# A systematic literature review informing the consensus statement on efficacy and safety of pharmacological treatment with interleukin-6 pathway inhibition with biological DMARDs in immune-mediated inflammatory diseases

**DOI:** 10.1136/rmdopen-2022-002359

**Published:** 2022-09-07

**Authors:** Kastriot Kastrati, Daniel Aletaha, Gerd R Burmester, Eva Chwala, Christian Dejaco, Maxime Dougados, Iain B McInnes, Angelo Ravelli, Naveed Sattar, Tanja A Stamm, Tsutomu Takeuchi, Michael Trauner, Desirée van der Heijde, Marieke J H Voshaar, Kevin Winthrop, Josef S Smolen, Andreas Kerschbaumer

**Affiliations:** 1 Division of Rheumatology, Department of Medicine III, Medical University of Vienna, Wien, Austria; 2 Rheumatology and Clinical Immunology, Charite University Hospital Berlin, Berlin, Germany; 3 University Library, Medical University of Vienna, Wien, Austria; 4 Rheumatology, Medical University of Graz, Graz, Austria; 5 Rheumatology, Hospital of Bruneck, Bruneck, Italy; 6 Hopital Cochin, Rheumatology, Université Paris Descartes, Paris, France; 7 Institute of Infection, Immunity & Inflammation, University of Glasgow, Glasgow, UK; 8 UO Pediatria II—Reumatologia, Istituto Giannina Gaslini, Genova, Italy; 9 Glasgow Cardiovascular Research Center, University of Glasgow, Glasgow, UK; 10 Section for Outcomes Research, Medical University of Vienna, Vienna, Austria; 11 Division of Rheumatology, Department of Internal Medicine, Keio University School of Medicine Graduate School of Medicine, Shinjuku-ku, Japan; 12 Division of Gastroenterology and Hepatology, Medical University of Vienna Department of Medicine III, Wien, Austria; 13 Rheumatology, LUMC, Leiden, The Netherlands; 14 Department of Pharmacy and Department of Research & Innovation, Sint Maartenskliniek, Ubbergen, The Netherlands; 15 Department of Pharmacy, Radboud University Medical Center, Nijmegen, The Netherlands; 16 Schools of Medicine and Public Health, Oregon Health and Science University, Portland, Oregon, USA

**Keywords:** Cytokines, Autoimmune Diseases, Inflammation

## Abstract

**Objectives:**

Informing an international task force updating the consensus statement on efficacy and safety of biological disease-modifying antirheumatic drugs (bDMARDs) selectively targeting interleukin-6 (IL-6) pathway in the context of immune-mediated inflammatory diseases.

**Methods:**

A systematic literature research of all publications on IL-6 axis inhibition with bDMARDs published between January 2012 and December 2020 was performed using MEDLINE, EMBASE and Cochrane CENTRAL databases. Efficacy and safety outcomes were assessed in clinical trials including their long-term extensions and observational studies. Meeting abstracts from ACR, EULAR conferences and results on clinicaltrials.gov were taken into consideration.

**Results:**

187 articles fulfilled the inclusion criteria. Evidence for positive effect of IL-6 inhibition was available in various inflammatory diseases such as rheumatoid arthritis, juvenile idiopathic arthritis, giant cell arteritis, Takayasu arteritis, adult-onset Still’s disease, cytokine release syndrome due to chimeric antigen receptor T cell therapy and systemic sclerosis-associated interstitial lung disease. Newcomers like satralizumab and anti-IL-6 ligand antibody siltuximab have expanded therapeutic approaches for Castleman’s disease and neuromyelitis optica, respectively. IL-6 inhibition did not provide therapeutic benefits in psoriatic arthritis, ankylosing spondylitis and certain connective tissue diseases. In COVID-19, tocilizumab (TCZ) has proven to be therapeutic in advanced disease. Safety outcomes did not differ from other bDMARDs, except higher risks of diverticulitis and lower gastrointestinal perforations. Inconsistent results were observed in several studies investigating the risk for infections when comparing TCZ to TNF-inhibitors.

**Conclusion:**

IL-6 inhibition is effective for treatment of several inflammatory diseases with a safety profile that is widely comparable to other bDMARDs.

**WHAT IS ALREADY KNOWN ON THIS TOPIC**

- Since the 2013 consensus statement on blocking the effects of interleukin-6 (IL-6) with biological disease-modifying antirheumatic drugs (bDMARDs), the body of evidence has grown widely.
- Data of clinical trials on several novel compounds targeting the IL-6 pathway in various immune-mediated inflammatory diseases have become available. This systematic literature review (SLR) was performed to inform an international consensus task force charged with updating the previous recommendations on pharmacological interventions with bDMARDs specifically targeting the IL-6 pathway.

**WHAT THIS STUDY ADDS**

- Anti-IL-6 bDMARDs were effective in various inflammatory diseases with an emphasis on rheumatic diseases, including rheumatoid arthritis, systemic and polyarticular-course juvenile idiopathic arthritis, giant cell arteritis, adult-onset Still’s disease, Takayasu arteritis as well as systemic sclerosis-associated interstitial lung disease. Clinically important differences in efficacy were further demonstrated in inflammatory conditions such as Castleman’s disease, neuromyelitis optica and chimeric antigen receptor T cell induced cytokine release syndrome. Use of tocilizumab resulted in better clinical outcomes and reduced mortality in patients with advanced stage of SARS-CoV-2 infection.
- Targeting IL-6 in osteoarthritis, psoriatic arthritis, ankylosing spondylitis and certain connective tissue diseases (systemic lupus erythematosus, myositis and Sjogren’s syndrome) was not beneficial.
- Safety outcomes regarding cardiovascular events, venous thromboembolism or malignancy did not differ from conventional DMARDs or bDMARDs with other modes of action. Risk of lower gastrointestinal perforations is low, but higher compared with other bDMARDs and in line with previously published reports.

**HOW THIS STUDY MIGHT AFFECT RESEARCH, PRACTICE OR POLICY**

- This SLR was conducted to inform the task force on ‘Consensus statement on blocking IL-6 receptor and IL-6 in inflammatory conditions: An update’ with the emerged evidence published from January 2012 onwards.

## Introduction

Basic research identified interleukin-6 (IL-6) as a cytokine with pleotropic activities and underlying abilities to promote inflammation and autoimmunity. The continuing development of biological agents that selectively target the IL-6 pathway has expanded the therapeutic armamentarium for various chronic inflammatory diseases over the last two decades.^1^ Tocilizumab (TCZ) was the first introduced humanised monoclonal antibody directed to membrane-bound as well as soluble IL-6 receptors (IL-6R), consequently inhibiting IL-6 from interacting with the IL-6R. By demonstrating therapeutic benefits in different clinical trials and indications, TCZ corroborated the concept of IL-6-related research.^2^ Previous evidence obtained by a systematic literature search in 2012, informed a consensus statement which was published in 2013 and provided points to consider when commencing TCZ and clinically relevant information regarding practical management.^3 4^ However, these statements addressed recommendations almost exclusively on TCZ and focusing on rheumatoid arthritis (RA) and to a minor extent on systemic juvenile idiopathic arthritis (sJIA), as other biological disease-modifying antirheumatic drugs (bDMARDs) targeting IL-6 (receptor or ligand) were in development and few or no other clinical data were available at that time.

Within the last decade, the body of evidence on bDMARDs blocking the IL-6-IL-6R axis has undergone a dynamic evolution. Data from clinical trials on novel compounds that target the IL-6 pathway in RA, such as sarilumab (SAR), and also on indications beyond RA and sJIA have become available.^5^ Furthermore, negative results of IL-6 pathway inhibition in diseases such as psoriatic arthritis, ankylosing spondylitis or some connective tissue diseases helped to shed more light on the immunopathology of these diseases.^6–11^ In RA, the body of evidence for using TCZ and other agents in different populations has grown extensively.^12–17^ Studies with emphasis on exploring tapering or cessation of IL-6 blockade in patients with RA who have achieved clinical remission have provided valuable evidence for clinicians.^18–23^ Safety data were derived from clinical trials of TCZ or SAR and from large prospective cohorts assessing long-term safety. This systematic literature review (SLR) was conducted to inform a consensus task force charged with developing an update of the original recommendations on pharmacological interventions with biological DMARDs targeting IL-6 pathway to account for the latest developments in indications, efficacy, safety as well as biomarker assessment, patient adherence and health economic aspects. This new SLR is considered an update of the SLR performed for the corresponding 2013 consensus statement.^3 4^

## Methods

### Literature search

The framework for this literature search and research questions were defined in the course of a steering group meeting with experts in various medical disciplines on 31 August 2020. A review protocol and definition of PICO (Population, Intervention, Comparator, Outcomes) were drafted and finally approved by the steering group. Details of the research questions developed by the steering group are provided in online supplemental tables 1.1.1–1.1.4. This SLR was conducted in adherence to the European League Against Rheumatism (EULAR) standardised operating procedures for recommendations and Preferred Reporting Items for Systematic reviews and Meta-Analyses (PRISMA) statement.^24 25^ Based on the research questions defined, five different searches on IL-6 inhibiting agents were carried out separately, namely: (1) efficacy in approved indications (including biomarker assessment); (2) efficacy in other studied diseases; (3) safety in approved indications; (4) safety in other studied diseases and, finally, (5) adherence, patient preferences and economic aspects. A professional librarian with longstanding experience (EC) conducted the database search using MEDLINE, EMBASE and the Cochrane Library’s Register of Controlled Trials (CENTRAL). Articles published in full and in English language were eligible for inclusion as well as conference abstracts presented at the EULAR and American College of Rheumatology (ACR) annual meetings from 2019 to 2020 (conference abstracts at ACR 2020 were hand-searched). Preliminary results from ongoing clinical trials, which were neither published in articles nor abstracts, were obtained from clincaltrials.gov insofar as sufficient data were available.^26^ As this SLR was performed to incorporate evidence since the last SLR for the corresponding 2013 consensus,^3 4^ articles published from 1 January 2012 to 15 January 2021 (last date searched) were included.

10.1136/rmdopen-2022-002359.supp1 Supplementary data

For articles to be eligible for inclusion, the following criteria were defined: (1) Efficacy: randomised, controlled, double-blind phase III trials (RCT) with a study duration of ≥3 months and sample size of ≥50 patients investigating IL-6 receptor or ligand inhibition with bDMARDs. (2) Open label studies addressing strategic, switching or dose-reduction issues or phase II trials if no phase III trial was available. Trials with earlier primary endpoint or trials with smaller sample size were eligible for inclusion if no other study fulfilled inclusion criteria. (3) Safety: observational studies, primarily, cohort studies, case control studies and registry data. (4) Safety data from RCTs, long-term extensions (LTEs) or postmarketing data of compounds of interest were further eligible for extracting safety data if registry data were not available. Description of PICOs and details on complete search strategy are listed in online supplemental section 1: 1.2.1–1.3.5.3.

### Study selection, data extraction and risk of bias assessment

During the study selection process, one reviewer (KK) evaluated all retrieved publications by title and abstract screening for eligibility. After the initial screening, a detailed assessment for inclusion of preselected articles was performed. In case of uncertainties, these were discussed with the methodologist (AK). Data of studies selected for inclusion were extracted based on disease-specific variables of interest which were predefined in the review protocol. Risk of bias (RoB) analysis was conducted using the Cochrane Collaboration’s Risk of Bias tool for RCTs and additionally the Newcastle-Ottawa Scale (NOS) for observational studies and case-control studies. Due to high heterogeneity of available studies, the steering group decided that no pooling of efficacy or safety outcomes by meta-analysis should be performed. Thus, the results in this manuscript are reported narratively.

## Results

**Figure 1:**
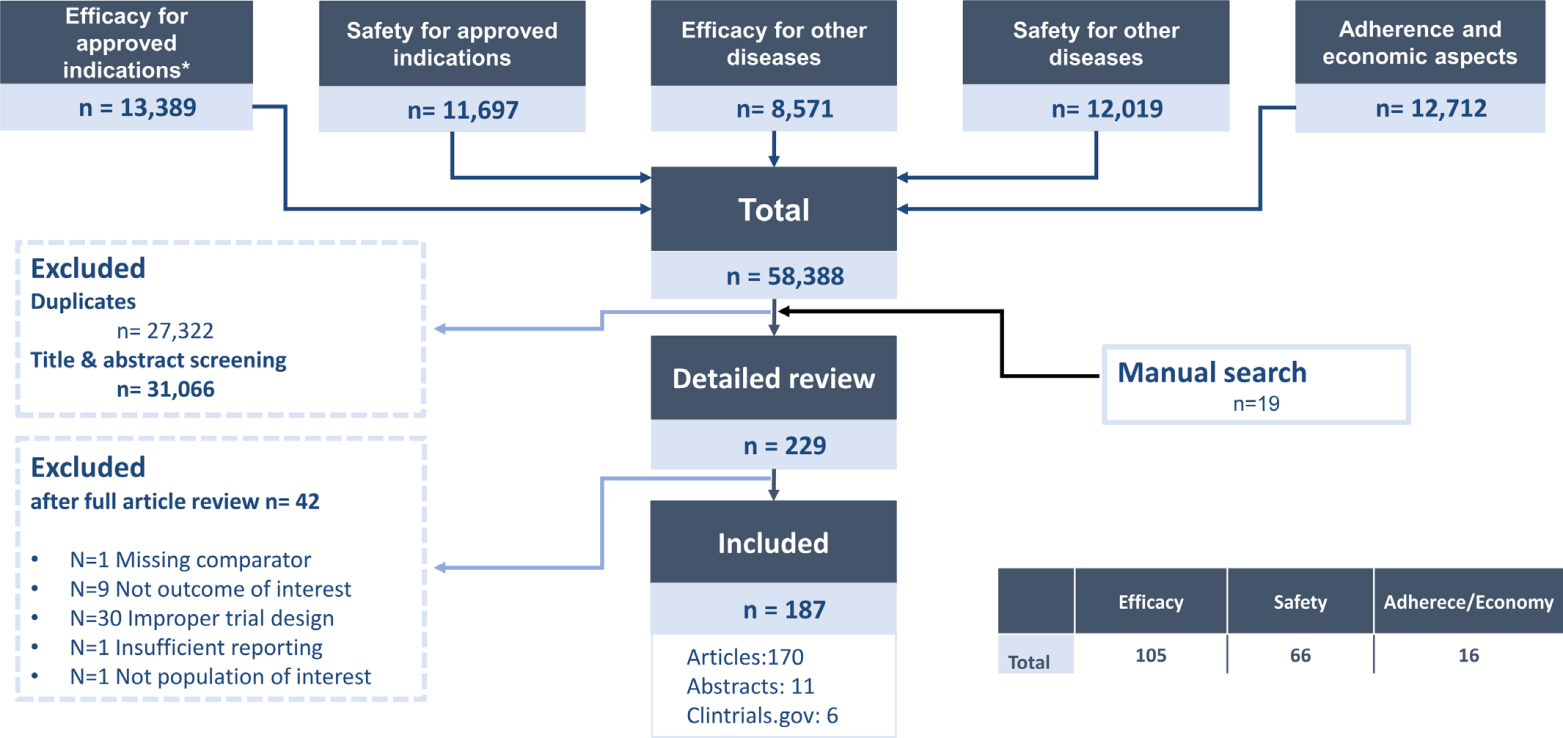
Flowchart describing the study selection process.

After deduplication, a total of 31 066 records remained for title and abstract screening. A total of 229 articles were selected for full-text review, of which 187 were finally included. Of these, 105 articles were eligible for extraction on efficacy including biomarker assessment, 66 on safety and 16 on adherence and health economic aspects. A flowchart with a detailed description of the selection process is shown in figure 1. Characteristics of each publication eligible for data-extraction, baseline characteristics, outcomes for each intervention group and the respective reference (section 8 in the online supplemental appendix) are shown in the supplement. For most of the RCTs included, RoB was considered as low. However, RoB on open-label studies was classified as high due to their unblinded design. RoB assessment was not performed on ACR/EULAR abstracts, trials available on clinicaltrial.gov or studies on biomarker assessment, patient adherence and economic aspects. Details of all articles and abstracts included and RoB assessment are provided in online supplemental appendix (Section 2: Efficacy for approved indications; Section 3: Efficacy for other studied diseases; Section 4: Safety aspects of interleukin-6 pathway inhibition; Section 5: Biomarkers for prediction of therapeutic response of interleukin-6 pathway inhibition; Section 6: Patient adherence/preferences and economic aspects of interleukin-6 pathway inhibition).

10.1136/rmdopen-2022-002359.supp2 Supplementary data

### Efficacy for approved indications

**Figure 2:**
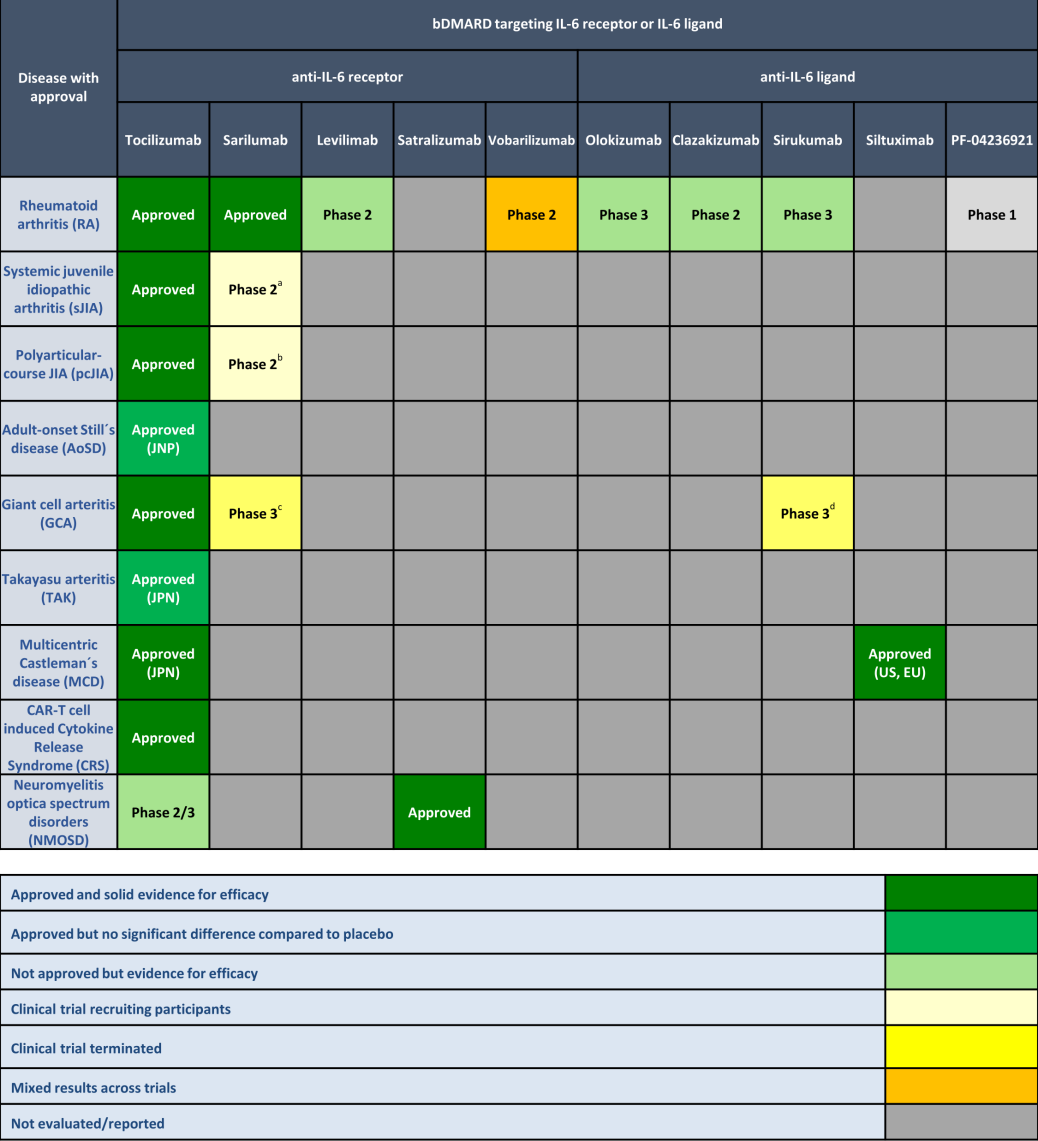
Efficacy of biological disease modifying antirheumatic drugs targeting the IL-6 receptor or ligand and their relative efficacy and/or regulatory approvals across immune-mediated inflammatory diseases (based on available data at end of December 2020). aNCT02991469 (recruiting); bNCT02776735 (recruiting); cNCT03600805 (study terminated, results are awaited); d trial terminated early due to sponsor decision. For colorblind readers, figure 2 is provided in the online supplemental appendix (section 7: S7.1). EU, European Union; JPN, Japan; RU: Russian Federation; US: United Stated of America.

A total 13 389 publications were retrieved for screening and 76 full text publications selected for inclusion regarding efficacy for approved indications. Studies evaluating biomarkers for prediction of therapeutic response of IL-6 pathway inhibition were incorporated in this search and are shown in online supplemental tables S5.1 and S5.2. Figure 2 shows bDMARDs specifically inhibiting IL-6 receptor or ligand and their approved indications based on available data at end of December 2020. At this date, TCZ was approved for RA, sJIA, polyarticular-course juvenile idiopathic arthritis (pcJIA), giant cell arteritis (GCA), chimeric antigen receptor (CAR)-T cell induced cytokine release syndrome (CRS), and in Japan for adult-onset Still’s disease (AoSD), Takayasu arteritis (TAK) as well as multicentric Castleman’s disease (MCD). Among the other anti-IL-6R antibodies, SAR is approved for RA in EU, Japan and USA and satralizumab (SAT) for treatment of seropositive neuromyelitis optica spectrum disorders (NMOSD). In addition, several compounds selectively binding the IL-6 ligand, including the humanised antibody olokizumab (OKZ) as well as chimeric monoclonal antibody siltuximab (SIL), were developed. OKZ received market authorisation for the treatment of RA in Russia in 2020. SIL is the first anti-IL-6 drug to be licensed for the treatment of MCD in the United States of America (USA) and the European Union (EU).

#### Rheumatoid arthritis (RA)

In total, 42 studies investigating bDMARDs selectively inhibiting IL-6 receptor or cytokine in patients with RA were included (low RoB: n=15; unclear RoB: n=7; high RoB: n=13, conference abstracts: n=3; not fully published with available study results on clinicaltrials.gov: n=4; for details of RoB analyses, see online supplemental section S2.2.1. Efficacy data were grouped into (1) efficacy trials (in combination with methotrexate (MTX) or other conventional synthetic DMARD (csDMARD) or as monotherapy); (2) head-to-head trials with other bDMARDs; (3) trials investigating routes of administration; (4) adding versus switching trials; (5) induction/strategic trials; and 6) stopping or dose reduction trials. Patients were grouped based on population (treatment naïve, previous insufficient response to conventional synthetic DMARDs and tumour necrosis factor inhibitors (TNF-i)). Details on study characteristics, baseline characteristics and efficacy outcomes are summarised in online supplemental tables S2.1.1, S2.3.1.1–S2.3.1.12 and S2.4.1.1–S2.4.1.12.

##### Efficacy trials

Trials comparing bDMARDs specifically inhibiting IL-6 receptor or ligand to placebo in patients with established RA with inadequate response (IR) to methotrexate (MTX) or conventional synthetic disease-modifying drugs (csDMARDs) showed effective reduction of signs and symptoms for several anti-IL-6R agents, including TCZ (Korean patients),^13^ SAR^12 27 28^ and BCD-089 (levilimab).^29^ Baseline characteristics and detailed efficacy outcomes are shown in online supplemental tables S2.3.1.1 and S2.4.1.1. A not yet published study of a phase II trial investigating ALX-0061 (vobarilizumab), an anti-IL-6R nanobody, did not show significant differences from placebo on achieving American College of Rheumatology 20% improvement (ACR20 response) in MTX-IR patients.^30^ In an early open-label phase II study, vobarilizumab monotherapy had similar numerical ACR response rates compared with TCZ.^31^ Compounds selectively targeting IL-6 cytokine such as clazakizumab (CLZ) (formerly ALD518 and BMS-945429),^32^ OKZ^33^ and sirukumab,^34^ respectively, were associated with significant improvement in signs, symptoms and physical function compared with placebo in patients with RA refractory to MTX or csDMARDs. Four trials (all with low RoB) studied the efficacy of agents binding IL-6 receptor or ligand in patients with an IR or intolerance to antitumour necrosis factor (TNF) therapy. All showed comparable results with improvements in signs and symptoms of RA, irrespective of the compound used (SAR,^35^ OKZ^36 37^ and sirukumab^38^). Three studies addressed the efficacy of TCZ in patients with previous insufficient treatment response to csDMARDs and TNF-inhibitors. In the BREVACTA study, the subcutaneous formulation of TCZ (TCZ-SC) was superior to placebo (PBO-SC) showing higher ACR responses (ACR20 response: 60.9% vs 31.5%) and inhibition of radiographic joint damage at week 24 in patients who had an IR to ≥1 DMARDs, including anti-TNF agents.^15^ Intravenous TCZ was more effective than placebo in reducing signs and symptoms in patients with RA who failed to respond adequately to DMARD therapy, among them approximately 38% in both groups with previous use of anti-TNF agents.^14^ The TORPEDO study failed to achieve its primary endpoint of improvement in Health Assessment Questionnaire—Disability Index (HAQ-DI) at week 4 when comparing intravenous TCZ to placebo in patients with IR to csDMARDs or anti-TNFs. The study was completed in 2014, but has not been published until the timepoint of submission of this manuscript (clinicaltrials.gov identifier: NCT00977106).^39^

##### Head-to-head trials

Three head-to-head studies were included (all three with high RoB). Efficacy results are summarised in table 1 (baseline characteristics and detailed efficacy outcomes are shown in online supplemental tables S2.3.1.4 and S2.4.1.4). TCZ and SAR monotherapy demonstrated clinical and functional superiority compared with adalimumab (ADA) monotherapy in patients who were intolerant or inadequate responders to MTX.^40 41^ The SIRROUND-H study assessed superiority of sirukumab monotherapy over ADA monotherapy in biologic-naïve patients who did not respond to MTX. The study failed to meet one of its coprimary endpoints with no significant differences in ACR50% response rates at week 24; however, sirukumab showed greater improvements with regard to the Disease Activity Score in 28 joints (DAS28) at week 24.^42^ Due to pronounced effects of IL-6 inhibition on hepatic acute phase reactant (C reactive protein (CRP)) production and erythrocyte sedimentation rate (ESR), an overestimation of improvement or response rates when using composite disease activity measures that include acute-phase reactants, such as the DAS28 or the Simplified Disease Activity Index (SDAI), can occur. Especially, the ESR is highly weighted in the DAS28, whereas CRP level exhibits low weight in SDAI.^43^ Thus, studies using composite disease activity measures comprising acute-phase reactants were judged as being at high RoB. In patients who had failed MTX, Weinblatt *et al* reported significantly greater ACR20 response rates at week 12 of CLZ over placebo in a randomised dose-ranging study. Herein, ADA was included as active reference; however, the study was not powered for head-to-head comparisons between CLZ and ADA.^44^

**Table 1:** Head-to-head studies comparing bDMARDs specifically targeting IL-6 receptor or ligand to tumour necrosis factor alpha inhibitors

Population	Study	Design	Risk of bias (RoB)	Treatment arm	No. of patients (n)	Primary endpoint		P value (primary endpoint)	ACR20 (%)	ACR50 (%)	ACR70 (%)	DAS28 <2.6 (%)	CDAI≤2.8 (%)	ACR/EULAR Boolean remission (%)	ΔHAQ (mean)
MTX-IR	Gabay *et al* (2013) (ADACTA)^40^	Superiority trial	High	ADA 40 mg Q2W	162	ΔDAS28-ESR at week 24	−1.8	<0.0001	49.4	27.8	17.9	10.5	9.3*	NR	−0.5
				TCZ 8 mg/kg Q4W	163		−3.3		65.0	47.2	32.5	39.9	17.2*	NR	−0.7
MTX-IR	Burmester *et al* (2017) (MONARCH) 41	Superiority trial	High	ADA 40 mg Q2W	185	ΔDAS28-ESR at week 24	−2.2	<0.0001	58.4	29.7	11.9	7.0	2.7	NR	−0.43
				SAR 200 mg Q2W	184		−3.28		71.7	45.7	23.4	26.6	7.1	NR	−0.61
MTX-IR	Taylor *et al* (2018) (SIRROUND-H)^42^	Superiority trial	High	ADA 40 mg Q2W	186	ΔDAS28-ESR+ACR 50 (%) at week 24	−2.19	Reference	56.5	31.7	12.9	7.5	NR	3.8	−0.52
				SRK 50 mg Q4W	186		−2.58	0.013	53.8	26.9	11.8	12.9	NR	3.8	−0.51
				SRK 100 mg Q2W	187		−2.96	<0.001	58.8	35.3	15.5	20.3	NR	3.7	−0.53
MTX-IR	Weinblatt *et al* (2015)^44^‡	Dose-ranging study with active comparator (ADA)	Unclear	Placebo+MTX	61	ACR 20 (%) at week 12	39.3	Reference	37.7†	NR	6.6†	1.6	3.3	3.3	−0.62†
				ADA 40 mg Q2W+MTX	59		76.3	<0.05*	66.1†	NR	18.6†	20.3	8.5	5.1	−0.66†
				CLZ 25 mg Q4W+MTX	59		76.3	<0.05	81.4†	NR	27.1†	35.6	11.9	8.5	−0.68†
				CLZ 100 mg Q4W+MTX	60		73.3	<0.05	65.0†	NR	40.0†	35.0	8.3	10.0	−0.79†
				CLZ 200 mg Q4W+MTX	60		60.0	<0.05	66.7†	NR	30.0†	26.7	3.3	5.0	−0.71†
				CLZ 100 mg Q4W+Placebo	60		55.0	<0.05	58.3†	NR	16.7†	21.7	8.3	6.7	−0.64†
				CLZ 200 mg Q4W+Placebo	59		61.0	<0.05	57.6†	NR	25.4†	25.4	3.4	1.7	−0.60†

Results of secondary efficacy outcomes are depicted at the time point of the primary endpoint. ADACTA-H, MONARCH-H and SIRROUND-H trial were judged as being at high RoB due to inclusion of acute phase reactants in the outcome of the primary endpoint.

*Posthoc analysis.

†Efficacy outcome at week 24.

‡Study not powered to compare CLZ with ADA.

ACR, American College of Rheumatology; ADA, adalimumab; CDAI, Clinical Disease Activity Index; CLZ, clazakizumab; DAS28, Disease Activity Score of 28 joints; ESR, erythrocyte sedimentation rate; HAQ, Health Assessment Questionnaire; MTX, methotrexate; NR, not reported; Q2W, every other week; Q4W, once every 4 weeks; SAR, sarilumab; SRK, sirukumab; TCZ, tocilizumab; Δ, change from baseline.

##### Trials investigating routes of administration

SUMMACTA compared subcutaneous (SC) versus intravenous formulations (IV) of TCZ in cs-/bDMARD-IR RA patients. TCZ-SC 162 mg weekly (QW) was non-inferior to TCZ-IV 8 mg/kg.^45^ In the multicentre, double-blind MUSASHI study, TCZ-SC every other week (Q2W) confirmed the non-inferiority of TCZ-SC monotherapy to TCZ-IV monotherapy in Japanese patients with an IR to csDMARDs and/or bDMARDs.^46^ An 84-week open-label extension period of the MUSASHI study evaluated the efficacy of switch from weight-titrated–dose TCZ-IV monotherapy to fixed-dose TCZ-SC monotherapy. Switching to another route of administration (IV to SC) was associated with comparable efficacy in the majority of patients, even though it was somewhat lower in patients with high body weight.^47^ In patients with RA with IR to TCZ-SC Q2W, TCZ-SC weekly was superior to TCZ-SC Q2W for adjusted mean change in DAS28-ESR from baseline to week 12 in a Japanese study. Therefore, shortening the dosing interval to QW improved efficacy with acceptable tolerability.^48^ Details on studies investigating route of administration are shown in online supplemental tables S2.3.1.5 and S2.4.1.5.

##### Adding versus switching trials

Pertaining to switching within the IL-6R blocker class, a posthoc analysis of the EXTEND trial, an open-label extension study of the ASCERTAIN trial, investigated patients switched from double-blind TCZ to open label SAR, showing sustained clinical efficacy over 96 weeks.^49^ Apart from that, two key studies (one with unclear and another with high RoB due to being not double-blinded and a lower number of patients enrolled as initially planned) compared the effectiveness between TCZ added to MTX and TCZ monotherapy (switched from MTX) in established RA. Clinical data suggested benefit of add-on (TCZ+MTX) over switching to TCZ, and data of radiographic progression were in favour of the add-on strategy in both studies, whereas in patients receiving TCZ monotherapy radiographic progression was numerically higher.^50–52^ Study details are depicted in online supplemental tables S2.3.1.6–S2.3.1.7 and S2.4.1.6–S2.4.1.7.

##### Induction and strategic trials

Three trials (two with high RoB due to including acute-phase reactants in the primary outcome and one with high RoB due to open label design) as well as one report with 2-year results on induction therapy with TCZ in early progressive RA were evaluated (for study details, see online supplemental tables S2.3.1.8-S2.3.1.9, S2.4.1.8-S2.4.1.9). The FUNCTION trial demonstrated significantly higher proportions of patients achieving DAS28-ESR≤2.6 when receiving 8 mg/kg TCZ+MTX or 8 mg/kg TCZ monotherapy in comparison with placebo+MTX at week 24 (45% and 39% vs 15%). Patients treated with 8 mg/kg TCZ plus MTX achieved significantly less radiographic progression and better HAQ-DI improvement at week 52 than did patients with MTX monotherapy.^16^ Furthermore, TCZ 8 mg/kg+MTX compared with TCZ monotherapy was associated with less radiographic progression at week 104 (mean change from baseline in van der Heijde-modified total Sharp score, 0.19 vs 0.62).^53^ However, treatment induction with TCZ monotherapy in early RA was not superior to MTX alone in FUNCTION in achievement of secondary endpoints, including ACR responses and Clinical Disease Activity Index (CDAI) remission at week 52.^16^

The U-Act-Early study was a 2-year RCT, in which DMARD-naïve patients with early RA were treated to the target of sustained remission defined as DAS28<2.6 with a swollen joint count less than or equal to four, persisting for at least 24 weeks. Patients were randomised to TCZ+MTX, TCZ monotherapy or MTX monotherapy using a treat-to-target approach. In the primary analysis (proportion of patients achieving sustained DAS28<2.6), significantly more patients achieved the target while being treated with their initial treatment regimen when TCZ was part of the regimen, compared with MTX monotherapy (TCZ+MTX: 86% vs TCZ mono: 84% vs MTX mono: 44%).^17^ However, following a treatment step-up according to a standardised treatment protocol during the entire study period, initial significant differences in achieving sustained DAS28<2.6 were no longer observed after the addition of TCZ in the MTX monotherapy group (TCZ+MTX: 86% vs TCZ mono: 88% vs MTX mono: 77%).^17^ A randomised, open-label strategic study (NORD-STAR) compared the proportion of patients with treatment-naïve, early RA achieving CDAI remission with active conventional therapy based on MTX combined with glucocorticoids (GC) with three different biological therapies, including TCZ, certolizumab-pegol (CZP) and abatacept (ABA) with background MTX. Non-inferiority was demonstrated for active conventional treatment regimens (including GC with rapid tapering or intra-articular GC, sulfasalazine and hydroxychloroquine) versus TCZ or CZP, but not for ABA at week 24.^54^

##### Stopping or dose reduction trials

Eight studies (two rated as having low RoB, three with unclear RoB and two judged as being at high RoB due to open-label study-design; one conference abstract) investigated tapering and discontinuation of IL-6 inhibiting agents or concomitant csDMARDs as a concept for achieving a more tailored treatment approach (for details, see online supplemental tables S2.3.1.10-S2.3.1.12, S2.4.1.10-S2.4.1.12). Four studies investigated a tapering and discontinuation strategy of concomitant MTX, one trial of GC in a subset of patients who achieved DAS≤3.2, whereas in three other studies TCZ was discontinued, or the interval of application extended once DAS28<2.6 was reached. The non-inferiority of MTX tapering versus continuation in patients receiving ongoing TCZ therapy without significant worsening of disease activity was demonstrated in three studies.^18–20^ Tapering or even stopping MTX was possible in some patients, but continuation of MTX led to numerically better results in different outcomes compared with TCZ monotherapy (DAS28-ESR≤3.2 at week 52: TCZ mono: 62.6% versus TCZ+MTX: 68% and DAS28-ESR<2.6 at week 52: TCZ mono: 48.3% vs TCZ+MTX: 55.1%).^19^ In a COMP-ACT substudy, MRI revealed similar intra-articular inflammation and damage in patients who discontinued MTX versus those who continued TCZ+MTX.^55^ The SEMIRA trial investigated patients treated with TCZ±csDMARD who achieved DAS28-ESR≤3.2 and had a stable GC dosage of 5 mg per day. Blinded tapering of GCs with continuation of TCZ resulted in a significant increase of disease activity in the discontinuation arm compared with patients who continued GCs (DAS28-ESR difference: 0.613; p<0.001). Sixty-five per cent of patients tapering GC remained in stable disease activity without experiencing a flare versus 77% of patients in the continued group. During tapering of GC, no difference in adverse events (AEs) was observed, especially no differences in the occurrence of adrenal insufficiency were reported.^56^ Other studies investigated the discontinuation of TCZ after achieving sustained DAS28-ESR<2.6. A follow-up of the ACT-RAY study showed that among patients who reached sustained DAS28-ESR<2.6 and discontinued TCZ, 238 of 472 patients (50.4%) achieved TCZ-free remission. However, a total of 200 patients (82.5%) subsequently flared following TCZ-free remission, with 82.5% of the TCZ+MTX combination group and 88.5% of the TCZ monotherapy group. The median time to flare once TCZ-free remission was reached was 113 days in the add-on group compared with 84 days in the switch group. The majority of patients (n=186; 94 patients from add-on and 92 from switch group) reinitiated TCZ after flaring and responded well to TCZ resumption by demonstrating improvements after restarting TCZ, with mean (SD) DAS28-ESR decreasing to 3.01 (1.25), 2.42 (1.18) and 2.19 (1.04) at three consecutive assessments visits 12 weeks apart after flare. Furthermore, the proportion of patients with high (DAS28-ESR>5.2) or moderate (DAS28-ESR>3.2 to≤5.2) disease activity was 29.7%, 18.6% and 11.7%, respectively, at these visits.^21^ In the second year of the SURPRISE study, at total of 105 patients who achieved DAS28-ESR<2.6 at week 52 discontinued TCZ. Among patients who had been previously receiving add-on treatment (TCZ+MTX) in the first year continued MTX alone, while patients in the switch arm (MTX switched to TCZ) continued without any DMARD. All patients were followed for additional 52 weeks. Numerically more patients who continued MTX (originally the add-on group) remained TCZ-free compared with patients in the switch group (67.3% vs 53.1%, p=0.22). Sustained DAS28 low disease activity states were observed more frequently in patients receiving MTX (add-on group). Radiological progression was numerically but not significantly higher in patients without DMARDs (switch group) at week 104 (mTSS; 0.37 vs 0.64, p=0.36).^22^

#### Systemic juvenile idiopathic arthritis (sJIA), polyarticular-course juvenile idiopathic arthritis (pcJIA) and adult-onset Still’s disease (AoSD)

In sJIA, one pivotal RCT of TCZ (TENDER, unclear RoB) including 112 children with IR to non-steroidal anti-inflammatory drugs (NSAIDs) and GC was published (for details, see online supplemental tables S2.2.2, S2.3.2.1 and S2.4.2.1). TENDER confirmed improvements in signs and symptoms of sJIA during TCZ treatment and showed the clinically relevant GC-sparing effect. The primary end point, defined as JIA ACR 30 response and absence of fever at week 12, was met in significantly more patients of the TCZ group (85%) compared with the placebo arm (24%).^57^

In pcJIA, results of a phase III study (CHERISH, low RoB) demonstrated that TCZ was efficacious for the management of MTX-IR patients (for details, see online supplemental tables S2.2.3, S2.3.3.1 and S2.4.3.1). In the first part of CHERISH, all 188 patients received open-label TCZ, followed by a second part of double-blind, 24-week withdrawal phase in patients who achieved JIA-ACR 30 response. After the first study part, by week 16, a high proportion (89%) of patients achieved JIA-ACR30 response and 26% even achieved JIA-ACR90 response. The CHERISH study met its primary endpoint showing that patients in the placebo group experienced significantly more JIA-flares than TCZ-treated patients during the withdrawal phase. JIA flare occurred in 48.1% of patients on placebo versus 25.6% continuing TCZ at week 40.^58^ One study investigated radiographic progression over 2 years in the TENDER and CHERISH trial, reporting the potential of TCZ to delay radiographic progression in children with sJIA and pcJIA.^59^

AoSD is regarded a counterpart of sJIA in adulthood. In a double-blind RCT (low RoB) including 27 patients published in 2018, treatment with TCZ in AoSD-patients refractory to GC did not result in a significant difference concerning the achievement of the primary endpoint (ACR50 response at week 4) compared with placebo (ACR 50 at week 4: 61.5% vs 30.8%; p=0.24). However, TCZ was associated with a therapeutic benefit in systemic symptoms and steroid-sparing effects compared with the placebo group.^60^ In 2019, this pivotal trial led to the approval of TCZ for the treatment of AoSD in Japan. Study details are shown in online supplemental tables S2.2.4, S2.3.4.1 and S2.4.4.1)

#### Giant cell arteritis (GCA) and Takayasu arteritis (TAK)

Several studies evaluated the efficacy of bDMARDs targeting IL-6 pathway in systemic large-vessel vasculitides, especially GCA and TAK. In total, four reports on patients suffering from GCA were included in this SLR (for details, see online supplemental tables S2.2.5, S2.3.5.1 and S2.4.5.1). In the phase III GiACTA trial (low RoB), 251 patients were randomly assigned to receive TCZ weekly or every other week combined with a 26-week prednisone taper or placebo combined with a prednisone taper of either 26 weeks or 52 weeks. TCZ was superior to placebo in achievement of GC-free remission at week 52 in patients with GCA. Sustained GC-free remission at 52 weeks was achieved in 56% of the patients treated with TCZ weekly and 53% in the TCZ every other week arm, compared with 14% of patients receiving placebo and a 26-week prednisone taper and 18% of those in the placebo group with the 52-week prednisone taper scheme (p<0.001 active treatments vs placebo). In addition, the cumulative median prednisone dose over the 52-week period was significantly lower in each TCZ group, as compared with placebo (p<0.001 for all comparisons).^61^ Based on a 3-year analysis of GiACTA (conference abstract), time to first flare favoured TCZ weekly over TCZ every other week in patients with new-onset and relapsing GCA.^62^ One multicentre study published as a conference abstract compared the efficacy of SC TCZ versus intravenous TCZ. 91.7% patients who received SC TCZ achieved prolonged remission after 12 months of treatment compared with patients receiving intravenous TCZ (61.4%, p=0.043). Patients in the SC TCZ group were able to successfully discontinue prednisone treatment after 24 months, whereas the median GC sparing effect of the intravenous group was 2.4 after 24 months (IQR 0–5).^63^

Sirukumab was investigated in one phase III RCT (including 161 patients) providing numerically lower proportions of flares at week 52 in the sirukumab arm versus placebo. However, the study was terminated early due to a sponsor decision.^64^

One key trial, the TAKT study, a double-blind RCT with 36 patients performed in Japan, investigated the efficacy of TCZ in patients with refractory Takayasu arteritis during GC tapering (for details, see online supplemental tables S2.2.6, S2.3.6.1 and S2.4.6.1). The TAKT study suggested efficacy in favour of TCZ over placebo for time to relapse, although the primary endpoint was not statistically different between TCZ and placebo.^65^

#### Multicentric Castleman’s disease (MCD)

TCZ has been licensed for the treatment of MCD in Japan since 2005. Studies on TCZ were discussed in the previous SLR and included in the previous consensus statement.^3 4^ The current SLR identified one new study (low RoB) published after 2013, confirming the efficacy of siltuximab (SIL), a monoclonal antibody selectively targeting the IL-6 cytokine, in MCD (see online supplemental tables S2.2.7, S2.3.7.1 and S2.4.7.1 for details of the included study). In this RCT including 79 patients, SIL in combination with best supportive care demonstrated superiority versus best supportive care alone regarding tumour and symptomatic response in HIV-negative and human herpesvirus-8-seronegative patients with symptomatic MCD.^66^ This study subsequently led to the approval of SIL in the EU and USA.

#### CAR-T cell induced cytokine release syndrome (CRS)

The approval of TCZ was based on a retrospective analysis of pooled data of adult and paediatric patients who developed CRS after treatment with CTL019 and KTE-C19 in prospective clinical trials including patients with B-cell lymphoma and acute lymphoblastic leukaemia. In this analysis, 45 patients with CTL019-induced CRS and 15 patients with CRS due to KTE-C19 were included. Among patients diagnosed with severe or life-threatening CAR T-cell induced CRS, in the majority of patients who developed CRS after receiving CTL019, symptoms resolved on therapy with TCZ (69%, median time to response 4 days). Likewise, of patients receiving TCZ after CRS due to KTE-C19 53% were considered responders (median time to response 4.5 days).^67^ While data on CAR-T cell induced CRS are rare, due to the retrospective nature and missing comparator arms, this analysis was judged as having a high RoB. Baseline characteristics and efficacy outcomes are depicted in online supplemental tables S2.2.8, S2.3.8.1 and S2.4.8.1, respectively.

#### Neuromyelitis optica spectrum disorders (NMOSD)

One open-label trial (high RoB) on TCZ and two RCTs (both with low RoB) on satralizumab (SAT) were available for efficacy evaluation in patients with relapsing NMOSD (for details, see online supplemental tables S2.2.9, S2.3.9.1 and S2.4.9.1). In the phase II/III TANGO trial, treatment with TCZ led to a significantly reduced risk of a subsequent NMOSD relapse compared with azathioprine. TCZ demonstrated a longer median time to the first relapse than azathioprine (78.9 vs 56.7 weeks; p=0.0026) as well as lower relapse rates at the end of the study (14% vs 59%; p<0.0001).^68^ In two phase III trials, SAT added to concomitant immunosuppressants^69^ or used as monotherapy^70^ resulted in a significant reduction of relapse rates compared with placebo. No differences in improvement of pain or fatigue with SAT were observed.^69^

### Efficacy for other studied diseases

Of 8571 records from the database search, additional ACR/EULAR conference abstracts and publications found eligible for inclusion after hand search, 26 articles and 2 trials with data available on clinicaltrial.gov were finally included.

Studies in line with the previously formulated inclusion criteria investigating the efficacy of bDMARDs specifically targeting IL-6 pathway in patients with ANCA-associated vasculitis, remitting seronegative symmetric synovitis with pitting oedema (RS3PE), refractory relapsing polychondritis, TNF-receptor associated periodic fever syndrome (TRAPS) or chronic infantile neurological cutaneous and articular (CINCA) syndrome were not available, as the body of evidence was limited to case reports.

Nevertheless, there is a plethora of immune-mediated diseases in which selective inhibition of IL-6 pathway has been evaluated with or without success in several investigator-initiated studies. Characteristics of each trial including study population, baseline characteristics, risk of bias assessment, results of studies and summary data for each intervention group are elaborated in online supplemental section 3. A summary of the efficacy of bDMARDs selectively inhibiting IL-6 axis across immune-mediated diseases of interest is shown in table 2.

**Table 2:** Efficacy outcomes of clinical trials published from 2012 to 2020 investigating bDMARDs specifically inhibiting IL-6 receptor or ligand compared against placebo or control group, shown across other studied immune-mediated diseases

Disease	Study	Target	Population	Intervention/ Control	Primary endpoint	Efficacy
Psoriatic arthritis	Mease *et al*^6^ (2016) phase IIb	IL-6	NSAID-IR and/or csDMARD bDMARD naïve	CLZ vs PBO	ACR 20 response at week 16	
Ankylosing spondylitis	Sieper *et al*^7^ (2014)(BUILDER) phase II/III	IL-6R	TNFi-naïve	TCZ vs PBO	ASAS 20 response at week 12	
	Sieper *et al*^8^ (2015) (ALIGN) phase II		NSAID-IR	SAR vs PBO	ASAS 20 response at week 12	
Osteoarthritis	Richette *et al*^71^ (2020) (TIDOA) phase III	IL-6R	Refractory to analgetics	TCZ vs PBO	ΔVAS pain at week 6	
Systemic lupus erythematosus	Wallace *et al*^9^ (2017)(BUTTERFLY) phase II	IL-6	Active disease (SLEDAI-2K/BILAG)	PF-04236921 vs PBO	SLE Responder Index (SRI-4) at week 24	
	Rovin *et al*^10^ (2016) phase II		Class III or class IV Lupus nephritis	SIR vs PBO	Reduction in proteinuria from baseline to week 24	
	NCT02437890 phase II	IL-6R	Moderate to severe active SLE	ALX-0061 vs PBO	mBICLA response rate at week 24	
Myositis	NCT02043548 phase II	IL-6R	Refractory PM/DM	TCZ vs PBO	Mean total improvement scores at visits 2–7	
Sjögren’s syndrome	Felten *et al*^11^ (2020) (ETAP) phase II/III	IL-6R	ESSDAI≥5	TCZ vs PBO	Response to treatment at week 24*	
Multiple myeloma	San-Miguel *et al*^75^ (2014) phase II	IL-6	Untreated MM and no candidate for stem cell transplantation	SIL+VMP vs VMP	Complete response rate†	
	Brighton *et al*^76^ (2019) phase II		High-risk smouldering multiple myeloma	SIL vs PBO	1-year progression-free survival rate	
Systemic sclerosis associated ILD	Khanna *et al*^74^ (2020) (focuSSced) phase III	IL-6R	Diffuse cutaneous-SSc; mRSS 10–35; inflammatory status	TCZ vs PBO	ΔmRSS from baseline to week 48; secondary outcome: ΔFVC% predicted from baseline to week 48	
Late antibody-mediated kidney transplant rejection	Doberer *et al*^77^ (2020) phase II	IL-6	Kidney transplant recipients with donor-specific, antibody-positive ABMR	CLZ vs PBO	Safety and tolerability; secondary outcomes: course of eGFR, protein/creatinine ratio	
AA-amyloidosis	Okuda *et al*^78 79^ (2014/2016) retrospective analyses	IL-6R	Amyloid A (AA) amyloidosis complicating rheumatic diseases	TCZ vs TNFi	Outcomes: retention rate, median ΔSAA, median ΔeGFR, mean ΔCDAI, mean ΔGC dose	
Polymyalgia rheumatica	Lally *et al*^80^ (2016) open label, phase IIa	IL-6R	PMR treated with GCs for ≤4 weeks	TCZ+GC vs GC	Relapse-free remission without GC treatment at 6 months	
	Devauchelle-Pensec *et al*^81^ (2016) (TENOR) open label, phase II		Active disease defined as PMR-AS >10	TCZ mono no control group	PMR-AS ≤10 at week 12	
COVID-19 CRS/pneumonia	Hermine *et al*^90^ (2020) (CORIMUNO-TOCI 1), open-label	IL-6R	Moderate to severe pneumonia	TCZ+SOC vs SOC	(1) % patients dead or needing NIV or mechanic ventilation on day 4 (scores>5 on WHO-CPS) and (2) survival without need of ventilation at day 14	
	Salvarani *et al*^93^ (2020) (RCT-TCZ-COVID-19), open-label		Mild pneumonia	TCZ+SOC vs SOC	Clinical worsening within 14 days‡	
	Stone *et al*^92^ (2020) (BACC Bay Tocilizumab Trial), phase III		Mild pneumonia	TCZ+SOC vs PBO+SOC	Mechanical ventilation or death (time frame: 28 days)	
	Salama *et al*^91^ (2020) (EMPACTA), phase III		Moderate to severe pneumonia	TCZ+SOC vs PBO+SOC	Mechanical ventilation or death by day 28	

Red denotes no difference comparted to placebo/control group; orange denotes promising results or rather mixed results across groups/trials and green denotes statistically superior compared with placebo or control group. For colorblind readers, table 2 is provided in the online supplemental appendix (section 7: S7.2).

*Response defined by combination of (1) decrease of at least 3 points in ESSDAI, (2) no new moderate/severe activity in any ESSDAI domain and (3) no worsening in physician’s global assessment on a visual numeric scale ≥1/10.

†Complete response (CR) based on European Group for Blood and Marrow Transplantation (EBMT) criteria

‡Defined by occurrence of 1 of the following events (whichever came first): (a) admission to intensive care unit with mechanical ventilation; (b) death or (c) paO_2_/FIO_2_ ratio <150 mm Hg.

ABMR, antibody-mediated rejection; ACR20, American College of Rheumatology 20% improvement criteria; ASAS20, Assessment in Ankylosing Spondylitis 20% response criteria; bDMARD, biological DMARD; BILAG, British Isles Lupus Assessment Group; CLZ, clazakizumab; csDMARD, conventional synthetic DMARD; DM, dermatomyositis; eGFR, estimated glomerular filtration rate; ESSDAI, EULAR Sjögren’s syndrome disease activity index; GC, glucocorticoids; ILD, interstitial lung disease; IR, inadequate response; mBICLA, modified BILAG-based Composite Lupus Assessment; MM, multiple myeloma; mRSS, modified Rodnan skin score; NIV, noninvasive ventilation; NSAID, non-steroidal anti-inflammatory drugs; %patients, proportion of patients; PBO, placebo; PM, polymyositis; PMR-AS, Polymyalgia Rheumatica (PMR) Activity Score; SAR, sarilumab; SIL, siltuximab; SIR, sirukumab; SLEDAI-2K, Systemic Lupus Erythematosus Disease Activity Index 2000; SOC, standard of care; SRI4, systemic lupus erythematosus (SLE) Responder Index (SRI); TCZ, tocilizumab; TNF-i, tumour necrosis factor inhibitor; VMP, bortezomib-melphalan-prednisone; WHO-CPS, World Health Organization-Clinical Progression Scale; ΔCDAI, change in Clinical Disease Activity Index; ΔFVC%, change in percentage of predicted forced vital capacity; ΔmRSS, change in modified Rodnan skin score; ΔSAA, change in serum amyloid A (SAA) levels; ΔVAS, change in visual analogue scale.

In this context, current evidence indicates that IL-6 blockade does not seem to be effective in psoriatic arthritis (PsA) or axial spondyloarthritis (axSpA). In a phase IIb, RCT in patients with active PsA, CLZ resolved musculoskeletal symptoms (arthritis, enthesitis and dactylitis) but did not improve skin manifestations.^6^ Currently, no further trials investigating the efficacy and appropriate dose of CLZ in PsA have been published. Also, TCZ and SAR failed to demonstrate therapeutic benefit in anti-TNF-naive patients with active axSpA.^7 8^ A phase III trial investigated the efficacy of TCZ in patients with hand osteoarthritis refractory to analgesics, exhibited no difference of TCZ over placebo to alleviate pain and improve function.^71^

Compounds specifically targeting IL-6 receptor or cytokine have undergone phase II or III clinical trials in patients with autoimmune diseases including systemic lupus erythematosus (SLE), primary Sjogren’s syndrome (pSS) and idiopathic inflammatory myopathy. Common to all these trials is the concept of targeting IL-6-mediated signalling did not show superiority in clinical responses versus placebo. In particular, neither PF-04236921 (a fully human antibody specifically binding IL-6 ligand) was superior to placebo in patients with SLE^9^ nor did sirukumab demonstrate any benefit in patients with active lupus nephritis and persistent proteinuria.^10^ Inhibiting IL-6R with vobarilizumab (ALX-0061) did not meet the primary endpoint of dose response in patients with active SLE.^72^ Likewise, TCZ failed to show clinical improvement in patients with refractory adult polymyositis and dermatomyositis^73^ as well as in patients with pSS with moderate or high systemic disease activity.^11^

Several studies investigating selective IL-6 pathway inhibition have not formally reached their primary endpoint but implied clinically meaningful benefits across several secondary outcomes. This includes TCZ in systemic sclerosis (SSc) associated interstitial lung disease (SSc-ILD). The primary endpoint of the phase III focuSSced trial was the difference in the mean change in modified Rodnan skin score at week 48. Despite the non-significant improvement of skin thickening as the primary outcome, change from baseline in forced vital capacity (FVC%) at week 48 favoured TCZ over placebo (difference in least squared mean FVC% change from baseline: 4.2; 95% CI 2.0 to 6.4; nominal p=0.0002), indicating a potentially beneficial effect of TCZ on preservation of lung function in SSc-ILD.^74^

The results of a phase II RCT of SIL in multiple myeloma (MM) did not show improved outcomes such as complete response, progression-free or overall survival in patients with untreated MM ineligible for high dose chemotherapy.^75^ SIL further failed to delay the transition from high-risk smouldering multiple myeloma to MM according to the prespecified protocol hypothesis criteria of increasing the 1-year progression-free survival rate by at least 14%.^76^

One phase II primarily safety powered RCT, evaluated the efficacy (secondary endpoint analysis) of clazakizumab versus placebo in late antibody-mediated kidney transplant rejection (ABMR). CLZ was associated with an early decrease in donor-specific antibody levels, modulated antibody-mediated rejection activity and delayed the decrease of renal function (eGFR decline: CLZ −0.96; placebo −2.43 mL/min per 1.73 m^2^ per month; p=0.04), suggesting a clinically meaningful effect in ABMR.^77^

Only limited data on IL-6 inhibition in amyloidosis are available. Two retrospective analyses compared the clinical utility of TCZ and anti-TNF therapy in patients with Amyloid A (AA) amyloidosis complicating rheumatic diseases.^78 79^ Compared with TNF-inhibitors, one study found TCZ superior in the suppression of serum-AA levels and improving renal function.^78^ Both studies indicated favourable results for TCZ, which needs to be confirmed in future randomised studies. However, given the retrospective design and therefore low hierarchy of evidence, efficacy of TCZ in amyloidosis was judged as ‘promising results’ (table 2).

TCZ has been effective in patients with polymyalgia rheumatica (PMR) according to case reports.^3^ This SLR yielded two reports on prospective, open-label phase II trials on TCZ emphasising its steroid-sparing effect^80^ as well as clinical and serological improvement in recent-onset PMR when used as monotherapy.^81^ In view of the high RoB of both studies and absence of a comparator arm in one report,^81^ results of phase III trials of TCZ (NCT03263715 and NCT02908217) and SAR (NCT03600818) are awaited as outcomes were not available in the public domain during the SLR’s time frame.

Finally, given the global crisis due to SARS-CoV-2 infection, causing the disease COVID-19 that is associated with heightened cytokine release including IL-6 and hyperinflammation,^82^ this SLR aimed to summarise the best available evidence on the use of agents selectively targeting IL-6 axis for the management of SARS-CoV-2 infection. A total of 11 studies at variable risk of bias, among them 4 RCTs, evaluating the therapeutic approach of TCZ or SAR in severe or critical COVID-19 were included (online supplemental table S3.1.14). For cohort studies (n=7), RoB was assessed using the validated NOS. Variability in inclusion criteria and therefore illness severity, study design and also size of the population enrolled, timepoint of treatment initiation, observation period, definition of primary endpoint and imbalances in the use of concomitant standard of care (SOC) treatment or rather steroids could affect the interpretation and comparability of the results. Most of the studies included moderately to severely ill patients with hyperinflammatory state requiring oxygen support. Treatment with TCZ was associated with lower hazards regarding intubation or death in two retrospective cohort studies.^83 84^ In case of requirement of intensive care unit (ICU) support for critically ill patients, three retrospective observational studies demonstrated positive effects of TCZ on hospital-related mortality.^85–87^ Furthermore, one prospective study with a historical control group investigated the efficacy of high-dose intravenous methylprednisolone (MP) with or without TCZ in hyperinflamed COVID-19 patients with rapid respiratory deterioration and requiring any oxygen support. The historical control group received supportive care only (no steroids) and in the intervention group, and TCZ was added if respiratory condition had not improved sufficiently through MP. Treatment with TCZ demonstrated a benefit of MP and TCZ concerning clinical improvement in respiratory status, likelihood to evolve to IMV, duration of hospitalisation and mortality.^88^ Conversely, one small prospective trial failed to show any mortality benefit for SAR.^89^ The promising results of observational studies led to execution of randomised clinical trials; four randomised trials (including two phase III trials) which, however, excluded critically ill patients, were available (table 2). The CORIMUNO-TOCI I trial, in patients requiring at least 3 L/min oxygen therapy but not admitted to the ICU, reported reduced risk of non-invasive ventilation, IMV or death at day 14, when TCZ was added to SOC. However, no difference was found between groups concerning day-28 mortality.^90^ A similar trial, the EMPACTA trial, a RCT assessing the efficacy of TCZ in ethnic minority populations, showed that TCZ+SOC was more efficacious than SOC alone in reducing a composite outcome of IMV or death, but no reduction in day-28 mortality was observed.^91^ Two additional randomised trails failed to provide any benefit of treatment strategies including TCZ on disease progression and other efficacy outcomes, respectively.^92 93^

### Safety

In total, 66 publications addressing the safety profile of bDMARDs targeting IL-6R or IL-6 ligand were included in this SLR. Safety data derived mainly from observational studies, such as cohort studies and real-word registries arising from TCZ in patients with RA and JIA, whereas for compounds with no registry data available (eg, SAR and sirukumab), randomised controlled trials and LTEs were applied as primary source of information. Characteristics of each individual article included, namely, inclusion and exclusion criteria, RoB and safety outcomes for each intervention group were extracted in detail and are listed in section 4 in the online supplemental appendix grouped according to the outcome of interest.

#### Cardiovascular events and venous thromboembolism

Six observational studies and one RCT addressing cardiovascular (CV) events and venous thromboembolism (VTE) were included (online supplemental table S4.1.1.1–S4.1.6.2). Overall, the risk of major adverse cardiac events (MACE), myocardial infarction, stroke or rather transient ischaemic attack, heart failure and coronary revascularisation was not increased in patients with RA receiving TCZ compared with those on TNFi, abatacept or rituximab.^94–100^ In ENTRACTE, a randomised controlled head-to-head trial assessing the CV safety of TCZ and etanercept (ETN) in patients with RA, for fatal and nonfatal stroke, 26 events occurred in the TCZ group (n=1538) compared with 16 events in the ETN treatment arm (n=1542). A total of 83 MACE occurred in TCZ recipients during follow-up compared with 78 in the ETN group. The estimated hazard ratio (HR) for occurrence of MACE in the TCZ group relative to the ETN group was 1.05 (95% CI 0.77–1.43). Additionally, risks of developing VTE (deep vein thrombosis and pulmonary embolism) were evaluated in ENTRACTE, which reported only a small number of events and no increased risk for TCZ compared with ETN-treated patients.^97^

#### Infections

In total, 15 observational studies investigated the risk of infections in patients receiving TCZ compared with those treated with TNF-inhibitors or non-TNF bDMARDs including abatacept and rituximab (see online supplemental tables S4.3.1.1–S4.3.5.3 for details of included reports). IL-6 inhibition with TCZ was associated with an increased risk of hospitalisation for overall serious bacterial infections (SI) compared with non-treated patients, which, however, was in line with rates seen with other bDMARDs.^101–106^ TCZ was found to have a significantly higher rate of SI compared with ETN in both the adjusted and unadjusted models (HR for adjusted models: 1.21, 95% CI 1.01 to 1.46) in a British observational cohort study.^107^ Similarly, in the above-mentioned ENTRACTE trial, infections (any infections and serious infections) were more frequently observed in the TCZ- than in the ETN-arm (HR for serious infections 1.39, 95% CI 1.08 to 1.79).^97^ An increased risk of certain types of serious infections (septicaemia, pneumonia, other upper respiratory tract and skin infections) was observed in TCZ versus TNF-i users in two cohort studies. Of note is, that these observations could not be consistently replicated in other studies and the estimates showed significant variability.^107 108^ Also, according to Pawar *et al*, the risk of composite serious infections was higher in TCZ initiators compared with abatacept initiators (pooled HR 1.40, 95% CI 1.20 to 1.63). However, composite risk of TCZ for serious infections requiring hospitalisation for infectious AE was comparable to TNF-i.^108^ Current evidence suggests that TCZ does not show an increased risk for herpes zoster, opportunistic infections or tuberculosis in comparison to TNF-inhibition or ABA.^108–114^

#### Malignancies

Data from four large RA observational studies suggested no increased risk of TCZ in total occurrence of malignant neoplasms, nor did TCZ show any signal of a higher risk for specific cancer types (including invasive melanoma and lymphoma) in comparison with csDMARDs, other bDMARDs or the general population.^115–118^ Details on studies investigating risks of malignancies are depicted in online supplemental tables S4.4.1.1–S4.4.6.3.

#### Gastrointestinal and hepatic events

The risk for diverticulitis and any gastrointestinal (GI) infections was investigated in three cohort studies (high RoB: n=1; low RoB: n=1; conference abstract: n=1; for details on baseline characteristics, RoB assessment and safety outcomes (see online supplemental tables S4.5.1.1–S4.5.1.3). One study reported an elevated risk of diverticulitis during TCZ treatment compared with TNF inhibitors (HR 2.34, 95% CI 1.64 to 3.34).^108^ Higher rates of any GI infections were observed with TCZ compared with ETN as reference compound in another study (HR 1.45, 95% CI 0.72 to 2.90).^107^ Similarly, a conference abstract assessed the risk of diverticulitis in patients with RA: three prospective observational French registries, confirmed an elevated risk of diverticulitis for TCZ compared with RTX-treated or ABA-treated patients.^119^

Events of GI perforations, in particular diverticular perforations as complications of diverticulitis have been increasingly reported in patients with RA with TCZ exposure.^3^ The overall risk of gastrointestinal perforations (GIP) including both the upper and lower GI tract was investigated in five cohort studies including populations with long-term exposure of TCZ (low RoB: n=1; high RoB: n=3; conference abstracts: n=1). GI perforations that required hospitalisation were significantly more frequent among TCZ than among TNF-inhibitors, RTX and ABA. In the majority of cases, the lower GI tract was involved.^119 120^ Overall incidence rates for lower GI perforations (LIP) under TCZ treatment were markedly increased compared with csDMARDs, TNF-inhibitors, RTX^121^ or the general population.^122^ Two of the included studies were further adjusted for concomitant treatment with GC and NSAIDs^120 121^ and two for history of diverticulitis.^120 123^ Diverticulitis itself was more often associated with perforation in TCZ exposed patients than in patients treated with other agents.^119 121^ Likewise in the ENTRACTE trial, eight confirmed GI perforation events occurred in the TCZ arm, compared with one event in the ETN arm. The estimated HR for occurrence of GI-perforations in the TCZ group relative to the ETN group was 8.43 (95% CI 1.06–67.26), accepting event numbers were small.^97^ Additionally, one observational study indicated a pronounced LIP risk for both TCZ (HR 2.55, 95% CI 1.33 to 4.88) and tofacitinib treatment, when compared with TNF-i (HR 3.24, 95% CI 1.05 to 10.04). However, incidence of upper tract GIP remained comparable between TCZ and other bDMARDs in the same study.^123^ Study details are shown in online supplemental tables S4.5.2.1–S4.5.2.3. Table 3 provides a summary of the data comparing GI perforation risk of TCZ versus other DMARDs stratified by any, upper and lower perforation.

**Table 3:** Safety outcomes in observational studies regarding GIP: comparison between TCZ and other bDMARDs/csDMARDs, tsDMARDs and general population

Study	Treatment group	N patients	N events	Incidence rate (95% CI)	aHR (I vs C)	
Monemi *et al* (2016)*^120^	Combined TNF-i (ADA, ETN, IFX)	17 333	10	0.6/1000 PY (0.3 to 1.2)	REF	REF
	TCZ	3602	6	1.8/1000 PY (0.7 to 4.0)	2.2 (0.7 to 6.6)	2.2 (0.9 to 5.4)
Monemi *et al* (2016)†^120^	Combined TNF-i (ADA, ETN, IFX)	17 333	6	0.4/1000 PY (0.1 to 0.8)	REF	REF
	TCZ	3602	5	1.5/1000 PY (0.5 to 3.6)	4.0 (1.1 to 14.1)	3.1 (1.1 to 8.4)
Rempenault *et al* (2019) (EULAR 2020)*^119^	TCZ	1496	9	2.3/1000 PY	TCZ vs RTX: 2.8 (1.5 to 5.1) TCZ vs ABA: 5.4 (1.4 to 19.9)	
	RTX	1986	8	1.3/1000 PY		
	ABA	1019	2	0.8/1000 PY		
Rempenault *et al* (2019) (EULAR 2020)§^119^	TCZ	1496	6	1.5/1000 PY	TCZ vs RTX: 3.8 (1.7 to 8.5) TCZ vs ABA: 6.9 (1.9 to 25.4)	
	RTX	1986	3	0.5/1000 PY		
	ABA	1019	2	0.8/1000 PY		
Rempenault *et al* (2019) (EULAR 2020)¶ 119	TCZ	1496	3	0.7/1000 PY	TCZ vs RTX: 1.4 (0.5 to 3.9) TCZ vs ABA: NAP	
	RTX	1986	5	0.8/1000 PY		
	ABA	1019	0	–		
Strangfeld *et al* (2017)†^121^	csDMARD	4423	11	0.61/1000 PY (0.3 to 1.1)	REF	
	TNF-i	6711	13	0.52/1000 PY (0.3 to 0.9)	1.04 (0.48 to 2.26)	
	TCZ	877	11	2.69/1000 PY (1.4 to 4.8)	4.48 (2.01 to 9.99)	
	other bDMARDs (RTX+ABA)	NR	NR	NR	0.33 (0.08 to 1.44)	
Barbulescu *et al* (2020)†^122^	General population	76 304	333	1.07/1000 PY (0.95 to 1.33)	REF	NAP
	Bionaïve patients with RA	62 532	570	1.60/1000 PY (1.46 to 1.74)	1.02	NAP
	TNF-i	17 594	57	1.84/1000 PY (1.38 to 3.63)	0.99	REF
	ABA	2527	13	3.32/1000 PY (1.66 to 16.6)	1.41	1.07 (0.55 to 2.10)
	RTX	3552	22	2.02/1000 PY (1.26 to 5.65)	1.07	0.89 (0.50 to 1.58)
	TCZ	2377	22	4.51/1000 PY (2.68 to 10.4)	2.36	2.20 (1.28 to 3.79)
Xie *et al* (2016)*^123^	Combined TNF-i	115 044	109	0.83/1000 PY (0.69 to 1.00)	NR	
	TCZ	11 705	16	1.55/1000 PY (0.95 to 2.54)	NR	
	TOFA	4755	3	0.86/1000 PY (0.10 to 3.60)	NR	
Xie *et al* (2016)†^123^	Combined TNF-i	115 044	59	0.46/1000 PY (0.35 to 0.58)	REF	
	ABA	31 214	30	0.76/1000 PY (0.53 to 1.09)	1.41 (0.90 to 2.21)	
	RTX	4391	2	0.48/1000 PY (0.06 to 1.75)	1.72 (0.52 to 5.69)	
	TCZ	11 705	13	1.26/1000 PY (0.73 to 2.18)	2.55 (1.33 to 4.88)	
	TOFA	4755	2	0.86/1000 PY (0.10 to 3.60)	3.24 (1.05 to 10.04)	
Xie *et al* (2016)‡^123^	Combined TNF-i	115 044	49	0.38/1000 PY (0.28 to 0.50)	NR	
	ABA	31 214	12	0.31/1000 PY (0.17 to 0.54)	NR	
	RTX	4391	1	0.24/1000 PY (0.01 to 1.35)	NR	
	TCZ	11 705	3	0.29/1000 PY (0.06 to 0.85)	NR	
	TOFA	4755	0	0.00/1000 PY (0.00 to 1.58)	NR	

Detailed results are shown in online supplemental tables S4.5.2.1–S4.5.2.3.

*Any GIP.

†Lower GIP.

‡Upper GIP.

§GIP due to diverticulitis (diverticular GIP).

¶GIP due to another aetiology.

ABA, abatacept; ADA, adalimumab; aHR, adjusted HR; bDMARD, biological DMARD; C, control; CI, confidence interval; csDMARD, conventional synthetic DMARD; DMARD, disease-modifying antirheumatic drug; ETN, etanercept; GIP, gastrointestinal perforations; I, intervention; IFX, infliximab; N, number; NAP, not applicable; NR, not reported; PY, patient years; REF, reference; RTX, rituximab; TCZ, tocilizumab; TNF-i, tumour necrosis factor inhibitor; TOFA, tofacitinib; tsDMARD, target synthetic DMARD.

The risk of hepatic events was investigated in two studies (moderate RoB: n=1; high RoB: n=1) (for details, see online supplemental tables S4.5.3.1–S4.5.3.4). Hepatic transaminase elevations with TCZ were frequent, but rates of hepatic severe AEs were low in a clinical trial setting. Of note, transaminase elevations were observed more frequently when TCZ was combined with MTX/DMARD compared with monotherapy.^124^ A postmarketing study from Japan investigated patients who had a history of hepatitis B/C virus or were virus carriers—no patient experienced a viral reactivation (with or without hepatitis) after exposure to TCZ.^125^

#### Vaccination

In total, five studies (all with high RoB) addressed the safety and efficacy of vaccination during TCZ therapy (online supplemental tables S4.2.1.1–S4.2.1.3). Overall, IL-6 inhibition with TCZ did not hamper antibody response to influenza, pneumococcal and tetanus toxoid vaccines in patients with RA.^126–128^ Similarly, safety and efficacy of influenza vaccination did not differ significantly between patients with sJIA and healthy controls.^129^ One study reported a negative impact of MTX on pneumococcal antibody response, when TCZ was used in combination with MTX in patients with RA.^130^

#### Adverse events of special interest

Several studies investigating AEs) of special interest could be included (details shown in online supplemental tables S4.6.1.1–S4.6.11.9). For completeness, all RCTs with a valid comparator and LTEs assessing the safety aspects of SAR and sirukumab were evaluated (online supplemental tables S4.6.10.1–S4.6.10.11). In terms of the other IL-6 receptor or ligand targeting compounds without observational data available, the overall safety profile or incidence of major AEs in all clinical trials was comparable to that of to TCZ.

Studies addressing the risk of withdrawals due to AEs and immunological reactions reported results in line with the available evidence of TCZ and other bDMARDs;^131–136^ however, one study showed a significantly higher discontinuation rate due to AEs in elderly patients with RA receiving TCZ compared with those treated with ABA. Nonetheless, apart from TCZ, several TNF inhibitors comprising ADA, ETN, golimumab and infliximab were associated with significantly higher discontinuation rate by AEs compared with ABA.^137^

As inhibition of the IL-6 axis is associated with higher risk for dyslipidaemia, two studies investigated the effect of TCZ on lipid-associated CV risk markers, reporting that median total-cholesterol, low-density lipoprotein-cholesterol and triglyceride levels increased in TCZ treatment arms when compared with placebo and ADA. Notably, TCZ modified high-density lipoprotein particles changed towards an anti-inflammatory composition and favourably altered some other vascular risk surrogates, such as lipoprotein (a).^138 139^ The overall net effect on CV risk of TCZ is therefore difficult to predict based on established or novel risk factor changes alone. For this reason, the results of the ENTRACTE trial, suggesting no increased risk of MACE with TCZ versus ETN, are reassuring.^97^

Moreover, TCZ showed no risk of worsening diabetes status in terms of therapy intensification and switching to insulin in comparison to other bDMARDs.^140^ Consistent results were observed with SAR in a posthoc analysis, where SAR was associated with a greater reduction in glycosylated haemoglobin (HbA1c) than placebo or ADA in patients with diabetes.^141^

Safety analyses of TCZ on haematological variables found significant increases in haemoglobin and haematocrit levels in anaemic and non-anaemic patients with RA compared with other biological and non-biological DMARDs. By contrast, TCZ-treated patients had higher rates of grade 1/2 or 3/4 neutropenia than placebo; however, neutropenia was not associated with serious infections and improved after dose reduction or discontinuation of TCZ.^142 143^

A stable safety and tolerability profile of TCZ in patients with RA was additionally reported in patients exhibiting renal insufficiency, regardless of MTX usage.^144^ No safety signal could be identified regarding the risk of ILD and its related complications,^145^ idiopathic facial nerve palsies^146^ or osteoporotic fractures.^147^

Only limited data regarding pregnancy outcomes were available. One safety database including clinical trials and postmarketing data as well as case series did not suggest a higher risk for malformations after exposure to TCZ shortly before conception or early pregnancy.^148 149^ Nevertheless, an increased rate of preterm birth (31.2%) was observed in TCZ users compared with the general population. No signal of adverse pregnancy outcomes was reported in the same analysis with paternal exposure to TCZ in 13 pregnancies.^148^ Overall, considering the currently insufficient evidence base, it cannot be suggested to continue TCZ therapy in case of pregnancy.

Safety data in terms of SAR were retrieved from three RCTs and one LTE, concluding comparable safety and tolerability profiles to TCZ without new signals emerging (online supplemental tables S4.6.10.1–S4.6.10.6).^28 150–152^

In contrast, numerical differences in mortality were observed in sirukumab-treated patients compared with control arms^34 153^ (for details, see online supplemental tables S4.6.10.7–S4.6.10.11). CV events, infections and malignancies were the underlying causes of deaths. In 2017, the FDA declined the approval of SIR for RA demanding ancillary clinical data for further safety evaluation.^154^ LTE safety outcomes of SAR and SIR are summarised in table 4.

**Table 4:** LTEs investigating the safety profile of SAR and SIR

Study	Trial	Treatment group	N patients	Any serious AEs Events: n (%)	Serious infections Events: n (%)	GI-perforations Events: n (%)	Any major CVE Events: n (%)	Any malignancy Events: n (%)	Deaths of any cause Events: n (%)
Fleischmann *et al*^152^	MOBILITY TARGET ASCERTAIN ONE COMPARE EASY EXTEND	SAR 150/200/100 mg Q2W or SAR 100/150 mg QW+DMARD	2887	685 (23.7)	232 (8.0)	9 (0.3)	41 (1.4)	52 (1.8)	31 (1.1)
		SAR Mono	471	52 (11.0)	7 (1.5)	0	2 (0.4)	4 (0.8)	5 (1.1)
Thorne *et al*^153^	SIRROUND-D (2 years)	Placebo	556	40 (7.2)	11 (2.0)	1 (0.2)	1 (0.2)	2 (0.4)	1 (0.2)
		SIR 50 mg Q4W	798	141 (17.7)	58 (7.3)	3 (0.4)	13 (1.6)	8 (1.0)	10 (1.3)
		SIR 100 mg Q2W	799	132 (16.5)	47 (5.9)	1 (0.1)	5 (0.6)	12 (1.5)	11 (1.4)
		SIR combined	1597	273 (17.1)	105 (6.6)	4 (0.3)	18 (1.1)	20 (1.3)	21 (1.3)

Detailed results are shown in online supplemental tables S4.6.10.4–S4.6.10.6 and S4.6.10.10–S4.6.10.11.

AE, adverse events; CVE, cardiovascular event; DMARD, disease-modifying antirheumatic drug; GIP, gastrointestinal perforations; LTEs, long-term extension studies; mono, monotherapy; N, number; Q2W, every other week; Q4W, every 4 weeks; QW, every week; SAR, sarilumab; SIR, sirukumab.

In JIA, this SLR presents the pertinent evidence from observational studies suggesting no increased risk of hospitalisation due to overall serious bacterial infections, tuberculosis, malignancies, GI perforations, hepatic events or rare events such as demyelination through TCZ as opposed to TNF-i and other bDMARDs. In addition, studies addressing the risk of withdrawals due to AEs and incidence of macrophage activation syndrome reported comparable results between TCZ and bDMARDs.^155–157^ Study outcomes are detailed in online supplemental tables S4.6.11.1–S4.6.11.9.

### Biomarkers for prediction of therapeutic response

A total of 20 reports (including three conference abstracts) investigating the applicability of predictors of treatment success and clinical outcome of bDMARDs selectively targeting the IL-6 pathway were included. Different variables including CRP,^158^ serum IL-6 concentration,^159–162^ baseline anticitrullinated peptide antibody status,^163^ type I interferon signalling and metallothionein proteins^164^ as well as cellular and synovial,^165–167^ cardiac,^168–171^ genetic markers,^172 173^ parameters of bone metabolism^174^ and body mass index^175–177^ were evaluated as possible predictors of clinical outcomes. Investigated biomarkers and detailed results are listed in online supplemental table S5.1 and S5.2, respectively. Current data suggest, to some extent, a certain association for different markers with clinical response. This includes low pretreatment IL-6 levels—which were predictive for response to TCZ or to sustained success after its cessation.^159 160^ Apart from that, a high pretreatment CRP level was indicative of good treatment responses in patients receiving TCZ.^158^ Data regarding obesity and treatment response, on the other hand, were more controversial.^175–177^ Generally, the quality of currently available evidence is considered as being low and further studies are required to confirm the validity of these analyses.

### Patient adherence/ preferences and economic aspects

Eleven studies on patient perspectives were included (online supplemental appendix table S6.1). Treatment with TCZ resulted in meaningful improvements of patient-reported outcomes (PROs), such as pain, physical function, activity impairment and quality of life.^178 179^ In RCTs, TCZ and SAR monotherapy yielded greater improvements across multiple PROs than csDMARD or TNF-i (ADA).^180 181^ Among patients with RA who had previously received ≥1 bDMARD, TCZ treated patients showed a similar or significantly better biological persistence than those receiving other bDMARDs.^182^ Moreover, fatigue and physical function significantly improved in TCZ treated GCA-patients compared with those receiving steroids only.^183^ In JIA, patients switching route of administration from intravenous to SC reported satisfaction in terms of life quality, school success and reduced school absenteeism.^184^ On the other side, in patients with RA, TCZ discontinuation was predicted by low initial CRP levels, high scale on the Health Assessment Questionnaire (HAQ), high fatigue and pain, smoking and exposure to bDMARD before TCZ therapy.^185 186^ Finally, five studies addressing the cost-effectiveness related to the usage of TCZ and SAR were included. Details of economic analyses and outcomes are shown in online supplemental appendix table S6.2. Overall, data on adjusted indirect comparison and cost-effectiveness suggested better results for TCZ and SAR compared with other bDMARDs, although these results should be interpreted with caution as quality of evidence is low.

## Discussion

With this SLR, we have obtained an update of the emerging evidence on agents that selectively target the IL-6 pathway by including new data from 2012 onwards. In retrospect, the scope of indications, both licensed and off-label, has extended over the past few years, and the availability of large-scale observational studies made it possible to gain a deep insight into long-term safety. Efficacy has been confirmed across various inflammatory diseases, especially in rheumatic diseases such as RA, JIA, AoSD, GCA, TAK and SSc-LD. Especially in RA, new data allowed us to refine the use of TCZ and other agents. Important findings in this SLR are the clinical efficacy of several novel compounds targeting both the IL-6 receptor (SAR) and IL-6 ligand (olokizumab, sirukumab) in patients with RA. Consistent efficacy results led to the approval of SAR as the second IL-6R blocker for RA in 2017. The humanised IL-6 ligand blocker OKZ is currently licensed for the treatment of RA in Russia only. Sirukumab was ultimately not approved by the FDA in 2017 due to safety concerns. Also, based on results from strategic studies, it can be concluded that in patients who are not suitable for csDMARD combination therapy, IL-6 pathway inhibitors may have some advantages over TNF-inhibitors. TCZ monotherapy may be a legitimate option in RA; however, trends for a worse clinical outcome and a more pronounced radiographic progression in the monotherapy group were observed when compared with combination therapy with MTX. Tapering concomitant MTX or steroids was possible in patients who were in sustained low disease activity or remission, but nevertheless constitutes a risk of flare.

In addition, TCZ was used to successfully treat CRS due to T cell therapy, and newcomers like SAT and the licensed anti-IL-6 ligand antibody SIL have expanded the therapeutic approaches for Castleman’s disease and neuromyelitis optica, respectively. Targeting IL-6 in several diseases (including PsA, axSpA or connective tissue diseases, except for ILD in SSc) was not effective in several clinical trials. In COVID-19, TCZ-treated critically ill patients showed a decreased mortality in subgroup analyses. Noteworthy is that the authorisations of TCZ for treatment of hospitalised adults and paediatric patients with severe acute respiratory syndrome coronavirus 2 pneumonia and SSc-associated interstitial lung disease (ILD), respectively, were effectuated by the U.S. Food and Drug Administration (FDA), but this happened after the last literature search update of this SLR.^187 188^ Likewise, in December 2021, TCZ received rapid European Commission approval for the treatment of COVID-19 in adults treated with systemic corticosteroids and requiring supplemental oxygen or mechanical ventilation.^189^ TCZ demonstrated better clinical outcomes and decreased mortality in severely ill patients, particularly those had received previous dexamethasone treatment.^190 191^ Meanwhile, treating critically ill patients with COVID-19 with either TCZ or baricitinib (a Janus Kinase inhibitor) after failing dexamethasone therapy has become standard of care.

Safety data deriving from observational studies allowed to gain further insights into long-term safety, especially focusing on less frequent or rare AEs. The risk of cardiac events, VTE or malignancy did not differ from other bDMARDs and csDMARDs. Observational studies suggested a potential increased risk for certain infections such as skin infections as well as diverticulitis in TCZ versus TNF-i treated patients. Observational studies on patients receiving TCZ showed that the risk of diverticulitis lower GI perforations is overall low, but higher compared with other bDMARDs and in line with previously published reports. Properly designed studies on other IL-6 blocking agents are required to study whether this increased risk of GI perforations is specific to TCZ or a potential class effect.

This SLR has several limitations: (1) only one researcher (KK) evaluated all retrieved publications by title and abstract screening for eligibility and assessed the risk of bias; however, whenever a question of uncertainty arose, the paper was discussed with the methodologist (AK); (2) due to the heterogeneity of the available studies, no pooling of efficacy or safety outcomes by meta-analysis were performed; (3) safety analyses are mainly based on observational studies on TCZ in patients with RA and JIA, limiting the interpretability of the safety profile with regard to other populations and other bDMARDs selectively targeting IL-6 receptor or cytokine.

The results of this SLR were presented to inform the consensus statement on efficacy and safety of pharmacological treatment with IL-6 pathway inhibition with biological DMARDs in immune-mediated inflammatory diseases.

## Data Availability

All data relevant to the study are included in the article or uploaded as supplementary information.
