# Supplementary material for: A systematic literature review informing the consensus statement on efficacy and safety of pharmacological treatment with interleukin-6 pathway inhibition with biological DMARDs in immune-mediated inflammatory diseases
Source: RMD Open. 2022 Sep 7;8(2):e002359. doi: 10.1136/rmdopen-2022-002359 (PMC9462104; doi:10.1136/rmdopen-2022-002359)
Supplement: Supplementary data [file rmdopen-2022-002359supp001.pdf]

A systematic literature review informing the consensus statement on efficacy and safety of pharmacological treatment with interleukin-6 pathway inhibition with biological DMARDs

Online Supplementary appendix

Section 2: Characteristics of articles and abstracts included: Efficacy for approved indications

Section 3: Characteristics of articles and abstracts included: Efficacy for other studied diseases

Section 4: Characteristics of articles and abstracts included: Safety aspects of interleukin-6 pathway inhibition

Section 5: Characteristics of articles and abstracts included:
Biomarkers for prediction of therapeutic response of interleukin-6 pathway inhibition

Section 6: Characteristics of articles and abstracts included: Patient adherence/preferences and economic aspects of interleukin-6 pathway inhibition

Section 7: Figures and tables for colorblind persons

Section 8: References

Kastrati K, *et al. RMD Open* 2022; 8:e002359. doi: 10.1136/rmdopen-2022-002359

Table of contents

Section 2: Characteristics of articles and abstracts included: Efficacy for approved indications 15

2.1. Details of articles and abstracts selected for inclusion 15

Table S2.1.1: Rheumatoid arthritis (RA) 15

Table S2.1.2: Systemic juvenile idiopathic arthritis (sJIA) 18

Table S2.1.3: Polyarticular-course juvenile idiopathic arthritis (pcJIA) 18

Table S2.1.4: Adult-onset Still's disease (AoSD) 18

Table S2.1.5: Giant cell arteritis (GCA) 19

Table S2.1.6: Takayasu arteritis (TAK) 20

Table S2.1.7: Multicentric Castleman´s disease (MCD) 20

Table S2.1.8: CAR-T cell induced Cytokine Release Syndrome (CRS) 20

Table S2.1.9: Neuromyelitis optica spectrum disorders (NMOSD) 21

2.2. Risk of bias analysis 22

Table S2.2.1: Rheumatoid arthritis (RA) 22

Table S2.2.2: Systemic juvenile idiopathic arthritis (sJIA) 26

Table S2.2.3: Polyarticular-course juvenile idiopathic arthritis (pcJIA) 26

Table S2.2.4: Adult-onset Still's disease (AoSD) 27

Table S2.2.5: Giant cell arteritis (GCA) 27

Table S2.2.6: Takayasu arteritis (TAK) 28

Table S2.2.7: Multicentric Castleman´s disease (MCD) 28

Table S2.2.8: CAR-T cell induced Cytokine Release Syndrome (CRS) 29

Table S2.2.9: Neuromyelitis optica spectrum disorders (NMOSD) 29

2.3. Baseline characteristics 30

2.3.1: Rheumatoid arthritis (RA) 30

Table S2.3.1.1: Baseline characteristics of trials investigating IL-6R/L blockers + MTX or csDMARDs versus placebo in patients with inadequate response or intolerance to MTX or csDMARDs 30

Table S2.3.1.2: Baseline characteristics of trials investigating IL-6R/L blockers in patients with inadequate response or intolerance to TNF-inhibitors. 33

Table S2.3.1.3: Baseline characteristics of trials investigating IL-6R/L blockers in patients with inadequate response or intolerance to csDMARDs or TNF-inhibitors. 34

Table S2.3.1.4: Baseline characteristics of trials investigating IL-6R/L blockers vs. other bDMARDs (Head-to-Head trials). 35

Table S2.3.1.5: Switch studies. Part 1: Baseline characteristics of trials investigating (switching) route of administration and dosage adaptation of IL-6R/L blockers. 36

Table S2.3.1.6: Switch studies. Part 2: Baseline characteristics of trials investigating add-on versus switching to IL-6R blockers. 37

Table S2.3.1.7: Switch studies. Part 3: Baseline characteristics of trials investigating switching to another IL-6R blocker. 38

Table S2.3.1.8: Induction/Strategic studies. Part 1: Baseline characteristics of trials comparing the effectiveness of IL-6R blocker monotherapy and combination therapy with MTX in early RA. 39

Table S2.3.1.9: Induction/Strategic studies. Part 2: Baseline characteristics of trials comparing the effectiveness of IL-6R blocker + MTX with conventional treatment in early RA. 40

Table S2.3.1.10: Tapering studies. Part 1: Baseline characteristics of trials investigating tapering of csDMARDs while on IL-6R blocker therapy. 41

Table S2.3.1.11: Tapering studies. Part 2: Baseline characteristics of trials investigating tapering of glucocorticoids while on IL-6R blocker therapy. 42

Table S2.3.1.12: Tapering studies. Part 3: Baseline characteristics of trials investigating tapering of IL-6R blockers. 42

2.3.2: Systemic juvenile idiopathic arthritis (sJIA) 44

Table S2.3.2.1: Baseline characteristics of trials investigating IL-6R/L blockers in sJIA. 44

2.3.3: Polyarticular-course juvenile idiopathic arthritis (pcJIA) 45

Table S2.3.3.1: Baseline characteristics of trials investigating IL-6R/L blockers in pcJIA. 45

2.3.4: Adult-onset Still’s disease (AoSD) 47

Table S2.3.4.1: Baseline characteristics of trials investigating IL-6R/L blockers in AoSD. 47

2.3.5: Giant cell arteritis (GCA) 48

Table S2.3.5.1: Baseline characteristics of trials investigating IL-6R/L blockers in GCA. 48

2.3.6: Takayasu arteritis (TAK) 50

Table S2.3.6.1: Baseline characteristics of trials investigating IL-6R/L blockers in TAK. 50

2.3.7: Multicentric Castleman´s disease (MCD) 51

Table S2.3.7.1: Baseline characteristics of trials investigating IL-6R/L blockers in MCD. 51

2.3.8: CAR-T cell induced Cytokine Release Syndrome (CRS) 52

Table S2.3.8.1: Baseline characteristics of trials investigating IL-6R/L blockers in CRS. 52

2.3.9: Neuromyelitis optica spectrum disorders (NMOSD) 53

Table S2.3.9.1: Baseline characteristics of trials investigating IL-6R/L blockers in NMOSD. 53

2.4. Efficacy outcomes 54

2.4.1: Rheumatoid arthritis (RA) 54

Table S2.4.1.1: Efficacy outcomes of trials investigating IL-6R/L blockers + MTX or csDMARDs versus placebo in patients with inadequate response or intolerance to MTX or csDMARDs. 54

Table S2.4.1.2: Efficacy outcomes of trials investigating IL-6R/L blockers in patients with inadequate response or intolerance to TNF-inhibitors. 56

Table S2.4.1.3: Efficacy outcomes of trials investigating IL-6R/L blockers in patients with inadequate response or intolerance to csDMARDs or TNF-inhibitors. 58

Table S2.4.1.4: Efficacy outcomes of trials investigating IL-6R/L blockers vs. other bDMARDs (Head-to-Head trials). 59

Table S2.4.1.5: Switch studies. Part 1: Efficacy outcomes of trials investigating (switching) route of administration and dosage adaptation of IL-6R/L blockers. 60

Table S2.4.1.6: Switch studies. Part 2: Efficacy outcomes of trials investigating add-on versus switching to IL-6R blockers. 61

Table S2.4.1.7: Switch studies. Part 3: Efficacy outcomes of trials investigating switching to another IL-6R blocker. 63

Table S2.4.1.8: Induction/Strategic studies. Part 1: Efficacy outcomes of trials comparing the effectiveness of IL-6R blocker monotherapy and combination therapy with MTX in early RA. 64

Table S2.4.1.9: Induction/Strategic studies. Part 2: Efficacy outcomes of trials comparing the effectiveness of IL-6R blocker + MTX with conventional treatment in early RA. 66

Table S2.4.1.10: Tapering studies. Part 1: Efficacy outcomes of trials investigating tapering of csDMARDs while on IL-6R blocker therapy. 66

Table S2.4.1.11: Tapering studies. Part 2: Efficacy outcomes of trials investigating tapering of glucocorticoids while on IL-6R blocker therapy. 68

Table S2.4.1.12: Tapering studies. Part 3: Efficacy outcomes of trials investigating tapering of IL-6R blockers. 69

2.4.2: Systemic juvenile idiopathic arthritis (sJIA) 71

Table S2.4.2.1: Efficacy outcomes of trials investigating IL-6R/L blockers in sJIA. 71

2.4.3: Polyarticular-course juvenile idiopathic arthritis (pcJIA) 72

Table S2.4.3.1: Efficacy outcomes of trials investigating IL-6R/L blockers in pcJIA 72

2.4.4: Adult-onset Still’s disease (AoSD) 73

Table S2.4.4.1: Efficacy outcomes of trials investigating IL-6R/L blockers in AoSD. 73

2.4.5: Giant cell arteritis (GCA) 74

Table S2.4.5.1: Efficacy outcomes of trials investigating IL-6R/L blockers in GCA. 74

2.4.6: Takayasu arteritis (TAK) 76

Table S2.4.6.1: Efficacy outcomes of trials investigating IL-6R/L blockers in TAK 76

2.4.7: Multicentric Castleman´s disease (MCD) 77

Table S2.4.7.1: Efficacy outcomes of trials investigating IL-6R/L blockers in MCD. 77

2.4.8: CAR-T cell induced Cytokine Release Syndrome (CRS) 78

Table S2.4.8.1: Efficacy outcome of trials investigating IL-6R/L blockers in CRS. 78

2.4.9: Neuromyelitis optica spectrum disorders (NMOSD) 79

Table S2.4.9.1: Efficacy outcomes of trials investigating IL-6R/L blockers in NMOSD. 79

Section 3: Characteristics of articles and abstracts included: Efficacy for other studied diseases 81

3.1. Details of articles and abstracts selected for inclusion 81

Table S3.1.1: Psoriatic arthritis (PsA) 81

Table S3.1.2: Axial spondyloarthritis (axSpA) 81

Table S3.1.3: Osteoarthritis (OA) 82

Table S3.1.4: Polymyalgia rheumatica (PMR) 82

Table S3.1.5: ANCA-associated vasculitis (GPA, MPA) 83

Table S3.1.6: Remitting seronegative symmetric synovitis with pitting edema (RS3PE) 83
Table S3.1.7: Systemic sclerosis associated interstitial lung disease (SSc-ILD) 84
Table S3.1.8: Idiopathic inflammatory myopathies (IIM) 84
Table S3.1.9: Systemic lupus erythematosus (SLE) 85
Table S3.1.10: Primary Sjögren's syndrome (pSS) 86
Table S3.1.11: Amyloid A (AA)- Amyloidosis (AAA) 86
Table S3.1.12: Multiple Myeloma (MM) 87
Table S3.1.13: Refractory relapsing polychondritis 88
Table S3.1.14: Cytokine release syndrome CRS (associated with severe SARS-CoV-2 infection, Macrophage activating syndrome) 89
Table S3.1.15: Tumor necrosis factor receptor-associated periodic fever syndrome (TRAPS) 92
Table S3.1.16: Chronic infantile neurological cutaneous and articular syndrome (CINCA) 92
Table S3.1.17: Late antibody-mediated kidney transplant rejection (ABMR) 92
3.2. Risk of bias analysis 93
Table S3.2.1: Psoriatic arthritis (PsA) 93
Table S3.2.2: Axial spondyloarthritis (axSpA) 93
Table S3.2.3: Osteoarthritis (OA) 94
Table S3.2.4: Polymyalgia rheumatica (PMR) 94
Table S3.2.5: ANCA-associated vasculitis (GPA, MPA) 95
Table S3.2.6: Remitting seronegative symmetric synovitis with pitting edema (RS3PE) 95
Table S3.2.7: Systemic sclerosis associated interstitial lung disease (SSc-ILD) 96
Table S3.2.8: Idiopathic inflammatory myopathies (IIM) 96
Table S3.2.9: Systemic lupus erythematosus (SLE) 97
Table S3.2.10: Primary Sjögren's syndrome (pSS) 97
Table S3.2.11: Amyloid A (AA)-Amyloidosis (AAA) 98
Table S3.2.12: Multiple Myeloma (MM) 98
Table S3.2.13: Refractory relapsing polychondritis 99
Table S3.2.14.1: Cytokine release syndrome CRS (associated with severe SARS-CoV-2 infection, Macrophage activating syndrome): Cohort studies/historically controlled comparison 99
Table S3.2.14.2: Cytokine release syndrome CRS (associated with severe SARS-CoV-2 infection, Macrophage activating syndrome): RCTs 100
Table S3.2.15: Tumor necrosis factor receptor-associated periodic fever syndrome (TRAPS) 101
Table S3.2.16: Chronic infantile neurological cutaneous and articular syndrome (CINCA) 101
Table S3.2.17: Late antibody-mediated kidney transplant rejection (ABMR) 102
3.3. Baseline characteristics 103
3.3.1: Psoriatic arthritis (PsA) 103
Table S3.3.1.1: Baseline characteristics of trials investigating IL-6R/L blockers in PsoA. 103

3.3.2: Axial spondyloarthritis (axSpA) 104

Table S3.3.2.1: Baseline characteristics of trials investigating IL-6R/L blockers in axSpA. 104

3.3.3: Osteoarthritis (OA) 105

Table S3.3.3.1: Baseline characteristics of trials investigating IL-6R/L blockers in OA. 105

3.3.4: Polymyalgia rheumatica (PMR) 106

Table S3.3.4.1: Baseline characteristics of trials investigating IL-6R/L blockers in PMR. 106

3.3.5: ANCA-associated vasculitis (GPA, MPA) 107

Table S3.3.5.1: Baseline characteristics of trials investigating IL-6R/L blockers in ANCA-associated vasculitis. 107

3.3.6: Remitting seronegative symmetric synovitis with pitting edema (RS3PE) 108

Table S3.3.6.1: Baseline characteristics of trials investigating IL-6R/L blockers in RS3PE. 108

3.3.7: Systemic sclerosis associated interstitial lung disease (SSc-ILD) 109

Table S3.3.7.1: Baseline characteristics of trials investigating IL-6R/L blockers in SSc-ILD. 109

3.3.8: Idiopathic inflammatory myopathies (IIM) 110

Table S3.3.8.1: Baseline characteristics of trials investigating IL-6R/L blockers in IIM. 110

3.3.9: Systemic lupus erythematosus (SLE) 111

Table S3.3.9.1: Baseline characteristics of trials investigating IL-6R/L blockers in SLE. 111

3.3.10: Primary Sjögren's syndrome (pSS) 112

Table S3.3.10.1: Baseline characteristics of trials investigating IL-6R/L blockers in pSS. 112

3.3.11: Amyloid A (AA)-Amyloidosis (AAA) 113

Table S3.3.11.1: Baseline characteristics of trials investigating IL-6R/L blockers in AAA. 113

3.3.12: Multiple Myeloma (MM) 114

Table S3.3.12.1: Baseline characteristics of trials investigating IL-6R/L blockers in MM. 114

3.3.13: Refractory relapsing polychondritis 115

Table S3.3.13.1: Baseline characteristics of trials investigating IL-6R/L blockers in refractory relapsing polychondritis. 115

3.3.13: Cytokine release syndrome CRS (associated with severe SARS-CoV-2 infection, Macrophage activating syndrome) 116

Table S3.3.14.1: Baseline characteristics of trials investigating IL-6R/L blockers in CRS (associated with severe SARS-CoV-2 infection, Macrophage activating syndrome): Cohort studies I 116

Table S3.3.14.2: Baseline characteristics of trials investigating IL-6R/L blockers in CRS (associated with severe SARS-CoV-2 infection, Macrophage activating syndrome): Cohort studies II 117

Table S3.3.14.3: Baseline characteristics of trials investigating IL-6R/L blockers in CRS (associated with severe SARS-CoV-2 infection, Macrophage activating syndrome): Cohort studies III 117

Table S3.3.14.4: Baseline characteristics of trials investigating IL-6R/L blockers in CRS (associated with severe SARS-CoV-2 infection, Macrophage activating syndrome): Cohort studies IV 118

Table S3.3.14.5: Baseline characteristics of trials investigating IL-6R/L blockers in CRS (associated with severe SARS-CoV-2 infection, Macrophage activating syndrome): Cohort studies V 118

Table S3.3.14.6: Baseline characteristics of trials investigating IL-6R/L blockers in CRS (associated with severe SARS-CoV-2 infection, Macrophage activating syndrome): Cohort studies VI 119

Table S3.3.14.7: Baseline characteristics of trials investigating IL-6R/L blockers in CRS (associated with severe SARS-CoV-2 infection, Macrophage activating syndrome): Historically controlled comparison 119

Table S3.3.14.8: Baseline characteristics of trials investigating IL-6R/L blockers in CRS (associated with severe SARS-CoV-2 infection, Macrophage activating syndrome): RCTs 120

3.3.15: Tumor necrosis factor receptor-associated periodic fever syndrome (TRAPS) 121

Table S3.3.15.1: Baseline characteristics of trials investigating IL-6R/L blockers in TRAPS. 121

3.3.16: Chronic infantile neurological cutaneous and articular syndrome (CINCA) 122

Table S3.3.16.1: Baseline characteristics of trials investigating IL-6R/L blockers in CINCA. 122

3.3.17: Late antibody-mediated kidney transplant rejection (ABMR) 123

Table S3.3.17.1: Baseline characteristics of trials investigating IL-6R/L blockers in ABMR. 123

3.4. Efficacy outcomes 124

3.4.1: Psoriatic arthritis (PsA) 124

Table S3.4.1.1: Efficacy outcomes of trials investigating IL-6R/L blockers in PsoA. 124

3.4.2: Axial spondyloarthritis (axSpA) 125

Table S3.4.2.1: Efficacy outcomes of trials investigating IL-6R/L blockers in axSpA. 125

3.4.3: Osteoarthritis (OA) 126

Table S3.4.3.1: Efficacy outcomes of trials investigating IL-6R/L blockers in OA. 126

3.4.4: Polymyalgia rheumatica (PMR) 127

Table S3.4.4.1: Efficacy outcomes of trials investigating IL-6R/L blockers in PMR. 127

3.4.5: ANCA-associated vasculitis (GPA, MPA) 128

Table S3.4.5.1: Efficacy outcomes of trials investigating IL-6R/L blockers in ANCA-associated vasculitis. 128

3.4.6: Remitting seronegative symmetric synovitis with pitting edema (RS3PE) 129

Table S3.4.6.1: Efficacy outcomes of trials investigating IL-6R/L blockers in RS3PE. 129

3.4.7: Systemic sclerosis associated interstitial lung disease (SSc-ILD) 130

Table S3.4.7.1: Efficacy outcomes of trials investigating IL-6R/L blockers in SSc-ILD. 130

3.4.8: Idiopathic inflammatory myopathies (IIM) 131

Table S3.4.8.1: Efficacy outcomes of trials investigating IL-6R/L blockers in IIM. 131

3.4.9: Systemic lupus erythematosus (SLE) 132

Table S3.4.9.1: Efficacy outcomes of trials investigating IL-6R/L blockers in SLE. 132

3.4.10: Primary Sjögren’s syndrome (pSS) 133

Table S3.4.10.1: Efficacy outcomes of trials investigating IL-6R/L blockers in pSS. 133

3.4.11: Amyloid A (AA)-Amyloidosis (AAA) 134

Table S3.4.11.1: Efficacy outcomes of trials investigating IL-6R/L blockers in AAA. 134

3.4.12: Multiple Myeloma (MM) 135

Table S3.4.12.1: Efficacy outcomes of trials investigating IL-6R/L blockers in MM. 135

3.4.13: Refractory relapsing polychondritis 136

Table S3.4.13.1: Efficacy outcomes of trials investigating IL-6R/L blockers in refractory relapsing polychondritis. 136

3.4.14: Cytokine release syndrome CRS (associated with severe SARS-CoV-2 infection, Macrophage activating syndrome) 137

Table S3.4.14.1: Efficacy outcome of trials investigating IL-6R/L blockers in CRS (associated with severe SARS-CoV-2 infection, Macrophage activating syndrome): Cohort studies I 137

Table S3.4.14.2: Efficacy outcome of trials investigating IL-6R/L blockers in CRS (associated with severe SARS-CoV-2 infection, Macrophage activating syndrome): Cohort studies II 138

Table S3.4.14.3: Efficacy outcome of trials investigating IL-6R/L blockers in CRS (associated with severe SARS-CoV-2 infection, Macrophage activating syndrome): Cohort studies III 138

Table S3.4.14.4: Efficacy outcome of trials investigating IL-6R/L blockers in CRS (associated with severe SARS-CoV-2 infection, Macrophage activating syndrome): Cohort studies IV 139

Table S3.4.14.5: Efficacy outcome of trials investigating IL-6R/L blockers in CRS (associated with severe SARS-CoV-2 infection, Macrophage activating syndrome): Cohort studies V 140

Table S3.4.14.6: Efficacy outcome of trials investigating IL-6R/L blockers in CRS (associated with severe SARS-CoV-2 infection, Macrophage activating syndrome): Cohort studies VI 141

Table S3.4.14.7: Efficacy outcomes of trials investigating IL-6R/L blockers in CRS (associated with severe SARS-CoV-2 infection, Macrophage activating syndrome): Historically controlled comparison 142

Table S3.4.14.8: Efficacy outcomes of trials investigating IL-6R/L blockers in CRS (associated with severe SARS-CoV-2 infection, Macrophage activating syndrome): RCTs 143

3.4.15: Tumor necrosis factor receptor-associated periodic fever syndrome (TRAPS) 146

Table S3.4.15.1: Efficacy outcomes of trials investigating IL-6R/L blockers in TRAPS......................... 146

3.4.16: Chronic infantile neurological cutaneous and articular syndrome (CINCA)............................ 147

Table S3.4.16.1: Efficacy outcomes of trials investigating IL-6R/L blockers in CINCA......................... 147

3.4.17: Late antibody-mediated kidney transplant rejection (ABMR) 148

Table S3.4.17.1: Efficacy outcomes of trials investigating IL-6R/L blockers in ABMR. 148

Section 4: Characteristics of articles and abstracts included: Safety aspects of interleukin-6 pathway inhibition 149

4.1. Cardiovascular events.. 149

4.1.1: Composite Outcome (MACE): Comparison between IL-6R/L blockers and different bDMARDs (observational studies/randomized controlled trials).. 149

Table S4.1.1.1: Baseline characteristics of observational studies investigating IL-6R/L blockers regarding MACE (major adverse cardiac events). 149

Table S4.1.1.2: Risk of bias analysis (Newcastle-Ottawa Scale [NOS] for Cohort studies).................. 150

Table S4.1.1.3: Safety outcomes of observational studies regarding MACE....................................... 151

Table S4.1.1.4: Baseline characteristics of RCTs investigating IL-6R/L blockers regarding MACE (major adverse cardiac events) 152

Table S4.1.1.5: Risk of bias analysis (Cochrane Risk of Bias Tool for RCTs).. 152

Table S4.1.1.6: Safety outcomes of RCTs regarding MACE. 153

4.1.2: Myocardial infarction: Comparison between IL-6R/L blockers and different bDMARDs (observational studies/randomized controlled trials)... 154

Table S4.1.2.1: Baseline characteristics of observational studies investigating IL-6R/L blockers regarding myocardial infarction. 154

Table S4.1.2.2: Risk of bias analysis (Newcastle-Ottawa Scale [NOS] for Cohort studies)................. 155

Table S4.1.2.3: Safety outcomes of observational studies regarding myocardial infarction. 155

Table S4.1.2.4: Baseline characteristics of RCTs investigating IL-6R/L blockers regarding myocardial infarction. 157

Table S4.1.2.5: Safety outcomes of RCTs regarding MACE. 157

4.1.3: Stroke/Transient ischemic attack: Comparison between IL-6R/L blockers and different bDMARDs (observational studies/randomized controlled trials) 158

Table S4.1.3.1: Baseline characteristics of observational studies investigating IL-6R/L blockers regarding stroke/transient ischemic attack. 158

Table S4.1.3.2: Safety outcomes of observational studies regarding stroke/transient ischemic attack. 159

Table S4.1.3.3: Baseline characteristics of RCTs investigating IL-6R/L blockers regarding stroke/transient ischemic attack... 160

Table S4.1.3.4: Safety outcomes of RCTs regarding stroke/transient ischemic attack. 160

4.1.4: Heart failure: Comparison between IL-6R/L blockers and different bDMARDs (observational studies/randomized controlled trials).. 161

Table S4.1.4.1: Baseline characteristics of observational studies investigating IL-6R/L blockers regarding heart failure. 161

Table S4.1.4.2: Safety outcomes of observational studies regarding heart failure. 161

Table S4.1.4.3: Baseline characteristics of RCTs investigating IL-6R/L blockers regarding stroke/transient ischemic attack... 162

Table S4.1.4.4: Safety outcomes of RCTs regarding stroke/transient ischemic attack. 162

4.1.5: Coronary Revascularisation: Comparison between IL-6R/L blockers and different bDMARDs (observational studies/randomized controlled trials)... 163

Table S4.1.5.1: Baseline characteristics of observational studies investigating IL-6R/L blockers regarding coronary revascularization.. 163

Table S4.1.5.2: Safety outcomes of observational studies regarding coronary revascularization. 163

4.1.6: Venous thromboembolism (VTE): Comparison between IL-6R/L blockers and different bDMARDs (randomized controlled trials) 164

Table S4.1.6.1: Baseline characteristics of RCTs investigating IL-6R/L blockers regarding VTE. 164

Table S4.1.6.2: Safety outcomes of RCTs regarding VTE. 164

4.2. Vaccination 165

4.2.1: Vaccination: Comparison between IL-6R/L blockers and different b/csDMARDs (clinical trials) 165

Table S4.2.1.1: Included clinical trials investigating IL-6R/L blockers regarding antibody response after vaccination.......... 165

Table S4.2.1.2: Risk of bias analysis.......... 166

Table S4.2.1.3: Outcomes of clinical trials investigating IL-6R/L blockers regarding antibody response after vaccination.......... 167

4.3. Infections.......... 170

4.3.1: Serious infections: Comparison between IL-6R/L blockers and different bDMARDs (observational studies/randomized controlled trials).......... 170

Table S4.3.1.1: Baseline characteristics of observational studies investigating IL-6R/L blockers regarding serious infections. 170

Table S4.3.1.2: Risk of bias analysis (Newcastle-Ottawa Scale [NOS] for Cohort studies).......... 171

Table S4.3.1.3: Safety outcomes of observational studies regarding serious infections. 172

Table S4.3.1.4.1: Safety outcomes of observational studies regarding subtypes of serious infections. 176

Table S4.3.1.4.2: Safety outcomes of observational studies regarding subtypes of serious infections. 177

4.3.2: Opportunistic infections: Comparison between IL-6R/L blockers and different bDMARDs (observational studies/randomized controlled trials).......... 178

Table S4.3.2.1: Baseline characteristics of observational studies investigating IL-6R/L blockers regarding opportunistic infections. 178

Table S4.3.2.2: Risk of bias analysis (Newcastle-Ottawa Scale [NOS] for Cohort studies).......... 179

Table S4.3.2.3: Safety outcomes of observational studies regarding opportunistic infections. 179

4.3.3: Tuberculosis: Comparison between IL-6R/L blockers and different bDMARDs (observational studies/randomized controlled trials).......... 181

Table S4.3.3.1: Baseline characteristics of observational studies investigating IL-6R/L blockers regarding tuberculosis. 181

Table S4.3.3.2: Risk of bias analysis (Newcastle-Ottawa Scale [NOS] for Cohort studies).......... 182

Table S4.3.3.3: Safety outcomes of observational studies regarding tuberculosis. 183

4.3.4: Pneumocystis jirovecii pneumonia: Comparison between IL-6R/L blockers and different bDMARDs (observational studies/randomized controlled trials) 185

Table S4.3.4.1: Baseline characteristics of observational studies investigating IL-6R/L blockers regarding pneumocystis jirovecii pneumonia. 185

Table S4.3.4.2: Safety outcomes of observational studies regarding pneumocystis jirovecii pneumonia. 185

4.3.5: Herpes zoster: Comparison between IL-6R/L blockers and different bDMARDs (observational studies/randomized controlled trials).......... 186

Table S4.3.5.1: Baseline characteristics of observational studies investigating IL-6R/L blockers regarding herpes zoster.......... 186

Table S4.3.5.2: Risk of bias analysis (Newcastle-Ottawa Scale [NOS] for Cohort studies).......... 187

Table S4.3.5.3: Safety outcomes of observational studies regarding herpes zoster. 187

4.4. Malignancies ... 190

4.4.1: All types of cancer: Comparison between IL-6R/L blockers and different bDMARDs (observational studies/randomized controlled trials) ... 190

Table S4.4.1.1: Baseline characteristics of observational studies investigating IL-6R/L blockers regarding all types of cancer (excluding NMSC). ... 190

Table S4.4.1.2: Risk of bias analysis (Newcastle-Ottawa Scale [NOS] for Cohort studies) ... 191

Table S4.4.1.3: Safety outcomes of observational studies regarding all types of cancer (invasive solid or hematologic malignant neoplasm excluding NMSC). ... 191

4.4.2: Invasive solid cancer: Comparison between IL-6R/L blockers and different bDMARDs (observational studies/randomized controlled trials) ... 193

Table S4.4.2.1: Baseline characteristics of observational studies investigating IL-6R/L blockers regarding invasive solid cancer. ... 193

Table S4.4.2.2: Safety outcomes of observational studies regarding invasive solid cancer. ... 193

4.4.3: Invasive hematologic cancer: Comparison between IL-6R/L blockers and different bDMARDs (observational studies/randomized controlled trials) ... 194

Table S4.4.3.1: Baseline characteristics of observational studies investigating IL-6R/L blockers regarding invasive hematologic cancer. ... 194

Table S4.4.3.2: Safety outcomes of observational studies regarding invasive hematologic cancer ... 195

4.4.4: Non-Hodgkin's Lymphoma: Comparison between IL-6R/L blockers and different bDMARDs (observational studies/randomized controlled trials) ... 196

Table S4.4.4.1: Baseline characteristics of observational studies investigating IL-6R/L blockers regarding Non-Hodgkin's Lymphoma. ... 196

Table S4.4.4.2: Risk of bias analysis (Newcastle-Ottawa Scale [NOS] for Cohort studies) ... 196

Table S4.4.4.3: Safety outcomes of observational studies regarding Non-Hodgkin's Lymphoma. ... 197

4.4.5: Non melanoma skin cancer (NMSC): Comparison between IL-6R/L blockers and different bDMARDs (observational studies/randomized controlled trials) ... 198

Table S4.4.5.1: Baseline characteristics of observational studies investigating IL-6R/L blockers regarding NMSC. ... 198

Table S4.4.5.2: Safety outcomes of observational studies regarding NMSC. ... 199

4.4.6: Melanoma: Comparison between IL-6R/L blockers and different bDMARDs (observational studies/randomized controlled trials) ... 200

Table S4.4.6.1: Baseline characteristics of observational studies investigating IL-6R/L blockers regarding invasive melanoma. ... 200

Table S4.4.6.2: Risk of bias analysis (Newcastle-Ottawa Scale [NOS] for Cohort studies) ... 201

Table S4.4.6.3: Safety outcomes of observational studies regarding invasive melanoma. ... 201

4.5. Gastrointestinal and hepatic events ... 203

4.5.1: Diverticulitis: Comparison between IL-6R/L blockers and different bDMARDs (observational studies/randomized controlled trials) ... 203

Table S4.5.1.1: Baseline characteristics of observational studies investigating IL-6R/L blockers regarding diverticulitis. ... 203

Table S4.5.1.2: Risk of bias analysis (Newcastle-Ottawa Scale [NOS] for Cohort studies)................. 204

Table S4.5.1.3: Safety outcomes of observational studies regarding diverticulitis. 204

4.5.2: Gastrointestinal perforation (GIP): Comparison between IL-6R/L blockers and different bDMARDs (observational studies/randomized controlled trials) 206

Table S4.5.2.1: Baseline characteristics of observational studies investigating IL-6R/L blockers regarding GIP. 206

Table S4.5.2.2: Risk of bias analysis (Newcastle-Ottawa Scale [NOS] for Cohort studies)................. 207

Table S4.5.2.3: Safety outcomes of observational studies regarding GIP. 208

4.5.3: Hepatic events: 213

Table S4.5.3.1: Baseline characteristics of observational studies investigating IL-6R/L blockers regarding hepatic events. 213

Table S4.5.3.2: Risk of bias analysis (Newcastle-Ottawa Scale [NOS] for Cohort studies)................. 214

Table S4.5.3.3: Safety outcomes of observational studies regarding hepatic events I....................... 214

Table S4.5.3.4: Safety outcomes of observational studies regarding hepatic events (transaminase elevations) II 215

4.6. Adverse events of special interest... 216

4.6.1: Withdrawal and immunologic events: Comparison between IL-6R/L blockers and different bDMARDs (observational studies/randomized controlled trials) 216

Table S4.6.1.1: Safety outcomes of observational studies investigating IL-6R/L blockers regarding withdrawals. 216

Table S4.6.1.2: Safety outcomes of observational studies investigating IL-6R/L blockers regarding immunologic events. 218

4.6.2: Changes in lipid profile: Comparison between IL-6R/L blockers and different bDMARDs (observational studies/randomized controlled trials).. 219

Table S4.6.2.1: Baseline characteristics of RCTs investigating IL-6R/L blockers regarding changes in lipid profile.. 219

Table S4.6.2.2: Outcomes of RCTs investigating IL-6R/L blockers regarding changes in lipid profile. 219

4.6.3: Diabetes and changes in HbA1c: Comparison between IL-6R/L blockers and different bDMARDs (observational studies/randomized controlled trials).. 221

Table S4.6.3.1: Safety outcomes of observational studies investigating IL-6R/L blockers regarding risk of diabetes treatment intensification and switching to insulin. 221

Table S4.6.3.2: Baseline characteristics of post hoc analyses investigating effects of IL-6R/L blockers vs. TNF-i on glycosylated hemoglobin (HbA1c) in patients with RA and subgroups of patients with RA and diabetes 221

Table S4.6.3.3: Outcomes of post hoc analyses investigating effects of IL-6R/L blockers vs. TNF-i on glycosylated hemoglobin (HbA1c) in patients with RA and subgroups of patients with RA and diabetes. 222

4.6.4: Effects on anemia and risk of neutropenia: Comparison between IL-6R/L blockers and different bDMARDs (observational studies/randomized controlled trials) 223

Table S4.6.4.1: Safety outcomes of observational studies investigating IL-6R/L blockers regarding changes in hemoglobin in patients with anemia at index date. 223

Table S4.6.4.2: Safety outcomes of observational studies investigating IL-6R/L blockers regarding changes in neutrophils and neutropenia associated risk of infection.. 224

4.6.5: Renal insufficiency: Comparison between IL-6R/L blockers and different bDMARDs (observational studies/randomized controlled trials)... 225

Table S4.6.5.1: Safety outcomes of observational studies investigating IL-6R/L blockers in patients with RA and renal insufficiency 225

4.6.6: Interstitial lung disease (ILD): Comparison between IL-6R/L blockers and different bDMARDs (observational studies/randomized controlled trials)... 227

Table S4.6.6.1: Safety outcomes of observational studies investigating IL-6R/L blockers regarding risk of ILD and is complications.. 227

4.6.7: Neurological AEs: Comparison between IL-6R/L blockers and different bDMARDs (observational studies/randomized controlled trials)... 228

Table S4.6.7.1: Safety outcomes of observational studies investigating IL-6R/L blockers regarding risk of idiopathic facial nerve palsy.. 228

4.6.8: Bone mineral density and osteoporosis: Comparison between IL-6R/L blockers and different bDMARDs (observational studies/randomized controlled trials) 229

Table S4.6.8.1: Safety outcomes of observational studies investigating IL-6R/L blockers regarding risk of osteoporotic fracture and other subtypes of fractures. 229

Table S4.6.8.2: Outcomes of prospective studies investigating effects of IL-6R/L blockers on bone mineral density... 230

4.6.9: Pregnancy: Clinical trials and post-marketing data.. 231

Table S4.6.9.1: Pregnancy outcome after exposure to IL-6R inhibition... 231

Table S4.6.9.2: Pregnancy outcome after exposure to IL-6R inhibition... 232

4.6.10: Randomized controlled trials (RCTs) and long-term extension studies (LTEs)........................ 232

Table S4.6.10.1: Sarilumab: Overview of RCTs... 232

Table S4.6.10.2: Sarilumab: Rates of serious AEs, serious infections, opportunistic infections, deaths, malignancies and CVE (RCTs).. 233

Table S4.6.10.3: Sarilumab: Rates of GIP, injection-site / infusion reactions, immunogenicity and neutropenia (RCTs)... 234

Table S4.6.10.4: Sarilumab: Rates of serious AEs, deaths, malignancies and CVE (LTE).................... 235

Table S4.6.10.5: Sarilumab: Rates of serious infections, opportunistic infections, serious demyelinating disorders and VTE (LTE)... 236

Table S4.6.10.6: Sarilumab: Rates of GIP, injection-site / infusion reactions, immunogenicity, neutropenia and hepatic disorders (LTE). 236

Table S4.6.10.7: Sirukumab: Overview of RCTs... 237

Table S4.6.10.8: Sirukumab: Rates of serious AEs, serious infections, opportunistic infections, deaths, malignancies and CVE (RCTs).. 238

Table S4.6.10.9: Sirukumab: Rates of GIP, injection-site / infusion reactions, immunogenicity, neutropenia and hepatic disorders (RCTs).. 239

Table S4.6.10.10: Sirukumab: Rates of serious AEs, serious infections, opportunistic infections, deaths, malignancies and CVE (LTE).. 240

Table S4.6.10.11: Sirukumab: Rates of GIP, injection-site / infusion reactions, immunogenicity, neutropenia and hepatic disorders (LTE). 240

4.6.11: Juvenile idiopathic arthritis (JIA): Comparison between IL-6R/L blockers and different bDMARDs (observational studies/randomized controlled trials) 241

Table S4.6.11.1: Safety outcomes of observational studies investigating IL-6R/L blockers in JIA regarding serious AEs. 241

Table S4.6.11.2: Safety outcomes of observational studies investigating IL-6R/L blockers in JIA regarding serious infections. 242

Table S4.6.11.3: Safety outcomes of observational studies investigating IL-6R/L blockers in JIA regarding malignancies. 243

Table S4.6.11.4: Safety outcomes of observational studies investigating IL-6R/L blockers in JIA regarding GIP. 244

Table S4.6.11.5: Safety outcomes of observational studies investigating IL-6R/L blockers in JIA regarding hepatic events. 244

Table S4.6.11.6: Safety outcomes of observational studies investigating IL-6R/L blockers in JIA regarding demyelination. 245

Table S4.6.11.7: Safety outcomes of observational studies investigating IL-6R/L blockers in JIA regarding tuberculosis. 246

Table S4.6.11.8: Safety outcomes of observational studies investigating IL-6R/L blockers in JIA regarding withdrawals. 247

Table S4.6.11.9: Safety outcomes of observational studies investigating IL-6R/L blockers in JIA regarding macrophage activation syndrome (MAS). 247

Section 5: Characteristics of articles and abstracts included: Biomarkers for prediction of therapeutic response of interleukin-6 pathway inhibition. 249

Table S5.1: Overview of included studies. 249

Table S5.2: Outcomes of studies investigating biomarkers for prediction of therapeutic response of interleukin-6 pathway inhibition. 250

Section 6: Characteristics of articles and abstracts included: Patient adherence/preferences and economic aspects of interleukin-6 pathway inhibition. 256

Table S6.1: Outcomes of studies investigating patient adherence and preferences in patients treated with IL-6R/L blockers. 256

Table S6.2: Outcomes of studies investigating economic aspects of treatment with IL-6R/L blockers. 261

Section 7: Figures and tables for colorblind persons 264

Figure S7.1: Efficacy of biological disease modifying anti-rheumatic drugs targeting the IL-6 receptor or ligand and their relative efficacy and or regulatory approvals 264

Table S7.2: Efficacy outcomes of clinical trials published from 2012 to 2020 investigating biologic disease modifying antirheumatic drugs (bDMARDs) specifically inhibiting IL-6 receptor or ligand compared against placebo or control group, shown across other studied immune-mediated diseases. 265

Section 8 References 266

Section 2: Characteristics of articles and abstracts included: Efficacy for approved indications

2.1. Details of articles and abstracts selected for inclusion

Table S2.1.1: Rheumatoid arthritis (RA)

**Study**	**Treatment**	**Target**	**Population**
Huizinga 2014 (MOBILITY Part A) (1)	Sarilumab	IL-6R	MTX-IR
Genovese 2015 (MOBILITY Part B) (2)	Sarilumab	IL-6R	MTX-IR
Tanaka 2019 (KAKEHASI) (3)	Sarilumab	IL-6R	MTX-IR
Mazurov 2020 (AURORA, 1-year) (4)	Levilimab (BCD-089)	IL-6R	MTX-IR
NCT02309359 (not published) (5)	Vobarilizumab (ALX-0061)	IL-6R	MTX-IR
NCT02287922 (not published) (6)	Vobarilizumab (ALX-0061)	IL-6R	MTX-IR
Nasonov 2020 (CREDO-1) (7)	Olokizumab	IL-6	MTX-IR
Mease 2012 (8)	Clazakizumab (BMS945429/ALD518)	IL-6	MTX-IR
Baek 2019 (9)	Tocilizumab	IL-6R	csDMARD
NCT00773461 (not published) (10)	Tocilizumab	IL-6R	csDMARD
Takeuchi 2017 (SIRROUND-D) (11)	Sirukumab	IL-6	csDMARD
Fleischmann 2017 (TARGET) (12)	Sarilumab	IL-6R	TNFi-IR
Takeuchi 2016 (RA0083) (13)	Olokizumab	IL-6	TNFi-IR

Aletaha 2017 (SIRROUND-T) (14)	Sirukumab	IL-6	TNFi-IR
Genovese 2014 (15)	Olokizumab	IL-6	TNFi-IR
Yazici 2012 (ROSE) (16)	Tocilizumab	IL-6R	cs-/bDMARD-IR (mixed)
Kivitz 2014 (BREVACTA) (17)	Tocilizumab	IL-6R	cs-/bDMARD-IR (mixed)
NCT00977106 (TORPEDO, not published) (18)	Tocilizumab	IL-6R	cs-/bDMARD-IR (mixed)
Gabay 2013 (ADACTA) (19)	Tocilizumab vs. Adalimumab	IL-6R vs. TNF	MTX-IR
Burmester 2017 (MONARCH) (20)	Sarilumab vs. Adalimumab	IL-6R vs. TNF	MTX-IR
Taylor 2018 (SIRROUND-H) (21)	Sirukumab vs. Adalimumab	IL-6 vs. TNF	MTX-IR
Weinblatt 2015 (22)	Clazakizumab (Adalimumab + MTX as active reference)	IL-6 (TNF+MTX as active reference)	MTX-IR
Burmester 2014 (SUMMACTA) (23)	Tocilizumab SC vs. Tocilizumab IV	IL-6R	cs-/bDMARD-IR
Ogata 2014 (MUSASHI) (24)	Tocilizumab SC vs. Tocilizumab IV	IL-6R	cs-/bDMARD-IR
Ogata 2015 (MUSASHI-OLE) (25)	Tocilizumab IV/SC vs. Tocilizumab SC/SC	IL-6R	MUSASHI:<br>TCZ-SC or TCZ-IV mono
Ogata 2018 (SHINOBI) (26)	Tocilizumab QW vs. Tocilizumab Q2W	IL-6R	TCZ-SC Q2W-IR
Dougados 2013 (ACT-RAY) (27)	Tocilizumab + MTX vs. Tocilizumab mono	IL-6R	MTX-IR
Dougados 2014 (ACT-RAY, 1-year) (28)	Tocilizumab + MTX vs. Tocilizumab mono	IL-6R	ACT-RAY, prespecified exploratory analyses (up to week 52)
Kaneko 2016 (SURPRISE) (29)	Tocilizumab + MTX vs. Tocilizumab mono	IL-6R	MTX-IR
Emery 2020 (EXTEND, OLE) (30)	Switching from Tocilizumab (or Sarilumab 150mg SC) to, or continuing, Sarilumab 200 mg SC Q2W	IL-6R	TNFi-IR, concom. csDMARD

Burmester 2016 (FUNCTION) (31)	Tocilizumab + MTX vs. Tocilizumab vs. MTX	IL-6R	MTX naïve, early RA
Burmester 2017 (FUNCTION, 2-years) (32)	Tocilizumab + MTX vs. Tocilizumab vs. MTX	IL-6R	MTX naïve, early RA
Bijlsma 2016 (U-ACT-EARLY) (33)	Tocilizumab + MTX vs. Tocilizumab vs. MTX	IL-6R	DMARD naïve, early RA
Hetland 2020 (NORD-STAR) (34)	MTX+ active conventional treatment vs. Tocilizumab + MTX vs. Abatacept + MTX vs. Certolizumab + MTX	IL-6R vs. TNF vs. CD-80/CD-86	treatment-naïve, early RA
Edwards 2018 (ACT-TAPER) (35)	Tocilizumab + MTX tapering vs. Tocilizumab + MTX continuation	IL-6R	DMARD-IR (bDMARD-naïve)
Kremer 2018 (COMP-ACT) (36)	Tocilizumab + MTX discontinuation vs. Tocilizumab + MTX continuation	IL-6R	MTX-IR
Pablos 2019 (JUST-ACT) (37)	Tocilizumab + MTX discontinuation vs. Tocilizumab + MTX continuation	IL-6R	MTX-IR (bDMARD- naïve)
Peterfy 2020 (COMP-ACT MRI Substudy) (38)	Tocilizumab + MTX discontinuation vs. Tocilizumab + MTX continuation	IL-6R	MTX-IR
Burmester 2020 (SEMIRA) (39)	Tocilizumab + GC tapering vs. Tocilizumab + GC continuation	IL-6R	TCZ SC/IV ± (cs)DMARD and GC
Huizinga 2015 (ACT-RAY, 2 and 3-years) (40)	Discontinuation of Tocilizumab + csDMARD/MTX	IL-6R	Tocilizumab + MTX (add-on) vs. Tocilizumab mono (switch)
Kaneko 2018 (SURPRISE, 2-years) (41)	After Tocilizumab discontinuation: MTX vs. no DMARD	IL-6R	Tocilizumab + MTX (add-on) vs. Tocilizumab mono (switch)
Kedra 2019 (TOLEDO) (42)	Tocilizumab (or Abatacept) maintenance vs. Tocilizumab (or Abatacept) progressive injection spacing up to discontinuation	IL-6R, CD-80/CD-86	ABA or TCZ for ≥ 1 year (mono +/-csDMARD, GC)

Table S2.1.2: Systemic juvenile idiopathic arthritis (sJIA)

Study	Treatment	Target	Population
De Benedetti 2012 (TENDER) (43)	Tocilizumab	IL-6R	NSAID-IR, GC-IR;
Malattia 2020 (44)	Tocilizumab	IL-6R	s/pc-JIA: TENDER/CHERISH

Table S2.1.3: Polyarticular-course juvenile idiopathic arthritis (pcJIA)

Study	Treatment	Target	Population
Brunner 2015 (CHERISH) (45)	Tocilizumab	IL-6R	MTX-IR
Malattia 2020 (44)	Tocilizumab	IL-6R	s/pc-JIA: TENDER/CHERISH

Table S2.1.4: Adult-onset Still’s disease (AoSD)

Study	Treatment	Target	Population
Kaneko 2018 (46)	Tocilizumab	IL-6R	GC-IR

Kastrati K, *et al. RMD Open* 2022; 8:e002359. doi: 10.1136/rmdopen-2022-002359

Table S2.1.5: Giant cell arteritis (GCA)

Study	Treatment	Target	Population
Stone 2017 (GiACTA) (47)	Tocilizumab	IL-6R	GCA patients ≥ 50 years of age, new-onset or relapsing GCA
Stone 2019 (3-year analysis) (48)	Tocilizumab	IL-6R	GiACTA: GCA patients ≥ 50 years of age, new-onset or relapsing GCA
Calderón-Goercke 2019 (49)	Tocilizumab IV vs. Tocilizumab SC	IL-6R	GC-IR
Schmidt 2020 (50)<br>study terminated early	Sirukumab	IL-6	New-onset GCA

Table S2.1.6: Takayasu arteritis (TAK)

Study	Treatment	Target	Population
Nakaoka 2018 (the TAKT study) (51)	Tocilizumab	IL-6R	TAK relapse and induced into remission with GC

Table S2.1.7: Multicentric Castleman´s disease (MCD)

Study	Treatment	Target	Population
Van Rhee 2014 (52)	Siltuximab	IL-6	Human immunodeficiency virus and human herpesvirus-8-seronegative patients with symptomatic MCD

Table S2.1.8: CAR-T cell induced Cytokine Release Syndrome (CRS)

Study	Treatment	Target	Population
Le 2018 (53)	Tocilizumab	IL-6R	severe or life-threatening CAR-T cell-induced CRS in adults and in pediatric patients ≥2 years of age

Table S2.1.9: Neuromyelitis optica spectrum disorders (NMOSD)

Study	Treatment	Target	Population
Zhang 2020 (TANGO) (54)	Tocilizumab vs. Azathioprine	IL-6R vs. inhibition of purine synthesis	AQP4-IgG seropositive/negative relapsing NMOSD
Yamamura 2019 (SAkuraStar) (55)	Satralizumab	IL-6R	AQP4-IgG seropositive/negative relapsing NMOSD, GC and/or DMARD allowed
Traboulsee 2020 (56)	Satralizumab	IL-6R	AQP4-IgG seropositive/negative relapsing NMOSD, no concomitant DMARD allowed

2.2. Risk of bias analysis

Table S2.2.1: Rheumatoid arthritis (RA)

Study	Random sequence generation	Allocation concealment	Blinding of participants and personnel	Blinding of outcome assessment	Incomplete outcome data	Selective reporting	Other sources of bias	Summary	Comment
Huizinga 2014 (MOBILITY Part A) (1)	Low	Low	Low	Low	Low	Low	Low	Low	
Genovese 2015 (MOBILITY Part B) (2)	Low	Low	Low	Low	Low	Low	Low	Low	
Tanaka 2019 (KAKEHASI) (3)	Low	Low	Low	Low	Low	Low	Low	Low	
Mazurov 2020 (AURORA, 1-year) (4)	Abstract	Abstract	Abstract	Abstract	Abstract	Abstract	Abstract	Abstract	Open label
NCT02309359 (not published) (5)	-	-	-	-	-	-	-	-	Not fully published
NCT02287922 (not published) (6)	-	-	-	-	-	-	-	-	Not fully published
Nasonov 2020 CREDO-1 (7)	Abstract	Abstract	Abstract	Abstract	Abstract	Abstract	Abstract	Abstract	
Mease 2012 (8)	Low	Low	Low	Low	Low	Low	Low	Low	
Baek 2019 (9)	Low	Low	Low	Low	Low	Low	Low	Low	

NCT00773461 (not published) (10)	-	-	-	-	-	-	-	-	Not fully published
Takeuchi 2017 (SIRROUND-D) (11)	Unclear	Unclear	Low	Low	Low	Low	Low	Unclear	
Fleischmann 2017 (TARGET) (12)	Low	Low	Low	Low	Low	Low	Low	Low	
Takeuchi 2016 (RA0083) (13)	Low	Low	Low	Low	Low	Low	Low	Low	
Aletaha 2017 (SIRROUND-T) (14)	Low	Low	Low	Low	Low	Low	Low	Low	
Genovese 2014 (15)	Low	Low	Low	Low	Low	Low	Low	Low	
Yazici 2012 (ROSE) (16)	Low	Low	Low	Low	Low	Low	Low	Low	
Kivitz 2014 (BREVACTA) (17)	Unclear	Unclear	Low	Low	Low	Low	Low	Unclear	
NCT00977106 (TORPEDO) (18)	-	-	-	-	-	-	-	-	Not fully published
Gabay 2013 (ADACTA) (19)	Low	Low	Low	Low	Low	Low	High	High	Δ DAS28-ESR as primary endpoint
Burmester 2017 (MONARCH) (20)	Low	Low	Low	Low	Low	Low	High	High	Δ DAS28-ESR as primary endpoint
Taylor 2018 (SIRROUND-H) (21)	Low	Low	Low	Low	Low	Low	High	High	Δ DAS28-ESR as primary endpoint
Weinblatt 2015 (22)	Unclear	Unclear	Low	Low	Low	Low	Low	Unclear	
Burmester 2014 (SUMMACTA) (23)	Low	Low	Low	Low	Low	Low	Low	Low	

Ogata 2014 (MUSASHI) (24)	Low	Low	Low	Low	Low	Low	Low	Low	
Ogata 2015 (MUSASHI-OLE) (25)	Low	Low	High	High	Low	Low	Low	High	Open label study
Ogata 2018 (SHINOBI) (26)	Low	Low	Low	Low	Low	Low	Low	Low	
Dougados 2013 (ACT-RAY) (27)	Unclear	Unclear	Low	Low	Low	Unclear	Low	Unclear	
Dougados 2014 (ACT-RAY, 1-year) (28)	Unclear	Unclear	High	High	Low	Low	Low	High	Open-label study; csDMARDs other than MTX were added at week 24 or later if DAS28 >3.2
Kaneko 2016 (SURPRISE) (29)	Low	Low	High	High	High	Low	Low	High	not double-blind, number of patients enrolled lower as planned
Emery 2020 (EXTEND, OLE) (30)	Unclear	Low	High	High	Low	Low	Low	High	Open label extension study of ASCERTAIN trial (57)
Burmester 2016 (FUNCTION) (31)	Low	Low	Low	Low	Low	Low	High	High	Δ DAS28-ESR as primary endpoint
Burmester 2017 (FUNCTION, 2-years) (32)	Low	Low	High	High	Low	Low	High	High	Patients not achieving DAS28-ESR ≤3.2 at week 52 switched to escape therapy (8 mg/kg

									TCZ+MTX). Analyses were exploratory.
Bijlsma 2016 (U-ACT-EARLY) (33)	Low	Low	Low	Low	Low	Low	High	High	Δ DAS28-ESR as primary endpoint
Hetland 2020 (NORD-STAR) (34)	Low	Low	Low	High	Low	Low	Low	High	Open label design
Edwards 2018 (ACT-TAPER) (35)	Unclear	Unclear	Low	Low	Unclear	Unclear	Unclear	Unclear	Study terminated early due to low recruitment
Kremer 2018 (COMP-ACT) (36)	Unclear	Unclear	Low	Low	Low	Low	Low	Unclear	
Pablos 2019 (JUST-ACT) (37)	Low	Low	Low	Low	Low	Low	Low	Low	
Peterfy 2020 (COMP-ACT MRI Substudy) (38)	Unclear	Unclear	Low	Low	Low	Low	Low	Unclear	
Burmester 2020 (SEMIRA) (39)	Low	Low	Low	Low	Low	Low	Low	Low	
Huizinga 2015 (ACT-RAY, 2 and 3-years) (40)	Unclear	Unclear	High	High	Low	Low	Low	High	
Kaneko 2018 (SURPRISE, 2-years) (41)	Low	Low	High	High	High	Low	Low	High	
Kedra 2019 (TOLEDO) (42)	Abstract	Abstract	Abstract	Abstract	Abstract	Abstract	Abstract	Abstract	

Table S2.2.2: Systemic juvenile idiopathic arthritis (sJIA)

Study	Random sequence generation	Allocation concealment	Blinding of participants and personnel	Blinding of outcome assessment	Incomplete outcome data	Selective reporting	Other sources of bias	Summary	Comment
De Benedetti 2012 (TENDER) (43)	Low	Low	Low	Low	Low	Unclear	Low	Unclear	ACR Pediatric 50,70,90 with inclusion of systemic features (fever, rash) only reported for tocilizumab group
Malattia 2020 (44)	Low	Low	High	High	Low	Low	Low	High	post hoc radiographic analysis from two randomized controlled trials

Table S2.2.3: Polyarticular-course juvenile idiopathic arthritis (pcJIA)

Study	Random sequence generation	Allocation concealment	Blinding of participants and personnel	Blinding of outcome assessment	Incomplete outcome data	Selective reporting	Other sources of bias	Summary	Comment
Brunner 2015 (CHERISH) (45)	Low	Low	Low	Low	Low	Low	Low	Low	In part 2, JIA-ACR30 responders were randomly assigned

									to PBO or continue TCZ as in part 1
Malattia 2020 (44)	Low	Low	High	High	Low	Low	Low	High	

Table S2.2.4: Adult-onset Still's disease (AoSD)

**Study**	**Random sequence generation**	**Allocation concealment**	**Blinding of participants and personnel**	**Blinding of outcome assessment**	**Incomplete outcome data**	**Selective reporting**	**Other sources of bias**	**Summary**	**Comment**
Kaneko 2018 (46)	Low	Low	Low	Low	Low	Low	Low	Low	

Table S2.2.5: Giant cell arteritis (GCA)

**Study**	**Random sequence generation**	**Allocation concealment**	**Blinding of participants and personnel**	**Blinding of outcome assessment**	**Incomplete outcome data**	**Selective reporting**	**Other sources of bias**	**Summary**	**Comment**
Stone 2017 (GiACTA) (47)	Low	Low	Low	Low	Low	Low	Low	Low	
Stone 2019 (3-year analysis) (48)	Abstract	Abstract	Abstract	Abstract	Abstract	Abstract	Abstract	Abstract	
Calderón-Goercke 2019 (49)	Abstract	Abstract	Abstract	Abstract	Abstract	Abstract	Abstract	Abstract	

Schmidt 2020 (50)	Low	Low	Low	Low	Unclear	Unclear	Unclear	Unclear	terminated early (October 2017; sponsor decision)

Table S2.2.6: Takayasu arteritis (TAK)

Study	Random sequence generation	Allocation concealment	Blinding of participants and personnel	Blinding of outcome assessment	Incomplete outcome data	Selective reporting	Other sources of bias	Summary	Comment
Nakaoka 2018 (the TAKT study) (51)	Low	Low	Low	Low	Low	Low	Low	Low	

Table S2.2.7: Multicentric Castleman´s disease (MCD)

Study	Random sequence generation	Allocation concealment	Blinding of participants and personnel	Blinding of outcome assessment	Incomplete outcome data	Selective reporting	Other sources of bias	Summary	Comment
Van Rhee 2014 (52)	Low	Low	Low	Low	Low	Low	Low	Low	

Table S2.2.8: CAR-T cell induced Cytokine Release Syndrome (CRS)

Study	Random sequence generation	Allocation concealment	Blinding of participants and personnel	Blinding of outcome assessment	Incomplete outcome data	Selective reporting	Other sources of bias	Summary	Comment
Le 2018 (53)	Unclear	Unclear	High	High	Low	High	Unclear	High	retrospective analysis of pooled data from prospective clinical trials

Table S2.2.9: Neuromyelitis optica spectrum disorders (NMOSD)

Study	Random sequence generation	Allocation concealment	Blinding of participants and personnel	Blinding of outcome assessment	Incomplete outcome data	Selective reporting	Other sources of bias	Summary	Comment
Zhang 2020 (TANGO) (54)	Low	Low	High	Unclear	Low	Low	Low	High	not blinded for investigators and patients
Yamamura 2019 (SAkuraStar) (55)	Low	Low	Low	Low	Low	Low	Low	Low	
Traboulsee 2020 (56)	Low	Low	Low	Low	Low	Low	Low	Low	

2.3. Baseline characteristics

2.3.1: Rheumatoid arthritis (RA)

Table S2.3.1.1: Baseline characteristics of trials investigating IL-6R/L blockers + MTX or csDMARDs versus placebo in patients with inadequate response or intolerance to MTX or csDMARDs.

Study	Treatment	No. of patients (n)	Mean age (years)	Mean disease duration (years)	Mean DAS28	Mean CDAI	Mean HAQ	Mean mTSS
Huizinga 2014 (MOBILITY Part A) (1)	Placebo + MTX	52	55.2	8.07	6.08	40.63		
	SAR 100 mg Q2W + MTX	51	53.5	9.76	6.28	44.74		
	SAR 150 mg Q2W + MTX	51	51.2	7.74	6.11	41.41		
	SAR 100 mg QW + MTX	50	53.9	8.07	6.05	40.32		
	SAR 200 mg Q2W + MTX	52	48.7	5.95	6.06	40.37		
	SAR 150 mg QW + MTX	50	50.9	7.30	6.07	40.48		
Genovese 2015 (MOBILITY Part B) (2)	Placebo + MTX	398	50.9	9.1	5.9		1.6	
	SAR 150 mg Q2W + MTX	400	50.1	9.5	6.0		1.6	
	SAR 200 mg Q2W + MTX	399	50.8	8.6	6.0		1.7	
Tanaka 2019 (KAKEHASI) (3)	Placebo to SAR 150 mg Q2W + MTX	42	51.9	7.6	5.6	34.4	1.1	

Kastrati K, *et al. RMD Open* 2022; 8:e002359. doi: 10.1136/rmdopen-2022-002359

	Placebo to SAR 200 mg Q2W + MTX	40	55.0	8.8	5.3	31.9	1.0	
	SAR 150 mg Q2W + MTX	81	56.1	7.0	5.7	35.9	1.2	
	SAR 200 mg Q2W + MTX	80	55.3	8.3	5.4	32.9	1.1	
Mazurov 2020 (AURORA, 1-year) (4)	LVL (BCD-089) 162 mg QW + MTX	35						
	LVL (BCD-089) 162 mg Q2W + MTX	35						
NCT02309359 (not published) (5)	Placebo + MTX	69	52.8					
	ALX-0061 75 mg Q4W + MTX	69	53.3					
	ALX-0061 150 mg Q4W + MTX	70	52					
	ALX-0061 150 mg Q2W + MTX	68	51.9					
	ALX-0061 225 mg Q2W + MTX	69	52.3					
NCT02287922 (not published) (6)	ALX-0061 150 mg Q4W Mono	62	53.0					
	ALX-0061 150 mg Q2W Mono	62	51.2					
	ALX-0061 225 mg Q2W Mono	63	51.3					
	TCZ 162 mg QW or Q2W	64	50.0					
Nasonov 2020 (CREDO-1) (7)	Placebo + MTX	143	52.7					
	OKZ 64 mg Q2W + MTX	143	52.0					
	OKZ 64 mg Q4W + MTX	142	49.1					
Mease 2012 (8)	Placebo + MTX	33	52	8	6.1		1.6	

	CLZ 80 mg (day 1 and wk 8) + MTX	32	53	7	6.3		1.7	
	CLZ 160 mg (day 1 and wk 8) + MTX	33	55	7	6.2		1.7	
	CLZ 320 mg (day 1 and wk 8) + MTX	29	50	6	6.2		1.7	
Baek 2019 (9)	Placebo + csDMARDs	48	52.0	8.9	6.1		1.4	
	TCZ 8 mg/kg Q4W + csDMARDs	47	52.6	10.8	6.1		1.3	
NCT00773461 (not published) (10)	Placebo + csDMARDs	69	47.8					
	TCZ 8 mg/kg Q4W + csDMARDs	139	46.8					
Takeuchi 2017 (SIRROUND-D) (11)	Placebo + csDMARDs	556	52.9	8.3	5.9		1.6	41.9
	SRK 50 mg Q4W + csDMARDs	557	52.9	8.7	5.9		1.5	41.8
	SRK 100 mg Q2W + csDMARDs	557	53	8.8	5.8		1.5	42.5
MTX: methotrexate; csDMARD: conventional synthetic disease modifying drug; DAS28: Disease Activity Score 28; CDAI: Clinical Disease Activity Index; HAQ: Health assessment Questionnaire, mTSS: modified Total Sharp Score; SAR: sarilumab; Q2W: every other week; QW: once weekly; LVL: levilimab; ALX-0061: vobarilizumab; Q4W: once every 4 weeks; OKZ: olokizumab; CLZ: clazakizumab; wk: week; TCZ: tocilizumab; SRK: sirukumab								

Table S2.3.1.2: Baseline characteristics of trials investigating IL-6R/L blockers in patients with inadequate response or intolerance to TNF-inhibitors.

Study	Treatment	No. of patients (n)	Mean age (years)	Mean disease duration (years)	Mean DAS28	Mean CDAI	Mean HAQ	Mean mTSS
Fleischmann 2017 (TARGET) (12)	Placebo + csDMARDs	181	51.9	12.0	6.2		1.8	
	SAR 150 mg Q2W + csDMARDs	181	54.0	11.6	6.1		1.7	
	SAR 200 mg Q2W + csDMARDs	184	52.9	12.7	6.3		1.8	
Takeuchi 2016 (RA0083) (13)	Placebo + MTX	29	52.6	6.5*	5.3*	35.7*	1.13*	
	OKZ 60 mg Q4W + MTX	32	53.9	7.6*	5.5*	34.3*	1.19*	
	OKZ 120 mg Q4W + MTX	32	55.7	6.9*	5.2*	27.3*	1.25*	
	OKZ 240 mg Q4W + MTX	26	56.7	6.9*	5.3*	29.8*	0.88*	
Aletaha 2017 (SIRROUND-T) (14)	Placebo ± csDMARDs	294	55.4	12.25	5.84	39.06	1.57	
	SRK 50 mg Q4W ± csDMARDs	292	55.8	12.85	5.94	40.41	1.65	
	SRK 100 mg Q2W ± csDMARDs	292	55.0	12.27	5.87	39.99	1.61	
Genovese 2014 (15)	Placebo Q2W ± MTX	22	59.36	10.56*	5.53	36.83*	1.56*	
	Placebo Q4W ± MTX	22	58.18	7.45*	5.69	36.25*	1.38*	
	OKZ 60 mg Q2W ± MTX	20	55.50	12.30*	5.57	36.28*	1.63*	
	OKZ 120 mg Q2W ± MTX	22	53.09	8.07*	5.96	42.90*	1.44*	
	OKZ 240 mg Q2W ± MTX	23	55.48	8.22*	5.94	45.20*	1.75*	

Kastrati K, et al. RMD Open 2022; 8:e002359. doi: 10.1136/rmdopen-2022-002359

	OKZ 60 mg Q4W ± MTX	22	52.64	10.89*	6.14	46.60*	1.81*	
	OKZ 120 mg Q4W ± MTX	23	53.52	11.58*	5.61	39.92*	1.50*	
	OKZ 240 mg Q4W ± MTX	22	54.55	7.83*	5.83	40.50*	1.69*	
	TCZ 8 mg/kg Q4W ± MTX	43	56.58	10.55*	5.72	35.65*	1.63*	

TNF: Tumor Necrosis Factor
* numbers reported as median

Table S2.3.1.3: Baseline characteristics of trials investigating IL-6R/L blockers in patients with inadequate response or intolerance to csDMARDs or TNF-inhibitors.

Study	Treatment	No. of patients (n)	Mean age (years)	Mean disease duration (years)	Mean DAS28	Mean CDAI	Mean HAQ	Mean mTSS
Yazici 2012 (ROSE) (16)	Placebo + csDMARDs	205	55.8	8.52	6.55		4.00*	
	TCZ 8 mg/kg Q4W + csDMARDs	409	55.2	8.62	6.53		4.07*	
Kivitz 2014 (BREVACTA) (17)	Placebo + csDMARDs	219	52.0	11.1	6.6		1.6	60.38
	TCZ 162 mg Q2W + csDMARDs	437	52.1	11.1	6.7		1.6	59.01
NCT00977106 (TORPEDO, not published) (18)	Placebo + csDMARDs	50	51.3					
	TCZ 8 mg/kg Q4W + csDMARDs	53	52.8					

* MDHAQ-PF, multidimensional health assessment questionnaire for physical function

Table S2.3.1.4: Baseline characteristics of trials investigating IL-6R/L blockers vs. other bDMARDs (Head-to-Head trials).

Study	Treatment	No. of patients (n)	Mean age (years)	Mean disease duration (years)	Mean DAS28	Mean CDAI	Mean HAQ	Mean mTSS
Gabay 2013 (ADACTA) (19)	ADA 40 mg Q2W	162	53.3	6.3	6.8	43.1	1.7	
	TCZ 8 mg/kg Q4W	163	54.4	7.3	6.7	40.8	1.6	
Burmester 2017 (MONARCH) (20)	ADA 40 mg Q2W	185	53.6	6.6	6.0	42.4	1.6	
	SAR 200 mg Q2W	184	50.9	8.1	6.0	43.6	1.6	
Taylor 2018 (SIRROUND-H) (21)	ADA 40mg Q2W	186	52.6	4.00	6.05	44.09	1.70	
	SRK 50 mg Q4W	186	52.5	4.24	6.12	44.62	1.75	
	SRK 100 mg Q2W	187	49.	4.60	6.08	45.39	1.62	
Weinblatt 2015 (22)	Placebo + MTX	61	51.4	6.4	6.1		1.6	
	ADA* 40 mg Q2W + MTX	59	52.8	6.1	6.3		1.9	
	CLZ 25 mg Q4W + MTX	59	47.4	5.0	5.7		1.5	
	CLZ 100 mg Q4W + MTX	60	49.9	5.6	5.8		1.5	
	CLZ 200 mg Q4W + MTX	60	46.4	6.0	5.8		1.4	
	CLZ 100 mg Q4W + Placebo	60	55.0	7.4	5.9		1.6	
	CLZ 200 mg Q4W + Placebo	59	50.0	5.0	6.1		1.7	

* adalimumab (40 mg) plus MTX was included as an active reference; ADA: adalimumab

Table S2.3.1.5: Switch studies. Part 1: Baseline characteristics of trials investigating (switching) route of administration and dosage adaptation of IL-6R/L blockers.

Study	Treatment	No. of patients (n)	Mean age (years)	Mean disease duration (years)	Mean DAS28	Mean CDAI	Mean HAQ	Mean mTSS
Burmester 2014 (SUMMACTA) (23)	TCZ SC 162 mg QW ± MTX	558	52.4	8.7	6.6		1.6	
	TCZ IV 8 mg/kg Q4W ± MTX	537	52.5	8.7	6.7		1.7	
Ogata 2014 (MUSASHI) (24)	TCZ SC 162 mg Q2W	159	52.1	7.3	6.1	34.2	1.18***	
	TCZ IV 8 mg/kg Q4W	156	51.8	8.0	6.2	33.7	1.25***	
Ogata 2015 (MUSASHI-OLE) (25)	TCZ SC/SC* 162 mg Q2W	159	52.5	7.4	6.1			
	TCZ IV 8 mg/kg Q4W switched to TCZ SC 162 mg Q2W (TCZ IV/SC)	160	51.5	8.0	6.2			
Ogata 2018 (SHINOBI) (26) **	TCZ SC 162 mg QW	21	56.4	9.7	5.9	35.3		
	TCZ SC 162 mg Q2W	20	55.1	7.0	5.5	31.5		

* TCZ SC/SC: continued TCZ-SC
** TCZ SC Q2W non-responders randomized to TCZ SC QW or TCZ SC Q2W
*** Japanese HAQ
IV: intravenously; SC: subcutaneously

Table S2.3.1.6: Switch studies. Part 2: Baseline characteristics of trials investigating add-on versus switching to IL-6R blockers.

Study	Treatment	No. of patients (n)	Mean age (years)	Mean disease duration (years)	Mean DAS28	Mean CDAI	Mean HAQ	Mean mTSS
Dougados 2013 (ACT-RAY) (27)	add-on strategy arm:<br>TCZ 8 mg/kg Q4W + MTX	277	53.0	8.2	6.33		1.46	30.4*
	switch strategy arm:<br>TCZ 8 mg/kg Q4W + Placebo	276	53.6	8.3	6.36		1.48	37.1*
Dougados 2014 (ACT-RAY, 1-year) ** (28)	add-on strategy arm:<br>TCZ 8 mg/kg Q4W + MTX	277	53.0	8.2	6.33		1.46	30.8*
	switch strategy arm:<br>TCZ 8 mg/kg Q4W + Placebo	276	53.6	8.3	6.36		1.48	37.2*
Kaneko 2016 (SURPRISE) (29)	add-on strategy arm:<br>TCZ 8 mg/kg Q4W + MTX	115	55.8	3.6	5.1	22.6	1.0	
	switch strategy arm:<br>TCZ 8 mg/kg Q4W	111	56.3	3.8	5.3	24.2	1.0	

* Genant-modified Sharp score (GSS)
** open-label csDMARDs other than MTX were added at week 24 or later in patients with DAS28 >3.2

Kastrati K, et al. RMD Open 2022; 8:e002359. doi: 10.1136/rmdopen-2022-002359

Table S2.3.1.7: Switch studies. Part 3: Baseline characteristics of trials investigating switching to another IL-6R blocker.

Study	Treatment	No. of patients (n)	Mean age (years)	Mean disease duration (years)	Mean DAS28	Mean CDAI	Mean HAQ	Mean mTSS
Emery 2020 (EXTEND, OLE) (30)*	SAR 150 mg SC Q2W + csDMARDs	37	53.5	12.9	3.0	11.9	1.1	5.66
	SAR 200 mg SC Q2W + csDMARDs	38	52.2	8.8	3.0	13.0	1.2	
	TCZ 4 mg/kg IV Q4W (no change in dose) + csDMARDs	35	51.2	9.1	3.3	10.6	1.0	
	TCZ 4→8 mg/kg IV Q4W + csDMARDs at wk 4, then continuing 8 mg/kg IV Q4W	38	50.1	11.5	3.2	13.7	1.3	
	All TCZ (including pat. changing dose after wk 4 of the RCT)	93	50.4	9.9	3.2	12.4	1.2	

* treatment during RCT, before switching to SAR 200 mg SC Q2W in the OLE

Table S2.3.1.8: Induction/Strategic studies. Part 1: Baseline characteristics of trials comparing the effectiveness of IL-6R blocker monotherapy and combination therapy with MTX in early RA.

Study	Treatment	No. of patients (n)	Mean age (years)	Mean disease duration (years)	Mean DAS28	Mean CDAI	Mean HAQ	Mean mTSS
Burmester 2016 (FUNCTION) (31)	Placebo + MTX	287	49.6	0.4	6.6		1.48	5.66
	TCZ 4 mg/kg Q4W + MTX	288	51.2	0.4	6.7		1.62	7.72
	TCZ 8 mg/kg Q4W + MTX	290	49.5	0.5	6.7		1.50	6.17
	TCZ 8 mg/kg Q4W + Placebo	292	49.9	0.5	6.7		1.58	6.85
Burmester 2017 (FUNCTION, 2-years) (32)*	Placebo + MTX pre-escape	142	49.9	0.5	6.7		1.5	7.04
	TCZ 4 mg/kg Q4W + MTX pre-escape	95	50.6	0.5	6.9		1.7	8.31
Bijlsma 2016 (U-ACT-EARLY) (33)	Placebo + MTX	108	53.5	< 0.1**	5.1		1.1	0.0
	TCZ 8 mg/kg Q4W + MTX	106	53.0	< 0.1**	5.2		1.1	0.0
	TCZ 8 mg/kg Q4W + Placebo	103	55.0	< 0.1**	5.3		1.3	0.0

* patients not receiving 8 mg/kg TCZ and not achieving Disease Activity Score-28 joints (DAS28-erythrocyte sedimentation rate (ESR)) ≤3.2 at week 52 switched to escape therapy (8 mg/kg TCZ+MTX)
** symptom duration

Kastrati K, *et al. RMD Open* 2022; 8:e002359. doi: 10.1136/rmdopen-2022-002359

Table S2.3.1.9: Induction/Strategic studies. Part 2: Baseline characteristics of trials comparing the effectiveness of IL-6R blocker + MTX with conventional treatment in early RA.

Study	Treatment	No. of patients (n)	Mean age (years)	Mean disease duration (years)	Mean DAS28	Mean CDAI	Mean HAQ	Mean mTSS
Hetland 2020 (NORD-STAR) (34)	MTX + active conventional treatment*	200	54.6	0.5*	5.1	28.6	1.1	
	CZP 200 mg Q2W** + MTX	203	55.3	0.5*	5.0	27.9	1	
	ABA 125 mg QW + MTX	204	54.7	0.6*	5.1	28.6	1.1	
	TCZ IV 8 mg/kg Q4W (or SC 162 mg QW) + MTX	188	52.4	0.6*	4.9	26.6	1.1	

* active conventional treatment: (a) oral prednisolone (tapered from 20 to 5 mg/day in nine weeks) or (b) sulfasalazine (2 g/day) combined with hydroxychloroquine (35 mg/kg every week or 200 mg/day) and mandatory intra-articular triamcinolone hexacetonide injection (or equivalent)
** CZP loading dose 400 mg at week 0, 2, and 4
*** symptom duration
CZP: certolizumab pegol; ABA: abatacept

Kastrati K, et al. RMD Open 2022; 8:e002359. doi: 10.1136/rmdopen-2022-002359

Table S2.3.1.10: Tapering studies. Part 1: Baseline characteristics of trials investigating tapering of csDMARDs while on IL-6R blocker therapy.

Study	Treatment	No. of patients (n)	Mean age (years)	Mean disease duration (years)	Mean DAS28	Mean CDAI	Mean HAQ	Mean mTSS
Edwards 2018 (ACT-TAPER) (35)	TCZ 8 mg/kg Q4W + Placebo (tapering MTX)	136	54.4	7.9	6.58			
	TCZ 8 mg/kg Q4W + MTX (stable MTX)	136	56.4	7.2	6.61			
Kremer 2018 (COMP-ACT) (36)	TCZ 162 mg QW/Q2W* + Placebo	147	54.6	6.8	6.2	37.3	1.3	
	TCZ 162 mg QW/Q2W* + MTX	147	56.4	6.8	6.3	39.1	1.4	
Pablos 2019 (JUST-ACT) (37)	TCZ 8 mg/kg Q4W + Placebo (switch to TCZ mono)	82	51.0	6.4	2.0		0.7	
	TCZ 8 mg/kg Q4W + MTX	82	50.2	5.8	1.8		0.5	
Peterfy 2020 (COMP-ACT MRI Substudy) (38)	TCZ 162 mg QW/Q2W* + Placebo	38	54.2	6.8	6.4	37.4		
	TCZ 162 mg QW/Q2W* + MTX	41	58.3	7.0	6.2	38.5		

* ≥100 kg start QW; DAS28-ESR > 3.2 increase frequency to QW

Table S2.3.1.11: Tapering studies. Part 2: Baseline characteristics of trials investigating tapering of glucocorticoids while on IL-6R blocker therapy.

Study	Treatment	No. of patients (n)	Mean age (years)	Mean disease duration (years)	Mean DAS28	Mean CDAI	Mean HAQ	Mean mTSS
Burmester 2020 (SEMIRA) (39)	TCZ IV 8 mg/kg Q4W or SC 162 mg QW ± csDMARDs + Glucocorticoid tapering	131	54.8	9.6	1.88	5.5		
	TCZ IV 8 mg/kg Q4W or SC 162 mg QW ± csDMARDs + Glucocorticoid continuation	128	54.0	8.6	1.95	5.7		

Table S2.3.1.12: Tapering studies. Part 3: Baseline characteristics of trials investigating tapering of IL-6R blockers.

Study	Treatment	No. of patients (n)	Mean age (years)	Mean disease duration (years)	Mean DAS28	Mean CDAI	Mean HAQ	Mean mTSS
Huizinga 2015 (ACT-RAY, 2 and 3-years) (40) *	add-on strategy arm: TCZ 8 mg/kg Q4W + MTX	277	53.0	8.2	6.33		1.46	36.9**
	switch strategy arm: TCZ 8 mg/kg Q4W + Placebo	276	53.6	8.3	6.36		1.48	41.2**

Kaneko 2018 (SURPRISE, 2-years) (41)	add-on arm (TCZ+MTX: discontinuing TCZ → MTX mono	49	57.5	3.6	1.4		0.32	
	switch arm (TCZ mono): discontinuing TCZ → no DMARD	53	54.4	3.5	1.4		0.31	
Kedra 2019 (TOLEDO) (42)	TCZ (or ABA) maintenance at full dose	116						
	progressive injection interval increase (by stage) up to bDMARD discontinuation	117						

* week 52-104, patients in sustained clinical remission (DAS28-ESR <2.6) discontinued TCZ. If sustained remission was maintained, csDMARDs, then MTX/PBO, were discontinued
** GSS, Genant-modified Sharp Score

2.3.2: Systemic juvenile idiopathic arthritis (sJIA)

Table S2.3.2.1: Baseline characteristics of trials investigating IL-6R/L blockers in sJIA.

Study	Treatment	No. of patients (n)	Mean age (years)	Mean disease duration (years)	Criteria for active disease	Fever (%)	Rash (%)	Mean CRP (mg/L)	Mean CHAQ	aSH*	Poznanski score*	Steroids at baseline (%)
De Benedetti 2012 (TENDER) (43)<br><br>12-week RCT followed by a long-term extension	Placebo	37	9.1	5.1	>5 active joints<br><br>or<br><br>>2 active joints and fever (>38°C; >5 days)	65	49		1.7			84
	TCZ IV 8mg/kg (if ≥30 kg) or 12mg/kg (<30kg) Q2W	75	10.0	5.2		55	29		1.7			93
Malattia 2020 (44)	TCZ (TENDER trial)	aSH: n=47**	9.9	5.2	see TENDER (43)				1.6	24.60		49
		Poznanski: n=33**	8.4	4.8					1.6		−2.38	36

CHAQ: Childhood Health Assessment Questionnaire; aSH: adapted Sharp–van der Heijde score; CRP: C-reactive protein
* numbers reported as median
** radiographic population

Kastrati K, et al. RMD Open 2022; 8:e002359. doi: 10.1136/rmdopen-2022-002359

2.3.3: Polyarticular-course juvenile idiopathic arthritis (pcJIA)

Table S2.3.3.1: Baseline characteristics of trials investigating IL-6R/L blockers in pcJIA.

Study	Treatment	No. of patients (n)	Mean age (years)	Mean disease duration (years)	Criteria for active disease	Joints with active arthritis (n)	Joints with LOM (n)	Mean CRP (mg/L)	Mean CHAQ	aSH**	Poznanski score**	Steroids at baseline (%)
Brunner 2015 (CHERISH) (45)	*Part 1: 16-week open-label, lead-in period:*<br>TCZ 8 mg/kg (if body weight ≥30 kg) Q2W ± MTX<br>or<br>10 mg or 8mg/kg (if weight <30kg) Q2W ± MTX<br>*Part 2: pat. with ≥JIA- (ACR) 30 improvement entered double-blind, randomized 24-week, withdrawal phase:*<br>Continue TCZ or Placebo	188*	11.0*	4.2*	≥ 5 active joints and MTX-IR	20.3*	17.6*	23.3*	1.4*			46*
Malattia 2020 (44)	TCZ (CHERISH trial)	aSH: n=45***	10.8	3.9	see CHERISH (45)	20.9	14.8		1.3	8.00		42
		Poznanski: n=35***	9.9	3.2		21.7	16.3		1.3		− 1.45	43

LOM: limitation of movement; MTX-IR: inadequate response to methotrexate
* baseline data study part 1

** numbers reported as median
*** radiographic population

2.3.4: Adult-onset Still's disease (AoSD)

Table S2.3.4.1: Baseline characteristics of trials investigating IL-6R/L blockers in AoSD.

Study	Treatment	No. of patients (n)	Mean age (years)	Mean disease duration (years)	Fever (%)	Skin rash (%)	Mean HAQ	SFS	Mean CRP (mg/dl)
Kaneko 2018 (46)	Placebo	13	55.5	0.1	46.2	53.8	1.0	5.1	4.7
	TCZ 8mg/kg Q2W	13	51.3	0.5	46.2	61.5	0.7	4.6	4.2

SFS: systemic feature score (consists of five clinical and five laboratory assessments; clinical assessment include fever, rash, lymphadenopathy, hepatosplenomegaly and serositis; laboratory aspects include ESR, CRP, leucocyte count, hb level and platelet count)

2.3.5: Giant cell arteritis (GCA)

Table S2.3.5.1: Baseline characteristics of trials investigating IL-6R/L blockers in GCA.

Study	Treatment	No. of patients (n)	Mean age (years)	GCA newly diagnosed (%)	Mean disease duration (years)	Cranial signs or symptoms (%)	PMR symptoms (%)	Mean CRP (mg/dl)	Mean ESR (mm/h)
Stone 2017 (GiACTA) (47)	Placebo + GC-26-Wk Taper	50	69.3	46	1	80	60		28.8
	Placebo + GC-52-Wk Taper	51	67.8	45	0.7	78	69		24.2
	TCZ 162 mg SC QW + GC-26-Wk taper	100	69.5	47	0.8	78	59		24.6
	TCZ 162 mg SC Q2W + GC-26-Wk taper	50	69.4	52	0.7	82	64		20.8
Stone 2019 (3-year analysis) (48)	Pooled Placebo (new onset)	46							
	Pooled Placebo (relapsing)	55							
	TCZ QW (new onset)	47							
	TCZ QW (relapsing)	53							
	TCZ Q2W (new onset)	26							
	TCZ Q2W (relapsing)	23							
Calderón-Goercke 2019 (49)	TCZ IV	104	73.4					3.3	41.8
	TCZ SC	30	71.9					2.1	35.9
Schmidt 2020 (50)	Placebo + GC-6-month Taper	27	71.6	59.3			40.7		

	Placebo + GC-12-month Taper	27	70.7	55.6			48.1		
	SRK 50 mg Q4W + GC-6-month Taper	26	67.5	46.2			61.5		
	SRK 100 mg Q2W + GC-3-month Taper	39	68.1	56.4			56.4		
	SRK 100 mg Q2W + GC-6-month Taper	42	70.5	59.5			59.5		

PMR: polymyalgia rheumatica; ESR: erythrocyte sedimentation rate; GC: glucocorticoid (i.e prednisone)

2.3.6: Takayasu arteritis (TAK)

Table S2.3.6.1: Baseline characteristics of trials investigating IL-6R/L blockers in TAK.

Study	Treatment	No. of patients (n)	Mean age (years)	Mean disease duration (years)	Mean CRP (mg/dl)	Mean GC dose (mg/kg)	HLA-B52 positive (%)
Nakaoka 2018 (the TAKT study) (51)	Placebo + GC Taper*	18	30.8	3.57		0.52	72.2
	TCZ 162 mg QW + GC taper*	18	31.1	6.46		0.57	38.9

* relapsing patients were induced into remission with oral glucocorticoid (GC) therapy; after randomization GC were tapered 10% per week from week 4 to a minimum of 0.1mg/kg per day
HLA: human leucocyte antigen

2.3.7: Multicentric Castleman´s disease (MCD)

Table S2.3.7.1: Baseline characteristics of trials investigating IL-6R/L blockers in MCD.

Study	Treatment	No. of patients (n)	Age* (years)	Disease duration (years)*	Overall symptom score	Steroids at baseline (%)	Hb level (g/l)*	CRP (mg/l)*	ESR (mm/h)*	Albumin (g/l)*
Van Rhee 2014 (52)	Placebo + BSC	26	48		10	35	134	4.2	23.5	36
	SIL 11mg/kg Q3W + BSC	53	47		6	25	118	17.6	62.0	35

* numbers reported as median
Hb: haemoglobin; BSC: best supportive care (management of disease related symptoms as well as conditions, infections, and infusion-related reactions referred to institutional guidelines, transfusions); SIL: siltuximab

Kastrati K, *et al. RMD Open* 2022; 8:e002359. doi: 10.1136/rmdopen-2022-002359

2.3.8: CAR-T cell induced Cytokine Release Syndrome (CRS)

Table S2.3.8.1: Baseline characteristics of trials investigating IL-6R/L blockers in CRS.

Study	Treatment	(CAR) T-cell therapy	No. of patients (n)	Age (years)*	Underlying malignancy (%)	1 dose of TCZ (%)	≥3 doses of TCZ (%)	Baseline CRS grade 3 (%)	Baseline CRS grade 4 (%)
Le 2018 (53)	TCZ 8 mg/kg (12 mg/kg for pts <30 kg)	CTL019 (Tisagenlecleucel) series	45	12	ALL (100)	55.5	15.6	22.2	77.8
		KTE-C19 (Axicabtagene Ciloleucel) series	15	60	ALL (13.3)<br>DLBCL (80.0)<br>PMBCL (6.7)	40.0	26.7	93.3	6.7

* numbers reported as median
CAR: chimeric antigen receptor; ALL: acute lymphoblastic leukemia; DLBCL: diffuse large B cell lymphoma; PMBCL: primary mediastinal B cell lymphoma

2.3.9: Neuromyelitis optica spectrum disorders (NMOSD)

Table S2.3.9.1: Baseline characteristics of trials investigating IL-6R/L blockers in NMOSD.

Study	Treatment	No. of patients (n)	Mean age (years)	Mean disease duration (years)	AQP4-IgG positivity (%)	EDSS score	Annualized relapse rate
Zhang 2020 (TANGO) (54)	AZA (2-3mg/kg) ± concomitant immunosuppressants	59	45.3	6.2	90	4.5	1.68*
	TCZ 8mg/kg Q4W + concomitant immunosuppressants for the first 12 wks; then TCZ monotherapy	59	48.1	6.0	85	4.5	1.71*
Yamamura 2019 (SAkuraStar) (55)	Placebo + concomitant immunosuppressants	42	43.4		67	3.63	1.4*
	SAT SC 120 mg wk 0, 2, 4; then Q4W + concomitant immunosuppressants	41	40.8		66	3.83	1.5*
Traboulsee 2020 (56)	Placebo	32	40.5	214.7**	72	3.7	1.5
	SAT SC 120 mg wk 0, 2, 4 and Q4W	63	45.3	317.8**	65	3.9	1.4

* annualized relapse rate in previous 2 years
** mean disease duration in weeks
AZA: azathioprine; AQP4-IgG: aquaporin-4 autoantibody; EDSS: Expanded Disability Status Scale, ranging from 0 (normal neurologic status) to 10 (death); SAT: satralizumab

2.4. Efficacy outcomes

2.4.1: Rheumatoid arthritis (RA)

Table S2.4.1.1: Efficacy outcomes of trials investigating IL-6R/L blockers + MTX or csDMARDs versus placebo in patients with inadequate response or intolerance to MTX or csDMARDs.

Study	Treatment	No. of patients (n)	Timepoint (weeks)	ACR20 (%)	ACR50 (%)	ACR70 (%)	DAS28 <2.6 (%)	CDAI ≤2.8 (%)	ACR/EULAR Boolean rem. (%)	ΔHAQ (mean)	ΔmTSS (mean)
Huizinga 2014 (MOBILITY Part A) (1)	Placebo + MTX	52	12	46	15	2	9			-0.26	
	SAR 100 mg Q2W + MTX	51		49	22	6	8			-0.35	
	SAR 150 mg Q2W + MTX	51		67	35	12	20			-0.62	
	SAR 100 mg QW + MTX	50		62	40	16	20			-0.42	
	SAR 200 mg Q2W + MTX	52		65	40	17	26			-0.57	
	SAR 150 mg QW + MTX	50		72	30	16	30			-0.45	
Genovese 2015 (MOBILITY Part B) (2)	Placebo + MTX	398	24	33.4	17	7	10.1	5.0		-0.29[a]	2.78[b]
	SAR 150 mg Q2W + MTX	400		58.0	37	20	27.8	10.3		-0.53[a]	0.90[b]
	SAR 200 mg Q2W + MTX	399		66.4	46	25	34.1	13.8		-0.55[a]	0.25[b]
Tanaka 2019 (KAKEHASI) (3)	Placebo to SAR 150 mg Q2W + MTX	42	24	14.8	9.9	3.7	7.4	1.2		-0.3	
	Placebo to SAR 200 mg Q2W + MTX	40									

Kastrati K, et al. RMD Open 2022; 8:e002359. doi: 10.1136/rmdopen-2022-002359

	SAR 150 mg Q2W + MTX	81		67.9	43.2	18.5	35.8	6.2		-0.5	
	SAR 200 mg Q2W + MTX	80		57.5	38.8	15.0	40.0	10.0		-0.6	
Mazurov 2020 (AURORA, 1-year) (4)	LVL (BCD-089) 162 mg QW + MTX	35	52	91.4	74.3	65.7					
	LVL (BCD-089) 162 mg Q2W + MTX	35		71.4	65.7	45.7					
NCT02309359 (not published) (5)	Placebo + MTX	69	12	62.3	27.5	8.7	8.7	4.3	4.3	-0.613	
	ALX-0061 75 mg Q4W + MTX	69		75.4	29.0	14.5	4.3	4.3	0.0	-0.696	
	ALX-0061 150 mg Q4W + MTX	70		81.4	44.3	21.4	37.1	10.0	7.1	-0.619	
	ALX-0061 150 mg Q2W + MTX	68		77.9	41.2	19.1	22.1	5.9	2.9	-0.771	
	ALX-0061 225 mg Q2W + MTX	69		72.5	44.9	17.4	30.4	7.2	5.8	-0.615	
NCT02287922 (not published) (6)	ALX-0061 150 mg Q4W Mono	62	12	72.6	43.5	16.1	33.9	9.7	3.2	-0.541	
	ALX-0061 150 mg Q2W Mono	62		77.4	37.1	24.2	21.0	4.8	4.8	-0.746	
	ALX-0061 225 mg Q2W Mono	63		81.0	49.2	20.6	39.7	6.3	6.3	-0.817	
	TCZ 162 mg QW or Q2W	64		78.1	45.3	23.4	25.0	9.4	6.3	-0.689	
Nasonov 2020 (CREDO-1) (7)	Placebo + MTX	143	12	25.9	7.7[c]			0[c]		-0.20	
	OKZ 64 mg Q2W + MTX	143		63.6	42.7[c]			8.4[c]		-0.54	
	OKZ 64 mg Q4W + MTX	142		70.4	48.6[c]			7.7[c]		-0.56	
Mease 2012 (8)	Placebo + MTX	33	12	27	9	3	0[a]			-0.47[a]	
	CLZ 80 mg (day 1 and wk 8) + MTX	32		81	34	13	14[a]			-0.57[a]	

	CLZ 160 mg (day 1 and wk 8) + MTX	33		71	27	12	28[a]			-0.58[a]	
	CLZ 320 mg (day 1 and wk 8) + MTX	29		82	50	25	44[a]			-0.67[a]	
Baek 2019 (9)	Placebo + csDMARDs	48	24	16.7	2.1	2.1	3.3			-0.2	
	TCZ 8 mg/kg Q4W + csDMARDs	47		61.7	29.8	4.3	42.5			-0.3	
NCT00773461 (not published) (10)	Placebo + csDMARDs	69	24	24.6	10.1	2.9	3.1			-0.06	
	TCZ 8 mg/kg Q4W + csDMARDs	139		69.8	38.8	12.9	30.5			-0.52	
Takeuchi 2017 (SIRROUND-D) (11)	Placebo + csDMARDs	556	16	26	10.8	4.0	5.6[c]	3.1[c]		-0.22[c]	1.96[c]
	SRK 50 mg Q4W + csDMARDs	557		55	30.0	13.5	26.0[c]	7.0[c]		-0.43[c]	0.35[c]
	SRK 100 mg Q2W + csDMARDs	557		54	26.2	13.5	25.5[c]	8.4[c]		-0.46[c]	0.30[c]

[a] efficacy outcome at week 16
[b] radiographic outcome at week 52
[c] efficacy outcome at week 24

Table S2.4.1.2: Efficacy outcomes of trials investigating IL-6R/L blockers in patients with inadequate response or intolerance to TNF-inhibitors.

Study	Treatment	No. of patients (n)	Timepoint (weeks)	ACR20 (%)	ACR50 (%)	ACR70 (%)	DAS28 <2.6 (%)	CDAI ≤2.8 (%)	ACR/EULAR Boolean rem. (%)	ΔHAQ (mean)	ΔmTSS (mean)
	Placebo + csDMARDs	181	24	33.7	18.2	7.2	7.2			-0.3	

Fleischmann 2017 (TARGET) (12)	SAR 150 mg Q2W + csDMARDs	181		55.8	37.0	19.9	24.9			-0.5	
	SAR 200 mg Q2W + csDMARDs	184		60.9	40.8	16.3	28.8			-0.6	
Takeuchi 2016 (RA0083) (13)	Placebo + MTX	29	12	21.9	8.6	3.8	3.4			0	
	OKZ 60 mg Q4W + MTX	32		58.7	35.7	9.6	21.9			-0.4	
	OKZ 120 mg Q4W + MTX	32		62.5	42.1	22.5	40.6			-0.4	
	OKZ 240 mg Q4W + MTX	26		73.8	39.1	17.1	53.8			-0.4	
Aletaha 2017 (SIRROUND-T) (14)	Placebo ± csDMARDs	294	16	24	9	3	5.8		1	-0.12	
	SRK 50 mg Q4W ± csDMARDs	292		40	21	6	17.5		1.7	-0.25	
	SRK 100 mg Q2W ± csDMARDs	292		45	22	10	15.8		3.1	-0.32	
Genovese 2014 (15)	Placebo Q2W ± MTX	22	12	29.9	4.9		4.5			0.0[a]	
	Placebo Q4W ± MTX	22		17.1	1.3		0.0			0.06[a]	
	OKZ 60 mg Q2W ± MTX	20		49.7	19.1		10.0			-0.25[a]	
	OKZ 120 mg Q2W ± MTX	22		55.5	24.9		13.6			-0.25[a]	
	OKZ 240 mg Q2W ± MTX	23		55.5	31.9		26.1			-0.38[a]	
	OKZ 60 mg Q4W ± MTX	22		60.7	33.2		13.6			-0.50[a]	
	OKZ 120 mg Q4W ± MTX	23		58.4	21.3		21.7			-0.25[a]	
	OKZ 240 mg Q4W ± MTX	22		32.5	11.5		9.1			0.0[a]	
	TCZ 8 mg/kg Q4W ± MTX	43		68.3	27.7		20.9			-0.25[a]	

[a] numbers reported as median

Table S2.4.1.3: Efficacy outcomes of trials investigating IL-6R/L blockers in patients with inadequate response or intolerance to csDMARDs or TNF-inhibitors.

Study	Treatment	No. of patients (n)	Timepoint (weeks)	ACR20 (%)	ACR50 (%)	ACR70 (%)	DAS28 <2.6 (%)	CDAI ≤2.8 (%)	ACR/EULAR Boolean rem. (%)	ΔHAQ (mean)	ΔmTSS (mean)
Yazici 2012 (ROSE) (16)	Placebo + csDMARDs	205	24		11.2						
	TCZ 8 mg/kg Q4W + csDMARDs	409			30.1						
Kivitz 2014 (BREVACTA) (17)	Placebo + csDMARDs	219	24	31.5	12	5	4				1.23
	TCZ 162 mg Q2W + csDMARDs	437		60.9	40	20	32				0.62
NCT00977106 (TORPEDO, not published) (18)	Placebo + csDMARDs	50	4								
	TCZ 8 mg/kg Q4W + csDMARDs	53									

Kastrati K, et al. RMD Open 2022; 8:e002359. doi: 10.1136/rmdopen-2022-002359

Table S2.4.1.4: Efficacy outcomes of trials investigating IL-6R/L blockers vs. other bDMARDs (Head-to-Head trials).

Study	Treatment	No. of patients (n)	Timepoint (weeks)	ACR20 (%)	ACR50 (%)	ACR70 (%)	DAS28 <2.6 (%)	CDAI ≤2.8 (%)	ACR/EULAR Boolean rem. (%)	ΔHAQ (mean)	ΔmTSS (mean)
Gabay 2013 (ADACTA) (19)	ADA 40 mg Q2W	162	24	49.4	27.8	17.9	10.5	9.3[a]		-0.5	
	TCZ 8 mg/kg Q4W	163		65.0	47.2	32.5	39.9	17.2[a]		-0.7	
Burmester 2017 (MONARCH) (20)	ADA 40 mg Q2W	185	24	58.4	29.7	11.9	7.0	2.7		-0.43	
	SAR 200 mg Q2W	184		71.7	45.7	23.4	26.6	7.1		-0.61	
Taylor 2018 (SIRROUND-H) (21)	ADA 40mg Q2W	186	24	56.5	31.7	12.9	7.5		3.8	-0.52	
	SRK 50 mg Q4W	186		53.8	26.9	11.8	12.9		3.8	-0.51	
	SRK 100 mg Q2W	187		58.8	35.3	15.5	20.3		3.7	-0.53	
Weinblatt 2015 (22)	Placebo + MTX	61	12	39.3		6.6[b]	1.6	3.3	3.3	-0.62[b]	
	ADA 40 mg Q2W + MTX	59		76.3		18.6[b]	20.3	8.5	5.1	-0.66[b]	
	CLZ 25 mg Q4W + MTX	59		76.3		27.1[b]	35.6	11.9	8.5	-0.68[b]	
	CLZ 100 mg Q4W + MTX	60		73.3		40.0[b]	35.0	8.3	10.0	-0.79[b]	
	CLZ 200 mg Q4W + MTX	60		60.0		30.0[b]	26.7	3.3	5.0	-0.71[b]	
	CLZ 100 mg Q4W + Placebo	60		55.0		16.7[b]	21.7	8.3	6.7	-0.64[b]	
	CLZ 200 mg Q4W + Placebo	59		61.0		25.4[b]	25.4	3.4	1.7	-0.60[b]	

[a] post-hoc analysis
[b] efficacy outcome at week 24

Table S2.4.1.5: Switch studies. Part 1: Efficacy outcomes of trials investigating (switching) route of administration and dosage adaptation of IL-6R/L blockers.

Study	Design	Primary / Secondary outcome	Timepoint (weeks)	Treatment arm	No. of patients (n)	Result	p / 95% CI
Burmester 2014 (SUMMACTA) (23)	NI (margin: 12%)	ACR20 response; ACR50/70 response, %DAS28-ESR<2.6, %of decrease ≥0.3 from baseline in HAQ	24	TCZ SC 162 mg QW ± MTX	558	69.4%; 47%, 24%, 38%, 65%	PE: ACR20: −4.0% (CI:−9.2, 1.2)<br><br>ACR50/70: -1.8% (CI:−7.5, 4.0) /-3.8% (CI:−9.0, 1.3) DAS28-rem: 0.9% (CI:−5.0, 6.8) HAQ: −2.3% (CI:−8.1, 3.4)
				TCZ IV 8 mg/kg Q4W ± MTX	537	73.4%; 49%, 28%, 36%, 67%	
Ogata 2014 (MUSASHI) (24)	NI (margin: 18%)	ACR20 response; ACR50/70 response, ACR/EULAR Boolean remission, CDAI ≤2.8, DAS28-ESR<2.6, mean ΔDAS28, mean ΔCDAI, %of decrease ≥0.3 from baseline in HAQ	24	TCZ SC 162 mg Q2W	159	79.2%; 63.5%, 37.1%, 15.7%, 16.4%, 49.7%, 6.1 to 2.8, 34.2 to 10.3, 56.6%	PE: ACR20: -9.4% (CI:-17.6, -1.2)<br><br>ACR50/70: - 4.3% (CI:-14.7, 6.0)/-3.8% (CI -14.5, 6.8)
				TCZ IV 8 mg/kg Q4W	156	88.5%; 67.3%, 41.0%, 16.0%, 23.1%, 62.2%, 6.2 to 2.5, 33.7 to 8.2, 67.9%	
	OLE	Mean DAS28-ESR, mean CDAI, ACR20/50/70 response,		TCZ SC/SC* 162 mg Q2W	159	2.6, 9.6, 86.1%, 65.8%, 39.9%, 57.0%	NR

Ogata 2015 (MUSASHI-OLE) (25)		%DAS28-ESR<2.6, CDAI ≤2.8 [a], CDAI >2.8-≤10 [a]	36 (12 wks after switching)	TCZ IV 8 mg/kg Q4W switched to TCZ SC 162 mg Q2W (TCZ IV/SC)	160	2.6, 8.7, 85.0%, 66.9%, 36.9%, 62.5%	
Ogata 2018 (SHINOBI) (26)	S (TCZ QW over Q2W)	ΔDAS28-ESR; %DAS28-ESR<2.6, CDAI ≤2.8, ΔCDAI, ACR20/50/70 response	12	TCZ SC 162 mg QW	21	-2.10; 19.0%, 4.8%, -16.0, 52.4%, 38.1%, 14.3%	difference in: PE: ΔDAS28-ESR: -1.21 (CI:-2.13, -0.30, p=0.0108) ΔCDAI: -7.26 (CI:-15.93, 1.40, p= 0.0979)
				TCZ SC 162 mg Q2W	20	-0.89; 10.0%, 0.0%, -8.7, 20.0%, 15.0%, 15.0%	
NI: non-inferiority; S: superiority; OLE: open-label extension; PE: primary endpoint; NR: not reported<br>* TCZ SC/SC: continued TCZ-SC<br>[a] numbers not shown or calculated							

Table S2.4.1.6: Switch studies. Part 2: Efficacy outcomes of trials investigating add-on versus switching to IL-6R blockers.

Study	Design	Primary / Secondary outcome	Timepoint (weeks)	Treatment arm	No. of patients (n)	Result	p / 95% CI
Dougados 2013 (ACT-RAY) (27)	S (add-on over switch)	DAS28-ESR<2.6; mean ΔDAS28, DAS28 <3.2, EULAR good plus moderate responders, ACR–EULAR Boolean remission, SDAI ≤3.3, CDAI ≤2.8, Δtotal GSS,	24	add-on strategy: TCZ 8 mg/kg Q4W + MTX	277	40.4%; −3.43, 61.7%, 89.5%, 6.9%, 11.9%, 11.9%, 0.08, 90.6%, 65.7%, 71.5%, 45.5%, 24.5%, 5.8%	PE: DAS28-ESR<2.6: p=0.19, 95% CI: -2.41, 13.71<br><br>0.051, 0.029, 0.30,

		%no progression in GSS (≤SDC), %no progression in GSS (≤0), ACR20/50/70/90 response		switch strategy: TCZ 8 mg/kg Q4W + Placebo	276	34.8%; −3.21, 51.4%, 86.2%, 5.4%, 9.8%, 7.6%, 0.22, 87.3%, 59.1%; 70.3%, 40.2%, 25.4%, 5.1%	0.53, 0.56, 0.12, 0.26, 0.18, 0.088, 0.87, 0.30, 0.68, 0.84
Dougados 2014 (ACT-RAY, 1-year) (28) *	NS (1-year data of ACT-RAY)	DAS28-ESR<2.6, mean ΔDAS28, DAS28 <3.2, EULAR good plus moderate responders, ACR–EULAR Boolean remission, SDAI ≤3.3, CDAI ≤2.8, ΔHAQ, Δtotal GSS, %no progression in GSS (SDC≤ 1.5), ACR20/50/70/90 response	52	add-on strategy: TCZ 8 mg/kg Q4W + MTX	277	45.5%, -3.74, 62.5%, 84.5%, 17.7%, 24.2%, 22.7%, -0.59, 0.35, 92.8%, 70.8%, 50.2%, 31.4%, 12.6%	PE: DAS28-ESR<2.6: 0.03; 0.39, 0.12, 0.12, 0.09, 0.10, 0.06, 0.14, 0.36, 0.016, 0.62, 0.22, 0.99, 0.65
				switch strategy: TCZ 8 mg/kg Q4W + Placebo	276	36.6%, -3.67, 57.2%, 78.2%, 12.3%, 18.1%, 15.9%, -0.67, 0.63, 86.1%, 69.2%, 55.4%, 31.2%, 11.2%	
Kaneko 2016 (SURPRISE) (29)	NI (margin: 10%)	DAS28-ESR<2.6; ΔDAS28, SDAI ≤3.3, CDAI ≤2.8, EULAR good/moderate responders, ACR–EULAR Boolean remission, ΔHAQ, %ΔmTSS ≤ 0.5[a], ACR20/50/70[b]; clinically relevant radiographic progression rates (CRRP; mTSS≥3), mean ΔmTSS in CRRP patients	24	add-on strategy: TCZ 8 mg/kg Q4W + MTX	115	69.6%; -2.9, 44.1%, 39.6%, 96.5%, 20.9%, -0.4, %ΔmTSS NR, 74.8%, 54.8%, 33.0%; NR, NR	PE: DAS28-ESR<2.6: p=0.03; 0.41, 0.07, 0.13, 0.06, 0.87, 0.75
				switch strategy: TCZ 8 mg/kg Q4W	111	55.0%; -2.7, 29.6%, 27.8%, 90.1%, 19.8%, -0.4, %ΔmTSS NR, 69.4%, 54.1%, 34.2%; NR, NR	
			52	add-on strategy: TCZ 8 mg/kg Q4W + MTX	115	72.2%, -3.0, 52.2%, 47.8%, 94.8%, 37.1%, -0.4, 66%, 73.9%,	0.77, 0.79, 0.43, 0.60, 0.10, 0.78,

						62.6%, 47.0%; 7% (7/95), 5.0/year	0.50, 0.92; 0.07, 0.04
				switch strategy: TCZ 8 mg/kg Q4W	111	70.3%; -3.0, 46.8%, 44.1%, 88.3%, 35.1%, -0.5, 64%, 77.5%, 63.1%, 44.1%; 15% (15/98), 9.0/year	

SDAI: Simplified Disease Activity Index; SDC: smallest detectable change; NS: not specified; NR: not reported; CRRP: clinically relevant radiographic progression rates
* open-label csDMARDs other than MTX were added at week 24 or later (week 36) in patients with DAS28 >3.2
[a] radiological outcomes (mTSS) reported at week 52
[b] ACR20/50/70 response rates at week 24/52 between both treatment arms not statistically significant (5% significance level was used)

Table S2.4.1.7: Switch studies. Part 3: Efficacy outcomes of trials investigating switching to another IL-6R blocker.

Study	Treatment*	No. of patients (n)	Timepoint (weeks)	ACR20 (%)	ACR50 (%)	ACR70 (%)	DAS28 <2.6 (%)	CDAI ≤2.8 (%)	ACR/EULAR Boolean rem. (%)	ΔHAQ (mean)	ΔmTSS (mean)
Emery 2020 (EXTEND, OLE) (30)	SAR 150 mg SC Q2W + csDMARDs	37	96				76.9	80.0			
	SAR 200 mg SC Q2W + csDMARDs	38					56.3	33.3			

	TCZ 4 mg/kg IV Q4W (no change in dose) + csDMARDs	35					80.0	55.6			
	TCZ 4→8 mg/kg IV Q4W + csDMARDs at wk 4, then continuing 8 mg/kg IV Q4W	38					72.7	50.0			
	All TCZ (including pat. changing dose after wk 4 of the RCT)	93					79.3	58.8			

EXTEND: all patients received open-label sarilumab 200 mg SC Q2W
* treatment during the RCT, before switch to sarilumab 200 mg SC Q2W in the OLE

Table S2.4.1.8: Induction/Strategic studies. Part 1: Efficacy outcomes of trials comparing the effectiveness of IL-6R blocker monotherapy and combination therapy with MTX in early RA.

Study	Treatment	No. of patients (n)	Timepoint (weeks)	ACR20 (%)	ACR50 (%)	ACR70 (%)	DAS28 <2.6 (%)	CDAI ≤2.8 (%)	ACR/EULAR Boolean rem. (%)	ΔHAQ (mean)	ΔmTSS (mean)
Burmester 2016 (FUNCTION) (31)	Placebo + MTX	287	24	65.2	43.2	25.4	15			-0.71	1.14[b]
	TCZ 4 mg/kg Q4W + MTX	288		73.6	47.9	34.7	31.9			-0.92	0.42[b]
	TCZ 8 mg/kg Q4W + MTX	290		74.5	56.9	38.6	44.8			-0.91	0.08[b]
	TCZ 8 mg/kg Q4W + Placebo	292		70.2	47.6	30.1	38.7			-0.82	0.26[b]
Burmester 2017	Placebo + MTX pre-escape	142	52[b]/104[c]	43.0[c]	30.3[c]	16.2[c]	51.4[c]				

(FUNCTION, 2-years) (32)[a]	TCZ 4 mg/kg Q4W + MTX pre-escape	95		29.5[c]	16.8[c]	6.3[c]	30.5[c]				
	Placebo + MTX	287		58.5[b]/25.4[c]	41.5[b]/22.0[c]	29.3[b]/17.4[c]	20.2[b]/16.0[c]	19.5[b]/20.2[c]	12.2[b]/10.1[c]		1.88[d]
	TCZ 4 mg/kg Q4W + MTX	288		65.3[b]/39.6[c]	54.9[b]/36.5[c]	37.8[b]/31.6[c]	36.1[b]/28.1[c]	25.3[b]/27.8[c]	17.0[b]/17.0[c]		1.43[d]
	TCZ 8 mg/kg Q4W + MTX	290		67.9[b]/65.2[c]	56.2[b]/57.6[c]	43.4[b]/46.6[c]	49.3[b]/47.6[c]	32.1[b]/37.9[c]	20.7[b]/23.1[c]		0.19[d]
	TCZ 8 mg/kg Q4W + Placebo	292		65.4[b]/61.6[c]	50.7[b]/53.1[c]	37.0[b]/39.4[c]	40.4[b]/43.5[c]	24.0[b]/32.5[c]	15.1[b]/19.2[c]		0.62[d]
Bijlsma 2016 (U-ACT-EARLY) (33) [e]	Placebo + MTX	108	24/52[b]/104[c]	59/69[b]/61[c]	34/51[b]/48[c]	15/33[b]/35[c]	39.6/61.2[b]/58.6[c]	13/36[b]/37[c]			0.96[b]/1.53[c]
	TCZ 8 mg/kg Q4W + MTX	106		75/75[b]/63[c]	64/62[b]/49[c]	44/44[b]/36[c]	80.0/71.0[b]/63.5[c]	30/42[b]/47[c]			0.50[b]/1.18[c]
	TCZ 8 mg/kg Q4W + Placebo	103		75/72[b]/65[c]	59/59[b]/55[c]	37/44[b]/39[c]	75.5/80.8[b]/70.5[c]	27/35[b]/40[c]			0.79[b]/1.45[c]

[a] patients not receiving 8 mg/kg TCZ and not achieving DAS28-ESR ≤3.2 at week 52 switched to escape therapy (8 mg/kg TCZ+MTX)
[b] efficacy outcome at week 52
[c] efficacy outcome at week 104
[d] mean change from baseline to week 104
[e] outcome definition: remission according DAS28 remission criteria and swollen joint count ≤ 4 joints; CDAI remission rate based on post-hoc analysis

Table S2.4.1.9: Induction/Strategic studies. Part 2: Efficacy outcomes of trials comparing the effectiveness of IL-6R blocker + MTX with conventional treatment in early RA.

Study	Treatment	No. of patients (n)	Timepoint (weeks)	ACR20 (%)	ACR50 (%)	ACR70 (%)	DAS28 <2.6 (%)	CDAI ≤2.8 (%)	ACR/EULAR Boolean rem. (%)	ΔHAQ (mean)	ΔmTSS (mean)
Hetland 2020 (NORD-STAR) (34)	MTX + active conventional treatment	200	24				reference	48.2	reference		
	CZP 200 mg Q2W + MTX	203					2.6[a]	52.6	3.6[a]		
	ABA 125 mg QW + MTX	204					4.5[a]	56.3	4.6[a]		
	TCZ IV 8 mg/kg Q4W (or SC 162 mg QW) + MTX	188					-0.7[a]	48.7	-3.8[a]		

[a] numbers are percentage differences in rates with active conventional treatment as reference

Table S2.4.1.10: Tapering studies. Part 1: Efficacy outcomes of trials investigating tapering of csDMARDs while on IL-6R blocker therapy.

Study	Design	Primary / Secondary outcome	Timepoint (weeks)	Treatment arm	No. of patients (n)	Result	p / 95% CI
Edwards 2018 (ACT-TAPER) (35)	NI (margin: 10%)		60	TCZ 8 mg/kg Q4W + Placebo (tapering MTX)	136	76.5%; 51.5%	PE: Maintenance of EULAR

		Maintenance of EULAR good/moderate response from week 24 to 60; DAS28<2.6		TCZ 8 mg/kg Q4W + MTX (stable MTX)	136	65.4%; 47.1%	good/moderate response from week 24 to 60: 0.036; 0.342
Kremer 2018 (COMP-ACT) (36)	NI (margin: 0.6)	ΔDAS28-ESR week 24 to 40; DAS28<2.6, DAS28≤3.2, ACR 20/50/70	40	TCZ 162 mg QW/Q2W + Placebo	147	0.46; 49.7%, 63.3%, 69%, 50%, 34%	PE: ΔDAS28-ESR week 24 to 40: 0.318 (CI: 0.045, 0.592)
				TCZ 162 mg QW/Q2W + MTX	147	0.14; 59.2%, 76.9%, 79%, 64%, 42%	
Pablos 2019 (JUST-ACT) (37)	NI (margin: 0.6)	ΔDAS28-ESR week 16 to 28, DAS28<2.6, CDAI<2.6, SDAI<3.3, ΔHAQ	28	TCZ 8 mg/kg Q4W + Placebo (switch to TCZ mono)	82	0.073; 75.9%, 35.8%, 28.2%, 0.02	PE: ΔDAS28-ESR week 16 to 28: -0.06 (CI: -0.40, 0.27) 0.328, 0.518, 0.358, 0.674
				TCZ 8 mg/kg Q4W + MTX	82	0.007; 82.3%, 40.7%, 35.1%, 0.06	
Peterfy 2020 (COMP-ACT MRI Substudy) (38)	Substudy of COMP-ACT	Mean ΔMRI-assessed synovitis, osteitis, erosion, and cartilage loss from week 24 to 40 (each outcome: both hands/dominant hand); %pat not progressing more than SDC in dominant hand and wrist at Week 40 for each outcome measure	40	TCZ 162 mg QW/Q2W + Placebo	38	−0.18/−0.11, 0.37/0.69, 0.18/0.49, −0.03/−0.05; 97%, 87.9%, 84.8%, 93.9%	Difference (95%CI): 0.06 (CI:−0.30, 0.41)/0.11 (CI:−0.18, 0.40), 0.53 (CI:−0.30, 1.36)/1.07 (CI:−0.18, 2.33), 0.24 (CI:−0.21, 0.68)/0.43 (CI:−0.14,1.01), −0.23 (CI:−0.58, 0.11)/−0.16 (CI:−0.59,0.27); -3.0 (CI: -8.9, 2.8), -4.4 (CI:-18.4, 9.5), -12.6 (CI:-25.8, 0.6), -3.5 (CI:-13.0, 6.0)
				TCZ 162 mg QW/Q2W + MTX	41	−0.24/−0.22, −0.16/−0.39, −0.06/0.06, 0.20/0.11; 100%, 92.3%, 97.4%, 97.4%	

Table S2.4.1.11: Tapering studies. Part 2: Efficacy outcomes of trials investigating tapering of glucocorticoids while on IL-6R blocker therapy.

Study	Design	Primary / Secondary outcome	Timepoint (weeks)	Treatment arm	No. of patients (n)	Result	p / 95% CI
Burmester 2020 (SEMIRA) (39)	S (margin: 0.6)	ΔDAS28-ESR; DAS28-ESR ≤3.2 + no flare + no confirmed adrenal insufficiency; ΔCDAI, %no flare, ΔHAQ	24	TCZ IV 8 mg/kg Q4W or SC 162 mg QW ± csDMARDs + Glucocorticoid tapering	131	0.538; 65%, 2.663, 74%, 0.17	PE: ΔDAS28-ESR: p<0.0001; relative risk 0.83 (CI:0.71 to 0.97); CI: 0.661 to 4.023, flare not assessed, p<0.0001
				TCZ IV 8 mg/kg Q4W or SC 162 mg QW ± csDMARDs + Glucocorticoid continuation	128	-0.075; 77%, 0.321, 89%, -0.09	

Table S2.4.1.12: Tapering studies. Part 3: Efficacy outcomes of trials investigating tapering of IL-6R blockers.

Study	Design	Primary / Secondary outcome	Timepoint (weeks)	Treatment arm	No. of patients (n)	Result	p / 95% CI
Huizinga 2015 (ACT-RAY, 2 and 3-years) (40)	NS	DAS28-ESR<2.6; mean ΔDAS28, EULAR good/moderate responders, ACR–EULAR Boolean remission, SDAI ≤3.3, CDAI ≤2.8, ΔHAQ, Δtotal GSS, %no progression in GSS (≤2.1), %TCZ-free remission, median time to TCZ-free rem., %total drug-free remission, %flare after TCZ-free rem., median time to flare after TCZ-free rem.	104	add-on strategy arm: TCZ 8 mg/kg Q4W + MTX	277	38.3%; -3.60, 75.8%, 14.8%, 22.0%, 22.7%, -0.67, 0.35, 94.4%, 53.1%, 645 d, 8.6%, 82.5%, 113 d	DAS28-ESR<2.6: 0.452; 0.934, 0.056, 0.048, 0.627, 0.203, 0.833, 0.034, 0.098, 0.170, p-values for time to TCZ-free rem not reported, 0.010, CI: 0.815, 0.973, p-values for time to flare after TCZ-rem. not reported
				switch strategy arm: TCZ 8 mg/kg Q4W + Placebo	276	35.1%; -3.61, 66.7%, 9.4%, 19.9%, 18.1%, -0.69, 0.95, 91.1%, 47.6%, 786 d, 3.1%, 88.5%, 84 d	
Kaneko 2018 (SURPRISE, 2-years) (41)	NS	TCZ free rate, TCZ free DAS28-ESR<2.6, TCZ-free DAS28≤3.2, HAQ, ΔmTSS	104	add-on arm (TCZ+MTX: discontinuing TCZ → MTX mono	49	67.3%, 24.4%; 55.1%, 0.30, 0.37	TCZ free rate: 0.001, 0.29, 0.005, 0.29, 0.36
				switch arm (TCZ mono): discontinuing TCZ → no DMARD	53	28.5%, 14.3%; 26.6%, 0.17, 0.64	
Kedra 2019 (TOLEDO) (42)	NI (margin: 0.25 for DAS44; 0.07 for flare rates)	DAS-44; flare (DAS28 > 3.2), major flare (DAS28 >3.2+ no recovery at following visit despite previous bDMARD escalation)	104	TCZ (or ABA) maintenance at full dose	116	DAS-44 slope difference for TCZ subgroup: 0.02 (95% CI: -0.22, 0.26) flare:	DAS-44: NI: 0.22, p=0.03
				progressive injection interval increase (by stage) up to bDMARD discontinuation	117		

						+0.42 (95% CI: 0.27, 0.57)<br>major flare: +0.07 (95%CI: -0.03, 0.16)	
d: days							

2.4.2: Systemic juvenile idiopathic arthritis (sJIA)

Table S2.4.2.1: Efficacy outcomes of trials investigating IL-6R/L blockers in sJIA.

Study	Treatment	No. of patients (n)	Timepoint (weeks)	JIA ACR 30 + no fever (%)	JIA ACR30 (%)	JIA ACR50 (%)	JIA ACR70 (%)	JIA ACR90 (%)	Fever/Rash (%)	Mean ESR (mm/h)	CFB in CHAQ score (%)	ΔaSH*	ΔPoznanski score*
De Benedetti 2012 (TENDER) (43)	Placebo	37	12	24	24.3	10.8	8.1	5.4	79/89	59.8	-10.3		
	TCZ IV 8mg/kg (if ≥30 kg) or 12mg/kg (<30kg) Q2W	75		85	90.7	85.3	70.7	37.3	15/36	4.4	-45.6		
Malattia 2020 (44)	TCZ (TENDER trial)	aSH: n=45[a]/37[b]	52[a]/104[b]									0.00[a]/0.50[b]	
		Poznanski: n=32[a]/26[b]											0.29[a]/0.16[b]

CFB: change from baseline
* values reported as median, change from baseline
[a] week 52
[b] week 104

2.4.3: Polyarticular-course juvenile idiopathic arthritis (pcJIA)

Table S2.4.3.1: Efficacy outcomes of trials investigating IL-6R/L blockers in pcJIA.

Study	Treatment	No. of patients (n)	Timepoint (weeks)	JIA-ACR30 flare (%)	JIA ACR30 (%)	JIA ACR50 (%)	JIA ACR70 (%)	JIA ACR90 (%)	ΔESR (mm/h)	ΔCHAQ	ΔaSH*	ΔPoznanski score*
Brunner 2015 (CHERISH) (45)	all Placebo	81	40 (16 wks open label TCZ + 24 wks withdrawal)	48.1	54.3	51.9	42.0	23.5	-12.0	−0.6		
	all TCZ	82		25.6	74.4	73.2	64.6	45.1	-26.3	−0.8		
Malattia 2020 (44)	TCZ (CHERISH trial)	aSH: n=40[a]/35[b]	52[a]/104[b]								0.50[a]/-1.00[b]	
		Poznanski: n=31[a]/25[b]										0.26[a]/0.55[b]

* values reported as median, change from baseline
[a] week 52
[b] week 104

2.4.4: Adult-onset Still's disease (AoSD)

Table S2.4.4.1: Efficacy outcomes of trials investigating IL-6R/L blockers in AoSD.

Study	Treatment	No. of patients (n)	Timepoint (weeks)	ACR20 (%)	ACR50 (%)	ACR70 (%)	Fever (%)	Rash (%)	ΔHAQ	ΔSFS	ΔCRP (mg/dl)	Decrease in GC dose (%)
Kaneko 2018 (46)*	Placebo	13	4[a]/12[b]	38.5[a]/30.8[b]	30.8[a]/30.8[b]	30.8[a]/30.8[b]	7.7[a]/15.4[b]	38.5[a]/38.5[b]		-2.7[a]/-2.3[b]		21.0[b]
	TCZ 8mg/kg Q2W	13		76.9[a]/61.5[b]	61.5[a]/61.5[b]	38.5[a]/46.2[b]	0.0[a]/0.0[b]	15.4[a]/15.4[b]		-4.1[a]/-4.1[b]		46.2[b]

* two coprimary endpoints of double-blind phase (part 1,2):
part 1[a]: proportion of patients who achieved ACR 50% improvement at 4 weeks.
part 2[b]: proportion of patients who achieved ACR 50% improvement at 12 weeks.

2.4.5: Giant cell arteritis (GCA)

Table S2.4.5.1: Efficacy outcomes of trials investigating IL-6R/L blockers in GCA.

Study	Primary / Secondary outcome	Timepoint (weeks)	Treatment arm	No. of patients (n)	Result	p / 95% CI
Stone 2017 (GiACTA) (47)	% of sustained GC-free remission at week 52 versus placebo + GC-26-wk taper (primary endpoint); % of sustained GC-free remission at week 52 vs placebo + GC-52-wk taper (key secondary outcome); cumulative GC dose (mg), first flare incidence after remission, Δquality of life (SF-36)	52	Placebo + GC-26-Wk Taper	50	14%; 3296, 68%, -0.28	PE: p<0.001 for the comparison of each TCZ group with PBO SE: p<0.001; GC-dose: 0.001 (for both comparisons), flare: HR 0.23 (CI:0.11,0.46) 0.28 (CI:0.12,0.66) p<0.001, SF-36: 0.002 (TCZ QW vs 52-wk taper)
			Placebo + GC-52-Wk Taper	51	18%; 3818, 49%, -1.49	
			TCZ 162 mg SC QW + GC-26-Wk taper	100	56%; 1862, 23%, 4.10	
			TCZ 162 mg SC Q2W + GC-26-Wk taper	50	53%; 1862, 26%, 2.76	
Stone 2019 (3-year analysis) (48)	Median time to first flare (days), %flare during entire 3-year study period	156	Pooled Placebo (new onset)	46	179; 72%	PBO new onset CI:149-331; PBO relapsing CI:148-322; TCZ QW new onset CI:499-NE; TCZ QW relapsing CI:463-NE; TCZ Q2W new onset CI:341-778; TCZ Q2W relapsing CI:162-645
			Pooled Placebo (relapsing)	55	224; 69%	
			TCZ QW (new onset)	47	577; 51%	
			TCZ QW (relapsing)	53	575; 53%	
			TCZ Q2W (new onset)	26	479; 73%	
			TCZ Q2W (relapsing)	23	428; 65%	

Kastrati K, *et al. RMD Open* 2022; 8:e002359. doi: 10.1136/rmdopen-2022-002359

Calderón-Goercke 2019 (49)	%prolonged remission, %relapse, GC-sparing effects (median)	26[a]/52[b]/104[c]	TCZ IV	104	56.3%[a], 61.4%[b], 63.6%[c]; 6.3%[a]; 15.8%[b], 21.2%[c]; 6.9[a], 3.7[b], 2.4[c]	0.712, 0.043, 0.257; 0.251, 0.140, 0.180; 0.032, 0.085, 0.021
			TCZ SC	30	65%[a], 91.7%[b], 85.7%[c]; 0.0%[a]; 0.0%[b], 0.0%[c]; 3.8[a], 1.7[b], 0.0[c]	
Schmidt 2020 (50)<br><br>study terminated early (October 2017)	% of sustained remission at week 52, %flare (wk 12- wk 52)	52	Placebo + GC-6-month Taper	9[d]	0%, 88.9%,	NA
			Placebo + GC-12-month Taper	7[d]	0%, 71.4%	
			SRK 50 mg Q4W + GC-6-month Taper	9[d]	11.1%, 55.6%	
			SRK 100 mg Q2W + GC-3-month Taper	13[d]	15.4%, 69.2%	
			SRK 100 mg Q2W + GC-6-month Taper	17[d]	17.6%, 52.9%	

SE: secondary endpoint; PBO: placebo; SF-36: 36- item Short Form Health Survey
[a] week 26
[b] week 52
[c] week 104
[d] patients in the revised intent-to-treat population (data presented with imputation; imputation rule: pat. withdrawing from the study early counted as flare)

2.4.6: Takayasu arteritis (TAK)

Table S2.4.6.1: Efficacy outcomes of trials investigating IL-6R/L blockers in TAK.

Study	Treatment	No. of patients (n)	Timepoint (weeks)	Relapse protocol definition* (%)	Relapse Kerr's definition (%)	Relapse clinical definition (%)	Time to relapse (weeks)[a]	Time to relapse (weeks)[b]	Time to relapse (weeks)[c]
Nakaoka 2018 (the TAKT study) (51)	Placebo + GC Taper	18	until 19 pat. relapsed	61.1	61.1	61.1	12.1	12.1	12.0
	TCZ 162 mg QW + GC taper	18		44.4	44.4	61.1	NE	NE	16.0

* defined as ≥2 of the following: objective systemic symptoms, subjective systemic symptoms, elevated inflammation markers, vascular signs/symptoms or ischemic symptoms
NE: not evaluable
[a] protocol definition, numbers reported as median
[b] Kerr's definition, numbers reported as median
[c] clinical definition, numbers reported as median

2.4.7: Multicentric Castleman´s disease (MCD)

Table S2.4.7.1: Efficacy outcomes of trials investigating IL-6R/L blockers in MCD.

Study	Treatment	No. of patients (n)	Timepoint (weeks)	Durable tumor + symptomatic response (%)[a]	Tumor response (%)[b]	Tumor response (%)[c]	Duration of durable tumor + symptomatic response (days)[d]	Time to durable symptomatic response (days)[e]	Time to treatment failure (days)[d]	Hb ≥15g/L (%)[f]	1-year survival (%)
Van Rhee 2014 (52)	Placebo + BSC	26	≥18 wks during masked treatment	0	4	0	NE	65	134	0	92
	SIL 11mg/kg Q3W + BSC	53		34	38	51	383	155	NE	61	100

NE: not evaluable

[a] by independent review, intention-to-treat population, defined as a complete or partial response by modified Cheson criteria with improvement or stabilization of disease-related symptoms for at least 18 weeks (= primary endpoint)

[b] according to independent review, response-evaluable population

[c] according to investigator assessment, response-evaluable population

[d] intention-to-treat population, numbers reported as median

[e] by independent review for responders (response-evaluable population), numbers reported as median

[f] week 13 compared with baseline (evaluable population, n=31 in SIL group vs n=11 in placebo group)

2.4.8: CAR-T cell induced Cytokine Release Syndrome (CRS)

Table S2.4.8.1: Efficacy outcome of trials investigating IL-6R/L blockers in CRS.

Study	Treatment	(CAR) T-cell therapy	No. of patients (n)	Response by day 14 (%)[a]	Time to response (days)[b]	Response by day 2 (%)	Response by day 7 (%)	Response by day 21 (%)
Le 2018 (53)	TCZ 8 mg/kg (12 mg/kg for pts <30 kg)	CTL019 (Tisagenlecleucel) series	45	68.9	4	20.0	57.8	68.9
		KTE-C19 (Axicabtagene Ciloleucel) series	15	53.3	4.5	20.0	53.3	53.3

[a] primary analysis (response defined as resolving of CRS within 14 days of 1. dose of TCZ, if no more than 2 doses of TCZ were needed, and if no drugs other than TCZ and GCs were used for treatment)
[b] median time from first dose to response

2.4.9: Neuromyelitis optica spectrum disorders (NMOSD)

Table S2.4.9.1: Efficacy outcomes of trials investigating IL-6R/L blockers in NMOSD.

Study	Primary / Secondary outcome	Timepoint	Treatment arm	No. of patients (n)	Result	Hazard Ratio or Difference (95% CI), p-value
Zhang 2020 (TANGO) (54)	Time to first relapse (weeks); % first relapse, %confirmed disease progression at 12 weeks, Δserum AQP4-IgG titres (change from baseline), Δserum AQP4-IgG titres (percentage change), %confirmed disease progression at 24 weeks	≥60 wks following randomization	AZA (2-3mg/kg) ± concomitant immunosuppressants	59	56.7; 47%, 25%, 0, 0%, 10%	PE: Time to first relapse (weeks): HR -14.3 (-26.7,-3.4) p=0.0026; HR 0.236 (0.107,0.518) p<0.0001; HR 0.288 (0.105,0.795) p=0.0087; HR -240 (-480,-240) p<0.0001; HR -33% (-50,-17) p<0.0001; HR 0.221 (0.047,1.042) p=0.0004
			TCZ 8mg/kg Q4W + concomitant immunosuppressants for the first 12 wks; then TCZ monotherapy	59	78.9; 14%, 8%, -240, -50%, 3%	
Yamamura 2019 (SAkuraStar) (55)	%protocol-defined relapse; ΔVAS pain score at wk 24, ΔFACIT-F score at wk 24, annualized relapse rate, ΔSF-36 score at wk 24 (physical component), ΔSF-36 score at wk 24 (mental component), ΔEDSS score at 24 wk, ΔEQ-5D score at 24 wk, Δmodified Rankin scale score at 24 wk	median treatment duration: 107.4 wks	Placebo + concomitant immunosuppressants	42	43%; -3.73, 3.12, 0.32, 2.46, 2.28, -0.21, 0.04, -0.05	PE: %protocol-defined relapse: 0.38 (0.16,0.88), p=0.02; 4.08 (-8.44,16.61), p=0.52; -3.10 (-8.38,2.18); 0.34 (0.15,0.77);
			SAT SC 120 mg wk 0, 2, 4; then Q4W + concomitant immunosuppressants	41	20%; 0.35, 0.02, 0.11, 1.10, -0.03, -0.10, -0.002, -0.03	

Traboulsee 2020 (56)	%protocol-defined relapse; ΔVAS pain score at wk 24, ΔFACIT-F score at wk 24, annualized relapse rate, ΔSF-36 score at wk 24 (physical component), ΔSF-36 score at wk 24 (mental component), ΔEDSS score at 24 wk, ΔEQ-5D score at 24 wk, Δmodified Rankin scale score at 24 wk	Occurrence of 44 protocol-defined relapses or 1.5 years after random assignment of the last enrolled patient	Placebo	32	50%; -5.95, 3.60, 0.41, 3.59, 1.39, -0.17, 0.04, -0.19	PE: %protocol-defined relapse: HR 0.45 (0.23,0.89), p=0.018; 3.21 (-5.09,11.52), p=0.44; 2.11 (-1.01, 5.22); 0.41 (0.21,0.79);
			SAT SC 120 mg wk 0, 2, 4 and Q4W	63	30%; -2.74, 5.71, 0.17, 2.54,4.84, 0.34, 0.04, -0.03	
VAS: Visual Analogue Scale; FACIT-F: Functional Assessment of Chronic Illness Therapy–Fatigue; EQ-5D: EuroQol-five dimensions						

Section 3: Characteristics of articles and abstracts included: Efficacy for other studied diseases

3.1. Details of articles and abstracts selected for inclusion

Table S3.1.1: Psoriatic arthritis (PsA)

**Study**	**Treatment**	**Target**	**Population**
Mease 2016 (58)	Clazakizumab	IL-6	NSAID-IR and/or csDMARD-IR; bDMARD naïve; all DMARDs except MTX discontinued

Table S3.1.2: Axial spondyloarthritis (axSpA)

**Study**	**Treatment**	**Target**	**Population**
Sieper 2014 (BUILDER-1) (59)	Tocilizumab	IL-6R	r-axSpA, NSAID-IR, active disease: BASDAI ≥4 + spinal pain ≥40 VAS (0-100 mm);<br>BUILDER-1: TNFi-naïve
Sieper 2015 (ALIGN) (60)	Sarilumab	IL-6R	r-axSpA, NSAID-IR, active disease: BASDAI + total back pain score ≥4

Table S3.1.3: Osteoarthritis (OA)

Study	Treatment	Target	Population
Richette 2020 (61)	Tocilizumab	IL-6R	painful hand OA; pain level ≥40 mm VAS pain (0-100 mm); at least 3 painful joints, Kellgren-Lawrence grade ≥2; pain not responding to acetaminophen or NSAID and weak opioids

Table S3.1.4: Polymyalgia rheumatica (PMR)

Study	Treatment	Target	Population
Lally 2016 (62)	Tocilizumab	IL-6R	newly diagnosed PMR, treated with glucocorticoids (GCs) for <1 month and ≤20 mg of prednisone daily or its equivalent
Devauchelle-Pensec 2016 (TENOR) (63)	Tocilizumab	IL-6R	PMR according to Chuang's PMR criteria, symptom onset within the last 12 months; active disease defined as PMR-AS>10; either no history of GC or GC for no longer than 1 month stopped at least 7 days before inclusion

Table S3.1.5: ANCA-associated vasculitis (GPA, MPA)

Study	Treatment	Target	Population
No study fulfilling criteria for inclusion was found	-	-	-

Table S3.1.6: Remitting seronegative symmetric synovitis with pitting edema (RS3PE)

Study	Treatment	Target	Population
No study fulfilling criteria for inclusion was found	-	-	-

Table S3.1.7: Systemic sclerosis associated interstitial lung disease (SSc-ILD)

Study	Treatment	Target	Population
Khanna 2020 (focuSSced) (64)	Tocilizumab	IL-6R	adult patients with:<br>• early diffuse cutaneous systemic sclerosis (dcSSc)<br>• classified according to 2013 ACR/EULAR criteria<br>• 60 months total disease duration or less (from first non-raynaud symptom)<br>• mRSS 10-35 units at baseline<br>• elevated acute-phase (CRP ≥6 mg/L, ESR ≥28 mm/h, or platelet count ≥330×10^9/L)<br>• patients with pulmonary disease with FVC (FVC% predicted) ≤55%, or a diffusing capacity for carbon monoxide (DLCo) ≤45% were excluded<br>• no immunosuppressive treatment

Table S3.1.8: Idiopathic inflammatory myopathies (IIM)

Study	Treatment	Target	Population
NCT02043548 (phase 2, not published) (65)	Tocilizumab	IL-6R	definite or probable polymyositis (PM) or dermatomyositis (DM) classified by Bohan and Peter criteria and refractory to treatment with GC or GC+DMARDs/intravenous immunoglobulin/anti-TNF/Rituximab

Table S3.1.9: Systemic lupus erythematosus (SLE)

Study	Treatment	Target	Population
Wallace 2017 (phase 2, BUTTERFLY) (66)	PF-04236921	IL-6	adult SLE patients with active disease:<br>• SLEDAI-2K ≥6<br>• BILAG Level A disease in ≥1 organ system (except renal or central nervous system)<br>or<br>• BILAG B disease in ≥2 organ systems if no level A disease
Rovin 2016 (phase 2) (67)	Sirukumab	IL-6	patients with biopsy proven class III or class IV lupus nephritis and persistent proteinuria (>0.5 g/day) despite immunosuppressive treatment (MMF/AZA±GC) and renin-angiotensin system blockade
NCT02437890 (phase 2, not published) (68)	Vobarilizumab (ALX-0061)	IL-6R	adult patients with moderate to severe active, seropositive systemic lupus erythematosus (SLE)

Table S3.1.10: Primary Sjögren's syndrome (pSS)

Study	Treatment	Target	Population
Felten 2020 (69)	Tocilizumab	IL-6R	pSS according to American European Consensus Group (AECG) criteria and an ESSDAI ≥ 5; concomitant GC and/or csDMARDs allowed

Table S3.1.11: Amyloid A (AA)- Amyloidosis (AAA)

Study	Treatment	Target	Population
Okuda 2014 (70)	Tocilizumab vs. TNF-i	IL-6R vs. TNF	patients with biopsy-proven AA amyloidosis complicating rheumatic diseases (n=39 rheumatoid arthritis, n=2 juvenile idiopathic arthritis carry-over, n=1 adult-onset Still's disease)
Okuda 2018 (71)	Tocilizumab vs. TNF-i vs. Abatacept	IL-6R vs. TNF vs. CD-80/CD-86	survey of 199 pat. with AAA with rheumatoid arthritis (60.3%), uncharacterized inflammatory disorders (11.1%), neoplasms (7.0%), other rheumatic diseases (6.5%) etc. TCZ was used in n=66 pat., anti-TNF in n=27 and ABA in n=4 cases.

Table S3.1.12: Multiple Myeloma (MM)

**Study**	**Treatment**	**Target**	**Population**
San-Miguel 2014 (phase 2) (72)	Siltuximab	IL-6	patients with untreated multiple myeloma and not candidate for high dose chemotherapy with stem cell transplantation due to age (≥65 years) or important comorbid conditions.
Brighton 2019 (phase 2) (73)	Siltuximab	IL-6	adult patients with High-Risk Smoldering Multiple Myeloma (SMM) for <4 years (defined as BMPC ≥10% and either serum M-protein ≥3 g/dL, or abnormal free light chain ratio [<0.126 or >8] and serum M-protein ≥1 <3g/dL) and an Eastern Cooperative Oncology Group (ECOG) Performance Status score of 0 or 1

Table S3.1.13: Refractory relapsing polychondritis

Study	Treatment	Target	Population
No study fulfilling criteria for inclusion was found	-	-	-

Table S3.1.14: Cytokine release syndrome CRS (associated with severe SARS-CoV-2 infection, Macrophage activating syndrome)

Study	Treatment	Target	Population
Rodriguez-Bano 2020 (SAM-COVID-19) (74)	Tocilizumab	IL-6R	adult patients with laboratory-confirmed COVID-19 infection by real-time polymerase chain reaction (RT-PCR) assay and admitted to hospital. COVID-19 infection- with at least one clinical criterion and one laboratory criterion suggestive of hyperinflammatory state<br>clinical criteria:<br>a) temperature ≥38°C and b) increase in oxygen support required to achieve O2 saturation >92%.<br>laboratory criteria:<br>a) ferritin >2000 ng/mL or increase >1000 ng/mL since admission, b) D-dimers >1500 mg/ mL (or doubled), and c) IL6 >50 pg/mL.
Ip 2020 (75)	Tocilizumab	IL-6R	patients with COVID-19 infection confirmed by polymerase chain reaction (PCR) assay and admitted to hospital, did not die during first day of hospitalization, and were not discharged to home within 24 hours. For tocilizumab, exposure was defined as receipt of the drug within the ICU setting

Guaraldi/Meschiari 2020 (TESEO) (76)	Tocilizumab	IL-6R	adult patients with PCR-confirmed severe COVID-19 pneumonia defined as at least one of the following: presence of a respiratory rate of ≥30 breaths per minute, peripheral blood oxygen saturation (SaO2) of < 93% in room air, a ratio of arterial oxygen partial pressure (PaO2) to fractional inspired oxygen (FiO2) of < 300 mm Hg in room air, and lung infiltrates of > 50% within 24–48 h.
Biran/Ip 2020 (77)	Tocilizumab	IL-6R	adult patients with PCR-confirmed COVID-19 infection and requiring intensive care unit (ICU) support
Gupta 2020 (STOP-COVID) (78)	Tocilizumab	IL-6R	adult patients with laboratory confirmed COVID-19 infection admitted to an ICU directly attributable to COVID-19
Della-Torre 2020 (79)	Sarilumab	IL-6R	patients with PCR-confirmed severe COVID-19 infection as defined by either ≤ 92% of oxygen saturation (room air) or by a partial pressure of arterial oxygen/fraction of inspired oxygen (PaO2/FiO2) ratio ≤ 300 mmHg on supplemental oxygen, and a hyper-inflamed phenotype as defined by an elevation of lactate dehydrogenase (LDH) above the upper limit of normal (ULN), and by at least one of the following: C-reactive protein (CRP) ≥ 100 mg/L; IL-6 ≥ 40 pg/ml; or ferritin (≥ 900 ng/ml).
Ramiro (CHIC study) 2020 (80)	Tocilizumab	IL-6R	patients with PCR-confirmed severe COVID-19-associated cytokine storm syndrome (CSS), defined as rapid respiratory deterioration + at least two out of three biomarkers with important elevations (C-reactive protein >100mg/L; ferritin >900 µg/L; D-dimer >1500 µg/L), received high-dose intravenous methylprednisolone for 5 consecutive days. If no clinical improvement or worsening in respiratory status, TCZ was added on or after day 2.
Hermine 2020 (CORIMUNO-TOCI 1) (81)	Tocilizumab	IL-6R	adults with confirmed COVID-19 infection (positive on RT-PCR and/or typical chest CT scan) with moderate to severe pneumonia (WHO Clinical Progression Scale [WHO-CPS] score

			of 5 with O_2 levels of ≥3 L/min but without noninvasive ventilation [NIV] or mechanical ventilation [MV] or admission to intensive care unit)
Salvarani 2020 (RCT-TCZ-COVID-19) (82)	Tocilizumab	IL-6R	adults with PCR-confirmed COVID-19 pneumonia and presence of acute respiratory failure with PaO_2/FIO_2 ratio 200-300mmHg, an inflammatory phenotype defined by a temperature > 38 °C during the last 2 days, and/or serum CRP levels ≥10 mg/dL and/or CRP level increased to at least twice the admission measurement
Stone 2020 (phase 3, BACC Bay Tocilizumab Trial) (83)	Tocilizumab	IL-6R	adults with PCR- or IgM antibody assay confirmed COVID-19 infection and:<br>• fever (body temperature >38°C) within 72 hours before enrollment<br>• pulmonary infiltrates, or need for supplemental oxygen to maintain oxygen saturation > 92%<br>• at least one of the following laboratory criteria: CRP > 50 mg/L, ferritin > 500 ng/mL, d-dimer level > 1000 ng/mL, or a lactate dehydrogenase (LDH) level >250 U/L.
Salama 2020 (phase 3, EMPACTA) (84)	Tocilizumab	IL-6R	adult patients with COVID-19 pneumonia confirmed by PCR and radiographic imaging and SpO2 < 94% while on ambient air

Table S3.1.15: Tumor necrosis factor receptor-associated periodic fever syndrome (TRAPS)

Study	Treatment	Target	Population
No study fulfilling criteria for inclusion was found	-	-	-

Table S3.1.16: Chronic infantile neurological cutaneous and articular syndrome (CINCA)

Study	Treatment	Target	Population
No study fulfilling criteria for inclusion was found	-	-	-

Table S3.1.17: Late antibody-mediated kidney transplant rejection (ABMR)

Study	Treatment	Target	Population
Doberer 2020 (phase 2) (85)	Clazakizumab	IL-6	adult kidney transplant recipients with biopsy-proven late active or chronic active antibody-mediated rejection (ABMR) ≥365 days after transplantation according to Banff 2013/2015 (with or without C4d deposits along the peritubular capillaries), associated with a molecular pattern of ABMR in gene array analysis, detection of HLA class I and/or II antigen-specific antibodies (preformed and/or de novo donor-specific antibodies [DSA]) and eGFR >30 ml/min/1.73 m^2

3.2. Risk of bias analysis

Table S3.2.1: Psoriatic arthritis (PsA)

Study	Random sequence generation	Allocation concealment	Blinding of participants and personnel	Blinding of outcome assessment	Incomplete outcome data	Selective reporting	Other sources of bias	Summary	Comment
Mease 2016 (58)	Low	Low	Low	Low	Low	Low	Unclear	Unclear	

Table S3.2.2: Axial spondyloarthritis (axSpA)

Study	Random sequence generation	Allocation concealment	Blinding of participants and personnel	Blinding of outcome assessment	Incomplete outcome data	Selective reporting	Other sources of bias	Summary	Comment
Sieper 2014 (BUILDER-1) (59)	Low	Low	Low	Low	Low	Low	Low	Low	
Sieper 2015 (ALIGN) (60)	Low	Low	Low	Low	Low	Low	Low	Low	

Table S3.2.3: Osteoarthritis (OA)

Study	Random sequence generation	Allocation concealment	Blinding of participants and personnel	Blinding of outcome assessment	Incomplete outcome data	Selective reporting	Other sources of bias	Summary	Comment
Richette 2020 (61)	Low	Low	Low	Low	Low	Low	Low	Low	

Table S3.2.4: Polymyalgia rheumatica (PMR)

Study	Random sequence generation	Allocation concealment	Blinding of participants and personnel	Blinding of outcome assessment	Incomplete outcome data	Selective reporting	Other sources of bias	Summary	Comment
Lally 2016 (62)	High	High	High	High	Low	Low	Unclear	High	phase 2a, non-blinded, single-center open-label prospective study
Devauchelle-Pensec 2016 (TENOR) (63)	High	High	High	High	Low	Low	Low	High	phase 2 study, no control group, non-randomized design

Table S3.2.5: ANCA-associated vasculitis (GPA, MPA)

Study	Random sequence generation	Allocation concealment	Blinding of participants and personnel	Blinding of outcome assessment	Incomplete outcome data	Selective reporting	Other sources of bias	Summary	Comment
No study found	-	-	-	-	-	-	-	-	

Table S3.2.6: Remitting seronegative symmetric synovitis with pitting edema (RS3PE)

Study	Random sequence generation	Allocation concealment	Blinding of participants and personnel	Blinding of outcome assessment	Incomplete outcome data	Selective reporting	Other sources of bias	Summary	Comment
No study found	-	-	-	-	-	-	-	-	

Table S3.2.7: Systemic sclerosis associated interstitial lung disease (SSc-ILD)

Study	Random sequence generation	Allocation concealment	Blinding of participants and personnel	Blinding of outcome assessment	Incomplete outcome data	Selective reporting	Other sources of bias	Summary	Comment
Khanna 2020 (focuSSced) (64)	Low	Low	Low	Low	Low	Low	Low	Low	

Table S3.2.8: Idiopathic inflammatory myopathies (IIM)

Study	Random sequence generation	Allocation concealment	Blinding of participants and personnel	Blinding of outcome assessment	Incomplete outcome data	Selective reporting	Other sources of bias	Summary	Comment
NCT02043548 (phase 2, not published) (65)	-	-	-	-	-	-	-	-	Not fully published

Table S3.2.9: Systemic lupus erythematosus (SLE)

Study	Random sequence generation	Allocation concealment	Blinding of participants and personnel	Blinding of outcome assessment	Incomplete outcome data	Selective reporting	Other sources of bias	Summary	Comment
Wallace 2017 (phase 2, BUTTERFLY) (66)	Low	Low	Low	Low	Unclear	Unclear	Low	Unclear	200 mg dosage group terminated prematurely due to safety issues
Rovin 2016 (phase 2) (67)	Unclear	Unclear	Low	Low	Low	Low	Low	Unclear	Randomization sequence generation and allocation not reported
NCT02437890 (phase 2, not published) (68)	-	-	-	-	-	-	-	-	Not fully published

Table S3.2.10: Primary Sjögren’s syndrome (pSS)

Study	Random sequence generation	Allocation concealment	Blinding of participants and personnel	Blinding of outcome assessment	Incomplete outcome data	Selective reporting	Other sources of bias	Summary	Comment
Felten 2020 (69)	Low	Low	Low	Low	Low	Low	Low	Low	

Table S3.2.11: Amyloid A (AA)-Amyloidosis (AAA)

Study	Representative-ness	Selection of non-exposed cohort	Ascertainment of exposure	Demonstration of outcome of interest	Comparability	Assessment of outcome	Follow-up length	Adequacy of follow-up of cohorts	Summary	Comment
Okuda 2014 (70)*	Low	Low	Low	Low	Low	Low	Low	Low	Low	
Okuda 2018 (71)*	Low	Low	Low	Low	Low	Low	Low	Low	Low	
* risk of bias assessment using the Newcastle-Ottawa Scale (NOS) for Case-control studies										

Table S3.2.12: Multiple Myeloma (MM)

Study	Random sequence generation	Allocation concealment	Blinding of participants and personnel	Blinding of outcome assessment	Incomplete outcome data	Selective reporting	Other sources of bias	Summary	Comment
San-Miguel 2014 (phase 2) (72)	Unclear	Unclear	High	High	Low	Low	Low	High	Open label
Brighton 2019 (phase 2) (73)	Low	Low	Low	Low	Low	Low	Low	Low	

Kastrati K, et al. RMD Open 2022; 8:e002359. doi: 10.1136/rmdopen-2022-002359

Table S3.2.13: Refractory relapsing polychondritis

Study	Random sequence generation	Allocation concealment	Blinding of participants and personnel	Blinding of outcome assessment	Incomplete outcome data	Selective reporting	Other sources of bias	Summary	Comment
No study found	-	-	-	-	-	-	-	-	

Table S3.2.14.1: Cytokine release syndrome CRS (associated with severe SARS-CoV-2 infection, Macrophage activating syndrome): Cohort studies/historically controlled comparison

Study	Representative-ness	Selection of non-exposed cohort	Ascertainment of exposure	Demonstration of outcome of interest	Comparability	Assessment of outcome	Follow-up length	Adequacy of follow-up of cohorts	Summary	Comment
Rodriguez-Bano 2020 (SAM-COVID) (74)	Low	Low	Low	Low	Low	Low	Low	Low	Low	
Ip 2020 (75)	Low	Low	Low	Low	Low	Low	Low	Low	Low	
Guaraldi/Meschiari 2020 (TESEO) (76)	Low	Low	Low	Low	Low	Low	Low	Low	Low	

Biran/Ip 2020 (77)	Low	Low	Low	Low	Low	Low	Low	Low	Low	
Gupta 2020 (STOP-COVID) (78)	Low	Low	Low	Low	Low	Low	Low	Low	Low	
Della-Torre 2020 (79)	Low	Low	Low	Low	Low	Low	Low	Low	Low	
Ramiro (CHIC study) 2020 (80)	Low	Low	Low	Low	Low	Low	Low	Low	Low	

Table S3.2.14.2: Cytokine release syndrome CRS (associated with severe SARS-CoV-2 infection, Macrophage activating syndrome): RCTs

Study	Random sequence generation	Allocation concealment	Blinding of participants and personnel	Blinding of outcome assessment	Incomplete outcome data	Selective reporting	Other sources of bias	Summary	Comment
Hermine 2020 (CORIMUNO-TOCI 1) (81)	Low	Low	High	Unclear	Low	Low	Low	High	Open label
Salvarani 2020 (RCT-TCZ-COVID-19) (82)	Low	Low	High	Low	Unclear	Low	Low	High	Open label; trial was prematurely interrupted after an interim analysis for futility

Stone 2020 (phase 3, BACC Bay Tocilizumab Trial) (83)	Low	Low	Low	Low	Low	Low	Low	Low	
Salama 2020 (phase 3, EMPACTA) (84)	Low	Low	Low	Low	Low	Low	Low	Low	

Table S3.2.15: Tumor necrosis factor receptor-associated periodic fever syndrome (TRAPS)

**Study**	**Random sequence generation**	**Allocation concealment**	**Blinding of participants and personnel**	**Blinding of outcome assessment**	**Incomplete outcome data**	**Selective reporting**	**Other sources of bias**	**Summary**	**Comment**
No study found	-	-	-	-	-	-	-	-	

Table S3.2.16: Chronic infantile neurological cutaneous and articular syndrome (CINCA)

**Study**	**Random sequence generation**	**Allocation concealment**	**Blinding of participants and personnel**	**Blinding of outcome assessment**	**Incomplete outcome data**	**Selective reporting**	**Other sources of bias**	**Summary**	**Comment**
No study found	-	-	-	-	-	-	-	-	

Table S3.2.17: Late antibody-mediated kidney transplant rejection (ABMR)

Study	Random sequence generation	Allocation concealment	Blinding of participants and personnel	Blinding of outcome assessment	Incomplete outcome data	Selective reporting	Other sources of bias	Summary	Comment
Doberer 2020 (phase 2) (85)<br>***part A****: 12-week randomized, placebo-controlled study period. part B: 40-week open-label extension, all participants received CLZ*	Low	Low	Low	Low	Low	Low	Low	Low/High*	randomized pilot trial to evaluate safety (primary endpoint) and efficacy (secondary endpoint analysis) of CLZ.<br>*RoB assessment regarding part A of study: low; RoB part B: high (open label)

3.3. Baseline characteristics

3.3.1: Psoriatic arthritis (PsA)

Table S3.3.1.1: Baseline characteristics of trials investigating IL-6R/L blockers in PsoA.

Study	Treatment	No. of patients (n)	Mean age (years)	Mean disease duration (years)	Mean SJC 66	Mean TJC 68	Mean EGA	Mean CRP (mg/L)	PASI (mean)	Dactylitis (%)	Enthesitis (%)*	Mean HAQ	Mean mTSS
Mease 2016 (58)	Placebo ± MTX	41	48.0	8.5	11.2	21.2	58.2	11.0	7.9	41.5	80.5	1.4	
	CLZ SC 25 mg Q4W ± MTX	41	49.8	9.6	12.4	23.0	64.0	13.2	9.1	36.6	75.6	1.4	
	CLZ SC 100 mg Q4W ± MTX	42	49.3	5.6	13.8	19.0	62.5	17.4	9.5	28.6	83.3	1.3	
	CLZ SC 200 mg Q4W ± MTX	41	44.7	4.7	10.8	16.6	57.8	16.2	8.7	31.7	75.6	1.4	

SJC 66: Swollen Joint Count (66 joints); TJC 68: Tender Joint Count (68 joints); EGA: Evaluator Global Assessment of disease activity; PASI: Psoriasis Area and Severity Index; mTSS: PsA modified total Sharp score
* based on Spondyloarthritis Research Consortium of Canada enthesitis index (SPARCC)

3.3.2: Axial spondyloarthritis (axSpA)

Table S3.3.2.1: Baseline characteristics of trials investigating IL-6R/L blockers in axSpA.

Study	Treatment	Study population	No. of patients (n)	Mean age (years)	Mean disease duration (years)	HLA-B27 positive (%)	BASDAI	SJ ≥1 (%)	CRP (mg/dL)	ASspiMRI total score (mean)
Sieper 2014 (BUILDER-1) (59)	Placebo	r-axSpA	51	42.7	7.5	88	6.8	59	1.7	
	TCZ 8 mg/kg Q4W		51	41.6	5.4	84	6.6	65	1.6	
Sieper 2015 (ALIGN) (60)	Placebo	r-axSpA	50	40.3	9.45	74.0			56.0*	8.8
	SAR SC 100 mg Q2W		49	42.4	8.50	78.7			55.1*	6.8
	SAR SC 150 mg Q2W		50	43.0	8.55	76.0			54.0*	7.8
	SAR SC 100 mg QW		52	40.4	7.13	78.8			55.8*	9.1
	SAR SC 200 mg Q2W		50	37.2	7.13	78.0			56.0*	9.2
	SAR SC 150 mg QW		50	41.1	5.55	81.6			54.0*	9.7

r-axSpA: radiographic axial spondyloarthritis, according to modified New York criteria; BASDAI: Bath Ankylosing Spondylitis Disease Activity Index; SJ: swollen joints; ASspiMRI: Ankylosing Spondylitis spine MRI-active score
* patients with CRP level ≤1.5 mg/dL (%)

3.3.3: Osteoarthritis (OA)

Table S3.3.3.1: Baseline characteristics of trials investigating IL-6R/L blockers in OA.

Study	Treatment	No. of patients (n)	Mean age (years)	Mean disease duration (years)	Mean VAS pain (0-100 mm)	Mean Morning stiffness (min)	PJ (mean)	SJ (mean)	Mean VAS PGA (0-100 mm)	Mean VAS PhGA (0-100 mm)	Mean FIHOA	Mean CHFS
Richette 2020 (61)	Placebo ± acetaminophen*	41	64.7	10.7	59.6	56.8	10.9	2.9	62.1	58.6	13.7	32.6
	TCZ 8 mg/kg (week 0 and week 4) ± acetaminophen*	42	64.1	9.1	57.6	33.4	12.5	2.9	60.3	57.6	13.2	29.8

VAS: Visual Analogue Scale; PJ: Painful joints (pressure); SJ: Swollen joints; PGA: Patient global assessment; PhGA: Physician global assessment; FIHOA: Functional Index for Hand Osteoarthritis; CHFS: Cochin Hand Function Scale score
* oral NSAIDs were not allowed until week 6 and were allowed thereafter

Kastrati K, *et al. RMD Open* 2022; 8:e002359. doi: 10.1136/rmdopen-2022-002359

3.3.4: Polymyalgia rheumatica (PMR)

Table S3.3.4.1: Baseline characteristics of trials investigating IL-6R/L blockers in PMR.

Study	Treatment	No. of patients (n)	Mean age (years)	Mean disease duration (days)	Mean ESR at diagnosis (mm/h)	Mean CRP at diagnosis (mg/dl)	Mean Initial prednisone dose (mg/day)	PMR-AS (ESR)	Patient VAS pain	Patient VAS fatigue	Patient VAS disease activity	Phys. VAS disease activity
Lally 2016 (62)	TCZ 8mg/kg Q4W for 1 year + rapid GC-12 wks taper	10	68		63.2	3.8[b]	16.5					
	Comparator group*	10	72		62.5	9.7[b]	16.5					
Devauchelle-Pensec 2016 (TENOR) (63)	TCZ 8 mg/kg week 0, 4 and 8	20	66.9[a]	99[a]	51.0[a]	65.1[a]		35.6[a]	6.4[a]	5.4[a]	6.6[a]	6.8[a]

PMR-AS: polymyalgia rheumatica activity score
* declined participation in the trial, or failed to meet inclusion criteria
[a] values reported as median
[b] levels provided are elevations above the upper limit of normal (ULN) of the laboratory reference range

Kastrati K, et al. RMD Open 2022; 8:e002359. doi: 10.1136/rmdopen-2022-002359

3.3.5: ANCA-associated vasculitis (GPA, MPA)

Table S3.3.5.1: Baseline characteristics of trials investigating IL-6R/L blockers in ANCA-associated vasculitis.

**Study**	**Treatment**	**Study population**	**No. of patients (n)**	**Mean age (years)**	**Mean disease duration (years)**	**CRP (mg/dL)**	**ESR (mm/h)**
No study found	-	-	-	-	-	-	-

BMJ Publishing Group Limited (BMJ) disclaims all liability and responsibility arising from any reliance placed on this supplemental material which has been supplied by the author(s)

3.3.6: Remitting seronegative symmetric synovitis with pitting edema (RS3PE)

Table S3.3.6.1: Baseline characteristics of trials investigating IL-6R/L blockers in RS3PE.

Study	Treatment	Study population	No. of patients (n)	Mean age (years)	Mean disease duration (years)	CRP (mg/dL)	ESR (mm/h)
No study found	-	-	-	-	-	-	-

Kastrati K, *et al. RMD Open* 2022; 8:e002359. doi: 10.1136/rmdopen-2022-002359

3.3.7: Systemic sclerosis associated interstitial lung disease (SSc-ILD)

Table S3.3.7.1: Baseline characteristics of trials investigating IL-6R/L blockers in SSc-ILD.

Study	Treatment	No. of patients (n)	Mean age (years)	Mean disease duration (months)	Mean mRSS	Mean FVC%	Mean DLCO%	Baseline SSc-ILD (%)	Mean CRP (mg/mL)	Mean ESR (mm/h)	Mean Platelet count ($\times10^9$/L)	Mean HAQ
Khanna 2020 (focuSSced) (64)	Placebo	106	49.3	23.1	20.4	83.9	76.8[a]	65 (68/104)	7.0	34.7[b]	298.7	1.3[d]
	TCZ 162 mg QW	104	47.0	22.2	20.3	80.3	74.4	67 (68/102)	8.9	34.8[c]	311.1	1.1

mRSS: modified Rodnan skin score; FVC: forced vital capacity (predicted); DLCO: diffusing capacity for carbon monoxide (predicted, hemoglobin corrected)
[a] n=105
[b] n=103
[c] n=100
[d] n=104

3.3.8: Idiopathic inflammatory myopathies (IIM)

Table S3.3.8.1: Baseline characteristics of trials investigating IL-6R/L blockers in IIM.

Study	Treatment	No. of patients (n)	Mean age (years)	Mean disease duration (years)	DM (%)	PM (%)
NCT02043548 (phase 2, not published) (65)	Placebo ± concomitant GC±csDMARDs±IVIG	18	50.4	NR	72.2	27.8
	TCZ 8mg/kg Q4W ± concomitant GC±csDMARDs±IVIG	18	52.3	NR	55.6	44.4
IVIG: Intravenous immunoglobulin; NR: not reported; DM: dermatomyositis; PM: polymyositis						

3.3.9: Systemic lupus erythematosus (SLE)

Table S3.3.9.1: Baseline characteristics of trials investigating IL-6R/L blockers in SLE.

Study	Treatment	No. of patients (n)	Mean age (years)	Mean SLE duration (years)	Mean LN duration (years)	Mean SLEDAI-2K score	BILAG A in ≥1 organ system (%)	BILAG B in ≥2 organ systems (%)	Mean PhGA score	Renal biopsy class III LN (%)	Renal biopsy class IV LN (%)
Wallace 2017 (phase 2, BUTTERFLY) (66)	Placebo ± GC+csDMARDs	45	42.3	9.1		9.5	44.4	55.6	1.6		
	PF-04236921 10 mg SC Q8W ± GC+csDMARDs	45	39.9	7.9		9.6	42.2	60.0	1.7		
	PF-04236921 50 mg SC Q8W ± GC+csDMARDs	47	38.3	7.5		9.0	34.0	70.2	1.6		
	PF-04236921 200 mg SC Q8W ± GC+csDMARDs *	46	41.3	8.6		10.1	54.3	56.5	1.8		
Rovin 2016 (phase 2) (67)	Placebo + GC+csDMARD	4	37.8	6.5	3.8	18.0			4.5	50	50
	SIR 10 mg/kg IV Q4W + GC+csDMARD	21	30.6	8.1	5.2	15.7			4.2	33.3	66.7
NCT02437890 (phase 2, not published) (68)	Placebo	62	42.3								
	ALX-0061 75 mg Q4W	64	42.0								
	ALX-0061 150 mg Q4W	62	41.8								
	ALX-0061 150 mg Q2W	62	39.2								

	ALX-0061 225 mg Q2W	62	42.0								

LN: lupus nephritis; SLEDAI-2K: Systemic Lupus Erythematosus Disease Activity Index 2000; BILAG: British Isles Lupus Assessment Group; PhGA: Physician global assessment
* treatment group terminated early due to safety issues

3.3.10: Primary Sjögren's syndrome (pSS)

Table S3.3.10.1: Baseline characteristics of trials investigating IL-6R/L blockers in pSS.

Study	Treatment	No. of patients (n)	Mean age (years)	Mean disease duration (months)	Median ESSDAI	Mean PhGA	Mean ESSPRI	Steroids at BL (%)	Other immune-modulatory drugs at BL (%)	Median CRP
Felten 2020 (69)	Placebo ± GC ±csDMARDs	55	54.8	4.9	10	5.1	6.4	9.1	10.9	4
	TCZ 8mg/kg IV Q4W ± GC ±csDMARDs	55	50.9	4.4	11	5.2	6.4	16.4	12.7	4.4

ESSDAI: European League Against Rheumatism Sjögren's Syndrome Disease Activity Index; ESSPRI: EULAR Sjögren's Syndrome Patient Reported Index; PhGA: Physician's global evaluation of systemic disease activity (Visual Numeric Scale)

3.3.11: Amyloid A (AA)-Amyloidosis (AAA)

Table S3.3.11.1: Baseline characteristics of trials investigating IL-6R/L blockers in AAA

Study	Treatment	No. of patients (n)	Median age (years)	Median disease duration (years)	Median SAA (μg/mL)	Median CRP (mg/dL)	Renal involvement (%)	GI symptoms or signs (%)	Cardiac involvement (%)	Median CDAI
Okuda 2014 (70)	TCZ	22	61.5	20.5	219.2	3.1	81.8	36.4	13.6	15.7
	TNF-i	32	68.5	18.0	143.6	2.0	31.3	28.1	3.1	19.1
Okuda 2018 (71)	All patients	199	65	NR	59.9	1.14	76.4	39.7	11.6	NR
	TCZ	66	NR	NR	NR	NR	NR	NR	NR	NR
	TNF-i	27	NR	NR	NR	NR	NR	NR	NR	NR
	ABA	4	NR	NR	NR	NR	NR	NR	NR	NR
SAA: serum amyloid A; GI: gastrointestinal; NR: not reported										

3.3.12: Multiple Myeloma (MM)

Table S3.3.12.1: Baseline characteristics of trials investigating IL-6R/L blockers in MM.

Study	Treatment	No. of patients (n)	Median age (years)	Type of myeloma IgG/IgA/Light chain/Biclonal (%)	ISS Staging I/II/III (%)	Cytogenetic abnormality: high risk (%)[a]	% Plasma cells, bone marrow biopsy/aspirate >30 (%)	Median hemoglobin (g/L)	Median platelet ($\times 10^9$/L)	Median Creatinine clearance (mL/min)
San-Miguel 2014 (phase 2) (72)*	VMP	54	70.0	68.5/18.5/ 11/2	5/41/ 54	10	68.5	101.50	225.5	56.40
	SIL 11 mg/kg IV Q3W + VMP	52	71.0	42/41/15/2	8/38/ 54	17	65	103.50	236.5	58.38
				high-risk cytogenetic abnormalities (%)[b]	ultra-high risk SMM[c]					
Brighton 2019 (phase 2) (73)	Placebo	42	62	82	41					
	SIL 15 mg/kg IV Q3W	43	62	65	23					

VMP: velcade (bortezomib)-melphalan-prednisone; ISS: International Staging System

* part 1 (single-arm lead-in for safety evaluation): VMP+Siltuximab 11 mg/kg IV Q3W
* part 2 (patients were randomized 1:1 to SIL+VMP or VMP): VMP+Siltuximab 8.3 mg/kg or 11 mg/kg Q3W
[a] high-risk abnormality defined as t(4;14), t(14;16), and del17p

[b] high-risk cytogenetic abnormalities defined as: t(4;14), t(14;16), 17p deletion by FISH; t(4;14), 17p deletion by karyotype
[c] ultra-highrisk SMM by IMWG 2014 criteria [60% plasma cells or highrisk FLC ratio (0.01 or 100) at baseline

3.3.13: Refractory relapsing polychondritis

Table S3.3.13.1: Baseline characteristics of trials investigating IL-6R/L blockers in refractory relapsing polychondritis.

Study	Treatment	Study population	No. of patients (n)	Mean age (years)	Mean disease duration (years)	CRP (mg/dL)	ESR (mm/h)
No study found	-	-	-	-	-	-	-

Kastrati K, *et al. RMD Open* 2022; 8:e002359. doi: 10.1136/rmdopen-2022-002359

3.3.13: Cytokine release syndrome CRS (associated with severe SARS-CoV-2 infection, Macrophage activating syndrome)

Table S3.3.14.1: Baseline characteristics of trials investigating IL-6R/L blockers in CRS (associated with severe SARS-CoV-2 infection, Macrophage activating syndrome): Cohort studies I

Study	Treatment	No. of patients (n)	Median age (years)	Comorbidity: hypertension (%)	Comorbidity: cardiac disease (%)	Comorbidity: obesity (%)	Comorbidity: chronic renal failure (%)	Comorbidity: chronic pulmonary disease (%)	Median days of symptoms	Fever (%)	Ferritin >2000 ng/mL (%)	D-dimers >1500 µg/mL (%)	Worsening in O_2 requirements (%)
Rodriguez-Bano 2020 (SAM-COVID) (74)	No treatment	344	69	50.9	18.0	11.4	3.8	10.8	8	58.7	49.0	61.7	66.9
	TCZ	88	66	34.1	12.5	14.3	0	6.8	10	47.7	32.2	52.4	92.0
	GC intermediate-high dose	117	71	52.1	17.9	17.1	2.6	15.4	10	55.6	43.6	49.1	74.4
	GC pulse dose	78	71	53.8	14.1	7.4	6.4	11.5	6	48.7	46.8	54.8	89.7
	GC + TCZ	151	65	48.3	11.3	17.2	0.7	11.3	11	51.0	51.0	55.7	90.1

Table S3.3.14.2: Baseline characteristics of trials investigating IL-6R/L blockers in CRS (associated with severe SARS-CoV-2 infection, Macrophage activating syndrome): Cohort studies II

Study	Treatment	No. of patients (n)	Median age (years)	Comorbidity: hypertension (%)	Comorbidity: coronary disease (%)	Comorbidity: obesity (%)	Comorbidity: renal failure (%)	Comorbidity: COPD/asthma (%)	Oxygenation < 94% (%)	Steroids (%)	HCQ+AZI (%)
Ip 2020 (75)	No TCZ	413	69	79	77	75	85	78	75	75	69
	TCZ*	134	62	21	23	25	15	22	25	25	31

HCQ: hydroxychloroquine; AZI: azithromycin
* TCZ administration of at least one dose, and if given after entering the ICU. TCZ was administered as a single dose in 104 (78%), with the majority receiving 400 mg (96%), followed by 800 mg (1%), 8 mg/kg (1%), 4 mg/kg (1%), and missing dosing (1%).

Table S3.3.14.3: Baseline characteristics of trials investigating IL-6R/L blockers in CRS (associated with severe SARS-CoV-2 infection, Macrophage activating syndrome): Cohort studies III

Study	Treatment	No. of patients (n)	Median age (years)	Median PaO2/FiO_2 (mmHg)	Median SOFA score	Duration of symptoms (median, days from symptom onset)
Guaraldi/Meschiari 2020 (TESEO) (76): *Characteristics of patients from all centres combined*	Standard care*	365	69	277	2	5
	TCZ* + Standard care	179	64	169	3	7

PaO2/FiO2: ratio of arterial oxygen partial pressure to fractional inspired oxygen; SOFA: Subsequent Organ Failure Assessment
* standard of care: supplemental oxygen, hydroxychloroquine, azithromycin, antiretrovirals, and low molecular weight heparin
** TCZ administered IV 8 mg/kg (up to a maximum of 800 mg) in two infusions, 12 h apart, or SC 162 mg in two simultaneous doses (ie, 324 mg in total), if IV was not available.

Kastrati K, et al. RMD Open 2022; 8:e002359. doi: 10.1136/rmdopen-2022-002359

Table S3.3.14.4: Baseline characteristics of trials investigating IL-6R/L blockers in CRS (associated with severe SARS-CoV-2 infection, Macrophage activating syndrome): Cohort studies IV

Study	Treatment	No. of patients (n)	Median age (years)	Comorbidity count ≥3 (%)	Fever (%)	Shortness of breath (%)	Oxygenation < 94% (%)	qSOFA score 2 (%)	Intubation or ventilator (%)	Steroids (%)	HCQ+AZI at BL(%)
Biran/Ip 2020 (77)	No TCZ*	420	65	35	71	73	49	6	93	45	46
	TCZ*	210	62	30	77	80	49	4	94	46	65

data reported as median; qSOFA: quickSOFA-Score
* propensity score-matched patients (variables used for matching: age, gender, diabetes, COPD or asthma, hypertension, cancer, renal failure, obesity, oxygenation 15 mg/dL, and intubation or mechanical ventilator support). Exposure to TCZ was defined as receipt of the drug as found in the electronic health record. The Pharmacy and Therapeutics Committee suggested one intravenous dose of 400 mg tocilizumab.

Table S3.3.14.5: Baseline characteristics of trials investigating IL-6R/L blockers in CRS (associated with severe SARS-CoV-2 infection, Macrophage activating syndrome): Cohort studies V

Study	Treatment	No. of patients (n)	Median age (years)	Comorbidity: hypertension (%)	Comorbidity: coronary disease (%)	Median BMI (kg/m²)	Symptom onset to ICU ≤3 days (%)	Fever >38°C (%)	PaO_2/FiO_2 ratio <200 mmHg (%)	HCQ at ICU (%)
Gupta 2020 (STOP-COVID) (78)	No TCZ*	3491	63	62.6	14.4	30.4	23.9	47.2	37.9	45.4
	TCZ IV/SC*	433	58	54.0	9.0	31.6	13.4	47.8	47.3	63.0

data reported as median and bevore IPW (inverse probability weighting)
* patients were categorized according to whether they received or did not receive tocilizumab during the first 2 days of ICU admission.

Table S3.3.14.6: Baseline characteristics of trials investigating IL-6R/L blockers in CRS (associated with severe SARS-CoV-2 infection, Macrophage activating syndrome): Cohort studies VI

Study	Treatment	No. of patients (n)	Median age (years)	Duration of symptoms before enrollment (days)	Non-invasive positive-pressure ventilation (%)	High-flow oxygen (FiO2 ≥40 mmHg)	PaO_2 /FiO_2 ratio <100 (%)	Fever >38°C (%)	CRP (mg/L)	LDH (IU/L)	CT-based lung consolidation (%)
Della-Torre 2020 (79)	Standard of care*	28	57	7	71	28	46	54	152	495	14.2[a]
	SAR** IV 400 mg + Standard of care	28	56	7	75	25	60	64	143	468	16.6[b]

data reported as median
* all patients received oral therapy with lopinavir/ritonavir, hydroxychloroquine and azithromycin as per local institutional standard of care at time of admission.
** SAR was initiated within 24hours from the fulfilment of inclusion criteria.
[a] n=6
[b] n=20

Table S3.3.14.7: Baseline characteristics of trials investigating IL-6R/L blockers in CRS (associated with severe SARS-CoV-2 infection, Macrophage activating syndrome): Historically controlled comparison

Study	Treatment	No. of patients (n)	Mean age (years)	Mean WHO Score	COPD (%)	Mean BMI (kg/m²)	Cardiovascular disease (%)	High-flow oxygen (%)	Mechanical ventilation (%)	Mean CO-RADS	Mean CRP (mg/L)	Chloroquine at BL (%)
Ramiro (CHIC study) 2020 (80)	Control group*	86	67	4.4	8	29.7	13	8	15	4.8	167	79
	Treated group** (TCZ 37/86;43%)	86	67	4.3	12	28.0	20	23	1	4.7	160	77

CO-RADS: COVID-19 CT Classification; WHO score (1-7): 1) non-hospitalised, able to resume normal activities; 2) non-hospitalised, but unable to resume normal activities; 3) hospitalised, not requiring oxygen therapy; 4) hospitalised, requiring additional oxygen therapy; 5) hospitalised, requiring high-flow nasal oxygen therapy, non-invasive mechanical ventilation or both; 6) hospitalised, requiring extracorporeal membrane oxygenation, mechanical ventilation or both; 7) death

BMI: body mass index; COPD: chronic obstructive pulmonary disease
* control patients with COVID-19-associated CSS (same definition) were retrospectively sampled from the pool of patients (n=350) admitted between 7 March and 31 March 2020, and matched 1:1 to treated patients on sex and age
** two-steps treatment: (1) methylprednisolone (MP) 250mg IV on day 1, followed by MP 80mg intravenously on days 2–5, and an option for a 2-day extension if considered necessary and safe; (2) escalation with TCZ, between day 2 and day 5 (single-dose TCZ, 8mg/kg body weight intravenous, max 800mg). Criteria for escalation with TCZ were lack of clinical improvement or worsening in respiratory status (assessed on WHO scale).

Table S3.3.14.8: Baseline characteristics of trials investigating IL-6R/L blockers in CRS (associated with severe SARS-CoV-2 infection, Macrophage activating syndrome): RCTs

Study	Treatment	No. of patients (n)	Median age (years)	Symptom onset to randomization (days)	Diabetes (%)	Hypertension (%)	COPD (%)	Respiratory rate (median bpm)	Median PaO_2/FIO_2 (mmHg)	Median CRP (mg/L)	Hydroxy-chloroquine at BL (%)
Hermine 2020 (CORIMUNO-TOCI 1) (81)	Usual care (UC)*	67	63.3	10	34	30.0[a]	5	26.0		127.0	
	TCZ** IV 8mg/kg on day 1 + UC	63	64.0	10	33	33[a]	5	24.0		119.5	
Salvarani 2020 (RCT-TCZ-COVID-19) (82)	Standard care (±TCZ as rescue)	66	60.0	8.0	13.6	43.9	3.0	20.0	268.2	6.5[b]	93.9
	Early administration of TCZ IV 8mg/kg on day 1 and 2nd dose after 12 hours	60	61.5	7.0	16.7	45.0	3.3	20.0	262.5	10.5[b]	88.3
Stone 2020 (phase 3,	Placebo + Standard care	82	56.5	10.0	37	46	9			94.3	

BACC Bay Tocilizumab Trial) (83)	TCZ IV 8mg/kg (max. 800 mg) + Standard care	161	61.6	9.0	28	50	9			116.0	
Salama 2020 (phase 3, EMPACTA) (84)	Placebo + Standard care	128	55.6[c]							143.40[c]	
	TCZ IV 8mg/kg (max. 800 mg) ***+ Standard care	249	56.0[c]							124.50[c]	

bpm: breaths per minute
* usual care: antibiotic agents, antiviral agents, corticosteroids, vasopressor support, anticoagulants
** additional administration of TCZ 400 mg IV on day 3 was recommended if oxygen requirement was not decreased by more than 50% (decision by treating physician)
*** up to one additional infusion may be given
[a] chronic cardiac disease
[b] values reported as mg/dL
[c] values reported as mean

3.3.15: Tumor necrosis factor receptor-associated periodic fever syndrome (TRAPS)

Table S3.3.15.1: Baseline characteristics of trials investigating IL-6R/L blockers in TRAPS.

Study	Treatment	Study population	No. of patients (n)	Mean age (years)	Mean disease duration (years)	CRP (mg/dL)	ESR (mm/h)
No study found	-	-	-	-	-	-	-

BMJ Publishing Group Limited (BMJ) disclaims all liability and responsibility arising from any reliance placed on this supplemental material which has been supplied by the author(s)

3.3.16: Chronic infantile neurological cutaneous and articular syndrome (CINCA)

Table S3.3.16.1: Baseline characteristics of trials investigating IL-6R/L blockers in CINCA.

Study	Treatment	Study population	No. of patients (n)	Mean age (years)	Mean disease duration (years)	CRP (mg/dL)	ESR (mm/h)
No study found	-	-	-	-	-	-	-

3.3.17: Late antibody-mediated kidney transplant rejection (ABMR)

Table S3.3.17.1: Baseline characteristics of trials investigating IL-6R/L blockers in ABMR.

Study	Treatment	No. of patients (n)	Median recipient age (years)	Median age of study patients (years)	HLA class I and II DSA (%)	Active ABMR (%)	Chronic/ active ABMR (%)	C4d-positive ABMR (%)	Median CRP (mg/dL)	Median eGFR (ml/min)	TTV load (copies/ml)
Doberer 2020 (phase 2) (85)<br>*part A: 12-week randomized, placebo-controlled study period. part B: 40-week open-label extension, all participants received CLZ*	Placebo + calcineurin- or mTOR inhibitor–based (triple) immunosuppressive therapy	10	31.4	39.6	20	0	100	30	0.42	39.2	6.0×10^5
	CLZ SC 25 mg Q4W + calcineurin- or mTOR inhibitor–based (triple) immunosuppressive therapy	10	37.4	47.2	20	20	80	40	0.13	40.5	7.2×10^4
TTV: Torque Teno virus											

3.4. Efficacy outcomes

3.4.1: Psoriatic arthritis (PsA)

Table S3.4.1.1: Efficacy outcomes of trials investigating IL-6R/L blockers in PsoA.

Study	Treatment	No. of patients (n)	Timepoint (weeks)	ACR20 (%)	ACR50 (%)	ACR70 (%)	ΔDAPSA (%)	MDA (%)	PASI 75 (%)	ΔHAQ	Dactylitis (%)*	ΔSPARCC	ΔmTSS
Mease 2016 (58)	Placebo ± MTX	41	16[a]/24[b]	29.3[a]/ 34.1[b]	7.3[a]/1 4.6[b]	2.4[a]/ 4.9[b]			14.6[a]/ 12.2[b]	-0.27[a]/ -0.26[b]	43.8[a]/61.5[b]	-2.0[a]/-2.4[b]	
	CLZ SC 25 mg Q4W ± MTX	41		46.3[a]/ 56.1[b]	29.3[a]/ 34.1[b]	17.1[a]/ 19.5[b]			12.2[a]/ 19.5[b]	-0.44[a]/ -0.46[b]	60.0[a]/42.9[b]	-3.3[a]/-4.7[b]	
	CLZ SC 100 mg Q4W ± MTX	42		52.4[a]/ 57.1[b]	35.7[a]/ 35.7[b]	14.3[a]/ 23.8[b]			16.7[a]/ 28.6[b]	-0.40[a]/ -0.43[b]	25.0[a]/18.2[b]	-3.0[a]/-3.4[b]	
	CLZ SC 200 mg Q4W ± MTX	41		39.0[a]/ 39.0[b]	17.1[a]/ 24.4[b]	4.9[a]/ 12.2[b]			4.9[a]/ 12.2[b]	-0.26[a]/ -0.34[b]	38.5[a]/33.3[b]	-2.9[a]/-3.3[b]	

DAPSA: Disease Activity Index for Psoriatic Arthritis; MDA: Minimal Disease Activity
* patients with dactylitis in those with dactylitis (≥1 tender digit) at baseline
[a] efficacy outcomes at week 16
[b] efficacy outcomes at week 24

Kastrati K, *et al. RMD Open* 2022; 8:e002359. doi: 10.1136/rmdopen-2022-002359

3.4.2: Axial spondyloarthritis (axSpA)

Table S3.4.2.1: Efficacy outcomes of trials investigating IL-6R/L blockers in axSpA.

Study	Treatment	No. of patients (n)	Timepoint (weeks)	ASAS 20 (%)	ASAS 40 (%)	ASAS 5/6 (%)	ASDAS partial rem. (%)	ΔASDAS (%)	ΔBASDAI (%)	ΔBASFI (%)	ΔBASMI (%)	ΔASspi-MRI total score	ΔCRP (mg/dL)
Sieper 2014 (BUILDER-1) (59)	Placebo	51	12	27.5	19.6	15.7	2.0						-0.17
	TCZ 8 mg/kg Q4W	51		37.3	11.8	25.5	0.0						-1.34
Sieper 2015 (ALIGN) (60)	Placebo	50	12	24.0	8.0	6.0	2.0	-0.4	-0.9		-0.2	-0.5	-3.7*
	SAR SC 100 mg Q2W	49		24.5	14.3	12.2	8.2	-0.5	-0.8		-0.2	-0.5	-1.2*
	SAR SC 150 mg Q2W	50		30.0	16.0	10.0	2.0	-0.8	-1.1		-0.2	-0.1	-5.8*
	SAR SC 100 mg QW	52		19.2	5.8	13.5	1.9	-1.1	-0.4		-0.4	0.1	-13.5*
	SAR SC 200 mg Q2W	50		30.0	18.0	14.0	2.0	-1.2	-0.9		-0.1	-0.3	-11.5*
	SAR SC 150 mg QW	50		38.0	20.0	32.0	8.0	-1.6	-1.2		-0.2	0.3	-14.3*

ASAS: Axial SpondyloArthritis international Society; ASDAS: Ankylosing Spondylitis Disease Activity Score; BASDAI: Bath Ankylosing Spondylitis Disease Activity Index; BASFI: Bath Ankylosing Spondylitis Functional Index; BASMI: Bath Ankylosing Spondylitis Metrology Index
* mg/L

3.4.3: Osteoarthritis (OA)

Table S3.4.3.1: Efficacy outcomes of trials investigating IL-6R/L blockers in OA.

Study	Treatment	No. of patients (n)	Timepoint (weeks)	ΔVAS pain	ΔMorning stiffness	ΔPJ	ΔSJ	ΔVAS PGA	ΔVAS PhGA	ΔFIHOA	ΔCHFS
Richette 2020 (61)	Placebo ± acetaminophen	41	4[a]/6[b]/8[c]/ 12[d]	-3.0[a]/ -9.7[b]/ -9.4[c]/ -11.6[d]	-11.9[a]/ -19.3[b]/ -17.2[c]/ -19.6[d]	-0.7[a]/ -2.4[b]/ -1.9[c]/ -1.6[d]	-0.7[a]/ -0.2[b]/ -0.8[c]/ -1.2[d]	-5.4[a]/-10.1[b]/ -10.6[c]/-12.9[d]	-4.2[a]/-8.0[b]/ -7.4[c]/-12.1[d]	0.3[a]/ 0.2[b]/ 0.5[c]/ -0.1[d]	0.2[a]/ -0.2[b]/ 0.4[c]/ -0.8[d]
	TCZ 8 mg/kg (week 0 and week 4) ± acetaminophen	42		-0.9[a]/ -8.3[b]/ -12.3[c]/ -13.5[d]	15.9[a]/ -2.3[b]/ -8.6[c]/-8.5[d]	-0.5[a]/ -2.0[b]/ -3.0[c]/ -2.6[d]	-0.2[a]/ -1.1[b]/ -1.6[c]/ -1.4[d]	-1.7[a]/-8.3[b]/ -10.4[c]/-13.4[d]	-3.7[a]/-7.3[b]/ -15.0[c]/-14.2[d]	0.4[a]/ -0.04[b]/ -0.3[c]/ -1.0[d]	1.1[a]/ 0.8[b]/ 0.3[c]/ -0.8[d]

[a] efficacy outcome at week 4
[b] efficacy outcome at week 6; primary endpoint: ΔVAS pain at week 6: -7.9 (SD 19.4) in TCZ and -9.9 (SD 20.1) in placebo; SD: standard deviation
[c] efficacy outcome at week 8
[d] efficacy outcome at week 12

3.4.4: Polymyalgia rheumatica (PMR)

Table S3.4.4.1: Efficacy outcomes of trials investigating IL-6R/L blockers in PMR.

Study	Primary / Secondary outcome	Timepoint (weeks)	Treatment arm	No. of patients (n)	Result	p / 95% CI
Lally 2016 (62)	% pat. with steroid-free remission at 6 months; %relapse at 12 months, mean cumulative prednisone dose (mg), mean duration of prednisone exposure (months)	26/52	TCZ 8mg/kg Q4W for 1 year + rapid GC-12 wks taper	9[a]	100%; 0%; 1,085.3mg; 3.9	PE: % pat. with steroid-free remission at 6 months:<0.0001; 0.03, 0.01, 0.002
			Comparator group	10	0%; 60%; 2,562.0mg; 14.1	
Devauchelle-Pensec 2016 (TENOR) (63)	% pat. with PMR-AS≤10; %PMR-AS<7;<br><br>PMR-AS, PMR-AS (ESR), median CRP (mg/dl), median ESR (mm/h), patient VAS pain, patient VAS fatigue, patient VAS disease activity, physician VAS disease activity, morning stiffness (minutes), EUL, SF-36: MCS, SF-36: PCS	12	TCZ 8 mg/kg week 0, 4 and 8	20	100%; 85%;<br><br>4.5, 4.7, 0.2 mg/dl, 2.00 mm/h, 1.7, 2.1, 2.0, 1.1, 4.0 min, 0.0, 47.7, 40.6	-<br><br>p Value Week 0 vs week 12: <0.001, <0.001, <0.001, <0.001, <0.001, <0.001, <0.001, <0.001, <0.001, 0.002, 0.058, <0.001

EUL: 0–3 scale for elevation of the upper limb
MCS: mental component summary of the SF36
PCS: physical component summary of the SF36
[a] one subject withdrew from study due to mild infusion reaction (after second TCZ infusion)

3.4.5: ANCA-associated vasculitis (GPA, MPA)

Table S3.4.5.1: Efficacy outcomes of trials investigating IL-6R/L blockers in ANCA-associated vasculitis.

**Study**	**Primary / Secondary outcome**	**Timepoint (weeks)**	**Treatment arm**	**No. of patients (n)**	**Result**	**p / 95% CI**
No study found	-	-	-	-	-	-

3.4.6: Remitting seronegative symmetric synovitis with pitting edema (RS3PE)

Table S3.4.6.1: Efficacy outcomes of trials investigating IL-6R/L blockers in RS3PE.

Study	Primary / Secondary outcome	Timepoint (weeks)	Treatment arm	No. of patients (n)	Result	p / 95% CI
No study found	-	-	-	-	-	-

Kastrati K, *et al. RMD Open* 2022; 8:e002359. doi: 10.1136/rmdopen-2022-002359

3.4.7: Systemic sclerosis associated interstitial lung disease (SSc-ILD)

Table S3.4.7.1: Efficacy outcomes of trials investigating IL-6R/L blockers in SSc-ILD.

Study	Primary / Secondary outcome	Timepoint (weeks)	Treatment arm	No. of patients (n)	Result	p / 95% CI
Khanna 2020 (focuSSced) (64)	ΔLSM in mRSS from BL to week 48; ΔLSM in mRSS from BL to week 24, % with improvement in mRSS from BL ≥20%/≥40%/≥60%, HAQ, ACR-CRISS (median); FVC% predicted change from BL (LSM, Intention-to-treat population), FVC% predicted change from BL (LSM, patients with SSc-ILD*), Δ from BL to week 48 in FVC (LSM ml, intention-to-treat population), Δ from BL to week 48 in FVC (LSM ml, SSc-ILD patients*); % pat. ≥15% decline in %DLCO predicted	48	Placebo	106	-4.4; -3.1, 50%/38%/23%, -0.06, 0.3; -4.6, -6.4, -190, -255, 10%	PE: ΔLSM in mRSS from BL to week 48: 0.10; 0.455, 0.0007/ 0.51/0.33, 0.45, 0.02, 0.0002, 0.0001, 0.0001, <0.0001, NA
			TCZ 162 mg QW	104	-6.1; -3.7, 72%/42%/17%, -0.11, 0.9; -0.4; 0.1, -24, -14, 9%	

LSM: least squares mean; BL: baseline; ACR-CRISS: American College of Rheumatology-Combined Response Index in Systemic Sclerosis
* placebo: n=68; TCZ-group: n=68

3.4.8: Idiopathic inflammatory myopathies (IIM)

Table S3.4.8.1: Efficacy outcomes of trials investigating IL-6R/L blockers in IIM.

Study	Primary / Secondary outcome	Timepoint (weeks)	Treatment arm	No. of patients (n)	Result	p / 95% CI
NCT02043548 (phase 2, not published) (65)	Mean Total Improvement Scores at Visits 2 Through 7 (during 6-month treatment period) *; time to first Definition of Improvement (DOI; median, days); Δ steroid dose prednisone equivalent from last visit to BL (mg); mean manual muscle test measures (0-150)	24	Placebo ± concomitant GC±csDMARD±IVIG	18	29.3; 55.5; 0; 137.3	PE: Mean Total Improvement Scores at Visits 2 Through 7 (during 6-month treatment period) *:0.86; 0.77; 0.40; 0.78
			TCZ 8mg/kg Q4W ± concomitant GC±csDMARD±IVIG	18	26.4; 42.4; 0; 130.7	
* total improvement score based on 2016 ACR/EULAR myositis response criteria.						

3.4.9: Systemic lupus erythematosus (SLE)

Table S3.4.9.1: Efficacy outcomes of trials investigating IL-6R/L blockers in SLE.

Study	Treatment	No. of patients (n)	Timepoint (weeks)	SRI response (%)	BICLA response (%)	SRI response (%)*	BICLA response (%)*	Severe BILAG flares (%)**	Mean % of proteinuria reduction from BL to wk 24	Reduction in proteinuria ≥50% from BL (%)	No eGFR worsening (%)
Wallace 2017 (phase 2, BUTTERFLY) (66)	Placebo + GC+csDMARDs	45	24	40.1	25.1	27.7	19.7	11.1			
	PF-04236921 10 mg SC Q8W + GC+csDMARDs	45		59.9	49.7	73.1	55.7	4.7			
	PF-04236921 50 mg SC Q8W + GC+csDMARDs	47		39.2	40.5	43.1	34.7	0.0			
Rovin 2016 (phase 2) (67)	Placebo + GC+csDMARDs	4	24						-73.6	0.0	75.0
	SIR 10 mg/kg IV Q4W + GC+csDMARDs	21							-37.1	20.0	45.0
NCT02437890 (phase 2, not published) (68)***	Placebo	62	24		46.8			12.9	6.17 ****		
	ALX-0061 75 mg Q4W	64			43.8			9.4	1.77****		
	ALX-0061 150 mg Q4W	62			38.7			9.7	1.03****		
	ALX-0061 150 mg Q2W	62			38.7			11.3	-3.02****		
	ALX-0061 225 mg Q2W	62			37.1			9.7	0.16****		

SRI: Systemic Lupus Erythematosus Responder Index; BICLA: BILAG-based Composite Lupus Assessment; BL: baseline; eGFR: estimated glomerular filtration rate
* summary of SRI and BICLA response at week 24 in enriched population: Placebo (n=33), 10 mg treatment arm (n=30), 50 mg treatment arm (n=38)
** defined as new BILAG A or two new BILAG B organ domain scores

*** primary endpoint defined as Modified British Isles Lupus Assessment Group (BILAG)-Based Composite Lupus Assessment (mBICLA) Score
**** Mean change From Baseline in Proteinuria at Week 24 and Week 48

3.4.10: Primary Sjögren's syndrome (pSS)

Table S3.4.10.1: Efficacy outcomes of trials investigating IL-6R/L blockers in pSS.

Study	Primary / Secondary outcome	Timepoint (weeks)	Treatment arm	No. of patients (n)	Result	Pr (diff>0)*
Felten 2020 (69)	Primary endpoint defined by combination of (1) a decrease of at least 3 points in ESSDAI, (2) no new moderate/severe activity in any ESSDAI domain and 3) no worsening in physician's global assessment on a visual numeric scale ≥1/10; 3-point decrease in ESSDAI, no new systemic complication, no worsening according to physician; ESSPRI at week 24	24	Placebo ± GC ± csDMARDs	55	63.6%; 70%, 84%, 84.8%, 6.2	PE: 0.86; 0.91, 0.79, 0.05, 0.125
			TCZ 8mg/kg IV Q4W ± GC ± csDMARDs	55	52.7%; 57.1%, 77.6%, 95.7%, 5.8	

* proportion difference: 95% credible interval (CrI) and the probability of difference >0 in favour of TCZ group (Pr[Toc >Pla])

3.4.11: Amyloid A (AA)-Amyloidosis (AAA)

Table S3.4.11.1: Efficacy outcomes of trials investigating IL-6R/L blockers in AAA.

Study	Primary / Secondary outcome	Timepoint (weeks)	Treatment arm	No. of patients (n)	Result	p[a]
Okuda 2014 (70)	1- and 5-year treatment retention rates (Kaplan–Meier method); median Δ SAA (μg/ml; median observation period: TCZ 22.5 months, TNF-i 21.0 months); median Δ eGFR (mL/min/1.73 m^2; median observation period: TCZ 22.5 months, TNF-I 21.0 months); mean Δ CDAI (last observation); mean Δ GC dose (mg/day, last observation)	described in "Primary / Secondary outcome"	TCZ	22	90.4%/90.4%; 219.2→5.0; 41.6→50.7; 16.04→7.98; 5.5→2.7	0.0154; 0.0194; 0.0062; 0.0201; 0.0057
			TNF-i	32	69.0%/34.3%; 143.6→38.1; 76.3→67.4; 19.11→12.31; 5.0→4.7	
Okuda 2018 (71)	%pat. with good response*	NA	TCZ	66	95.5	0.007
			TNF-i	27	74.1	
			ABA	4	75	

* varibales of survey questionnaire included: age, gender, family history, medical history, underlying diseases, histological evidence for the diagnosis, manifestation at the diagnosis, therapeutic modality and response, laboratory data [CRP, SAA, creatinine, albumin, urinary protein], and echocardiographic results.
[a] efficacy of TCZ was significantly superior to that of TNF inhibitors

3.4.12: Multiple Myeloma (MM)

Table S3.4.12.1: Efficacy outcomes of trials investigating IL-6R/L blockers in MM.

Study	Treatment	No. of patients (n)	Median treatment duration (months)	Overall response (CR or PR) (%)[a]	Complete response (%)	Partial response (%)	VGPR (%)[b]	Progressive disease (%)	100% M-protein response in serum (%)	100% M-protein response in urine (%)	Median time to first response (months)	Median PFS (months)*	1-year survival rate (%)
San-Miguel 2014 (phase 2) (72)	VMP	54	12.9	80	22	57	51	0	38	57	1.4	17	88
	SIL 13 mg/kg IV Q3W + VMP	52	12.5	88	27	61	71	0	61	100	0.8	17	88
				**ITT population PFS (days) Median**	**1-year PFS rate %**	**1-year PFS rate %; Risk factor <2**	**1-year PFS rate %; Risk factor ≥2**	**PFS events, %**					
Brighton 2019 (phase 2) (73)	Placebo	42	29.2	715.0	74.4	100.0	48.1	42.9					
	SIL 15 mg/kg IV Q3W	43		NE	84.5	89.3	79.8	32.6					

* PFS: progression-free survival
NE: not estimable
[a] overall response: complete response (CR) or partial response (PR) based on European Group for Blood and Marrow Transplantation (EBMT) criteria
[b] very good partial response (VGPR) based on International Myeloma Working Group (IMWG) criteria

3.4.13: Refractory relapsing polychondritis

Table S3.4.13.1: Efficacy outcomes of trials investigating IL-6R/L blockers in refractory relapsing polychondritis.

Study	Primary / Secondary outcome	Timepoint (weeks)	Treatment arm	No. of patients (n)	Result	p / 95% CI
No study found	-	-	-	-	-	-

3.4.14: Cytokine release syndrome CRS (associated with severe SARS-CoV-2 infection, Macrophage activating syndrome)

Table S3.4.14.1: Efficacy outcome of trials investigating IL-6R/L blockers in CRS (associated with severe SARS-CoV-2 infection, Macrophage activating syndrome): Cohort studies I

Study	Treatment	No. of patients (n)	Median follow-up without endpoint (days)	Primary outcome (%)[a]	Scale[b] ≤ 3 (%)	Scale 7 (death) (%)	Digestive tract bleeding	Secondary bacterial infection
Rodriguez-Bano 2020 (SAM-COVID) (74)	No treatment	344	20	20.1	81.1	11.9	0.6	10.3
	TCZ	88	21	11.4	90.9	2.3	1.1	12.5
	GC intermediate-high dose	117	21	23.1	78.6	18.8	1.4	8.7
	GC pulse dose	78	21	15.4	83.3	10.3	1.4	10.7
	GC + TCZ	151	20	26.5	80.8	12.6	2.0	12.0

[a] intubation or death (whichever occurred first)
[b] seven-point ordinal scale at day 21: 1 not hospitalized; 2 hospitalized without supplemental oxygen; 3 hospitalized with supplemental oxygen; 4 hospitalized and requiring supplemental oxygen with a high nasal flow cannula or non-invasive ventilation; 5 hospitalized and requiring mechanical ventilation; 6 hospitalized and requiring extracorporeal membrane oxygenation (ECMO) or invasive mechanical ventilation with amine support; 7 death).

Table S3.4.14.2: Efficacy outcome of trials investigating IL-6R/L blockers in CRS (associated with severe SARS-CoV-2 infection, Macrophage activating syndrome): Cohort studies II

Study	Primary / Secondary outcome	Timepoint (days)	Treatment arm	No. of patients (n)	Result	p/95% CI
Ip 2020 (75)	unadjusted 30-day mortality	30	No TCZ	413	56%	HR, 0.76 [95% CI, 0.57–1.00]
			TCZ	134	46%	

Table S3.4.14.3: Efficacy outcome of trials investigating IL-6R/L blockers in CRS (associated with severe SARS-CoV-2 infection, Macrophage activating syndrome): Cohort studies III

Study	Treatment	No. of patients (n)	Median Follow-up (days)	Mechanical ventilation (%)	Deaths after mechanical ventilation (%)	Death (%)	Cumulative probability of mechanical ventilation or death at day 14
Guaraldi/Meschiari 2020 (TESEO) (76): *Characteristics of patients from all centers combined*	Standard care	365	8	16	25	20	36.5
	TCZ + Standard care	179	12	18	15	7	22.6

Table S3.4.14.4: Efficacy outcome of trials investigating IL-6R/L blockers in CRS (associated with severe SARS-CoV-2 infection, Macrophage activating syndrome): Cohort studies IV

Study	Primary / Secondary outcome	Median follow-up (days)	Treatment arm	No. of patients (n)	Result	p/95% CI
Biran/Ip 2020 (77)	median overall survival from time of admission; % death; mechanical ventilation (TCZ yes vs no); hospital-related mortality (TCZ yes vs no)	22	No TCZ	420	19; 61%;	HR 0.71, (CI 0.56–0.89) log-rank p=0.0027;<br>NR<br>HR 0.63 (CI 0.46–0.85) p=0.0029<br>HR 0.64 (0.47–0.87) p=0.0040
			TCZ	210	23; 49%;	

Table S3.4.14.5: Efficacy outcome of trials investigating IL-6R/L blockers in CRS (associated with severe SARS-CoV-2 infection, Macrophage activating syndrome): Cohort studies V

Study	Primary / Secondary outcome	Median follow-up (days)	Treatment arm	No. of patients (n)	Result	Adjusted HR* (95% CI)
Gupta 2020 (STOP-COVID) (78)	primary analysis: death (%)<br>estimated 30-day mortality (%)	27	No TCZ*	3491	40.6<br>37.1	0.71 (0.56-0.92)
			TCZ IV/SC*	433	28.9<br>27.5	risk difference, 9.6% (95% CI: 3.1,16.0)

* adjusted for: age, sex, race, ethnicity, BMI, hypertension, diabetes, coronary artery disease, congestive heart failure, current tobacco use, active cancer, home medications (statin, angiotensin-converting enzyme inhibitor, angiotensin 2 receptor blocker), days from symptom onset to (ICU) admission, severity-of-illness covariates assessed on ICU admission (fever, renal/liver components of SOFAscore, PaO2/FiO2 ratio, # vasopressors received, white blood cell count, and inflammation (assessed by CRP, IL-6, and ferritin), and therapies received on ICU admission (HCQ, AZI, GCs, therapeutic anticoagulants, etc.)

Table S3.4.14.6: Efficacy outcome of trials investigating IL-6R/L blockers in CRS (associated with severe SARS-CoV-2 infection, Macrophage activating syndrome): Cohort studies VI

Study	Primary / Secondary outcome	Timepoint (days)	Treatment arm	No. of patients (n)	Result	p/95% CI
Della-Torre 2020 (79)	% clinical improvement; time to clinical improvement (days); % death; time to death (days); % live discharge; time to discharge (days); % mechanical ventilation; time to mechanical ventilation (days); % fever resolution; time to fever resolution (days); % CRP normalisation; time to CRP normalisation (days); median time to clinical improvement in patients with lung consolidation <17% (days)	28	Standard of care	28	64; 19; 18; 4; 60; 13; 25; 0.99; 3; 100; 4; 61; 12; 24	% clinical improvement: 0.99; 0.89; 0.42; 0.006; 0.99; 0.35; 0.52; 0.99; <0.0001; 0.06; <0.0001; 0.01
			SAR IV 400 mg + Standard of care	28	60; 18; 7; 19; 60; 12; 21; 5; 100; 1; 86; 6; 10	

Table S3.4.14.7: Efficacy outcomes of trials investigating IL-6R/L blockers in CRS (associated with severe SARS-CoV-2 infection, Macrophage activating syndrome): Historically controlled comparison

Study	Primary / Secondary outcome	Timepoint (days)	Treatment arm	No. of patients (n)	Result	p/95% CI
Ramiro (CHIC study) 2020 (80)	Clinical improvement (2 points) in WHO score (n); hospital mortality (n); mechanical ventilation (n); Clinical improvement (1 point) in WHO score (n); % WHO score 2 at day 7 (no hospitalization); % WHO score 2 at day 14; duration of mechanical ventilation in survivors (days); duration of hospitalisation in survivors and discharged (days)	described in "Primary / Secondary outcome"	Control group	86	44; 41; 24; 45; 11; 24; 18.8; 15.9	Clinical improvement (2 points) in WHO score (n): 0.0025; 0.0004; 0.0003; 0.0003; <0.0001; <0.0001; 0.5809; 0.0196
			Treated group (TCZ 37/86;43%)	86	64; 14; 10; 69; 21; 58; 16.3; 10.8	
						**Univariable analysis HR /coefficient (95%CI)**
	Clinical improvement (2 points) in WHO score; hospital mortality; mechanical ventilation; clinical		Effect of treatment versus control			1.79 (1.20, 2.67); 0.35 (0.19, 0.65);

	improvement (1 point) in WHO score; independence from oxygen therapy; duration of mechanical ventilation in survivors; duration of hospitalisation in survivors		0.29 (0.14, 0.60);<br>1.95 (1.33, 2.87);<br>1.80 (1.19, 2.71);<br>-2.57 (-12.08, 6.93);<br>-5.23 (-8.99, -1.46)

Table S3.4.14.8: Efficacy outcomes of trials investigating IL-6R/L blockers in CRS (associated with severe SARS-CoV-2 infection, Macrophage activating syndrome): RCTs

Study	Primary / Secondary outcome	Timepoint (days)	Treatment arm	No. of patients (n)	Result	p/95% CI
Hermine 2020 (CORIMUNO-TOCI 1) (81)	patients (n) with scores > 5 on WHO-CPS on day 4*; survival without need of ventilation including noninvasive ventilation at day 14 (primary outcome by day 14, cumulative incidence)**; mechanical ventilation or death by day 14***; survival day 14 (%;95% CI); survival day 28 (%;95% CI)	4/14/28	Usual care (UC)	67	19 (28%); 36%; 27%; 91% (84 to 98); 88% (80 to 96)	* median posterior absolute risk difference (ARD) -9.0%; 90% CI −21.0 to 3.1;<br>difference:<br>**-12 (-28 to 4), HR 0.58 (90% CrI, 0.33-1.00);<br>***-9 (-24 to 5)
			TCZ + UC	63	12 (19%); 24%; 17%; 89% (81 to 97); 89% (81 to 97)	
Salvarani 2020 (RCT-TCZ-COVID-19) (82)	PE: clinical worsening within 14 days[a]; overall events at 14 d: admissions to ICU, deaths,	14/30	Standard care	66	27.0%; 7.9%, 1.6%, 57.1%, 7.9%, 1.6%, 92.1%	Relative ratio 1.05 (0.59,1.86) p=0.87; 1.26 (0.41,3.91),

	discharges; overall events at 30 d: admissions to ICU, deaths, discharges		Early TCZ	60	28.3%; 10.0%, 1.7%, 56.7%, 10.0%, 3.3%, 90.0%	1.05 (0.07,16.4), 0.99 (0.73,1.35), 1.26 (0.41,3.91), 2.10 (0.20,22.6), 0.98 (0.87,1.09)
Stone 2020 (phase 3, BACC Bay Tocilizumab Trial) (83)	Primary outcome: mechanical ventilation or death at day 14 (%, 95%CI), mechanical ventilation or death day 28 (%, 95%CI); clinical worsening on ordinal scale[b] at day 14 (%;95%CI), clinical worsening on ordinal scale day 28 (%;95%CI) discontinuation of supplemental oxygen among patients receiving it at baseline at day 14 (%;95%CI), discontinuation of supplemental oxygen among patients receiving it at baseline at day 28 (%;95%CI) median duration of receipt of supplemental oxygen (days), median duration of mechanical ventilation	14/28	Placebo + Standard care	82	10.0 (5.1,18.9), 12.5 (6.9,22.0) 14.9 (8.7,24.7), 17.4 (10.7,27.7) 78.8 (68.3,87.7), 84.9 (75.2,92.2) 3.9, 27.9 15.8	PE: HR 0.83 (0.38,1.81) p=0.64 HR 1.11 (0.59,2.10) p=0.73 HR 0.94 (0.67,1.30) p=0.69 HR 0.97 (0.50,1.88)
			TCZ + Standard care	161	9.9 (6.2,15.7), 10.6 (6.7,16.6) 18.0 (12.9,24.9), 19.3 (14.0,26.2) 75.4 (67.9,82.2), 82.6 (75.9,88.4)	

	admission to ICU or death (days)				4.0, 15.0<br><br>15.9	
Salama 2020 (phase 3, EMPACTA) (84)	Primary outcome: mechanical ventilation or death (%;95% CI)<br>median time to hospital discharge or readiness for discharge (days)<br>median time to improvement in clinical status (days)[c]<br>median time to clinical failure (days)<br>death no. (%;95% CI)		Placebo + Standard care	128	19.3 (13.3,27.4)<br>7.5<br>7.0<br>NE<br>11 (8.6%; CI 4.9,14.7)	HR 0.56 (0.33,0.97) p=0.04<br>HR 1.16 (0.91,1.48)<br>HR 1.15 (0.90,1.48)<br>HR 0.55 (0.33, 0.93)<br>weighted difference: 2.0 (-5.2, 7.8)
			TCZ + Standard care	249	12.0 (8.5,16.9)<br>6.0<br>6.0<br>NE<br>26 (10.4%; CI 7.2, 14.9)	

NE: not estimated
[a] primary end point: defined by occurrence of 1 of the following events, whichever occurred first: a) admission to ICU with mechanical ventilation; b) death; c) paO_2/FIO_2 ratio <150 mmHg.
[b] worsening defined as increase in score on the ordinal clinical improvement scale by at least 1 point among patients receiving supplemental oxygen at baseline or at least 2 points among patients not receiving supplemental oxygen at baseline.
[c] clinical status was determined with the use of the seven-category ordinal scale

3.4.15: Tumor necrosis factor receptor-associated periodic fever syndrome (TRAPS)

Table S3.4.15.1: Efficacy outcomes of trials investigating IL-6R/L blockers in TRAPS.

Study	Treatment	Study population	No. of patients (n)	Mean age (years)	Mean disease duration (years)	CRP (mg/dL)	ESR (mm/h)
No study found	-	-	-	-	-	-	-

3.4.16: Chronic infantile neurological cutaneous and articular syndrome (CINCA)

Table S3.4.16.1: Efficacy outcomes of trials investigating IL-6R/L blockers in CINCA.

**Study**	**Treatment**	**Study population**	**No. of patients (n)**	**Mean age (years)**	**Mean disease duration (years)**	**CRP (mg/dL)**	**ESR (mm/h)**
No study found	-	-	-	-	-	-	-

3.4.17: Late antibody-mediated kidney transplant rejection (ABMR)

Table S3.4.17.1: Efficacy outcomes of trials investigating IL-6R/L blockers in ABMR.

Study	Efficacy outcomes	Timepoint (weeks)	Treatment arm	No. of patients (n)	Result	p/95% CI
Doberer 2020 (phase 2) (85)	*HLA Antibody and Ig levels*:<br>DSA MFI mean fluorescence intensity (MFI) week 12/52<br>*Evolution of Rejection:*<br>-51-week-biopsy decrease in molecular ABMR/“all rejection” scores<br>-T cell–mediated rejection scores<br>*Clinical outcomes:*<br>mean slope of eGFR	12[a]/52[b]	Placebo + calcineurin- or mTOR inhibitor–based (triple) immunosuppressive therapy	10	103%[a]<br>-2.43 (95% CI: -3.4, -1.46)[a]<br>-0.64 (95% CI: -1.13, -0.14)[b]	p=0.035[a]; p=0.001[b]<br>p=0.020[b]/ p=0.037[b]<br>p=0.97[b]/ p=0.93[b]<br>p=0.04[a]<br>p=0.37[b]
			CLZ SC 25 mg Q4W + calcineurin- or mTOR inhibitor–based (triple) immunosuppressive therapy	10	77%[a]<br>-0.96 (95% CI: -1.96, 0.03)[a]<br>-0.29 (95% CI: -0.85, 0.26)[b]	

[a] part A
[b] part B

Section 4: Characteristics of articles and abstracts included: Safety aspects of interleukin-6 pathway inhibition

4.1. Cardiovascular events

4.1.1: Composite Outcome (MACE): Comparison between IL-6R/L blockers and different bDMARDs (observational studies/randomized controlled trials)

Table S4.1.1.1: Baseline characteristics of observational studies investigating IL-6R/L blockers regarding MACE (major adverse cardiac events).

Study	Registry	Inclusion criteria	Exclusion criteria
Kim 2017 (86)<br>Kim 2018 (87)	US health care claims databases: Medicare, IMS PharMetrics Plus, Truven MarketScan	RA (ICD9 codes) patients, ≥ 18 years, starting TCZ or TNFi after failure of at least 1 bDMARD or tsDMARD	Nursing home residents, patients with HIV/ AIDS, malignancy other than NMSC, end-stage renal disease, patients undergone dialysis or renal transplant prior index date; patients who received RTX, patients with hospitalizations for MI, stroke, ACS, or heart failure in the 90 days prior index date
Xie 2019 (88)	US health care claims databases: Medicare, MarketScan	RA (ICD9 codes) patients, initiated at least 1 bDMARD for RA	ICD9-CM diagnosis code(s) for other autoimmune/inflammatory diseases, including inflammatory bowel disease, psoriatic arthritis, psoriasis, or ankylosing spondylitis, to ensure that biologics were used to treat RA; 2) had any ICD-9-CM diagnosis code for past myocardial infarction (MI), stroke, ICD-9 procedure code or current procedural terminology code for percutaneous coronary intervention or

			coronary artery bypass grafting; history of malignancy (except non-melanoma skin cancer), HIV infection, or organ transplantation.

Table S4.1.1.2: Risk of bias analysis (Newcastle-Ottawa Scale [NOS] for Cohort studies)

**Study**	**Representative-ness of exposed cohort**	**Selection of non-exposed cohort**	**Ascertainment of exposure**	**Outcome not presented at start**	**Comparability of cohort**	**Assessment of outcome**	**Follow-up length**	**Adequacy of follow-up**	**Summary**
Kim 2017 (86)	Low	Low	High	Low	Low	High	Low	Low	High
Kim 2018 (87)	Low	Low	High	Low	Low	High	Low	Low	High
Xie 2019 (88)	Low	Low	High	Low	Low	High	Low	Low	High

Table S4.1.1.3: Safety outcomes of observational studies regarding MACE.

Study	Treatment group		N patients	N events	Incidence rate (95% CI)	age/gender aHR (I vs C)	aHR (I vs C)	Adjusted for
Kim 2017 (86)	Combined TCZ		9,218	36	0.52/100PY (0.37; 0.71)	NR	0.84 (0.56; 1.26)	demographics (age, sex, region, race/ethnicity [only available in the Medicare data]), prior DMARD use, cardiovascular comorbidities, other chronic diseases, cardiovascular medications, other long-term medications, and markers of health care utilization intensity
	Combined TNF-i		18,810	89	0.59/100PY (0.47; 0.72)	NR	REF	
Kim 2018 (87)	Combined TCZ		6,237	32	0.70/100PY (0.49; 0.97)	NR	0.82 (0.55; 1.22)	
	Combined ABA		14,685	112	0.96/100PY (0.79; 1.15)	NR	REF	
Xie 2019 (88)	Medicare	TCZ	7,369	104	12.9/1000 PY (10.7;15.7)	NR	REF	demographic characteristics (age, sex), co-morbidities (history of CVD, heart failure, atrial fibrillation, abdominal aortic aneurism, peripheral arterial disease, diabetes mellitus, hyperlipidemia, hypertension, obesity, chronic kidney disease, chronic obstructive pulmonary disease, fibromyalgia, any hospitalized infection), health care utilization (any hospitalization, number of physician visits), drug use (methotrexate, nonsteroidal antiinflammatory drugs [NSAIDs], statin potency, other lipid-lowering drug use at baseline, number of
		all TNF-i	6,895	600	15.0/1000 PY (13.9;16.3)	NR	1.27 (1.02;1.59)	
		ABA	11,979	199	13.7/1000 PY (11.9;15.7)	NR	1.01 (0.79;1.28)	
		RTX	5,472	105	16.6/1000 PY (13.7;20.1)	NR	1.16 (0.89;1.53)	
	MarketScan	TCZ	4,523	21	5.2/1000 PY (3.4;7.9)	NR	REF	
		all TNF-i	40,153	222	5.8 (5.1;6.6)	NR	1.29 (0.81;2.05)	
		ABA	8,105	67	8.7 (6.9;11.1)	NR	1.60 (0.98; 2.61)	

		RTX	2,997	35	11.0 (7.9;15.3)	NR	1.69 (0.98; 2.90)	biologics used prior to initiation of treatment, and oral steroid dose in the 6 months before initiation of treatment), smoking
I: intervention; C: control; aHR: adjusted hazard ratio; HR: hazard ratio; REF: reference; PY: patient years; NR: not reported								

Table S4.1.1.4: Baseline characteristics of RCTs investigating IL-6R/L blockers regarding MACE (major adverse cardiac events)

Study	Treatment	Target	Population
Giles 2020 (ENTRACTE) (89)	Tocilizumab vs. Etanercept	IL-6R vs. TNF	csDMARD-IR; TNFi-IR

Table S4.1.1.5: Risk of bias analysis (Cochrane Risk of Bias Tool for RCTs)

Study	Random sequence generation	Allocation concealment	Blinding of participants and personnel	Blinding of outcome assessment	Incomplete outcome data	Selective reporting	Other sources of bias	Summary	Comment
Giles 2020 (ENTRACTE) (89)	Low	Low	High	Unclear	Low	Low	Low	High	Open label

Table S4.1.1.6: Safety outcomes of RCTs regarding MACE.

Study	Treatment group	N patients	N events	Incidence rate (95% CI)	age/gender aHR (I vs C)	HR	Adjusted for
Giles 2020 (ENTRACTE) (89)	TCZ	1,538	83	1.82/100 PY (1.46;2.24)	NR	1.05 (0.77;1.43)	stratified by previous exposure to TNF-i therapy and history of cardiovascular (CV) events
	ETN	1,542	78	1.70/100 PY (1.35;2.10)	NR	REF	

4.1.2: Myocardial infarction: Comparison between IL-6R/L blockers and different bDMARDs (observational studies/randomized controlled trials)

Table S4.1.2.1: Baseline characteristics of observational studies investigating IL-6R/L blockers regarding myocardial infarction.

<table>
<tr><th>Study</th><th>Registry</th><th>Inclusion criteria</th><th>Exclusion criteria</th></tr>
<tr><td>Kim 2017 (86)</td><td rowspan="2">US health care claims databases: Medicare, IMS PharMetrics Plus, Truven MarketScan</td><td rowspan="2">RA (ICD9 codes) patients, ≥ 18 years, starting TCZ or TNFi after failure of at least 1 bDMARD or tsDMARD</td><td rowspan="2">Nursing home residents, patients with HIV/ AIDS, malignancy other than NMSC, end-stage renal disease, patients undergone dialysis or renal transplant prior index date; patients who received RTX, patients with hospitalizations for MI, stroke, ACS, or heart failure in the 90 days prior index date</td></tr>
<tr><td>Kim 2018 (87)</td></tr>
<tr><td>Zhang 2016 (90)</td><td>US health care claims database: Medicare</td><td>RA (ICD codes); initiated an anti-TNF (ADA, certolizumab, ETN, GOL, infliximab) or any non-TNF biologics (ABA, RTX, TCZ)</td><td>History of coronary heart disease</td></tr>
<tr><td>Generali 2018 (91)</td><td>Administrative healthcare database Italy</td><td>RA (ICD9 codes), patients starting treatment with TCZ or ETN for the first time</td><td>none</td></tr>
<tr><td>Lukas 2020 (92)</td><td>REGATE (French)</td><td>RA patients treated with TCZ</td><td>none</td></tr>
</table>

Table S4.1.2.2: Risk of bias analysis (Newcastle-Ottawa Scale [NOS] for Cohort studies)

Study	Representative-ness of exposed cohort	Selection of non-exposed cohort	Ascertainment of exposure	Outcome not presented at start	Comparability of cohort	Assessment of outcome	Follow-up length	Adequacy of follow-up	Summary
Zhang 2016 (90)	Low	Low	High	Low	Low	High	Low	Low	High
Generali 2018 (91)	Low	Low	High	Low	Low	High	Low	Low	High
Lukas 2020 (92)	Low	High	Low	Low	Low	Low	Low	Low	High

Table S4.1.2.3: Safety outcomes of observational studies regarding myocardial infarction.

Study	Treatment group	N patients	N events	Incidence rate (95% CI)	age/gender aHR (I vs C)	aHR (I vs C)	Adjusted for
Kim 2017 (86)	Combined TCZ	9,218	NR	NR	NR	0.70 (0.37;1.34)	demographics (age, sex, region, race/ethnicity [only available in the Medicare data]), prior DMARD use, cardiovascular comorbidities, other chronic diseases, cardiovascular medications, other long-term medications, and markers of health care utilization intensity
	Combined TNF-i	18,810	41	0.27/100 PY (0.20; 0.36)	NR	REF	
Kim 2018 (87)	Combined TCZ	6,237	NR	NR	NR	1.11 (0.65;1.89)	
	Combined ABA	14,685	NR	NR	NR	REF	

Zhang 2016 (90)	ABA	13,608	138	7.36/1000 PY (6.23; 8.70)	NR	REF	age, sex, race, original reason for Medicare enrolment (old age or disability), receipt of subsidised Medicare premium (a surrogate for low income), CV risk factors, other comorbid diseases (heart failure, COPD) and use of CV medications (antihypertense medications categorised into ACE inhibitors, β blockers, and other; statins; NSAIDs); acute myocardial infarction comparing biologics with different mechanisms to Abatacept
	ADA	10,241	77	6.82/1000 PY (5.46; 8.53)	NR	NAP	
	CZP	2,956	19	8.02/1000 PY (5.11; 12.57)	NR	NAP	
	ETN	9763	92	7.91/1000 PY (6.45; 9.71)	NR	NAP	
	GOL	1,774	<11	5.71/1000 PY (2.97; 10.97)	NR	NAP	
	INF	12,758	171	8.78/1000 PY (7.56; 10.20)	NR	NAP	
	RTX	7,475	71	8.43/1000 PY (6.68; 10.64)	NR	1.07 (0.80;1.42)	
	TCZ	3,332	17	6.23/1000 PY (3.87; 10.02)	NR	0.88 (0.51;1.51)	
	TNF-i	NR	NR	NR	NR	1.28 (1.04;1.56)	
Generali 2018 (91)	ETN	1,086	NR	NR	NR	REF	sex, age, disease duration, methotrexate (MTX), corticosteroids, non-steroidal anti-inflammatory drugs (NSAIDs), number of previous biologics, presence of hypertension, dyslipidaemia, diabetes and previous CV events
	TCZ	666	NR	NR	NR	0.39 (0.15;1.06)	
Lukas 2020 (92)	TCZ	5,591	12	0.21/100 PY	NAP	NAP	

NAP: not applicable

Table S4.1.2.4: Baseline characteristics of RCTs investigating IL-6R/L blockers regarding myocardial infarction.

Study	Treatment	Target	Population
Giles 2020 (ENTRACTE) (89)	Tocilizumab vs. Etanercept	IL-6R vs. TNF	csDMARD-IR; TNFi-IR

Table S4.1.2.5: Safety outcomes of RCTs regarding MACE.

Study	Treatment group	N patients	N events	Incidence rate (95% CI)	age/gender aHR (I vs C)	HR	Adjusted for
Giles 2020 (ENTRACTE) (89)	TCZ	1,538	29	0.61/100 PY (0.41;0.87)	NR	0.90 (0.54;1.48)	stratified by previous exposure to TNF-i therapy and history of cardiovascular (CV) events
	ETN	1,542	32	0.67/100 PY (0.46;0.95)	NR	REF	

4.1.3: Stroke/Transient ischemic attack: Comparison between IL-6R/L blockers and different bDMARDs (observational studies/randomized controlled trials)

Table S4.1.3.1: Baseline characteristics of observational studies investigating IL-6R/L blockers regarding stroke/transient ischemic attack.

Study	Registry	Inclusion criteria	Exclusion criteria
Kim 2017 (86)	US health care claims databases: Medicare, IMS PharMetrics Plus, Truven MarketScan	RA (ICD9 codes) patients, ≥ 18 years, starting TCZ or TNFi after failure of at least 1 bDMARD or tsDMARD	Nursing home residents, patients with HIV/ AIDS, malignancy other than NMSC, end-stage renal disease, patients undergone dialysis or renal transplant prior index date; patients who received RTX, patients with hospitalizations for MI, stroke, ACS, or heart failure in the 90 days prior index date
Kim 2018 (87)			
Generali 2018 (91)	Administrative healthcare database Italy	RA (ICD9 codes), patients starting treatment with TCZ or ETN for the first time	none
Lukas 2020 (92)	REGATE (French)	RA patients treated with TCZ	none

Table S4.1.3.2: Safety outcomes of observational studies regarding stroke/transient ischemic attack.

Study	Treatment group	N patients	N events	Incidence rate (95% CI)	age/gender aHR (I vs C)	aHR (I vs C)	Adjusted for
Kim 2017 (86)	Combined TCZ	9,218	23	0.33/100 PY (0.21; 0.49)	NR	0.94 (0.56; 1.59)	demographics (age, sex, region, race/ethnicity [only available in the Medicare data]), prior DMARD use, cardiovascular comorbidities, other chronic diseases, cardiovascular medications, other long-term medications, and markers of health care utilization intensity
	Combined TNF-i	18,810	49	0.32/100 PY (0.24; 0.42)	NR	REF	
Kim 2018 (87)	Combined TCZ	6,237	NR	NR	NR	0.73 (0.39; 1.39)	
	Combined ABA	14,685	NR	NR	NR	REF	
Generali 2018 (91)	ETN	1,086	NR	NR	NR	REF	sex, age, disease duration, methotrexate (MTX), corticosteroids, non-steroidal anti-inflammatory drugs (NSAIDs), number of previous biologics, presence of hypertension, dyslipidaemia, diabetes and previous CV events
	TCZ	666	NR	NR	NR	1.44 (0.24;8.68)	
Lukas 2020 (92)	TCZ	5,591	23	0.41/100 PY	NAP	NAP	

Table S4.1.3.3: Baseline characteristics of RCTs investigating IL-6R/L blockers regarding stroke/transient ischemic attack.

Study	Treatment	Target	Population
Giles 2020 (ENTRACTE) (89)	Tocilizumab vs. Etanercept	IL-6R vs. TNF	csDMARD-IR; TNFi-IR

Table S4.1.3.4: Safety outcomes of RCTs regarding stroke/transient ischemic attack.

Study	Treatment group	N patients	N events	Incidence rate (95% CI)	age/gender aHR (I vs C)	HR	Adjusted for
Giles 2020 (ENTRACTE) (89)	TCZ	1,538	26	0.53/100 PY (0.35;0.78)	NR	1.55 (0.83;2.90)	stratified by previous exposure to TNF-i therapy and history of cardiovascular (CV) events
	ETN	1,542	16	0.35/100 PY (0.20;0.56)	NR	REF	

4.1.4: Heart failure: Comparison between IL-6R/L blockers and different bDMARDs (observational studies/randomized controlled trials)

Table S4.1.4.1: Baseline characteristics of observational studies investigating IL-6R/L blockers regarding heart failure.

Study	Registry	Inclusion criteria	Exclusion criteria
Kim 2018 (87)	US health care claims databases: Medicare, IMS PharMetrics Plus, Truven MarketScan	RA (ICD9 codes) patients, ≥ 18 years, starting TCZ or TNFi after failure of at least 1 bDMARD or tsDMARD	Nursing home residents, patients with HIV/ AIDS, malignancy other than NMSC, end-stage renal disease, patients undergone dialysis or renal transplant prior index date; patients who received RTX, patients with hospitalizations for MI, stroke, ACS, or heart failure in the 90 days prior index date

Table S4.1.4.2: Safety outcomes of observational studies regarding heart failure.

Study	Treatment group	N patients	N events	Incidence rate (95% CI)	age/gender aHR (I vs C)	aHR (I vs C)	Adjusted for
Kim 2018 (87)	Combined TCZ	6,237	32	NR	NR	1.18 (0.71; 1.97)	demographics (age, sex, region, race/ethnicity [only available in the Medicare data]), prior DMARD use, cardiovascular comorbidities, other chronic diseases, cardiovascular medications, other long-term
	Combined ABA	14,685	112	NR	NR	REF	

							medications, and markers of health care utilization intensity

Table S4.1.4.3: Baseline characteristics of RCTs investigating IL-6R/L blockers regarding stroke/transient ischemic attack.

Study	Treatment	Target	Population
Giles 2020 (ENTRACTE) (89)	Tocilizumab vs. Etanercept	IL-6R vs. TNF	csDMARD-IR; TNFi-IR

Table S4.1.4.4: Safety outcomes of RCTs regarding stroke/transient ischemic attack.

Study	Treatment group	N patients	N events	Incidence rate (95% CI)	age/gender aHR (I vs C)	HR	Adjusted for
Giles 2020 (ENTRACTE) (89)	TCZ	1,538	12	0.31/100 PY (0.17;0.50)	NR	1.50 (0.61;3.67)	stratified by previous exposure to TNF-i therapy and history of cardiovascular (CV) events
	ETN	1,542	8	0.20/100 PY (0.10;0.38)	NR	REF	

4.1.5: Coronary Revascularisation: Comparison between IL-6R/L blockers and different bDMARDs (observational studies/randomized controlled trials)

Table S4.1.5.1: Baseline characteristics of observational studies investigating IL-6R/L blockers regarding coronary revascularization.

Study	Registry	Inclusion criteria	Exclusion criteria
Kim 2018 (87)	US health care claims databases: Medicare, IMS PharMetrics Plus, Truven MarketScan	RA (ICD9 codes) patients, ≥ 18 years, starting TCZ or TNFi after failure of at least 1 bDMARD or tsDMARD	Nursing home residents, patients with HIV/ AIDS, malignancy other than NMSC, end-stage renal disease, patients undergone dialysis or renal transplant prior index date; patients who received RTX, patients with hospitalizations for MI, stroke, ACS, or heart failure in the 90 days prior index date

Table S4.1.5.2: Safety outcomes of observational studies regarding coronary revascularization.

Study	Treatment group	N patients	N events	Incidence rate (95% CI)	age/gender aHR (I vs C)	aHR (I vs C)	Adjusted for
Kim 2018 (87)	Combined TCZ	6,237	NR	NR	NR	0.97 (0.56; 1.68)	demographics (age, sex, region, race/ethnicity [only available in the Medicare data]), prior DMARD use, cardiovascular comorbidities, other chronic diseases, cardiovascular medications, other long-term
	Combined ABA	14,685	NR	NR	NR	REF	

							medications, and markers of health care utilization intensity

4.1.6: Venous thromboembolism (VTE): Comparison between IL-6R/L blockers and different bDMARDs (randomized controlled trials)

Table S4.1.6.1: Baseline characteristics of RCTs investigating IL-6R/L blockers regarding VTE.

**Study**	**Treatment**	**Target**	**Population**
Giles 2020 (ENTRACTE) (89)	Tocilizumab vs. Etanercept	IL-6R vs. TNF	csDMARD-IR; TNFi-IR

Table S4.1.6.2: Safety outcomes of RCTs regarding VTE.

**Study**	**Treatment group**	**N patients**	**N events**	**Incidence rate (95% CI)**	**age/gender aHR (I vs C)**	**HR**	**Adjusted for**
Giles 2020 (ENTRACTE) (89)	TCZ	1,538	**DVT**: 10	0.2/100 PY	NR	0.83 (0.34;2.03)	stratified by previous exposure to TNF-i therapy and history of cardiovascular (CV) events
			**PE**: 1	0.06/100 PY	NR	0.13 (0.02;1.04)	
	ETN	1,542	**DVT**: 12	0.3/100 PY	NR	REF	
			**PE**: 8	0.2/100 PY	NR	REF	
DVT: deep vein thrombosis; PE: pulmonary embolism							

4.2. Vaccination

4.2.1: Vaccination: Comparison between IL-6R/L blockers and different b/csDMARDs (clinical trials)

Table S4.2.1.1: Included clinical trials investigating IL-6R/L blockers regarding antibody response after vaccination.

Study	Treatment	Population	Antibody response to	Intervention	Outcome
Crnkic Kapetanovic 2013 (93)	Rituximab Mono vs. Rituximab + MTX vs. Abatacept vs. Tocilizumab vs. MTX vs. Controls	RA patients receiving MTX or bDAMRDs other than TNF-i	pneumococcal conjugate vaccine	one dose (0.5 ml) of heptavalent pneumococcal conjugate vaccine (Prevenar) intramuscularly	IgG Streptococcus pneumoniae capsular polysaccharides 6B and 23F (before and week 4 and 6)
Mori 2013 (94)	Tocilizumab vs. Tocilizumab+MTX vs. MTX vs. Controls	RA patients receiving TCZ (at least the first dose of 8 mg/kg IV Q4W) and/or MTX for ≥12 weeks	pneumococcal polysaccharide vaccine	23-valent pneumococcal polysaccharide vaccine (PPV23)	IgG Streptococcus pneumoniae capsular polysaccharides 6B and 23F (before and week 4 and 6) and functional antibody activity reported as opsonisation indices (OIs)
Tsuru 2014 (95)	Tocilizumab vs. TNF-I vs. csDMARDs	Patients (n=28 RA and n=10 Castleman's disease) were treated with TCZ, 15 RA patients treated with TNF-i +	influenza and pneumococcal polysaccharide vaccine	single dose containing A (New Caledonia (NC):H1N1), A (Hiroshima (HIR):H3N2) and B (Malaysia (MAL) strain;	Antibody titers were measured every 4 weeks for a total of 12 weeks after vaccination

		MTX and 24 patients treated with csDMARDs		23-valent pneumococcal polysaccharide vaccine (PPV23) (TCZ group, n=21)	
Bingham III 2015 (VISARA) (96)	Tocilizumab + MTX vs. MTX	RA patients, TNFi-IR	pneumococcal polysaccharide vaccine (PPV23) and tetanus toxoid vaccine (TTV)	Week 3:<br>PPV23 administered i.m or s.c<br>TTV administered i.m in opposite deltoid	% of patients responding to ≥6/12 (PPV23) serotypes (primary) and % responding to TTV (secondary) at week 8
Shinoki 2012 (97)	Tocilizumab vs. healthy controls	sJIA patients treated with TCZ±GC±NSAID (no csDMARD/MTX)	influenza vaccine	standard doses of A/Solomon/3/2006(H1N1), A/Hiroshima/52/ 2005(H3N2), and B/Malaysia/2506/2004	seroprotection after vaccination; Blood samples were drawn before and 4–7 weeks (mean 5.2 weeks) after the last vaccination

Table S4.2.1.2: Risk of bias analysis

Study	Random sequence generation	Allocation concealment	Blinding of participants and personnel	Blinding of outcome assessment	Incomplete outcome data	Selective reporting	Other sources of bias	Summary	Comment
Crnkic Kapetanovic 2013 (93)	Unclear	Unclear	High	High	Low	Low	Low	High	Not randomized
Mori 2013 (94)	Unclear	Unclear	High	High	Low	Low	Low	High	Open label

Tsuru 2014 (95)	Unclear	Unclear	High	High	Low	Low	Low	High	Open label
Bingham III 2015 (VISARA) (96)	Unclear	Unclear	High	Unclear	Low	Low	Low	High	Open label
Shinoki 2012 (97)	Unclear	Unclear	High	High	Low	Low	Low	High	Not randomized

Table S4.2.1.3: Outcomes of clinical trials investigating IL-6R/L blockers regarding antibody response after vaccination.

Study	Primary / Secondary outcome	Treatment arm	No. of patients (n)	Result	p/95% CI
Crnkic Kapetanovic 2013 (93)	antibody response (AR) defined as post- and pre-vaccination ratio of antibody levels and positive antibody response (posAR) was AR ≥2	RTX monotherapy	29	10.3%; 0.3; 0.4; 0.2; 0.3	
		RTX + MTX	26	0%; 0.4; 0.4; 0.3; 0.4	
		ABA	17	17.6%; 0.6; 1.1; 0.4; 1.1	
		TCZ	16	50%; 0.4; 1.7; 0.2; 2.2	
	prevaccination antibody levels for 6B, mg/L, geometric mean antibody levels (GML;95% CI); postvaccination antibody levels for 6B, mg/L, GML (95% CI); prevaccination antibody levels for 23F, mg/L, GML (95% CI);	MTX	85	NR numerically; 2.0; 3.5; 0.7; 1.9	
		Controls (SpA patients on NSAIDs)	86	NR numerically; 2.9; 9.5; 0.97; 6.4	

	postvaccination antibody levels for 23F, mg/L, GML (95% CI)				
Mori 2013 (94)	Positive antibody response was defined as a 2-fold or more increase in the IgG concentration or as a ≥10-fold or more increase in the OI<br><br>% of patients with twofold or more increases in serotype-specific IgG concentrations for serotypes 6B and 23F; Percentages of patients with 10-fold or more increases in OIs for serotypes 6B and 23F in the RA treatment groups	MTX	62	21%; 16%*	p=0.046 (TCZ vs MTX); p=0.0009 (TCZ vs TCZ+MTX); p=0.005 (TCZ vs TCZ+MTX); p=0.044 (TCZ vs Cont)<br><br>% of patients with 10-fold or more increases in OIs for serotypes 6B and 23F in the RA treatment groups. p=0.019 (TCZ vs MTX); p=0.027 (TCZ vs MTX); p=0.020 (TCZ vs TCZ+MTX). *p=0.028 (TCZ vs MTX)
		TCZ+MTX	54	20%	
		TCZ	50	46%; 34%*	
		RA control group	24	21%;	
Tsuru 2014 (95)	seropositive response was defined as the HI titer at the post-vaccination ≥4-fold increase from the baseline titer against influenza antigen; seroprotective defined as post-vaccination HI titer ≥1:40. For pneumococcal vaccine, seropositive response was defined as ≥2-fold increase in antibody concentration from the baseline antibody levels in 6/12 serotypes of pneumococcal vaccine:<br><br>seropositive response A(NC), A(HIR), B(MAL); seroprotective level after	TCZ (RA+CD)	38	17/38; 18/38; 24/38; 36/38; 35/38; 32/38	
		TNF-i (RA)	15	6/15; 8/15; 4/15; 11/15; 12/15; 8/15	
		DMARDs (RA)	24	18/24; 13/24; 19/24; 22/24; 23/24; 21/24	
		Pneumococcal vaccine (TCZ)	21	21/21	

	vaccination A(NC), A(HIR), B(MAL); pneumococcal vaccine: seropositive response				
Bingham III 2015 (VISARA) (96)	% of responders to ≥6 of 12 anti-pneumococcal antibody serotype; % of responders to tetanus toxoid vaccine; %patients with ≥2-fold increases in anti-tetanus toxoid antibody levels; % patients with ≥4-fold increases in anti-tetanus toxoid antibody levels	MTX*	26	70.8%; 39.1%; 65.2%; 39.1%	95% CI: 52.6 to 89.0; 19.2 to 59.1; NR; NR
		TCZ IV 8mg/kg Q4W + MTX*	50	60.0%; 42.0%; 72.0%; 42.0%	95% CI: 46.4 to 73.6; 28.3 to 55.7; NR; NR
Shinoki 2012 (97)	seroprotection rate (%) after vaccination A/H1N1; seroprotection rate (%) after vaccination A/H3N2; seroprotection rate (%) after vaccination B	TCZ	27	88.9%; 85.2%, 40.7%	p=0.40; 0.15; 0.76
		age-matched healthy control	17	76.5%; 100.0%; 35.3%	
* all patients					

4.3. Infections

4.3.1: Serious infections: Comparison between IL-6R/L blockers and different bDMARDs (observational studies/randomized controlled trials)

Table S4.3.1.1: Baseline characteristics of observational studies investigating IL-6R/L blockers regarding serious infections.

Study	Registry	Inclusion criteria	Exclusion criteria
Carrara 2019 (98)	RECORD (health databases of Lombardy Region, Italy)	RA (ICD9 codes) patients treated with bDMDARDs	none
Mori 2017 (99)	SARABA (multiple medical centers in Japan)	RA patients (ACR 1987; ACR/EULAR 2010 criteria) starting first bDMARD	patients receiving bDMARDs previously
Rutherford 2018 a (100)	BSRBR-RA (British)	RA patients treated with bDMDARDs	none
Pawar 2019 (101)	US health care claims databases: Medicare, IMS PharMetrics Plus, Truven MarketScan	RA (ICD9/10 codes) patients treated with TCZ, ABA or TNF-i; ≥1 different biologic agent or tofacitinib any time prior	nursing home residents and patients with pre-existing malignancy prior and at the index date; RTX users
Grøn 2019 (102)	DANBIO (Danish); ARTIS (Swedish)	RA patients treated with ABA, TCZ or RTX	none
Grøn 2020 (103)	DANIBO	RA patients treated with ABA, TCZ or RTX	none
Morel 2017 (104)	REGATE (French)	RA patients treated with TCZ	none
Sakai 2015 (105)	REAL (Japanese)	RA patients (1987 ACR criteria), treatment with csDMARDs or bDMARDs	none

Yun 2016 (106)	US health care claims database: Medicare	RA (ICD9 codes) patients treated with bDMDARDs after having been treated with a different biologic agent at any time (i.e., biologic switchers)	patients with PsA, psoriasis, ankylosing spondylitis (AS), or inflammatory bowel disease

Table S4.3.1.2: Risk of bias analysis (Newcastle-Ottawa Scale [NOS] for Cohort studies)

Study	Representative-ness of exposed cohort	Selection of non-exposed cohort	Ascertainment of exposure	Outcome not presented at start	Comparability of cohort	Assessment of outcome	Follow-up length	Adequacy of follow-up	Summary
Carrara 2019 (98)	Low	Low	High	Low	Low	High	Low	Low	High
Mori 2017 (99)	Low	Low	Low	Low	Low	Low	Low	Low	Low
Rutherford 2018 a (100)	Low	Low	Low	Low	Low	Low	Low	Low	Low
Pawar 2019 (101)	Low	Low	High	Low	Low	High	Low	Low	High
Grøn 2019 (102)	Low	Low	Low	Low	Low	High	Low	Low	High
Grøn 2020 (103)	Low	Low	Low	Low	Low	High	Low	Low	High
Morel 2017 (104)	Low	High	Low	Low	High	Low	Low	Low	High
Sakai 2015 (105)	Low	Low	Low	Low	Low	Low	Low	Low	Low

Yun 2016 (106)	Low	Low	High	Low	Low	High	Low	Low	High

Table S4.3.1.3: Safety outcomes of observational studies regarding serious infections.

Study	Treatment group	N patients	N events	Incidence rate (95% CI)	age/gender aHR (I vs C)	aHR (I vs C)	Adjusted for
Carrara 2019 (98)	ETN	NR	68	8.2/1000 PY (6.4;10.4)	NR	REF	sex, age, disease duration, Charlson Comorbidity Index, concomitant use of MTX, leflunomide, GC, NSAIDs, number of previous bDMARDs and previous infections
	ADA	NR	52	10.7/1000 PY (8;14.1)	NR	1.37 (0.95;1.96)	
	IFX	NR	26	8.1/1000 PY (5.3;11.9)	NR	0.96 (0.60;1.56)	
	CZP	NR	4	9. /1000 PY (2.7;25.2)	NR	1.31 (0.48;3.58)	
	GOL	NR	4	8.8/1000 PY (2.4;22.7)	NR	1.09 (0.37;3.21	
	ABA	NR	4	2.8/1000 PY (0.8;7.3)	NR	0.29 (0.10;0.82)	
	RTX	NR	13	13.2/1000 PY (7.0;22.6)	NR	0.95 (0.48;1.91)	
	TCZ	NR	10	10.8 /1000 PY (5.2;19.8)	NR	1.24 (0.59;2.61)	

Mori 2017 (99)	ETN	413	25	8.0/100 PY (5.4;11.9)	NR	REF	age, sex, BMI, smoking history, RA duration, RA stage III/IV, RA class 3/4, previous use of biological agents, concurrent use of MTX, concurrent use of prednisolone, and comorbid diseases (chronic kidney disease, diabetes mellitus, chronic lung disease)
	IFX	335	15	5.7/100 PY (3.5;9.5)	NR	1.54 (0.78;3.04)	
	ADA	264	15	7.4/100 PY (4.5;12.3	NR	1.72 (0.88;3.34)	
	ABA	189	12	8.4/100 PY (4.8;14.8)	NR	1.11 (0.55;2.21)	
	TCZ	395	19	6.0/100 PY (3.8;9.4)	NR	1.02 (0.55;1.87)	
Rutherford 2018 a (100)	ETN	8,630	852	5.56/100 PY (5.20;5.95)	NR	REF	age, gender, DAS28-ESR, HAQ, disease duration, smoking, seropositivity, polypharmacy, baseline steroid usage
	IFX	4,908	472	5.35/100 PY (4.89;5.85)	NR	0.89 (0.79;1.00)	
	ADA	7,818	709	5.42/100 PY (5.04;5.84)	NR	1.00 (0.90;1.10)	
	RTX	5,101	372	6.29/100 PY (5.69;6.97)	NR	0.91 (0.80;1.03)	
	TCZ	2,174	137	6.98/100 PY (5.90;8.25)	NR	1.21 (1.01;1.46)	
	CZP	1,446	64	3.80/100 PY (2.97;4.85)	NR	0.75 (0.58;0.97)	
Pawar 2019 (101)	Combined TCZ	16,074	618	4.68/100 PY (4.31;5.05)	NR	1.05 (0.95;1.16)	index year, demographics, comorbid conditions, combined comorbidity index, claims-based index of RA severity index, use of DMARDs (during all available data) and other prescription drugs
	Combined TNF-i	33,109	1,155	3.99/100 PY (3.76;4.22)	NR	REF	

	Combined ABA	10,414	295	3.24/100 PY (2.87;3.61)	NR	1.40 (1.20; 1.63)	including GC, NSAIDs and analgesics, use of prophylactic antibiotics/antivirals, vaccination, history of any invasive procedures or surgery; cancer screening tests; physician orders of outpatient laboratory tests for acute phase reactants
Grøn 2019 (102)	Combined ABA	2,725	NR	NR	0.93 (0.74;1.15)	0.88 (0.69; 1.12)	age, gender, DAS28, disease duration, HAQ, smoking, previous malignancy, previous serious infection, previous number of prescriptions, previous COPD, tertiles of prescriptions
	Combined RTX	3,363	NR	NR	REF	REF	
	Combined TCZ	2,899	NR	NR	0.75 (0.60;0.95)	0.78 (0.61; 1.01)	
Grøn 2020 (103)*	ABA	1,115	598	76/100 PY (70;80)	NR	0.94 (0.81; 1.08)	calendar year, RA disease duration (years), number of previous bDMARDs, GCs, DAS28, HAQ, CRP, use of concomitant MTX, IgM RF status, current smoking status, history of cancer, hospitalized, knee or hip prosthesis, COPD, diabetes, myocardial infarction, or chronic kidney disease, reimbursement of a prescription of antibiotics and antivirals
	RTX	1,017	579	66/100 PY (61;72)	NR	REF	
	TCZ	1,564	883	69/100 PY (65;75)	NR	0.94 (0.81; 1.03)	
Morel 2017 (104)	TCZ	1,491	125	4.7/100 PY (3.8; 5.5)	NR	no control group/reference	-
Sakai 2015 (105)	TNF-i	304	7	3.03/100 PY (1.35;5.95)	NR	REF	age, gender, DAS28-CRP, comorbidity, corticosteroids ≥5 mg/day
	TCZ	302	24	10.68/100 PY (7.02;15.63)	NR	2.23 (0.93;5.37)	

Yun 2016 (106)	ADA	4,845	317	14.6/100 PY (13.1;16.3)	NR	1.08 (0.93;1.25)	infection risk score decile, number of previous bDMARDs, disability status, GC use during baseline, MTX use during baseline, most recent bDMARD used during baseline, and Medicaid eligibility.
	CZP	1,866	106	14.2/100 PY (11.7;17.2)	NR	1.07 (0.86;1.32)	
	ETN	3,814	87	14.1/100 PY (11.5;17.4)	NR	1.24 (1.07;1.45)	
	GOL	1,394	275	15.9/100 PY (14.2;17.9)	NR	1.14 (0.90;1.44)	
	IFX	3,944	370	17.0/100 PY (15.3;18.8)	NR	1.39 (1.21;1.60)	
	RTX	4,718	541	18.7/100 PY (17.2;20.3)	NR	1.36 (1.21;1.53)	
	TCZ	2,016	129	14.9/100 PY (12.6;17.8)	NR	1.10 (0.89;1.34)	
	ABA	9,204	705	13.1/100 PY (12.2;14.1)	NR	REF	
* overall infections; defined as a prescription of antibiotics or hospitalization due to infection; data reported as 0-24 months after starting treatment; incidence rate adjusted for age and gender							

Table S4.3.1.4.1: Safety outcomes of observational studies regarding subtypes of serious infections.

Study	Treatment group	Incidence rate (95% CI) Sepsis	aHR (95% CI) Sepsis	Incidence rate (95% CI) Respiratory infection	aHR (95% CI) Respiratory infection	Incidence rate (95% CI) Skin infection	aHR (95% CI) Skin infection	Incidence rate (95% CI) GI-infection	aHR (95% CI) GI-infection
Rutherford 2018 a (100)	ETN	0.15/100 PY (0.10;0.23)	REF	1.82/100 PY (1.61;2.04)	REF	1.31/100 PY (1.14;1.51)	REF	0.5/100 PY (0.40;0.63)	REF
	IFX	0.14/100 PY (0.08;0.24)	0.83 (0.41;1.66)	2.25/100 PY (1.96;2.59)	1.16 (0.96;1.39)	1.21/100 PY (1.00;1.46)	0.84 (0.66;1.06)	0.51/100 PY (0.38;0.68)	0.95 (0.66;1.38)
	ADA	0.16/100 PY (0.10;0.25)	1.04 (0.57;1.91)	2.28/100 PY (2.04;2.55)	1.23 (1.04;1.45)	0.89/100 PY (0.74;1.06)	0.65 (0.52;0.82)	0.38/100 PY (0.29;0.50)	0.77 (0.54;1.11)
	RTX	0.44/100 PY (0.30;0.64)	2.08 (1.14;3.80)	2.71/100 PY (2.32;3.16)	1.03 (0.83;1.28)	0.9/100 PY (0.69;1.17)	0.54 (0.39;0.75)	0.58/100 PY (0.41;0.81	0.93 (0.61;1.42)
	TCZ	0.31/100 PY (0.14;0.68)	1.83 (0.63;5.35)	3.16/100 PY (2.46;4.05)	1.61 (1.15;2.25)	1.38/100 PY (0.94;2.01)	0.71 (0.40;1.24)	0.76/100 PY (0.46;1.27)	1.45 (0.72;2.90)
	CZP	0.12/100 PY (0.03;0.47)	1.03 (0.24;4.41)	1.72/100 PY (1.20;2.48)	0.96 (0.63;1.46)	0.42/100 PY (0.20;0.87)	0.27 (0.11;0.67)	0.18/100 PY (0.06;0.55)	0.51 (0.16;1.63)
Pawar 2019 (101)	Combined TCZ	1.72/100 PY (1.50;1.95)	1.04 (0.88;1.22)	1.39/100 PY (1.19;1.59)	0.92 (0.77;1.10)	0.28/100 PY (0.19;0.36)	2.38 (1.47;3.86)	0.52/100 PY (0.40;0.64)*	2.34 (1.64; 3.34)*
	Combined TNF-i	1.51/100 PY (1.37;1.65)	REF	1.34/100 PY (1.21;1.48)	REF	0.12/100 PY (0.08;0.15)	REF	0.21 /100 PY (0.16;0.26)*	REF*
	Combined ABA	NR	NR	NR	NR	NR	NR	NR*	NR*

Sakai 2015 (105)	TCZ	1.34/100 PY (0.37;3.56)	NA	3.12/100 PY (1.39;6.12	NR	1.78/100 PY (0.60; 4.23)	NR	0.89/100 PY (0.18; 2.85)	NR
	TNF-i	0	NA	1.30/100 PY (0.36;3.46)	NR	0.43/100 PY (0.04;2.02)	NR	0.43/100 PY (0.04; 2.02)	NR
* diverticulitis									

Table S4.3.1.4.2: Safety outcomes of observational studies regarding subtypes of serious infections.

Study	Treatment group	Incidence rate (95% CI) Bone/joint infection	aHR (95% CI) Bone/joint infection	Incidence rate (95% CI) Genitourinary infection	aHR (95% CI) Genitourinary infection	Incidence rate (95% CI) Other infection	aHR (95% CI) Other infection
Rutherford 2018 a (100)	ETN	0.67/100 PY (0.55;0.81)	REF	0.61/100 PY (0.50;0.75)	REF	0.36/100 PY (0.28;0.47)	REF
	IFX	0.43/100 PY (0.31;0.59)	0.56 (0.38; 0.83)	0.48/100 PY (0.35;0.64)	0.74 (0.50;1.07)	0.25/100 PY (0.16;0.38)	0.54 (0.31;0.91)
	ADA	0.51/100 PY (0.40;0.65)	0.80 (0.58; 1.09)	0.68/100 PY (0.55;0.84)	1.18 (0.87;1.59)	0.41/100 PY (0.31;0.53)	1.08 (0.74 1.58)
	RTX	0.49/100 PY (0.34;0.71)	0.67 (0.43; 1.02)	0.81/100 PY (0.61;1.08)	1.15 (0.79;1.68)	0.32/100 PY (0.21;0.50)	0.72 (0.41;1.29)
	TCZ	0.46/100 PY (0.24;0.88	0.46 (0.17; 1.27)	0.46/100 PY (0.24;0.88)	0.67 (0.27;1.66)	0.41/100 PY (0.20;0.81)	1.15 (0.49;2.67)

	CZP	0.53/100 PY (0.28;1.03)	0.73 (0.32; 1.68)	0.36/100 PY (0.16;0.79)	0.55 (0.20;1.52)	0.3/100 PY (0.12;0.71)	0.50 (0.16;1.60)
Pawar 2019 (101)	Combined TCZ	NR	NR	0.49/100 PY (0.37;0.60)	0.77 (0.58;1.04)	NR	NR
	Combined TNF-i	NR	NR	0.54/100 PY (0.46;0.63)	REF	NR	NR
	Combined ABA	NR	NR	NR	NR	NR	NR
Sakai 2015 (105)	TCZ	2.23/100 PY (0.84;4.88)	NA	0.89/100 PY (0.18;2.85)	NR	0.45/100 PY (0.04;2.08)	NR
	TNF-i	0	NA	0.43/100 PY (0.04;2.02)	NR	0.43/100 PY (0.04; 2.02)	NR

4.3.2: Opportunistic infections: Comparison between IL-6R/L blockers and different bDMARDs (observational studies/randomized controlled trials)

Table S4.3.2.1: Baseline characteristics of observational studies investigating IL-6R/L blockers regarding opportunistic infections.

Study	Registry	Inclusion criteria	Exclusion criteria
Pawar 2019 (101)	US health care claims databases: Medicare, IMS PharMetrics Plus, Truven MarketScan	RA (ICD9/10 codes) patients treated with TCZ, ABA or TNF-i; ≥1 different biologic agent or tofacitinib any time prior	nursing home residents and patients with pre-existing malignancy prior and at the index date; RTX users

Rutherford 2018 b (107)	BSRBR-RA	RA patients treated with bDMDARDs	a priori decision by authors to exclude tuberculosis (TB) from main analysis
Leon 2019 (108)	Hospital Clínico San Carlos, Madrid, Spain	RA (ICD 10 codes) patients treated with bDMARDs	none

Table S4.3.2.2: Risk of bias analysis (Newcastle-Ottawa Scale [NOS] for Cohort studies)

Study	Representative-ness of exposed cohort	Selection of non-exposed cohort	Ascertainment of exposure	Outcome not presented at start	Comparability of cohort	Assessment of outcome	Follow-up length	Adequacy of follow-up	Summary
Rutherford 2018 b (107)	Low	Low	Low	Low	Low	Low	Low	Low	Low
Leon 2019 (108)	Low	Low	Low	Low	Low	Low	Low	Low	Low

Table S4.3.2.3: Safety outcomes of observational studies regarding opportunistic infections.

Study	Treatment group	N patients	N events	Incidence rate (95% CI)	age/gender aHR (I vs C)	aHR (I vs C)	Adjusted for
Pawar 2019 (101)	Combined TCZ	16,074	18	0.13/100 PY (0.07;0.20)	NR	0.99 (0.55; 1.79)	index year, demographics, comorbid conditions, combined comorbidity index, claims-based index of RA severity index, use of DMARDs (during all available

	Combined TNF-i	33,109	37	NR	NR	REF	data) and other prescription drugs including GC, NSAIDs and analgesics, use of prophylactic antibiotics/antivirals, vaccination, history of any invasive procedures or surgery; cancer screening tests; physician orders of outpatient laboratory tests for acute phase reactants
	Combined ABA	10,414	NR	0.13/100 PY (0.09;0.17	NR	NR	
Rutherford 2018 b (107)	TNF-i	16,742	114	134 /100 000 PY (111; 161)	NR	REF	age, gender, disease severity and duration, smoking, seropositivity, polypharmacy (as a surrogate for comorbidity)
	RTX	5,072	25	146/100 000 PY (98; 217)	NR	0.96 (0.62; 1.50)	
	TCZ	2,171	3	78/100 000 PY (25; 241)	NR	0.52 (0.17; 1.65)	
Leon 2019 (108)	ADA	28.4*	11	26.3/1000 PY (8.4; 81.6)	NR	NR	
	ETN	23.5`*	9	20.7/1000 PY (10.7; 39.8)	NR	NR	
	IFX	7*	3	26.3/1000 PY (8.4;81.6)	NR	NR	
	RTX	17.3*	9	40.3/1000 PY (20.9; 77.4)	NR	NR	
	ABA	6.2*	2	22.5/1000 PY (5.6; 90)	NR	NR	
	CZP	10.6*	2	23.2/1000 PY (5.8; 92.8)	NR	NR	

	TCZ	5.3*	1	15.1/1000 PY (2.1; 107.6)	NR	NR	
	GOL	1.7*	0	-	NR	NR	
* data reported as percentage of total n=441							

4.3.3: Tuberculosis: Comparison between IL-6R/L blockers and different bDMARDs (observational studies/randomized controlled trials)

Table S4.3.3.1: Baseline characteristics of observational studies investigating IL-6R/L blockers regarding tuberculosis.

Study	Registry	Inclusion criteria	Exclusion criteria
Pawar 2019 (101)	US health care claims databases: Medicare, IMS PharMetrics Plus, Truven MarketScan	RA (ICD9/10 codes) patients treated with TCZ, ABA or TNF-i; ≥1 different biologic agent or tofacitinib any time prior	nursing home residents and patients with pre-existing malignancy prior and at the index date; RTX users
Rutherford 2018 b (107)	BSRBR-RA	RA patients treated with bDMDARDs	a priori decision by authors to exclude tuberculosis (TB) from main analysis
Lim 2017 (109)	Taichung Veterans General Hospital, Taiwan	RA (ICD9 codes), ≧18 years old, first bDMARDs or tDMARDs; in Taiwan, latent TB screening and treatment policy before initiation of biologics commenced in 2012. As per TB risk management plan, every patient must undergo TB screening before initiation of biologics	concomitant diagnosis of psoriatic arthritis, spondyloarthritis, inflammatory bowel diseases or Behcet's disease. Patients who had used bDMARDs or tDMARDs prior

Wang 2019 (110)	multiple medical centers, computerized database in Hong Kong	Rheumatology disease (RA, ankylosing spondylitis, psoriatic arthritis, Felty's syndrome and other miscellaneous); IBD (ulcerative colitis, Crohn's disease and undetermined colitis); and dermatology disease (psoriasis); *it is possible that some TB cases who had taken anti-TB treatment in private institutions were missed, and this could result in an underestimation of the IR of TB (all patients should undergo latent TB screening prior to biologic initiation based on Hong Kong's local guideline)*	two or more of the mentioned disease subtypes

Table S4.3.3.2: Risk of bias analysis (Newcastle-Ottawa Scale [NOS] for Cohort studies)

**Study**	**Representative-ness of exposed cohort**	**Selection of non-exposed cohort**	**Ascertainment of exposure**	**Outcome not presented at start**	**Comparability of cohort**	**Assessment of outcome**	**Follow-up length**	**Adequacy of follow-up**	**Summary**
Lim 2017 (109)	Low	Low	High	Low	Low	High	Low	Low	High
Wang 2019 (110)	Low	Low	Low	Low	Low	Low	Low	Low	Low

Table S4.3.3.3: Safety outcomes of observational studies regarding tuberculosis.

Study	Treatment group	N patients	N events	Incidence rate (95% CI)	age/gender aHR (I vs C)	aHR (I vs C)	Adjusted for
Pawar 2019 (101)	Combined TCZ	16,074	0	0.00/100 PY (0.00;0.00)	NR	NAP	index year, demographics, comorbid conditions, combined comorbidity index, claims-based index of RA severity index, use of DMARDs (during all available data) and other prescription drugs including GC, NSAIDs and analgesics, use of prophylactic antibiotics/antivirals, vaccination, history of any invasive procedures or surgery; cancer screening tests; physician orders of outpatient laboratory tests for acute phase reactants
	Combined TNF-i	33,109	0	0.00/100 PY (0.00;0.00)	NR	NR	
	Combined ABA	10,414	NR	NR	NR	REF	
Rutherford 2018 b (107)	TNF-i	16,742	56	65/100,000 PY (50;85)	NR	REF	age, gender, disease severity and duration, smoking, seropositivity, polypharmacy (as a surrogate for comorbidity)
	RTX	5,072	2	12/100,000 PY (3;46)	NR	0.16 (0.04; 0.67)	
	TCZ	2,171	1	26/100,000 PY (4;183)	NR	0.35 (0.05; 2.55)	
Lim 2017 (109)	ETN	443	13	889.3/100,000 PY	REF	NR	age, gender
	ADA	332	11	1055.6/100,000 PY	1.27 (0.76;2.13)*	NR	
	GOL	60	0	0	NAP	NR	

	TCZ	31	0	0	NAP	NR	
	ABA	74	0	0	NAP	NR	
	TOF	11	0	0	NAP	NR	
Wang 2019 (110)	Combined TNF-i	2840	57	956.1/100,000 PY	NR	4.34 (1.31;14.39)**	age, sex, comorbidity and concurrent use of immunosuppressant when comparing risk of TB among different disease subtypes and biologics
	ABA	147	0	0/100,000 PY	NR	NR	
	RTX	167	2	1404.5/100,000 PY	NR	NR	
	TCZ	371	4	633.8/100,000 PY	NR	NR	
	TOF	38	0	0/100,000 PY	NR	NR	
	UST	19	0	0/100,000 PY	NR	NR	

TOF: tofacitinib; UST: ustekinumab
* TB HR for ADA was 1.87 (95% CI:1.27;2.73) in patients without TB infection history
** aHR: risk of TB with TNF inhibitor vs. a non-TNF biologic as reference

4.3.4: Pneumocystis jirovecii pneumonia: Comparison between IL-6R/L blockers and different bDMARDs (observational studies/randomized controlled trials)

Table S4.3.4.1: Baseline characteristics of observational studies investigating IL-6R/L blockers regarding pneumocystis jirovecii pneumonia.

Study	Registry	Inclusion criteria	Exclusion criteria
Rutherford 2018 b (107)	BSRBR-RA	RA patients treated with bDMARDs	a priori decision by authors to exclude tuberculosis (TB) from main analysis

Table S4.3.4.2: Safety outcomes of observational studies regarding pneumocystis jirovecii pneumonia.

Study	Treatment group	N patients	N events	Incidence rate (95% CI)	age/gender aHR (I vs C)	aHR (I vs C)	Adjusted for
Rutherford 2018 b (107)	TNF-i	16,742	15	NR	NR	REF	age, gender, disease severity and duration, smoking, seropositivity, polypharmacy (as a surrogate for comorbidity)
	RTX	5,072	9	52/100,000 PY	NR	3.2 (1.4;7.5)	
	TCZ	2,171	NR	NR	NR	NR	

4.3.5: Herpes zoster: Comparison between IL-6R/L blockers and different bDMARDs (observational studies/randomized controlled trials)

Table S4.3.5.1: Baseline characteristics of observational studies investigating IL-6R/L blockers regarding herpes zoster.

Study	Registry	Inclusion criteria	Exclusion criteria
Pawar 2019 (101)	US health care claims databases: Medicare, IMS PharMetrics Plus, Truven MarketScan	RA (ICD9/10 codes) patients treated with TCZ, ABA or TNF-i; ≥1 different biologic agent or tofacitinib any time prior	nursing home residents and patients with pre-existing malignancy prior and at the index date; RTX users
Curtis 2016 (111)	US health care claims databases: Medicare, MarketScan	RA (ICD9 codes) patients, first use of TOFA or bDMARD	prior diagnosis of herpes infection (ICD 9 codes), any diagnostic of mucocutaneous ulcers (ICD9), or any prior use of acyclovir, valacyclovir, or famciclovir. Diagnosis for ankylosing spondylitis, psoriasis, psoriatic arthritis, or IBD; any cancer diagnosis, other nonmelanoma skin cancer
Yun 2015 (112)	US health care claims database: Medicare	RA (ICD9 codes) patients, history of prior biologic agent use	diagnosis of cancer or other autoimmune diseases (i.e., psoriatic arthritis, psoriasis, ankylosing spondylitis, or inflammatory bowel disease); patients who used antiviral medication (famciclovir, aciclovir, or valaciclovir) during the 3 months before the index date or who had a diagnosis code of HZ at any time before the index date (not just the 12-month baseline)

Table S4.3.5.2: Risk of bias analysis (Newcastle-Ottawa Scale [NOS] for Cohort studies)

Study	Representative-ness of exposed cohort	Selection of non-exposed cohort	Ascertainment of exposure	Outcome not presented at start	Comparability of cohort	Assessment of outcome	Follow-up length	Adequacy of follow-up	Summary
Curtis 2016 (111)	Low	Low	High	Low	Low	High	Low	Low	High
Yun 2015 (112)	Low	Low	High	Low	Low	High	Low	Low	High

Table S4.3.5.3: Safety outcomes of observational studies regarding herpes zoster.

Study	Treatment group	N patients	N events	Incidence rate (95% CI)	age/gender aHR (I vs C)	aHR (I vs C)	Adjusted for
Pawar 2019 (101)	Combined TCZ	16,074	15	0.11/100 PY (0.06;0.17)	NR	0.90 (0.48; 1.69)	index year, demographics, comorbid conditions, combined comorbidity index, claims-based index of RA severity index, use of DMARDs (during all available data) and other prescription drugs including GC, NSAIDs and analgesics, use of prophylactic antibiotics/antivirals, vaccination, history of any invasive procedures or surgery; cancer screening tests; physician orders of outpatient laboratory tests for acute phase reactants
	Combined TNF-i	33,109	33	0.11/100 PY (0.07;0.15)	NR	REF	
	Combined ABA	10,414	NR	NR	NR	NR	

Curtis 2016 (111)*	ADA	NR	NR	1.95/100 PY (1.65;2.31)	NR	1.00 (0.80; 1.25)	age, gender, GC, MTX, number of biologics, prior hospitalized infection, prior hospitalization for other reasons, prior outpatient infection (other than varicella), zoster vaccination
	CZP	NR	NR	2.55/100 PY (2.04;3.20)	NR	1.14 (0.87; 1.48)	
	ETN	NR	NR	2.08/100 PY (1.77;2.45)	NR	1.06 (0.85; 1.32)	
	GOL	NR	NR	2.12/100 PY (1.53;2.94)	NR	1.09 (0.76; 1.57)	
	IFX	NR	NR	2.71/100 PY (2.33;3.08)	NR	1.17 (0.97; 1.43)	
	RXT	4,785	NR	2.67/100 PY (2.22;3.22)	NR	1.12 (0.89; 1.41)	
	TCZ	6,266	NR	2.48/100 PY (2.07;2.98)	NR	1.12 (0.88; 1.42)	
	TOFA	1,746	NR	3.87/100 PY (2.82;5.32)	NR	2.01 (1.40; 2.88)	
	ABA	11,434	NR	2.33/100 PY (2.04;2.67)	NR	REF	
Yun 2015 (112)	ABA	NR	142	1.87/100 PY (1.58;2.20)	NR	REF	age, sex, race, oral glucocorticoids use during baseline, methotrexate use during baseline, number of hospitalizations during baseline, previous biologic agent type, disabled status, number of hospitalizations during baseline, and HZ vaccination before new biologic agent treatment initiation
	RTX	NR	82	2.27/100 PY (1.83;2.82)	NR	1.20 (0.88;1.63)	
	TCZ	NR	18	2.15/100 PY (1.35;3.40)	NR	1.05 (0.60;1.84)	
	ADA	NR	46	1.74/100 PY (1.31;2.33)	NR	1.04 (0.72;1.51)	

	CZP	NR	19	2.45/100 PY (1.57;3.85)	NR	1.30 (0.77;2.23)	
	ETN	NR	48	2.15/100 PY (1.62;2.86)	NR	1.26 (0.87;1.81)	
	GOL	NR	11	1.61/100 PY (0.89;2.91)	NR	0.91 (0.47;1.76)	
	IFX	NR	57	1.82/100 PY (1.40;2.36)	NR	0.98 (0.69;1.39)	
	MTX yes	NR	251	1.94/100 PY (1.67;2.26)	NR	REF[a]	
	MTX no	NR	172	1.98/100 PY (1.75;2.24)	NR	1.07 (0.88–1.29)[a]	
	No GC	NR	128	1.50/100 PY (1.26;1.78)	NR	REF[b]	
	≤7.5mg/d GC	NR	209	2.12/100 PY (1.85;2.43)	NR	1.55 (1.25;1.93)[b]	
	>7.5mg/d GC	NR	86	2.74/100 PY (2.22;3.39)	NR	2.35 (1.81;3.04)[b]	
* Glucocorticoids:<br>≤7.5 mg/day vs. 0 mg/day: HR: 1.05 (0.91; 1.20)<br>>7.5 mg/day vs. 0 mg/day: HR: 1.40 (1.19; 1.65)							

4.4. Malignancies

4.4.1: All types of cancer: Comparison between IL-6R/L blockers and different bDMARDs (observational studies/randomized controlled trials)

Table S4.4.1.1: Baseline characteristics of observational studies investigating IL-6R/L blockers regarding all types of cancer (excluding NMSC).

Study	Registry	Inclusion criteria	Exclusion criteria
Wadström 2017 (113)	Swedish Rheumatology Quality of Care Register (SRQ)/ARTIS, Swedish Patient Register Swedish Cancer Register Prescribed Drug Register Total Population Register	RA patients treated with TNF-i, non-TNF-i; bDMARD-naive patients with csDMARDs	juvenile idiopathic arthritis, systemic lupus erythematosus, psoriatic arthritis, spondyloarthropathy
Kim 2019 (114)	US health care claims databases: Medicare, IMS PharMetrics Plus, Truven MarketScan	RA (ICD9/10 codes) patients treated with TCZ or TNF-i; ≥1 different biologic agent or tofacitinib prior	nursing home residents, patients with preexisting malignancies at baseline, RTX users

Table S4.4.1.2: Risk of bias analysis (Newcastle-Ottawa Scale [NOS] for Cohort studies)

Study	Representative-ness of exposed cohort	Selection of non-exposed cohort	Ascertainment of exposure	Outcome not presented at start	Comparability of cohort	Assessment of outcome	Follow-up length	Adequacy of follow-up	Summary
Wadström 2017 (113)	Low	Low	Low	Low	Low	Low	Low	Low	Low
Kim 2019 (114)	Low	Low	High	Low	Low	High	Low	Low	High

Table S4.4.1.3: Safety outcomes of observational studies regarding all types of cancer (invasive solid or hematologic malignant neoplasm excluding NMSC).

Study	Treatment group	N patients	N events	Incidence rate (95% CI)	age/gender aHR (I vs C)	aHR (I vs C)		Adjusted for
Wadström 2017 (113)	TCZ	1,798	50	959/100,000 PY	0.87 (0.66;1.16)	0.89 (0.67; 1.18)	1.12 (0.81; 1.54)	age, sex, and start-year, comorbidities, educational level, number of hospitalizations and days spent in inpatient care, use of GCs, use of NSAIDs at baseline, number of prescription drugs at baseline, and sick leave and disability year before cohort entry; disease duration, DAS28-CRP, CRP, erythrocyte sedimentation rate, HAQ, previous bDMARD use
	ABA	2,021	61	1026/100,000 PY	0.88 (0.68;1.13)	0.88 (0.68; 1.14)	1.10 (0.82; 1.48)	
	RTX	3,586	141	1074/100,000 PY	0.86 (0.72;1.02)	0.86 (0.73; 1.03)	1.06 (0.86; 1.30)	

	TNF-i (first bDMARD)	10,782	478	978/100,000 PY	0.92 (0.84;1.01)	0.93 (0.85; 1.01)	REF	
	TNF-i (second bDMARD)	4,347	169	917/100,000 PY	0.88 (0.76;1.03)	0.89 (0.76; 1.04	NR	
	csDMARD	46,610	3,260	1,328/100,000 PY	REF	REF	NR	
	General population	107,491	4,193	953/100,000 PY	0.90 (0.82;0.99)	NAP	NR	
Kim 2019 (114)	Combined TCZ	13,102	162	14.77/1000 PY (12.49;17.04)	NR	0.96 (0.79, 1.17)		sex, age, baseline use of MTX, baseline use of GC, and receipt of any cancer screening tests
	Combined TNF-i	26,727	322	14.60/1000 PY (13.00;16.19	NR	REF		

4.4.2: Invasive solid cancer: Comparison between IL-6R/L blockers and different bDMARDs (observational studies/randomized controlled trials)

Table S4.4.2.1: Baseline characteristics of observational studies investigating IL-6R/L blockers regarding invasive solid cancer.

Study	Registry	Inclusion criteria	Exclusion criteria
Wadström 2017 (113)	Swedish Rheumatology Quality of Care Register (SRQ)/ARTIS, Swedish Patient Register Swedish Cancer Register Prescribed Drug Register Total Population Register	RA patients treated with TNF-i, non-TNF-i; bDMARD-naive patients with csDMARDs	juvenile idiopathic arthritis, systemic lupus erythematosus, psoriatic arthritis, spondyloarthropathy

Table S4.4.2.2: Safety outcomes of observational studies regarding invasive solid cancer.

Study	Treatment group	N patients	N events	Incidence rate (95% CI)	age/gender aHR (I vs C)	aHR (I vs C)		Adjusted for
Wadström 2017 (113)	TCZ	1,798	47	899/100,000 PY	0.92 (0.69;1.23)	0.95 (0.71; 1.27)	1.14 (0.81; 1.59)	age, sex, and start-year, comorbidities, educational level, number of hospitalizations and days spent in inpatient care, use of GCs, use of NSAIDs at baseline, number of prescription drugs at baseline, and sick leave and disability year before cohort entry;
	ABA	2,021	54	903/100,000 PY	0.86 (0.66;1.13)	0.88 (0.67; 1.16)	1.04 (0.76; 1.42)	

	RTX	3,586	132	985/100,000 PY	0.88 (0.74;1.05)	0.90 (0.75; 1.08)	1.05 (0.84;1.31)	disease duration, DAS28-CRP, CRP, erythrocyte sedimentation rate, HAQ, previous bDMARD use
	TNF-i (first bDMARD)	10,782	434	884/100,000 PY	0.94 (0.85;1.03)	0.94 (0.86; 1.04)	REF	
	TNF-i (second bDMARD)	4,347	153	827/100,000 PY	0.89 (0.76;1.05)	0.91 (0.77; 1.07)	NR	
	csDMARD	46,610	2,910	1,175/100,000 PY	REF	REF	NR	
	General population	107,491	3,883	877/100,000 PY	0.93 (0.84;1.03)	NAP	NR	

4.4.3: Invasive hematologic cancer: Comparison between IL-6R/L blockers and different bDMARDs (observational studies/randomized controlled trials)

Table S4.4.3.1: Baseline characteristics of observational studies investigating IL-6R/L blockers regarding invasive hematologic cancer.

Study	Registry	Inclusion criteria	Exclusion criteria
Wadström 2017 (113)	Swedish Rheumatology Quality of Care Register (SRQ)/ARTIS, Swedish Patient Register Swedish	RA patients treated with TNF-i, non-TNF-i; bDMARD-naive patients with csDMARDs	juvenile idiopathic arthritis, systemic lupus erythematosus, psoriatic arthritis, spondyloarthropathy

	Cancer Register Prescribed Drug Register Total Population Register		

Table S4.4.3.2: Safety outcomes of observational studies regarding invasive hematologic cancer.

Study	Treatment group	N patients	N events	Incidence rate (95% CI)	age/gender aHR (I vs C)	aHR (I vs C)		Adjusted for
Wadström 2017 (113)	TCZ	1,798	3	54/100,000 PY	<5 events	<5 events	<5 events	age, sex, and start-year, comorbidities, educational level, number of hospitalizations and days spent in inpatient care, use of GCs, use of NSAIDs at baseline, number of prescription drugs at baseline, and sick leave and disability year before cohort entry; disease duration, DAS28-CRP, CRP, erythrocyte sedimentation rate, HAQ, previous bDMARD use
	ABA	2,021	9	141 /100,000 PY	1.07 (0.55;2.06)	1.04 (0.53; 2.03)	1.82 (0.81; 4.05)	
	RTX	3,586	17	114 /100,000 PY	0.78 (0.48;1.27)	0.74 (0.45; 1.22)	1.12 (0.62; 2.04)	
	TNF-i (first bDMARD)	10,782	54	104 /100,000 PY	0.85 (0.65;1.10)	0.85 (0.65; 1.10)	REF	
	TNF-i (second bDMARD)	4,347	20	102/100,000 PY	0.85 (0.54;1.33)	0.84 (0.54; 1.32)	NR	
	csDMARD	46,610	448	164/100,000 PY	REF	REF	NR	
	General population	107,491	403	84/100,000 PY	0.71 (0.59;0.85)	NAP	NR	

4.4.4: Non-Hodgkin's Lymphoma: Comparison between IL-6R/L blockers and different bDMARDs (observational studies/randomized controlled trials)

Table S4.4.4.1: Baseline characteristics of observational studies investigating IL-6R/L blockers regarding Non-Hodgkin's Lymphoma.

Study	Registry	Inclusion criteria	Exclusion criteria
Kim 2019 (114)	US health care claims databases: Medicare, IMS PharMetrics Plus, Truven MarketScan	RA (ICD9/10 codes) patients treated with TCZ or TNF-i; ≥1 different biologic agent or tofacitinib prior	nursing home residents, patients with preexisting malignancies at baseline, RTX users
Hellgren 2020 (115)	Swedish Rheumatology Quality Register (SRQ)/ARTIS; Swedish National Patient Register (NPR)	RA patients, ≥18 years of age treated with bDMARDs (TNF-i/non-TNF-i)	patients diagnosed with SLE, JIA, AS

Table S4.4.4.2: Risk of bias analysis (Newcastle-Ottawa Scale [NOS] for Cohort studies)

Study	Representative-ness of exposed cohort	Selection of non-exposed cohort	Ascertainment of exposure	Outcome not presented at start	Comparability of cohort	Assessment of outcome	Follow-up length	Adequacy of follow-up	Summary
Hellgren 2020 (115)	Low	Low	Low	Low	Low	High	Low	Low	High

Table S4.4.4.3: Safety outcomes of observational studies regarding Non-Hodgkin's Lymphoma.

Study	Treatment group	N patients	N events	Incidence rate (95% CI)	age/gender aHR (I vs C)	aHR (I vs C)	Adjusted for
Kim 2019 (114)	Combined TCZ	13,102	11	0.99/1000 PY (0.41;1.58)	NR	1.31 (0.60, 2.88)	sex, age, baseline use of MTX, baseline use of GC, and receipt of any cancer screening tests
	Combined TNF-i	26,727	22	0.91/1000 PY (0.53;1.28)	NR	REF	
Hellgren 2020 (115)*	ETN	6,384	17	51.9/100,000 PY	NR	REF	age, sex, educational level, number of previous bDMARDs and comorbidities until start of follow-up and DAS28 and HAQ at start of bDMARD
	ADA	3,806	15	69.2/100,000 PY	NR	1.02 (0.52;1.99)	
	IFX	3,257	9	51.6/100,000 PY	NR	0.64 (0.27;1.56)	
	CZP	1,644	2	34.4/100,000 PY	NR	NR	
	GOL	1,577	3	57.8/100,000 PY	NR	NR	
	ABA	2,115	7	95.3/100,000 PY	NR	1.61 (0.50;5.22)	
	RTX	3,188	3	20.8/100,000 PY	NR	NR	

Kastrati K, et al. RMD Open 2022; 8:e002359. doi: 10.1136/rmdopen-2022-002359

	TCZ	1,895	2	30.7/100,000 PY	NR	NR	
	ANR	83	1	243.8/100,000 PY	NR	NR	
* reported data include different subtypes of lymphomas<br>ANR: anakinra							

4.4.5: Non melanoma skin cancer (NMSC): Comparison between IL-6R/L blockers and different bDMARDs (observational studies/randomized controlled trials)

Table S4.4.5.1: Baseline characteristics of observational studies investigating IL-6R/L blockers regarding NMSC.

Study	Registry	Inclusion criteria	Exclusion criteria
Wadström 2017 (113)	Swedish Rheumatology Quality of Care Register (SRQ)/ARTIS, Swedish Patient Register Swedish Cancer Register Prescribed Drug Register Total Population Register	RA patients treated with TNF-i, non-TNF-i; bDMARD-naive patients with csDMARDs	juvenile idiopathic arthritis, systemic lupus erythematosus, psoriatic arthritis, spondyloarthropathy

Table S4.4.5.2: Safety outcomes of observational studies regarding NMSC.

Study	Treatment group	N patients	N events	Incidence rate (95% CI)	age/gender aHR (I vs C)	aHR (I vs C)		Adjusted for
Wadström 2017 (113)	TCZ	1,798	5	90/100,000 PY	1.16 (0.48;2.80)	0.93 (0.39; 2.21)	1.04 (0.39; 2.80)	age, sex, and start-year, comorbidities, educational level, number of hospitalizations and days spent in inpatient care, use of GCs, use of NSAIDs at baseline, number of prescription drugs at baseline, and sick leave and disability year before cohort entry; disease duration, DAS28-CRP, CRP, erythrocyte sedimentation rate, HAQ, previous bDMARD use
	ABA	2,021	17	266/100,000 PY	2.98 (1.81;4.90)	2.15 (1.31; 3.52)	2.12 (1.14; 3.95)	
	RTX	3,586	24	159 /100,000 PY	1.38 (0.90;2.11)	1.01 (0.66; 1.55)	1.05 (0.62; 1.77)	
	TNF-i (first bDMARD)	10,782	54	104 /100,000 PY	1.24 (0.95;1.62)	1.09 (0.84; 1.42)	REF	
	TNF-i (second bDMARD)	4,347	17	86/100,000 PY	1.05 (0.66;1.69)	0.86 (0.54; 1.39)	NR	
	csDMARD	46,610	467	171/100,000 PY	REF	REF	NR	
	General population	107,491	263	55/100,000 PY	0.64 (0.46;0.88)	NAP	NR	

4.4.6: Melanoma: Comparison between IL-6R/L blockers and different bDMARDs (observational studies/randomized controlled trials)

Table S4.4.6.1: Baseline characteristics of observational studies investigating IL-6R/L blockers regarding invasive melanoma.

Study	Registry	Inclusion criteria	Exclusion criteria
Wadström 2017 (113)	Swedish Rheumatology Quality of Care Register (SRQ)/ARTIS, Swedish Patient Register Swedish Cancer Register Prescribed Drug Register Total Population Register	RA patients treated with TNF-i, non-TNF-i; bDMARD-naive patients with csDMARDs	juvenile idiopathic arthritis, systemic lupus erythematosus, psoriatic arthritis, spondyloarthropathy
Kim 2019 (114)	US health care claims databases: Medicare, IMS PharMetrics Plus, Truven MarketScan	RA (ICD9/10 codes) patients treated with TCZ or TNF-i; ≥1 different biologic agent or tofacitinib prior	nursing home residents, patients with preexisting malignancies at baseline, RTX users
Mercer 2017 (116)	EULAR RODS Study Group: AIR, ARTIS, ATTRA, BSRBR-RA, DANBIO, GISEA, Orencia and RA, RABBIT, REGistry— RoAcTEmra, Reuma.pt, SCQM	RA patients treated with TNF-i, non-TNF-i; bDMARDs	history of invasive melanoma prior to registration

Table S4.4.6.2: Risk of bias analysis (Newcastle-Ottawa Scale [NOS] for Cohort studies)

Study	Representative-ness of exposed cohort	Selection of non-exposed cohort	Ascertainment of exposure	Outcome not presented at start	Comparability of cohort	Assessment of outcome	Follow-up length	Adequacy of follow-up	Summary
Mercer 2017 (116)	Low	Low	Low	Low	Low	Low	Low	Low	Low

Table S4.4.6.3: Safety outcomes of observational studies regarding invasive melanoma.

Study	Treatment group	N patients	N events	Incidence rate (95% CI)	age/gender aHR (I vs C)	aHR (I vs C)		Adjusted for
Wadström 2017 (113)	TCZ	1,798	3	54/100,000 PY	<5 events	<5 events	<5 events	age, sex, and start-year, comorbidities, educational level, number of hospitalizations and days spent in inpatient care, use of GCs, use of NSAIDs at baseline, number of prescription drugs at baseline, and sick leave and disability year before cohort entry; disease duration, DAS28-CRP, CRP, erythrocyte sedimentation rate, HAQ, previous bDMARD use
	ABA	2,021	7	110/100,000 PY	1.33 (0.61;2.90)	1.43 (0.66; 3.09)	2.39 (0.90; 6.33)	
	RTX	3,586	9	60/100,000 PY	0.69 (0.36;1.35)	0.73 (0.38; 1.39)	1.07 (0.47; 2.45)	
	TNF-i (first bDMARD)	10,782	32	62/100,000 PY	0.85 (0.60;1.18)	0.84 (0.60; 1.18)	REF	

	TNF-i (second bDMARD)	4,347	13	66/100,000 PY	0.92 (0.52;1.61)	0.94 (0.53; 1.66)	NR	
	csDMARD	46,610	234	86/100,000 PY	REF	REF	NR	
	General population	107,491	290	61/100,000 PY	0.84 (0.57;1.23)	NAP	NR	
Kim 2019 (114)	Combined TCZ	13,102	12	1.09/1000 PY (0.47;1.70)	NR	0.71 (0.36;1.40)		sex, age, baseline use of MTX, baseline use of GC, and receipt of any cancer screening tests
	Combined TNF-i	26,727	322	1.36/1000 PY (0.90;1.82)	NR	REF		
Mercer 2017 (116)	TCZ	2,606	4	4.1/1000 PY	NR	NR		NAP
	ABA	1,563	2	4.4/1000 PY	NR	NR		
	RTX	9,431	13	29/1000 PY	NR	NR		
	TNF-i	48,304	106	242/1000 PY	NR	NR		
	bDMARD naive	68,411	160	300/1000 PY	NR	NR		

4.5. Gastrointestinal and hepatic events

4.5.1: Diverticulitis: Comparison between IL-6R/L blockers and different bDMARDs (observational studies/randomized controlled trials)

Table S4.5.1.1: Baseline characteristics of observational studies investigating IL-6R/L blockers regarding diverticulitis.

Study	Registry	Inclusion criteria	Exclusion criteria
Pawar 2019 (101)	US health care claims databases: Medicare, IMS PharMetrics Plus, Truven MarketScan	RA (ICD9/10 codes) patients treated with TCZ, ABA or TNF-i; ≥1 different biologic agent or tofacitinib any time prior	nursing home residents and patients with pre-existing malignancy prior and at the index date; RTX users
Rutherford 2018 a (100)	BSRBR-RA (British)	RA patients treated with bDMARDs	none
Rempenault (EULAR 2020) (117)	French registries: AIR (Autoimmunity and Rituximab), ABA (Orencia and Rheumatoid Arthritis -ORA), TCZ (REGistry–RoAcTEmra-REGATE)	RA patients treated with RTX, TCZ or ABA	NR

Table S4.5.1.2: Risk of bias analysis (Newcastle-Ottawa Scale [NOS] for Cohort studies)

Study	Representative-ness of exposed cohort	Selection of non-exposed cohort	Ascertainment of exposure	Outcome not presented at start	Comparability of cohort	Assessment of outcome	Follow-up length	Adequacy of follow-up	Summary
Rempenault (EULAR 2020) (117)	Abstract	Abstract	Abstract	Abstract	Abstract	Abstract	Abstract	Abstract	Abstract

Table S4.5.1.3: Safety outcomes of observational studies regarding diverticulitis.

Study	Treatment group	N patients	N events	Incidence rate (95% CI)	age/gender aHR (I vs C)	aHR (I vs C)	Adjusted for
Pawar 2019 (101)	Combined TCZ	16,074	70	0.52/100 PY (0.40;0.64)	NR	2.34 (1.64;3.34)	index year, demographics, comorbid conditions, combined comorbidity index, claims-based index of RA severity index, use of DMARDs (during all available data) and other prescription drugs including GC, NSAIDs and analgesics, use of prophylactic antibiotics/antivirals, vaccination, history of any invasive procedures or surgery; cancer screening tests; physician orders of outpatient laboratory tests for acute phase reactants
	Combined TNF-i	33,109	61	0.21/100 PY (0.16; 0.26)	NR	REF	
	Combined ABA	10,414	NR	NR	NR	NR	

Rutherford 2018 a (100)*	ETN	8,630	NR	0.5/100 PY (0.40;0.63)	NR	REF	age, gender, DAS28-ESR, HAQ, disease duration, smoking, seropositivity, polypharmacy, baseline steroid usage
	IFX	4,908	NR	0.51/100 PY (0.38;0.68)	NR	0.95 (0.66;1.38)	
	ADA	7,818	NR	0.38/100 PY (0.29;0.50)	NR	0.77 (0.54;1.11)	
	RTX	5,101	NR	0.58/100 PY (0.41;0.81	NR	0.93 (0.61;1.42)	
	TCZ	2,174	NR	0.76/100 PY (0.46;1.27)	NR	1.45 (0.72;2.90)	
	CZP	1,446	NR	0.18/100 PY (0.06;0.55)	NR	0.51 (0.16;1.63)	
Rempenault (EULAR 2020) (117)	TCZ	1,496	21	5.3/1000 PY	NR	TCZ vs RTX: 4.5 (2.6;7.6) TCZ vs ABA: 3.4 (1.7;6.5)	age, sex, history of diabetes and neoplasia, Charlson Comorbidity Index, number of previous csDMARDs and TNFi, history of TNFi, daily dose of GC at baseline, co-treatment with a csDMARDs, average DAS28 during follow-up, duration of RA, and exposure time to the considered bDMARDs
	RTX	1,986	10	1.6/1000 PY	NR		
	ABA	1,019	10	4.2/1000 PY	NR		
* any GI-infection							

4.5.2: Gastrointestinal perforation (GIP): Comparison between IL-6R/L blockers and different bDMARDs (observational studies/randomized controlled trials)

Table S4.5.2.1: Baseline characteristics of observational studies investigating IL-6R/L blockers regarding GIP.

Study	Registry	Inclusion criteria	Exclusion criteria
Monemi 2016 (118)	US health care claims databases: Truven Health MarketScan Commercial Claims and Encounters (commercial), Medicare Supplemental and Coordination of Benefits (Medicare)	TCZ-IV RA clinical trial all-exposure population, global TCZ postmarketing safety database population, and a US healthcare claims database population of patients with RA, including patients who received TCZ, TNF-i, or ABA.	history of GIP, GI cancer, ulcerative colitis, or Crohn disease during the 12 months prior to the index date
Rempenault (EULAR 2020) (117)	French registries: AIR (Autoimmunity and Rituximab), ABA (Orencia and Rheumatoid Arthritis -ORA), TCZ (REGistry–RoAcTEmra-REGATE)	RA patients treated with RTX, TCZ or ABA	NR
Strangfeld 2017 (119)	RABBIT (German)	RA patients starting atreatment with a bDMARD, or csDMARD after failure of at least one csDMARD.	none
Barbulescu 2020 (120)	SRQ/ARTIS	RA patients (ICD10 codes) treated with bDMARDs	history of any gastrointestinal perforation (upper GIP excluded from main analysis)
Xie 2016 (121)	US health care claims databases: Medicare, MarketScan	RA patients (ICD9 codes) ≥ 18 years, bDMARD or TOFA	any prior diagnosis of GI-perforation (inpatient or outpatient) using all available previous data (minimum of 6 months), diagnosis of inflammatory bowel disease,

			any cancer diagnosis, other than nonmelanoma skin cancer

Table S4.5.2.2: Risk of bias analysis (Newcastle-Ottawa Scale [NOS] for Cohort studies)

**Study**	**Representative-ness of exposed cohort**	**Selection of non-exposed cohort**	**Ascertainment of exposure**	**Outcome not presented at start**	**Comparability of cohort**	**Assessment of outcome**	**Follow-up length**	**Adequacy of follow-up**	**Summary**
Monemi 2016 (118)	Low	Low	High	Low	Low	High	Low	Low	High
Rempenault (EULAR 2020) (117)	Abstract	Abstract	Abstract	Abstract	Abstract	Abstract	Abstract	Abstract	Abstract
Strangfeld 2017 (119)	Low	Low	Low	Low	Low	Low	Low	Low	Low
Barbulescu 2020 (120)	Low	Low	High	Low	Low	High	Low	Low	High
Xie 2016 (121)	Low	Low	High	Low	Low	High	Low	Low	High

Kastrati K, *et al. RMD Open* 2022; 8:e002359. doi: 10.1136/rmdopen-2022-002359

Table S4.5.2.3: Safety outcomes of observational studies regarding GIP.

Study	Treatment group	N patients	N events	Incidence rate (95% CI)[a]	age/gender aHR (I vs C)	aHR (I vs C)		Adjusted for
Monemi 2016 (118)*	Combined TNF-i (ADA, ETN, IFX)	17,333	10	0.6/1000 PY (0.3;1.2)	NR	REF	REF	age, sex, cumulative oral GC and NSAID use 180 days prior to index date, history of diverticulitis, number of prior biologics, and observed duration of RA
	TCZ	3,602	6	1.8/1000 PY (0.7;4.0)	NR	2.2 (0.7;6.6)[a]	2.2 (0.9;5.4)[b]	
	ABA	6,320	5	0.8/1000 PY (0.3;2.0)	NR	NR	NR	
Monemi 2016 (118)**	Combined TNF-i (ADA, ETN, IFX)	17,333	5	0.4/1000 PY (0.1;0.8)	NR	REF	REF	
	TCZ	3,602	5	1.5/1000 PY (0.5;3.6)	NR	4.0 (1.1;14.1)[a]	3.1 (1.1;8.4)[b]	
	ABA	6,320	5	0.8/1000 PY (0.3;2.0)	NR	NR	NR	
Rempenault (EULAR 2020) (117)*	TCZ	1,496	9	2.3/1000 PY	NR	TCZ vs RTX: 2.8 (1.5;5.1)<br>TCZ vs ABA: 5.4 (1.4;19.9)<br>older age (p=0.05), GCs at baseline (p=0.10) and average daily dose of GCs during follow-up (p=0.08) associated with		age, sex, history of diabetes and neoplasia, Charlson Comorbidity Index, number of previous csDMARDs and TNFi, history of TNFi, daily dose of GC at baseline, co-treatment with a csDMARDs, average DAS28 during follow-up, duration of RA, and exposure time to the considered bDMARDs
	RTX	1,986	8	1.3/1000 PY	NR			
	ABA	1,019	2	0.8/1000 PY	NR			

						GIP only in univariate analysis		
Rempenault (EULAR 2020) (117)***	TCZ	1,496	6	1.5/1000 PY	NR	TCZ vs RTX: 3.8(1.7;8.5)		
	RTX	1,986	3	0.5/1000 PY	NR			
	ABA	1,019	2	0.8/1000 PY	NR	TCZ vs ABA: 6.9(1.9;25.4)		
Rempenault (EULAR 2020) (117)****	TCZ	1,496	3	0.7/1000 PY	NR	TCZ vs RTX: 1.4 (0.5;3.9)		
	RTX	1,986	5	0.8/1000 PY	NR			
	ABA	1,019	0	-	NR	TCZ vs ABA: NAP		
Strangfeld 2017 (119)**	csDMARD	4,423	11	0.61/1000 PY (0.3;1.1)	NR	REF		age, sex, treatment with GCs and NSAIDs
	TNF-i	6,711	13	0.52/1000 PY (0.3;0.9)	NR	1.04 (0.48;2.26)		
	TCZ	877	11	2.69/1000 PY (1.4;4.8)	NR	4.48 (2.01;9.99)		
	ABA	371	1	0.51/1000 PY (0.01;2.8)	NR	NR		
	RTX	928	1	0.2/1000 PY (0.01;1.1)	NR	NR		
	other bDMARDs (RTX+ABA)	NR	NR	NR	NR	0.33 (0.08;1.44)		
Barbulescu 2020 (120)**	General population	76,304	333	1.07/1000 PY (0.95;1.33)	NR	REF	NAP[c]	Incidence rates per 1000 person-years were standardized for sex and age

	Bionaïve RA pat.	62,532	570	1.60/1000 PY (1.46;1.74)	NR	1.02	NAP[c]	(categorised in 10-years groups). HRs adjusted (by multivariable Cox regression) for demographic characteristics (age, sex) and cumulated use of GC
	TNF-i	17,594	57	1.84/1000 PY (1.38;3.63)	NR	0.99	REF[c]	
	ABA	2,527	13	3.32/1000 PY (1.66;16.6)	NR	1.41	1.07 (0.55;2.10)[c]	
	RTX	3,552	22	2.02/1000 PY (1.26;5.65)	NR	1.07	0.89 (0.50;1.58)[c]	
	TCZ	2,377	22	4.51/1000 PY (2.68;10.4)	NR	2.36	2.20 (1.28;3.79)[c]	
Xie 2016 (121)*	Combined TNF-i	115,044	109	0.83/1000 PY (0.69;1.00)	NR	NR		age, sex, race, concurrent medications, diabetes, peptic ulcer disease, gastroesophageal reflux disease, diverticulitis, and other gastrointestinal conditions
	ADA	34,787	28	0.97/1000 PY (0.69;1.35)	NR	NR		
	ETN	35,076	34	0.74/1000 PY (0.51;1.07)	NR	NR		
	IFX	28,722	38	0.85/1000 PY (0.62;1.18)	NR	NR		
	ABA	31,214	3	1.07/1000 PY (0.79;1.45)	NR	NR		
	RTX	4,391	43	0.73/1000 PY (0.15;2.12)	NR	NR		
	TCZ	11,705	16	1.55/1000 PY (0.95;2.54)	NR	NR		
	TOFA	4,755	3	0.86/1000 PY (0.10;3.60)	NR	NR		

Xie 2016 (121)**	Combined TNF-i	115,044	59	0.46/1000 PY (0.35;0.58)	NR	REF	
	ADA	34,787	17	0.48/1000 PY (0.30;0.78)	NR	NR	
	ETN	35,076	18	0.47/1000 PY (0.30;0.75)	NR	NR	
	IFX	28,722	20	0.46/1000 PY (0.30;0.71)	NR	NR	
	ABA	31,214	30	0.76/1000 PY (0.53;1.09)	NR	1.41 (0.90;2.21)	
	RTX	4,391	2	0.48/1000 PY (0.06;1.75)	NR	1.72 (0.52;5.69)	
	TCZ	11,705	13	1.26/1000 PY (0.73;2.18)	NR	2.55 (1.33;4.88)	
	TOFA	4,755	2	0.86/1000 PY (0.10;3.60)	NR	3.24 (1.05;10.04)	
Xie 2016 (121)#	Combined TNF-i	115,044	49	0.38/1000 PY (0.28;0.50)	NR	NR	
	ADA	34,787	17	0.48/1000 PY (0.30;0.78)	NR	NR	
	ETN	35,076	10	0.26/1000 PY (0.14;0.49)	NR	NR	
	IFX	28,722	17	0.39/1000 PY (0.24;0.63)	NR	NR	
	ABA	31,214	12	0.31/1000 PY (0.17;0.54)	NR	NR	

	RTX	4,391	1	0.24/1000 PY (0.01;1.35)	NR	NR	
	TCZ	11,705	3	0.29/1000 PY (0.06;0.85)	NR	NR	
	TOFA	4,755	0	0.00/1000 PY (0.00;1.58)	NR	NR	

* any GIP
** lower GIP
*** GIP due to diverticulitis (diverticular GIP)
**** GIP due to another etiology
# upper GIP

[a] specific definition: inpatient admissions with evidence of perforation based on presence of the word perforation in ICD-9-CM diagnosis for esophageal rupture; gastric, duodenal, peptic, or gastrojejunal ulcers; and unspecified GIP (appendicitis, diverticulitis, diverticulosis, or ischemic colitis associated with surgical GI procedures not included)

[b] sensitive definition: inpatient admissions with evidence of perforation based on (1) presence of the word perforation in ICD-9-CM diagnosis descriptions: esophageal rupture; gastric, duodenal, peptic, or gastrojejunal ulcers; appendicitis; and GI perforation of an unspecific location in the large intestine or (2) an ICD-9-CM diagnosis of diverticulitis, diverticulosis, or ischemic colitis plus a Current Procedural Terminology code for suture or resection of the small or large intestine

[c] lower GI perforations, crude and IPTW-adjusted incidence rates and contrasts between non-TNFi and TNFi. IPTW adjustment for age, sex, education level, year of treatment start, disease history (GI perforations, diverticular disease, intestinal vascular disease, inflammatory bowel disease, other GI disorders, diabetes, chronic obstructive pulmonary disease, hospitalised infections, cardiovascular disease, cancer, joint surgery, number of hospitalisations), RA duration, rheumatoid factor, erythrocyte sedimentation rate, CRP, DAS28-CRP, HAQ, comedication with MTX, other csDMARDs, selective COX2 inhibitors, NSAIDs, GC and cumulated use of GCs and of NSAIDs

4.5.3: Hepatic events:

Table S4.5.3.1: Baseline characteristics of observational studies investigating IL-6R/L blockers regarding hepatic events.

Study	Registry	Inclusion criteria	Exclusion criteria
Koike 2014 (122)	Post-marketing data, Japan	RA patients treated with TCZ	NR
Genovese 2017 (123)	Pooled data from RCTs (five phase 3 studies and one phase 4 study) and long-term extension studies	All-exposure population, RA patients who received ≥1 dose of TCZ at 4 mg/kg, 8 mg/kg, or 10 mg/kg and who had ≥1 postbaseline safety assessment	in all trials, pat. were excluded from entering the study if they had alanine aminotransferase (ALT) or aspartate aminotransferase (AST) levels >1.5x the upper limit of normal (ULN) at screening. Patients with known active current or history of recurrent hepatitis B and C, history of alcohol or chemical abuse during the 6 months before screening, evidence of serious uncontrolled concomitant hepatic disease, or current hepatic disease as determined by the principal investigator were also excluded from the clinical trials

Table S4.5.3.2: Risk of bias analysis (Newcastle-Ottawa Scale [NOS] for Cohort studies)

Study	Representative-ness of exposed cohort	Selection of non-exposed cohort	Ascertainment of exposure	Outcome not presented at start	Comparability of cohort	Assessment of outcome	Follow-up length	Adequacy of follow-up	Summary
Koike 2014 (122)	Low	Low	High	Low	High	High	Low	Low	High
Genovese 2017 (123)	Low	Low	Low	Low	Low	Moderate	Low	Low	Moderate

Table S4.5.3.3: Safety outcomes of observational studies regarding hepatic events I

Study	Treatment group	N patients	N events hepatic events	Incidence rate hepatic events	N patients history or carrier of hepatitis B/C	N events hepatitis B/C virus reactivation	Incidence rate hepatitis B/C virus reactivation	Adjusted for
Koike 2014 (122)	TCZ	7,901	28	0.84/100 PY	Hep B n= 52<br>Hep C n=76	0	NAP	-
Genovese 2017 (123)	TCZ	4,171	NR	0.78/100 PY<br><br>hepatic serious AEs: 0.04/100 PY	NR	NR	NR	-

Table S4.5.3.4: Safety outcomes of observational studies regarding hepatic events (transaminase elevations) II

Study	Treatment group	N patients	AST elevation greater ULN (%)	ALT elevation greater ULN (%)	AST elevation >1-3x ULN (%)	ALT elevation >1-3x ULN (%)	AST elevation >3-5x ULN (%)	ALT elevation >3-5x ULN (%)	AST elevation >5x ULN (%)	ALT elevation >5x ULN (%)	Single ALT/AST elevation >3x ULN (%)	Elevations >3x ULN returning to normal (%)
Genovese 2017 (123)	TCZ	4,171	70.6	59.4	59	55	8.9	3.3	2.9	0.9	7.7/3.6	80
ALT: alanine aminotransferase; AST: aspartate aminotransferase; ULN: upper limit of normal												

4.6. Adverse events of special interest

4.6.1: Withdrawal and immunologic events: Comparison between IL-6R/L blockers and different bDMARDs (observational studies/randomized controlled trials)

Table S4.6.1.1: Safety outcomes of observational studies investigating IL-6R/L blockers regarding withdrawals.

Study	Registry	Treatment group	Type of ratio	Incidence rate (95% CI)	age/gender aHR (I vs C)	aHR (I vs C)	Risk of bias assessment
Brodszky 2017 (124)	Debrecen Medical and Health Sciences Center	TCZ	HR	NR	NR	0.474 (p=0.151)	Low
		CZP		NR	NR	REF	
		ETA		NR	NR	0.63 (p=0.265)	
		ADA		NR	NR	0.554 (p=0.152)	
		RTX		NR	NR	0.505 (p=0.139)	
		IFX		NR	NR	1.04 (p=0.923)	
		GOL		NR	NR	0.918 (p=0.854)	
Ebina 2018 (125)	ANSWER cohort	TCZ	HR	NR	NR	0.90 (0.44;1.84)	Low
		IFX		NR	NR	REF	
		ABA		NR	NR	0.53 (0.24;1.19)	
		ADA		NR	NR	1.06 (0.46;2.40)	
		CZP		NR	NR	0.77 (0.32;1.84)	
		ETN		NR	NR	0.73 (0.33;1.64)	

		GOL		NR	NR	0.85 (0.39;1.83)	
Ebina 2020 (126)	ANSWER cohort	ADA	HR	NR	NR	1.8 (1.0;3.1)	Low
		CZP		NR	NR	0.8 (0.3;2.0)	
		ETN		NR	NR	0.4 (0.2;0.9)	
		GOL		NR	NR	1.0 (0.6;1.9)	
		IFX		NR	NR	1.2 (0.5;2.7)	
		TCZ		NR	NR	1.4 (0.9;2.3)	
		TOF		NR	NR	1.8 (0.9;3.5)	
		ABA		NR	NR	REF	
Ebina 2019 (127)	ANSWER cohort (elderly ≥65 years of age)	ADA	HR	NR	NR	3.16 (1.36;7.35)	Low
		CZP		NR	NR	2.23 (0.61;8.15)	
		ETN		NR	NR	2.50 (1.15;5.43	
		GOL		NR	NR	3.58 (1.63;7.82)	
		IFX		NR	NR	3.62 (1.58;8.26)	
		TCZ		NR	NR	3.04 (1.45;6.38)	
		ABA		NR	NR	REF	
Gottenberg 2019 (128)	AIR ORA REGATE	TCZ	life expectancy difference without failure after IPW	NR	NR	0.5 (−0.4;1.4)	Low
		ABA		NR	NR	REF	
		RTX		NR	NR	0.3 (−0.4;1.0)	

Table S4.6.1.2: Safety outcomes of observational studies investigating IL-6R/L blockers regarding immunologic events.

Study	Registry	Treatment group	N events	Type of ratio	Incidence rate (95% CI)	age/gender aHR (I vs C)	aHR (I vs C)	Risk of bias assessment
Yun 2017 (129)	US claims data (Medicare)	TCZ (iv)	13	Adjusted RR (95% CI) with first dose	155.5/10^6 (90.3;267.8)	NR	22.2 (11.6;42.4)	High
		RXT (iv)	16		239.5/10^6 (146.7;390.9)	NR	18.0 (8.9; 36.2)	
		ABA (iv)	16		41.1/10^6 (25.2;67.1)	NR	7.1 (3.9; 12.8)	
		IFX (iv)	48		145.1/10^6 (109.3;192.5)	NR	26.9 (17.4–41.5)	
		GOL (iv)	0		0/10^6 (0;153.703)	NR	NAP	
		ABA (sc)	0		0/10^6 (0; 175.8)	NR	NAP	
		TCZ (sc)	0		0/10^6 (0; 38.425)	NR	NAP	
		TNFi (sc)	44		5.8/10^6 (4.3; 7.8)	NR	REF	
Salmon 2018 (130)	AIR	RTX (iv)	56	-	0.7/100 PY	NR	NR	Low
	ORA	TCZ (iv)	29		1/100 PY	NR	NR	
	REGATE	ABA (iv)	15		0.6/100 PY	NR	NR	

4.6.2: Changes in lipid profile: Comparison between IL-6R/L blockers and different bDMARDs (observational studies/randomized controlled trials)

Table S4.6.2.1: Baseline characteristics of RCTs investigating IL-6R/L blockers regarding changes in lipid profile.

Study	Registry	Inclusion criteria	Exclusion criteria	Risk of bias assessment
McInnes 2015 (MEASURE) (131)	RCT	moderately to severely active RA, MTX-IR	initiation of lipid-lowering, oral antidiabetic or antihypertensive medications or change in dose within 12 weeks of baseline was prohibited, and GC (≤10 mg) had to remain stable.	Low
Gabay 2016 (132)	Post-hoc analysis of ADACTA trial (phase IV)	RA patients, MTX-IR, receiving ADA or TCZ	none	High

Table S4.6.2.2: Outcomes of RCTs investigating IL-6R/L blockers regarding changes in lipid profile.

Study	Primary / Secondary outcome	Timepoint (weeks)	Treatment arm	No. of patients (n)	Result	p / 95% CI
McInnes 2015 (MEASURE) (131)*	total cholesterol (median Δ from baseline); LDL-C (median Δ from baseline); HDL-C (median Δ from baseline); triglycerides (median Δ from baseline); total cholesterol/HDL ratio (median Δ	12	Placebo + MTX	63	-; -1.9; 2.4; 2.2; 0.9; 2.5; -0.99; -0.47	10.4 (4.8;16.9) p=0.0004; 11.0 (3.8;18.6) p=0.0076; 3.0 (-2.4;8.6) p=0.2753;
			TCZ + MTX	69	12.6; 10.6; 3.1; 28.1; 11.3; 4.7; -0.21; -0.17	

	from baseline); ApoB/ApoA1 ratio (median Δ from baseline); mean change from baseline in PWV (pulse wave velocity) m/s (week 12); mean change from baseline in PWV (pulse wave velocity) m/s (week 24)					25.4 (10.1;40.8) p=0.0011; 9.7 (4.3;14.5) p=0.0008; 2.1(–4.1;7.9) p=0.5108; 0.22 to 1.35 (p=0.0067); -0.27 to 0.87 (p=0.3042)
Gabay 2016 (132)	total cholesterol (mean Δ from baseline); triglycerides (mean Δ from baseline); LDL-C (mean Δ from baseline); HDL-C (mean Δ from baseline); total cholesterol/HDL ratio (mean Δ from baseline); HDL-SAA (median Δ from baseline); sPLA$_2$ IIA (median Δ from baseline); Lp(a) (mean Δ from baseline)	8	ADA 40 mg SC Q2W	162	0.17; 0.07; 0.07; 0.07; -0.01; -1.1; -1.3; -1.1	0.67 (0.47;0.86) p<0.0001; 0.24 (0.10;0.38) p= 0.0008; 0.46 (0.30;0.62) p<0.0001; 0.07 (0.001;0.14) p=0.0453; 0.27 (0.12;0.42) p=0.0005; p=0.0077; p<0.0001; p<0.0001
			TCZ 8 mg/kg IV Q4W	162	0.79; 0.29; 0.52; 0.14; 0.24; -3.2; -4.1; -7.6	

HDL: high-density lipoprotein; HDL-C: high-density lipoprotein cholesterol; LDL: low-density lipoprotein; LDL-C: low-density lipoprotein cholesterol; HDL-SAA: high-density lipoprotein-associated serum amyloid A; sPLA$_2$ IIA: secretory phospholipase A$_2$ II; Lp(a): lipoprotein (a)

* part 1: 24 wks; TCZ 8 mg/kg q4w (n=69) or placebo (n=63) (MTX continued in both groups); part 2: open label follow-up 24-104 wks; HDL-associated serum amyloid A content decreased in TCZ recipients. TCZ induced reductions (>30%) in secretory phospholipase A2-IIA, lipoprotein(a), fibrinogen and D-dimers and elevation of paraoxonase (all p<0.0001 vs PBO).

4.6.3: Diabetes and changes in HbA1c: Comparison between IL-6R/L blockers and different bDMARDs (observational studies/randomized controlled trials)

Table S4.6.3.1: Safety outcomes of observational studies investigating IL-6R/L blockers regarding risk of diabetes treatment intensification and switching to insulin.

Study	Registry	Treatment group	N events	Type of ratio	Incidence rate (95% CI)	age/gender aHR (I vs C)	aHR (I vs C)	Risk of bias assessment
Chen 2020 (133)	US claims data (MarketScan)	TCZ	94	HR	182.7/1000 PY (149.2; 223.6)	NR	0.94 (0.74;1.19)	High
		TNF-i	875		185.4/1000 PY (173.5; 198.1	NR	0.97 (0.82;1.15)	
		RTX	124		198.0/1000 PY (166.0; 236.1)	NR	0.99 (0.79;1.23)	
		ABA	248		196.2/1000 PY (173.2; 222.2)	NR	REF	
		TOFA	58		148.2/1000 PY (114.6; 191.7	NR	0.67 (0.50;0.90)	

Table S4.6.3.2: Baseline characteristics of post hoc analyses investigating effects of IL-6R/L blockers vs. TNF-i on glycosylated hemoglobin (HbA1c) in patients with RA and subgroups of patients with RA and diabetes

Study	Registry	Inclusion criteria	Exclusion criteria	Risk of bias assessment
Genovese/Burmester 2020 (134)	post-hoc analysis of phase III study (MOBILITY, TARGET, MONARCH)	moderately to severely active RA, MTX-IR, TNFi-IR; pat. with diabetes were identified by medical history or use of antidiabetic medication	pat. with HbA1c ≥ 9% were excluded from all 3 studies	High

Table S4.6.3.3: Outcomes of post hoc analyses investigating effects of IL-6R/L blockers vs. TNF-i on glycosylated hemoglobin (HbA1c) in patients with RA and subgroups of patients with RA and diabetes.

Study	Primary / Secondary outcome	Timepoint (weeks)	Treatment arm	No. of patients (n)	Result	p-value vs. placebo/adalimumab
Genovese/Burmester 2020 (134)	*Change from baseline at week 24:*<br>a) Patients with a medical history of diabetes or baseline use of antidiabetic medication: LS mean difference (95% CI)	24	Placebo + csDMARDs	15		
			SAR 150 mg Q2W + csDMARDs	16	-0.47	0.0021
			SAR 200 mg Q2W + csDMARDs	15	-0.67	< 0.0001
			ADA 40 mg Q2W monotherapy	14		
			SAR 200 mg Q2W monotherapy	6	-0.43	0.0257
	b) baseline HbA1c ≥ 7.0%: LS mean difference (95% CI)	24	Placebo + csDMARDs	11		
			SAR 150 mg Q2W + csDMARDs	10	-0.48	0.0097
			SAR 200 mg Q2W + csDMARDs	9	-0.69	0.0003
			ADA 40 mg Q2W monotherapy	6		
			SAR 200 mg Q2W monotherapy	4	-0.96	0.0002
LS: least squares						

4.6.4: Effects on anemia and risk of neutropenia: Comparison between IL-6R/L blockers and different bDMARDs (observational studies/randomized controlled trials)

Table S4.6.4.1: Safety outcomes of observational studies investigating IL-6R/L blockers regarding changes in hemoglobin in patients with anemia at index date.

Study	Registry	Treatment group	N patients	Mean Hb (95% CI) (g/dL) index date	Δ Hb at 6 months (g/dL) mean (95% CI)	Δ Hb at 12 months (g/dL) mean (95% CI)	Δ Hb at 24 months (g/dL) mean (95% CI)	Risk of bias assessment
Paul 2018 (135)	Centricity Electronic Medical Record (CEMR)	TCZ	3,732	12.06 (11.98;12.14	0.40 (0.24;0.56)	0.55 (0.32;0.78)	0.72 (0.36;1.08)	High
		TOFA	3,126	11.89 (11.81;11.97)	0.40 (0.22;0.58)	0.46 (0.15;0.76)	0.58 (0.05;1.11)	
		obDMARD	55,964	11.90 (11.87;11.92)	0.20 (0.16;0.24)	0.25 (0.21;0.30)	0.35 (0.29;0.41)	
		onbDMARD	91,236)	11.86 (11.84;11.88)	0.17 (0.14;0.19)	0.21 (0.18;0.24)	0.26 (0.22;0.30)	

Hb: hemoglobin; obDMARD: biologic DMARDs excluding tocilizumab; onbDMARD: non-biologic DMARDs excluding tofacitinib;
treatment groups balanced on sex and baseline measures, analysis was adjusted for age, sex, and duration of RA, history of CVD, CKD, cancer, and diabetes prior to index date

Table S4.6.4.2: Safety outcomes of observational studies investigating IL-6R/L blockers regarding changes in neutrophils and neutropenia associated risk of infection.

Study	Registry	Treatment group	N patients	Neutrophils Grade 1 (n;%)	Neutrophils Grade 2 (n;%)	Neutrophils Grade 3 (n;%)	Neutrophils Grade 4 (n;%)	Serious infections around grade 1/2 neutrophil count (100 PY [95% CI])	Serious infections around grade 3/4 neutrophil count (100 PY [95% CI])	Risk of bias assessment
Moots 2017 (136)	pooled analysis of data from phase II and IV clinical trials	Placebo controlled pooled: Placebo+ DMARDs	1454	88 (6.1)	41 (2.8)	3 (0.2)	0	10.48 (2.16;30.62)	0	High
		Placebo controlled pooled: all TCZ	2644	461 (17.4)	284 (10.7)	73 (2.8)	8 (0.3)	2.40 (0.88;5.22)	0	
		LTE all-exposure population: DMARD-IR	2904	655 (22.6)	554 (19.1)	164 (5.6)	17 (0.6)	2.22 (1.49;3.19)	1.97 (0.05;10.99)	
		LTE all-exposure population: TNFi-IR	464	101 (21.8)	56 (12.1)	22 (4.7)	5 (1.1)	3.68 (1.19;8.59)	0	

		LTE all-exposure population: MTX-naive	417	83 (19.9)	84 (20.1)	28 (6.7)	2 (0.5)	3.32 (1.22;7.22)	9.70 (0.25;54.05)	
		LTE all-exposure population: all TCZ*	4163	900 (21.6)	757 (18.2)	223 (5.4)	27 (0.6)	2.48 (1.79;3.34)	2.77 (0.34;10.01)	
Grade 1 neutrophil count is defined as ANC < lower limit of normal to $1.5x10^9$/l; grade 2, ANC <1.5 to $1.0x10^9$; grade 3, ANC <1.0 to $0.5x10^9$/l; grade 4, ANC <$0.5x10^9$/l. ANC: absolute neutrophil count<br>* number of patients who had neutrophil measurements										

4.6.5: Renal insufficiency: Comparison between IL-6R/L blockers and different bDMARDs (observational studies/randomized controlled trials)

Table S4.6.5.1: Safety outcomes of observational studies investigating IL-6R/L blockers in patients with RA and renal insufficiency

Study	Registry	Treatment group	N patients	Hemoglobin, g/dL, mean (95% CI) Week 0	Hemoglobin, g/dL, mean (95% CI) Week 24	Anemia, n (%) Week 0	Anemia, n (%) Week 24	Δ hemoglobin, mean (95% CI)	Risk of bias assessment
Mori 2015 (ACTRA-RI) (137)	ACTRA-RI study	Patients with renal insufficiency: TCZ	64	11.5 (11.1;11.9)	12.5 (12.1;12.9)	36 (56.3)	24 (37.5)	0.96 (0.67;1.26)	High

		Patients with renal insufficiency: TCZ + MTX	28	11.5 (10.9;12.0)	12.0 (11.5;12.4)	12 (42.9)	9 (32.1)	0.48 (0.16;0.81)	
		Patients without renal insufficiency: TCZ	106	12.3 (12;12.6)	13.2 (12.9;13.5)	36 (34)	21 (19.8)	0.89 (0.61;1.16)	
		Patients without renal insufficiency: TCZ + MTX	173	12.1 (11.9;12.4)	12.9 (12.7;13.2)	65 (37.6)	25 (14.5)	0.81 (0.65;0.98)	
				**Discontinuation within the first 24 weeks**	**Adverse events within the first 24 weeks, number (%)**	**Severe adverse events, number (%)**			
		With renal insufficiency (n=102) MTX user, number (%)		5/33 (15.2)	4/33 (12.1)	1/33 (3.0)			
		With renal insufficiency (n=102) MTX non-user, number (%)		4/69 (5.8)	5/69 (7.2)	4/69 (5.8)			
		Without renal insufficiency		15/188 (8.0)	9/188 (4.8)	4/188 (2.1)			

		(n=303) MTX user, number (%)						
		Without renal insufficiency (n=303) MTX non-user, number (%)		10/115 (8.7)	7/115 (6.1)	2/115 (1.7)		

4.6.6: Interstitial lung disease (ILD): Comparison between IL-6R/L blockers and different bDMARDs (observational studies/randomized controlled trials)

Table S4.6.6.1: Safety outcomes of observational studies investigating IL-6R/L blockers regarding risk of ILD and is complications.

Study	Registry	Treatment group	N patients	N events	Type of ratio	Incidence rate (95% CI)	age/gender aHR (I vs C)	aHR (I vs C)	Risk of bias assessment
Curtis 2015 (138)	US claims data (MarketScan; Medicare)	TNFi	7,951	9	HR	1.6/1000 PY (0.8;3.1)	NR	REF	High
		TCZ	1,528	1		1/1000 PY (0;5.5)	NR	0.5 (0.06;4.0)	
		RTX	1,134	4		4.7/1000 PY (1.3;12.1)	NR	2.2 (0.67;7.25)	
		ABA	2,683	2		1.1/1000 PY (0.1;4.1)	NR	0.6 (0.13;2.84)	
		ETN	NR	0		0/1000 PY (0;3)	NR	NR	
		ADA	NR	3		1.8/1000 PY (0.4;5.2)	NR	NR	
		IFX	NR	3		4.1/1000 PY (0.8;12.0)	NR	NR	

		CZP	NR	3		3.2/1000 PY (0.7;9.3)	NR	NR	
		GOL	NR	0		0/1000 PY (0;2.7)	NR	NR	

4.6.7: Neurological AEs: Comparison between IL-6R/L blockers and different bDMARDs (observational studies/randomized controlled trials)

Table S4.6.7.1: Safety outcomes of observational studies investigating IL-6R/L blockers regarding risk of idiopathic facial nerve palsy.

Study	Registry	Treatment group	N patients	N events	Type of ratio	Incidence rate (95% CI)	age/gender aHR (I vs C)	aHR (I vs C)	Risk of bias assessment
Strangfeld 2019 (EULAR Abstract) (139)	RABBIT	csDMARDs	NR	3	HR	0.2/1000 PY (0.0;0.5)	NR	NR	Abstract
		ETN* (original)	NR	4		0.7/1000 PY (0.2;1.6)	NR	NR	
		ETN (biosimilar, SB4)	NR	1		1.9/1000 PY (0.1;6.9)	NR	NR	
		GOL	NR	1		0.7/1000 PY (0.0;2.4)	NR	NR	
		RTX*	NR	5		0.8/1000 PY (0.3;1.6)	NR	NR	
		ABA	NR	1		0.3/1000 PY (0.0;1.2)	NR	NR	
		TCZ	NR	3		0.5/1000 PY (0.1;1.1)	NR	NR	
* n=1 patient was exposed to both ENT (original) and RTX at the time of event.									

4.6.8: Bone mineral density and osteoporosis: Comparison between IL-6R/L blockers and different bDMARDs (observational studies/randomized controlled trials)

Table S4.6.8.1: Safety outcomes of observational studies investigating IL-6R/L blockers regarding risk of osteoporotic fracture and other subtypes of fractures.

Study	Registry	Treatment group	N patients	N events osteoporotic fracture	Type of ratio	Incidence rate (95% CI)	age/gender aHR (I vs C)	HR (95% CI) TNF-i vs. TCZ	Risk of bias assessment
Shin 2019 (EULAR Abstract) (140)	Korean National Health Insurance Service datasets	TNF-i	2,339	54	HR	1.69/100 PY	NR	1.00 (0.53;1.92)	Abstract
		TCZ	647	4		0.7/100 PY	NR		
		**Treatment group**	**N patients**	**N events spinal fracture**	**Type of ratio**	**Incidence rate (95% CI)**	**age/gender aHR (I vs C)**	**HR (95% CI) TNF-i vs. TCZ**	
		TNF-i	2,339	29	HR	0.90/100 PY	NR	0.98 (0.43;2.24)	
		TCZ	647	10		1.27/100 PY	NR		
		**Treatment group**	**N patients**	**N events no-spinal fracture**	**Type of ratio**	**Incidence rate (95% CI)**	**age/gender aHR (I vs C)**	**HR (95% CI) TNF-i vs. TCZ**	
		TNF-i	2,339	25	HR	0.78/100 PY	NR	1.03 (0.36;2.90)	

		TCZ	647	6		0.76/100 PY	NR		

Table S4.6.8.2: Outcomes of prospective studies investigating effects of IL-6R/L blockers on bone mineral density.

Study	Primary / Secondary outcome	Timepoint (weeks)	Treatment arm	No. of patients (n)	Result	p-value	Risk of bias assessment
Kume 2014 (141)	Lumbar spine:<br>BMD at week 52, mean (S.D.), g/cm^2<br>-all patients<br>-Normal BMD at baseline<br>-Osteopenia at baseline	52	TCZ 8mg/kg IV Q4W+MTX<br>(no GC, no bisphosphonates or PTH)	86	0.986 (0.21), 1.091 (0.14), 0.843 (0.18)	0.12, 0.24, 0.02	High
	Femoral neck:<br>BMD at week 52, mean (S.D.), g/cm^2<br>-all patients<br>-Normal BMD at baseline<br>-Osteopenia at baseline	52	TCZ 8mg/kg IV Q4W+MTX<br>(no GC, no bisphosphonates or PTH)	86	0.826 (0.12), 0.919 (0.14), 0.698 (0.21)	0.27, 0.19, 0.03	
Chen 2017 (142)	Lumbar spine<br>-BMD (g/cm2)<br>- T-score	104	TCZ 4 or 8mg/kg IV Q4W +csDMARD + stable GC<br>(no antiosteoporosis medication)<br>**ACPA-positive**	54	0.93, -0.99;<br>0.67, -1.76;<br>0.66, -1.76	0.087, 0.027;	High

Kastrati K, et al. RMD Open 2022; 8:e002359. doi: 10.1136/rmdopen-2022-002359

	Femoral neck, right<br>-BMD (g/cm2)<br>- T-score<br><br>Femoral neck, left<br>-BMD (g/cm2)<br>- T-score		TCZ 4 or 8mg/kg IV Q4W +csDMARD + stable GC<br>(no antiosteoporosis medication)<br>**ACPA-negative**	22	1.08, -0.17;<br>0.81, -0.98;<br>0.82, -0.88	0.046, 0.043;<br>0.064; 0.036	
ACPA: anticitrullinated protein antibody; BMD: bone mineral density							

4.6.9: Pregnancy: Clinical trials and post-marketing data.

Table S4.6.9.1: Pregnancy outcome after exposure to IL-6R inhibition.

Study	Registry	Treatment group	N patients	Live birth, n (%)	Liveborn children, n	Spontaneous abortion, n (%)	ETOP, n (%)	Stillbirth, n	Mal-formation, n (%)	Preterm birth, n (%)	Risk of bias assessment
Hoeltzenbein 2016 (143)	Roche Global Safety Database	TCZ Exposure Prospective	180	109 (60.6%)	111	39 (21.7%)	31 (17.2%)	1	5/111 (4.5%)	29/93 (31.1%)	High
		TCZ Exposure Retrospective	108	55 (50.9%)	56	31 (28.7%)	22 (20.4%)	0	NR	2/56 (20.0%)	
ETOP: elective termination of pregnancy.<br><br>no increased risks for adverse pregnancy outcomes were observed after paternal exposure in n=13 pregnancies with known outcome.											

Table S4.6.9.2: Pregnancy outcome after exposure to IL-6R inhibition.

Study	Registry	Treatment group	N patients	Liveborn children, n	Spontaneous abortion, n	Induced abortion, n	Congenital malformations, n	Risk of bias assessment
Weber-Schoendorfer 2016 (144)	Pharmakovigilanzzentrum<br><br>Embryonaltoxikologie (further referred to as Embryotox Berlin)	TCZ Exposure Prospective	16 (maternal)	11	4	1	0	High

4.6.10: Randomized controlled trials (RCTs) and long-term extension studies (LTEs)

Table S4.6.10.1: Sarilumab: Overview of RCTs.

Study	Trial	Treatment	Risk of bias assessment
Emery 2019 (57)	ASCERTAIN	TCZ 4 or 8 IV Q4W + DMARD	Unclear
		SAR 150 Q2W + DMARD	
		SAR 200 Q2W + DMARD	
	1309 (OLE)	TCZ 4 IV Q4W + MTX	High
		TCZ 8 IV Q4W + MTX	
		SAR 150 + MTX	
		SAR 200 + MTX	

Tanaka 2019 (3)	KAKEHASI<br>(PBO period)	PBO + MTX	Low
		SAR 150 Q2W + MTX	
		SAR 200 Q2W + MTX	
Kameda 2020 (145)	HARUKA	SAR 150 Q2W	Low
		SAR 200 Q2W	
		SAR 150 Q2W + nMTX	
		SAR 200 Q2W + nMTX	

Table S4.6.10.2: Sarilumab: Rates of serious AEs, serious infections, opportunistic infections, deaths, malignancies and CVE (RCTs).

Study	Trial	Treatment group	N patients	Any serious AE n (%)	Serious infections n (%)	OI n (%)	Any major CVE n (%)	Any malignancy n (%)	Deaths of any cause n (%)
Emery 2019 (57)	ASCERTAIN	TCZ 4 or 8 IV Q4W + DMARD	101	7 (6.9)	2 (2.0)	0	0	NR	1 (1.0)
		SAR 150 Q2W + DMARD	49	1 (2.0)	0	0	0	NR	NR
		SAR 200 Q2W + DMARD	51	3 (5.9)	1 (2.0)	0	0	NR	NR
	1309 (OLE)	TCZ 4 IV Q4W + MTX	25	0	0	0	0	NR	NR
		TCZ 8 IV Q4W + MTX	24	1 (4.2)	0	0	0	NR	NR
		SAR 150 + MTX	26	0	0	0	0	NR	NR
		SAR 200 + MTX	26	0	0	0	0	NR	NR
		PBO + MTX	81	6 (7.4)	0	0	0	0	0

Kastrati K, *et al. RMD Open* 2022; 8:e002359. doi: 10.1136/rmdopen-2022-002359

Tanaka 2019 (3)	KAKEHASI (PBO period)	SAR 150 Q2W + MTX	81	4 (4.9)	5 (6.2)	1 (1.2)	0	0	0
		SAR 200 Q2W + MTX	80	4 (5.0)	0	0	0	1 (1.3)	0
Kameda 2020 (145)	HARUKA	SAR 150 Q2W	30	1 (3.3)	1 (3.3)	0	0	0	0
		SAR 200 Q2W	31	2 (6.5)	1 (3.2)	0	0	2 (13.3)	0
		SAR 150 Q2W + nMTX	15	0	0	0	0	0	0
		SAR 200 Q2W + nMTX	15	3 (20.0)	1 (6.7)	0	0	0	0

Table S4.6.10.3: Sarilumab: Rates of GIP, injection-site / infusion reactions, immunogenicity and neutropenia (RCTs).

Study	Trial	Treatment group	N patients	GIP n (%)	Inj/Inf reaction n (%)	ADA n (%)	Any neutropenia n (%)
Emery 2019 (57)	ASCERTAIN	TCZ 4 or 8 IV Q4W + DMARD	101	NR	1 (1.0)	NR	4 (3.9)
		SAR 150 Q2W + DMARD	49	NR	4 (8.2)	NR	6 (12.2)
		SAR 200 Q2W + DMARD	51	NR	4 (7.8)	NR	8 (15.7)
	1309 (OLE)	TCZ 4 IV Q4W + MTX	25	NR	NR	NR	NR
		TCZ 8 IV Q4W + MTX	24	NR	NR	NR	NR
		SAR 150 + MTX	26	NR	NR	NR	NR
		SAR 200 + MTX	26	NR	NR	NR	NR
Tanaka 2019 (3)	KAKEHASI (PBO period)	PBO + MTX	81	0	3 (3.7)	1 (1.2)	0
		SAR 150 Q2W + MTX	81	0	11 (13.6)	1 (1.2)	7 (8.6)

		SAR 200 Q2W + MTX	80	0	12 (15.0)	1 (1.3)	9 (11.3)
Kameda 2020 (145)	HARUKA	SAR 150 Q2W	30	0	1 (3.3)	5 (16.7)	1 (3.3)
		SAR 200 Q2Q	31	0	6 (19.4)	2 (6.5)	3 (9.7)
		SAR 150 Q2W + nMTX	15	0	0	0	5 (33.3)
		SAR 200 Q2W + nMTX	15	0	1 (6.7)	0	3 (20.0)

Table S4.6.10.4: Sarilumab: Rates of serious AEs, deaths, malignancies and CVE (LTE).

Study	Trial	Treatment group	N patients	Any serious AE n (%), IR	Any major CVE n (%), IR	Any malignancy n (%), IR	Deaths of any cause n (%), IR
Fleischmann 2020 (146)	MOBILITY<br>TARGET<br>ASCERTAIN<br>ONE<br>COMPARE<br>EASY<br>EXTEND	SAR 150/200/100 Q2W<br>or<br>SAR 100/150 QW + DMARD	2887	685 (23.7)<br>IR/100 PY (nE): 9.4 (685)	41 (1.4)<br>IR/100 PY (nE): 0.5 (45)	52 (1.8)<br>IR/100 PY (nE): 0.7 (56)	31 (1.1)<br>IR: 0.4 (31)
		SAR Mono	471	52 (11.0)<br>IR/100 PY (nE): 6.7 (52)	2 (0.4)<br>IR/100 PY (nE): 0.2 (2)	4 (0.8)<br>IR/100 PY (nE): 0.6 (5)	5 (1.1)<br>IR: 0.6 (5)

Table S4.6.10.5: Sarilumab: Rates of serious infections, opportunistic infections, serious demyelinating disorders and VTE (LTE).

Study	Trial	Treatment group	N patients	Serious infections n (%), IR	OI n (%), IR	demyelinating disorders n (%), IR	VTE n (%), IR
Fleischmann 2020 (146)	MOBILITY TARGET ASCERTAIN ONE COMPARE EASY EXTEND	SAR 150/200/100 Q2W or SAR 100/150 QW + DMARD	2887	232 (8.0) IR/100 PY (nE): 3.7 (301)	72 (2.5) IR/100 PY (nE): 0.9 (76)	0	46 (1.6) IR/100 PY (nE): 0.8 (67)
		SAR Mono	471	7 (1.5) IR/100 PY (nE): 1.0 (8)	6 (1.3) IR/100 PY (nE): 0.7 (6)	1 (0.2) IR/100 PY (nE): 0.1 (1)	3 (0.6) IR/100 PY (nE): 0.4 (3)

Table S4.6.10.6: Sarilumab: Rates of GIP, injection-site / infusion reactions, immunogenicity, neutropenia and hepatic disorders (LTE).

Study	Trial	Treatment group	N patients	GIP n (%), IR	Inj/Inf reaction n (%), IR	ADA n (%), IR	Any neutropenia n (%), IR	Hepatic disorders n (%), IR
Fleischmann 2020 (146)	MOBILITY TARGET ASCERTAIN ONE COMPARE EASY EXTEND	SAR 150/200/100 Q2W or SAR 100/150 QW + DMARD	2887	9 (0.3) IR/100 PY (nE): 0.1 (9)	333 (11.5) IR: 23.6 (1934)	NR	536 (18.6) IR: 13.8 (1132)	448 (15.5) IR: 8.9 (726)
		SAR Mono	471	0	4 (8.2)	NR	85 (18.0) IR: 27.7 (225)	39 (8.3) IR: 7.1 (58)

Kastrati K, et al. RMD Open 2022; 8:e002359. doi: 10.1136/rmdopen-2022-002359

Table S4.6.10.7: Sirukumab: Overview of RCTs.

Study	Trial	Treatment	Risk of bias assessment
Aletaha 2017 (14)	SIRROUND-T	PBO	Low
		SIR 50 Q4W	
		SIR 100 Q2W	
Takeuchi 2017 (11)	SIRROUND-D	PBO	Unclear
		SIR 50 Q4W	
		SIR 100 Q2W	
		SIR combined	
Takeuchi 2018 (147)	no name available	SIR 50 Q4W	Low
		SIR 100 Q2W	
		SIR combined	
Taylor 2018 (21)	SIRROUND-T	ADA 40 Q2W	Low
		SIR 50 Q4W	
		SIR 100 Q2W	

Table S4.6.10.8: Sirukumab: Rates of serious AEs, serious infections, opportunistic infections, deaths, malignancies and CVE (RCTs).

Study	Trial	Treatment group	N patients	Any serious AE n (%)	Serious infections n (%)	OI n (%)	Any major CVE n (%)	Any malignancy n (%)	Deaths of any cause n (%)
Aletaha 2017 (14)	SIRROUND-T	PBO	294	15 (5)	2 (<1)	0	2 (<1)	1 (<1)	0
		SIR 50 Q4W	292	28 (10)	13 (4)	0	1 (<1)	4 (1)	0
		SIR 100 Q2W	292	22 (8)	8 (3)	0	0	1 (<1)	0
Takeuchi 2017 (11)	SIRROUND-D	PBO	556	38 (6.8)	10 (1.8)	0	2 (0.4)	2 (0.4)	1 (0.2)
		SIR 50 Q4W	663	73 (11.0)	27 (4.1)	0	8 (1.2)	2 (0.3)	7 (1.1)
		SIR 100 Q2W	662	65 (9.8)	22 (3.3)	0	3 (0.5)	5 (0.8)	3 (0.5)
		SIR combined	1325	138 (10.4)	49 (3.7)	0	11 (0.8)	7 (0.5)	10 (0.8)
Takeuchi 2018 (147)	no name available	SIR 50 Q4W	61	4 (6.6)	1 (1.6)	0	0	0	0
		SIR 100 Q2W	61	5 (8.2)	2 (3.3)	0	0	1 (1.6)	0
		SIR combined	122	9 (7.4)	3 (2.5)	0	0	1 (0.8)	0
Taylor 2018 (21)	SIRROUND-T	ADA 40 Q2W	186	16 (8.6)	4 (2.2)	0	0	1 (0.5)	0
		SIR 50 Q4W	186	29 (15.6)	14 (7.5)	1	1 (0.5)	3 (1.6)	3 (1.6)
		SIR 100 Q2W	187	22 (11.8)	5 (2.7)	0	2 (1.1)	2 (1.1)	1 (0.5)

Table S4.6.10.9: Sirukumab: Rates of GIP, injection-site / infusion reactions, immunogenicity, neutropenia and hepatic disorders (RCTs).

Study	Trial	Treatment group	N patients	GIP n (%)	Inj/Inf reaction n (%)	Antidrug antibody n (%)	Any neutropenia n (%)
Aletaha 2017 (14)	SIRROUND-T	PBO	294	0	9 (3)	NR	9 (3)
		SIR 50 Q4W	292	2(<1)	29 (10)	NR	94 (32)
		SIR 100 Q2W	292	3 (1)	68 (23)	NR	103 (35)
Takeuchi 2017 (11)	SIRROUND-D	PBO	556	1 (0.2)	14 (2.5)	NR	5 (0.9)
		SIR 50 Q4W	663	1 (0.2)	71 (10.7)	NR	38 (5.7)
		SIR 100 Q2W	662	0	108 (16.3)	NR	29 (4.4)
		SIR combined	1325	1 (0.2)	179 (13.5)	NR	67 (5.1)
Takeuchi 2018 (147)	no name available	SIR 50 Q4W	61	0	26 (42.6)	NR	7 (11.5)
		SIR 100 Q2W	61	0	27 (44.3)	NR	4 (6.6)
		SIR combined	122	0	53 (43.4)	NR	11 (9.0)
Taylor 2018 (21)	SIRROUND-T	ADA 40 Q2W	186	0	16 (8.6)	171 (91.9)	4 (2.2)
		SIR 50 Q4W	186	1 (0.5)	20 (10.8)	7 (3.8)	17 (9.1)
		SIR 100 Q2W	187	1 (0.5)	43 (23.0)	9 (4.9)	11 (5.9)

Table S4.6.10.10: Sirukumab: Rates of serious AEs, serious infections, opportunistic infections, deaths, malignancies and CVE (LTE).

Study	Trial	Treatment group	N patients	Any serious AE n (%)	Serious infections n (%)	OI n (%)	Any major CVE n (%)	Any malignancy n (%)	Deaths of any cause n (%)
Thorne 2018 (148)	SIRROUND-D (2 years)	PBO	556	40 (7.2)	11 (2.0)	0	1 (0.2)	2 (0.4)	1 (0.2)
		SIR 50 Q4W	798	141 (17.7)	58 (7.3)	1 (0.1)	13 (1.6)	8 (1.0)	10 (1.3)
		SIR 100 Q2W	799	132 (16.5)	47 (5.9)	4 (0.5)	5 (0.6)	12 (1.5)	11 (1.4)
		SIR combined	1597	273 (17.1)	105 (6.6)	5 (0.3)	18 (1.1)	20 (1.3)	21 (1.3)

Table S4.6.10.11: Sirukumab: Rates of GIP, injection-site / infusion reactions, immunogenicity, neutropenia and hepatic disorders (LTE).

Study	Trial	Treatment group	N patients	GIP n (%)	Inj/Inf reaction n (%)	ADA n (%)	Any neutropenia n (%)
Thorne 2018 (148)	SIRROUND-D (2 years)	PBO	556	1 (0.2)	14 (2.5)	0	5 (0.9)
		SIR 50 Q4W	798	3 (0.4)	84 (10.5)	14 (1.7)	52 (6.5)
		SIR 100 Q2W	799	1 (0.1)	135 (16.9)	6 (0.7)	45 (5.6)
		SIR combined	1597	4 (0.3)	219 (13.7)	20 (2.4)	97 (6.1)

4.6.11: Juvenile idiopathic arthritis (JIA): Comparison between IL-6R/L blockers and different bDMARDs (observational studies/randomized controlled trials)

Table S4.6.11.1: Safety outcomes of observational studies investigating IL-6R/L blockers in JIA regarding serious AEs.

pcJIA Study	Registry	Treatment group	N patients	N events	Incidence rate (95% CI)	age/gender aHR	RR	Risk of bias assessment
Horneff 2016 (149)	BIKER	TCZ	74	3	4.1/100 PY (1.3;12.8)	NR	ETN vs ADA: 2.06 (1.35;3.16)<br><br>ADA vs TCZ: ns<br><br>ETN vs TCZ: 5.48 (1.74;17.25)	High
		ADA	236	26	11.0/100 PY (7.5;16.2)	NR		
		ETN	419	119	22.07/100 PY (19.0;27.2)	NR		
Grönlund 2020 (150)	JIA database, Finland	TCZ	56	11	12.9/100 PY	No control	No control	High
**sJIA Study**	**Registry**	**Treatment group**	**N patients**	**N events**	**Incidence rate (95% CI)**	**age/gender aHR**	**RR***	**Risk of bias assessment**
Klein 2020 (151)	BIKER	TCZ	109	51	20.91/100 PY (15.56;27.48)	NR	1.33 (1.06;1.66)	High
		ETA	151	14	3.53/100 PY (1.93;5.92)	NR	0.47 (0.33;0.68)	
		ANR	71	8	6.61/100 PY (2.85;13.03)	NR	0.69 (0.46;1.09)	
		CAM	51	19	20.26/100 PY (12.17;31.56)	NR	1.41 (1.09;1.83)	

Kastrati K, et al. RMD Open 2022; 8:e002359. doi: 10.1136/rmdopen-2022-002359

CAM: canakinumab
* relative risk for an adverse event for each biologic in study by Klein et al. 2020 (BIKER) was estimated in comparison with the other three bDMARDs combined (applies to all further risk information in the tables below).

Table S4.6.11.2: Safety outcomes of observational studies investigating IL-6R/L blockers in JIA regarding serious infections.

pcJIA Study	Registry	Treatment group	N patients	N events	Incidence rate (95% CI)	age/gender aHR	RR	Risk of bias assessment
Horneff 2016 (149)	BIKER	TCZ	74	3	4.14/100 PY (1.31;12.57)	NR	ADA vs TCZ: ns<br>ETN vs TCZ: ns<br>ETN vs ADA: 1.73 (0.94;3.19)	High
		ADA	236	13	5.5/100 PY (3.19;9.47)	NR		
		ETN	419	50	9.54/100 PY (7.23;12.59)	NR		
Grönlund 2020 (150)	JIA database, Finland	TCZ	56	3	NR	No control	No control	High
**sJIA Study**	**Registry**	**Treatment group**	**N patients**	**N events**	**Incidence rate (95% CI)**	**age/gender aHR**	**RR**	**Risk of bias assessment**
Klein 2020 (151)	BIKER	TCZ	109	13	5.33/100 PY (2.84;9.11)	NR	1.31 (0.49;3.48)	High
		ETA	151	4	1.01/100 PY (0.27;2.58)	NR	0.23 (0.05;1.03)	
		ANR	71	6	4.96/100 PY (1.82;10.79)	NR	2.82 (1.05;7.60)	
		CAM	51	3	3.2/100 PY (0.66;9.33)	NR	0.54 (0.14;2.01)	

Table S4.6.11.3: Safety outcomes of observational studies investigating IL-6R/L blockers in JIA regarding malignancies.

pcJIA Study	Registry	Treatment group	N patients	N events	Incidence rate (95% CI)	age/gender aHR	RR	Risk of bias assessment
Horneff 2016 (149)	BIKER	TCZ	74	0	NAP	NR	ETN vs ADA: ns<br>ADA vs TCZ: ns<br>ETN vs TCZ: ns	High
		ADA	236	0	NAP	NR		
		ETN	419	1	0.19/100 PY (0.03;1.35)	NR		
Grönlund 2020 (150)	JIA database, Finland	TCZ	56	0	NAP	No control	No control	High
**sJIA Study**	**Registry**	**Treatment group**	**N patients**	**N events**	**Incidence rate (95% CI)**	**age/gender aHR**	**RR**	**Risk of bias assessment**
Klein 2020 (151)	BIKER	TCZ	109	1	0.41/100 PY (0.01;2.28)	NR	5.85 (0.09;381.76)	High
		ETA	151	1	0.25/100 PY (0.01;1.40)	NR	1.84 (0.01;7763.91)	
		ANR	71	0	NAP	NR	NAP	
		CAM	51	0	NAP	NR	NAP	

Kastrati K, et al. RMD Open 2022; 8:e002359. doi: 10.1136/rmdopen-2022-002359

Table S4.6.11.4: Safety outcomes of observational studies investigating IL-6R/L blockers in JIA regarding GIP.

sJIA Study	Registry	Treatment group	N patients	N events	Incidence rate (95% CI)	age/gender aHR	RR	Risk of bias assessment
Klein 2020 (151)	BIKER	TCZ	109	0	0	NAP	NAP	High
		ETA	151	0	0	NAP	NAP	
		ANR	71	0	0	NAP	NAP	
		CAM	51	0	0	NAP	NAP	

Table S4.6.11.5: Safety outcomes of observational studies investigating IL-6R/L blockers in JIA regarding hepatic events.

pcJIA Study	Registry	Treatment group	N patients	N events	Incidence rate (95% CI)	age/gender aHR	RR	Risk of bias assessment
Horneff 2016 (149)	BIKER	TCZ	74	3	4.14/100 PY (1.31;12.57)	NR	ETA vs ADA: ns<br>ADA vs TCZ: ns<br>ETA vs TCZ: ns	High
		ADA	236	6	2.54/100 PY (1.14;5.65)	NR		
		ETN	419	10	1.91/100 PY (1.03;3.55)	NR		
Grönlund 2020 (150)	JIA database, Finland	TCZ	56	11	NR	No control	No control	High

sJIA Study	Registry	Treatment group	N patients	N events	Incidence rate (95% CI)	age/gender aHR	RR	Risk of bias assessment
Klein 2020 (151)	BIKER	TCZ	109	6	2.46/100 PY (0.90;5.35)	NR	2.12 (0.37;12.07)	High
		ETA	151	1	0.25/100 PY (0.01;1.40)	NR	0.14 (0.01;3.19)	
		ANR	71	0	NAP	NR	NAP	
		CAM	51	2	2.13/100 PY (0.26;7.69)	NR	1.65 (0.26;10.51)	

Table S4.6.11.6: Safety outcomes of observational studies investigating IL-6R/L blockers in JIA regarding demyelination.

pcJIA Study	Registry	Treatment group	N patients	N events	Incidence rate (95% CI)	age/gender aHR	RR	Risk of bias assessment
Horneff 2016 (149)	BIKER	TCZ	74	0	NAP	NR	ETA vs ADA: ns<br>ADA vs TCZ: ns<br>ETA vs TCZ: ns	High
		ADA	236	0	NAP	NR		
		ETN	419	1	0.19/100 PY (0.03;1.35)	NR		
Grönlund 2020 (150)	JIA database, Finland	TCZ	56	0	NAP	No control	No control	High

sJIA Study	Registry	Treatment group	N patients	N events	Incidence rate (95% CI)	age/gender aHR	RR	Risk of bias assessment
Klein 2020 (151)	BIKER	TCZ	109	0	NAP	NR	NAP	High
		ETA	151	1	0.25/100 PY (0.01;1.40)	NR	0.14 (0.01;3.19)	

Kastrati K, et al. RMD Open 2022; 8:e002359. doi: 10.1136/rmdopen-2022-002359

		ANR	71	0	NAP	NR	NAP	
		CAM	51	0	NAP	NR	NAP	

Table S4.6.11.7: Safety outcomes of observational studies investigating IL-6R/L blockers in JIA regarding tuberculosis.

pcJIA Study	Registry	Treatment group	N patients	N events	Incidence rate (95% CI)	age/gender aHR	RR	Risk of bias assessment
Horneff 2016 (149)	BIKER	TCZ	74	0	NAP	NR	ETA vs ADA: ns<br>ADA vs TCZ: ns<br>ETA vs TCZ: ns	High
		ADA	236	0	NAP	NR		
		ETN	419	0	NAP	NR		
Grönlund 2020 (150)	JIA database, Finland	TCZ	56	0	NAP	No control	No control	High
**sJIA Study**	**Registry**	**Treatment group**	**N patients**	**N events**	**Incidence rate (95% CI)**	**age/gender aHR**	**RR**	**Risk of bias assessment**
Klein 2020 (151)	BIKER	TCZ	109	0	NAP	NR	NAP	High
		ETA	151	0	NAP	NR	NAP	
		ANR	71	0	NAP	NR	NAP	
		CAM	51	0	NAP	NR	NAP	

Table S4.6.11.8: Safety outcomes of observational studies investigating IL-6R/L blockers in JIA regarding withdrawals.

pcJIA Study	Registry	Treatment group	N patients	N (%) events	Incidence rate (95% CI)	age/gender aHR	RR; p value	Risk of bias assessment
Horneff 2016 (149)	BIKER	TCZ	74	2 (2.7)	NR	NR	ADA vs ETN: 2.28 (1.03; 5.04); 0.042<br>TCZ vs ADA: 0.37 (0.08; 1.79); 0.216<br>TCZ vs ETN: 0.84 (0.18; 4.01); 0.826	High
		ADA	236	15 (3.6)	NR	NR		
		ETN	419	15 (6.4)	NR	NR		
Grönlund 2020 (150)	JIA database, Finland	TCZ	56	2 (12.5)	NAP	No control	No control	High

Table S4.6.11.9: Safety outcomes of observational studies investigating IL-6R/L blockers in JIA regarding macrophage activation syndrome (MAS).

sJIA Study	Registry	Treatment group	N patients	N events	Incidence rate (95% CI)	age/gender aHR	RR	Risk of bias assessment
Klein 2020 (151)	BIKER	TCZ	109	6	2.46/100 PY (0.90;5.35)	NR	1.91 (0.49;7.46)	High
		ETA	151	2	0.5/100 PY (0.06;1.82)	NR	0.32 (0.04;2.91)	
		ANR	71	1	0.83/100 PY (0.02;4.60)	NR	0.62 (0.08;4.93)	

		CAM	51	3	3.2/100 PY (0.66;9.33)	NR	1.07 (0.24;4.87)	

Section 5: Characteristics of articles and abstracts included: Biomarkers for prediction of therapeutic response of interleukin-6 pathway inhibition.

Table S5.1: Overview of included studies.

Biomarker	Study	Agent
**CRP**	Shafran 2020 (152)	Tocilizumab (anti-IL-6R)
**IL-6**	Shimamoto 2013 (153)	Tocilizumab (anti-IL-6R)
	Nishimoto 2014 (DREAM) (154)	Tocilizumab (anti-IL-6R)
	Strand 2020 (155)	Sarilumab (anti-IL-6R) vs ADA (TNF-i)
	Boyapati 2020 (156)	Sarilumab (anti-IL-6R) vs ADA (TNF-i)
**anti-CCP status**	Cappelli 2017 (157)	Tocilizumab (anti-IL-6R)
**Genetic**	Sanayama 2014 (158)	Tocilizumab (anti-IL-6R)
	Maldonado-Montoro 2016 (159)	Tocilizumab (anti-IL-6R)
	Jiménez Morales 2019 (160)	Tocilizumab (anti-IL-6R) vs. RTX (anti-CD20)
**Cellular**	Daien 2015 (161)	Tocilizumab (anti-IL-6R) vs. TNF-i
	Humby 2020 (ACR Abstract) (162)	Tocilizumab (anti-IL-6R) vs. RTX (anti-CD20)
	Dulic 2017 (163)	Tocilizumab (anti-IL6R) vs. TNF-i
**others**	Gabay 2018 (164)	Sarilumab (anti-IL-6R)
	Gabay 2020 (165)	Sarilumab (anti-IL-6R) vs ADA (TNF-i)
	Toussirot 2020 (166)	Tocilizumab (anti-IL-6R)

	Fioravanti 2019 (167)	Tocilizumab (anti-IL-6R)
	Gerasimova 2020 (EULAR Abstract) (168)	Tocilizumab (anti-IL-6R) and TOFA (JAK-i)
**Biometric (Body Mass Index)**	Gardette 2016 (169)	Tocilizumab (anti-IL-6R)
	Schaefer 2020 (170)	Tocilizumab (anti-IL-6R) vs csDMARDs vs RTX vs ABA (CD-80/CD-86)
	Davies 2020 (EULAR Abstract) (171)	Tocilizumab (anti-IL-6R)

Table S5.2: Outcomes of studies investigating biomarkers for prediction of therapeutic response of interleukin-6 pathway inhibition.

Study	Treatment group	N patients	Biomarker	Outcome measures	Results	Conclusion
Shafran 2020 (152)	TCZ pooled (8mg/kg)	1126	CRP	Comparing CDAI values and change along 24 weeks follow-up to CRP values at BL or its early change	CDAI remission at wk 24 on TCZ associated with highest CRP at BL<br>Pat with highest DA had lowest CRP at BL<br>Pat with CDAI Rem at wk 24 had largest reductions of CRP by wk 4<br>Early CRP non-response indicative for achieving clinical treatment goals (p=0.038)	baseline CRP positive predictor of response for TCZ (negative RTX/MTX)<br>CRP reduction of <20% from BL by 4 wks during TCZ : poor prognostic marker
	RTX	250				
	MTX	249				
Shimamoto 2013 (153)	TCZ	32	s IL-6	DAS 28 at BL and 4 wks after treatment	Pre-treatment IL-6 levels significant lower in TCZ responsive pts (DAS28<3.2) than TCZ-non responders	low serum IL-6 is associated with a favorable response to TCZ
	IFX	29	TNF-α			
	healthy controls	13				

Nishimoto 2014 (DREAM) (154)	Cessation of TCZ (monotherapy)	187	s IL-6<br>MMP-3	DAS-28 for 52 wks	Pat with low serum IL-6 (<12.9 pg/mL) and normal MMP-3 levels, the rate of continued LDA reached 38.0% at 52 weeks.	low serum IL-6 associated with favorable progression after TCZ cessation
Strand 2020 (155)<br>MONARCH- post hoc	SAR	148	s IL-6	HRQoL BL, wk 24 and wk 52<br>• Short Form 36 (SF-36)<br>• (FACIT)-fatigue<br>• AM stiffness VAS	high baseline IL-6 levels reported better improvements in HRQoL (PCS, physical functioning domain, and AM-stiffness VAS) with SAR versus ADA	high levels of IL-6 at BL are associated with greater improvements in health-related quality of life
	ADA	152				
Boyapati 2020 (156)	SAR 200 Q2W	184	s IL-6	Efficacy and patient-reported outcomes were compared between and within groups according to IL-6 tertile using linear and logistic regression	Pat. with high BL IL-6 levels (all >=3 times the upper limit of normal; n = 100): higher disease activity at BL vs pat. with low IL-6 levels (n = 100).<br>clinical improvement over 24 weeks with SAR versus ADA greater in pat. with high compared to low BL IL-6 levels.<br>MOBILITY: patients with low IL-6 levels (n = 397) vs. pat with high IL-6 levels (n = 398) higher disease activity and joint damage at BL, were more likely to have joint progression, and had less clinical improvement over 52 weeks' treatment with PBO plus MTX compared to SAR 150 mg or 200 mg plus MTX.	IL-6 may be prognostic marker of disease progression and severity<br>Pat. with high IL-6 levels likely to benefit from SAR compared to ADA or MTX
	ADA 40 Q2W	185				
	SAR 150 Q2W	400				
	SAR 200 Q2W	399				
	PBO	398				

					BL IL-6 and C-reactive protein levels predictors of outcomes	
Cappelli 2017 (157)	TCZ	316	anti CCP status (negative/positive)	Visit 1 and 2 (4-8 months)<br>• Disease Activity (CDAI)<br>• mDAS<br>• VAS fatigue, global DA, pain, HAQ	both groups significant improvement<br>magnitude of improvement did not differ significantly by CCP status	anti CCP status did not predict treatment response
Sanayama 2014 (158)	TCZ	Training Cohort n=40<br>Valid. Cohort n=20	PBMC gene expression using DNA microarray	physician's global assessment (good/ moderate/no response) at 6 months	type I interferon response genes (IFI6, MX2, and OASL) and MT1G associated with TCZ-response	type I interferon signaling and metallothioneins are candidate biomarkers to predict TCZ-response
Maldonado-Montoro 2016 (159)	TCZ	79	gene polymorphisms	EULAR response, remission, LDA and DAS28 improvement rates 6/18 months	GALNT18 C-allele or the CD69 A-allele associated with good TCZ response	genetic biomarkers could predict TCZ response
Jiménez Morales 2019 (160)	TCZ	87	gene polymorphisms	EULAR response, remission, LDA and DAS28 improvement rates at 6/12/18 months	FCGR3A rs396991-TT genotype treated with TCZ: higher EULAR response (OR, 5.075; 95%CI, 1.20–21.33; p = .027) at 12 months	genetic biomarkers could predict TCZ response
	RTX	55				
Daien 2015 (161)	TCZ	20	B, T, NK and NK T (NKT) cells at BL, 3 and 6 months	DAS 28	Pts with TCZ significantly increased proportion of Tregs at 3 but not at 6 months.<br>% of NK cells higher at BL for TCZ-treated patients with disease	NK cells at baseline could be a predictive factor of TCZ response
	TNFi (6 ETN, 8 CZP, 1 ADA)	15				
	Controls	25				

					remission than active disease at 3 months	
Humby 2020 (ACR Abstract) (162)	TCZ	81	Synovial tissue at trial entry histologically classified:<br>B-cell rich (BCR) or B-cell poor (BCP)	Week 16:<br>CDAI ≥50% improvement from BL<br>and<br>Major Treatment response (MTR)= CDAI improvement ≥ 50% and CDAI ≤10.1	PEP (TCZ):<br>23 (56.1) BCP vs 16 (51.6) BCR<br>Co-PEP (TCZ):<br>19 (46.3) BCP vs. 11 (35.5) BCR	in RA BCP population failing csDMARDs and TNFi therapy, TCZ is more effective than RTX in achieving significant falls in disease activity
	RTX	83				
Dulic 2017 (163)	TNF-i responders	30	PBMC: helper T-cells, Th1/Th2/Th17 cells, Treg, naïve T cells, memory T-cells; before, 8 weeks and at least 6 months after biological therapy	DAS 28	% of regulatory T-cells (Tregs) becomes normal in all long-term-treated groups;<br>TNF-i responders/non-responders frequencies of naïve CD4+ and CD8+ cells are lower, whereas those of proinflammatory Th1, Th2, and Th17 cells and HLA-DR+-activated cells are higher than those in untreated RA or healthy controls;<br>TCZ responders, Th1 proportion was decreased; Th2 and Th17 is increased vs. TNF-i patients and controls	CD4CD69 ratio < 2.43 at BL, could be predictive for therapeutic response to TNF-i
	TNF-i non responders	19				
	TCZ responders	43				
	Treatment naïve RA	19				
Gabay 2018 (164)<br>TARGET-substudy	SAR 150 q2w	97		CDAI		sICAM-1 was predictive of achieving LDA with SAR
	SAR 200 q2w	97				

	csDMARD	97	Circulating markers for synovial inflammation		SAR significantly decreased C1M, C3M, CXCL13, MMP-3 and total RANKL levels at wk 24 versus PBO<br><br>sICAM-1 predictive by C-reactive protein CDAI low disease activity (LDA) response in the SAR 200mg q2w group at week 12	
Gabay 2020 (165)<br><br>MONARCH post hoc	SAR	153	circulating biomarkers associated with<br><br>acute-phase response<br><br>bone remodelling<br><br>atherothrombosis<br><br>anaemia of chronic disease<br><br>synovial markers	ACR 20<br><br>DAS28	Week 24: SAR vs ADA<br><br>sign. ↓ CRP, SAA, RANKL, Lp(a)<br><br>sign. ↑ procollagen type 1 N-terminal propeptide (P1NP)<br><br>high baseline SAA, CRP (MMP-3) more likely for clinical improvement and PRO SAR vs. ADA	SAR associated with greater positive effects on bone remodelling, synovial inflammation and cardiovascular risk vs. ADA
	ADA	154				
Toussirot 2020 (166)	csDMARD/bDMARD IR→TCZ	107	BMI<br><br>Lipid and metabolic parameters<br><br>Body composition	BL, 1/3/6 months	signif. ↑ in total and HMW adiponectin at the onset of treatment<br><br>significant ↑ in lean mass, while fat mass did not change	↑ adiponectin levels could have positive effects on the CV risk<br><br>TCZ may have anabolic impact on lean mass/skeletal muscle
Fioravanti 2019 (167)	TCZ	44	Lipid and metabolic parameters BL and 6 months	DAS 28<br><br>HAQ	signif. ↑ total cholesterol<br><br>signif. ↑ adiponectin	↑ adiponectin levels could have positive effects on the CV risk

					signif. ↓ chemerin<br>no significant correlations with clinical and biochemical parameters	
Gerasimova 2020 (EULAR Abstract) (168)	TCZ	29	NT-proBNP in pts with no history of CVD and normal TTE	DAS 28	↓ NT-proBNP associated with positive dynamics DAS 28 and inflammatory markers (CRP, ESR)	↑ NT-proBNP considered as a component of disease activity
	TOFA	31				
Gardette 2016 (169)	TCZ	115	BMI BL and after 6 months	↓DAS28 ≥ 1.2<br>EULAR good response<br>DAS28 < 2.6	median BMI did not differ between responders and non-responders for DAS28	BMI did not affect the response to TCZ in RA
Schaefer 2020 (170)<br>RABBIT Registry	TCZ	1173	BMI	DAS28-ESR improvement after 6 months of treatment	Obesity BMI 30 kg/m2 reduced real-world effectiveness of TCZ<br>-0.22 (95% CI: –0.42; –0.03) units for women and –0.41 (95% CI: –0.74; –0.07) units for men receiving TCZ	Obesity has a negative impact on the effectiveness of TCZ
Davies 2020 (EULAR Abstract) (171)	TCZ IV or SC	1241	BMI	DAS28-ESR improvement after 6 months of treatment	no significant effect of BMI in change of DAS28 for pat starting IV or SC TCZ	BMI does not affect initial response to IV or SC TCZ

Kastrati K, et al. RMD Open 2022; 8:e002359. doi: 10.1136/rmdopen-2022-002359

Section 6: Characteristics of articles and abstracts included: Patient adherence/preferences and economic aspects of interleukin-6 pathway inhibition.

Table S6.1: Outcomes of studies investigating patient adherence and preferences in patients treated with IL-6R/L blockers.

RA Study	Registry/trial	Treatment group	N patients	Outcome measures	Results	Conclusion
Forsblad-d'Elia 2015 (172)	ARTIS	TCZ	530	Adherence/drug continuation<br>Predictors for discontinuing	6 month, 1 and 2 year estimated drug continuations were 79%, 64% and 50%<br>Predictors:<br>• low initial CRP: HR 0.76 (0.63;0.91)<br>• high HAQ: HR 1.23 (1.06;1.44)<br>• prior bDMARD HR 1.43 (1.12;1.83)	TCZ discontinuation was predicted by low CRP, high HAQ and exposure to biologics in RA
Pappas 2020 (EULAR Abstract) (173)	CORRONA registry	TCZ	1789<br>N=1303 with reported reason	Adherence/drug continuation<br>Predictors for discontinuing	median (95% CI) duration of persistence: 46 (38 to 55) mths<br>Predictors:<br>• Smoking previous or current: HR 1.32 (1.03;1.75)<br>• use of 1 prior non-TNFi: HR 1.25 (1.03;1.52)	TCZ most frequently initiated after IR to ≥ 2 bDMARDs<br>Smoking, use of 1 prior non-TNFi and higher baseline pain score associated with discontinuation

					• Patient pain: HR 1.07 (1.01;1.22)<br>• IV TCZ: no insurance: HR 2.51 (1.02; 6.18)<br>• high fatigue at BL: HR 1.04 (1.00;1.08)	
Best 2020 (174)	US data: ≥1 bDMARD-IR<br><br>MarketScan, Medicare	TCZ	1630	Days of prim. persistance: adjusted mean (95% CI)	TCZ 333 (311–356)<br>ADA 280 (268–293)<br>CZP 262 (241–284)<br>ETA 289 (274–304)<br>ABA 320 (305–335)<br>GOL 304 (274–333)	among patients with RA with ≥1 bDMARD-IR pat with TCZ exhibited a similar or significantly better bDMARD persistance
		GOL	745			
		ETA	2760			
		CZP	982			
		ADA	3599			
		ABA	2899			
Saraux 2019 (EULAR Abstract) (175)	multicenter, observational	TCZ	291	drug retention rate of TCZ sc at 12 months<br><br>Qol using EQ5D	drug retention rate month 12 63.6%; 62.6% in Mono, 64.3% in csDMARD combination<br><br>EQ-5D improved in all domains with a change from baseline of 0.11 ±0.29	at 12 months, drug retention rate was 63.6% in patients receiving TCZ SC in real life, with no difference between monotherapy and combination with csDMARDs groups<br><br>QoL improved in all EQ-5D domains
Haraoui 2019 (176)	multicenter, observational	TCZ	n= 639 Mono<br><br>n=1273 Combi	drug retention rate after 6 months	1504 patients (78.7%) continued to receive TCZ<br><br>no difference between bDMARD exposed or naïve or combination vs mono	in routine clinical practice, TCZ discontinuation rates were low and unaffected by prior use of bDMARDs;

						PROs in bDMARD naïve were numerically better
Tanaka 2018 (177)	multicenter, observational	TCZ SC	377	change in % overall work impairment (OWI) among PWs at week 52 assessed using the Work Productivity and Activity Impairment Questionnaire (WPAI)	OWI at week 52:<br>• −18.9% (TCZ-SC group) and −19.0% (csDMARDs group) (ns)<br>• WPAI activity impairment in the overall group and HWs for TCZ significant bette than csDMARD<br>• TCZ-SC-treated HWs sign. Improvement in QoL<br>• no difference in PW regarding QoL	despite lack of differences in OWI between groups at week 52, overall group (particularly HWs) receiving TCZ-SC in addition to csDMARDs showed significant improvements in activity impairment, disease activity, and QOL vs csDMARD alone
		csDMARD	347			
Strand 2017 (178)	ADACTA	TCZ vs. ADA	265/259	PtGA<br>(FACIT)-Fatigue<br>SF-36<br>% of pat with improvements from baseline ≥minimum clinically important differences (MCID) for each PRO<br>≥age-matched and gender-matched normative values	ADACTA<br>• TCZ sign greater improvements in PtGA, pain, SF-36,<br>• more TCZ-treated patients reported improvements ≥MCID, and reported scores ≥normative values across all PROs vs ADA.<br>AMBITION<br>• TCZ significant improvement in HAQ, fatigue, SF-36	TCZ monotherapy resulted in more patients reporting clinically meaningful PRO improvements and PRO scores ≥normative values compared with MTX or ADA monotherapy
	AMBITION	TCZ vs. MTX	163/162			

					• ≥MCID and reported scores ≥normative values across all PROs vs MTX	
Strand 2018a (179)	MONARCH	SAR	184	PtGA<br>VAS pain<br>Chronic Illness Therapy-Fatigue (FACIT-F)<br>SF-36<br>HAQ<br>Rheumatoid Arthritis Impact of Disease (RAID)<br>Work Productivity Survey (WPS-RA)	at week 24:<br>SAR: sign. Improvement vs ADA in<br>• HAQ, PtGA, pain VAS, MS VAS, SF-36 PCS, WPS-RA	SAR monotherapy resulted in greater improvements across multiple PROs than ADA mono
		ADA	185			
Strand 2018b (180)	OPTION<br>BREVACTA<br>SUMMACTA	OPTION: TCZ IV	205	PtGA<br>VAS pain<br>Chronic Illness Therapy-Fatigue (FACIT-F)<br>SF-36<br>HAQ<br>% of pat with improvements from baseline ≥minimum clinically important	Pat. with TCZ-IV reported improvements in PROs >=MCID (50%-82% vs 31%-57%) and scores >= normative values (16%-44% vs 5%-28%) at week 16 vs PBO.<br><br>greater % of pat. in BREVACTA with TCZ-SC reported improvements >= MCID (54%-73% vs 42%-55%) and scores >= normative values (8%-34% vs 4%-25%) at week 12 vs. PBO;<br><br>SUMMACTA: 61%-84% of pat. with TCZ-SC and 64%-84% of pat.	TCZ-IV or TCZ-SC with csDMARDs: more pat. reported clinically meaningful improvements and PRO scores >= normative values compared with PBO; improvements similar with TCZ-IV and TCZ-SC
		OPTION: PBO	204			
		BREVACTA: TCZ SC	437			
		BREVACTA: PBO	219			
		SUMMACTA: TCZ SC	558			
		SUMMACTA: TCZ IV	537			

				differences (MCID) for each PRO<br>≥age-matched and gender-matched normative values	treated with TCZ-IV reported improvements >= MCID and 14%-41% and 15%-24%, respectively, scores >= normative values at week 24	
**GCA Study**	**Registry/trial**	**Treatment group**	**N patients**	**Outcome measures**	**Results**	**Conclusion**
Strand 2019 (181)	GiACTA	TCZ-QW + Pred-26	100	SF-36 PCS and MCS and all eight individual domains<br>PtGA<br>FACIT-Fatigue	TCZ-QW + Pred-26: signif. greater improvement in 4/8 SF-36 domains vs PBO + Pred-26 and 6/8 domains compared with PBO + Pred-52 (p < 0.01). I<br>improvement with TCZ-QW + Pred-26 met or exceeded minimum clinically important differences (MCID) in all 8 domains compared with 5 domains with PBO + Pred-26 and 0 with PBO + Pred-52<br>Domain scores in TCZ-QW + Pred-26 group at wk 52 met or exceeded age- and gender-matched normative values (A/G norms)<br>LSM changes from BL in FACIT-Fatigue scores increased significantly with TCZ-QW + Pred-	pat. with TCZ-QW + Pred-26 reported statistically significant and clinically meaningful improvement in SF-36 and FACIT-Fatigue scores compared with those receiving prednisone only
		PBO + Pred-26	50			
		PBO + Pred-52	51			

					26, exceeding MCID and A/G norms (p < 0.001)	
**JIA Study**	**Registry/trial**	**Treatment group**	**N patients**	**Outcome measures**	**Results**	**Conclusion**
Ayaz 2020 (182)	Single center	TCZ	9	JADAS71<br><br>satisfaction questionnaire	no deterioration in terms of active joint counts, physician's VAS, patient's VAS and JADAS71.<br><br>satisfaction in life quality, school success and reduced school absenteeism.	TCZ effective treatment option in JIA and switching from IV to SC route when necessary was found to be an effective and acceptable alternative by pat.

Table S6.2: Outcomes of studies investigating economic aspects of treatment with IL-6R/L blockers.

Study	Registry/trial	Treatment group	N patients	Outcome measures	Results	Conclusion
Soini 2012 (183)	Patient profiles (OPTION, TOWARD, LITHE) for a probabilistic microsimulation model<br><br>≥csDMARD-IR	TCZ<br>ADA<br>ETN	3000	Δ costs and QALYs<br><br>ACR 20/50/70	TCZ+MTX more cost-effective than ADA+MTX or ETN+MTX or MTX alone	Tocilizumab + MTX is a potentially cost-effective bDMARD treatment for moderate-to-severe rheumatoid arthritis (msRA)
Johnston 2015 (184)	MarketScan<br><br>Medicare	TCZ	1090	per-patient per-month (PPPM) healthcare costs, including biologic costs, RA-related healthcare costs, and	TCZ had significantly lower (all P<0.05) PPPM biologic costs (ABA = $2,597, IFX = $3,141, TCZ = $1,894)<br>RA-related healthcare costs (ABA	TCZ had the lowest real-world healthcare costs, largely driven by lower costs directly related to bDMARD treatment
		ABA	1759			
		IFX	922			

				all-cause healthcare costs	= $2,929, IFX = $3,598, TCZ = $2,236), and all-cause healthcare costs (ABA = $3,735, IFX = $4,600, TCZ = $3,042)	
Verhoeven 2020 (185)	U-ACT Early	TCZ	total 317	QALYs calculated based on the EQ5D<br>Δ costs and QALYs calculated for TCZ+MTX vs. MTX and TCZ vs. MTX over 2 and 5 year time horizon	QALYs increased between 2 and 5 years, without becoming statistically significant:<br>TCZ+MTX vs. MTX: 0.06 (-0.10; 0.22)<br>TCZ vs. MTX: -0.03 (-0.20; 0.13)<br>probability of TCZ (+MTX) being cost-effective intervention over 5 years, using different WTP thresholds for a QALY, was in general low	early initiation TCZ ± MTX, is not cost-effective vs MTX initiation in a step-up T2T strategy over 2 or 5 years in early RA
		TCZ+MTX				
		MTX				
Best 2020 (186)	ADACTA	TCZ	163	patient-level drug costs<br>cost of hospitalization due to AE<br>cost per response (DAS28, ACR20/50/70)	mean drug and administration costs per each clinical response achieved were lower with TCZ vs ADA	in comparative assessment, the cost to achieve all 4 clinical endpoints was lower for TCZ vs ADA
		ADA	162			
Muszbek 2019 (187)	Microsimulation based on patient profiles from MOBILITY<br>via a 6-month decision tree and lifetime Markov model	SAR 150<br>SAR 200<br>MTX<br>treatment comparators in the model included	total 1197	QALYs were estimated via mapping 6-month ACR20/50/70 response to relative change in HAQ	Lifetime QALYs and costs for treatment sequences on the efficiency frontier were 3.43 and $115,019 for active csDMARD, 5.79 and $430,918 for SAR, and 5.94 and $524,832 for etanercept (all others dominated).	SAR dominated ADA, CZP, GOL and TOFA treatment sequences (i.e., more effective and less costly)

		bDMARDs and the tsDMARD, tofacitinib			SAR vs TCZ and DMARD: \$84,079/QALY and \$134,286/QALY	

Section 7: Figures and tables for colorblind persons

Figure S7.1: Efficacy of biological disease modifying anti-rheumatic drugs targeting the IL-6 receptor or ligand and their relative efficacy and or regulatory approvals

Disease with approval	bDMARD targeting IL-6 receptor or IL-6 ligand									
	anti-IL-6 receptor					anti-IL-6 ligand				
	Tocilizumab	Sarilumab	Levilimab	Satralizumab	Vobarilizumab	Olokizumab	Clazakizumab	Sirukumab	Siltuximab	PF-04236921
Rheumatoid arthritis (RA)	Approved	Approved	Phase 2		Phase 2	Phase 3	Phase 2	Phase 3		Phase 1
Systemic juvenile idiopathic arthritis (sJIA)	Approved	Phase 2[a]								
Polyarticular-course JIA (pcJIA)	Approved	Phase 2[b]								
Adult-onset Still´s disease (AoSD)	Approved (JPN)									
Giant cell arteritis (GCA)	Approved	Phase 3[c]						Phase 3[d]		
Takayasu arteritis (TAK)	Approved (JPN)									
Multicentric Castleman´s disease (MCD)	Approved (JPN)								Approved (US, EU)	
CAR-T cell induced Cytokine Release Syndrome (CRS)	Approved									
Neuromyelitis optica spectrum disorders (NMOSD)	Phase 2/3			Approved						

Approved and solid evidence for efficacy	
Approved but no significant difference compared to placebo	
Not approved but evidence for efficacy	
Clinical trial recruiting participants	
Clinical trial terminated	
Mixed results across trials	
Not evaluated/reported	

Table S7.2: Efficacy outcomes of clinical trials published from 2012 to 2020 investigating biologic disease modifying antirheumatic drugs (bDMARDs) specifically inhibiting IL-6 receptor or ligand compared against placebo or control group, shown across other studied immune-mediated diseases.

Disease	Study	Target	Population	Intervention / Control	Primary endpoint	Efficacy
Psoriatic arthritis	Mease et al. 2016 phase 2b	IL-6	NSAID-IR and/or csDMARD bDMARD naïve	CLZ vs PBO	ACR 20 response at week 16	no difference compared to placebo/control group
Ankylosing spondylitis	Sieper et al. 2014 (BUILDER) phase 2/3	IL-6R	TNFi-naïve	TCZ vs PBO	ASAS 20 response at week 12	
	Sieper et al. 2015 (ALIGN) phase 2		NSAID-IR	SAR vs PBO	ASAS 20 response at week 12	
Osteoarthritis	Richette et al. 2020 (TIDOA) phase 3	IL-6R	refractory to analgetics	TCZ vs PBO	ΔVAS pain at week 6	
Systemic lupus erythematosus	Wallace et al. 2017 (BUTTERFLY) phase 2	IL-6	active disease (SLEDAI-2K/BILAG)	PF-04236921 vs PBO	SLE Responder Index (SRI-4) at week 24	
	Rovin et al. 2016 phase 2		class III or class IV Lupus nephritis	SIR vs PBO	reduction in proteinuria from baseline to week 24	
	NCT02437890 phase 2	IL-6R	moderate to severe active SLE	ALX-0061 vs PBO	mBICLA response rate at week 24	
Myositis	NCT02043548 phase 2	IL-6R	refractory PM/DM	TCZ vs PBO	Mean Total Improvement Scores at visits 2-7	
Sjögren's syndrome	Felten et al. 2020 (ETAP) phase 2/3	IL-6R	ESSDAI ≥ 5	TCZ vs PBO	Response to treatment at week 24*	
Multiple Myeloma	San-Miguel et al. 2014 phase 2	IL-6	untreated MM and no candidate for stem cell transplantation	SIL +VMP vs VMP	Complete response rate**	
	Brighton et al. 2019 phase 2		high-Risk Smoldering multiple Myeloma	SIL vs PBO	1-year progression-free survival rate	
Systemic sclerosis associated ILD	Khanna et al. 2020 (focuSSced) phase 3	IL-6R	diffuse cutaneous-SSc; mRSS 10-35; inflammatory status	TCZ vs PBO	ΔmRSS from baseline to week 48; secondary outcome: ΔFVC% predicted from baseline to week 48	promising results or rather mixed results across groups/trials
Late Antibody-Mediated Kidney Transplant Rejection	Doberer et al. 2020 phase 2	IL-6	kidney transplant recipients with donor-specific, antibody-positive ABMR	CLZ vs PBO	safety and tolerability; secondary outcomes: course of eGFR, protein/creatinine ratio	
AA-Amyloidosis	Okuda et al. 2014/2016 retrospective analyses	IL-6R	Amyloid A (AA) amyloidosis complicating rheumatic diseases	TCZ vs TNFi	Outcomes: retention rate, median ΔSAA, median ΔeGFR, mean ΔCDAI, mean ΔGC dose	
Polymyalgia rheumatica	Lally et al. 2016 open label, phase 2a	IL-6R	PMR treated with GCs for ≤ 4 weeks	TCZ+GC vs GC	relapse-free remission without GC treatment at 6 months	superior versus control
	Devauchelle-Pensec et al. 2016 (TENOR) open label, phase 2		active disease defined as PMR-AS>10	TCZ mono no control group	PMR-AS≤10 at week 12	promising results or rather mixed results across groups/trials
COVID-19 CRS/pneumonia	Hermine et al. 2020 (CORIMUNO-TOCI 1),open-label	IL-6R	moderate to severe pneumonia	TCZ + SOC vs SOC	(1) %patients dead or needing NIV or mechanic ventilation on day 4 (scores >5 on WHO-CPS); and (2) survival without need of ventilation at day 14	
	Salvarani et al. 2020 (RCT-TCZ-COVID-19), open-label		mild pneumonia	TCZ + SOC vs SOC	clinical worsening within 14 days***	
	Stone et al. 2020 (BACC Bay Tocilizumab Trial), phase 3		mild pneumonia	TCZ + SOC vs PBO + SOC	mechanical ventilation or death (time frame: 28 days)	

Section 8 References

1. Huizinga TWJ, Fleischmann RM, Jasson M, Radin AR, Van Adelsberg J, Fiore S, et al. Sarilumab, a fully human monoclonal antibody against IL-6R alphain patients with rheumatoid arthritis and an inadequate response to methotrexate: efficacy and safety results from the randomised SARIL-RA-MOBILITY part a trial. Annals of the rheumatic diseases. 2014;73(9):1626-34.
2. Genovese MC, Fleischmann R, Kivitz AJ, Rell-Bakalarska M, Martincova R, Fiore S, et al. Sarilumab Plus Methotrexate in Patients With Active Rheumatoid Arthritis and Inadequate Response to Methotrexate: Results of a Phase III Study. Arthritis & Rheumatology. 2015;67(6):1424-37.
3. Tanaka Y, Wada K, Takahashi Y, Hagino O, van Hoogstraten H, Graham NMH, et al. Sarilumab plus methotrexate in patients with active rheumatoid arthritis and inadequate response to methotrexate: results of a randomized, placebo-controlled phase III trial in Japan. Arthritis Research & Therapy. 2019;21(1):79.
4. Mazurov V, Zotkin E, Ilivanova E, Kropotina T, Plaksina T, Nesmeyanova O, et al. Efficacy of levilimab, novel monoclonal anti-IL-6 receptor antibody, in combination with methotrexate in patients with rheumatoid arthritis: 1-year results of phase 2 aurora study. Annals of the Rheumatic Diseases Conference: annual european congress of rheumatology of the european league against rheumatism, EULAR 2020, Germany. 2020;79(SUPPL 1):637-8.
5. Ablynx. A Dose-Range Finding Study for ALX-0061 Combination Therapy in Subjects With Rheumatoid Arthritis. Available: https://clinicaltrialsgov/ct2/show/NCT02309359 [Accessed 09 Nov 2020].
6. Ablynx A Phase IIb Study for ALX-0061 Monotherapy in Subjects With Rheumatoid Arthritis Available: https://clinicaltrialsgov/ct2/show/study/NCT02287922 [Accessed 09 Nov 2021].
7. Nasonov E, Stoilov R, Tyabut T, Genovese MC. Olokizumab, monoclonal antibody against il6, in patients with moderately to severely active rheumatoid arthritis inadequately controlled by methotrexate: Efficacy and safety results of phase III credo-1 study. Annals of the Rheumatic Diseases Conference: annual european congress of rheumatology of the european league against rheumatism, EULAR 2020, Germany. 2020;79(SUPPL 1):16-7.
8. Mease PJ, Strand V, Shalamberidze L, Dimic A, Raskina T, Xu LA, et al. A phase II, double-blind, randomised, placebo-controlled study of BMS945429 (ALD518) in patients with rheumatoid arthritis with an inadequate response to methotrexate. Annals of the Rheumatic Diseases. 2012;71(7):1183-9.
9. Baek HJ, Lim MJ, Park W, Park SH, Shim SC, Yoo DH, et al. Efficacy and safety of tocilizumab in Korean patients with active rheumatoid arthritis. Korean Journal of Internal Medicine. 2019;34(4):917-31.
10. Hoffmann-La Roche. A Study of Tocilizumab in Combination With DMARD Therapy in Patients With Active Rheumatoid Arthritis. Available: https://clinicaltrialsgov/ct2/show/NCT00773461 [Accessed 10 Nov 2020].
11. Takeuchi T, Thorne C, Karpouzas G, Sheng S, Xu W, Rao R, et al. Sirukumab for rheumatoid arthritis: the phase III SIRROUND-D study. Annals of the Rheumatic Diseases. 2017;76(12):2001-8.
12. Fleischmann R, van Adelsberg J, Lin Y, Castelar-Pinheiro GDR, Brzezicki J, Hrycaj P, et al. Sarilumab and Nonbiologic Disease-Modifying Antirheumatic Drugs in Patients With Active Rheumatoid Arthritis and Inadequate Response or Intolerance to Tumor Necrosis Factor Inhibitors. Arthritis and Rheumatology. 2017;69(2):277-90.
13. Takeuchi T, Tanaka Y, Yamanaka H, Amano K, Nagamine R, Park W, et al. Efficacy and safety of olokizumab in Asian patients with moderate-to-severe rheumatoid arthritis, previously exposed to anti-TNF therapy: Results from a randomized phase II trial. Modern Rheumatology. 2016;26(1):15-23.
14. Aletaha D, Bingham CO, 3rd, Tanaka Y, Agarwal P, Kurrasch R, Tak PP, et al. Efficacy and safety of sirukumab in patients with active rheumatoid arthritis refractory to anti-TNF therapy (SIRROUND-T): a randomised, double-blind, placebo-controlled, parallel-group, multinational, phase 3 study. Lancet. 2017;389(10075):1206-17.

15. Genovese MC, Fleischmann R, Furst D, Janssen N, Carter J, Dasgupta B, et al. Efficacy and safety of olokizumab in patients with rheumatoid arthritis with an inadequate response to TNF inhibitor therapy: outcomes of a randomised Phase IIb study. Annals of the Rheumatic Diseases. 2014;73(9):1607-15.
16. Yazici Y, Curtis JR, Ince A, Baraf H, Malamet RL, Teng LL, et al. Efficacy of tocilizumab in patients with moderate to severe active rheumatoid arthritis and a previous inadequate response to disease-modifying antirheumatic drugs: the ROSE study. Annals of the Rheumatic Diseases. 2012;71(2):198-205.
17. Kivitz A, Olech E, Borofsky M, Zazueta BM, Navarro-Sarabia F, Radominski SC, et al. Subcutaneous tocilizumab versus placebo in combination with disease-modifying antirheumatic drugs in patients with rheumatoid arthritis. Arthritis care & research. 2014;66(11):1653-61.
18. Roche H-L. TORPEDO Study: A Study on Rapid Effect of Tocilizumab in Patients With Rheumatoid Arthritis With an Inadequate Response to Disease-Modifying Antirheumatic Drugs (DMARDs) or Anti-TNF. Available: https://clinicaltrials.gov/ct2/show/NCT00977106 [Accessed 10 Nov 2020].
19. Gabay C, Emery P, van Vollenhoven R, Dikranian A, Alten R, Pavelka K, et al. Tocilizumab monotherapy versus adalimumab monotherapy for treatment of rheumatoid arthritis (ADACTA): a randomised, double-blind, controlled phase 4 trial. Lancet. 2013;381(9877):1541-50.
20. Burmester GR, Lin Y, Patel R, Van Adelsberg J, Mangan EK, Graham NMH, et al. Efficacy and safety of sarilumab monotherapy versus adalimumab monotherapy for the treatment of patients with active rheumatoid arthritis (MONARCH): A randomised, double-blind, parallel-group phase III trial. Annals of the Rheumatic Diseases. 2017;76(5):840-7.
21. Taylor PC, Schiff MH, Wang Q, Jiang Y, Zhuang Y, Kurrasch R, et al. Efficacy and safety of monotherapy with sirukumab compared with adalimumab monotherapy in biologic-naïve patients with active rheumatoid arthritis (SIRROUND-H): A randomised, double-blind, parallel-group, multinational, 52-week, phase 3 study. Annals of the Rheumatic Diseases. 2018;77(5):658-66.
22. Weinblatt ME, Mease P, Mysler E, Takeuchi T, Drescher E, Berman A, et al. The efficacy and safety of subcutaneous clazakizumab in patients with moderate-to-severe rheumatoid arthritis and an inadequate response to methotrexate: results from a multinational, phase IIb, randomized, double-blind, placebo/active-controlled, dose-ranging study. Arthritis & Rheumatology. 2015;67(10):2591-600.
23. Burmester GR, Rubbert-Roth A, Cantagrel A, Hall S, Leszczynski P, Feldman D, et al. A randomised, double-blind, parallel-group study of the safety and efficacy of subcutaneous tocilizumab versus intravenous tocilizumab in combination with traditional disease-modifying antirheumatic drugs in patients with moderate to severe rheumatoid arthritis (SUMMACTA study). Annals of the Rheumatic Diseases. 2014;73(1):69-74.
24. Ogata A, Tanimura K, Sugimoto T, Inoue H, Urata Y, Matsubara T, et al. Phase III study of the efficacy and safety of subcutaneous versus intravenous tocilizumab monotherapy in patients with rheumatoid arthritis. Arthritis care & research. 2014;66(3):344-54.
25. Ogata A, Atsumi T, Fukuda T, Hirabayashi Y, Inaba M, Ishiguro N, et al. Sustainable Efficacy of Switching From Intravenous to Subcutaneous Tocilizumab Monotherapy in Patients With Rheumatoid Arthritis. Arthritis care & research. 2015;67(10):1354-62.
26. Ogata A, Tanaka Y, Ishii T, Kaneko M, Miwa H, Ohsawa S, et al. A randomized, double-blind, parallel-group, phase III study of shortening the dosing interval of subcutaneous tocilizumab monotherapy in patients with rheumatoid arthritis and an inadequate response to subcutaneous tocilizumab every other week: Results of the 12-week double-blind period. Modern Rheumatology. 2018;28(1):76-84.
27. Dougados M, Kissel K, Sheeran T, Tak PP, Conaghan PG, Mola EM, et al. Adding tocilizumab or switching to tocilizumab monotherapy in methotrexate inadequate responders: 24-week symptomatic and structural results of a 2-year randomised controlled strategy trial in rheumatoid arthritis (ACT-RAY). Annals of the Rheumatic Diseases. 2013;72(1):43-50.

28. Dougados M, Kissel K, Conaghan PG, Mola EM, Schett G, Gerli R, et al. Clinical, radiographic and immunogenic effects after 1 year of tocilizumab-based treatment strategies in rheumatoid arthritis: the ACT-RAY study. Annals of the Rheumatic Diseases. 2014;73(5):803-9.
29. Kaneko Y, Atsumi T, Tanaka Y, Inoo M, Kobayashi-Haraoka H, Amano K, et al. Comparison of adding tocilizumab to methotrexate with switching to tocilizumab in patients with rheumatoid arthritis with inadequate response to methotrexate: 52-week results from a prospective, randomised, controlled study (SURPRISE study). Annals of the Rheumatic Diseases. 2016;75(11):1917-23.
30. Emery P, van Hoogstraten H, Thangavelu K, Mangan E, St John G, Verschueren P. Subcutaneous Sarilumab in Patients With Rheumatoid Arthritis who Previously Received Subcutaneous Sarilumab or Intravenous Tocilizumab: An Open-Label Extension of a Randomized Clinical Trial. ACR Open Rheumatology. 2020;2(11):672-80.
31. Burmester GR, Rigby WF, van Vollenhoven RF, Kay J, Rubbert-Roth A, Kelman A, et al. Tocilizumab in early progressive rheumatoid arthritis: FUNCTION, a randomised controlled trial. Annals of the Rheumatic Diseases. 2016;75(6):1081-91.
32. Burmester GR, Rigby WF, van Vollenhoven RF, Kay J, Rubbert-Roth A, Blanco R, et al. Tocilizumab combination therapy or monotherapy or methotrexate monotherapy in methotrexate-naive patients with early rheumatoid arthritis: 2-year clinical and radiographic results from the randomised, placebo-controlled FUNCTION trial. Annals of the Rheumatic Diseases. 2017;76(7):1279-84.
33. Bijlsma JWJ, Welsing PMJ, Woodworth TG, Middelink LM, Petho-Schramm A, Bernasconi C, et al. Early rheumatoid arthritis treated with tocilizumab, methotrexate, or their combination (U-Act-Early): a multicentre, randomised, double-blind, double-dummy, strategy trial. Lancet. 2016;388(10042):343-55.
34. Hetland ML, Haavardsholm EA, Rudin A, Nordstrom D, Nurmohamed M, Gudbjornsson B, et al. Active conventional treatment and three different biological treatments in early rheumatoid arthritis: phase IV investigator initiated, randomised, observer blinded clinical trial. BMJ. 2020;371:m4328.
35. Edwards CJ, Östör AJK, Naisbett-Groet B, Kiely P. Tapering versus steady-state methotrexate in combination with tocilizumab for rheumatoid arthritis: A randomized, double-blind trial. Rheumatology (United Kingdom). 2018;57(1):84-91.
36. Kremer JM, Rigby W, Singer NG, Birchwood C, Gill D, Reiss W, et al. Sustained Response Following Discontinuation of Methotrexate in Patients With Rheumatoid Arthritis Treated With Subcutaneous Tocilizumab: Results From a Randomized, Controlled Trial. Arthritis & Rheumatology. 2018;70(8):1200-8.
37. Pablos JL, Navarro F, Blanco FJ, Román-Ivorra JA, Alonso A, Martín Mola E, et al. Efficacy of tocilizumab monotherapy after response to combined tocilizumab and methotrexate in patients with rheumatoid arthritis: the randomised JUST-ACT study. Clinical and experimental rheumatology. 2019;37(3):437-44.
38. Peterfy C, Kremer J, Rigby W, Singer N, Birchwood C, Gill D, et al. Magnetic Resonance Imaging (MRI) Results Following Discontinuation of Methotrexate in Rheumatoid Arthritis Treated with Subcutaneous Tocilizumab: The COMP-ACT MRI Substudy. Journal of Rheumatology. 2020;47(3):325-32.
39. Burmester GR, Buttgereit F, Bernasconi C, Alvaro-Gracia JM, Castro N, Dougados M, et al. Continuing versus tapering glucocorticoids after achievement of low disease activity or remission in rheumatoid arthritis (SEMIRA): a double-blind, multicentre, randomised controlled trial. Lancet. 2020;396(10246):267-76.
40. Huizinga TWJ, Conaghan PG, Martin-Mola E, Schett G, Amital H, Xavier RM, et al. Clinical and radiographic outcomes at 2 years and the effect of tocilizumab discontinuation following sustained remission in the second and third year of the ACT-RAY study. Annals of the rheumatic diseases. 2015;74(1):35-43.

41. Kaneko Y, Kato M, Tanaka Y, Inoo M, Kobayashi-Haraoka H, Amano K, et al. Tocilizumab discontinuation after attaining remission in patients with rheumatoid arthritis who were treated with tocilizumab alone or in combination with methotrexate: results from a prospective randomised controlled study (the second year of the SURPRISE study). Annals of the Rheumatic Diseases. 2018;77(9):1268-75.
42. Kedra J, Dieudé P, Marotte H, Lafourcade A, Ducourau E, Schaeverbeke T, et al. Towards the lowest efficacious dose (toledo): Results of a multicenter non-inferiority randomized open-label controlled Trial Assessing Tocilizumab or Abatacept Injection Spacing in Rheumatoid Arthritis Patients in Remission. American College of Rheumatology Conference: Annual Meeting, ACR, USA, Atlanta. 2019;71:4916-9.
43. De Benedetti F, Brunner HI, Ruperto N, Kenwright A, Wright S, Calvo I, et al. Randomized trial of tocilizumab in systemic juvenile idiopathic arthritis. New England Journal of Medicine. 2012;367(25):2385-95.
44. Malattia C, Ruperto N, Pederzoli S, Palmisani E, Pistorio A, Wouters C, et al. Tocilizumab may slow radiographic progression in patients with systemic or polyarticular-course juvenile idiopathic arthritis: post hoc radiographic analysis from two randomized controlled trials. Arthritis Research & Therapy. 2020;22(1):211.
45. Brunner HI, Ruperto N, Zuber Z, Keane C, Harari O, Kenwright A, et al. Efficacy and safety of tocilizumab in patients with polyarticular-course juvenile idiopathic arthritis: Results from a phase 3, randomised, double-blind withdrawal trial. Annals of the Rheumatic Diseases. 2015;74(6):1110-7.
46. Kaneko Y, Kameda H, Ikeda K, Ishii T, Murakami K, Takamatsu H, et al. Tocilizumab in patients with adult-onset still's disease refractory to glucocorticoid treatment: a randomised, double-blind, placebo-controlled phase III trial. Annals of the Rheumatic Diseases. 2018;77(12):1720-9.
47. Stone JH, Tuckwell K, Dimonaco S, Klearman M, Aringer M, Blockmans D, et al. Trial of Tocilizumab in Giant-Cell Arteritis. New England Journal of Medicine. 2017;377(4):317-28.
48. Stone J, Spotswood H, Unizony S, Aringer M, Blockmans D, Brouwer E, et al. Time to flare in patients with new-onset versus relapsing giant cell arteritis treated with tocilizumab or placebo plus prednisone Tapering: 3-Year Results from a Randomized Controlled Phase 3 Trial. American College of Rheumatology Conference: Annual Meeting, ACR, USA, Atlanta 2019;71:3278-80.
49. Calderón-Goercke M, Loricera J, Prieto-Pena D, Castañeda S, Caceres VA, Villa I, et al. Tocilizumab in giant cell arteritis: Route of administration: Intravenous or subcutaneous. American College of Rheumatology Conference: Annual Meeting, ACR, USA, Atlanta. 2019;71:4744-6.
50. Schmidt WA, Dasgupta B, Luqmani R, Unizony SH, Blockmans D, Lai Z, et al. A Multicentre, Randomised, Double-Blind, Placebo-Controlled, Parallel-Group Study to Evaluate the Efficacy and Safety of Sirukumab in the Treatment of Giant Cell Arteritis. Rheumatology & Therapy. 2020;25:25.
51. Nakaoka Y, Isobe M, Takei S, Tanaka Y, Ishii T, Yokota S, et al. Efficacy and safety of tocilizumab in patients with refractory Takayasu arteritis: results from a randomised, double-blind, placebo-controlled, phase 3 trial in Japan (the TAKT study). Annals of the Rheumatic Diseases. 2018;77(3):348-54.
52. van Rhee F, Wong RS, Munshi N, Rossi JF, Ke XY, Fossa A, et al. Siltuximab for multicentric Castleman's disease: a randomised, double-blind, placebo-controlled trial. Lancet Oncology. 2014;15(9):966-74.
53. Le RQ, Li L, Yuan W, Shord SS, Nie L, Habtemariam BA, et al. FDA Approval Summary: Tocilizumab for Treatment of Chimeric Antigen Receptor T Cell-Induced Severe or Life-Threatening Cytokine Release Syndrome. Oncologist. 2018;23(8):943-7.
54. Zhang C, Zhang M, Qiu W, Ma H, Zhang X, Zhu Z, et al. Safety and efficacy of tocilizumab versus azathioprine in highly relapsing neuromyelitis optica spectrum disorder (TANGO): an open-label, multicentre, randomised, phase 2 trial. Lancet neurol. 2020;19(5):391-401.
55. Yamamura T, Kleiter I, Fujihara K, Palace J, Greenberg B, Zakrzewska-Pniewska B, et al. Trial of Satralizumab in Neuromyelitis Optica Spectrum Disorder. New England Journal of Medicine. 2019;381(22):2114-24.

56. Traboulsee A GB, Bennett JL, Szczechowski L, Fox E, Shkrobot S, Yamamura T, Terada Y, Kawata Y, Wright P, Gianella-Borradori A, Garren H, Weinshenker BG. Safety and efficacy of satralizumab monotherapy in neuromyelitis optica spectrum disorder: a randomised, double-blind, multicentre, placebo-controlled phase 3 trial. Lancet Neurol 2020;19(5):402-412
57. Emery P, Rondon J, Parrino J, Lin Y, Pena-Rossi C, van Hoogstraten H, et al. Safety and tolerability of subcutaneous sarilumab and intravenous tocilizumab in patients with rheumatoid arthritis. Rheumatology. 2019;58(5):849-58.
58. Mease PJ, Gottlieb AB, Berman A, Drescher E, Xing J, Wong R, et al. The Efficacy and Safety of Clazakizumab, an Anti-Interleukin-6 Monoclonal Antibody, in a Phase IIb Study of Adults With Active Psoriatic Arthritis. Arthritis & Rheumatology. 2016;68(9):2163-73.
59. Sieper J, Porter-Brown B, Thompson L, Harari O, Dougados M. Assessment of short-term symptomatic efficacy of tocilizumab in ankylosing spondylitis: results of randomised, placebo-controlled trials. Annals of the Rheumatic Diseases. 2014;73(1):95-100.
60. Sieper J, Braun J, Kay J, Badalamenti S, Radin AR, Jiao L, et al. Sarilumab for the treatment of ankylosing spondylitis: Results of a Phase II, randomised, double-blind, placebo-controlled study (ALIGN). Annals of the Rheumatic Diseases. 2015;74(6):1051-7.
61. Richette P LA, Sellam J, Wendling D, Piperno M, Goupille P, Pers YM, Eymard F, Ottaviani S, Ornetti P, Flipo RM, Fautrel B, Peyr O, Bertola JP, Vicaut E, Chevalier X. . Efficacy of tocilizumab in patients with hand osteoarthritis: double blind, randomised, placebo-controlled, multicentre trial. Ann Rheum Dis. 2020 Oct 14:annrheumdis-2020-218547. doi: 10.1136/annrheumdis-2020-218547. Epub ahead of print. PMID: 33055078.
62. Lally L, Forbess L, Hatzis C, Spiera R. Brief Report: A Prospective Open-Label Phase IIa Trial of Tocilizumab in the Treatment of Polymyalgia Rheumatica. Arthritis and Rheumatology. 2016;68(10):2550-4.
63. Devauchelle-Pensec V, Berthelot JM, Cornec D, Renaudineau Y, Marhadour T, Jousse-Joulin S, et al. Efficacy of first-line tocilizumab therapy in early polymyalgia rheumatica: A prospective longitudinal study. Annals of the Rheumatic Diseases. 2016;75(8):1506-10.
64. Khanna D, Lin CJF, Furst DE, Goldin J, Kim G, Kuwana M, et al. Tocilizumab in systemic sclerosis: a randomised, double-blind, placebo-controlled, phase 3 trial. The Lancet Respiratory Medicine. 2020;8(10):963-74.
65. Oddis C. Tocilizumab in the Treatment of Refractory Polymyositis and Dermatomyositis (TIM). Available: https://clinicaltrialsgov/ct2/show/study/NCT02043548 [Accessed 15 Nov 2020].
66. Wallace DJ, Strand V, Merrill JT, Popa S, Spindler AJ, Eimon A, et al. Efficacy and safety of an interleukin 6 monoclonal antibody for the treatment of systemic lupus erythematosus: a phase II dose-ranging randomised controlled trial. Annals of the Rheumatic Diseases. 2017;76(3):534-42.
67. Rovin BH, van Vollenhoven RF, Aranow C, Wagner C, Gordon R, Zhuang Y, et al. A Multicenter, Randomized, Double-Blind, Placebo-Controlled Study to Evaluate the Efficacy and Safety of Treatment With Sirukumab (CNTO 136) in Patients With Active Lupus Nephritis. Arthritis & Rheumatology. 2016;68(9):2174-83.
68. Ablynx. A Phase II Study to Evaluate Safety and Efficacy of ALX-0061 in Subjects With Systemic Lupus Erythematosus Available: https://clinicaltrials.gov/ct2/show/NCT02437890 [Accessed 09 Nov 2021].
69. Felten R, Devauchelle-Pensec V, Seror R, Duffau P, Saadoun D, Hachulla E, et al. Interleukin 6 receptor inhibition in primary Sjögren syndrome: a multicentre double-blind randomised placebo-controlled trial. Annals of the Rheumatic Diseases.2020 Nov 18;annrheumdis-2020-218467. doi: 10.1136/annrheumdis-2020-218467. Online ahead of print.
70. Okuda Y, Ohnishi M, Matoba K, Jouyama K, Yamada A, Sawada N, et al. Comparison of the clinical utility of tocilizumab and anti-TNF therapy in AA amyloidosis complicating rheumatic diseases. Modern Rheumatology. 2014;24(1):137-43.
71. Okuda Y, Yamada T, Ueda M, Ando Y. First nationwide survey of 199 patients with amyloid a amyloidosis in Japan. Internal Medicine. 2018;57(23):3351-5.

72. San-Miguel J, Bladé J, Shpilberg O, Grosicki S, Maloisel F, Min C-K, et al. Phase 2 randomized study of bortezomib-melphalan-prednisone with or without siltuximab (anti–IL-6) in multiple myeloma. Blood. 2014;123(26):4136-42.
73. Brighton TA, Khot A, Harrison SJ, Ghez D, Weiss BM, Kirsch A, et al. Randomized, Double-Blind, Placebo-Controlled, Multicenter Study of Siltuximab in High-Risk Smoldering Multiple Myeloma. Clin Cancer Res. 2019;25(13):3772-5.
74. Rodriguez-Bano J, Pachon J, Carratala J, Ryan P, Jarrin I, Yllescas M, et al. Treatment with tocilizumab or corticosteroids for COVID-19 patients with hyperinflammatory state: a multicentre cohort study (SAM-COVID-19). Clin Microbiol Infect. 2020;27:27.
75. Ip A, Berry DA, Hansen E, Goy AH, Pecora AL, Sinclaire BA, et al. Hydroxychloroquine and tocilizumab therapy in COVID-19 patients-An observational study. PLoS ONE. 2020;15(8 August).
76. Guaraldi G, Meschiari M, Cozzi-Lepri A, Milic J, Tonelli R, Menozzi M, et al. Tocilizumab in patients with severe COVID-19: a retrospective cohort study. The Lancet Rheumatology. 2020;2(8):e474-e84.
77. Biran N, Ip A, Ahn J, Go RC, Wang S, Mathura S, et al. Tocilizumab among patients with COVID-19 in the intensive care unit: a multicentre observational study. The Lancet Rheumatology. 2020;2(10):e603-e12.
78. Gupta S, Wang W, Hayek SS, Chan L, Mathews KS, Melamed ML, et al. Association Between Early Treatment With Tocilizumab and Mortality Among Critically Ill Patients With COVID-19. JAMA Internal Medicine. 2020;20:20.
79. Della-Torre E, Campochiaro C, Cavalli G, De Luca G, Napolitano A, La Marca S, et al. Interleukin-6 blockade with sarilumab in severe COVID-19 pneumonia with systemic hyperinflammation: An open-label cohort study. Annals of the Rheumatic Diseases. 2020;79(10):1277-85.
80. Ramiro S, Mostard RLM, Magro-Checa C, van Dongen CMP, Dormans T, Buijs J, et al. Historically controlled comparison of glucocorticoids with or without tocilizumab versus supportive care only in patients with COVID-19-associated cytokine storm syndrome: results of the CHIC study. Annals of the Rheumatic Diseases. 2020;79(9):1143-51.
81. Hermine O, Mariette X, Tharaux PL, Resche-Rigon M, Porcher R, Ravaud P, et al. Effect of Tocilizumab vs Usual Care in Adults Hospitalized With COVID-19 and Moderate or Severe Pneumonia: A Randomized Clinical Trial. JAMA Internal Medicine. 2020;20:20.
82. Salvarani C, Dolci G, Massari M, Merlo DF, Cavuto S, Savoldi L, et al. Effect of Tocilizumab vs Standard Care on Clinical Worsening in Patients Hospitalized with COVID-19 Pneumonia: A Randomized Clinical Trial. JAMA Internal Medicine. 2020;Article in Press.
83. Stone JH, Frigault MJ, Serling-Boyd NJ, Fernandes AD, Harvey L, Foulkes AS, et al. Efficacy of Tocilizumab in Patients Hospitalized with Covid-19. New England journal of medicine. 2020;Article in Press.
84. Salama C, Han J, Yau L, Reiss WG, Kramer B, Neidhart JD, et al. Tocilizumab in Patients Hospitalized with Covid-19 Pneumonia. New England Journal of Medicine. 2020;384(1):20-30.
85. Doberer K, Duerr M, Halloran PF, Eskandary F, Budde K, Regele H, et al. A Randomized Clinical Trial of Anti–IL-6 Antibody Clazakizumab in Late Antibody-Mediated Kidney Transplant Rejection. Journal of the American Society of Nephrology. published online ahead of print Dec 18, 2020.
86. Kim SC, Solomon DH, Rogers JR, Gale S, Klearman M, Sarsour K, et al. Cardiovascular Safety of Tocilizumab Versus Tumor Necrosis Factor Inhibitors in Patients With Rheumatoid Arthritis: A Multi-Database Cohort Study. Arthritis and Rheumatology. 2017;69(6):1154-64.
87. Kim SC, Solomon DH, Rogers JR, Gale S, Klearman M, Sarsour K, et al. No difference in cardiovascular risk of tocilizumab versus abatacept for rheumatoid arthritis: A multi-database cohort study. Seminars in Arthritis and Rheumatism. 2018;48(3):399-405.
88. Xie F, Yun H, Levitan EB, Muntner P, Curtis JR. Tocilizumab and the Risk of Cardiovascular Disease: Direct Comparison Among Biologic Disease-Modifying Antirheumatic Drugs for Rheumatoid Arthritis Patients. Arthritis Care and Research. 2019;71(8):1004-18.

89. Giles JT, Sattar N, Gabriel S, Ridker PM, Gay S, Warne C, et al. Cardiovascular Safety of Tocilizumab Versus Etanercept in Rheumatoid Arthritis: A Randomized Controlled Trial. Arthritis & Rheumatology. 2020;72(1):31-40.
90. Zhang J, Xie F, Yun H, Chen L, Muntner P, Levitan EB, et al. Comparative effects of biologics on cardiovascular risk among older patients with rheumatoid arthritis. Annals of the Rheumatic Diseases. 2016;75(10):1813-8.
91. Generali E, Carrara G, Selmi C, Verstappen S, Zambon A, Bortoluzzi A, et al. Comparison of the risks of hospitalisation for cardiovascular events in patients with rheumatoid arthritis treated with tocilizumab and etanercept. Clinical and Experimental Rheumatology. 2018;36(2):310-3.
92. Lukas C, Redondin M, Pane I, Soubrier M, Houvenagel E, Sibilia J, et al. Cardiovascular events and change in cholesterol levels in patients with rheumatoid arthritis treated with tocilizumab: data from the REGATE Registry. Clinical and experimental rheumatology. 2020.
93. Crnkic Kapetanovic M, Saxne T, Jonsson G, Truedsson L, Geborek P. Rituximab and abatacept but not tocilizumab impair antibody response to pneumococcal conjugate vaccine in patients with rheumatoid arthritis. Arthritis Research & Therapy. 2013;15(5):R171.
94. Mori S, Ueki Y, Akeda Y, Hirakata N, Oribe M, Shiohira Y, et al. Pneumococcal polysaccharide vaccination in rheumatoid arthritis patients receiving tocilizumab therapy. Annals of the Rheumatic Diseases. 2013;72(8):1362-6.
95. Tsuru T, Terao K, Murakami M, Matsutani T, Suzaki M, Amamoto T, et al. Immune response to influenza vaccine and pneumococcal polysaccharide vaccine under IL-6 signal inhibition therapy with tocilizumab. Modern Rheumatology. 2014;24(3):511-6.
96. Bingham CO, 3rd, Rizzo W, Kivitz A, Hassanali A, Upmanyu R, Klearman M. Humoral immune response to vaccines in patients with rheumatoid arthritis treated with tocilizumab: results of a randomised controlled trial (VISARA). Annals of the Rheumatic Diseases. 2015;74(5):818-22.
97. Shinoki T, Hara R, Kaneko U, Miyamae T, Imagawa T, Mori M, et al. Safety and response to influenza vaccine in patients with systemic-onset juvenile idiopathic arthritis receiving tocilizumab. Modern Rheumatology. 2012;22(6):871-6.
98. Carrara G, Bortoluzzi A, Sakellariou G, Silvagni E, Zanetti A, Govoni M, et al. Risk of hospitalisation for serious bacterial infections in patients with rheumatoid arthritis treated with biologics. Analysis from the RECORD linkage on rheumatic disease study of the Italian Society for Rheumatology. Clinical and Experimental Rheumatology. 2019;37(1):60-6.
99. Mori S, Yoshitama T, Hidaka T, Sakai F, Hasegawa M, Hashiba Y, et al. Comparative risk of hospitalized infection between biological agents in rheumatoid arthritis patients: A multicenter retrospective cohort study in Japan. PLoS ONE. 2017;12(6).
100. Rutherford AI, Subesinghe S, Hyrich KL, Galloway JB. Serious infection across biologic-treated patients with rheumatoid arthritis: Results from the British Society for Rheumatology Biologics Register for Rheumatoid Arthritis. Annals of the Rheumatic Diseases. 2018;77(6):905-10.
101. Pawar A, Desai RJ, Solomon DH, Santiago Ortiz AJ, Gale S, Bao M, et al. Risk of serious infections in tocilizumab versus other biologic drugs in patients with rheumatoid arthritis: A multidatabase cohort study. Annals of the Rheumatic Diseases. 2019;78(4):456-64.
102. Grøn KL, Arkema EV, Glintborg B, Mehnert F, Østergaard M, Dreyer L, et al. Risk of serious infections in patients with rheumatoid arthritis treated in routine care with abatacept, rituximab and tocilizumab in Denmark and Sweden. Annals of the Rheumatic Diseases. 2019;78(3):320-7.
103. Grøn KL, Glintborg B, Nørgaard M, Mehnert F, Østergaard M, Dreyer L, et al. Overall infection risk in rheumatoid arthritis during treatment with abatacept, rituximab and tocilizumab; an observational cohort study. Rheumatology (Oxford, England). 2020;59(8):1949-56.
104. Morel J, Constantin A, Baron G, Dernis E, Flipo RM, Rist S, et al. Risk factors of serious infections in patients with rheumatoid arthritis treated with tocilizumab in the French Registry REGATE. Rheumatology (United Kingdom). 2017;56(10):1746-54.
105. Sakai R, Cho SK, Nanki T, Watanabe K, Yamazaki H, Tanaka M, et al. Head-to-head comparison of the safety of tocilizumab and tumor necrosis factor inhibitors in rheumatoid arthritis

patients (RA) in clinical practice: results from the registry of Japanese RA patients on biologics for long-term safety (REAL) registry. Arthritis Research & Therapy. 2015;17:74.
106. Yun H, Xie F, Delzell E, Levitan EB, Chen L, Lewis JD, et al. Comparative Risk of Hospitalized Infection Associated with Biologic Agents in Rheumatoid Arthritis Patients Enrolled in Medicare. Arthritis and Rheumatology. 2016;68(1):56-66.
107. Rutherford AI, Patarata E, Subesinghe S, Hyrich KL, Galloway JB. Opportunistic infections in rheumatoid arthritis patients exposed to biologic therapy: Results from the British Society for Rheumatology Biologics Register for Rheumatoid Arthritis. Rheumatology (United Kingdom). 2018;57(6):997-1001.
108. Leon L, Penuelas M, Candel FJ, Freites D, Rodriguez-Rodriguez L, Fernandez-Gutierrez B, et al. Indicator opportunistic infections after biological treatment in rheumatoid arthritis, 10 years follow-up in a real-world setting. Therapeutic Advances in Musculoskeletal Disease. 2019;11:1759720X19878004.
109. Lim CH, Chen HH, Chen YH, Chen DY, Huang WN, Tsai JJ, et al. The risk of tuberculosis disease in rheumatoid arthritis patients on biologics and targeted therapy: A 15-year real world experience in Taiwan. PLoS ONE. 2017;12(6).
110. Wang X, Wong SH, Wang XS, Tang W, Liu CQ, Niamul G, et al. Risk of tuberculosis in patients with immune-mediated diseases on biological therapies: A population-based study in a tuberculosis endemic region. Rheumatology (United Kingdom). 2019;58(5):803-10.
111. Curtis JR, Xie F, Yun H, Bernatsky S, Winthrop KL. Real-world comparative risks of herpes virus infections in tofacitinib and biologic-treated patients with rheumatoid arthritis. Annals of the Rheumatic Diseases. 2016;75(10):1843-7.
112. Yun H, Xie F, Delzell E, Chen L, Levitan EB, Lewis JD, et al. Risks of herpes zoster in patients with rheumatoid arthritis according to biologic disease-modifying therapy. Arthritis Care and Research. 2015;67(5):731-6.
113. Wadström H, Frisell T, Askling J. Malignant neoplasms in patients with rheumatoid arthritis treated with tumor necrosis factor inhibitors, tocilizumab, abatacept, or rituximab in clinical practice: A nationwide cohort study from Sweden. JAMA Internal Medicine. 2017;177(11):1605-12.
114. Kim SC, Pawar A, Desai RJ, Solomon DH, Gale S, Bao M, et al. Risk of malignancy associated with use of tocilizumab versus other biologics in patients with rheumatoid arthritis: A multi-database cohort study. Seminars in Arthritis and Rheumatism. 2019;49(2):222-8.
115. Hellgren K, Di Giuseppe D, Smedby KE, Sundström C, Askling J, Baecklund E. Lymphoma risks in patients with rheumatoid arthritis treated with biological drugs-a Swedish cohort study of risks by time, drug and lymphoma subtype. Rheumatology (Oxford, England). 2020.
116. Mercer LK, Askling J, Raaschou P, Dixon WG, Dreyer L, Hetland ML, et al. Risk of invasive melanoma in patients with rheumatoid arthritis treated with biologics: Results from a collaborative project of 11 European biologic registers. Annals of the Rheumatic Diseases. 2017;76(2):386-91.
117. Rempenault C, Lukas C, Combe B, Schaeverbeke T, Wendling D, Pham T, et al. RISK OF DIVERTICULITIS AND GASTRO-INTESTINAL PERFORATION IN RHEUMATOID ARTHRITIS TREATED WITH TOCILIZUMAB COMPARED TO RITUXIMAB AND ABATACEPT: A PROSPECTIVE PROPENSITY-MATCHED COHORT STUDY. Annals of the Rheumatic Diseases Conference: annual european congress of rheumatology of the european league against rheumatism, EULAR 2020, Germany. 2020;79(Suppl 1):17-.
118. Monemi S, Berber E, Sarsour K, Wang J, Lampl K, Bharucha K, et al. Incidence of Gastrointestinal Perforations in Patients with Rheumatoid Arthritis Treated with Tocilizumab from Clinical Trial, Postmarketing, and Real-World Data Sources. Rheumatology & Therapy. 2016;3(2):337-52.
119. Strangfeld A, Richter A, Siegmund B, Herzer P, Rockwitz K, Demary W, et al. Risk for lower intestinal perforations in patients with rheumatoid arthritis treated with tocilizumab in comparison to treatment with other biologic or conventional synthetic DMARDs. Annals of the Rheumatic Diseases. 2017;76(3):504-10.

120. Barbulescu A, Delcoigne B, Askling J, Frisell T. Gastrointestinal perforations in patients with rheumatoid arthritis treated with biological disease-modifying antirheumatic drugs in Sweden: A nationwide cohort study. RMD Open. 2020;6(2).
121. Xie F, Yun H, Bernatsky S, Curtis JR. Brief Report: Risk of Gastrointestinal Perforation Among Rheumatoid Arthritis Patients Receiving Tofacitinib, Tocilizumab, or Other Biologic Treatments. Arthritis and Rheumatology. 2016;68(11):2612-7.
122. Koike T, Harigai M, Inokuma S, Ishiguro N, Ryu J, Takeuchi T, et al. Effectiveness and safety of tocilizumab: postmarketing surveillance of 7901 patients with rheumatoid arthritis in Japan. Journal of Rheumatology. 2014;41(1):15-23.
123. Genovese MC, Kremer JM, van Vollenhoven RF, Alten R, Scali JJ, Kelman A, et al. Transaminase Levels and Hepatic Events During Tocilizumab Treatment: Pooled Analysis of Long-Term Clinical Trial Safety Data in Rheumatoid Arthritis. Arthritis & Rheumatology. 2017;69(9):1751-61.
124. Brodszky V, Biro A, Szekanecz Z, Soos B, Baji P, Rencz F, et al. Determinants of biological drug survival in rheumatoid arthritis: evidence from a Hungarian rheumatology center over 8 years of retrospective data. Clinicoeconomics & Outcomes Research. 2017;9:139-47.
125. Ebina K, Hashimoto M, Yamamoto W, Ohnishi A, Kabata D, Hirano T, et al. Drug retention and discontinuation reasons between seven biologics in patients with rheumatoid arthritis -The ANSWER cohort study. PLoS ONE. 2018;13(3).
126. Ebina K, Hirano T, Maeda Y, Yamamoto W, Hashimoto M, Murata K, et al. Drug retention of 7 biologics and tofacitinib in biologics-naive and biologics-switched patients with rheumatoid arthritis: the ANSWER cohort study. Arthritis Research & Therapy. 2020;22(1):142.
127. Ebina K, Hashimoto M, Yamamoto W, Hirano T, Hara R, Katayama M, et al. Drug tolerability and reasons for discontinuation of seven biologics in elderly patients with rheumatoid arthritis -The ANSWER cohort study. PLoS ONE. 2019;14(5).
128. Gottenberg JE, Morel J, Perrodeau E, Bardin T, Combe B, Dougados M, et al. Comparative effectiveness of rituximab, abatacept, and tocilizumab in adults with rheumatoid arthritis and inadequate response to TNF inhibitors: prospective cohort study. BMJ. 2019;364:l67.
129. Yun H, Xie F, Beyl RN, Chen L, Lewis JD, Saag KG, et al. Risk of Hypersensitivity to Biologic Agents Among Medicare Patients With Rheumatoid Arthritis. Arthritis care & research. 2017;69(10):1526-34.
130. Salmon JH, Perotin JM, Morel J, Dramé M, Cantagrel A, Ziegler LE, et al. Serious infusion-related reaction after rituximab, abatacept and tocilizumab in rheumatoid arthritis: prospective registry data. Rheumatology (Oxford, England). 2018;57(1):134-9.
131. McInnes IB, Thompson L, Giles JT, Bathon JM, Salmon JE, Beaulieu AD, et al. Effect of interleukin-6 receptor blockade on surrogates of vascular risk in rheumatoid arthritis: MEASURE, a randomised, placebo-controlled study. Annals of the Rheumatic Diseases. 2015;74(4):694-702.
132. Gabay C, McInnes IB, Kavanaugh A, Tuckwell K, Klearman M, Pulley J, et al. Comparison of lipid and lipid-associated cardiovascular risk marker changes after treatment with tocilizumab or adalimumab in patients with rheumatoid arthritis. Annals of the Rheumatic Diseases. 2016;75(10):1806-12.
133. Chen SK, Lee H, Jin Y, Liu J, Kim SC. Use of biologic or targeted-synthetic disease-modifying anti-rheumatic drugs and risk of diabetes treatment intensification in patients with rheumatoid arthritis and diabetes mellitus. Rheumatol Adv Pract. 2020;4(2):rkaa027.
134. Genovese MC, Burmester GR, Hagino O, Thangavelu K, Iglesias-Rodriguez M, John GS, et al. Interleukin-6 receptor blockade or TNFα inhibition for reducing glycaemia in patients with RA and diabetes: Post hoc analyses of three randomised, controlled trials. Arthritis Research and Therapy. 2020;22(1).
135. Paul SK, Montvida O, Best JH, Gale S, Pethoe-Schramm A, Sarsour K. Effectiveness of biologic and non-biologic antirheumatic drugs on anaemia markers in 153,788 patients with rheumatoid arthritis: New evidence from real-world data. Seminars in Arthritis and Rheumatism. 2018;47(4):478-84.

136. Moots RJ, Sebba A, Rigby W, Ostor A, Porter-Brown B, Donaldson F, et al. Effect of tocilizumab on neutrophils in adult patients with rheumatoid arthritis: pooled analysis of data from phase 3 and 4 clinical trials. Rheumatology. 2017;56(4):541-9.
137. Mori S, Yoshitama T, Hidaka T, Hirakata N, Ueki Y. Effectiveness and safety of tocilizumab therapy for patients with rheumatoid arthritis and renal insufficiency: a real-life registry study in Japan (the ACTRA-RI study). Annals of the rheumatic diseases. 2015;74(3):627-30.
138. Curtis JR, Sarsour K, Napalkov P, Costa LA, Schulman KL. Incidence and complications of interstitial lung disease in users of tocilizumab, rituximab, abatacept and anti-tumor necrosis factor aα agents, a retrospective cohort study. Arthritis Research and Therapy. 2015;17(1).
139. Strangfeld A, Meißner Y, Schaefer M, Baganz L, Schneider M, Wilden E, et al. No confirmation of increased risk of idiopathic facial nerve palsy under tocilizumab. Annals of the Rheumatic Diseases Conference: annual european congress of rheumatology of the european league against rheumatism, EULAR 2019, Spain 2019;78:351-2.
140. Shin A, Dong YH, Shin S, Ha YJ, Lee YJ, Lee EB, et al. The comparative risk of osteoporotic fractures among patients with rheumatoid arthritis receiving TNF inhibitors versus other biologics: A nation-wide cohort study in Korea. 2019;78(Annals of the Rheumatic Diseases Conference: annual european congress of rheumatology of the european league against rheumatism, EULAR 2019, Spain 2019):726.
141. Kume K, Amano K, Yamada S, Kanazawa T, Ohta H, Hatta K, et al. The effect of tocilizumab on bone mineral density in patients with methotrexate-resistant active rheumatoid arthritis. Rheumatology. 2014;53(5):900-3.
142. Chen YM, Chen HH, Huang WN, Liao TL, Chen JP, Chao WC, et al. Tocilizumab potentially prevents bone loss in patients with anticitrullinated protein antibody-positive rheumatoid arthritis. PLoS ONE. 2017;12(11).
143. Hoeltzenbein M, Beck E, Rajwanshi R, Gøtestam Skorpen C, Berber E, Schaefer C, et al. Tocilizumab use in pregnancy: Analysis of a global safety database including data from clinical trials and post-marketing data. Seminars in Arthritis and Rheumatism. 2016;46(2):238-45.
144. Weber-Schoendorfer C, Schaefer C. Pregnancy outcome after tocilizumab therapy in early pregnancy-a case series from the German Embryotox Pharmacovigilance Center. Reproductive Toxicology. 2016;60:29-32.
145. Kameda H, Wada K, Takahashi Y, Hagino O, van Hoogstraten H, Graham N, et al. Sarilumab monotherapy or in combination with non-methotrexate disease-modifying antirheumatic drugs in active rheumatoid arthritis: A Japan phase 3 trial (HARUKA). Modern Rheumatology. 2020;30(2):239-48.
146. Fleischmann R, Genovese MC, Lin Y, John GS, van der Heijde D, Wang S, et al. Long-term safety of sarilumab in rheumatoid arthritis: An integrated analysis with up to 7 years' follow-up. Rheumatology (United Kingdom). 2020;59(2):292-302.
147. Takeuchi T, Yamanaka H, Harigai M, Tamamura R, Kato Y, Ukyo Y, et al. Sirukumab in rheumatoid arthritis refractory to sulfasalazine or methotrexate: a randomized phase 3 safety and efficacy study in Japanese patients. Arthritis Research & Therapy. 2018;20(1):42.
148. Thorne C, Takeuchi T, Karpouzas GA, Sheng S, Kurrasch R, Fei K, et al. Investigating sirukumab for rheumatoid arthritis: 2-year results from the phase III SIRROUND-D study. RMD Open. 2018;4(2):e000731.
149. Horneff G, Klein A, Klotsche J, Minden K, Huppertz HI, Weller-Heinemann F, et al. Comparison of treatment response, remission rate and drug adherence in polyarticular juvenile idiopathic arthritis patients treated with etanercept, adalimumab or tocilizumab. Arthritis Research and Therapy. 2016;18(1).
150. Grönlund MM, Remes-Pakarinen T, Kröger L, Markula-Patjas K, Backström M, Putto-Laurila A, et al. Efficacy and safety of tocilizumab in a real-life observational cohort of patients with polyarticular juvenile idiopathic arthritis. Rheumatology (United Kingdom). 2020;59(4):732-41.

151. Klein A, Klotsche J, Hügle B, Minden K, Hospach A, Weller-Heinemann F, et al. Long-term surveillance of biologic therapies in systemic-onset juvenile idiopathic arthritis: data from the German BIKER registry. Rheumatology (Oxford, England). 2020;59(9):2287-98.
152. Shafran IH, Alasti F, Smolen JS, Aletaha D. Implication of baseline levels and early changes of C-reactive protein for subsequent clinical outcomes of patients with rheumatoid arthritis treated with tocilizumab. Annals of the Rheumatic Diseases. 2020;79(7):874-82.
153. Shimamoto K, Ito T, Ozaki Y, Amuro H, Tanaka A, Nishizawa T, et al. Serum interleukin 6 before and after therapy with tocilizumab is a principal biomarker in patients with rheumatoid arthritis. Journal of Rheumatology. 2013;40(7):1074-81.
154. Nishimoto N, Amano K, Hirabayashi Y, Horiuchi T, Ishii T, Iwahashi M, et al. Drug free REmission/low disease activity after cessation of tocilizumab (Actemra) Monotherapy (DREAM) study. Modern Rheumatology. 2014;24(1):17-25.
155. Strand V, Boklage SH, Kimura T, Joly F, Boyapati A, Msihid J. High levels of interleukin-6 in patients with rheumatoid arthritis are associated with greater improvements in health-related quality of life for sarilumab compared with adalimumab. Arthritis Research & Therapy. 2020;22(1):250.
156. Boyapati A, Schwartzman S, Msihid J, Choy E, Genovese MC, Burmester GR, et al. Association of High Serum Interleukin-6 Levels With Severe Progression of Rheumatoid Arthritis and Increased Treatment Response Differentiating Sarilumab From Adalimumab or Methotrexate in a Post Hoc Analysis. Arthritis & Rheumatology. 2020;28:28.
157. Cappelli LC, Palmer JL, Kremer J, Bingham CO, 3rd. Tocilizumab treatment leads to improvement in disease activity regardless of CCP status in rheumatoid arthritis. Seminars in Arthritis & Rheumatism. 2017;47(2):165-9.
158. Sanayama Y, Ikeda K, Saito Y, Kagami S, Yamagata M, Furuta S, et al. Prediction of therapeutic responses to tocilizumab in patients with rheumatoid arthritis: biomarkers identified by analysis of gene expression in peripheral blood mononuclear cells using genome-wide DNA microarray. Arthritis & Rheumatology. 2014;66(6):1421-31.
159. Maldonado-Montoro M, Canadas-Garre M, Gonzalez-Utrilla A, Plaza-Plaza JC, Calleja-Hernandez MY. Genetic and clinical biomarkers of tocilizumab response in patients with rheumatoid arthritis. Pharmacological Research. 2016;111:264-71.
160. Jiménez Morales A, Maldonado-Montoro M, Martínez de la Plata JE, Pérez Ramírez C, Daddaoua A, Alarcón Payer C, et al. FCGR2A/FCGR3A Gene Polymorphisms and Clinical Variables as Predictors of Response to Tocilizumab and Rituximab in Patients With Rheumatoid Arthritis. Journal of Clinical Pharmacology. 2019;59(4):517-31.
161. Daien CI, Gailhac S, Audo R, Mura T, Hahne M, Combe B, et al. High levels of natural killer cells are associated with response to tocilizumab in patients with severe rheumatoid arthritis. Rheumatology. 2015;54(4):601-8.
162. Humby F, Buch MH, Durez P, Lewis M, Bombardieri M, Rizvi H, et al. A randomised, open labelled clinical trial to investigate synovial mechanisms determining response-resistance to rituximab versus tocilizumab in rheumatoid arthritis patients failing TNF inhibitor therapy. American College of Rheumatology Conference: Annual Meeting, ACR, USA, Atlanta. 2019;71:5180-3.
163. Dulic S, Vásárhelyi Z, Sava F, Berta L, Szalay B, Toldi G, et al. T-Cell Subsets in Rheumatoid Arthritis Patients on Long-Term Anti-TNF or IL-6 Receptor Blocker Therapy. Mediators of Inflammation. 2017;2017.
164. Gabay C, Msihid J, Zilberstein M, Paccard C, Lin Y, Graham NMH, et al. Identification of sarilumab pharmacodynamic and predictive markers in patients with inadequate response to TNF inhibition: a biomarker substudy of the phase 3 TARGET study. RMD Open. 2018;4(1):e000607.
165. Gabay C, Burmester GR, Strand V, Msihid J, Zilberstein M, Kimura T, et al. Sarilumab and adalimumab differential effects on bone remodelling and cardiovascular risk biomarkers, and predictions of treatment outcomes. Arthritis Research & Therapy. 2020;22(1):70.

166. Toussirot E, Marotte H, Mulleman D, Cormier G, Coury F, Gaudin P, et al. Increased high molecular weight adiponectin and lean mass during tocilizumab treatment in patients with rheumatoid arthritis: a 12-month multicentre study. Arthritis Research & Therapy. 2020;22(1):224.
167. Fioravanti A, Tenti S, Bacarelli MR, Damiani A, Li Gobbi F, Bandinelli F, et al. Tocilizumab modulates serum levels of adiponectin and chemerin in patients with rheumatoid arthritis: potential cardiovascular protective role of IL-6 inhibition. Clinical & Experimental Rheumatology. 2019;37(2):293-300.
168. Gerasimova H, Popkova T, Kirillova I, Cherkasova M, Martynova A, Novikova D, et al. Significant improvement of NT-probnp levels in rheumatoid arthritis patients treated with tocilizumab and tofacitinib. Annals of the Rheumatic Diseases Conference: annual european congress of rheumatology of the european league against rheumatism, EULAR 2020, Germany. 2020;79(SUPPL 1):625-6.
169. Gardette A, Ottaviani S, Sellam J, Berenbaum F, Lioté F, Meyer A, et al. Body mass index and response to tocilizumab in rheumatoid arthritis: A real life study. Clinical Rheumatology. 2016;35(4):857-61.
170. Schäfer M, Meißner Y, Kekow J, Berger S, Remstedt S, Manger B, et al. Obesity reduces the real-world effectiveness of cytokine-targeted but not cell-targeted disease-modifying agents in rheumatoid arthritis. Rheumatology (Oxford, England). 2020;59(8):1916-26.
171. Davies R, Vivekanantham A, Lunt M, Watson K, Hyrich K, Bluett J. The effect of bodyweight on response to intravenous or subcutaneous tocilizumab in patients with rheumatoid arthritis. Annals of the Rheumatic Diseases Conference: annual european congress of rheumatology of the european league against rheumatism, EULAR 2020, Germany. 2020;79(SUPPL 1):985.
172. Forsblad-d'Elia H, Bengtsson K, Kristensen LE, Jacobsson LTH. Drug adherence, response and predictors thereof for tocilizumab in patients with rheumatoid arthritis: Results from the Swedish biologics register. Rheumatology (United Kingdom). 2015;54(7):1186-93.
173. Pappas DA, Blachley T, Best JH, Zlotnick S, Emeanuru K, Kremer JM. Persistence of tocilizumab therapy among patients with rheumatoid arthritis: Data from the us-based corrona rheumatoid arthritis registry. Annals of the Rheumatic Diseases Conference: annual european congress of rheumatology of the european league against rheumatism, EULAR 2020, Germany. 2020;79(SUPPL 1):631-2.
174. Best JH, Vlad SC, Tominna L, Abbass I. Real-World Persistence with Tocilizumab Compared to Other Subcutaneous Biologic Disease-Modifying Antirheumatic Drugs Among Patients with Rheumatoid Arthritis Switching from Another Biologic. Rheumatology and Therapy. 2020;7(2):345-55.
175. Saraux A, Barnetche T, Baudens G, Idier I, Delaporte F, Hilliquin P. Subcutaneous tocilizumab in monotherapy or in combination with csDMARD in patients with moderate to severe rheumatoid arthritis: Observational study to describe real-world drug retention rate at 12 months. Annals of the Rheumatic Diseases Conference: annual european congress of rheumatology of the european league against rheumatism, EULAR 2019, Spain. 2019;78:725-6.
176. Haraoui B, Casado G, Czirják L, Taylor A, Dong L, Button P, et al. Tocilizumab Patterns of Use, Effectiveness, and Safety in Patients with Rheumatoid Arthritis: Final Results from a Set of Multi-National Non-Interventional Studies. Rheumatology and Therapy. 2019;6(2):231-43.
177. Tanaka Y, Kameda H, Saito K, Kaneko Y, Tanaka E, Yasuda S, et al. Effect of subcutaneous tocilizumab treatment on work/housework status in biologic-naïve rheumatoid arthritis patients using inverse probability of treatment weighting: FIRST ACT-SC study. Arthritis Research and Therapy. 2018;20(1).
178. Strand V, Michalska M, Birchwood C, Pei J, Tuckwell K, Finch R, et al. Impact of tocilizumab monotherapy on patient-reported outcomes in patients with rheumatoid arthritis from two randomised controlled trials. RMD Open. 2017;3(2):e000496.
179. Strand V, Gossec L, Proudfoot CWJ, Chen CI, Reaney M, Guillonneau S, et al. Patient-reported outcomes from a randomized phase III trial of sarilumab monotherapy versus adalimumab monotherapy in patients with rheumatoid arthritis. Arthritis Research & Therapy. 2018;20(1):129.

180. Strand V, Michalska M, Birchwood C, Pei J, Tuckwell K, Finch R, et al. Impact of tocilizumab administered intravenously or subcutaneously on patient-reported quality-of-life outcomes in patients with rheumatoid arthritis. RMD Open. 2018;4(1):e000602.
181. Strand V, Dimonaco S, Tuckwell K, Klearman M, Collinson N, Stone JH. Health-related quality of life in patients with giant cell arteritis treated with tocilizumab in a phase 3 randomised controlled trial. Arthritis Research & Therapy. 2019;21(1):64.
182. Ayaz NA, Karadağ ŞG, Koç R, Demirkan FG, Çakmak F, Sönmez HE. Patient satisfaction and clinical effectiveness of switching from intravenous tocilizumab to subcutaneous tocilizumab in patients with juvenile idiopathic arthritis: an observational study. Rheumatology International. 2020;40(7):1111-6.
183. Soini EJ, Hallinen TA, Puolakka K, Vihervaara V, Kauppi MJ. Cost-effectiveness of adalimumab, etanercept, and tocilizumab as first-line treatments for moderate-to-severe rheumatoid arthritis. Journal of Medical Economics. 2012;15(2):340-51.
184. Johnston SS, McMorrow D, Farr AM, Juneau P, Ogale S. Comparison of Healthcare Costs Between Rheumatoid Arthritis Patients Treated with Infused Biologics After Switching from Another Biologic. Drugs - Real World Outcomes. 2015;2(1):99-109.
185. Verhoeven MMA, Tekstra J, van Laar JM, Pethö-Schramm A, Borm MEA, Bijlsma JWJ, et al. Impact on costs and quality-adjusted-life-years of treat-to-target treatment strategies initiating methotrexate, or tocilizumab, or their combination in early rheumatoid arthritis. 5 year economic evaluation. The Journal of rheumatology. 2020.
186. Best JH, Vlad SC, Pei J. Comparative Cost per Response for 4 Clinical Endpoints with Tocilizumab Monotherapy vs Adalimumab Monotherapy in a Head-to-Head Randomized Double-Blind Superiority Trial (ADACTA) in Patients with Rheumatoid Arthritis. Rheumatology & Therapy. 2020;7(1):165-71.
187. Muszbek N, Proudfoot C, Fournier M, Chen CI, Kuznik A, Kiss Z, et al. Economic Evaluation of Sarilumab in the Treatment of Adult Patients with Moderately-to-Severely Active Rheumatoid Arthritis Who Have an Inadequate Response to Conventional Synthetic Disease-Modifying Antirheumatic Drugs. Advances in Therapy. 2019;36(6):1337-57.
