# Supplementary material for: A systematic literature review informing the consensus statement on efficacy and safety of pharmacological treatment with interleukin-6 pathway inhibition with biological DMARDs in immune-mediated inflammatory diseases
Source: RMD Open. 2022 Sep 7;8(2):e002359. doi: 10.1136/rmdopen-2022-002359 (PMC9462104; doi:10.1136/rmdopen-2022-002359)
Supplement: Supplementary data [file rmdopen-2022-002359supp002.pdf]

A systematic literature review informing the consensus statement on efficacy and safety of pharmacological treatment with interleukin-6 pathway inhibition with biological DMARDs

Online Supplementary appendix

Section 1: Research questions, PICOs and Search strategy

Table of contents

Section 1: Research questions, PICOs and Search strategy 4

1.1 Research questions 4

1.1.1 Research questions: Efficacy for approved indications 4

1.1.2 Research questions: Safety for approved indications 5

1.1.3 Research questions: Efficacy and safety for other studies diseases 5

1.1.4 Research questions: Adherence, patient preference and economic aspects 6

1.2 Patients, Interventions, Control, Outcomes (PICOs) 7

1.2.1 Definitions 7

1.2.1.1 Populations and efficacy measures for approved indications 7

1.2.1.1.1 Rheumatoid Arthritis (RA) 7

1.2.1.1.2 Systemic juvenile idiopathic arthritis (sJIA) 7

1.2.1.1.3 Polyarticular-course juvenile idiopathic arthritis (pcJIA) 7

1.2.1.1.4 Adult-onset Still's disease (AoSD) 8

1.2.1.1.5 Giant cell arteritis (GCA) 8

1.2.1.1.6 Takayasu arteritis (TAK) 8

1.2.1.1.7 Multicentric Castleman's disease (MCD) 8

1.2.1.1.8 CAR-T cell induced Cytokine Release Syndrome (CRS) 8

1.2.1.1.9 Neuromyelitis optica spectrum disorders (NMOSD) 9

1.2.1.2 Populations and efficacy measures for other studied diseases 9

1.2.1.2.1 Psoriatic arthritis (PsA) 9

1.2.1.2.2 Axial spondyloarthritis (axSpA) 9

1.2.1.2.3 Osteoarthritis (OA) 9

1.2.1.2.4 Polymyalgia rheumatica (PMR) 10

1.2.1.2.5 ANCA-associated vasculitis (GPA, MPA) 10

1.2.1.2.6 Remitting seronegative symmetric synovitis with pitting edema (RS3PE) 10

1.2.1.2.7 Systemic sclerosis associated interstitial lung disease (SSc-ILD) 10

1.2.1.2.8 Idiopathic inflammatory myopathies (IIM) 11

1.2.1.2.9 Systemic lupus erythematosus (SLE) 11

1.2.1.2.10 Primary Sjögren's syndrome (pSS) 11

1.2.1.2.11 Amyloid A (AA)-Amyloidosis (AAA) 11

1.2.1.2.12 Multiple myeloma (MM) 11

1.2.1.2.13 Refractory relapsing polychondritis 12

1.2.1.2.14 Cytokine release syndrome CRS (associated with severe SARS-CoV-2 infection, Macrophage activating syndrome) 12

1.2.1.2.15 Tumor necrosis factor receptor-associated periodic fever syndrome (TRAPS) 12

1.2.1.2.16 Chronic infantile neurological cutaneous and articular syndrome (CINCA) 12

1.2.1.2.17 Late antibody-mediated kidney transplant rejection (ABMR) ... 12
1.2.1.3 Safety measures ... 13
1.2.1.4 Interventions: Compounds targeting IL-6 receptor or ligand ... 14
1.2.1.4.1 Interleukin-6-receptor (IL-6R)-inhibitors ... 14
1.2.1.4.2 Interleukin-6-ligand (IL-6L)-inhibitors ... 14
1.2.1.5 Controls ... 14
1.3 Search strategy ... 15
1.3.1 Efficacy: approved indications ... 15
1.3.1.1 Medline ... 15
1.3.1.2 EMBASE ... 27
1.3.1.3 Cochrane CENTRAL ... 75
1.3.2 Efficacy: other studied diseases ... 87
1.3.2.1 Medline ... 87
1.3.2.2 EMBASE ... 102
1.3.2.3 Cochrane CENTRAL ... 140
1.3.3 Safety: approved indications ... 154
1.3.3.1 Medline ... 154
1.3.3.2 EMBASE ... 183
1.3.3.3 Cochrane CENTRAL ... 236
1.3.4 Safety: other studied diseases ... 265
1.3.4.1 Medline ... 265
1.3.4.2 EMBASE ... 297
1.3.4.3 Cochrane CENTRAL ... 354
1.3.5 Patient adherence and economic aspects ... 386
1.3.5.1 Medline ... 386
1.3.5.2 EMBASE ... 402
1.3.5.3 Cochrane CENTRAL ... 443

Section 1: Research questions, PICOs and Search strategy

1.1 Research questions

1.1.1 Research questions: Efficacy for approved indications

#	Research question	Population	Intervention	Control	Outcome
1	What is the evidence on efficacy of IL-6 receptor/ligand inhibition with bDMARDs for approved indications such as RA, sJIA, pcJIA, AoSD, GCA, Castleman's disease, CAR-T cell induced CRS and NMOSD (trials; update from 2012 onwards & include data from before)?	See 1.6.1.1.1-1.6.1.1.8	See 1.6.1.4	See 1.6.1.5	See 1.6.1.1.1-1.6.1.1.8

1.1.2 Research questions: Safety for approved indications

#	Research question	Population	Intervention	Control	Outcome
1	What are the safety aspects of IL-6 receptor/ligand inhibition with bDMARDs (trials & registries; update from 2012 onwards & include data from before)?	See 1.6.1.1.1-1.6.1.1.8	See 1.6.1.4	See 1.6.1.5	See 1.6.1.3

1.1.3 Research questions: Efficacy and safety for other studies diseases

#	Research question	Population	Intervention	Control	Outcome
1	What is the evidence for efficacy as well as safety of IL-6 receptor/ligand inhibition with bDMARDs regarding the following studied diseases without approval?<br>• PsoA, axSpA, OA, PMR, large vessel vasculitis, ANCA-associated vasculitis, RS3PE, SSc-ILD, IIM, SLE, pSS, AA-Amyloidosis, multiple myeloma, refractory relapsing polychondritis, CRS associated with severe SARS-CoV-2 infection or macrophage activating syndrome, TRAPS, CINCA and ABMR	See 1.6.1.2.1-1.6.1.2.18	See 1.6.1.4	See 1.6.1.5	See 1.6.1.2.1-1.6.1.2.18

1.1.4 Research questions: Adherence, patient preference and economic aspects

#	Research question	Population	Intervention	Control	Outcome
1	What is the data on adherence issues, patient preferences and economic aspects in patients with RA, sJIA, pcJIA and GCA receiving IL-6 receptor/ligand inhibition with bDMARDs?	See 1.6.1.1.1-1.6.1.1.3; 1.6.1.1.5	See 1.6.1.4	See 1.6.1.5	cost of therapy, utility, patient preferences, adherence, persistence, drug survival, treatment survival, maintenance, dose / interval adjustment towards target and after reaching the target

1.2 Patients, Interventions, Control, Outcomes (PICOs)

1.2.1 Definitions

1.2.1.1 Populations and efficacy measures for approved indications

1.2.1.1.1 Rheumatoid Arthritis (RA)

- Core set variables: SJC, TJC, Pain, Patient global assessment, Physician global assessment, HAQ, CRP, ESR
- Composite measures: ACR 20/50/70, DAS28-CRP, DAS28-ESR, CDAI, SDAI, EULAR responses, ACR/EULAR remission
- Patient reported outcomes: SF-36, FACIT-F, Ability of work, participation, QoL
- Structural damage: Sharp score (including modifications), number of patients achieving radiographic non-progression (as defined in individual studies)

1.2.1.1.2 Systemic juvenile idiopathic arthritis (sJIA)

- Core set variables: JIA-core response variables (JIA-CRV) including number of joints with active joints, number of joints with limitation of movement (LOM) plus pain on motion and/or tenderness with palpation, Patient/Parent global assessment of overall well-being, Physician global assessment, CHAQ and ESR; CRP, JIA-flare, height velocity, survival
- Composite measures: ACR Pediatric Criteria (ACR Pedi 30/50/70/90). ACR Pedi 30 defined as at least 30% improvement from baseline in three of any six variables in core set, while no more than one of remaining variables can worsen by >30%, Juvenile Arthritis Disease Activity Score (JADAS: Physician global assessment, Parent/patient global assessment of well-being, active joint count 0-71/0-27/0-10, ESR), Wallace criteria (≥6 months without joint affection, systemic features, or active uveitis, normal ESR and CRP, Physician global indicating inactive disease)

1.2.1.1.3 Polyarticular-course juvenile idiopathic arthritis (pcJIA)

- Core set variables: active joints, joints with LOM, Patient/Parent global assessment, Physician global, CHAQ, CRP, ESR
- Composite measures: JIA-ACR30/50/70/90
- Radiographic outcomes: Larsen score, van der Heijde Score, Poznanski score

1.2.1.1.4 Adult-onset Still's disease (AoSD)

- Core set variables: SJC, TJC, Pain, Patient global assessment, Physician global assessment, HAQ, CRP, ESR
- Systemic features: fever, skin rash, lymphadenopathy, hepatosplenomegaly, serositis
- Composite measures: ACR 20/50/70, systemic feature score (SFS)

1.2.1.1.5 Giant cell arteritis (GCA)

- Core set parameters: GCA-related signs and symptoms, Patient global assessment, Physician global assessment, Quality of life, sustained glucocorticoid-free remission, relapse, recurrence, cumulative median prednisone dose, serious adverse events, GC-related adverse effects, GC toxicity index, ESR, CRP, Hemoglobin
- Radiographic outcomes: Sonographic evidence of arteritis (Ultrasound), Large vessel involvement: peripheral pulses, blood pressure, Dilatation/aneurysm (US/MR/CT), Inflammatory wall thickening (US/MR/CT), Stenosis (US/MR/CT), PET

1.2.1.1.6 Takayasu arteritis (TAK)

- Core set parameters: Related signs and symptoms, Patient global assessment, Physician global assessment, Quality of life, sustained glucocorticoid-free remission, relapse, recurrence, cumulative median prednisone dose, serious adverse events, GC-related adverse effects, GC toxicity index, ESR, CRP, Hemoglobin
- Radiographic outcomes: Sonographic evidence of arteritis (Ultrasound), Large vessel involvement: peripheral pulses, blood pressure, Dilatation/aneurysm (US/MR/CT), Inflammatory wall thickening (US/MR/CT), Stenosis (US/MR/CT), PET

1.2.1.1.7 Multicentric Castleman's disease (MCD)

- Outcome measures: VAS fatigue, CRP, SSA, Hb, Albumin, IgG, total cholesterol
- Radiographic outcomes: size changes of index lesions/swollen lymph nodes

1.2.1.1.8 CAR-T cell induced Cytokine Release Syndrome (CRS)

- Outcome measures: PaO_2/FiO_2 ratio, fever, hospital-related mortality, overall survival and clinical improvement (including changes in oxygen-support requirements), time to hospital discharge, mechanical ventilation-free survival, time to fever, time to fever resolution, incidence of CRP normalization, changes in IL-6 levels, ferritin, D-Dimer, ALT, LDH, Creatinine, INR, lactate, platelets count, major organ impairment (cardiac, respiratory, gastrointestinal, hepatic, renal, dermatologic, coagulopathy/DIC, neurologic)

1.2.1.1.9 Neuromyelitis optica spectrum disorders (NMOSD)

- Outcome measures: relapse rate, expanded disability status scale (EDSS), intrathecal IL-6 level
- Imaging: gadolinium (Gd)- enhancing T1 lesions

1.2.1.2 Populations and efficacy measures for other studied diseases

1.2.1.2.1 Psoriatic arthritis (PsA)

- Core set variables: SJC, TJC, Pain, Patient global assessment, Physician global, HAQ, CRP, ESR, Enthesitis (MASES, LEI), Dactylitis, PASI
- Composite measures: ACR 20/50/70, DAPSA, PASDAS, PsARC, MDA
- Structural damage: PsA modified Sharp van der Heijde Score, No. of patients achieving radiographic non-progression (as defined in individual studies)

1.2.1.2.2 Axial spondyloarthritis (axSpA)

- Core set variables: Patient global assessment, back pain, nocturnal pain, duration of morning stiffness, fatigue, CRP Enthesitis (MASES), SJC, Spinal mobility (Chest expansion, lateral spinal flexion, modified Schober, occiput to wall distance, cervical rotation), BASDAI, BASFI, BASMI
- Composite measures: ASAS 20/40, ASAS 5/6, ASDAS(CRP)
- Patient reported outcomes: SF-36, FACIT-F, Work, Participation, QoL
- Inflammation: SPARCC MRI Index of the SI Joints, Berlin modification of ASspiMRI
- Structural damage: mSASSS. mNY SI joints

1.2.1.2.3 Osteoarthritis (OA)

- Outcome measures: Patient global assessment, Physician global, VAS pain, SJ, TJ, morning stiffness duration, Dreiser's algofunctional index and Functional Cochin hand index, HAQ, Functional Index for Hand Osteoarthritis (FIHOA)
- Structural damage: Kellgren-Lawrence-Score

1.2.1.2.4 Polymyalgia rheumatica (PMR)

- Core set variables: Patients pain, Morning stiffness, Patient global assessment, Physician global assessment, concomitant prednisolone doses, cumulative glucocorticoid doses, glucocorticoid-free remission rate, relapse rate, HAQ, ESR, CRP
- Patient reported outcomes: SF-36, FACIT-F, Ability of work, participation, QoL
- Composite measures: PMR-AS (CRP, VAS pain, VAS physician, MST, ability to elevate upper limbs EUL)

  Others:
  - Ultrasonographic monitoring of response
  - PET
  - MRI

1.2.1.2.5 ANCA-associated vasculitis (GPA, MPA)

- Core set parameters: related signs and symptoms, Quality of life SF-36, relapse, recurrence, serious adverse events, GC remission, cumulative prednisone dose GC-related adverse effects, GC toxicity index, CRP, IL-6 serum levels, MPO-ANCA level, serum creatinine
- Composite measures: Birmingham vasculitis activity score (BVAS), Vasculitis damage index (VDI)
- Patient reported outcomes: SF-36, FACIT-F
- Radiographic outcomes: CT, PET

1.2.1.2.6 Remitting seronegative symmetric synovitis with pitting edema (RS3PE)

- Outcome measures: pain, morning stiffness, swelling/edema, CRP, SAA
- Radiographic outcomes: US, MRI

1.2.1.2.7 Systemic sclerosis associated interstitial lung disease (SSc-ILD)

- Outcome measures: predicted forced vital capacity (% pFVC), Patient global assessment, Physician global assessment, HAQ
- Patient reported outcomes: SF-36, FACIT-F
- Radiographic outcomes: computer-based score for quantitative lung fibrosis in the zone of maximum fibrosis (QLF-ZM) from hrCT scans

1.2.1.2.8 Idiopathic inflammatory myopathies (IIM)

- Core set variables: core set measures (CSM) including Patient global assessment, Physician global assessment, HAQ, MMT-8 and Extra-muscular Disease Activity score and muscle enzyme (CK/Aldolase, lactate dehydrogenase, aspartate aminotransferase/alanine aminotransferase); Disease Activity score, Myositis Damage Index, Cutaneous Dermatomyositis Disease Area and Severity Index (CDASI),
- Composite measures: ACR/EULAR Criteria for Minimal, Moderate, and Major Clinical Response in Adult Dermatomyositis and Polymyositis and Juvenile Dermatomyositis, definition of improvement (DOI) by International Myositis Assessment and Clinical Studies Group (IMACS) of a ≥ 20% improvement in 3 of 6 CSM, with no more than 2 worsening by ≥ 25%

1.2.1.2.9 Systemic lupus erythematosus (SLE)

- Core set variables: serologic markers of SLE (anti-dsDNA antibodies, anti-cardiolipin, ANA, ENA and quantitative Igs (IgG/IgA/IgM); complement C3/C4, CRP, ESR, fibrinogen, ferritin, swollen joint count, IFN gene signature, prednisone dose, physician global assessment
- Composite measures: BILAG, BICLA, LLDAS, CLASI, SRI-4/5/6, SLEDAI
- Patient reported outcomes: SF-36, FACIT-F, QoL, Participation, Work

1.2.1.2.10 Primary Sjögren’s syndrome (pSS)

- Core set variables: related signs and symptoms: pain, fatigue, dryness, ocular symptoms, oral symptoms, ocular signs (Shirmer test, Rose bengal score), swollen joints, tender joints, Histopathologic features (focus score >1), HAQ
- Composite measures: ESSDAI, ESSPRI

1.2.1.2.11 Amyloid A (AA)-Amyloidosis (AAA)

- Outcome measures: changes in serum amyloid A (SSA), regression of amyloid A protein deposits, changes in estimated glomerular filtration rate (eGFR)

1.2.1.2.12 Multiple myeloma (MM)

- Outcome measures: overall response, complete response, partial response using European Group for Blood and Marrow Transplantation (EBMT) criteria, very good partial response (VGRP) good partial response in MM, progression-free survival, overall survival

1.2.1.2.13 Refractory relapsing polychondritis

- Outcome measures: clinical signs, CRP, MMP-3
- Imaging: laryngoscopy, CT/MR

1.2.1.2.14 Cytokine release syndrome CRS (associated with severe SARS-CoV-2 infection, Macrophage activating syndrome)

- Outcome measures: PaO_2/FiO_2 ratio, fever, hospital-related mortality, overall survival and clinical improvement (including changes in oxygen-support requirements), time to hospital discharge, mechanical ventilation-free survival, time to fever, time to fever resolution, incidence of CRP normalization, changes in IL-6 levels, ferritin, D-Dimer, ALT, LDH, Creatinine, INR, lactate, platelets count, major organ impairment (cardiac, respiratory, gastrointestinal, hepatic, renal, dermatologic, coagulopathy/DIC, neurologic)
- Imaging: CT

1.2.1.2.15 Tumor necrosis factor receptor-associated periodic fever syndrome (TRAPS)

- Outcome measures: time to flare, pain, stiffness, CRP

1.2.1.2.16 Chronic infantile neurological cutaneous and articular syndrome (CINCA)

- Outcome measures: fever, rash intensity, ESR, SSA, CRP

1.2.1.2.17 Late antibody-mediated kidney transplant rejection (ABMR)

- Outcome measures: eGFR decline, biopsy results, DSA-MFI (donor specific antibody-mean fluorescence intensity) and C1q formation, acute rejection; treatment failure (death, graft loss, loss to follow-up or withdrawal from trial), proteinuria

1.2.1.3 Safety measures

- Global: Number of AEs, treatment emergent AEs, serious adverse events, deaths, withdrawals
- Infections: Overall infections, serious infections, opportunistic infections, tuberculosis, herpes zoster, CMV, nontuberculous mycobacteria, Fungal, Hepatitis-B virus reactivation, laboratory indicators of infection beyond CRP/ESR during treatment with IL6(R) inhibiting agents
- Malignancies: lymphoma, non-melanoma skin cancer, solid tumors, other hematological malignancies
- Gastro-intestinal: diverticulitis, gastrointestinal perforations
- Hepatic function: transaminase elevation (>3x ULN/ >5xULN)
- Lipid levels
- Metabolic effects: diabetes, HbA1c levels, Body-Mass-Index (BMI)
- Venous thromboembolic events: DVT, pulmonary embolism
- Hematological abnormalities: anemia, thrombocytopenia (G3/G4), neutropenia (G3/G4), lymphopenia (G3/G4)
- Induction of s-JIA-associated macrophage activation syndrome (MAS)
- Cardiac: Congestive heart failure, MACE (coronary heart disease including fatal and non-fatal MI, stroke), revascularization, incidence of hospitalizations due to heart failure, peripheral artery disease
- Renal function: Serum creatinine, eGFR, corrected eGFR (using patients body surface area), hemoglobin
- Osteoporosis
- Hypersensitivity reactions and anaphylaxis
- Neurological: leukoencephalopathy, CNS demyelinating disorders (ie multiple sclerosis, optic neuritis, acute transverse myelitis), peripheral nervous system disorders (Guillain-Barre syndrome, Miller Fischer syndrome, CIDP, multifocal motor neuropathy with conduction block, mononeuropathy multiplex, axonal sensorimotor polyneuropathies)
- Vaccination and immune response to vaccination
- Pregnancy
- Teratogenicity

1.2.1.4 Interventions: Compounds targeting IL-6 receptor or ligand

1.2.1.4.1 Interleukin-6-receptor (IL-6R)-inhibitors

- Tocilizumab (humanized)
- Sarilumab (human)
- Levilimab (human)
- Satralizumab (humanized)
- Vobarilizumab (humanized, nanobody)

1.2.1.4.2 Interleukin-6-ligand (IL-6L)-inhibitors

- Olokizumab (humanized)
- Clazakizumab (humanized)
- Sirukumab (human)
- Siltuximab (chimeric)
- Ziltivekimab (human)
- PF-04236921 (human)
- Elsilimomab (murine)

1.2.1.5 Controls

- Biologic disease modifying anti-rheumatic drugs (DMARD): Anakinra, Infliximab, Etanercept, Adalimumab, Golimumab, Certolizumab pegol, Rituximab, Ofatumumab, Abatacept, Tabalumab, Pateclizumab, Secukinumab, Ixekizumab, Brodalumab, Guselkumab, Ustekinumab, Mavrilimumab) – all formulations and duration (biosimilars included, if any)
- Conventional synthetic DMARD (including Methotrexate, Leflunomide, Sulfasalazine, Hydroxychloroquine, injectable gold, Chloroquine)
- Interleukin-6 inhibitors in combination with DMARD and/or steroids OR steroid monotherapy
- JAK-Inhibitors
- Placebo (for randomized controlled trials)

1.3 Search strategy

1.3.1 Efficacy: approved indications

1.3.1.1 Medline

1 Arthritis, Rheumatoid/ (99940)

2 ((rheumat* or reumat* or revmat* or rheumo or reumo) adj3 (arthrit* or artrit* or polyarthrit* or polyartrit*)).ti,ot,ab,kw,kf. (115241)

3 (r?eumarthrit* or r?eumartrit* or r?eum arthrit* or r?eum artrit* or r?evmarthrit* or r?evmartrit* or r?evm arthrit* or r?evm artrit* or r?eum polyarthrit* or r?eum polyartrit* or r?eum poly arthrit* or r?eum poly artrit* or r?eumpolyarthrit* or r?eumpolyartrit*).ti,ot,ab,kf,kw. (5)

4 (chronic* adj2 (polyarthrit* or poly arthrit* or polyartrit* or poly artrit*)).ti,ot,ab,kf,kw. (2408)

5 (inflammator* adj2 (arthrit* or artrit*)).ti,ot,ab,kf,kw. (6728)

6 ((r?eum* or r?evm*) adj2 (chronic* adj2 articular*)).ti,ot,ab,kf,kw. (39)

7 ((rheumat* or reumat* or revmat* or rheumo or reumo) adj2 (condition* or diseas*)).ti,ot,ab,kf,kw. (33309)

8 Arthritis, Juvenile/ (10479)

9 ((arthrit* or artrit*) adj6 juvenile*).ti,ab,kf,kw. (10615)

10 JIA.ti,ot,ab,kf,kw. (4049)

11 8 or 9 or 10 (14057)

12 (systemic or polyart* or poly art* or oligoart* or oligo art*).ti,ab,kf,kw,hw. (528939)

13 11 and 12 (4783)

14 ((polyarthr* or polyartrit* or poly arthrit* or poly artrit* or oligoarthrit* or oligoartrit* or oligo arthrit* or oligo artrit*) adj6 juvenile*).ti,ab,kf,kw. (233)

15 (((stills or still's or still) adj disease) and juvenile*).ti,ab,kf,kw. (306)

16 ((stills or still's or still) adj disease adj6 child*).ti,ab,kf,kw. (58)

17 ((systemic adj6 JIA) or sJIA).ti,ab,kf. (852)

18 (((polyart* or oligoart* or poly art* or oligo art) adj6 JIA) or pJIA).ti,ab,kf. (746)

19 Still's Disease, Adult-Onset/ (1371)

20 ((stills or still's or still) adj disease adj6 adult*).ti,ab,kf,kw. (1853)

21 Giant Cell Arteritis/ (6530)

22 (giant cell adj6 (arterit* or aortit* or horton*)).ti,ab,kf. (5139)

23 ((temporal or cranial) adj2 arterit*).ti,ab,kf. (2409)

24 ((horton or horton's or hortons) adj3 disease?).ti,ab,kf. (490)

25 (large vessel adj3 (vasculit* or arterit*)).ti,ab,kf. (1057)

26 GCA.ti,ab,kf. and (arterit* or vasculit* or rheum*).mp,jw. (1834)

27 Takayasu Arteritis/ (4016)

28 (takayasu* adj3 (arterit* or syndrome* or disease*)).ti,ab,kf. (4421)

29 (young female adj6 arterit*).ti,ab,kf. (21)

30 (brachiocephal* adj3 arterit*).ti,ab,kf. (11)

31 Cytokine Release Syndrome/ (416)

32 ((cytokine releas* or cytokine storm) adj3 syndrome*).ti,ab,kf. (1333)

33 crs.ti,ab,kf. and (cytokine releas* or cytokine storm).mp. [mp=title, abstract, original title, name of substance word, subject heading word, floating sub-heading word, keyword heading word, organism supplementary concept word, protocol supplementary concept word, rare disease supplementary concept word, unique identifier, synonyms] (410)

34 Castleman Disease/ (2598)

35 (castleman* adj3 (disease* or tumo?r*)).ti,ab,kf. (3490)

36 ((angiofollicul* or angio follicul*) adj3 lymph* adj3 hyperplas*).ti,ab,kf. (282)

37 (lymph* node adj3 hyperplas* adj3 giant).ti,ab,kf. (213)

38 (castleman* adj3 (disease* or tumo?r* or pseudo-tumo?r* or pseudotumo?r* or lymph* or pseudolymphom* or hyperplas*)).ti,ab,kf,kw. (3590)

39 1 or 2 or 3 or 4 or 5 or 6 or 7 or 13 or 14 or 15 or 16 or 17 or 18 or 19 or 20 or 21 or 22 or 23 or 24 or 25 or 26 or 27 or 28 or 29 or 30 or 31 or 32 or 33 or 34 or 35 or 36 or 37 or 38 (184253)

40 (tocilizumab* or actemra* or atlizumab* or lusinex* or r1569 or r 1569 or roactemra* or mra or 375823 41 9 or i031v2h011* or rhpm-1 or rhpm1 or rg-1569 or rg1569 or msb-11456 or msb11456 or ro-4877533 or ro4877533).mp,rn. (12356)

41 (sarilumab* or kevzara* or regn 88 or regn88 or sar 153191 or sar153191 or 1189541 98 7 or nu90v55f8i*).mp,rn. (166)

42 (levilimab* or BCD-089 or bcd089 or 2035008 70 7 or P7UV3L2H80*).mp,rn. (0)

43 (olokizumab* or cdp 6038 or cdp6038 or 1007223 17 7 or pai71r1d2w*).mp,rn. (17)

44 (clazakizumab* or ald 518 or ald518 or bms 945429 or bms945429 or 1236278 28 6 or 4s38z8ra9o*).mp,rn. (30)

45 (sirukumab* or cnto 136 or cnto136 or pilvensia or 1194585 53 9 or 640443FU93*).mp,rn. (55)

46 (siltuximab* or sylvant or cnto 328 or cnto-328 or cnto328 or cllb8 or ccib8 monoclonal antibody or sylvant or t4h8fma7im* or 541502 14 1).mp,rn. (180)

47 (ziltivekimab* or zilti or "cor-001" or "2226654 05 1").mp,rn. (2)

48 (elsilimomab* or "b-e8" or 468715 71 1).mp,rn. (12)

49 40 or 41 or 42 or 43 or 44 or 45 or 46 or 47 or 48 (12632)

50 39 and 49 (2772)

51 Interleukin 1 Receptor Antagonist Protein/ (5210)

52 (anakinra* or kineret or ((interleukin 1 receptor or il1 receptor or il 1 receptor) adj1 antagonist protein*) or (recombinant interleukin 1 receptor adj1 (antagonist* or blocker* or blocking agent*)) or antril or il 1ra or il1ra or il 1 ra or ((urin* or febrile) adj2 (interleukin 1 inhibitor* or il1 inhibitor* or il 1 inhibitor*)) or "143090 92 0" or 9013duq28k*).mp,rn. (9540)

53 Infliximab/ (10363)

54 (infliximab* or ifx or avakine or flixabi or inflectra or inflecta or ixifi or remicade or remsima or revellex or renflexis or zessly or mab ca2 or monoclonal antibody ca2 or antibody ca2 monoclonal or ca2 monoclonal antibody or ct p13 or ctp13 or ct-p-13 gp-1111 or gp1111 or ta-650 or ta650 or abp 710 or abp710 or bow-015 or bow015 or 170277 31 3 or b72hh48flu* or sb2 or "pf 06438179" or pf 6438179 or pf06438179 or pf6438179 or hsdb 7850 or hsdb7850).mp,rn. (15745)

55 Etanercept/ (5884)

56 (etanercept* or benepali or embrel or enbrel or enia 11 or enia11 or erelzi or lifmior or opinercept or recombinant tumo?r necrosis factor receptor fc fusion protein or "tnr 001" or tnr001 or tnfr-fc or tnrfr:fc or rhu tnfr:fc or rhu-tnfr:fc or tnfr-immunoadhesin or tnt receptor fusion protein or tum?or necrosis factor receptor fc fusion protein or tunex or "185243 69 0" or op401g7ojc* or brenzys or tnf receptor fusion protein or tnfr fc or recombinant human tnf or recombinant human dimeric tnf receptor type ii igg fusion protein or chs-0214 or chs0214 or dwp-422 or dwp422 or enia-11 or enia11 or gp-2015 or gp2015 or gp2015c or gp-2015c or hd-203 or hd203 or lbec-0101 or lbec0101 or sb-4 or sb4).mp,rn. (9436)

57 Adalimumab/ (5434)

58 (adalimumab* or humira or abp 501 or abp501 or abtd2e7 or amjevita or amgevita or bax 2923 or bax2923 gp 2017 or gp2017 or halimatoz or hefiya or hulio or hyrimoz or ibi 303 or ibi303 or imraldi or m 923 or m923 or msb 11022 or msb11022 or ons3010 or ons 3010 or "pf 06410293" or pf 6410293 or pf06410293 or pf6410293 or d2e7 or truxeda or cyltezo or solymbic or fys6t7f842* or 331731 18 1 or lu 200134 or lu200134 or d2e7 or hsdb 7851 or hsdb7851 or abp-501 or abp501 or bcd-057 or bcd057 or bi-695501 or bi695501 or chs-1420 or chs1420 or gp-2017 or gp2017 or sb-5 or sb5).mp,rn. (9029)

59 (golimumab* or cnto 148 or cnto148 or simponi or 476181 74 5 or 91x1klu43e*).mp,rn. (1298)

60 Certolizumab Pegol/ (611)

61 (certolizumab* or cdp 870 or cdp870 or cimzia or pegylated tumor necrosis factor alpha antibody fab fragment or pegylated tumour necrosis factor alpha antibody fab fragment or pha 738144 or pha738144 or czp or 428863 50 7 or g6adw90r16* or umd07x179e* or hsdb 7848 or hsdb7848).mp,rn. (1555)

62 Rituximab/ (14951)

63 (rituximab* or ct p10 or ctp10 or idec 102 or idec102 or idecc2b8 or idec c2b8 or monoclonal antibody idec c2b8 or gp2013 or gp 2013 or "pf 05280586" or pf05280586 or mabthera or mab thera or r 105 or r105 or reditux or rg 105 or rg105 or rituxan or rituxin or ritemvia or rituzena or rixathon or riximyo or ro 452294 or ro452294 or truxima or 174722 31 7 or 4f4x42syq6* or hsdb 7455 or hsdb7455).mp,rn. (24654)

64 (ofatumumab* or humax cd20 or humax-cd20 or humaxcd20 or humax-cd20-2f2 or arzerra or gsk 1841157 or gsk1841157 or gski841157 or humac cd20 or omb 157 or omb157 or 679818 59 8 or m95kg522r0* or hsdb 8170 or hsdb8170).mp,rn. (585)

65 Abatacept/ (2933)

66 (abatacept* or ctla4 ig or ctla4 immunglobulin or ctla4 immunoglobulin g or ctla4ig or 'ctla 4 ig' or ctla-igg4m or ctla4-fc or bms 188667 or bms188667 or orencia or '332348 12 6' or 7d0yb67s97* or rg 2077 or rg2077 or rg-1046 or rg1046 or nulojix or lea29y or lea 29y or ctl4 fc or ctl4fc or belatacept or bms 224818 or bms224818 or belatacept or cytotoxic t lymphocyte associat* antigen 4).mp,rn. (5401)

67 (tabalumab* or ly 2127399 or ly2127399 or 1143503 67 6 or pqp8vh3mjw*).mp,rn. (58)

68 (pateclizumab* or mlta 3698a or mlta3698a or pro 283698 or pro283698 or 12202526 59 7 or qok1yyh7j2* or rg 7415 or rg7415).mp,rn. (6)

69 (secukinumab* or cosentyx or ain 457 or ain457 or 1229022 83 6 or dlg4eml025*).mp,rn. (1207)

70 (ixekizumab* or ly 2439821 or ly2439821 or taltz or 1329632 62 3 or 1143503 69 8 or bty153760o*).mp,rn. (597)

71 (brodalumab* or amg 827 or amg827 or khk 4827 or khk4827 or siliq or kyntheum or 1174395 19 7 or 6za31y954z*).mp,rn. (346)

72 (guselkumab* or cnto 1959 or cnto1959 or 1350289 85 8 or tremfya or 1350289 85 8 or 089658a12d*).mp,rn. (262)

73 Ustekinumab/ (1093)

74 (ustekinumab* or cnto 1275 or cnto1275 or stelara or "815610 63 0" or fu77b4u5z0* or 1275 cnto or 1275cnto or l04ac05 or tt-20 or tt20).mp,rn. (2159)

75 (mavrilimumab or "1085337 57 0" or 1158jdp9a* or cam 3001 or cam3001).mp,rn. (32)

76 exp Antirheumatic Agents/ (429922)

77 (dmard* or csdmard* or cs-dmard* or sdmard* or s-dmard* or tsdmard* or ts-dmard* or bdmard* or b-dmard*).ti,ab,kf. (5744)

78 (disease modifying adj3 (antirheum* or anti rheum*) adj3 (drug? or agent?)).ti,ab,kf. (6391)

79 ((antirheum* or anti rheum*) adj5 (drug? or agent?)).ti,ab,kf. (8996)

80 Methotrexate/ (38138)

81 (mxt or methotrexat* or metotrexat* or methoblastin or methylaminopterin* or amet?opterin* or abitrexate or antifolan or brimexate or a-met?opterin* or a-methpterin* or ai3-25299 or ai325299 or alpha-methopterin or biotrexate or emtexate or emtrexate* or emthexat* or enthexate* or farmitrexat* or farmotrex or fauldexato or folex or folex pfs or hdmtx or ifamet or imeth or maxtrex or metotressat* or methotrate* or methohexate* or mexate* or metrotex or metatrexato* or metex or metrex or metatrexan or maxtrex or meticil or metoject or metotrexin or novatrex* or neotrexate* or nordimet or lumexon or ledertrexate* or lanterel or rasuvo or r?eumatrex* or reditrex or rasuvo or texate or texate-t or tremetex or trexeron or trixilem or texorate or trexall or xaken or xatmep or otrexup or mpi-2505 or mpi2505 or mpi 5004 or mpi5004 or amethopterin* or 133073-73-1 or 15475-56-6 or 3ig1e710zn* or yl5fz2y5u1* or cl 14377 or cl14377 or ccris 1109 or ccris1109 or emt 25299 or emt25299 or nsc-740 or nsc740 or r-9985 or r9885 or x-133 or wr-19039 or wr19039).mp,rn. (55414)

82 Leflunomide/ (1528)

83 (leflunomid* or hwa 486 or hwa486 or su101 or su 101 or arava or arabloc or hsdb 7289 or hsdb7289 or g162gk9u4w* or 75706-12-6 or rs 34821 or rs34821).mp,rn. (2601)

84 lef.ti,ab,kf. (2538)

85 Sulfasalazine/ (4132)

86 (sulfasalazin* or sasp or ssz or salicylazosulfapyridin* or salicyl azo sulfapyridin* or sulphasalazin* or salazosulfapyridin* or salazosulfapiridin* or 'pyralin en' or azulfadin* or azulfidin* or asulfidin* or azulfide* or azulfin or colo-pleon or colopleon or disalazin or gastropyrin or pleon or "pleon ra" or "pyralin en" or rorasul or rosulfant or ulcol or ucine or salazopyrin* or ratio-sulfosalazin* or sulfasalazin* or 3xc8guz6cb* or accucol or asulfidin* or azopyrin* or azosulfidin* or benzosulfa or "brn 0356241" or brn0356241 or ccris 4713 or ccris4713 or einecs 209-974-3 or hsdb 3395 or hsdb3395 or nsc 203730 or nsc203730 or nsc 667219 or nsc667219 or reupirin or rorasul or salazo-sulfapyridin* or salazosulfpyridin* or salazopiridazin* or salazopyridin* or salazopirin* or salazodin or salisulf or salipyr or saridine or si-88 or si88 or sulculon or sulfasalazin* or sulfasalizin* or sulphasalazin* or w-t sasp oral or 599-79-1 or "s.a.s. 500" or "s.a.s.-500" or sas-500 or zopyrin*).mp,rn. (8002)

87 Hydroxychloroquine/ (3826)

88 (hydroxychloroquin* or hcq or 747-3-4 or 8q2869cnvh* or 118-42-3 or 4qwg6n8qkh* or hydroxychlorochin* or hydrochloroquin* or hydrocloroquin* or oxychlorochin or oxychloroquin* or plaquenil or plaquinol or "brn 0253894" or brn0253894 or einecs 204-249-8 or einecs204-249-8 or hidroxicloroquin* or idrossiclorochin* or oxichlorochin* or oxichloroquin* or oxiklorin or win1258 or win 1258 or z0188 or einecs 212-019-3 or einecs212-019-3 or chloroquinol or dimard or ercoquin or evoquin or erquin or geniquin or quensyl or sn 8137 or sn8137 or toremonil or yuma).mp,rn. (7309)

89 Gold Sodium Thiomalate/ (1262)

90 (gold sodium thiomalate* or aurothiomalate* or aurolate* or gold thiomalate* or gold disodium thiomalate* or gold thiomalic acid* or miocrin or miocrisin or monogold disodium thiomalate* or myochrysin* or myocrisin* or myocrysin* or sodium aurothiomalate* or sodium gold thiomalate* or sodium thiomalate* gold or sodium thiomalatoaurate* or tauredon or taureodon or tauredone or thiomalate gold or thiomalatoaurate sodium or 1244-57-4 or 39377-38-3 or 4846-27-9 or 74916-57-7 or e4768zy6gm* or aurothiomala-natrium or aurot?iomalato sodico or monogold* sodium salt or dinatrium 2-aurothio-succinat or einecs 235-479-7 or einecs235-479-7 or hsdb 7173 or hsdb7173 or kidon or natrii aurothiomalas* or shiosol or gold mercatpsuccinate*).mp,rn. (1545)

91 Aurothioglucose/ (834)

92 (thioglucosoaurate* or gold thioglucose* or thioglucose gold or aureotan or solganal or solganol or gold-50 or gold50 or solganal b or b oleosum solganal or auromyose* or aurothioglucose* or aurotan or aurumine or authron or brenol or ccris 59 or ccris59 or einecs 235-365-7 or einecs235-365-7 or glysanol b or goldthioglucose* or hsdb 7174 or hsdb7174 or oronol or romosol or skf 10056 or skf10056 or 2p2v9q0e78* or 12192-57-3).mp,rn. (1132)

93 (thioglucosoaurate* or gold thioglucose* or gold thio glucose* or thioglucose gold or aureotan or solganal or solganol or gold-50 or gold50 or solganal b or b oleosum solganal or auromyose* or aurothioglucose* or auro thioglucose* or aurotan or aurumine or aurumin or authron or brenol or ccris 59 or ccris59 or einecs 235-365-7 or einecs235-365-7 or glysanol b or goldthioglucose* or goldtioglucose* or hsdb 7174 or hsdb7174 or oronol or romosol or skf 10056 or skf10056 or 2p2v9q0e78* or 12192-57-3).mp,rn. (1133)

94 ((gold* or auro*) adj10 (inject* or intramuscular* or im or intra muscular*)).ti,ab,kf,kw. (2752)

95 (aurothiopropanol* or allochrysine or allocrisine or aurotiopoe or aurotioprol or 27279-43-2 or g7097j63e9* or auromercapto propanol sulfonate sodium or auromercaptopropanolsulfonate sodium or aurothioisopropanolsulfonate sodium or gold sodium thiopropanol sulfonate or sodium auromercaptopropanol sulfonate).mp,rn. (91)

96 Organogold Compounds/ (934)

97 (aurothiopropanol* or allochrysine or allocrisine or aurotiopoe or aurotioprol or 27279-43-2 or g7097j63e9* or auromercapto propanol sulfonate sodium or auromercaptopropanolsulfonate sodium or aurothioisopropanolsulfonate sodium or sodium aurothiopropanolsulfonate or gold sodium thiopropanol sulfonate or sodium auromercaptopropanol sulfonate).mp,rn. (91)

98 Chloroquine/ (14521)

99 (chloroquin* or cq or 54-04-7 or 886u3h6uff* or aralen or aralene or arechin or arechine or arequin or chingamin or chlorochin* or khingamin or nivaquine or oe48649kh6n* or amokin or amokine or anaclor or aralan or arequine or artriquine or avloclor or avoclor or arthrochin or artrichin or bemaco or bemaphata or bemaphate or bemasulph or benaquin or bipiquin or cadiquin or "brn 0482809" or brn0482809 or capquin or ccris 3439 or ccris3439 or

chemochin or chemochine or chingamine or chingaminum or chloraquin* or chlorquin or chlorquine or choloquine or choroquine sulfate or choroquine sulphate or cindacin or clo-kit junior or clorichina or clorichine or cloriquine or clorochina or delagil or delagyl or diclokin or chlorofoz or cindachin or clorochina or cloroquina or cocatrit or dichinalex or diquinalex or diroquine or emquin or genocin or gontochin or gontochine or gontoquine or einecs 200-191-2 or einecs200-191-2 or elestol or gontochin or heliopar or hsdb 3029 or hsdb3029 or imagon or iroquine or klorokin or klorokine or klorokinfosfat or lagaquin or lapaquin or malaquin or malaren or malarex or malarivon or malaviron or maliaquine or maquine or mesylith or mexaquin or mirquin or neochin or nivaquin or nivachine or nivaquine b or nivaquine dp or nivaquine forte or "p roquine" or nsc 187208 or nsc187208 or pfizerquine or quensyl or quinachlor or quinercyl or quingamine or quinilon or quinoline or quinoscan or repal or resochen or resochene or resochin or resochin junior or resochina or resochine or resochinon or resoquina or resoquine or reumachlor or ronaquine or roquine or rp 3377 or rp3377 or sanoquin or sanoquine or silbesan or siragan or sirajan or sn 7618 or sn7818 or solprina or solprine or sopaquin or st 21 or tresochin or tresoquine or trochin or trochine or troquine or weimerquin or w 7618 or w7618 or win 244 or win244).mp,rn. (34786)

100 exp Adrenal Cortex Hormones/ (398367)

101 exp Prednisolone/ (51327)

102 Prednisone/ (39543)

103 Cortisone/ (19606)

104 Hydrocortisone/ (72277)

105 exp Steroids/ (859975)

106 adrenal cortex hormones.ti,ab,kf,rn. (65185)

107 prednisolone*.mp,rn. (46453)

108 methylprednisolone*.mp,rn. (26936)

109 (prednisolone* or predonine or di-adreson-f or diadresonf or 9phq9y1olm* or prednisolona* or prednisolonum* or nsc-9120 or nsc9120 or nsc 9900 or nsc9900 or delta-dehydrocortisol* or delta-dehydrocortisone* or delta-hydrocortisone or hydroretrocortine or metacortandralone* or brn 1354103 or brn1354103 or bubbli-pred or ccris 980 or ccris980 or co-hydeltra or codelcortone or cordrol or cortalone or cotogesic or cotolone or decaprednil or decortin h or delcortol or dehydrohydrocortisone* or delta-cortef or deltacortef or delta-ef-cortelan or delta-stab or deltacortenol or deltacortril or deltahydrocortisone* or deltisilone or derpo pd or dexa-dortidelt hostacortin h or di-adreson f or dicortol or donisolone or dydeltrone or eazolin d or ec 200-021-7 or ec200-021-7 or einecs 200-021-7 or einecs200-021-7 or erbacort or erbasona or estilsona or fernisolone or fernisolone p or hostacortin h or hsdb 3385 or hsdb3385 or hydeltra

or hydeltrone or hydrodeltalone or hydrodeltisone or hydroretrocortin or hydroretrocortine* or k 1557 or k1557 or lentosone or meti-drm or meticortelone or orapred or orapred odt or paracortol or paracotol or pediapred or precortancyl or precortilon or precortisyl or predne-dome or prednelan or prednicen or predniliderm or predniretard or prednis or predonin or predonine* or prelon or prelone or prenolone or rolisone or scherisolon? or solone or sterolone or ulacort or ultracorten h or ultracortene h adelcort or antisolon or antisolone or aprednisolon? or benisolon? or caberdelta or co hydeltra or codelcortone or ompresolon).mp,rn. (46541)

110 (cortadeltona or cortelinter or cortisolone or dacortin or dacortin h or dacrotin or decaprednil or decortil or delta cortril or delta hycortol or deltacortenolo or deltacortil or deltacortoil or deltaderm or deltadglycortril or deltahycortol or deltahydrocortison? or deltaophticor or deltasolone or deltastab or deltidrosol or deltisilone or deltisolon? or deltolasson? or deltosona or deltosone or depo-predate or dermosolon or dhasolone or di adreson? f or diadreson? f or dicortol or domucortone or encortelon? or encortolon or equisolon or glistelone or hefasolon or hydrelta or hydrocortancyl or hydrocortidelt or hydradeltalone or hydradeltisone or inflanefran or insolone or keteocort h or key-pred or key-pred sp or lenisolone or leocortol or liquipred or mediasolone or meprisolon? or metacortalon? or metacortelone or meti derm or metiderm or meticortelone or morlone or mydrapred or nisolon? or opredsone or panafcortelone or panafcortolone or panafort or paracortol pr phlogex or pre cortisyl or preconin or precortalon or precortancyl or precortisyl or pred ject 50 or predacort 50 or predaject 50 or predalone 50 or predartrina or predartrine or predate 50 or predeltilone or predisole or predisyr or predne dome or prednecort or prednedome or prednelan or predni coelin or predni h tablinen or predni helvacort or prednicoelin or prednicort or prednicortelone or prednifor drops or predniment or prednorsolon? or predorgasolon? or prenilone or preventan or prezolon or rubycort or serilone or solonda or solupren? or spiricort or spolotane or supercorti#ol or taracortelone or walesolone or wysolone).mp,rn. (63)

111 (methylprednisolone * or methylprednisolona* or methylprednisolonum* or metipred or urbason or medrol or x4w7zr7023* or 83-43-2 or besonia or brn 23403300 or brn2340300 or a-methapred or artisone-wyeth or besonia or depo-medrol or dopomedrol ec 201-4764 pr ec2014764 or einecs 201-476-4 or einecs201-476-4 or esametone or firmacort or hsdb3127 or hsdb3127 or lemod or medesone or medixon or medlone 21 or medrate or medrol or medrone or mesopren or metastab or methyleneprednisolone* or metilbetasone or metrisone or metrocort or metysolon or moderin or nirypan or noretona or nsc 19987 or nsc19987 or predni n tablinen or prednol-l or promacortine or reactenol or sieropresol or solomet or summicort or suprametil or u 7532 or u7532 or "u 67 590a" or urbason or urbasone or wyacort or adlone 40 or adlone 80 or beta methylprednisolone or medalone 80 or depmedalone or deproject 80 or depopred or esametone or firmacort or med-jec-40 or medixon or mednin or medralone or mepredprednisolone or meprelon or mesopren or methacort 40 or methacort 80 or methyxlcotol or methylcotolone or methylpred dp or methylsterolone or metidrol or metycortin or metypred or metypresol or neomedrone or solu decortin or urbason).mp,rn. (19764)

112 (prednison or prednisone or prednisona or prednisonum or dehydrocortisone or delta cortisone or rectodelt or sterapred or ultracorten or winpred or apo prednisone or cortan or cartancyl or panafcort or cutason or decortin or dacortin or decortisyl or deltasone or encortone or encorton or enkorton or enkortolon or kortancyl or liquid pred or meticorten or orasone or panasol or predni tablinen or prednidib or predniment or pronisone or vb0r961hzt* or 53-03-2 or adasone or al3-52939 or ancortone or bicortone or cartancyl or ccris 2646 or ccrs2646 or colisone or cortan or cortidelt or cotone or dacorten or

dacortin or decortancyl or dekortin or dellacort or delta cortelan or delta cortisone or delta dome or deltacortene or deltacortisone or deltacortone or deltasone or deltison or deltisona or deltra or di adreson or diadreson or econosone or einecs 200-160-3 or einecs200-160-3 or fernisone or fiasone or hostacortin or hsdb 3168 or hsdb3168 or incocortyl or juvason or lisacort or lodotra or lodtra).mp,rn. (53935)

113 (me-korti or metacortandracin or metacorten or nci c04897 or ncic04897 or nisona or nizon or novoprednisone or nsc 10023 or nsc10023 or nurison or orasone or panafcort or paracort or prarmenison or pehacort or predeltin or prednicen-m or prednicorm or prednicort or prednicot or prednilonga or prednitone or prednizon or prednovister or presone or pronison or rayos or rectodelt or retrocortine or servisone or sterapred or supercortio or u 6020 or u6020 or ultracorten or ultracortene or winpred or wojtab or zenadrid).mp,rn. (353)

114 cortisone?.mp,rn. (23529)

115 corticosteroid*.mp,rn. (105757)

116 corticoid*.mp,rn. (6570)

117 glucocorticoid*.mp,rn. (113752)

118 corticosterone*.mp,rn. (34708)

119 hydrocortisone*.mp,rn. (77716)

120 dexamethasone*.mp,rn. (72603)

121 beclomethasone*.mp,rn. (3844)

122 (triamcinolone* or 1zk20vi6ty* or 124-94-7 or aristocort or volon or fluoxyprednisolon* or tiamcinolonum or triamcinolonum* or triamcinolona* or brn 2341955 or brn2341955 or cl 19823 or cl19823 or einecs 204-718-7 or einecs204-718-7 or hsdb 3194 or hsdb3194 or nsc 13397 or nsc13397).mp,rn. (11925)

123 (steroid? adj10 (therap* or treat* or drug? or inject* or intramuscular* or intra muscular* or im or intravenous* or intra venous* or iv or intraarticular* or intra articular* or ia or administrat* or oral or orally or parenteral*)).ti,ab,kf. (73759)

124 Janus Kinase Inhibitors/ (424)

125 ((jak or jak1 or jak2 or jak3 or janus kinase) adj10 inhibitor*).mp,rn. (6368)

126 (janus adj4 kinase adj10 inhibitor?).mp,rn. (2514)

127 exp Janus Kinases/ai [Antagonists & Inhibitors] (2278)

128 (baricitinib* or 1187594-09-7 or isp4442i3y* or "incb 028050" or incb028050 or ly-3009104 or ly3009104).mp,rn. (420)

129 (tofacitinib* or 477600-75-2 or 87la6fu830* or hsdb 8311 or hsdb8311 or cp-690 550 or cp 690550 or cp690550).mp,rn. (1582)

130 (filgotinib* or 1206161-97-8 or 3xvl385q0m* or "glpg 0634" or glpg0634).mp,rn. (108)

131 (upadacitinib* or 1310726-60-3 or 4RA0KN46E0* or abt 494 or abt494).mp,rn. (133)

132 (ruxolitinib* or rux or 941678-49-5 or 82s8x8xx8h* or hsdb 8259 or hsdb8259 or inc 424 or inc424 or incb424 or "incb 018424" or incb018424 or incb 18424 or incb18424 or r-ruxolitinib* or jakafi or jakavi).mp,rn. (1675)

133 (itacitinib or "incb 039110" or incb039110 or incb 39110 or incb39110 or 1334298-90-6 or 19j3781lpm*).mp,rn. (24)

134 (ag490 or ag 490 or tyrphostin b42 or tyrphostin ag 490 or tyrphostin ag490 or 133550-30-8).mp,rn. (1517)

135 (fedratinib* or tg101348 or tg 101348 or sar302503 or sar 302503 or 936091-26-8 or 6l1xp550i6* or inrebic).mp,rn. (145)

136 (ritlecitinib* or pf-06651600 or pf06651600 or pf 6651600 or pf6651600 or 2140301-97-7 or 132lf5wgh4* or 2192215-81-7 or eag4t1459k* or ritlecitinib malonate or ritlecitinib toslate or ritlecitinib tosylate or ritlecitinib propanedioate).mp,rn. (10)

137 (peficitinib* or peficitinib hydrobromide).mp,rn. (62)

138 or/51-137 (1523761)

139 39 and 138 (55066)

140 50 or 139 (55805)

141 randomized controlled trial.pt. (516561)

142 controlled clinical trial.pt. (93916)

143 randomi*.ti,ab,kf. (658948)

144 placebo*.ti,ab,kf,hw. (233561)

145 exp placebo effect/ (4860)

146 randomly.ti,ab,kf. (345241)

147 exp Randomized Controlled Trials as Topic/ (141058)

148 Controlled Clinical Trials as Topic/ (5547)

149 clinical trial/ or clinical trial, phase ii/ or clinical trial, phase iii/ or clinical trial, phase iv/ (563754)

150 clinical trials as topic/ or clinical trials, phase ii as topic/ or clinical trials, phase iii as topic/ or clinical trials, phase iv as topic/ (208102)

151 trial.ti,ab. (614102)

152 ((singl* or doubl* or trebl* or tripl*) adj3 (blind* or dummy or mask*)).ti,ab,kf. (177364)

153 Single-Blind Method/ (29248)

154 Double-Blind Method/ (160719)

155 Cross-Over Studies/ (48864)

156 (crossover or cross over).ti,ab,kf. (86890)

157 or/141-156 (1838792)

158 140 and 157 (10588)

159 exp animals/ not humans/ (4753760)

160 158 not 159 (10506)

161 limit 160 to english (9274)

162 limit 161 to yr="2012 - 2020" (3543)

163 review.pt. (2714658)

164 162 not 163 (2761)

165 from 164 keep 1-2000 (2000)

166 164 not 165 (761)

1.3.1.2 EMBASE

#194,"#191 OR #193",5116,18 Nov 2020

#193,"#186 NOT #192",4549,18 Nov 2020

#192,"#186 AND ('conference abstract'/it OR 'conference review'/it OR 'conference paper'/it OR 'editorial'/it OR 'letter'/it)",7044,18 Nov 2020

#191,"#186 AND #189 AND [2019-2020]/py",567,18 Nov 2020

#190,"#186 AND #189",3950,18 Nov 2020

#189,"#187 OR #188",50862,18 Nov 2020

#188,"acr:nc AND annual:nc AND meeting:nc AND rheumatology:nc",19451,18 Nov 2020

#187,"'eular':nc AND 'annual european congress of rheumatology':nc",31411,18 Nov 2020

#186,"#180 NOT #185",11593,18 Nov 2020

#185,"#181 OR #182 OR #183 OR #184",948516,18 Nov 2020

#184,"('case control*':ti,ab OR 'case control*':tt) AND (random*:ti,ab OR random*:tt) NOT ('randomi?ed controlled':ti,ab OR 'randomi?ed controlled':tt)",17681,18 Nov 2020

#183,"('systematic review':ti OR 'systematic review':tt) NOT (trial:ti OR trial:tt OR study:ti OR study:tt)",156791,18 Nov 2020

#182,"'we searched':ab AND (review:it OR review:ti OR review:tt)",33801,18 Nov 2020

#181,"'review':ab AND 'review':it NOT (trial:ti OR trial:tt)",828498,18 Nov 2020

#180,"#178 NOT #179",12933,18 Nov 2020

#179,"(mouse:ti OR mice:ti OR murine$:ti OR rat:ti OR rats:ti OR swine:ti OR bovine:ti OR porcine:ti OR pigs:ti OR piglets:ti OR rabbit$:ti OR dog:ti OR dogs:ti OR cat:ti OR cats:ti OR lamb:ti OR lambs:ti OR sheep:ti OR cattle:ti OR monkey:ti OR monkeys:ti OR trout:ti OR marmoset*:ti OR mouse:tt OR

mice:tt OR murine$:tt OR rat:tt OR rats:tt OR swine:tt OR bovine:tt OR porcine:tt OR pigs:tt OR piglets:tt OR rabbit$:tt OR dog:tt OR dogs:tt OR cat:tt OR cats:tt OR lamb:tt OR lambs:tt OR sheep:tt OR cattle:tt OR monkey:tt OR monkeys:tt OR trout:tt OR marmoset*:tt) AND 'animal experiment'/de",1088314,18 Nov 2020

#178,"#174 NOT #177",12943,18 Nov 2020

#177,"#175 NOT #176",5708414,18 Nov 2020

#176,"'human'/exp OR 'human experiment'/exp",22654307,18 Nov 2020

#175,"'animal'/exp OR 'animal experiment'/exp OR 'animal model'/exp",28361407,18 Nov 2020

#174,"#150 AND #171 AND [english]/lim AND [2012-2020]/py",13177,18 Nov 2020

#173,"#150 AND #171 AND [english]/lim",23340,18 Nov 2020

#172,"#150 AND #171",24625,18 Nov 2020

#171,"#151 OR #152 OR #153 OR #154 OR #155 OR #156 OR #157 OR #158 OR #159 OR #160 OR #161 OR #162 OR #163 OR #164 OR #165 OR #166 OR #167 OR #168 OR #169 OR #170",2667932,18 Nov 2020

#170,"(('head to head' NEAR/3 (study OR comparison OR trial)):ti,ab,kw) AND random*:ti,ab,kw,de",1594,18 Nov 2020

#169,"((parallel NEXT/1 group*):ti,ab) OR ((parallel NEXT/1 group*):tt)",26319,18 Nov 2020

#168,"'crossover procedure'/de OR crossover:ti,ab,kw OR 'cross over':ti,ab,kw OR crossover:tt OR 'cross over':tt",119419,18 Nov 2020

#167,"(((singl* OR doubl* OR trebl* OR tripl*) NEAR/3 (blind* OR dumm* OR mask*)):ti,ab,kw) OR (((singl* OR doubl* OR trebl* OR tripl*) NEAR/3 (blind* OR dumm* OR mask*)):tt)",248514,18 Nov 2020

#166,"'single blind procedure'/de OR 'double blind procedure'/de OR 'triple blind procedure'/de",216950,18 Nov 2020

#165,"(controlled NEAR/8 (study OR trial OR design)):tt",60,18 Nov 2020

#164,"(controlled NEAR/8 (study OR trial OR design)):ti,ab,kw",375383,18 Nov 2020

#163,"((clin* NEAR/6 trial):ti,ab) OR ((clin* NEAR/6 trial):tt)",259124,18 Nov 2020

#162,"trial:ti OR trial:tt",319336,18 Nov 2020

#161,"'phase 2':ti,ab,kw OR 'phase 3':ti,ab,kw OR 'phase 4':ti,ab,kw OR 'phase ll':ti,ab,kw OR 'phase lll':ti,ab,kw OR 'phase lv':ti,ab,kw OR 'phase ii':ti,ab,kw OR 'phase iii':ti,ab,kw OR 'phase iv':ti,ab,kw OR 'phase 2':tt OR 'phase 3':tt OR 'phase 4':tt OR 'phase ll':tt OR 'phase lll':tt OR 'phase lv':tt OR 'phase ii':tt OR 'phase iii':tt OR 'phase iv':tt",218201,18 Nov 2020

#160,"'clinical trial'/de OR 'phase 2 clinical trial'/exp OR 'clinical trial, phase 2' OR 'phase 2 clinical study' OR 'phase 2 clinical trial' OR 'phase 2 study' OR 'phase 2 trial' OR 'phase ii clinical study' OR 'phase ii clinical trial' OR 'phase ii study' OR 'phase ii trial' OR 'phase 3 clinical trial'/exp OR 'clinical trial, phase 3' OR 'phase 3 clinical study' OR 'phase 3 clinical trial' OR 'phase 3 study' OR 'phase 3 trial' OR 'phase iii clinical study' OR 'phase iii clinical trial' OR 'phase iii study' OR 'phase iii trial' OR 'phase 4 clinical trial'/exp OR 'clinical trial, phase 4' OR 'phase 4 clinical study' OR 'phase 4 clinical trial' OR 'phase 4 study' OR 'phase 4 trial' OR 'phase iv clinical study' OR 'phase iv clinical trial' OR 'phase iv study' OR 'phase iv trial'",1151189,18 Nov 2020

#159,"'controlled clinical trial (topic)'/exp",197801,18 Nov 2020

#158,"randomly:ti,ab,kw OR randomly:tt",457066,18 Nov 2020

#157,"'placebo effect'/de",6260,18 Nov 2020

#156,"'placebo*':ti,ab,kw OR 'placebo*':tt",316818,18 Nov 2020

#155,"'placebo'/de",364393,18 Nov 2020

#154,"'randomization'/de",88357,18 Nov 2020

#153,"randomi*:ti,ab,kw OR randomi*:tt",947166,18 Nov 2020

#152,"'controlled clinical trial'/de",431650,18 Nov 2020

#151,"'randomized controlled trial'/de",629374,18 Nov 2020

#150,"#72 OR #149",107747,18 Nov 2020

#149,"#61 AND #148",106050,18 Nov 2020

#148,"#73 OR #74 OR #75 OR #76 OR #77 OR #78 OR #79 OR #80 OR #81 OR #82 OR #83 OR #84 OR #85 OR #86 OR #87 OR #88 OR #89 OR #90 OR #91 OR #92 OR #93 OR #94 OR #95 OR #96 OR #97 OR #98 OR #99 OR #100 OR #101 OR #102 OR #103 OR #104 OR #105 OR #106 OR #107 OR #108 OR #109 OR #110 OR #111 OR #112 OR #113 OR #114 OR #115 OR #116 OR #117 OR #118 OR #119 OR #120 OR #121 OR #122 OR #123 OR #124 OR #125 OR #126 OR #127 OR #128 OR #129 OR #130 OR #131 OR #132 OR #133 OR #134 OR #135 OR #136 OR #137 OR #138 OR #139 OR #140 OR #141 OR #142 OR #143 OR #144 OR #145 OR #146 OR #147",1460320,18 Nov 2020

#147,"'peficitinib'/de OR '4 [(5 hydroxyadamantan 2 yl) amino] 1h pyrrolo [2, 3 b] pyridine 5 carboxamide' OR '4 [(5 hydroxytricyclo [3.3.1.1 3, 7] dec 2 yl) amino] 1h pyrrolo [2, 3 b] pyridine 5 carboxamide' OR 'asp 015k' OR 'asp015k' OR 'peficitinib' OR 'peficitinib hydrobromide'",182,18 Nov 2020

#146,"'ritlecitinib'/exp OR '1 [2 methyl 5 (7h pyrrolo [2, 3 d] pyrimidin 4 ylamino) 1 piperidinyl] prop 2 en 1 one' OR '1 [2 methyl 5 (7h pyrrolo [2, 3 d] pyrimidin 4 ylamino) piperidin 1 yl] prop 2 en 1 one' OR '1 [2 methyl 5 [(7h pyrrolo [2, 3 d] pyrimidin 4 yl) amino] 1 piperidinyl] prop 2 en 1 one' OR '1 [2 methyl 5 [(7h pyrrolo [2, 3 d] pyrimidin 4 yl) amino] piperidin 1 yl] prop 2 en 1 one' OR '1 [5

(7h pyrrolo [2, 3 d] pyrimidin 4 ylamino) 2 methyl 1 piperidinyl] prop 2 en 1 one' OR '1 [5 (7h pyrrolo [2, 3 d] pyrimidin 4 ylamino) 2 methylpiperidin 1 yl] prop 2 en 1 one' OR '1 [5 [(7h pyrrolo [2, 3 d] pyrimidin 4 yl) amino] 2 methyl 1 piperidinyl] prop 2 en 1 one' OR '1 [5 [(7h pyrrolo [2, 3 d] pyrimidin 4 yl) amino] 2 methylpiperidin 1 yl] prop 2 en 1 one' OR 'pf 06651600' OR 'pf 6651600' OR 'pf06651600' OR 'pf6651600' OR 'ritlecitinib' OR 'ritlecitinib malonate' OR 'ritlecitinib propanedioate'",39,18 Nov 2020

#145,"'fedratinib'/de OR 'fedratinib' OR 'fedratinib dihydrochloride' OR 'fedratinib dihydrochloride monohydrate' OR 'fedratinib hydrochloride' OR 'inrebic' OR 'n (1, 1 dimethylethyl) 3 [[5 methyl 2 [[4 [2 (1 pyrrolidinyl) ethoxy] phenyl] amino] 4 pyrimidinyl] amino] benzenesulfonamide' OR 'n tert butyl 3 [[5 methyl 2 [[4 (2 pyrrolidin 1 ylethoxy) phenyl] amino] pyrimidin 4 yl] amino] benzenesulfonamide' OR 'n tert butyl 3 [[5 methyl 2 [4 [2 (pyrrolidin 1 yl) ethoxy] anilino] pyrimidin 4 yl] amino] benzenesulfonamide' OR 'n tert butyl 3 [5 methyl 2 [4 (2 pyrrolidin 1 ylethoxy) phenylamino] pyrimidin 4 ylamino] benzenesulfonamide' OR 'n tert butyl 3 [5 methyl 2 [4 [2 (1 pyrrolidinyl) ethoxy] phenylamino] 4 pyrimidinylamino] benzenesulfonamide' OR 'sar 302503' OR 'sar 302503a' OR 'sar302503' OR 'sar302503a' OR 'tg 101348' OR 'tg101348'",648,18 Nov 2020

#144,"'n benzyl 2 cyano 3 (3,4 dihydroxyphenyl)acrylamide'/exp OR '2 cyano 3 (3, 4 dihydroxyphenyl) n (phenylmethyl) 2 propenamide' OR 'ag 490' OR 'ag490' OR 'n benzyl 2 cyano 3 (3,4 dihydroxyphenyl)acrylamide' OR 'tyrphostin ag 490' OR 'tyrphostin ag490'",2476,18 Nov 2020

#143,"'itacitinib'/de OR '1 [1 [[3 fluoro 2 (trifluoromethyl) 4 pyridinyl] carbonyl] 4 piperidinyl] 3 [4 (7h pyrrolo [2, 3 d] pyrimidin 4 yl) 1h pyrazol 1 yl] 3 azetidineacetonitrile' OR '1 [1 [[3 fluoro 2 (trifluoromethyl) pyridin 4 yl] carbonyl] piperidin 4 yl] 3 [4 (7h pyrrolo [2, 3 d] pyrimidin 4 yl) 1h pyrazol 1 yl] 3 azetidineacetonitrile' OR '[1 [1 (3 fluoro 2 trifluoromethylpyridine 4 carbonyl) 4

piperidinyl] 3 [4 (7h pyrrolo [2, 3 d] pyrimidin 4 yl) 1h pyrazol 1 yl] 3 azetidinyl] ethanenitrile' OR '[1 [1 [3 fluoro 2 (trifluoromethyl) pyridine 4 carbonyl] piperidin 4 yl] 3 [4 (7h pyrrolo [2, 3 d] pyrimidin 4 yl) 1h pyrazol 1 yl] azetidin 3 yl] ethanenitrile' OR 'incb 039110' OR 'incb 39110' OR 'incb039110' OR 'incb39110' OR 'itacitinib' OR 'itacitinib adipate' OR 'itacitinib hexanedioate'",183,18 Nov 2020

#142,"'ruxolitinib'/de OR '3 [4 (7h pyrrolo [2, 3 d] pyrimidin 4 yl) 1h pyrazol 1 yl] 3 cyclopentylpropanenitrile' OR '3 [4 (7h pyrrolo [2, 3 d] pyrimidin 4 yl) 1h pyrazol 1 yl] 3 cyclopentylpropanenitrile phosphate' OR '3 cyclopentyl 3 [4 (7h pyrrolo [2, 3 d] pyrimidin 4 yl) 1h pyrazol 1 yl] propanenitrile' OR 'beta cyclopentyl 4 (7h pyrrolo [2, 3 d] pyrimidin 4 yl) 1h pyrazole 1 propanenitrile' OR 'incb 018424' OR 'incb 18424' OR 'incb 424' OR 'incb018424' OR 'incb18424' OR 'incb424' OR 'jakafi' OR 'jakavi' OR 'ruxolitinib' OR 'ruxolitinib maleate' OR 'ruxolitinib phosphate'",5326,18 Nov 2020

#141,"'upadacitinib'/de OR '3 ethyl 4 (3h imidazo [1, 2 a] pyrrolo [2, 3 e] pyrazin 8 yl) n (2, 2, 2 trifluoroethyl) 1 pyrrolidinecarboxamide' OR '3 ethyl 4 (3h imidazo [1, 2 a] pyrrolo [2, 3 e] pyrazin 8 yl) n (2, 2, 2 trifluoroethyl) 1 pyrrolidinecarboxamide 2, 3 dihydroxybutanedioate' OR '3 ethyl 4 (3h imidazo [1, 2 a] pyrrolo [2, 3 e] pyrazin 8 yl) n (2, 2, 2 trifluoroethyl) 1 pyrrolidinecarboxamide tartrate' OR '3 ethyl 4 (3h imidazo [1, 2 a] pyrrolo [2, 3 e] pyrazin 8 yl) n (2, 2, 2 trifluoroethyl) pyrrolidine 1 carboxamide' OR '3 ethyl 4 (3h imidazo [1, 2 a] pyrrolo [2, 3 e] pyrazin 8 yl) n (2, 2, 2 trifluoroethyl) pyrrolidine 1 carboxamide 2, 3 dihydroxybutanedioate' OR '3 ethyl 4 (3h imidazo [1, 2 a] pyrrolo [2, 3 e] pyrazin 8 yl) n (2, 2, 2 trifluoroethyl) pyrrolidine 1 carboxamide tartrate' OR 'abt 494' OR 'abt494' OR 'rinvoq' OR 'upadacitinib' OR 'upadacitinib 2, 3 dihydroxybutanedioate' OR 'upadacitinib hemihydrate' OR 'upadacitinib hydrate' OR 'upadacitinib tartrate'",545,18 Nov 2020

#140,"'filgotinib'/de OR 'filgotinib' OR 'filgotinib 2 butenedioate' OR 'filgotinib hydrochloride' OR 'filgotinib maleate' OR 'g 146034' OR 'g 146034 101' OR 'g 146034-101' OR 'g146034' OR 'g146034 101' OR 'g146034-101' OR 'glpg 0634' OR 'glpg0634' OR 'gs 6034' OR 'gs6034' OR 'n [5 [4 (1, 1 dioxothiomorpholinomethyl) phenyl] 1, 2, 4 triazolo [1, 5 a] pyridin 2 yl] cyclopropanecarboxamide' OR 'n [5 [4 (1, 1 dioxothiomorpholinomethyl) phenyl] 1, 2, 4 triazolo [1, 5 a] pyridin 2 yl] cyclopropanecarboxamide 2 butenedioate' OR 'n [5 [4 (1, 1 dioxothiomorpholinomethyl) phenyl] 1, 2, 4 triazolo [1, 5 a] pyridin 2 yl] cyclopropanecarboxamide but 2 enedioate' OR 'n [5 [4 [(1, 1 dioxido 4 thiomorpholinyl) methyl] phenyl] 1, 2, 4 triazolo [1, 5 a] pyridin 2 yl] cyclopropanecarboxamide' OR 'n [5 [4 [(1, 1 dioxido 4 thiomorpholinyl) methyl] phenyl] 1, 2, 4 triazolo [1, 5 a] pyridin 2 yl] cyclopropanecarboxamide 2 butenedioate' OR 'n [5 [4 [(1, 1 dioxothiomorpholin 4 yl) methyl] phenyl] 1, 2, 4 triazolo [1, 5 a] pyridin 2 yl] cyclopropanecarboxamide' OR 'n [5 [4 [(1, 1 dioxothiomorpholin 4 yl) methyl] phenyl] 1, 2, 4 triazolo [1, 5 a] pyridin 2 yl] cyclopropanecarboxamide but 2 enedioate' OR 'n [5 [4 [(1, 1 dioxothiomorpholin 4 yl) methyl] phenyl] [1, 2, 4] triazolo [1, 5 a] pyridin 2 yl] cyclopropanecarboxamide' OR 'n [5 [4 [(1, 1 dioxothiomorpholin 4 yl) methyl] phenyl] [1, 2, 4] triazolo [1, 5 a] pyridin 2 yl] cyclopropanecarboxamide but 2 enedioate'",502,18 Nov 2020

#139,"'tofacitinib'/de OR '1 cyanoacetyl 4 methyl n methyl n (1h pyrrolo [2, 3 d] pyrimidin 4 yl) 3 piperidinamine' OR '3 [4 methyl 3 [methyl (7h pyrrolo [2, 3 d] pyrimidin 4 yl) amino] 1 piperidinyl] 3 oxopropanenitrile' OR '4 [n [1 (2 cyano 1 oxoethyl) 4 methyl 3 piperidinyl] n methylamino] pyrrolo [2, 3 d] pyrimidine' OR '4 methyl 3 [methyl (7h pyrrolo [2, 3 d] pyrimidin 4 yl) amino] beta oxo 1 piperidinepropanenitrile' OR 'cp 690 550' OR 'cp 690, 550' OR 'cp 690550' OR 'cp 690550 10' OR 'cp 690550-10' OR 'cp690 550' OR 'cp690, 550' OR 'cp690550' OR 'cp690550 10' OR 'cp690550-10' OR

'tasocitinib' OR 'tasocitinib citrate' OR 'tofacitinib' OR 'tofacitinib citrate' OR 'xeljanz' OR 'xeljanz xr'",5239,18 Nov 2020

#138,"'baricitinib'/de OR '1 (ethylsulfonyl) 3 [4 (7h pyrrolo [2, 3 d] pyrimidin 4 yl) 1h pyrazol 1 yl] 3 azetidineacetonitrile' OR '[1 (ethanesulfonyl) 3 [4 (7h pyrrolo [2, 3 d] pyrimidin 4 yl) 1h pyrazol 1 yl] azetidin 3 yl] ethanenitrile' OR '[1 (ethylsulfonyl) 3 [4 (1h pyrrolo [2, 3 d] pyrimidin 4 yl) 1h pyrazol 1 yl] 3 azetidinyl] acetonitrile' OR '[1 (ethylsulfonyl) 3 [4 (7h pyrrolo [2, 3 d] pyrimidin 4 yl) 1h pyrazol 1 yl] azetidin 3 yl] ethanenitrile' OR 'baricitinib' OR 'incb 028050' OR 'incb 28050' OR 'incb028050' OR 'incb28050' OR 'ly 3009104' OR 'ly3009104' OR 'olumiant'",1525,18 Nov 2020

#137,"(janus NEAR/4 kinase NEAR/10 inhibitor$):tt",4,18 Nov 2020

#136,"(janus NEAR/4 kinase NEAR/10 inhibitor$):ti,ab,kw,tn",3309,18 Nov 2020

#135,"((jak OR jak1 OR jak2 OR jak3 OR 'janus kinase') NEAR/10 inhibitor*):tt",21,18 Nov 2020

#134,"((jak OR jak1 OR jak2 OR jak3 OR 'janus kinase') NEAR/10 inhibitor*):ti,ab,kw,tn",11228,18 Nov 2020

#133,"'janus kinase inhibitor'/exp OR 'jak inhibitor' OR 'janus kinase inhibitor' OR 'janus kinase inhibitors' OR 'janus tyrosine kinase inhibitor'",16132,18 Nov 2020

#132,"(steroid$ NEAR/10 (therap* OR treat* OR drug$ OR inject* OR intramuscular* OR 'intra muscular*' OR im OR intravenous* OR 'intra venous*' OR iv OR intraarticular* OR 'intra articular*' OR ia OR administrat* OR oral OR orally OR parenteral*)):tt",147,18 Nov 2020

#131,"(steroid$ NEAR/10 (therap* OR treat* OR drug$ OR inject* OR intramuscular* OR 'intra muscular*' OR im OR intravenous* OR 'intra venous*' OR iv OR intraarticular* OR 'intra articular*' OR ia OR administrat* OR oral OR orally OR parenteral*)):ti,ab,kw",118045,18 Nov 2020

Kastrati K, *et al. RMD Open* 2022; 8:e002359. doi: 10.1136/rmdopen-2022-002359

#130,"'triamcinolone'/exp OR '9alpha fluoro 1, 4 pregnadiene 3, 20 dione 11beta, 16alpha, 17alpha, 21 tetrol' OR '9alpha fluoro 11beta, 16alpha, 17alpha, 21 tetrahydroxypregna 1, 4 diene 3, 20 dione' OR '9alpha fluoro 16alpha hydroxyhydrocortisone' OR '9alpha fluoro 16alpha hydroxyprednisolone' OR 'acetocot' OR 'adcortyl' OR 'aristocort' OR 'aristocort forte' OR 'aristodan' OR 'azmacor' OR 'celeste' OR 'cl 19823' OR 'cl19823' OR 'clinacort' OR 'clinalog' OR 'delphicort' OR 'fluoxiprednisolone' OR 'fluoxyprednisolone' OR 'ken-jec 40' OR 'kenacort' OR 'kenacort retard' OR 'korticoid' OR 'ledercort' OR 'omcilon' OR 'polcortolon' OR 'rp 8357' OR 'rp8357' OR 'simacort' OR 'sterocort' OR 'tac 3' OR 'tramcinolone' OR 'triacortyl' OR 'triam-a' OR 'triam-forte' OR 'triamcinolon' OR 'triamcinolona' OR 'triamcinolone*' OR 'triamcort' OR 'triamcot' OR 'triamonide 40' OR 'triamsicort' OR 'triancinolon' OR 'u-tri-lone' OR 'volon'",39545,18 Nov 2020
#129,"'beclometasone'/exp OR '9 chloro 11beta, 17, 21 trihydroxy 16beta methylpregna 1, 4 diene 3, 20 dione' OR '9alpha chloro 16beta methylprednisolone' OR 'beclometasone' OR 'beclomethasone' OR 'prednisolone, 9alpha chloro 16beta methyl' OR 'pregna 1, 4 diene 11beta, 17, 21 triol 3, 20 dione, 9 chloro 16beta methyl'",15089,18 Nov 2020
#128,"'dexamethasone'/de OR '16alpha methyl 9alpha fluoroprednisolone' OR '9 alpha fluoro 16 alpha methyl delta corticosterone' OR '9alpha fluoro 11beta, 17alpha, 21 trihydroxy 16alpha methyl 1, 4 pregnadiene 3, 20 dione' OR '9alpha fluoro 11beta, 17alpha, 21 trihydroxy 16alpha methylpregna 1, 4 diene 3, 20 dione' OR '9alpha fluoro 16alpha methyl delta corticosterone' OR 'adrecort' OR 'adrenocot' OR 'aeroseb dex' OR 'aeroseb-dex' OR 'aflucoson' OR 'aflucosone' OR 'alfalyl' OR 'anaflogistico' OR 'anaflogistico novobios' OR 'arcodexan' OR 'arcodexane' OR 'artrosone' OR 'azium' OR 'bidexol' OR 'calonat' OR 'cebedex' OR 'cetadexon' OR 'colofoam' OR 'corsona' OR 'cortastat' OR 'cortastat 10' OR 'cortastat la' OR 'cortidex' OR 'cortidexason' OR 'cortidrona' OR

'cortidrone' OR 'cortisumman' OR 'dacortina fuerte' OR 'dacortine fuerte' OR 'dalalone' OR 'dalalone d.p.' OR 'dalalone l.a.' OR 'danasone' OR 'de-sone la' OR 'decacortin' OR 'decadeltosona' OR 'decadeltosone' OR 'decaderm' OR 'decadion' OR 'decadran' OR 'decadron' OR 'decadron 5-12 pak' OR 'decadron la' OR 'decadronal' OR 'decadrone' OR 'decaesadril' OR 'decaject' OR 'decamethasone' OR 'decasone' OR 'decaspray' OR 'decasterolone' OR 'decdan' OR 'decilone' OR 'decilone forte' OR 'decofluor' OR 'dectancyl' OR 'dekacort' OR 'delladec' OR 'deltafluoren' OR 'deltafluorene' OR 'dergramin' OR 'deronil' OR 'desacort' OR 'desacortone' OR 'desadrene' OR 'desalark' OR 'desameton' OR 'desametone' OR 'desigdron' OR 'dexa cortisyl' OR 'dexa dabrosan' OR 'dexa korti' OR 'dexa scherosan' OR 'dexa scherozon' OR 'dexa scherozone' OR 'dexa-p' OR 'dexacen 4' OR 'dexacen-4' OR 'dexachel' OR 'dexacort' OR 'dexacortal' OR 'dexacorten' OR 'dexacortin' OR 'dexacortisyl' OR 'dexadabroson' OR 'dexadecadrol' OR 'dexadrol' OR 'dexagel' OR 'dexagen' OR 'dexahelvacort' OR 'dexakorti' OR 'dexalien' OR 'dexalocal' OR 'dexame' OR 'dexamecortin' OR 'dexameson' OR 'dexamesone' OR 'dexametason' OR 'dexametasone' OR 'dexameth' OR 'dexamethason' OR 'dexamethasone' OR 'dexamethasone alcohol' OR 'dexamethasone intensol' OR 'dexamethazon' OR 'dexamethazone' OR 'dexamethonium' OR 'dexamonozon' OR 'dexan' OR 'dexane' OR 'dexano' OR 'dexapot' OR 'dexascheroson' OR 'dexascherozon' OR 'dexascherozone' OR 'dexason' OR 'dexasone' OR 'dexasone la' OR 'dexasone s' OR 'dexinoral' OR 'dexionil' OR 'dexmethsone' OR 'dexona' OR 'dexone' OR 'dexone 0.5' OR 'dexone 0.75' OR 'dexone 1.5' OR 'dexone 4' OR 'dexpak taperpak' OR 'dextelan' OR 'dextenza' OR 'dextrasone' OR 'dexycu' OR 'dezone' OR 'dibasona' OR 'doxamethasone' OR 'esacortene' OR 'ex s1' OR 'exadion' OR 'exadione' OR 'firmalone' OR 'fluormethyl prednisolone' OR 'fluormethylprednisolon' OR 'fluormethylprednisolone' OR 'fluormone' OR 'fluorocort' OR 'fluorodelta' OR

'fluoromethylprednisolone' OR 'fortecortin' OR 'gammacorten' OR 'gammacortene' OR 'grosodexon' OR 'grosodexone' OR 'hemady' OR 'hexadecadiol' OR 'hexadecadrol' OR 'hexadiol' OR 'hexadrol' OR 'isnacort' OR 'isopto dex' OR 'isopto maxidex' OR 'isopto-dex' OR 'isopto-maxidex' OR 'isoptodex' OR 'isoptomaxidex' OR 'lokalison f' OR 'loverine' OR 'luxazone' OR 'marvidione' OR 'maxidex' OR 'mediamethasone' OR 'megacortin' OR 'mephameson' OR 'mephamesone' OR 'metasolon' OR 'metasolone' OR 'methazon ion' OR 'methazone ion' OR 'methazonion' OR 'methazonione' OR 'metisone lafi' OR 'mexasone' OR 'millicorten' OR 'millicortenol' OR 'mk 125' OR 'mk125' OR 'mymethasone' OR 'neoforderx' OR 'neofordex' OR 'nisomethasona' OR 'novocort' OR 'nsc 34521' OR 'nsc34521' OR 'oftan-dexa' OR 'opticorten' OR 'opticortinol' OR 'oradexan' OR 'oradexon' OR 'oradexone' OR 'orgadrone' OR 'ozurdex' OR 'pidexon' OR 'policort' OR 'posurdex' OR 'predni f tablinen' OR 'predni-f' OR 'prednisolone f' OR 'prodexona' OR 'prodexone' OR 'sanamethasone' OR 'santenson' OR 'santeson' OR 'sawasone' OR 'solurex' OR 'solurex la' OR 'spoloven' OR 'sterasone' OR 'thilodexine' OR 'triamcimetil' OR 'vexamet' OR 'visumetazone' OR 'visumethazone'",170905,18 Nov 2020

#127,"'hydrocortisone'/de OR '11beta, 17, 21 trihydroxypregn 4 ene 3, 20 dione' OR '11beta, 17alpha, 21 trihydroxypregn 4 ene 3, 20 dione' OR '17 hydroxycorticosterone' OR '4 pregnene 11beta, 17alpha, 21 triol 3, 20 dione' OR '4 pregnene 3, 20 dione n beta, 17alpha, 21 triol' OR 'acticort' OR 'acticort 100' OR 'aeroseb hc' OR 'aeroseb-hc' OR 'ala-cort' OR 'ala-scalp' OR 'ala-scalp hp' OR 'alfacort' OR 'algicortis' OR 'alkindi' OR 'alpha derm' OR 'alphaderm' OR 'anucort-hc' OR 'anumed-hc' OR 'anutone-hc' OR 'aquanil hc' OR 'balneol-hc' OR 'barseb hc' OR 'beta-hc' OR 'biacort' OR 'cetacort' OR 'cobadex' OR 'colocort' OR 'compound f' OR 'cordicare lotion' OR 'coripen' OR 'cort dome' OR 'cort-dome' OR 'cort-dome high potency' OR 'cortef' OR 'cortef cream' OR

'cortenema' OR 'cortibel' OR 'corticorenol' OR 'cortifan' OR 'cortiphate' OR 'cortisol' OR 'cortisole' OR 'cortispray' OR 'cortoderm' OR 'cortril' OR 'cotacort' OR 'covocort' OR 'cremicort-h' OR 'cutaderm' OR 'derm-aid cream' OR 'dermacrin hc lotion' OR 'dermaid' OR 'dermaid soft cream' OR 'dermocare' OR 'dermocortal' OR 'dermolate' OR 'dioderm' OR 'eczacort' OR 'ef cortelan' OR 'efcortelan' OR 'egocort' OR 'egocort cream' OR 'eksalb' OR 'eldecort' OR 'emo-cort' OR 'epicort' OR 'ficortril' OR 'filocot' OR 'flexicort' OR 'gly-cort' OR 'glycort' OR 'h-cort' OR 'hc (hydrocortisone)' OR 'hc no. 1' OR 'hc no. 4' OR 'hebcort' OR 'hebcort v' OR 'hemorrhoidal hc' OR 'hemril-30' OR 'hemril-hc uniserts' OR 'hi-cor' OR 'hidrotisona' OR 'hycor' OR 'hycort' OR 'hydracort' OR 'hydrasson' OR 'hydro ricortex' OR 'hydro-rx' OR 'hydrocort' OR 'hydrocorticosteroid' OR 'hydrocortisate' OR 'hydrocortison' OR 'hydrocortisone' OR 'hydrocortisone 1% in absorbase' OR 'hydrocortisone acetonide' OR 'hydrocortisone astier' OR 'hydrocortisone in absorbase' OR 'hydrocortisone ointment' OR 'hydrocortisone plus saline' OR 'hydrocortisone steroid' OR 'hydrocortisone, topical' OR 'hydrocortisonum' OR 'hydrocortisyl' OR 'hydrocortone' OR 'hydrogalen' OR 'hydrokort' OR 'hydrokortison' OR 'hydrotopic' OR 'hysone' OR 'hytisone' OR 'hytone' OR 'hytone lotion' OR 'incortin h' OR 'instacort 10' OR 'kyypakkaus' OR 'lacticare hc' OR 'lacticare-hc' OR 'lemnis fatty cream hc' OR 'lenirit' OR 'medihaler cort' OR 'medihaler duo' OR 'medrocil' OR 'mildison' OR 'mildison fet krem' OR 'mildison lipocream' OR 'mildison-fatty' OR 'mitocortyl demangeaisons' OR 'munitren' OR 'nogenic hc' OR 'novohydrocort' OR 'nsc 10483' OR 'nsc 741' OR 'nsc10483' OR 'nutracort' OR 'optef' OR 'otosone f' OR 'penecort' OR 'plenadren' OR 'prepcort' OR 'prevex hc' OR 'pro cort' OR 'procort' OR 'procto-kit 1%' OR 'procto-kit 2.5%' OR 'proctocort' OR 'proctosert hc' OR 'proctosol-hc' OR 'proctosone' OR 'proctozone hc' OR 'procutan' OR 'rectasol-hc' OR 'rectocort' OR 'rederm' OR 'sanatison' OR 'scalp-aid' OR 'schericur' OR 'schericur 0.25%' OR 'scherosone f' OR

'sistral hydrocort' OR 'skincalm' OR 'stie-cort' OR 'substance m' OR 'synacort' OR 'texacort' OR 'triburon-hc' OR 'unicort' OR 'vasocort'",165665,18 Nov 2020

#126,"'corticosterone'/de OR '11 beta, 21 dihydroxy pregn 4 ene 3, 20 dione' OR '11beta, 21 dihydroxy 4 pregnene 3, 20 dione' OR '11beta, 21 dihydroxypregn 4 ene 3, 20 dione' OR '4 pregnene 11beta, 21 diol 3, 20 dione' OR 'compound b' OR 'corticosterone*' OR 'corticosterone function' OR 'corticosterone response' OR 'kendall compound b' OR 'nsc 9705' OR 'nsc9705' OR 'pregn 4 ene 11beta, 21 diol 3, 20 dione' OR 'reichstein substance h'",45670,18 Nov 2020

#125,"'glucocorticoid'/exp OR 'glucocorticoid*' OR 'glucocorticoid drug' OR 'glucocorticoid hormone' OR 'glucocorticoid steroid' OR 'glucocorticoids' OR 'glucocorticoids, synthetic' OR 'glucocorticoids, topical' OR 'glucocorticoidsteroid' OR 'glucocorticosteroid' OR 'glucocortoid' OR 'glycocorticoid' OR 'glycocorticosteroid'",796299,18 Nov 2020

#124,"'methylprednisolone'/de OR '11beta, 17alpha, 21 trihydroxy 6alpha methyl 1, 4 pregnadiene 3, 20 dione' OR '2 methylprednisolone' OR '5 methylprednisolone' OR '6 alpha methylprednisolone' OR '6 methyl delta 1 hydrocortisone' OR '6 methyl prednisolone' OR '6 methylprednisolone' OR '6alpha methyl delta1 hydrocortisone' OR '6alpha methylprednisolone' OR 'adlone-40' OR 'adlone-80' OR 'beta methylprednisolone' OR 'dep medalone 80' OR 'depmedalone' OR 'depoject-80' OR 'depopred' OR 'esametone' OR 'firmacort' OR 'med-jec-40' OR 'medixon' OR 'mednin' OR 'medralone 80' OR 'medrate' OR 'medrol' OR 'medrol a' OR 'medrol adt pak' OR 'medrol compositum' OR 'medrol dosepak' OR 'medrol medules' OR 'medrol pak' OR 'medrone' OR 'meprednisolone' OR 'meprelon' OR 'mesopren' OR 'methacort 40' OR 'methacort 80' OR 'methyl prednisolone' OR 'methylcotol' OR 'methylcotolone' OR 'methylpred dp' OR 'methylprednisolone*' OR 'methylsterolone' OR 'metidrol' OR 'metrisone' OR 'metycortin' OR 'metypred' OR 'metypresol'

OR 'neomedrone' OR 'nsc 19987' OR 'nsc19987' OR 'prednol' OR 'solomet' OR 'solu decortin' OR 'urbason' OR '83 43 2':rn",109096,18 Nov 2020

#123,"'cortisone'/de OR '11 dehydro 17 hydroxy corticosterone' OR '11 dehydro 17 hydroxycorticosterone' OR '17 hydroxy 11 dehydrocortisone' OR '17alpha, 21 dihydroxy 4 pregnene 3, 11, 20 trione' OR '4 pregnene 17alpha, 21 diol 3, 11, 20 trione' OR 'adrenalex' OR 'compound e (kendall)' OR 'corlin' OR 'cortadren' OR 'cortagen' OR 'cortandren' OR 'cortane' OR 'cortisal' OR 'cortisate' OR 'cortison' OR 'cortisone$' OR 'cortisone sodium' OR 'cortistal' OR 'cortivite' OR 'cortogen' OR 'cortone' OR 'delta4 pregnene 17alpha, 21 diol 3, 11, 20 trione' OR 'incorlin' OR 'incortin' OR 'kendall compound e' OR 'nsc 9703' OR 'nsc9703' OR 'pregn 4 en 17 alpha, 21 diol 3, 11, 20 trione' OR 'pregn 4 ene 17alpha, 21 diol 3, 11, 20 trione' OR 'reichstein substance fa' OR 'ricortex' OR 'scheroson' OR 'scherosone' OR 'wintersteiner compound f'",32696,18 Nov 2020

#122,"'cortisone therapeutic use'/de",3404,18 Nov 2020

#121,"'prednisone'/de OR '1, 2 dehydrocortisone' OR '17, 21 dihydroxypregna 1, 4 diene 3, 11, 20 trione' OR 'ancortone' OR 'apo-prednisone' OR 'biocortone' OR 'colisone' OR 'cortan' OR 'cortidelt' OR 'cortiprex' OR 'cutason' OR 'dacorten' OR 'de cortisyl' OR 'decortancyl' OR 'decortin' OR 'decortin e merck' OR 'decortine' OR 'decortisyl' OR 'dehydrocortisone' OR 'dekortin' OR 'delitisone' OR 'dellacort a' OR 'delta 1 dehydrocortisone' OR 'delta cortelan' OR 'delta cortisone' OR 'delta dome' OR 'delta e' OR 'delta prenovis' OR 'delta-dome' OR 'deltacorten' OR 'deltacortene' OR 'deltacortisone' OR 'deltacortone' OR 'deltasone' OR 'deltison' OR 'deltisona' OR 'deltra' OR 'di adreson' OR 'di-adreson' OR 'diadreson' OR 'drazone' OR 'encorton' OR 'encortone' OR 'enkorton' OR 'fernisone' OR 'hostacortin' OR 'insone' OR 'liquid pred' OR 'lodotra' OR 'me-korti' OR 'meprison' OR 'metacortandracin' OR 'meticorten' OR 'meticortine' OR 'nisona' OR 'nsc 10023' OR 'nsc10023'

OR 'orasone' OR 'orisane' OR 'panafcort' OR 'paracort' OR 'pehacort' OR 'precort' OR 'precortal' OR 'prednicen-m' OR 'prednicorm' OR 'prednicot' OR 'prednidib' OR 'prednison' OR 'prednisone*' OR 'prednisone alcohol' OR 'prednisone intensol' OR 'prednisone test' OR 'prednitone' OR 'pregna 1, 4 diene 3, 11, 20 trione 17, 21 diol' OR 'pronison' OR 'pronisone' OR 'pronizone' OR 'pulmison' OR 'rayos' OR 'rectodelt' OR 'servisone' OR 'steerometz' OR 'sterapred' OR 'sterapred ds' OR 'ultracorten' OR 'urtilone' OR 'winpred'",187108,18 Nov 2020

#120,"'prednisolone'/de OR '1, 2 dehydro hydrocortison' OR '1, 4 pregnadien 11beta, 17alpha, 21 triol 3, 20 dion' OR '1, 4 pregnadiene 11beta, 17alpha, 21 triol 3, 20 dione' OR '1, 4 pregnadiene 3, 20 dione 11beta, 17alpha, 21 triol' OR '11beta, 17, 21 trihydroxypregna 1, 4 diene 3, 20 dione' OR '11beta, 17alpha, 21 trihydroxy 1, 4 pregnadien 3, 20 dion' OR '11beta, 17alpha, 21 trihydroxypregna 1, 4 diene 3, 20 dione' OR '3, 20 dioxo 11beta, 17alpha, 21 trihydroxy 1, 4 pregnadiene' OR 'adelcort' OR 'antisolon' OR 'antisolone' OR 'aprednislon' OR 'aprednislone' OR 'benisolon' OR 'benisolone' OR 'berisolon' OR 'berisolone' OR 'caberdelta' OR 'capsoid' OR 'co hydeltra' OR 'codelcortone' OR 'compresolon' OR 'cortadeltona' OR 'cortadeltone' OR 'cortalone' OR 'cortelinter' OR 'cortisolone' OR 'cotolone' OR 'dacortin' OR 'dacortin h' OR 'dacrotin' OR 'decaprednil' OR 'decortin h' OR 'decortril' OR 'dehydro cortex' OR 'dehydro hydrocortison' OR 'dehydro hydrocortisone' OR 'dehydrocortex' OR 'dehydrocortisol' OR 'dehydrocortisole' OR 'dehydrohydrocortison' OR 'dehydrohydrocortisone' OR 'delcortol' OR 'delta 1 17 hydroxycorticosterone 21 acetate' OR 'delta 1 hydrocortisone' OR 'delta cortef' OR 'delta cortril' OR 'delta ef cortelan' OR 'delta f' OR 'delta hycortol' OR 'delta hydrocortison' OR 'delta hydrocortisone' OR 'delta ophticor' OR 'delta stab' OR 'delta-cortef' OR 'delta1 dehydrocortisol' OR 'delta1 dehydrohydrocortisone' OR 'delta1 hydrocortisone' OR 'deltacortef' OR 'deltacortenolo' OR

'deltacortil' OR 'deltacortoil' OR 'deltacortril' OR 'deltaderm' OR 'deltaglycortril' OR 'deltahycortol' OR 'deltahydrocortison' OR 'deltahydrocortisone' OR 'deltaophticor' OR 'deltasolone' OR 'deltastab' OR 'deltidrosol' OR 'deltisilone' OR 'deltisolon' OR 'deltisolone' OR 'deltolasson' OR 'deltolassone' OR 'deltosona' OR 'deltosone' OR 'depo-predate' OR 'dermosolon' OR 'dhasolone' OR 'di adreson f' OR 'di adresone f' OR 'di-adreson-f' OR 'diadreson f' OR 'diadresone f' OR 'dicortol' OR 'domucortone' OR 'encortelon' OR 'encortelone' OR 'encortolon' OR 'equisolon' OR 'fernisolone-p' OR 'glistelone' OR 'hefasolon' OR 'hostacortin h' OR 'hostacortin h vet' OR 'hydeltra' OR 'hydeltrone' OR 'hydrelta' OR 'hydrocortancyl' OR 'hydrocortidelt' OR 'hydrodeltalone' OR 'hydrodeltisone' OR 'hydroretrocortin' OR 'hydroretrocortine' OR 'inflanefran' OR 'insolone' OR 'keteocort h' OR 'key-pred' OR 'key-pred sp' OR 'lenisolone' OR 'leocortol' OR 'liquipred' OR 'lygal kopftinktur n' OR 'mediasolone' OR 'meprisolon' OR 'meprisolone' OR 'metacortalon' OR 'metacortalone' OR 'metacortandralon' OR 'metacortandralone' OR 'metacortelone' OR 'meti derm' OR 'meti-derm' OR 'meticortelone' OR 'metiderm' OR 'morlone' OR 'mydrapred' OR 'neo delta' OR 'nisolon' OR 'nisolone' OR 'nsc 9120' OR 'nsc9120' OR 'opredsone' OR 'panafcortelone' OR 'panafcortolone' OR 'panafort' OR 'paracortol' OR 'phlogex' OR 'pre cortisyl' OR 'preconin' OR 'precortalon' OR 'precortancyl' OR 'precortisyl' OR 'pred-ject-50' OR 'predacort 50' OR 'predaject-50' OR 'predalone 50' OR 'predartrina' OR 'predartrine' OR 'predate-50' OR 'predeltilone' OR 'predisole' OR 'predisyr' OR 'predne dome' OR 'prednecort' OR 'prednedome' OR 'prednelan' OR 'predni coelin' OR 'predni h tablinen' OR 'predni-helvacort' OR 'prednicoelin' OR 'prednicort' OR 'prednicortelone' OR 'prednifor drops' OR 'predniment' OR 'predniretard' OR 'prednis' OR 'prednisil' OR 'prednisolon' OR 'prednisolona' OR 'prednisolone*' OR 'prednisolone alcohol' OR 'prednisolone h' OR 'prednisolone oleosae sr 82' OR 'prednisolone, topical' OR 'prednivet' OR 'prednorsolon' OR 'prednorsolone' OR

'predonine' OR 'predorgasolona' OR 'predorgasolone' OR 'pregna 1, 4 diene 11beta, 17alpha, 21 triol 3, 20 dione' OR 'prelon' OR 'prelone' OR 'prenilone' OR 'prenin' OR 'prenolone' OR 'preventan' OR 'prezolon' OR 'rubycort' OR 'scherisolon' OR 'scherisolona' OR 'serilone' OR 'solondo' OR 'solone' OR 'solupren' OR 'soluprene' OR 'spiricort' OR 'spolotane' OR 'sterane' OR 'sterolone' OR 'supercortisol' OR 'supercortizol' OR 'taracortelone' OR 'walesolone' OR 'wysolone'",147230,18 Nov 2020

#119,"'corticosteroid'/exp OR 'adrenal cortex hormone' OR 'adrenal cortex hormones' OR 'adrenal cortical hormone' OR 'adrenal cortical hormones' OR 'adrenal cortical steroid' OR 'adrenal steroid' OR 'adrenal steroid hormone' OR 'adreno cortical steroid' OR 'adreno corticosteroid' OR 'adrenocortical hormone' OR 'adrenocortical steroid' OR 'adrenocorticosteroid' OR 'cortical steroid' OR 'cortico steroid' OR 'corticoid*' OR 'corticosteroid*' OR 'corticosteroid agent' OR 'corticosteroid calcium' OR 'corticosteroid hormone' OR 'corticosteroids' OR 'corticosteroids, inhalation' OR 'corticosteroids, ophthalmic' OR 'corticosteroids, otic' OR 'corticosteroids, systemic' OR 'corticosteroids, topical' OR 'dermocorticosteroid' OR 'fluorinated corticosteroid'",1038787,18 Nov 2020

#118,"chloroquin*:ti,ab,kw,rn,tn OR cq:ti,ab,kw,rn,tn OR '54-04-7':ti,ab,kw,rn,tn OR 886u3h6uff*:ti,ab,kw,rn,tn OR arequin:ti,ab,kw,rn,tn OR chingamin:ti,ab,kw,rn,tn OR chlorochin*:ti,ab,kw,rn,tn OR khingamin:ti,ab,kw,rn,tn OR oe48649kh6n*:ti,ab,kw,rn,tn OR anaclor:ti,ab,kw,rn,tn OR bemaco:ti,ab,kw,rn,tn OR benaquin:ti,ab,kw,rn,tn OR 'brn 0482809':ti,ab,kw,rn,tn OR brn0482809:ti,ab,kw,rn,tn OR capquin:ti,ab,kw,rn,tn OR 'ccris 3439':ti,ab,kw,rn,tn OR ccris3439:ti,ab,kw,rn,tn OR chloraquin*:ti,ab,kw,rn,tn OR cindacin:ti,ab,kw,rn,tn OR cindachin:ti,ab,kw,rn,tn OR cloroquina:ti,ab,kw,rn,tn OR

cocatrit:ti,ab,kw,rn,tn OR 'einecs 200-191-2':ti,ab,kw,rn,tn OR 'einecs200 191 2':ti,ab,kw,rn,tn OR elestol:ti,ab,kw,rn,tn OR 'hsdb 3029':ti,ab,kw,rn,tn OR hsdb3029:ti,ab,kw,rn,tn OR lapaquin:ti,ab,kw,rn,tn OR malaren:ti,ab,kw,rn,tn OR neochin:ti,ab,kw,rn,tn OR 'nsc 187208':ti,ab,kw,rn,tn OR nsc187208:ti,ab,kw,rn,tn OR pfizerquine:ti,ab,kw,rn,tn OR quensyl:ti,ab,kw,rn,tn OR quinercyl:ti,ab,kw,rn,tn OR quinilon:ti,ab,kw,rn,tn OR quinoline:ti,ab,kw,rn,tn OR quinoscan:ti,ab,kw,rn,tn OR ronaquine:ti,ab,kw,rn,tn OR sopaquin:ti,ab,kw,rn,tn OR 'st 21':ti,ab,kw,rn,tn OR weimerquin:ti,ab,kw,rn,tn",36649,18 Nov 2020

#117,"'chloroquine'/de OR '4 (4 diethylamino 1 methylbutylamino) 7 chlorchinolin diphosphate' OR '4 (4 diethylamino 1 methylbutylamino) 7 chlorchinolin sulfate' OR '4 (4 diethylamino 1 methylbutylamino) 7 chlorchinolin sulphate' OR '4 (4 diethylamino 1 methylbutylamino) 7 chloroquinoline' OR '7 chloro 4 (4 diethylamino 1 methylbutylamino) quinoline' OR '7 chloro 4 (4 diethylamino 1 methylbutylamino) quinoline diphosphate' OR 'a-cq' OR 'amokin' OR 'amokine' OR 'anoclor' OR 'aralan' OR 'aralen' OR 'aralen hydrochloride' OR 'aralen phosphate' OR 'aralene' OR 'arechin' OR 'arechine' OR 'arequine' OR 'arthrochin' OR 'arthrochine' OR 'arthroquine' OR 'artrichin' OR 'artrichine' OR 'artriquine' OR 'avloclor' OR 'avoclor' OR 'bemaphata' OR 'bemaphate' OR 'bemasulph' OR 'bipiquin' OR 'cadiquin' OR 'chemochin' OR 'chemochine' OR 'chingamine' OR 'chingaminum' OR 'chloraquine' OR 'chlorochin' OR 'chlorochine' OR 'chlorofoz' OR 'chloroquin' OR 'chloroquin phosphate' OR 'chloroquine' OR 'chloroquine diphosphate' OR 'chloroquine disulfate' OR 'chloroquine disulphate' OR 'chloroquine hydrochloride' OR 'chloroquine phosphate' OR 'chloroquine streuli' OR 'chloroquine sulfate' OR 'chloroquine sulphate' OR 'chloroquinesulphate' OR 'chloroquini diphosphas' OR 'chloroquinum diphosphoricum' OR 'chlorquin' OR 'chlorquine' OR 'choloquine' OR 'choroquine sulfate' OR 'choroquine sulphate' OR 'cidanchin' OR 'clo-kit junior' OR

'clorichina' OR 'clorichine' OR 'cloriquine' OR 'clorochina' OR 'delagil' OR 'delagyl' OR 'dichinalex' OR 'diclokin' OR 'diquinalex' OR 'diroquine' OR 'emquin' OR 'genocin' OR 'gontochin' OR 'gontochine' OR 'gontoquine' OR 'heliopar' OR 'imagon' OR 'iroquine' OR 'klorokin' OR 'klorokine' OR 'klorokinfosfat' OR 'lagaquin' OR 'malaquin' OR 'malarex' OR 'malarivon' OR 'malaviron' OR 'maliaquine' OR 'maquine' OR 'mesylith' OR 'mexaquin' OR 'mirquin' OR 'nivachine' OR 'nivaquin' OR 'nivaquine' OR 'nivaquine (b)' OR 'nivaquine b' OR 'nivaquine dp' OR 'nivaquine forte' OR 'p roquine' OR 'quinachlor' OR 'quingamine' OR 'repal' OR 'resochen' OR 'resochene' OR 'resochin' OR 'resochin junior' OR 'resochina' OR 'resochine' OR 'resochinon' OR 'resoquina' OR 'resoquine' OR 'reumachlor' OR 'roquine' OR 'rp 3377' OR 'rp3377' OR 'sanoquin' OR 'sanoquine' OR 'silbesan' OR 'siragan' OR 'sirajan' OR 'sn 7618' OR 'sn7618' OR 'solprina' OR 'solprine' OR 'tresochin' OR 'tresochine' OR 'tresoquine' OR 'trochin' OR 'trochine' OR 'troquine' OR 'w 7618' OR 'w7618' OR 'win 244' OR 'win244'",44472,18 Nov 2020

#116,"'organogold compound'/exp OR 'organogold compound' OR 'organogold compounds' OR 'organogold derivative'",482,18 Nov 2020

#115,"aurothiopropanol*:ti,ab,kw,rn,tn OR allochrysine:ti,ab,kw,rn,tn OR allocrisine:ti,ab,kw,rn,tn OR aurotiopoe:ti,ab,kw,rn,tn OR aurotioprol:ti,ab,kw,rn,tn OR '27279-43-2':ti,ab,kw,rn,tn OR g7097j63e9*:ti,ab,kw,rn,tn OR 'sodium aurothiopropanolsulfonate':ti,ab,kw,rn,tn OR 'gold sodium thiopropanol sulfonate':ti,ab,kw,rn,tn OR 'sodium auromercaptopropanol sulfonate':ti,ab,kw,rn,tn",274,18 Nov 2020

#114,"'allochrysine'/de OR '1 auromercapto 2 propanol 3 sulfonate' OR '1 auromercapto 2 propanol 3 sulfonate sodium' OR '1 auromercepto 2 propanol 3 sulfonate' OR 'allochrysine' OR 'auromercapto propanol sulfonate sodium' OR 'auromercaptopropanolsulfonate sodium' OR

'aurothioisopropanolsulfonate sodium' OR 'aurothiopropanol sulfonate' OR 'aurothiopropanol sulfonate sodium' OR 'gold sodium thiopropanol sulfonate' OR 'sodium 1 auromercapto 2 propanol 3 sulfonate' OR 'sodium auromercapto 2 propanol 3 sulfonate' OR 'sodium auromercaptopropanol sulfonate' OR 'sodium aurothiopropanol sulfonate'",271,18 Nov 2020

#113,"((gold* OR auro*) NEAR/10 (inject* OR intramuscular* OR 'im' OR 'intra muscular*')):ti,ab,kw",3122,18 Nov 2020

#112,"(thioglucosoaurate*:ti,ab,kw,rn,tn OR 'gold thioglucose*':ti,ab,kw,rn,tn OR 'gold thio glucose*':ti,ab,kw,rn,tn OR 'thioglucose gold':ti,ab,kw,rn,tn OR aureotan:ti,ab,kw,rn,tn OR 'gold-50':ti,ab,kw,rn,tn OR gold50:ti,ab,kw,rn,tn OR 'b oleosum solganal':ti,ab,kw,rn,tn OR aurotan:ti,ab,kw,rn,tn OR authron:ti,ab,kw,rn,tn OR brenol:ti,ab,kw,rn,tn OR 'ccris 59':ti,ab,kw,rn,tn OR ccris59:ti,ab,kw,rn,tn OR 'einecs 235-365-7':ti,ab,kw,rn,tn OR 'einecs235 365 7':ti,ab,kw,rn,tn OR glysanol:ti,ab,kw,rn,tn) AND b:ti,ab,kw,rn,tn OR goldthioglucose*:ti,ab,kw,rn,tn OR goldtioglucose*:ti,ab,kw,rn,tn OR 'hsdb 7174':ti,ab,kw,rn,tn OR hsdb7174:ti,ab,kw,rn,tn OR oronol:ti,ab,kw,rn,tn OR romosol:ti,ab,kw,rn,tn OR 'skf 10056':ti,ab,kw,rn,tn OR skf10056:ti,ab,kw,rn,tn OR 2p2v9q0e78*:ti,ab,kw,rn,tn OR '12192-57-3':ti,ab,kw,rn,tn",1507,18 Nov 2020

#111,"'aurothioglucose sodium'/de",40,18 Nov 2020

#110,"'aurothioglucose'/de OR '(1 d glucosylthio) gold' OR '(1 glucosylthio) gold' OR 'anitur' OR 'aureotan' OR 'auro thioglucose*' OR 'auromyose*' OR 'aurothioglucose*' OR 'aurumin' OR 'aurumine' OR 'gold thio glucose' OR 'gold thioglucose' OR 'gold-50' OR 'goldthioglucose' OR 'goldtioglucose' OR 'oronol' OR 'solganal' OR 'solganal b' OR 'solganol'",1706,18 Nov 2020

#109,"aurolate*:ti,ab,kw,rn,tn OR 'gold disodium thiomalate*':ti,ab,kw,rn,tn OR 'gold thiomalic acid*':ti,ab,kw,rn,tn OR miocrisin:ti,ab,kw,rn,tn OR 'monogold disodium thiomalate*':ti,ab,kw,rn,tn OR myochrysin*:ti,ab,kw,rn,tn OR myocrisin*:ti,ab,kw,rn,tn OR myocrysin*:ti,ab,kw,rn,tn OR 'sodium aurothiomalate*':ti,ab,kw,rn,tn OR 'sodium gold thiomalate*':ti,ab,kw,rn,tn OR 'sodium thiomalate* gold':ti,ab,kw,rn,tn OR 'sodium thiomalatoaurate*':ti,ab,kw,rn,tn OR taureodon:ti,ab,kw,rn,tn OR 'thiomalatoaurate sodium':ti,ab,kw,rn,tn OR '1244-57-4':ti,ab,kw,rn,tn OR '39377-38-3':ti,ab,kw,rn,tn OR '4846-27-9':ti,ab,kw,rn,tn OR '74916-57-7':ti,ab,kw,rn,tn OR e4768zy6gm*:ti,ab,kw,rn,tn OR 'aurothiomala-natrium':ti,ab,kw,rn,tn OR 'aurot$iomalato sodico':ti,ab,kw,rn,tn OR 'monogold* sodium salt':ti,ab,kw,rn,tn OR 'dinatrium 2-aurothio-succinat':ti,ab,kw,rn,tn OR 'einecs 235-479-7':ti,ab,kw,rn,tn OR 'einecs235 479 7':ti,ab,kw,rn,tn OR 'hsdb 7173':ti,ab,kw,rn,tn OR hsdb7173:ti,ab,kw,rn,tn OR kidon:ti,ab,kw,rn,tn OR 'natrii aurothiomalas*':ti,ab,kw,rn,tn OR shiosol:ti,ab,kw,rn,tn OR 'gold mercatpsuccinate*':ti,ab,kw,rn,tn",885,18 Nov 2020

#108,"'aurothiomalate'/de OR '(1, 2 dicarboxyethylthio) gold disodium' OR '(1, 2 dicarboxyethylthio) gold disodium salt' OR 'aurothio' OR 'aurothiomalate*' OR 'aurothiomalate sodium' OR 'disodium aurothiomalate' OR 'gold mercaptosuccinate' OR 'gold sodium thiomalate*' OR 'gold thiomalate*' OR 'gold thiomalate sodium' OR 'miocrin' OR 'myochrisin' OR 'myochrisine' OR 'myochrysin' OR 'myochrysine' OR 'myocrisin' OR 'myocrysin' OR 'shiosol' OR 'sodium aurothiomalate' OR 'sodium gold thiomalate' OR 'tauredon' OR 'tauredone' OR 'thiomalate gold'",3453,18 Nov 2020

#107,"hydroxychloroquin*:ti,ab,kw,rn,tn OR hcq:ti,ab,kw,rn,tn OR '747-3-4':ti,ab,kw,rn,tn OR 8q2869cnvh*:ti,ab,kw,rn,tn OR '118-42-3':ti,ab,kw,rn,tn OR 4qwg6n8qkh*:ti,ab,kw,rn,tn OR

hydroxychlorochin*:ti,ab,kw,rn,tn OR hydrochloroquin*:ti,ab,kw,rn,tn OR hydrocloroquin*:ti,ab,kw,rn,tn OR oxychlorochin:ti,ab,kw,rn,tn OR oxychloroquin*:ti,ab,kw,rn,tn OR plaquinol:ti,ab,kw,rn,tn OR 'brn 0253894':ti,ab,kw,rn,tn OR brn0253894:ti,ab,kw,rn,tn OR 'einecs 204-249-8':ti,ab,kw,rn,tn OR 'einecs204 249 8':ti,ab,kw,rn,tn OR hidroxicloroquin*:ti,ab,kw,rn,tn OR idrossiclorochin*:ti,ab,kw,rn,tn OR oxichlorochin*:ti,ab,kw,rn,tn OR oxichloroquin*:ti,ab,kw,rn,tn OR win1258:ti,ab,kw,rn,tn OR 'win 1258':ti,ab,kw,rn,tn OR z0188:ti,ab,kw,rn,tn OR 'einecs 212-019-3':ti,ab,kw,rn,tn OR 'einecs212-019-3':ti,ab,kw,rn,tn OR erquin:ti,ab,kw,rn,tn OR quensyl:ti,ab,kw,rn,tn OR 'sn 8137':ti,ab,kw,rn,tn OR sn8137:ti,ab,kw,rn,tn",29547,18 Nov 2020

#106,"'hydroxychloroquine sulfate'/de OR '1 (7 chloro 4 quinolylamino) 3 diethylamino 2 propanol sulfate' OR '1 (7 chloro 4 quinolylamino) 3 diethylamino 2 propanol sulphate' OR '2 [[4 [(7 chloro 4 quinolinyl) amino] pentyl] (ethyl) amino] ethanol sulfate' OR '2 [[4 [(7 chloro 4 quinolinyl) amino] pentyl] (ethyl) amino] ethanol sulphate' OR '2 [[4 [(7 chloroquinolin 4 yl) amino] pentyl] (ethyl) amino] ethanol sulfate' OR '2 [[4 [(7 chloroquinolin 4 yl) amino] pentyl] (ethyl) amino] ethanol sulphate' OR '7 chloro 4 (3 diethylamino 2 hydroxypropylamino) quinoline sulfate' OR '7 chloro 4 (3 diethylamino 2 hydroxypropylamino) quinoline sulphate' OR 'dimard' OR 'erquin' OR 'evoquin' OR 'geniquin' OR 'hydroxychloroquine sulfate' OR 'hydroxychloroquine sulphate' OR 'oxiklorin' OR 'oxychloroquine sulfate' OR 'oxychloroquine sulphate' OR 'plaquenil' OR 'plaquenil sulfate' OR 'plaquenil sulphate' OR 'plaquinol' OR 'toremonil' OR 'yuma'",3641,18 Nov 2020

#105,"'hydroxychloroquine'/de OR '7 chloro 4 [4 [ethyl (2 hydroxyethyl) amino] 1 methylbutylamino] quinoline' OR '7 chloro 4 [4 [ethyl (2 hydroxyethyl) amino] 1 methylbutylamino] quinoline diphosphate' OR 'apo-hydroxychloroquine' OR 'chloroquinol' OR 'ercoquin' OR

'hydrochloroquine' OR 'hydrocloroquine' OR 'hydroxychloroquine' OR 'oxychloroquine' OR 'quensyl' OR 'sn 8137'",29719,18 Nov 2020

#104,"sasp:ti,ab,kw,rn,tn OR ssz:ti,ab,kw,rn,tn OR salicylazosulfapyridin*:ti,ab,kw,rn,tn OR 'salicyl azo sulfapyridin*':ti,ab,kw,rn,tn OR salazosulfapyridin*:ti,ab,kw,rn,tn OR salazosulfapiridin*:ti,ab,kw,rn,tn OR azulfadin*:ti,ab,kw,rn,tn OR azulfidin*:ti,ab,kw,rn,tn OR pleon:ti,ab,kw,rn,tn OR ulcol:ti,ab,kw,rn,tn OR ucine:ti,ab,kw,rn,tn OR salazopyrin*:ti,ab,kw,rn,tn OR 'ratio-sulfosalazin*':ti,ab,kw,rn,tn OR 3xc8guz6cb*:ti,ab,kw,rn,tn OR accucol:ti,ab,kw,rn,tn OR asulfidin*:ti,ab,kw,rn,tn OR azopyrin*:ti,ab,kw,rn,tn OR azosulfidin*:ti,ab,kw,rn,tn OR 'brn 0356241':ti,ab,kw,rn,tn OR brn0356241:ti,ab,kw,rn,tn OR 'ccris 4713':ti,ab,kw,rn,tn OR ccris4713:ti,ab,kw,rn,tn OR 'einecs 209-974-3':ti,ab,kw,rn,tn OR 'hsdb 3395':ti,ab,kw,rn,tn OR hsdb3395:ti,ab,kw,rn,tn OR 'nsc 203730':ti,ab,kw,rn,tn OR nsc203730:ti,ab,kw,rn,tn OR 'nsc 667219':ti,ab,kw,rn,tn OR nsc667219:ti,ab,kw,rn,tn OR reupirin:ti,ab,kw,rn,tn OR 'salazo-sulfapyridin*':ti,ab,kw,rn,tn OR salazosulfpyridin*:ti,ab,kw,rn,tn OR salazopiridazin*:ti,ab,kw,rn,tn OR salazopyridin*:ti,ab,kw,rn,tn OR salazopirin*:ti,ab,kw,rn,tn OR salipyr:ti,ab,kw,rn,tn OR 'si-88':ti,ab,kw,rn,tn OR si88:ti,ab,kw,rn,tn OR sulfasalazin*:ti,ab,kw,rn,tn OR sulphasalazin*:ti,ab,kw,rn,tn OR 'w-t sasp oral':ti,ab,kw,rn,tn OR '599-79-1':ti,ab,kw,rn,tn",28690,18 Nov 2020

#103,"'salazosulfapyridine'/de OR '4 (2 pyridylaminosulfonyl) 3` carboxy 4` hydroxyazobenzene' OR '4 hydroxy 4` (pyrid 2 ylsulfamoyl) azobenzene 3 carboxylic acid' OR '5 [4 (2 pyridylsulfamoyl) phenylazo] 2 hydroxybenzoic acid' OR '5 [para (2 pyridylsulfamoyl) phenylazo] salicylic acid' OR 'azlufidine en-tabs' OR 'azopyrin' OR 'azopyrine' OR 'azosulfidine' OR 'azulfide' OR 'azulfidina' OR 'azulfidine' OR 'azulfidine en tabs' OR 'azulfidine en-tabs' OR 'azulfidine ra' OR 'azulfin' OR

'benzosulfa' OR 'colo pleon' OR 'colo-pleon' OR 'colopleon' OR 'disalazin' OR 'gastropyrin' OR 'pleon ra' OR 'pyralin en' OR 'rorasul' OR 'rosulfant' OR 's.a.s.-500' OR 'salazine' OR 'salazo sulfapyridine' OR 'salazodin' OR 'salazopirina' OR 'salazopyridin' OR 'salazopyridine' OR 'salazopyrin' OR 'salazopyrin entabs' OR 'salazopyrin-en' OR 'salazopyrina' OR 'salazopyrine' OR 'salazopyrine ec' OR 'salazosulfa pyridine' OR 'salazosulfapyridine' OR 'salazosulfpyridine' OR 'salicyl azo sulfapyridine' OR 'salicylazosulfapyridin' OR 'salicylazosulfapyridine' OR 'salisulf' OR 'salopyr' OR 'saridine' OR 'sas 500' OR 'sulcolon' OR 'sulfasalazine' OR 'sulfasalizine' OR 'sulfosalazine' OR 'sulphasalazine' OR 'zopyrin'",26735,18 Nov 2020

#102,"'lef':ti,ab,kw",4107,18 Nov 2020

#101,"'leflunomide'/de OR '5 methyl 4` trifluoromethyl 4 isoxazolecarboxanilide' OR '5 methyl n [4 (trifluoromethyl) phenyl] 4 isoxazolecarboxamide' OR '5 methyl n [4 (trifluoromethyl) phenyl] isoxazole 4 carboxamide' OR '5 methyl n [para (trifluoromethyl) phenyl] 4 isoxazolecarboxamide' OR 'alpha, alpha, alpha trifluoro 5 methyl 4 isoxazolecarboxy para toluidide' OR 'arabloc' OR 'arava' OR 'hwa 486' OR 'hwa486' OR 'leflunomid*' OR 'leflunomide winthrop' OR 'n (4 trifluoromethylphenyl) 5 methylisoxazole 4 carboxamide' OR 'repso' OR 'rs 34821' OR 'rs34821' OR 'su 101' OR 'su101' OR 'hsdb 7289' OR hsdb7289 OR g162gk9u4w* OR '75706-12-6'",12968,18 Nov 2020

#100,"'mtx':ti,ab,kw,rn,tn OR methotrex*:ti,ab,kw,rn,tn OR metotrexat*:ti,ab,kw,rn,tn OR methylaminopterin*:ti,ab,kw,rn,tn OR amet$opterin*:ti,ab,kw,rn,tn OR brimexate:ti,ab,kw,rn,tn OR 'a-met$opterin*':ti,ab,kw,rn,tn OR 'a-methpterin*':ti,ab,kw,rn,tn OR 'ai3-25299':ti,ab,kw,rn,tn OR ai325299:ti,ab,kw,rn,tn OR 'alpha-methopterin':ti,ab,kw,rn,tn OR farmitrexat*:ti,ab,kw,rn,tn OR fauldexato:ti,ab,kw,rn,tn OR hdmtx:ti,ab,kw,rn,tn OR metotressat*:ti,ab,kw,rn,tn OR

methotrate*:ti,ab,kw,rn,tn OR methohexate*:ti,ab,kw,rn,tn OR mexat*:ti,ab,kw,rn,tn OR metrotex:ti,ab,kw,rn,tn OR metatrexato*:ti,ab,kw,rn,tn OR metatrexan:ti,ab,kw,rn,tn OR maxtrex:ti,ab,kw,rn,tn OR meticil:ti,ab,kw,rn,tn OR metoject:ti,ab,kw,rn,tn OR metotrexin:ti,ab,kw,rn,tn OR lumexon:ti,ab,kw,rn,tn OR r$eumatrex*:ti,ab,kw,rn,tn OR tremetex:ti,ab,kw,rn,tn OR trexeron:ti,ab,kw,rn,tn OR trixilem:ti,ab,kw,rn,tn OR 'mpi-2505':ti,ab,kw,rn,tn OR mpi2505:ti,ab,kw,rn,tn OR amethopterin*:ti,ab,kw,rn,tn OR '133073-73-1':ti,ab,kw,rn,tn OR '15475-56-6':ti,ab,kw,rn,tn OR 3ig1e710zn*:ti,ab,kw,rn,tn OR yl5fz2y5u1*:ti,ab,kw,rn,tn OR 'ccris 1109':ti,ab,kw,rn,tn OR ccris1109:ti,ab,kw,rn,tn OR 'emt 25299':ti,ab,kw,rn,tn OR emt25299:ti,ab,kw,rn,tn OR 'r-9985':ti,ab,kw,rn,tn OR r9885:ti,ab,kw,rn,tn OR 'x-133':ti,ab,kw,rn,tn OR 'wr-19039':ti,ab,kw,rn,tn OR wr19039:ti,ab,kw,rn,tn",190962,18 Nov 2020

#99,"'methotrexate'/de OR '2 [[4 [[(2, 4 diamino 6 pteridinyl) methyl] (methyl) amino] benzoyl] amino] pentanedioic acid' OR '2 [[4 [[(2, 4 diaminopteridin 6 yl) methyl] (methyl) amino] benzoyl] amino] pentanedioic acid' OR '4 amino 10 methylfolic acid' OR '4 amino 10 methylpteroylglutamic acid' OR '4 amino n10 methylpteroylglutamic acid' OR 'mtx' OR 'a methopterine' OR 'abitrexate' OR 'amethopterin' OR 'amethopterine' OR 'ametopterine' OR 'antifolan' OR 'biotrexate' OR 'canceren' OR 'cl 14377' OR 'cl14377' OR 'emtexate' OR 'emthexat' OR 'emthexate' OR 'emtrexate' OR 'enthexate' OR 'farmitrexat' OR 'farmitrexate' OR 'farmotrex' OR 'folex' OR 'folex pfs' OR 'ifamet' OR 'imeth' OR 'intradose mtx' OR 'jylamvo' OR 'lantarel' OR 'ledertrexate' OR 'maxtrex' OR 'metex' OR 'methoblastin' OR 'methohexate' OR 'methotrate' OR 'methotrexat' OR 'methotrexat ebewe' OR 'methotrexate' OR 'methotrexate lpf' OR 'methotrexate preservative free' OR 'methotrexate sodium' OR 'methotrexate sodium preservative free' OR 'methotrexato' OR 'methoxtrexate' OR

'methrotrexate' OR 'methylaminopterin' OR 'methylaminopterine' OR 'meticil' OR 'metoject' OR 'metothrexate' OR 'metothrexate sodium' OR 'metotrexat' OR 'metotrexate' OR 'metotrexin' OR 'metrex' OR 'mexate' OR 'mexate-aq' OR 'mexate-aq preserved' OR 'mpi 5004' OR 'mpi5004' OR 'n [4 [(2, 4 diamino 6 pteridylmethyl) methylamino] benzoyl] glutamic acid' OR 'neotrexate' OR 'nordimet' OR 'novatrex' OR 'nsc 740' OR 'nsc740' OR 'otrexup' OR 'otrexup pfs' OR 'rasuvo' OR 'reditrex' OR 'reumatrex' OR 'rheumatrex' OR 'rheumatrex dose pack' OR 'sodium methotrexate' OR 'texate' OR 'texate-t' OR 'texorate' OR 'trexall' OR 'xaken' OR 'xatmep' OR 'zexate'",191230,18 Nov 2020

#98,"(('disease modifying' NEAR/3 (antir$eum* OR 'anti r$eum*') NEAR/3 (drug$ OR agent$)):ti,ab,kw) OR (('disease modifying' NEAR/3 (antir$eum* OR 'anti r$eum*') NEAR/3 (drug$ OR agent$)):tt)",10967,18 Nov 2020

#97,"dmard*:ti,ab,kw OR csdmard*:ti,ab,kw OR 'cs-dmard*':ti,ab,kw OR sdmard*:ti,ab,kw OR 's-dmard*':ti,ab,kw OR tsdmard*:ti,ab,kw OR 'ts-dmard*':ti,ab,kw OR bdmard*:ti,ab,kw OR 'b-dmard*':ti,ab,kw OR dmard*:tt OR csdmard*:tt OR 'cs-dmard*':tt OR sdmard*:tt OR 's-dmard*':tt OR tsdmard*:tt OR 'ts-dmard*':tt OR bdmard*:tt OR 'b-dmard*':tt",17411,18 Nov 2020

#96,"'disease modifying antirheumatic drug'/de OR 'disease modifying antirheumatic agent' OR 'disease modifying antirheumatic drug' OR 'disease modifying antirheumatic drugs'",20074,18 Nov 2020

#95,"'mavrilimumab'/de OR 'mavrilimumab*' OR '1085337 57 0' OR 1158jdp9a* OR 'cam 3001' OR cam3001",129,18 Nov 2020

#94,"'ustekinumab'/de OR 'cnto 1275' OR 'cnto1275' OR 'monoclonal antibody cnto 1275' OR 'stelara' OR 'ustekinumab*' OR '815610 63 0' OR fu77b4u5z0* OR 'stelera'",7677,18 Nov 2020

#93,"'guselkumab'/de OR 'cnto 1959' OR 'cnto1959' OR 'guselkumab' OR 'tremfya' OR '1350289 85 8' OR 089658a12d*",825,18 Nov 2020

#92,"'brodalumab'/de OR 'amg 827' OR 'amg827' OR 'brodalumab*' OR 'kyntheum' OR 'siliq' OR 'khk 4827' OR khk4827 OR '1174395 19 7' OR 6za31y954z*",1168,18 Nov 2020

#91,"'ixekizumab'/de OR 'ixekizumab' OR 'ly 2439821' OR 'ly2439821' OR 'taltz' OR '1329632 62 3' OR '1143503 69 8' OR bty153760o*",1947,18 Nov 2020

#90,"'secukinumab'/de OR 'ain 457' OR 'ain457' OR 'cosentyx' OR 'secukinumab*' OR '1229022 83 6' OR dlg4eml025*",4049,18 Nov 2020

#89,"'pateclizumab'/de OR 'mlta 3698a' OR 'mlta3698a' OR 'pateclizumab' OR 'pro 283698' OR 'pro283698' OR '12202526 59 7' OR qok1yyh7j2* OR 'rg 7415' OR rg7415",28,18 Nov 2020

#88,"'tabalumab'/de OR 'ly 2127399' OR 'ly2127399' OR 'tabalumab*' OR '1143503 67 6' OR pqp8vh3mjw*",272,18 Nov 2020

#87,"abatacept*:ti,ab,kw,rn,tn OR 'ctla 4 ig':ti,ab,kw,rn,tn OR 'ctla-igg4m':ti,ab,kw,rn,tn OR 'ctla4-fc':ti,ab,kw,rn,tn OR '332348 12 6':ti,ab,kw,rn,tn OR 7d0yb67s97*:ti,ab,kw,rn,tn OR 'rg 2077':ti,ab,kw,rn,tn OR rg2077:ti,ab,kw,rn,tn OR 'rg-1046':ti,ab,kw,rn,tn OR rg1046:ti,ab,kw,rn,tn OR nulojix:ti,ab,kw,rn,tn OR lea29y:ti,ab,kw,rn,tn OR 'lea 29y':ti,ab,kw,rn,tn OR 'ctl4 fc':ti,ab,kw,rn,tn OR ctl4fc:ti,ab,kw,rn,tn OR belatacept:ti,ab,kw,rn,tn OR 'bms 224818':ti,ab,kw,rn,tn OR bms224818:ti,ab,kw,rn,tn OR 'cytotoxic t lymphocyte associat* antigen 4 immunglobulin*':ti,ab,kw,rn,tn",11011,18 Nov 2020

#86,"'abatacept'/de OR 'ctla4 ig' OR 'ctla4 immunoglobulin' OR 'ctla4 immunoglobulin g' OR 'ctla4ig' OR 'abatacept' OR 'bms 188667' OR 'bms188667' OR 'orencia'",11011,18 Nov 2020

#85,"'ofatumumab'/de OR 'humax cd20' OR 'humax-cd20' OR 'humaxcd20' OR 'arzerra' OR 'gsk 1841157' OR 'gsk1841157' OR 'humac cd20' OR 'ofatumumab*' OR 'omb 157' OR 'omb157' OR 'humax-cd20-2f2' OR '679818 59 8' OR m95kg522r0* OR 'hsdb 8170' OR hsdb8170",3079,18 Nov 2020

#84,"'rituximab'/de OR 'abp 798' OR 'abp798' OR 'blitzima' OR 'ct p10' OR 'ctp10' OR 'gp 2013' OR 'gp2013' OR 'hlx 01' OR 'hlx01' OR 'idec 102' OR 'idec c2b8' OR 'idec102' OR 'idecc2b8' OR 'mabthera' OR 'mab thera' OR 'mk 8808' OR 'mk8808' OR 'monoclonal antibody idec c2b8' OR 'pf 05280586' OR 'pf 5280586' OR 'pf05280586' OR 'pf5280586' OR 'r 105' OR 'r105' OR 'reditux' OR 'rg 105' OR 'rg105' OR 'ritemvia' OR 'ritumax' OR 'rituxan' OR 'rituximab*' OR 'rituximab abbs' OR 'rituximab pvvr' OR 'rituximab-abbs' OR 'rituximab-pvvr' OR 'rituxin' OR 'rituzena' OR 'rixathon' OR 'riximyo' OR 'ro 452294' OR 'ro452294' OR 'ruxience' OR 'truxima' OR 'tuxella' OR '174722 31 7' OR 4f4x42syq6* OR 'hsdb 7455' OR hsdb7455",85892,18 Nov 2020

#83,"'czp':ti,ab,kw,rn,tn OR '428863 50 7':ti,ab,kw,rn,tn OR g6adw90r16*:ti,ab,kw,rn,tn OR umd07x179e*:ti,ab,kw,rn,tn OR 'hsdb 7848':ti,ab,kw,rn,tn OR hsdb7848:ti,ab,kw,rn,tn",6864,18 Nov 2020

#82,"'certolizumab pegol'/de OR 'cdp 870' OR 'cdp870' OR 'certolizumab pegol' OR 'cimzia' OR 'pegylated tumor necrosis factor alpha antibody fab fragment' OR 'pegylated tumour necrosis factor alpha antibody fab fragment' OR 'pha 738144' OR 'pha738144'",6943,18 Nov 2020

#81,"'golimumab'/de OR 'cnto 148' OR 'cnto148' OR 'golimumab*' OR 'simponi' OR 'simponi aria' OR '476181 74 5' OR 91x1klu43e*",7360,18 Nov 2020

#80,"adalimumab*:ti,ab,kw,rn,tn OR fys6t7f842*:ti,ab,kw,rn,tn OR '331731 18 1':ti,ab,kw,rn,tn OR d2e7:ti,ab,kw,rn,tn OR 'hsdb 7851':ti,ab,kw,rn,tn OR hsdb7851:ti,ab,kw,rn,tn OR 'bcd-

057':ti,ab,kw,rn,tn OR bcd057:ti,ab,kw,rn,tn OR 'chs-1420':ti,ab,kw,rn,tn OR chs1420:ti,ab,kw,rn,tn OR 'sb-5':ti,ab,kw,rn,tn OR sb5:ti,ab,kw,rn,tn",35405,18 Nov 2020

#79,"'adalimumab'/de OR 'abp 501' OR 'abp501' OR 'abrilada' OR 'abt d2e7' OR 'abtd2e7' OR 'adalimumab' OR 'adalimumab adaz' OR 'adalimumab adbm' OR 'adalimumab afzb' OR 'adalimumab atto' OR 'adalimumab beta' OR 'adalimumab bwwd' OR 'adalimumab-adaz' OR 'adalimumab-adbm' OR 'adalimumab-afzb' OR 'adalimumab-atto' OR 'adalimumab-bwwd' OR 'adaly' OR 'amgevita' OR 'amjevita' OR 'amsparity' OR 'avt 02' OR 'avt02' OR 'bat 1406' OR 'bat1406' OR 'bax 2923' OR 'bax 923' OR 'bax2923' OR 'bax923' OR 'bi 695501' OR 'bi695501' OR 'chs 1420' OR 'chs1420' OR 'cinnora' OR 'ct p17' OR 'ctp17' OR 'cyltezo' OR 'da 3113' OR 'da3113' OR 'dmb 3113' OR 'dmb3113' OR 'exemptia' OR 'fkb 327' OR 'fkb327' OR 'fyzoclad' OR 'gp 2017' OR 'gp2017' OR 'hadlima' OR 'halimatoz' OR 'hefiya' OR 'hlx 03' OR 'hlx03' OR 'hulio' OR 'humira' OR 'hyrimoz' OR 'ibi 303' OR 'ibi303' OR 'idacio' OR 'imraldi' OR 'kromeya' OR 'lu 200134' OR 'lu200134' OR 'm 923' OR 'm923' OR 'mabura' OR 'monoclonal antibody d2e7' OR 'msb 11022' OR 'msb11022' OR 'ons 3010' OR 'ons3010' OR 'pf 06410293' OR 'pf 6410293' OR 'pf06410293' OR 'pf6410293' OR 'raheara' OR 'solymbic' OR 'trudexa' OR 'zrc 3197' OR 'zrc3197'",35338,18 Nov 2020

#78,"etanercept*:ti,ab,kw,tn,rn OR 'rhu tnfr fc':ti,ab,kw,tn,rn OR 'tnfr-immunoadhesin':ti,ab,kw,tn,rn OR 'tnt receptor fusion protein':ti,ab,kw,tn,rn OR 'tum$or necrosis factor receptor fc fusion protein':ti,ab,kw,tn,rn OR 'tnf receptor fusion protein':ti,ab,kw,tn,rn OR 'tnfr fc':ti,ab,kw,tn,rn OR 'recombinant human tnf':ti,ab,kw,tn,rn OR 'recombinant human dimeric tnf receptor type ii igg fusion protein':ti,ab,kw,tn,rn OR 'dwp-422':ti,ab,kw,tn,rn OR dwp422:ti,ab,kw,tn,rn OR 'hd-203':ti,ab,kw,tn,rn OR hd203:ti,ab,kw,tn,rn OR 'lbec

0101':ti,ab,kw,tn,rn OR lbec0101:ti,ab,kw,tn,rn OR 'sb 4':ti,ab,kw,tn,rn OR 'sb4':ti,ab,kw,tn,rn",16014,18 Nov 2020

#77,"'etanercept'/de OR 'avent' OR 'benepali' OR 'brenzys' OR 'chs 0214' OR 'chs0214' OR 'embrel' OR 'enbrel' OR 'enerceptan' OR 'enia 11' OR 'enia11' OR 'erelzi' OR 'etanercept*' OR 'etanercept szzs' OR 'etanercept ykro' OR 'etanercept-szzs' OR 'etanercept-ykro' OR 'eticovo' OR 'gp 2015' OR 'gp 2015c' OR 'gp2015' OR 'gp2015c' OR 'hd 203' OR 'hd203' OR 'infinitam' OR 'lbec 0101' OR 'lbec0101' OR 'lifmior' OR 'nepexto' OR 'opinercept' OR 'recombinant tumor necrosis factor receptor fc fusion protein' OR 'recombinant tumour necrosis factor receptor fc fusion protein' OR 'tnr 001' OR 'tnr001' OR 'tumor necrosis factor receptor fc fusion protein' OR 'tumour necrosis factor receptor fc fusion protein' OR 'tunex' OR 'ylb 113' OR 'ylb113' OR '185243 69 0' OR 'op401g7ojc*'",33157,18 Nov 2020

#76,"ifx:ti,ab,kw,de,tn,rn OR 'mab ca2':ti,ab,kw,de,tn,rn OR 'monoclonal antibody ca2':ti,ab,kw,de,tn,rn OR 'antibody ca2 monoclonal':ti,ab,kw,de,tn,rn OR 'ca2 monoclonal antibody':ti,ab,kw,de,tn,rn OR 'ct p13':ti,ab,kw,de,tn,rn OR ctp13:ti,ab,kw,de,tn,rn OR 'ct-p-13':ti,ab,kw,de,tn,rn OR sb2:ti,ab,kw,de,tn,rn OR 'hsdb 7850':ti,ab,kw,de,tn,rn OR hsdb7850:ti,ab,kw,de,tn,rn",5755,18 Nov 2020

#75,"'infliximab'/de OR 'abp 710' OR 'abp710' OR 'avakine' OR 'avsola' OR 'flixabi' OR 'gp 1111' OR 'gp1111' OR 'inflectra' OR 'infliximab*' OR 'infliximab abda' OR 'infliximab axxq' OR 'infliximab dyyb' OR 'infliximab qbtx' OR 'infliximab-abda' OR 'infliximab-axxq' OR 'infliximab-dyyb' OR 'infliximab-qbtx' OR 'ixifi' OR 'pf 06438179' OR 'pf 6438179' OR 'pf06438179' OR 'pf6438179' OR 'remicade' OR 'remsima' OR 'renflexis' OR 'revellex' OR 'ta 650' OR 'ta650' OR 'zessly' OR '170277 31 3' OR 'b72hh48flu*' OR 'inflecta'",52703,18 Nov 2020

#74,"((('il-1ra' OR il1ra OR 'il-1-ra') NEAR/10 (antagonist* OR 'blocking agent*' OR blocker* OR inhibitor* OR anakinra*)):ti,ab,kw,tn) AND (therap*:ti,ab,kw,de OR treat*:ti,ab,kw,de OR trial$:ti,ab,kw,de)",2220,18 Nov 2020

#73,"'anakinra'/de OR 'anakinra*' OR 'kineret' OR 'recombinant interleukin 1 receptor antagonist' OR 'recombinant interleukin 1 receptor blocker' OR 'recombinant interleukin 1 receptor blocking agent' OR '143090 92 0'",9224,18 Nov 2020

#72,"#61 AND #71",10749,18 Nov 2020

#71,"#62 OR #63 OR #64 OR #65 OR #66 OR #67 OR #68 OR #69 OR #70",30096,18 Nov 2020

#70,"'elsilimomab'/de OR 'b e8' OR 'elsilimomab*' OR '468715 71 1'",38,18 Nov 2020

#69,"'ziltivekimab'/de OR 'cor 001' OR 'cor001' OR 'ziltivekimab*' OR ziltivekimab* OR zilti OR '2226654 05 1'",4,18 Nov 2020

#68,"'siltuximab'/de OR 'cnto 328' OR 'cnto328' OR 'monoclonal antibody cnto 328' OR 'monoclonal antibody cnto328' OR 'siltuximab*' OR 'sylvant' OR cllb8 OR 'ccib8 monoclonal antibody' OR t4h8fma7im* OR '541502 14 1'",851,18 Nov 2020

#67,"'sirukumab'/exp OR 'cnto 136' OR 'cnto136' OR 'plivensia' OR 'sirukumab*' OR '1194585 53 9' OR 640443fu93*",320,18 Nov 2020

#66,"'clazakizumab'/de OR 'ald 518' OR 'ald518' OR 'bms 945429' OR 'bms945429' OR 'clazakizumab*' OR '1236278 28 6' OR 4s38z8ra9o*",216,18 Nov 2020

#65,"'olokizumab'/de OR 'olokizumab*' OR 'cdp 6038' OR cdp6038 OR '1007223 17 7' OR pai71r1d2w*",95,18 Nov 2020

#64,"'levilimab'/de OR 'bcd 089' OR 'bcd089' OR 'levilimab*' OR '2035008 70 7' OR p7uv3l2h80*",5,18 Nov 2020

#63,"'sarilumab'/de OR 'kevzara*' OR 'regn 88' OR 'regn88' OR 'sar 153191' OR 'sar153191' OR 'sarilumab*' OR '1189541 98 7' OR nu90v55f8i*",810,18 Nov 2020

#62,"'tocilizumab'/de OR 'actemra*' OR 'actemra 200' OR 'atlizumab*' OR 'lusinex*' OR 'r1569' OR 'roactemra*' OR 'tocilizumab*' OR r1569 OR 'r 1569' OR 'mra':ti,ab,kw,tn OR mra:tt OR '375823 41 9' OR i031v2h011* OR 'rhpm-1' OR rhpm1 OR 'rg-1569' OR rg1569 OR 'msb-11456' OR msb11456 OR 'ro-4877533' OR ro4877533",29019,18 Nov 2020

#61,"#1 OR #2 OR #3 OR #4 OR #5 OR #6 OR #7 OR #8 OR #9 OR #10 OR #11 OR #12 OR #13 OR #14 OR #15 OR #16 OR #22 OR #23 OR #24 OR #25 OR #26 OR #27 OR #28 OR #29 OR #30 OR #31 OR #32 OR #33 OR #34 OR #35 OR #36 OR #37 OR #38 OR #39 OR #40 OR #41 OR #42 OR #43 OR #44 OR #45 OR #46 OR #47 OR #48 OR #49 OR #50 OR #51 OR #54 OR #55 OR #56 OR #57 OR #58 OR #59 OR #60",323204,18 Nov 2020

#60,"(('lymph* node' NEAR/3 hyperplas* NEAR/3 giant):ti,ab,kw) OR (('lymph* node' NEAR/3 hyperplas* NEAR/3 giant):tt)",309,18 Nov 2020

#59,"((angiofollicul* OR 'angio follicul*') NEAR/3 lymph* NEAR/3 hyperplas*):tt",12,18 Nov 2020

#58,"((angiofollicul* OR 'angio follicul*') NEAR/3 lymph* NEAR/3 hyperplas*):ti,ab,kw",409,18 Nov 2020

#57,"(castleman* NEAR/3 (disease* OR tumo$r* OR 'pseudo tumo$r*' OR pseudotumo$r* OR lymph* OR pseudolymphom* OR 'pseudo lymphom*')):tt",42,18 Nov 2020

#56,"(castleman* NEAR/3 (disease* OR tumo$r* OR 'pseudo tumo$r*' OR pseudotumo$r* OR lymph* OR pseudolymphom* OR 'pseudo lymphom*')):ti,ab,kw",4589,18 Nov 2020

#55,"'angiofollicular lymph node hyperplasia'/de",5350,18 Nov 2020

#54,"#52 NOT #53",1298,18 Nov 2020

#53,"(('chronic sinusitis' OR 'chronic rhinosinusitis' OR 'cytoreductive surg*' OR 'complete response' OR 'complete responders') NEAR/6 crs):ti,ab,kw",5798,18 Nov 2020

#52,"(crs:ti,ab,kw OR crs:tt) AND ('cytokine releas*':ti,ab,kw,de OR 'cytokine storm':ti,ab,kw,de)",1355,18 Nov 2020

#51,"((('cytokine releas*' OR 'cytokine storm') NEAR/3 syndrom*):ti,ab,kw,de) OR ((('cytokine releas*' OR 'cytokine storm') NEAR/3 syndrom*):tt)",3999,18 Nov 2020

#50,"'cytokine release syndrome'/de",3347,18 Nov 2020

#49,"(('brachiocephal*' NEAR/6 arterit*):ti,ab,kw) OR (('brachiocephal*' NEAR/6 arterit*):tt)",29,18 Nov 2020

#48,"(('young female' NEAR/6 arterit*):ti,ab,kw) OR (('young female' NEAR/6 arterit*):tt)",29,18 Nov 2020

#47,"(takayasu* NEAR/3 (arterit* OR syndrom* OR disease*)):tt",255,18 Nov 2020

#46,"(takayasu* NEAR/3 (arterit* OR syndrom* OR disease*)):ti,ab,kw",6467,18 Nov 2020

#45,"'aortic arch syndrome'/de",9131,18 Nov 2020

#44,"(gca:ti,ab,kw OR gca:tt) AND (arterit*:ti,ab,kw,de OR vasculit*:ti,ab,kw,de OR r$eum*:ti,ab,kw,de,jt)",3569,18 Nov 2020

#43,"(('large vessel' NEAR/3 (vasculit* OR arterit*)):ti,ab,kw) OR (('large vessel' NEAR/3 (vasculit* OR arterit*)):tt)",2118,18 Nov 2020

#42,"'large vessel vasculitis'/de",233,18 Nov 2020

#41,"'temporal arteritis'/de",4254,18 Nov 2020

#40,"((horton OR 'horton s' OR hortons) NEAR/3 disease*):ti,ab,kw",578,18 Nov 2020

#39,"((temporal OR cranial) NEAR/2 arterit*):ti,ab,kw",3148,18 Nov 2020

#38,"('giant cell' NEAR/6 (arterit* OR aortit* OR horton*)):ti,ab,kw",7687,18 Nov 2020

#37,"'giant cell arteritis'/de",8282,18 Nov 2020

#36,"(aosd:ti,ab,kw OR aosd:tt) AND (inflammator*:ti,ab,kw,de OR autoinflammator*:ti,ab,kw,de OR r$eumat*:ti,ab,kw,de,jt)",963,18 Nov 2020

#35,"'still s disease*':tt OR 'stll s disease*':tt OR 'stills disease*':tt OR 'stlls disease*':tt",5,18 Nov 2020

#34,"'still s disease*':ti,ab,kw OR 'stll s disease*':ti,ab,kw OR 'stills disease*':ti,ab,kw OR 'stlls disease*':ti,ab,kw",3023,18 Nov 2020

#33,"(('stills disease*' OR 'still s disease*' OR 'still disease*') NEAR/6 (adult* OR 'adult onset')):tt",5,18 Nov 2020

#32,"(('stills disease*' OR 'still s disease*' OR 'still disease*') NEAR/6 (adult* OR 'adult onset')):ti,ab,kw",2656,18 Nov 2020

#31,"'adult onset still disease'/de",2279,18 Nov 2020

#30,"(polyart*:tt OR oligoart*:tt OR 'poly art*':tt OR 'oligo art*':tt) AND (jia:tt OR 'juvenile idiopathic arthritis':tt OR 'juvenile onset idiopathic arthritis':tt) OR pjia:tt",1,18 Nov 2020

#29,"(polyart*:ti,ab,kw OR oligoart*:ti,ab,kw OR 'poly art*':ti,ab,kw OR 'oligo art*':ti,ab,kw) AND (jia:ti,ab,kw OR 'juvenile idiopathic arthritis':ti,ab,kw OR 'juvenile onset idiopathic arthritis':ti,ab,kw) OR pjia:ti,ab,kw",3365,18 Nov 2020

#28,"(('stills disease*' OR 'still s disease*' OR 'still disease*') NEAR/6 child*):tt",0,18 Nov 2020

#27,"(('stills disease*' OR 'still s disease*' OR 'still disease*') NEAR/6 child*):ti,ab,kw",89,18 Nov 2020

#26,"('stills disease*':tt OR 'still s disease*':tt OR 'still disease*':tt) AND juvenile*:tt",1,18 Nov 2020

#25,"('stills disease*':ti,ab,kw OR 'still s disease*':ti,ab,kw OR 'still disease*':ti,ab,kw) AND juvenile*:ti,ab,kw",514,18 Nov 2020

#24,"((polyarthr* OR polyartrit* OR 'poly arthrit*' OR 'poly artrit*' OR oligoarthrit* OR oligoartrit* OR 'oligo arthrit*' OR 'oligo artrit*') NEAR/6 juvenile*):tt",80,18 Nov 2020

#23,"((polyarthr* OR polyartrit* OR 'poly arthrit*' OR 'poly artrit*' OR oligoarthrit* OR oligoartrit* OR 'oligo arthrit*' OR 'oligo artrit*') NEAR/6 juvenile*):ti,ab,kw",326,18 Nov 2020

#22,"#20 AND #21",9195,18 Nov 2020

#21,"systemic:ti,ab,kw OR polyart*:ti,ab,kw OR 'poly art*':ti,ab,kw OR oligoart*:ti,ab,kw OR 'oligo art*':ti,ab,kw OR systemic:tt OR polyart*:tt OR 'poly art*':tt OR oligoart*:tt OR 'oligo art*':tt",718275,18 Nov 2020

#20,"#17 OR #18 OR #19",24662,18 Nov 2020

#19,"jia:ti OR jia:tt",1258,18 Nov 2020

#18,"(((arthrit* OR artrit*) NEAR/6 juvenile*):ti,ab,kw) OR (((arthrit* OR artrit*) NEAR/6 juvenile*):tt)",17869,18 Nov 2020

#17,"'juvenile rheumatoid arthritis'/de",21365,18 Nov 2020

#16,"((systemic NEAR/6 (jia OR 'juvenile idiopathic arthritis' OR 'juvenile onset idiopathic arthriti')):ti,ab,kw) OR sjia:ti,ab,kw",3323,18 Nov 2020

#15,"'systemic juvenile idiopathic arthritis'/de",1399,18 Nov 2020

#14,"'rheumatic disease'/de",50350,18 Nov 2020

#13,"((rheumat* OR reumat* OR revmat* OR rheumo OR reumo) NEAR/2 (condition* OR diseas*)):tt",13,18 Nov 2020

#12,"((rheumat* OR reumat* OR revmat* OR rheumo OR reumo) NEAR/2 (condition* OR diseas*)):ti,ab,kw",50782,18 Nov 2020

#11,"((r$eum* OR r$evm*) NEAR/2 (chronic* OR articular*)):tt",87,18 Nov 2020

#10,"((r$eum* OR r$evm*) NEAR/2 (chronic* OR articular*)):ti,ab,kw",5684,18 Nov 2020

#9,"(inflammator* NEAR/2 (arthrit* OR artrit*)):tt",3,18 Nov 2020

#8,"(inflammator* NEAR/2 (arthrit* OR artrit*)):ti,ab,kw",12368,18 Nov 2020

#7,"(chronic* NEAR/2 (polyarthrit* OR 'poly arthrit*' OR polyartrit* OR 'poly artrit*')):tt",86,18 Nov 2020

#6,"(chronic* NEAR/2 (polyarthrit* OR 'poly arthrit*' OR polyartrit* OR 'poly artrit*')):ti,ab,kw",2521,18 Nov 2020

#5,"r$eumarthrit*:tt OR r$eumartrit*:tt OR 'r$eum arthrit*':tt OR 'r$eum artrit*':tt OR r$evmarthrit*:tt OR r$evmartrit*:tt OR 'r$evm arthrit*':tt OR 'r$evm artrit*':tt OR 'r$eum polyarthrit*':tt OR 'r$eum polyartrit*':tt OR 'r$eum poly arthrit*':tt OR 'r$eum poly artrit*':tt OR r$eumpolyarthrit*:tt OR r$eumpolyartrit*:tt",0,18 Nov 2020

#4,"r$eumarthrit*:ti,ab,kw OR r$eumartrit*:ti,ab,kw OR 'r$eum arthrit*':ti,ab,kw OR 'r$eum artrit*':ti,ab,kw OR r$evmarthrit*:ti,ab,kw OR r$evmartrit*:ti,ab,kw OR 'r$evm arthrit*':ti,ab,kw OR 'r$evm artrit*':ti,ab,kw OR 'r$eum polyarthrit*':ti,ab,kw OR 'r$eum polyartrit*':ti,ab,kw OR 'r$eum poly arthrit*':ti,ab,kw OR 'r$eum poly artrit*':ti,ab,kw OR r$eumpolyarthrit*:ti,ab,kw OR r$eumpolyartrit*:ti,ab,kw",10,18 Nov 2020

#3,"((rheumat* OR reumat* OR revmat* OR rheumo OR reumo) NEAR/3 (arthrit* OR artrit* OR polyarthrit* OR polyartrit*)):tt",6608,18 Nov 2020

#2,"((rheumat* OR reumat* OR revmat* OR rheumo OR reumo) NEAR/3 (arthrit* OR artrit* OR polyarthrit* OR polyartrit*)):ti,ab,kw",171077,18 Nov 2020

#1,"'rheumatoid arthritis'/de",198935,18 Nov 2020

EBM-Reviews incl. CDSR (OVID)

IL-6 inhibitiors//RA, sJIA, pJIA, adult Still’s disease, GCA, CRS, Castleman’s disease//PY=2012-2020 (nicht möglich für CDSR)

Search Strategy (11 November 2020)

Database: EBM Reviews - Cochrane Database of Systematic Reviews <2005 to November 04, 2020>, EBM Reviews - ACP Journal Club <1991 to October 2020>, EBM Reviews - Cochrane Clinical Answers <October 2020>, EBM Reviews - Database of Abstracts of Reviews of Effects <1st Quarter 2016>

Search Strategy:

----

1 ((rheumat* or reumat* or revmat* or rheumo or reumo) adj3 (arthrit* or artrit* or polyarthrit* or polyartrit*)).ti,ot,ab,kw,ct. (558)

2 (r?eumarthrit* or r?eumartrit* or r?eum arthrit* or r?eum artrit* or r?evmarthrit* or r?evmartrit* or r?evm arthrit* or r?evm artrit* or r?eum polyarthrit* or r?eum polyartrit* or r?eum poly arthrit* or r?eum poly artrit* or r?eumpolyarthrit* or r?eumpolyartrit*).ti,ot,ab,kw,ct. (0)

3 (chronic* adj2 (polyarthrit* or poly arthrit* or polyartrit* or poly artrit*)).ti,ot,ab,kw,ct. (1)

4 (inflammator* adj2 (arthrit* or artrit*)).ti,ot,ab,kw,ct. (40)

5 ((r?eum* or r?evm*) adj2 (chronic* adj2 articular*)).ti,ot,ab,kw,ct. (0)

6 ((rheumat* or reumat* or revmat* or rheumo or reumo) adj2 (condition* or diseas*)).ti,ot,ab,kw,ct. (151)

7 ((arthrit* or artrit*) adj6 juvenile*).ti,ot,ab,kw,ct. (34)

8 JIA.ti,ot,ab,ct. (11)

9 7 or 8 (38)

10 (systemic or polyart* or poly art* or oligoart* or oligo art*).ti,ot,ab,kw,ct. (1142)

11 9 and 10 (5)

12 ((polyarthr* or polyartrit* or poly arthrit* or poly artrit* or oligoarthrit* or oligoartrit* or oligo arthrit* or oligo artrit*) adj6 juvenile*).ti,ot,ab,kw,ct. (1)

13 (((stills or still's or still) adj disease) and juvenile*).ti,ot,ab,kw,ct. (1)

14 ((stills or still's or still) adj disease adj6 child*).ti,ot,ab,kw,ct. (0)

15 ((systemic adj6 JIA) or sJIA).ti,ot,ab,ct. (1)

16 (((polyart* or oligoart* or poly art* or oligo art) adj6 JIA) or pJIA).ti,ot,ab,ct. (0)

17 ((stills or still's or still) adj disease adj6 adult*).ti,ot,ab,kw,ct. (1)

18 (giant cell adj6 (arterit* or aortit* or horton*)).ti,ot,ab,kw,ct. (19)

19 ((temporal or cranial) adj2 arterit*).ti,ot,ab,kw,ct. (2)

20 ((horton or horton's or hortons) adj3 disease?).ti,ot,ab,kw,ct. (0)

21 (large vessel adj3 (vasculit* or arterit*)).ti,ot,ab,kw,ct. (1)

22 GCA.ti,ot,ab,ct. and (arterit* or vasculit* or rheum*).mp,jw. (5)

23 (takayasu* adj3 (arterit* or syndrome* or disease*)).ti,ot,ab,kw,ct. (7)

24 (young female adj6 arterit*).ti,ot,ab,kw,ct. (0)

25 (brachiocephal* adj3 arterit*).ti,ot,ab,kw,ct. (0)

26 ((cytokine releas* or cytokine storm) adj3 syndrome*).ti,ot,ab,kw,ct. (1)

27 crs.ti,ab,ct. and (cytokine releas* or cytokine storm).mp. (0)

28 (castleman* adj3 (disease* or tumo?r*)).ti,ot,ab,kw,ct. (0)

29 ((angiofollicul* or angio follicul*) adj3 lymph* adj3 hyperplas*).ti,ot,ab,kw,ct. (0)

30 (lymph* node adj3 hyperplas* adj3 giant).ti,ot,ab,kw,ct. (0)

31 (castleman* adj3 (disease* or tumo?r* or pseudo-tumo?r* or pseudotumo?r* or lymph* or pseudolymphom* or hyperplas*)).ti,ot,ab,kw,ct. (0)

32 1 or 2 or 3 or 4 or 5 or 6 or 11 or 12 or 13 or 14 or 15 or 16 or 17 or 18 or 19 or 20 or 21 or 22 or 23 or 24 or 25 or 26 or 27 or 28 or 29 or 30 or 31 (719)

33 (tocilizumab* or actemra* or atlizumab* or lusinex* or r1569 or r 1569 or roactemra* or mra or 375823 41 9 or i031v2h011* or rhpm-1 or rhpm1 or rg-1569 or rg1569 or msb-11456 or msb11456 or ro-4877533 or ro4877533).mp. (135)

34 (sarilumab* or kevzara* or regn 88 or regn88 or sar 153191 or sar153191 or 1189541 98 7 or nu90v55f8i*).mp. (2)

35 (levilimab* or BCD-089 or bcd089 or 2035008 70 7 or P7UV3L2H80*).mp. (0)

36 (olokizumab* or cdp 6038 or cdp6038 or 1007223 17 7 or pai71r1d2w*).mp. (1)

37 (clazakizumab* or ald 518 or ald518 or bms 945429 or bms945429 or 1236278 28 6 or 4s38z8ra9o*).mp. (1)

38 (sirukumab* or cnto 136 or cnto136 or pilvensia or 1194585 53 9 or 640443FU93*).mp. (3)

39 (siltuximab* or sylvant or cnto 328 or cnto-328 or cnto328 or cllb8 or ccib8 monoclonal antibody or sylvant or t4h8fma7im* or 541502 14 1).mp. (2)

40 (ziltivekimab* or zilti or "cor-001" or "2226654 05 1").mp. (0)

41 (elsilimomab* or "b-e8" or 468715 71 1).mp. (1)

42 33 or 34 or 35 or 36 or 37 or 38 or 39 or 40 or 41 (138)

43 32 and 42 (44)

44 (anakinra* or kineret or ((interleukin 1 receptor or il1 receptor or il 1 receptor) adj1 antagonist protein*) or (recombinant interleukin 1 receptor adj1 (antagonist* or blocker* or blocking agent*)) or antril or il 1ra or il1ra or il 1 ra or ((urin* or febrile) adj2 (interleukin 1 inhibitor* or il1 inhibitor* or il 1 inhibitor*)) or "143090 92 0" or 9013duq28k*).mp. (66)

45 (infliximab* or ifx or avakine or flixabi or inflectra or inflecta or ixifi or remicade or remsima or revellex or renflexis or zessly or mab ca2 or monoclonal antibody ca2 or antibody ca2 monoclonal or ca2 monoclonal antibody or ct p13 or ctp13 or ct-p-13 gp-1111 or gp1111 or ta-650 or ta650 or abp 710 or abp710 or bow-015 or bow015 or 170277 31 3 or b72hh48flu* or sb2 or "pf 06438179" or pf 6438179 or pf06438179 or pf6438179 or hsdb 7850 or hsdb7850).mp. (261)

46 (etanercept* or benepali or embrel or enbrel or enia 11 or enia11 or erelzi or lifmior or opinercept or recombinant tumo?r necrosis factor receptor fc fusion protein or "tnr 001" or tnr001 or tnfr-fc or tnrfr:fc or rhu tnfr:fc or rhu-tnfr:fc or tnfr-immunoadhesin or tnt receptor fusion protein or tum?or necrosis factor receptor fc fusion protein or tunex or "185243 69 0" or op401g7ojc* or brenzys or tnf receptor fusion protein or tnfr fc or recombinant human tnf or recombinant human dimeric tnf receptor type ii igg fusion protein or chs-0214 or chs0214 or dwp-422 or dwp422 or enia-11 or enia11 or gp-2015 or gp2015 or gp2015c or gp-2015c or hd-203 or hd203 or lbec-0101 or lbec0101 or sb-4 or sb4).mp. (165)

47 (adalimumab* or humira or abp 501 or abp501 or abtd2e7 or amjevita or amgevita or bax 2923 or bax2923 gp 2017 or gp2017 or halimatoz or hefiya or hulio or hyrimoz or ibi 303 or ibi303 or imraldi or m 923 or m923 or msb 11022 or msb11022 or ons3010 or ons 3010 or "pf 06410293" or pf 6410293 or pf06410293 or pf6410293 or d2e7 or truxeda or cyltezo or solymbic or fys6t7f842* or 331731 18 1 or lu 200134 or lu200134 or d2e7 or hsdb 7851 or hsdb7851 or abp-501

or abp501 or bcd-057 or bcd057 or bi-695501 or bi695501 or chs-1420 or chs1420 or gp-2017 or gp2017 or sb-5 or sb5).mp. (182)

48 (golimumab* or cnto 148 or cnto148 or simponi or 476181 74 5 or 91x1klu43e*).mp. (63)

49 (certolizumab* or cdp 870 or cdp870 or cimzia or pegylated tumor necrosis factor alpha antibody fab fragment or pegylated tumour necrosis factor alpha antibody fab fragment or pha 738144 or pha738144 or czp or 428863 50 7 or g6adw90r16* or umd07x179e* or hsdb 7848 or hsdb7848).mp. (85)

50 (rituximab* or ct p10 or ctp10 or idec 102 or idec102 or idecc2b8 or idec c2b8 or monoclonal antibody idec c2b8 or gp2013 or gp 2013 or "pf 05280586" or pf05280586 or mabthera or mab thera or r 105 or r105 or reditux or rg 105 or rg105 or rituxan or rituxin or ritemvia or rituzena or rixathon or riximyo or ro 452294 or ro452294 or truxima or 174722 31 7 or 4f4x42syq6* or hsdb 7455 or hsdb7455).mp. (190)

51 (ofatumumab* or humax cd20 or humax-cd20 or humaxcd20 or humax-cd20-2f2 or arzerra or gsk 1841157 or gsk1841157 or gski841157 or humac cd20 or omb 157 or omb157 or 679818 59 8 or m95kg522r0* or hsdb 8170 or hsdb8170).mp. (9)

52 (abatacept* or ctla4 ig or ctla4 immunglobulin or ctla4 immunoglobulin g or ctla4ig or 'ctla 4 ig' or ctla-igg4m or ctla4-fc or bms 188667 or bms188667 or orencia or '332348 12 6' or 7d0yb67s97* or rg 2077 or rg2077 or rg-1046 or rg1046 or nulojix or lea29y or lea 29y or ctl4 fc or ctl4fc or belatacept or bms 224818 or bms224818 or belatacept or cytotoxic t lymphocyte associat* antigen 4).mp. (73)

53 (tabalumab* or ly 2127399 or ly2127399 or 1143503 67 6 or pqp8vh3mjw*).mp. (0)

54 (pateclizumab* or mlta 3698a or mlta3698a or pro 283698 or pro283698 or 12202526 59 7 or qok1yyh7j2* or rg 7415 or rg7415).mp. (0)

55 (secukinumab* or cosentyx or ain 457 or ain457 or 1229022 83 6 or dlg4eml025*).mp. (16)

56 (ixekizumab* or ly 2439821 or ly2439821 or taltz or 1329632 62 3 or 1143503 69 8 or bty153760o*).mp. (8)

57 (brodalumab* or amg 827 or amg827 or khk 4827 or khk4827 or siliq or kyntheum or 1174395 19 7 or 6za31y954z*).mp. (6)

58 (guselkumab* or cnto 1959 or cnto1959 or 1350289 85 8 or tremfya or 1350289 85 8 or 089658a12d*).mp. (5)

59 (ustekinumab* or cnto 1275 or cnto1275 or stelara or "815610 63 0" or fu77b4u5z0* or 1275 cnto or 1275cnto or l04ac05 or tt-20 or tt20).mp. (43)

60 (mavrilimumab or "1085337 57 0" or 1158jdp9a* or cam 3001 or cam3001).mp. (0)

61 (dmard* or csdmard* or cs-dmard* or sdmard* or s-dmard* or tsdmard* or ts-dmard* or bdmard* or b-dmard*).ti,ot,ab,kw,ct. (75)

62 (disease modifying adj3 (antirheum* or anti rheum*) adj3 (drug? or agent?)).mp. (191)

63 ((antirheum* or anti rheum*) adj5 (drug? or agent?)).ti,ot,ab,ct. (90)

64 (mxt or methotrex* or metotrexat* or methoblastin or methylaminopterin* or amet?opterin* or abitrexate or antifolan or brimexate or a-met?opterin* or a-methpterin* or ai3-25299 or ai325299 or alpha-methopterin or biotrexate or emtexate or emtrexate* or emthexat* or enthexate* or farmitrexat* or farmotrex or fauldexato or folex or folex pfs or hdmtx or ifamet or imeth or maxtrex or metotressat* or methotrate* or methohexate* or mexat* or metrotex or metatrexato* or metex or metrex or metatrexan or maxtrex or meticil or metoject or metotrexin or novatrex* or neotrexate* or nordimet or lumexon or ledertrexate* or lanterel or rasuvo or r?eumatrex* or reditrex or rasuvo or texate or texate-t or tremetex or trexeron or trixilem or texorate or trexall or xaken or xatmep or otrexup or mpi-2505 or mpi2505 or mpi 5004 or mpi5004 or amethopterin* or 133073-73-1 or 15475-56-6 or 3ig1e710zn* or yl5fz2y5u1* or cl 14377 or cl14377 or ccris 1109 or ccris1109 or emt 25299 or emt25299 or nsc-740 or nsc740 or r-9985 or r9885 or x-133 or wr-19039 or wr19039).mp. (592)

65 (leflunomid* or hwa 486 or hwa486 or su101 or su 101 or arava or arabloc or hsdb 7289 or hsdb7289 or g162gk9u4w* or 75706-12-6 or rs 34821 or rs34821).mp. (60)

66 lef.ti,ot,ab. (0)

67 (sulfasalazin* or sasp or ssz or salicylazosulfapyridin* or salicyl azo sulfapyridin* or sulphasalazin* or salazosulfapyridin* or salazosulfapiridin* or 'pyralin en' or azulfadin* or azulfidin* or asulfidin* or azulfide* or azulfin or colo-pleon or colopleon or disalazin or gastropyrin or pleon or "pleon ra" or "pyralin en" or rorasul or rosulfant or ulcol or ucine or salazopyrin* or ratio-sulfosalazin* or sulfasalazin* or 3xc8guz6cb* or accucol or asulfidin* or azopyrin* or azosulfidin* or benzosulfa or "brn 0356241" or brn0356241 or ccris 4713 or ccris4713 or einecs 209-974-3 or hsdb 3395 or hsdb3395 or nsc 203730 or nsc203730 or nsc 667219 or nsc667219 or reupirin or rorasul or salazo-sulfapyridin* or salazosulfpyridin* or salazopiridazin* or salazopyridin* or salazopirin* or salazodin or salisulf or salipyr or saridine or si-88 or si88 or sulculon or sulfasalazin* or sulfasalizin* or sulphasalazin* or w-t sasp oral or 599-79-1 or "s.a.s. 500" or "s.a.s.-500" or sas-500 or zopyrin*).mp. (196)

68 (hydroxychloroquin* or hcq or 747-3-4 or 8q2869cnvh* or 118-42-3 or 4qwg6n8qkh* or hydroxychlorochin* or hydrochloroquin* or hydrocloroquin* or oxychlorochin or oxychloroquin* or plaquenil or plaquinol or "brn 0253894" or brn0253894 or einecs 204-249-8 or einecs204-249-8 or hidroxicloroquin* or idrossiclorochin* or oxichlorochin* or oxichloroquin* or oxiklorin or win1258 or win 1258 or z0188 or einecs 212-019-3 or einecs212-019-3 or chloroquinol or dimard or ercoquin or evoquin or erquin or geniquin or quensyl or sn 8137 or sn8137 or toremonil or yuma).mp. (79)

69 (gold sodium thiomalate* or aurothiomalate* or aurolate* or gold thiomalate* or gold disodium thiomalate* or gold thiomalic acid* or miocrin or miocrisin or monogold disodium thiomalate* or myochrysin* or myocrisin* or myocrysin* or sodium aurothiomalate* or sodium gold thiomalate* or sodium thiomalate* gold or sodium thiomalatoaurate* or tauredon or taureodon or tauredone or thiomalate gold or thiomalatoaurate sodium or 1244-57-4 or 39377-38-3 or 4846-27-9 or 74916-57-7 or e4768zy6gm* or aurothiomala-natrium or aurot?iomalato sodico or monogold* sodium salt or dinatrium 2-aurothio-succinat or einecs 235-479-7 or einecs235-479-7 or hsdb 7173 or hsdb7173 or kidon or natrii aurothiomalas* or shiosol or gold mercatpsuccinate*).mp. (6)

70 (thioglucosoaurate* or gold thioglucose* or gold thio glucose* or thioglucose gold or aureotan or solganal or solganol or gold-50 or gold50 or solganal b or b oleosum solganal or auromyose* or aurothioglucose* or auro thioglucose* or aurotan or aurumine or aurumin or authron or brenol or ccris 59 or ccris59 or einecs 235-365-7 or einecs235-365-7 or glysanol b or goldthioglucose* or

goldtioglucose* or hsdb 7174 or hsdb7174 or oronol or romosol or skf 10056 or skf10056 or 2p2v9q0e78* or 12192-57-3).mp. (5)

71 ((gold* or auro*) adj10 (inject* or intramuscular* or im or intra muscular*)).ti,ot,ab. (6)

72 (aurothiopropanol* or allochrysine or allocrisine or aurotiopoe or aurotioprol or 27279-43-2 or g7097j63e9* or auromercapto propanol sulfonate sodium or auromercaptopropanolsulfonate sodium or aurothioisopropanolsulfonate sodium or sodium aurothiopropanolsulfonate or gold sodium thiopropanol sulfonate or sodium auromercaptopropanol sulfonate).mp. (1)

73 (chloroquin* or cq or 54-04-7 or 886u3h6uff* or aralen or aralene or arechin or arechine or arequin or chingamin or chlorochin* or khingamin or nivaquine or oe48649kh6n* or amokin or amokine or anaclor or aralan or arequine or artriquine or avloclor or avoclor or arthrochin or artrichin or bemaco or bemaphata or bemaphate or bemasulph or benaquin or bipiquin or cadiquin or "brn 0482809" or brn0482809 or capquin or ccris 3439 or ccris3439 or chemochin or chemochine or chingamine or chingaminum or chloraquin* or chlorquin or chlorquine or choloquine or choroquine sulfate or choroquine sulphate or cindacin or clo-kit junior or clorichina or clorichine or cloriquine or clorochina or delagil or delagyl or diclokin or chlorofoz or cindachin or clorochina or cloroquina or cocatrit or dichinalex or diquinalex or diroquine or emquin or genocin or gontochin or gontochine or gontoquine or einecs 200-191-2 or einecs200-191-2 or elestol or gontochin or heliopar or hsdb 3029 or hsdb3029 or imagon or iroquine or klorokin or klorokine or klorokinfosfat or lagaquin or lapaquin or malaquin or malaren or malarex or malarivon or malaviron or maliaquine or maquine or mesylith or mexaquin or mirquin or neochin or nivaquin or nivachine or nivaquine b or nivaquine dp or nivaquine forte or "p roquine" or nsc 187208 or nsc187208 or pfizerquine or quensyl or quinachlor or quinercyl or quingamine or quinilon or quinoline or quinoscan or repal or resochen or resochene or resochin or resochin junior or resochina or resochine or resochinon or resoquina or resoquine or reumachlor or ronaquine or roquine or rp 3377 or rp3377 or sanoquin or sanoquine or silbesan or siragan or sirajan or sn 7618 or sn7818 or solprina or solprine or sopaquin or st 21 or tresochin or tresoquine or trochin or trochine or troquine or weimerquin or w 7618 or w7618 or win 244 or win244).mp. (147)

74 adrenal cortex hormones.mp. (595)

75 prednisolone*.mp. (662)

76 methylprednisolone*.mp. (437)

77 (prednisolone* or predonine or di-adreson-f or diadresonf or 9phq9y1olm* or prednisolona* or prednisolonum* or nsc-9120 or nsc9120 or nsc 9900 or nsc9900 or delta-dehydrocortisol* or delta-dehydrocortisone* or delta-hydrocortisone or hydroretrocortine or metacortandralone* or brn 1354103 or brn1354103 or bubbli-pred or ccris 980 or ccris980 or co-hydeltra or codelcortone or cordrol or cortalone or cotogesic or cotolone or decaprednil or decortin h or delcortol or dehydrohydrocortisone* or delta-cortef or deltacortef or delta-ef-cortelan or delta-stab or deltacortenol or deltacortril or deltahydrocortisone* or deltisilone or derpo pd or dexa-dortidelt hostacortin h or di-adreson f or dicortol or donisolone or dydeltrone or eazolin d or ec 200-021-7 or ec200-021-7 or einecs 200-021-7 or einecs200-021-7 or erbacort or erbasona or estilsona or fernisolone or fernisolone p or hostacortin h or hsdb 3385 or hsdb3385 or hydeltra or hydeltrone or hydrodeltalone or hydrodeltisone or hydroretrocortin or hydroretrocortine* or k 1557 or k1557 or lentosone or meti-drm or meticortelone or orapred or orapred odt or paracortol or paracotol or pediapred or precortancyl or precortilon or precortisyl or predne-dome or prednelan or prednicen or predniliderm or predniretard or prednis or predonin or predonine* or prelon or prelone or prenolone or rolisone or scherisolon? or solone or sterolone or ulacort or ultracorten h or ultracortene h adelcort or antisolon or antisolone or aprednisolon? or benisolon? or caberdelta or co hydeltra or codelcortone or ompresolon).mp. (664)

78 (cortadeltona or cortelinter or cortisolone or dacortin or dacortin h or dacrotin or decaprednil or decortil or delta cortril or delta hycortol or deltacortenolo or deltacortil or deltacortoil or deltaderm or deltadglycortril or deltahycortol or deltahydrocortison? or deltaophticor or deltasolone or deltastab or deltidrosol or deltisilone or deltisolon? or deltolasson? or deltosona or deltosone or depo-predate or dermosolon or dhasolone or di adreson? f or diadreson? f or dicortol or domucortone or encortelon? or encortolon or equisolon or glistelone or hefasolon or hydrelta or hydrocortancyl or hydrocortidelt or hydradeltalone or hydradeltisone or inflanefran or insolone or keteocort h or key-pred or key-pred sp or lenisolone or leocortol or liquipred or mediasolone or meprisolon? or metacortalon? or metacortelone or meti derm or metiderm or meticortelone or morlone or mydrapred or nisolon? or opredsone or panafcortelone or panafcortolone or panafort or paracortol pr phlogex or pre cortisyl or preconin or precortalon or precortancyl or precortisyl or pred ject 50 or predacort 50 or predaject 50 or predalone 50 or predartrina or predartrine or predate 50 or predeltilone or predisole or predisyr or predne dome or prednecort or prednedome or prednelan or predni coelin or predni h tablinen or predni helvacort or prednicoelin or prednicort

or prednicortelone or prednifor drops or predniment or prednorsolon? or predorgasolon? or prenilone or preventan or prezolon or rubycort or serilone or solonda or solupren? or spiricort or spolotane or supercorti#ol or taracortelone or walesolone or wysolone).mp. (33)

79 (methylprednisolone * or methylprednisolona* or methylprednisolonum* or metipred or urbason or medrol or x4w7zr7023* or 83-43-2 or besonia or brn 23403300 or brn2340300 or a-methapred or artisone-wyeth or besonia or depo-medrol or dopomedrol ec 201-4764 pr ec2014764 or einecs 201-476-4 or einecs201-476-4 or esametone or firmacort or hsdb3127 or hsdb3127 or lemod or medesone or medixon or medlone 21 or medrate or medrol or medrone or mesopren or metastab or methyleneprednisolone* or metilbetasone or metrisone or metrocort or metysolon or moderin or nirypan or noretona or nsc 19987 or nsc19987 or predni n tablinen or prednol-l or promacortine or reactenol or sieropresol or solomet or summicort or suprametil or u 7532 or u7532 or "u 67 590a" or urbason or urbasone or wyacort or adlone 40 or adlone 80 or beta methylprednisolone or medalone 80 or depmedalone or deproject 80 or depopred or esametone or firmacort or med-jec-40 or medixon or mednin or medralone or mepredprednisolone or meprelon or mesopren or methacort 40 or methacort 80 or methyxlcotol or methylcotolone or methylpred dp or methylsterolone or metidrol or metycortin or metypred or metypresol or neomedrone or solu decortin or urbason).mp. (26)

80 (prednison or prednisone or prednisona or prednisonum or dehydrocortisone or delta cortisone or rectodelt or sterapred or ultracorten or winpred or apo prednisone or cortan or cartancyl or panafcort or cutason or decortin or dacortin or decortisyl or deltasone or encortone or encorton or enkorton or enkortolon or kortancyl or liquid pred or meticorten or orasone or panasol or predni tablinen or prednidib or predniment or pronisone or vb0r961hzt* or 53-03-2 or adasone or al3-52939 or ancortone or bicortone or cartancyl or ccris 2646 or ccrs2646 or colisone or cortan or cortidelt or cotone or dacorten or dacortin or decortancyl or dekortin or dellacort or delta cortelan or delta cortisone or delta dome or deltacortene or deltacortisone or deltacortone or deltasone or deltison or deltisona or deltra or di adreson or diadreson or econosone or einecs 200-160-3 or einecs200-160-3 or fernisone or fiasone or hostacortin or hsdb 3168 or hsdb3168 or incocortyl or juvason or lisacort or lodotra or lodtra).mp. (578)

81 (me-korti or metacortandracin or metacorten or nci c04897 or ncic04897 or nisona or nizon or novoprednisone or nsc 10023 or nsc10023 or nurison or orasone or panafcort or paracort or prarmenison or pehacort or predeltin or prednicen-m or prednicorm or prednicort or prednicot or

prednilonga or prednitone or prednizon or prednovister or presone or pronison or rayos or rectodelt or retrocortine or servisone or sterapred or supercortio or u 6020 or u6020 or ultracorten or ultracortene or winpred or wojtab or zenadrid).mp. (40)

82 cortisone?.mp. (123)

83 corticosteroid*.mp. (2283)

84 corticoid*.mp. (56)

85 glucocorticoid*.mp. (812)

86 corticosterone*.mp. (18)

87 hydrocortisone*.mp. (345)

88 dexamethasone*.mp. (552)

89 beclomethasone*.mp. (260)

90 (triamcinolone* or 1zk20vi6ty* or 124-94-7 or aristocort or volon or fluoxyprednisolon* or tiamcinolonum or triamcinolonum* or triamcinolona* or brn 2341955 or brn2341955 or cl 19823 or cl19823 or einecs 204-718-7 or einecs204-718-7 or hsdb 3194 or hsdb3194 or nsc 13397 or nsc13397).mp. (293)

91 (steroid? adj10 (therap* or treat* or drug? or inject* or intramuscular* or intra muscular* or im or intravenous* or intra venous* or iv or intraarticular* or intra articular* or ia or administrat* or oral or orally or parenteral*)).mp. (1489)

92 ((jak or jak1 or jak2 or jak3 or janus kinase) adj10 inhibitor*).mp. (21)

93 (janus adj4 kinase adj10 inhibitor?).mp. (18)

94 (baricitinib* or 1187594-09-7 or isp4442i3y* or "incb 028050" or incb028050 or ly-3009104 or ly3009104).mp. (7)

95 (tofacitinib* or 477600-75-2 or 87la6fu830* or hsdb 8311 or hsdb8311 or cp-690 550 or cp 690550 or cp690550).mp. (37)

96 (filgotinib* or 1206161-97-8 or 3xvl385q0m* or "glpg 0634" or glpg0634).mp. (3)

97 (upadacitinib* or 1310726-60-3 or 4RA0KN46E0* or abt 494 or abt494).mp. (3)

98 (ruxolitinib* or rux or 941678-49-5 or 82s8x8xx8h* or hsdb 8259 or hsdb8259 or inc 424 or inc424 or incb424 or "incb 018424" or incb018424 or incb 18424 or incb18424 or r-ruxolitinib* or jakafi or jakavi).mp. (6)

99 (itacitinib or "incb 039110" or incb039110 or incb 39110 or incb39110 or 1334298-90-6 or 19j3781lpm*).mp. (1)

100 (ag490 or ag 490 or tyrphostin b42 or tyrphostin ag 490 or tyrphostin ag490 or 133550-30-8).mp. (0)

101 (fedratinib* or tg101348 or tg 101348 or sar302503 or sar 302503 or 936091-26-8 or 6l1xp550i6* or inrebic).mp. (2)

102 (ritlecitinib* or pf-06651600 or pf06651600 or pf 6651600 or pf6651600 or 2140301-97-7 or 132lf5wgh4* or 2192215-81-7 or eag4t1459k* or ritlecitinib malonate or ritlecitinib toslate or ritlecitinib tosylate or ritlecitinib propanedioate).mp. (0)

103 (peficitinib* or peficitinib hydrobromide).mp. (1)

104 or/44-103 (4373)

105 32 and 104 (372)

106 43 or 105 (373)

107 limit 106 to full systematic reviews [Limit not valid in ACP Journal Club,CCA,DARE; records were retained] (346)

108 limit 107 to yr="2012 - 2020" [Limit not valid in DARE; records were retained] (233)

109 from 107 keep 1-111 (111)

110 108 or 109 (296)

111 remove duplicates from 110 (296)

1.3.1.3 Cochrane CENTRAL

1 Arthritis, Rheumatoid/ (5811)

2 ((rheumat* or reumat* or revmat* or rheumo or reumo) adj3 (arthrit* or artrit* or polyarthrit* or polyartrit*)).ti,ot,ab,kw,hw. (16870)

3 (r?eumarthrit* or r?eumartrit* or r?eum arthrit* or r?eum artrit* or r?evmarthrit* or r?evmartrit* or r?evm arthrit* or r?evm artrit* or r?eum polyarthrit* or r?eum polyartrit* or r?eum poly arthrit* or r?eum poly artrit* or r?eumpolyarthrit* or r?eumpolyartrit*).ti,ot,ab,kw,hw. (3)

4 (chronic* adj2 (polyarthrit* or poly arthrit* or polyartrit* or poly artrit*)).ti,ot,ab,kw,hw. (143)

5 (inflammator* adj2 (arthrit* or artrit*)).ti,ot,ab,kw,hw. (649)

6 ((r?eum* or r?evm*) adj2 (chronic* adj2 articular*)).ti,ot,ab,kw,hw. (14)

7 ((rheumat* or reumat* or revmat* or rheumo or reumo) adj2 (condition* or diseas*)).ti,ot,ab,kw,hw. (4967)

8 Arthritis, Juvenile/ (298)

9 ((arthrit* or artrit*) adj6 juvenile*).ti,ot,ab,kw,hw. (989)

10 JIA.ti,ot,ab. (612)

11 8 or 9 or 10 (1106)

12 (systemic or polyart* or poly art* or oligoart* or oligo art*).ti,ot,ab,kw,hw. (52024)

13 11 and 12 (506)

14 ((polyarthr* or polyartrit* or poly arthrit* or poly artrit* or oligoarthrit* or oligoartrit* or oligo arthrit* or oligo artrit*) adj6 juvenile*).ti,ot,ab,kw,hw. (9)

15 (((stills or still's or still) adj disease) and juvenile*).ti,ot,ab,kw,hw. (31)

16 ((stills or still's or still) adj disease adj6 child*).ti,ot,ab,kw,hw. (1)

17 ((systemic adj6 JIA) or sJIA).ti,ot,ab. (164)

18 (((polyart* or oligoart* or poly art* or oligo art) adj6 JIA) or pJIA).ti,ot,ab. (169)

19 Still's Disease, Adult-Onset/ (8)

20 ((stills or still's or still) adj disease adj6 adult*).ti,ot,ab,kw,hw. (51)

21 Giant Cell Arteritis/ (90)

22 (giant cell adj6 (arterit* or aortit* or horton*)).ti,ot,ab,kw,hw. (289)

23 ((temporal or cranial) adj2 arterit*).ti,ot,ab,kw,hw. (44)

24 ((horton or horton's or hortons) adj3 disease?).ti,ot,ab,kw,hw. (4)

25 (large vessel adj3 (vasculit* or arterit*)).ti,ot,ab. (55)

26 GCA.ti,ab. and (arterit* or vasculit* or rheum*).mp,jw. (201)

27 Takayasu Arteritis/ (27)

28 (takayasu* adj3 (arterit* or syndrome* or disease*)).ti,ot,ab,kw,hw. (99)

29 (young female adj6 arterit*).ti,ot,ab,kw,hw. (0)

30 (brachiocephal* adj3 arterit*).ti,ot,ab,kw,hw. (0)

31 Cytokine Release Syndrome/ (2)

32 ((cytokine releas* or cytokine storm) adj3 syndrome*).ti,ot,ab,kw,hw. (249)

33 crs.ti,ab. and (cytokine releas* or cytokine storm).mp. (93)

34 Castleman Disease/ (12)

35 (castleman* adj3 (disease* or tumo?r*)).ti,ot,ab,kw,hw. (48)

36 ((angiofollicul* or angio follicul*) adj3 lymph* adj3 hyperplas*).ti,ot,ab,kw,hw. (40)

37 (lymph* node adj3 hyperplas* adj3 giant).ti,ot,ab,kw,hw. (2)

38 (castleman* adj3 (disease* or tumo?r* or pseudo-tumo?r* or pseudotumo?r* or lymph* or pseudolymphom* or hyperplas*)).ti,ot,ab,kw,hw. (48)

39 1 or 2 or 3 or 4 or 5 or 6 or 7 or 13 or 14 or 15 or 16 or 17 or 18 or 19 or 20 or 21 or 22 or 23 or 24 or 25 or 26 or 27 or 28 or 29 or 30 or 31 or 32 or 33 or 34 or 35 or 36 or 37 or 38 (20053)

40 (tocilizumab* or actemra* or atlizumab* or lusinex* or r1569 or r 1569 or roactemra* or mra or 375823 41 9 or i031v2h011* or rhpm-1 or rhpm1 or rg-1569 or rg1569 or msb-11456 or msb11456 or ro-4877533 or ro4877533).mp. (1934)

41 (sarilumab* or kevzara* or regn 88 or regn88 or sar 153191 or sar153191 or 1189541 98 7 or nu90v55f8i*).mp. (250)

42 (levilimab* or BCD-089 or bcd089 or 2035008 70 7 or P7UV3L2H80*).mp. (5)

43 (olokizumab* or cdp 6038 or cdp6038 or 1007223 17 7 or pai71r1d2w*).mp. (34)

44 (clazakizumab* or ald 518 or ald518 or bms 945429 or bms945429 or 1236278 28 6 or 4s38z8ra9o*).mp. (38)

45 (sirukumab* or cnto 136 or cnto136 or pilvensia or 1194585 53 9 or 640443FU93*).mp. (118)

46 (siltuximab* or sylvant or cnto 328 or cnto-328 or cnto328 or cllb8 or ccib8 monoclonal antibody or sylvant or t4h8fma7im* or 541502 14 1).mp. (76)

47 (ziltivekimab* or zilti or "cor-001" or "2226654 05 1").mp. (4)

48 (elsilimomab* or "b-e8" or 468715 71 1).mp. (2)

49 40 or 41 or 42 or 43 or 44 or 45 or 46 or 47 or 48 (2402)

50 39 and 49 (1293)

51 Interleukin 1 Receptor Antagonist Protein/ (304)

52 (anakinra* or kineret or ((interleukin 1 receptor or il1 receptor or il 1 receptor) adj1 antagonist protein*) or (recombinant interleukin 1 receptor adj1 (antagonist* or blocker* or blocking agent*)) or antril or il 1ra or il1ra or il 1 ra or ((urin* or febrile) adj2 (interleukin 1 inhibitor* or il1 inhibitor* or il 1 inhibitor*)) or "143090 92 0" or 9013duq28k*).mp. (1072)

53 Infliximab/ (729)

54 (infliximab* or ifx or avakine or flixabi or inflectra or inflecta or ixifi or remicade or remsima or revellex or renflexis or zessly or mab ca2 or monoclonal antibody ca2 or antibody ca2 monoclonal or ca2 monoclonal antibody or ct p13 or ctp13 or ct-p-13 gp-1111 or gp1111 or ta-650 or ta650 or abp 710 or abp710 or bow-015 or bow015 or 170277 31 3 or b72hh48flu* or sb2 or "pf 06438179" or pf 6438179 or pf06438179 or pf6438179 or hsdb 7850 or hsdb7850).mp. (2747)

55 Etanercept/ (755)

56 (etanercept* or benepali or embrel or enbrel or enia 11 or enia11 or erelzi or lifmior or opinercept or recombinant tumo?r necrosis factor receptor fc fusion protein or "tnr 001" or tnr001 or tnfr-fc or tnrfr:fc or rhu tnfr:fc or rhu-tnfr:fc or tnfr-immunoadhesin or tnt receptor fusion protein or tum?or necrosis factor receptor fc fusion protein or tunex or "185243 69 0" or op401g7ojc* or brenzys or tnf receptor fusion protein or tnfr fc or recombinant human tnf or recombinant human dimeric tnf receptor type ii igg fusion protein or chs-0214 or chs0214 or dwp-422 or dwp422 or enia-11 or enia11 or gp-2015 or gp2015 or gp2015c or gp-2015c or hd-203 or hd203 or lbec-0101 or lbec0101 or sb-4 or sb4).mp. (2426)

57 Adalimumab/ (747)

58 (adalimumab* or humira or abp 501 or abp501 or abtd2e7 or amjevita or amgevita or bax 2923 or bax2923 gp 2017 or gp2017 or halimatoz or hefiya or hulio or hyrimoz or ibi 303 or ibi303 or imraldi or m 923 or m923 or msb 11022 or msb11022 or ons3010 or ons 3010 or "pf 06410293" or pf 6410293 or pf06410293 or pf6410293 or d2e7 or truxeda or cyltezo or solymbic or fys6t7f842* or 331731 18 1 or lu 200134 or lu200134 or d2e7 or hsdb 7851 or hsdb7851 or abp-501 or abp501 or bcd-057 or bcd057 or bi-695501 or bi695501 or chs-1420 or chs1420 or gp-2017 or gp2017 or sb-5 or sb5).mp. (3343)

59 (golimumab* or cnto 148 or cnto148 or simponi or 476181 74 5 or 91x1klu43e*).mp. (739)

60 Certolizumab Pegol/ (166)

61 (certolizumab* or cdp 870 or cdp870 or cimzia or pegylated tumor necrosis factor alpha antibody fab fragment or pegylated tumour necrosis factor alpha antibody fab fragment or pha 738144 or pha738144 or czp or 428863 50 7 or g6adw90r16* or umd07x179e* or hsdb 7848 or hsdb7848).mp. (693)

62 Rituximab/ (1261)

63 (rituximab* or ct p10 or ctp10 or idec 102 or idec102 or idecc2b8 or idec c2b8 or monoclonal antibody idec c2b8 or gp2013 or gp 2013 or "pf 05280586" or pf05280586 or mabthera or mab thera or r 105 or r105 or reditux or rg 105 or rg105 or rituxan or rituxin or ritemvia or rituzena or rixathon or riximyo or ro 452294 or ro452294 or truxima or 174722 31 7 or 4f4x42syq6* or hsdb 7455 or hsdb7455).mp. (5262)

64 (ofatumumab* or humax cd20 or humax-cd20 or humaxcd20 or humax-cd20-2f2 or arzerra or gsk 1841157 or gsk1841157 or gski841157 or humac cd20 or omb 157 or omb157 or 679818 59 8 or m95kg522r0* or hsdb 8170 or hsdb8170).mp. (294)

65 Abatacept/ (277)

66 (abatacept* or ctla4 ig or ctla4 immunglobulin or ctla4 immunoglobulin g or ctla4ig or 'ctla 4 ig' or ctla-igg4m or ctla4-fc or bms 188667 or bms188667 or orencia or '332348 12 6' or 7d0yb67s97* or rg 2077 or rg2077 or rg-1046 or rg1046 or nulojix or lea29y or lea 29y or ctl4 fc or ctl4fc or belatacept or bms 224818 or bms224818 or belatacept or cytotoxic t lymphocyte associat* antigen 4).mp. (1122)

67 (tabalumab* or ly 2127399 or ly2127399 or 1143503 67 6 or pqp8vh3mjw*).mp. (70)

68 (pateclizumab* or mlta 3698a or mlta3698a or pro 283698 or pro283698 or 12202526 59 7 or qok1yyh7j2* or rg 7415 or rg7415).mp. (7)

69 (secukinumab* or cosentyx or ain 457 or ain457 or 1229022 83 6 or dlg4eml025*).mp. (892)

70 (ixekizumab* or ly 2439821 or ly2439821 or taltz or 1329632 62 3 or 1143503 69 8 or bty153760o*).mp. (461)

71 (brodalumab* or amg 827 or amg827 or khk 4827 or khk4827 or siliq or kyntheum or 1174395 19 7 or 6za31y954z*).mp. (176)

72 (guselkumab* or cnto 1959 or cnto1959 or 1350289 85 8 or tremfya or 1350289 85 8 or 089658a12d*).mp. (216)

73 Ustekinumab/ (188)

74 (ustekinumab* or cnto 1275 or cnto1275 or stelara or "815610 63 0" or fu77b4u5z0* or 1275 cnto or 1275cnto or l04ac05 or tt-20 or tt20).mp. (872)

75 (mavrilimumab or "1085337 57 0" or 1158jdp9a* or cam 3001 or cam3001).mp. (52)

76 exp Antirheumatic Agents/ (34789)

77 (dmard* or csdmard* or cs-dmard* or sdmard* or s-dmard* or tsdmard* or ts-dmard* or bdmard* or b-dmard*).ti,ab. (3021)

78 (disease modifying adj3 (antirheum* or anti rheum*) adj3 (drug? or agent?)).mp. (2093)

79 ((antirheum* or anti rheum*) adj5 (drug? or agent?)).ti,ot,ab. (2138)

80 Methotrexate/ (4132)

81 (mxt or methotrex* or metotrexat* or methoblastin or methylaminopterin* or amet?opterin* or abitrexate or antifolan or brimexate or a-met?opterin* or a-methpterin* or ai3-25299 or ai325299 or alpha-methopterin or biotrexate or emtexate or emtrexate* or emthexat* or enthexate* or farmitrexat* or farmotrex or fauldexato or folex or folex pfs or hdmtx or ifamet or imeth or maxtrex or metotressat* or methotrate* or methohexate* or mexat* or metrotex or metatrexato* or metex or metrex or metatrexan or maxtrex or meticil or metoject or metotrexin or novatrex* or neotrexate* or nordimet or lumexon or ledertrexate* or lanterel or rasuvo or r?eumatrex* or reditrex or rasuvo or texate or texate-t or tremetex or trexeron or trixilem or texorate or trexall or xaken or xatmep or otrexup or mpi-2505 or mpi2505 or mpi 5004 or mpi5004 or amethopterin* or 133073-73-1 or 15475-56-6 or 3ig1e710zn* or yl5fz2y5u1* or cl 14377 or cl14377 or ccris 1109 or ccris1109 or emt 25299 or emt25299 or nsc-740 or nsc740 or r-9985 or r9885 or x-133 or wr-19039 or wr19039).mp. (12000)

82 Leflunomide/ (151)

83 (leflunomid* or hwa 486 or hwa486 or su101 or su 101 or arava or arabloc or hsdb 7289 or hsdb7289 or g162gk9u4w* or 75706-12-6 or rs 34821 or rs34821).mp. (682)

84 lef.ti,ot,ab. (171)

85 Sulfasalazine/ (468)

86 (sulfasalazin* or sasp or ssz or salicylazosulfapyridin* or salicyl azo sulfapyridin* or sulphasalazin* or salazosulfapyridin* or salazosulfapiridin* or 'pyralin en' or azulfadin* or azulfidin* or asulfidin* or azulfide* or azulfin or colo-pleon or colopleon or disalazin or gastropyrin or pleon or "pleon ra" or "pyralin en" or rorasul or rosulfant or ulcol or ucine or salazopyrin* or ratio-sulfosalazin* or sulfasalazin* or 3xc8guz6cb* or accucol or asulfidin* or azopyrin* or azosulfidin* or benzosulfa or "brn 0356241" or brn0356241 or ccris 4713 or ccris4713 or einecs 209-974-3 or hsdb 3395 or hsdb3395 or nsc 203730 or nsc203730 or nsc 667219 or nsc667219 or reupirin or rorasul or salazo-sulfapyridin* or salazosulfpyridin* or salazopiridazin* or salazopyridin* or salazopirin* or salazodin or salisulf or salipyr or saridine or si-88 or si88 or sulculon or sulfasalazin* or sulfasalizin* or sulphasalazin* or w-t sasp oral or 599-79-1 or "s.a.s. 500" or "s.a.s.-500" or sas-500 or zopyrin*).mp. (1585)

87 Hydroxychloroquine/ (493)

88 (hydroxychloroquin* or hcq or 747-3-4 or 8q2869cnvh* or 118-42-3 or 4qwg6n8qkh* or hydroxychlorochin* or hydrochloroquin* or hydrocloroquin* or oxychlorochin or oxychloroquin* or plaquenil or plaquinol or "brn 0253894" or brn0253894 or einecs 204-249-8 or einecs204-249-8 or hidroxicloroquin* or idrossiclorochin* or oxichlorochin* or oxichloroquin* or oxiklorin or win1258 or win 1258 or z0188 or einecs 212-019-3 or einecs212-019-3 or chloroquinol or dimard or ercoquin or evoquin or erquin or geniquin or quensyl or sn 8137 or sn8137 or toremonil or yuma).mp. (1580)

89 Gold Sodium Thiomalate/ (91)

90 (gold sodium thiomalate* or aurothiomalate* or aurolate* or gold thiomalate* or gold disodium thiomalate* or gold thiomalic acid* or miocrin or miocrisin or monogold disodium thiomalate* or myochrysin* or myocrisin* or myocrysin* or sodium aurothiomalate* or sodium gold thiomalate* or sodium thiomalate* gold or sodium thiomalatoaurate* or tauredon or taureodon or tauredone or thiomalate gold or thiomalatoaurate sodium or 1244-57-4 or 39377-38-3 or 4846-27-9 or 74916-57-7 or e4768zy6gm* or aurothiomala-natrium or aurot?iomalato sodico or monogold* sodium salt or dinatrium 2-aurothio-succinat or einecs 235-479-7 or einecs235-479-7 or hsdb 7173 or hsdb7173 or kidon or natrii aurothiomalas* or shiosol or gold mercatpsuccinate*).mp. (156)

91 Aurothioglucose/ (36)

92 (thioglucosoaurate* or gold thioglucose* or thioglucose gold or aureotan or solganal or solganol or gold-50 or gold50 or solganal b or b oleosum solganal or auromyose* or aurothioglucose* or aurotan or aurumine or authron or brenol or ccris 59 or ccris59 or einecs 235-365-7 or einecs235-365-7 or glysanol b or goldthioglucose* or hsdb 7174 or hsdb7174 or oronol or romosol or skf 10056 or skf10056 or 2p2v9q0e78* or 12192-57-3).mp. (49)

93 (thioglucosoaurate* or gold thioglucose* or gold thio glucose* or thioglucose gold or aureotan or solganal or solganol or gold-50 or gold50 or solganal b or b oleosum solganal or auromyose* or aurothioglucose* or auro thioglucose* or aurotan or aurumine or aurumin or authron or brenol or ccris 59 or ccris59 or einecs 235-365-7 or einecs235-365-7 or glysanol b or goldthioglucose* or goldtioglucose* or hsdb 7174 or hsdb7174 or oronol or romosol or skf 10056 or skf10056 or 2p2v9q0e78* or 12192-57-3).mp. (49)

94 ((gold* or auro*) adj10 (inject* or intramuscular* or im or intra muscular*)).ti,ot,ab. (209)

95 (aurothiopropanol* or allochrysine or allocrisine or aurotiopoe or aurotioprol or 27279-43-2 or g7097j63e9* or auromercapto propanol sulfonate sodium or auromercaptopropanolsulfonate sodium or aurothioisopropanolsulfonate sodium or gold sodium thiopropanol sulfonate or sodium auromercaptopropanol sulfonate).mp. (3)

96 Organogold Compounds/ (15)

97 (aurothiopropanol* or allochrysine or allocrisine or aurotiopoe or aurotioprol or 27279-43-2 or g7097j63e9* or auromercapto propanol sulfonate sodium or auromercaptopropanolsulfonate sodium or aurothioisopropanolsulfonate sodium or sodium aurothiopropanolsulfonate or gold sodium thiopropanol sulfonate or sodium auromercaptopropanol sulfonate).mp. (3)

98 Chloroquine/ (708)

99 (chloroquin* or cq or 54-04-7 or 886u3h6uff* or aralen or aralene or arechin or arechine or arequin or chingamin or chlorochin* or khingamin or nivaquine or oe48649kh6n* or amokin or amokine or anaclor or aralan or arequine or artriquine or avloclor or avoclor or arthrochin or artrichin or bemaco or bemaphata or bemaphate or bemasulph or benaquin or bipiquin or cadiquin or "brn 0482809" or brn0482809 or capquin or ccris 3439 or ccris3439 or chemochin or chemochine or chingamine or chingaminum or chloraquin* or chlorquin or chlorquine or choloquine or choroquine sulfate or choroquine sulphate or cindacin or clo-kit junior or clorichina or clorichine or cloriquine or clorochina or delagil or delagyl or diclokin or chlorofoz or cindachin or clorochina or cloroquina or cocatrit or dichinalex or diquinalex or diroquine or emquin or genocin or gontochin or gontochine or gontoquine or einecs 200-191-2 or einecs200-191-2 or elestol or gontochin or heliopar or hsdb 3029 or hsdb3029 or imagon or iroquine or klorokin or klorokine or klorokinfosfat or lagaquin or lapaquin or malaquin or malaren or malarex or malarivon or malaviron or maliaquine or maquine or mesylith or mexaquin or mirquin or neochin or nivaquin or nivachine or nivaquine b or nivaquine dp or nivaquine forte or "p roquine" or nsc 187208 or nsc187208 or pfizerquine or quensyl or quinachlor or quinercyl or quingamine or quinilon or quinoline or quinoscan or repal or resochen or resochene or resochin or resochin junior or resochina or resochine or resochinon or resoquina or resoquine or reumachlor or ronaquine or roquine or rp 3377 or rp3377 or sanoquin or sanoquine or silbesan or siragan or sirajan or sn 7618 or sn7818 or solprina or solprine or sopaquin or st 21 or tresochin or tresoquine or trochin or trochine or troquine or weimerquin or w 7618 or w7618 or win 244 or win244).mp. (2059)

100 exp Adrenal Cortex Hormones/ (27497)

101 exp Prednisolone/ (4829)

102 Prednisone/ (3972)

103 Cortisone/ (148)

104 Hydrocortisone/ (5928)

105 exp Steroids/ (53428)

106 adrenal cortex hormones.mp. (2206)

107 prednisolone*.mp. (7359)

108 methylprednisolone*.mp. (5436)

109 (prednisolone* or predonine or di-adreson-f or diadresonf or 9phq9y1olm* or prednisolona* or prednisolonum* or nsc-9120 or nsc9120 or nsc 9900 or nsc9900 or delta-dehydrocortisol* or delta-dehydrocortisone* or delta-hydrocortisone or hydroretrocortine or metacortandralone* or brn 1354103 or brn1354103 or bubbli-pred or ccris 980 or ccris980 or co-hydeltra or codelcortone or cordrol or cortalone or cotogesic or cotolone or decaprednil or decortin h or delcortol or dehydrohydrocortisone* or delta-cortef or deltacortef or delta-ef-cortelan or delta-stab or deltacortenol or deltacortril or deltahydrocortisone* or deltisilone or derpo pd or dexa-dortidelt hostacortin h or di-adreson f or dicortol or donisolone or dydeltrone or eazolin d or ec 200-021-7 or ec200-021-7 or einecs 200-021-7 or einecs200-021-7 or erbacort or erbasona or estilsona or fernisolone or fernisolone p or hostacortin h or hsdb 3385 or hsdb3385 or hydeltra or hydeltrone or hydrodeltalone or hydrodeltisone or hydroretrocortin or hydroretrocortine* or k 1557 or k1557 or lentosone or meti-drm or meticortelone or orapred or orapred odt or paracortol or paracotol or pediapred or precortancyl or precortilon or precortisyl or predne-dome or prednelan or prednicen or predniliderm or predniretard or prednis or predonin or predonine* or prelon or prelone or prenolone or rolisone or scherisolon? or solone or sterolone or ulacort or ultracorten h or ultracortene h adelcort or antisolon or antisolone or aprednisolon? or benisolon? or caberdelta or co hydeltra or codelcortone or ompresolon).mp. (7377)

110 (cortadeltona or cortelinter or cortisolone or dacortin or dacortin h or dacrotin or decaprednil or decortil or delta cortril or delta hycortol or deltacortenolo or deltacortil or deltacortoil or deltaderm or deltadglycortril or deltahycortol or deltahydrocortison? or deltaophticor or deltasolone or deltastab or deltidrosol or deltisilone or deltisolon? or deltolasson? or deltosona or deltosone or depo-predate or dermosolon or dhasolone or di adreson? f or diadreson? f or dicortol or domucortone or encortelon? or encortolon or equisolon or glistelone or hefasolon or hydrelta or hydrocortancyl or hydrocortidelt or hydradeltalone or hydradeltisone or inflanefran or insolone or keteocort h or key-pred or key-pred sp or lenisolone or leocortol or liquipred or mediasolone or meprisolon? or metacortalon? or metacortelone or meti derm or metiderm or meticortelone or morlone or mydrapred or nisolon? or opredsone or panafcortelone or panafcortolone or panafort or paracortol pr phlogex or pre cortisyl or preconin or precortalon or precortancyl or precortisyl or pred ject 50

or predacort 50 or predaject 50 or predalone 50 or predartrina or predartrine or predate 50 or predeltilone or predisole or predisyr or predne dome or prednecort or prednedome or prednelan or predni coelin or predni h tablinen or predni helvacort or prednicoelin or prednicort or prednicortelone or prednifor drops or predniment or prednorsolon? or predorgasolon? or prenilone or preventan or prezolon or rubycort or serilone or solonda or solupren? or spiricort or spolotane or supercorti#ol or taracortelone or walesolone or wysolone).mp. (22)

111 (methylprednisolone * or methylprednisolona* or methylprednisolonum* or metipred or urbason or medrol or x4w7zr7023* or 83-43-2 or besonia or brn 23403300 or brn2340300 or a-methapred or artisone-wyeth or besonia or depo-medrol or dopomedrol ec 201-4764 pr ec2014764 or einecs 201-476-4 or einecs201-476-4 or esametone or firmacort or hsdb3127 or hsdb3127 or lemod or medesone or medixon or medlone 21 or medrate or medrol or medrone or mesopren or metastab or methyleneprednisolone* or metilbetasone or metrisone or metrocort or metysolon or moderin or nirypan or noretona or nsc 19987 or nsc19987 or predni n tablinen or prednol-l or promacortine or reactenol or sieropresol or solomet or summicort or suprametil or u 7532 or u7532 or "u 67 590a" or urbason or urbasone or wyacort or adlone 40 or adlone 80 or beta methylprednisolone or medalone 80 or depmedalone or deproject 80 or depopred or esametone or firmacort or med-jec-40 or medixon or mednin or medralone or mepredprednisolone or meprelon or mesopren or methacort 40 or methacort 80 or methyxlcotol or methylcotolone or methylpred dp or methylsterolone or metidrol or metycortin or metypred or metypresol or neomedrone or solu decortin or urbason).mp. (1204)

112 (prednison or prednisone or prednisona or prednisonum or dehydrocortisone or delta cortisone or rectodelt or sterapred or ultracorten or winpred or apo prednisone or cortan or cartancyl or panafcort or cutason or decortin or dacortin or decortisyl or deltasone or encortone or encorton or enkorton or enkortolon or kortancyl or liquid pred or meticorten or orasone or panasol or predni tablinen or prednidib or predniment or pronisone or vb0r961hzt* or 53-03-2 or adasone or al3-52939 or ancortone or bicortone or cartancyl or ccris 2646 or ccrs2646 or colisone or cortan or cortidelt or cotone or dacorten or dacortin or decortancyl or dekortin or dellacort or delta cortelan or delta cortisone or delta dome or deltacortene or deltacortisone or deltacortone or deltasone or deltison or deltisona or deltra or di adreson or diadreson or econosone or einecs 200-160-3 or einecs200-160-3 or fernisone or fiasone or hostacortin or hsdb 3168 or hsdb3168 or incocortyl or juvason or lisacort or lodotra or lodtra).mp. (10042)

113 (me-korti or metacortandracin or metacorten or nci c04897 or ncic04897 or nisona or nizon or novoprednisone or nsc 10023 or nsc10023 or nurison or orasone or panafcort or paracort or prarmenison or pehacort or predeltin or prednicen-m or prednicorm or prednicort or prednicot or prednilonga or prednitone or prednizon or prednovister or presone or pronison or rayos or rectodelt or retrocortine or servisone or sterapred or supercortio or u 6020 or u6020 or ultracorten or ultracortene or winpred or wojtab or zenadrid).mp. (19)

114 cortisone?.mp. (568)

115 corticosteroid*.mp. (22857)

116 corticoid*.mp. (554)

117 glucocorticoid*.mp. (8802)

118 corticosterone*.mp. (152)

119 hydrocortisone*.mp. (9660)

120 dexamethasone*.mp. (12204)

121 beclomethasone*.mp. (2291)

122 (triamcinolone* or 1zk20vi6ty* or 124-94-7 or aristocort or volon or fluoxyprednisolon* or tiamcinolonum or triamcinolonum* or triamcinolona* or brn 2341955 or brn2341955 or cl 19823 or cl19823 or einecs 204-718-7 or einecs204-718-7 or hsdb 3194 or hsdb3194 or nsc 13397 or nsc13397).mp. (3192)

123 (steroid? adj10 (therap* or treat* or drug? or inject* or intramuscular* or intra muscular* or im or intravenous* or intra venous* or iv or intraarticular* or intra articular* or ia or administrat* or oral or orally or parenteral*)).ti,ot,ab. (12844)

124 Janus Kinase Inhibitors/ (46)

125 ((jak or jak1 or jak2 or jak3 or janus kinase) adj10 inhibitor*).mp. (1479)

126 (janus adj4 kinase adj10 inhibitor?).mp. (894)

127 exp Janus Kinases/ai [Antagonists & Inhibitors] (94)

128 (baricitinib* or 1187594-09-7 or isp4442i3y* or "incb 028050" or incb028050 or ly-3009104 or ly3009104).mp. (393)

129 (tofacitinib* or 477600-75-2 or 87la6fu830* or hsdb 8311 or hsdb8311 or cp-690 550 or cp 690550 or cp690550).mp. (808)

130 (filgotinib* or 1206161-97-8 or 3xvl385q0m* or "glpg 0634" or glpg0634).mp. (165)

131 (upadacitinib* or 1310726-60-3 or 4RA0KN46E0* or abt 494 or abt494).mp. (241)

132 (ruxolitinib* or rux or 941678-49-5 or 82s8x8xx8h* or hsdb 8259 or hsdb8259 or inc 424 or inc424 or incb424 or "incb 018424" or incb018424 or incb 18424 or incb18424 or r-ruxolitinib* or jakafi or jakavi).mp. (478)

133 (itacitinib or "incb 039110" or incb039110 or incb 39110 or incb39110 or 1334298-90-6 or 19j3781lpm*).mp. (33)

134 (ag490 or ag 490 or tyrphostin b42 or tyrphostin ag 490 or tyrphostin ag490 or 133550-30-8).mp. (1)

135 (fedratinib* or tg101348 or tg 101348 or sar302503 or sar 302503 or 936091-26-8 or 6l1xp550i6* or inrebic).mp. (29)

136 (ritlecitinib* or pf-06651600 or pf06651600 or pf 6651600 or pf6651600 or 2140301-97-7 or 132lf5wgh4* or 2192215-81-7 or eag4t1459k* or ritlecitinib malonate or ritlecitinib toslate or ritlecitinib tosylate or ritlecitinib propanedioate).mp. (24)

137 (peficitinib* or peficitinib hydrobromide).mp. (25)

138 or/51-137 (148626)

139 39 and 138 (11830)

140 50 or 139 (12041)

141 randomized controlled trial.pt. (505478)

142 controlled clinical trial.pt. (91759)

143 randomi*.ti,ot,ab,kw,hw. (1014515)

144 Placebos/ (24072)

145 placebo*.mp. (329033)

146 exp placebo effect/ (1543)

147 randomly.ti,ot,ab. (257382)

148 exp Randomized Controlled Trials as Topic/ (7991)

149 Controlled Clinical Trials as Topic/ (71)

150 clinical trial/ or clinical trial, phase ii/ or clinical trial, phase iii/ or clinical trial, phase iv/ (33)

151 clinical trials as topic/ or clinical trials, phase ii as topic/ or clinical trials, phase iii as topic/ or clinical trials, phase iv as topic/ (33505)

152 (phase 2 or phase 3 or phase 4 or phase ii or phase iii or phase iv or phase II or phase III or phase IV or phase l or phase ll or phase lll or phase lV).ti,ot,ab,kw,hw. (130831)

153 trial.ti,ot,ab,kw,hw. (924215)

154 ((singl* or doubl* or trebl* or tripl*) adj3 (blind* or dumm* or mask*)).ti,ot,ab,kw,hw. (388185)

155 (singl*blind or doubl*blind* or trebl*blind* or tripl*blind* or singl*mask* or doubl*mask* or trebl*mask* or tripl*mask*).ti,ot,ab,kw,hw. (2475)

156 Single-Blind Method/ (21109)

157 Double-Blind Method/ (138830)

158 Cross-Over Studies/ (37577)

159 (crossover or cross over).ti,ot,ab,kw,hw. (106538)

160 or/141-159 (1453894)

161 140 and 160 (11445)

162 exp animals/ not humans/ (12)

163 161 not 162 (11445)

164 limit 163 to english (8238)

165 limit 164 to yr="2012 - 2020" (5216)

166 from 165 keep 1-2000 (2000)

167 from 165 keep 2001-4000 (2000)

168 from 165 keep 4001-5216 (1216)

1.3.2 Efficacy: other studied diseases

1.3.2.1 Medline

1 exp Coronavirus/ (43712)

2 exp Coronavirus Infections/ (47706)

3 (coronavirus* or corona virus* or OC43 or NL63 or 229E or HKU1 or HCoV* or ncov* or covid* or sars-cov* or sarscov* or Sars-coronavirus* or Severe Acute Respiratory Syndrome Coronavirus*).mp. (92228)

4 (or/1-3) and ((20191* or 202*).dp. or 20190101:20301231.(ep).) (79238)

5 4 not (SARS or SARS-CoV or MERS or MERS-CoV or Middle East respiratory syndrome or camel* or dromedar* or equine or coronary or coronal or covidence* or covidien or influenza virus or HIV or bovine or calves or TGEV or feline or porcine or BCoV or PED or PEDV or PDCoV or FIPV or FCoV or SADS-CoV or canine or CCov or zoonotic or avian influenza or H1N1 or H5N1 or H5N6 or IBV or murine corona*).mp. (51189)

6 ((pneumonia or covid* or coronavirus* or corona virus* or ncov* or 2019-ncov or sars*).mp. or exp pneumonia/) and Wuhan.mp. (3698)

7 (2019-ncov* or 2019-n-cov*or 2019ncov* or 2019n-cov* or ncov19 or ncov-19 or 2019-novel CoV*or ncov2019* or n-cov2019* or ncov-2019* or n-cov-2019* or novel cov-2019* or novel-cov-19* or ncov19* or n-cov19* or ncov-19* or n-cov-19* or sars-cov2* or sars-cov-2* or sarscov2* or sarscov-2* or sars-covid-2* or sars-2-cov* or Sars-coronavirus2* or Sars-coronavirus-2* or SARS-like coronavirus* or coronavirus-19 or covid19 or covid-19 or covid 2019 or ((novel or new or newly or nouveau or "19?" or "2019*" or wuhan) adj2 (CoV on nCoV or n-cov or covid or coronavirus* or corona virus or betacoronavirus* or Pandemi*2)) or ((covid or covid19 or covid-19) and pandemic*2) or (coronavirus* and pneumonia)).mp. (78956)

8 (severe acute respiratory syndrome coronavirus 2* or severe acute respiratory syndrome cov* 2* or severe acute respiratory syndrome coronavirus 2019* or severe acute respiratory syndrome cov* 2019* or severe acute respiratory syndrome coronavirus 19* or severe acute respiratory syndrome cov* 19*).mp. [mp=title, abstract, original title, name of substance word, subject heading word, floating sub-heading word, keyword heading word, organism supplementary concept word, protocol supplementary concept word, rare disease supplementary concept word, unique identifier, synonyms] (36004)

9 COVID-19.rx,px,ox. or severe acute respiratory syndrome coronavirus 2.os. (37817)

10 ("32240632" or "32236488" or "32268021" or "32267941" or "32169616" or "32267649" or "32267499" or "32267344" or "32248853" or "32246156" or "32243118" or "32240583" or "32237674" or "32234725" or "32173381" or "32227595" or "32185863" or "32221979" or "32213260" or "32205350" or "32202721" or "32197097" or "32196032" or "32188729" or "32176889" or "32088947" or "32277065" or "32273472" or "32273444" or "32145185" or "31917786" or "32267384" or "32265186" or "32253187" or "32265567" or "32231286" or "32105468" or "32179788" or "32152361" or "32152148" or

"32140676" or "32053580" or "32029604" or "32127714" or "32047315" or "32020111" or "32267950" or "32249952" or "32172715").ui. [Articles not captured by this search when created in April 2020, pending further indexing by NLM] (49)

11 or/6-10 (79089)

12 5 or 11 (80298)

13 12 and 20191201:20301231.(dt). (78041)

14 exp Lung Diseases, Interstitial/ (56349)

15 exp Scleroderma, Systemic/ (20798)

16 14 and 15 (1009)

17 (((systemic adj3 (sclerosis* or sleroderma*)) or ssc) and (interstitial adj3 (lung disease* or pneumoni*))).ti,ab,kf. (1453)

18 (ssc-ild* or sscild*).ti,ab,kf. (296)

19 Polymyalgia Rheumatica/ (2533)

20 pmr.ti,ab,kw. and rheum*.mp. [mp=title, abstract, original title, name of substance word, subject heading word, floating sub-heading word, keyword heading word, organism supplementary concept word, protocol supplementary concept word, rare disease supplementary concept word, unique identifier, synonyms] (917)

21 ((rheumat* or arteritic*) adj1 (polymyalgia* or myalgia*)).ti,ab,kf. (2774)

22 ((pseudopolyarthrit* or pseudo polyarthrit* or inflammat* rheumat* or rheumat* inflammat*) adj1 rhizomelic).ti,ab,kf. (86)

23 (forestier-certonciny adj1 syndrom*).ti,ab,kf. (2)

24 (remitting adj3 seronegative adj3 synovitis adj6 (edem* or oedem*)).ti,ab,kw. (189)

25 rs3pe*.ti,ab,kw. (191)

26 exp Synovitis/ (8377)

27 Edema/ (40300)

28 26 and 27 (370)

29 (remitting or seronegativ* or symmetric*).ti,ab,kf. (103569)

30 28 and 29 (156)

31 exp Lupus Erythematosus, Systemic/ (60103)

32 (lupus erythemato* adj3 (systemic* or disseminat* or visceral*)).ti,ab,kf. (54723)

33 (sle or 's l e').ti,ab,kf. (34714)

34 (lupus or autoimmun* or auto immun* or autoantibod* or antibod* or inflamm* or immun* or rheumat* or arthrit* or nephritis or nephrol* or cutan* or dermatol* or venerol* or neuromyotoni* or neuroscience? or neurolog* or patient? or cell debris).mp,jw. (11359926)

35 33 and 34 (33879)

36 (libman sacks adj1 (disease* or endocarditis*)).ti,ab,kf. (259)

37 exp Myositis/ (19912)

38 (myositis or neuromyositis or polymyositis or dermatomysitis).ti,ab,kf. (14578)

39 (inflammat* adj1 (myopath* or muscle? or myosit*)).ti,ab,kf. (4510)

40 (inflammat* adj3 idiopath* adj3 (myopath* or myosit*)).ti,ab,kf. (1629)

41 ((infect* or focal or proliferativ*) adj3 (myopath* or myosit*)).ti,ab,kw. (702)

42 exp Amyloidosis/ (24819)

43 (amyloidos#s or amyloidoma* or beta fibrillos#s or paraamyloidos#s).ti,ab,kf. (24594)

44 Polychondritis, Relapsing/ (1390)

45 (polychondrit* adj3 (relapsing* or atrophic*)).ti,ab,kf. (1467)

46 (chondromalac* adj3 systemic*).ti,ab,kf. (3)

47 (von meyenburg adj3 disease*).ti,ab,kf. (3)

48 (periodic fever adj1 familial adj1 autosomal dominant).rx. (98)

49 ((tnf receptor or tnfr1 or tnfr) adj3 associated periodic syndrom*).ti,ab,kf. (155)

50 (traps and (tumo?r necrosis factor receptor or tnf receptor)).ti,ab,kf. (312)

51 (tumo?r necrosis factor receptor adj3 associated periodic adj2 (syndrom* or fever)).ti,ab,kf. (292)

52 (autosomal dominant adj3 periodic fever).ti,ab,kf. (13)

53 (hibernian fever adj3 familial).ti,ab,kf. (16)

54 ((tnf receptor associated periodic or tnfr1 associated or tumo?r necrosis factor receptor associated or tnfr associated) adj3 (syndrom* or fever*)).ti,ab,kf. (394)

55 Cryopyrin-Associated Periodic Syndromes/ (462)

56 (cinca or cinca syndrom*).ti,ab,kf. (183)

57 (chronic infantile adj3 neurolog* cutan* adj3 (articul* syndrom* or arthropath*)).ti,ab,kf. (98)

58 (neonatal onset adj3 multisyst* inflamm* adj1 (diseas* or disorder*)).ti,ab,kf. (146)

59 nomid.ti,ab,kf. (126)

60 iomid.ti,ab,kf. (4)

61 cryopyrin associated periodic syndrom*.ti,ab,kf. (433)

62 Neuromyelitis Optica/ (3027)

63 (neuromyelit* adj1 optic*).ti,ab,kf. (4287)

64 (myelooptic* adj1 neuropath*).ti,ab,kf. (3)

65 ((nmo or nmosd) and (neuromyelit* or neuropath*)).ti,ab,kf. (2726)

66 ((devic or devics or devic's) adj2 (diseas* or syndrom* or neuromyelit* optic*)).ti,ab,kf. (456)

67 (myeloopticoneuropath* or myelooptic* neuropath* or neuroopticomyelit*).ti,ab,kf. (6)

68 exp Anti-Neutrophil Cytoplasmic Antibody-Associated Vasculitis/ (10596)

69 ((anca or antineutrophil cytoplasmic or anti-neutrophil cytoplasmic) adj6 vasculit*).ti,ab,kf. (4585)

70 (pauci immun* adj6 vasculit*).ti,ab,kf. (145)

71 exp Arthritis, Psoriatic/ (6269)

72 (psoria* adj2 (arthrit* or polyarthrit* or poly arthrit* or arthropath*)).ti,ab,kf. (10720)

73 (psoria* adj1 rheumat*).ti,ab,kf. (283)

74 (psoria* adj6 (inflammat* arthrit* or inflammat* polyarthrit* or inflammat* poly arthrit*)).ti,ab,kf. (283)

75 Spondylitis, Ankylosing/ (14819)

76 ((spondylitis* or spondylarthrit* or spndyloarthrit* or spine or vertebral) adj1 ankyl*).ti,ab,kf. (15689)

77 (rheumat* spondylit* or axial spondyl?arthrit* or bechterew* disease* or bechterews disease* or bechterew's disease* or marie struempell disease*).ti,ab,kf. (2097)

78 (axspa* or ax-spa*).ti,ab,kf. (782)

79 (axial adj3 spondyl?arthrit*).ti,ab,kf. (1591)

80 13 or 16 or 18 or 19 or 20 or 21 or 22 or 23 or 24 or 25 or 26 or 31 or 32 or 33 or 36 or 37 or 38 or 39 or 40 or 41 or 42 or 43 or 44 or 45 or 46 or 47 or 48 or 49 or 50 or 51 or 52 or 53 or 54 or 55 or 56 or 57 or 58 or 59 or 60 or 61 or 62 or 63 or 64 or 65 or 66 or 67 or 68 or 69 or 70 or 71 or 72 or 73 or 74 or 75 or 76 or 77 or 78 or 79 (269674)

81 (tocilizumab* or actemra* or atlizumab* or lusinex* or r1569 or r 1569 or roactemra* or mra or 375823 41 9 or i031v2h011* or rhpm-1 or rhpm1 or rg-1569 or rg1569 or msb-11456 or msb11456 or ro-4877533 or ro4877533).mp,rn. (12483)

82 (sarilumab* or kevzara* or regn 88 or regn88 or sar 153191 or sar153191 or 1189541 98 7 or nu90v55f8i*).mp,rn. (171)

83 (levilimab* or BCD-089 or bcd089 or 2035008 70 7 or P7UV3L2H80*).mp,rn. (0)

84 (olokizumab* or cdp 6038 or cdp6038 or 1007223 17 7 or pai71r1d2w*).mp,rn. (17)

85 (clazakizumab* or ald 518 or ald518 or bms 945429 or bms945429 or 1236278 28 6 or 4s38z8ra9o*).mp,rn. (28)

86 (sirukumab* or cnto 136 or cnto136 or pilvensia or 1194585 53 9 or 640443FU93*).mp,rn. (55)

87 (siltuximab* or sylvant or cnto 328 or cnto-328 or cnto328 or cllb8 or ccib8 monoclonal antibody or sylvant or t4h8fma7im* or 541502 14 1).mp,rn. (182)

88 (ziltivekimab* or zilti or "cor-001" or "2226654 05 1").mp,rn. (2)

89 (elsilimomab* or "b-e8" or 468715 71 1).mp,rn. (12)

90 81 or 82 or 83 or 84 or 85 or 86 or 87 or 88 or 89 (12766)

91 80 and 90 (1184)

92 Interleukin 1 Receptor Antagonist Protein/ (5221)

93 (anakinra* or kineret or ((interleukin 1 receptor or il1 receptor or il 1 receptor) adj1 antagonist protein*) or (recombinant interleukin 1 receptor adj1 (antagonist* or blocker* or blocking agent*)) or antril or il 1ra or il1ra or il 1 ra or ((urin* or febrile) adj2 (interleukin 1 inhibitor* or il1 inhibitor* or il 1 inhibitor*)) or "143090 92 0" or 9013duq28k*).mp,rn. (9574)

94 Infliximab/ (10393)

95 (infliximab* or ifx or avakine or flixabi or inflectra or inflecta or ixifi or remicade or remsima or revellex or renflexis or zessly or mab ca2 or monoclonal antibody ca2 or antibody ca2 monoclonal or ca2 monoclonal antibody or ct p13 or ctp13 or ct-p-13 gp-1111 or gp1111 or ta-650 or ta650 or abp 710 or abp710 or bow-015 or bow015 or 170277 31 3 or b72hh48flu* or sb2 or "pf 06438179" or pf 6438179 or pf06438179 or pf6438179 or hsdb 7850 or hsdb7850).mp,rn. (15807)

96 Etanercept/ (5897)

97 (etanercept* or benepali or embrel or enbrel or enia 11 or enia11 or erelzi or lifmior or opinercept or recombinant tumo?r necrosis factor receptor fc fusion protein or "tnr 001" or tnr001 or tnfr-fc or tnrfr:fc or rhu tnfr:fc or rhu-tnfr:fc or tnfr-immunoadhesin or tnt receptor fusion protein or tum?or necrosis factor receptor fc fusion protein or tunex or "185243 69 0" or op401g7ojc* or brenzys or tnf receptor fusion protein or tnfr fc or recombinant human tnf or

recombinant human dimeric tnf receptor type ii igg fusion protein or chs-0214 or chs0214 or dwp-422 or dwp422 or enia-11 or enia11 or gp-2015 or gp2015 or gp2015c or gp-2015c or hd-203 or hd203 or lbec-0101 or lbec0101 or sb-4 or sb4).mp,rn. (9460)

98 Adalimumab/ (5464)

99 (adalimumab* or humira or abp 501 or abp501 or abtd2e7 or amjevita or amgevita or bax 2923 or bax2923 gp 2017 or gp2017 or halimatoz or hefiya or hulio or hyrimoz or ibi 303 or ibi303 or imraldi or m 923 or m923 or msb 11022 or msb11022 or ons3010 or ons 3010 or "pf 06410293" or pf 6410293 or pf06410293 or pf6410293 or d2e7 or truxeda or cyltezo or solymbic or fys6t7f842* or 331731 18 1 or lu 200134 or lu200134 or d2e7 or hsdb 7851 or hsdb7851 or abp-501 or abp501 or bcd-057 or bcd057 or bi-695501 or bi695501 or chs-1420 or chs1420 or gp-2017 or gp2017 or sb-5 or sb5).mp,rn. (9079)

100 (golimumab* or cnto 148 or cnto148 or simponi or 476181 74 5 or 91x1klu43e*).mp,rn. (1307)

101 Certolizumab Pegol/ (613)

102 (certolizumab* or cdp 870 or cdp870 or cimzia or pegylated tumor necrosis factor alpha antibody fab fragment or pegylated tumour necrosis factor alpha antibody fab fragment or pha 738144 or pha738144 or czp or 428863 50 7 or g6adw90r16* or umd07x179e* or hsdb 7848 or hsdb7848).mp,rn. (1560)

103 Rituximab/ (15011)

104 (rituximab* or ct p10 or ctp10 or idec 102 or idec102 or idecc2b8 or idec c2b8 or monoclonal antibody idec c2b8 or gp2013 or gp 2013 or "pf 05280586" or pf05280586 or mabthera or mab thera or r 105 or r105 or reditux or rg 105 or rg105 or rituxan or rituxin or ritemvia or rituzena or rixathon or riximyo or ro 452294 or ro452294 or truxima or 174722 31 7 or 4f4x42syq6* or hsdb 7455 or hsdb7455).mp,rn. (24750)

105 (ofatumumab* or humax cd20 or humax-cd20 or humaxcd20 or humax-cd20-2f2 or arzerra or gsk 1841157 or gsk1841157 or gski841157 or humax cd20 or omb 157 or omb157 or 679818 59 8 or m95kg522r0* or hsdb 8170 or hsdb8170).mp,rn. (587)

106 Abatacept/ (2943)

107 (abatacept* or ctla4 ig or ctla4 immunglobulin or ctla4 immunoglobulin g or ctla4ig or 'ctla 4 ig' or ctla-igg4m or ctla4-fc or bms 188667 or bms188667 or orencia or '332348 12 6' or 7d0yb67s97* or rg 2077 or rg2077 or rg-1046 or rg1046 or nulojix or lea29y or lea 29y or ctl4 fc or ctl4fc or belatacept or bms 224818 or bms224818 or belatacept or cytotoxic t lymphocyte associat* antigen 4).mp,rn. (5423)

108 (tabalumab* or ly 2127399 or ly2127399 or 1143503 67 6 or pqp8vh3mjw*).mp,rn. (58)

109 (pateclizumab* or mlta 3698a or mlta3698a or pro 283698 or pro283698 or 12202526 59 7 or qok1yyh7j2* or rg 7415 or rg7415).mp,rn. (6)

110 (secukinumab* or cosentyx or ain 457 or ain457 or 1229022 83 6 or dlg4eml025*).mp,rn. (1220)

111 (ixekizumab* or ly 2439821 or ly2439821 or taltz or 1329632 62 3 or 1143503 69 8 or bty153760o*).mp,rn. (608)

112 (brodalumab* or amg 827 or amg827 or khk 4827 or khk4827 or siliq or kyntheum or 1174395 19 7 or 6za31y954z*).mp,rn. (352)

113 (guselkumab* or cnto 1959 or cnto1959 or 1350289 85 8 or tremfya or 1350289 85 8 or 089658a12d*).mp,rn. (265)

114 Ustekinumab/ (1105)

115 (ustekinumab* or cnto 1275 or cnto1275 or stelara or "815610 63 0" or fu77b4u5z0* or 1275 cnto or 1275cnto or l04ac05 or tt-20 or tt20).mp,rn. (2180)

116 (mavrilimumab or "1085337 57 0" or 1158jdp9a* or cam 3001 or cam3001).mp,rn. (33)

117 exp Antirheumatic Agents/ (430636)

118 (dmard* or csdmard* or cs-dmard* or sdmard* or s-dmard* or tsdmard* or ts-dmard* or bdmard* or b-dmard*).ti,ab,kf. (5791)

119 (disease modifying adj3 (antirheum* or anti rheum*) adj3 (drug? or agent?)).ti,ab,kf. (6420)

120 ((antirheum* or anti rheum*) adj5 (drug? or agent?)).ti,ab,kf. (9036)

121 Methotrexate/ (38192)

122 (mxt or methotrexat* or metotrexat* or methoblastin or methylaminopterin* or amet?opterin* or abitrexate or antifolan or brimexate or a-met?opterin* or a-methpterin* or ai3-25299 or ai325299 or alpha-methopterin or biotrexate or emtexate or emtrexate* or emthexat* or enthexate* or farmitrexat* or farmotrex or fauldexato or folex or folex pfs or hdmtx or ifamet or imeth or maxtrex or metotressat* or methotrate* or methohexate* or mexate* or metrotex or metatrexato* or metex or metrex or metatrexan or maxtrex or meticil or metoject or metotrexin or novatrex* or neotrexate* or nordimet or lumexon or ledertrexate* or lanterel or rasuvo or r?eumatrex* or reditrex or rasuvo or texate or texate-t or tremetex or trexeron or trixilem or texorate or trexall or xaken or xatmep or otrexup or mpi-2505 or mpi2505 or mpi 5004 or mpi5004 or amethopterin* or 133073-73-1 or 15475-56-6 or 3ig1e710zn* or yl5fz2y5u1* or cl 14377 or cl14377 or ccris 1109 or ccris1109 or emt 25299 or emt25299 or nsc-740 or nsc740 or r-9985 or r9885 or x-133 or wr-19039 or wr19039).mp,rn. (55522)

123 Leflunomide/ (1533)

124 (leflunomid* or hwa 486 or hwa486 or su101 or su 101 or arava or arabloc or hsdb 7289 or hsdb7289 or g162gk9u4w* or 75706-12-6 or rs 34821 or rs34821).mp,rn. (2609)

125 lef.ti,ab,kf. (2544)

126 Sulfasalazine/ (4137)

127 (sulfasalazin* or sasp or ssz or salicylazosulfapyridin* or salicyl azo sulfapyridin* or sulphasalazin* or salazosulfapyridin* or salazosulfapiridin* or 'pyralin en' or azulfadin* or azulfidin* or asulfidin* or azulfide* or azulfin or colo-pleon or colopleon or disalazin or gastropyrin or pleon or "pleon ra" or "pyralin en" or rorasul or rosulfant or ulcol or ucine or salazopyrin* or ratio-sulfosalazin* or sulfasalazin* or 3xc8guz6cb* or accucol or asulfidin* or azopyrin* or azosulfidin* or benzosulfa or "brn 0356241" or brn0356241 or ccris 4713 or ccris4713 or einecs 209-974-3 or hsdb 3395 or hsdb3395 or nsc 203730 or nsc203730 or nsc 667219 or nsc667219 or reupirin or rorasul or salazo-sulfapyridin* or salazosulfpyridin* or salazopiridazin* or salazopyridin* or salazopirin* or salazodin or salisulf or salipyr or saridine or si-88 or si88 or sulculon or sulfasalazin* or sulphasalazin* or w-t sasp oral or 599-79-1 or "s.a.s. 500" or "s.a.s.-500" or sas-500 or zopyrin*).mp,rn. (8027)

128 Hydroxychloroquine/ (3886)

129 (hydroxychloroquin* or hcq or 747-3-4 or 8q2869cnvh* or 118-42-3 or 4qwg6n8qkh* or hydroxychlorochin* or hydrochloroquin* or hydrocloroquin* or oxychlorochin or oxychloroquin* or plaquenil or plaquinol or "brn 0253894" or brn0253894 or einecs 204-249-8 or einecs204-249-8 or hidroxicloroquin* or idrossiclorochin* or oxichlorochin* or oxichloroquin* or oxiklorin or win1258 or win 1258 or z0188 or einecs 212-019-3 or einecs212-019-3 or chloroquinol or dimard or ercoquin or evoquin or erquin or geniquin or quensyl or sn 8137 or sn8137 or toremonil or yuma).mp,rn. (7447)

130 Gold Sodium Thiomalate/ (1262)

131 (gold sodium thiomalate* or aurothiomalate* or aurolate* or gold thiomalate* or gold disodium thiomalate* or gold thiomalic acid* or miocrin or miocrisin or monogold disodium thiomalate* or myochrysin* or myocrisin* or myocrysin* or sodium aurothiomalate* or sodium gold thiomalate* or sodium thiomalate* gold or sodium thiomalatoaurate* or tauredon or taureodon or tauredone or thiomalate gold or thiomalatoaurate sodium or 1244-57-4 or 39377-38-3 or 4846-27-9 or 74916-57-7 or e4768zy6gm* or aurothiomala-natrium or aurot?iomalato sodico or monogold* sodium salt or dinatrium 2-aurothio-succinat or einecs 235-479-7 or einecs235-479-7 or hsdb 7173 or hsdb7173 or kidon or natrii aurothiomalas* or shiosol or gold mercatpsuccinate*).mp,rn. (1545)

132 Aurothioglucose/ (834)

133 (thioglucosoaurate* or gold thioglucose* or gold thio glucose* or thioglucose gold or aureotan or solganal or solganol or gold-50 or gold50 or solganal b or b oleosum solganal or auromyose* or aurothioglucose* or auro thioglucose* or aurotan or aurumine or aurumin or authron or brenol or ccris 59 or ccris59 or einecs 235-365-7 or einecs235-365-7 or glysanol b or goldthioglucose* or goldtioglucose* or hsdb 7174 or hsdb7174 or oronol or romosol or skf 10056 or skf10056 or 2p2v9q0e78* or 12192-57-3).mp,rn. (1133)

134 ((gold* or auro*) adj10 (inject* or intramuscular* or im or intra muscular*)).ti,ab,kf,kw. (2755)

135 Organogold Compounds/ (935)

136 (aurothiopropanol* or allochrysine or allocrisine or aurotiopoe or aurotioprol or 27279-43-2 or g7097j63e9* or auromercapto propanol sulfonate sodium or auromercaptopropanolsulfonate sodium or aurothioisopropanolsulfonate sodium or sodium aurothiopropanolsulfonate or gold sodium thiopropanol sulfonate or sodium auromercaptopropanol sulfonate).mp,rn. (91)

137 Chloroquine/ (14555)

138 (chloroquin* or cq or 54-04-7 or 886u3h6uff* or aralen or aralene or arechin or arechine or arequin or chingamin or chlorochin* or khingamin or nivaquine or oe48649kh6n* or amokin or amokine or anaclor or aralan or arequine or artriquine or avloclor or avoclor or arthrochin or artrichin or bemaco or bemaphata or bemaphate or bemasulph or benaquin or bipiquin or cadiquin or "brn 0482809" or brn0482809 or capquin or ccris 3439 or ccris3439 or chemochin or chemochine or chingamine or chingaminum or chloraquin* or chlorquin or chlorquine or choloquine or choroquine sulfate or choroquine sulphate or cindacin or clo-kit junior or clorichina or clorichine or cloriquine or clorochina or delagil or delagyl or diclokin or chlorofoz or cindachin or clorochina or cloroquina or cocatrit or dichinalex or diquinalex or diroquine or emquin or genocin or gontochin or gontochine or gontoquine or einecs 200-191-2 or einecs200-191-2 or elestol or gontochin or heliopar or hsdb 3029 or hsdb3029 or imagon or iroquine or klorokin or klorokine or klorokinfosfat or lagaquin or lapaquin or malaquin or malaren or malarex or malarivon or malaviron or maliaquine or maquine or mesylith or mexaquin or mirquin or neochin or nivaquin or nivachine or nivaquine b or nivaquine dp or nivaquine forte or "p roquine" or nsc 187208 or nsc187208 or pfizerquine or quensyl or quinachlor or quinercyl or quingamine or quinilon or quinoline or quinoscan or repal or resochen or resochene or resochin or resochin junior or resochina or resochine or resochinon or resoquina or resoquine or reumachlor or ronaquine or roquine or rp 3377 or rp3377 or sanoquin or sanoquine or silbesan or siragan or sirajan or sn 7618 or sn7618 or solprina or solprine or sopaquin or st 21 or tresochin or tresoquine or trochin or trochine or troquine or weimerquin or w 7618 or w7618 or win 244 or win244).mp,rn. (34915)

139 exp Adrenal Cortex Hormones/ (398870)

140 exp Prednisolone/ (51393)

141 Prednisone/ (39584)

142 Cortisone/ (19608)

143 Hydrocortisone/ (72351)

144 exp Steroids/ (861054)

145 adrenal cortex hormones.ti,ab,kf,rn. (65291)

146 prednisolone*.mp,rn. (46528)

147 methylprednisolone*.mp,rn. (27014)

148 (prednisolone* or predonine or di-adreson-f or diadresonf or 9phq9y1olm* or prednisolona* or prednisolonum* or nsc-9120 or nsc9120 or nsc 9900 or nsc9900 or delta-dehydrocortisol* or delta-dehydrocortisone* or delta-hydrocortisone or hydroretrocortine or metacortandralone* or brn 1354103 or brn1354103 or bubbli-pred or ccris 980 or ccris980 or co-hydeltra or codelcortone or cordrol or cortalone or cotogesic or cotolone or decaprednil or decortin h or delcortol or dehydrohydrocortisone* or delta-cortef or deltacortef or delta-ef-cortelan or delta-stab or deltacortenol or deltacortril or deltahydrocortisone* or deltisilone or derpo pd or dexa-dortidelt hostacortin h or di-adreson f or dicortol or donisolone or dydeltrone or eazolin d or ec 200-021-7 or ec200-021-7 or einecs 200-021-7 or einecs200-021-7 or erbacort or erbasona or estilsona or fernisolone or fernisolone p or hostacortin h or hsdb 3385 or hsdb3385 or hydeltra or hydeltrone or hydrodeltalone or hydrodeltisone or hydroretrocortin or hydroretrocortine* or k 1557 or k1557 or lentosone or meti-drm or meticortelone or orapred or orapred odt or paracortol or paracotol or pediapred or precortancyl or precortilon or precortisyl or predne-dome or prednelan or prednicen or predniliderm or predniretard or prednis or predonin or predonine* or prelon or prelone or prenolone or rolisone or scherisolon? or solone or sterolone or ulacort or ultracorten h or ultracortene h adelcort or antisolon or antisolone or aprednisolon? or benisolon? or caberdelta or co hydeltra or codelcortone or ompresolon).mp,rn. (46616)

149 (cortadeltona or cortelinter or cortisolone or dacortin or dacortin h or dacrotin or decaprednil or decortil or delta cortril or delta hycortol or deltacortenolo or deltacortil or deltacortoil or deltaderm or deltadglycortril or deltahycortol or deltahydrocortison? or deltaophticor or deltasolone or deltastab or deltidrosol or deltisilone or deltisolon? or deltolasson? or deltosona or deltosone or depo-predate or dermosolon or dhasolone or di adreson? f or diadreson? f or dicortol or domucortone or encortelon? or encortolon or equisolon or glistelone or hefasolon or hydrelta or hydrocortancyl or hydrocortidelt or hydradeltalone or hydradeltisone or inflanefran or insolone or keteocort h or key-pred or key-pred sp or lenisolone or leocortol or liquipred or mediasolone or meprisolon? or metacortalon? or metacortelone or meti derm or metiderm or meticortelone or morlone or mydrapred or nisolon? or opredsone or panafcortelone or panafcortolone or panafort or paracortol pr phlogex or pre cortisyl or preconin or precortalon or precortancyl or precortisyl or pred ject 50 or predacort 50 or predaject 50 or predalone 50 or predartrina or predartrine or predate 50 or predeltilone or predisole or predisyr or predne dome or prednecort or prednedome or prednelan or predni coelin or predni h tablinen or predni helvacort or prednicoelin or prednicort or prednicortelone or prednifor drops or predniment or prednorsolon? or predorgasolon? or prenilone or preventan or prezolon or rubycort or serilone or solonda or solupren? or spiricort or spolotane or supercorti#ol or taracortelone or walesolone or wysolone).mp,rn. (63)

150 (methylprednisolone * or methylprednisolona* or methylprednisolonum* or metipred or urbason or medrol or x4w7zr7023* or 83-43-2 or besonia or brn 23403300 or brn2340300 or a-methapred or artisone-wyeth or besonia or depo-medrol or dopomedrol ec 201-4764 pr ec2014764 or einecs 201-476-4 or einecs201-476-4 or esametone or firmacort or hsdb3127 or hsdb3127 or lemod or medesone or medixon or medlone 21 or medrate or medrol or medrone or mesopren or metastab or methyleneprednisolone* or metilbetasone or metrisone or metrocort or metysolon or moderin or nirypan or noretona or nsc 19987 or nsc19987 or predni n tablinen or prednol-l or promacortine or reactenol or sieropresol or solomet or summicort or suprametil or u 7532 or u7532 or "u 67

590a" or urbason or urbasone or wyacort or adlone 40 or adlone 80 or beta methylprednisolone or medalone 80 or depmedalone or deproject 80 or depopred or esametone or firmacort or med-jec-40 or medixon or mednin or medralone or mepredprednisolone or meprelon or mesopren or methacort 40 or methacort 80 or methyxlcotol or methylcotolone or methylpred dp or methylsterolone or metidrol or metycortin or metypred or metypresol or neomedrone or solu decortin or urbason).mp,rn. (19800)

151 (prednison or prednisone or prednisona or prednisonum or dehydrocortisone or delta cortisone or rectodelt or sterapred or ultracorten or winpred or apo prednisone or cortan or cartancyl or panafcort or cutason or decortin or dacortin or decortisyl or deltasone or encortone or encorton or enkorton or enkortolon or kortancyl or liquid pred or meticorten or orasone or panasol or predni tablinen or prednidib or predniment or pronisone or vb0r961hzt* or 53-03-2 or adasone or al3-52939 or ancortone or bicortone or cartancyl or ccris 2646 or ccrs2646 or colisone or cortan or cortidelt or cotone or dacorten or dacortin or decortancyl or dekortin or dellacort or delta cortelan or delta cortisone or delta dome or deltacortene or deltacortisone or deltacortone or deltasone or deltison or deltisona or deltra or di adreson or diadreson or econosone or einecs 200-160-3 or einecs200-160-3 or fernisone or fiasone or hostacortin or hsdb 3168 or hsdb3168 or incocortyl or juvason or lisacort or lodotra or lodtra).mp,rn. (54014)

152 (me-korti or metacortandracin or metacorten or nci c04897 or ncic04897 or nisona or nizon or novoprednisone or nsc 10023 or nsc10023 or nurison or orasone or panafcort or paracort or prarmenison or pehacort or predeltin or prednicen-m or prednicorm or prednicort or prednicot or prednilonga or prednitone or prednizon or prednovister or presone or pronison or rayos or rectodelt or retrocortine or servisone or sterapred or supercortio or u 6020 or u6020 or ultracorten or ultracortene or winpred or wojtab or zenadrid).mp,rn. (354)

153 cortisone?.mp,rn. (23536)

154 corticosteroid*.mp,rn. (106098)

155 corticoid*.mp,rn. (6580)

156 glucocorticoid*.mp,rn. (114028)

157 corticosterone*.mp,rn. (34763)

158 hydrocortisone*.mp,rn. (77800)

159 dexamethasone*.mp,rn. (72756)

160 beclomethasone*.mp,rn. (3844)

161 (triamcinolone* or 1zk20vi6ty* or 124-94-7 or aristocort or volon or fluoxyprednisolon* or tiamcinolonum or triamcinolonum* or triamcinolona* or brn 2341955 or brn2341955 or cl 19823 or cl19823 or einecs 204-718-7 or einecs204-718-7 or hsdb 3194 or hsdb3194 or nsc 13397 or nsc13397).mp,rn. (11949)

162 (steroid? adj10 (therap* or treat* or drug? or inject* or intramuscular* or intra muscular* or im or intravenous* or intra venous* or iv or intraarticular* or intra articular* or ia or administrat* or oral or orally or parenteral*)).ti,ab,kf. (73951)

163 Janus Kinase Inhibitors/ (438)

164 ((jak or jak1 or jak2 or jak3 or janus kinase) adj10 inhibitor*).mp,rn. (6424)

165 (janus adj4 kinase adj10 inhibitor?).mp,rn. (2539)

166 exp Janus Kinases/ai [Antagonists & Inhibitors] (2291)

167 (baricitinib* or 1187594-09-7 or isp4442i3y* or "incb 028050" or incb028050 or ly-3009104 or ly3009104).mp,rn. (429)

168 (tofacitinib* or 477600-75-2 or 87la6fu830* or hsdb 8311 or hsdb8311 or cp-690 550 or cp 690550 or cp690550).mp,rn. (1600)

169 (filgotinib* or 1206161-97-8 or 3xvl385q0m* or "glpg 0634" or glpg0634).mp,rn. (111)

170 (upadacitinib* or 1310726-60-3 or 4RA0KN46E0* or abt 494 or abt494).mp,rn. (138)

171 (ruxolitinib* or rux or 941678-49-5 or 82s8x8xx8h* or hsdb 8259 or hsdb8259 or inc 424 or inc424 or incb424 or "incb 018424" or incb018424 or incb 18424 or incb18424 or r-ruxolitinib* or jakafi or jakavi).mp,rn. (1699)

172 (itacitinib or "incb 039110" or incb039110 or incb 39110 or incb39110 or 1334298-90-6 or 19j3781lpm*).mp,rn. (26)

173 (ag490 or ag 490 or tyrphostin b42 or tyrphostin ag 490 or tyrphostin ag490 or 133550-30-8).mp,rn. (1521)

174 (fedratinib* or tg101348 or tg 101348 or sar302503 or sar 302503 or 936091-26-8 or 6l1xp550i6* or inrebic).mp,rn. (146)

175 (ritlecitinib* or pf-06651600 or pf06651600 or pf 6651600 or pf6651600 or 2140301-97-7 or 132lf5wgh4* or 2192215-81-7 or eag4t1459k* or ritlecitinib malonate or ritlecitinib toslate or ritlecitinib tosylate or ritlecitinib propanedioate).mp,rn. (10)

176 (peficitinib* or peficitinib hydrobromide).mp,rn. (64)

177 or/92-176 (1526572)

178 80 and 177 (44139)

179 91 or 178 (44617)

180 randomized controlled trial.pt. (517933)

181 controlled clinical trial.pt. (93945)

182 randomi*.ti,ab,kf. (662146)

183 placebo*.ti,ab,kf,hw. (234149)

184 exp placebo effect/ (4871)

185 Random Allocation/ (104095)

186 randomly.ti,ab,kf. (346537)

187 exp Randomized Controlled Trials as Topic/ (141609)

188 clinical trial/ or clinical trial, phase ii/ or clinical trial, phase iii/ or clinical trial, phase iv/ (564238)

189 clinical trials as topic/ or clinical trials, phase ii as topic/ or clinical trials, phase iii as topic/ or clinical trials, phase iv as topic/ (208310)

190 trial.ti,ab. (617309)

191 ((singl* or doubl* or trebl* or tripl*) adj3 (blind* or dumm* or mask*)).ti,ab,kf. (177877)

192 Single-Blind Method/ (29353)

193 Double-Blind Method/ (161093)

194 Cross-Over Studies/ (49007)

195 (crossover or cross over).ti,ab,kf. (87176)

196 (parallel adj1 group*).ti,ab,kf. (19439)

197 (controlled adj8 (study or trial or design)).ti,ab,kf. (274759)

198 (assigned or allocated).ti,ab. (313725)

199 ((assign* or match or matched or allocation) adj6 (alternate or group or groups or intervention? or patient? or subject? or participant?)).ti,ab. (265570)

200 drug therapy.fs. (2254909)

201 groups.ti,ab. (2147169)

202 ('head to head' adj3 (study or comparison or trial)).ti,ab,kf. (2392)

203 200 or 201 or 202 (4186558)

204 random*.mp. [mp=title, abstract, original title, name of substance word, subject heading word, floating sub-heading word, keyword heading word, organism supplementary concept word, protocol supplementary concept word, rare disease supplementary concept word, unique identifier, synonyms] (1413319)

205 203 and 204 (623722)

206 180 or 181 or 182 or 183 or 184 or 185 or 186 or 187 or 188 or 189 or 190 or 191 or 192 or 193 or 194 or 195 or 196 or 197 or 198 or 199 or 205 (2193707)

207 179 and 206 (6699)

208 exp animals/ not humans/ (4760027)

209 207 not 208 (6598)

210 limit 209 to english (6025)

211 limit 210 to yr="2012 - 2020" (3262)

212 review.pt. (2722892)

213 editorial.pt. (548187)

214 letter.pt. (1109554)

215 congress.pt. (66444)

216 212 or 213 or 214 or 215 (4419539)

217 211 not 216 (2257

1.3.2.2 EMBASE

#211,"#209 NOT #210",3324,27 Nov 2020

#210,"(#206 OR #208) AND [review]/lim",930,27 Nov 2020

#209,"#206 OR #208",4254,27 Nov 2020

#208,"#201 NOT #207",3825,27 Nov 2020

#207,"#195 NOT #200 AND ([conference abstract]/lim OR [conference paper]/lim OR [conference review]/lim OR [editorial]/lim OR [letter]/lim)",4637,27 Nov 2020

#206,"#201 AND #204 AND [2019-2020]/py",429,27 Nov 2020

#205,"#201 AND #204",1982,27 Nov 2020

#204,"#202 OR #203",50862,27 Nov 2020

#203,"acr:nc AND annual:nc AND meeting:nc AND rheumatology:nc",19451,27 Nov 2020

#202,"'eular':nc AND 'annual european congress of rheumatology':nc",31411,27 Nov 2020

#201,"#195 NOT #200",8462,27 Nov 2020

#200,"#196 OR #197 OR #198 OR #199",951075,27 Nov 2020

#199,"('case control*':ti,ab OR 'case control*':tt) AND (random*:ti,ab OR random*:tt) NOT ('randomi?ed controlled':ti,ab OR 'randomi?ed controlled':tt)",17715,27 Nov 2020

#198,"('systematic review':ti OR 'systematic review':tt) NOT (trial:ti OR trial:tt OR study:ti OR study:tt)",157626,27 Nov 2020

#197,"'we searched':ab AND (review:it OR review:ti OR review:tt)",33899,27 Nov 2020

#196,"'review':ab AND 'review':it NOT (trial:ti OR trial:tt)",830417,27 Nov 2020

#195,"#193 NOT #194",9772,27 Nov 2020

#194,"(mouse:ti OR mice:ti OR murine$:ti OR rat:ti OR rats:ti OR swine:ti OR bovine:ti OR porcine:ti OR pigs:ti OR piglets:ti OR rabbit$:ti OR dog:ti OR dogs:ti OR cat:ti OR cats:ti OR lamb:ti OR lambs:ti OR sheep:ti OR cattle:ti OR monkey:ti OR monkeys:ti OR trout:ti OR marmoset*:ti OR mouse:tt OR mice:tt OR murine$:tt OR rat:tt OR rats:tt OR swine:tt OR bovine:tt OR porcine:tt OR pigs:tt OR piglets:tt OR rabbit$:tt OR dog:tt OR dogs:tt OR cat:tt OR cats:tt OR lamb:tt OR lambs:tt OR sheep:tt OR cattle:tt OR monkey:tt OR monkeys:tt OR trout:tt OR marmoset*:tt) AND 'animal experiment'/de",1089428,27 Nov 2020

#193,"#189 NOT #192",9773,27 Nov 2020

#192,"#190 NOT #191",5712932,27 Nov 2020

#191,"'human'/exp OR 'human experiment'/exp",22688490,27 Nov 2020

#190,"'animal'/exp OR 'animal experiment'/exp OR 'animal model'/exp",28400105,27 Nov 2020

#189,"#165 AND #186 AND [english]/lim AND [2012-2020]/py",9885,27 Nov 2020

#188,"#165 AND #186 AND [english]/lim",15654,27 Nov 2020

#187,"#165 AND #186",16282,27 Nov 2020

#186,"#166 OR #167 OR #168 OR #169 OR #170 OR #171 OR #172 OR #173 OR #174 OR #175 OR #176 OR #177 OR #178 OR #179 OR #180 OR #181 OR #182 OR #183 OR #184 OR #185",2672046,27 Nov 2020

#185,"(('head to head' NEAR/3 (study OR comparison OR trial)):ti,ab,kw) AND random*:ti,ab,kw,de",1601,27 Nov 2020

#184,"((parallel NEXT/1 group*):ti,ab) OR ((parallel NEXT/1 group*):tt)",26381,27 Nov 2020

#183,"'crossover procedure'/de OR crossover:ti,ab,kw OR 'cross over':ti,ab,kw OR crossover:tt OR 'cross over':tt",119608,27 Nov 2020

#182,"(((singl* OR doubl* OR trebl* OR tripl*) NEAR/3 (blind* OR dumm* OR mask*)):ti,ab,kw) OR (((singl* OR doubl* OR trebl* OR tripl*) NEAR/3 (blind* OR dumm* OR mask*)):tt)",248877,27 Nov 2020

#181,"'single blind procedure'/de OR 'double blind procedure'/de OR 'triple blind procedure'/de",217336,27 Nov 2020

#180,"(controlled NEAR/8 (study OR trial OR design)):tt",61,27 Nov 2020

#179,"(controlled NEAR/8 (study OR trial OR design)):ti,ab,kw",376237,27 Nov 2020

#178,"((clin* NEAR/6 trial):ti,ab) OR ((clin* NEAR/6 trial):tt)",259886,27 Nov 2020

#177,"trial:ti OR trial:tt",320118,27 Nov 2020

#176,"'phase 2':ti,ab,kw OR 'phase 3':ti,ab,kw OR 'phase 4':ti,ab,kw OR 'phase ll':ti,ab,kw OR 'phase lll':ti,ab,kw OR 'phase lv':ti,ab,kw OR 'phase ii':ti,ab,kw OR 'phase iii':ti,ab,kw OR 'phase iv':ti,ab,kw OR 'phase 2':tt OR 'phase 3':tt OR 'phase 4':tt OR 'phase ll':tt OR 'phase lll':tt OR 'phase lv':tt OR 'phase ii':tt OR 'phase iii':tt OR 'phase iv':tt",218644,27 Nov 2020

#175,"'clinical trial'/de OR 'phase 2 clinical trial'/exp OR 'clinical trial, phase 2' OR 'phase 2 clinical study' OR 'phase 2 clinical trial' OR 'phase 2 study' OR 'phase 2 trial' OR 'phase ii clinical study' OR 'phase ii clinical trial' OR 'phase ii study' OR 'phase ii trial' OR 'phase 3 clinical trial'/exp OR 'clinical trial, phase 3' OR 'phase 3 clinical study' OR 'phase 3 clinical trial' OR 'phase 3 study' OR 'phase 3 trial' OR 'phase iii clinical study' OR 'phase iii clinical trial' OR 'phase iii study' OR 'phase iii trial' OR 'phase 4 clinical trial'/exp OR 'clinical trial, phase 4' OR 'phase 4 clinical study' OR 'phase 4 clinical trial' OR 'phase 4 study' OR 'phase 4 trial' OR 'phase iv clinical study' OR 'phase iv clinical trial' OR 'phase iv study' OR 'phase iv trial'",1152294,27 Nov 2020

#174,"'controlled clinical trial (topic)'/exp",198345,27 Nov 2020

#173,"randomly:ti,ab,kw OR randomly:tt",457932,27 Nov 2020

#172,"'placebo effect'/de",6270,27 Nov 2020

#171,"'placebo*':ti,ab,kw OR 'placebo*':tt",317297,27 Nov 2020

#170,"'placebo'/de",364796,27 Nov 2020

#169,"'randomization'/de",88511,27 Nov 2020

#168,"randomi*:ti,ab,kw OR randomi*:tt",949281,27 Nov 2020

#167,"'controlled clinical trial'/de",431732,27 Nov 2020

#166,"'randomized controlled trial'/de",630730,27 Nov 2020

#165,"#87 OR #164",93537,27 Nov 2020

#164,"#76 AND #163",92785,27 Nov 2020

#163,"#88 OR #89 OR #90 OR #91 OR #92 OR #93 OR #94 OR #95 OR #96 OR #97 OR #98 OR #99 OR #100 OR #101 OR #102 OR #103 OR #104 OR #105 OR #106 OR #107 OR #108 OR #109 OR #110 OR #111 OR #112 OR #113 OR #114 OR #115 OR #116 OR #117 OR #118 OR #119 OR #120 OR #121 OR #122 OR #123 OR #124 OR #125 OR #126 OR #127 OR #128 OR #129 OR #130 OR #131 OR #132 OR #133 OR #134 OR #135 OR #136 OR #137 OR #138 OR #139 OR #140 OR #141 OR #142 OR #143 OR #144 OR #145 OR #146 OR #147 OR #148 OR #149 OR #150 OR #151 OR #152 OR #153 OR #154 OR #155 OR #156 OR #157 OR #158 OR #159 OR #160 OR #161 OR #162",1462236,27 Nov 2020

#162,"'peficitinib'/de OR '4 [(5 hydroxyadamantan 2 yl) amino] 1h pyrrolo [2, 3 b] pyridine 5 carboxamide' OR '4 [(5 hydroxytricyclo [3.3.1.1 3, 7] dec 2 yl) amino] 1h pyrrolo [2, 3 b] pyridine 5 carboxamide' OR 'asp 015k' OR 'asp015k' OR 'peficitinib' OR 'peficitinib hydrobromide'",186,27 Nov 2020

#161,"'ritlecitinib'/exp OR '1 [2 methyl 5 (7h pyrrolo [2, 3 d] pyrimidin 4 ylamino) 1 piperidinyl] prop 2 en 1 one' OR '1 [2 methyl 5 (7h pyrrolo [2, 3 d] pyrimidin 4 ylamino) piperidin 1 yl] prop 2 en 1 one' OR '1 [2 methyl 5 [(7h pyrrolo [2, 3 d] pyrimidin 4 yl) amino] 1 piperidinyl] prop 2 en 1 one' OR '1 [2 methyl 5 [(7h pyrrolo [2, 3 d] pyrimidin 4 yl) amino] piperidin 1 yl] prop 2 en 1 one' OR '1 [5 (7h pyrrolo [2, 3 d] pyrimidin 4 ylamino) 2 methyl 1 piperidinyl] prop 2 en 1 one' OR '1 [5 (7h pyrrolo [2, 3 d] pyrimidin 4 ylamino) 2 methylpiperidin 1 yl] prop 2 en 1 one' OR '1 [5 [(7h pyrrolo [2, 3 d] pyrimidin 4 yl) amino] 2 methyl 1 piperidinyl] prop 2 en 1 one' OR '1 [5 [(7h pyrrolo [2, 3 d] pyrimidin 4 yl) amino] 2 methylpiperidin 1 yl] prop 2 en 1 one' OR 'pf 06651600' OR 'pf 6651600' OR 'pf06651600' OR 'pf6651600' OR 'ritlecitinib' OR 'ritlecitinib malonate' OR 'ritlecitinib propanedioate'",40,27 Nov 2020

#160,"'fedratinib'/de OR 'fedratinib' OR 'fedratinib dihydrochloride' OR 'fedratinib dihydrochloride monohydrate' OR 'fedratinib hydrochloride' OR 'inrebic' OR 'n (1, 1 dimethylethyl) 3 [[5 methyl 2 [[4 [2 (1 pyrrolidinyl) ethoxy] phenyl] amino] 4 pyrimidinyl] amino] benzenesulfonamide' OR 'n tert butyl 3 [[5 methyl 2 [[4 (2 pyrrolidin 1 ylethoxy) phenyl] amino] pyrimidin 4 yl] amino] benzenesulfonamide' OR 'n tert butyl 3 [[5 methyl 2 [4 [2 (pyrrolidin 1 yl) ethoxy] anilino] pyrimidin 4 yl] amino] benzenesulfonamide' OR 'n tert butyl 3 [5 methyl 2 [4 (2 pyrrolidin 1 ylethoxy) phenylamino] pyrimidin 4 ylamino] benzenesulfonamide' OR 'n tert butyl 3 [5 methyl 2 [4 [2 (1 pyrrolidinyl) ethoxy] phenylamino] 4 pyrimidinylamino] benzenesulfonamide' OR 'sar 302503' OR 'sar 302503a' OR 'sar302503' OR 'sar302503a' OR 'tg 101348' OR 'tg101348'",653,27 Nov 2020

#159,"'n benzyl 2 cyano 3 (3,4 dihydroxyphenyl)acrylamide'/exp OR '2 cyano 3 (3, 4 dihydroxyphenyl) n (phenylmethyl) 2 propenamide' OR 'ag 490' OR 'ag490' OR 'n benzyl 2 cyano 3 (3,4 dihydroxyphenyl)acrylamide' OR 'tyrphostin ag 490' OR 'tyrphostin ag490'",2477,27 Nov 2020

#158,"'itacitinib'/de OR '1 [1 [[3 fluoro 2 (trifluoromethyl) 4 pyridinyl] carbonyl] 4 piperidinyl] 3 [4 (7h pyrrolo [2, 3 d] pyrimidin 4 yl) 1h pyrazol 1 yl] 3 azetidineacetonitrile' OR '1 [1 [[3 fluoro 2 (trifluoromethyl) pyridin 4 yl] carbonyl] piperidin 4 yl] 3 [4 (7h pyrrolo [2, 3 d] pyrimidin 4 yl) 1h pyrazol 1 yl] 3 azetidineacetonitrile' OR '[1 [1 (3 fluoro 2 trifluoromethylpyridine 4 carbonyl) 4 piperidinyl] 3 [4 (7h pyrrolo [2, 3 d] pyrimidin 4 yl) 1h pyrazol 1 yl] 3 azetidinyl] ethanenitrile' OR '[1 [1 [3 fluoro 2 (trifluoromethyl) pyridine 4 carbonyl] piperidin 4 yl] 3 [4 (7h pyrrolo [2, 3 d] pyrimidin 4 yl) 1h pyrazol 1 yl] azetidin 3 yl] ethanenitrile' OR 'incb 039110' OR 'incb 39110' OR 'incb039110' OR 'incb39110' OR 'itacitinib' OR 'itacitinib adipate' OR 'itacitinib hexanedioate'",183,27 Nov 2020

#157,"'ruxolitinib'/de OR '3 [4 (7h pyrrolo [2, 3 d] pyrimidin 4 yl) 1h pyrazol 1 yl] 3 cyclopentylpropanenitrile' OR '3 [4 (7h pyrrolo [2, 3 d] pyrimidin 4 yl) 1h pyrazol 1 yl] 3 cyclopentylpropanenitrile phosphate' OR '3 cyclopentyl 3 [4 (7h pyrrolo [2, 3 d] pyrimidin 4 yl) 1h pyrazol 1 yl] propanenitrile' OR 'beta cyclopentyl 4 (7h pyrrolo [2, 3 d] pyrimidin 4 yl) 1h pyrazole 1 propanenitrile' OR 'incb 018424' OR 'incb 18424' OR 'incb 424' OR 'incb018424' OR 'incb18424' OR 'incb424' OR 'jakafi' OR 'jakavi' OR 'ruxolitinib' OR 'ruxolitinib maleate' OR 'ruxolitinib phosphate'",5351,27 Nov 2020

#156,"'upadacitinib'/de OR '3 ethyl 4 (3h imidazo [1, 2 a] pyrrolo [2, 3 e] pyrazin 8 yl) n (2, 2, 2 trifluoroethyl) 1 pyrrolidinecarboxamide' OR '3 ethyl 4 (3h imidazo [1, 2 a] pyrrolo [2, 3 e] pyrazin 8 yl) n (2, 2, 2 trifluoroethyl) 1 pyrrolidinecarboxamide 2, 3 dihydroxybutanedioate' OR '3 ethyl 4 (3h imidazo [1, 2 a] pyrrolo [2, 3 e] pyrazin 8 yl) n (2, 2, 2 trifluoroethyl) 1 pyrrolidinecarboxamide tartrate' OR '3 ethyl 4 (3h imidazo [1, 2 a] pyrrolo [2, 3 e] pyrazin 8 yl) n (2, 2, 2 trifluoroethyl) pyrrolidine 1 carboxamide' OR '3 ethyl 4 (3h imidazo [1, 2 a] pyrrolo [2, 3 e] pyrazin 8 yl) n (2, 2, 2 trifluoroethyl) pyrrolidine 1 carboxamide 2, 3 dihydroxybutanedioate' OR '3 ethyl 4 (3h imidazo [1, 2 a] pyrrolo [2, 3 e]

pyrazin 8 yl) n (2, 2, 2 trifluoroethyl) pyrrolidine 1 carboxamide tartrate' OR 'abt 494' OR 'abt494' OR 'rinvoq' OR 'upadacitinib' OR 'upadacitinib 2, 3 dihydroxybutanedioate' OR 'upadacitinib hemihydrate' OR 'upadacitinib hydrate' OR 'upadacitinib tartrate'",553,27 Nov 2020

#155,"'filgotinib'/de OR 'filgotinib' OR 'filgotinib 2 butenedioate' OR 'filgotinib hydrochloride' OR 'filgotinib maleate' OR 'g 146034' OR 'g 146034 101' OR 'g 146034-101' OR 'g146034' OR 'g146034 101' OR 'g146034-101' OR 'glpg 0634' OR 'glpg0634' OR 'gs 6034' OR 'gs6034' OR 'n [5 [4 (1, 1 dioxothiomorpholinomethyl) phenyl] 1, 2, 4 triazolo [1, 5 a] pyridin 2 yl] cyclopropanecarboxamide' OR 'n [5 [4 (1, 1 dioxothiomorpholinomethyl) phenyl] 1, 2, 4 triazolo [1, 5 a] pyridin 2 yl] cyclopropanecarboxamide 2 butenedioate' OR 'n [5 [4 (1, 1 dioxothiomorpholinomethyl) phenyl] 1, 2, 4 triazolo [1, 5 a] pyridin 2 yl] cyclopropanecarboxamide but 2 enedioate' OR 'n [5 [4 [(1, 1 dioxido 4 thiomorpholinyl) methyl] phenyl] 1, 2, 4 triazolo [1, 5 a] pyridin 2 yl] cyclopropanecarboxamide' OR 'n [5 [4 [(1, 1 dioxido 4 thiomorpholinyl) methyl] phenyl] 1, 2, 4 triazolo [1, 5 a] pyridin 2 yl] cyclopropanecarboxamide 2 butenedioate' OR 'n [5 [4 [(1, 1 dioxothiomorpholin 4 yl) methyl] phenyl] 1, 2, 4 triazolo [1, 5 a] pyridin 2 yl] cyclopropanecarboxamide' OR 'n [5 [4 [(1, 1 dioxothiomorpholin 4 yl) methyl] phenyl] 1, 2, 4 triazolo [1, 5 a] pyridin 2 yl] cyclopropanecarboxamide but 2 enedioate' OR 'n [5 [4 [(1, 1 dioxothiomorpholin 4 yl) methyl] phenyl] [1, 2, 4] triazolo [1, 5 a] pyridin 2 yl] cyclopropanecarboxamide' OR 'n [5 [4 [(1, 1 dioxothiomorpholin 4 yl) methyl] phenyl] [1, 2, 4] triazolo [1, 5 a] pyridin 2 yl] cyclopropanecarboxamide but 2 enedioate'",506,27 Nov 2020

#154,"'tofacitinib'/de OR '1 cyanoacetyl 4 methyl n methyl n (1h pyrrolo [2, 3 d] pyrimidin 4 yl) 3 piperidinamine' OR '3 [4 methyl 3 [methyl (7h pyrrolo [2, 3 d] pyrimidin 4 yl) amino] 1 piperidinyl] 3 oxopropanenitrile' OR '4 [n [1 (2 cyano 1 oxoethyl) 4 methyl 3 piperidinyl] n methylamino] pyrrolo [2, 3 d] pyrimidine' OR '4 methyl 3 [methyl (7h pyrrolo [2, 3 d] pyrimidin 4 yl) amino] beta oxo 1

piperidinepropanenitrile' OR 'cp 690 550' OR 'cp 690, 550' OR 'cp 690550' OR 'cp 690550 10' OR 'cp 690550-10' OR 'cp690 550' OR 'cp690, 550' OR 'cp690550' OR 'cp690550 10' OR 'cp690550-10' OR 'tasocitinib' OR 'tasocitinib citrate' OR 'tofacitinib' OR 'tofacitinib citrate' OR 'xeljanz' OR 'xeljanz xr'",5261,27 Nov 2020

#153,"'baricitinib'/de OR '1 (ethylsulfonyl) 3 [4 (7h pyrrolo [2, 3 d] pyrimidin 4 yl) 1h pyrazol 1 yl] 3 azetidineacetonitrile' OR '[1 (ethanesulfonyl) 3 [4 (7h pyrrolo [2, 3 d] pyrimidin 4 yl) 1h pyrazol 1 yl] azetidin 3 yl] ethanenitrile' OR '[1 (ethylsulfonyl) 3 [4 (1h pyrrolo [2, 3 d] pyrimidin 4 yl) 1h pyrazol 1 yl] 3 azetidinyl] acetonitrile' OR '[1 (ethylsulfonyl) 3 [4 (7h pyrrolo [2, 3 d] pyrimidin 4 yl) 1h pyrazol 1 yl] azetidin 3 yl] ethanenitrile' OR 'baricitinib' OR 'incb 028050' OR 'incb 28050' OR 'incb028050' OR 'incb28050' OR 'ly 3009104' OR 'ly3009104' OR 'olumiant'",1543,27 Nov 2020

#152,"(janus NEAR/4 kinase NEAR/10 inhibitor$):tt",4,27 Nov 2020

#151,"(janus NEAR/4 kinase NEAR/10 inhibitor$):ti,ab,kw,tn",3319,27 Nov 2020

#150,"((jak OR jak1 OR jak2 OR jak3 OR 'janus kinase') NEAR/10 inhibitor*):tt",22,27 Nov 2020

#149,"((jak OR jak1 OR jak2 OR jak3 OR 'janus kinase') NEAR/10 inhibitor*):ti,ab,kw,tn",11251,27 Nov 2020

#148,"'janus kinase inhibitor'/exp OR 'jak inhibitor' OR 'janus kinase inhibitor' OR 'janus kinase inhibitors' OR 'janus tyrosine kinase inhibitor'",16188,27 Nov 2020

#147,"(steroid$ NEAR/10 (therap* OR treat* OR drug$ OR inject* OR intramuscular* OR 'intra muscular*' OR im OR intravenous* OR 'intra venous*' OR iv OR intraarticular* OR 'intra articular*' OR ia OR administrat* OR oral OR orally OR parenteral*)):tt",148,27 Nov 2020

#146,"(steroid$ NEAR/10 (therap* OR treat* OR drug$ OR inject* OR intramuscular* OR 'intra muscular*' OR im OR intravenous* OR 'intra venous*' OR iv OR intraarticular* OR 'intra articular*' OR ia OR administrat* OR oral OR orally OR parenteral*)):ti,ab,kw",118206,27 Nov 2020

#145,"'triamcinolone'/exp OR '9alpha fluoro 1, 4 pregnadiene 3, 20 dione 11beta, 16alpha, 17alpha, 21 tetrol' OR '9alpha fluoro 11beta, 16alpha, 17alpha, 21 tetrahydroxypregna 1, 4 diene 3, 20 dione' OR '9alpha fluoro 16alpha hydroxyhydrocortisone' OR '9alpha fluoro 16alpha hydroxyprednisolone' OR 'acetocot' OR 'adcortyl' OR 'aristocort' OR 'aristocort forte' OR 'aristodan' OR 'azmacor' OR 'celeste' OR 'cl 19823' OR 'cl19823' OR 'clinacort' OR 'clinalog' OR 'delphicort' OR 'fluoxiprednisolone' OR 'fluoxyprednisolone' OR 'ken-jec 40' OR 'kenacort' OR 'kenacort retard' OR 'korticoid' OR 'ledercort' OR 'omcilon' OR 'polcortolon' OR 'rp 8357' OR 'rp8357' OR 'simacort' OR 'sterocort' OR 'tac 3' OR 'tramcinolone' OR 'triacortyl' OR 'triam-a' OR 'triam-forte' OR 'triamcinolon' OR 'triamcinolona' OR 'triamcinolone*' OR 'triamcort' OR 'triamcot' OR 'triamonide 40' OR 'triamsicort' OR 'triancinolon' OR 'u-tri-lone' OR 'volon'",39582,27 Nov 2020

#144,"'beclometasone'/exp OR '9 chloro 11beta, 17, 21 trihydroxy 16beta methylpregna 1, 4 diene 3, 20 dione' OR '9alpha chloro 16beta methylprednisolone' OR 'beclometasone' OR 'beclomethasone' OR 'prednisolone, 9alpha chloro 16beta methyl' OR 'pregna 1, 4 diene 11beta, 17, 21 triol 3, 20 dione, 9 chloro 16beta methyl'",15095,27 Nov 2020

#143,"'dexamethasone'/de OR '16alpha methyl 9alpha fluoroprednisolone' OR '9 alpha fluoro 16 alpha methyl delta corticosterone' OR '9alpha fluoro 11beta, 17alpha, 21 trihydroxy 16alpha methyl 1, 4 pregnadiene 3, 20 dione' OR '9alpha fluoro 11beta, 17alpha, 21 trihydroxy 16alpha methylpregna 1, 4 diene 3, 20 dione' OR '9alpha fluoro 16alpha methyl delta corticosterone' OR 'adrecort' OR 'adrenocot' OR 'aeroseb dex' OR 'aeroseb-dex' OR 'aflucoson' OR 'aflucosone' OR 'alfalyl' OR 'anaflogistico' OR

'anaflogistico novobios' OR 'arcodexan' OR 'arcodexane' OR 'artrosone' OR 'azium' OR 'bidexol' OR 'calonat' OR 'cebedex' OR 'cetadexon' OR 'colofoam' OR 'corsona' OR 'cortastat' OR 'cortastat 10' OR 'cortastat la' OR 'cortidex' OR 'cortidexason' OR 'cortidrona' OR 'cortidrone' OR 'cortisumman' OR 'dacortina fuerte' OR 'dacortine fuerte' OR 'dalalone' OR 'dalalone d.p.' OR 'dalalone l.a.' OR 'danasone' OR 'de-sone la' OR 'decacortin' OR 'decadeltosona' OR 'decadeltosone' OR 'decaderm' OR 'decadion' OR 'decadran' OR 'decadron' OR 'decadron 5-12 pak' OR 'decadron la' OR 'decadronal' OR 'decadrone' OR 'decaesadril' OR 'decaject' OR 'decamethasone' OR 'decasone' OR 'decaspray' OR 'decasterolone' OR 'decdan' OR 'decilone' OR 'decilone forte' OR 'decofluor' OR 'dectancyl' OR 'dekacort' OR 'delladec' OR 'deltafluoren' OR 'deltafluorene' OR 'dergramin' OR 'deronil' OR 'desacort' OR 'desacortone' OR 'desadrene' OR 'desalark' OR 'desameton' OR 'desametone' OR 'desigdron' OR 'dexa cortisyl' OR 'dexa dabrosan' OR 'dexa korti' OR 'dexa scherosan' OR 'dexa scherozon' OR 'dexa scherozone' OR 'dexa-p' OR 'dexacen 4' OR 'dexacen-4' OR 'dexachel' OR 'dexacort' OR 'dexacortal' OR 'dexacorten' OR 'dexacortin' OR 'dexacortisyl' OR 'dexadabroson' OR 'dexadecadrol' OR 'dexadrol' OR 'dexagel' OR 'dexagen' OR 'dexahelvacort' OR 'dexakorti' OR 'dexalien' OR 'dexalocal' OR 'dexame' OR 'dexamecortin' OR 'dexameson' OR 'dexamesone' OR 'dexametason' OR 'dexametasone' OR 'dexameth' OR 'dexamethason' OR 'dexamethasone' OR 'dexamethasone alcohol' OR 'dexamethasone intensol' OR 'dexamethazon' OR 'dexamethazone' OR 'dexamethonium' OR 'dexamonozon' OR 'dexan' OR 'dexane' OR 'dexano' OR 'dexapot' OR 'dexascheroson' OR 'dexascherozon' OR 'dexascherozone' OR 'dexason' OR 'dexasone' OR 'dexasone la' OR 'dexasone s' OR 'dexinoral' OR 'dexionil' OR 'dexmethsone' OR 'dexona' OR 'dexone' OR 'dexone 0.5' OR 'dexone 0.75' OR 'dexone 1.5' OR 'dexone 4' OR 'dexpak taperpak' OR 'dextelan' OR 'dextenza' OR 'dextrasone' OR 'dexycu' OR 'dezone' OR 'dibasona' OR 'doxamethasone' OR 'esacortene' OR 'ex s1' OR 'exadion' OR 'exadione' OR 'firmalone' OR 'fluormethyl

prednisolone' OR 'fluormethylprednisolon' OR 'fluormethylprednisolone' OR 'fluormone' OR 'fluorocort' OR 'fluorodelta' OR 'fluoromethylprednisolone' OR 'fortecortin' OR 'gammacorten' OR 'gammacortene' OR 'grosodexon' OR 'grosodexone' OR 'hemady' OR 'hexadecadiol' OR 'hexadecadrol' OR 'hexadiol' OR 'hexadrol' OR 'isnacort' OR 'isopto dex' OR 'isopto maxidex' OR 'isopto-dex' OR 'isopto-maxidex' OR 'isoptodex' OR 'isoptomaxidex' OR 'lokalison f' OR 'loverine' OR 'luxazone' OR 'marvidione' OR 'maxidex' OR 'mediamethasone' OR 'megacortin' OR 'mephameson' OR 'mephamesone' OR 'metasolon' OR 'metasolone' OR 'methazon ion' OR 'methazone ion' OR 'methazonion' OR 'methazonione' OR 'metisone lafi' OR 'mexasone' OR 'millicorten' OR 'millicortenol' OR 'mk 125' OR 'mk125' OR 'mymethasone' OR 'neoforderx' OR 'neofordex' OR 'nisomethasona' OR 'novocort' OR 'nsc 34521' OR 'nsc34521' OR 'oftan-dexa' OR 'opticorten' OR 'opticortinol' OR 'oradexan' OR 'oradexon' OR 'oradexone' OR 'orgadrone' OR 'ozurdex' OR 'pidexon' OR 'policort' OR 'posurdex' OR 'predni f tablinen' OR 'predni-f' OR 'prednisolone f' OR 'prodexona' OR 'prodexone' OR 'sanamethasone' OR 'santenson' OR 'santeson' OR 'sawasone' OR 'solurex' OR 'solurex la' OR 'spoloven' OR 'sterasone' OR 'thilodexine' OR 'triamcimetil' OR 'vexamet' OR 'visumetazone' OR 'visumethazone'",171141,27 Nov 2020

#142,"'hydrocortisone'/de OR '11beta, 17, 21 trihydroxypregn 4 ene 3, 20 dione' OR '11beta, 17alpha, 21 trihydroxypregn 4 ene 3, 20 dione' OR '17 hydroxycorticosterone' OR '4 pregnene 11beta, 17alpha, 21 triol 3, 20 dione' OR '4 pregnene 3, 20 dione n beta, 17alpha, 21 triol' OR 'acticort' OR 'acticort 100' OR 'aeroseb hc' OR 'aeroseb-hc' OR 'ala-cort' OR 'ala-scalp' OR 'ala-scalp hp' OR 'alfacort' OR 'algicortis' OR 'alkindi' OR 'alpha derm' OR 'alphaderm' OR 'anucort-hc' OR 'anumed-hc' OR 'anutone-hc' OR 'aquanil hc' OR 'balneol-hc' OR 'barseb hc' OR 'beta-hc' OR 'biacort' OR 'cetacort' OR 'cobadex' OR 'colocort' OR 'compound f' OR 'cordicare lotion' OR 'coripen' OR 'cort dome' OR 'cort-dome' OR 'cort-dome high potency' OR 'cortef' OR 'cortef cream' OR 'cortenema' OR 'cortibel' OR 'corticorenol' OR

'cortifan' OR 'cortiphate' OR 'cortisol' OR 'cortisole' OR 'cortispray' OR 'cortoderm' OR 'cortril' OR 'cotacort' OR 'covocort' OR 'cremicort-h' OR 'cutaderm' OR 'derm-aid cream' OR 'dermacrin hc lotion' OR 'dermaid' OR 'dermaid soft cream' OR 'dermocare' OR 'dermocortal' OR 'dermolate' OR 'dioderm' OR 'eczacort' OR 'ef cortelan' OR 'efcortelan' OR 'egocort' OR 'egocort cream' OR 'eksalb' OR 'eldecort' OR 'emo-cort' OR 'epicort' OR 'ficortril' OR 'filocot' OR 'flexicort' OR 'gly-cort' OR 'glycort' OR 'h-cort' OR 'hc (hydrocortisone)' OR 'hc no. 1' OR 'hc no. 4' OR 'hebcort' OR 'hebcort v' OR 'hemorrhoidal hc' OR 'hemril-30' OR 'hemril-hc uniserts' OR 'hi-cor' OR 'hidrotisona' OR 'hycor' OR 'hycort' OR 'hydracort' OR 'hydrasson' OR 'hydro ricortex' OR 'hydro-rx' OR 'hydrocort' OR 'hydrocorticosteroid' OR 'hydrocortisate' OR 'hydrocortison' OR 'hydrocortisone' OR 'hydrocortisone 1% in absorbase' OR 'hydrocortisone acetonide' OR 'hydrocortisone astier' OR 'hydrocortisone in absorbase' OR 'hydrocortisone ointment' OR 'hydrocortisone plus saline' OR 'hydrocortisone steroid' OR 'hydrocortisone, topical' OR 'hydrocortisonum' OR 'hydrocortisyl' OR 'hydrocortone' OR 'hydrogalen' OR 'hydrokort' OR 'hydrokortison' OR 'hydrotopic' OR 'hysone' OR 'hytisone' OR 'hytone' OR 'hytone lotion' OR 'incortin h' OR 'instacort 10' OR 'kyypakkaus' OR 'lacticare hc' OR 'lacticare-hc' OR 'lemnis fatty cream hc' OR 'lenirit' OR 'medihaler cort' OR 'medihaler duo' OR 'medrocil' OR 'mildison' OR 'mildison fet krem' OR 'mildison lipocream' OR 'mildison-fatty' OR 'mitocortyl demangeaisons' OR 'munitren' OR 'nogenic hc' OR 'novohydrocort' OR 'nsc 10483' OR 'nsc 741' OR 'nsc10483' OR 'nutracort' OR 'optef' OR 'otosone f' OR 'penecort' OR 'plenadren' OR 'prepcort' OR 'prevex hc' OR 'pro cort' OR 'procort' OR 'procto-kit 1%' OR 'procto-kit 2.5%' OR 'proctocort' OR 'proctosert hc' OR 'proctosol-hc' OR 'proctosone' OR 'proctozone hc' OR 'procutan' OR 'rectasol-hc' OR 'rectocort' OR 'rederm' OR 'sanatison' OR 'scalp-aid' OR 'schericur' OR 'schericur 0.25%' OR 'scherosone f' OR 'sistral

hydrocort' OR 'skincalm' OR 'stie-cort' OR 'substance m' OR 'synacort' OR 'texacort' OR 'triburon-hc' OR 'unicort' OR 'vasocort'",165843,27 Nov 2020

#141,"'corticosterone'/de OR '11 beta, 21 dihydroxy pregn 4 ene 3, 20 dione' OR '11beta, 21 dihydroxy 4 pregnene 3, 20 dione' OR '11beta, 21 dihydroxypregn 4 ene 3, 20 dione' OR '4 pregnene 11beta, 21 diol 3, 20 dione' OR 'compound b' OR 'corticosterone*' OR 'corticosterone function' OR 'corticosterone response' OR 'kendall compound b' OR 'nsc 9705' OR 'nsc9705' OR 'pregn 4 ene 11beta, 21 diol 3, 20 dione' OR 'reichstein substance h'",45705,27 Nov 2020

#140,"'glucocorticoid'/exp OR 'glucocorticoid*' OR 'glucocorticoid drug' OR 'glucocorticoid hormone' OR 'glucocorticoid steroid' OR 'glucocorticoids' OR 'glucocorticoids, synthetic' OR 'glucocorticoids, topical' OR 'glucocorticoidsteroid' OR 'glucocorticosteroid' OR 'glucocortoid' OR 'glycocorticoid' OR 'glycocorticosteroid'",797162,27 Nov 2020

#139,"'methylprednisolone'/de OR '11beta, 17alpha, 21 trihydroxy 6alpha methyl 1, 4 pregnadiene 3, 20 dione' OR '2 methylprednisolone' OR '5 methylprednisolone' OR '6 alpha methylprednisolone' OR '6 methyl delta 1 hydrocortisone' OR '6 methyl prednisolone' OR '6 methylprednisolone' OR '6alpha methyl delta1 hydrocortisone' OR '6alpha methylprednisolone' OR 'adlone-40' OR 'adlone-80' OR 'beta methylprednisolone' OR 'dep medalone 80' OR 'depmedalone' OR 'depoject-80' OR 'depopred' OR 'esametone' OR 'firmacort' OR 'med-jec-40' OR 'medixon' OR 'mednin' OR 'medralone 80' OR 'medrate' OR 'medrol' OR 'medrol a' OR 'medrol adt pak' OR 'medrol compositum' OR 'medrol dosepak' OR 'medrol medules' OR 'medrol pak' OR 'medrone' OR 'meprednisolone' OR 'meprelon' OR 'mesopren' OR 'methacort 40' OR 'methacort 80' OR 'methyl prednisolone' OR 'methylcotol' OR 'methylcotolone' OR 'methylpred dp' OR 'methylprednisolone*' OR 'methylsterolone' OR 'metidrol' OR 'metrisone' OR

'metycortin' OR 'metypred' OR 'metypresol' OR 'neomedrone' OR 'nsc 19987' OR 'nsc19987' OR 'prednol' OR 'solomet' OR 'solu decortin' OR 'urbason' OR '83 43 2':rn",109222,27 Nov 2020

#138,"'cortisone'/de OR '11 dehydro 17 hydroxy corticosterone' OR '11 dehydro 17 hydroxycorticosterone' OR '17 hydroxy 11 dehydrocortisone' OR '17alpha, 21 dihydroxy 4 pregnene 3, 11, 20 trione' OR '4 pregnene 17alpha, 21 diol 3, 11, 20 trione' OR 'adrenalex' OR 'compound e (kendall)' OR 'corlin' OR 'cortadren' OR 'cortagen' OR 'cortandren' OR 'cortane' OR 'cortisal' OR 'cortisate' OR 'cortison' OR 'cortisone$' OR 'cortisone sodium' OR 'cortistal' OR 'cortivite' OR 'cortogen' OR 'cortone' OR 'delta4 pregnene 17alpha, 21 diol 3, 11, 20 trione' OR 'incorlin' OR 'incortin' OR 'kendall compound e' OR 'nsc 9703' OR 'nsc9703' OR 'pregn 4 en 17 alpha, 21 diol 3, 11, 20 trione' OR 'pregn 4 ene 17alpha, 21 diol 3, 11, 20 trione' OR 'reichstein substance fa' OR 'ricortex' OR 'scheroson' OR 'scherosone' OR 'wintersteiner compound f'",32707,27 Nov 2020

#137,"'cortisone therapeutic use'/de",3404,27 Nov 2020

#136,"'prednisone'/de OR '1, 2 dehydrocortisone' OR '17, 21 dihydroxypregna 1, 4 diene 3, 11, 20 trione' OR 'ancortone' OR 'apo-prednisone' OR 'biocortone' OR 'colisone' OR 'cortan' OR 'cortidelt' OR 'cortiprex' OR 'cutason' OR 'dacorten' OR 'de cortisyl' OR 'decortancyl' OR 'decortin' OR 'decortin e merck' OR 'decortine' OR 'decortisyl' OR 'dehydrocortisone' OR 'dekortin' OR 'delitisone' OR 'dellacort a' OR 'delta 1 dehydrocortisone' OR 'delta cortelan' OR 'delta cortisone' OR 'delta dome' OR 'delta e' OR 'delta prenovis' OR 'delta-dome' OR 'deltacorten' OR 'deltacortene' OR 'deltacortisone' OR 'deltacortone' OR 'deltasone' OR 'deltison' OR 'deltisona' OR 'deltra' OR 'di adreson' OR 'di-adreson' OR 'diadreson' OR 'drazone' OR 'encorton' OR 'encortone' OR 'enkorton' OR 'fernisone' OR 'hostacortin' OR 'insone' OR 'liquid pred' OR 'lodotra' OR 'me-korti' OR 'meprison' OR 'metacortandracin' OR 'meticorten' OR 'meticortine' OR 'nisona' OR 'nsc 10023' OR 'nsc10023' OR 'orasone' OR 'orisane' OR

'panafcort' OR 'paracort' OR 'pehacort' OR 'precort' OR 'precortal' OR 'prednicen-m' OR 'prednicorm' OR 'prednicot' OR 'prednidib' OR 'prednison' OR 'prednisone*' OR 'prednisone alcohol' OR 'prednisone intensol' OR 'prednisone test' OR 'prednitone' OR 'pregna 1, 4 diene 3, 11, 20 trione 17, 21 diol' OR 'pronison' OR 'pronisone' OR 'pronizone' OR 'pulmison' OR 'rayos' OR 'rectodelt' OR 'servisone' OR 'steerometz' OR 'sterapred' OR 'sterapred ds' OR 'ultracorten' OR 'urtilone' OR 'winpred'",187259,27 Nov 2020

#135,"'prednisolone'/de OR '1, 2 dehydro hydrocortison' OR '1, 4 pregnadien 11beta, 17alpha, 21 triol 3, 20 dion' OR '1, 4 pregnadiene 11beta, 17alpha, 21 triol 3, 20 dione' OR '1, 4 pregnadiene 3, 20 dione 11beta, 17alpha, 21 triol' OR '11beta, 17, 21 trihydroxypregna 1, 4 diene 3, 20 dione' OR '11beta, 17alpha, 21 trihydroxy 1, 4 pregnadien 3, 20 dion' OR '11beta, 17alpha, 21 trihydroxypregna 1, 4 diene 3, 20 dione' OR '3, 20 dioxo 11beta, 17alpha, 21 trihydroxy 1, 4 pregnadiene' OR 'adelcort' OR 'antisolon' OR 'antisolone' OR 'aprednislon' OR 'aprednislone' OR 'benisolon' OR 'benisolone' OR 'berisolon' OR 'berisolone' OR 'caberdelta' OR 'capsoid' OR 'co hydeltra' OR 'codelcortone' OR 'compresolon' OR 'cortadeltona' OR 'cortadeltone' OR 'cortalone' OR 'cortelinter' OR 'cortisolone' OR 'cotolone' OR 'dacortin' OR 'dacortin h' OR 'dacrotin' OR 'decaprednil' OR 'decortin h' OR 'decortril' OR 'dehydro cortex' OR 'dehydro hydrocortison' OR 'dehydro hydrocortisone' OR 'dehydrocortex' OR 'dehydrocortisol' OR 'dehydrocortisole' OR 'dehydrohydrocortison' OR 'dehydrohydrocortisone' OR 'delcortol' OR 'delta 1 17 hydroxycorticosterone 21 acetate' OR 'delta 1 hydrocortisone' OR 'delta cortef' OR 'delta cortril' OR 'delta ef cortelan' OR 'delta f' OR 'delta hycortol' OR 'delta hydrocortison' OR 'delta hydrocortisone' OR 'delta ophticor' OR 'delta stab' OR 'delta-cortef' OR 'delta1 dehydrocortisol' OR 'delta1 dehydrohydrocortisone' OR 'delta1 hydrocortisone' OR 'deltacortef' OR 'deltacortenolo' OR 'deltacortil' OR 'deltacortoil' OR 'deltacortril' OR 'deltaderm' OR 'deltaglycortril' OR

'deltahycortol' OR 'deltahydrocortison' OR 'deltahydrocortisone' OR 'deltaophticor' OR 'deltasolone' OR 'deltastab' OR 'deltidrosol' OR 'deltisilone' OR 'deltisolon' OR 'deltisolone' OR 'deltolasson' OR 'deltolassone' OR 'deltosona' OR 'deltosone' OR 'depo-predate' OR 'dermosolon' OR 'dhasolone' OR 'di adreson f' OR 'di adresone f' OR 'di-adreson-f' OR 'diadreson f' OR 'diadresone f' OR 'dicortol' OR 'domucortone' OR 'encortelon' OR 'encortelone' OR 'encortolon' OR 'equisolon' OR 'fernisolone-p' OR 'glistelone' OR 'hefasolon' OR 'hostacortin h' OR 'hostacortin h vet' OR 'hydeltra' OR 'hydeltrone' OR 'hydrelta' OR 'hydrocortancyl' OR 'hydrocortidelt' OR 'hydrodeltalone' OR 'hydrodeltisone' OR 'hydroretrocortin' OR 'hydroretrocortine' OR 'inflanefran' OR 'insolone' OR 'keteocort h' OR 'key-pred' OR 'key-pred sp' OR 'lenisolone' OR 'leocortol' OR 'liquipred' OR 'lygal kopftinktur n' OR 'mediasolone' OR 'meprisolon' OR 'meprisolone' OR 'metacortalon' OR 'metacortalone' OR 'metacortandralon' OR 'metacortandralone' OR 'metacortelone' OR 'meti derm' OR 'meti-derm' OR 'meticortelone' OR 'metiderm' OR 'morlone' OR 'mydrapred' OR 'neo delta' OR 'nisolon' OR 'nisolone' OR 'nsc 9120' OR 'nsc9120' OR 'opredsone' OR 'panafcortelone' OR 'panafcortolone' OR 'panafort' OR 'paracortol' OR 'phlogex' OR 'pre cortisyl' OR 'preconin' OR 'precortalon' OR 'precortancyl' OR 'precortisyl' OR 'pred-ject-50' OR 'predacort 50' OR 'predaject-50' OR 'predalone 50' OR 'predartrina' OR 'predartrine' OR 'predate-50' OR 'predeltilone' OR 'predisole' OR 'predisyr' OR 'predne dome' OR 'prednecort' OR 'prednedome' OR 'prednelan' OR 'predni coelin' OR 'predni h tablinen' OR 'predni-helvacort' OR 'prednicoelin' OR 'prednicort' OR 'prednicortelone' OR 'prednifor drops' OR 'predniment' OR 'predniretard' OR 'prednis' OR 'prednisil' OR 'prednisolon' OR 'prednisolona' OR 'prednisolone*' OR 'prednisolone alcohol' OR 'prednisolone h' OR 'prednisolone oleosae sr 82' OR 'prednisolone, topical' OR 'prednivet' OR 'prednorsolon' OR 'prednorsolone' OR 'predonine' OR 'predorgasolona' OR 'predorgasolone' OR 'pregna 1, 4 diene 11beta, 17alpha, 21 triol 3, 20 dione' OR 'prelon' OR 'prelone'

OR 'prenilone' OR 'prenin' OR 'prenolone' OR 'preventan' OR 'prezolon' OR 'rubycort' OR 'scherisolon' OR 'scherisolona' OR 'serilone' OR 'solondo' OR 'solone' OR 'solupren' OR 'soluprene' OR 'spiricort' OR 'spolotane' OR 'sterane' OR 'sterolone' OR 'supercortisol' OR 'supercortizol' OR 'taracortelone' OR 'walesolone' OR 'wysolone'",147404,27 Nov 2020

#134,"'corticosteroid'/exp OR 'adrenal cortex hormone' OR 'adrenal cortex hormones' OR 'adrenal cortical hormone' OR 'adrenal cortical hormones' OR 'adrenal cortical steroid' OR 'adrenal steroid' OR 'adrenal steroid hormone' OR 'adreno cortical steroid' OR 'adreno corticosteroid' OR 'adrenocortical hormone' OR 'adrenocortical steroid' OR 'adrenocorticosteroid' OR 'cortical steroid' OR 'cortico steroid' OR 'corticoid*' OR 'corticosteroid*' OR 'corticosteroid agent' OR 'corticosteroid calcium' OR 'corticosteroid hormone' OR 'corticosteroids' OR 'corticosteroids, inhalation' OR 'corticosteroids, ophthalmic' OR 'corticosteroids, otic' OR 'corticosteroids, systemic' OR 'corticosteroids, topical' OR 'dermocorticosteroid' OR 'fluorinated corticosteroid'",1039987,27 Nov 2020

#133,"chloroquin*:ti,ab,kw,rn,tn OR cq:ti,ab,kw,rn,tn OR '54-04-7':ti,ab,kw,rn,tn OR 886u3h6uff*:ti,ab,kw,rn,tn OR arequin:ti,ab,kw,rn,tn OR chingamin:ti,ab,kw,rn,tn OR chlorochin*:ti,ab,kw,rn,tn OR khingamin:ti,ab,kw,rn,tn OR oe48649kh6n*:ti,ab,kw,rn,tn OR anaclor:ti,ab,kw,rn,tn OR bemaco:ti,ab,kw,rn,tn OR benaquin:ti,ab,kw,rn,tn OR 'brn 0482809':ti,ab,kw,rn,tn OR brn0482809:ti,ab,kw,rn,tn OR capquin:ti,ab,kw,rn,tn OR 'ccris 3439':ti,ab,kw,rn,tn OR ccris3439:ti,ab,kw,rn,tn OR chloraquin*:ti,ab,kw,rn,tn OR cindacin:ti,ab,kw,rn,tn OR cindachin:ti,ab,kw,rn,tn OR cloroquina:ti,ab,kw,rn,tn OR cocatrit:ti,ab,kw,rn,tn OR 'einecs 200-191-2':ti,ab,kw,rn,tn OR 'einecs200 191 2':ti,ab,kw,rn,tn OR elestol:ti,ab,kw,rn,tn OR 'hsdb 3029':ti,ab,kw,rn,tn OR hsdb3029:ti,ab,kw,rn,tn OR lapaquin:ti,ab,kw,rn,tn OR malaren:ti,ab,kw,rn,tn OR neochin:ti,ab,kw,rn,tn OR 'nsc

187208':ti,ab,kw,rn,tn OR nsc187208:ti,ab,kw,rn,tn OR pfizerquine:ti,ab,kw,rn,tn OR quensyl:ti,ab,kw,rn,tn OR quinercyl:ti,ab,kw,rn,tn OR quinilon:ti,ab,kw,rn,tn OR quinoline:ti,ab,kw,rn,tn OR quinoscan:ti,ab,kw,rn,tn OR ronaquine:ti,ab,kw,rn,tn OR sopaquin:ti,ab,kw,rn,tn OR 'st 21':ti,ab,kw,rn,tn OR weimerquin:ti,ab,kw,rn,tn",36707,27 Nov 2020

#132,"'chloroquine'/de OR '4 (4 diethylamino 1 methylbutylamino) 7 chlorchinolin diphosphate' OR '4 (4 diethylamino 1 methylbutylamino) 7 chlorchinolin sulfate' OR '4 (4 diethylamino 1 methylbutylamino) 7 chlorchinolin sulphate' OR '4 (4 diethylamino 1 methylbutylamino) 7 chloroquinoline' OR '7 chloro 4 (4 diethylamino 1 methylbutylamino) quinoline' OR '7 chloro 4 (4 diethylamino 1 methylbutylamino) quinoline diphosphate' OR 'a-cq' OR 'amokin' OR 'amokine' OR 'anoclor' OR 'aralan' OR 'aralen' OR 'aralen hydrochloride' OR 'aralen phosphate' OR 'aralene' OR 'arechin' OR 'arechine' OR 'arequine' OR 'arthrochin' OR 'arthrochine' OR 'arthroquine' OR 'artrichin' OR 'artrichine' OR 'artriquine' OR 'avloclor' OR 'avoclor' OR 'bemaphata' OR 'bemaphate' OR 'bemasulph' OR 'bipiquin' OR 'cadiquin' OR 'chemochin' OR 'chemochine' OR 'chingamine' OR 'chingaminum' OR 'chloraquine' OR 'chlorochin' OR 'chlorochine' OR 'chlorofoz' OR 'chloroquin' OR 'chloroquin phosphate' OR 'chloroquine' OR 'chloroquine diphosphate' OR 'chloroquine disulfate' OR 'chloroquine disulphate' OR 'chloroquine hydrochloride' OR 'chloroquine phosphate' OR 'chloroquine streuli' OR 'chloroquine sulfate' OR 'chloroquine sulphate' OR 'chloroquinesulphate' OR 'chloroquini diphosphas' OR 'chloroquinum diphosphoricum' OR 'chlorquin' OR 'chlorquine' OR 'choloquine' OR 'choroquine sulfate' OR 'choroquine sulphate' OR 'cidanchin' OR 'clo-kit junior' OR 'clorichina' OR 'clorichine' OR 'cloriquine' OR 'clorochina' OR 'delagil' OR 'delagyl' OR 'dichinalex' OR 'diclokin' OR 'diquinalex' OR 'diroquine' OR 'emquin' OR 'genocin' OR 'gontochin' OR 'gontochine' OR 'gontoquine' OR 'heliopar' OR 'imagon' OR 'iroquine' OR 'klorokin' OR 'klorokine' OR 'klorokinfosfat' OR 'lagaquin' OR 'malaquin' OR 'malarex' OR

'malarivon' OR 'malaviron' OR 'maliaquine' OR 'maquine' OR 'mesylith' OR 'mexaquin' OR 'mirquin' OR 'nivachine' OR 'nivaquin' OR 'nivaquine' OR 'nivaquine (b)' OR 'nivaquine b' OR 'nivaquine dp' OR 'nivaquine forte' OR 'p roquine' OR 'quinachlor' OR 'quingamine' OR 'repal' OR 'resochen' OR 'resochene' OR 'resochin' OR 'resochin junior' OR 'resochina' OR 'resochine' OR 'resochinon' OR 'resoquina' OR 'resoquine' OR 'reumachlor' OR 'roquine' OR 'rp 3377' OR 'rp3377' OR 'sanoquin' OR 'sanoquine' OR 'silbesan' OR 'siragan' OR 'sirajan' OR 'sn 7618' OR 'sn7618' OR 'solprina' OR 'solprine' OR 'tresochin' OR 'tresochine' OR 'tresoquine' OR 'trochin' OR 'trochine' OR 'troquine' OR 'w 7618' OR 'w7618' OR 'win 244' OR 'win244'",44555,27 Nov 2020

#131,"'organogold compound'/exp OR 'organogold compound' OR 'organogold compounds' OR 'organogold derivative'",482,27 Nov 2020

#130,"aurothiopropanol*:ti,ab,kw,rn,tn OR allochrysine:ti,ab,kw,rn,tn OR allocrisine:ti,ab,kw,rn,tn OR aurotiopoe:ti,ab,kw,rn,tn OR aurotioprol:ti,ab,kw,rn,tn OR '27279-43-2':ti,ab,kw,rn,tn OR g7097j63e9*:ti,ab,kw,rn,tn OR 'sodium aurothiopropanolsulfonate':ti,ab,kw,rn,tn OR 'gold sodium thiopropanol sulfonate':ti,ab,kw,rn,tn OR 'sodium auromercaptopropanol sulfonate':ti,ab,kw,rn,tn",274,27 Nov 2020

#129,"'allochrysine'/de OR '1 auromercapto 2 propanol 3 sulfonate' OR '1 auromercapto 2 propanol 3 sulfonate sodium' OR '1 auromercepto 2 propanol 3 sulfonate' OR 'allochrysine' OR 'auromercapto propanol sulfonate sodium' OR 'auromercaptopropanolsulfonate sodium' OR 'aurothioisopropanolsulfonate sodium' OR 'aurothiopropanol sulfonate' OR 'aurothiopropanol sulfonate sodium' OR 'gold sodium thiopropanol sulfonate' OR 'sodium 1 auromercapto 2 propanol 3 sulfonate' OR 'sodium auromercapto 2 propanol 3 sulfonate' OR 'sodium auromercaptopropanol sulfonate' OR 'sodium aurothiopropanol sulfonate'",272,27 Nov 2020

#128,"((gold* OR auro*) NEAR/10 (inject* OR intramuscular* OR 'im' OR 'intra muscular*')):ti,ab,kw",3123,27 Nov 2020

#127,"(thioglucosoaurate*:ti,ab,kw,rn,tn OR 'gold thioglucose*':ti,ab,kw,rn,tn OR 'gold thio glucose*':ti,ab,kw,rn,tn OR 'thioglucose gold':ti,ab,kw,rn,tn OR aureotan:ti,ab,kw,rn,tn OR 'gold-50':ti,ab,kw,rn,tn OR gold50:ti,ab,kw,rn,tn OR 'b oleosum solganal':ti,ab,kw,rn,tn OR aurotan:ti,ab,kw,rn,tn OR authron:ti,ab,kw,rn,tn OR brenol:ti,ab,kw,rn,tn OR 'ccris 59':ti,ab,kw,rn,tn OR ccris59:ti,ab,kw,rn,tn OR 'einecs 235-365-7':ti,ab,kw,rn,tn OR 'einecs235 365 7':ti,ab,kw,rn,tn OR glysanol:ti,ab,kw,rn,tn) AND b:ti,ab,kw,rn,tn OR goldthioglucose*:ti,ab,kw,rn,tn OR goldtioglucose*:ti,ab,kw,rn,tn OR 'hsdb 7174':ti,ab,kw,rn,tn OR hsdb7174:ti,ab,kw,rn,tn OR oronol:ti,ab,kw,rn,tn OR romosol:ti,ab,kw,rn,tn OR 'skf 10056':ti,ab,kw,rn,tn OR skf10056:ti,ab,kw,rn,tn OR 2p2v9q0e78*:ti,ab,kw,rn,tn OR '12192-57-3':ti,ab,kw,rn,tn",1509,27 Nov 2020

#126,"'aurothioglucose sodium'/de",40,27 Nov 2020

#125,"'aurothioglucose'/de OR '(1 d glucosylthio) gold' OR '(1 glucosylthio) gold' OR 'anitur' OR 'aureotan' OR 'auro thioglucose*' OR 'auromyose*' OR 'aurothioglucose*' OR 'aurumin' OR 'aurumine' OR 'gold thio glucose' OR 'gold thioglucose' OR 'gold-50' OR 'goldthioglucose' OR 'goldtioglucose' OR 'oronol' OR 'solganal' OR 'solganal b' OR 'solganol'",1709,27 Nov 2020

#124,"aurolate*:ti,ab,kw,rn,tn OR 'gold disodium thiomalate*':ti,ab,kw,rn,tn OR 'gold thiomalic acid*':ti,ab,kw,rn,tn OR miocrisin:ti,ab,kw,rn,tn OR 'monogold disodium thiomalate*':ti,ab,kw,rn,tn OR myochrysin*:ti,ab,kw,rn,tn OR myocrisin*:ti,ab,kw,rn,tn OR myocrysin*:ti,ab,kw,rn,tn OR 'sodium aurothiomalate*':ti,ab,kw,rn,tn OR 'sodium gold thiomalate*':ti,ab,kw,rn,tn OR 'sodium thiomalate* gold':ti,ab,kw,rn,tn OR 'sodium thiomalatoaurate*':ti,ab,kw,rn,tn OR taureodon:ti,ab,kw,rn,tn OR 'thiomalatoaurate sodium':ti,ab,kw,rn,tn OR '1244-57-4':ti,ab,kw,rn,tn OR '39377-38-3':ti,ab,kw,rn,tn

OR '4846-27-9':ti,ab,kw,rn,tn OR '74916-57-7':ti,ab,kw,rn,tn OR e4768zy6gm*:ti,ab,kw,rn,tn OR 'aurothiomala-natrium':ti,ab,kw,rn,tn OR 'aurot$iomalato sodico':ti,ab,kw,rn,tn OR 'monogold* sodium salt':ti,ab,kw,rn,tn OR 'dinatrium 2-aurothio-succinat':ti,ab,kw,rn,tn OR 'einecs 235-479-7':ti,ab,kw,rn,tn OR 'einecs235 479 7':ti,ab,kw,rn,tn OR 'hsdb 7173':ti,ab,kw,rn,tn OR hsdb7173:ti,ab,kw,rn,tn OR kidon:ti,ab,kw,rn,tn OR 'natrii aurothiomalas*':ti,ab,kw,rn,tn OR shiosol:ti,ab,kw,rn,tn OR 'gold mercatpsuccinate*':ti,ab,kw,rn,tn",885,27 Nov 2020

#123,"'aurothiomalate'/de OR '(1, 2 dicarboxyethylthio) gold disodium' OR '(1, 2 dicarboxyethylthio) gold disodium salt' OR 'aurothio' OR 'aurothiomalate*' OR 'aurothiomalate sodium' OR 'disodium aurothiomalate' OR 'gold mercaptosuccinate' OR 'gold sodium thiomalate*' OR 'gold thiomalate*' OR 'gold thiomalate sodium' OR 'miocrin' OR 'myochrisin' OR 'myochrisine' OR 'myochrysin' OR 'myochrysine' OR 'myocrisin' OR 'myocrysin' OR 'shiosol' OR 'sodium aurothiomalate' OR 'sodium gold thiomalate' OR 'tauredon' OR 'tauredone' OR 'thiomalate gold'",3454,27 Nov 2020

#122,"hydroxychloroquin*:ti,ab,kw,rn,tn OR hcq:ti,ab,kw,rn,tn OR '747-3-4':ti,ab,kw,rn,tn OR 8q2869cnvh*:ti,ab,kw,rn,tn OR '118-42-3':ti,ab,kw,rn,tn OR 4qwg6n8qkh*:ti,ab,kw,rn,tn OR hydroxychlorochin*:ti,ab,kw,rn,tn OR hydrochloroquin*:ti,ab,kw,rn,tn OR hydrocloroquin*:ti,ab,kw,rn,tn OR oxychlorochin:ti,ab,kw,rn,tn OR oxychloroquin*:ti,ab,kw,rn,tn OR plaquinol:ti,ab,kw,rn,tn OR 'brn 0253894':ti,ab,kw,rn,tn OR brn0253894:ti,ab,kw,rn,tn OR 'einecs 204-249-8':ti,ab,kw,rn,tn OR 'einecs204 249 8':ti,ab,kw,rn,tn OR hidroxicloroquin*:ti,ab,kw,rn,tn OR idrossiclorochin*:ti,ab,kw,rn,tn OR oxichlorochin*:ti,ab,kw,rn,tn OR oxichloroquin*:ti,ab,kw,rn,tn OR win1258:ti,ab,kw,rn,tn OR 'win 1258':ti,ab,kw,rn,tn OR z0188:ti,ab,kw,rn,tn OR 'einecs 212-019-3':ti,ab,kw,rn,tn OR 'einecs212-019-3':ti,ab,kw,rn,tn OR erquin:ti,ab,kw,rn,tn OR quensyl:ti,ab,kw,rn,tn OR 'sn 8137':ti,ab,kw,rn,tn OR sn8137:ti,ab,kw,rn,tn",29714,27 Nov 2020

#121,"'hydroxychloroquine sulfate'/de OR '1 (7 chloro 4 quinolylamino) 3 diethylamino 2 propanol sulfate' OR '1 (7 chloro 4 quinolylamino) 3 diethylamino 2 propanol sulphate' OR '2 [[4 [(7 chloro 4 quinolinyl) amino] pentyl] (ethyl) amino] ethanol sulfate' OR '2 [[4 [(7 chloro 4 quinolinyl) amino] pentyl] (ethyl) amino] ethanol sulphate' OR '2 [[4 [(7 chloroquinolin 4 yl) amino] pentyl] (ethyl) amino] ethanol sulfate' OR '2 [[4 [(7 chloroquinolin 4 yl) amino] pentyl] (ethyl) amino] ethanol sulphate' OR '7 chloro 4 (3 diethylamino 2 hydroxypropylamino) quinoline sulfate' OR '7 chloro 4 (3 diethylamino 2 hydroxypropylamino) quinoline sulphate' OR 'dimard' OR 'erquin' OR 'evoquin' OR 'geniquin' OR 'hydroxychloroquine sulfate' OR 'hydroxychloroquine sulphate' OR 'oxiklorin' OR 'oxychloroquine sulfate' OR 'oxychloroquine sulphate' OR 'plaquenil' OR 'plaquenil sulfate' OR 'plaquenil sulphate' OR 'plaquinol' OR 'toremonil' OR 'yuma'",3651,27 Nov 2020

#120,"'hydroxychloroquine'/de OR '7 chloro 4 [4 [ethyl (2 hydroxyethyl) amino] 1 methylbutylamino] quinoline' OR '7 chloro 4 [4 [ethyl (2 hydroxyethyl) amino] 1 methylbutylamino] quinoline diphosphate' OR 'apo-hydroxychloroquine' OR 'chloroquinol' OR 'ercoquin' OR 'hydrochloroquine' OR 'hydrocloroquine' OR 'hydroxychloroquine' OR 'oxychloroquine' OR 'quensyl' OR 'sn 8137'",29882,27 Nov 2020

#119,"sasp:ti,ab,kw,rn,tn OR ssz:ti,ab,kw,rn,tn OR salicylazosulfapyridin*:ti,ab,kw,rn,tn OR 'salicyl azo sulfapyridin*':ti,ab,kw,rn,tn OR salazosulfapyridin*:ti,ab,kw,rn,tn OR salazosulfapiridin*:ti,ab,kw,rn,tn OR azulfadin*:ti,ab,kw,rn,tn OR azulfidin*:ti,ab,kw,rn,tn OR pleon:ti,ab,kw,rn,tn OR ulcol:ti,ab,kw,rn,tn OR ucine:ti,ab,kw,rn,tn OR salazopyrin*:ti,ab,kw,rn,tn OR 'ratio-sulfosalazin*':ti,ab,kw,rn,tn OR 3xc8guz6cb*:ti,ab,kw,rn,tn OR accucol:ti,ab,kw,rn,tn OR asulfidin*:ti,ab,kw,rn,tn OR azopyrin*:ti,ab,kw,rn,tn OR azosulfidin*:ti,ab,kw,rn,tn OR 'brn 0356241':ti,ab,kw,rn,tn OR brn0356241:ti,ab,kw,rn,tn OR 'ccris 4713':ti,ab,kw,rn,tn OR ccris4713:ti,ab,kw,rn,tn OR 'einecs 209-

974-3':ti,ab,kw,rn,tn OR 'hsdb 3395':ti,ab,kw,rn,tn OR hsdb3395:ti,ab,kw,rn,tn OR 'nsc 203730':ti,ab,kw,rn,tn OR nsc203730:ti,ab,kw,rn,tn OR 'nsc 667219':ti,ab,kw,rn,tn OR nsc667219:ti,ab,kw,rn,tn OR reupirin:ti,ab,kw,rn,tn OR 'salazo-sulfapyridin*':ti,ab,kw,rn,tn OR salazosulfpyridin*:ti,ab,kw,rn,tn OR salazopiridazin*:ti,ab,kw,rn,tn OR salazopyridin*:ti,ab,kw,rn,tn OR salazopirin*:ti,ab,kw,rn,tn OR salipyr:ti,ab,kw,rn,tn OR 'si-88':ti,ab,kw,rn,tn OR si88:ti,ab,kw,rn,tn OR sulfasalazin*:ti,ab,kw,rn,tn OR sulphasalazin*:ti,ab,kw,rn,tn OR 'w-t sasp oral':ti,ab,kw,rn,tn OR '599-79-1':ti,ab,kw,rn,tn",28724,27 Nov 2020

#118,"'salazosulfapyridine'/de OR '4 (2 pyridylaminosulfonyl) 3` carboxy 4` hydroxyazobenzene' OR '4 hydroxy 4` (pyrid 2 ylsulfamoyl) azobenzene 3 carboxylic acid' OR '5 [4 (2 pyridylsulfamoyl) phenylazo] 2 hydroxybenzoic acid' OR '5 [para (2 pyridylsulfamoyl) phenylazo] salicylic acid' OR 'azlufidine en-tabs' OR 'azopyrin' OR 'azopyrine' OR 'azosulfidine' OR 'azulfide' OR 'azulfidina' OR 'azulfidine' OR 'azulfidine en tabs' OR 'azulfidine en-tabs' OR 'azulfidine ra' OR 'azulfin' OR 'benzosulfa' OR 'colo pleon' OR 'colo-pleon' OR 'colopleon' OR 'disalazin' OR 'gastropyrin' OR 'pleon ra' OR 'pyralin en' OR 'rorasul' OR 'rosulfant' OR 's.a.s.-500' OR 'salazine' OR 'salazo sulfapyridine' OR 'salazodin' OR 'salazopirina' OR 'salazopyridin' OR 'salazopyridine' OR 'salazopyrin' OR 'salazopyrin entabs' OR 'salazopyrin-en' OR 'salazopyrina' OR 'salazopyrine' OR 'salazopyrine ec' OR 'salazosulfa pyridine' OR 'salazosulfapyridine' OR 'salazosulfpyridine' OR 'salicyl azo sulfapyridine' OR 'salicylazosulfapyridin' OR 'salicylazosulfapyridine' OR 'salisulf' OR 'salopyr' OR 'saridine' OR 'sas 500' OR 'sulcolon' OR 'sulfasalazine' OR 'sulfasalizine' OR 'sulfosalazine' OR 'sulphasalazine' OR 'zopyrin'",26755,27 Nov 2020

#117,"'lef':ti,ab,kw",4109,27 Nov 2020

#116,"'leflunomide'/de OR '5 methyl 4` trifluoromethyl 4 isoxazolecarboxanilide' OR '5 methyl n [4 (trifluoromethyl) phenyl] 4 isoxazolecarboxamide' OR '5 methyl n [4 (trifluoromethyl) phenyl] isoxazole

4 carboxamide' OR '5 methyl n [para (trifluoromethyl) phenyl] 4 isoxazolecarboxamide' OR 'alpha, alpha, alpha trifluoro 5 methyl 4 isoxazolecarboxy para toluidide' OR 'arabloc' OR 'arava' OR 'hwa 486' OR 'hwa486' OR 'leflunomid*' OR 'leflunomide winthrop' OR 'n (4 trifluoromethylphenyl) 5 methylisoxazole 4 carboxamide' OR 'repso' OR 'rs 34821' OR 'rs34821' OR 'su 101' OR 'su101' OR 'hsdb 7289' OR hsdb7289 OR g162gk9u4w* OR '75706-12-6'",12984,27 Nov 2020

#115,"'mtx':ti,ab,kw,rn,tn OR methotrex*:ti,ab,kw,rn,tn OR metotrexat*:ti,ab,kw,rn,tn OR methylaminopterin*:ti,ab,kw,rn,tn OR amet$opterin*:ti,ab,kw,rn,tn OR brimexate:ti,ab,kw,rn,tn OR 'a-met$opterin*':ti,ab,kw,rn,tn OR 'a-methpterin*':ti,ab,kw,rn,tn OR 'ai3-25299':ti,ab,kw,rn,tn OR ai325299:ti,ab,kw,rn,tn OR 'alpha-methopterin':ti,ab,kw,rn,tn OR farmitrexat*:ti,ab,kw,rn,tn OR fauldexato:ti,ab,kw,rn,tn OR hdmtx:ti,ab,kw,rn,tn OR metotressat*:ti,ab,kw,rn,tn OR methotrate*:ti,ab,kw,rn,tn OR methohexate*:ti,ab,kw,rn,tn OR mexat*:ti,ab,kw,rn,tn OR metrotex:ti,ab,kw,rn,tn OR metatrexato*:ti,ab,kw,rn,tn OR metatrexan:ti,ab,kw,rn,tn OR maxtrex:ti,ab,kw,rn,tn OR meticil:ti,ab,kw,rn,tn OR metoject:ti,ab,kw,rn,tn OR metotrexin:ti,ab,kw,rn,tn OR lumexon:ti,ab,kw,rn,tn OR r$eumatrex*:ti,ab,kw,rn,tn OR tremetex:ti,ab,kw,rn,tn OR trexeron:ti,ab,kw,rn,tn OR trixilem:ti,ab,kw,rn,tn OR 'mpi-2505':ti,ab,kw,rn,tn OR mpi2505:ti,ab,kw,rn,tn OR amethopterin*:ti,ab,kw,rn,tn OR '133073-73-1':ti,ab,kw,rn,tn OR '15475-56-6':ti,ab,kw,rn,tn OR 3ig1e710zn*:ti,ab,kw,rn,tn OR yl5fz2y5u1*:ti,ab,kw,rn,tn OR 'ccris 1109':ti,ab,kw,rn,tn OR ccris1109:ti,ab,kw,rn,tn OR 'emt 25299':ti,ab,kw,rn,tn OR emt25299:ti,ab,kw,rn,tn OR 'r-9985':ti,ab,kw,rn,tn OR r9885:ti,ab,kw,rn,tn OR 'x-133':ti,ab,kw,rn,tn OR 'wr-19039':ti,ab,kw,rn,tn OR wr19039:ti,ab,kw,rn,tn",191138,27 Nov 2020

#114,"'methotrexate'/de OR '2 [[4 [[(2, 4 diamino 6 pteridinyl) methyl] (methyl) amino] benzoyl] amino] pentanedioic acid' OR '2 [[4 [[(2, 4 diaminopteridin 6 yl) methyl] (methyl) amino] benzoyl]

amino] pentanedioic acid' OR '4 amino 10 methylfolic acid' OR '4 amino 10 methylpteroylglutamic acid' OR '4 amino n10 methylpteroylglutamic acid' OR 'mtx' OR 'a methopterine' OR 'abitrexate' OR 'amethopterin' OR 'amethopterine' OR 'ametopterine' OR 'antifolan' OR 'biotrexate' OR 'canceren' OR 'cl 14377' OR 'cl14377' OR 'emtexate' OR 'emthexat' OR 'emthexate' OR 'emtrexate' OR 'enthexate' OR 'farmitrexat' OR 'farmitrexate' OR 'farmotrex' OR 'folex' OR 'folex pfs' OR 'ifamet' OR 'imeth' OR 'intradose mtx' OR 'jylamvo' OR 'lantarel' OR 'ledertrexate' OR 'maxtrex' OR 'metex' OR 'methoblastin' OR 'methohexate' OR 'methotrate' OR 'methotrexat' OR 'methotrexat ebewe' OR 'methotrexate' OR 'methotrexate lpf' OR 'methotrexate preservative free' OR 'methotrexate sodium' OR 'methotrexate sodium preservative free' OR 'methotrexato' OR 'methoxtrexate' OR 'methrotrexate' OR 'methylaminopterin' OR 'methylaminopterine' OR 'meticil' OR 'metoject' OR 'metothrexate' OR 'metothrexate sodium' OR 'metotrexat' OR 'metotrexate' OR 'metotrexin' OR 'metrex' OR 'mexate' OR 'mexate-aq' OR 'mexate-aq preserved' OR 'mpi 5004' OR 'mpi5004' OR 'n [4 [(2, 4 diamino 6 pteridylmethyl) methylamino] benzoyl] glutamic acid' OR 'neotrexate' OR 'nordimet' OR 'novatrex' OR 'nsc 740' OR 'nsc740' OR 'otrexup' OR 'otrexup pfs' OR 'rasuvo' OR 'reditrex' OR 'reumatrex' OR 'rheumatrex' OR 'rheumatrex dose pack' OR 'sodium methotrexate' OR 'texate' OR 'texate-t' OR 'texorate' OR 'trexall' OR 'xaken' OR 'xatmep' OR 'zexate'",191406,27 Nov 2020

#113,"(('disease modifying' NEAR/3 (antir$eum* OR 'anti r$eum*') NEAR/3 (drug$ OR agent$)):ti,ab,kw) OR (('disease modifying' NEAR/3 (antir$eum* OR 'anti r$eum*') NEAR/3 (drug$ OR agent$)):tt)",10989,27 Nov 2020

#112,"dmard*:ti,ab,kw OR csdmard*:ti,ab,kw OR 'cs-dmard*':ti,ab,kw OR sdmard*:ti,ab,kw OR 's-dmard*':ti,ab,kw OR tsdmard*:ti,ab,kw OR 'ts-dmard*':ti,ab,kw OR bdmard*:ti,ab,kw OR 'b-

dmard*':ti,ab,kw OR dmard*:tt OR csdmard*:tt OR 'cs-dmard*':tt OR sdmard*:tt OR 's-dmard*':tt OR tsdmard*:tt OR 'ts-dmard*':tt OR bdmard*:tt OR 'b-dmard*':tt",17435,27 Nov 2020

#111,"'disease modifying antirheumatic drug'/de OR 'disease modifying antirheumatic agent' OR 'disease modifying antirheumatic drug' OR 'disease modifying antirheumatic drugs'",20106,27 Nov 2020

#110,"'mavrilimumab'/de OR 'mavrilimumab*' OR '1085337 57 0' OR 1158jdp9a* OR 'cam 3001' OR cam3001",130,27 Nov 2020

#109,"'ustekinumab'/de OR 'cnto 1275' OR 'cnto1275' OR 'monoclonal antibody cnto 1275' OR 'stelara' OR 'ustekinumab*' OR '815610 63 0' OR fu77b4u5z0* OR 'stelera'",7706,27 Nov 2020

#108,"'guselkumab'/de OR 'cnto 1959' OR 'cnto1959' OR 'guselkumab' OR 'tremfya' OR '1350289 85 8' OR 089658a12d*",831,27 Nov 2020

#107,"'brodalumab'/de OR 'amg 827' OR 'amg827' OR 'brodalumab*' OR 'kyntheum' OR 'siliq' OR 'khk 4827' OR khk4827 OR '1174395 19 7' OR 6za31y954z*",1173,27 Nov 2020

#106,"'ixekizumab'/de OR 'ixekizumab' OR 'ly 2439821' OR 'ly2439821' OR 'taltz' OR '1329632 62 3' OR '1143503 69 8' OR bty153760o*",1962,27 Nov 2020

#105,"'secukinumab'/de OR 'ain 457' OR 'ain457' OR 'cosentyx' OR 'secukinumab*' OR '1229022 83 6' OR dlg4eml025*",4070,27 Nov 2020

#104,"'pateclizumab'/de OR 'mlta 3698a' OR 'mlta3698a' OR 'pateclizumab' OR 'pro 283698' OR 'pro283698' OR '12202526 59 7' OR qok1yyh7j2* OR 'rg 7415' OR rg7415",28,27 Nov 2020

#103,"'tabalumab'/de OR 'ly 2127399' OR 'ly2127399' OR 'tabalumab*' OR '1143503 67 6' OR pqp8vh3mjw*",272,27 Nov 2020

#102,"abatacept*:ti,ab,kw,rn,tn OR 'ctla 4 ig':ti,ab,kw,rn,tn OR 'ctla-igg4m':ti,ab,kw,rn,tn OR 'ctla4-fc':ti,ab,kw,rn,tn OR '332348 12 6':ti,ab,kw,rn,tn OR 7d0yb67s97*:ti,ab,kw,rn,tn OR 'rg 2077':ti,ab,kw,rn,tn OR rg2077:ti,ab,kw,rn,tn OR 'rg-1046':ti,ab,kw,rn,tn OR rg1046:ti,ab,kw,rn,tn OR nulojix:ti,ab,kw,rn,tn OR lea29y:ti,ab,kw,rn,tn OR 'lea 29y':ti,ab,kw,rn,tn OR 'ctl4 fc':ti,ab,kw,rn,tn OR ctl4fc:ti,ab,kw,rn,tn OR belatacept:ti,ab,kw,rn,tn OR 'bms 224818':ti,ab,kw,rn,tn OR bms224818:ti,ab,kw,rn,tn OR 'cytotoxic t lymphocyte associat* antigen 4 immunglobulin*':ti,ab,kw,rn,tn",11029,27 Nov 2020

#101,"'abatacept'/de OR 'ctla4 ig' OR 'ctla4 immunoglobulin' OR 'ctla4 immunoglobulin g' OR 'ctla4ig' OR 'abatacept' OR 'bms 188667' OR 'bms188667' OR 'orencia'",11027,27 Nov 2020

#100,"'ofatumumab'/de OR 'humax cd20' OR 'humax-cd20' OR 'humaxcd20' OR 'arzerra' OR 'gsk 1841157' OR 'gsk1841157' OR 'humac cd20' OR 'ofatumumab*' OR 'omb 157' OR 'omb157' OR 'humax-cd20-2f2' OR '679818 59 8' OR m95kg522r0* OR 'hsdb 8170' OR hsdb8170",3083,27 Nov 2020

#99,"'rituximab'/de OR 'abp 798' OR 'abp798' OR 'blitzima' OR 'ct p10' OR 'ctp10' OR 'gp 2013' OR 'gp2013' OR 'hlx 01' OR 'hlx01' OR 'idec 102' OR 'idec c2b8' OR 'idec102' OR 'idecc2b8' OR 'mabthera' OR 'mab thera' OR 'mk 8808' OR 'mk8808' OR 'monoclonal antibody idec c2b8' OR 'pf 05280586' OR 'pf 5280586' OR 'pf05280586' OR 'pf5280586' OR 'r 105' OR 'r105' OR 'reditux' OR 'rg 105' OR 'rg105' OR 'ritemvia' OR 'ritumax' OR 'rituxan' OR 'rituximab*' OR 'rituximab abbs' OR 'rituximab pvvr' OR 'rituximab-abbs' OR 'rituximab-pvvr' OR 'rituxin' OR 'rituzena' OR 'rixathon' OR 'riximyo' OR 'ro 452294' OR 'ro452294' OR 'ruxience' OR 'truxima' OR 'tuxella' OR '174722 31 7' OR 4f4x42syq6* OR 'hsdb 7455' OR hsdb7455",86075,27 Nov 2020

#98,"'czp':ti,ab,kw,rn,tn OR '428863 50 7':ti,ab,kw,rn,tn OR g6adw90r16*:ti,ab,kw,rn,tn OR umd07x179e*:ti,ab,kw,rn,tn OR 'hsdb 7848':ti,ab,kw,rn,tn OR hsdb7848:ti,ab,kw,rn,tn",6879,27 Nov 2020

#97,"'certolizumab pegol'/de OR 'cdp 870' OR 'cdp870' OR 'certolizumab pegol' OR 'cimzia' OR 'pegylated tumor necrosis factor alpha antibody fab fragment' OR 'pegylated tumour necrosis factor alpha antibody fab fragment' OR 'pha 738144' OR 'pha738144'",6961,27 Nov 2020

#96,"'golimumab'/de OR 'cnto 148' OR 'cnto148' OR 'golimumab*' OR 'simponi' OR 'simponi aria' OR '476181 74 5' OR 91x1klu43e*",7380,27 Nov 2020

#95,"adalimumab*:ti,ab,kw,rn,tn OR fys6t7f842*:ti,ab,kw,rn,tn OR '331731 18 1':ti,ab,kw,rn,tn OR d2e7:ti,ab,kw,rn,tn OR 'hsdb 7851':ti,ab,kw,rn,tn OR hsdb7851:ti,ab,kw,rn,tn OR 'bcd-057':ti,ab,kw,rn,tn OR bcd057:ti,ab,kw,rn,tn OR 'chs-1420':ti,ab,kw,rn,tn OR chs1420:ti,ab,kw,rn,tn OR 'sb-5':ti,ab,kw,rn,tn OR sb5:ti,ab,kw,rn,tn",35470,27 Nov 2020

#94,"'adalimumab'/de OR 'abp 501' OR 'abp501' OR 'abrilada' OR 'abt d2e7' OR 'abtd2e7' OR 'adalimumab' OR 'adalimumab adaz' OR 'adalimumab adbm' OR 'adalimumab afzb' OR 'adalimumab atto' OR 'adalimumab beta' OR 'adalimumab bwwd' OR 'adalimumab-adaz' OR 'adalimumab-adbm' OR 'adalimumab-afzb' OR 'adalimumab-atto' OR 'adalimumab-bwwd' OR 'adaly' OR 'amgevita' OR 'amjevita' OR 'amsparity' OR 'avt 02' OR 'avt02' OR 'bat 1406' OR 'bat1406' OR 'bax 2923' OR 'bax 923' OR 'bax2923' OR 'bax923' OR 'bi 695501' OR 'bi695501' OR 'chs 1420' OR 'chs1420' OR 'cinnora' OR 'ct p17' OR 'ctp17' OR 'cyltezo' OR 'da 3113' OR 'da3113' OR 'dmb 3113' OR 'dmb3113' OR 'exemptia' OR 'fkb 327' OR 'fkb327' OR 'fyzoclad' OR 'gp 2017' OR 'gp2017' OR 'hadlima' OR 'halimatoz' OR 'hefiya' OR 'hlx 03' OR 'hlx03' OR 'hulio' OR 'humira' OR 'hyrimoz' OR 'ibi 303' OR 'ibi303' OR 'idacio' OR 'imraldi' OR 'kromeya' OR 'lu 200134' OR 'lu200134' OR 'm 923' OR 'm923' OR 'mabura' OR 'monoclonal antibody

d2e7' OR 'msb 11022' OR 'msb11022' OR 'ons 3010' OR 'ons3010' OR 'pf 06410293' OR 'pf 6410293' OR 'pf06410293' OR 'pf6410293' OR 'raheara' OR 'solymbic' OR 'trudexa' OR 'zrc 3197' OR 'zrc3197'",35401,27 Nov 2020

#93,"etanercept*:ti,ab,kw,tn,rn OR 'rhu tnfr fc':ti,ab,kw,tn,rn OR 'tnfr-immunoadhesin':ti,ab,kw,tn,rn OR 'tnt receptor fusion protein':ti,ab,kw,tn,rn OR 'tum$or necrosis factor receptor fc fusion protein':ti,ab,kw,tn,rn OR 'tnf receptor fusion protein':ti,ab,kw,tn,rn OR 'tnfr fc':ti,ab,kw,tn,rn OR 'recombinant human tnf':ti,ab,kw,tn,rn OR 'recombinant human dimeric tnf receptor type ii igg fusion protein':ti,ab,kw,tn,rn OR 'dwp-422':ti,ab,kw,tn,rn OR dwp422:ti,ab,kw,tn,rn OR 'hd-203':ti,ab,kw,tn,rn OR hd203:ti,ab,kw,tn,rn OR 'lbec 0101':ti,ab,kw,tn,rn OR lbec0101:ti,ab,kw,tn,rn OR 'sb 4':ti,ab,kw,tn,rn OR 'sb4':ti,ab,kw,tn,rn",16030,27 Nov 2020

#92,"'etanercept'/de OR 'avent' OR 'benepali' OR 'brenzys' OR 'chs 0214' OR 'chs0214' OR 'embrel' OR 'enbrel' OR 'enerceptan' OR 'enia 11' OR 'enia11' OR 'erelzi' OR 'etanercept*' OR 'etanercept szzs' OR 'etanercept ykro' OR 'etanercept-szzs' OR 'etanercept-ykro' OR 'eticovo' OR 'gp 2015' OR 'gp 2015c' OR 'gp2015' OR 'gp2015c' OR 'hd 203' OR 'hd203' OR 'infinitam' OR 'lbec 0101' OR 'lbec0101' OR 'lifmior' OR 'nepexto' OR 'opinercept' OR 'recombinant tumor necrosis factor receptor fc fusion protein' OR 'recombinant tumour necrosis factor receptor fc fusion protein' OR 'tnr 001' OR 'tnr001' OR 'tumor necrosis factor receptor fc fusion protein' OR 'tumour necrosis factor receptor fc fusion protein' OR 'tunex' OR 'ylb 113' OR 'ylb113' OR '185243 69 0' OR 'op401g7ojc*'",33194,27 Nov 2020

#91,"ifx:ti,ab,kw,de,tn,rn OR 'mab ca2':ti,ab,kw,de,tn,rn OR 'monoclonal antibody ca2':ti,ab,kw,de,tn,rn OR 'antibody ca2 monoclonal':ti,ab,kw,de,tn,rn OR 'ca2 monoclonal antibody':ti,ab,kw,de,tn,rn OR 'ct p13':ti,ab,kw,de,tn,rn OR ctp13:ti,ab,kw,de,tn,rn OR 'ct-p-13':ti,ab,kw,de,tn,rn OR sb2:ti,ab,kw,de,tn,rn OR 'hsdb 7850':ti,ab,kw,de,tn,rn OR hsdb7850:ti,ab,kw,de,tn,rn",5765,27 Nov 2020

#90,"'infliximab'/de OR 'abp 710' OR 'abp710' OR 'avakine' OR 'avsola' OR 'flixabi' OR 'gp 1111' OR 'gp1111' OR 'inflectra' OR 'infliximab*' OR 'infliximab abda' OR 'infliximab axxq' OR 'infliximab dyyb' OR 'infliximab qbtx' OR 'infliximab-abda' OR 'infliximab-axxq' OR 'infliximab-dyyb' OR 'infliximab-qbtx' OR 'ixifi' OR 'pf 06438179' OR 'pf 6438179' OR 'pf06438179' OR 'pf6438179' OR 'remicade' OR 'remsima' OR 'renflexis' OR 'revellex' OR 'ta 650' OR 'ta650' OR 'zessly' OR '170277 31 3' OR 'b72hh48flu*' OR 'inflecta'",52777,27 Nov 2020

#89,"((('il-1ra' OR il1ra OR 'il-1-ra') NEAR/10 (antagonist* OR 'blocking agent*' OR blocker* OR inhibitor* OR anakinra*)):ti,ab,kw,tn) AND (therap*:ti,ab,kw,de OR treat*:ti,ab,kw,de OR trial$:ti,ab,kw,de)",2221,27 Nov 2020

#88,"'anakinra'/de OR 'anakinra*' OR 'kineret' OR 'recombinant interleukin 1 receptor antagonist' OR 'recombinant interleukin 1 receptor blocker' OR 'recombinant interleukin 1 receptor blocking agent' OR '143090 92 0'",9249,27 Nov 2020

#87,"#76 AND #86",4583,27 Nov 2020

#86,"#77 OR #78 OR #79 OR #80 OR #81 OR #82 OR #83 OR #84 OR #85",30224,27 Nov 2020

#85,"'elsilimomab'/de OR 'b e8' OR 'elsilimomab*' OR '468715 71 1'",38,27 Nov 2020

#84,"'ziltivekimab'/de OR 'cor 001' OR 'cor001' OR 'ziltivekimab*' OR ziltivekimab* OR zilti OR '2226654 05 1'",4,27 Nov 2020

#83,"'siltuximab'/de OR 'cnto 328' OR 'cnto328' OR 'monoclonal antibody cnto 328' OR 'monoclonal antibody cnto328' OR 'siltuximab*' OR 'sylvant' OR cllb8 OR 'ccib8 monoclonal antibody' OR t4h8fma7im* OR '541502 14 1'",856,27 Nov 2020

#82,"'sirukumab'/exp OR 'cnto 136' OR 'cnto136' OR 'plivensia' OR 'sirukumab*' OR '1194585 53 9' OR 640443fu93*",320,27 Nov 2020

#81,"'clazakizumab'/de OR 'ald 518' OR 'ald518' OR 'bms 945429' OR 'bms945429' OR 'clazakizumab*' OR '1236278 28 6' OR 4s38z8ra9o*",217,27 Nov 2020

#80,"'olokizumab'/de OR 'olokizumab*' OR 'cdp 6038' OR cdp6038 OR '1007223 17 7' OR pai71r1d2w*",95,27 Nov 2020

#79,"'levilimab'/de OR 'bcd 089' OR 'bcd089' OR 'levilimab*' OR '2035008 70 7' OR p7uv3l2h80*",5,27 Nov 2020

#78,"'sarilumab'/de OR 'kevzara*' OR 'regn 88' OR 'regn88' OR 'sar 153191' OR 'sar153191' OR 'sarilumab*' OR '1189541 98 7' OR nu90v55f8i*",816,27 Nov 2020

#77,"'tocilizumab'/de OR 'actemra*' OR 'actemra 200' OR 'atlizumab*' OR 'lusinex*' OR 'r1569' OR 'roactemra*' OR 'tocilizumab*' OR r1569 OR 'r 1569' OR 'mra':ti,ab,kw,tn OR mra:tt OR '375823 41 9' OR i031v2h011* OR 'rhpm-1' OR rhpm1 OR 'rg-1569' OR rg1569 OR 'msb-11456' OR msb11456 OR 'ro-4877533' OR ro4877533",29142,27 Nov 2020

#76,"#1 OR #2 OR #3 OR #4 OR #5 OR #6 OR #7 OR #8 OR #9 OR #10 OR #13 OR #14 OR #15 OR #16 OR #17 OR #18 OR #19 OR #20 OR #21 OR #22 OR #23 OR #24 OR #25 OR #26 OR #27 OR #28 OR #29 OR #30 OR #31 OR #32 OR #33 OR #34 OR #35 OR #36 OR #37 OR #38 OR #39 OR #40 OR #41 OR #42 OR #43 OR #44 OR #45 OR #46 OR #47 OR #48 OR #49 OR #50 OR #51 OR #52 OR #53 OR #54 OR #55 OR #56 OR #57 OR #58 OR #59 OR #60 OR #61 OR #62 OR #63 OR #64 OR #65 OR #66 OR #67 OR #68 OR #69 OR #70 OR #71 OR #72 OR #73 OR #74 OR #75",368076,27 Nov 2020

#75,"((axial NEAR/3 spondyl$arthrit*):ti,ab,kw) OR ((axial NEAR/3 spondyl$arthrit*):tt)",3877,27 Nov 2020

#74,"axspa*:ti,ab,kw OR axspa*:tt",2558,27 Nov 2020

#73,"'bath ankylosing spondylitis disease activity index'/de",4414,27 Nov 2020

#72,"'rheumat* spondylit*':tt OR 'axial spondyl$arthrit*':tt OR 'bechterew* disease*':tt OR 'bechterew s disease*':tt OR 'bechterews disease*':tt OR 'marie struempell disease*':tt",8,27 Nov 2020

#71,"'rheumat* spondylit*':ti,ab,kw OR 'axial spondyl$arthrit*':ti,ab,kw OR 'bechterew* disease*':ti,ab,kw OR 'bechterew s disease*':ti,ab,kw OR 'bechterews disease*':ti,ab,kw OR 'marie struempell disease*':ti,ab,kw",4416,27 Nov 2020

#70,"((spondylitis* OR spondylarthrit* OR spondyloarthrit* OR spine OR vertebral) NEAR/1 ankyl*):tt",1139,27 Nov 2020

#69,"((spondylitis* OR spondylarthrit* OR spondyloarthrit* OR spine OR vertebral) NEAR/1 ankyl*):ti,ab,kw",25483,27 Nov 2020

#68,"'ankylosing spondylitis disease activity score'/de",1891,27 Nov 2020

#67,"'ankylosing spondylitis'/de OR 'bechterew disease' OR 'ankylating spondylitis' OR 'ankylopoietic spondylarthritis' OR 'ankylopoietic spondylitis' OR 'ankylosing spine' OR 'ankylosing spondilitis' OR 'ankylosing spondylarthritis' OR 'ankylosing spondylarthrosis' OR 'ankylosing spondylitis' OR 'ankylosis spondylitis' OR 'ankylotic spondylitis' OR 'bekhterev disease' OR 'morbus bechterew' OR 'spinal ankylosis' OR 'spine ankylosis' OR 'spondylarthritis ankylopoietica' OR 'spondylarthritis ankylosans' OR 'spondylarthrosis ankylopoietica' OR 'spondylitis ankylopoetica' OR 'spondylitis ankylopoietica' OR 'spondylitis, ankylosing' OR 'spondyloarthritis ankylopoietica' OR 'vertebral ankylosis'",35176,27 Nov 2020

#66,"'ankylosing spondylitis'/de",29599,27 Nov 2020

#65,"((psoria* NEXT/6 ('inflammat* arthrit*' OR 'inflammat* polyarthrit*')):ti,ab,kw) OR ((psoria* NEXT/6 ('inflammat* arthrit*' OR 'inflammat* polyarthrit*')):tt)",323,27 Nov 2020

#64,"((psoria* NEXT/1 rheumat*):ti,ab,kw) OR ((psoria* NEXT/1 rheumat*):tt)",388,27 Nov 2020

#63,"((psoria* NEXT/2 arthrit*):tt) OR ((psoria* NEXT/2 polyarthrit*):tt) OR ((psoria* NEXT/2 arthropath*):tt) OR ((psoria* NEXT/2 'poly arthrit*'):tt)",184,27 Nov 2020

#62,"((psoria* NEXT/2 arthrit*):ti,ab,kw) OR ((psoria* NEXT/2 polyarthrit*):ti,ab,kw) OR ((psoria* NEXT/2 arthropath*):ti,ab,kw) OR ((psoria* NEXT/2 'poly arthrit*'):ti,ab,kw)",20191,27 Nov 2020

#61,"'psoriatic arthritis'/exp OR 'alibert bazin disease' OR 'arthritis psoriatica' OR 'arthritis, psoriasis' OR 'arthritis, psoriatic' OR 'arthropathic psoriasis' OR 'arthropathy, psoriatic' OR 'disease, alibert bazin' OR 'polyarthritis, psoriatic' OR 'psoriasis arthropathica' OR 'psoriasis pustulosa arthropathica' OR 'psoriasis, arthritis' OR 'psoriatic arthritis' OR 'psoriatic arthropathy' OR 'psoriatic polyarthritis' OR 'psoriatic rheumatism' OR 'psoriatic rheumatoid arthritis' OR 'rheumatoid arthritis, psoriatic'",26345,27 Nov 2020

#60,"(('pauci immune*' NEAR/6 vasculit*):ti,ab,kw) OR (('pauci immune*' NEAR/6 vasculit*):tt)",266,27 Nov 2020

#59,"((anca OR 'antineutrophil cytoplasmic' OR 'anti-neutrophil cytoplasmic') NEAR/6 vasculit*):tt",66,27 Nov 2020

#58,"((anca OR 'antineutrophil cytoplasmic' OR 'anti-neutrophil cytoplasmic') NEAR/6 vasculit*):ti,ab,kw",8172,27 Nov 2020

#57,"'anca associated vasculitis'/de OR 'anca associated vasculitis' OR 'anca vasculitis' OR 'anca-associated vasculitis' OR 'anti neutrophil cytoplasmic antibody associated vasculitis' OR 'anti-neutrophil cytoplasmic antibody-associated vasculitis' OR 'antineutrophil cytoplasmic antibody associated vasculitis' OR 'neutrophil cytoplasmic antibody associated vasculitis'",7508,27 Nov 2020

#56,"'myelopticoneuropath*':tt OR 'myelooptic* neuropath*':tt OR neuroopticomyelit*:tt",8,27 Nov 2020

#55,"'myeloopticoneuropath*':ti,ab,kw OR 'myelooptic* neuropath*':ti,ab,kw OR neuroopticomyelit*:ti,ab,kw",167,27 Nov 2020

#54,"((devic OR devics OR 'devic s') NEAR/2 (disease$ OR syndrom* OR 'neuromyelit* optic*')):tt",10,27 Nov 2020

#53,"((devic OR devics OR 'devic s') NEAR/2 (disease$ OR syndrom* OR 'neuromyelit* optic*')):ti,ab,kw",769,27 Nov 2020

#52,"((nmo:ti,ab,kw OR nmosd:ti,ab,kw) AND (neuromyelit*:ti,ab,kw OR neuropath*:ti,ab,kw,de) OR nmo:ti,ab,kw OR nmosd:tt) AND (neuromyelit*:ti,ab,kw OR neuropath*:ti,ab,kw,de)",6137,27 Nov 2020

#51,"((neuromyelit* NEAR/1 optic*):ti,ab,kw) OR ((neuromyelit* NEAR/1 optic*):tt)",8308,27 Nov 2020

#50,"'myelooptic neuropathy'/de OR 'devic disease' OR 'devic syndrome' OR 'myelooptic neuropathy' OR 'myeloopticoneuropathy' OR 'myeloptico neuropathy' OR 'myelopticoneuropathy' OR 'neuromyelitis optica' OR 'neuromyelitis optica spectrum disorder' OR 'neuropticomyelitis' OR 'optic neuromyelitis'",10191,27 Nov 2020

#49,"('neonatal onset' NEAR/3 'multisystem* inflammat*' NEAR/1 (disease* OR disorder*)):ti,ab,kw",264,27 Nov 2020

#48,"(('chronic infantile' NEAR/3 'neurolog* cutaneous' NEAR/3 ('articular* syndrom*' OR arthropath*)):ti,ab,kw) OR (('chronic infantile' NEAR/3 'neurolog* cutaneous' NEAR/3 ('articular* syndrom*' OR arthropath*)):tt)",231,27 Nov 2020

#47,"'cinca syndrome'/exp OR 'cinca' OR 'cinca syndrome' OR 'nomid' OR 'chronic infantile neurologic cutaneous and articular syndrome' OR 'chronic infantile neurologic cutaneous articular syndrome' OR 'chronic infantile neurologic, cutaneous and articular syndrome' OR 'chronic infantile neurologic,

cutaneous, articular syndrome' OR 'chronic infantile neurological cutaneous and articular syndrome' OR 'chronic infantile neurological cutaneous arthropathy' OR 'chronic infantile neurological cutaneous articular syndrome' OR 'chronic infantile neurological, cutaneous and articular syndrome' OR 'chronic infantile neurological, cutaneous, articular syndrome' OR 'neonatal onset multisystem inflammatory disease' OR 'neonatal onset multisystem inflammatory disorder' OR 'neonatal onset multisystemic inflammatory disease' OR 'neonatal onset multisystemic inflammatory disorder'",2214,27 Nov 2020
#46,"(('tnf receptor-associated periodic' OR 'tnfr1 associated' OR 'tumo$r necrosis factor receptor associated' OR 'tnfr associated') NEAR/3 (syndrome* OR fever)):tt",2,27 Nov 2020
#45,"(('tnf receptor-associated periodic' OR 'tnfr1 associated' OR 'tumo$r necrosis factor receptor associated' OR 'tnfr associated') NEAR/3 (syndrome* OR fever)):ti,ab,kw",713,27 Nov 2020
#44,"(traps:ti,ab,kw OR traps:tt) AND ('tnf receptor':ti,ab,kw,de OR 'tumo$r necrosis factor receptor':ti,ab,kw,de)",695,27 Nov 2020
#43,"'tumor necrosis factor receptor associated periodic syndrome'/de OR 'tnf receptor 1 associated periodic syndrome' OR 'tnf receptor associated periodic syndrome' OR 'tnf receptor superfamily 1a associated periodic syndrome' OR 'tnfr1 associated periodic syndrome' OR 'tnfr associated periodic syndrome' OR 'traps (tumor necrosis factor receptor associated periodic syndrome)' OR 'traps (tumour necrosis factor receptor associated periodic syndrome)' OR 'autosomal dominant periodic fever with amyloidosis' OR 'benign autosomal dominant familial periodic fever' OR 'familial hibernian fever' OR 'tumor necrosis factor receptor 1 associated periodic syndrome' OR 'tumor necrosis factor receptor associated periodic fever' OR 'tumor necrosis factor receptor associated periodic fever syndrome' OR 'tumor necrosis factor receptor associated periodic syndrome' OR 'tumor necrosis factor receptor superfamily 1a associated periodic syndrome' OR 'tumour necrosis factor receptor 1 associated

periodic syndrome' OR 'tumour necrosis factor receptor associated periodic fever' OR 'tumour necrosis factor receptor associated periodic fever syndrome' OR 'tumour necrosis factor receptor associated periodic syndrome' OR 'tumour necrosis factor receptor superfamily 1a associated periodic syndrome'",1124,27 Nov 2020

#42,"(('von meyenburg' NEAR/3 disease*):ti,ab,kw) OR (('von meyenburg' NEAR/3 disease*):tt)",13,27 Nov 2020

#41,"((chondromalac* NEAR/3 systemic*):ti,ab,kw) OR ((chondromalac* NEAR/3 systemic*):tt)",5,27 Nov 2020

#40,"((polychondrit* NEAR/3 (relapsing* OR atrophic*)):ti,ab,kw) OR ((polychondrit* NEAR/3 (relapsing* OR atrophic*)):tt)",1962,27 Nov 2020

#39,"'relapsing polychondritis'/de",2112,27 Nov 2020

#38,"((amyloid* NEAR/3 (neuropath* OR tumo$r* OR angiopath* OR senile*)):ti,ab,kw) OR ((amyloid* NEAR/3 (neuropath* OR tumo$r* OR angiopath* OR senile*)):tt)",8207,27 Nov 2020

#37,"'amyloidos$s':ti,ab,kw OR amyloidoma*:ti,ab,kw OR 'beta fibrillos$s':ti,ab,kw OR paraamyloidos$s:ti,ab,kw OR 'amyloidos$s':tt OR amyloidoma*:tt OR 'beta fibrillos$s':tt OR paraamyloidos$s:tt",34314,27 Nov 2020

#36,"'amyloidosis'/exp",51266,27 Nov 2020

#35,"((infectious* OR focal OR proliferativ*) NEAR/3 myopath*):ti,ab,kw",165,27 Nov 2020

#34,"((inflammat* NEAR/3 idiopath* NEAR/3 (myopath* OR myosit*)):ti,ab,kw) OR ((inflammat* NEAR/3 idiopath* NEAR/3 (myopath* OR myosit*)):tt)",2724,27 Nov 2020

#33,"((inflammat* NEAR/1 (myopath* OR muscle$)):ti,ab,kw) OR ((inflammat* NEAR/1 (myopath* OR muscle$)):tt)",6811,27 Nov 2020

#32,"myositis:ti,ab,kw OR neuromyositis:ti,ab,kw OR myositis:tt OR neuromyositis:tt",15287,27 Nov 2020

#31,"'myositis'/exp",41179,27 Nov 2020

#30,"'libman sacks endocarditis'/de",112,27 Nov 2020

#29,"'libman sacks disease'/de",24,27 Nov 2020

#28,"'libman sacks disease*':ti,ab,kw OR 'libman sacks disease*':tt",19,27 Nov 2020

#27,"(sle:ti,ab,kw OR 's l e':ti,ab,kw OR sle:tt OR 's l e':tt) AND lupus",52367,27 Nov 2020

#26,"(('lupus erythemato*' NEAR/3 (systemic* OR disseminat* OR visceral*)):ti,ab,kw) OR (('lupus erythemato*' NEAR/3 (systemic* OR disseminat* OR visceral*)):tt)",77370,27 Nov 2020

#25,"'systemic lupus erythematosus'/exp",98941,27 Nov 2020

#24,"(('remitting seronegative' NEAR/3 synovitis NEAR/6 ('edem*' OR 'oedem*')):ti,ab,kw) OR (('remitting seronegative' NEAR/3 synovitis NEAR/6 ('edem*' OR 'oedem*')):tt)",255,27 Nov 2020

#23,"rs3pe*:ti,ab,kw OR rs3pe*:tt",242,27 Nov 2020

#22,"'rs3pe syndrome'/de",15,27 Nov 2020

#21,"'remitting seronegative symmetrical synovitis with pitting edema syndrome'/de",42,27 Nov 2020

#20,"'remitting seronegative symmetrical synovitis with pitting edema'/de",67,27 Nov 2020

#19,"(('forestier-certonciny' NEAR/1 syndrom*):ti,ab,kw) OR (('forestier certonciny' NEAR/1 syndrom*):tt)",2,27 Nov 2020

#18,"(((pseudopolyarthrit* OR 'pseudo polyarthrit*' OR 'inflammat* rheumat*' OR 'rheumat* inflammat*') NEAR/1 rhizomelic):ti,ab,kw) OR (((pseudopolyarthrit* OR 'pseudo polyarthrit*' OR 'inflammat* rheumat*' OR 'rheumat* inflammat*') NEAR/1 rhizomelic):tt)",88,27 Nov 2020

#17,"(((rheumat* OR arteritic*) NEAR/1 (polymyalgia* OR myalgia*)):ti,ab,kw) OR (((rheumat* OR arteritic*) NEAR/1 (polymyalgia* OR myalgia*)):tt)",4334,27 Nov 2020

#16,"'rheumatic polymyalgia'/de",5810,27 Nov 2020

#15,"('systemic scleros$s*':ti,ab,kw OR 'systemic scleros$s*':tt OR ssc:ti,ab,kw) AND ('interstitial lung disease$':ti,ab,kw,de OR 'interstitial pneumoni*':ti,ab,kw,de OR 'interstitial lung disease$':tt OR 'interstitial pneumoni*':tt)",3841,27 Nov 2020

#14,"'ssc-ild*':ti,ab,kw OR 'sscild*':ti,ab,kw",789,27 Nov 2020

#13,"#11 AND #12",4503,27 Nov 2020

#12,"'interstitial lung disease'/exp",85995,27 Nov 2020

#11,"'systemic sclerosis'/exp",33079,27 Nov 2020

#10,"'severe acute respiratory syndrome coronavirus 2':tt OR 'sars coronavirus 2':tt OR 'sars-cov-2*':tt OR 'sars-cov2*':tt OR 'sars-covid-2*':tt OR sarscov2*:tt OR 'sars-2-cov*':tt",665,27 Nov 2020

#9,"'severe acute respiratory syndrome coronavirus 2':ti,ab,kw OR 'sars coronavirus 2':ti,ab,kw OR 'sars-cov-2*':ti,ab,kw OR 'sars-cov2*':ti,ab,kw OR 'sars-covid-2*':ti,ab,kw OR sarscov2*:ti,ab,kw OR 'sars-2-cov*':ti,ab,kw",24248,27 Nov 2020

#8,"'severe acute respiratory syndrome coronavirus 2'/exp",17605,27 Nov 2020

#7,"((covid OR ncov OR 'n-cov' OR cov OR coronavir* OR 'corona vir*' OR betacoronavir*) NEAR/3 ('19$' OR '2019*' OR wuhan OR novel* OR new OR newly)):tt",3083,27 Nov 2020

#6,"((covid OR ncov OR 'n-cov' OR cov OR coronavir* OR 'corona vir*' OR betacoronavir*) NEAR/3 ('19$' OR '2019*' OR wuhan OR novel* OR new OR newly)):ti,ab,kw",68310,27 Nov 2020

#5,"'2019-ncov*':tt OR '2019-n-cov*':tt OR 2019ncov*:tt OR '2019-novel-cov*':tt OR ncov2019*:tt OR 'n-cov2019*':tt OR 'ncov-2019*':tt OR 'n-cov-2019*':tt OR 'novel cov-2019*':tt OR 'novel cov-19*':tt OR ncov19*:tt OR 'n-cov19*':tt OR 'ncov-19*':tt OR 'n-cov-19*':tt OR 'hcov-19':tt",21,27 Nov 2020

#4,"'2019-ncov*':ti,ab,kw OR '2019-n-cov*':ti,ab,kw OR 2019ncov*:ti,ab,kw OR '2019-novel-cov*':ti,ab,kw OR ncov2019*:ti,ab,kw OR 'n-cov2019*':ti,ab,kw OR 'ncov-2019*':ti,ab,kw OR 'n-cov-2019*':ti,ab,kw OR 'novel cov-2019*':ti,ab,kw OR 'novel cov-19*':ti,ab,kw OR ncov19*:ti,ab,kw OR 'n-cov19*':ti,ab,kw OR 'ncov-19*':ti,ab,kw OR 'n-cov-19*':ti,ab,kw OR 'hcov-19':ti,ab,kw",1407,27 Nov 2020

#3,"'covid-19*':tt OR 'covid19*':tt OR 'covid-2019*':tt OR 'covid2019*':tt",3068,27 Nov 2020

#2,"'covid-19*':ti,ab,kw OR 'covid19*':ti,ab,kw OR 'covid-2019*':ti,ab,kw OR 'covid2019*':ti,ab,kw",64846,27 Nov 2020

#1,"'coronavirus disease 2019'/de",65249,27 Nov 2020

1.3.2.3 Cochrane CENTRAL

1 exp Coronavirus/ (10)

2 exp Coronavirus Infections/ (513)

3 (coronavirus* or corona virus* or OC43 or NL63 or 229E or HKU1 or HCoV* or ncov* or covid* or sars-cov* or sarscov* or Sars-coronavirus* or Severe Acute Respiratory Syndrome Coronavirus*).mp. (4896)

4 (or/1-3) and (20191* or 202*).up. (3779)

5 4 not (SARS or SARS-CoV or MERS or MERS-CoV or Middle East respiratory syndrome or camel* or dromedar* or equine or coronary or coronal or covidence* or covidien or influenza virus or HIV or bovine or calves or TGEV or feline or porcine or BCoV or PED or PEDV or PDCoV or FIPV or FCoV or SADS-CoV or canine or CCov or zoonotic or avian influenza or H1N1 or H5N1 or H5N6 or IBV or murine corona*).mp. (2117)

6 ((pneumonia or covid* or coronavirus* or corona virus* or ncov* or 2019-ncov or sars*).mp. or exp pneumonia/) and Wuhan.mp. (148)

7 (2019-ncov* or 2019-n-cov*or 2019ncov* or 2019n-cov* or ncov19 or ncov-19 or 2019-novel CoV*or ncov2019* or n-cov2019* or ncov-2019* or n-cov-2019* or novel cov-2019* or novel-cov-19* or ncov19* or n-cov19* or ncov-19* or n-cov-19* or sars-cov2* or sars-cov-2* or sarscov2* or sarscov-2* or sars-covid-2* or sars-2-cov* or Sars-coronavirus2* or Sars-coronavirus-2* or SARS-like coronavirus* or coronavirus-19 or covid19 or covid-19 or covid 2019 or ((novel or new or newly or nouveau or "19?" or "2019*" or wuhan) adj2 (CoV on nCoV or n-cov or covid or coronavirus* or corona virus or betacoronavirus* or Pandemi*2)) or ((covid or covid19 or covid-19) and pandemic*2) or (coronavirus* and pneumonia)).mp. (3582)

8 (severe acute respiratory syndrome coronavirus 2* or severe acute respiratory syndrome cov* 2* or severe acute respiratory syndrome coronavirus 2019* or severe acute respiratory syndrome cov* 2019* or severe acute respiratory syndrome coronavirus 19* or severe acute respiratory syndrome cov* 19*).mp. [mp=title, original title, abstract, mesh headings, heading words, keyword] (252)

9 6 or 7 or 8 (3585)

10 5 or 9 (3676)

11 10 and (20191* or 202*).up. (3649)

12 exp Lung Diseases, Interstitial/ (824)

13 exp Scleroderma, Systemic/ (559)

14 12 and 13 (40)

15 (((systemic adj3 (sclerosis* or sleroderma*)) or ssc) and (interstitial adj3 (lung disease* or pneumoni*))).mp. (255)

16 (ssc-ild* or sscild*).ti,ot,ab. (141)

17 Polymyalgia Rheumatica/ (81)

18 pmr.ti,ot,ab. and rheum*.mp. [mp=title, original title, abstract, mesh headings, heading words, keyword] (106)

19 ((rheumat* or arteritic*) adj1 (polymyalgia* or myalgia*)).mp. (224)

20 ((pseudopolyarthrit* or pseudo polyarthrit* or inflammat* rheumat* or rheumat* inflammat*) adj1 rhizomelic).mp. (0)

21 (forestier-certonciny adj1 syndrom*).mp. (0)

22 (remitting adj3 seronegative adj3 synovitis adj6 (edem* or oedem*)).mp. (1)

23 rs3pe*.ti,ot,ab. (1)

24 exp Synovitis/ (186)

25 Edema/ (1773)

26 24 and 25 (7)

27 (remitting or seronegativ* or symmetric*).mp. (7459)

28 26 and 27 (0)

29 exp Lupus Erythematosus, Systemic/ (1050)

30 (lupus erythemato* adj3 (systemic* or disseminat* or visceral*)).mp. (2518)

31 (sle or 's l e').ti,ot,ab. (2010)

32 (lupus or autoimmun* or auto immun* or autoantibod* or antibod* or inflamm* or immun* or rheumat* or arthrit* or nephritis or nephrol* or cutan* or dermatol* or venerol* or neuromyotoni* or neuroscience? or neurolog* or patient? or cell debris).mp,jw. (1135159)

33 31 and 32 (1981)

34 (libman sacks adj1 (disease* or endocarditis*)).mp. (3)

35 exp Myositis/ (193)

36 (myositis or neuromyositis or polymyositis or dermatomyositis).mp. (870)

37 (inflammat* adj1 (myopath* or muscle? or myosit*)).mp. (361)

38 (inflammat* adj3 idiopath* adj3 (myopath* or myosit*)).mp. (78)

39 ((infect* or focal or proliferativ*) adj3 (myopath* or myosit*)).ti,ot,ab. (10)

40 exp Amyloidosis/ (165)

41 (amyloidos#s or amyloidoma* or beta fibrillos#s or paraamyloidos#s).mp. (647)

42 Polychondritis, Relapsing/ (0)

43 (polychondrit* adj3 (relapsing* or atrophic*)).mp. (22)

44 (chondromalac* adj3 systemic*).mp. (0)

45 (von meyenburg adj3 disease*).mp. (0)

46 (periodic fever adj1 familial adj1 autosomal dominant).mp. (0)

47 ((tnf receptor or tnfr1 or tnfr) adj3 associated periodic syndrom*).mp. (23)

48 (traps and (tumo?r necrosis factor receptor or tnf receptor)).ti,ot,ab. (30)

49 (tumo?r necrosis factor receptor adj3 associated periodic adj2 (syndrom* or fever)).mp. (33)

50 (autosomal dominant adj3 periodic fever).mp. (0)

51 (hibernian fever adj3 familial).mp. (0)

52 ((tnf receptor associated periodic or tnfr1 associated or tumo?r necrosis factor receptor associated or tnfr associated) adj3 (syndrom* or fever*)).mp. (33)

53 Cryopyrin-Associated Periodic Syndromes/ (20)

54 (cinca or cinca syndrom*).mp. (16)

55 (chronic infantile adj3 neurolog* cutan* adj3 (articul* syndrom* or arthropath*)).mp. (2)

56 (neonatal onset adj3 multisyst* inflamm* adj1 (diseas* or disorder*)).mp. (3)

57 nomid.ti,ot,ab. (4)

58 iomid.ti,ot,ab. (0)

59 cryopyrin associated periodic syndrom*.mp. (42)

60 Neuromyelitis Optica/ (33)

61 (neuromyelit* adj1 optic*).mp. (222)

62 (myelooptic* adj1 neuropath*).mp. (164)

63 ((nmo or nmosd) and (neuromyelit* or neuropath*)).ti,ot,ab. (179)

64 ((devic or devics or devic's) adj2 (diseas* or syndrom* or neuromyelit* optic*)).mp. (5)

65 (myeloopticoneuropath* or myelooptic* neuropath* or neuroopticomyelit*).mp. (164)

66 exp Anti-Neutrophil Cytoplasmic Antibody-Associated Vasculitis/ (121)

67 ((anca or antineutrophil cytoplasmic or anti-neutrophil cytoplasmic) adj6 vasculit*).mp. (522)

68 (pauci immun* adj6 vasculit*).ti,ot,ab. (4)

69 exp Arthritis, Psoriatic/ (445)

70 (psoria* adj2 (arthrit* or polyarthrit* or poly arthrit* or arthropath*)).ti,ot,ab. (2204)

71 (psoria* adj1 rheumat*).ti,ot,ab. (65)

72 (psoria* adj6 (inflammat* arthrit* or inflammat* polyarthrit* or inflammat* poly arthrit*)).ti,ot,ab. (43)

73 Spondylitis, Ankylosing/ (687)

74 ((spondylitis* or spondylarthrit* or spndyloarthrit* or spine or vertebral) adj1 ankyl*).mp. (2309)

75 (rheumat* spondylit* or axial spondyl?arthrit* or bechterew* disease* or bechterews disease* or bechterew's disease* or marie struempell disease*).mp. (587)

76 (axspa* or ax-spa*).ti,ot,ab. (428)

77 (axial adj3 spondyl?arthrit*).ti,ot,ab. (598)

78 11 or 14 or 15 or 16 or 17 or 18 or 19 or 20 or 21 or 22 or 23 or 28 or 29 or 30 or 33 or 34 or 35 or 36 or 37 or 38 or 39 or 40 or 41 or 42 or 43 or 44 or 45 or 46 or 47 or 48 or 49 or 50 or 51 or 52 or 53 or 54 or 55 or 56 or 57 or 58 or 59 or 60 or 61 or 62 or 63 or 64 or 65 or 66 or 67 or 68 or 69 or 70 or 71 or 72 or 73 or 74 or 75 or 76 or 77 (13874)

79 (tocilizumab* or actemra* or atlizumab* or lusinex* or r1569 or r 1569 or roactemra* or mra or 375823 41 9 or i031v2h011* or rhpm-1 or rhpm1 or rg-1569 or rg1569 or msb-11456 or msb11456 or ro-4877533 or ro4877533).mp. (1989)

80 (sarilumab* or kevzara* or regn 88 or regn88 or sar 153191 or sar153191 or 1189541 98 7 or nu90v55f8i*).mp. (255)

81 (levilimab* or BCD-089 or bcd089 or 2035008 70 7 or P7UV3L2H80*).mp. (5)

82 (olokizumab* or cdp 6038 or cdp6038 or 1007223 17 7 or pai71r1d2w*).mp. (35)

83 (clazakizumab* or ald 518 or ald518 or bms 945429 or bms945429 or 1236278 28 6 or 4s38z8ra9o*).mp. (38)

84 (sirukumab* or cnto 136 or cnto136 or pilvensia or 1194585 53 9 or 640443FU93*).mp. (118)

85 (siltuximab* or sylvant or cnto 328 or cnto-328 or cnto328 or cllb8 or ccib8 monoclonal antibody or sylvant or t4h8fma7im* or 541502 14 1).mp. (78)

86 (ziltivekimab* or zilti or "cor-001" or "2226654 05 1").mp. (5)

87 (elsilimomab* or "b-e8" or 468715 71 1).mp. (2)

88 79 or 80 or 81 or 82 or 83 or 84 or 85 or 86 or 87 (2464)

89 78 and 88 (282)

90 Interleukin 1 Receptor Antagonist Protein/ (305)

91 (anakinra* or kineret or ((interleukin 1 receptor or il1 receptor or il 1 receptor) adj1 antagonist protein*) or (recombinant interleukin 1 receptor adj1 (antagonist* or blocker* or blocking agent*)) or antril or il 1ra or il1ra or il 1 ra or ((urin* or febrile) adj2 (interleukin 1 inhibitor* or il1 inhibitor* or il 1 inhibitor*)) or "143090 92 0" or 9013duq28k*).mp. (1087)

92 Infliximab/ (732)

93 (infliximab* or ifx or avakine or flixabi or inflectra or inflecta or ixifi or remicade or remsima or revellex or renflexis or zessly or mab ca2 or monoclonal antibody ca2 or antibody ca2 monoclonal or ca2 monoclonal antibody or ct p13 or ctp13 or ct-p-13 gp-1111 or gp1111 or ta-650 or ta650 or abp 710 or abp710 or bow-015 or bow015 or 170277 31 3 or b72hh48flu* or sb2 or "pf 06438179" or pf 6438179 or pf06438179 or pf6438179 or hsdb 7850 or hsdb7850).mp. (2769)

94 Etanercept/ (758)

95 (etanercept* or benepali or embrel or enbrel or enia 11 or enia11 or erelzi or lifmior or opinercept or recombinant tumo?r necrosis factor receptor fc fusion protein or "tnr 001" or tnr001 or tnfr-fc or tnrfr:fc or rhu tnfr:fc or rhu-tnfr:fc or tnfr-immunoadhesin or tnt receptor fusion protein or tum?or necrosis factor receptor fc fusion protein or tunex or "185243 69 0" or op401g7ojc* or brenzys or tnf receptor fusion protein or tnfr fc or recombinant human tnf or recombinant human dimeric tnf receptor type ii igg fusion protein or chs-0214 or chs0214 or dwp-422 or dwp422 or enia-11 or enia11 or gp-2015 or gp2015 or gp2015c or gp-2015c or hd-203 or hd203 or lbec-0101 or lbec0101 or sb-4 or sb4).mp. (2451)

96 Adalimumab/ (752)

97 (adalimumab* or humira or abp 501 or abp501 or abtd2e7 or amjevita or amgevita or bax 2923 or bax2923 gp 2017 or gp2017 or halimatoz or hefiya or hulio or hyrimoz or ibi 303 or ibi303 or imraldi or m 923 or m923 or msb 11022 or msb11022 or ons3010 or ons 3010 or "pf 06410293" or pf 6410293 or pf06410293 or pf6410293 or d2e7 or truxeda or cyltezo or solymbic or fys6t7f842* or 331731 18 1 or lu 200134 or lu200134 or d2e7 or hsdb 7851 or hsdb7851 or abp-501 or abp501 or bcd-057 or bcd057 or bi-695501 or bi695501 or chs-1420 or chs1420 or gp-2017 or gp2017 or sb-5 or sb5).mp. (3403)

98 (golimumab* or cnto 148 or cnto148 or simponi or 476181 74 5 or 91x1klu43e*).mp. (751)

99 Certolizumab Pegol/ (167)

100 (certolizumab* or cdp 870 or cdp870 or cimzia or pegylated tumor necrosis factor alpha antibody fab fragment or pegylated tumour necrosis factor alpha antibody fab fragment or pha 738144 or pha738144 or czp or 428863 50 7 or g6adw90r16* or umd07x179e* or hsdb 7848 or hsdb7848).mp. (705)

101 Rituximab/ (1268)

102 (rituximab* or ct p10 or ctp10 or idec 102 or idec102 or idecc2b8 or idec c2b8 or monoclonal antibody idec c2b8 or gp2013 or gp 2013 or "pf 05280586" or pf05280586 or mabthera or mab thera or r 105 or r105 or reditux or rg 105 or rg105 or rituxan or rituxin or ritemvia or rituzena or rixathon or riximyo or ro 452294 or ro452294 or truxima or 174722 31 7 or 4f4x42syq6* or hsdb 7455 or hsdb7455).mp. (5326)

103 (ofatumumab* or humax cd20 or humax-cd20 or humaxcd20 or humax-cd20-2f2 or arzerra or gsk 1841157 or gsk1841157 or gski841157 or humax cd20 or omb 157 or omb157 or 679818 59 8 or m95kg522r0* or hsdb 8170 or hsdb8170).mp. (298)

104 Abatacept/ (280)

105 (abatacept* or ctla4 ig or ctla4 immunglobulin or ctla4 immunoglobulin g or ctla4ig or 'ctla 4 ig' or ctla-igg4m or ctla4-fc or bms 188667 or bms188667 or orencia or '332348 12 6' or 7d0yb67s97* or rg 2077 or rg2077 or rg-1046 or rg1046 or nulojix or lea29y or lea 29y or ctl4 fc or ctl4fc or belatacept or bms 224818 or bms224818 or belatacept or cytotoxic t lymphocyte associat* antigen 4).mp. (1151)

106 (tabalumab* or ly 2127399 or ly2127399 or 1143503 67 6 or pqp8vh3mjw*).mp. (70)

107 (pateclizumab* or mlta 3698a or mlta3698a or pro 283698 or pro283698 or 12202526 59 7 or qok1yyh7j2* or rg 7415 or rg7415).mp. (7)

108 (secukinumab* or cosentyx or ain 457 or ain457 or 1229022 83 6 or dlg4eml025*).mp. (917)

109 (ixekizumab* or ly 2439821 or ly2439821 or taltz or 1329632 62 3 or 1143503 69 8 or bty153760o*).mp. (489)

110 (brodalumab* or amg 827 or amg827 or khk 4827 or khk4827 or siliq or kyntheum or 1174395 19 7 or 6za31y954z*).mp. (178)

111 (guselkumab* or cnto 1959 or cnto1959 or 1350289 85 8 or tremfya or 1350289 85 8 or 089658a12d*).mp. (227)

112 Ustekinumab/ (195)

113 (ustekinumab* or cnto 1275 or cnto1275 or stelara or "815610 63 0" or fu77b4u5z0* or 1275 cnto or 1275cnto or l04ac05 or tt-20 or tt20).mp. (897)

114 (mavrilimumab or "1085337 57 0" or 1158jdp9a* or cam 3001 or cam3001).mp. (53)

115 exp Antirheumatic Agents/ (34893)

116 (dmard* or csdmard* or cs-dmard* or sdmard* or s-dmard* or tsdmard* or ts-dmard* or bdmard* or b-dmard*).mp. (3147)

117 (disease modifying adj3 (antirheum* or anti rheum*) adj3 (drug? or agent?)).mp. (2123)

118 ((antirheum* or anti rheum*) adj5 (drug? or agent?)).mp. (4248)

119 Methotrexate/ (4137)

120 (mxt or methotrexat* or metotrexat* or methoblastin or methylaminopterin* or amet?opterin* or abitrexate or antifolan or brimexate or a-met?opterin* or a-methpterin* or ai3-25299 or ai325299 or alpha-methopterin or biotrexate or emtexate or emtrexate* or emthexat* or enthexate* or farmitrexat* or farmotrex or fauldexato or folex or folex pfs or hdmtx or ifamet or imeth or maxtrex or metotressat* or methotrate* or methohexate* or mexate* or metrotex or metatrexato* or metex or metrex or metatrexan or maxtrex or meticil or metoject or metotrexin or novatrex* or neotrexate* or nordimet or lumexon or ledertrexate* or lanterel or rasuvo or r?eumatrex* or reditrex or rasuvo or texate or texate-t or tremetex or trexeron or trixilem or texorate or trexall or xaken or xatmep or otrexup or mpi-2505 or mpi2505 or mpi 5004 or mpi5004 or amethopterin* or 133073-73-1 or 15475-56-6 or 3ig1e710zn* or yl5fz2y5u1* or cl 14377 or cl14377 or ccris 1109 or ccris1109 or emt 25299 or emt25299 or nsc-740 or nsc740 or r-9985 or r9885 or x-133 or wr-19039 or wr19039).mp. (12111)

121 Leflunomide/ (153)

122 (leflunomid* or hwa 486 or hwa486 or su101 or su 101 or arava or arabloc or hsdb 7289 or hsdb7289 or g162gk9u4w* or 75706-12-6 or rs 34821 or rs34821).mp. (688)

123 lef.ti,ot,ab. (173)

124 Sulfasalazine/ (469)

125 (sulfasalazin* or sasp or ssz or salicylazosulfapyridin* or salicyl azo sulfapyridin* or sulphasalazin* or salazosulfapyridin* or salazosulfapiridin* or 'pyralin en' or azulfadin* or azulfidin* or asulfidin* or azulfide* or azulfin or colo-pleon or colopleon or disalazin or gastropyrin or pleon or "pleon ra" or "pyralin en" or rorasul or rosulfant or ulcol or ucine or salazopyrin* or ratio-sulfosalazin* or sulfasalazin* or 3xc8guz6cb* or accucol or asulfidin* or azopyrin* or azosulfidin* or benzosulfa or "brn 0356241" or brn0356241 or ccris 4713 or ccris4713 or einecs 209-974-3 or hsdb 3395 or hsdb3395 or nsc 203730 or nsc203730 or nsc 667219 or nsc667219 or reupirin or rorasul or salazo-sulfapyridin* or salazosulfpyridin* or salazopiridazin* or salazopyridin* or salazopirin* or salazodin or salisulf or salipyr or saridine or si-88 or si88 or sulculon or sulfasalazin* or sulphasalazin* or w-t sasp oral or 599-79-1 or "s.a.s. 500" or "s.a.s.-500" or sas-500 or zopyrin*).mp. (1596)

126 Hydroxychloroquine/ (505)

127 (hydroxychloroquin* or hcq or 747-3-4 or 8q2869cnvh* or 118-42-3 or 4qwg6n8qkh* or hydroxychlorochin* or hydrochloroquin* or hydrocloroquin* or oxychlorochin or oxychloroquin* or plaquenil or plaquinol or "brn 0253894" or brn0253894 or einecs 204-249-8 or einecs204-249-8 or hidroxicloroquin* or idrossiclorochin* or oxichlorochin* or oxichloroquin* or oxiklorin or win1258 or win 1258 or z0188 or einecs 212-019-3 or einecs212-019-3 or chloroquinol or dimard or ercoquin or evoquin or erquin or geniquin or quensyl or sn 8137 or sn8137 or toremonil or yuma).mp. (1666)

128 Gold Sodium Thiomalate/ (91)

129 (gold sodium thiomalate* or aurothiomalate* or aurolate* or gold thiomalate* or gold disodium thiomalate* or gold thiomalic acid* or miocrin or miocrisin or monogold disodium thiomalate* or myochrysin* or myocrisin* or myocrysin* or sodium aurothiomalate* or sodium gold thiomalate* or sodium thiomalate* gold or sodium thiomalatoaurate* or tauredon or taureodon or tauredone or thiomalate gold or thiomalatoaurate sodium or 1244-57-4 or 39377-38-3 or 4846-27-9 or 74916-57-7 or e4768zy6gm* or aurothiomala-natrium or aurot?iomalato sodico or monogold* sodium salt or dinatrium 2-aurothio-succinat or einecs 235-479-7 or einecs235-479-7 or hsdb 7173 or hsdb7173 or kidon or natrii aurothiomalas* or shiosol or gold mercatpsuccinate*).mp. (156)

130 Aurothioglucose/ (36)

131 (thioglucosoaurate* or gold thioglucose* or gold thio glucose* or thioglucose gold or aureotan or solganal or solganol or gold-50 or gold50 or solganal b or b oleosum solganal or auromyose* or aurothioglucose* or auro thioglucose* or aurotan or aurumine or aurumin or authron or brenol or ccris 59 or ccris59

or einecs 235-365-7 or einecs235-365-7 or glysanol b or goldthioglucose* or goldtioglucose* or hsdb 7174 or hsdb7174 or oronol or romosol or skf 10056 or skf10056 or 2p2v9q0e78* or 12192-57-3).mp. (49)

132 ((gold* or auro*) adj10 (inject* or intramuscular* or im or intra muscular*)).ti,ot,ab. (210)

133 Organogold Compounds/ (15)

134 (aurothiopropanol* or allochrysine or allocrisine or aurotiopoe or aurotioprol or 27279-43-2 or g7097j63e9* or auromercapto propanol sulfonate sodium or auromercaptopropanolsulfonate sodium or aurothioisopropanolsulfonate sodium or sodium aurothiopropanolsulfonate or gold sodium thiopropanol sulfonate or sodium auromercaptopropanol sulfonate).mp. (3)

135 Chloroquine/ (709)

136 (chloroquin* or cq or 54-04-7 or 886u3h6uff* or aralen or aralene or arechin or arechine or arequin or chingamin or chlorochin* or khingamin or nivaquine or oe48649kh6n* or amokin or amokine or anaclor or aralan or arequine or artriquine or avloclor or avoclor or arthrochin or artrichin or bemaco or bemaphata or bemaphate or bemasulph or benaquin or bipiquin or cadiquin or "brn 0482809" or brn0482809 or capquin or ccris 3439 or ccris3439 or chemochin or chemochine or chingamine or chingaminum or chloraquin* or chlorquin or chlorquine or choloquine or choroquine sulfate or choroquine sulphate or cindacin or clo-kit junior or clorichina or clorichine or cloriquine or clorochina or delagil or delagyl or diclokin or chlorofoz or cindachin or clorochina or cloroquina or cocatrit or dichinalex or diquinalex or diroquine or emquin or genocin or gontochin or gontochine or gontoquine or einecs 200-191-2 or einecs200-191-2 or elestol or gontochin or heliopar or hsdb 3029 or hsdb3029 or imagon or iroquine or klorokin or klorokine or klorokinfosfat or lagaquin or lapaquin or malaquin or malaren or malarex or malarivon or malaviron or maliaquine or maquine or mesylith or mexaquin or mirquin or neochin or nivaquin or nivachine or nivaquine b or nivaquine dp or nivaquine forte or "p roquine" or nsc 187208 or nsc187208 or pfizerquine or quensyl or quinachlor or quinercyl or quingamine or quinilon or quinoline or quinoscan or repal or resochen or resochene or resochin or resochin junior or resochina or resochine or resochinon or resoquina or resoquine or reumachlor or ronaquine or roquine or rp 3377 or rp3377 or sanoquin or sanoquine or silbesan or siragan or sirajan or sn 7618 or sn7618 or solprina or solprine or sopaquin or st 21 or tresochin or tresoquine or trochin or trochine or troquine or weimerquin or w 7618 or w7618 or win 244 or win244).mp. (2086)

137 exp Adrenal Cortex Hormones/ (27587)

138 exp Prednisolone/ (4835)

139 Prednisone/ (3978)

140 Cortisone/ (148)

141 Hydrocortisone/ (5944)

142 exp Steroids/ (53553)

143 adrenal cortex hormones.mp. (2224)

144 prednisolone*.mp. (7414)

145 methylprednisolone*.mp. (5485)

146 (prednisolone* or predonine or di-adreson-f or diadresonf or 9phq9y1olm* or prednisolona* or prednisolonum* or nsc-9120 or nsc9120 or nsc 9900 or nsc9900 or delta-dehydrocortisol* or delta-dehydrocortisone* or delta-hydrocortisone or hydroretrocortine or metacortandralone* or brn 1354103 or brn1354103 or bubbli-pred or ccris 980 or ccris980 or co-hydeltra or codelcortone or cordrol or cortalone or cotogesic or cotolone or decaprednil or decortin h or delcortol or dehydrohydrocortisone* or delta-cortef or deltacortef or delta-ef-cortelan or delta-stab or deltacortenol or deltacortril or deltahydrocortisone* or deltisilone or derpo pd or dexa-dortidelt hostacortin h or di-adreson f or dicortol or donisolone or dydeltrone or eazolin d or ec 200-021-7 or ec200-021-7 or einecs 200-021-7 or einecs200-021-7 or erbacort or erbasona or estilsona or fernisolone or fernisolone p or hostacortin h or hsdb 3385 or hsdb3385 or hydeltra or hydeltrone or hydrodeltalone or hydrodeltisone or hydroretrocortin or hydroretrocortine* or k 1557 or k1557 or lentosone or meti-drm or meticortelone or orapred or orapred odt or paracortol or paracotol or pediapred or precortancyl or precortilon or precortisyl or predne-dome or prednelan or prednicen or predniliderm or predniretard or prednis or predonin or predonine* or prelon or prelone or prenolone or rolisone or scherisolon? or solone or sterolone or ulacort or ultracorten h or ultracortene h adelcort or antisolon or antisolone or aprednisolon? or benisolon? or caberdelta or co hydeltra or codelcortone or ompresolon).mp. (7432)

147 (cortadeltona or cortelinter or cortisolone or dacortin or dacortin h or dacrotin or decaprednil or decortil or delta cortril or delta hycortol or deltacortenolo or deltacortil or deltacortoil or deltaderm or deltadglycortril or deltahycortol or deltahydrocortison? or deltaophticor or deltasolone or deltastab or deltidrosol or deltisilone or deltisolon? or deltolasson? or deltosona or deltosone or depo-predate or dermosolon or dhasolone or di adreson? f or diadreson? f or dicortol or domucortone or encortelon? or encortolon or equisolon or glistelone or hefasolon or hydrelta or hydrocortancyl or hydrocortidelt or hydradeltalone or hydradeltisone or inflanefran or insolone or keteocort h or key-pred or key-pred sp or lenisolone or leocortol or liquipred or mediasolone or meprisolon? or metacortalon? or metacortelone or meti derm or metiderm or meticortelone or morlone or mydrapred or nisolon? or opredsone or panafcortelone or panafcortolone or panafort or paracortol pr phlogex or pre cortisyl or preconin or precortalon or precortancyl or precortisyl or pred ject 50 or predacort 50 or predaject 50 or predalone 50 or predartrina or predartrine or predate 50 or predeltilone or predisole or predisyr or predne dome or prednecort or prednedome or prednelan or predni coelin or predni h tablinen or predni helvacort or prednicoelin or prednicort or prednicortelone or prednifor drops or predniment or prednorsolon? or predorgasolon? or prenilone or preventan or prezolon or rubycort or serilone or solonda or solupren? or spiricort or spolotane or supercorti#ol or taracortelone or walesolone or wysolone).mp. (23)

148 (methylprednisolone * or methylprednisolona* or methylprednisolonum* or metipred or urbason or medrol or x4w7zr7023* or 83-43-2 or besonia or brn 23403300 or brn2340300 or a-methapred or artisone-wyeth or besonia or depo-medrol or dopomedrol ec 201-4764 pr ec2014764 or einecs 201-476-4 or einecs201-476-4 or esametone or firmacort or hsdb3127 or hsdb3127 or lemod or medesone or medixon or medlone 21 or medrate or medrol or medrone or mesopren or metastab or methyleneprednisolone* or metilbetasone or metrisone or metrocort or metysolon or moderin or nirypan or noretona or nsc 19987 or nsc19987 or predni n tablinen or prednol-l or promacortine or reactenol or sieropresol or solomet or summicort or suprametil or u 7532 or u7532 or "u 67 590a" or urbason or urbasone or wyacort or adlone 40 or adlone 80 or beta methylprednisolone or medalone 80 or depmedalone or deproject 80 or depopred or esametone or firmacort or med-jec-40 or medixon or mednin or medralone or mepredprednisolone or meprelon or mesopren or methacort 40 or methacort 80 or methyxlcotol or methylcotolone or methylpred dp or methylsterolone or metidrol or metycortin or metypred or metypresol or neomedrone or solu decortin or urbason).mp. (1215)

149 (prednison or prednisone or prednisona or prednisonum or dehydrocortisone or delta cortisone or rectodelt or sterapred or ultracorten or winpred or apo prednisone or cortan or cartancyl or panafcort or cutason or decortin or dacortin or decortisyl or deltasone or encortone or encorton or enkorton or enkortolon or kortancyl or liquid pred or meticorten or orasone or panasol or predni tablinen or prednidib or predniment or pronisone or vb0r961hzt* or 53-03-2 or adasone or al3-52939 or ancortone or bicortone or cartancyl or ccris 2646 or ccrs2646 or colisone or cortan or cortidelt or cotone or dacorten or dacortin or decortancyl or dekortin or dellacort or delta cortelan or delta cortisone or delta dome or deltacortene or deltacortisone or deltacortone or deltasone or deltison or deltisona or deltra or di adreson or diadreson or econosone or einecs 200-160-3 or einecs200-160-3 or fernisone or fiasone or hostacortin or hsdb 3168 or hsdb3168 or incocortyl or juvason or lisacort or lodotra or lodtra).mp. (10119)

150 (me-korti or metacortandracin or metacorten or nci c04897 or ncic04897 or nisona or nizon or novoprednisone or nsc 10023 or nsc10023 or nurison or orasone or panafcort or paracort or prarmenison or pehacort or predeltin or prednicen-m or prednicorm or prednicort or prednicot or prednilonga or prednitone or prednizon or prednovister or presone or pronison or rayos or rectodelt or retrocortine or servisone or sterapred or supercortio or u 6020 or u6020 or ultracorten or ultracortene or winpred or wojtab or zenadrid).mp. (19)

151 cortisone?.mp. (569)

152 corticosteroid*.mp. (23083)

153 corticoid*.mp. (556)

154 glucocorticoid*.mp. (8890)

155 corticosterone*.mp. (153)

156 hydrocortisone*.mp. (9702)

157 dexamethasone*.mp. (12333)

158 beclomethasone*.mp. (2295)

159 (triamcinolone* or 1zk20vi6ty* or 124-94-7 or aristocort or volon or fluoxyprednisolon* or tiamcinolonum or triamcinolonum* or triamcinolona* or brn 2341955 or brn2341955 or cl 19823 or cl19823 or einecs 204-718-7 or einecs204-718-7 or hsdb 3194 or hsdb3194 or nsc 13397 or nsc13397).mp. (3241)

160 (steroid? adj10 (therap* or treat* or drug? or inject* or intramuscular* or intra muscular* or im or intravenous* or intra venous* or iv or intraarticular* or intra articular* or ia or administrat* or oral or orally or parenteral*)).ti,ot,ab. (12978)

161 Janus Kinase Inhibitors/ (50)

162 ((jak or jak1 or jak2 or jak3 or janus kinase) adj10 inhibitor*).mp. (1538)

163 (janus adj4 kinase adj10 inhibitor?).mp. (927)

164 exp Janus Kinases/ai [Antagonists & Inhibitors] (95)

165 (baricitinib* or 1187594-09-7 or isp4442i3y* or "incb 028050" or incb028050 or ly-3009104 or ly3009104).mp. (409)

166 (tofacitinib* or 477600-75-2 or 87la6fu830* or hsdb 8311 or hsdb8311 or cp-690 550 or cp 690550 or cp690550).mp. (825)

167 (filgotinib* or 1206161-97-8 or 3xvl385q0m* or "glpg 0634" or glpg0634).mp. (173)

168 (upadacitinib* or 1310726-60-3 or 4RA0KN46E0* or abt 494 or abt494).mp. (267)

169 (ruxolitinib* or rux or 941678-49-5 or 82s8x8xx8h* or hsdb 8259 or hsdb8259 or inc 424 or inc424 or incb424 or "incb 018424" or incb018424 or incb 18424 or incb18424 or r-ruxolitinib* or jakafi or jakavi).mp. (486)

170 (itacitinib or "incb 039110" or incb039110 or incb 39110 or incb39110 or 1334298-90-6 or 19j3781lpm*).mp. (34)

171 (ag490 or ag 490 or tyrphostin b42 or tyrphostin ag 490 or tyrphostin ag490 or 133550-30-8).mp. (1)

172 (fedratinib* or tg101348 or tg 101348 or sar302503 or sar 302503 or 936091-26-8 or 6l1xp550i6* or inrebic).mp. (29)

173 (ritlecitinib* or pf-06651600 or pf06651600 or pf 6651600 or pf6651600 or 2140301-97-7 or 132lf5wgh4* or 2192215-81-7 or eag4t1459k* or ritlecitinib malonate or ritlecitinib toslate or ritlecitinib tosylate or ritlecitinib propanedioate).mp. (24)

174 (peficitinib* or peficitinib hydrobromide).mp. (26)

175 or/90-174 (149813)

176 78 and 175 (6580)

177 89 or 176 (6684)

178 exp animals/ not humans/ (12)

179 177 not 178 (6684)

180 ((eular or (european adj4 rheumat*)) adj8 (meeting or conference or congress) adj8 annual).af. (702)

181 ((acr or (american college adj4 rheumat*)) adj8 (meeting or conference or congress) adj8 annual).af. (801)

182 180 or 181 (1502)

183 179 and 182 (294)

184 limit 183 to yr="2019 - 2020" (0)

185 limit 179 to english (4204)

186 review.pt. (3088)

187 editorial.pt. (462)

188 letter.pt. (7334)

189 congress.pt. (44)

190 conference abstract.pt. (16804)

191 conference review.pt. (173)

192 or/186-191 (27884)

193 185 not 192 (3955)

194 limit 193 to yr="2012 - 2020" (2990)

195 184 or 194 (2990)

196 from 195 keep 1-2000 (2000)

197 from 195 keep 2001-2990 (990)

1.3.3 Safety: approved indications

1.3.3.1 Medline

1 Arthritis, Rheumatoid/ (100354)

2 ((rheumat* or reumat* or revmat* or rheumo or reumo) adj3 (arthrit* or artrit* or polyarthrit* or polyartrit*)).ti,ot,ab,kw,kf. (116281)

3 (r?eumarthrit* or r?eumartrit* or r?eum arthrit* or r?eum artrit* or r?evmarthrit* or r?evmartrit* or r?evm arthrit* or r?evm artrit* or r?eum polyarthrit* or r?eum polyartrit* or r?eum poly arthrit* or r?eum poly artrit* or r?eumpolyarthrit* or r?eumpolyartrit*).ti,ot,ab,kf,kw. (5)

4 (chronic* adj2 (polyarthrit* or poly arthrit* or polyartrit* or poly artrit*)).ti,ot,ab,kf,kw. (2455)

5 (inflammator* adj2 (arthrit* or artrit*)).ti,ot,ab,kf,kw. (6818)

6 ((r?eum* or r?evm*) adj2 (chronic* adj2 articular*)).ti,ot,ab,kf,kw. (42)

7 ((rheumat* or reumat* or revmat* or rheumo or reumo) adj2 (condition* or diseas*)).ti,ot,ab,kf,kw. (33754)

8 Arthritis, Juvenile/ (10532)

9 ((arthrit* or artrit*) adj6 juvenile*).ti,ab,kf,kw. (10710)

10 JIA.ti,ot,ab,kf,kw. (4121)

11 8 or 9 or 10 (14166)

12 (systemic or polyart* or poly art* or oligoart* or oligo art*).ti,ab,kf,kw,hw. (535563)

13 11 and 12 (4826)

14 ((polyarthr* or polyartrit* or poly arthrit* or poly artrit* or oligoarthrit* or oligoartrit* or oligo arthrit* or oligo artrit*) adj6 juvenile*).ti,ab,kf,kw. (233)

15 (((stills or still's or still) adj disease) and juvenile*).ti,ab,kf,kw. (309)

16 ((stills or still's or still) adj disease adj6 child*).ti,ab,kf,kw. (59)

17 ((systemic adj6 JIA) or sJIA or s-JIA).ti,ab,kf. (869)

18 (((polyart* or oligoart* or poly art* or oligo art*) and JIA) or pJIA).ti,ab,kf. (1057)

19 Still's Disease, Adult-Onset/ (1384)

20 ((stills or still's or still) adj disease adj6 adult*).ti,ab,kf,kw. (1888)

21 Giant Cell Arteritis/ (6552)

22 (giant cell adj6 (arterit* or aortit* or horton*)).ti,ab,kf. (5211)

23 ((temporal or cranial) adj2 arterit*).ti,ab,kf. (2432)

24 ((horton or horton's or hortons) adj3 disease?).ti,ab,kf. (490)

25 (large vessel adj3 (vasculit* or arterit*)).ti,ab,kf. (1085)

26 GCA.ti,ab,kf. and (arterit* or vasculit* or rheum*).mp,jw. (1868)

27 Takayasu Arteritis/ (4029)

28 (takayasu* adj3 (arterit* or syndrom* or disease*)).ti,ab,kf. (4468)

29 (young female adj6 arterit*).ti,ab,kf. (21)

30 (brachiocephal* adj6 arterit*).ti,ab,kf. (21)

31 Cytokine Release Syndrome/ (572)

32 ((cytokine releas* or cytokine storm) adj3 syndrome*).ti,ab,kf. (1495)

33 crs.ti,ab,kf. and (cytokine releas* or cytokine storm).mp. [mp=title, abstract, original title, name of substance word, subject heading word, floating sub-heading word, keyword heading word, organism supplementary concept word, protocol supplementary concept word, rare disease supplementary concept word, unique identifier, synonyms] (450)

34 ((chronic sinusitis or chronic rhinosinusitis or chronic rhino sinusitis or cytoreductive surg* or complete response or complete responders) adj6 crs).ti,ab,kf. (4201)

35 33 not 34 (448)

36 Castleman Disease/ (2615)

37 ((angiofollicul* or angio follicul*) adj3 lymph* adj3 hyperplas*).ti,ab,kf. (283)

38 (lymph* node adj3 hyperplas* adj3 giant).ti,ab,kf. (216)

39 (castleman* adj3 (disease* or tumo?r* or pseudo-tumo?r* or pseudotumo?r* or lymph* or pseudolymphom* or hyperplas*)).ti,ab,kf,kw. (3643)

40 1 or 2 or 3 or 4 or 5 or 6 or 7 or 13 or 14 or 15 or 16 or 17 or 18 or 19 or 20 or 21 or 22 or 23 or 24 or 25 or 26 or 27 or 28 or 29 or 30 or 31 or 32 or 35 or 36 or 37 or 38 or 39 (186239)

41 (tocilizumab* or actemra* or atlizumab* or lusinex* or r1569 or r 1569 or roactemra* or mra or 375823 41 9 or i031v2h011* or rhpm-1 or rhpm1 or rg-1569 or rg1569 or msb-11456 or msb11456 or ro-4877533 or ro4877533).mp,rn. (12726)

42 (sarilumab* or kevzara* or regn 88 or regn88 or sar 153191 or sar153191 or 1189541 98 7 or nu90v55f8i*).mp,rn. (178)

43 (levilimab* or BCD-089 or bcd089 or 2035008 70 7 or P7UV3L2H80*).mp,rn. (0)

44 (olokizumab* or cdp 6038 or cdp6038 or 1007223 17 7 or pai71r1d2w*).mp,rn. (18)

45 (clazakizumab* or ald 518 or ald518 or bms 945429 or bms945429 or 1236278 28 6 or 4s38z8ra9o*).mp,rn. (29)

46 (sirukumab* or cnto 136 or cnto136 or pilvensia or 1194585 53 9 or 640443FU93*).mp,rn. (55)

47 (siltuximab* or sylvant or cnto 328 or cnto-328 or cnto328 or cllb8 or ccib8 monoclonal antibody or sylvant or t4h8fma7im* or 541502 14 1).mp,rn. (184)

48 (ziltivekimab* or zilti or "cor-001" or "2226654 05 1").mp,rn. (3)

49 (elsilimomab* or "b-e8" or 468715 71 1).mp,rn. (12)

50 41 or 42 or 43 or 44 or 45 or 46 or 47 or 48 or 49 (13014)

51 40 and 50 (2882)

52 Interleukin 1 Receptor Antagonist Protein/ (5241)

53 (anakinra* or kineret or ((interleukin 1 receptor or il1 receptor or il 1 receptor) adj1 antagonist protein*) or (recombinant interleukin 1 receptor adj1 (antagonist* or blocker* or blocking agent*)) or antril or il 1ra or il1ra or il 1 ra or ((urin* or febrile) adj2 (interleukin 1 inhibitor* or il1 inhibitor* or il 1 inhibitor*)) or "143090 92 0" or 9013duq28k*).mp,rn. (9674)

54 Infliximab/ (10432)

55 (infliximab* or ifx or avakine or flixabi or inflectra or inflecta or ixifi or remicade or remsima or revellex or renflexis or zessly or mab ca2 or monoclonal antibody ca2 or antibody ca2 monoclonal or ca2 monoclonal antibody or ct p13 or ctp13 or ct-p-13 gp-1111 or gp1111 or ta-650 or ta650 or abp 710 or abp710 or bow-015 or bow015 or 170277 31 3 or b72hh48flu* or sb2 or "pf 06438179" or pf 6438179 or pf06438179 or pf6438179 or hsdb 7850 or hsdb7850).mp,rn. (15967)

56 Etanercept/ (5917)

57 (etanercept* or benepali or embrel or enbrel or enia 11 or enia11 or erelzi or lifmior or opinercept or recombinant tumo?r necrosis factor receptor fc fusion protein or "tnr 001" or tnr001 or tnfr-fc or tnrfr:fc or rhu tnfr:fc or rhu-tnfr:fc or tnfr-immunoadhesin or tnt receptor fusion protein or tum?or necrosis factor receptor fc fusion protein or tunex or "185243 69 0" or op401g7ojc* or brenzys or tnf receptor fusion protein or tnfr fc or recombinant human tnf or recombinant human dimeric tnf receptor type ii igg fusion protein or chs-0214 or chs0214 or dwp-422 or dwp422 or enia-11 or enia11 or gp-2015 or gp2015 or gp2015c or gp-2015c or hd-203 or hd203 or lbec-0101 or lbec0101 or sb-4 or sb4).mp,rn. (9522)

58 Adalimumab/ (5507)

59 (adalimumab* or humira or abp 501 or abp501 or abtd2e7 or amjevita or amgevita or bax 2923 or bax2923 gp 2017 or gp2017 or halimatoz or hefiya or hulio or hyrimoz or ibi 303 or ibi303 or imraldi or m 923 or m923 or msb 11022 or msb11022 or ons3010 or ons 3010 or "pf 06410293" or pf 6410293 or pf06410293 or pf6410293 or d2e7 or truxeda or cyltezo or solymbic or fys6t7f842* or 331731 18 1 or lu 200134 or lu200134 or d2e7 or hsdb 7851 or hsdb7851 or abp-501 or abp501 or bcd-057 or bcd057 or bi-695501 or bi695501 or chs-1420 or chs1420 or gp-2017 or gp2017 or sb-5 or sb5).mp,rn. (9187)

60 (golimumab* or cnto 148 or cnto148 or simponi or 476181 74 5 or 91x1klu43e*).mp,rn. (1328)

61 Certolizumab Pegol/ (619)

62 (certolizumab* or cdp 870 or cdp870 or cimzia or pegylated tumor necrosis factor alpha antibody fab fragment or pegylated tumour necrosis factor alpha antibody fab fragment or pha 738144 or pha738144 or czp or 428863 50 7 or g6adw90r16* or umd07x179e* or hsdb 7848 or hsdb7848).mp,rn. (1575)

63 Rituximab/ (15105)

64 (rituximab* or ct p10 or ctp10 or idec 102 or idec102 or idecc2b8 or idec c2b8 or monoclonal antibody idec c2b8 or gp2013 or gp 2013 or "pf 05280586" or pf05280586 or mabthera or mab thera or r 105 or r105 or reditux or rg 105 or rg105 or rituxan or rituxin or ritemvia or rituzena or rixathon or riximyo or ro 452294 or ro452294 or truxima or 174722 31 7 or 4f4x42syq6* or hsdb 7455 or hsdb7455).mp,rn. (25091)

65 (ofatumumab* or humax cd20 or humax-cd20 or humaxcd20 or humax-cd20-2f2 or arzerra or gsk 1841157 or gsk1841157 or gski841157 or human cd20 or omb 157 or omb157 or 679818 59 8 or m95kg522r0* or hsdb 8170 or hsdb8170).mp,rn. (775)

66 Abatacept/ (2952)

67 (abatacept* or ctla4 ig or ctla4 immunglobulin or ctla4 immunoglobulin g or ctla4ig or 'ctla 4 ig' or ctla-igg4m or ctla4-fc or bms 188667 or bms188667 or orencia or '332348 12 6' or 7d0yb67s97* or rg 2077 or rg2077 or rg-1046 or rg1046 or nulojix or lea29y or lea 29y or ctl4 fc or ctl4fc or belatacept or bms 224818 or bms224818 or belatacept or cytotoxic t lymphocyte associat* antigen 4).mp,rn. (5479)

68 (tabalumab* or ly 2127399 or ly2127399 or 1143503 67 6 or pqp8vh3mjw*).mp,rn. (58)

69 (pateclizumab* or mlta 3698a or mlta3698a or pro 283698 or pro283698 or 12202526 59 7 or qok1yyh7j2* or rg 7415 or rg7415).mp,rn. (6)

70 (secukinumab* or cosentyx or ain 457 or ain457 or 1229022 83 6 or dlg4eml025*).mp,rn. (1263)

71 (ixekizumab* or ly 2439821 or ly2439821 or taltz or 1329632 62 3 or 1143503 69 8 or bty153760o*).mp,rn. (627)

72 (brodalumab* or amg 827 or amg827 or khk 4827 or khk4827 or siliq or kyntheum or 1174395 19 7 or 6za31y954z*).mp,rn. (362)

73 (guselkumab* or cnto 1959 or cnto1959 or 1350289 85 8 or tremfya or 1350289 85 8 or 089658a12d*).mp,rn. (278)

74 Ustekinumab/ (1121)

75 (ustekinumab* or cnto 1275 or cnto1275 or stelara or "815610 63 0" or fu77b4u5z0* or 1275 cnto or 1275cnto or l04ac05 or tt-20 or tt20).mp,rn. (2230)

76 (mavrilimumab or "1085337 57 0" or 1158jdp9a* or cam 3001 or cam3001).mp,rn. (33)

77 exp Antirheumatic Agents/ (432001)

78 (dmard* or csdmard* or cs-dmard* or sdmard* or s-dmard* or tsdmard* or ts-dmard* or bdmard* or b-dmard*).ti,ab,kf. (5877)

79 (disease modifying adj3 (antirheum* or anti rheum*) adj3 (drug? or agent?)).ti,ab,kf. (6499)

80 ((antirheum* or anti rheum*) adj5 (drug? or agent?)).ti,ab,kf. (9124)

81 Methotrexate/ (38291)

82 (mxt or methotrexat* or metotrexat* or methoblastin or methylaminopterin* or amet?opterin* or abitrexate or antifolan or brimexate or a-met?opterin* or a-methpterin* or ai3-25299 or ai325299 or alpha-methopterin or biotrexate or emtexate or emtrexate* or emthexat* or enthexate* or farmitrexat* or farmotrex or fauldexato or folex or folex pfs or hdmtx or ifamet or imeth or maxtrex or metotressat* or methotrate* or methohexate* or mexate* or metrotex or metatrexato* or metex or metrex or metatrexan or maxtrex or meticil or metoject or metotrexin or novatrex* or neotrexate* or nordimet or lumexon or ledertrexate* or lanterel or rasuvo or r?eumatrex* or reditrex or rasuvo or texate or texate-t or tremetex or trexeron or trixilem or texorate or trexall or xaken or xatmep or otrexup or mpi-2505 or mpi2505 or mpi 5004 or mpi5004 or amethopterin* or 133073-73-1 or 15475-56-6 or 3ig1e710zn* or yl5fz2y5u1* or cl 14377 or cl14377 or ccris 1109 or ccris1109 or emt 25299 or emt25299 or nsc-740 or nsc740 or r-9985 or r9885 or x-133 or wr-19039 or wr19039).mp,rn. (55797)

83 Leflunomide/ (1536)

84 (leflunomid* or hwa 486 or hwa486 or su101 or su 101 or arava or arabloc or hsdb 7289 or hsdb7289 or g162gk9u4w* or 75706-12-6 or rs 34821 or rs34821).mp,rn. (2626)

85 lef.ti,ab,kf. (2564)

86 Sulfasalazine/ (4144)

87 (sulfasalazin* or sasp or ssz or salicylazosulfapyridin* or salicyl azo sulfapyridin* or sulphasalazin* or salazosulfapyridin* or salazosulfapiridin* or 'pyralin en' or azulfadin* or azulfidin* or asulfidin* or azulfide* or azulfin or colo-pleon or colopleon or disalazin or gastropyrin or pleon or "pleon ra" or "pyralin en" or rorasul or rosulfant or ulcol or ucine or salazopyrin* or ratio-sulfosalazin* or sulfasalazin* or 3xc8guz6cb* or accucol or asulfidin* or azopyrin* or azosulfidin* or benzosulfa or "brn 0356241" or brn0356241 or ccris 4713 or ccris4713 or einecs 209-974-3 or hsdb 3395 or hsdb3395 or nsc 203730 or nsc203730 or nsc 667219 or nsc667219 or reupirin or rorasul or salazo-sulfapyridin* or salazosulfpyridin* or salazopiridazin* or salazopyridin* or salazopirin* or salazodin or salisulf or salipyr or saridine or si-88 or si88 or sulculon or sulfasalazin* or sulfasalizin* or sulphasalazin* or w-t sasp oral or 599-79-1 or "s.a.s. 500" or "s.a.s.-500" or sas-500 or zopyrin*).mp,rn. (8109)

88 Hydroxychloroquine/ (4080)

89 (hydroxychloroquin* or hcq or 747-3-4 or 8q2869cnvh* or 118-42-3 or 4qwg6n8qkh* or hydroxychlorochin* or hydrochloroquin* or hydrocloroquin* or oxychlorochin or oxychloroquin* or plaquenil or plaquinol or "brn 0253894" or brn0253894 or einecs 204-249-8 or einecs204-249-8 or hidroxicloroquin* or idrossiclorochin* or oxichlorochin* or oxichloroquin* or oxiklorin or win1258 or win 1258 or z0188 or einecs 212-019-3 or einecs212-019-3 or chloroquinol or dimard or ercoquin or evoquin or erquin or geniquin or quensyl or sn 8137 or sn8137 or toremonil or yuma).mp,rn. (7797)

90 Gold Sodium Thiomalate/ (1262)

91 (gold sodium thiomalate* or aurothiomalate* or aurolate* or gold thiomalate* or gold disodium thiomalate* or gold thiomalic acid* or miocrin or miocrisin or monogold disodium thiomalate* or myochrysin* or myocrisin* or myocrysin* or sodium aurothiomalate* or sodium gold thiomalate* or sodium thiomalate* gold or sodium thiomalatoaurate* or tauredon or taureodon or tauredone or thiomalate gold or thiomalatoaurate sodium or 1244-57-4 or 39377-38-3 or 4846-27-9 or 74916-57-7 or e4768zy6gm* or aurothiomala-natrium or aurot?iomalato sodico or monogold* sodium salt or dinatrium 2-aurothio-succinat or einecs 235-479-7 or einecs235-479-7 or hsdb 7173 or hsdb7173 or kidon or natrii aurothiomalas* or shiosol or gold mercatpsuccinate*).mp,rn. (1547)

92 Aurothioglucose/ (834)

93 (thioglucosoaurate* or gold thioglucose* or gold thio glucose* or thioglucose gold or aureotan or solganal or solganol or gold-50 or gold50 or solganal b or b oleosum solganal or auromyose* or aurothioglucose* or auro thioglucose* or aurotan or aurumine or aurumin or authron or brenol or ccris 59 or ccris59 or einecs 235-365-7 or einecs235-365-7 or glysanol b or goldthioglucose* or goldtioglucose* or hsdb 7174 or hsdb7174 or oronol or romosol or skf 10056 or skf10056 or 2p2v9q0e78* or 12192-57-3).mp,rn. (1133)

94 ((gold* or auro*) adj10 (inject* or intramuscular* or im or intra muscular*)).ti,ab,kf,kw. (2766)

95 Organogold Compounds/ (941)

96 (aurothiopropanol* or allochrysine or allocrisine or aurotiopoe or aurotioprol or 27279-43-2 or g7097j63e9* or auromercapto propanol sulfonate sodium or auromercaptopropanolsulfonate sodium or aurothioisopropanolsulfonate sodium or sodium aurothiopropanolsulfonate or gold sodium thiopropanol sulfonate or sodium auromercaptopropanol sulfonate).mp,rn. (91)

97 Chloroquine/ (14620)

98 (chloroquin* or cq or 54-04-7 or 886u3h6uff* or aralen or aralene or arechin or arechine or arequin or chingamin or chlorochin* or khingamin or nivaquine or oe48649kh6n* or amokin or amokine or anaclor or aralan or arequine or artriquine or avloclor or avoclor or arthrochin or artrichin or bemaco or bemaphata or bemaphate or bemasulph or benaquin or bipiquin or cadiquin or "brn 0482809" or brn0482809 or capquin or ccris 3439 or ccris3439 or chemochin or chemochine or chingamine or chingaminum or chloraquin* or chlorquin or chlorquine or choloquine or choroquine sulfate or choroquine

sulphate or cindacin or clo-kit junior or clorichina or clorichine or cloriquine or clorochina or delagil or delagyl or diclokin or chlorofoz or cindachin or clorochina or cloroquina or cocatrit or dichinalex or diquinalex or diroquine or emquin or genocin or gontochin or gontochine or gontoquine or einecs 200-191-2 or einecs200-191-2 or elestol or gontochin or heliopar or hsdb 3029 or hsdb3029 or imagon or iroquine or klorokin or klorokine or klorokinfosfat or lagaquin or lapaquin or malaquin or malaren or malarex or malarivon or malaviron or maliaquine or maquine or mesylith or mexaquin or mirquin or neochin or nivaquin or nivachine or nivaquine b or nivaquine dp or nivaquine forte or "p roquine" or nsc 187208 or nsc187208 or pfizerquine or quensyl or quinachlor or quinercyl or quingamine or quinilon or quinoline or quinoscan or repal or resochen or resochene or resochin or resochin junior or resochina or resochine or resochinon or resoquina or resoquine or reumachlor or ronaquine or roquine or rp 3377 or rp3377 or sanoquin or sanoquine or silbesan or siragan or sirajan or sn 7618 or sn7618 or solprina or solprine or sopaquin or st 21 or tresochin or tresoquine or trochin or trochine or troquine or weimerquin or w 7618 or w7618 or win 244 or win244).mp,rn. (35229)

99 exp Adrenal Cortex Hormones/ (399791)

100 exp Prednisolone/ (51515)

101 Prednisone/ (39649)

102 Cortisone/ (19618)

103 Hydrocortisone/ (72496)

104 exp Steroids/ (862957)

105 adrenal cortex hormones.ti,ab,kf,rn. (65503)

106 prednisolone*.mp,rn. (46716)

107 methylprednisolone*.mp,rn. (27162)

108 (prednisolone* or predonine or di-adreson-f or diadresonf or 9phq9y1olm* or prednisolona* or prednisolonum* or nsc-9120 or nsc9120 or nsc 9900 or nsc9900 or delta-dehydrocortisol* or delta-dehydrocortisone* or delta-hydrocortisone or hydroretrocortine or metacortandralone* or brn 1354103 or brn1354103 or bubbli-pred or ccris 980 or ccris980 or co-hydeltra or codelcortone or cordrol or cortalone or cotogesic or cotolone or decaprednil or decortin h or delcortol or dehydrohydrocortisone* or delta-cortef or deltacortef or delta-ef-cortelan or delta-stab or deltacortenol or deltacortril or deltahydrocortisone* or deltisilone or derpo pd or dexa-dortidelt hostacortin h or di-adreson f or dicortol or donisolone or dydeltrone or eazolin d or ec 200-021-7 or ec200-021-7 or einecs 200-021-7 or einecs200-021-7 or erbacort or erbasona or estilsona or fernisolone or fernisolone p or hostacortin h or hsdb 3385 or hsdb3385 or hydeltra or hydeltrone or hydrodeltalone or hydrodeltisone or hydroretrocortin or hydroretrocortine* or k 1557 or k1557 or lentosone or meti-drm or meticortelone or

orapred or orapred odt or paracortol or paracotol or pediapred or precortancyl or precortilon or precortisyl or predne-dome or prednelan or prednicen or predniliderm or predniretard or prednis or predonin or predonine* or prelon or prelone or prenolone or rolisone or scherisolon? or solone or sterolone or ulacort or ultracorten h or ultracortene h adelcort or antisolon or antisolone or aprednisolon? or benisolon? or caberdelta or co hydeltra or codelcortone or ompresolon).mp,rn. (46804)

109 (cortadeltona or cortelinter or cortisolone or dacortin or dacortin h or dacrotin or decaprednil or decortil or delta cortril or delta hycortol or deltacortenolo or deltacortil or deltacortoil or deltaderm or deltadglycortril or deltahycortol or deltahydrocortison? or deltaophticor or deltasolone or deltastab or deltidrosol or deltisilone or deltisolon? or deltolasson? or deltosona or deltosone or depo-predate or dermosolon or dhasolone or di adreson? f or diadreson? f or dicortol or domucortone or encortelon? or encortolon or equisolon or glistelone or hefasolon or hydrelta or hydrocortancyl or hydrocortidelt or hydradeltalone or hydradeltisone or inflanefran or insolone or keteocort h or key-pred or key-pred sp or lenisolone or leocortol or liquipred or mediasolone or meprisolon? or metacortalon? or metacortelone or meti derm or metiderm or meticortelone or morlone or mydrapred or nisolon? or opredsone or panafcortelone or panafcortolone or panafort or paracortol pr phlogex or pre cortisyl or preconin or precortalon or precortancyl or precortisyl or pred ject 50 or predacort 50 or predaject 50 or predalone 50 or predartrina or predartrine or predate 50 or predeltilone or predisole or predisyr or predne dome or prednecort or prednedome or prednelan or predni coelin or predni h tablinen or predni helvacort or prednicoelin or prednicort or prednicortelone or prednifor drops or predniment or prednorsolon? or predorgasolon? or prenilone or preventan or prezolon or rubycort or serilone or solonda or solupren? or spiricort or spolotane or supercorti#ol or taracortelone or walesolone or wysolone).mp,rn. (63)

110 (methylprednisolone * or methylprednisolona* or methylprednisolonum* or metipred or urbason or medrol or x4w7zr7023* or 83-43-2 or besonia or brn 23403300 or brn2340300 or a-methapred or artisone-wyeth or besonia or depo-medrol or dopomedrol ec 201-4764 pr ec2014764 or einecs 201-476-4 or einecs201-476-4 or esametone or firmacort or hsdb3127 or hsdb3127 or lemod or medesone or medixon or medlone 21 or medrate or medrol or medrone or mesopren or metastab or methyleneprednisolone* or metilbetasone or metrisone or metrocort or metysolon or moderin or nirypan or noretona or nsc 19987 or nsc19987 or predni n tablinen or prednol-l or promacortine or reactenol or sieropresol or solomet or summicort or suprametil or u 7532 or u7532 or "u 67 590a" or urbason or urbasone or wyacort or adlone 40 or adlone 80 or beta methylprednisolone or medalone 80 or depmedalone or deproject 80 or depopred or esametone or firmacort or med-jec-40 or medixon or mednin or medralone or mepredprednisolone or meprelon or mesopren or methacort 40 or methacort 80 or methyxlcotol or methylcotolone or methylpred dp or methylsterolone or metidrol or metycortin or metypred or metypresol or neomedrone or solu decortin or urbason).mp,rn. (19855)

111 (prednison or prednisone or prednisona or prednisonum or dehydrocortisone or delta cortisone or rectodelt or sterapred or ultracorten or winpred or apo prednisone or cortan or cartancyl or panafcort or cutason or decortin or dacortin or decortisyl or deltasone or encortone or encorton or enkorton or enkortolon or kortancyl or liquid pred or meticorten or orasone or panasol or predni tablinen or prednidib or predniment or pronisone or vb0r961hzt* or 53-03-2 or adasone or al3-52939 or ancortone or bicortone or cartancyl or ccris 2646 or ccrs2646 or colisone or cortan or cortidelt or cotone or dacorten or dacortin or decortancyl or dekortin or dellacort or delta cortelan or delta cortisone or delta dome or deltacortene or deltacortisone or deltacortone or

deltasone or deltison or deltisona or deltra or di adreson or diadreson or econosone or einecs 200-160-3 or einecs200-160-3 or fernisone or fiasone or hostacortin or hsdb 3168 or hsdb3168 or incocortyl or juvason or lisacort or lodotra or lodtra).mp,rn. (54226)

112 (me-korti or metacortandracin or metacorten or nci c04897 or ncic04897 or nisona or nizon or novoprednisone or nsc 10023 or nsc10023 or nurison or orasone or panafcort or paracort or prarmenison or pehacort or predeltin or prednicen-m or prednicorm or prednicort or prednicot or prednilonga or prednitone or prednizon or prednovister or presone or pronison or rayos or rectodelt or retrocortine or servisone or sterapred or supercortio or u 6020 or u6020 or ultracorten or ultracortene or winpred or wojtab or zenadrid).mp,rn. (356)

113 cortisone?.mp,rn. (23574)

114 corticosteroid*.mp,rn. (106931)

115 corticoid*.mp,rn. (6605)

116 glucocorticoid*.mp,rn. (114710)

117 corticosterone*.mp,rn. (34899)

118 hydrocortisone*.mp,rn. (77985)

119 dexamethasone*.mp,rn. (73124)

120 beclomethasone*.mp,rn. (3845)

121 (triamcinolone* or 1zk20vi6ty* or 124-94-7 or aristocort or volon or fluoxyprednisolon* or tiamcinolonum or triamcinolonum* or triamcinolona* or brn 2341955 or brn2341955 or cl 19823 or cl19823 or einecs 204-718-7 or einecs204-718-7 or hsdb 3194 or hsdb3194 or nsc 13397 or nsc13397).mp,rn. (12000)

122 (steroid? adj10 (therap* or treat* or drug? or inject* or intramuscular* or intra muscular* or im or intravenous* or intra venous* or iv or intraarticular* or intra articular* or ia or administrat* or oral or orally or parenteral*)).ti,ab,kf. (74479)

123 Janus Kinase Inhibitors/ (465)

124 ((jak or jak1 or jak2 or jak3 or janus kinase) adj10 inhibitor*).mp,rn. (6545)

125 (janus adj4 kinase adj10 inhibitor?).mp,rn. (2599)

126 exp Janus Kinases/ai [Antagonists & Inhibitors] (2315)

127 (baricitinib* or 1187594-09-7 or isp4442i3y* or "incb 028050" or incb028050 or ly-3009104 or ly3009104).mp,rn. (451)

128 (tofacitinib* or 477600-75-2 or 87la6fu830* or hsdb 8311 or hsdb8311 or cp-690 550 or cp 690550 or cp690550).mp,rn. (1648)

129 (filgotinib* or 1206161-97-8 or 3xvl385q0m* or "glpg 0634" or glpg0634).mp,rn. (115)

130 (upadacitinib* or 1310726-60-3 or 4RA0KN46E0* or abt 494 or abt494).mp,rn. (145)

131 (ruxolitinib* or rux or 941678-49-5 or 82s8x8xx8h* or hsdb 8259 or hsdb8259 or inc 424 or inc424 or incb424 or "incb 018424" or incb018424 or incb 18424 or incb18424 or r-ruxolitinib* or jakafi or jakavi).mp,rn. (1738)

132 (itacitinib or "incb 039110" or incb039110 or incb 39110 or incb39110 or 1334298-90-6 or 19j3781lpm*).mp,rn. (25)

133 (ag490 or ag 490 or tyrphostin b42 or tyrphostin ag 490 or tyrphostin ag490 or 133550-30-8).mp,rn. (1527)

134 (fedratinib* or tg101348 or tg 101348 or sar302503 or sar 302503 or 936091-26-8 or 6l1xp550i6* or inrebic).mp,rn. (155)

135 (ritlecitinib* or pf-06651600 or pf06651600 or pf 6651600 or pf6651600 or 2140301-97-7 or 132lf5wgh4* or 2192215-81-7 or eag4t1459k* or ritlecitinib malonate or ritlecitinib toslate or ritlecitinib tosylate or ritlecitinib propanedioate).mp,rn. (10)

136 (peficitinib* or peficitinib hydrobromide).mp,rn. (64)

137 or/52-136 (1532937)

138 40 and 137 (55642)

139 51 or 138 (56425)

140 ae.fs. (1765248)

141 (adverse adj3 (event* or effect* or reaction* or incident* or outcome*)).ti,ab,kf. (465450)

142 ((serious* or severe* or severit*) adj3 (adverse or saes or aes or adrs or sae or "ae" or adr)).ti,ab,kf. (47904)

143 (saes or aes or adrs or sae or "ae" or adr).ti,ab,kf. and adverse.mp. (21882)

144 (adverseevent* or adversedrugreaction* or adversereaction* or adverseincident* or adverseoutcome*).ti,ab,kf. (13)

145 side effect*.ti,ab,kf. (261469)

Kastrati K, *et al. RMD Open* 2022; 8:e002359. doi: 10.1136/rmdopen-2022-002359

146 exp "Drug-Related Side Effects and Adverse Reactions"/ (117944)

147 Adverse Drug Reaction Reporting Systems/ (7724)

148 exp Product Surveillance, Postmarketing/ (15909)

149 Substance Withdrawal Syndrome/ (21480)

150 withdraw*.ti,ab,kf. (129409)

151 safety.ti,ab,kf. (541929)

152 exp Drug Hypersensitivity/ (46443)

153 (hypersensitiv* or hyper sensitiv*).ti,ab,kf. (78946)

154 Anaphylaxis/ (21211)

155 anaphyla*.ti,ab,kf. (30444)

156 (toxic* adj6 (drug* or medication*)).ti,ab,kf. (37610)

157 intoxication*.ti,ab,kf. (42357)

158 Abnormalities, Drug-Induced/ (14574)

159 Teratogenesis/de (94)

160 teratogen*.ti,ab,kf. (17246)

161 ((drug or chemical*) adj1 induced).ti,ab,kf. (53926)

162 exp substance related disorders/ (282361)

163 (treatment adj3 emergent).ti,ab,kf. (6006)

164 exp Mortality/ (391059)

165 exp Death, Sudden/ (35406)

166 exp Death/ (150454)

167 (fatal or fatalit*).ti,ab,kf. (151028)

168 ((mortality or death?) adj4 (rate? or ratio or caus* or risk or sudden or number* or amount* or count*)).ti,ab,kf. (550712)

169 mortality.ti,ab,kf. (800003)

170 exp *Infections/ (2272669)

171 infection rate?.ti,ab,kf. (25595)

172 ((serious* or severe* or severit*) adj8 (infect* or bacter?emi* or septic?emi* or sepsis or fung?emi* or fungal infect* or fungal disease* or mycoses or mycosis or mycotic)).ti,ab,kf. (101810)

173 exp Opportunistic Infections/ (34831)

174 ((opportunistic* or opportune) adj6 (infect* or bacter?emi* or septic?emi* or bacterial infect* or bacterial diseas* or viral infect* or virus diseas* or virus infect* or microbial infect* or mycos#s or fung* infect* or mycotic diseas* or mycos#s infect* or fung?emi*)).ti,ab,kf. (20333)

175 ((overall or rate or rates or risk?) adj8 (infect* or bacter?emi* or septic?emi* or sepsis or bacterial infect* or bacterial diseas* or bacterious infect* or viral infect* or virus diseas* or virus infect* or microbial infect* or mycos#s or fung* infect* or mycotic diseas* or mycos#s infect* or fung?emi*)).ti,ab,kf. (214246)

176 infect*.ti,ab,kf. (1838106)

177 exp Tuberculosis/ (193709)

178 (tuberculosis or tuberculous).ti,ab,kf. (230536)

179 exp Cytomegalovirus Infections/ (25987)

180 ((cytomegalovirus or cytomegaly virus or cytomegaloviral or cmv) adj (infection? or disease?)).ti,ab,kf. (17400)

181 (cytomegalic inclusion adj3 disease?).ti,ab,kf. (576)

182 cytomegaloinfection?.ti,ab,kf. (1)

183 congenital cytomegalic inclusion.ti,ab,kf. (39)

184 cmv.ti,ab,kf. and (virus or viral or infect*).mp. (23190)

185 exp Mycobacterium Infections, Nontuberculous/ (35270)

186 exp Nontuberculous Mycobacteria/ (11805)

187 ((atypical or nontuberculous* or non-tuberculous*) adj3 (mycobacterios* or mycobacter*)).ti,ab,kf. (8144)

188 (mycobacter* adj3 intracellulare adj3 infection*).ti,ab,kf. (538)

189 exp Mycoses/ (127986)

190 (mycosis or mycoses or mycotic or fung*).ti,ab,kf. (235014)

191 (aspergillos#s or (aspergill* adj3 infect*)).ti,ab,kf. (15734)

192 (candidias#s* or candidemi* or (candid* adj3 infect*)).ti,ab,kf. (24202)

193 pneumocyst*.ti,ab,kf. (11780)

194 exp Hepatitis B/ (58983)

195 Hepatitis B virus/ (27519)

196 hepatitis b.ti,ab,kf. (80583)

197 (hepatitis adj1 (type b or injection or serum)).ti,ab,kf. (3066)

198 (hepatitis adj3 "b").ti,ab,kf. (84715)

199 hbv.ti,ab,kf. (43685)

200 exp Infections/ (2663858)

201 (opportunistic or serious* or severe* or severit*).ti,ab,kf. (1725934)

202 200 and 201 (248496)

203 exp Varicella Zoster Virus Infection/ (18307)

204 ((herpes or varicella*) adj1 zoster*).ti,ab,kf. (17684)

205 (zoster or shingles or varicellovirus infect*).ti,ab,kf. (19011)

206 exp Herpes Simplex/ (24393)

207 (herpes adj3 (simplex or vulgaris or infection*)).ti,ab,kf. (44044)

208 herpes.ti,ab,kf. (62587)

209 hsv.ti,ab,kf. and (infect* or herpes).mp. (23178)

210 203 or 204 or 205 or 206 or 207 or 208 or 209 (82606)

211 Virus Activation/ (7650)

212 (reactivat* or re-activat*).ti,ab,kf. (44526)

213 (activat* adj3 (virus or viral* or vir?emi*)).ti,ab,kf. (6326)

214 211 or 212 or 213 (53446)

215 210 and 214 (5397)

216 C-Reactive Protein/ (46917)

217 9007-41-4.rn. (46917)

218 ((c-reactive or creactive or c reaction or creaction) adj2 protein).ti,ab,kf. (72849)

219 crp.ti,ab,kf. (50775)

220 Blood Sedimentation/ (11926)

221 ((erythrocyte or blood) adj3 sedimentation*).ti,ab,kf. (16545)

222 esr.ti,ab,kf. and sedimentation.mp. (6469)

223 (DAS28-ESR or DAS-28-ESR or DAS28ESR).ti,ab,kf. (627)

224 ((DAS28 or "DAS 28" or disease activity score or das or disease activity) and ESR).ti,ab,kf. (2995)

225 esr.ti,ab,kf. (19875)

226 Procalcitonin/ (704)

227 Calcitonin/ (15827)

228 (procalcitonin or pro-calcitonin or pct).ti,ab,kf. (11002)

229 (calcitonin adj3 precursor?).ti,ab,kf. (134)

230 exp Leukocyte Count/ (98474)

231 ((leukocyte* or white blood cell or white cell or white blood or wbc) adj3 count*).ti,ab,kf. (39828)

232 wbc.ti,ab,kf. (17678)

233 exp Neoplasms/ (3398940)

234 exp Hematologic Neoplasms/ (21567)

235 exp histiocytic disorders, malignant/ or exp leukemia/ or exp lymphoma/ (386022)

236 exp Multiple Myeloma/ (41814)

237 (lymphom* or leuk?em* or myeloma*).ti,ab,kf. (477469)

238 ((h?ematolog* or blood disease) adj6 (malignan* or neoplas*)).ti,ab,kf. (31360)

239 (cancer or cancers).ti,ab,kf. (1864129)

240 neoplas*.ti,ab,kf. (408399)

241 (tumor? or tumour?).ti,ab,kf. (1719910)

242 (carcinom* or karzinom*).ti,ab,kf. (686631)

243 hodgkin*.ti,ab,kf. (68829)

244 (non-hodgkin* or nonhodgkin*).ti,ab,kf. (39094)

245 (mesotheliom* or mesoteliom*).ti,ab,kf. (16806)

246 malignan*.ti,ab,kf. (593227)

247 adenocarcinom*.ti,ab,kf. (154063)

248 metasta*.ti,ab,kf. (529491)

249 sarcom*.ti,ab,kf. (113112)

250 adenosarcom*.ti,ab,kf. (587)

251 osteosarcom*.ti,ab,kf. (24723)

252 reticulosarcom*.ti,ab,kf. (1297)

253 lymphosarcom*.ti,ab,kf. (5203)

254 (blastom* or neuroblastom*).ti,ab,kf. (47113)

255 (gliom* or glioblastom*).ti,ab,kf. (84729)

256 melanom*.ti,ab,kf. (122230)

257 ((non melanom* or nonmelanom*) adj3 skin adj3 (cancer or cancers or neoplas* or tumo?r* or carcino*)).ti,ab,kf. (5551)

258 (solid adj6 (cancer or cancers or neoplas* or tumo?r* or carcino* or malignan*)).ti,ab,kf. (74913)

259 exp Diverticulitis/ (6590)

260 diverticulitis*.ti,ab,kf. (6953)

261 Intestinal Perforation/ (13205)

262 ((digestive system or gastrointestinal* or gastro intestinal* or intestinal*) adj3 perforation*).ti,ab,kf. (5298)

263 exp Liver/ (445501)

264 Liver Function Tests/ (29140)

265 ((liver or hepat*) adj1 function?).ti,ab,kf. (48432)

266 (lft or lfts).ti,ab,kf. (1288)

267 (hypertransaminasemi* or hyper transaminasemi*).ti,ab,kf. (381)

268 uln.ti,ab,kf. (1236)

269 exp "Chemical and Drug Induced Liver Injury"/ (30011)

270 (dili or drug induc* liver injur*).ti,ab,kf. (3418)

271 Alanine Transaminase/ (31693)

272 exp Aspartate Aminotransferases/ (30019)

273 (transaminas* or alanin* aminotransaminas* or alt or aspartat* aminotransaminas* or ast or serum glutam* pyruv* transaminas* or sgpt or serum glutam* oxalacet* transaminas* or sgot or liver enzyme* or hepat* enzyme*).ti,ab,kf. (86198)

274 transaminitis*.ti,ab,kf. (750)

275 (hepat*tox* or ((liver or hepat*) adj3 toxic*)).ti,ab,kf. (38535)

276 exp Lipoproteins/bl [Blood] (77427)

277 exp Triglycerides/ (77586)

278 exp "lipoprotein(a)"/ or exp lipoproteins, hdl/ or exp lipoproteins, ldl/ (82221)

279 exp Cholesterol/ (161543)

280 ((lipid? or cholesterol* or cholesterin* or triglycerid* or lipoprotein*) adj3 (level? or blood or plasma or serum)).ti,ab,kf. (190160)

281 (ldl or low density lipoprotein*).ti,ab,kf. (113534)

282 (hdl or high density lipoprotein*).ti,ab,kf. (91058)

283 (hdl2 or "hdl(2)" or "hdl 2").ti,ab,kf. (2596)

284 (hdl3 or "hdl(3)" or "hdl 3").ti,ab,kf. (2532)

285 (lipoprotein a or lipoproteina).ti,ab,kf. (7679)

286 alpha lipoprotein.ti,ab,kf. (334)

287 (ldl1 or "ldl(1)" or "ldl 1").ti,ab,kf. (307)

288 (ldl2 or "ldl(2)" or "ldl 2").ti,ab,kf. (314)

289 exp Lipids/bl [Blood] (220133)

290 exp Hyperlipidemias/ (66675)

291 (hyperlipid?emi* or lipid?emi*).ti,ab,kf. (32753)

292 exp Diabetes Mellitus/ (434059)

293 exp Obesity/ (218348)

294 (metabolic adj3 (effect? or alterat* or chang* or risk? or transition* or transformat* or parameter? or marker? or biomarker?)).ti,ab,kf. (85591)

295 (diabetes* or diabetic*).ti,ab,kf. (657434)

296 Glycated Hemoglobin A/ (35830)

297 (hba1c or hbaic or "hb a1c" or "hb a 1c" or "hb aic" or "hb a ic" or "hba 1c" or "hba ic").ti,ab,kf. (40581)

298 ("h?emoglobin a1c" or "h?emoglobin a 1c" or h?emoglobin aic or "h?emoglobin a ic").ti,ab,kf. (13836)

299 body mass index/ (129993)

300 (body mass index or bmi).ti,ab,kf. (257363)

301 Venous Thromboembolism/ (11366)

302 ((venous* or venos* or vein*) adj1 (thrombos#s* or thrombotic*or thromboemboli* or thrombo emboli* or thrombus or thrombi or microthromb*)).ti,ab,kf. (53682)

303 (dvt or dvts or vte or vtes).ti,ab,kf. (20619)

304 exp Pulmonary Embolism/ (39482)

305 ((lung* or pulmonary) adj3 (embol* or thromboemboli* or thromb* emboli* or microemboli* or clot* or thrombus or thrombi or microthromb* or infarct*)).ti,ab,kf. (52142)

306 (pe or pes).ti,ab,kf. and (lung* or pulmo* or pneumo* or embol* or thromboembol* or thrombot* or thrombus or thrombi or microembol).mp. (11921)

307 exp Anemia/ (162737)

308 an?emi*.ti,ab,kf. (158664)

309 exp Thrombocytopenia/ (48327)

310 (thrombocytopeni* or thrombopeni*).ti,ab,kf. (64088)

311 exp Neutropenia/ (18884)

312 neutropeni*.ti,ab,kf. (42114)

313 exp Lymphopenia/ (5869)

314 (lymphocytopeni* or lymphopeni*).ti,ab,kf. (9473)

315 Macrophage Activation Syndrome/ (469)

316 mas.ti,ab,kf. (23057)

317 (macrophage activat* adj3 syndrome?).ti,ab,kf. (1071)

318 315 or 316 or 317 (23713)

319 (Rheumatoid Arthritis adj3 Systemic adj3 Juvenile).mp. (303)

320 Arthritis, Juvenile/ (10532)

321 ((arthrit* or artrit*) adj6 juvenile).mp. (13601)

322 JIA.ti,ab,kf,kw. (4120)

323 320 or 321 or 322 (14161)

324 (systemic or polyart* or poly art* or oligoart* or oligo art*).mp. (536618)

325 323 and 324 (4848)

326 ((systemic adj6 jia) or sjia or s-jia).ti,ab,kf. (869)

327 319 or 325 or 326 (4856)

328 318 and 327 (342)

329 exp Heart Failure/ (124580)

330 (congestive adj2 (heart or cardiac) adj2 failure*).ti,ab,kf. (44412)

331 (heart failure* or cardiac failure* or myocard* failure*).ti,ab,kf. (191253)

332 ((heart or cardiac or cordis or cardio* or myocard*) adj1 decompensatio*).ti,ab,kf. (1599)

333 (chf or ccf or hf).ti,ab,kf. and (heart or cardiac or cardio* or myocard*).mp. (41458)

334 exp Myocardial Ischemia/ (435508)

335 ((heart or cardio* or cardiac or myocardial) adj1 infarct*).ti,ab,kf. (205731)

336 exp Myocardial Infarction/ (177667)

337 cardiovascular* stroke?.ti,ab,kf. (47)

338 (MACE or MACEs).ti,ab,kf. (8829)

339 major adverse card* event*.ti,ab,kf. (11434)

340 acute coronary syndrome*.ti,ab,kf. (33652)

341 (heart attack or heart attacks).ti,ab,kf. (5731)

342 (mi or mis).ti,ab,kf. and (myocard* or cardio* or coronary or heart or cardiac or mace or maces or fatal* or non-fatal* or nonfatal*).mp. (33035)

343 (angina or anginas or stenocardia?).mp. (71053)

344 exp Stroke/ (139170)

345 ((cerebrovascular* or cerebr* vascular*) adj1 (accident? or failure? or injury or injuries or insult? or insufficienc* or apoplex*)).ti,ab,kf. (10671)

346 (stroke or strokes or apoplexi* or apoplexy*).ti,ab,kf. (264795)

347 (cva or cvas).ti,ab,kf. (3116)

348 (brain insult* or brain isch?emic attack? or cerebral insult* or brain vascular accident? or isch?emic cerebr* attack? or isch?emic seizure? or cerebr* vascular accident?).ti,ab,kf. (2968)

349 exp Myocardial Revascularization/ (92799)

350 (revascularizat* or re-vascularizat* or revascularisat* or re-vascularisat*).ti,ab,kf,hw. (66500)

351 Peripheral Arterial Disease/ (8307)

352 Arterial Occlusive Diseases/ (27413)

353 (peripher* adj2 (arter* or vascular*) adj2 disease*).ti,ab,kf. (30445)

354 ((pad or pvd) adj6 (peripher* or arter* or vascular*)).ti,ab,kf. (7140)

355 exp Kidney Function Tests/ (80541)

356 ((kidney or renal) adj1 function*).ti,ab,kf. (110390)

357 Creatinine/bl [Blood] (35721)

358 creatinin*.ti,ab,kf. and (serum or plasma or blood).mp. (93100)

359 (creatinin* adj15 (level? or concentration? or ratio or clearance or value?)).ti,ab,kf. (74571)

360 (creatinin* adj15 (decreas* or increas* or elevat* or lower* or low or high or higher)).ti,ab,kf. (48216)

361 AYI8EX34EU.rn. (56939)

362 (estimated glomerul* filtrat* rate? or estimated glomerulofiltration rate?).ti,ab,kf. (18873)

363 (egfr or egfrs or e-gfr or e-gfrs or estimat* gfr or estimat* gfrs or estimat*gfr or estimat*gfrs).ti,ab,kf. (64485)

364 Glomerular Filtration Rate/ (45131)

365 exp Renal Insufficiency/ (176735)

366 ((renal or kidney) adj1 (insufficien* or failure? or injur*)).ti,ab,kf. (155330)

367 exp Hemoglobins/ (128060)

368 h?emoglobin*.ti,ab,kf. (175065)

369 exp Osteoporosis/ (56494)

370 (osteoporos* or osteoporotic*).ti,ab,kf. (80428)

371 exp Leukoencephalopathies/ (32187)

372 (leukoencephalopath* or leuko encephalopath*).ti,ab,kf. (7874)

373 exp Demyelinating Diseases/ (102212)

374 ((demyelinat* or demyeliniz*) adj3 (disease* or disorder* or cns or central nervous system* or encephalopath* or autoimmune disease*)).ti,ab,kf. (11111)

375 demyelinat*.mp. (37619)

376 demyeliniz*.mp. (499)

377 demyelinis*.mp. (304)

378 exp Multiple Sclerosis/ (59955)

379 multiple scleros#s.ti,ab,kf. (78274)

380 exp Optic Neuritis/ (8726)

381 (optic* adj3 (neuritis* or neuritides*)).ti,ab,kf. (6315)

382 (optic* adj3 (inflammat* adj3 neuropath*)).ti,ab,kf. (126)

383 exp Myelitis, Transverse/ (4314)

384 (transverse adj3 (myelitis* or myelitides* or myelopath* syndrome*)).ti,ab,kf. (2280)

385 (necrotiz* adj3 (myelitis* or myelitides*)).ti,ab,kf. (41)

386 (demyelinat* adj3 (myelitis* or myelitides*)).ti,ab,kf. (48)

387 ((postinfect* or infect* or postvaccin* or vaccin*) adj10 (myelitis or myelitides)).ti,ab,kf. (649)

388 exp Peripheral Nervous System Diseases/ (149885)

389 (peripheral adj2 (nervous system or nerve) adj2 (disease* or disorder* or neuropath* or mononeuropath* or polyneuropath* or polyradiculaneuropath*)).ti,ab,kf. (2104)

390 exp Guillain-Barre Syndrome/ (5260)

391 (guillain barre* or fisher? syndrome*).ti,ab,kf. (10513)

392 ((landry? or landry guillain barre?) adj3 (syndrom* or paralys*)).ti,ab,kf. (230)

393 (miller fisher? adj3 (syndrom* or polyneuritis* or variant?)).ti,ab,kf. (876)

394 ((polyradiculoneuropath* or poly radiculoneuropath* or polyradiculo neuropath* or polyneuropath* or poly neuropath* or neuronitis* or polyneuritis* or poly neuritis*) adj15 (inflammat* or postinfect* or infect* or postvaccin* or vaccin*)).ti,ab,kf. (4229)

395 Polyradiculoneuropathy, Chronic Inflammatory Demyelinating/ (1559)

396 ((polyradiculoneuropath* or poly radiculoneuropath* or polyradiculo neuropath* or polyneuropath* or poly neuropath* or polyradiculopath* or poly radiculopath*) adj6 chronic* adj3 inflammat*).ti,ab,kf. (2694)

397 cidp.ti,ab,kf. (1851)

398 exp Polyradiculoneuropathy/ (12782)

399 (polyradiculoneuropath* or poly radiculoneuropath* or polyradiculo neuropath*).ti,ab,kf. (1700)

400 ((demyelinat* or inflammat*) adj1 (neuropath* or polyneuropath* or poly neuropath* or polyradiculoneuropath* or poly radiculoneuropath* or polyradiculo neuropath*)).ti,ab,kf. (5652)

401 (multifocal motor neuropath* adj6 conduction block*).ti,ab,kf. (133)

402 (motor adj3 neuropath* adj6 conduction block*).ti,ab,kf. (216)

403 exp Motor Neuron Disease/ (28488)

404 (motor adj3 neuropath*).ti,ab,kf. (4617)

405 exp Mononeuropathies/ (20363)

406 (mononeuropath* or mono neuropath* or mononeuritis* or mono neuritis* or mononeuritides* or mono neuritides*).ti,ab,kf. (2413)

407 exp Polyneuropathies/ (28012)

408 ((polyneuropath* or poly neuropath*) adj6 axonal adj3 (sensor* motor or sensorimotor* or sensorymotor)).ti,ab,kf. (242)

409 (polyneuropath* or poly neuropath*).ti,ab,kf. (14987)

410 exp Vaccination/ (86812)

411 exp Vaccines/ (232416)

412 vaccinat*.ti,ab,kf. (160370)

413 (active adj2 immuni#at*).ti,ab,kf. (3777)

414 vaccine*.ti,ab,kf. (247383)

415 exp Pregnancy/ (905498)

416 exp Pregnancy Complications/ (431899)

417 pregnan*.ti,ab,kf. (537837)

418 tolerabilit*.ti,ab,kf. (50985)

419 safe.ti,ab,kf. (379201)

420 ((injurious* or undesirabl*) adj3 (effect? or reaction* or event? or outcome? or incident?)).ti,ab,kf. (20838)

421 exp Postoperative Complications/ (556098)

422 exp Intraoperative Complications/ (53530)

423 complication*.ti,ab,kf. (1014283)

424 co.fs. (2004332)

425 risk.ti,ab,kf. (2207646)

426 de.fs. (3025692)

427 exp risk/ (1239045)

428 140 or 141 or 142 or 143 or 144 or 145 or 146 or 147 or 148 or 149 or 150 or 151 or 152 or 153 or 154 or 155 or 156 or 157 or 158 or 159 or 160 or 161 or 162 or 163 or 164 or 165 or 166 or 167 or 168 or 169 or 170 or 171 or 172 or 173 or 174 or 175 or 176 or 177 or 178 or 179 or 180 or 181 or 182 or 183 or 184 or 185 or 186 or 187 or 188 or 189 or 190 or 191 or 192 or 193 or 194 or 195 or 196 or 197 or 198 or 199 or 202 or 215 or 216 or 217 or 218 or 219 or 220 or 221 or 222 or 223 or 224 or 225 or 226 or 227 or 228 or 229 or 230 or 231 or 232 or 233 or 234 or 235 or 236 or 237 or 238 or 239 or 240 or 241 or 242 or 243 or 244 or 245 or 246 or 247 or 248 or 249 or 250 or 251 or 252 or 253 or 254 or 255 or 256 or 257 or 258 or 259 or 260 or 261 or 262 or 263 or 264 or 265 or 266 or 267 or 268 or 269 or 270 or 271 or 272 or 273 or 274 or 275 or 276 or 277 or 278 or 279 or 280 or 281 or 282 or 283 or 284 or 285 or 286 or 287 or 288 or 289 or 290 or 291 or 292 or 293 or 294 or 295 or 296 or 297 or 298 or 299 or 300 or 301 or 302 or 303 or 304 or 305 or 306 or 307 or 308 or 309 or 310 or 311 or 312 or 313 or 314 or 328 or 329 or 330 or 331 or 332 or 333 or 334 or 335 or 336 or 337 or 338 or 339 or 340 or 341 or 342 or 343 or 344 or 345 or 346 or 347 or 348 or 349 or 350 or 351 or 352 or 353 or 354 or 355 or 356 or 357 or 358 or 359 or 360 or 361 or 362 or 363 or 364 or 365 or 366 or 367 or 368 or 369 or 370 or 371 or 372 or 373 or 374 or 375 or 376 or 377 or 378 or 379 or 380 or 381 or 382 or 383 or 384 or 385 or 386 or 387 or 388 or 389 or 390 or 391 or 392 or 393 or 394 or 395 or 396 or 397 or 398 or 399 or 400 or 401 or 402 or 403 or 404 or 405 or 406 or 407 or 408 or 409 or 410 or 411 or 412 or 413 or 414 or 415 or 416 or 417 or 418 or 419 or 420 or 421 or 422 or 423 or 424 or 425 or 426 or 427 (16675282)

429 139 and 428 (42533)

430 ((eular or (european adj4 rheumat*)) adj8 (meeting* or conference* or congress*) adj8 annual).af. (67)

431 ((acr or (american college adj4 rheumat*)) adj8 (meeting* or conference* or congress*) adj8 annual).af. (137)

432 430 or 431 (172)

433 429 and 432 (25)

434 limit 433 to yr="2019 - 2020" (4)

435 limit 429 to yr="2012-2020" (16514)

436 limit 435 to english (15523)

437 exp Registries/ (99914)

438 (register or registry or registries or registered or registration*).ti,ab,kf. (406182)

439 Cohort Studies/ (274744)

440 (cohort* adj6 (study* or studies* or analy*)).ti,ab,kf. (315251)

441 (cohortstudy* or cohortstudies* or cohortanaly*).ti,ab,kf. (4)

442 exp case-control studies/ (1131012)

443 (case? adj3 control* adj3 (study or match*)).ti,ab,kf. (104186)

444 case referent.ti,ab,kf. (660)

445 (case? adj2 control?).ti,ab,kf. (152426)

446 Prospective Studies/ (559046)

447 exp Longitudinal Studies/ (140858)

448 Retrospective Studies/ (861564)

449 (longitudinal or prospective* or retrospective*).ti,ab,kf. (1665493)

450 Follow-Up Studies/ (653467)

451 ((follow up or followup) adj6 (trial or trials or study or studies or analy*)).ti,ab,kf. (146679)

452 450 or 451 (742053)

453 (long term or longterm).ti,ab,kf. (832736)

454 (extend* or extension?).ti,ab,kf. (648046)

455 (continuing or continue* or continuat*).ti,ab,kf. (505555)

456 453 or 454 or 455 (1899474)

457 452 and 456 (155625)

458 ((long term or longterm) adj3 data).ti,ab,kf. (16112)

459 457 or 458 (168189)

460 437 or 438 or 439 or 440 or 441 or 442 or 443 or 444 or 445 or 446 or 447 or 448 or 449 or 459 (3025213)

461 436 and 460 (5029)

462 randomized controlled trial.pt. (519987)

463 exp randomized controlled trial/ (521007)

464 exp Randomized Controlled Trials as Topic/ (142623)

465 randomi*.ti,ab,kf. (669713)

466 randomly.ti,ab,kf. (349683)

467 trial.ti. (232626)

468 trial.ab. and random*.mp. (315455)

469 (rct or rcts).ti,ab,kf. and random*.mp. (47700)

470 Random Allocation/ (104297)

471 controlled clinical trial/ (93997)

472 Controlled Clinical Trials as Topic/ (5555)

473 Single-Blind Method/ (29501)

474 Double-Blind Method/ (161600)

475 ((singl* or doubl* or tripl* or trebl*) adj3 (blind* or mask*)).ti,ab,kf. (178910)

476 (singl*blind* or doubl*blind* or trebl*blind* or tripl*blind*).ti,ab,kf. (252)

477 (singl*mask* or doubl*mask* or tripl*mask* or trebl*mask*).ti,ab,kf. (4)

478 (controlled adj8 (study or design or trial)).ti,ab,kf. (277888)

479 (parallel adj1 group*).ti,ab,kf. (19660)

480 exp placebo effect/ (4889)

481 Placebos/ (35260)

482 placebo*.ti,ab,kf. (222177)

483 (allocated or allocation or assigned).ti,ab,kf. and random*.mp. (194135)

484 groups.ti,ab,kf. (2176015)

485 dt.fs. (2264973)

486 ((assign* or match or matched or allocation) adj6 (alternate or group or groups or intervention? or patient? or subject? or participant?)).ti,ab,kf. (268235)

487 ('head to head' adj3 (study or comparison or trial)).ti,ab,kf. and random*.mp. (863)

488 or/462-487 (5043679)

489 456 and 488 (479344)

490 436 and 489 (1753)

491 461 or 490 (5909)

492 exp animals/ not humans/ (4772383)

493 491 not 492 (5878)

494 review.pt. (2742419)

495 congress.pt. (66480)

496 editorial.pt. (552831)

497 letter.pt. (1116218)

498 494 or 495 or 496 or 497 (4450357)

499 493 not 498 (5044)

500 434 or 499 (5046)

501 from 500 keep 1-2000 (2000)

502 from 500 keep 2001-4000 (2000)

503 from 500 keep 4001-5046 (1046)

1.3.3.2 EMBASE

#410,"#404 NOT #409",5525,24 Nov 2020

#409,"#407 AND #408",1106559,24 Nov 2020

#408,"'animal experiment'/de",2606288,24 Nov 2020

#407,"#405 OR #406",2451378,24 Nov 2020

#406,"mouse:tt OR mice:tt OR rat:tt OR rats:tt OR murine$:tt OR swine:tt OR porcine:tt OR sheep:tt OR lamb:tt OR lambs:tt OR pig:tt OR pigs:tt OR piglets:tt OR rabbit:tt OR rabbits:tt OR cat:tt OR cats:tt OR dog:tt OR dogs:tt OR cattle:tt OR bovine:tt OR monkey:tt OR monkeys:ti OR trout:tt OR marmoset*:tt",39752,24 Nov 2020

#405,"mouse:ti OR mice:ti OR rat:ti OR rats:ti OR murine$:ti OR swine:ti OR porcine:ti OR sheep:ti OR lamb:ti OR lambs:ti OR pig:ti OR pigs:ti OR piglets:ti OR rabbit:ti OR rabbits:ti OR cat:ti OR cats:ti OR dog:ti OR dogs:ti OR cattle:ti OR bovine:ti OR monkey:ti OR monkeys:ti OR trout:ti OR marmoset*:ti",2450524,24 Nov 2020

#404,"#402 NOT #403",5529,24 Nov 2020

#403,"('animal experiment'/de OR 'animal model'/de OR 'animal tissue'/de OR 'animal cel') NOT ('human experiment'/de OR 'human'/de)",2762877,24 Nov 2020

#402,"#400 NOT #401",5535,24 Nov 2020

#401,"#351 AND #397 AND [2012-2020]/py AND [english]/lim AND ([conference abstract]/lim OR [conference paper]/lim OR [conference review]/lim OR [editorial]/lim OR [letter]/lim OR [review]/lim)",9437,24 Nov 2020

#400,"#351 AND #397 AND [2012-2020]/py AND [english]/lim",14972,24 Nov 2020

#399,"#351 AND #397 AND [2012-2020]/py",15151,24 Nov 2020

#398,"#351 AND #397",20215,24 Nov 2020

#397,"#352 OR #353 OR #354 OR #355 OR #356 OR #357 OR #358 OR #359 OR #360 OR #361 OR #364 OR #365 OR #373 OR #375 OR #396",4028419,24 Nov 2020

#396,"#372 AND #395",152421,24 Nov 2020

#395,"#376 OR #377 OR #378 OR #379 OR #380 OR #381 OR #382 OR #383 OR #384 OR #385 OR #386 OR #387 OR #388 OR #389 OR #390 OR #391 OR #392 OR #393 OR #394",1871770,24 Nov 2020

#394,"(((allocated OR allocation) NEAR/3 random*):ti,ab,kw) OR (((allocated OR allocation) NEAR/3 random*):tt)",45967,24 Nov 2020

#393,"placebo*:ti,ab,kw OR placebo*:tt",317096,24 Nov 2020

#392,"((parallel NEXT/1 group*):ti,ab,kw) OR ((parallel NEXT/1 group*):tt)",26365,24 Nov 2020

#391,"((controlled NEAR/8 (study OR design OR trial)):ti,ab,kw) OR ((controlled NEAR/8 (study OR design OR trial)):tt)",375879,24 Nov 2020

#390,"singl*blind:tt OR doubl*blind*:tt OR trebl*blind*:tt OR tripl*blind*:tt OR singl*mask*:tt OR doubl*mask*:tt OR trebl*mask*:tt OR tripl*mask*:tt",33,24 Nov 2020

#389,"singl*blind:ti,ab,kw OR doubl*blind*:ti,ab,kw OR trebl*blind*:ti,ab,kw OR tripl*blind*:ti,ab,kw OR singl*mask*:ti,ab,kw OR doubl*mask*:ti,ab,kw OR trebl*mask*:ti,ab,kw OR tripl*mask*:ti,ab,kw",207671,24 Nov 2020

#388,"((singl* OR doubl* OR tripl* OR trebl*) NEAR/3 (blind* OR mask*)):tt",69,24 Nov 2020

#387,"((singl* OR doubl* OR tripl* OR trebl*) NEAR/3 (blind* OR mask*)):ti,ab,kw",248452,24 Nov 2020

#386,"'triple blind procedure'/de",267,24 Nov 2020

#385,"'double blind procedure'/de",178172,24 Nov 2020

#384,"'single blind procedure'/de",40746,24 Nov 2020

#383,"'controlled clinical trial'/de",431689,24 Nov 2020

#382,"'randomization'/exp",88694,24 Nov 2020

#381,"(rct:ti,ab,kw OR rcts:ti,ab,kw OR rct:tt OR rcts:tt) AND random*",68341,24 Nov 2020

#380,"randomly:ti,ab,kw OR randomly:tt",457608,24 Nov 2020

#379,"trial:ab AND random*:ti,ab,kw,de",421206,24 Nov 2020

#378,"trial:ti OR trial:tt",319769,24 Nov 2020

#377,"randomi*:ti,ab,kw OR randomi*:tt",948361,24 Nov 2020

#376,"'randomized controlled trial'/de",630140,24 Nov 2020

#375,"#363 AND #374",22892,24 Nov 2020

#374,"((('long-term' OR longterm) NEAR/3 data):ti,ab,kw) OR ((('long-term' OR longterm) NEAR/3 data):tt)",26046,24 Nov 2020

#373,"#368 AND #372",284858,24 Nov 2020

#372,"#369 OR #370 OR #371",1380709,24 Nov 2020

#371,"continuation:ti,ab,kw OR continuation:tt",31570,24 Nov 2020

#370,"extension:ti,ab,kw OR extension:tt",216725,24 Nov 2020

#369,"'long term':ti,ab,kw OR longterm:ti,ab,kw OR 'long term':tt OR longterm:tt",1151023,24 Nov 2020

#368,"#366 OR #367",1682407,24 Nov 2020

#367,"(('follow up' OR followup) NEAR/6 (trial OR trials OR study OR studies OR analy* OR design)):ti,ab,kw",234641,24 Nov 2020

#366,"'follow up'/de",1621131,24 Nov 2020

#365,"'long term follow up'/de",27,24 Nov 2020

#364,"#362 AND #363",915318,24 Nov 2020

#363,"study:ti,ab,kw,de OR trial:ti,ab,kw,de OR studies:ti,ab,kw,de OR trials:ti,ab,kw,de OR analys$s:ti,ab,kw,de OR design:ti,ab,kw,de",22225808,24 Nov 2020

#362,"longitudinal:ti,ab,kw OR 'prospective':ti,ab,kw OR retrospective:ti,ab,kw OR longitudinal:tt OR 'prospective':tt OR retrospective:tt",2028463,24 Nov 2020

#361,"'retrospective study'/de",992323,24 Nov 2020

#360,"'longitudinal study'/exp",147024,24 Nov 2020

#359,"'prospective study'/de",641228,24 Nov 2020

#358,"(case$:ti,ab,kw OR case$:tt) AND (control*:ti,ab,kw OR control*:tt) AND (study:ti,ab,kw,de OR studies:ti,ab,kw,de OR match*:ti,ab,kw,de OR trial:ti,ab,kw,de OR trials:ti,ab,kw,de OR design:ti,ab,kw,de OR analy*:ti,ab,kw,de)",640672,24 Nov 2020

#357,"(('case control' NEAR/3 (study OR match*)):ti,ab,kw) OR (('case control' NEAR/3 (study OR match*)):tt)",128121,24 Nov 2020

#356,"'case control study'/exp",180271,24 Nov 2020

#355,"((cohort* NEAR/6 (study* OR studies* OR analy*)):ti,ab,kw) OR ((cohort* NEAR/6 (study* OR studies* OR analy*)):tt) OR cohortstudy*:ti,ab,kw OR cohortstudies*:ti,ab,kw OR cohortanaly*:ti,ab,kw OR cohortstudy:tt OR cohortstudies:tt OR cohortanaly*:tt",478327,24 Nov 2020

#354,"'cohort analysis'/de",636397,24 Nov 2020

#353,"register:ti,ab,kw OR registry:ti,ab,kw OR registries:ti,ab,kw OR registered:ti,ab,kw OR registration$:ti,ab,kw OR register:tt OR registry:tt OR registries:tt OR registered:tt OR registration$:tt",556840,24 Nov 2020

#352,"'register'/de",113713,24 Nov 2020

#351,"#150 AND #350",77593,24 Nov 2020

#350,"#151 OR #152 OR #153 OR #154 OR #155 OR #156 OR #157 OR #158 OR #159 OR #160 OR #161 OR #162 OR #163 OR #164 OR #165 OR #166 OR #167 OR #168 OR #169 OR #170 OR #171 OR #172 OR #173 OR #174 OR #175 OR #176 OR #177 OR #178 OR #179 OR #180 OR #181 OR #182 OR #183 OR #184 OR #185 OR #186 OR #187 OR #188 OR #189 OR #190 OR #191 OR #192 OR #193 OR #194 OR #195 OR #196 OR #197 OR #198 OR #199 OR #200 OR #201 OR #202 OR #203 OR #204 OR #205 OR #206 OR #207 OR #208 OR #209 OR #212 OR #220 OR #221 OR #222 OR #223 OR #224 OR #225 OR #226 OR #227 OR #228 OR #229 OR #230 OR #231 OR #232 OR #233 OR #234 OR #235 OR #236 OR #237 OR #238 OR #239 OR #240 OR #241 OR #242 OR #243 OR #244 OR #245 OR #246 OR #247 OR #248 OR #249 OR #250 OR #251 OR #252 OR #253 OR #254 OR #255 OR #256 OR #257 OR #258 OR #259 OR #260 OR #261 OR #262 OR #263 OR #264 OR #265 OR #266 OR #267 OR #268 OR #269 OR #270 OR #271 OR #272 OR #273 OR #274 OR #275 OR #276 OR #277 OR #278 OR #279 OR #280 OR #281 OR #282 OR #283 OR #284 OR #290 OR #291 OR #292 OR #293 OR #294 OR #295 OR #296 OR #297 OR #298 OR #299 OR #300 OR #301 OR #302 OR #303 OR #304 OR #305 OR #306 OR #307 OR #308 OR #309 OR #310 OR #311 OR #312 OR #313 OR #314 OR #315 OR #316 OR #317 OR #318 OR #319 OR #320 OR #321 OR #322 OR #323 OR #324 OR #325 OR #326 OR #327 OR #328 OR #329 OR #330 OR #331 OR #332 OR #333 OR #334 OR #335 OR #336 OR #337 OR #338 OR #339 OR #340 OR #341 OR #342 OR #343 OR #344 OR #345 OR #346 OR #347 OR #348 OR #349",16440415,24 Nov 2020

#349,"((injurious OR undesirabl*) NEAR/3 (effect$ OR reaction$ OR event$ OR outcome$ OR incident$)):tt",0,24 Nov 2020

#348,"((injurious OR undesirabl*) NEAR/3 (effect$ OR reaction$ OR event$ OR outcome$ OR incident$)):ti,ab,kw",18213,24 Nov 2020

#347,"safe:ti,ab,kw OR safe:tt",563138,24 Nov 2020

#346,"tolerability:ti,ab,kw OR tolerability:tt",90740,24 Nov 2020

#345,"'drug tolerability'/de",132915,24 Nov 2020

#344,"'pregnancy complication'/exp",153357,24 Nov 2020

#343,"pregnan*:ti,ab,kw OR pregnan*:tt",694467,24 Nov 2020

#342,"'pregnancy'/exp",779817,24 Nov 2020

#341,"'vaccine*':ti,ab,kw OR vaccine*:tt",293299,24 Nov 2020

#340,"'vaccine'/exp",355013,24 Nov 2020

#339,"vaccinat*:ti,ab,kw OR vaccinat*:tt",192630,24 Nov 2020

#338,"'vaccination'/exp",181688,24 Nov 2020

#337,"('peripheral nervous system' NEAR/1 (disorder$ OR diseas* OR neuropath* OR mononeuropath* OR polyneuropath* OR polyradiculoneuropath*)):tt",0,24 Nov 2020

#336,"('peripheral nervous system' NEAR/1 (disorder$ OR diseas* OR neuropath* OR mononeuropath* OR polyneuropath* OR polyradiculoneuropath*)):ti,ab,kw",701,24 Nov 2020

#335,"'axonal sensorimotor polyneuropathy'/de",17,24 Nov 2020

#334,"'mononeuropathy'/exp OR 'mononeuritis' OR 'mononeuropathies' OR 'mononeuropathy'",80626,24 Nov 2020

#333,"'motor neuropathy'/exp OR 'motor neuropathy' OR 'multifocal motor neuropathy' OR 'neuropathy, motor'",6488,24 Nov 2020

#332,"(((demyelinat* OR inflammator*) NEAR/1 (neuropath* OR polyneuropath* OR polyradiculoneuropath*)):ti,ab,kw) OR ((demyelinat* NEAR/1 (neuropath* OR polyneuropath* OR polyradiculoneuropath*)):tt)",9036,24 Nov 2020

#331,"cidp:ti,ab,kw OR cidp:tt",3757,24 Nov 2020

#330,"'polyradiculoneuropathy'/exp OR 'chronic inflammatory demyelinating polyradiculoneuropathy' OR 'polyradiculoneuropathy' OR 'polyradiculoneuropathy, chronic inflammatory demyelinating' OR 'radiculoneuropathy'",18702,24 Nov 2020

#329,"'chronic inflammatory neuropathy'",34,24 Nov 2020

#328,"'guillain barre syndrome'/de OR 'fisher syndrome' OR 'guillain barre' OR 'guillain barre disease' OR 'guillain barre polyradiculitis' OR 'guillain barre polyradiculoneuritis' OR 'guillain barre syndrome' OR 'guillain-barre syndrome' OR 'landry guillain barre strohl syndrome' OR 'landry guillain barre syndrome' OR 'landry paralysis' OR 'landry syndrome' OR 'miller fisher syndrome' OR 'acute febrile polyneuritis' OR 'acute postinfective polyradiculoneuropathy' OR 'infectious neuronitis' OR 'inflammatory acute polyradiculoneuropathy' OR 'polyradiculoneuritis guillain-barre' OR 'polyradiculoneuropathy, acute postinfective' OR 'polyradiculoneuropathy, inflammatory acute'",19516,24 Nov 2020

#327,"'peripheral neuropathy'/exp",75994,24 Nov 2020

#326,"'acute transverse myelitis':ti,ab,kw OR 'transverse myelitis':ti,ab,kw OR 'demyelinat* myelit*':ti,ab,kw OR 'acute transverse myelitis':tt OR 'transverse myelitis':tt OR 'demyelinat* myelit*':tt",4070,24 Nov 2020

#325,"'acute transverse myelitis'/de",65,24 Nov 2020

#324,"'optic neuritis'/exp OR 'acute optic neuritis' OR 'inflammatory optic neuropathies' OR 'inflammatory optic neuropathy' OR 'neuritis optica' OR 'neuritis, optic' OR 'optic neuritis' OR 'opticus neuritis'",14212,24 Nov 2020

#323,"((demyelinat* NEAR/3 (brain OR cns OR disease$ OR 'nervous system' OR disorder$)):ti,ab,kw) OR ((demyelinat* NEAR/3 (brain OR cns OR disease$ OR 'nervous system' OR disorder$)):tt)",17655,24 Nov 2020

#322,"'multiple scleros$s*':ti,ab,kw OR 'multiple scleros$s*':tt",121917,24 Nov 2020

#321,"'demyelinating disease'/exp OR 'brain demyelination' OR 'clinically isolated syndrome' OR 'cns demyelinating autoimmune diseases' OR 'demyelinating autoimmune diseases, cns' OR 'demyelinating brain disease' OR 'demyelinating disease' OR 'demyelinating disease, hereditary central nervous system' OR 'demyelinating diseases' OR 'demyelinating encephalopathy' OR 'demyelination disease' OR 'demyelinizing disease' OR 'hereditary central nervous system demyelinating disease' OR 'hereditary central nervous system demyelinating diseases' OR 'heredodegenerative disorders, nervous system' OR 'nervous system heredodegenerative disorders'",184194,24 Nov 2020

#320,"leukoencephalopath*:ti,ab,kw OR leukoencephalopath*:tt",11540,24 Nov 2020

#319,"'leukoencephalopathy'/de",7297,24 Nov 2020

#318,"osteoporos*:ti,ab,kw OR osteoporotic:ti,ab,kw OR osteoporos*:tt OR osteoporotic:tt",125112,24 Nov 2020

#317,"'osteoporosis'/exp",133281,24 Nov 2020

#316,"egfr:ti,ab,kw,de OR egfr:tt",119758,24 Nov 2020

#315,"'estimated glomerular* filtrat* rate$':ti,ab,kw OR 'estimated glomerular* filtrat* rate$':tt",27800,24 Nov 2020

#314,"((creatinin* NEAR/3 (serum OR plasma OR blood)):ti,ab,kw) OR ((creatinin* NEAR/3 (serum OR plasma OR blood)):tt)",86869,24 Nov 2020

#313,"'creatinine blood level'/de",114555,24 Nov 2020

#312,"'kidney function'/exp",201806,24 Nov 2020

#311,"'peripheral occlusive artery disease'/exp",178846,24 Nov 2020

#310,"revascularizat*:ti,ab,kw OR revascularisat*:ti,ab,kw OR revascuarizat*:tt OR revascularisat*:tt",94167,24 Nov 2020

#309,"'heart muscle revascularization'/de",32774,24 Nov 2020

#308,"'revascularization'/exp",56773,24 Nov 2020

#307,"'brain insult*':tt OR 'brain isch$emic attack$':tt OR 'cerebral insult*':tt OR 'brain vascular accident$':tt OR 'isch$emic cerebral attack$':tt OR 'isch$emic seizure$':tt OR 'cerebrum vascular accident$':tt",3,24 Nov 2020

#306,"'brain insult*':ti,ab,kw OR 'brain isch$emic attack$':ti,ab,kw OR 'cerebral insult*':ti,ab,kw OR 'brain vascular accident$':ti,ab,kw OR 'isch$emic cerebral attack$':ti,ab,kw OR 'isch$emic seizure$':ti,ab,kw OR 'cerebrum vascular accident$':ti,ab,kw",2333,24 Nov 2020

#305,"cva:ti,ab,kw OR cvas:ti,ab,kw OR cva:tt OR cvas:tt",6932,24 Nov 2020

#304,"stroke:ti,ab,kw OR strokes:ti,ab,kw OR apoplexi*:ti,ab,kw OR apoplexy*:ti,ab,kw OR stroke:tt OR strokes:tt OR apoplexi*:tt OR apoplexy*:tt",418055,24 Nov 2020

#303,"((cerebrovascular* OR 'cerebro vascular*' OR 'cerebral vascular*') NEAR/1 (accident$ OR failure$ OR injury OR injuries OR insult$ OR insufficienc* OR apoplexi*)):tt",199,24 Nov 2020

#302,"((cerebrovascular* OR 'cerebro vascular*' OR 'cerebral vascular*') NEAR/1 (accident$ OR failure$ OR injury OR injuries OR insult$ OR insufficienc* OR apoplexi*)):ti,ab,kw",17186,24 Nov 2020

#301,"'cerebrovascular accident'/exp",335644,24 Nov 2020

#300,"'heart attack$':ti,ab,kw OR 'heart attack$':tt",8276,24 Nov 2020

#299,"(((myocardial OR heart) NEAR/1 (infarct$ OR infarction$)):ti,ab,kw) OR (((myocardial OR heart) NEAR/1 (infarct$ OR infarction$)):tt)",298736,24 Nov 2020

#298,"'non fatal myocardial infarction'/de",27,24 Nov 2020

#297,"'acute heart infarction'/de",75630,24 Nov 2020

#296,"'stroke patient'/exp",33114,24 Nov 2020

#295,"'heart infarction'/exp",403581,24 Nov 2020

#294,"'ischemic heart disease'/exp",700345,24 Nov 2020

#293,"'heart failure$':ti,ab,kw OR 'cardiac failure$':ti,ab,kw OR 'heart failure$':tt OR 'cardiac failure$':tt",310170,24 Nov 2020

#292,"((congestive NEAR/2 (heart OR cardiac) NEAR/2 failure$):ti,ab,kw) OR ((congestive NEAR/2 (heart OR cardiac) NEAR/2 failure$):tt)",59274,24 Nov 2020

#291,"'congestive heart failure'/exp",112582,24 Nov 2020

#290,"#22 AND #289",882,24 Nov 2020

#289,"#285 OR #286 OR #287 OR #288",27206,24 Nov 2020

#288,"('macrophag* activat*' NEAR/3 (syndrome* OR factor$)):ti,ab,kw",2885,24 Nov 2020

#287,"'macrophage activating factor'/de",956,24 Nov 2020

#286,"'macrophage activation syndrome*':ti,ab,kw OR mas:ti,ab,kw OR 'macrophage activation syndrome*':tt OR mas:tt",25147,24 Nov 2020

#285,"'macrophage activation syndrome'/de",2241,24 Nov 2020

#284,"an$emi*:ti,ab,kw OR thrombocytopeni*:ti,ab,kw OR neutropeni*:ti,ab,kw OR lymphopeni*:ti,ab,kw OR lymphocytopeni*:ti,ab,kw OR an$emi*:tt OR thrombocytopeni*:tt OR neutropeni*:tt OR lymphopeni*:tt OR lymphocytopeni*:tt",368433,24 Nov 2020

#283,"((g3 NEAR/2 g4):ti,ab,kw) OR ((g3 NEAR/2 g4):tt)",2427,24 Nov 2020

#282,"'lymphocytopenia'/exp",24029,24 Nov 2020

#281,"'neutropenia'/exp",121899,24 Nov 2020

#280,"'thrombocytopenia'/exp",182167,24 Nov 2020

#279,"'anemia'/exp",402922,24 Nov 2020

#278,"(pe:ti,ab,kw OR pes:ti,ab,kw OR pe:tt OR pes:tt) AND (lung* OR pulmo* OR pneumo* OR embol* OR thromboembol* OR thrombot* OR thrombus OR thrombi OR microembol*)",23243,24 Nov 2020

#277,"(((lung* OR pulmonary) NEAR/3 (embol* OR thromboemboli* OR 'thromb* emboli*' OR microemboli* OR clot* OR thrombus OR thrombi OR microthromb*)):ti,ab,kw) OR (((lung* OR pulmonary) NEAR/3 (embol* OR thromboemboli* OR 'thromb* emboli*' OR microemboli* OR clot* OR thrombus OR thrombi OR microthromb*)):tt)",77601,24 Nov 2020

#276,"'pulmonary infarct*':ti,ab,kw OR 'pulmonary infarct*':tt",1453,24 Nov 2020

#275,"dvt:ti,ab,kw OR dvts:ti,ab,kw OR vte:ti,ab,kw OR vtes:ti,ab,kw OR dvt:tt OR dvts:tt OR vte:tt OR vtes:tt",37981,24 Nov 2020

#274,"((venous* OR venos* OR vein*) NEAR/1 (thrombos?s* OR thrombotic* OR thromboemboli* OR 'thrombo emboli*' OR thrombus OR thrombi OR microthromb*)):tt",1445,24 Nov 2020

#273,"((venous* OR venos* OR vein*) NEAR/1 (thrombos?s* OR thrombotic* OR thromboemboli* OR 'thrombo emboli*' OR thrombus OR thrombi OR microthromb*)):ti,ab,kw",108846,24 Nov 2020

#272,"'venous thromboembolism'/exp",163231,24 Nov 2020

#271,"'bmi'/de OR 'body mass index':ti,ab,kw OR bmi:ti,ab,kw OR 'body mass index':tt OR bmi:tt",462705,24 Nov 2020

#270,"'body mass'/de",455875,24 Nov 2020

#269,"'hemoglobin a1c'/exp OR 'hb a1c' OR 'glycated haemoglobin a1c' OR 'glycated hemoglobin a1c' OR 'glycosylated haemoglobin a1c' OR 'glycosylated hemoglobin a1c' OR 'haemoglobin a1c' OR 'haemoglobin a (1c)' OR 'haemoglobin a 1c' OR 'haemoglobin aic' OR 'hb a (1c)' OR 'hba 1c' OR 'hba1c'

OR 'hemoglobin a1c' OR 'hemoglobin a (1c)' OR 'hemoglobin a 1c' OR 'hemoglobin aic'",125583,24 Nov 2020

#268,"diabetes*:ti,ab,kw OR diabetic*:ti,ab,kw OR diabetes*:tt OR diabetic*:tt",991196,24 Nov 2020

#267,"'diabetes mellitus'/exp",1024534,24 Nov 2020

#266,"(metabolic NEAR/3 (effect$ OR alterat* OR chang* OR risk$ OR transition* OR transformat* OR parameter$ OR marker$ OR biomarker$)):ti,ab,kw",116211,24 Nov 2020

#265,"'metabolic parameters'/exp",317917,24 Nov 2020

#264,"'metabolic effects'/de",223,24 Nov 2020

#263,"hyperlipid$emi*:ti,ab,kw OR lip$edemi*:ti,ab,kw OR hyperlipid$emi*:tt OR lip$edemi*:tt",52557,24 Nov 2020

#262,"'hyperlipidemia'/exp",165050,24 Nov 2020

#261,"'low density lipoprotein cholesterol'/exp OR 'ldl cholesterol' OR 'cholesterol, ldl' OR 'lipoproteins, ldl cholesterol' OR 'low density lipoprotein cholesterol'",123396,24 Nov 2020

#260,"'high density lipoprotein cholesterol'/de OR 'hdl cholesterol' OR 'cholesterol, hdl' OR 'high density lipoprotein cholesterol' OR 'lipoproteins, hdl cholesterol'",127555,24 Nov 2020

#259,"'lipoprotein a'/exp OR 'lipoprotein (a)' OR 'lipoprotein a'",12688,24 Nov 2020

#258,"hdl:ti,ab,kw OR ldl:ti,ab,kw OR hdl:tt OR ldl:tt",167427,24 Nov 2020

#257,"((lipid OR cholesterol* OR cholesterin* OR triglyceride* OR triaglycer* OR lipoprotein* OR fat) NEAR/3 (level$ OR blood OR plasma OR serum)):tt",271,24 Nov 2020

#256,"((lipid OR cholesterol* OR cholesterin* OR triglyceride* OR triaglycer* OR lipoprotein* OR fat) NEAR/3 (level$ OR blood OR plasma OR serum)):ti,ab,kw",238088,24 Nov 2020

#255,"'lipid blood level'/exp",209025,24 Nov 2020

#254,"'lipid level'/exp",20947,24 Nov 2020

#253,"'liver function':ti,ab,kw OR 'liver function':tt",58581,24 Nov 2020

#252,"uln:ti,ab,kw,de AND (liver*:ti,ab,kw,de OR hepat*:ti,ab,kw,de)",2605,24 Nov 2020

#251,"((elevat* NEAR/6 transaminas*):ti,ab,kw) OR ((elevat* NEAR/6 transaminas*):tt)",8914,24 Nov 2020

#250,"'hypertransaminasemi*':ti,ab,kw OR 'hyper transaminasemi*':ti,ab,kw OR 'hypertransaminasemi*':tt OR 'hyper transaminasemi*':tt",758,24 Nov 2020

#249,"'hypertransaminasemia'/de",18203,24 Nov 2020

#248,"'liver function test'/de",48385,24 Nov 2020

#247,"'liver function'/exp",83870,24 Nov 2020

#246,"((('digestive system' OR gastrointestinal OR 'gastro intestinal*' OR intestinal*) NEAR/3 perforation$):ti,ab,kw) OR ((('digestive system' OR gastrointestinal* OR 'gastro intestinal*' OR intestinal*l) NEAR/3 perforation$):tt)",7050,24 Nov 2020

#245,"'digestive system perforation'/exp",51307,24 Nov 2020

#244,"diverticulitis*:ti,ab,kw OR diverticulitis*:tt",9856,24 Nov 2020

#243,"'digestive system perforation'/exp",51307,24 Nov 2020

#242,"'diverticulitis'/de",8715,24 Nov 2020

#241,"'malignanc*':ti,ab,kw OR 'malignanc*':tt",392042,24 Nov 2020

#240,"'malignancies'/de",29,24 Nov 2020

#239,"((solid NEAR/6 malignan* NEAR/3 (tumo$r$ OR cancer OR neoplasm$)):ti,ab,kw) OR ((solid NEAR/6 malignan* NEAR/3 (tumo$r$ OR cancer OR neoplasm$)):tt)",6409,24 Nov 2020

#238,"((h$ematol* NEAR/6 malignan*):ti,ab,kw) OR ((h$ematol* NEAR/6 malignan*):tt)",51047,24 Nov 2020

#237,"(malignan* NEAR/6 (tumo$r$ OR cancer OR neoplasm$)):tt",18,24 Nov 2020

#236,"(malignan* NEAR/6 (tumo$r$ OR cancer OR neoplasm$)):ti,ab,kw",241515,24 Nov 2020

#235,"(('non melanoma' OR nonmelanoma) NEAR/3 'skin' NEAR/3 (cancer OR tumo$r* OR neoplasm$)):tt",2,24 Nov 2020

#234,"(('non melanoma' OR nonmelanoma) NEAR/3 'skin' NEAR/3 (cancer OR tumo$r* OR neoplasm$)):ti,ab,kw",6407,24 Nov 2020

#233,"'non melanoma skin cancer'/exp",74264,24 Nov 2020

#232,"lymphom*:ti,ab,kw OR leuk$emi*:ti,ab,kw OR myelom*:ti,ab,kw OR lymphom*:tt OR leuk$emi*:tt OR myelom*:tt",664357,24 Nov 2020

#231,"'hematologic malignancy'/exp OR 'blood disease, malignant' OR 'haematologic malignancy' OR 'haematological malignancy' OR 'hematologic malignancy' OR 'hematological malignancy' OR 'malignant blood disease' OR 'malignant haematologic disease' OR 'malignant haematological disease' OR 'malignant hematologic disease' OR 'malignant hematological disease'",736636,24 Nov 2020

#230,"'malignant neoplasm'/exp",3825208,24 Nov 2020

#229,"(wbc:ti,ab,kw OR wbc:tt) AND ('leucocyte* count*':ti,ab,kw,de OR 'white blood cell count*':ti,ab,kw,de OR 'white cell count*':ti,ab,kw,de)",4289,24 Nov 2020

#228,"(wcc:ti,ab,kw OR wcc:tt) AND ('leucocyte*' OR 'white cell count*')",555,24 Nov 2020

#227,"'leukocyte count'/exp OR 'wbc count' OR 'wbc counts' OR 'leucocyte count' OR 'leucocyte counting' OR 'leucocyte counts' OR 'leukocyte count' OR 'leukocyte counts' OR 'total leucocyte count' OR 'total leukocyte count' OR 'white blood cell (wbc) count' OR 'white blood cell (wbc) counts' OR

'white blood cell count' OR 'white blood cell counts' OR 'white blood count' OR 'white cell count' OR 'white cell counts'",245173,24 Nov 2020

#226,"'procalcitonin'/de OR 'calcitonin precursor' OR 'procalcitonin'",14501,24 Nov 2020

#225,"'procalcitonin blood level'/de",71,24 Nov 2020

#224,"(esr:ti,ab,kw OR esr:tt) AND 'sedimentation rate$':ti,ab,kw",9260,24 Nov 2020

#223,"'erythrocyte sedimentation rate'/de OR 'blood sedimentation' OR 'blood sedimentation rate' OR 'erythrocyte sedimentation rate' OR 'sedimentation rate, erythrocyte'",54360,24 Nov 2020

#222,"'c reactive protein'/exp OR 'c reactive protein' OR 'c reaction protein' OR 'c-reactive protein' OR 'creactive protein' OR 'crp' OR 'protein, c reactive' OR 'serum c reactive protein'",229058,24 Nov 2020

#221,"'c reactive protein blood level'/de",208,24 Nov 2020

#220,"#218 AND #219",8411,24 Nov 2020

#219,"reactivat*:ti,ab,kw,de OR reactivat*:tt",64828,24 Nov 2020

#218,"#213 OR #214 OR #215 OR #216 OR #217",130571,24 Nov 2020

#217,"(herpes:ti,ab,kw OR 'herpes vulgaris':ti,ab,kw OR 'herpes simplex':ti,ab,kw OR herpes:tt OR 'herpes vulgaris':tt OR 'herpes simplex':tt) AND infect*",49549,24 Nov 2020

#216,"(hsv:ti,ab,kw OR hsv:tt) AND (infect*:ti,ab,kw OR herpes:ti,ab,kw,de)",28988,24 Nov 2020

#215,"'herpes simplex'/exp OR 'herpes' OR 'herpes infection' OR 'herpes simplex' OR 'herpes simplex infection' OR 'herpes simplex viral infection' OR 'herpes simplex virus infection' OR 'herpes vulgaris'",121760,24 Nov 2020

#214,"'herpes zoster':ti,ab,kw,de OR 'varicella zoster':ti,ab,kw,de OR zoster:ti,ab,kw,de OR shingles:ti,ab,kw,de OR 'varicellovirus infect*':ti,ab,kw,de OR 'herpes zoster':tt OR 'varicella zoster':tt OR zoster:tt OR shingles:tt OR 'varicellovirus infect*':tt",41395,24 Nov 2020

#213,"'herpes zoster'/exp",27143,24 Nov 2020

#212,"#210 AND #211",467285,24 Nov 2020

#211,"opportunistic:ti,ab,kw OR serious*:ti,ab,kw OR severe*:ti,ab,kw OR severit*:ti,ab,kw OR opportunistic:tt OR serious*:tt OR severe*:tt OR severit*:tt",2438020,24 Nov 2020

#210,"'infection'/exp",3779142,24 Nov 2020

#209,"fung*:tt OR mycosis:tt OR mycoses:tt OR mycotic:tt OR 'hepatitis b':tt OR 'hepatitis type b':tt OR 'type b hepatitis':tt OR hbv:tt OR ((hepatitis NEAR/3 'b'):tt)",5420,24 Nov 2020

#208,"fung*:ti,ab,kw OR mycosis:ti,ab,kw OR mycoses:ti,ab,kw OR mycotic:ti,ab,kw OR 'hepatitis b':ti,ab,kw OR 'hepatitis type b':ti,ab,kw OR 'type b hepatitis':ti,ab,kw OR hbv:ti,ab,kw OR ((hepatitis NEAR/3 'b'):ti,ab,kw)",410769,24 Nov 2020

#207,"'hepatitis b'/exp OR 'hepatitis b' OR 'hepatitis b virus infection' OR 'hepatitis, serum' OR 'hippie hepatitis' OR 'injection hepatitis' OR 'serum hepatitis' OR 'type b hepatitis' OR 'viral hepatitis type b' OR 'virus hepatitis type b'",166943,24 Nov 2020

#206,"'mycosis'/exp OR 'deep mycosis' OR 'fungal disease' OR 'fungal infection' OR 'fungal infections' OR 'fungus infection' OR 'mycoses' OR 'mycosis' OR 'mycosis infection' OR 'mycotic disease' OR 'mycotic infection'",230545,24 Nov 2020

#205,"'fungus reactivation'",2,24 Nov 2020

#204,"'fungal reactivation'",5,24 Nov 2020

#203,"'atypical mycobacteriosis'/de OR 'mycobacterium avium complex infection' OR 'mycobacterium avium infection' OR 'mycobacterium avium-intracellulare complex infection' OR 'mycobacterium avium-intracellulare infection' OR 'mycobacterium infections, atypical' OR 'mycobacterium intracellulare infection' OR 'mycobacterium kansasii infection' OR 'atypical mycobacterium infection'

OR 'atypical mycobacterial infection' OR 'atypical mycobacteriosis' OR 'atypical mycobacterium infections' OR 'atypical tuberculosis' OR 'mycobacteriosis, atypical' OR 'mycobacterium infections, nontuberculous' OR 'nontuberculous mycobacterium infection' OR 'nontuberculous mycobacterium infections'",7000,24 Nov 2020

#202,"(cmv:ti,ab,kw OR cmv:tt) AND (virus:ti,ab,kw OR viral:ti,ab,kw OR infect*:ti,ab,kw)",33935,24 Nov 2020

#201,"'cytomegalovirus infection'/exp OR 'cmv disease' OR 'cmv infection' OR 'cmv infections' OR 'cmv syndrome' OR 'cytomegalovirus disease' OR 'cytomegalovirus infections' OR 'cytomegaly virus disease' OR 'cytomegaly virus infection' OR 'congenital cytomegalic inclusion' OR 'congenital cytomegalic inclusion body disease' OR 'congenital cytomegalic inclusion disease' OR 'cytomegalic inclusion body disease' OR 'cytomegalic inclusion disease' OR 'cytomegaloinfection' OR 'cytomegaloviral infection' OR 'cytomegalovirus infection'",47316,24 Nov 2020

#200,"'tuberculosis':ti,ab,kw OR 'tuberculous':ti,ab,kw OR 'tuberculosis':tt OR 'tuberculous':tt",257093,24 Nov 2020

#199,"'tuberculosis'/exp",268935,24 Nov 2020

#198,"infect*:ti,ab,kw OR infect*:tt",2310510,24 Nov 2020

#197,"((((overall OR rate OR risk$) NEAR/8 (viral* OR virus OR bacterial* OR bacterious OR microbial* OR fungal OR mycos$s OR zoonosis)):ti,ab,kw) OR (((overall OR rate OR risk$) NEAR/3 (viral* OR virus OR bacterial* OR bacterious OR microbial* OR fungal OR mycos$s OR zoonosis)):tt)) AND infect*",51543,24 Nov 2020

#196,"(((overall OR rate OR risk$) NEAR/8 (infect* OR bacter$emi* OR septic$emi* OR sepsis OR 'bacterial infect*' OR 'bacterial diseas*' OR 'bacterious infect*' OR 'viral infect*' OR 'virus diseas*' OR

'virus infect*' OR 'microbial infect*' OR mycos$s OR 'fung* infect*' OR 'mycotic diseas*' OR 'mycos$s infect*' OR fung$emi*)):ti,ab,kw) OR (((overall OR rate OR risk$) NEAR/6 (infect* OR bacter$emi* OR septic$emi* OR sepsis OR 'bacterial infect*' OR 'bacterial diseas*' OR 'bacterious infect*' OR 'viral infect*' OR 'virus diseas*' OR 'virus infect*' OR 'microbial infect*' OR mycos$s OR 'fung* infect*' OR 'mycotic diseas*' OR 'mycos$s infect*' OR fung$emi*)):tt)",254441,24 Nov 2020

#195,"(((opportunistic* OR opportune) NEAR/6 (infect* OR bacter$emi* OR septic$emi* OR 'bacterial infect*' OR 'bacterial diseas*' OR 'viral infect*' OR 'virus diseas*' OR 'virus infect*' OR 'microbial infect*' OR mycos$s OR 'fung* infect*' OR 'mycotic diseas*' OR 'mycos$s infect*' OR fung$emi*)):ti,ab,kw) OR (((opportunistic* OR opportune) NEAR/6 (infect* OR bacter$emi* OR septic$emi* OR 'bacteri* infect*' OR 'bacteri* diseas*' OR 'viral infect*' OR 'virus diseas*' OR 'virus infect*' OR 'microbial infect*' OR mycos$s OR 'fung* infect*' OR 'mycotic diseas*' OR 'mycos$s infect*' OR fung$emi*)):tt)",28170,24 Nov 2020

#194,"'opportunistic infection'/de",27120,24 Nov 2020

#193,"(((serious* OR severe* OR severit*) NEAR/8 (infect* OR bacter$emi* OR septic$emi* OR sepsis OR fung$emi* OR 'fungal infect*' OR 'fungal disease$' OR mycos$s)):ti,ab,kw) OR (((serious* OR severe* OR severit*) NEAR/8 (infect* OR bacter$emi* OR septic$emi* OR sepsis OR fung$emi* OR 'fungal infect*' OR mycos$s OR 'fungal disease$')):tt)",141542,24 Nov 2020

#192,"'serious infection'/de",11,24 Nov 2020

#191,"'infection rate'/de",31767,24 Nov 2020

#190,"'infection'/exp/mj",2416047,24 Nov 2020

#189,"mortality:ti,ab,kw OR mortality:tt",1156774,24 Nov 2020

#188,"((mortality OR death$) NEAR/4 (rate$ OR ratio OR caus* OR risk OR sudden OR number* OR amount* OR count*)):tt",60,24 Nov 2020

#187,"((mortality OR death$) NEAR/4 (rate$ OR ratio OR caus* OR risk OR sudden OR number* OR amount* OR count*)):ti,ab,kw",795953,24 Nov 2020

#186,"'fatality'/de",90014,24 Nov 2020

#185,"death:ti,ab,kw OR deaths:ti,ab,kw OR death:tt OR deaths:tt",1209305,24 Nov 2020

#184,"'death'/de OR 'death'/exp/mj",386631,24 Nov 2020

#183,"'sudden death'/de",44326,24 Nov 2020

#182,"'mortality'/exp/mj",164167,24 Nov 2020

#181,"'mortality'/de",783670,24 Nov 2020

#180,"'cause of death'/de",117909,24 Nov 2020

#179,"'standardized mortality ratio'/de",3016,24 Nov 2020

#178,"'mortality risk'/de",21895,24 Nov 2020

#177,"'all cause mortality'/de",33252,24 Nov 2020

#176,"'mortality rate'/exp",68171,24 Nov 2020

#175,"((treatment NEAR/3 emergent):ti,ab,kw) OR ((treatment NEAR/3 emergent):tt)",13302,24 Nov 2020

#174,"teratogen*:ti,ab,kw OR teratogen*:tt",23832,24 Nov 2020

#173,"'teratogenicity'/de",17398,24 Nov 2020

#172,"(((drug OR chemical*) NEAR/1 induced):ti,ab,kw) OR (((drug OR chemical*) NEAR/1 induced):tt)",71433,24 Nov 2020

#171,"'drug induced malformation'/de",110,24 Nov 2020

#170,"((toxic* NEAR/6 (drug$ OR medication$)):ti,ab,kw) OR ((toxic* NEAR/6 (drug$ OR medication$)):tt)",53083,24 Nov 2020

#169,"'drug toxicity and intoxication'/exp",178224,24 Nov 2020

#168,"'anaphyla*':ti,ab,kw OR anaphyla*:tt",43461,24 Nov 2020

#167,"'anaphylaxis'/exp",54207,24 Nov 2020

#166,"hypersensitiv*:ti,ab,kw OR 'hyper sensitiv*':ti,ab,kw OR hypersensitiv*:tt OR 'hyper sensitiv*':tt",101554,24 Nov 2020

#165,"'drug hypersensitivity'/exp",62723,24 Nov 2020

#164,"'side effect$':ti,ab,kw OR 'side effect$':tt",378161,24 Nov 2020

#163,"'side effect'/exp",574650,24 Nov 2020

#162,"safety:ti,ab,kw OR safety:tt",826797,24 Nov 2020

#161,"'drug safety'/exp",423068,24 Nov 2020

#160,"withdraw*:ti,ab,kw OR withdraw*:tt",181298,24 Nov 2020

#159,"'drug withdrawal'/de",202021,24 Nov 2020

#158,"adverseevent*:ti,ab,kw OR adversedrugreaction*:ti,ab,kw OR adversereaction*:ti,ab,kw OR adverseincident*:ti,ab,kw OR adverseoutcome*:ti,ab,kw",1173,24 Nov 2020

#157,"(saes:tt OR aes:tt OR adrs:tt OR sae:tt OR 'ae':tt OR adr:tt) AND adverse:ti,ab,kw,de",14,24 Nov 2020

#156,"(saes:ti,ab,kw OR aes:ti,ab,kw OR adrs:ti,ab,kw OR sae:ti,ab,kw OR 'ae':ti,ab,kw OR adr:ti,ab,kw) AND adverse:ti,ab,kw,de",55573,24 Nov 2020

#155,"(((serious* OR severe* OR severit*) NEAR/3 (adverse OR saes OR aes OR adrs OR sae OR 'ae' OR adr)):ti,ab,kw) OR (((serious* OR severe* OR severit*) NEAR/3 (adverse OR saes OR aes OR adrs OR sae OR 'ae' OR adr)):tt)",84186,24 Nov 2020

#154,"(adverse NEAR/3 (event$ OR effect$ OR reaction$ OR incident$ OR outcome$)):tt",53,24 Nov 2020

#153,"(adverse NEAR/3 (event$ OR effect$ OR reaction$ OR incident$ OR outcome$)):ti,ab,kw",720474,24 Nov 2020

#152,"'adverse event'/de OR 'adverse drug reaction'/exp",617132,24 Nov 2020

#151,"'adverse drug reaction'/lnk OR 'complication'/lnk OR 'drug toxicity'/lnk OR 'side effect'/lnk",3550888,24 Nov 2020

#150,"#72 OR #149",107829,24 Nov 2020

#149,"#61 AND #148",106163,24 Nov 2020

#148,"#73 OR #74 OR #75 OR #76 OR #77 OR #78 OR #79 OR #80 OR #81 OR #82 OR #83 OR #84 OR #85 OR #86 OR #87 OR #88 OR #89 OR #90 OR #91 OR #92 OR #93 OR #94 OR #95 OR #96 OR #97 OR #98 OR #99 OR #100 OR #101 OR #102 OR #103 OR #104 OR #105 OR #106 OR #107 OR #108 OR #109 OR #110 OR #111 OR #112 OR #113 OR #114 OR #115 OR #116 OR #117 OR #118 OR #119 OR #120 OR #121 OR #122 OR #123 OR #124 OR #125 OR #126 OR #127 OR #128 OR #129 OR #130 OR #131 OR #132 OR #133 OR #134 OR #135 OR #136 OR #137 OR #138 OR #139 OR #140 OR #141 OR #142 OR #143 OR #144 OR #145 OR #146 OR #147",1461517,24 Nov 2020

#147,"'peficitinib'/de OR '4 [(5 hydroxyadamantan 2 yl) amino] 1h pyrrolo [2, 3 b] pyridine 5 carboxamide' OR '4 [(5 hydroxytricyclo [3.3.1.1 3, 7] dec 2 yl) amino] 1h pyrrolo [2, 3 b] pyridine 5

carboxamide' OR 'asp 015k' OR 'asp015k' OR 'peficitinib' OR 'peficitinib hydrobromide'",185,24 Nov 2020

#146,"'ritlecitinib'/exp OR '1 [2 methyl 5 (7h pyrrolo [2, 3 d] pyrimidin 4 ylamino) 1 piperidinyl] prop 2 en 1 one' OR '1 [2 methyl 5 (7h pyrrolo [2, 3 d] pyrimidin 4 ylamino) piperidin 1 yl] prop 2 en 1 one' OR '1 [2 methyl 5 [(7h pyrrolo [2, 3 d] pyrimidin 4 yl) amino] 1 piperidinyl] prop 2 en 1 one' OR '1 [2 methyl 5 [(7h pyrrolo [2, 3 d] pyrimidin 4 yl) amino] piperidin 1 yl] prop 2 en 1 one' OR '1 [5 (7h pyrrolo [2, 3 d] pyrimidin 4 ylamino) 2 methyl 1 piperidinyl] prop 2 en 1 one' OR '1 [5 (7h pyrrolo [2, 3 d] pyrimidin 4 ylamino) 2 methylpiperidin 1 yl] prop 2 en 1 one' OR '1 [5 [(7h pyrrolo [2, 3 d] pyrimidin 4 yl) amino] 2 methyl 1 piperidinyl] prop 2 en 1 one' OR '1 [5 [(7h pyrrolo [2, 3 d] pyrimidin 4 yl) amino] 2 methylpiperidin 1 yl] prop 2 en 1 one' OR 'pf 06651600' OR 'pf 6651600' OR 'pf06651600' OR 'pf6651600' OR 'ritlecitinib' OR 'ritlecitinib malonate' OR 'ritlecitinib propanedioate'",39,24 Nov 2020

#145,"'fedratinib'/de OR 'fedratinib' OR 'fedratinib dihydrochloride' OR 'fedratinib dihydrochloride monohydrate' OR 'fedratinib hydrochloride' OR 'inrebic' OR 'n (1, 1 dimethylethyl) 3 [[5 methyl 2 [[4 [2 (1 pyrrolidinyl) ethoxy] phenyl] amino] 4 pyrimidinyl] amino] benzenesulfonamide' OR 'n tert butyl 3 [[5 methyl 2 [[4 (2 pyrrolidin 1 ylethoxy) phenyl] amino] pyrimidin 4 yl] amino] benzenesulfonamide' OR 'n tert butyl 3 [[5 methyl 2 [4 [2 (pyrrolidin 1 yl) ethoxy] anilino] pyrimidin 4 yl] amino] benzenesulfonamide' OR 'n tert butyl 3 [5 methyl 2 [4 (2 pyrrolidin 1 ylethoxy) phenylamino] pyrimidin 4 ylamino] benzenesulfonamide' OR 'n tert butyl 3 [5 methyl 2 [4 [2 (1 pyrrolidinyl) ethoxy] phenylamino] 4 pyrimidinylamino] benzenesulfonamide' OR 'sar 302503' OR 'sar 302503a' OR 'sar302503' OR 'sar302503a' OR 'tg 101348' OR 'tg101348'",649,24 Nov 2020

#144,"'n benzyl 2 cyano 3 (3,4 dihydroxyphenyl)acrylamide'/exp OR '2 cyano 3 (3, 4 dihydroxyphenyl) n (phenylmethyl) 2 propenamide' OR 'ag 490' OR 'ag490' OR 'n benzyl 2 cyano 3 (3,4 dihydroxyphenyl)acrylamide' OR 'tyrphostin ag 490' OR 'tyrphostin ag490'",2477,24 Nov 2020
#143,"'itacitinib'/de OR '1 [1 [[3 fluoro 2 (trifluoromethyl) 4 pyridinyl] carbonyl] 4 piperidinyl] 3 [4 (7h pyrrolo [2, 3 d] pyrimidin 4 yl) 1h pyrazol 1 yl] 3 azetidineacetonitrile' OR '1 [1 [[3 fluoro 2 (trifluoromethyl) pyridin 4 yl] carbonyl] piperidin 4 yl] 3 [4 (7h pyrrolo [2, 3 d] pyrimidin 4 yl) 1h pyrazol 1 yl] 3 azetidineacetonitrile' OR '[1 [1 (3 fluoro 2 trifluoromethylpyridine 4 carbonyl) 4 piperidinyl] 3 [4 (7h pyrrolo [2, 3 d] pyrimidin 4 yl) 1h pyrazol 1 yl] 3 azetidinyl] ethanenitrile' OR '[1 [1 [3 fluoro 2 (trifluoromethyl) pyridine 4 carbonyl] piperidin 4 yl] 3 [4 (7h pyrrolo [2, 3 d] pyrimidin 4 yl) 1h pyrazol 1 yl] azetidin 3 yl] ethanenitrile' OR 'incb 039110' OR 'incb 39110' OR 'incb039110' OR 'incb39110' OR 'itacitinib' OR 'itacitinib adipate' OR 'itacitinib hexanedioate'",183,24 Nov 2020
#142,"'ruxolitinib'/de OR '3 [4 (7h pyrrolo [2, 3 d] pyrimidin 4 yl) 1h pyrazol 1 yl] 3 cyclopentylpropanenitrile' OR '3 [4 (7h pyrrolo [2, 3 d] pyrimidin 4 yl) 1h pyrazol 1 yl] 3 cyclopentylpropanenitrile phosphate' OR '3 cyclopentyl 3 [4 (7h pyrrolo [2, 3 d] pyrimidin 4 yl) 1h pyrazol 1 yl] propanenitrile' OR 'beta cyclopentyl 4 (7h pyrrolo [2, 3 d] pyrimidin 4 yl) 1h pyrazole 1 propanenitrile' OR 'incb 018424' OR 'incb 18424' OR 'incb 424' OR 'incb018424' OR 'incb18424' OR 'incb424' OR 'jakafi' OR 'jakavi' OR 'ruxolitinib' OR 'ruxolitinib maleate' OR 'ruxolitinib phosphate'",5337,24 Nov 2020
#141,"'upadacitinib'/de OR '3 ethyl 4 (3h imidazo [1, 2 a] pyrrolo [2, 3 e] pyrazin 8 yl) n (2, 2, 2 trifluoroethyl) 1 pyrrolidinecarboxamide' OR '3 ethyl 4 (3h imidazo [1, 2 a] pyrrolo [2, 3 e] pyrazin 8 yl) n (2, 2, 2 trifluoroethyl) 1 pyrrolidinecarboxamide 2, 3 dihydroxybutanedioate' OR '3 ethyl 4 (3h imidazo [1, 2 a] pyrrolo [2, 3 e] pyrazin 8 yl) n (2, 2, 2 trifluoroethyl) 1 pyrrolidinecarboxamide tartrate' OR '3

ethyl 4 (3h imidazo [1, 2 a] pyrrolo [2, 3 e] pyrazin 8 yl) n (2, 2, 2 trifluoroethyl) pyrrolidine 1 carboxamide' OR '3 ethyl 4 (3h imidazo [1, 2 a] pyrrolo [2, 3 e] pyrazin 8 yl) n (2, 2, 2 trifluoroethyl) pyrrolidine 1 carboxamide 2, 3 dihydroxybutanedioate' OR '3 ethyl 4 (3h imidazo [1, 2 a] pyrrolo [2, 3 e] pyrazin 8 yl) n (2, 2, 2 trifluoroethyl) pyrrolidine 1 carboxamide tartrate' OR 'abt 494' OR 'abt494' OR 'rinvoq' OR 'upadacitinib' OR 'upadacitinib 2, 3 dihydroxybutanedioate' OR 'upadacitinib hemihydrate' OR 'upadacitinib hydrate' OR 'upadacitinib tartrate'",548,24 Nov 2020

#140,"'filgotinib'/de OR 'filgotinib' OR 'filgotinib 2 butenedioate' OR 'filgotinib hydrochloride' OR 'filgotinib maleate' OR 'g 146034' OR 'g 146034 101' OR 'g 146034-101' OR 'g146034' OR 'g146034 101' OR 'g146034-101' OR 'glpg 0634' OR 'glpg0634' OR 'gs 6034' OR 'gs6034' OR 'n [5 [4 (1, 1 dioxothiomorpholinomethyl) phenyl] 1, 2, 4 triazolo [1, 5 a] pyridin 2 yl] cyclopropanecarboxamide' OR 'n [5 [4 (1, 1 dioxothiomorpholinomethyl) phenyl] 1, 2, 4 triazolo [1, 5 a] pyridin 2 yl] cyclopropanecarboxamide 2 butenedioate' OR 'n [5 [4 (1, 1 dioxothiomorpholinomethyl) phenyl] 1, 2, 4 triazolo [1, 5 a] pyridin 2 yl] cyclopropanecarboxamide but 2 enedioate' OR 'n [5 [4 [(1, 1 dioxido 4 thiomorpholinyl) methyl] phenyl] 1, 2, 4 triazolo [1, 5 a] pyridin 2 yl] cyclopropanecarboxamide' OR 'n [5 [4 [(1, 1 dioxido 4 thiomorpholinyl) methyl] phenyl] 1, 2, 4 triazolo [1, 5 a] pyridin 2 yl] cyclopropanecarboxamide 2 butenedioate' OR 'n [5 [4 [(1, 1 dioxothiomorpholin 4 yl) methyl] phenyl] 1, 2, 4 triazolo [1, 5 a] pyridin 2 yl] cyclopropanecarboxamide' OR 'n [5 [4 [(1, 1 dioxothiomorpholin 4 yl) methyl] phenyl] 1, 2, 4 triazolo [1, 5 a] pyridin 2 yl] cyclopropanecarboxamide but 2 enedioate' OR 'n [5 [4 [(1, 1 dioxothiomorpholin 4 yl) methyl] phenyl] [1, 2, 4] triazolo [1, 5 a] pyridin 2 yl] cyclopropanecarboxamide' OR 'n [5 [4 [(1, 1 dioxothiomorpholin 4 yl) methyl] phenyl] [1, 2, 4] triazolo [1, 5 a] pyridin 2 yl] cyclopropanecarboxamide but 2 enedioate'",505,24 Nov 2020

#139,"'tofacitinib'/de OR '1 cyanoacetyl 4 methyl n methyl n (1h pyrrolo [2, 3 d] pyrimidin 4 yl) 3 piperidinamine' OR '3 [4 methyl 3 [methyl (7h pyrrolo [2, 3 d] pyrimidin 4 yl) amino] 1 piperidinyl] 3 oxopropanenitrile' OR '4 [n [1 (2 cyano 1 oxoethyl) 4 methyl 3 piperidinyl] n methylamino] pyrrolo [2, 3 d] pyrimidine' OR '4 methyl 3 [methyl (7h pyrrolo [2, 3 d] pyrimidin 4 yl) amino] beta oxo 1 piperidinepropanenitrile' OR 'cp 690 550' OR 'cp 690, 550' OR 'cp 690550' OR 'cp 690550 10' OR 'cp 690550-10' OR 'cp690 550' OR 'cp690, 550' OR 'cp690550' OR 'cp690550 10' OR 'cp690550-10' OR 'tasocitinib' OR 'tasocitinib citrate' OR 'tofacitinib' OR 'tofacitinib citrate' OR 'xeljanz' OR 'xeljanz xr'",5249,24 Nov 2020

#138,"'baricitinib'/de OR '1 (ethylsulfonyl) 3 [4 (7h pyrrolo [2, 3 d] pyrimidin 4 yl) 1h pyrazol 1 yl] 3 azetidineacetonitrile' OR '[1 (ethanesulfonyl) 3 [4 (7h pyrrolo [2, 3 d] pyrimidin 4 yl) 1h pyrazol 1 yl] azetidin 3 yl] ethanenitrile' OR '[1 (ethylsulfonyl) 3 [4 (1h pyrrolo [2, 3 d] pyrimidin 4 yl) 1h pyrazol 1 yl] 3 azetidinyl] acetonitrile' OR '[1 (ethylsulfonyl) 3 [4 (7h pyrrolo [2, 3 d] pyrimidin 4 yl) 1h pyrazol 1 yl] azetidin 3 yl] ethanenitrile' OR 'baricitinib' OR 'incb 028050' OR 'incb 28050' OR 'incb028050' OR 'incb28050' OR 'ly 3009104' OR 'ly3009104' OR 'olumiant'",1534,24 Nov 2020

#137,"(janus NEAR/4 kinase NEAR/10 inhibitor$):tt",4,24 Nov 2020

#136,"(janus NEAR/4 kinase NEAR/10 inhibitor$):ti,ab,kw,tn",3315,24 Nov 2020

#135,"((jak OR jak1 OR jak2 OR jak3 OR 'janus kinase') NEAR/10 inhibitor*):tt",22,24 Nov 2020

#134,"((jak OR jak1 OR jak2 OR jak3 OR 'janus kinase') NEAR/10 inhibitor*):ti,ab,kw,tn",11237,24 Nov 2020

#133,"'janus kinase inhibitor'/exp OR 'jak inhibitor' OR 'janus kinase inhibitor' OR 'janus kinase inhibitors' OR 'janus tyrosine kinase inhibitor'",16156,24 Nov 2020

#132,"(steroid$ NEAR/10 (therap* OR treat* OR drug$ OR inject* OR intramuscular* OR 'intra muscular*' OR im OR intravenous* OR 'intra venous*' OR iv OR intraarticular* OR 'intra articular*' OR ia OR administrat* OR oral OR orally OR parenteral*)):tt",147,24 Nov 2020

#131,"(steroid$ NEAR/10 (therap* OR treat* OR drug$ OR inject* OR intramuscular* OR 'intra muscular*' OR im OR intravenous* OR 'intra venous*' OR iv OR intraarticular* OR 'intra articular*' OR ia OR administrat* OR oral OR orally OR parenteral*)):ti,ab,kw",118160,24 Nov 2020

#130,"'triamcinolone'/exp OR '9alpha fluoro 1, 4 pregnadiene 3, 20 dione 11beta, 16alpha, 17alpha, 21 tetrol' OR '9alpha fluoro 11beta, 16alpha, 17alpha, 21 tetrahydroxypregna 1, 4 diene 3, 20 dione' OR '9alpha fluoro 16alpha hydroxyhydrocortisone' OR '9alpha fluoro 16alpha hydroxyprednisolone' OR 'acetocot' OR 'adcortyl' OR 'aristocort' OR 'aristocort forte' OR 'aristodan' OR 'azmacor' OR 'celeste' OR 'cl 19823' OR 'cl19823' OR 'clinacort' OR 'clinalog' OR 'delphicort' OR 'fluoxiprednisolone' OR 'fluoxyprednisolone' OR 'ken-jec 40' OR 'kenacort' OR 'kenacort retard' OR 'korticoid' OR 'ledercort' OR 'omcilon' OR 'polcortolon' OR 'rp 8357' OR 'rp8357' OR 'simacort' OR 'sterocort' OR 'tac 3' OR 'tramcinolone' OR 'triacortyl' OR 'triam-a' OR 'triam-forte' OR 'triamcinolon' OR 'triamcinolona' OR 'triamcinolone*' OR 'triamcort' OR 'triamcot' OR 'triamonide 40' OR 'triamsicort' OR 'triancinolon' OR 'u-tri-lone' OR 'volon'",39563,24 Nov 2020

#129,"'beclometasone'/exp OR '9 chloro 11beta, 17, 21 trihydroxy 16beta methylpregna 1, 4 diene 3, 20 dione' OR '9alpha chloro 16beta methylprednisolone' OR 'beclometasone' OR 'beclomethasone' OR 'prednisolone, 9alpha chloro 16beta methyl' OR 'pregna 1, 4 diene 11beta, 17, 21 triol 3, 20 dione, 9 chloro 16beta methyl'",15089,24 Nov 2020

#128,"'dexamethasone'/de OR '16alpha methyl 9alpha fluoroprednisolone' OR '9 alpha fluoro 16 alpha methyl delta corticosterone' OR '9alpha fluoro 11beta, 17alpha, 21 trihydroxy 16alpha methyl 1, 4

pregnadiene 3, 20 dione' OR '9alpha fluoro 11beta, 17alpha, 21 trihydroxy 16alpha methylpregna 1, 4 diene 3, 20 dione' OR '9alpha fluoro 16alpha methyl delta corticosterone' OR 'adrecort' OR 'adrenocot' OR 'aeroseb dex' OR 'aeroseb-dex' OR 'aflucoson' OR 'aflucosone' OR 'alfalyl' OR 'anaflogistico' OR 'anaflogistico novobios' OR 'arcodexan' OR 'arcodexane' OR 'artrosone' OR 'azium' OR 'bidexol' OR 'calonat' OR 'cebedex' OR 'cetadexon' OR 'colofoam' OR 'corsona' OR 'cortastat' OR 'cortastat 10' OR 'cortastat la' OR 'cortidex' OR 'cortidexason' OR 'cortidrona' OR 'cortidrone' OR 'cortisumman' OR 'dacortina fuerte' OR 'dacortine fuerte' OR 'dalalone' OR 'dalalone d.p.' OR 'dalalone l.a.' OR 'danasone' OR 'de-sone la' OR 'decacortin' OR 'decadeltosona' OR 'decadeltosone' OR 'decaderm' OR 'decadion' OR 'decadran' OR 'decadron' OR 'decadron 5-12 pak' OR 'decadron la' OR 'decadronal' OR 'decadrone' OR 'decaesadril' OR 'decaject' OR 'decamethasone' OR 'decasone' OR 'decaspray' OR 'decasterolone' OR 'decdan' OR 'decilone' OR 'decilone forte' OR 'decofluor' OR 'dectancyl' OR 'dekacort' OR 'delladec' OR 'deltafluoren' OR 'deltafluorene' OR 'dergramin' OR 'deronil' OR 'desacort' OR 'desacortone' OR 'desadrene' OR 'desalark' OR 'desameton' OR 'desametone' OR 'desigdron' OR 'dexa cortisyl' OR 'dexa dabrosan' OR 'dexa korti' OR 'dexa scherosan' OR 'dexa scherozon' OR 'dexa scherozone' OR 'dexa-p' OR 'dexacen 4' OR 'dexacen-4' OR 'dexachel' OR 'dexacort' OR 'dexacortal' OR 'dexacorten' OR 'dexacortin' OR 'dexacortisyl' OR 'dexadabroson' OR 'dexadecadrol' OR 'dexadrol' OR 'dexagel' OR 'dexagen' OR 'dexahelvacort' OR 'dexakorti' OR 'dexalien' OR 'dexalocal' OR 'dexame' OR 'dexamecortin' OR 'dexameson' OR 'dexamesone' OR 'dexametason' OR 'dexametasone' OR 'dexameth' OR 'dexamethason' OR 'dexamethasone' OR 'dexamethasone alcohol' OR 'dexamethasone intensol' OR 'dexamethazon' OR 'dexamethazone' OR 'dexamethonium' OR 'dexamonozon' OR 'dexan' OR 'dexane' OR 'dexano' OR 'dexapot' OR 'dexascheroson' OR 'dexascherozon' OR 'dexascherozone' OR 'dexason' OR 'dexasone' OR 'dexasone la' OR 'dexasone s' OR 'dexinoral' OR 'dexionil' OR 'dexmethsone' OR

'dexona' OR 'dexone' OR 'dexone 0.5' OR 'dexone 0.75' OR 'dexone 1.5' OR 'dexone 4' OR 'dexpak taperpak' OR 'dextelan' OR 'dextenza' OR 'dextrasone' OR 'dexycu' OR 'dezone' OR 'dibasona' OR 'doxamethasone' OR 'esacortene' OR 'ex s1' OR 'exadion' OR 'exadione' OR 'firmalone' OR 'fluormethyl prednisolone' OR 'fluormethylprednisolon' OR 'fluormethylprednisolone' OR 'fluormone' OR 'fluorocort' OR 'fluorodelta' OR 'fluoromethylprednisolone' OR 'fortecortin' OR 'gammacorten' OR 'gammacortene' OR 'grosodexon' OR 'grosodexone' OR 'hemady' OR 'hexadecadiol' OR 'hexadecadrol' OR 'hexadiol' OR 'hexadrol' OR 'isnacort' OR 'isopto dex' OR 'isopto maxidex' OR 'isopto-dex' OR 'isopto-maxidex' OR 'isoptodex' OR 'isoptomaxidex' OR 'lokalison f' OR 'loverine' OR 'luxazone' OR 'marvidione' OR 'maxidex' OR 'mediamethasone' OR 'megacortin' OR 'mephameson' OR 'mephamesone' OR 'metasolon' OR 'metasolone' OR 'methazon ion' OR 'methazone ion' OR 'methazonion' OR 'methazonione' OR 'metisone lafi' OR 'mexasone' OR 'millicorten' OR 'millicortenol' OR 'mk 125' OR 'mk125' OR 'mymethasone' OR 'neoforderx' OR 'neofordex' OR 'nisomethasona' OR 'novocort' OR 'nsc 34521' OR 'nsc34521' OR 'oftan-dexa' OR 'opticorten' OR 'opticortinol' OR 'oradexan' OR 'oradexon' OR 'oradexone' OR 'orgadrone' OR 'ozurdex' OR 'pidexon' OR 'policort' OR 'posurdex' OR 'predni f tablinen' OR 'predni-f' OR 'prednisolone f' OR 'prodexona' OR 'prodexone' OR 'sanamethasone' OR 'santenson' OR 'santeson' OR 'sawasone' OR 'solurex' OR 'solurex la' OR 'spoloven' OR 'sterasone' OR 'thilodexine' OR 'triamcimetil' OR 'vexamet' OR 'visumetazone' OR 'visumethazone'",171053,24 Nov 2020

#127,"'hydrocortisone'/de OR '11beta, 17, 21 trihydroxypregn 4 ene 3, 20 dione' OR '11beta, 17alpha, 21 trihydroxypregn 4 ene 3, 20 dione' OR '17 hydroxycorticosterone' OR '4 pregnene 11beta, 17alpha, 21 triol 3, 20 dione' OR '4 pregnene 3, 20 dione n beta, 17alpha, 21 triol' OR 'acticort' OR 'acticort 100' OR 'aeroseb hc' OR 'aeroseb-hc' OR 'ala-cort' OR 'ala-scalp' OR 'ala-scalp hp' OR 'alfacort' OR 'algicortis' OR 'alkindi' OR 'alpha derm' OR 'alphaderm' OR 'anucort-hc' OR 'anumed-hc' OR 'anutone-hc' OR

'aquanil hc' OR 'balneol-hc' OR 'barseb hc' OR 'beta-hc' OR 'biacort' OR 'cetacort' OR 'cobadex' OR 'colocort' OR 'compound f' OR 'cordicare lotion' OR 'coripen' OR 'cort dome' OR 'cort-dome' OR 'cort-dome high potency' OR 'cortef' OR 'cortef cream' OR 'cortenema' OR 'cortibel' OR 'corticorenol' OR 'cortifan' OR 'cortiphate' OR 'cortisol' OR 'cortisole' OR 'cortispray' OR 'cortoderm' OR 'cortril' OR 'cotacort' OR 'covocort' OR 'cremicort-h' OR 'cutaderm' OR 'derm-aid cream' OR 'dermacrin hc lotion' OR 'dermaid' OR 'dermaid soft cream' OR 'dermocare' OR 'dermocortal' OR 'dermolate' OR 'dioderm' OR 'eczacort' OR 'ef cortelan' OR 'efcortelan' OR 'egocort' OR 'egocort cream' OR 'eksalb' OR 'eldecort' OR 'emo-cort' OR 'epicort' OR 'ficortril' OR 'filocot' OR 'flexicort' OR 'gly-cort' OR 'glycort' OR 'h-cort' OR 'hc (hydrocortisone)' OR 'hc no. 1' OR 'hc no. 4' OR 'hebcort' OR 'hebcort v' OR 'hemorrhoidal hc' OR 'hemril-30' OR 'hemril-hc uniserts' OR 'hi-cor' OR 'hidrotisona' OR 'hycor' OR 'hycort' OR 'hydracort' OR 'hydrasson' OR 'hydro ricortex' OR 'hydro-rx' OR 'hydrocort' OR 'hydrocorticosteroid' OR 'hydrocortisate' OR 'hydrocortison' OR 'hydrocortisone' OR 'hydrocortisone 1% in absorbase' OR 'hydrocortisone acetonide' OR 'hydrocortisone astier' OR 'hydrocortisone in absorbase' OR 'hydrocortisone ointment' OR 'hydrocortisone plus saline' OR 'hydrocortisone steroid' OR 'hydrocortisone, topical' OR 'hydrocortisonum' OR 'hydrocortisyl' OR 'hydrocortone' OR 'hydrogalen' OR 'hydrokort' OR 'hydrokortison' OR 'hydrotopic' OR 'hysone' OR 'hytisone' OR 'hytone' OR 'hytone lotion' OR 'incortin h' OR 'instacort 10' OR 'kyypakkaus' OR 'lacticare hc' OR 'lacticare-hc' OR 'lemnis fatty cream hc' OR 'lenirit' OR 'medihaler cort' OR 'medihaler duo' OR 'medrocil' OR 'mildison' OR 'mildison fet krem' OR 'mildison lipocream' OR 'mildison-fatty' OR 'mitocortyl demangeaisons' OR 'munitren' OR 'nogenic hc' OR 'novohydrocort' OR 'nsc 10483' OR 'nsc 741' OR 'nsc10483' OR 'nutracort' OR 'optef' OR 'otosone f' OR 'penecort' OR 'plenadren' OR 'prepcort' OR 'prevex hc' OR 'pro cort' OR 'procort' OR 'procto-kit 1%' OR 'procto-kit 2.5%' OR 'proctocort' OR 'proctosert hc' OR

'proctosol-hc' OR 'proctosone' OR 'proctozone hc' OR 'procutan' OR 'rectasol-hc' OR 'rectocort' OR 'rederm' OR 'sanatison' OR 'scalp-aid' OR 'schericur' OR 'schericur 0.25%' OR 'scherosone f' OR 'sistral hydrocort' OR 'skincalm' OR 'stie-cort' OR 'substance m' OR 'synacort' OR 'texacort' OR 'triburon-hc' OR 'unicort' OR 'vasocort'",165764,24 Nov 2020

#126,"'corticosterone'/de OR '11 beta, 21 dihydroxy pregn 4 ene 3, 20 dione' OR '11beta, 21 dihydroxy 4 pregnene 3, 20 dione' OR '11beta, 21 dihydroxypregn 4 ene 3, 20 dione' OR '4 pregnene 11beta, 21 diol 3, 20 dione' OR 'compound b' OR 'corticosterone*' OR 'corticosterone function' OR 'corticosterone response' OR 'kendall compound b' OR 'nsc 9705' OR 'nsc9705' OR 'pregn 4 ene 11beta, 21 diol 3, 20 dione' OR 'reichstein substance h'",45687,24 Nov 2020

#125,"'glucocorticoid'/exp OR 'glucocorticoid*' OR 'glucocorticoid drug' OR 'glucocorticoid hormone' OR 'glucocorticoid steroid' OR 'glucocorticoids' OR 'glucocorticoids, synthetic' OR 'glucocorticoids, topical' OR 'glucocorticoidsteroid' OR 'glucocorticosteroid' OR 'glucocortoid' OR 'glycocorticoid' OR 'glycocorticosteroid'",796824,24 Nov 2020

#124,"'methylprednisolone'/de OR '11beta, 17alpha, 21 trihydroxy 6alpha methyl 1, 4 pregnadiene 3, 20 dione' OR '2 methylprednisolone' OR '5 methylprednisolone' OR '6 alpha methylprednisolone' OR '6 methyl delta 1 hydrocortisone' OR '6 methyl prednisolone' OR '6 methylprednisolone' OR '6alpha methyl delta1 hydrocortisone' OR '6alpha methylprednisolone' OR 'adlone-40' OR 'adlone-80' OR 'beta methylprednisolone' OR 'dep medalone 80' OR 'depmedalone' OR 'depoject-80' OR 'depopred' OR 'esametone' OR 'firmacort' OR 'med-jec-40' OR 'medixon' OR 'mednin' OR 'medralone 80' OR 'medrate' OR 'medrol' OR 'medrol a' OR 'medrol adt pak' OR 'medrol compositum' OR 'medrol dosepak' OR 'medrol medules' OR 'medrol pak' OR 'medrone' OR 'meprednisolone' OR 'meprelon' OR 'mesopren' OR 'methacort 40' OR 'methacort 80' OR 'methyl prednisolone' OR 'methylcotol' OR 'methylcotolone' OR

'methylpred dp' OR 'methylprednisolone*' OR 'methylsterolone' OR 'metidrol' OR 'metrisone' OR 'metycortin' OR 'metypred' OR 'metypresol' OR 'neomedrone' OR 'nsc 19987' OR 'nsc19987' OR 'prednol' OR 'solomet' OR 'solu decortin' OR 'urbason' OR '83 43 2':rn",109165,24 Nov 2020

#123,"'cortisone'/de OR '11 dehydro 17 hydroxy corticosterone' OR '11 dehydro 17 hydroxycorticosterone' OR '17 hydroxy 11 dehydrocortisone' OR '17alpha, 21 dihydroxy 4 pregnene 3, 11, 20 trione' OR '4 pregnene 17alpha, 21 diol 3, 11, 20 trione' OR 'adrenalex' OR 'compound e (kendall)' OR 'corlin' OR 'cortadren' OR 'cortagen' OR 'cortandren' OR 'cortane' OR 'cortisal' OR 'cortisate' OR 'cortison' OR 'cortisone$' OR 'cortisone sodium' OR 'cortistal' OR 'cortivite' OR 'cortogen' OR 'cortone' OR 'delta4 pregnene 17alpha, 21 diol 3, 11, 20 trione' OR 'incorlin' OR 'incortin' OR 'kendall compound e' OR 'nsc 9703' OR 'nsc9703' OR 'pregn 4 en 17 alpha, 21 diol 3, 11, 20 trione' OR 'pregn 4 ene 17alpha, 21 diol 3, 11, 20 trione' OR 'reichstein substance fa' OR 'ricortex' OR 'scheroson' OR 'scherosone' OR 'wintersteiner compound f'",32703,24 Nov 2020

#122,"'cortisone therapeutic use'/de",3404,24 Nov 2020

#121,"'prednisone'/de OR '1, 2 dehydrocortisone' OR '17, 21 dihydroxypregna 1, 4 diene 3, 11, 20 trione' OR 'ancortone' OR 'apo-prednisone' OR 'biocortone' OR 'colisone' OR 'cortan' OR 'cortidelt' OR 'cortiprex' OR 'cutason' OR 'dacorten' OR 'de cortisyl' OR 'decortancyl' OR 'decortin' OR 'decortin e merck' OR 'decortine' OR 'decortisyl' OR 'dehydrocortisone' OR 'dekortin' OR 'delitisone' OR 'dellacort a' OR 'delta 1 dehydrocortisone' OR 'delta cortelan' OR 'delta cortisone' OR 'delta dome' OR 'delta e' OR 'delta prenovis' OR 'delta-dome' OR 'deltacorten' OR 'deltacortene' OR 'deltacortisone' OR 'deltacortone' OR 'deltasone' OR 'deltison' OR 'deltisona' OR 'deltra' OR 'di adreson' OR 'di-adreson' OR 'diadreson' OR 'drazone' OR 'encorton' OR 'encortone' OR 'enkorton' OR 'fernisone' OR 'hostacortin' OR 'insone' OR 'liquid pred' OR 'lodotra' OR 'me-korti' OR 'meprison' OR 'metacortandracin' OR

'meticorten' OR 'meticortine' OR 'nisona' OR 'nsc 10023' OR 'nsc10023' OR 'orasone' OR 'orisane' OR 'panafcort' OR 'paracort' OR 'pehacort' OR 'precort' OR 'precortal' OR 'prednicen-m' OR 'prednicorm' OR 'prednicot' OR 'prednidib' OR 'prednison' OR 'prednisone*' OR 'prednisone alcohol' OR 'prednisone intensol' OR 'prednisone test' OR 'prednitone' OR 'pregna 1, 4 diene 3, 11, 20 trione 17, 21 diol' OR 'pronison' OR 'pronisone' OR 'pronizone' OR 'pulmison' OR 'rayos' OR 'rectodelt' OR 'servisone' OR 'steerometz' OR 'sterapred' OR 'sterapred ds' OR 'ultracorten' OR 'urtilone' OR 'winpred'",187211,24 Nov 2020

#120,"'prednisolone'/de OR '1, 2 dehydro hydrocortison' OR '1, 4 pregnadien 11beta, 17alpha, 21 triol 3, 20 dion' OR '1, 4 pregnadiene 11beta, 17alpha, 21 triol 3, 20 dione' OR '1, 4 pregnadiene 3, 20 dione 11beta, 17alpha, 21 triol' OR '11beta, 17, 21 trihydroxypregna 1, 4 diene 3, 20 dione' OR '11beta, 17alpha, 21 trihydroxy 1, 4 pregnadien 3, 20 dion' OR '11beta, 17alpha, 21 trihydroxypregna 1, 4 diene 3, 20 dione' OR '3, 20 dioxo 11beta, 17alpha, 21 trihydroxy 1, 4 pregnadiene' OR 'adelcort' OR 'antisolon' OR 'antisolone' OR 'aprednislon' OR 'aprednislone' OR 'benisolon' OR 'benisolone' OR 'berisolon' OR 'berisolone' OR 'caberdelta' OR 'capsoid' OR 'co hydeltra' OR 'codelcortone' OR 'compresolon' OR 'cortadeltona' OR 'cortadeltone' OR 'cortalone' OR 'cortelinter' OR 'cortisolone' OR 'cotolone' OR 'dacortin' OR 'dacortin h' OR 'dacrotin' OR 'decaprednil' OR 'decortin h' OR 'decortril' OR 'dehydro cortex' OR 'dehydro hydrocortison' OR 'dehydro hydrocortisone' OR 'dehydrocortex' OR 'dehydrocortisol' OR 'dehydrocortisole' OR 'dehydrohydrocortison' OR 'dehydrohydrocortisone' OR 'delcortol' OR 'delta 1 17 hydroxycorticosterone 21 acetate' OR 'delta 1 hydrocortisone' OR 'delta cortef' OR 'delta cortril' OR 'delta ef cortelan' OR 'delta f' OR 'delta hycortol' OR 'delta hydrocortison' OR 'delta hydrocortisone' OR 'delta ophticor' OR 'delta stab' OR 'delta-cortef' OR 'delta1 dehydrocortisol' OR 'delta1 dehydrohydrocortisone' OR 'delta1 hydrocortisone' OR 'deltacortef' OR

'deltacortenolo' OR 'deltacortil' OR 'deltacortoil' OR 'deltacortril' OR 'deltaderm' OR 'deltaglycortril' OR 'deltahycortol' OR 'deltahydrocortison' OR 'deltahydrocortisone' OR 'deltaophticor' OR 'deltasolone' OR 'deltastab' OR 'deltidrosol' OR 'deltisilone' OR 'deltisolon' OR 'deltisolone' OR 'deltolasson' OR 'deltolassone' OR 'deltosona' OR 'deltosone' OR 'depo-predate' OR 'dermosolon' OR 'dhasolone' OR 'di adreson f' OR 'di adresone f' OR 'di-adreson-f' OR 'diadreson f' OR 'diadresone f' OR 'dicortol' OR 'domucortone' OR 'encortelon' OR 'encortelone' OR 'encortolon' OR 'equisolon' OR 'fernisolone-p' OR 'glistelone' OR 'hefasolon' OR 'hostacortin h' OR 'hostacortin h vet' OR 'hydeltra' OR 'hydeltrone' OR 'hydrelta' OR 'hydrocortancyl' OR 'hydrocortidelt' OR 'hydrodeltalone' OR 'hydrodeltisone' OR 'hydroretrocortin' OR 'hydroretrocortine' OR 'inflanefran' OR 'insolone' OR 'keteocort h' OR 'key-pred' OR 'key-pred sp' OR 'lenisolone' OR 'leocortol' OR 'liquipred' OR 'lygal kopftinktur n' OR 'mediasolone' OR 'meprisolon' OR 'meprisolone' OR 'metacortalon' OR 'metacortalone' OR 'metacortandralon' OR 'metacortandralone' OR 'metacortelone' OR 'meti derm' OR 'meti-derm' OR 'meticortelone' OR 'metiderm' OR 'morlone' OR 'mydrapred' OR 'neo delta' OR 'nisolon' OR 'nisolone' OR 'nsc 9120' OR 'nsc9120' OR 'opredsone' OR 'panafcortelone' OR 'panafcortolone' OR 'panafort' OR 'paracortol' OR 'phlogex' OR 'pre cortisyl' OR 'preconin' OR 'precortalon' OR 'precortancyl' OR 'precortisyl' OR 'pred-ject-50' OR 'predacort 50' OR 'predaject-50' OR 'predalone 50' OR 'predartrina' OR 'predartrine' OR 'predate-50' OR 'predeltilone' OR 'predisole' OR 'predisyr' OR 'predne dome' OR 'prednecort' OR 'prednedome' OR 'prednelan' OR 'predni coelin' OR 'predni h tablinen' OR 'predni-helvacort' OR 'prednicoelin' OR 'prednicort' OR 'prednicortelone' OR 'prednifor drops' OR 'predniment' OR 'predniretard' OR 'prednis' OR 'prednisil' OR 'prednisolon' OR 'prednisolona' OR 'prednisolone*' OR 'prednisolone alcohol' OR 'prednisolone h' OR 'prednisolone oleosae sr 82' OR 'prednisolone, topical' OR 'prednivet' OR 'prednorsolon' OR 'prednorsolone' OR 'predonine' OR 'predorgasolona' OR

'predorgasolone' OR 'pregna 1, 4 diene 11beta, 17alpha, 21 triol 3, 20 dione' OR 'prelon' OR 'prelone' OR 'prenilone' OR 'prenin' OR 'prenolone' OR 'preventan' OR 'prezolon' OR 'rubycort' OR 'scherisolon' OR 'scherisolona' OR 'serilone' OR 'solondo' OR 'solone' OR 'solupren' OR 'soluprene' OR 'spiricort' OR 'spolotane' OR 'sterane' OR 'sterolone' OR 'supercortisol' OR 'supercortizol' OR 'taracortelone' OR 'walesolone' OR 'wysolone'",147347,24 Nov 2020

#119,"'corticosteroid'/exp OR 'adrenal cortex hormone' OR 'adrenal cortex hormones' OR 'adrenal cortical hormone' OR 'adrenal cortical hormones' OR 'adrenal cortical steroid' OR 'adrenal steroid' OR 'adrenal steroid hormone' OR 'adreno cortical steroid' OR 'adreno corticosteroid' OR 'adrenocortical hormone' OR 'adrenocortical steroid' OR 'adrenocorticosteroid' OR 'cortical steroid' OR 'cortico steroid' OR 'corticoid*' OR 'corticosteroid*' OR 'corticosteroid agent' OR 'corticosteroid calcium' OR 'corticosteroid hormone' OR 'corticosteroids' OR 'corticosteroids, inhalation' OR 'corticosteroids, ophthalmic' OR 'corticosteroids, otic' OR 'corticosteroids, systemic' OR 'corticosteroids, topical' OR 'dermocorticosteroid' OR 'fluorinated corticosteroid'",1039553,24 Nov 2020

#118,"chloroquin*:ti,ab,kw,rn,tn OR cq:ti,ab,kw,rn,tn OR '54-04-7':ti,ab,kw,rn,tn OR 886u3h6uff*:ti,ab,kw,rn,tn OR arequin:ti,ab,kw,rn,tn OR chingamin:ti,ab,kw,rn,tn OR chlorochin*:ti,ab,kw,rn,tn OR khingamin:ti,ab,kw,rn,tn OR oe48649kh6n*:ti,ab,kw,rn,tn OR anaclor:ti,ab,kw,rn,tn OR bemaco:ti,ab,kw,rn,tn OR benaquin:ti,ab,kw,rn,tn OR 'brn 0482809':ti,ab,kw,rn,tn OR brn0482809:ti,ab,kw,rn,tn OR capquin:ti,ab,kw,rn,tn OR 'ccris 3439':ti,ab,kw,rn,tn OR ccris3439:ti,ab,kw,rn,tn OR chloraquin*:ti,ab,kw,rn,tn OR cindacin:ti,ab,kw,rn,tn OR cindachin:ti,ab,kw,rn,tn OR cloroquina:ti,ab,kw,rn,tn OR cocatrit:ti,ab,kw,rn,tn OR 'einecs 200-191-2':ti,ab,kw,rn,tn OR 'einecs200 191 2':ti,ab,kw,rn,tn OR elestol:ti,ab,kw,rn,tn OR 'hsdb 3029':ti,ab,kw,rn,tn OR hsdb3029:ti,ab,kw,rn,tn OR

lapaquin:ti,ab,kw,rn,tn OR malaren:ti,ab,kw,rn,tn OR neochin:ti,ab,kw,rn,tn OR 'nsc 187208':ti,ab,kw,rn,tn OR nsc187208:ti,ab,kw,rn,tn OR pfizerquine:ti,ab,kw,rn,tn OR quensyl:ti,ab,kw,rn,tn OR quinercyl:ti,ab,kw,rn,tn OR quinilon:ti,ab,kw,rn,tn OR quinoline:ti,ab,kw,rn,tn OR quinoscan:ti,ab,kw,rn,tn OR ronaquine:ti,ab,kw,rn,tn OR sopaquin:ti,ab,kw,rn,tn OR 'st 21':ti,ab,kw,rn,tn OR weimerquin:ti,ab,kw,rn,tn",36685,24 Nov 2020

#117,"'chloroquine'/de OR '4 (4 diethylamino 1 methylbutylamino) 7 chlorchinolin diphosphate' OR '4 (4 diethylamino 1 methylbutylamino) 7 chlorchinolin sulfate' OR '4 (4 diethylamino 1 methylbutylamino) 7 chlorchinolin sulphate' OR '4 (4 diethylamino 1 methylbutylamino) 7 chloroquinoline' OR '7 chloro 4 (4 diethylamino 1 methylbutylamino) quinoline' OR '7 chloro 4 (4 diethylamino 1 methylbutylamino) quinoline diphosphate' OR 'a-cq' OR 'amokin' OR 'amokine' OR 'anoclor' OR 'aralan' OR 'aralen' OR 'aralen hydrochloride' OR 'aralen phosphate' OR 'aralene' OR 'arechin' OR 'arechine' OR 'arequine' OR 'arthrochin' OR 'arthrochine' OR 'arthroquine' OR 'artrichin' OR 'artrichine' OR 'artriquine' OR 'avloclor' OR 'avoclor' OR 'bemaphata' OR 'bemaphate' OR 'bemasulph' OR 'bipiquin' OR 'cadiquin' OR 'chemochin' OR 'chemochine' OR 'chingamine' OR 'chingaminum' OR 'chloraquine' OR 'chlorochin' OR 'chlorochine' OR 'chlorofoz' OR 'chloroquin' OR 'chloroquin phosphate' OR 'chloroquine' OR 'chloroquine diphosphate' OR 'chloroquine disulfate' OR 'chloroquine disulphate' OR 'chloroquine hydrochloride' OR 'chloroquine phosphate' OR 'chloroquine streuli' OR 'chloroquine sulfate' OR 'chloroquine sulphate' OR 'chloroquinesulphate' OR 'chloroquini diphosphas' OR 'chloroquinum diphosphoricum' OR 'chlorquin' OR 'chlorquine' OR 'choloquine' OR 'choroquine sulfate' OR 'choroquine sulphate' OR 'cidanchin' OR 'clo-kit junior' OR 'clorichina' OR 'clorichine' OR 'cloriquine' OR 'clorochina' OR 'delagil' OR 'delagyl' OR 'dichinalex' OR 'diclokin' OR 'diquinalex' OR 'diroquine' OR 'emquin' OR 'genocin' OR 'gontochin' OR 'gontochine' OR 'gontoquine' OR 'heliopar' OR 'imagon' OR

'iroquine' OR 'klorokin' OR 'klorokine' OR 'klorokinfosfat' OR 'lagaquin' OR 'malaquin' OR 'malarex' OR 'malarivon' OR 'malaviron' OR 'maliaquine' OR 'maquine' OR 'mesylith' OR 'mexaquin' OR 'mirquin' OR 'nivachine' OR 'nivaquin' OR 'nivaquine' OR 'nivaquine (b)' OR 'nivaquine b' OR 'nivaquine dp' OR 'nivaquine forte' OR 'p roquine' OR 'quinachlor' OR 'quingamine' OR 'repal' OR 'resochen' OR 'resochene' OR 'resochin' OR 'resochin junior' OR 'resochina' OR 'resochine' OR 'resochinon' OR 'resoquina' OR 'resoquine' OR 'reumachlor' OR 'roquine' OR 'rp 3377' OR 'rp3377' OR 'sanoquin' OR 'sanoquine' OR 'silbesan' OR 'siragan' OR 'sirajan' OR 'sn 7618' OR 'sn7618' OR 'solprina' OR 'solprine' OR 'tresochin' OR 'tresochine' OR 'tresoquine' OR 'trochin' OR 'trochine' OR 'troquine' OR 'w 7618' OR 'w7618' OR 'win 244' OR 'win244'",44514,24 Nov 2020

#116,"'organogold compound'/exp OR 'organogold compound' OR 'organogold compounds' OR 'organogold derivative'",482,24 Nov 2020

#115,"aurothiopropanol*:ti,ab,kw,rn,tn OR allochrysine:ti,ab,kw,rn,tn OR allocrisine:ti,ab,kw,rn,tn OR aurotiopoe:ti,ab,kw,rn,tn OR aurotioprol:ti,ab,kw,rn,tn OR '27279-43-2':ti,ab,kw,rn,tn OR g7097j63e9*:ti,ab,kw,rn,tn OR 'sodium aurothiopropanolsulfonate':ti,ab,kw,rn,tn OR 'gold sodium thiopropanol sulfonate':ti,ab,kw,rn,tn OR 'sodium auromercaptopropanol sulfonate':ti,ab,kw,rn,tn",274,24 Nov 2020

#114,"'allochrysine'/de OR '1 auromercapto 2 propanol 3 sulfonate' OR '1 auromercapto 2 propanol 3 sulfonate sodium' OR '1 auromercepto 2 propanol 3 sulfonate' OR 'allochrysine' OR 'auromercapto propanol sulfonate sodium' OR 'auromercaptopropanolsulfonate sodium' OR 'aurothioisopropanolsulfonate sodium' OR 'aurothiopropanol sulfonate' OR 'aurothiopropanol sulfonate sodium' OR 'gold sodium thiopropanol sulfonate' OR 'sodium 1 auromercapto 2 propanol 3

sulfonate' OR 'sodium auromercapto 2 propanol 3 sulfonate' OR 'sodium auromercaptopropanol sulfonate' OR 'sodium aurothiopropanol sulfonate'",272,24 Nov 2020

#113,"((gold* OR auro*) NEAR/10 (inject* OR intramuscular* OR 'im' OR 'intra muscular*')):ti,ab,kw",3123,24 Nov 2020

#112,"(thioglucosoaurate*:ti,ab,kw,rn,tn OR 'gold thioglucose*':ti,ab,kw,rn,tn OR 'gold thio glucose*':ti,ab,kw,rn,tn OR 'thioglucose gold':ti,ab,kw,rn,tn OR aureotan:ti,ab,kw,rn,tn OR 'gold-50':ti,ab,kw,rn,tn OR gold50:ti,ab,kw,rn,tn OR 'b oleosum solganal':ti,ab,kw,rn,tn OR aurotan:ti,ab,kw,rn,tn OR authron:ti,ab,kw,rn,tn OR brenol:ti,ab,kw,rn,tn OR 'ccris 59':ti,ab,kw,rn,tn OR ccris59:ti,ab,kw,rn,tn OR 'einecs 235-365-7':ti,ab,kw,rn,tn OR 'einecs235 365 7':ti,ab,kw,rn,tn OR glysanol:ti,ab,kw,rn,tn) AND b:ti,ab,kw,rn,tn OR goldthioglucose*:ti,ab,kw,rn,tn OR goldtioglucose*:ti,ab,kw,rn,tn OR 'hsdb 7174':ti,ab,kw,rn,tn OR hsdb7174:ti,ab,kw,rn,tn OR oronol:ti,ab,kw,rn,tn OR romosol:ti,ab,kw,rn,tn OR 'skf 10056':ti,ab,kw,rn,tn OR skf10056:ti,ab,kw,rn,tn OR 2p2v9q0e78*:ti,ab,kw,rn,tn OR '12192-57-3':ti,ab,kw,rn,tn",1509,24 Nov 2020

#111,"'aurothioglucose sodium'/de",40,24 Nov 2020

#110,"'aurothioglucose'/de OR '(1 d glucosylthio) gold' OR '(1 glucosylthio) gold' OR 'anitur' OR 'aureotan' OR 'auro thioglucose*' OR 'auromyose*' OR 'aurothioglucose*' OR 'aurumin' OR 'aurumine' OR 'gold thio glucose' OR 'gold thioglucose' OR 'gold-50' OR 'goldthioglucose' OR 'goldtioglucose' OR 'oronol' OR 'solganal' OR 'solganal b' OR 'solganol'",1709,24 Nov 2020

#109,"aurolate*:ti,ab,kw,rn,tn OR 'gold disodium thiomalate*':ti,ab,kw,rn,tn OR 'gold thiomalic acid*':ti,ab,kw,rn,tn OR miocrisin:ti,ab,kw,rn,tn OR 'monogold disodium thiomalate*':ti,ab,kw,rn,tn OR myochrysin*:ti,ab,kw,rn,tn OR myocrisin*:ti,ab,kw,rn,tn OR myocrysin*:ti,ab,kw,rn,tn OR 'sodium aurothiomalate*':ti,ab,kw,rn,tn OR 'sodium gold thiomalate*':ti,ab,kw,rn,tn OR 'sodium thiomalate*

gold':ti,ab,kw,rn,tn OR 'sodium thiomalatoaurate*':ti,ab,kw,rn,tn OR taureodon:ti,ab,kw,rn,tn OR 'thiomalatoaurate sodium':ti,ab,kw,rn,tn OR '1244-57-4':ti,ab,kw,rn,tn OR '39377-38-3':ti,ab,kw,rn,tn OR '4846-27-9':ti,ab,kw,rn,tn OR '74916-57-7':ti,ab,kw,rn,tn OR e4768zy6gm*:ti,ab,kw,rn,tn OR 'aurothiomala-natrium':ti,ab,kw,rn,tn OR 'aurot$iomalato sodico':ti,ab,kw,rn,tn OR 'monogold* sodium salt':ti,ab,kw,rn,tn OR 'dinatrium 2-aurothio-succinat':ti,ab,kw,rn,tn OR 'einecs 235-479-7':ti,ab,kw,rn,tn OR 'einecs235 479 7':ti,ab,kw,rn,tn OR 'hsdb 7173':ti,ab,kw,rn,tn OR hsdb7173:ti,ab,kw,rn,tn OR kidon:ti,ab,kw,rn,tn OR 'natrii aurothiomalas*':ti,ab,kw,rn,tn OR shiosol:ti,ab,kw,rn,tn OR 'gold mercatpsuccinate*':ti,ab,kw,rn,tn",885,24 Nov 2020

#108,"'aurothiomalate'/de OR '(1, 2 dicarboxyethylthio) gold disodium' OR '(1, 2 dicarboxyethylthio) gold disodium salt' OR 'aurothio' OR 'aurothiomalate*' OR 'aurothiomalate sodium' OR 'disodium aurothiomalate' OR 'gold mercaptosuccinate' OR 'gold sodium thiomalate*' OR 'gold thiomalate*' OR 'gold thiomalate sodium' OR 'miocrin' OR 'myochrisin' OR 'myochrisine' OR 'myochrysin' OR 'myochrysine' OR 'myocrisin' OR 'myocrysin' OR 'shiosol' OR 'sodium aurothiomalate' OR 'sodium gold thiomalate' OR 'tauredon' OR 'tauredone' OR 'thiomalate gold'",3454,24 Nov 2020

#107,"hydroxychloroquin*:ti,ab,kw,rn,tn OR hcq:ti,ab,kw,rn,tn OR '747-3-4':ti,ab,kw,rn,tn OR 8q2869cnvh*:ti,ab,kw,rn,tn OR '118-42-3':ti,ab,kw,rn,tn OR 4qwg6n8qkh*:ti,ab,kw,rn,tn OR hydroxychlorochin*:ti,ab,kw,rn,tn OR hydrochloroquin*:ti,ab,kw,rn,tn OR hydrocloroquin*:ti,ab,kw,rn,tn OR oxychlorochin:ti,ab,kw,rn,tn OR oxychloroquin*:ti,ab,kw,rn,tn OR plaquinol:ti,ab,kw,rn,tn OR 'brn 0253894':ti,ab,kw,rn,tn OR brn0253894:ti,ab,kw,rn,tn OR 'einecs 204-249-8':ti,ab,kw,rn,tn OR 'einecs204 249 8':ti,ab,kw,rn,tn OR hidroxicloroquin*:ti,ab,kw,rn,tn OR idrossiclorochin*:ti,ab,kw,rn,tn OR oxichlorochin*:ti,ab,kw,rn,tn OR oxichloroquin*:ti,ab,kw,rn,tn OR win1258:ti,ab,kw,rn,tn OR 'win 1258':ti,ab,kw,rn,tn OR z0188:ti,ab,kw,rn,tn OR 'einecs 212-019-

3':ti,ab,kw,rn,tn OR 'einecs212-019-3':ti,ab,kw,rn,tn OR erquin:ti,ab,kw,rn,tn OR quensyl:ti,ab,kw,rn,tn OR 'sn 8137':ti,ab,kw,rn,tn OR sn8137:ti,ab,kw,rn,tn",29651,24 Nov 2020

#106,"'hydroxychloroquine sulfate'/de OR '1 (7 chloro 4 quinolylamino) 3 diethylamino 2 propanol sulfate' OR '1 (7 chloro 4 quinolylamino) 3 diethylamino 2 propanol sulphate' OR '2 [[4 [(7 chloro 4 quinolinyl) amino] pentyl] (ethyl) amino] ethanol sulfate' OR '2 [[4 [(7 chloro 4 quinolinyl) amino] pentyl] (ethyl) amino] ethanol sulphate' OR '2 [[4 [(7 chloroquinolin 4 yl) amino] pentyl] (ethyl) amino] ethanol sulfate' OR '2 [[4 [(7 chloroquinolin 4 yl) amino] pentyl] (ethyl) amino] ethanol sulphate' OR '7 chloro 4 (3 diethylamino 2 hydroxypropylamino) quinoline sulfate' OR '7 chloro 4 (3 diethylamino 2 hydroxypropylamino) quinoline sulphate' OR 'dimard' OR 'erquin' OR 'evoquin' OR 'geniquin' OR 'hydroxychloroquine sulfate' OR 'hydroxychloroquine sulphate' OR 'oxiklorin' OR 'oxychloroquine sulfate' OR 'oxychloroquine sulphate' OR 'plaquenil' OR 'plaquenil sulfate' OR 'plaquenil sulphate' OR 'plaquinol' OR 'toremonil' OR 'yuma'",3647,24 Nov 2020

#105,"'hydroxychloroquine'/de OR '7 chloro 4 [4 [ethyl (2 hydroxyethyl) amino] 1 methylbutylamino] quinoline' OR '7 chloro 4 [4 [ethyl (2 hydroxyethyl) amino] 1 methylbutylamino] quinoline diphosphate' OR 'apo-hydroxychloroquine' OR 'chloroquinol' OR 'ercoquin' OR 'hydrochloroquine' OR 'hydrocloroquine' OR 'hydroxychloroquine' OR 'oxychloroquine' OR 'quensyl' OR 'sn 8137'",29818,24 Nov 2020

#104,"sasp:ti,ab,kw,rn,tn OR ssz:ti,ab,kw,rn,tn OR salicylazosulfapyridin*:ti,ab,kw,rn,tn OR 'salicyl azo sulfapyridin*':ti,ab,kw,rn,tn OR salazosulfapyridin*:ti,ab,kw,rn,tn OR salazosulfapiridin*:ti,ab,kw,rn,tn OR azulfadin*:ti,ab,kw,rn,tn OR azulfidin*:ti,ab,kw,rn,tn OR pleon:ti,ab,kw,rn,tn OR ulcol:ti,ab,kw,rn,tn OR ucine:ti,ab,kw,rn,tn OR salazopyrin*:ti,ab,kw,rn,tn OR 'ratio-sulfosalazin*':ti,ab,kw,rn,tn OR 3xc8guz6cb*:ti,ab,kw,rn,tn OR accucol:ti,ab,kw,rn,tn OR asulfidin*:ti,ab,kw,rn,tn OR

azopyrin*:ti,ab,kw,rn,tn OR azosulfidin*:ti,ab,kw,rn,tn OR 'brn 0356241':ti,ab,kw,rn,tn OR brn0356241:ti,ab,kw,rn,tn OR 'ccris 4713':ti,ab,kw,rn,tn OR ccris4713:ti,ab,kw,rn,tn OR 'einecs 209-974-3':ti,ab,kw,rn,tn OR 'hsdb 3395':ti,ab,kw,rn,tn OR hsdb3395:ti,ab,kw,rn,tn OR 'nsc 203730':ti,ab,kw,rn,tn OR nsc203730:ti,ab,kw,rn,tn OR 'nsc 667219':ti,ab,kw,rn,tn OR nsc667219:ti,ab,kw,rn,tn OR reupirin:ti,ab,kw,rn,tn OR 'salazo-sulfapyridin*':ti,ab,kw,rn,tn OR salazosulfpyridin*:ti,ab,kw,rn,tn OR salazopiridazin*:ti,ab,kw,rn,tn OR salazopyridin*:ti,ab,kw,rn,tn OR salazopirin*:ti,ab,kw,rn,tn OR salipyr:ti,ab,kw,rn,tn OR 'si-88':ti,ab,kw,rn,tn OR si88:ti,ab,kw,rn,tn OR sulfasalazin*:ti,ab,kw,rn,tn OR sulphasalazin*:ti,ab,kw,rn,tn OR 'w-t sasp oral':ti,ab,kw,rn,tn OR '599-79-1':ti,ab,kw,rn,tn",28714,24 Nov 2020

#103,"'salazosulfapyridine'/de OR '4 (2 pyridylaminosulfonyl) 3` carboxy 4` hydroxyazobenzene' OR '4 hydroxy 4` (pyrid 2 ylsulfamoyl) azobenzene 3 carboxylic acid' OR '5 [4 (2 pyridylsulfamoyl) phenylazo] 2 hydroxybenzoic acid' OR '5 [para (2 pyridylsulfamoyl) phenylazo] salicylic acid' OR 'azlufidine en-tabs' OR 'azopyrin' OR 'azopyrine' OR 'azosulfidine' OR 'azulfide' OR 'azulfidina' OR 'azulfidine' OR 'azulfidine en tabs' OR 'azulfidine en-tabs' OR 'azulfidine ra' OR 'azulfin' OR 'benzosulfa' OR 'colo pleon' OR 'colo-pleon' OR 'colopleon' OR 'disalazin' OR 'gastropyrin' OR 'pleon ra' OR 'pyralin en' OR 'rorasul' OR 'rosulfant' OR 's.a.s.-500' OR 'salazine' OR 'salazo sulfapyridine' OR 'salazodin' OR 'salazopirina' OR 'salazopyridin' OR 'salazopyridine' OR 'salazopyrin' OR 'salazopyrin entabs' OR 'salazopyrin-en' OR 'salazopyrina' OR 'salazopyrine' OR 'salazopyrine ec' OR 'salazosulfa pyridine' OR 'salazosulfapyridine' OR 'salazosulfpyridine' OR 'salicyl azo sulfapyridine' OR 'salicylazosulfapyridin' OR 'salicylazosulfapyridine' OR 'salisulf' OR 'salopyr' OR 'saridine' OR 'sas 500' OR 'sulcolon' OR 'sulfasalazine' OR 'sulfasalizine' OR 'sulfosalazine' OR 'sulphasalazine' OR 'zopyrin'",26747,24 Nov 2020

#102,"'lef':ti,ab,kw",4107,24 Nov 2020

#101,"'leflunomide'/de OR '5 methyl 4` trifluoromethyl 4 isoxazolecarboxanilide' OR '5 methyl n [4 (trifluoromethyl) phenyl] 4 isoxazolecarboxamide' OR '5 methyl n [4 (trifluoromethyl) phenyl] isoxazole 4 carboxamide' OR '5 methyl n [para (trifluoromethyl) phenyl] 4 isoxazolecarboxamide' OR 'alpha, alpha, alpha trifluoro 5 methyl 4 isoxazolecarboxy para toluidide' OR 'arabloc' OR 'arava' OR 'hwa 486' OR 'hwa486' OR 'leflunomid*' OR 'leflunomide winthrop' OR 'n (4 trifluoromethylphenyl) 5 methylisoxazole 4 carboxamide' OR 'repso' OR 'rs 34821' OR 'rs34821' OR 'su 101' OR 'su101' OR 'hsdb 7289' OR hsdb7289 OR g162gk9u4w* OR '75706-12-6'",12977,24 Nov 2020

#100,"'mtx':ti,ab,kw,rn,tn OR methotrex*:ti,ab,kw,rn,tn OR metotrexat*:ti,ab,kw,rn,tn OR methylaminopterin*:ti,ab,kw,rn,tn OR amet$opterin*:ti,ab,kw,rn,tn OR brimexate:ti,ab,kw,rn,tn OR 'a-met$opterin*':ti,ab,kw,rn,tn OR 'a-methpterin*':ti,ab,kw,rn,tn OR 'ai3-25299':ti,ab,kw,rn,tn OR ai325299:ti,ab,kw,rn,tn OR 'alpha-methopterin':ti,ab,kw,rn,tn OR farmitrexat*:ti,ab,kw,rn,tn OR fauldexato:ti,ab,kw,rn,tn OR hdmtx:ti,ab,kw,rn,tn OR metotressat*:ti,ab,kw,rn,tn OR methotrate*:ti,ab,kw,rn,tn OR methohexate*:ti,ab,kw,rn,tn OR mexat*:ti,ab,kw,rn,tn OR metrotex:ti,ab,kw,rn,tn OR metatrexato*:ti,ab,kw,rn,tn OR metatrexan:ti,ab,kw,rn,tn OR maxtrex:ti,ab,kw,rn,tn OR meticil:ti,ab,kw,rn,tn OR metoject:ti,ab,kw,rn,tn OR metotrexin:ti,ab,kw,rn,tn OR lumexon:ti,ab,kw,rn,tn OR r$eumatrex*:ti,ab,kw,rn,tn OR tremetex:ti,ab,kw,rn,tn OR trexeron:ti,ab,kw,rn,tn OR trixilem:ti,ab,kw,rn,tn OR 'mpi-2505':ti,ab,kw,rn,tn OR mpi2505:ti,ab,kw,rn,tn OR amethopterin*:ti,ab,kw,rn,tn OR '133073-73-1':ti,ab,kw,rn,tn OR '15475-56-6':ti,ab,kw,rn,tn OR 3ig1e710zn*:ti,ab,kw,rn,tn OR yl5fz2y5u1*:ti,ab,kw,rn,tn OR 'ccris 1109':ti,ab,kw,rn,tn OR ccris1109:ti,ab,kw,rn,tn OR 'emt 25299':ti,ab,kw,rn,tn OR emt25299:ti,ab,kw,rn,tn OR 'r-9985':ti,ab,kw,rn,tn OR r9885:ti,ab,kw,rn,tn OR 'x-133':ti,ab,kw,rn,tn OR 'wr-19039':ti,ab,kw,rn,tn OR wr19039:ti,ab,kw,rn,tn",191063,24 Nov 2020

#99,"'methotrexate'/de OR '2 [[4 [[(2, 4 diamino 6 pteridinyl) methyl] (methyl) amino] benzoyl] amino] pentanedioic acid' OR '2 [[4 [[(2, 4 diaminopteridin 6 yl) methyl] (methyl) amino] benzoyl] amino] pentanedioic acid' OR '4 amino 10 methylfolic acid' OR '4 amino 10 methylpteroylglutamic acid' OR '4 amino n10 methylpteroylglutamic acid' OR 'mtx' OR 'a methopterine' OR 'abitrexate' OR 'amethopterin' OR 'amethopterine' OR 'ametopterine' OR 'antifolan' OR 'biotrexate' OR 'canceren' OR 'cl 14377' OR 'cl14377' OR 'emtexate' OR 'emthexat' OR 'emthexate' OR 'emtrexate' OR 'enthexate' OR 'farmitrexat' OR 'farmitrexate' OR 'farmotrex' OR 'folex' OR 'folex pfs' OR 'ifamet' OR 'imeth' OR 'intradose mtx' OR 'jylamvo' OR 'lantarel' OR 'ledertrexate' OR 'maxtrex' OR 'metex' OR 'methoblastin' OR 'methohexate' OR 'methotrate' OR 'methotrexat' OR 'methotrexat ebewe' OR 'methotrexate' OR 'methotrexate lpf' OR 'methotrexate preservative free' OR 'methotrexate sodium' OR 'methotrexate sodium preservative free' OR 'methotrexato' OR 'methoxtrexate' OR 'methrotrexate' OR 'methylaminopterin' OR 'methylaminopterine' OR 'meticil' OR 'metoject' OR 'metothrexate' OR 'metothrexate sodium' OR 'metotrexat' OR 'metotrexate' OR 'metotrexin' OR 'metrex' OR 'mexate' OR 'mexate-aq' OR 'mexate-aq preserved' OR 'mpi 5004' OR 'mpi5004' OR 'n [4 [(2, 4 diamino 6 pteridylmethyl) methylamino] benzoyl] glutamic acid' OR 'neotrexate' OR 'nordimet' OR 'novatrex' OR 'nsc 740' OR 'nsc740' OR 'otrexup' OR 'otrexup pfs' OR 'rasuvo' OR 'reditrex' OR 'reumatrex' OR 'rheumatrex' OR 'rheumatrex dose pack' OR 'sodium methotrexate' OR 'texate' OR 'texate-t' OR 'texorate' OR 'trexall' OR 'xaken' OR 'xatmep' OR 'zexate'",191331,24 Nov 2020

#98,"(('disease modifying' NEAR/3 (antir$eum* OR 'anti r$eum*') NEAR/3 (drug$ OR agent$)):ti,ab,kw) OR (('disease modifying' NEAR/3 (antir$eum* OR 'anti r$eum*') NEAR/3 (drug$ OR agent$)):tt)",10975,24 Nov 2020

#97,"dmard*:ti,ab,kw OR csdmard*:ti,ab,kw OR 'cs-dmard*':ti,ab,kw OR sdmard*:ti,ab,kw OR 's-dmard*':ti,ab,kw OR tsdmard*:ti,ab,kw OR 'ts-dmard*':ti,ab,kw OR bdmard*:ti,ab,kw OR 'b-dmard*':ti,ab,kw OR dmard*:tt OR csdmard*:tt OR 'cs-dmard*':tt OR sdmard*:tt OR 's-dmard*':tt OR tsdmard*:tt OR 'ts-dmard*':tt OR bdmard*:tt OR 'b-dmard*':tt",17423,24 Nov 2020

#96,"'disease modifying antirheumatic drug'/de OR 'disease modifying antirheumatic agent' OR 'disease modifying antirheumatic drug' OR 'disease modifying antirheumatic drugs'",20086,24 Nov 2020

#95,"'mavrilimumab'/de OR 'mavrilimumab*' OR '1085337 57 0' OR 1158jdp9a* OR 'cam 3001' OR cam3001",130,24 Nov 2020

#94,"'ustekinumab'/de OR 'cnto 1275' OR 'cnto1275' OR 'monoclonal antibody cnto 1275' OR 'stelara' OR 'ustekinumab*' OR '815610 63 0' OR fu77b4u5z0* OR 'stelera'",7691,24 Nov 2020

#93,"'guselkumab'/de OR 'cnto 1959' OR 'cnto1959' OR 'guselkumab' OR 'tremfya' OR '1350289 85 8' OR 089658a12d*",826,24 Nov 2020

#92,"'brodalumab'/de OR 'amg 827' OR 'amg827' OR 'brodalumab*' OR 'kyntheum' OR 'siliq' OR 'khk 4827' OR khk4827 OR '1174395 19 7' OR 6za31y954z*",1170,24 Nov 2020

#91,"'ixekizumab'/de OR 'ixekizumab' OR 'ly 2439821' OR 'ly2439821' OR 'taltz' OR '1329632 62 3' OR '1143503 69 8' OR bty153760o*",1953,24 Nov 2020

#90,"'secukinumab'/de OR 'ain 457' OR 'ain457' OR 'cosentyx' OR 'secukinumab*' OR '1229022 83 6' OR dlg4eml025*",4061,24 Nov 2020

#89,"'pateclizumab'/de OR 'mlta 3698a' OR 'mlta3698a' OR 'pateclizumab' OR 'pro 283698' OR 'pro283698' OR '12202526 59 7' OR qok1yyh7j2* OR 'rg 7415' OR rg7415",28,24 Nov 2020

#88,"'tabalumab'/de OR 'ly 2127399' OR 'ly2127399' OR 'tabalumab*' OR '1143503 67 6' OR pqp8vh3mjw*",272,24 Nov 2020

#87,"abatacept*:ti,ab,kw,rn,tn OR 'ctla 4 ig':ti,ab,kw,rn,tn OR 'ctla-igg4m':ti,ab,kw,rn,tn OR 'ctla4-fc':ti,ab,kw,rn,tn OR '332348 12 6':ti,ab,kw,rn,tn OR 7d0yb67s97*:ti,ab,kw,rn,tn OR 'rg 2077':ti,ab,kw,rn,tn OR rg2077:ti,ab,kw,rn,tn OR 'rg-1046':ti,ab,kw,rn,tn OR rg1046:ti,ab,kw,rn,tn OR nulojix:ti,ab,kw,rn,tn OR lea29y:ti,ab,kw,rn,tn OR 'lea 29y':ti,ab,kw,rn,tn OR 'ctl4 fc':ti,ab,kw,rn,tn OR ctl4fc:ti,ab,kw,rn,tn OR belatacept:ti,ab,kw,rn,tn OR 'bms 224818':ti,ab,kw,rn,tn OR bms224818:ti,ab,kw,rn,tn OR 'cytotoxic t lymphocyte associat* antigen 4 immunglobulin*':ti,ab,kw,rn,tn",11021,24 Nov 2020

#86,"'abatacept'/de OR 'ctla4 ig' OR 'ctla4 immunoglobulin' OR 'ctla4 immunoglobulin g' OR 'ctla4ig' OR 'abatacept' OR 'bms 188667' OR 'bms188667' OR 'orencia'",11019,24 Nov 2020

#85,"'ofatumumab'/de OR 'humax cd20' OR 'humax-cd20' OR 'humaxcd20' OR 'arzerra' OR 'gsk 1841157' OR 'gsk1841157' OR 'humac cd20' OR 'ofatumumab*' OR 'omb 157' OR 'omb157' OR 'humax-cd20-2f2' OR '679818 59 8' OR m95kg522r0* OR 'hsdb 8170' OR hsdb8170",3082,24 Nov 2020

#84,"'rituximab'/de OR 'abp 798' OR 'abp798' OR 'blitzima' OR 'ct p10' OR 'ctp10' OR 'gp 2013' OR 'gp2013' OR 'hlx 01' OR 'hlx01' OR 'idec 102' OR 'idec c2b8' OR 'idec102' OR 'idecc2b8' OR 'mabthera' OR 'mab thera' OR 'mk 8808' OR 'mk8808' OR 'monoclonal antibody idec c2b8' OR 'pf 05280586' OR 'pf 5280586' OR 'pf05280586' OR 'pf5280586' OR 'r 105' OR 'r105' OR 'reditux' OR 'rg 105' OR 'rg105' OR 'ritemvia' OR 'ritumax' OR 'rituxan' OR 'rituximab*' OR 'rituximab abbs' OR 'rituximab pvvr' OR 'rituximab-abbs' OR 'rituximab-pvvr' OR 'rituxin' OR 'rituzena' OR 'rixathon' OR 'riximyo' OR 'ro 452294' OR 'ro452294' OR 'ruxience' OR 'truxima' OR 'tuxella' OR '174722 31 7' OR 4f4x42syq6* OR 'hsdb 7455' OR hsdb7455",86018,24 Nov 2020

#83,"'czp':ti,ab,kw,rn,tn OR '428863 50 7':ti,ab,kw,rn,tn OR g6adw90r16*:ti,ab,kw,rn,tn OR umd07x179e*:ti,ab,kw,rn,tn OR 'hsdb 7848':ti,ab,kw,rn,tn OR hsdb7848:ti,ab,kw,rn,tn",6872,24 Nov 2020

#82,"'certolizumab pegol'/de OR 'cdp 870' OR 'cdp870' OR 'certolizumab pegol' OR 'cimzia' OR 'pegylated tumor necrosis factor alpha antibody fab fragment' OR 'pegylated tumour necrosis factor alpha antibody fab fragment' OR 'pha 738144' OR 'pha738144'",6951,24 Nov 2020

#81,"'golimumab'/de OR 'cnto 148' OR 'cnto148' OR 'golimumab*' OR 'simponi' OR 'simponi aria' OR '476181 74 5' OR 91x1klu43e*",7369,24 Nov 2020

#80,"adalimumab*:ti,ab,kw,rn,tn OR fys6t7f842*:ti,ab,kw,rn,tn OR '331731 18 1':ti,ab,kw,rn,tn OR d2e7:ti,ab,kw,rn,tn OR 'hsdb 7851':ti,ab,kw,rn,tn OR hsdb7851:ti,ab,kw,rn,tn OR 'bcd-057':ti,ab,kw,rn,tn OR bcd057:ti,ab,kw,rn,tn OR 'chs-1420':ti,ab,kw,rn,tn OR chs1420:ti,ab,kw,rn,tn OR 'sb-5':ti,ab,kw,rn,tn OR sb5:ti,ab,kw,rn,tn",35443,24 Nov 2020

#79,"'adalimumab'/de OR 'abp 501' OR 'abp501' OR 'abrilada' OR 'abt d2e7' OR 'abtd2e7' OR 'adalimumab' OR 'adalimumab adaz' OR 'adalimumab adbm' OR 'adalimumab afzb' OR 'adalimumab atto' OR 'adalimumab beta' OR 'adalimumab bwwd' OR 'adalimumab-adaz' OR 'adalimumab-adbm' OR 'adalimumab-afzb' OR 'adalimumab-atto' OR 'adalimumab-bwwd' OR 'adaly' OR 'amgevita' OR 'amjevita' OR 'amsparity' OR 'avt 02' OR 'avt02' OR 'bat 1406' OR 'bat1406' OR 'bax 2923' OR 'bax 923' OR 'bax2923' OR 'bax923' OR 'bi 695501' OR 'bi695501' OR 'chs 1420' OR 'chs1420' OR 'cinnora' OR 'ct p17' OR 'ctp17' OR 'cyltezo' OR 'da 3113' OR 'da3113' OR 'dmb 3113' OR 'dmb3113' OR 'exemptia' OR 'fkb 327' OR 'fkb327' OR 'fyzoclad' OR 'gp 2017' OR 'gp2017' OR 'hadlima' OR 'halimatoz' OR 'hefiya' OR 'hlx 03' OR 'hlx03' OR 'hulio' OR 'humira' OR 'hyrimoz' OR 'ibi 303' OR 'ibi303' OR 'idacio' OR 'imraldi' OR 'kromeya' OR 'lu 200134' OR 'lu200134' OR 'm 923' OR 'm923' OR 'mabura' OR 'monoclonal antibody

d2e7' OR 'msb 11022' OR 'msb11022' OR 'ons 3010' OR 'ons3010' OR 'pf 06410293' OR 'pf 6410293' OR 'pf06410293' OR 'pf6410293' OR 'raheara' OR 'solymbic' OR 'trudexa' OR 'zrc 3197' OR 'zrc3197'",35374,24 Nov 2020

#78,"etanercept*:ti,ab,kw,tn,rn OR 'rhu tnfr fc':ti,ab,kw,tn,rn OR 'tnfr-immunoadhesin':ti,ab,kw,tn,rn OR 'tnt receptor fusion protein':ti,ab,kw,tn,rn OR 'tum$or necrosis factor receptor fc fusion protein':ti,ab,kw,tn,rn OR 'tnf receptor fusion protein':ti,ab,kw,tn,rn OR 'tnfr fc':ti,ab,kw,tn,rn OR 'recombinant human tnf':ti,ab,kw,tn,rn OR 'recombinant human dimeric tnf receptor type ii igg fusion protein':ti,ab,kw,tn,rn OR 'dwp-422':ti,ab,kw,tn,rn OR dwp422:ti,ab,kw,tn,rn OR 'hd-203':ti,ab,kw,tn,rn OR hd203:ti,ab,kw,tn,rn OR 'lbec 0101':ti,ab,kw,tn,rn OR lbec0101:ti,ab,kw,tn,rn OR 'sb 4':ti,ab,kw,tn,rn OR 'sb4':ti,ab,kw,tn,rn",16020,24 Nov 2020

#77,"'etanercept'/de OR 'avent' OR 'benepali' OR 'brenzys' OR 'chs 0214' OR 'chs0214' OR 'embrel' OR 'enbrel' OR 'enerceptan' OR 'enia 11' OR 'enia11' OR 'erelzi' OR 'etanercept*' OR 'etanercept szzs' OR 'etanercept ykro' OR 'etanercept-szzs' OR 'etanercept-ykro' OR 'eticovo' OR 'gp 2015' OR 'gp 2015c' OR 'gp2015' OR 'gp2015c' OR 'hd 203' OR 'hd203' OR 'infinitam' OR 'lbec 0101' OR 'lbec0101' OR 'lifmior' OR 'nepexto' OR 'opinercept' OR 'recombinant tumor necrosis factor receptor fc fusion protein' OR 'recombinant tumour necrosis factor receptor fc fusion protein' OR 'tnr 001' OR 'tnr001' OR 'tumor necrosis factor receptor fc fusion protein' OR 'tumour necrosis factor receptor fc fusion protein' OR 'tunex' OR 'ylb 113' OR 'ylb113' OR '185243 69 0' OR 'op401g7ojc*'",33175,24 Nov 2020

#76,"ifx:ti,ab,kw,de,tn,rn OR 'mab ca2':ti,ab,kw,de,tn,rn OR 'monoclonal antibody ca2':ti,ab,kw,de,tn,rn OR 'antibody ca2 monoclonal':ti,ab,kw,de,tn,rn OR 'ca2 monoclonal antibody':ti,ab,kw,de,tn,rn OR 'ct p13':ti,ab,kw,de,tn,rn OR ctp13:ti,ab,kw,de,tn,rn OR 'ct-p-13':ti,ab,kw,de,tn,rn OR sb2:ti,ab,kw,de,tn,rn OR 'hsdb 7850':ti,ab,kw,de,tn,rn OR hsdb7850:ti,ab,kw,de,tn,rn",5760,24 Nov 2020

#75,"'infliximab'/de OR 'abp 710' OR 'abp710' OR 'avakine' OR 'avsola' OR 'flixabi' OR 'gp 1111' OR 'gp1111' OR 'inflectra' OR 'infliximab*' OR 'infliximab abda' OR 'infliximab axxq' OR 'infliximab dyyb' OR 'infliximab qbtx' OR 'infliximab-abda' OR 'infliximab-axxq' OR 'infliximab-dyyb' OR 'infliximab-qbtx' OR 'ixifi' OR 'pf 06438179' OR 'pf 6438179' OR 'pf06438179' OR 'pf6438179' OR 'remicade' OR 'remsima' OR 'renflexis' OR 'revellex' OR 'ta 650' OR 'ta650' OR 'zessly' OR '170277 31 3' OR 'b72hh48flu*' OR 'inflecta'",52744,24 Nov 2020

#74,"((('il-1ra' OR il1ra OR 'il-1-ra') NEAR/10 (antagonist* OR 'blocking agent*' OR blocker* OR inhibitor* OR anakinra*)):ti,ab,kw,tn) AND (therap*:ti,ab,kw,de OR treat*:ti,ab,kw,de OR trial$:ti,ab,kw,de)",2221,24 Nov 2020

#73,"'anakinra'/de OR 'anakinra*' OR 'kineret' OR 'recombinant interleukin 1 receptor antagonist' OR 'recombinant interleukin 1 receptor blocker' OR 'recombinant interleukin 1 receptor blocking agent' OR '143090 92 0'",9236,24 Nov 2020

#72,"#61 AND #71",10768,24 Nov 2020

#71,"#62 OR #63 OR #64 OR #65 OR #66 OR #67 OR #68 OR #69 OR #70",30175,24 Nov 2020

#70,"'elsilimomab'/de OR 'b e8' OR 'elsilimomab*' OR '468715 71 1'",38,24 Nov 2020

#69,"'ziltivekimab'/de OR 'cor 001' OR 'cor001' OR 'ziltivekimab*' OR ziltivekimab* OR zilti OR '2226654 05 1'",4,24 Nov 2020

#68,"'siltuximab'/de OR 'cnto 328' OR 'cnto328' OR 'monoclonal antibody cnto 328' OR 'monoclonal antibody cnto328' OR 'siltuximab*' OR 'sylvant' OR cllb8 OR 'ccib8 monoclonal antibody' OR t4h8fma7im* OR '541502 14 1'",853,24 Nov 2020

#67,"'sirukumab'/exp OR 'cnto 136' OR 'cnto136' OR 'plivensia' OR 'sirukumab*' OR '1194585 53 9' OR 640443fu93*",320,24 Nov 2020

#66,"'clazakizumab'/de OR 'ald 518' OR 'ald518' OR 'bms 945429' OR 'bms945429' OR 'clazakizumab*' OR '1236278 28 6' OR 4s38z8ra9o*",216,24 Nov 2020

#65,"'olokizumab'/de OR 'olokizumab*' OR 'cdp 6038' OR cdp6038 OR '1007223 17 7' OR pai71r1d2w*",95,24 Nov 2020

#64,"'levilimab'/de OR 'bcd 089' OR 'bcd089' OR 'levilimab*' OR '2035008 70 7' OR p7uv3l2h80*",5,24 Nov 2020

#63,"'sarilumab'/de OR 'kevzara*' OR 'regn 88' OR 'regn88' OR 'sar 153191' OR 'sar153191' OR 'sarilumab*' OR '1189541 98 7' OR nu90v55f8i*",812,24 Nov 2020

#62,"'tocilizumab'/de OR 'actemra*' OR 'actemra 200' OR 'atlizumab*' OR 'lusinex*' OR 'r1569' OR 'roactemra*' OR 'tocilizumab*' OR r1569 OR 'r 1569' OR 'mra':ti,ab,kw,tn OR mra:tt OR '375823 41 9' OR i031v2h011* OR 'rhpm-1' OR rhpm1 OR 'rg-1569' OR rg1569 OR 'msb-11456' OR msb11456 OR 'ro-4877533' OR ro4877533",29097,24 Nov 2020

#61,"#1 OR #2 OR #3 OR #4 OR #5 OR #6 OR #7 OR #8 OR #9 OR #10 OR #11 OR #12 OR #13 OR #14 OR #15 OR #16 OR #22 OR #23 OR #24 OR #25 OR #26 OR #27 OR #28 OR #29 OR #30 OR #31 OR #32 OR #33 OR #34 OR #35 OR #36 OR #37 OR #38 OR #39 OR #40 OR #41 OR #42 OR #43 OR #44 OR #45 OR #46 OR #47 OR #48 OR #49 OR #50 OR #51 OR #54 OR #55 OR #56 OR #57 OR #58 OR #59 OR #60",323425,24 Nov 2020

#60,"(('lymph* node' NEAR/3 hyperplas* NEAR/3 giant):ti,ab,kw) OR (('lymph* node' NEAR/3 hyperplas* NEAR/3 giant):tt)",310,24 Nov 2020

#59,"((angiofollicul* OR 'angio follicul*') NEAR/3 lymph* NEAR/3 hyperplas*):tt",12,24 Nov 2020

#58,"((angiofollicul* OR 'angio follicul*') NEAR/3 lymph* NEAR/3 hyperplas*):ti,ab,kw",409,24 Nov 2020

#57,"(castleman* NEAR/3 (disease* OR tumo$r* OR 'pseudo tumo$r*' OR pseudotumo$r* OR lymph* OR pseudolymphom* OR 'pseudo lymphom*')):tt",42,24 Nov 2020

#56,"(castleman* NEAR/3 (disease* OR tumo$r* OR 'pseudo tumo$r*' OR pseudotumo$r* OR lymph* OR pseudolymphom* OR 'pseudo lymphom*')):ti,ab,kw",4592,24 Nov 2020

#55,"'angiofollicular lymph node hyperplasia'/de",5353,24 Nov 2020

#54,"#52 NOT #53",1299,24 Nov 2020

#53,"(('chronic sinusitis' OR 'chronic rhinosinusitis' OR 'cytoreductive surg*' OR 'complete response' OR 'complete responders') NEAR/6 crs):ti,ab,kw",5808,24 Nov 2020

#52,"(crs:ti,ab,kw OR crs:tt) AND ('cytokine releas*':ti,ab,kw,de OR 'cytokine storm':ti,ab,kw,de)",1356,24 Nov 2020

#51,"((('cytokine releas*' OR 'cytokine storm') NEAR/3 syndrom*):ti,ab,kw,de) OR ((('cytokine releas*' OR 'cytokine storm') NEAR/3 syndrom*):tt)",4027,24 Nov 2020

#50,"'cytokine release syndrome'/de",3368,24 Nov 2020

#49,"(('brachiocephal*' NEAR/6 arterit*):ti,ab,kw) OR (('brachiocephal*' NEAR/6 arterit*):tt)",29,24 Nov 2020

#48,"(('young female' NEAR/6 arterit*):ti,ab,kw) OR (('young female' NEAR/6 arterit*):tt)",29,24 Nov 2020

#47,"(takayasu* NEAR/3 (arterit* OR syndrom* OR disease*)):tt",255,24 Nov 2020

#46,"(takayasu* NEAR/3 (arterit* OR syndrom* OR disease*)):ti,ab,kw",6471,24 Nov 2020

#45,"'aortic arch syndrome'/de",9136,24 Nov 2020

#44,"(gca:ti,ab,kw OR gca:tt) AND (arterit*:ti,ab,kw,de OR vasculit*:ti,ab,kw,de OR r$eum*:ti,ab,kw,de,jt)",3570,24 Nov 2020

#43,"(('large vessel' NEAR/3 (vasculit* OR arterit*)):ti,ab,kw) OR (('large vessel' NEAR/3 (vasculit* OR arterit*)):tt)",2120,24 Nov 2020

#42,"'large vessel vasculitis'/de",234,24 Nov 2020

#41,"'temporal arteritis'/de",4255,24 Nov 2020

#40,"((horton OR 'horton s' OR hortons) NEAR/3 disease*):ti,ab,kw",578,24 Nov 2020

#39,"((temporal OR cranial) NEAR/2 arterit*):ti,ab,kw",3149,24 Nov 2020

#38,"('giant cell' NEAR/6 (arterit* OR aortit* OR horton*)):ti,ab,kw",7692,24 Nov 2020

#37,"'giant cell arteritis'/de",8286,24 Nov 2020

#36,"(aosd:ti,ab,kw OR aosd:tt) AND (inflammator*:ti,ab,kw,de OR autoinflammator*:ti,ab,kw,de OR r$eumat*:ti,ab,kw,de,jt)",964,24 Nov 2020

#35,"'still s disease*':tt OR 'stll s disease*':tt OR 'stills disease*':tt OR 'stlls disease*':tt",5,24 Nov 2020

#34,"'still s disease*':ti,ab,kw OR 'stll s disease*':ti,ab,kw OR 'stills disease*':ti,ab,kw OR 'stlls disease*':ti,ab,kw",3025,24 Nov 2020

#33,"(('stills disease*' OR 'still s disease*' OR 'still disease*') NEAR/6 (adult* OR 'adult onset')):tt",5,24 Nov 2020

#32,"(('stills disease*' OR 'still s disease*' OR 'still disease*') NEAR/6 (adult* OR 'adult onset')):ti,ab,kw",2658,24 Nov 2020

#31,"'adult onset still disease'/de",2281,24 Nov 2020

#30,"(polyart*:tt OR oligoart*:tt OR 'poly art*':tt OR 'oligo art*':tt) AND (jia:tt OR 'juvenile idiopathic arthritis':tt OR 'juvenile onset idiopathic arthritis':tt) OR pjia:tt",1,24 Nov 2020

#29,"(polyart*:ti,ab,kw OR oligoart*:ti,ab,kw OR 'poly art*':ti,ab,kw OR 'oligo art*':ti,ab,kw) AND (jia:ti,ab,kw OR 'juvenile idiopathic arthritis':ti,ab,kw OR 'juvenile onset idiopathic arthritis':ti,ab,kw) OR pjia:ti,ab,kw",3367,24 Nov 2020

#28,"(('stills disease*' OR 'still s disease*' OR 'still disease*') NEAR/6 child*):tt",0,24 Nov 2020

#27,"(('stills disease*' OR 'still s disease*' OR 'still disease*') NEAR/6 child*):ti,ab,kw",89,24 Nov 2020

#26,"('stills disease*':tt OR 'still s disease*':tt OR 'still disease*':tt) AND juvenile*:tt",1,24 Nov 2020

#25,"('stills disease*':ti,ab,kw OR 'still s disease*':ti,ab,kw OR 'still disease*':ti,ab,kw) AND juvenile*:ti,ab,kw",514,24 Nov 2020

#24,"((polyarthr* OR polyartrit* OR 'poly arthrit*' OR 'poly artrit*' OR oligoarthrit* OR oligoartrit* OR 'oligo arthrit*' OR 'oligo artrit*') NEAR/6 juvenile*):tt",80,24 Nov 2020

#23,"((polyarthr* OR polyartrit* OR 'poly arthrit*' OR 'poly artrit*' OR oligoarthrit* OR oligoartrit* OR 'oligo arthrit*' OR 'oligo artrit*') NEAR/6 juvenile*):ti,ab,kw",326,24 Nov 2020

#22,"#20 AND #21",9198,24 Nov 2020

#21,"systemic:ti,ab,kw OR polyart*:ti,ab,kw OR 'poly art*':ti,ab,kw OR oligoart*:ti,ab,kw OR 'oligo art*':ti,ab,kw OR systemic:tt OR polyart*:tt OR 'poly art*':tt OR oligoart*:tt OR 'oligo art*':tt",719006,24 Nov 2020

#20,"#17 OR #18 OR #19",24676,24 Nov 2020

#19,"jia:ti OR jia:tt",1260,24 Nov 2020

#18,"(((arthrit* OR artrit*) NEAR/6 juvenile*):ti,ab,kw) OR (((arthrit* OR artrit*) NEAR/6 juvenile*):tt)",17877,24 Nov 2020

#17,"'juvenile rheumatoid arthritis'/de",21376,24 Nov 2020

#16,"((systemic NEAR/6 (jia OR 'juvenile idiopathic arthritis' OR 'juvenile onset idiopathic arthriti')):ti,ab,kw) OR sjia:ti,ab,kw",3323,24 Nov 2020

#15,"'systemic juvenile idiopathic arthritis'/de",1401,24 Nov 2020

#14,"'rheumatic disease'/de",50367,24 Nov 2020

#13,"((rheumat* OR reumat* OR revmat* OR rheumo OR reumo) NEAR/2 (condition* OR diseas*)):tt",13,24 Nov 2020

#12,"((rheumat* OR reumat* OR revmat* OR rheumo OR reumo) NEAR/2 (condition* OR diseas*)):ti,ab,kw",50814,24 Nov 2020

#11,"((r$eum* OR r$evm*) NEAR/2 (chronic* OR articular*)):tt",87,24 Nov 2020

#10,"((r$eum* OR r$evm*) NEAR/2 (chronic* OR articular*)):ti,ab,kw",5686,24 Nov 2020

#9,"(inflammator* NEAR/2 (arthrit* OR artrit*)):tt",3,24 Nov 2020

#8,"(inflammator* NEAR/2 (arthrit* OR artrit*)):ti,ab,kw",12372,24 Nov 2020

#7,"(chronic* NEAR/2 (polyarthrit* OR 'poly arthrit*' OR polyartrit* OR 'poly artrit*')):tt",86,24 Nov 2020

#6,"(chronic* NEAR/2 (polyarthrit* OR 'poly arthrit*' OR polyartrit* OR 'poly artrit*')):ti,ab,kw",2521,24 Nov 2020

#5,"r$eumarthrit*:tt OR r$eumartrit*:tt OR 'r$eum arthrit*':tt OR 'r$eum artrit*':tt OR r$evmarthrit*:tt OR r$evmartrit*:tt OR 'r$evm arthrit*':tt OR 'r$evm artrit*':tt OR 'r$eum polyarthrit*':tt OR 'r$eum polyartrit*':tt OR 'r$eum poly arthrit*':tt OR 'r$eum poly artrit*':tt OR r$eumpolyarthrit*:tt OR r$eumpolyartrit*:tt",0,24 Nov 2020

#4,"r$eumarthrit*:ti,ab,kw OR r$eumartrit*:ti,ab,kw OR 'r$eum arthrit*':ti,ab,kw OR 'r$eum artrit*':ti,ab,kw OR r$evmarthrit*:ti,ab,kw OR r$evmartrit*:ti,ab,kw OR 'r$evm arthrit*':ti,ab,kw OR

'r$evm artrit*':ti,ab,kw OR 'r$eum polyarthrit*':ti,ab,kw OR 'r$eum polyartrit*':ti,ab,kw OR 'r$eum poly arthrit*':ti,ab,kw OR 'r$eum poly artrit*':ti,ab,kw OR r$eumpolyarthrit*:ti,ab,kw OR r$eumpolyartrit*:ti,ab,kw",10,24 Nov 2020

#3,"((rheumat* OR reumat* OR revmat* OR rheumo OR reumo) NEAR/3 (arthrit* OR artrit* OR polyarthrit* OR polyartrit*)):tt",6608,24 Nov 2020

#2,"((rheumat* OR reumat* OR revmat* OR rheumo OR reumo) NEAR/3 (arthrit* OR artrit* OR polyarthrit* OR polyartrit*)):ti,ab,kw",171191,24 Nov 2020

#1,"'rheumatoid arthritis'/de",199060,24 Nov 2020

1.3.3.3 Cochrane CENTRAL

1 Arthritis, Rheumatoid/ (5819)

2 ((rheumat* or reumat* or revmat* or rheumo or reumo) adj3 (arthrit* or artrit* or polyarthrit* or polyartrit*)).mp. (17097)

3 (r?eumarthrit* or r?eumartrit* or r?eum arthrit* or r?eum artrit* or r?evmarthrit* or r?evmartrit* or r?evm arthrit* or r?evm artrit* or r?eum polyarthrit* or r?eum polyartrit* or r?eum poly arthrit* or r?eum poly artrit* or r?eumpolyarthrit* or r?eumpolyartrit*).mp. (3)

4 (chronic* adj2 (polyarthrit* or poly arthrit* or polyartrit* or poly artrit*)).mp. (143)

5 (inflammator* adj2 (arthrit* or artrit*)).mp. (668)

6 ((r?eum* or r?evm*) adj2 (chronic* adj2 articular*)).mp. (14)

7 ((rheumat* or reumat* or revmat* or rheumo or reumo) adj2 (condition* or diseas*)).mp. (5045)

8 Arthritis, Juvenile/ (299)

9 ((arthrit* or artrit*) adj6 juvenile*).mp. (1001)

10 JIA.ti,ot,ab. (627)

11 8 or 9 or 10 (1123)

12 (systemic or polyart* or poly art* or oligoart* or oligo art*).mp. (52595)

13 11 and 12 (515)

14 ((polyarthr* or polyartrit* or poly arthrit* or poly artrit* or oligoarthrit* or oligoartrit* or oligo arthrit* or oligo artrit*) adj6 juvenile*).mp. (9)

15 (((stills or still's or still) adj disease) and juvenile*).mp. (31)

16 ((stills or still's or still) adj disease adj6 child*).mp. (1)

17 ((systemic adj6 JIA) or sJIA or s-JIA).mp. (167)

18 (((polyart* or oligoart* or poly art* or oligo art*) and JIA) or pJIA or p-JIA).mp. (211)

19 Still's Disease, Adult-Onset/ (8)

20 ((stills or still's or still) adj disease adj6 adult*).mp. (52)

21 Giant Cell Arteritis/ (90)

22 (giant cell adj6 (arterit* or aortit* or horton*)).mp. (297)

23 ((temporal or cranial) adj2 arterit*).mp. (44)

24 ((horton or horton's or hortons) adj3 disease?).mp. (4)

25 (large vessel adj3 (vasculit* or arterit*)).mp. (55)

26 GCA.ti,ot,ab. and (arterit* or vasculit* or rheum*).mp,jw. (209)

27 Takayasu Arteritis/ (27)

28 (takayasu* adj3 (arterit* or syndrom* or disease*)).mp. (102)

29 (young female adj6 arterit*).mp. (0)

30 (brachiocephal* adj6 arterit*).mp. (0)

31 Cytokine Release Syndrome/ (2)

32 ((cytokine releas* or cytokine storm) adj3 syndrome*).mp. (257)

33 crs.ti,ot,ab. and (cytokine releas* or cytokine storm).mp. (97)

34 ((chronic sinusitis or chronic rhinosinusitis or chronic rhino sinusitis or cytoreductive surg* or complete response or complete responders) adj6 crs).ti,ot,ab. (453)

35 33 not 34 (93)

36 Castleman Disease/ (12)

37 ((angiofollicul* or angio follicul*) adj3 lymph* adj3 hyperplas*).mp. (40)

38 (lymph* node adj3 hyperplas* adj3 giant).mp. (2)

39 (castleman* adj3 (disease* or tumo?r* or pseudo-tumo?r* or pseudotumo?r* or lymph* or pseudolymphom* or hyperplas*)).mp. (48)

40 1 or 2 or 3 or 4 or 5 or 6 or 7 or 13 or 14 or 15 or 16 or 17 or 18 or 19 or 20 or 21 or 22 or 23 or 24 or 25 or 26 or 27 or 28 or 29 or 30 or 31 or 32 or 35 or 36 or 37 or 38 or 39 (20328)

41 (tocilizumab* or actemra* or atlizumab* or lusinex* or r1569 or r 1569 or roactemra* or mra or 375823 41 9 or i031v2h011* or rhpm-1 or rhpm1 or rg-1569 or rg1569 or msb-11456 or msb11456 or ro-4877533 or ro4877533).mp. (1989)

42 (sarilumab* or kevzara* or regn 88 or regn88 or sar 153191 or sar153191 or 1189541 98 7 or nu90v55f8i*).mp. (255)

43 (levilimab* or BCD-089 or bcd089 or 2035008 70 7 or P7UV3L2H80*).mp. (5)

44 (olokizumab* or cdp 6038 or cdp6038 or 1007223 17 7 or pai71r1d2w*).mp. (35)

45 (clazakizumab* or ald 518 or ald518 or bms 945429 or bms945429 or 1236278 28 6 or 4s38z8ra9o*).mp. (38)

46 (sirukumab* or cnto 136 or cnto136 or pilvensia or 1194585 53 9 or 640443FU93*).mp. (118)

47 (siltuximab* or sylvant or cnto 328 or cnto-328 or cnto328 or cllb8 or ccib8 monoclonal antibody or sylvant or t4h8fma7im* or 541502 14 1).mp. (78)

48 (ziltivekimab* or zilti or "cor-001" or "2226654 05 1").mp. (5)

49 (elsilimomab* or "b-e8" or 468715 71 1).mp. (2)

50 or/41-49 (2464)

51 40 and 50 (1320)

52 Interleukin 1 Receptor Antagonist Protein/ (305)

53 (anakinra* or kineret or ((interleukin 1 receptor or il1 receptor or il 1 receptor) adj1 antagonist protein*) or (recombinant interleukin 1 receptor adj1 (antagonist* or blocker* or blocking agent*)) or antril or il 1ra or il1ra or il 1 ra or ((urin* or febrile) adj2 (interleukin 1 inhibitor* or il1 inhibitor* or il 1 inhibitor*)) or "143090 92 0" or 9013duq28k*).mp. (1087)

54 Infliximab/ (732)

55 (infliximab* or ifx or avakine or flixabi or inflectra or inflecta or ixifi or remicade or remsima or revellex or renflexis or zessly or mab ca2 or monoclonal antibody ca2 or antibody ca2 monoclonal or ca2 monoclonal antibody or ct p13 or ctp13 or ct-p-13 gp-1111 or gp1111 or ta-650 or ta650 or abp 710 or abp710 or bow-015 or bow015 or 170277 31 3 or b72hh48flu* or sb2 or "pf 06438179" or pf 6438179 or pf06438179 or pf6438179 or hsdb 7850 or hsdb7850).mp. (2769)

56 Etanercept/ (758)

57 (etanercept* or benepali or embrel or enbrel or enia 11 or enia11 or erelzi or lifmior or opinercept or recombinant tumo?r necrosis factor receptor fc fusion protein or "tnr 001" or tnr001 or tnfr-fc or tnrfr:fc or rhu tnfr:fc or rhu-tnfr:fc or tnfr-immunoadhesin or tnt receptor fusion protein or tum?or necrosis factor receptor fc fusion protein or tunex or "185243 69 0" or op401g7ojc* or brenzys or tnf receptor fusion protein or tnfr fc or recombinant human tnf or recombinant human dimeric tnf receptor type ii igg fusion protein or chs-0214 or chs0214 or dwp-422 or dwp422 or enia-11 or enia11 or gp-2015 or gp2015 or gp2015c or gp-2015c or hd-203 or hd203 or lbec-0101 or lbec0101 or sb-4 or sb4).mp. (2451)

58 Adalimumab/ (752)

59 (adalimumab* or humira or abp 501 or abp501 or abtd2e7 or amjevita or amgevita or bax 2923 or bax2923 gp 2017 or gp2017 or halimatoz or hefiya or hulio or hyrimoz or ibi 303 or ibi303 or imraldi or m 923 or m923 or msb 11022 or msb11022 or ons3010 or ons 3010 or "pf 06410293" or pf 6410293 or pf06410293 or pf6410293 or d2e7 or truxeda or cyltezo or solymbic or fys6t7f842* or 331731 18 1 or lu 200134 or lu200134 or d2e7 or hsdb 7851 or hsdb7851 or abp-501 or abp501 or bcd-057 or bcd057 or bi-695501 or bi695501 or chs-1420 or chs1420 or gp-2017 or gp2017 or sb-5 or sb5).mp. (3403)

60 (golimumab* or cnto 148 or cnto148 or simponi or 476181 74 5 or 91x1klu43e*).mp. (751)

61 Certolizumab Pegol/ (167)

62 (certolizumab* or cdp 870 or cdp870 or cimzia or pegylated tumor necrosis factor alpha antibody fab fragment or pegylated tumour necrosis factor alpha antibody fab fragment or pha 738144 or pha738144 or czp or 428863 50 7 or g6adw90r16* or umd07x179e* or hsdb 7848 or hsdb7848).mp. (705)

63 Rituximab/ (1268)

64 (rituximab* or ct p10 or ctp10 or idec 102 or idec102 or idecc2b8 or idec c2b8 or monoclonal antibody idec c2b8 or gp2013 or gp 2013 or "pf 05280586" or pf05280586 or mabthera or mab thera or r 105 or r105 or reditux or rg 105 or rg105 or rituxan or rituxin or ritemvia or rituzena or rixathon or riximyo or ro 452294 or ro452294 or truxima or 174722 31 7 or 4f4x42syq6* or hsdb 7455 or hsdb7455).mp. (5326)

65 (ofatumumab* or humax cd20 or humax-cd20 or humaxcd20 or humax-cd20-2f2 or arzerra or gsk 1841157 or gsk1841157 or gski841157 or human cd20 or omb 157 or omb157 or 679818 59 8 or m95kg522r0* or hsdb 8170 or hsdb8170).mp. (299)

66 Abatacept/ (280)

67 (abatacept* or ctla4 ig or ctla4 immunglobulin or ctla4 immunoglobulin g or ctla4ig or 'ctla 4 ig' or ctla-igg4m or ctla4-fc or bms 188667 or bms188667 or orencia or '332348 12 6' or 7d0yb67s97* or rg 2077 or rg2077 or rg-1046 or rg1046 or nulojix or lea29y or lea 29y or ctl4 fc or ctl4fc or belatacept or bms 224818 or bms224818 or belatacept or cytotoxic t lymphocyte associat* antigen 4).mp. (1151)

68 (tabalumab* or ly 2127399 or ly2127399 or 1143503 67 6 or pqp8vh3mjw*).mp. (70)

69 (pateclizumab* or mlta 3698a or mlta3698a or pro 283698 or pro283698 or 12202526 59 7 or qok1yyh7j2* or rg 7415 or rg7415).mp. (7)

70 (secukinumab* or cosentyx or ain 457 or ain457 or 1229022 83 6 or dlg4eml025*).mp. (917)

71 (ixekizumab* or ly 2439821 or ly2439821 or taltz or 1329632 62 3 or 1143503 69 8 or bty153760o*).mp. (489)

72 (brodalumab* or amg 827 or amg827 or khk 4827 or khk4827 or siliq or kyntheum or 1174395 19 7 or 6za31y954z*).mp. (178)

73 (guselkumab* or cnto 1959 or cnto1959 or 1350289 85 8 or tremfya or 1350289 85 8 or 089658a12d*).mp. (227)

74 Ustekinumab/ (195)

75 (ustekinumab* or cnto 1275 or cnto1275 or stelara or "815610 63 0" or fu77b4u5z0* or 1275 cnto or 1275cnto or l04ac05 or tt-20 or tt20).mp. (897)

76 (mavrilimumab or "1085337 57 0" or 1158jdp9a* or cam 3001 or cam3001).mp. (53)

77 exp Antirheumatic Agents/ (34893)

78 (dmard* or csdmard* or cs-dmard* or sdmard* or s-dmard* or tsdmard* or ts-dmard* or bdmard* or b-dmard*).mp. (3147)

79 (disease modifying adj3 (antirheum* or anti rheum*) adj3 (drug? or agent?)).mp. (2123)

80 ((antirheum* or anti rheum*) adj5 (drug? or agent?)).mp. (4248)

81 Methotrexate/ (4137)

82 (mxt or methotrexat* or metotrexat* or methoblastin or methylaminopterin* or amet?opterin* or abitrexate or antifolan or brimexate or a-met?opterin* or a-methpterin* or ai3-25299 or ai325299 or alpha-methopterin or biotrexate or emtexate or emtrexate* or emthexat* or enthexate* or farmitrexat* or farmotrex or fauldexato or folex or folex pfs or hdmtx or ifamet or imeth or maxtrex or metotressat* or methotrate* or methohexate* or mexate* or metrotex or metatrexato* or metex or metrex or metatrexan or maxtrex or meticil or metoject or metotrexin or novatrex* or neotrexate* or nordimet or lumexon or ledertrexate* or lanterel or rasuvo or r?eumatrex* or reditrex or rasuvo or texate or texate-t or tremetex or trexeron or trixilem or texorate or trexall or xaken or xatmep or otrexup or mpi-2505 or mpi2505 or mpi 5004 or mpi5004 or amethopterin* or 133073-73-1 or 15475-56-6 or 3ig1e710zn* or yl5fz2y5u1* or cl 14377 or cl14377 or ccris 1109 or ccris1109 or emt 25299 or emt25299 or nsc-740 or nsc740 or r-9985 or r9885 or x-133 or wr-19039 or wr19039).mp. (12111)

83 Leflunomide/ (153)

84 (leflunomid* or hwa 486 or hwa486 or su101 or su 101 or arava or arabloc or hsdb 7289 or hsdb7289 or g162gk9u4w* or 75706-12-6 or rs 34821 or rs34821).mp. (688)

85 lef.ti,ot,ab. (173)

86 Sulfasalazine/ (469)

87 (sulfasalazin* or sasp or ssz or salicylazosulfapyridin* or salicyl azo sulfapyridin* or sulphasalazin* or salazosulfapyridin* or salazosulfapiridin* or 'pyralin en' or azulfadin* or azulfidin* or asulfidin* or azulfide* or azulfin or colo-pleon or colopleon or disalazin or gastropyrin or pleon or "pleon ra" or "pyralin en" or rorasul or rosulfant or ulcol or ucine or salazopyrin* or ratio-sulfosalazin* or sulfasalazin* or 3xc8guz6cb* or accucol or asulfidin* or azopyrin* or azosulfidin* or benzosulfa or "brn 0356241" or brn0356241 or ccris 4713 or ccris4713 or einecs 209-974-3 or hsdb 3395 or hsdb3395 or nsc 203730 or nsc203730 or nsc 667219 or nsc667219 or reupirin or rorasul or salazo-sulfapyridin* or salazosulfpyridin* or salazopiridazin* or salazopyridin* or salazopirin* or salazodin or salisulf or salipyr or saridine or si-88 or si88 or sulculon or sulfasalazin* or sulfasalizin* or sulphasalazin* or w-t sasp oral or 599-79-1 or "s.a.s. 500" or "s.a.s.-500" or sas-500 or zopyrin*).mp. (1596)

88 Hydroxychloroquine/ (505)

89 (hydroxychloroquin* or hcq or 747-3-4 or 8q2869cnvh* or 118-42-3 or 4qwg6n8qkh* or hydroxychlorochin* or hydrochloroquin* or hydrocloroquin* or oxychlorochin or oxychloroquin* or plaquenil or plaquinol or "brn 0253894" or brn0253894 or einecs 204-249-8 or einecs204-249-8 or hidroxicloroquin* or idrossiclorochin* or oxichlorochin* or oxichloroquin* or oxiklorin or win1258 or win 1258 or z0188 or einecs 212-019-3 or einecs212-019-3 or chloroquinol or dimard or ercoquin or evoquin or erquin or geniquin or quensyl or sn 8137 or sn8137 or toremonil or yuma).mp. (1666)

90 Gold Sodium Thiomalate/ (91)

91 (gold sodium thiomalate* or aurothiomalate* or aurolate* or gold thiomalate* or gold disodium thiomalate* or gold thiomalic acid* or miocrin or miocrisin or monogold disodium thiomalate* or myochrysin* or myocrisin* or myocrysin* or sodium aurothiomalate* or sodium gold thiomalate* or sodium thiomalate* gold or sodium thiomalatoaurate* or tauredon or taureodon or tauredone or thiomalate gold or thiomalatoaurate sodium or 1244-57-4 or 39377-38-3 or 4846-27-9 or 74916-57-7 or e4768zy6gm* or aurothiomala-natrium or aurot?iomalato sodico or monogold* sodium salt or dinatrium 2-aurothio-succinat or einecs 235-479-7 or einecs235-479-7 or hsdb 7173 or hsdb7173 or kidon or natrii aurothiomalas* or shiosol or gold mercatpsuccinate*).mp. (156)

92 Aurothioglucose/ (36)

93 (thioglucosoaurate* or gold thioglucose* or gold thio glucose* or thioglucose gold or aureotan or solganal or solganol or gold-50 or gold50 or solganal b or b oleosum solganal or auromyose* or aurothioglucose* or auro thioglucose* or aurotan or aurumine or aurumin or authron or brenol or ccris 59 or ccris59 or einecs 235-365-7 or einecs235-365-7 or glysanol b or goldthioglucose* or goldtioglucose* or hsdb 7174 or hsdb7174 or oronol or romosol or skf 10056 or skf10056 or 2p2v9q0e78* or 12192-57-3).mp. (49)

94 ((gold* or auro*) adj10 (inject* or intramuscular* or im or intra muscular*)).mp. (268)

95 Organogold Compounds/ (15)

96 (aurothiopropanol* or allochrysine or allocrisine or aurotiopoe or aurotioprol or 27279-43-2 or g7097j63e9* or auromercapto propanol sulfonate sodium or auromercaptopropanolsulfonate sodium or aurothioisopropanolsulfonate sodium or sodium aurothiopropanolsulfonate or gold sodium thiopropanol sulfonate or sodium auromercaptopropanol sulfonate).mp. (3)

97 Chloroquine/ (709)

98 (chloroquin* or cq or 54-04-7 or 886u3h6uff* or aralen or aralene or arechin or arechine or arequin or chingamin or chlorochin* or khingamin or nivaquine or oe48649kh6n* or amokin or amokine or anaclor or aralan or arequine or artriquine or avloclor or avoclor or arthrochin or artrichin or bemaco or bemaphata or bemaphate or bemasulph or benaquin or bipiquin or cadiquin or "brn 0482809" or brn0482809 or capquin or ccris 3439 or ccris3439 or chemochin or chemochine or chingamine or chingaminum or chloraquin* or chlorquin or chlorquine or choloquine or choroquine sulfate or choroquine sulphate or cindacin or clo-kit junior or clorichina or clorichine or cloriquine or clorochina or delagil or delagyl or diclokin or chlorofoz or cindachin or clorochina

or cloroquina or cocatrit or dichinalex or diquinalex or diroquine or emquin or genocin or gontochin or gontochine or gontoquine or einecs 200-191-2 or einecs200-191-2 or elestol or gontochin or heliopar or hsdb 3029 or hsdb3029 or imagon or iroquine or klorokin or klorokine or klorokinfosfat or lagaquin or lapaquin or malaquin or malaren or malarex or malarivon or malaviron or maliaquine or maquine or mesylith or mexaquin or mirquin or neochin or nivaquin or nivachine or nivaquine b or nivaquine dp or nivaquine forte or "p roquine" or nsc 187208 or nsc187208 or pfizerquine or quensyl or quinachlor or quinercyl or quingamine or quinilon or quinoline or quinoscan or repal or resochen or resochene or resochin or resochin junior or resochina or resochine or resochinon or resoquina or resoquine or reumachlor or ronaquine or roquine or rp 3377 or rp3377 or sanoquin or sanoquine or silbesan or siragan or sirajan or sn 7618 or sn7618 or solprina or solprine or sopaquin or st 21 or tresochin or tresoquine or trochin or trochine or troquine or weimerquin or w 7618 or w7618 or win 244 or win244).mp. (2086)

99 exp Adrenal Cortex Hormones/ (27587)

100 exp Prednisolone/ (4835)

101 Prednisone/ (3978)

102 Cortisone/ (148)

103 Hydrocortisone/ (5944)

104 exp Steroids/ (53553)

105 adrenal cortex hormones.mp. (2224)

106 prednisolone*.mp. (7414)

107 methylprednisolone*.mp. (5485)

108 (prednisolone* or predonine or di-adreson-f or diadresonf or 9phq9y1olm* or prednisolona* or prednisolonum* or nsc-9120 or nsc9120 or nsc 9900 or nsc9900 or delta-dehydrocortisol* or delta-dehydrocortisone* or delta-hydrocortisone or hydroretrocortine or metacortandralone* or brn 1354103 or brn1354103 or bubbli-pred or ccris 980 or ccris980 or co-hydeltra or codelcortone or cordrol or cortalone or cotogesic or cotolone or decaprednil or decortin h or delcortol or dehydrohydrocortisone* or delta-cortef or deltacortef or delta-ef-cortelan or delta-stab or deltacortenol or deltacortril or deltahydrocortisone* or deltisilone or derpo pd or dexa-dortidelt hostacortin h or di-adreson f or dicortol or donisolone or dydeltrone or eazolin d or ec 200-021-7 or ec200-021-7 or einecs 200-021-7 or einecs200-021-7 or erbacort or erbasona or estilsona or fernisolone or fernisolone p or hostacortin h or hsdb 3385 or hsdb3385 or hydeltra or hydeltrone or hydrodeltalone or hydrodeltisone or hydroretrocortin or hydroretrocortine* or k 1557 or k1557 or lentosone or meti-drm or meticortelone or orapred or orapred odt or paracortol or paracotol or pediapred or precortancyl or precortilon or precortisyl or predne-dome or prednelan or prednicen or

predniliderm or predniretard or prednis or predonin or predonine* or prelon or prelone or prenolone or rolisone or scherisolon? or solone or sterolone or ulacort or ultracorten h or ultracortene h adelcort or antisolon or antisolone or aprednisolon? or benisolon? or caberdelta or co hydeltra or codelcortone or ompresolon).mp. (7432)

109 (cortadeltona or cortelinter or cortisolone or dacortin or dacortin h or dacrotin or decaprednil or decortil or delta cortril or delta hycortol or deltacortenolo or deltacortil or deltacortoil or deltaderm or deltadglycortril or deltahycortol or deltahydrocortison? or deltaophticor or deltasolone or deltastab or deltidrosol or deltisilone or deltisolon? or deltolasson? or deltosona or deltosone or depo-predate or dermosolon or dhasolone or di adreson? f or diadreson? f or dicortol or domucortone or encortelon? or encortolon or equisolon or glistelone or hefasolon or hydrelta or hydrocortancyl or hydrocortidelt or hydradeltalone or hydradeltisone or inflanefran or insolone or keteocort h or key-pred or key-pred sp or lenisolone or leocortol or liquipred or mediasolone or meprisolon? or metacortalon? or metacortelone or meti derm or metiderm or meticortelone or morlone or mydrapred or nisolon? or opredsone or panafcortelone or panafcortolone or panafort or paracortol pr phlogex or pre cortisyl or preconin or precortalon or precortancyl or precortisyl or pred ject 50 or predacort 50 or predaject 50 or predalone 50 or predartrina or predartrine or predate 50 or predeltilone or predisole or predisyr or predne dome or prednecort or prednedome or prednelan or predni coelin or predni h tablinen or predni helvacort or prednicoelin or prednicort or prednicortelone or prednifor drops or predniment or prednorsolon? or predorgasolon? or prenilone or preventan or prezolon or rubycort or serilone or solonda or solupren? or spiricort or spolotane or supercorti#ol or taracortelone or walesolone or wysolone).mp. (23)

110 (methylprednisolone * or methylprednisolona* or methylprednisolonum* or metipred or urbason or medrol or x4w7zr7023* or 83-43-2 or besonia or brn 23403300 or brn2340300 or a-methapred or artisone-wyeth or besonia or depo-medrol or dopomedrol ec 201-4764 pr ec2014764 or einecs 201-476-4 or einecs201-476-4 or esametone or firmacort or hsdb3127 or hsdb3127 or lemod or medesone or medixon or medlone 21 or medrate or medrol or medrone or mesopren or metastab or methyleneprednisolone* or metilbetasone or metrisone or metrocort or metysolon or moderin or nirypan or noretona or nsc 19987 or nsc19987 or predni n tablinen or prednol-l or promacortine or reactenol or sieropresol or solomet or summicort or suprametil or u 7532 or u7532 or "u 67 590a" or urbason or urbasone or wyacort or adlone 40 or adlone 80 or beta methylprednisolone or medalone 80 or depmedalone or deproject 80 or depopred or esametone or firmacort or med-jec-40 or medixon or mednin or medralone or mepredprednisolone or meprelon or mesopren or methacort 40 or methacort 80 or methyxlcotol or methylcotolone or methylpred dp or methylsterolone or metidrol or metycortin or metypred or metypresol or neomedrone or solu decortin or urbason).mp. (1215)

111 (prednison or prednisone or prednisona or prednisonum or dehydrocortisone or delta cortisone or rectodelt or sterapred or ultracorten or winpred or apo prednisone or cortan or cartancyl or panafcort or cutason or decortin or dacortin or decortisyl or deltasone or encortone or encorton or enkorton or enkortolon or kortancyl or liquid pred or meticorten or orasone or panasol or predni tablinen or prednidib or predniment or pronisone or vb0r961hzt* or 53-03-2 or adasone or al3-52939 or ancortone or bicortone or cartancyl or ccris 2646 or ccrs2646 or colisone or cortan or cortidelt or cotone or dacorten or dacortin or decortancyl or dekortin or dellacort or delta cortelan or delta cortisone or delta dome or deltacortene or deltacortisone or deltacortone or

deltasone or deltison or deltisona or deltra or di adreson or diadreson or econosone or einecs 200-160-3 or einecs200-160-3 or fernisone or fiasone or hostacortin or hsdb 3168 or hsdb3168 or incocortyl or juvason or lisacort or lodotra or lodtra).mp. (10119)

112 (me-korti or metacortandracin or metacorten or nci c04897 or ncic04897 or nisona or nizon or novoprednisone or nsc 10023 or nsc10023 or nurison or orasone or panafcort or paracort or prarmenison or pehacort or predeltin or prednicen-m or prednicorm or prednicort or prednicot or prednilonga or prednitone or prednizon or prednovister or presone or pronison or rayos or rectodelt or retrocortine or servisone or sterapred or supercortio or u 6020 or u6020 or ultracorten or ultracortene or winpred or wojtab or zenadrid).mp. (19)

113 cortisone?.mp. (569)

114 corticosteroid*.mp. (23083)

115 corticoid*.mp. (556)

116 glucocorticoid*.mp. (8890)

117 corticosterone*.mp. (153)

118 hydrocortisone*.mp. (9702)

119 dexamethasone*.mp. (12333)

120 beclomethasone*.mp. (2295)

121 (triamcinolone* or 1zk20vi6ty* or 124-94-7 or aristocort or volon or fluoxyprednisolon* or tiamcinolonum or triamcinolonum* or triamcinolona* or brn 2341955 or brn2341955 or cl 19823 or cl19823 or einecs 204-718-7 or einecs204-718-7 or hsdb 3194 or hsdb3194 or nsc 13397 or nsc13397).mp. (3241)

122 (steroid? adj10 (therap* or treat* or drug? or inject* or intramuscular* or intra muscular* or im or intravenous* or intra venous* or iv or intraarticular* or intra articular* or ia or administrat* or oral or orally or parenteral*)).mp. (14182)

123 Janus Kinase Inhibitors/ (50)

124 ((jak or jak1 or jak2 or jak3 or janus kinase) adj10 inhibitor*).mp. (1538)

125 (janus adj4 kinase adj10 inhibitor?).mp. (927)

126 exp Janus Kinases/ai [Antagonists & Inhibitors] (95)

127 (baricitinib* or 1187594-09-7 or isp4442i3y* or "incb 028050" or incb028050 or ly-3009104 or ly3009104).mp. (409)

128 (tofacitinib* or 477600-75-2 or 87la6fu830* or hsdb 8311 or hsdb8311 or cp-690 550 or cp 690550 or cp690550).mp. (825)

129 (filgotinib* or 1206161-97-8 or 3xvl385q0m* or "glpg 0634" or glpg0634).mp. (173)

130 (upadacitinib* or 1310726-60-3 or 4RA0KN46E0* or abt 494 or abt494).mp. (267)

131 (ruxolitinib* or rux or 941678-49-5 or 82s8x8xx8h* or hsdb 8259 or hsdb8259 or inc 424 or inc424 or incb424 or "incb 018424" or incb018424 or incb 18424 or incb18424 or r-ruxolitinib* or jakafi or jakavi).mp. (486)

132 (itacitinib or "incb 039110" or incb039110 or incb 39110 or incb39110 or 1334298-90-6 or 19j3781lpm*).mp. (34)

133 (ag490 or ag 490 or tyrphostin b42 or tyrphostin ag 490 or tyrphostin ag490 or 133550-30-8).mp. (1)

134 (fedratinib* or tg101348 or tg 101348 or sar302503 or sar 302503 or 936091-26-8 or 6l1xp550i6* or inrebic).mp. (29)

135 (ritlecitinib* or pf-06651600 or pf06651600 or pf 6651600 or pf6651600 or 2140301-97-7 or 132lf5wgh4* or 2192215-81-7 or eag4t1459k* or ritlecitinib malonate or ritlecitinib toslate or ritlecitinib tosylate or ritlecitinib propanedioate).mp. (24)

136 (peficitinib* or peficitinib hydrobromide).mp. (26)

137 or/52-136 (150442)

138 40 and 137 (12026)

139 51 or 138 (12238)

140 ae.fs. (128872)

141 (adverse adj3 (event* or effect* or reaction* or incident* or outcome*)).mp. (205801)

142 ((serious* or severe* or severit*) adj3 (adverse or saes or aes or adrs or sae or "ae" or adr)).mp. (34986)

143 (saes or aes or adrs or sae or "ae" or adr).ti,ot,ab. and adverse.mp. (21053)

144 (adverseevent* or adversedrugreaction* or adversereaction* or adverseincident* or adverseoutcome*).mp. (27)

145 side effect*.mp. (150434)

146 exp "Drug-Related Side Effects and Adverse Reactions"/ (3172)

147 Adverse Drug Reaction Reporting Systems/ (89)

148 exp Product Surveillance, Postmarketing/ (207)

149 Substance Withdrawal Syndrome/ (2005)

150 withdraw*.mp. (44162)

151 safety.mp. (251564)

152 exp Drug Hypersensitivity/ (983)

153 (hypersensitiv* or hyper sensitiv*).mp. (10145)

154 Anaphylaxis/ (183)

155 anaphyla*.mp. (2258)

156 (toxic* adj6 (drug* or medication*)).mp. (8807)

157 intoxication*.mp. (3388)

158 Abnormalities, Drug-Induced/ (45)

159 Teratogens/ (9)

160 teratogen*.mp. (374)

161 ((drug or chemical*) adj1 induced).mp. (7968)

162 exp substance related disorders/ (15176)

163 (treatment adj3 emergent).mp. (8208)

164 exp Mortality/ (13372)

165 exp Death, Sudden/ (859)

166 exp Death/ (2028)

167 (fatal or fatalit*).mp. (10324)

168 ((mortality or death?) adj4 (rate? or ratio or caus* or risk or sudden or number* or amount* or count*)).mp. (60394)

169 mortality.mp. (83073)

170 exp *Infection/ (14338)

171 infection rate?.mp. (4145)

172 ((serious* or severe* or severit*) adj8 (infect* or bacter?emi* or septic?emi* or sepsis or fung?emi* or fungal infect* or fungal disease* or mycoses or mycosis or mycotic)).mp. (11905)

173 exp Opportunistic Infections/ (1005)

174 ((opportunistic* or opportune) adj6 (infect* or bacter?emi* or septic?emi* or bacterial infect* or bacterial diseas* or viral infect* or virus diseas* or virus infect* or microbial infect* or mycos#s or fung* infect* or mycotic diseas* or mycos#s infect* or fung?emi*)).mp. (2625)

175 ((overall or rate or rates or risk?) adj8 (infect* or bacter?emi* or septic?emi* or sepsis or bacterial infect* or bacterial diseas* or bacterious infect* or viral infect* or virus diseas* or virus infect* or microbial infect* or mycos#s or fung* infect* or mycotic diseas* or mycos#s infect* or fung?emi*)).mp. (27132)

176 infect*.mp. (137368)

177 exp Tuberculosis/ (2387)

178 (tuberculosis or tuberculous).mp. (7367)

179 exp Cytomegalovirus Infections/ (777)

180 ((cytomegalovirus or cytomegaly virus or cytomegaloviral or cmv) adj (infection? or disease?)).mp. (2019)

181 (cytomegalic inclusion adj3 disease?).mp. (4)

182 cytomegaloinfection?.mp. (0)

183 congenital cytomegalic inclusion.mp. (0)

184 (cmv and (virus or viral or infect*)).mp. (1614)

185 exp Mycobacterium Infections, Nontuberculous/ (153)

186 exp Nontuberculous Mycobacteria/ (62)

187 ((atypical or nontuberculous* or non-tuberculous*) adj3 (mycobacterios* or mycobacter*)).mp. (205)

188 (mycobacter* adj3 intracellulare adj3 infection*).mp. (125)

189 exp Mycoses/ (2785)

190 (mycosis or mycoses or mycotic or fung*).mp. (5837)

191 (aspergillos#s or (aspergill* adj3 infect*)).mp. (664)

192 (candidias#s* or candidemi* or (candid* adj3 infect*)).mp. (2778)

193 pneumocyst*.mp. (723)

194 exp Hepatitis B/ (2724)

195 Hepatitis B virus/ (776)

196 hepatitis b.mp. (9349)

197 (hepatitis adj1 (type b or injection or serum)).mp. (523)

198 (hepatitis adj3 "b").ti,ot,ab. (8799)

199 hbv.ti,ot,ab. (4611)

200 exp Infection/ (24558)

201 (opportunistic or serious* or severe* or severit*).mp. (235906)

202 200 and 201 (5313)

203 exp Herpes Zoster/ (563)

204 ((herpes or varicella*) adj1 zoster*).mp. (2376)

205 (zoster or shingles or varicellovirus infect*).mp. (2443)

206 exp Herpes Simplex/ (976)

207 (herpes adj3 (simplex or vulgaris or infection*)).mp. (2126)

208 herpes.mp. (4620)

209 hsv.ti,ot,ab. and (infect* or herpes).mp. (781)

210 203 or 204 or 205 or 206 or 207 or 208 or 209 (4962)

211 Virus Activation/ (85)

212 (reactivat* or re-activat*).mp. (1660)

213 (activat* adj3 (virus or viral* or vir?emi*)).mp. (259)

214 211 or 212 or 213 (1846)

215 210 and 214 (245)

216 C-Reactive Protein/ (4676)

217 9007-41-4.mp. (1)

218 ((c-reactive or creactive or c reaction or creaction) adj2 protein).mp. (18672)

219 crp.mp. (15495)

220 Blood Sedimentation/ (507)

221 ((erythrocyte or blood) adj3 sedimentation*).mp. (2798)

222 esr.ti,ot,ab. and sedimentation.mp. (1248)

223 (DAS28-ESR or DAS-28-ESR or DAS28ESR).mp. (605)

224 ((DAS28 or "DAS 28" or disease activity score or das or disease activity) and ESR).mp. (1537)

225 esr.ti,ot,ab. (3034)

226 Procalcitonin/ (22)

227 Calcitonin/ (645)

228 (procalcitonin or pro-calcitonin or pct).mp. (1707)

229 (calcitonin adj3 precursor?).mp. (4)

230 exp Leukocyte Count/ (5342)

231 ((leukocyte* or whithe blood cell or white cell or white blood or wbc) adj3 count*).mp. (8905)

232 wbc.ti,ot,ab. (3986)

233 exp Neoplasms/ (79793)

234 exp Hematologic Neoplasms/ (481)

235 exp histiocytic disorders, malignant/ or exp leukemia/ or exp lymphoma/ (7510)

236 exp Multiple Myeloma/ (1618)

237 (lymphom* or leuk?em* or myeloma*).mp. (29835)

238 ((h?ematolog* or blood disease) adj6 (malignan* or neoplas*)).mp. (3004)

239 (cancer or cancers).mp. (176613)

240 neoplas*.mp. (85051)

241 (tumor? or tumour?).mp. (81133)

242 (carcinom* or karzinom*).mp. (44961)

243 hodgkin*.mp. (5949)

244 (non-hodgkin* or nonhodgkin*).mp. (3882)

245 (mesotheliom* or mesoteliom*).mp. (842)

246 malignan*.mp. (29048)

247 adenocarcinom*.mp. (11782)

248 metasta*.mp. (46561)

249 sarcom*.mp. (2964)

250 adenosarcom*.mp. (8)

251 osteosarcom*.mp. (616)

252 reticulosarcom*.mp. (5)

253 lymphosarcom*.mp. (27)

254 (blastom* or neuroblastom*).mp. (823)

255 (gliom* or glioblastom*).mp. (3695)

256 melanom*.mp. (6084)

257 ((non melanom* or nonmelanom*) adj3 skin adj3 (cancer or cancers or neoplas* or tumo?r* or carcino*)).mp. (634)

258 (solid adj6 (cancer or cancers or neoplas* or tumo?r* or carcino* or malignan*)).mp. (8239)

259 exp Diverticulitis/ (91)

260 diverticulitis*.mp. (568)

261 Intestinal Perforation/ (140)

262 ((digestive system or gastrointestinal* or gastro intestinal* or intestinal*) adj3 perforation*).mp. (805)

263 exp Liver/ (3141)

264 Liver Function Tests/ (1160)

265 ((liver or hepat*) adj1 function?).mp. (10870)

266 (lft or lfts).ti,ot,ab. (570)

267 (hypertransaminasemi* or hyper transaminasemi*).mp. (1157)

268 uln.ti,ot,ab. (2730)

269 exp Drug-Induced Liver Injury/ (10)

270 (dili or drug induc* liver injur*).mp. (484)

271 Alanine Transaminase/ (1605)

272 exp Aspartate Aminotransferases/ (1021)

273 (transaminas* or alanin* aminotransaminas* or alt or aspartat* aminotransaminas* or ast or serum glutam* pyruv* transaminas* or sgpt or serum glutam* oxalacet* transaminas* or sgot or liver enzyme* or hepat* enzyme*).mp. (17316)

274 transaminitis*.mp. (164)

275 (hepat*tox* or ((liver or hepat*) adj3 toxic*)).mp. (3522)

276 exp Triglycerides/ (6344)

277 exp Lipoproteins/bl [Blood] (1639)

278 exp "lipoprotein(a)"/ or exp lipoproteins, hdl/ or exp lipoproteins, ldl/ (7556)

279 exp Cholesterol/ (10207)

280 ((lipid? or cholesterol* or cholesterin* or triglycerid* or lipoprotein*) adj3 (level? or blood or plasma or serum)).mp. (37529)

281 (ldl or low density lipoprotein*).mp. (27032)

282 (hdl or high density lipoprotein*).mp. (22341)

283 (hdl2 or "hdl(2)" or "hdl 2").mp. (425)

284 (hdl3 or "hdl(3)" or "hdl 3").mp. (342)

285 (lipoprotein a or lipoproteina).mp. (22259)

286 alpha lipoprotein.mp. (13)

287 (ldl1 or "ldl(1)" or "ldl 1").mp. (48)

288 (ldl2 or "ldl(2)" or "ldl 2").mp. (65)

289 exp Lipids/bl [Blood] (3506)

290 exp Hyperlipidemias/ (6430)

291 (hyperlipid?emi* or lipid?emi*).mp. (6732)

292 exp Diabetes Mellitus/ (31627)

293 exp Obesity/ (14018)

294 (metabolic adj3 (effect? or alterat* or chang* or risk? or transition* or transformat* or parameter? or marker? or biomarker?)).mp. (14742)

295 (diabetes* or diabetic*).mp. (99239)

296 Glycated Hemoglobin A/ (5742)

297 (hba1c or hbaic or "hb a1c" or "hb a 1c" or "hb aic" or "hb a ic" or "hba 1c" or "hba ic").ti,ot,ab. (19312)

298 ("h?emoglobin a1c" or "h?emoglobin a 1c" or h?emoglobin aic or "h?emoglobin a ic").mp. (8888)

299 body mass index/ (10319)

300 (body mass index or bmi).mp. (64171)

301 Venous Thromboembolism/ (640)

302 ((venous* or venos* or vein*) adj1 (thrombos#s* or thrombotic*or thromboemboli* or thrombo emboli* or thrombus or thrombi or microthromb*)).mp. (8802)

303 (dvt or dvts or vte or vtes).ti,ot,ab. (4121)

304 exp Pulmonary Embolism/ (968)

305 ((lung* or pulmonary) adj3 (embol* or thromboemboli* or thromb* emboli* or microemboli* or clot* or thrombus or thrombi or microthromb* or infarct*)).mp. (6183)

306 (pe or pes).ti,ot,ab. and (lung* or pulmo* or pneumo* or embol* or thromboembol* or thrombot* or thrombus or thrombi or microembol).mp. (1709)

307 exp Anemia/ (5209)

308 an?emi*.mp. (21980)

309 exp Thrombocytopenia/ (1259)

310 (thrombocytopeni* or thrombopeni*).mp. (11302)

311 exp Neutropenia/ (1712)

312 neutropeni*.mp. (14658)

313 exp Lymphopenia/ (87)

314 (lymphocytopeni* or lymphopeni*).mp. (1515)

315 Macrophage Activation Syndrome/ (7)

316 mas.ti,ot,ab. (2036)

317 (macrophage activat* adj3 syndrome?).mp. (55)

318 315 or 316 or 317 (2071)

319 (Rheumatoid Arthritis adj3 Systemic adj3 Juvenile).mp. (18)

320 Arthritis, Juvenile/ (299)

321 ((arthrit* or artrit*) adj6 juvenile).mp. (1001)

322 JIA.ti,ot,ab. (627)

323 320 or 321 or 322 (1123)

324 (systemic or polyart* or poly art* or oligoart* or oligo art*).mp. (52595)

325 323 and 324 (515)

326 ((systemic adj6 jia) or sjia or s-jia).ti,ot,ab. (167)

327 319 or 325 or 326 (526)

328 318 and 327 (32)

329 exp Heart Failure/ (9513)

330 (congestive adj2 (heart or cardiac) adj2 failure*).mp. (6600)

331 (heart failure* or cardiac failure* or myocard* failure*).mp. (31749)

332 ((heart or cardiac or cordis or cardio* or myocard*) adj1 decompensatio*).mp. (144)

333 (chf or ccf or hf).ti,ot,ab. and (heart or cardiac or cardio* or myocard*).mp. (10356)

334 exp Myocardial Ischemia/ (28078)

335 ((heart or cardio* or cardiac or myocardial) adj1 infarct*).mp. (36483)

336 exp Myocardial Infarction/ (10847)

337 cardiovascular* stroke?.mp. (29)

338 (MACE or MACEs).ti,ot,ab. (3217)

339 major adverse card* event*.mp. (4170)

340 acute coronary syndrome*.mp. (7735)

341 (heart attack or heart attacks).mp. (1232)

342 (mi or mis).ti,ot,ab. and (myocard* or cardio* or coronary or heart or cardiac or mace or maces).mp. (6685)

343 (angina or anginas or stenocardia?).mp. (14212)

344 ((cerebrovascular* or cerebr* vascular*) adj1 (accident? or failure? or injury or injuries or insult? or insufficienc* or apoplex*)).mp. (15128)

345 (stroke or strokes or apoplexi* or apoplexy*).mp. (59567)

346 (cva or cvas).ti,ot,ab. (594)

347 (brain insult* or brain isch?emic attack? or cerebral insult* or brain vascular accident? or isch?emic cerebr* attack? or isch?emic seizure? or cerebr* vascular accident?).mp. (210)

348 exp Myocardial Revascularization/ (9219)

349 (revascularizat* or re-vascularizat* or revascularisat* or re-vascularisat*).mp. (13109)

350 Peripheral Arterial Disease/ (1066)

351 Arterial Occlusive Diseases/ (863)

352 (peripher* adj2 (arter* or vascular*) adj2 disease*).mp. (5834)

353 ((pad or pvd) adj6 (peripher* or arter* or vascular*)).ti,ot,ab. (1342)

354 exp Kidney Function Tests/ (4263)

355 ((kidney or renal) adj1 function*).mp. (20279)

356 Creatinine/bl [Blood] (132)

357 (creatinin* and (serum or plasma or blood)).mp. (22626)

358 (creatinin* adj15 (level? or concentration? or ratio or clearance or value?)).mp. (18351)

359 (creatinin* adj15 (decreas* or increas* or elevat* or lower* or low or high or higher)).ti,ot,ab. (8238)

360 AYI8EX34EU.af. (0)

361 (estimated glomerul* filtrat* rate? or estimated glomerulofiltration rate?).mp. (5087)

362 (egfr or egfrs or e-gfr or e-gfrs or estimat* gfr or estimat* gfrs or estimat*gfr or estimat*gfrs).mp. (9842)

363 Glomerular Filtration Rate/ (2787)

364 exp Renal Insufficiency/ (9129)

365 ((renal or kidney) adj1 (insufficien* or failure ?or injur*)).mp. (5694)

366 ((cardio renal or cardiorenal or renocardiac or reno cardiac) adj1 syndrom*).mp. (132)

367 exp Hemoglobins/ (3695)

368 h?emoglobin*.mp. (39881)

369 exp Osteoporosis/ (4109)

370 (osteoporos* or osteoporotic*).mp. (11834)

371 exp Leukoencephalopathies/ (371)

372 (leukoencephalopath* or leuko encephalopath*).mp. (354)

373 exp Demyelinating Diseases/ (3812)

374 ((demyelinat* or demyeliniz*) adj3 (disease* or disorder* or cns or central nervous system* or encephalopath* or autoimmune disease*)).mp. (640)

375 demyelinat*.mp. (1318)

376 demyeliniz*.mp. (13)

377 demyelinis*.mp. (9)

378 exp Multiple Sclerosis/ (3525)

379 multiple scleros#s.mp. (10869)

380 exp Optic Neuritis/ (167)

381 (optic* adj3 (neuritis* or neuritides*)).mp. (495)

382 (optic* adj3 (inflammat* adj3 neuropath*)).mp. (6)

383 exp Myelitis, Transverse/ (43)

384 (transverse adj3 (myelitis* or myelitides* or myelopath* syndrome*)).mp. (67)

385 (necrotiz* adj3 (myelitis* or myelitides*)).mp. (0)

386 (demyelinat* adj3 (myelitis* or myelitides*)).mp. (2)

387 ((postinfect* or infect* or postvaccin* or vaccin*) adj10 (myelitis or myelitides)).mp. (21)

388 exp Peripheral Nervous System Diseases/ (5421)

389 (peripheral adj2 (nervous system or nerve) adj2 (disease* or disorder* or neuropath* or mononeuropath* or polyneuropath* or polyradiculaneuropath*)).mp. (1199)

390 exp Guillain-Barre Syndrome/ (48)

391 (guillain barre or fisher syndrome*).mp. (398)

392 ((landry or landry guillain barre) adj3 (syndrom* or paralys*)).mp. (0)

393 (miller fisher adj3 (syndrom* or polyneuritis* or variant?)).mp. (1)

394 ((polyradiculoneuropath* or polyneuropath* or neuronitis* or polyneuritis*) adj15 (inflammat* or postinfect* or infect* or postvaccin* or vaccin*)).mp. (378)

395 Polyradiculoneuropathy, Chronic Inflammatory Demyelinating/ (63)

396 ((polyradiculoneuropath* or polyneuropath* or polyradiculopath*) adj6 chronic* adj3 inflammat*).mp. (303)

397 cidp.ti,ot,ab. (256)

398 exp Polyradiculoneuropathy/ (207)

399 polyradiculoneuropath*.mp. (249)

400 ((demyelinat* or inflammat*) adj1 (neuropath* or polyneuropath* or polyradiculoneuropath*)).mp. (427)

401 (multifocal motor neuropath* adj6 conduction block*).mp. (8)

402 (motor adj3 neuropath* adj6 conduction block*).mp. (12)

403 exp Motor Neuron Disease/ (712)

404 (motor adj3 neuropath*).mp. (538)

405 exp Mononeuropathies/ (1097)

406 (mononeuropath* or mononeuritis* or mononeuritides*).mp. (76)

407 exp Polyneuropathies/ (499)

408 (polyneuropath* adj6 axonal adj3 (sensor* motor or sensorimotor* or sensorymotor)).mp. (3)

409 polyneuropath*.mp. (1508)

410 exp Vaccination/ (2544)

411 exp Vaccines/ (12922)

412 vaccinat*.mp. (16843)

413 (active adj2 immuni#at*).mp. (351)

414 vaccine*.mp. (24496)

415 exp Pregnancy/ (21326)

416 exp Pregnancy Complications/ (11338)

417 pregnan*.mp. (68002)

418 tolerabilit*.mp. (68763)

419 safe.ti,ot,ab. (68592)

420 ((injurious* or undesirabl*) adj3 (effect? or reaction* or event? or outcome? or incident?)).ti,ot,ab. (1226)

421 exp Postoperative Complications/ (39488)

422 exp Intraoperative Complications/ (4298)

423 complication*.mp. (147097)

424 co.fs. (54137)

425 risk.ti,ot,ab. (222306)

426 de.fs. (120976)

427 exp risk/ (38514)

428 140 or 141 or 142 or 143 or 144 or 145 or 146 or 147 or 148 or 149 or 150 or 151 or 152 or 153 or 154 or 155 or 156 or 157 or 158 or 159 or 160 or 161 or 162 or 163 or 164 or 165 or 166 or 167 or 168 or 169 or 170 or 171 or 172 or 173 or 174 or 175 or 176 or 177 or 178 or 179 or 180 or 181 or 182 or 183 or 184 or 185 or 186 or 187 or 188 or 189 or 190 or 191 or 192 or 193 or 194 or 195 or 196 or 197 or 198 or 199 or 202 or 215 or 216 or 217 or 218 or 219 or 220 or 221 or 222 or 223 or 224 or 225 or 226 or 227 or 228 or 229 or 230 or 231 or 232 or 233 or 234 or 235 or 236 or 237 or 238 or 239 or 240 or 241 or 242 or 243 or 244 or 245 or 246 or 247 or 248 or 249 or 250 or 251 or 252 or 253 or 254 or 255 or 256 or 257 or 258 or 259 or 260 or 261 or 262 or 263 or 264 or 265 or 266 or 267 or 268 or 269 or 270 or 271 or 272 or 273 or 274 or 275 or 276 or 277 or 278 or 279 or 280 or 281 or 282 or 283 or 284 or 285 or 286 or 287 or 288 or 289 or 290 or 291 or 292 or 293 or 294 or 295 or 296 or 297 or 298 or 299 or 300 or 301 or 302 or 303 or 304 or 305 or 306 or 307 or 308 or 309 or 310 or 311 or 312 or 313 or 314 or 328 or 329 or 330 or 331 or 332 or 333 or 334 or 335 or 336 or 337 or 338 or 339 or 340 or 341 or 342 or 343 or 344 or 345 or 346 or 347 or 348 or 349 or 350 or 351 or 352 or 353 or 354 or 355 or 356 or 357 or 358 or 359 or 360 or 361 or 362 or 363 or 364 or 365 or 366 or 367 or 368 or 369 or 370 or 371 or 372 or 373 or 374 or 375 or 376 or 377 or 378 or 379 or 380 or 381 or 382 or 383 or 384 or 385 or 386 or 387 or 388 or 389 or 390 or 391 or 392 or 393 or 394 or 395 or 396 or 397 or 398 or 399 or 400 or 401 or 402 or 403 or 404 or 405 or 406 or 407 or 408 or 409 or 410 or 411 or 412 or 413 or 414 or 415 or 416 or 417 or 418 or 419 or 420 or 421 or 422 or 423 or 424 or 425 or 426 or 427 (1196351)

429 139 and 428 (9994)

430 ((eular or (european adj4 rheumat*)) adj8 (meeting* or conference* or congress*) adj8 annual).af. (704)

431 ((acr or (american college adj4 rheumat*)) adj8 (meeting* or conference* or congress*) adj8 annual).af. (806)

432 430 or 431 (1506)

433 429 and 432 (715)

434 limit 433 to yr="2019 - 2020" (0)

435 limit 429 to yr="2012 - 2020" (6051)

436 limit 435 to english (4522)

437 exp Registries/ (1029)

438 (register or registry or registries or registered or registration*).mp. (101650)

439 Cohort Studies/ (7646)

440 (cohort* adj6 (study* or studies* or analy*)).mp. (43916)

441 (cohortstudy* or cohortstudies* or cohortanaly*).mp. (6)

442 exp case-control studies/ (13933)

443 (case control adj3 (study or match*)).mp. (7196)

444 case referent.mp. (12)

445 case control.mp. (12677)

446 Prospective Studies/ (93051)

447 exp Longitudinal studies/ (142382)

448 Retrospective Studies/ (8831)

449 (longitudinal or prospective or retrospective).mp. (287054)

450 (study or trial or studies or trials or analys#s or design).mp. (1510562)

451 449 and 450 (284910)

452 Follow-Up Studies/ (60349)

453 (follow up or followup).mp. (267009)

454 452 or 453 (267009)

455 (long term or longterm).mp. (103284)

456 (extend* or extension?).mp. (49928)

457 (continuing or continue* or continuat*).mp. (73980)

458 455 or 456 or 457 (204772)

459 454 and 458 (62283)

460 ((long term or longterm) adj3 data).ti,ot,ab. (3554)

461 459 or 460 (63887)

462 437 or 438 or 439 or 440 or 441 or 442 or 443 or 444 or 445 or 446 or 447 or 448 or 451 or 461 (457392)

463 436 and 462 (1711)

464 randomized controlled trial.pt. (507674)

465 exp randomized controlled trial/ (131)

466 exp Randomized Controlled Trials as Topic/ (8017)

467 randomi*.mp. (1025624)

468 randomly.ti,ot,ab. (259754)

469 trial.ti. (336020)

470 trial.ab. and random*.mp. (412280)

471 (rct or rcts).ti,ot,ab. (34111)

472 Random Allocation/ (20645)

473 controlled clinical trial/ (36)

474 Single-Blind Method/ (21220)

475 Double-Blind Method/ (139303)

476 ((singl* or doubl* or tripl* or trebl*) adj3 (blind* or mask*)).mp. (390906)

477 (singl*blind* or doubl*blind* or trebl*blind* or tripl*blind*).ti,ot,ab. (2483)

478 (singl*mask* or doubl*mask* or tripl*mask* or trebl*mask*).ti,ot,ab. (72)

479 (controlled adj8 (study or design or trial)).ti,ot,ab. (444798)

480 (parallel adj1 group*).ti,ot,ab. (43843)

481 exp placebo effect/ (1548)

482 Placebos/ (24107)

483 placebo*.mp. (331869)

484 (allocated or allocation or assigned).ti,ot,ab. and random*.mp. (230516)

485 groups.ti,ot,ab. (489767)

486 dt.fs. (200514)

487 464 or 465 or 466 or 467 or 468 or 469 or 470 or 471 or 472 or 473 or 474 or 475 or 476 or 477 or 478 or 479 or 480 or 481 or 482 or 483 or 484 or 485 or 486 (1410768)

488 458 and 487 (181428)

489 436 and 488 (1378)

490 463 or 489 (2483)

491 review.pt. (3088)

492 conference abstract.pt. (16804)

493 conference review.pt. (173)

494 conference paper.pt. (134)

495 congress.pt. (44)

496 congresses.pt. (5)

497 editorial.pt. (462)

498 letter.pt. (7334)

499 491 or 492 or 493 or 494 or 495 or 496 or 497 or 498 (28023)

500 490 not 499 (2270)

501 (journal conference abstract or journal conference paper or "journal conference review" or journal editorial or journal letter).pt. (178394)

502 500 not 501 (1126)

1.3.4 Safety: other studied diseases

1.3.4.1 Medline

1 exp Coronavirus/ (45349)

2 exp Coronavirus Infections/ (49593)

3 (coronavirus* or corona virus* or OC43 or NL63 or 229E or HKU1 or HCoV* or ncov* or covid* or sars-cov* or sarscov* or Sars-coronavirus* or Severe Acute Respiratory Syndrome Coronavirus*).mp. (103896)

4 (or/1-3) and ((20191* or 202*).dp. or 20190101:20301231.(ep).) (90904)

5 4 not (SARS or SARS-CoV or MERS or MERS-CoV or Middle East respiratory syndrome or camel* or dromedar* or equine or coronary or coronal or covidence* or covidien or influenza virus or HIV or bovine or calves or TGEV or feline or porcine or BCoV or PED or PEDV or PDCoV or FIPV or FCoV or SADS-CoV or canine or CCov or zoonotic or avian influenza or H1N1 or H5N1 or H5N6 or IBV or murine corona*).mp. (55424)

6 ((pneumonia or covid* or coronavirus* or corona virus* or ncov* or 2019-ncov or sars*).mp. or exp pneumonia/) and Wuhan.mp. (4026)

7 (2019-ncov* or 2019-n-cov*or 2019ncov* or 2019n-cov* or ncov19 or ncov-19 or 2019-novel CoV*or ncov2019* or n-cov2019* or ncov-2019* or n-cov-2019* or novel cov-2019* or novel-cov-19* or ncov19* or n-cov19* or ncov-19* or n-cov-19* or sars-cov2* or sars-cov-2* or sarscov2* or sarscov-2* or sars-covid-2* or sars-2-cov* or Sars-coronavirus2* or Sars-coronavirus-2* or SARS-like coronavirus* or coronavirus-19 or covid19 or covid-19 or covid 2019 or ((novel or new or newly or nouveau or "19?" or "2019*" or wuhan) adj2 (CoV on nCoV or n-cov or covid or coronavirus* or corona virus or betacoronavirus* or Pandemi*2)) or ((covid or covid19 or covid-19) and pandemic*2) or (coronavirus* and pneumonia)).mp. (90477)

8 (severe acute respiratory syndrome coronavirus 2* or severe acute respiratory syndrome cov* 2* or severe acute respiratory syndrome coronavirus 2019* or severe acute respiratory syndrome cov* 2019* or severe acute respiratory syndrome coronavirus 19* or severe acute respiratory syndrome cov* 19*).mp. [mp=title, abstract, original title, name of substance word, subject heading word, floating sub-heading word, keyword heading word, organism supplementary concept word, protocol supplementary concept word, rare disease supplementary concept word, unique identifier, synonyms] (38437)

9 COVID-19.rx,px,ox. or severe acute respiratory syndrome coronavirus 2.os. (39791)

10 ("32240632" or "32236488" or "32268021" or "32267941" or "32169616" or "32267649" or "32267499" or "32267344" or "32248853" or "32246156" or "32243118" or "32240583" or "32237674" or "32234725" or "32173381" or "32227595" or "32185863" or "32221979" or "32213260" or "32205350" or "32202721" or "32197097" or "32196032" or "32188729" or "32176889" or "32088947" or "32277065" or "32273472" or "32273444" or "32145185" or "31917786" or "32267384" or "32265186" or "32253187" or "32265567" or "32231286" or "32105468" or "32179788" or "32152361" or "32152148" or "32140676" or "32053580" or "32029604" or "32127714" or "32047315" or "32020111" or "32267950" or "32249952" or "32172715").ui. [Articles not captured by this search when created in April 2020, pending further indexing by NLM] (49)

11 or/6-10 (90608)

12 5 or 11 (91906)

13 12 and 20191201:20301231.(dt). (89648)

14 exp Lung Diseases, Interstitial/ (56498)

15 exp Scleroderma, Systemic/ (20851)

16 14 and 15 (1018)

17 (((systemic adj3 (sclerosis* or sleroderma*)) or ssc) and (interstitial adj3 (lung disease* or pneumoni*))).ti,ab,kf. (1481)

18 (ssc-ild* or sscild*).ti,ab,kf. (313)

19 Polymyalgia Rheumatica/ (2541)

20 pmr.ti,ab,kf. and rheum*.mp. [mp=title, abstract, original title, name of substance word, subject heading word, floating sub-heading word, keyword heading word, organism supplementary concept word, protocol supplementary concept word, rare disease supplementary concept word, unique identifier, synonyms] (923)

21 ((rheumat* or arteritic*) adj1 (polymyalgia* or myalgia*)).ti,ab,kf. (2786)

22 ((pseudopolyarthrit* or pseudo polyarthrit* or inflammat* rheumat* or rheumat* inflammat*) adj1 rhizomelic).ti,ab,kf. (86)

23 (forestier-certonciny adj1 syndrom*).ti,ab,kf. (2)

24 (remitting adj3 seronegative adj3 synovitis adj6 (edem* or oedem*)).ti,ab,kw. (190)

25 rs3pe*.ti,ab,kw. (192)

26 exp Synovitis/ (8401)

27 Edema/ (40385)

28 26 and 27 (374)

29 (remitting or seronegativ* or symmetric*).ti,ab,kf. (104376)

30 28 and 29 (159)

31 exp Lupus Erythematosus, Systemic/ (60234)

32 (lupus erythemato* adj3 (systemic* or disseminat* or visceral*)).ti,ab,kf. (55011)

33 (sle or 's l e').ti,ab,kf. (34923)

34 (lupus or autoimmun* or auto immun* or autoantibod* or antibod* or inflamm* or immun* or rheumat* or arthrit* or nephritis or nephrol* or cutan* or dermatol* or venerol* or neuromyotoni* or neuroscience? or neurolog* or patient? or cell debris).mp,jw. (11440078)

35 33 and 34 (34081)

36 (libman sacks adj1 (disease* or endocarditis*)).ti,ab,kf. (259)

37 exp Myositis/ (19973)

38 (myositis or neuromyositis or polymyositis or dermatomyositis).ti,ab,kf. (20548)

39 (inflammat* adj1 (myopath* or muscle? or myosit*)).ti,ab,kf. (4556)

40 (inflammat* adj3 idiopath* adj3 (myopath* or myosit*)).ti,ab,kf. (1652)

41 ((infect* or focal or proliferativ*) adj3 (myopath* or myosit*)).ti,ab,kw. (707)

42 exp Amyloidosis/ (24905)

43 (amyloidos#s or amyloidoma* or beta fibrillos#s or paraamyloidos#s).ti,ab,kf. (24765)

44 Polychondritis, Relapsing/ (1398)

45 (polychondrit* adj3 (relapsing* or atrophic*)).ti,ab,kf. (1476)

46 (chondromalac* adj3 systemic*).ti,ab,kf. (3)

47 (von meyenburg adj3 disease*).ti,ab,kf. (3)

48 (periodic fever adj1 familial adj1 autosomal dominant).rx. (98)

49 ((tnf receptor or tnfr1 or tnfr) adj3 associated periodic syndrom*).ti,ab,kf. (156)

50 (traps and (tumo?r necrosis factor receptor or tnf receptor)).ti,ab,kf. (313)

51 (tumo?r necrosis factor receptor adj3 associated periodic adj2 (syndrom* or fever)).ti,ab,kf. (294)

52 (autosomal dominant adj3 periodic fever).ti,ab,kf. (13)

53 (hibernian fever adj3 familial).ti,ab,kf. (16)

54 ((tnf receptor associated periodic or tnfr1 associated or tumo?r necrosis factor receptor associated or tnfr associated) adj3 (syndrom* or fever*)).ti,ab,kf. (397)

55 Cryopyrin-Associated Periodic Syndromes/ (466)

56 (cinca or cinca syndrom*).ti,ab,kf. (184)

57 (chronic infantile adj3 neurolog* cutan* adj3 (articul* syndrom* or arthropath*)).ti,ab,kf. (99)

58 (neonatal onset adj3 multisyst* inflamm* adj1 (diseas* or disorder*)).ti,ab,kf. (148)

59 nomid.ti,ab,kf. (128)

60 iomid.ti,ab,kf. (4)

61 cryopyrin associated periodic syndrom*.ti,ab,kf. (437)

62 Neuromyelitis Optica/ (3052)

63 (neuromyelit* adj1 optic*).ti,ab,kf. (4369)

64 (myelooptic* adj1 neuropath*).ti,ab,kf. (3)

65 ((nmo or nmosd) and (neuromyelit* or neuropath*)).ti,ab,kf. (2786)

66 ((devic or devics or devic's) adj2 (diseas* or syndrom* or neuromyelit* optic*)).ti,ab,kf. (457)

67 (myeloopticoneuropath* or myelooptic* neuropath* or neuroopticomyelit*).ti,ab,kf. (6)

68 exp Anti-Neutrophil Cytoplasmic Antibody-Associated Vasculitis/ (10622)

69 ((anca or antineutrophil cytoplasmic or anti-neutrophil cytoplasmic) adj6 vasculit*).ti,ab,kf. (4648)

70 (pauci immun* adj6 vasculit*).ti,ab,kf. (145)

71 exp Arthritis, Psoriatic/ (6317)

72 (psoria* adj2 (arthrit* or polyarthrit* or poly arthrit* or arthropath*)).ti,ab,kf. (10836)

73 (psoria* adj1 rheumat*).ti,ab,kf. (285)

74 (psoria* adj6 (inflammat* arthrit* or inflammat* polyarthrit* or inflammat* poly arthrit*)).ti,ab,kf. (285)

75 Spondylitis, Ankylosing/ (14854)

76 ((spondylitis* or spondylarthrit* or spndyloarthrit* or spine or vertebral) adj1 ankyl*).ti,ab,kf. (15807)

77 (rheumat* spondylit* or axial spondyl?arthrit* or bechterew* disease* or bechterews disease* or bechterew's disease* or marie struempell disease*).ti,ab,kf. (2145)

78 (axspa* or ax-spa*).ti,ab,kf. (811)

79 (axial adj3 spondyl?arthrit*).ti,ab,kf. (1639)

80 13 or 16 or 17 or 18 or 19 or 20 or 21 or 22 or 23 or 24 or 25 or 30 or 31 or 32 or 35 or 36 or 37 or 38 or 39 or 40 or 41 or 42 or 43 or 44 or 45 or 46 or 47 or 48 or 49 or 50 or 51 or 52 or 53 or 54 or 55 or 56 or 57 or 58 or 59 or 60 or 61 or 62 or 63 or 64 or 65 or 66 or 67 or 68 or 69 or 70 or 71 or 72 or 73 or 74 or 75 or 76 or 77 or 78 or 79 (275377)

81 (tocilizumab* or actemra* or atlizumab* or lusinex* or r1569 or r 1569 or roactemra* or mra or 375823 41 9 or i031v2h011* or rhpm-1 or rhpm1 or rg-1569 or rg1569 or msb-11456 or msb11456 or ro-4877533 or ro4877533).mp,rn. (12697)

82 (sarilumab* or kevzara* or regn 88 or regn88 or sar 153191 or sar153191 or 1189541 98 7 or nu90v55f8i*).mp,rn. (178)

83 (levilimab* or BCD-089 or bcd089 or 2035008 70 7 or P7UV3L2H80*).mp,rn. (0)

84 (olokizumab* or cdp 6038 or cdp6038 or 1007223 17 7 or pai71r1d2w*).mp,rn. (18)

85 (clazakizumab* or ald 518 or ald518 or bms 945429 or bms945429 or 1236278 28 6 or 4s38z8ra9o*).mp,rn. (29)

86 (sirukumab* or cnto 136 or cnto136 or pilvensia or 1194585 53 9 or 640443FU93*).mp,rn. (55)

87 (siltuximab* or sylvant or cnto 328 or cnto-328 or cnto328 or cllb8 or ccib8 monoclonal antibody or sylvant or t4h8fma7im* or 541502 14 1).mp,rn. (184)

88 (ziltivekimab* or zilti or "cor-001" or "2226654 05 1").mp,rn. (3)

89 (elsilimomab* or "b-e8" or 468715 71 1).mp,rn. (12)

90 81 or 82 or 83 or 84 or 85 or 86 or 87 or 88 or 89 (12985)

91 80 and 90 (1283)

92 Interleukin 1 Receptor Antagonist Protein/ (5239)

93 (anakinra* or kineret or ((interleukin 1 receptor or il1 receptor or il 1 receptor) adj1 antagonist protein*) or (recombinant interleukin 1 receptor adj1 (antagonist* or blocker* or blocking agent*)) or antril or il 1ra or il1ra or il 1 ra or ((urin* or febrile) adj2 (interleukin 1 inhibitor* or il1 inhibitor* or il 1 inhibitor*)) or "143090 92 0" or 9013duq28k*).mp,rn. (9660)

94 Infliximab/ (10429)

95 (infliximab* or ifx or avakine or flixabi or inflectra or inflecta or ixifi or remicade or remsima or revellex or renflexis or zessly or mab ca2 or monoclonal antibody ca2 or antibody ca2 monoclonal or ca2 monoclonal antibody or ct p13 or ctp13 or ct-p-13 gp-1111 or gp1111 or ta-650 or ta650 or abp 710 or abp710 or bow-015 or bow015 or 170277 31 3 or b72hh48flu* or sb2 or "pf 06438179" or pf 6438179 or pf06438179 or pf6438179 or hsdb 7850 or hsdb7850).mp,rn. (15957)

96 Etanercept/ (5917)

97 (etanercept* or benepali or embrel or enbrel or enia 11 or enia11 or erelzi or lifmior or opinercept or recombinant tumo?r necrosis factor receptor fc fusion protein or "tnr 001" or tnr001 or tnfr-fc or tnrfr:fc or rhu tnfr:fc or rhu-tnfr:fc or tnfr-immunoadhesin or tnt receptor fusion protein or tum?or necrosis factor receptor fc fusion protein or tunex or "185243 69 0" or op401g7ojc* or brenzys or tnf receptor fusion protein or tnfr fc or recombinant human tnf or recombinant human dimeric tnf receptor type ii igg fusion protein or chs-0214 or chs0214 or dwp-422 or dwp422 or enia-11 or enia11 or gp-2015 or gp2015 or gp2015c or gp-2015c or hd-203 or hd203 or lbec-0101 or lbec0101 or sb-4 or sb4).mp,rn. (9517)

98 Adalimumab/ (5507)

99 (adalimumab* or humira or abp 501 or abp501 or abtd2e7 or amjevita or amgevita or bax 2923 or bax2923 gp 2017 or gp2017 or halimatoz or hefiya or hulio or hyrimoz or ibi 303 or ibi303 or imraldi or m 923 or m923 or msb 11022 or msb11022 or ons3010 or ons 3010 or "pf 06410293" or pf 6410293 or pf06410293 or pf6410293 or d2e7 or truxeda or cyltezo or solymbic or fys6t7f842* or 331731 18 1 or lu 200134 or lu200134 or d2e7 or hsdb 7851 or hsdb7851 or abp-501 or abp501 or bcd-057 or bcd057 or bi-695501 or bi695501 or chs-1420 or chs1420 or gp-2017 or gp2017 or sb-5 or sb5).mp,rn. (9182)

100 (golimumab* or cnto 148 or cnto148 or simponi or 476181 74 5 or 91x1klu43e*).mp,rn. (1328)

101 Certolizumab Pegol/ (619)

102 (certolizumab* or cdp 870 or cdp870 or cimzia or pegylated tumor necrosis factor alpha antibody fab fragment or pegylated tumour necrosis factor alpha antibody fab fragment or pha 738144 or pha738144 or czp or 428863 50 7 or g6adw90r16* or umd07x179e* or hsdb 7848 or hsdb7848).mp,rn. (1574)

103 Rituximab/ (15102)

104 (rituximab* or ct p10 or ctp10 or idec 102 or idec102 or idecc2b8 or idec c2b8 or monoclonal antibody idec c2b8 or gp2013 or gp 2013 or "pf 05280586" or pf05280586 or mabthera or mab thera or r 105 or r105 or reditux or rg 105 or rg105 or rituxan or rituxin or ritemvia or rituzena or rixathon or riximyo or ro 452294 or ro452294 or truxima or 174722 31 7 or 4f4x42syq6* or hsdb 7455 or hsdb7455).mp,rn. (25061)

105 (ofatumumab* or humax cd20 or humax-cd20 or humaxcd20 or humax-cd20-2f2 or arzerra or gsk 1841157 or gsk1841157 or gski841157 or humax cd20 or omb 157 or omb157 or 679818 59 8 or m95kg522r0* or hsdb 8170 or hsdb8170).mp,rn. (602)

106 Abatacept/ (2952)

107 (abatacept* or ctla4 ig or ctla4 immunglobulin or ctla4 immunoglobulin g or ctla4ig or 'ctla 4 ig' or ctla-igg4m or ctla4-fc or bms 188667 or bms188667 or orencia or '332348 12 6' or 7d0yb67s97* or rg 2077 or rg2077 or rg-1046 or rg1046 or nulojix or lea29y or lea 29y or ctl4 fc or ctl4fc or belatacept or bms 224818 or bms224818 or belatacept or cytotoxic t lymphocyte associat* antigen 4).mp,rn. (5476)

108 (tabalumab* or ly 2127399 or ly2127399 or 1143503 67 6 or pqp8vh3mjw*).mp,rn. (58)

109 (pateclizumab* or mlta 3698a or mlta3698a or pro 283698 or pro283698 or 12202526 59 7 or qok1yyh7j2* or rg 7415 or rg7415).mp,rn. (6)

110 (secukinumab* or cosentyx or ain 457 or ain457 or 1229022 83 6 or dlg4eml025*).mp,rn. (1263)

111 (ixekizumab* or ly 2439821 or ly2439821 or taltz or 1329632 62 3 or 1143503 69 8 or bty153760o*).mp,rn. (626)

112 (brodalumab* or amg 827 or amg827 or khk 4827 or khk4827 or siliq or kyntheum or 1174395 19 7 or 6za31y954z*).mp,rn. (362)

113 (guselkumab* or cnto 1959 or cnto1959 or 1350289 85 8 or tremfya or 1350289 85 8 or 089658a12d*).mp,rn. (278)

114 Ustekinumab/ (1121)

115 (ustekinumab* or cnto 1275 or cnto1275 or stelara or "815610 63 0" or fu77b4u5z0* or 1275 cnto or 1275cnto or l04ac05 or tt-20 or tt20).mp,rn. (2227)

116 (mavrilimumab or "1085337 57 0" or 1158jdp9a* or cam 3001 or cam3001).mp,rn. (33)

117 exp Antirheumatic Agents/ (431920)

118 (dmard* or csdmard* or cs-dmard* or sdmard* or s-dmard* or tsdmard* or ts-dmard* or bdmard* or b-dmard*).ti,ab,kf. (5873)

119 (disease modifying adj3 (antirheum* or anti rheum*) adj3 (drug? or agent?)).ti,ab,kf. (6494)

120 ((antirheum* or anti rheum*) adj5 (drug? or agent?)).ti,ab,kf. (9119)

121 Methotrexate/ (38283)

122 (mxt or methotrexat* or metotrexat* or methoblastin or methylaminopterin* or amet?opterin* or abitrexate or antifolan or brimexate or a-met?opterin* or a-methpterin* or ai3-25299 or ai325299 or alpha-methopterin or biotrexate or emtexate or emtrexate* or emthexat* or enthexate* or farmitrexat* or farmotrex or fauldexato or folex or folex pfs or hdmtx or ifamet or imeth or maxtrex or metotressat* or methotrate* or methohexate* or mexate* or metrotex or metatrexato* or metex or metrex or metatrexan or maxtrex or meticil or metoject or metotrexin or novatrex* or neotrexate* or nordimet or lumexon or ledertrexate* or lanterel or rasuvo or r?eumatrex* or reditrex or rasuvo or texate or texate-t or tremetex or trexeron or trixilem or texorate or trexall or xaken or xatmep or otrexup or mpi-2505 or mpi2505 or mpi 5004 or mpi5004 or amethopterin* or 133073-73-1 or 15475-56-6 or 3ig1e710zn* or yl5fz2y5u1* or cl 14377 or cl14377 or ccris 1109 or ccris1109 or emt 25299 or emt25299 or nsc-740 or nsc740 or r-9985 or r9885 or x-133 or wr-19039 or wr19039).mp,rn. (55776)

123 Leflunomide/ (1536)

124 (leflunomid* or hwa 486 or hwa486 or su101 or su 101 or arava or arabloc or hsdb 7289 or hsdb7289 or g162gk9u4w* or 75706-12-6 or rs 34821 or rs34821).mp,rn. (2626)

125 lef.ti,ab,kf. (2563)

126 Sulfasalazine/ (4144)

127 (sulfasalazin* or sasp or ssz or salicylazosulfapyridin* or salicyl azo sulfapyridin* or sulphasalazin* or salazosulfapyridin* or salazosulfapiridin* or 'pyralin en' or azulfadin* or azulfidin* or asulfidin* or azulfide* or azulfin or colo-pleon or colopleon or disalazin or gastropyrin or pleon or "pleon ra" or "pyralin en" or rorasul or rosulfant or ulcol or ucine or salazopyrin* or ratio-sulfosalazin* or sulfasalazin* or 3xc8guz6cb* or accucol or asulfidin* or azopyrin* or azosulfidin* or benzosulfa or "brn 0356241" or brn0356241 or ccris 4713 or ccris4713 or einecs 209-974-3 or hsdb 3395 or hsdb3395 or nsc 203730 or nsc203730 or nsc 667219 or nsc667219 or reupirin or rorasul or salazo-sulfapyridin* or salazosulfpyridin* or salazopiridazin* or salazopyridin* or salazopirin* or salazodin or salisulf or salipyr or saridine or si-88 or si88 or sulculon or sulfasalazin* or sulphasalazin* or w-t sasp oral or 599-79-1 or "s.a.s. 500" or "s.a.s.-500" or sas-500 or zopyrin*).mp,rn. (8102)

128 Hydroxychloroquine/ (4067)

129 (hydroxychloroquin* or hcq or 747-3-4 or 8q2869cnvh* or 118-42-3 or 4qwg6n8qkh* or hydroxychlorochin* or hydrochloroquin* or hydrocloroquin* or oxychlorochin or oxychloroquin* or plaquenil or plaquinol or "brn 0253894" or brn0253894 or einecs 204-249-8 or einecs204-249-8 or hidroxicloroquin* or idrossiclorochin* or oxichlorochin* or oxichloroquin* or oxiklorin or win1258 or win 1258 or z0188 or einecs 212-019-3 or einecs212-019-3 or chloroquinol or dimard or ercoquin or evoquin or erquin or geniquin or quensyl or sn 8137 or sn8137 or toremonil or yuma).mp,rn. (7770)

130 Gold Sodium Thiomalate/ (1262)

131 (gold sodium thiomalate* or aurothiomalate* or aurolate* or gold thiomalate* or gold disodium thiomalate* or gold thiomalic acid* or miocrin or miocrisin or monogold disodium thiomalate* or myochrysin* or myocrisin* or myocrysin* or sodium aurothiomalate* or sodium gold thiomalate* or sodium thiomalate* gold or sodium thiomalatoaurate* or tauredon or taureodon or tauredone or thiomalate gold or thiomalatoaurate sodium or 1244-57-4 or 39377-38-3 or 4846-27-9 or 74916-57-7 or e4768zy6gm* or aurothiomala-natrium or aurot?iomalato sodico or monogold* sodium salt or dinatrium 2-aurothio-succinat or einecs 235-479-7 or einecs235-479-7 or hsdb 7173 or hsdb7173 or kidon or natrii aurothiomalas* or shiosol or gold mercatpsuccinate*).mp,rn. (1547)

132 Aurothioglucose/ (834)

133 (thioglucosoaurate* or gold thioglucose* or gold thio glucose* or thioglucose gold or aureotan or solganal or solganol or gold-50 or gold50 or solganal b or b oleosum solganal or auromyose* or aurothioglucose* or auro thioglucose* or aurotan or aurumine or aurumin or authron or brenol or ccris 59 or ccris59 or einecs 235-365-7 or einecs235-365-7 or glysanol b or goldthioglucose* or goldtioglucose* or hsdb 7174 or hsdb7174 or oronol or romosol or skf 10056 or skf10056 or 2p2v9q0e78* or 12192-57-3).mp,rn. (1133)

134 ((gold* or auro*) adj10 (inject* or intramuscular* or im or intra muscular*)).ti,ab,kf,kw. (2766)

135 Organogold Compounds/ (941)

136 (aurothiopropanol* or allochrysine or allocrisine or aurotiopoe or aurotioprol or 27279-43-2 or g7097j63e9* or auromercapto propanol sulfonate sodium or auromercaptopropanolsulfonate sodium or aurothioisopropanolsulfonate sodium or sodium aurothiopropanolsulfonate or gold sodium thiopropanol sulfonate or sodium auromercaptopropanol sulfonate).mp,rn. (91)

137 Chloroquine/ (14614)

138 (chloroquin* or cq or 54-04-7 or 886u3h6uff* or aralen or aralene or arechin or arechine or arequin or chingamin or chlorochin* or khingamin or nivaquine or oe48649kh6n* or amokin or amokine or anaclor or aralan or arequine or artriquine or avloclor or avoclor or arthrochin or artrichin or bemaco or

bemaphata or bemaphate or bemasulph or benaquin or bipiquin or cadiquin or "brn 0482809" or brn0482809 or capquin or ccris 3439 or ccris3439 or chemochin or chemochine or chingamine or chingaminum or chloraquin* or chlorquin or chlorquine or choloquine or choroquine sulfate or choroquine sulphate or cindacin or clo-kit junior or clorichina or clorichine or cloriquine or clorochina or delagil or delagyl or diclokin or chlorofoz or cindachin or clorochina or cloroquina or cocatrit or dichinalex or diquinalex or diroquine or emquin or genocin or gontochin or gontochine or gontoquine or einecs 200-191-2 or einecs200-191-2 or elestol or gontochin or heliopar or hsdb 3029 or hsdb3029 or imagon or iroquine or klorokin or klorokine or klorokinfosfat or lagaquin or lapaquin or malaquin or malaren or malarex or malarivon or malaviron or maliaquine or maquine or mesylith or mexaquin or mirquin or neochin or nivaquin or nivachine or nivaquine b or nivaquine dp or nivaquine forte or "p roquine" or nsc 187208 or nsc187208 or pfizerquine or quensyl or quinachlor or quinercyl or quingamine or quinilon or quinoline or quinoscan or repal or resochen or resochene or resochin or resochin junior or resochina or resochine or resochinon or resoquina or resoquine or reumachlor or ronaquine or roquine or rp 3377 or rp3377 or sanoquin or sanoquine or silbesan or siragan or sirajan or sn 7618 or sn7618 or solprina or solprine or sopaquin or st 21 or tresochin or tresoquine or trochin or trochine or troquine or weimerquin or w 7618 or w7618 or win 244 or win244).mp,rn. (35198)

139 exp Adrenal Cortex Hormones/ (399728)

140 exp Prednisolone/ (51507)

141 Prednisone/ (39644)

142 Cortisone/ (19618)

143 Hydrocortisone/ (72490)

144 exp Steroids/ (862842)

145 adrenal cortex hormones.ti,ab,kf,rn. (65488)

146 prednisolone*.mp,rn. (46695)

147 methylprednisolone*.mp,rn. (27149)

148 (prednisolone* or predonine or di-adreson-f or diadresonf or 9phq9y1olm* or prednisolona* or prednisolonum* or nsc-9120 or nsc9120 or nsc 9900 or nsc9900 or delta-dehydrocortisol* or delta-dehydrocortisone* or delta-hydrocortisone or hydroretrocortine or metacortandralone* or brn 1354103 or brn1354103 or bubbli-pred or ccris 980 or ccris980 or co-hydeltra or codelcortone or cordrol or cortalone or cotogesic or cotolone or decaprednil or decortin h or delcortol or dehydrohydrocortisone* or delta-cortef or deltacortef or delta-ef-cortelan or delta-stab or deltacortenol or deltacortril or deltahydrocortisone* or deltisilone or derpo pd or dexa-dortidelt hostacortin h or di-adreson f or dicortol or donisolone or dydeltrone or eazolin d or ec 200-021-7 or ec200-021-7 or

einecs 200-021-7 or einecs200-021-7 or erbacort or erbasona or estilsona or fernisolone or fernisolone p or hostacortin h or hsdb 3385 or hsdb3385 or hydeltra or hydeltrone or hydrodeltalone or hydrodeltisone or hydroretrocortin or hydroretrocortine* or k 1557 or k1557 or lentosone or meti-drm or meticortelone or orapred or orapred odt or paracortol or paracotol or pediapred or precortancyl or precortilon or precortisyl or predne-dome or prednelan or prednicen or predniliderm or predniretard or prednis or predonin or predonine* or prelon or prelone or prenolone or rolisone or scherisolon? or solone or sterolone or ulacort or ultracorten h or ultracortene h adelcort or antisolon or antisolone or aprednisolon? or benisolon? or caberdelta or co hydeltra or codelcortone or ompresolon).mp,rn. (46783)

149 (cortadeltona or cortelinter or cortisolone or dacortin or dacortin h or dacrotin or decaprednil or decortil or delta cortril or delta hycortol or deltacortenolo or deltacortil or deltacortoil or deltaderm or deltadglycortril or deltahycortol or deltahydrocortison? or deltaophticor or deltasolone or deltastab or deltidrosol or deltisilone or deltisolon? or deltolasson? or deltosona or deltosone or depo-predate or dermosolon or dhasolone or di adreson? f or diadreson? f or dicortol or domucortone or encortelon? or encortolon or equisolon or glistelone or hefasolon or hydrelta or hydrocortancyl or hydrocortidelt or hydradeltalone or hydradeltisone or inflanefran or insolone or keteocort h or key-pred or key-pred sp or lenisolone or leocortol or liquipred or mediasolone or meprisolon? or metacortalon? or metacortelone or meti derm or metiderm or meticortelone or morlone or mydrapred or nisolon? or opredsone or panafcortelone or panafcortolone or panafort or paracortol pr phlogex or pre cortisyl or preconin or precortalon or precortancyl or precortisyl or pred ject 50 or predacort 50 or predaject 50 or predalone 50 or predartrina or predartrine or predate 50 or predeltilone or predisole or predisyr or predne dome or prednecort or prednedome or prednelan or predni coelin or predni h tablinen or predni helvacort or prednicoelin or prednicort or prednicortelone or prednifor drops or predniment or prednorsolon? or predorgasolon? or prenilone or preventan or prezolon or rubycort or serilone or solonda or solupren? or spiricort or spolotane or supercorti#ol or taracortelone or walesolone or wysolone).mp,rn. (63)

150 (methylprednisolone * or methylprednisolona* or methylprednisolonum* or metipred or urbason or medrol or x4w7zr7023* or 83-43-2 or besonia or brn 23403300 or brn2340300 or a-methapred or artisone-wyeth or besonia or depo-medrol or dopomedrol ec 201-4764 pr ec2014764 or einecs 201-476-4 or einecs201-476-4 or esametone or firmacort or hsdb3127 or hsdb3127 or lemod or medesone or medixon or medlone 21 or medrate or medrol or medrone or mesopren or metastab or methyleneprednisolone* or metilbetasone or metrisone or metrocort or metysolon or moderin or nirypan or noretona or nsc 19987 or nsc19987 or predni n tablinen or prednol-l or promacortine or reactenol or sieropresol or solomet or summicort or suprametil or u 7532 or u7532 or "u 67 590a" or urbason or urbasone or wyacort or adlone 40 or adlone 80 or beta methylprednisolone or medalone 80 or depmedalone or deproject 80 or depopred or esametone or firmacort or med-jec-40 or medixon or mednin or medralone or mepredprednisolone or meprelon or mesopren or methacort 40 or methacort 80 or methyxlcotol or methylcotolone or methylpred dp or methylsterolone or metidrol or metycortin or metypred or metypresol or neomedrone or solu decortin or urbason).mp,rn. (19852)

151 (prednison or prednisone or prednisona or prednisonum or dehydrocortisone or delta cortisone or rectodelt or sterapred or ultracorten or winpred or apo prednisone or cortan or cartancyl or panafcort or cutason or decortin or dacortin or decortisyl or deltasone or encortone or encorton or enkorton or enkortolon or kortancyl or liquid pred or meticorten or orasone or panasol or predni tablinen or prednidib or predniment or pronisone or vb0r961hzt* or 53-

03-2 or adasone or al3-52939 or ancortone or bicortone or cartancyl or ccris 2646 or ccrs2646 or colisone or cortan or cortidelt or cotone or dacorten or dacortin or decortancyl or dekortin or dellacort or delta cortelan or delta cortisone or delta dome or deltacortene or deltacortisone or deltacortone or deltasone or deltison or deltisona or deltra or di adreson or diadreson or econosone or einecs 200-160-3 or einecs200-160-3 or fernisone or fiasone or hostacortin or hsdb 3168 or hsdb3168 or incocortyl or juvason or lisacort or lodotra or lodtra).mp,rn. (54206)

152 (me-korti or metacortandracin or metacorten or nci c04897 or ncic04897 or nisona or nizon or novoprednisone or nsc 10023 or nsc10023 or nurison or orasone or panafcort or paracort or prarmenison or pehacort or predeltin or prednicen-m or prednicorm or prednicort or prednicot or prednilonga or prednitone or prednizon or prednovister or presone or pronison or rayos or rectodelt or retrocortine or servisone or sterapred or supercortio or u 6020 or u6020 or ultracorten or ultracortene or winpred or wojtab or zenadrid).mp,rn. (356)

153 cortisone?.mp,rn. (23572)

154 corticosteroid*.mp,rn. (106849)

155 corticoid*.mp,rn. (6605)

156 glucocorticoid*.mp,rn. (114642)

157 corticosterone*.mp,rn. (34886)

158 hydrocortisone*.mp,rn. (77972)

159 dexamethasone*.mp,rn. (73076)

160 beclomethasone*.mp,rn. (3845)

161 (triamcinolone* or 1zk20vi6ty* or 124-94-7 or aristocort or volon or fluoxyprednisolon* or tiamcinolonum or triamcinolonum* or triamcinolona* or brn 2341955 or brn2341955 or cl 19823 or cl19823 or einecs 204-718-7 or einecs204-718-7 or hsdb 3194 or hsdb3194 or nsc 13397 or nsc13397).mp,rn. (11996)

162 (steroid? adj10 (therap* or treat* or drug? or inject* or intramuscular* or intra muscular* or im or intravenous* or intra venous* or iv or intraarticular* or intra articular* or ia or administrat* or oral or orally or parenteral*)).ti,ab,kf. (74426)

163 Janus Kinase Inhibitors/ (463)

164 ((jak or jak1 or jak2 or jak3 or janus kinase) adj10 inhibitor*).mp,rn. (6531)

165 (janus adj4 kinase adj10 inhibitor?).mp,rn. (2593)

166 exp Janus Kinases/ai [Antagonists & Inhibitors] (2313)

167 (baricitinib* or 1187594-09-7 or isp4442i3y* or "incb 028050" or incb028050 or ly-3009104 or ly3009104).mp,rn. (448)

168 (tofacitinib* or 477600-75-2 or 87la6fu830* or hsdb 8311 or hsdb8311 or cp-690 550 or cp 690550 or cp690550).mp,rn. (1644)

169 (filgotinib* or 1206161-97-8 or 3xvl385q0m* or "glpg 0634" or glpg0634).mp,rn. (115)

170 (upadacitinib* or 1310726-60-3 or 4RA0KN46E0* or abt 494 or abt494).mp,rn. (145)

171 (ruxolitinib* or rux or 941678-49-5 or 82s8x8xx8h* or hsdb 8259 or hsdb8259 or inc 424 or inc424 or incb424 or "incb 018424" or incb018424 or incb 18424 or incb18424 or r-ruxolitinib* or jakafi or jakavi).mp,rn. (1735)

172 (itacitinib or "incb 039110" or incb039110 or incb 39110 or incb39110 or 1334298-90-6 or 19j3781lpm*).mp,rn. (25)

173 (ag490 or ag 490 or tyrphostin b42 or tyrphostin ag 490 or tyrphostin ag490 or 133550-30-8).mp,rn. (1525)

174 (fedratinib* or tg101348 or tg 101348 or sar302503 or sar 302503 or 936091-26-8 or 6l1xp550i6* or inrebic).mp,rn. (155)

175 (ritlecitinib* or pf-06651600 or pf06651600 or pf 6651600 or pf6651600 or 2140301-97-7 or 132lf5wgh4* or 2192215-81-7 or eag4t1459k* or ritlecitinib malonate or ritlecitinib toslate or ritlecitinib tosylate or ritlecitinib propanedioate).mp,rn. (10)

176 (peficitinib* or peficitinib hydrobromide).mp,rn. (64)

177 or/92-176 (1532367)

178 80 and 177 (44310)

179 91 or 178 (44837)

180 ae.fs. (1764893)

181 (adverse adj3 (event* or effect* or reaction* or incident* or outcome*)).ti,ab,kf. (464870)

182 ((serious* or severe* or severit*) adj3 (adverse or saes or aes or adrs or sae or "ae" or adr)).ti,ab,kf. (47848)

183 (saes or aes or adrs or sae or "ae" or adr).ti,ab,kf. and adverse.mp. (21835)

184 (adverseevent* or adversedrugreaction* or adversereaction* or adverseincident* or adverseoutcome*).ti,ab,kf. (13)

185 side effect*.ti,ab,kf. (261262)

186 exp "Drug-Related Side Effects and Adverse Reactions"/ (117928)

187 Adverse Drug Reaction Reporting Systems/ (7722)

188 exp Product Surveillance, Postmarketing/ (15906)

189 Substance Withdrawal Syndrome/ (21479)

190 withdraw*.ti,ab,kf. (129331)

191 safety.ti,ab,kf. (541227)

192 exp Drug Hypersensitivity/ (46442)

193 (hypersensitiv* or hyper sensitiv*).ti,ab,kf. (78902)

194 Anaphylaxis/ (21208)

195 anaphyla*.ti,ab,kf. (30424)

196 (toxic* adj6 (drug* or medication*)).ti,ab,kf. (37582)

197 intoxication*.ti,ab,kf. (42337)

198 Abnormalities, Drug-Induced/ (14573)

199 Teratogenesis/de (94)

200 teratogen*.ti,ab,kf. (17235)

201 ((drug or chemical*) adj1 induced).ti,ab,kf. (53898)

202 exp substance related disorders/ (282326)

203 (treatment adj3 emergent).ti,ab,kf. (6005)

204 exp Mortality/ (390908)

205 exp Death, Sudden/ (35398)

206 exp Death/ (150433)

207 (fatal or fatalit*).ti,ab,kf. (150887)

208 ((mortality or death?) adj4 (rate? or ratio or caus* or risk or sudden or number* or amount* or count*)).ti,ab,kf. (550074)

209 mortality.ti,ab,kf. (799095)

210 exp *Infections/ (2272322)

211 infection rate?.ti,ab,kf. (25559)

212 ((serious* or severe* or severit*) adj8 (infect* or bacter?emi* or septic?emi* or sepsis or fung?emi* or fungal infect* or fungal disease* or mycoses or mycosis or mycotic)).ti,ab,kf. (101643)

213 exp Opportunistic Infections/ (34824)

214 ((opportunistic* or opportune) adj6 (infect* or bacter?emi* or septic?emi* or bacterial infect* or bacterial diseas* or viral infect* or virus diseas* or virus infect* or microbial infect* or mycos#s or fung* infect* or mycotic diseas* or mycos#s infect* or fung?emi*)).ti,ab,kf. (20320)

215 ((overall or rate or rates or risk?) adj8 (infect* or bacter?emi* or septic?emi* or sepsis or bacterial infect* or bacterial diseas* or bacterious infect* or viral infect* or virus diseas* or virus infect* or microbial infect* or mycos#s or fung* infect* or mycotic diseas* or mycos#s infect* or fung?emi*)).ti,ab,kf. (213961)

216 infect*.ti,ab,kf. (1836501)

217 exp Tuberculosis/ (193696)

218 (tuberculosis or tuberculous).ti,ab,kf. (230442)

219 exp Cytomegalovirus Infections/ (25981)

220 ((cytomegalovirus or cytomegaly virus or cytomegaloviral or cmv) adj (infection? or disease?)).ti,ab,kf. (17392)

221 (cytomegalic inclusion adj3 disease?).ti,ab,kf. (576)

222 cytomegaloinfection?.ti,ab,kf. (1)

223 congenital cytomegalic inclusion.ti,ab,kf. (39)

224 cmv.ti,ab,kf. and (virus or viral or infect*).mp. (23171)

225 exp Mycobacterium Infections, Nontuberculous/ (35268)

226 exp Nontuberculous Mycobacteria/ (11804)

227 ((atypical or nontuberculous* or non-tuberculous*) adj3 (mycobacterios* or mycobacter*)).ti,ab,kf. (8135)

228 (mycobacter* adj3 intracellulare adj3 infection*).ti,ab,kf. (538)

229 exp Mycoses/ (127951)

230 (mycosis or mycoses or mycotic or fung*).ti,ab,kf. (234801)

231 (aspergillos#s or (aspergill* adj3 infect*)).ti,ab,kf. (15725)

232 (candidias#s* or candidemi* or (candid* adj3 infect*)).ti,ab,kf. (24187)

233 pneumocyst*.ti,ab,kf. (11778)

234 exp Hepatitis B/ (58975)

235 Hepatitis B virus/ (27517)

236 hepatitis b.ti,ab,kf. (80542)

237 (hepatitis adj1 (type b or injection or serum)).ti,ab,kf. (3066)

238 (hepatitis adj3 "b").ti,ab,kf. (84674)

239 hbv.ti,ab,kf. (43661)

240 exp Infections/ (2663468)

241 (opportunistic or serious* or severe* or severit*).ti,ab,kf. (1724016)

242 240 and 241 (248423)

243 exp Varicella Zoster Virus Infection/ (18305)

244 ((herpes or varicella*) adj1 zoster*).ti,ab,kf. (17679)

245 (zoster or shingles or varicellovirus infect*).ti,ab,kf. (19006)

246 exp Herpes Simplex/ (24391)

247 (herpes adj3 (simplex or vulgaris or infection*)).ti,ab,kf. (44028)

248 herpes.ti,ab,kf. (62569)

249 hsv.ti,ab,kf. and (infect* or herpes).mp. (23170)

250 243 or 244 or 245 or 246 or 247 or 248 or 249 (82586)

251 Virus Activation/ (7648)

252 (reactivat* or re-activat*).ti,ab,kf. (44493)

253 (activat* adj3 (virus or viral* or vir?emi*)).ti,ab,kf. (6323)

254 251 or 252 or 253 (53410)

255 250 and 254 (5395)

256 C-Reactive Protein/ (46897)

257 9007-41-4.rn. (46897)

258 ((c-reactive or creactive or c reaction or creaction) adj2 protein).ti,ab,kf. (72750)

259 crp.ti,ab,kf. (50714)

260 Blood Sedimentation/ (11925)

261 ((erythrocyte or blood) adj3 sedimentation*).ti,ab,kf. (16536)

262 esr.ti,ab,kf. and sedimentation.mp. (6463)

263 (DAS28-ESR or DAS-28-ESR or DAS28ESR).ti,ab,kf. (627)

264 ((DAS28 or "DAS 28" or disease activity score or das or disease activity) and ESR).ti,ab,kf. (2993)

265 esr.ti,ab,kf. (19862)

266 Procalcitonin/ (702)

267 Calcitonin/ (15827)

268 (procalcitonin or pro-calcitonin or pct).ti,ab,kf. (10984)

269 (calcitonin adj3 precursor?).ti,ab,kf. (134)

270 exp Leukocyte Count/ (98452)

271 ((leukocyte* or white blood cell or white cell or white blood or wbc) adj3 count*).ti,ab,kf. (39791)

272 wbc.ti,ab,kf. (17663)

273 exp Neoplasms/ (3398253)

274 exp Hematologic Neoplasms/ (21558)

275 exp histiocytic disorders, malignant/ or exp leukemia/ or exp lymphoma/ (385954)

276 exp Multiple Myeloma/ (41805)

277 (lymphom* or leuk?em* or myeloma*).ti,ab,kf. (477116)

278 ((h?ematolog* or blood disease) adj6 (malignan* or neoplas*)).ti,ab,kf. (31306)

279 (cancer or cancers).ti,ab,kf. (1861769)

280 neoplas*.ti,ab,kf. (408153)

281 (tumor? or tumour?).ti,ab,kf. (1718026)

282 (carcinom* or karzinom*).ti,ab,kf. (686061)

283 hodgkin*.ti,ab,kf. (68794)

284 (non-hodgkin* or nonhodgkin*).ti,ab,kf. (39071)

285 (mesotheliom* or mesoteliom*).ti,ab,kf. (16773)

286 malignan*.ti,ab,kf. (592632)

287 adenocarcinom*.ti,ab,kf. (153910)

288 metasta*.ti,ab,kf. (528864)

289 sarcom*.ti,ab,kf. (113039)

290 adenosarcom*.ti,ab,kf. (586)

291 osteosarcom*.ti,ab,kf. (24702)

292 reticulosarcom*.ti,ab,kf. (1297)

293 lymphosarcom*.ti,ab,kf. (5203)

294 (blastom* or neuroblastom*).ti,ab,kf. (47062)

295 (gliom* or glioblastom*).ti,ab,kf. (84577)

296 melanom*.ti,ab,kf. (122051)

297 ((non melanom* or nonmelanom*) adj3 skin adj3 (cancer or cancers or neoplas* or tumo?r* or carcino*)).ti,ab,kf. (5547)

298 (solid adj6 (cancer or cancers or neoplas* or tumo?r* or carcino* or malignan*)).ti,ab,kf. (74784)

299 exp Diverticulitis/ (6588)

300 diverticulitis*.ti,ab,kf. (6946)

301 Intestinal Perforation/ (13203)

302 ((digestive system or gastrointestinal* or gastro intestinal* or intestinal*) adj3 perforation*).ti,ab,kf. (5290)

303 exp Liver/ (445443)

304 Liver Function Tests/ (29134)

305 ((liver or hepat*) adj1 function?).ti,ab,kf. (48393)

306 (lft or lfts).ti,ab,kf. (1288)

307 (hypertransaminasemi* or hyper transaminasemi*).ti,ab,kf. (381)

308 uln.ti,ab,kf. (1235)

309 exp "Chemical and Drug Induced Liver Injury"/ (30002)

310 (dili or drug induc* liver injur*).ti,ab,kf. (3416)

311 Alanine Transaminase/ (31689)

312 exp Aspartate Aminotransferases/ (30015)

313 (transaminas* or alanin* aminotransaminas* or alt or aspartat* aminotransaminas* or ast or serum glutam* pyruv* transaminas* or sgpt or serum glutam* oxalacet* transaminas* or sgot or liver enzyme* or hepat* enzyme*).ti,ab,kf. (86123)

314 transaminitis*.ti,ab,kf. (749)

315 (hepat*tox* or ((liver or hepat*) adj3 toxic*)).ti,ab,kf. (38509)

316 exp Lipoproteins/bl [Blood] (77408)

317 exp Triglycerides/ (77574)

318 exp "lipoprotein(a)"/ or exp lipoproteins, hdl/ or exp lipoproteins, ldl/ (82202)

319 exp Cholesterol/ (161519)

320 ((lipid? or cholesterol* or cholesterin* or triglycerid* or lipoprotein*) adj3 (level? or blood or plasma or serum)).ti,ab,kf. (190068)

321 (ldl or low density lipoprotein*).ti,ab,kf. (113469)

322 (hdl or high density lipoprotein*).ti,ab,kf. (91014)

323 (hdl2 or "hdl(2)" or "hdl 2").ti,ab,kf. (2596)

324 (hdl3 or "hdl(3)" or "hdl 3").ti,ab,kf. (2532)

325 (lipoprotein a or lipoproteina).ti,ab,kf. (7677)

326 alpha lipoprotein.ti,ab,kf. (334)

327 (ldl1 or "ldl(1)" or "ldl 1").ti,ab,kf. (306)

328 (ldl2 or "ldl(2)" or "ldl 2").ti,ab,kf. (314)

329 exp Lipids/bl [Blood] (220090)

330 exp Hyperlipidemias/ (66656)

331 (hyperlipid?emi* or lipid?emi*).ti,ab,kf. (32731)

332 exp Diabetes Mellitus/ (433946)

333 exp Obesity/ (218277)

334 (metabolic adj3 (effect? or alterat* or chang* or risk? or transition* or transformat* or parameter? or marker? or biomarker?)).ti,ab,kf. (85521)

335 (diabetes* or diabetic*).ti,ab,kf. (656872)

336 Glycated Hemoglobin A/ (35812)

337 (hba1c or hbaic or "hb a1c" or "hb a 1c" or "hb aic" or "hb a ic" or "hba 1c" or "hba ic").ti,ab,kf. (40552)

338 ("h?emoglobin a1c" or "h?emoglobin a 1c" or h?emoglobin aic or "h?emoglobin a ic").ti,ab,kf. (13821)

339 body mass index/ (129951)

340 (body mass index or bmi).ti,ab,kf. (257072)

341 Venous Thromboembolism/ (11354)

342 ((venous* or venos* or vein*) adj1 (thrombos#s* or thrombotic*or thromboemboli* or thrombo emboli* or thrombus or thrombi or microthromb*)).ti,ab,kf. (53636)

343 (dvt or dvts or vte or vtes).ti,ab,kf. (20587)

344 exp Pulmonary Embolism/ (39476)

345 ((lung* or pulmonary) adj3 (embol* or thromboemboli* or thromb* emboli* or microemboli* or clot* or thrombus or thrombi or microthromb* or infarct*)).ti,ab,kf. (52100)

346 (pe or pes).ti,ab,kf. and (lung* or pulmo* or pneumo* or embol* or thromboembol* or thrombot* or thrombus or thrombi or microembol*).mp. (11909)

347 exp Anemia/ (162709)

348 an?emi*.ti,ab,kf. (158577)

349 exp Thrombocytopenia/ (48322)

350 (thrombocytopeni* or thrombopeni*).ti,ab,kf. (64047)

351 exp Neutropenia/ (18879)

352 neutropeni*.ti,ab,kf. (42083)

353 exp Lymphopenia/ (5866)

354 (lymphocytopeni* or lymphopeni*).ti,ab,kf. (9455)

355 Macrophage Activation Syndrome/ (468)

356 mas.ti,ab,kf. (23022)

357 (macrophage activat* adj3 syndrome?).ti,ab,kf. (1065)

358 355 or 356 or 357 (23677)

359 (Rheumatoid Arthritis adj3 Systemic adj3 Juvenile).mp. (303)

360 Arthritis, Juvenile/ (10531)

361 ((arthrit* or artrit*) adj6 juvenile).mp. (13591)

362 JIA.ti,ab,kf,kw. (4114)

363 360 or 361 or 362 (14150)

364 (systemic or polyart* or poly art* or oligoart* or oligo art*).mp. (536171)

365 363 and 364 (4843)

366 ((systemic adj6 jia) or sjia or s-jia).ti,ab,kf. (867)

367 359 or 365 or 366 (4851)

368 358 and 367 (339)

369 exp Heart Failure/ (124536)

370 (congestive adj2 (heart or cardiac) adj2 failure*).ti,ab,kf. (44398)

371 (heart failure* or cardiac failure* or myocard* failure*).ti,ab,kf. (191061)

372 ((heart or cardiac or cordis or cardio* or myocard*) adj1 decompensatio*).ti,ab,kf. (1597)

373 (chf or ccf or hf).ti,ab,kf. and (heart or cardiac or cardio* or myocard*).mp. (41407)

374 exp Myocardial Ischemia/ (435420)

375 ((heart or cardio* or cardiac or myocardial) adj1 infarct*).ti,ab,kf. (205613)

376 exp Myocardial Infarction/ (177633)

377 cardiovascular* stroke?.ti,ab,kf. (47)

378 (MACE or MACEs).ti,ab,kf. (8808)

379 major adverse card* event*.ti,ab,kf. (11413)

380 acute coronary syndrome*.ti,ab,kf. (33616)

381 (heart attack or heart attacks).ti,ab,kf. (5729)

382 (mi or mis).ti,ab,kf. and (myocard* or cardio* or coronary or heart or cardiac or mace or maces or fatal* or non-fatal* or nonfatal*).mp. (33006)

383 (angina or anginas or stenocardia?).mp. (71037)

384 exp Stroke/ (139133)

385 ((cerebrovascular* or cerebr* vascular*) adj1 (accident? or failure? or injury or injuries or insult? or insufficienc* or apoplex*)).ti,ab,kf. (10666)

386 (stroke or strokes or apoplexi* or apoplexy*).ti,ab,kf. (264504)

387 (cva or cvas).ti,ab,kf. (3114)

388 (brain insult* or brain isch?emic attack? or cerebral insult* or brain vascular accident? or isch?emic cerebr* attack? or isch?emic seizure? or cerebr* vascular accident?).ti,ab,kf. (2965)

389 exp Myocardial Revascularization/ (92791)

390 (revascularizat* or re-vascularizat* or revascularisat* or re-vascularisat*).ti,ab,kf,hw. (66451)

391 Peripheral Arterial Disease/ (8299)

392 Arterial Occlusive Diseases/ (27411)

393 (peripher* adj2 (arter* or vascular*) adj2 disease*).ti,ab,kf. (30418)

394 ((pad or pvd) adj6 (peripher* or arter* or vascular*)).ti,ab,kf. (7127)

395 exp Kidney Function Tests/ (80530)

396 ((kidney or renal) adj1 function*).ti,ab,kf. (110288)

397 Creatinine/bl [Blood] (35714)

398 creatinin*.ti,ab,kf. and (serum or plasma or blood).mp. (93043)

399 (creatinin* adj15 (level? or concentration? or ratio or clearance or value?)).ti,ab,kf. (74525)

400 (creatinin* adj15 (decreas* or increas* or elevat* or lower* or low or high or higher)).ti,ab,kf. (48186)

401 AYI8EX34EU.rn. (56931)

402 (estimated glomerul* filtrat* rate? or estimated glomerulofiltration rate?).ti,ab,kf. (18833)

403 (egfr or egfrs or e-gfr or e-gfrs or estimat* gfr or estimat* gfrs or estimat*gfr or estimat*gfrs).ti,ab,kf. (64378)

404 Glomerular Filtration Rate/ (45122)

405 exp Renal Insufficiency/ (176694)

406 ((renal or kidney) adj1 (insufficien* or failure? or injur*)).ti,ab,kf. (155188)

407 exp Hemoglobins/ (128028)

408 h?emoglobin*.ti,ab,kf. (174953)

409 exp Osteoporosis/ (56475)

410 (osteoporos* or osteoporotic*).ti,ab,kf. (80349)

411 exp Leukoencephalopathies/ (32186)

412 (leukoencephalopath* or leuko encephalopath*).ti,ab,kf. (7870)

413 exp Demyelinating Diseases/ (102186)

414 ((demyelinat* or demyeliniz*) adj3 (disease* or disorder* or cns or central nervous system* or encephalopath* or autoimmune disease*)).ti,ab,kf. (11100)

415 demyelinat*.mp. (37590)

416 demyeliniz*.mp. (499)

417 demyelinis*.mp. (304)

418 exp Multiple Sclerosis/ (59938)

419 multiple scleros#s.ti,ab,kf. (78203)

420 exp Optic Neuritis/ (8721)

421 (optic* adj3 (neuritis* or neuritides*)).ti,ab,kf. (6306)

422 (optic* adj3 (inflammat* adj3 neuropath*)).ti,ab,kf. (125)

423 exp Myelitis, Transverse/ (4309)

424 (transverse adj3 (myelitis* or myelitides* or myelopath* syndrome*)).ti,ab,kf. (2275)

425 (necrotiz* adj3 (myelitis* or myelitides*)).ti,ab,kf. (41)

426 (demyelinat* adj3 (myelitis* or myelitides*)).ti,ab,kf. (48)

427 ((postinfect* or infect* or postvaccin* or vaccin*) adj10 (myelitis or myelitides)).ti,ab,kf. (648)

428 exp Peripheral Nervous System Diseases/ (149861)

429 (peripheral adj2 (nervous system or nerve) adj2 (disease* or disorder* or neuropath* or mononeuropath* or polyneuropath* or polyradiculaneuropath*)).ti,ab,kf. (2104)

430 exp Guillain-Barre Syndrome/ (5258)

431 (guillain barre* or fisher? syndrome*).ti,ab,kf. (10508)

432 ((landry? or landry guillain barre?) adj3 (syndrom* or paralys*)).ti,ab,kf. (230)

433 (miller fisher? adj3 (syndrom* or polyneuritis* or variant?)).ti,ab,kf. (876)

434 ((polyradiculoneuropath* or polyneuropath* or neuronitis* or polyneuritis*) adj15 (inflammat* or postinfect* or infect* or postvaccin* or vaccin*)).ti,ab,kf. (4220)

435 Polyradiculoneuropathy, Chronic Inflammatory Demyelinating/ (1558)

436 ((polyradiculoneuropath* or polyneuropath* or polyradiculopath*) adj6 chronic* adj3 inflammat*).ti,ab,kf. (2690)

437 cidp.ti,ab,kf. (1849)

438 exp Polyradiculoneuropathy/ (12779)

439 polyradiculoneuropath*.ti,ab,kf. (1688)

440 ((demyelinat* or inflammat*) adj1 (neuropath* or polyneuropath* or polyradiculoneuropath*)).ti,ab,kf. (5647)

441 (multifocal motor neuropath* adj6 conduction block*).ti,ab,kf. (133)

442 (motor adj3 neuropath* adj6 conduction block*).ti,ab,kf. (216)

443 exp Motor Neuron Disease/ (28482)

444 (motor adj3 neuropath*).ti,ab,kf. (4617)

445 exp Mononeuropathies/ (20361)

446 (mononeuropath* or mono neuropath* or mononeuritis* or mono neuritis* or mononeuritides* or mono neuritides*).ti,ab,kf. (2413)

447 exp Polyneuropathies/ (28002)

448 (polyneuropath* adj6 axonal adj3 (sensor* motor or sensorimotor* or sensorymotor*)).ti,ab,kf. (240)

449 polyneuropath*.ti,ab,kf. (14959)

450 exp Vaccination/ (86794)

451 exp Vaccines/ (232392)

452 vaccinat*.ti,ab,kf. (160178)

453 (active adj2 immuni#at*).ti,ab,kf. (3776)

454 vaccine*.ti,ab,kf. (247081)

455 exp Pregnancy/ (905362)

456 exp Pregnancy Complications/ (431829)

457 pregnan*.ti,ab,kf. (537500)

458 tolerabilit*.ti,ab,kf. (50959)

459 safe.ti,ab,kf. (378779)

460 ((injurious* or undesirabl*) adj3 (effect? or reaction* or event? or outcome? or incident?)).ti,ab,kf. (20828)

461 exp Postoperative Complications/ (555964)

462 exp Intraoperative Complications/ (53524)

463 complication*.ti,ab,kf. (1013363)

464 co.fs. (2003906)

465 risk.ti,ab,kf. (2204872)

466 de.fs. (3025206)

467 exp risk/ (1238723)

468 180 or 181 or 182 or 183 or 184 or 185 or 186 or 187 or 188 or 189 or 190 or 191 or 192 or 193 or 194 or 195 or 196 or 197 or 198 or 199 or 200 or 201 or 202 or 203 or 204 or 205 or 206 or 207 or 208 or 209 or 210 or 211 or 212 or 213 or 214 or 215 or 216 or 217 or 218 or 219 or 220 or 221 or 222 or 223 or 224 or 225 or 226 or 227 or 228 or 229 or 230 or 231 or 232 or 233 or 234 or 235 or 236 or 237 or 238 or 239 or 242 or 255 or 256 or 257 or 258 or 259 or 260 or 261 or 262 or 263 or 264 or 265 or 266 or 267 or 268 or 269 or 270 or 271 or 272 or 273 or 274 or 275 or 276 or 277 or 278 or 279 or 280 or 281 or 282 or 283 or 284 or 285 or 286 or 287 or 288 or 289 or 290 or 291 or 292 or 293 or 294 or 295 or 296 or 297 or 298 or 299 or 300 or 301 or 302 or 303 or 304 or 305 or 306 or 307 or 308 or 309 or 310 or 311 or 312 or 313 or 314 or 315 or 316 or 317 or 318 or 319 or 320 or 321 or 322 or 323 or 324 or 325 or 326 or 327 or 328 or 329 or 330 or 331 or 332 or 333 or 334 or 335 or 336 or 337 or 338 or 339 or 340 or 341 or 342 or 343 or 344 or 345 or 346 or 347 or 348 or 349 or 350 or 351 or 352 or 353 or 354 or 368 or 369 or 370 or 371 or 372 or 373 or 374 or 375 or 376 or 377 or 378 or 379 or 380 or 381 or 382 or 383 or 384 or 385 or 386 or 387 or 388 or 389 or 390 or 391 or 392 or 393 or 394 or 395 or 396 or 397 or 398 or 399 or 400 or 401 or 402 or 403 or 404 or 405 or 406 or 407 or 408 or 409 or 410 or 411 or 412 or 413 or 414 or 415 or 416 or 417 or 418 or 419 or 420 or 421 or 422 or 423 or 424 or 425 or 426 or 427 or 428 or 429 or 430 or 431 or 432 or 433 or 434 or 435 or 436 or 437 or 438 or 439 or 440 or 441 or 442 or 443 or 444 or 445 or 446 or 447 or 448 or 449 or 450 or 451 or 452 or 453 or 454 or 455 or 456 or 457 or 458 or 459 or 460 or 461 or 462 or 463 or 464 or 465 or 466 or 467 (16665073)

469 179 and 468 (36405)

470 ((eular or (european adj4 rheumat*)) adj8 (meeting* or conference* or congress*) adj8 annual).af. (67)

471 ((acr or "acr/arhp" or (american college adj4 rheumat*)) adj8 (meeting* or conference* or congress*) adj8 annual).af. (137)

472 470 or 471 (172)

473 469 and 472 (13)

474 limit 473 to yr="2019 - 2020" (3)

475 limit 469 to yr="2012 - 2020" (16988)

476 limit 475 to english (15948)

477 exp Registries/ (99877)

478 (register or registry or registries or registered or registration*).ti,ab,kf. (405714)

479 Cohort Studies/ (274628)

480 (cohort* adj6 (study* or studies* or analy*)).ti,ab,kf. (314635)

481 (cohortstudy* or cohortstudies* or cohortanaly*).ti,ab,kf. (4)

482 exp case-control studies/ (1130493)

483 (case? adj3 control* adj3 (study* or match*)).ti,ab,kf. (104105)

484 case referent.ti,ab,kf. (660)

485 (case? adj2 control?).ti,ab,kf. (152262)

486 Prospective Studies/ (558848)

487 exp Longitudinal Studies/ (140801)

488 Retrospective Studies/ (861131)

489 (longitudinal or prospective* or retrospective*).ti,ab,kf. (1663270)

490 Follow-Up Studies/ (653351)

491 ((follow up or followup) adj6 (trial or trials or study or studies or analy*)).ti,ab,kf. (146525)

492 490 or 491 (741792)

493 (long term or longterm).ti,ab,kf. (831940)

494 (extend* or extension?).ti,ab,kf. (647544)

495 (continuing or continue* or continuat*).ti,ab,kf. (505112)

496 493 or 494 or 495 (1897822)

497 492 and 496 (155560)

498 ((long term or longterm) adj3 data).ti,ab,kf. (16096)

499 497 or 498 (168109)

500 477 or 478 or 479 or 480 or 481 or 482 or 483 or 484 or 485 or 486 or 487 or 488 or 489 or 499 (3022097)

501 476 and 500 (4643)

502 randomized controlled trial.pt. (519902)

503 exp randomized controlled trial/ (520921)

504 exp Randomized Controlled Trials as Topic/ (142582)

505 randomi*.ti,ab,kf. (669047)

506 randomly.ti,ab,kf. (349385)

507 trial.ti. (232377)

508 trial.ab. and random*.mp. (315155)

509 (rct or rcts).ti,ab,kf. and random*.mp. (47623)

510 Random Allocation/ (104289)

511 controlled clinical trial/ (93994)

512 Controlled Clinical Trials as Topic/ (5555)

513 Single-Blind Method/ (29491)

514 Double-Blind Method/ (161580)

515 ((singl* or doubl* or tripl* or trebl*) adj3 (blind* or mask*)).ti,ab,kf. (178833)

516 (singl*blind* or doubl*blind* or trebl*blind* or tripl*blind*).ti,ab,kf. (252)

517 (singl*mask* or doubl*mask* or tripl*mask* or trebl*mask*).ti,ab,kf. (4)

518 (controlled adj8 (study or design or trial)).ti,ab,kf. (277654)

519 (parallel adj1 group*).ti,ab,kf. (19652)

520 exp placebo effect/ (4888)

521 Placebos/ (35259)

522 placebo*.ti,ab,kf. (222081)

523 (allocated or allocation or assigned).ti,ab,kf. and random*.mp. (193965)

524 groups.ti,ab,kf. (2174116)

525 dt.fs. (2264407)

526 ((assign* or match or matched or allocation) adj6 (alternate or group or groups or intervention? or patient? or subject? or participant?)).ti,ab,kf. (267999)

527 ('head to head' adj3 (study or comparison or trial)).ti,ab,kf. and random*.mp. (863)

528 502 or 503 or 504 or 505 or 506 or 507 or 508 or 509 or 510 or 511 or 512 or 513 or 514 or 515 or 516 or 517 or 518 or 519 or 520 or 521 or 522 or 523 or 524 or 525 or 526 or 527 (5040565)

529 496 and 528 (479030)

530 476 and 529 (1378)

531 501 or 530 (5368)

532 exp animals/ not humans/ (4771716)

533 531 not 532 (5359)

534 review.pt. (2740585)

535 congress.pt. (66476)

536 editorial.pt. (552345)

537 letter.pt. (1115441)

538 534 or 535 or 536 or 537 (4447258)

539 533 not 538 (4493)

540 539 or 474 (4495)

1.3.4.2 EMBASE

#443,"#440 OR #442",6871,27 Nov 2020

#442,"#435 NOT #441",6030,27 Nov 2020

#441,"#429 NOT #434 AND ([conference abstract]/lim OR [conference paper]/lim OR [conference review]/lim OR [editorial]/lim OR [letter]/lim OR [review]/lim)",7329,27 Nov 2020

#440,"#435 AND #438 AND [2019-2020]/py",841,27 Nov 2020

#439,"#435 AND #438",2600,27 Nov 2020

#438,"#436 OR #437",50862,27 Nov 2020

#437,"acr:nc AND annual:nc AND meeting:nc AND rheumatology:nc",19451,27 Nov 2020

#436,"'eular':nc AND 'annual european congress of rheumatology':nc",31411,27 Nov 2020

#435,"#429 NOT #434",13359,27 Nov 2020

#434,"#432 AND #433",1116683,27 Nov 2020

#433,"'animal experiment'/de",2607791,27 Nov 2020

#432,"#430 OR #431",2477250,27 Nov 2020

#431,"mouse:tt OR mice:tt OR rat:tt OR rats:tt OR murine$:tt OR swine:tt OR porcine:tt OR sheep:tt OR lamb:tt OR lambs:tt OR pig:tt OR pigs:tt OR piglets:tt OR rabbit:tt OR rabbits:tt OR cat:tt OR cats:tt OR dog:tt OR dogs:tt OR cattle:tt OR bovine:tt OR monkey:tt OR monkeys:ti OR trout:tt OR marmoset*:tt OR 'zebra fish*':tt OR zebrafish*:tt",39760,27 Nov 2020

#430,"mouse:ti OR mice:ti OR rat:ti OR rats:ti OR murine$:ti OR swine:ti OR porcine:ti OR sheep:ti OR lamb:ti OR lambs:ti OR pig:ti OR pigs:ti OR piglets:ti OR rabbit:ti OR rabbits:ti OR cat:ti OR cats:ti OR dog:ti OR dogs:ti OR cattle:ti OR bovine:ti OR monkey:ti OR monkeys:ti OR trout:ti OR marmoset*:ti OR 'zebra fish*':ti OR zebrafish*:ti",2476396,27 Nov 2020

#429,"#427 NOT #428",13362,27 Nov 2020

#428,"('animal experiment'/de OR 'animal model'/de OR 'animal tissue'/de OR 'animal cel') NOT ('human experiment'/de OR 'human'/de)",2763835,27 Nov 2020

#427,"#378 AND #424 AND [2012-2020]/py AND [english]/lim",13430,27 Nov 2020

#426,"#378 AND #424 AND [2012-2020]/py",13621,27 Nov 2020

#425,"#378 AND #424",17578,27 Nov 2020

#424,"#379 OR #380 OR #381 OR #382 OR #383 OR #384 OR #385 OR #386 OR #387 OR #388 OR #391 OR #392 OR #400 OR #402 OR #423",4032272,27 Nov 2020

#423,"#399 AND #422",152554,27 Nov 2020

#422,"#403 OR #404 OR #405 OR #406 OR #407 OR #408 OR #409 OR #410 OR #411 OR #412 OR #413 OR #414 OR #415 OR #416 OR #417 OR #418 OR #419 OR #420 OR #421",1873160,27 Nov 2020

#421,"(((allocated OR allocation) NEAR/3 random*):ti,ab,kw) OR (((allocated OR allocation) NEAR/3 random*):tt)",46003,27 Nov 2020

#420,"placebo*:ti,ab,kw OR placebo*:tt",317297,27 Nov 2020

#419,"((parallel NEXT/1 group*):ti,ab,kw) OR ((parallel NEXT/1 group*):tt)",26390,27 Nov 2020

#418,"((controlled NEAR/8 (study OR design OR trial)):ti,ab,kw) OR ((controlled NEAR/8 (study OR design OR trial)):tt)",376243,27 Nov 2020

#417,"singl*blind:tt OR doubl*blind*:tt OR trebl*blind*:tt OR tripl*blind*:tt OR singl*mask*:tt OR doubl*mask*:tt OR trebl*mask*:tt OR tripl*mask*:tt",33,27 Nov 2020

#416,"singl*blind:ti,ab,kw OR doubl*blind*:ti,ab,kw OR trebl*blind*:ti,ab,kw OR tripl*blind*:ti,ab,kw OR singl*mask*:ti,ab,kw OR doubl*mask*:ti,ab,kw OR trebl*mask*:ti,ab,kw OR tripl*mask*:ti,ab,kw",207825,27 Nov 2020

#415,"((singl* OR doubl* OR tripl* OR trebl*) NEAR/3 (blind* OR mask*)):tt",69,27 Nov 2020

#414,"((singl* OR doubl* OR tripl* OR trebl*) NEAR/3 (blind* OR mask*)):ti,ab,kw",248619,27 Nov 2020

#413,"'triple blind procedure'/de",267,27 Nov 2020

#412,"'double blind procedure'/de",178320,27 Nov 2020

#411,"'single blind procedure'/de",40787,27 Nov 2020

#410,"'controlled clinical trial'/de",431732,27 Nov 2020

#409,"'randomization'/exp",88773,27 Nov 2020

#408,"(rct:ti,ab,kw OR rcts:ti,ab,kw OR rct:tt OR rcts:tt) AND random*",68454,27 Nov 2020

#407,"randomly:ti,ab,kw OR randomly:tt",457932,27 Nov 2020

#406,"trial:ab AND random*:ti,ab,kw,de",421684,27 Nov 2020

#405,"trial:ti OR trial:tt",320118,27 Nov 2020

#404,"randomi*:ti,ab,kw OR randomi*:tt",949281,27 Nov 2020

#403,"'randomized controlled trial'/de",630730,27 Nov 2020

#402,"#390 AND #401",22917,27 Nov 2020

#401,"((('long-term' OR longterm) NEAR/3 data):ti,ab,kw) OR ((('long-term' OR longterm) NEAR/3 data):tt)",26070,27 Nov 2020

#400,"#395 AND #399",285043,27 Nov 2020

#399,"#396 OR #397 OR #398",1381581,27 Nov 2020

#398,"continuation:ti,ab,kw OR continuation:tt",31593,27 Nov 2020

#397,"extension:ti,ab,kw OR extension:tt",216845,27 Nov 2020

#396,"'long term':ti,ab,kw OR longterm:ti,ab,kw OR 'long term':tt OR longterm:tt",1151771,27 Nov 2020

#395,"#393 OR #394",1683760,27 Nov 2020

#394,"(('follow up' OR followup) NEAR/6 (trial OR trials OR study OR studies OR analy* OR design)):ti,ab,kw",234805,27 Nov 2020

#393,"'follow up'/de",1622453,27 Nov 2020

#392,"'long term follow up'/de",27,27 Nov 2020

#391,"#389 AND #390",1958383,27 Nov 2020

#390,"study:ti,ab,kw,de OR trial:ti,ab,kw,de OR studies:ti,ab,kw,de OR trials:ti,ab,kw,de OR analys$s:ti,ab,kw,de OR design:ti,ab,kw,de",22240025,27 Nov 2020

#389,"longitudinal:ti,ab,kw OR 'prospective':ti,ab,kw OR retrospective:ti,ab,kw OR longitudinal:tt OR 'prospective':tt OR retrospective:tt",2030346,27 Nov 2020

#388,"'retrospective study'/de",993704,27 Nov 2020

#387,"'longitudinal study'/exp",147182,27 Nov 2020

#386,"'prospective study'/de",642001,27 Nov 2020

#385,"(case$:ti,ab,kw OR case$:tt) AND (control*:ti,ab,kw OR control*:tt) AND (study:ti,ab,kw,de OR studies:ti,ab,kw,de OR match*:ti,ab,kw,de OR trial:ti,ab,kw,de OR trials:ti,ab,kw,de OR design:ti,ab,kw,de OR analy*:ti,ab,kw,de)",641122,27 Nov 2020

#384,"(('case control' NEAR/3 (study OR match*)):ti,ab,kw) OR (('case control' NEAR/3 (study OR match*)):tt)",128210,27 Nov 2020

#383,"'case control study'/exp",180422,27 Nov 2020

#382,"((cohort* NEAR/6 (study* OR studies* OR analy*)):ti,ab,kw) OR ((cohort* NEAR/6 (study* OR studies* OR analy*)):tt) OR cohortstudy*:ti,ab,kw OR cohortstudies*:ti,ab,kw OR cohortanaly*:ti,ab,kw OR cohortstudy:tt OR cohortstudies:tt OR cohortanaly*:tt",478927,27 Nov 2020

#381,"'cohort analysis'/de",637513,27 Nov 2020

#380,"register:ti,ab,kw OR registry:ti,ab,kw OR registries:ti,ab,kw OR registered:ti,ab,kw OR registration$:ti,ab,kw OR register:tt OR registry:tt OR registries:tt OR registered:tt OR registration$:tt",557474,27 Nov 2020

#379,"'register'/de",113724,27 Nov 2020

#378,"#165 AND #377",73799,27 Nov 2020

#377,"#166 OR #167 OR #168 OR #169 OR #170 OR #171 OR #172 OR #173 OR #174 OR #175 OR #176 OR #177 OR #178 OR #179 OR #180 OR #181 OR #182 OR #183 OR #184 OR #185 OR #186 OR #187 OR #188 OR #189 OR #190 OR #191 OR #192 OR #193 OR #194 OR #195 OR #196 OR #197 OR #198 OR #199 OR #200 OR #201 OR #202 OR #203 OR #204 OR #205 OR #206 OR #207 OR #208 OR #209 OR #210 OR #211 OR #212 OR #213 OR #214 OR #215 OR #216 OR #217 OR #218 OR #219 OR #220 OR #221 OR #222 OR #223 OR #224 OR #227 OR #235 OR #236 OR #237 OR #238 OR #239 OR #240 OR #241 OR #242 OR #243 OR #244 OR #245 OR #246 OR #247 OR #248 OR #249 OR #250 OR #251 OR #252 OR #253 OR #254 OR #255 OR #256 OR #257 OR #258 OR #259 OR #260 OR #261 OR #262 OR #263 OR #264 OR #265 OR #266 OR #267 OR #268 OR #269 OR #270 OR #271 OR #272 OR #273 OR #274 OR #275 OR #276 OR #277 OR #278 OR #279 OR #280 OR #281 OR #282 OR #283 OR #284 OR #285 OR #286 OR #287 OR #288 OR #289 OR #290 OR #291 OR #292 OR #293 OR #294 OR #295 OR #296 OR #297 OR #298 OR #299 OR #313 OR #314 OR #315 OR #316 OR #317 OR #318 OR #319 OR #320 OR #321 OR #322 OR #323 OR #324 OR #325 OR #326 OR #327 OR #328 OR #329 OR #330 OR #331 OR #332 OR #333 OR #334 OR #335 OR #336 OR #337 OR #338 OR #339 OR #340 OR #341 OR #342 OR #343 OR #344 OR #345 OR #346 OR #347 OR #348 OR #349 OR #350 OR #351 OR #352 OR #353 OR #354 OR #355 OR #356 OR #357 OR #358 OR #359 OR #360 OR #361 OR #362 OR #363 OR #364 OR #365 OR #366 OR #367 OR #368 OR #369 OR #370 OR #371 OR #372 OR #373 OR #374 OR #375 OR #376",16904453,27 Nov 2020

#376,"complication$:ti,ab,kw OR complication$:tt",1415795,27 Nov 2020

#375,"'complication'/exp",1260879,27 Nov 2020

#374,"'postoperative complication'/exp",736468,27 Nov 2020

#373,"'infectious complication'/exp",187741,27 Nov 2020

#372,"((injurious OR undesirabl*) NEAR/3 (effect$ OR reaction$ OR event$ OR outcome$ OR incident$)):tt",0,27 Nov 2020

#371,"((injurious OR undesirabl*) NEAR/3 (effect$ OR reaction$ OR event$ OR outcome$ OR incident$)):ti,ab,kw",18225,27 Nov 2020

#370,"safe:ti,ab,kw OR safe:tt",563570,27 Nov 2020

#369,"tolerability:ti,ab,kw OR tolerability:tt",90823,27 Nov 2020

#368,"'drug tolerability'/de",132960,27 Nov 2020

#367,"'pregnancy complication'/exp",153423,27 Nov 2020

#366,"pregnan*:ti,ab,kw OR pregnan*:tt",694841,27 Nov 2020

#365,"'pregnancy'/exp",780117,27 Nov 2020

#364,"'vaccine*':ti,ab,kw OR vaccine*:tt",293559,27 Nov 2020

#363,"'vaccine'/exp",355213,27 Nov 2020

#362,"vaccinat*:ti,ab,kw OR vaccinat*:tt",192756,27 Nov 2020

#361,"'vaccination'/exp",181813,27 Nov 2020

#360,"('peripheral nervous system' NEAR/1 (disorder$ OR diseas* OR neuropath* OR mononeuropath* OR polyneuropath* OR polyradiculoneuropath*)):tt",0,27 Nov 2020

#359,"('peripheral nervous system' NEAR/1 (disorder$ OR diseas* OR neuropath* OR mononeuropath* OR polyneuropath* OR polyradiculoneuropath*)):ti,ab,kw",701,27 Nov 2020

#358,"'axonal sensorimotor polyneuropathy'/de",17,27 Nov 2020

#357,"'mononeuropathy'/exp OR 'mononeuritis' OR 'mononeuropathies' OR 'mononeuropathy'",80657,27 Nov 2020

#356,"'motor neuropathy'/exp OR 'motor neuropathy' OR 'multifocal motor neuropathy' OR 'neuropathy, motor'",6490,27 Nov 2020

#355,"(((demyelinat* OR inflammator*) NEAR/1 (neuropath* OR polyneuropath* OR polyradiculoneuropath*)):ti,ab,kw) OR ((demyelinat* NEAR/1 (neuropath* OR polyneuropath* OR polyradiculoneuropath*)):tt)",9038,27 Nov 2020

#354,"cidp:ti,ab,kw OR cidp:tt",3759,27 Nov 2020

#353,"'polyradiculoneuropathy'/exp OR 'chronic inflammatory demyelinating polyradiculoneuropathy' OR 'polyradiculoneuropathy' OR 'polyradiculoneuropathy, chronic inflammatory demyelinating' OR 'radiculoneuropathy'",18709,27 Nov 2020

#352,"'chronic inflammatory neuropathy'",34,27 Nov 2020

#351,"'guillain barre syndrome'/de OR 'fisher syndrome' OR 'guillain barre' OR 'guillain barre disease' OR 'guillain barre polyradiculitis' OR 'guillain barre polyradiculoneuritis' OR 'guillain barre syndrome' OR 'guillain-barre syndrome' OR 'landry guillain barre strohl syndrome' OR 'landry guillain barre syndrome' OR 'landry paralysis' OR 'landry syndrome' OR 'miller fisher syndrome' OR 'acute febrile polyneuritis' OR 'acute postinfective polyradiculoneuropathy' OR 'infectious neuronitis' OR 'inflammatory acute polyradiculoneuropathy' OR 'polyradiculoneuritis guillain-barre' OR 'polyradiculoneuropathy, acute postinfective' OR 'polyradiculoneuropathy, inflammatory acute'",19528,27 Nov 2020

#350,"'peripheral neuropathy'/exp",76028,27 Nov 2020

#349,"'acute transverse myelitis':ti,ab,kw OR 'transverse myelitis':ti,ab,kw OR 'demyelinat* myelit*':ti,ab,kw OR 'acute transverse myelitis':tt OR 'transverse myelitis':tt OR 'demyelinat* myelit*':tt",4071,27 Nov 2020

#348,"'acute transverse myelitis'/de",65,27 Nov 2020

#347,"'optic neuritis'/exp OR 'acute optic neuritis' OR 'inflammatory optic neuropathies' OR 'inflammatory optic neuropathy' OR 'neuritis optica' OR 'neuritis, optic' OR 'optic neuritis' OR 'opticus neuritis'",14224,27 Nov 2020

#346,"((demyelinat* NEAR/3 (brain OR cns OR disease$ OR 'nervous system' OR disorder$)):ti,ab,kw) OR ((demyelinat* NEAR/3 (brain OR cns OR disease$ OR 'nervous system' OR disorder$)):tt)",17665,27 Nov 2020

#345,"'multiple scleros$s*':ti,ab,kw OR 'multiple scleros$s*':tt",121991,27 Nov 2020

#344,"'demyelinating disease'/exp OR 'brain demyelination' OR 'clinically isolated syndrome' OR 'cns demyelinating autoimmune diseases' OR 'demyelinating autoimmune diseases, cns' OR 'demyelinating brain disease' OR 'demyelinating disease' OR 'demyelinating disease, hereditary central nervous system' OR 'demyelinating diseases' OR 'demyelinating encephalopathy' OR 'demyelination disease' OR 'demyelinizing disease' OR 'hereditary central nervous system demyelinating disease' OR 'hereditary central nervous system demyelinating diseases' OR 'heredodegenerative disorders, nervous system' OR 'nervous system heredodegenerative disorders'",184289,27 Nov 2020

#343,"leukoencephalopath*:ti,ab,kw OR leukoencephalopath*:tt",11547,27 Nov 2020

#342,"'leukoencephalopathy'/de",7296,27 Nov 2020

#341,"osteoporos*:ti,ab,kw OR osteoporotic:ti,ab,kw OR osteoporos*:tt OR osteoporotic:tt",125178,27 Nov 2020

#340,"'osteoporosis'/exp",133355,27 Nov 2020

#339,"egfr:ti,ab,kw,de OR egfr:tt",119907,27 Nov 2020

#338,"'estimated glomerular* filtrat* rate$':ti,ab,kw OR 'estimated glomerular* filtrat* rate$':tt",27837,27 Nov 2020

#337,"((creatinin* NEAR/3 (serum OR plasma OR blood)):ti,ab,kw) OR ((creatinin* NEAR/3 (serum OR plasma OR blood)):tt)",86917,27 Nov 2020

#336,"'creatinine blood level'/de",114637,27 Nov 2020

#335,"'kidney function'/exp",201901,27 Nov 2020

#334,"'peripheral occlusive artery disease'/exp",178923,27 Nov 2020

#333,"revascularizat*:ti,ab,kw OR revascularisat*:ti,ab,kw OR revascularizat*:tt OR revascularisat*:tt",94201,27 Nov 2020

#332,"'heart muscle revascularization'/de",32786,27 Nov 2020

#331,"'revascularization'/exp",56800,27 Nov 2020

#330,"'brain insult*':tt OR 'brain isch$emic attack$':tt OR 'cerebral insult*':tt OR 'brain vascular accident$':tt OR 'isch$emic cerebral attack$':tt OR 'isch$emic seizure$':tt OR 'cerebrum vascular accident$':tt",3,27 Nov 2020

#329,"'brain insult*':ti,ab,kw OR 'brain isch$emic attack$':ti,ab,kw OR 'cerebral insult*':ti,ab,kw OR 'brain vascular accident$':ti,ab,kw OR 'isch$emic cerebral attack$':ti,ab,kw OR 'isch$emic seizure$':ti,ab,kw OR 'cerebrum vascular accident$':ti,ab,kw",2334,27 Nov 2020

#328,"cva:ti,ab,kw OR cvas:ti,ab,kw OR cva:tt OR cvas:tt",6937,27 Nov 2020

#327,"stroke:ti,ab,kw OR strokes:ti,ab,kw OR apoplexi*:ti,ab,kw OR apoplexy*:ti,ab,kw OR stroke:tt OR strokes:tt OR apoplexi*:tt OR apoplexy*:tt",418365,27 Nov 2020

#326,"((cerebrovascular* OR 'cerebro vascular*' OR 'cerebral vascular*') NEAR/1 (accident$ OR failure$ OR injury OR injuries OR insult$ OR insufficienc* OR apoplexi*)):tt",199,27 Nov 2020

#325,"((cerebrovascular* OR 'cerebro vascular*' OR 'cerebral vascular*') NEAR/1 (accident$ OR failure$ OR injury OR injuries OR insult$ OR insufficienc* OR apoplexi*)):ti,ab,kw",17196,27 Nov 2020

#324,"'cerebrovascular accident'/exp",335826,27 Nov 2020

#323,"'heart attack$':ti,ab,kw OR 'heart attack$':tt",8279,27 Nov 2020

#322,"(((myocardial OR heart) NEAR/1 (infarct$ OR infarction$)):ti,ab,kw) OR (((myocardial OR heart) NEAR/1 (infarct$ OR infarction$)):tt)",298850,27 Nov 2020

#321,"'non fatal myocardial infarction'/de",27,27 Nov 2020

#320,"'acute heart infarction'/de",75653,27 Nov 2020

#319,"'stroke patient'/exp",33161,27 Nov 2020

#318,"'heart infarction'/exp",403727,27 Nov 2020

#317,"'ischemic heart disease'/exp",700619,27 Nov 2020

#316,"'heart failure$':ti,ab,kw OR 'cardiac failure$':ti,ab,kw OR 'heart failure$':tt OR 'cardiac failure$':tt",310369,27 Nov 2020

#315,"((congestive NEAR/2 (heart OR cardiac) NEAR/2 failure$):ti,ab,kw) OR ((congestive NEAR/2 (heart OR cardiac) NEAR/2 failure$):tt)",59293,27 Nov 2020

#314,"'congestive heart failure'/exp",112632,27 Nov 2020

#313,"#304 AND #312",883,27 Nov 2020

#312,"#310 AND #311",9205,27 Nov 2020

#311,"systemic:ti,ab,kw OR polyart*:ti,ab,kw OR 'poly art*':ti,ab,kw OR oligoart*:ti,ab,kw OR 'oligo art*':ti,ab,kw OR systemic:tt OR polyart*:tt OR 'poly art*':tt OR oligoart*:tt OR 'oligo art*':tt",719551,27 Nov 2020

#310,"#307 OR #308 OR #309",24687,27 Nov 2020

#309,"jia:ti OR jia:tt",1260,27 Nov 2020

#308,"(((arthrit* OR artrit*) NEAR/6 juvenile*):ti,ab,kw) OR (((arthrit* OR artrit*) NEAR/6 juvenile*):tt)",17888,27 Nov 2020

#307,"'juvenile rheumatoid arthritis'/de",21383,27 Nov 2020

#306,"((systemic NEAR/6 (jia OR 'juvenile idiopathic arthritis' OR 'juvenile onset idiopathic arthriti')):ti,ab,kw) OR sjia:ti,ab,kw",3330,27 Nov 2020

#305,"'systemic juvenile idiopathic arthritis'/de",1404,27 Nov 2020

#304,"#300 OR #301 OR #302 OR #303",27221,27 Nov 2020

#303,"('macrophag* activat*' NEAR/3 (syndrome* OR factor$)):ti,ab,kw",2890,27 Nov 2020

#302,"'macrophage activating factor'/de",956,27 Nov 2020

#301,"'macrophage activation syndrome*':ti,ab,kw OR mas:ti,ab,kw OR 'macrophage activation syndrome*':tt OR mas:tt",25163,27 Nov 2020

#300,"'macrophage activation syndrome'/de",2245,27 Nov 2020

#299,"an$emi*:ti,ab,kw OR thrombocytopeni*:ti,ab,kw OR neutropeni*:ti,ab,kw OR lymphopeni*:ti,ab,kw OR lymphocytopeni*:ti,ab,kw OR an$emi*:tt OR thrombocytopeni*:tt OR neutropeni*:tt OR lymphopeni*:tt OR lymphocytopeni*:tt",368650,27 Nov 2020

#298,"((g3 NEAR/2 g4):ti,ab,kw) OR ((g3 NEAR/2 g4):tt)",2429,27 Nov 2020

#297,"'lymphocytopenia'/exp",24073,27 Nov 2020

#296,"'neutropenia'/exp",121982,27 Nov 2020

#295,"'thrombocytopenia'/exp",182278,27 Nov 2020

#294,"'anemia'/exp",403123,27 Nov 2020

#293,"(pe:ti,ab,kw OR pes:ti,ab,kw OR pe:tt OR pes:tt) AND (lung* OR pulmo* OR pneumo* OR embol* OR thromboembol* OR thrombot* OR thrombus OR thrombi OR microembol*)",23258,27 Nov 2020

#292,"(((lung* OR pulmonary) NEAR/3 (embol* OR thromboemboli* OR 'thromb* emboli*' OR microemboli* OR clot* OR thrombus OR thrombi OR microthromb*)):ti,ab,kw) OR (((lung* OR pulmonary) NEAR/3 (embol* OR thromboemboli* OR 'thromb* emboli*' OR microemboli* OR clot* OR thrombus OR thrombi OR microthromb*)):tt)",77655,27 Nov 2020

#291,"'pulmonary infarct*':ti,ab,kw OR 'pulmonary infarct*':tt",1453,27 Nov 2020

#290,"dvt:ti,ab,kw OR dvts:ti,ab,kw OR vte:ti,ab,kw OR vtes:ti,ab,kw OR dvt:tt OR dvts:tt OR vte:tt OR vtes:tt",38011,27 Nov 2020

#289,"((venous* OR venos* OR vein*) NEAR/1 (thrombos?s* OR thrombotic* OR thromboemboli* OR 'thrombo emboli*' OR thrombus OR thrombi OR microthromb*)):tt",1457,27 Nov 2020

#288,"((venous* OR venos* OR vein*) NEAR/1 (thrombos?s* OR thrombotic* OR thromboemboli* OR 'thrombo emboli*' OR thrombus OR thrombi OR microthromb*)):ti,ab,kw",108920,27 Nov 2020

#287,"'venous thromboembolism'/exp",163340,27 Nov 2020

#286,"'bmi'/de OR 'body mass index':ti,ab,kw OR bmi:ti,ab,kw OR 'body mass index':tt OR bmi:tt",463320,27 Nov 2020

#285,"'body mass'/de",456521,27 Nov 2020

#284,"'hemoglobin a1c'/exp OR 'hb a1c' OR 'glycated haemoglobin a1c' OR 'glycated hemoglobin a1c' OR 'glycosylated haemoglobin a1c' OR 'glycosylated hemoglobin a1c' OR 'haemoglobin a1c' OR 'haemoglobin a (1c)' OR 'haemoglobin a 1c' OR 'haemoglobin aic' OR 'hb a (1c)' OR 'hba 1c' OR 'hba1c' OR 'hemoglobin a1c' OR 'hemoglobin a (1c)' OR 'hemoglobin a 1c' OR 'hemoglobin aic'",125702,27 Nov 2020

#283,"diabetes*:ti,ab,kw OR diabetic*:ti,ab,kw OR diabetes*:tt OR diabetic*:tt",991940,27 Nov 2020

#282,"'diabetes mellitus'/exp",1025246,27 Nov 2020

#281,"(metabolic NEAR/3 (effect$ OR alterat* OR chang* OR risk$ OR transition* OR transformat* OR parameter$ OR marker$ OR biomarker$)):ti,ab,kw",116324,27 Nov 2020

#280,"'metabolic parameters'/exp",318119,27 Nov 2020

#279,"'metabolic effects'/de",223,27 Nov 2020

#278,"hyperlipid$emi*:ti,ab,kw OR lip$edemi*:ti,ab,kw OR hyperlipid$emi*:tt OR lip$edemi*:tt",52585,27 Nov 2020

#277,"'hyperlipidemia'/exp",165121,27 Nov 2020

#276,"'low density lipoprotein cholesterol'/exp OR 'ldl cholesterol' OR 'cholesterol, ldl' OR 'lipoproteins, ldl cholesterol' OR 'low density lipoprotein cholesterol'",123484,27 Nov 2020

#275,"'high density lipoprotein cholesterol'/de OR 'hdl cholesterol' OR 'cholesterol, hdl' OR 'high density lipoprotein cholesterol' OR 'lipoproteins, hdl cholesterol'",127619,27 Nov 2020

#274,"'lipoprotein a'/exp OR 'lipoprotein (a)' OR 'lipoprotein a'",12698,27 Nov 2020

#273,"hdl:ti,ab,kw OR ldl:ti,ab,kw OR hdl:tt OR ldl:tt",167514,27 Nov 2020

#272,"((lipid OR cholesterol* OR cholesterin* OR triglyceride* OR triaglycer* OR lipoprotein* OR fat) NEAR/3 (level$ OR blood OR plasma OR serum)):tt",271,27 Nov 2020

#271,"((lipid OR cholesterol* OR cholesterin* OR triglyceride* OR triaglycer* OR lipoprotein* OR fat) NEAR/3 (level$ OR blood OR plasma OR serum)):ti,ab,kw",238219,27 Nov 2020

#270,"'lipid blood level'/exp",209151,27 Nov 2020

#269,"'lipid level'/exp",20990,27 Nov 2020

#268,"'liver function':ti,ab,kw OR 'liver function':tt",58607,27 Nov 2020

#267,"uln:ti,ab,kw,de AND (liver*:ti,ab,kw,de OR hepat*:ti,ab,kw,de)",2607,27 Nov 2020

#266,"((elevat* NEAR/6 transaminas*):ti,ab,kw) OR ((elevat* NEAR/6 transaminas*):tt)",8919,27 Nov 2020

#265,"'hypertransaminasemi*':ti,ab,kw OR 'hyper transaminasemi*':ti,ab,kw OR 'hypertransaminasemi*':tt OR 'hyper transaminasemi*':tt",759,27 Nov 2020

#264,"'hypertransaminasemia'/de",18228,27 Nov 2020

#263,"'liver function test'/de",48409,27 Nov 2020

#262,"'liver function'/exp",83903,27 Nov 2020

#261,"((('digestive system' OR gastrointestinal OR 'gastro intestinal*' OR intestinal*) NEAR/3 perforation$):ti,ab,kw) OR ((('digestive system' OR gastrointestinal* OR 'gastro intestinal*' OR intestinal*l) NEAR/3 perforation$):tt)",7057,27 Nov 2020

#260,"'digestive system perforation'/exp",51327,27 Nov 2020

#259,"diverticulitis*:ti,ab,kw OR diverticulitis*:tt",9867,27 Nov 2020

#258,"'digestive system perforation'/exp",51327,27 Nov 2020

#257,"'diverticulitis'/de",8725,27 Nov 2020

#256,"'malignanc*':ti,ab,kw OR 'malignanc*':tt",392313,27 Nov 2020

#255,"'malignancies'/de",29,27 Nov 2020

#254,"((solid NEAR/6 malignan* NEAR/3 (tumo$r$ OR cancer OR neoplasm$)):ti,ab,kw) OR ((solid NEAR/6 malignan* NEAR/3 (tumo$r$ OR cancer OR neoplasm$)):tt)",6415,27 Nov 2020

#253,"((h$ematol* NEAR/6 malignan*):ti,ab,kw) OR ((h$ematol* NEAR/6 malignan*):tt)",51083,27 Nov 2020

#252,"(malignan* NEAR/6 (tumo$r$ OR cancer OR neoplasm$)):tt",18,27 Nov 2020

#251,"(malignan* NEAR/6 (tumo$r$ OR cancer OR neoplasm$)):ti,ab,kw",241702,27 Nov 2020

#250,"(('non melanoma' OR nonmelanoma) NEAR/3 'skin' NEAR/3 (cancer OR tumo$r* OR neoplasm$)):tt",2,27 Nov 2020

#249,"(('non melanoma' OR nonmelanoma) NEAR/3 'skin' NEAR/3 (cancer OR tumo$r* OR neoplasm$)):ti,ab,kw",6418,27 Nov 2020

#248,"'non melanoma skin cancer'/exp",74320,27 Nov 2020

#247,"lymphom*:ti,ab,kw OR leuk$emi*:ti,ab,kw OR myelom*:ti,ab,kw OR lymphom*:tt OR leuk$emi*:tt OR myelom*:tt",664603,27 Nov 2020

#246,"'hematologic malignancy'/exp OR 'blood disease, malignant' OR 'haematologic malignancy' OR 'haematological malignancy' OR 'hematologic malignancy' OR 'hematological malignancy' OR 'malignant blood disease' OR 'malignant haematologic disease' OR 'malignant haematological disease' OR 'malignant hematologic disease' OR 'malignant hematological disease'",736917,27 Nov 2020

#245,"'malignant neoplasm'/exp",3827904,27 Nov 2020

#244,"(wbc:ti,ab,kw OR wbc:tt) AND ('leucocyte* count*':ti,ab,kw,de OR 'white blood cell count*':ti,ab,kw,de OR 'white cell count*':ti,ab,kw,de)",4296,27 Nov 2020

#243,"(wcc:ti,ab,kw OR wcc:tt) AND ('leucocyte*' OR 'white cell count*')",555,27 Nov 2020

#242,"'leukocyte count'/exp OR 'wbc count' OR 'wbc counts' OR 'leucocyte count' OR 'leucocyte counting' OR 'leucocyte counts' OR 'leukocyte count' OR 'leukocyte counts' OR 'total leucocyte count' OR 'total leukocyte count' OR 'white blood cell (wbc) count' OR 'white blood cell (wbc) counts' OR 'white blood cell count' OR 'white blood cell counts' OR 'white blood count' OR 'white cell count' OR 'white cell counts'",245408,27 Nov 2020

#241,"'procalcitonin'/de OR 'calcitonin precursor' OR 'procalcitonin'",14531,27 Nov 2020

#240,"'procalcitonin blood level'/de",71,27 Nov 2020

#239,"(esr:ti,ab,kw OR esr:tt) AND 'sedimentation rate$':ti,ab,kw",9272,27 Nov 2020

#238,"'erythrocyte sedimentation rate'/de OR 'blood sedimentation' OR 'blood sedimentation rate' OR 'erythrocyte sedimentation rate' OR 'sedimentation rate, erythrocyte'",54414,27 Nov 2020

#237,"'c reactive protein'/exp OR 'c reactive protein' OR 'c reaction protein' OR 'c-reactive protein' OR 'creactive protein' OR 'crp' OR 'protein, c reactive' OR 'serum c reactive protein'",229346,27 Nov 2020

#236,"'c reactive protein blood level'/de",208,27 Nov 2020

#235,"#233 AND #234",8417,27 Nov 2020

#234,"reactivat*:ti,ab,kw,de OR reactivat*:tt",64862,27 Nov 2020

#233,"#228 OR #229 OR #230 OR #231 OR #232",130624,27 Nov 2020

#232,"(herpes:ti,ab,kw OR 'herpes vulgaris':ti,ab,kw OR 'herpes simplex':ti,ab,kw OR herpes:tt OR 'herpes vulgaris':tt OR 'herpes simplex':tt) AND infect*",49569,27 Nov 2020

#231,"(hsv:ti,ab,kw OR hsv:tt) AND (infect*:ti,ab,kw OR herpes:ti,ab,kw,de)",28999,27 Nov 2020

#230,"'herpes simplex'/exp OR 'herpes' OR 'herpes infection' OR 'herpes simplex' OR 'herpes simplex infection' OR 'herpes simplex viral infection' OR 'herpes simplex virus infection' OR 'herpes vulgaris'",121807,27 Nov 2020

#229,"'herpes zoster':ti,ab,kw,de OR 'varicella zoster':ti,ab,kw,de OR zoster:ti,ab,kw,de OR shingles:ti,ab,kw,de OR 'varicellovirus infect*':ti,ab,kw,de OR 'herpes zoster':tt OR 'varicella zoster':tt OR zoster:tt OR shingles:tt OR 'varicellovirus infect*':tt",41423,27 Nov 2020

#228,"'herpes zoster'/exp",27157,27 Nov 2020

#227,"#225 AND #226",467876,27 Nov 2020

#226,"opportunistic:ti,ab,kw OR serious*:ti,ab,kw OR severe*:ti,ab,kw OR severit*:ti,ab,kw OR opportunistic:tt OR serious*:tt OR severe*:tt OR severit*:tt",2439976,27 Nov 2020

#225,"'infection'/exp",3781783,27 Nov 2020

#224,"fung*:tt OR mycosis:tt OR mycoses:tt OR mycotic:tt OR 'hepatitis b':tt OR 'hepatitis type b':tt OR 'type b hepatitis':tt OR hbv:tt OR ((hepatitis NEAR/3 'b'):tt)",5421,27 Nov 2020

#223,"fung*:ti,ab,kw OR mycosis:ti,ab,kw OR mycoses:ti,ab,kw OR mycotic:ti,ab,kw OR 'hepatitis b':ti,ab,kw OR 'hepatitis type b':ti,ab,kw OR 'type b hepatitis':ti,ab,kw OR hbv:ti,ab,kw OR ((hepatitis NEAR/3 'b'):ti,ab,kw)",411027,27 Nov 2020

#222,"'hepatitis b'/exp OR 'hepatitis b' OR 'hepatitis b virus infection' OR 'hepatitis, serum' OR 'hippie hepatitis' OR 'injection hepatitis' OR 'serum hepatitis' OR 'type b hepatitis' OR 'viral hepatitis type b' OR 'virus hepatitis type b'",167024,27 Nov 2020

#221,"'mycosis'/exp OR 'deep mycosis' OR 'fungal disease' OR 'fungal infection' OR 'fungal infections' OR 'fungus infection' OR 'mycoses' OR 'mycosis' OR 'mycosis infection' OR 'mycotic disease' OR 'mycotic infection'",230639,27 Nov 2020

#220,"'fungus reactivation'",2,27 Nov 2020

#219,"'fungal reactivation'",5,27 Nov 2020

#218,"'atypical mycobacteriosis'/de OR 'mycobacterium avium complex infection' OR 'mycobacterium avium infection' OR 'mycobacterium avium-intracellulare complex infection' OR 'mycobacterium avium-intracellulare infection' OR 'mycobacterium infections, atypical' OR 'mycobacterium intracellulare infection' OR 'mycobacterium kansasii infection' OR 'atypical mycobacterium infection' OR 'atypical mycobacterial infection' OR 'atypical mycobacteriosis' OR 'atypical mycobacterium infections' OR 'atypical tuberculosis' OR 'mycobacteriosis, atypical' OR 'mycobacterium infections, nontuberculous' OR 'nontuberculous mycobacterium infection' OR 'nontuberculous mycobacterium infections'",7007,27 Nov 2020

#217,"(cmv:ti,ab,kw OR cmv:tt) AND (virus:ti,ab,kw OR viral:ti,ab,kw OR infect*:ti,ab,kw)",33950,27 Nov 2020

#216,"'cytomegalovirus infection'/exp OR 'cmv disease' OR 'cmv infection' OR 'cmv infections' OR 'cmv syndrome' OR 'cytomegalovirus disease' OR 'cytomegalovirus infections' OR 'cytomegaly virus disease' OR 'cytomegaly virus infection' OR 'congenital cytomegalic inclusion' OR 'congenital cytomegalic inclusion body disease' OR 'congenital cytomegalic inclusion disease' OR 'cytomegalic inclusion body disease' OR 'cytomegalic inclusion disease' OR 'cytomegaloinfection' OR 'cytomegaloviral infection' OR 'cytomegalovirus infection'",47330,27 Nov 2020

#215,"'tuberculosis':ti,ab,kw OR 'tuberculous':ti,ab,kw OR 'tuberculosis':tt OR 'tuberculous':tt",257191,27 Nov 2020

#214,"'tuberculosis'/exp",269025,27 Nov 2020

#213,"infect*:ti,ab,kw OR infect*:tt",2312204,27 Nov 2020

#212,"((((overall OR rate OR risk$) NEAR/8 (viral* OR virus OR bacterial* OR bacterious OR microbial* OR fungal OR mycos$s OR zoonosis)):ti,ab,kw) OR (((overall OR rate OR risk$) NEAR/3 (viral* OR virus OR bacterial* OR bacterious OR microbial* OR fungal OR mycos$s OR zoonosis)):tt)) AND infect*",51588,27 Nov 2020

#211,"(((overall OR rate OR risk$) NEAR/8 (infect* OR bacter$emi* OR septic$emi* OR sepsis OR 'bacterial infect*' OR 'bacterial diseas*' OR 'bacterious infect*' OR 'viral infect*' OR 'virus diseas*' OR 'virus infect*' OR 'microbial infect*' OR mycos$s OR 'fung* infect*' OR 'mycotic diseas*' OR 'mycos$s infect*' OR fung$emi*)):ti,ab,kw) OR (((overall OR rate OR risk$) NEAR/6 (infect* OR bacter$emi* OR septic$emi* OR sepsis OR 'bacterial infect*' OR 'bacterial diseas*' OR 'bacterious infect*' OR 'viral

infect*' OR 'virus diseas*' OR 'virus infect*' OR 'microbial infect*' OR mycos$s OR 'fung* infect*' OR 'mycotic diseas*' OR 'mycos$s infect*' OR fung$emi*)):tt)",254660,27 Nov 2020

#210,"(((opportunistic* OR opportune) NEAR/6 (infect* OR bacter$emi* OR septic$emi* OR 'bacterial infect*' OR 'bacterial diseas*' OR 'viral infect*' OR 'virus diseas*' OR 'virus infect*' OR 'microbial infect*' OR mycos$s OR 'fung* infect*' OR 'mycotic diseas*' OR 'mycos$s infect*' OR fung$emi*)):ti,ab,kw) OR (((opportunistic* OR opportune) NEAR/6 (infect* OR bacter$emi* OR septic$emi* OR 'bacteri* infect*' OR 'bacteri* diseas*' OR 'viral infect*' OR 'virus diseas*' OR 'virus infect*' OR 'microbial infect*' OR mycos$s OR 'fung* infect*' OR 'mycotic diseas*' OR 'mycos$s infect*' OR fung$emi*)):tt)",28179,27 Nov 2020

#209,"'opportunistic infection'/de",27120,27 Nov 2020

#208,"(((serious* OR severe* OR severit*) NEAR/8 (infect* OR bacter$emi* OR septic$emi* OR sepsis OR fung$emi* OR 'fungal infect*' OR 'fungal disease$' OR mycos$s)):ti,ab,kw) OR (((serious* OR severe* OR severit*) NEAR/8 (infect* OR bacter$emi* OR septic$emi* OR sepsis OR fung$emi* OR 'fungal infect*' OR mycos$s OR 'fungal disease$')):tt)",141705,27 Nov 2020

#207,"'serious infection'/de",11,27 Nov 2020

#206,"'infection rate'/de",31798,27 Nov 2020

#205,"'infection'/exp/mj",2417876,27 Nov 2020

#204,"mortality:ti,ab,kw OR mortality:tt",1157877,27 Nov 2020

#203,"((mortality OR death$) NEAR/4 (rate$ OR ratio OR caus* OR risk OR sudden OR number* OR amount* OR count*)):tt",60,27 Nov 2020

#202,"((mortality OR death$) NEAR/4 (rate$ OR ratio OR caus* OR risk OR sudden OR number* OR amount* OR count*)):ti,ab,kw",796762,27 Nov 2020

#201,"'fatality'/de",90034,27 Nov 2020

#200,"death:ti,ab,kw OR deaths:ti,ab,kw OR death:tt OR deaths:tt",1210269,27 Nov 2020

#199,"'death'/de OR 'death'/exp/mj",386700,27 Nov 2020

#198,"'sudden death'/de",44336,27 Nov 2020

#197,"'mortality'/exp/mj",164359,27 Nov 2020

#196,"'mortality'/de",784077,27 Nov 2020

#195,"'cause of death'/de",117964,27 Nov 2020

#194,"'standardized mortality ratio'/de",3022,27 Nov 2020

#193,"'mortality risk'/de",21965,27 Nov 2020

#192,"'all cause mortality'/de",33351,27 Nov 2020

#191,"'mortality rate'/exp",68389,27 Nov 2020

#190,"((treatment NEAR/3 emergent):ti,ab,kw) OR ((treatment NEAR/3 emergent):tt)",13332,27 Nov 2020

#189,"teratogen*:ti,ab,kw OR teratogen*:tt",23836,27 Nov 2020

#188,"'teratogenicity'/de",17400,27 Nov 2020

#187,"(((drug OR chemical*) NEAR/1 induced):ti,ab,kw) OR (((drug OR chemical*) NEAR/1 induced):tt)",71433,27 Nov 2020

#186,"'drug induced malformation'/de",110,27 Nov 2020

#185,"((toxic* NEAR/6 (drug$ OR medication$)):ti,ab,kw) OR ((toxic* NEAR/6 (drug$ OR medication$)):tt)",53083,27 Nov 2020

#184,"'drug toxicity and intoxication'/exp",178224,27 Nov 2020

#183,"'anaphyla*':ti,ab,kw OR anaphyla*:tt",43461,27 Nov 2020

#182,"'anaphylaxis'/exp",54207,27 Nov 2020

#181,"hypersensitiv*:ti,ab,kw OR 'hyper sensitiv*':ti,ab,kw OR hypersensitiv*:tt OR 'hyper sensitiv*':tt",101554,27 Nov 2020

#180,"'drug hypersensitivity'/exp",62723,27 Nov 2020

#179,"'side effect$':ti,ab,kw OR 'side effect$':tt",378161,27 Nov 2020

#178,"'side effect'/exp",574650,27 Nov 2020

#177,"safety:ti,ab,kw OR safety:tt",826797,27 Nov 2020

#176,"'drug safety'/exp",423068,27 Nov 2020

#175,"withdraw*:ti,ab,kw OR withdraw*:tt",181375,27 Nov 2020

#174,"'drug withdrawal'/de",202175,27 Nov 2020

#173,"adverseevent*:ti,ab,kw OR adversedrugreaction*:ti,ab,kw OR adversereaction*:ti,ab,kw OR adverseincident*:ti,ab,kw OR adverseoutcome*:ti,ab,kw",1173,27 Nov 2020

#172,"(saes:tt OR aes:tt OR adrs:tt OR sae:tt OR 'ae':tt OR adr:tt) AND adverse:ti,ab,kw,de",14,27 Nov 2020

#171,"(saes:ti,ab,kw OR aes:ti,ab,kw OR adrs:ti,ab,kw OR sae:ti,ab,kw OR 'ae':ti,ab,kw OR adr:ti,ab,kw) AND adverse:ti,ab,kw,de",55573,27 Nov 2020

#170,"(((serious* OR severe* OR severit*) NEAR/3 (adverse OR saes OR aes OR adrs OR sae OR 'ae' OR adr)):ti,ab,kw) OR (((serious* OR severe* OR severit*) NEAR/3 (adverse OR saes OR aes OR adrs OR sae OR 'ae' OR adr)):tt)",84186,27 Nov 2020

#169,"(adverse NEAR/3 (event$ OR effect$ OR reaction$ OR incident$ OR outcome$)):tt",53,27 Nov 2020

#168,"(adverse NEAR/3 (event$ OR effect$ OR reaction$ OR incident$ OR outcome$)):ti,ab,kw",720474,27 Nov 2020

#167,"'adverse event'/de OR 'adverse drug reaction'/exp",617132,27 Nov 2020

#166,"'adverse drug reaction'/lnk OR 'complication'/lnk OR 'drug toxicity'/lnk OR 'side effect'/lnk",3550888,27 Nov 2020

#165,"#87 OR #164",93537,27 Nov 2020

#164,"#76 AND #163",92785,27 Nov 2020

#163,"#88 OR #89 OR #90 OR #91 OR #92 OR #93 OR #94 OR #95 OR #96 OR #97 OR #98 OR #99 OR #100 OR #101 OR #102 OR #103 OR #104 OR #105 OR #106 OR #107 OR #108 OR #109 OR #110 OR #111 OR #112 OR #113 OR #114 OR #115 OR #116 OR #117 OR #118 OR #119 OR #120 OR #121 OR #122 OR #123 OR #124 OR #125 OR #126 OR #127 OR #128 OR #129 OR #130 OR #131 OR #132 OR #133 OR #134 OR #135 OR #136 OR #137 OR #138 OR #139 OR #140 OR #141 OR #142 OR #143 OR #144 OR #145 OR #146 OR #147 OR #148 OR #149 OR #150 OR #151 OR #152 OR #153 OR #154 OR #155 OR #156 OR #157 OR #158 OR #159 OR #160 OR #161 OR #162",1462236,27 Nov 2020

#162,"'peficitinib'/de OR '4 [(5 hydroxyadamantan 2 yl) amino] 1h pyrrolo [2, 3 b] pyridine 5 carboxamide' OR '4 [(5 hydroxytricyclo [3.3.1.1 3, 7] dec 2 yl) amino] 1h pyrrolo [2, 3 b] pyridine 5 carboxamide' OR 'asp 015k' OR 'asp015k' OR 'peficitinib' OR 'peficitinib hydrobromide'",186,27 Nov 2020

#161,"'ritlecitinib'/exp OR '1 [2 methyl 5 (7h pyrrolo [2, 3 d] pyrimidin 4 ylamino) 1 piperidinyl] prop 2 en 1 one' OR '1 [2 methyl 5 (7h pyrrolo [2, 3 d] pyrimidin 4 ylamino) piperidin 1 yl] prop 2 en 1 one' OR '1 [2 methyl 5 [(7h pyrrolo [2, 3 d] pyrimidin 4 yl) amino] 1 piperidinyl] prop 2 en 1 one' OR '1 [2 methyl 5 [(7h pyrrolo [2, 3 d] pyrimidin 4 yl) amino] piperidin 1 yl] prop 2 en 1 one' OR '1 [5 (7h pyrrolo [2, 3 d]

pyrimidin 4 ylamino) 2 methyl 1 piperidinyl] prop 2 en 1 one' OR '1 [5 (7h pyrrolo [2, 3 d] pyrimidin 4 ylamino) 2 methylpiperidin 1 yl] prop 2 en 1 one' OR '1 [5 [(7h pyrrolo [2, 3 d] pyrimidin 4 yl) amino] 2 methyl 1 piperidinyl] prop 2 en 1 one' OR '1 [5 [(7h pyrrolo [2, 3 d] pyrimidin 4 yl) amino] 2 methylpiperidin 1 yl] prop 2 en 1 one' OR 'pf 06651600' OR 'pf 6651600' OR 'pf06651600' OR 'pf6651600' OR 'ritlecitinib' OR 'ritlecitinib malonate' OR 'ritlecitinib propanedioate'",40,27 Nov 2020
#160,"'fedratinib'/de OR 'fedratinib' OR 'fedratinib dihydrochloride' OR 'fedratinib dihydrochloride monohydrate' OR 'fedratinib hydrochloride' OR 'inrebic' OR 'n (1, 1 dimethylethyl) 3 [[5 methyl 2 [[4 [2 (1 pyrrolidinyl) ethoxy] phenyl] amino] 4 pyrimidinyl] amino] benzenesulfonamide' OR 'n tert butyl 3 [[5 methyl 2 [[4 (2 pyrrolidin 1 ylethoxy) phenyl] amino] pyrimidin 4 yl] amino] benzenesulfonamide' OR 'n tert butyl 3 [[5 methyl 2 [4 [2 (pyrrolidin 1 yl) ethoxy] anilino] pyrimidin 4 yl] amino] benzenesulfonamide' OR 'n tert butyl 3 [5 methyl 2 [4 (2 pyrrolidin 1 ylethoxy) phenylamino] pyrimidin 4 ylamino] benzenesulfonamide' OR 'n tert butyl 3 [5 methyl 2 [4 [2 (1 pyrrolidinyl) ethoxy] phenylamino] 4 pyrimidinylamino] benzenesulfonamide' OR 'sar 302503' OR 'sar 302503a' OR 'sar302503' OR 'sar302503a' OR 'tg 101348' OR 'tg101348'",653,27 Nov 2020
#159,"'n benzyl 2 cyano 3 (3,4 dihydroxyphenyl)acrylamide'/exp OR '2 cyano 3 (3, 4 dihydroxyphenyl) n (phenylmethyl) 2 propenamide' OR 'ag 490' OR 'ag490' OR 'n benzyl 2 cyano 3 (3,4 dihydroxyphenyl)acrylamide' OR 'tyrphostin ag 490' OR 'tyrphostin ag490'",2477,27 Nov 2020
#158,"'itacitinib'/de OR '1 [1 [[3 fluoro 2 (trifluoromethyl) 4 pyridinyl] carbonyl] 4 piperidinyl] 3 [4 (7h pyrrolo [2, 3 d] pyrimidin 4 yl) 1h pyrazol 1 yl] 3 azetidineacetonitrile' OR '1 [1 [[3 fluoro 2 (trifluoromethyl) pyridin 4 yl] carbonyl] piperidin 4 yl] 3 [4 (7h pyrrolo [2, 3 d] pyrimidin 4 yl) 1h pyrazol 1 yl] 3 azetidineacetonitrile' OR '[1 [1 (3 fluoro 2 trifluoromethylpyridine 4 carbonyl) 4 piperidinyl] 3 [4 (7h pyrrolo [2, 3 d] pyrimidin 4 yl) 1h pyrazol 1 yl] 3 azetidinyl] ethanenitrile' OR '[1 [1 [3 fluoro 2

(trifluoromethyl) pyridine 4 carbonyl] piperidin 4 yl] 3 [4 (7h pyrrolo [2, 3 d] pyrimidin 4 yl) 1h pyrazol 1 yl] azetidin 3 yl] ethanenitrile' OR 'incb 039110' OR 'incb 39110' OR 'incb039110' OR 'incb39110' OR 'itacitinib' OR 'itacitinib adipate' OR 'itacitinib hexanedioate'",183,27 Nov 2020

#157,"'ruxolitinib'/de OR '3 [4 (7h pyrrolo [2, 3 d] pyrimidin 4 yl) 1h pyrazol 1 yl] 3 cyclopentylpropanenitrile' OR '3 [4 (7h pyrrolo [2, 3 d] pyrimidin 4 yl) 1h pyrazol 1 yl] 3 cyclopentylpropanenitrile phosphate' OR '3 cyclopentyl 3 [4 (7h pyrrolo [2, 3 d] pyrimidin 4 yl) 1h pyrazol 1 yl] propanenitrile' OR 'beta cyclopentyl 4 (7h pyrrolo [2, 3 d] pyrimidin 4 yl) 1h pyrazole 1 propanenitrile' OR 'incb 018424' OR 'incb 18424' OR 'incb 424' OR 'incb018424' OR 'incb18424' OR 'incb424' OR 'jakafi' OR 'jakavi' OR 'ruxolitinib' OR 'ruxolitinib maleate' OR 'ruxolitinib phosphate'",5351,27 Nov 2020

#156,"'upadacitinib'/de OR '3 ethyl 4 (3h imidazo [1, 2 a] pyrrolo [2, 3 e] pyrazin 8 yl) n (2, 2, 2 trifluoroethyl) 1 pyrrolidinecarboxamide' OR '3 ethyl 4 (3h imidazo [1, 2 a] pyrrolo [2, 3 e] pyrazin 8 yl) n (2, 2, 2 trifluoroethyl) 1 pyrrolidinecarboxamide 2, 3 dihydroxybutanedioate' OR '3 ethyl 4 (3h imidazo [1, 2 a] pyrrolo [2, 3 e] pyrazin 8 yl) n (2, 2, 2 trifluoroethyl) 1 pyrrolidinecarboxamide tartrate' OR '3 ethyl 4 (3h imidazo [1, 2 a] pyrrolo [2, 3 e] pyrazin 8 yl) n (2, 2, 2 trifluoroethyl) pyrrolidine 1 carboxamide' OR '3 ethyl 4 (3h imidazo [1, 2 a] pyrrolo [2, 3 e] pyrazin 8 yl) n (2, 2, 2 trifluoroethyl) pyrrolidine 1 carboxamide 2, 3 dihydroxybutanedioate' OR '3 ethyl 4 (3h imidazo [1, 2 a] pyrrolo [2, 3 e] pyrazin 8 yl) n (2, 2, 2 trifluoroethyl) pyrrolidine 1 carboxamide tartrate' OR 'abt 494' OR 'abt494' OR 'rinvoq' OR 'upadacitinib' OR 'upadacitinib 2, 3 dihydroxybutanedioate' OR 'upadacitinib hemihydrate' OR 'upadacitinib hydrate' OR 'upadacitinib tartrate'",553,27 Nov 2020

#155,"'filgotinib'/de OR 'filgotinib' OR 'filgotinib 2 butenedioate' OR 'filgotinib hydrochloride' OR 'filgotinib maleate' OR 'g 146034' OR 'g 146034 101' OR 'g 146034-101' OR 'g146034' OR 'g146034 101'

OR 'g146034-101' OR 'glpg 0634' OR 'glpg0634' OR 'gs 6034' OR 'gs6034' OR 'n [5 [4 (1, 1 dioxothiomorpholinomethyl) phenyl] 1, 2, 4 triazolo [1, 5 a] pyridin 2 yl] cyclopropanecarboxamide' OR 'n [5 [4 (1, 1 dioxothiomorpholinomethyl) phenyl] 1, 2, 4 triazolo [1, 5 a] pyridin 2 yl] cyclopropanecarboxamide 2 butenedioate' OR 'n [5 [4 (1, 1 dioxothiomorpholinomethyl) phenyl] 1, 2, 4 triazolo [1, 5 a] pyridin 2 yl] cyclopropanecarboxamide but 2 enedioate' OR 'n [5 [4 [(1, 1 dioxido 4 thiomorpholinyl) methyl] phenyl] 1, 2, 4 triazolo [1, 5 a] pyridin 2 yl] cyclopropanecarboxamide' OR 'n [5 [4 [(1, 1 dioxido 4 thiomorpholinyl) methyl] phenyl] 1, 2, 4 triazolo [1, 5 a] pyridin 2 yl] cyclopropanecarboxamide 2 butenedioate' OR 'n [5 [4 [(1, 1 dioxothiomorpholin 4 yl) methyl] phenyl] 1, 2, 4 triazolo [1, 5 a] pyridin 2 yl] cyclopropanecarboxamide' OR 'n [5 [4 [(1, 1 dioxothiomorpholin 4 yl) methyl] phenyl] 1, 2, 4 triazolo [1, 5 a] pyridin 2 yl] cyclopropanecarboxamide but 2 enedioate' OR 'n [5 [4 [(1, 1 dioxothiomorpholin 4 yl) methyl] phenyl] [1, 2, 4] triazolo [1, 5 a] pyridin 2 yl] cyclopropanecarboxamide' OR 'n [5 [4 [(1, 1 dioxothiomorpholin 4 yl) methyl] phenyl] [1, 2, 4] triazolo [1, 5 a] pyridin 2 yl] cyclopropanecarboxamide but 2 enedioate'",506,27 Nov 2020

#154,"'tofacitinib'/de OR '1 cyanoacetyl 4 methyl n methyl n (1h pyrrolo [2, 3 d] pyrimidin 4 yl) 3 piperidinamine' OR '3 [4 methyl 3 [methyl (7h pyrrolo [2, 3 d] pyrimidin 4 yl) amino] 1 piperidinyl] 3 oxopropanenitrile' OR '4 [n [1 (2 cyano 1 oxoethyl) 4 methyl 3 piperidinyl] n methylamino] pyrrolo [2, 3 d] pyrimidine' OR '4 methyl 3 [methyl (7h pyrrolo [2, 3 d] pyrimidin 4 yl) amino] beta oxo 1 piperidinepropanenitrile' OR 'cp 690 550' OR 'cp 690, 550' OR 'cp 690550' OR 'cp 690550 10' OR 'cp 690550-10' OR 'cp690 550' OR 'cp690, 550' OR 'cp690550' OR 'cp690550 10' OR 'cp690550-10' OR 'tasocitinib' OR 'tasocitinib citrate' OR 'tofacitinib' OR 'tofacitinib citrate' OR 'xeljanz' OR 'xeljanz xr'",5261,27 Nov 2020

#153,"'baricitinib'/de OR '1 (ethylsulfonyl) 3 [4 (7h pyrrolo [2, 3 d] pyrimidin 4 yl) 1h pyrazol 1 yl] 3 azetidineacetonitrile' OR '[1 (ethanesulfonyl) 3 [4 (7h pyrrolo [2, 3 d] pyrimidin 4 yl) 1h pyrazol 1 yl] azetidin 3 yl] ethanenitrile' OR '[1 (ethylsulfonyl) 3 [4 (1h pyrrolo [2, 3 d] pyrimidin 4 yl) 1h pyrazol 1 yl] 3 azetidinyl] acetonitrile' OR '[1 (ethylsulfonyl) 3 [4 (7h pyrrolo [2, 3 d] pyrimidin 4 yl) 1h pyrazol 1 yl] azetidin 3 yl] ethanenitrile' OR 'baricitinib' OR 'incb 028050' OR 'incb 28050' OR 'incb028050' OR 'incb28050' OR 'ly 3009104' OR 'ly3009104' OR 'olumiant'",1543,27 Nov 2020

#152,"(janus NEAR/4 kinase NEAR/10 inhibitor$):tt",4,27 Nov 2020

#151,"(janus NEAR/4 kinase NEAR/10 inhibitor$):ti,ab,kw,tn",3319,27 Nov 2020

#150,"((jak OR jak1 OR jak2 OR jak3 OR 'janus kinase') NEAR/10 inhibitor*):tt",22,27 Nov 2020

#149,"((jak OR jak1 OR jak2 OR jak3 OR 'janus kinase') NEAR/10 inhibitor*):ti,ab,kw,tn",11251,27 Nov 2020

#148,"'janus kinase inhibitor'/exp OR 'jak inhibitor' OR 'janus kinase inhibitor' OR 'janus kinase inhibitors' OR 'janus tyrosine kinase inhibitor'",16188,27 Nov 2020

#147,"(steroid$ NEAR/10 (therap* OR treat* OR drug$ OR inject* OR intramuscular* OR 'intra muscular*' OR im OR intravenous* OR 'intra venous*' OR iv OR intraarticular* OR 'intra articular*' OR ia OR administrat* OR oral OR orally OR parenteral*)):tt",148,27 Nov 2020

#146,"(steroid$ NEAR/10 (therap* OR treat* OR drug$ OR inject* OR intramuscular* OR 'intra muscular*' OR im OR intravenous* OR 'intra venous*' OR iv OR intraarticular* OR 'intra articular*' OR ia OR administrat* OR oral OR orally OR parenteral*)):ti,ab,kw",118206,27 Nov 2020

#145,"'triamcinolone'/exp OR '9alpha fluoro 1, 4 pregnadiene 3, 20 dione 11beta, 16alpha, 17alpha, 21 tetrol' OR '9alpha fluoro 11beta, 16alpha, 17alpha, 21 tetrahydroxypregna 1, 4 diene 3, 20 dione' OR '9alpha fluoro 16alpha hydroxyhydrocortisone' OR '9alpha fluoro 16alpha hydroxyprednisolone' OR

'acetocot' OR 'adcortyl' OR 'aristocort' OR 'aristocort forte' OR 'aristodan' OR 'azmacor' OR 'celeste' OR 'cl 19823' OR 'cl19823' OR 'clinacort' OR 'clinalog' OR 'delphicort' OR 'fluoxiprednisolone' OR 'fluoxyprednisolone' OR 'ken-jec 40' OR 'kenacort' OR 'kenacort retard' OR 'korticoid' OR 'ledercort' OR 'omcilon' OR 'polcortolon' OR 'rp 8357' OR 'rp8357' OR 'simacort' OR 'sterocort' OR 'tac 3' OR 'tramcinolone' OR 'triacortyl' OR 'triam-a' OR 'triam-forte' OR 'triamcinolon' OR 'triamcinolona' OR 'triamcinolone*' OR 'triamcort' OR 'triamcot' OR 'triamonide 40' OR 'triamsicort' OR 'triancinolon' OR 'u-tri-lone' OR 'volon'",39582,27 Nov 2020

#144,"'beclometasone'/exp OR '9 chloro 11beta, 17, 21 trihydroxy 16beta methylpregna 1, 4 diene 3, 20 dione' OR '9alpha chloro 16beta methylprednisolone' OR 'beclometasone' OR 'beclomethasone' OR 'prednisolone, 9alpha chloro 16beta methyl' OR 'pregna 1, 4 diene 11beta, 17, 21 triol 3, 20 dione, 9 chloro 16beta methyl'",15095,27 Nov 2020

#143,"'dexamethasone'/de OR '16alpha methyl 9alpha fluoroprednisolone' OR '9 alpha fluoro 16 alpha methyl delta corticosterone' OR '9alpha fluoro 11beta, 17alpha, 21 trihydroxy 16alpha methyl 1, 4 pregnadiene 3, 20 dione' OR '9alpha fluoro 11beta, 17alpha, 21 trihydroxy 16alpha methylpregna 1, 4 diene 3, 20 dione' OR '9alpha fluoro 16alpha methyl delta corticosterone' OR 'adrecort' OR 'adrenocot' OR 'aeroseb dex' OR 'aeroseb-dex' OR 'aflucoson' OR 'aflucosone' OR 'alfalyl' OR 'anaflogistico' OR 'anaflogistico novobios' OR 'arcodexan' OR 'arcodexane' OR 'artrosone' OR 'azium' OR 'bidexol' OR 'calonat' OR 'cebedex' OR 'cetadexon' OR 'colofoam' OR 'corsona' OR 'cortastat' OR 'cortastat 10' OR 'cortastat la' OR 'cortidex' OR 'cortidexason' OR 'cortidrona' OR 'cortidrone' OR 'cortisumman' OR 'dacortina fuerte' OR 'dacortine fuerte' OR 'dalalone' OR 'dalalone d.p.' OR 'dalalone l.a.' OR 'danasone' OR 'de-sone la' OR 'decacortin' OR 'decadeltosona' OR 'decadeltosone' OR 'decaderm' OR 'decadion' OR 'decadran' OR 'decadron' OR 'decadron 5-12 pak' OR 'decadron la' OR 'decadronal' OR 'decadrone' OR

'decaesadril' OR 'decaject' OR 'decamethasone' OR 'decasone' OR 'decaspray' OR 'decasterolone' OR 'decdan' OR 'decilone' OR 'decilone forte' OR 'decofluor' OR 'dectancyl' OR 'dekacort' OR 'delladec' OR 'deltafluoren' OR 'deltafluorene' OR 'dergramin' OR 'deronil' OR 'desacort' OR 'desacortone' OR 'desadrene' OR 'desalark' OR 'desameton' OR 'desametone' OR 'desigdron' OR 'dexa cortisyl' OR 'dexa dabrosan' OR 'dexa korti' OR 'dexa scherosan' OR 'dexa scherozon' OR 'dexa scherozone' OR 'dexa-p' OR 'dexacen 4' OR 'dexacen-4' OR 'dexachel' OR 'dexacort' OR 'dexacortal' OR 'dexacorten' OR 'dexacortin' OR 'dexacortisyl' OR 'dexadabroson' OR 'dexadecadrol' OR 'dexadrol' OR 'dexagel' OR 'dexagen' OR 'dexahelvacort' OR 'dexakorti' OR 'dexalien' OR 'dexalocal' OR 'dexame' OR 'dexamecortin' OR 'dexameson' OR 'dexamesone' OR 'dexametason' OR 'dexametasone' OR 'dexameth' OR 'dexamethason' OR 'dexamethasone' OR 'dexamethasone alcohol' OR 'dexamethasone intensol' OR 'dexamethazon' OR 'dexamethazone' OR 'dexamethonium' OR 'dexamonozon' OR 'dexan' OR 'dexane' OR 'dexano' OR 'dexapot' OR 'dexascheroson' OR 'dexascherozon' OR 'dexascherozone' OR 'dexason' OR 'dexasone' OR 'dexasone la' OR 'dexasone s' OR 'dexinoral' OR 'dexionil' OR 'dexmethsone' OR 'dexona' OR 'dexone' OR 'dexone 0.5' OR 'dexone 0.75' OR 'dexone 1.5' OR 'dexone 4' OR 'dexpak taperpak' OR 'dextelan' OR 'dextenza' OR 'dextrasone' OR 'dexycu' OR 'dezone' OR 'dibasona' OR 'doxamethasone' OR 'esacortene' OR 'ex s1' OR 'exadion' OR 'exadione' OR 'firmalone' OR 'fluormethyl prednisolone' OR 'fluormethylprednisolon' OR 'fluormethylprednisolone' OR 'fluormone' OR 'fluorocort' OR 'fluorodelta' OR 'fluoromethylprednisolone' OR 'fortecortin' OR 'gammacorten' OR 'gammacortene' OR 'grosodexon' OR 'grosodexone' OR 'hemady' OR 'hexadecadiol' OR 'hexadecadrol' OR 'hexadiol' OR 'hexadrol' OR 'isnacort' OR 'isopto dex' OR 'isopto maxidex' OR 'isopto-dex' OR 'isopto-maxidex' OR 'isoptodex' OR 'isoptomaxidex' OR 'lokalison f' OR 'loverine' OR 'luxazone' OR 'marvidione' OR 'maxidex' OR 'mediamethasone' OR 'megacortin' OR 'mephameson' OR 'mephamesone' OR 'metasolon' OR

'metasolone' OR 'methazon ion' OR 'methazone ion' OR 'methazonion' OR 'methazonione' OR 'metisone lafi' OR 'mexasone' OR 'millicorten' OR 'millicortenol' OR 'mk 125' OR 'mk125' OR 'mymethasone' OR 'neoforderx' OR 'neofordex' OR 'nisomethasona' OR 'novocort' OR 'nsc 34521' OR 'nsc34521' OR 'oftan-dexa' OR 'opticorten' OR 'opticortinol' OR 'oradexan' OR 'oradexon' OR 'oradexone' OR 'orgadrone' OR 'ozurdex' OR 'pidexon' OR 'policort' OR 'posurdex' OR 'predni f tablinen' OR 'predni-f' OR 'prednisolone f' OR 'prodexona' OR 'prodexone' OR 'sanamethasone' OR 'santenson' OR 'santeson' OR 'sawasone' OR 'solurex' OR 'solurex la' OR 'spoloven' OR 'sterasone' OR 'thilodexine' OR 'triamcimetil' OR 'vexamet' OR 'visumetazone' OR 'visumethazone'",171141,27 Nov 2020
#142,"'hydrocortisone'/de OR '11beta, 17, 21 trihydroxypregn 4 ene 3, 20 dione' OR '11beta, 17alpha, 21 trihydroxypregn 4 ene 3, 20 dione' OR '17 hydroxycorticosterone' OR '4 pregnene 11beta, 17alpha, 21 triol 3, 20 dione' OR '4 pregnene 3, 20 dione n beta, 17alpha, 21 triol' OR 'acticort' OR 'acticort 100' OR 'aeroseb hc' OR 'aeroseb-hc' OR 'ala-cort' OR 'ala-scalp' OR 'ala-scalp hp' OR 'alfacort' OR 'algicortis' OR 'alkindi' OR 'alpha derm' OR 'alphaderm' OR 'anucort-hc' OR 'anumed-hc' OR 'anutone-hc' OR 'aquanil hc' OR 'balneol-hc' OR 'barseb hc' OR 'beta-hc' OR 'biacort' OR 'cetacort' OR 'cobadex' OR 'colocort' OR 'compound f' OR 'cordicare lotion' OR 'coripen' OR 'cort dome' OR 'cort-dome' OR 'cort-dome high potency' OR 'cortef' OR 'cortef cream' OR 'cortenema' OR 'cortibel' OR 'corticorenol' OR 'cortifan' OR 'cortiphate' OR 'cortisol' OR 'cortisole' OR 'cortispray' OR 'cortoderm' OR 'cortril' OR 'cotacort' OR 'covocort' OR 'cremicort-h' OR 'cutaderm' OR 'derm-aid cream' OR 'dermacrin hc lotion' OR 'dermaid' OR 'dermaid soft cream' OR 'dermocare' OR 'dermocortal' OR 'dermolate' OR 'dioderm' OR 'eczacort' OR 'ef cortelan' OR 'efcortelan' OR 'egocort' OR 'egocort cream' OR 'eksalb' OR 'eldecort' OR 'emo-cort' OR 'epicort' OR 'ficortril' OR 'filocot' OR 'flexicort' OR 'gly-cort' OR 'glycort' OR 'h-cort' OR 'hc (hydrocortisone)' OR 'hc no. 1' OR 'hc no. 4' OR 'hebcort' OR 'hebcort v' OR 'hemorrhoidal hc' OR

'hemril-30' OR 'hemril-hc uniserts' OR 'hi-cor' OR 'hidrotisona' OR 'hycor' OR 'hycort' OR 'hydracort' OR 'hydrasson' OR 'hydro ricortex' OR 'hydro-rx' OR 'hydrocort' OR 'hydrocorticosteroid' OR 'hydrocortisate' OR 'hydrocortison' OR 'hydrocortisone' OR 'hydrocortisone 1% in absorbase' OR 'hydrocortisone acetonide' OR 'hydrocortisone astier' OR 'hydrocortisone in absorbase' OR 'hydrocortisone ointment' OR 'hydrocortisone plus saline' OR 'hydrocortisone steroid' OR 'hydrocortisone, topical' OR 'hydrocortisonum' OR 'hydrocortisyl' OR 'hydrocortone' OR 'hydrogalen' OR 'hydrokort' OR 'hydrokortison' OR 'hydrotopic' OR 'hysone' OR 'hytisone' OR 'hytone' OR 'hytone lotion' OR 'incortin h' OR 'instacort 10' OR 'kyypakkaus' OR 'lacticare hc' OR 'lacticare-hc' OR 'lemnis fatty cream hc' OR 'lenirit' OR 'medihaler cort' OR 'medihaler duo' OR 'medrocil' OR 'mildison' OR 'mildison fet krem' OR 'mildison lipocream' OR 'mildison-fatty' OR 'mitocortyl demangeaisons' OR 'munitren' OR 'nogenic hc' OR 'novohydrocort' OR 'nsc 10483' OR 'nsc 741' OR 'nsc10483' OR 'nutracort' OR 'optef' OR 'otosone f' OR 'penecort' OR 'plenadren' OR 'prepcort' OR 'prevex hc' OR 'pro cort' OR 'procort' OR 'procto-kit 1%' OR 'procto-kit 2.5%' OR 'proctocort' OR 'proctosert hc' OR 'proctosol-hc' OR 'proctosone' OR 'proctozone hc' OR 'procutan' OR 'rectasol-hc' OR 'rectocort' OR 'rederm' OR 'sanatison' OR 'scalp-aid' OR 'schericur' OR 'schericur 0.25%' OR 'scherosone f' OR 'sistral hydrocort' OR 'skincalm' OR 'stie-cort' OR 'substance m' OR 'synacort' OR 'texacort' OR 'triburon-hc' OR 'unicort' OR 'vasocort'",165843,27 Nov 2020

#141,"'corticosterone'/de OR '11 beta, 21 dihydroxy pregn 4 ene 3, 20 dione' OR '11beta, 21 dihydroxy 4 pregnene 3, 20 dione' OR '11beta, 21 dihydroxypregn 4 ene 3, 20 dione' OR '4 pregnene 11beta, 21 diol 3, 20 dione' OR 'compound b' OR 'corticosterone*' OR 'corticosterone function' OR 'corticosterone response' OR 'kendall compound b' OR 'nsc 9705' OR 'nsc9705' OR 'pregn 4 ene 11beta, 21 diol 3, 20 dione' OR 'reichstein substance h'",45705,27 Nov 2020

#140,"'glucocorticoid'/exp OR 'glucocorticoid*' OR 'glucocorticoid drug' OR 'glucocorticoid hormone' OR 'glucocorticoid steroid' OR 'glucocorticoids' OR 'glucocorticoids, synthetic' OR 'glucocorticoids, topical' OR 'glucocorticoidsteroid' OR 'glucocorticosteroid' OR 'glucocortoid' OR 'glycocorticoid' OR 'glycocorticosteroid'",797162,27 Nov 2020

#139,"'methylprednisolone'/de OR '11beta, 17alpha, 21 trihydroxy 6alpha methyl 1, 4 pregnadiene 3, 20 dione' OR '2 methylprednisolone' OR '5 methylprednisolone' OR '6 alpha methylprednisolone' OR '6 methyl delta 1 hydrocortisone' OR '6 methyl prednisolone' OR '6 methylprednisolone' OR '6alpha methyl delta1 hydrocortisone' OR '6alpha methylprednisolone' OR 'adlone-40' OR 'adlone-80' OR 'beta methylprednisolone' OR 'dep medalone 80' OR 'depmedalone' OR 'depoject-80' OR 'depopred' OR 'esametone' OR 'firmacort' OR 'med-jec-40' OR 'medixon' OR 'mednin' OR 'medralone 80' OR 'medrate' OR 'medrol' OR 'medrol a' OR 'medrol adt pak' OR 'medrol compositum' OR 'medrol dosepak' OR 'medrol medules' OR 'medrol pak' OR 'medrone' OR 'meprednisolone' OR 'meprelon' OR 'mesopren' OR 'methacort 40' OR 'methacort 80' OR 'methyl prednisolone' OR 'methylcotol' OR 'methylcotolone' OR 'methylpred dp' OR 'methylprednisolone*' OR 'methylsterolone' OR 'metidrol' OR 'metrisone' OR 'metycortin' OR 'metypred' OR 'metypresol' OR 'neomedrone' OR 'nsc 19987' OR 'nsc19987' OR 'prednol' OR 'solomet' OR 'solu decortin' OR 'urbason' OR '83 43 2':rn",109222,27 Nov 2020

#138,"'cortisone'/de OR '11 dehydro 17 hydroxy corticosterone' OR '11 dehydro 17 hydroxycorticosterone' OR '17 hydroxy 11 dehydrocortisone' OR '17alpha, 21 dihydroxy 4 pregnene 3, 11, 20 trione' OR '4 pregnene 17alpha, 21 diol 3, 11, 20 trione' OR 'adrenalex' OR 'compound e (kendall)' OR 'corlin' OR 'cortadren' OR 'cortagen' OR 'cortandren' OR 'cortane' OR 'cortisal' OR 'cortisate' OR 'cortison' OR 'cortisone$' OR 'cortisone sodium' OR 'cortistal' OR 'cortivite' OR 'cortogen' OR 'cortone' OR 'delta4 pregnene 17alpha, 21 diol 3, 11, 20 trione' OR 'incorlin' OR 'incortin' OR

'kendall compound e' OR 'nsc 9703' OR 'nsc9703' OR 'pregn 4 en 17 alpha, 21 diol 3, 11, 20 trione' OR 'pregn 4 ene 17alpha, 21 diol 3, 11, 20 trione' OR 'reichstein substance fa' OR 'ricortex' OR 'scheroson' OR 'scherosone' OR 'wintersteiner compound f'",32707,27 Nov 2020

#137,"'cortisone therapeutic use'/de",3404,27 Nov 2020

#136,"'prednisone'/de OR '1, 2 dehydrocortisone' OR '17, 21 dihydroxypregna 1, 4 diene 3, 11, 20 trione' OR 'ancortone' OR 'apo-prednisone' OR 'biocortone' OR 'colisone' OR 'cortan' OR 'cortidelt' OR 'cortiprex' OR 'cutason' OR 'dacorten' OR 'de cortisyl' OR 'decortancyl' OR 'decortin' OR 'decortin e merck' OR 'decortine' OR 'decortisyl' OR 'dehydrocortisone' OR 'dekortin' OR 'delitisone' OR 'dellacort a' OR 'delta 1 dehydrocortisone' OR 'delta cortelan' OR 'delta cortisone' OR 'delta dome' OR 'delta e' OR 'delta prenovis' OR 'delta-dome' OR 'deltacorten' OR 'deltacortene' OR 'deltacortisone' OR 'deltacortone' OR 'deltasone' OR 'deltison' OR 'deltisona' OR 'deltra' OR 'di adreson' OR 'di-adreson' OR 'diadreson' OR 'drazone' OR 'encorton' OR 'encortone' OR 'enkorton' OR 'fernisone' OR 'hostacortin' OR 'insone' OR 'liquid pred' OR 'lodotra' OR 'me-korti' OR 'meprison' OR 'metacortandracin' OR 'meticorten' OR 'meticortine' OR 'nisona' OR 'nsc 10023' OR 'nsc10023' OR 'orasone' OR 'orisane' OR 'panafcort' OR 'paracort' OR 'pehacort' OR 'precort' OR 'precortal' OR 'prednicen-m' OR 'prednicorm' OR 'prednicot' OR 'prednidib' OR 'prednison' OR 'prednisone*' OR 'prednisone alcohol' OR 'prednisone intensol' OR 'prednisone test' OR 'prednitone' OR 'pregna 1, 4 diene 3, 11, 20 trione 17, 21 diol' OR 'pronison' OR 'pronisone' OR 'pronizone' OR 'pulmison' OR 'rayos' OR 'rectodelt' OR 'servisone' OR 'steerometz' OR 'sterapred' OR 'sterapred ds' OR 'ultracorten' OR 'urtilone' OR 'winpred'",187259,27 Nov 2020

#135,"'prednisolone'/de OR '1, 2 dehydro hydrocortison' OR '1, 4 pregnadien 11beta, 17alpha, 21 triol 3, 20 dion' OR '1, 4 pregnadiene 11beta, 17alpha, 21 triol 3, 20 dione' OR '1, 4 pregnadiene 3, 20 dione

11beta, 17alpha, 21 triol' OR '11beta, 17, 21 trihydroxypregna 1, 4 diene 3, 20 dione' OR '11beta, 17alpha, 21 trihydroxy 1, 4 pregnadien 3, 20 dion' OR '11beta, 17alpha, 21 trihydroxypregna 1, 4 diene 3, 20 dione' OR '3, 20 dioxo 11beta, 17alpha, 21 trihydroxy 1, 4 pregnadiene' OR 'adelcort' OR 'antisolon' OR 'antisolone' OR 'aprednislon' OR 'aprednislone' OR 'benisolon' OR 'benisolone' OR 'berisolon' OR 'berisolone' OR 'caberdelta' OR 'capsoid' OR 'co hydeltra' OR 'codelcortone' OR 'compresolon' OR 'cortadeltona' OR 'cortadeltone' OR 'cortalone' OR 'cortelinter' OR 'cortisolone' OR 'cotolone' OR 'dacortin' OR 'dacortin h' OR 'dacrotin' OR 'decaprednil' OR 'decortin h' OR 'decortril' OR 'dehydro cortex' OR 'dehydro hydrocortison' OR 'dehydro hydrocortisone' OR 'dehydrocortex' OR 'dehydrocortisol' OR 'dehydrocortisole' OR 'dehydrohydrocortison' OR 'dehydrohydrocortisone' OR 'delcortol' OR 'delta 1 17 hydroxycorticosterone 21 acetate' OR 'delta 1 hydrocortisone' OR 'delta cortef' OR 'delta cortril' OR 'delta ef cortelan' OR 'delta f' OR 'delta hycortol' OR 'delta hydrocortison' OR 'delta hydrocortisone' OR 'delta ophticor' OR 'delta stab' OR 'delta-cortef' OR 'delta1 dehydrocortisol' OR 'delta1 dehydrohydrocortisone' OR 'delta1 hydrocortisone' OR 'deltacortef' OR 'deltacortenolo' OR 'deltacortil' OR 'deltacortoil' OR 'deltacortril' OR 'deltaderm' OR 'deltaglycortril' OR 'deltahycortol' OR 'deltahydrocortison' OR 'deltahydrocortisone' OR 'deltaophticor' OR 'deltasolone' OR 'deltastab' OR 'deltidrosol' OR 'deltisilone' OR 'deltisolon' OR 'deltisolone' OR 'deltolasson' OR 'deltolassone' OR 'deltosona' OR 'deltosone' OR 'depo-predate' OR 'dermosolon' OR 'dhasolone' OR 'di adreson f' OR 'di adresone f' OR 'di-adreson-f' OR 'diadreson f' OR 'diadresone f' OR 'dicortol' OR 'domucortone' OR 'encortelon' OR 'encortelone' OR 'encortolon' OR 'equisolon' OR 'fernisolone-p' OR 'glistelone' OR 'hefasolon' OR 'hostacortin h' OR 'hostacortin h vet' OR 'hydeltra' OR 'hydeltrone' OR 'hydrelta' OR 'hydrocortancyl' OR 'hydrocortidelt' OR 'hydrodeltalone' OR 'hydrodeltisone' OR 'hydroretrocortin' OR 'hydroretrocortine' OR 'inflanefran' OR 'insolone' OR 'keteocort h' OR 'key-pred'

OR 'key-pred sp' OR 'lenisolone' OR 'leocortol' OR 'liquipred' OR 'lygal kopftinktur n' OR 'mediasolone' OR 'meprisolon' OR 'meprisolone' OR 'metacortalon' OR 'metacortalone' OR 'metacortandralon' OR 'metacortandralone' OR 'metacortelone' OR 'meti derm' OR 'meti-derm' OR 'meticortelone' OR 'metiderm' OR 'morlone' OR 'mydrapred' OR 'neo delta' OR 'nisolon' OR 'nisolone' OR 'nsc 9120' OR 'nsc9120' OR 'opredsone' OR 'panafcortelone' OR 'panafcortolone' OR 'panafort' OR 'paracortol' OR 'phlogex' OR 'pre cortisyl' OR 'preconin' OR 'precortalon' OR 'precortancyl' OR 'precortisyl' OR 'pred-ject-50' OR 'predacort 50' OR 'predaject-50' OR 'predalone 50' OR 'predartrina' OR 'predartrine' OR 'predate-50' OR 'predeltilone' OR 'predisole' OR 'predisyr' OR 'predne dome' OR 'prednecort' OR 'prednedome' OR 'prednelan' OR 'predni coelin' OR 'predni h tablinen' OR 'predni-helvacort' OR 'prednicoelin' OR 'prednicort' OR 'prednicortelone' OR 'prednifor drops' OR 'predniment' OR 'predniretard' OR 'prednis' OR 'prednisil' OR 'prednisolon' OR 'prednisolona' OR 'prednisolone*' OR 'prednisolone alcohol' OR 'prednisolone h' OR 'prednisolone oleosae sr 82' OR 'prednisolone, topical' OR 'prednivet' OR 'prednorsolon' OR 'prednorsolone' OR 'predonine' OR 'predorgasolona' OR 'predorgasolone' OR 'pregna 1, 4 diene 11beta, 17alpha, 21 triol 3, 20 dione' OR 'prelon' OR 'prelone' OR 'prenilone' OR 'prenin' OR 'prenolone' OR 'preventan' OR 'prezolon' OR 'rubycort' OR 'scherisolon' OR 'scherisolona' OR 'serilone' OR 'solondo' OR 'solone' OR 'solupren' OR 'soluprene' OR 'spiricort' OR 'spolotane' OR 'sterane' OR 'sterolone' OR 'supercortisol' OR 'supercortizol' OR 'taracortelone' OR 'walesolone' OR 'wysolone'",147404,27 Nov 2020

#134,"'corticosteroid'/exp OR 'adrenal cortex hormone' OR 'adrenal cortex hormones' OR 'adrenal cortical hormone' OR 'adrenal cortical hormones' OR 'adrenal cortical steroid' OR 'adrenal steroid' OR 'adrenal steroid hormone' OR 'adreno cortical steroid' OR 'adreno corticosteroid' OR 'adrenocortical hormone' OR 'adrenocortical steroid' OR 'adrenocorticosteroid' OR 'cortical steroid' OR 'cortico steroid'

OR 'corticoid*' OR 'corticosteroid*' OR 'corticosteroid agent' OR 'corticosteroid calcium' OR 'corticosteroid hormone' OR 'corticosteroids' OR 'corticosteroids, inhalation' OR 'corticosteroids, ophthalmic' OR 'corticosteroids, otic' OR 'corticosteroids, systemic' OR 'corticosteroids, topical' OR 'dermocorticosteroid' OR 'fluorinated corticosteroid'",1039987,27 Nov 2020

#133,"chloroquin*:ti,ab,kw,rn,tn OR cq:ti,ab,kw,rn,tn OR '54-04-7':ti,ab,kw,rn,tn OR 886u3h6uff*:ti,ab,kw,rn,tn OR arequin:ti,ab,kw,rn,tn OR chingamin:ti,ab,kw,rn,tn OR chlorochin*:ti,ab,kw,rn,tn OR khingamin:ti,ab,kw,rn,tn OR oe48649kh6n*:ti,ab,kw,rn,tn OR anaclor:ti,ab,kw,rn,tn OR bemaco:ti,ab,kw,rn,tn OR benaquin:ti,ab,kw,rn,tn OR 'brn 0482809':ti,ab,kw,rn,tn OR brn0482809:ti,ab,kw,rn,tn OR capquin:ti,ab,kw,rn,tn OR 'ccris 3439':ti,ab,kw,rn,tn OR ccris3439:ti,ab,kw,rn,tn OR chloraquin*:ti,ab,kw,rn,tn OR cindacin:ti,ab,kw,rn,tn OR cindachin:ti,ab,kw,rn,tn OR cloroquina:ti,ab,kw,rn,tn OR cocatrit:ti,ab,kw,rn,tn OR 'einecs 200-191-2':ti,ab,kw,rn,tn OR 'einecs200 191 2':ti,ab,kw,rn,tn OR elestol:ti,ab,kw,rn,tn OR 'hsdb 3029':ti,ab,kw,rn,tn OR hsdb3029:ti,ab,kw,rn,tn OR lapaquin:ti,ab,kw,rn,tn OR malaren:ti,ab,kw,rn,tn OR neochin:ti,ab,kw,rn,tn OR 'nsc 187208':ti,ab,kw,rn,tn OR nsc187208:ti,ab,kw,rn,tn OR pfizerquine:ti,ab,kw,rn,tn OR quensyl:ti,ab,kw,rn,tn OR quinercyl:ti,ab,kw,rn,tn OR quinilon:ti,ab,kw,rn,tn OR quinoline:ti,ab,kw,rn,tn OR quinoscan:ti,ab,kw,rn,tn OR ronaquine:ti,ab,kw,rn,tn OR sopaquin:ti,ab,kw,rn,tn OR 'st 21':ti,ab,kw,rn,tn OR weimerquin:ti,ab,kw,rn,tn",36707,27 Nov 2020

#132,"'chloroquine'/de OR '4 (4 diethylamino 1 methylbutylamino) 7 chlorchinolin diphosphate' OR '4 (4 diethylamino 1 methylbutylamino) 7 chlorchinolin sulfate' OR '4 (4 diethylamino 1 methylbutylamino) 7 chlorchinolin sulphate' OR '4 (4 diethylamino 1 methylbutylamino) 7 chloroquinoline' OR '7 chloro 4 (4 diethylamino 1 methylbutylamino) quinoline' OR '7 chloro 4 (4

diethylamino 1 methylbutylamino) quinoline diphosphate' OR 'a-cq' OR 'amokin' OR 'amokine' OR 'anoclor' OR 'aralan' OR 'aralen' OR 'aralen hydrochloride' OR 'aralen phosphate' OR 'aralene' OR 'arechin' OR 'arechine' OR 'arequine' OR 'arthrochin' OR 'arthrochine' OR 'arthroquine' OR 'artrichin' OR 'artrichine' OR 'artriquine' OR 'avloclor' OR 'avoclor' OR 'bemaphata' OR 'bemaphate' OR 'bemasulph' OR 'bipiquin' OR 'cadiquin' OR 'chemochin' OR 'chemochine' OR 'chingamine' OR 'chingaminum' OR 'chloraquine' OR 'chlorochin' OR 'chlorochine' OR 'chlorofoz' OR 'chloroquin' OR 'chloroquin phosphate' OR 'chloroquine' OR 'chloroquine diphosphate' OR 'chloroquine disulfate' OR 'chloroquine disulphate' OR 'chloroquine hydrochloride' OR 'chloroquine phosphate' OR 'chloroquine streuli' OR 'chloroquine sulfate' OR 'chloroquine sulphate' OR 'chloroquinesulphate' OR 'chloroquini diphosphas' OR 'chloroquinum diphosphoricum' OR 'chlorquin' OR 'chlorquine' OR 'choloquine' OR 'choroquine sulfate' OR 'choroquine sulphate' OR 'cidanchin' OR 'clo-kit junior' OR 'clorichina' OR 'clorichine' OR 'cloriquine' OR 'clorochina' OR 'delagil' OR 'delagyl' OR 'dichinalex' OR 'diclokin' OR 'diquinalex' OR 'diroquine' OR 'emquin' OR 'genocin' OR 'gontochin' OR 'gontochine' OR 'gontoquine' OR 'heliopar' OR 'imagon' OR 'iroquine' OR 'klorokin' OR 'klorokine' OR 'klorokinfosfat' OR 'lagaquin' OR 'malaquin' OR 'malarex' OR 'malarivon' OR 'malaviron' OR 'maliaquine' OR 'maquine' OR 'mesylith' OR 'mexaquin' OR 'mirquin' OR 'nivachine' OR 'nivaquin' OR 'nivaquine' OR 'nivaquine (b)' OR 'nivaquine b' OR 'nivaquine dp' OR 'nivaquine forte' OR 'p roquine' OR 'quinachlor' OR 'quingamine' OR 'repal' OR 'resochen' OR 'resochene' OR 'resochin' OR 'resochin junior' OR 'resochina' OR 'resochine' OR 'resochinon' OR 'resoquina' OR 'resoquine' OR 'reumachlor' OR 'roquine' OR 'rp 3377' OR 'rp3377' OR 'sanoquin' OR 'sanoquine' OR 'silbesan' OR 'siragan' OR 'sirajan' OR 'sn 7618' OR 'sn7618' OR 'solprina' OR 'solprine' OR 'tresochin' OR 'tresochine' OR 'tresoquine' OR 'trochin' OR 'trochine' OR 'troquine' OR 'w 7618' OR 'w7618' OR 'win 244' OR 'win244'",44555,27 Nov 2020

#131,"'organogold compound'/exp OR 'organogold compound' OR 'organogold compounds' OR 'organogold derivative'",482,27 Nov 2020

#130,"aurothiopropanol*:ti,ab,kw,rn,tn OR allochrysine:ti,ab,kw,rn,tn OR allocrisine:ti,ab,kw,rn,tn OR aurotiopoe:ti,ab,kw,rn,tn OR aurotioprol:ti,ab,kw,rn,tn OR '27279-43-2':ti,ab,kw,rn,tn OR g7097j63e9*:ti,ab,kw,rn,tn OR 'sodium aurothiopropanolsulfonate':ti,ab,kw,rn,tn OR 'gold sodium thiopropanol sulfonate':ti,ab,kw,rn,tn OR 'sodium auromercaptopropanol sulfonate':ti,ab,kw,rn,tn",274,27 Nov 2020

#129,"'allochrysine'/de OR '1 auromercapto 2 propanol 3 sulfonate' OR '1 auromercapto 2 propanol 3 sulfonate sodium' OR '1 auromercepto 2 propanol 3 sulfonate' OR 'allochrysine' OR 'auromercapto propanol sulfonate sodium' OR 'auromercaptopropanolsulfonate sodium' OR 'aurothioisopropanolsulfonate sodium' OR 'aurothiopropanol sulfonate' OR 'aurothiopropanol sulfonate sodium' OR 'gold sodium thiopropanol sulfonate' OR 'sodium 1 auromercapto 2 propanol 3 sulfonate' OR 'sodium auromercapto 2 propanol 3 sulfonate' OR 'sodium auromercaptopropanol sulfonate' OR 'sodium aurothiopropanol sulfonate'",272,27 Nov 2020

#128,"((gold* OR auro*) NEAR/10 (inject* OR intramuscular* OR 'im' OR 'intra muscular*')):ti,ab,kw",3123,27 Nov 2020

#127,"(thioglucosoaurate*:ti,ab,kw,rn,tn OR 'gold thioglucose*':ti,ab,kw,rn,tn OR 'gold thio glucose*':ti,ab,kw,rn,tn OR 'thioglucose gold':ti,ab,kw,rn,tn OR aureotan:ti,ab,kw,rn,tn OR 'gold-50':ti,ab,kw,rn,tn OR gold50:ti,ab,kw,rn,tn OR 'b oleosum solganal':ti,ab,kw,rn,tn OR aurotan:ti,ab,kw,rn,tn OR authron:ti,ab,kw,rn,tn OR brenol:ti,ab,kw,rn,tn OR 'ccris 59':ti,ab,kw,rn,tn OR ccris59:ti,ab,kw,rn,tn OR 'einecs 235-365-7':ti,ab,kw,rn,tn OR 'einecs235 365 7':ti,ab,kw,rn,tn OR glysanol:ti,ab,kw,rn,tn) AND b:ti,ab,kw,rn,tn OR goldthioglucose*:ti,ab,kw,rn,tn OR

goldtioglucose*:ti,ab,kw,rn,tn OR 'hsdb 7174':ti,ab,kw,rn,tn OR hsdb7174:ti,ab,kw,rn,tn OR oronol:ti,ab,kw,rn,tn OR romosol:ti,ab,kw,rn,tn OR 'skf 10056':ti,ab,kw,rn,tn OR skf10056:ti,ab,kw,rn,tn OR 2p2v9q0e78*:ti,ab,kw,rn,tn OR '12192-57-3':ti,ab,kw,rn,tn",1509,27 Nov 2020

#126,"'aurothioglucose sodium'/de",40,27 Nov 2020

#125,"'aurothioglucose'/de OR '(1 d glucosylthio) gold' OR '(1 glucosylthio) gold' OR 'anitur' OR 'aureotan' OR 'auro thioglucose*' OR 'auromyose*' OR 'aurothioglucose*' OR 'aurumin' OR 'aurumine' OR 'gold thio glucose' OR 'gold thioglucose' OR 'gold-50' OR 'goldthioglucose' OR 'goldtioglucose' OR 'oronol' OR 'solganal' OR 'solganal b' OR 'solganol'",1709,27 Nov 2020

#124,"aurolate*:ti,ab,kw,rn,tn OR 'gold disodium thiomalate*':ti,ab,kw,rn,tn OR 'gold thiomalic acid*':ti,ab,kw,rn,tn OR miocrisin:ti,ab,kw,rn,tn OR 'monogold disodium thiomalate*':ti,ab,kw,rn,tn OR myochrysin*:ti,ab,kw,rn,tn OR myocrisin*:ti,ab,kw,rn,tn OR myocrysin*:ti,ab,kw,rn,tn OR 'sodium aurothiomalate*':ti,ab,kw,rn,tn OR 'sodium gold thiomalate*':ti,ab,kw,rn,tn OR 'sodium thiomalate* gold':ti,ab,kw,rn,tn OR 'sodium thiomalatoaurate*':ti,ab,kw,rn,tn OR taureodon:ti,ab,kw,rn,tn OR 'thiomalatoaurate sodium':ti,ab,kw,rn,tn OR '1244-57-4':ti,ab,kw,rn,tn OR '39377-38-3':ti,ab,kw,rn,tn OR '4846-27-9':ti,ab,kw,rn,tn OR '74916-57-7':ti,ab,kw,rn,tn OR e4768zy6gm*:ti,ab,kw,rn,tn OR 'aurothiomala-natrium':ti,ab,kw,rn,tn OR 'aurot$iomalato sodico':ti,ab,kw,rn,tn OR 'monogold* sodium salt':ti,ab,kw,rn,tn OR 'dinatrium 2-aurothio-succinat':ti,ab,kw,rn,tn OR 'einecs 235-479-7':ti,ab,kw,rn,tn OR 'einecs235 479 7':ti,ab,kw,rn,tn OR 'hsdb 7173':ti,ab,kw,rn,tn OR hsdb7173:ti,ab,kw,rn,tn OR kidon:ti,ab,kw,rn,tn OR 'natrii aurothiomalas*':ti,ab,kw,rn,tn OR shiosol:ti,ab,kw,rn,tn OR 'gold mercatpsuccinate*':ti,ab,kw,rn,tn",885,27 Nov 2020

#123,"'aurothiomalate'/de OR '(1, 2 dicarboxyethylthio) gold disodium' OR '(1, 2 dicarboxyethylthio) gold disodium salt' OR 'aurothio' OR 'aurothiomalate*' OR 'aurothiomalate sodium' OR 'disodium

aurothiomalate' OR 'gold mercaptosuccinate' OR 'gold sodium thiomalate*' OR 'gold thiomalate*' OR 'gold thiomalate sodium' OR 'miocrin' OR 'myochrisin' OR 'myochrisine' OR 'myochrysin' OR 'myochrysine' OR 'myocrisin' OR 'myocrysin' OR 'shiosol' OR 'sodium aurothiomalate' OR 'sodium gold thiomalate' OR 'tauredon' OR 'tauredone' OR 'thiomalate gold'",3454,27 Nov 2020

#122,"hydroxychloroquin*:ti,ab,kw,rn,tn OR hcq:ti,ab,kw,rn,tn OR '747-3-4':ti,ab,kw,rn,tn OR 8q2869cnvh*:ti,ab,kw,rn,tn OR '118-42-3':ti,ab,kw,rn,tn OR 4qwg6n8qkh*:ti,ab,kw,rn,tn OR hydroxychlorochin*:ti,ab,kw,rn,tn OR hydrochloroquin*:ti,ab,kw,rn,tn OR hydrocloroquin*:ti,ab,kw,rn,tn OR oxychlorochin:ti,ab,kw,rn,tn OR oxychloroquin*:ti,ab,kw,rn,tn OR plaquinol:ti,ab,kw,rn,tn OR 'brn 0253894':ti,ab,kw,rn,tn OR brn0253894:ti,ab,kw,rn,tn OR 'einecs 204-249-8':ti,ab,kw,rn,tn OR 'einecs204 249 8':ti,ab,kw,rn,tn OR hidroxicloroquin*:ti,ab,kw,rn,tn OR idrossiclorochin*:ti,ab,kw,rn,tn OR oxichlorochin*:ti,ab,kw,rn,tn OR oxichloroquin*:ti,ab,kw,rn,tn OR win1258:ti,ab,kw,rn,tn OR 'win 1258':ti,ab,kw,rn,tn OR z0188:ti,ab,kw,rn,tn OR 'einecs 212-019-3':ti,ab,kw,rn,tn OR 'einecs212-019-3':ti,ab,kw,rn,tn OR erquin:ti,ab,kw,rn,tn OR quensyl:ti,ab,kw,rn,tn OR 'sn 8137':ti,ab,kw,rn,tn OR sn8137:ti,ab,kw,rn,tn",29714,27 Nov 2020

#121,"'hydroxychloroquine sulfate'/de OR '1 (7 chloro 4 quinolylamino) 3 diethylamino 2 propanol sulfate' OR '1 (7 chloro 4 quinolylamino) 3 diethylamino 2 propanol sulphate' OR '2 [[4 [(7 chloro 4 quinolinyl) amino] pentyl] (ethyl) amino] ethanol sulfate' OR '2 [[4 [(7 chloro 4 quinolinyl) amino] pentyl] (ethyl) amino] ethanol sulphate' OR '2 [[4 [(7 chloroquinolin 4 yl) amino] pentyl] (ethyl) amino] ethanol sulfate' OR '2 [[4 [(7 chloroquinolin 4 yl) amino] pentyl] (ethyl) amino] ethanol sulphate' OR '7 chloro 4 (3 diethylamino 2 hydroxypropylamino) quinoline sulfate' OR '7 chloro 4 (3 diethylamino 2 hydroxypropylamino) quinoline sulphate' OR 'dimard' OR 'erquin' OR 'evoquin' OR 'geniquin' OR 'hydroxychloroquine sulfate' OR 'hydroxychloroquine sulphate' OR 'oxiklorin' OR 'oxychloroquine

sulfate' OR 'oxychloroquine sulphate' OR 'plaquenil' OR 'plaquenil sulfate' OR 'plaquenil sulphate' OR 'plaquinol' OR 'toremonil' OR 'yuma'",3651,27 Nov 2020

#120,"'hydroxychloroquine'/de OR '7 chloro 4 [4 [ethyl (2 hydroxyethyl) amino] 1 methylbutylamino] quinoline' OR '7 chloro 4 [4 [ethyl (2 hydroxyethyl) amino] 1 methylbutylamino] quinoline diphosphate' OR 'apo-hydroxychloroquine' OR 'chloroquinol' OR 'ercoquin' OR 'hydrochloroquine' OR 'hydrocloroquine' OR 'hydroxychloroquine' OR 'oxychloroquine' OR 'quensyl' OR 'sn 8137'",29882,27 Nov 2020

#119,"sasp:ti,ab,kw,rn,tn OR ssz:ti,ab,kw,rn,tn OR salicylazosulfapyridin*:ti,ab,kw,rn,tn OR 'salicyl azo sulfapyridin*':ti,ab,kw,rn,tn OR salazosulfapyridin*:ti,ab,kw,rn,tn OR salazosulfapiridin*:ti,ab,kw,rn,tn OR azulfadin*:ti,ab,kw,rn,tn OR azulfidin*:ti,ab,kw,rn,tn OR pleon:ti,ab,kw,rn,tn OR ulcol:ti,ab,kw,rn,tn OR ucine:ti,ab,kw,rn,tn OR salazopyrin*:ti,ab,kw,rn,tn OR 'ratio-sulfosalazin*':ti,ab,kw,rn,tn OR 3xc8guz6cb*:ti,ab,kw,rn,tn OR accucol:ti,ab,kw,rn,tn OR asulfidin*:ti,ab,kw,rn,tn OR azopyrin*:ti,ab,kw,rn,tn OR azosulfidin*:ti,ab,kw,rn,tn OR 'brn 0356241':ti,ab,kw,rn,tn OR brn0356241:ti,ab,kw,rn,tn OR 'ccris 4713':ti,ab,kw,rn,tn OR ccris4713:ti,ab,kw,rn,tn OR 'einecs 209-974-3':ti,ab,kw,rn,tn OR 'hsdb 3395':ti,ab,kw,rn,tn OR hsdb3395:ti,ab,kw,rn,tn OR 'nsc 203730':ti,ab,kw,rn,tn OR nsc203730:ti,ab,kw,rn,tn OR 'nsc 667219':ti,ab,kw,rn,tn OR nsc667219:ti,ab,kw,rn,tn OR reupirin:ti,ab,kw,rn,tn OR 'salazo-sulfapyridin*':ti,ab,kw,rn,tn OR salazosulfpyridin*:ti,ab,kw,rn,tn OR salazopiridazin*:ti,ab,kw,rn,tn OR salazopyridin*:ti,ab,kw,rn,tn OR salazopirin*:ti,ab,kw,rn,tn OR salipyr:ti,ab,kw,rn,tn OR 'si-88':ti,ab,kw,rn,tn OR si88:ti,ab,kw,rn,tn OR sulfasalazin*:ti,ab,kw,rn,tn OR sulphasalazin*:ti,ab,kw,rn,tn OR 'w-t sasp oral':ti,ab,kw,rn,tn OR '599-79-1':ti,ab,kw,rn,tn",28724,27 Nov 2020

#118,"'salazosulfapyridine'/de OR '4 (2 pyridylaminosulfonyl) 3` carboxy 4` hydroxyazobenzene' OR '4 hydroxy 4` (pyrid 2 ylsulfamoyl) azobenzene 3 carboxylic acid' OR '5 [4 (2 pyridylsulfamoyl) phenylazo] 2 hydroxybenzoic acid' OR '5 [para (2 pyridylsulfamoyl) phenylazo] salicylic acid' OR 'azlufidine en-tabs' OR 'azopyrin' OR 'azopyrine' OR 'azosulfidine' OR 'azulfide' OR 'azulfidina' OR 'azulfidine' OR 'azulfidine en tabs' OR 'azulfidine en-tabs' OR 'azulfidine ra' OR 'azulfin' OR 'benzosulfa' OR 'colo pleon' OR 'colo-pleon' OR 'colopleon' OR 'disalazin' OR 'gastropyrin' OR 'pleon ra' OR 'pyralin en' OR 'rorasul' OR 'rosulfant' OR 's.a.s.-500' OR 'salazine' OR 'salazo sulfapyridine' OR 'salazodin' OR 'salazopirina' OR 'salazopyridin' OR 'salazopyridine' OR 'salazopyrin' OR 'salazopyrin entabs' OR 'salazopyrin-en' OR 'salazopyrina' OR 'salazopyrine' OR 'salazopyrine ec' OR 'salazosulfa pyridine' OR 'salazosulfapyridine' OR 'salazosulfpyridine' OR 'salicyl azo sulfapyridine' OR 'salicylazosulfapyridin' OR 'salicylazosulfapyridine' OR 'salisulf' OR 'salopyr' OR 'saridine' OR 'sas 500' OR 'sulcolon' OR 'sulfasalazine' OR 'sulfasalizine' OR 'sulfosalazine' OR 'sulphasalazine' OR 'zopyrin'",26755,27 Nov 2020

#117,"'lef':ti,ab,kw",4109,27 Nov 2020

#116,"'leflunomide'/de OR '5 methyl 4` trifluoromethyl 4 isoxazolecarboxanilide' OR '5 methyl n [4 (trifluoromethyl) phenyl] 4 isoxazolecarboxamide' OR '5 methyl n [4 (trifluoromethyl) phenyl] isoxazole 4 carboxamide' OR '5 methyl n [para (trifluoromethyl) phenyl] 4 isoxazolecarboxamide' OR 'alpha, alpha, alpha trifluoro 5 methyl 4 isoxazolecarboxy para toluidide' OR 'arabloc' OR 'arava' OR 'hwa 486' OR 'hwa486' OR 'leflunomid*' OR 'leflunomide winthrop' OR 'n (4 trifluoromethylphenyl) 5 methylisoxazole 4 carboxamide' OR 'repso' OR 'rs 34821' OR 'rs34821' OR 'su 101' OR 'su101' OR 'hsdb 7289' OR hsdb7289 OR g162gk9u4w* OR '75706-12-6'",12984,27 Nov 2020

#115,"'mtx':ti,ab,kw,rn,tn OR methotrex*:ti,ab,kw,rn,tn OR metotrexat*:ti,ab,kw,rn,tn OR methylaminopterin*:ti,ab,kw,rn,tn OR amet$opterin*:ti,ab,kw,rn,tn OR brimexate:ti,ab,kw,rn,tn OR 'a-

met$opterin*':ti,ab,kw,rn,tn OR 'a-methpterin*':ti,ab,kw,rn,tn OR 'ai3-25299':ti,ab,kw,rn,tn OR ai325299:ti,ab,kw,rn,tn OR 'alpha-methopterin':ti,ab,kw,rn,tn OR farmitrexat*:ti,ab,kw,rn,tn OR fauldexato:ti,ab,kw,rn,tn OR hdmtx:ti,ab,kw,rn,tn OR metotressat*:ti,ab,kw,rn,tn OR methotrate*:ti,ab,kw,rn,tn OR methohexate*:ti,ab,kw,rn,tn OR mexat*:ti,ab,kw,rn,tn OR metrotex:ti,ab,kw,rn,tn OR metatrexato*:ti,ab,kw,rn,tn OR metatrexan:ti,ab,kw,rn,tn OR maxtrex:ti,ab,kw,rn,tn OR meticil:ti,ab,kw,rn,tn OR metoject:ti,ab,kw,rn,tn OR metotrexin:ti,ab,kw,rn,tn OR lumexon:ti,ab,kw,rn,tn OR r$eumatrex*:ti,ab,kw,rn,tn OR tremetex:ti,ab,kw,rn,tn OR trexeron:ti,ab,kw,rn,tn OR trixilem:ti,ab,kw,rn,tn OR 'mpi-2505':ti,ab,kw,rn,tn OR mpi2505:ti,ab,kw,rn,tn OR amethopterin*:ti,ab,kw,rn,tn OR '133073-73-1':ti,ab,kw,rn,tn OR '15475-56-6':ti,ab,kw,rn,tn OR 3ig1e710zn*:ti,ab,kw,rn,tn OR yl5fz2y5u1*:ti,ab,kw,rn,tn OR 'ccris 1109':ti,ab,kw,rn,tn OR ccris1109:ti,ab,kw,rn,tn OR 'emt 25299':ti,ab,kw,rn,tn OR emt25299:ti,ab,kw,rn,tn OR 'r-9985':ti,ab,kw,rn,tn OR r9885:ti,ab,kw,rn,tn OR 'x-133':ti,ab,kw,rn,tn OR 'wr-19039':ti,ab,kw,rn,tn OR wr19039:ti,ab,kw,rn,tn",191138,27 Nov 2020
#114,"'methotrexate'/de OR '2 [[4 [[(2, 4 diamino 6 pteridinyl) methyl] (methyl) amino] benzoyl] amino] pentanedioic acid' OR '2 [[4 [[(2, 4 diaminopteridin 6 yl) methyl] (methyl) amino] benzoyl] amino] pentanedioic acid' OR '4 amino 10 methylfolic acid' OR '4 amino 10 methylpteroylglutamic acid' OR '4 amino n10 methylpteroylglutamic acid' OR 'mtx' OR 'a methopterine' OR 'abitrexate' OR 'amethopterin' OR 'amethopterine' OR 'ametopterine' OR 'antifolan' OR 'biotrexate' OR 'canceren' OR 'cl 14377' OR 'cl14377' OR 'emtexate' OR 'emthexat' OR 'emthexate' OR 'emtrexate' OR 'enthexate' OR 'farmitrexat' OR 'farmitrexate' OR 'farmotrex' OR 'folex' OR 'folex pfs' OR 'ifamet' OR 'imeth' OR 'intradose mtx' OR 'jylamvo' OR 'lantarel' OR 'ledertrexate' OR 'maxtrex' OR 'metex' OR 'methoblastin' OR 'methohexate' OR 'methotrate' OR 'methotrexat' OR 'methotrexat ebewe' OR 'methotrexate' OR

'methotrexate lpf' OR 'methotrexate preservative free' OR 'methotrexate sodium' OR 'methotrexate sodium preservative free' OR 'methotrexato' OR 'methoxtrexate' OR 'methrotrexate' OR 'methylaminopterin' OR 'methylaminopterine' OR 'meticil' OR 'metoject' OR 'metothrexate' OR 'metothrexate sodium' OR 'metotrexat' OR 'metotrexate' OR 'metotrexin' OR 'metrex' OR 'mexate' OR 'mexate-aq' OR 'mexate-aq preserved' OR 'mpi 5004' OR 'mpi5004' OR 'n [4 [(2, 4 diamino 6 pteridylmethyl) methylamino] benzoyl] glutamic acid' OR 'neotrexate' OR 'nordimet' OR 'novatrex' OR 'nsc 740' OR 'nsc740' OR 'otrexup' OR 'otrexup pfs' OR 'rasuvo' OR 'reditrex' OR 'reumatrex' OR 'rheumatrex' OR 'rheumatrex dose pack' OR 'sodium methotrexate' OR 'texate' OR 'texate-t' OR 'texorate' OR 'trexall' OR 'xaken' OR 'xatmep' OR 'zexate'",191406,27 Nov 2020

#113,"(('disease modifying' NEAR/3 (antir$eum* OR 'anti r$eum*') NEAR/3 (drug$ OR agent$)):ti,ab,kw) OR (('disease modifying' NEAR/3 (antir$eum* OR 'anti r$eum*') NEAR/3 (drug$ OR agent$)):tt)",10989,27 Nov 2020

#112,"dmard*:ti,ab,kw OR csdmard*:ti,ab,kw OR 'cs-dmard*':ti,ab,kw OR sdmard*:ti,ab,kw OR 's-dmard*':ti,ab,kw OR tsdmard*:ti,ab,kw OR 'ts-dmard*':ti,ab,kw OR bdmard*:ti,ab,kw OR 'b-dmard*':ti,ab,kw OR dmard*:tt OR csdmard*:tt OR 'cs-dmard*':tt OR sdmard*:tt OR 's-dmard*':tt OR tsdmard*:tt OR 'ts-dmard*':tt OR bdmard*:tt OR 'b-dmard*':tt",17435,27 Nov 2020

#111,"'disease modifying antirheumatic drug'/de OR 'disease modifying antirheumatic agent' OR 'disease modifying antirheumatic drug' OR 'disease modifying antirheumatic drugs'",20106,27 Nov 2020

#110,"'mavrilimumab'/de OR 'mavrilimumab*' OR '1085337 57 0' OR 1158jdp9a* OR 'cam 3001' OR cam3001",130,27 Nov 2020

#109,"'ustekinumab'/de OR 'cnto 1275' OR 'cnto1275' OR 'monoclonal antibody cnto 1275' OR 'stelara' OR 'ustekinumab*' OR '815610 63 0' OR fu77b4u5z0* OR 'stelera'",7706,27 Nov 2020

#108,"'guselkumab'/de OR 'cnto 1959' OR 'cnto1959' OR 'guselkumab' OR 'tremfya' OR '1350289 85 8' OR 089658a12d*",831,27 Nov 2020

#107,"'brodalumab'/de OR 'amg 827' OR 'amg827' OR 'brodalumab*' OR 'kyntheum' OR 'siliq' OR 'khk 4827' OR khk4827 OR '1174395 19 7' OR 6za31y954z*",1173,27 Nov 2020

#106,"'ixekizumab'/de OR 'ixekizumab' OR 'ly 2439821' OR 'ly2439821' OR 'taltz' OR '1329632 62 3' OR '1143503 69 8' OR bty153760o*",1962,27 Nov 2020

#105,"'secukinumab'/de OR 'ain 457' OR 'ain457' OR 'cosentyx' OR 'secukinumab*' OR '1229022 83 6' OR dlg4eml025*",4070,27 Nov 2020

#104,"'pateclizumab'/de OR 'mlta 3698a' OR 'mlta3698a' OR 'pateclizumab' OR 'pro 283698' OR 'pro283698' OR '12202526 59 7' OR qok1yyh7j2* OR 'rg 7415' OR rg7415",28,27 Nov 2020

#103,"'tabalumab'/de OR 'ly 2127399' OR 'ly2127399' OR 'tabalumab*' OR '1143503 67 6' OR pqp8vh3mjw*",272,27 Nov 2020

#102,"abatacept*:ti,ab,kw,rn,tn OR 'ctla 4 ig':ti,ab,kw,rn,tn OR 'ctla-igg4m':ti,ab,kw,rn,tn OR 'ctla4-fc':ti,ab,kw,rn,tn OR '332348 12 6':ti,ab,kw,rn,tn OR 7d0yb67s97*:ti,ab,kw,rn,tn OR 'rg 2077':ti,ab,kw,rn,tn OR rg2077:ti,ab,kw,rn,tn OR 'rg-1046':ti,ab,kw,rn,tn OR rg1046:ti,ab,kw,rn,tn OR nulojix:ti,ab,kw,rn,tn OR lea29y:ti,ab,kw,rn,tn OR 'lea 29y':ti,ab,kw,rn,tn OR 'ctl4 fc':ti,ab,kw,rn,tn OR ctl4fc:ti,ab,kw,rn,tn OR belatacept:ti,ab,kw,rn,tn OR 'bms 224818':ti,ab,kw,rn,tn OR bms224818:ti,ab,kw,rn,tn OR 'cytotoxic t lymphocyte associat* antigen 4 immunglobulin*':ti,ab,kw,rn,tn",11029,27 Nov 2020

#101,"'abatacept'/de OR 'ctla4 ig' OR 'ctla4 immunoglobulin' OR 'ctla4 immunoglobulin g' OR 'ctla4ig' OR 'abatacept' OR 'bms 188667' OR 'bms188667' OR 'orencia'",11027,27 Nov 2020

#100,"'ofatumumab'/de OR 'humax cd20' OR 'humax-cd20' OR 'humaxcd20' OR 'arzerra' OR 'gsk 1841157' OR 'gsk1841157' OR 'humac cd20' OR 'ofatumumab*' OR 'omb 157' OR 'omb157' OR 'humax-cd20-2f2' OR '679818 59 8' OR m95kg522r0* OR 'hsdb 8170' OR hsdb8170",3083,27 Nov 2020

#99,"'rituximab'/de OR 'abp 798' OR 'abp798' OR 'blitzima' OR 'ct p10' OR 'ctp10' OR 'gp 2013' OR 'gp2013' OR 'hlx 01' OR 'hlx01' OR 'idec 102' OR 'idec c2b8' OR 'idec102' OR 'idecc2b8' OR 'mabthera' OR 'mab thera' OR 'mk 8808' OR 'mk8808' OR 'monoclonal antibody idec c2b8' OR 'pf 05280586' OR 'pf 5280586' OR 'pf05280586' OR 'pf5280586' OR 'r 105' OR 'r105' OR 'reditux' OR 'rg 105' OR 'rg105' OR 'ritemvia' OR 'ritumax' OR 'rituxan' OR 'rituximab*' OR 'rituximab abbs' OR 'rituximab pvvr' OR 'rituximab-abbs' OR 'rituximab-pvvr' OR 'rituxin' OR 'rituzena' OR 'rixathon' OR 'riximyo' OR 'ro 452294' OR 'ro452294' OR 'ruxience' OR 'truxima' OR 'tuxella' OR '174722 31 7' OR 4f4x42syq6* OR 'hsdb 7455' OR hsdb7455",86075,27 Nov 2020

#98,"'czp':ti,ab,kw,rn,tn OR '428863 50 7':ti,ab,kw,rn,tn OR g6adw90r16*:ti,ab,kw,rn,tn OR umd07x179e*:ti,ab,kw,rn,tn OR 'hsdb 7848':ti,ab,kw,rn,tn OR hsdb7848:ti,ab,kw,rn,tn",6879,27 Nov 2020

#97,"'certolizumab pegol'/de OR 'cdp 870' OR 'cdp870' OR 'certolizumab pegol' OR 'cimzia' OR 'pegylated tumor necrosis factor alpha antibody fab fragment' OR 'pegylated tumour necrosis factor alpha antibody fab fragment' OR 'pha 738144' OR 'pha738144'",6961,27 Nov 2020

#96,"'golimumab'/de OR 'cnto 148' OR 'cnto148' OR 'golimumab*' OR 'simponi' OR 'simponi aria' OR '476181 74 5' OR 91x1klu43e*",7380,27 Nov 2020

#95,"adalimumab*:ti,ab,kw,rn,tn OR fys6t7f842*:ti,ab,kw,rn,tn OR '331731 18 1':ti,ab,kw,rn,tn OR d2e7:ti,ab,kw,rn,tn OR 'hsdb 7851':ti,ab,kw,rn,tn OR hsdb7851:ti,ab,kw,rn,tn OR 'bcd-057':ti,ab,kw,rn,tn OR bcd057:ti,ab,kw,rn,tn OR 'chs-1420':ti,ab,kw,rn,tn OR chs1420:ti,ab,kw,rn,tn OR 'sb-5':ti,ab,kw,rn,tn OR sb5:ti,ab,kw,rn,tn",35470,27 Nov 2020

#94,"'adalimumab'/de OR 'abp 501' OR 'abp501' OR 'abrilada' OR 'abt d2e7' OR 'abtd2e7' OR 'adalimumab' OR 'adalimumab adaz' OR 'adalimumab adbm' OR 'adalimumab afzb' OR 'adalimumab atto' OR 'adalimumab beta' OR 'adalimumab bwwd' OR 'adalimumab-adaz' OR 'adalimumab-adbm' OR 'adalimumab-afzb' OR 'adalimumab-atto' OR 'adalimumab-bwwd' OR 'adaly' OR 'amgevita' OR 'amjevita' OR 'amsparity' OR 'avt 02' OR 'avt02' OR 'bat 1406' OR 'bat1406' OR 'bax 2923' OR 'bax 923' OR 'bax2923' OR 'bax923' OR 'bi 695501' OR 'bi695501' OR 'chs 1420' OR 'chs1420' OR 'cinnora' OR 'ct p17' OR 'ctp17' OR 'cyltezo' OR 'da 3113' OR 'da3113' OR 'dmb 3113' OR 'dmb3113' OR 'exemptia' OR 'fkb 327' OR 'fkb327' OR 'fyzoclad' OR 'gp 2017' OR 'gp2017' OR 'hadlima' OR 'halimatoz' OR 'hefiya' OR 'hlx 03' OR 'hlx03' OR 'hulio' OR 'humira' OR 'hyrimoz' OR 'ibi 303' OR 'ibi303' OR 'idacio' OR 'imraldi' OR 'kromeya' OR 'lu 200134' OR 'lu200134' OR 'm 923' OR 'm923' OR 'mabura' OR 'monoclonal antibody d2e7' OR 'msb 11022' OR 'msb11022' OR 'ons 3010' OR 'ons3010' OR 'pf 06410293' OR 'pf 6410293' OR 'pf06410293' OR 'pf6410293' OR 'raheara' OR 'solymbic' OR 'trudexa' OR 'zrc 3197' OR 'zrc3197'",35401,27 Nov 2020

#93,"etanercept*:ti,ab,kw,tn,rn OR 'rhu tnfr fc':ti,ab,kw,tn,rn OR 'tnfr-immunoadhesin':ti,ab,kw,tn,rn OR 'tnt receptor fusion protein':ti,ab,kw,tn,rn OR 'tum$or necrosis factor receptor fc fusion protein':ti,ab,kw,tn,rn OR 'tnf receptor fusion protein':ti,ab,kw,tn,rn OR 'tnfr fc':ti,ab,kw,tn,rn OR 'recombinant human tnf':ti,ab,kw,tn,rn OR 'recombinant human dimeric tnf receptor type ii igg fusion protein':ti,ab,kw,tn,rn OR 'dwp-422':ti,ab,kw,tn,rn OR dwp422:ti,ab,kw,tn,rn OR 'hd-203':ti,ab,kw,tn,rn

OR hd203:ti,ab,kw,tn,rn OR 'lbec 0101':ti,ab,kw,tn,rn OR lbec0101:ti,ab,kw,tn,rn OR 'sb 4':ti,ab,kw,tn,rn OR 'sb4':ti,ab,kw,tn,rn",16030,27 Nov 2020

#92,"'etanercept'/de OR 'avent' OR 'benepali' OR 'brenzys' OR 'chs 0214' OR 'chs0214' OR 'embrel' OR 'enbrel' OR 'enerceptan' OR 'enia 11' OR 'enia11' OR 'erelzi' OR 'etanercept*' OR 'etanercept szzs' OR 'etanercept ykro' OR 'etanercept-szzs' OR 'etanercept-ykro' OR 'eticovo' OR 'gp 2015' OR 'gp 2015c' OR 'gp2015' OR 'gp2015c' OR 'hd 203' OR 'hd203' OR 'infinitam' OR 'lbec 0101' OR 'lbec0101' OR 'lifmior' OR 'nepexto' OR 'opinercept' OR 'recombinant tumor necrosis factor receptor fc fusion protein' OR 'recombinant tumour necrosis factor receptor fc fusion protein' OR 'tnr 001' OR 'tnr001' OR 'tumor necrosis factor receptor fc fusion protein' OR 'tumour necrosis factor receptor fc fusion protein' OR 'tunex' OR 'ylb 113' OR 'ylb113' OR '185243 69 0' OR 'op401g7ojc*'",33194,27 Nov 2020

#91,"ifx:ti,ab,kw,de,tn,rn OR 'mab ca2':ti,ab,kw,de,tn,rn OR 'monoclonal antibody ca2':ti,ab,kw,de,tn,rn OR 'antibody ca2 monoclonal':ti,ab,kw,de,tn,rn OR 'ca2 monoclonal antibody':ti,ab,kw,de,tn,rn OR 'ct p13':ti,ab,kw,de,tn,rn OR ctp13:ti,ab,kw,de,tn,rn OR 'ct-p-13':ti,ab,kw,de,tn,rn OR sb2:ti,ab,kw,de,tn,rn OR 'hsdb 7850':ti,ab,kw,de,tn,rn OR hsdb7850:ti,ab,kw,de,tn,rn",5765,27 Nov 2020

#90,"'infliximab'/de OR 'abp 710' OR 'abp710' OR 'avakine' OR 'avsola' OR 'flixabi' OR 'gp 1111' OR 'gp1111' OR 'inflectra' OR 'infliximab*' OR 'infliximab abda' OR 'infliximab axxq' OR 'infliximab dyyb' OR 'infliximab qbtx' OR 'infliximab-abda' OR 'infliximab-axxq' OR 'infliximab-dyyb' OR 'infliximab-qbtx' OR 'ixifi' OR 'pf 06438179' OR 'pf 6438179' OR 'pf06438179' OR 'pf6438179' OR 'remicade' OR 'remsima' OR 'renflexis' OR 'revellex' OR 'ta 650' OR 'ta650' OR 'zessly' OR '170277 31 3' OR 'b72hh48flu*' OR 'inflecta'",52777,27 Nov 2020

#89,"((('il-1ra' OR il1ra OR 'il-1-ra') NEAR/10 (antagonist* OR 'blocking agent*' OR blocker* OR inhibitor* OR anakinra*)):ti,ab,kw,tn) AND (therap*:ti,ab,kw,de OR treat*:ti,ab,kw,de OR trial$:ti,ab,kw,de)",2221,27 Nov 2020

#88,"'anakinra'/de OR 'anakinra*' OR 'kineret' OR 'recombinant interleukin 1 receptor antagonist' OR 'recombinant interleukin 1 receptor blocker' OR 'recombinant interleukin 1 receptor blocking agent' OR '143090 92 0'",9249,27 Nov 2020

#87,"#76 AND #86",4583,27 Nov 2020

#86,"#77 OR #78 OR #79 OR #80 OR #81 OR #82 OR #83 OR #84 OR #85",30224,27 Nov 2020

#85,"'elsilimomab'/de OR 'b e8' OR 'elsilimomab*' OR '468715 71 1'",38,27 Nov 2020

#84,"'ziltivekimab'/de OR 'cor 001' OR 'cor001' OR 'ziltivekimab*' OR ziltivekimab* OR zilti OR '2226654 05 1'",4,27 Nov 2020

#83,"'siltuximab'/de OR 'cnto 328' OR 'cnto328' OR 'monoclonal antibody cnto 328' OR 'monoclonal antibody cnto328' OR 'siltuximab*' OR 'sylvant' OR cllb8 OR 'ccib8 monoclonal antibody' OR t4h8fma7im* OR '541502 14 1'",856,27 Nov 2020

#82,"'sirukumab'/exp OR 'cnto 136' OR 'cnto136' OR 'plivensia' OR 'sirukumab*' OR '1194585 53 9' OR 640443fu93*",320,27 Nov 2020

#81,"'clazakizumab'/de OR 'ald 518' OR 'ald518' OR 'bms 945429' OR 'bms945429' OR 'clazakizumab*' OR '1236278 28 6' OR 4s38z8ra9o*",217,27 Nov 2020

#80,"'olokizumab'/de OR 'olokizumab*' OR 'cdp 6038' OR cdp6038 OR '1007223 17 7' OR pai71r1d2w*",95,27 Nov 2020

#79,"'levilimab'/de OR 'bcd 089' OR 'bcd089' OR 'levilimab*' OR '2035008 70 7' OR p7uv3l2h80*",5,27 Nov 2020

#78,"'sarilumab'/de OR 'kevzara*' OR 'regn 88' OR 'regn88' OR 'sar 153191' OR 'sar153191' OR 'sarilumab*' OR '1189541 98 7' OR nu90v55f8i*",816,27 Nov 2020

#77,"'tocilizumab'/de OR 'actemra*' OR 'actemra 200' OR 'atlizumab*' OR 'lusinex*' OR 'r1569' OR 'roactemra*' OR 'tocilizumab*' OR r1569 OR 'r 1569' OR 'mra':ti,ab,kw,tn OR mra:tt OR '375823 41 9' OR i031v2h011* OR 'rhpm-1' OR rhpm1 OR 'rg-1569' OR rg1569 OR 'msb-11456' OR msb11456 OR 'ro-4877533' OR ro4877533",29142,27 Nov 2020

#76,"#1 OR #2 OR #3 OR #4 OR #5 OR #6 OR #7 OR #8 OR #9 OR #10 OR #13 OR #14 OR #15 OR #16 OR #17 OR #18 OR #19 OR #20 OR #21 OR #22 OR #23 OR #24 OR #25 OR #26 OR #27 OR #28 OR #29 OR #30 OR #31 OR #32 OR #33 OR #34 OR #35 OR #36 OR #37 OR #38 OR #39 OR #40 OR #41 OR #42 OR #43 OR #44 OR #45 OR #46 OR #47 OR #48 OR #49 OR #50 OR #51 OR #52 OR #53 OR #54 OR #55 OR #56 OR #57 OR #58 OR #59 OR #60 OR #61 OR #62 OR #63 OR #64 OR #65 OR #66 OR #67 OR #68 OR #69 OR #70 OR #71 OR #72 OR #73 OR #74 OR #75",368076,27 Nov 2020

#75,"((axial NEAR/3 spondyl$arthrit*):ti,ab,kw) OR ((axial NEAR/3 spondyl$arthrit*):tt)",3877,27 Nov 2020

#74,"axspa*:ti,ab,kw OR axspa*:tt",2558,27 Nov 2020

#73,"'bath ankylosing spondylitis disease activity index'/de",4414,27 Nov 2020

#72,"'rheumat* spondylit*':tt OR 'axial spondyl$arthrit*':tt OR 'bechterew* disease*':tt OR 'bechterew s disease*':tt OR 'bechterews disease*':tt OR 'marie struempell disease*':tt",8,27 Nov 2020

#71,"'rheumat* spondylit*':ti,ab,kw OR 'axial spondyl$arthrit*':ti,ab,kw OR 'bechterew* disease*':ti,ab,kw OR 'bechterew s disease*':ti,ab,kw OR 'bechterews disease*':ti,ab,kw OR 'marie struempell disease*':ti,ab,kw",4416,27 Nov 2020

Kastrati K, et al. RMD Open 2022; 8:e002359. doi: 10.1136/rmdopen-2022-002359

#70,"((spondylitis* OR spondylarthrit* OR spondyloarthrit* OR spine OR vertebral) NEAR/1 ankyl*):tt",1139,27 Nov 2020

#69,"((spondylitis* OR spondylarthrit* OR spondyloarthrit* OR spine OR vertebral) NEAR/1 ankyl*):ti,ab,kw",25483,27 Nov 2020

#68,"'ankylosing spondylitis disease activity score'/de",1891,27 Nov 2020

#67,"'ankylosing spondylitis'/de OR 'bechterew disease' OR 'ankylating spondylitis' OR 'ankylopoietic spondylarthritis' OR 'ankylopoietic spondylitis' OR 'ankylosing spine' OR 'ankylosing spondilitis' OR 'ankylosing spondylarthritis' OR 'ankylosing spondylarthrosis' OR 'ankylosing spondylitis' OR 'ankylosis spondylitis' OR 'ankylotic spondylitis' OR 'bekhterev disease' OR 'morbus bechterew' OR 'spinal ankylosis' OR 'spine ankylosis' OR 'spondylarthritis ankylopoietica' OR 'spondylarthritis ankylosans' OR 'spondylarthrosis ankylopoietica' OR 'spondylitis ankylopoetica' OR 'spondylitis ankylopoietica' OR 'spondylitis, ankylosing' OR 'spondyloarthritis ankylopoietica' OR 'vertebral ankylosis'",35176,27 Nov 2020

#66,"'ankylosing spondylitis'/de",29599,27 Nov 2020

#65,"((psoria* NEXT/6 ('inflammat* arthrit*' OR 'inflammat* polyarthrit*')):ti,ab,kw) OR ((psoria* NEXT/6 ('inflammat* arthrit*' OR 'inflammat* polyarthrit*')):tt)",323,27 Nov 2020

#64,"((psoria* NEXT/1 rheumat*):ti,ab,kw) OR ((psoria* NEXT/1 rheumat*):tt)",388,27 Nov 2020

#63,"((psoria* NEXT/2 arthrit*):tt) OR ((psoria* NEXT/2 polyarthrit*):tt) OR ((psoria* NEXT/2 arthropath*):tt) OR ((psoria* NEXT/2 'poly arthrit*'):tt)",184,27 Nov 2020

#62,"((psoria* NEXT/2 arthrit*):ti,ab,kw) OR ((psoria* NEXT/2 polyarthrit*):ti,ab,kw) OR ((psoria* NEXT/2 arthropath*):ti,ab,kw) OR ((psoria* NEXT/2 'poly arthrit*'):ti,ab,kw)",20191,27 Nov 2020

#61,"'psoriatic arthritis'/exp OR 'alibert bazin disease' OR 'arthritis psoriatica' OR 'arthritis, psoriasis' OR 'arthritis, psoriatic' OR 'arthropathic psoriasis' OR 'arthropathy, psoriatic' OR 'disease, alibert bazin' OR 'polyarthritis, psoriatic' OR 'psoriasis arthropathica' OR 'psoriasis pustulosa arthropathica' OR 'psoriasis, arthritis' OR 'psoriatic arthritis' OR 'psoriatic arthropathy' OR 'psoriatic polyarthritis' OR 'psoriatic rheumatism' OR 'psoriatic rheumatoid arthritis' OR 'rheumatoid arthritis, psoriatic'",26345,27 Nov 2020
#60,"(('pauci immune*' NEAR/6 vasculit*):ti,ab,kw) OR (('pauci immune*' NEAR/6 vasculit*):tt)",266,27 Nov 2020
#59,"((anca OR 'antineutrophil cytoplasmic' OR 'anti-neutrophil cytoplasmic') NEAR/6 vasculit*):tt",66,27 Nov 2020
#58,"((anca OR 'antineutrophil cytoplasmic' OR 'anti-neutrophil cytoplasmic') NEAR/6 vasculit*):ti,ab,kw",8172,27 Nov 2020
#57,"'anca associated vasculitis'/de OR 'anca associated vasculitis' OR 'anca vasculitis' OR 'anca-associated vasculitis' OR 'anti neutrophil cytoplasmic antibody associated vasculitis' OR 'anti-neutrophil cytoplasmic antibody-associated vasculitis' OR 'antineutrophil cytoplasmic antibody associated vasculitis' OR 'neutrophil cytoplasmic antibody associated vasculitis'",7508,27 Nov 2020
#56,"'myeloopticoneuropath*':tt OR 'myelooptic* neuropath*':tt OR neuroopticomyelit*:tt",8,27 Nov 2020
#55,"'myeloopticoneuropath*':ti,ab,kw OR 'myelooptic* neuropath*':ti,ab,kw OR neuroopticomyelit*:ti,ab,kw",167,27 Nov 2020
#54,"((devic OR devics OR 'devic s') NEAR/2 (disease$ OR syndrom* OR 'neuromyelit* optic*')):tt",10,27 Nov 2020

#53,"((devic OR devics OR 'devic s') NEAR/2 (disease$ OR syndrom* OR 'neuromyelit* optic*')):ti,ab,kw",769,27 Nov 2020

#52,"((nmo:ti,ab,kw OR nmosd:ti,ab,kw) AND (neuromyelit*:ti,ab,kw OR neuropath*:ti,ab,kw,de) OR nmo:ti,ab,kw OR nmosd:tt) AND (neuromyelit*:ti,ab,kw OR neuropath*:ti,ab,kw,de)",6137,27 Nov 2020

#51,"((neuromyelit* NEAR/1 optic*):ti,ab,kw) OR ((neuromyelit* NEAR/1 optic*):tt)",8308,27 Nov 2020

#50,"'myelooptic neuropathy'/de OR 'devic disease' OR 'devic syndrome' OR 'myelooptic neuropathy' OR 'myeloopticoneuropathy' OR 'myeloptico neuropathy' OR 'myelopticoneuropathy' OR 'neuromyelitis optica' OR 'neuromyelitis optica spectrum disorder' OR 'neuropticomyelitis' OR 'optic neuromyelitis'",10191,27 Nov 2020

#49,"('neonatal onset' NEAR/3 'multisystem* inflammat*' NEAR/1 (disease* OR disorder*)):ti,ab,kw",264,27 Nov 2020

#48,"(('chronic infantile' NEAR/3 'neurolog* cutaneous' NEAR/3 ('articular* syndrom*' OR arthropath*)):ti,ab,kw) OR (('chronic infantile' NEAR/3 'neurolog* cutaneous' NEAR/3 ('articular* syndrom*' OR arthropath*)):tt)",231,27 Nov 2020

#47,"'cinca syndrome'/exp OR 'cinca' OR 'cinca syndrome' OR 'nomid' OR 'chronic infantile neurologic cutaneous and articular syndrome' OR 'chronic infantile neurologic cutaneous articular syndrome' OR 'chronic infantile neurologic, cutaneous and articular syndrome' OR 'chronic infantile neurologic, cutaneous, articular syndrome' OR 'chronic infantile neurological cutaneous and articular syndrome' OR 'chronic infantile neurological cutaneous arthropathy' OR 'chronic infantile neurological cutaneous articular syndrome' OR 'chronic infantile neurological, cutaneous and articular syndrome' OR 'chronic infantile neurological, cutaneous, articular syndrome' OR 'neonatal onset multisystem inflammatory

disease' OR 'neonatal onset multisystem inflammatory disorder' OR 'neonatal onset multisystemic inflammatory disease' OR 'neonatal onset multisystemic inflammatory disorder'",2214,27 Nov 2020
#46,"(('tnf receptor-associated periodic' OR 'tnfr1 associated' OR 'tumo$r necrosis factor receptor associated' OR 'tnfr associated') NEAR/3 (syndrome* OR fever)):tt",2,27 Nov 2020
#45,"(('tnf receptor-associated periodic' OR 'tnfr1 associated' OR 'tumo$r necrosis factor receptor associated' OR 'tnfr associated') NEAR/3 (syndrome* OR fever)):ti,ab,kw",713,27 Nov 2020
#44,"(traps:ti,ab,kw OR traps:tt) AND ('tnf receptor':ti,ab,kw,de OR 'tumo$r necrosis factor receptor':ti,ab,kw,de)",695,27 Nov 2020
#43,"'tumor necrosis factor receptor associated periodic syndrome'/de OR 'tnf receptor 1 associated periodic syndrome' OR 'tnf receptor associated periodic syndrome' OR 'tnf receptor superfamily 1a associated periodic syndrome' OR 'tnfr1 associated periodic syndrome' OR 'tnfr associated periodic syndrome' OR 'traps (tumor necrosis factor receptor associated periodic syndrome)' OR 'traps (tumour necrosis factor receptor associated periodic syndrome)' OR 'autosomal dominant periodic fever with amyloidosis' OR 'benign autosomal dominant familial periodic fever' OR 'familial hibernian fever' OR 'tumor necrosis factor receptor 1 associated periodic syndrome' OR 'tumor necrosis factor receptor associated periodic fever' OR 'tumor necrosis factor receptor associated periodic fever syndrome' OR 'tumor necrosis factor receptor associated periodic syndrome' OR 'tumor necrosis factor receptor superfamily 1a associated periodic syndrome' OR 'tumour necrosis factor receptor 1 associated periodic syndrome' OR 'tumour necrosis factor receptor associated periodic fever' OR 'tumour necrosis factor receptor associated periodic fever syndrome' OR 'tumour necrosis factor receptor associated periodic syndrome' OR 'tumour necrosis factor receptor superfamily 1a associated periodic syndrome'",1124,27 Nov 2020

#42,"(('von meyenburg' NEAR/3 disease*):ti,ab,kw) OR (('von meyenburg' NEAR/3 disease*):tt)",13,27 Nov 2020

#41,"((chondromalac* NEAR/3 systemic*):ti,ab,kw) OR ((chondromalac* NEAR/3 systemic*):tt)",5,27 Nov 2020

#40,"((polychondrit* NEAR/3 (relapsing* OR atrophic*)):ti,ab,kw) OR ((polychondrit* NEAR/3 (relapsing* OR atrophic*)):tt)",1962,27 Nov 2020

#39,"'relapsing polychondritis'/de",2112,27 Nov 2020

#38,"((amyloid* NEAR/3 (neuropath* OR tumo$r* OR angiopath* OR senile*)):ti,ab,kw) OR ((amyloid* NEAR/3 (neuropath* OR tumo$r* OR angiopath* OR senile*)):tt)",8207,27 Nov 2020

#37,"'amyloidos$s':ti,ab,kw OR amyloidoma*:ti,ab,kw OR 'beta fibrillos$s':ti,ab,kw OR paraamyloidos$s:ti,ab,kw OR 'amyloidos$s':tt OR amyloidoma*:tt OR 'beta fibrillos$s':tt OR paraamyloidos$s:tt",34314,27 Nov 2020

#36,"'amyloidosis'/exp",51266,27 Nov 2020

#35,"((infectious* OR focal OR proliferativ*) NEAR/3 myopath*):ti,ab,kw",165,27 Nov 2020

#34,"((inflammat* NEAR/3 idiopath* NEAR/3 (myopath* OR myosit*)):ti,ab,kw) OR ((inflammat* NEAR/3 idiopath* NEAR/3 (myopath* OR myosit*)):tt)",2724,27 Nov 2020

#33,"((inflammat* NEAR/1 (myopath* OR muscle$)):ti,ab,kw) OR ((inflammat* NEAR/1 (myopath* OR muscle$)):tt)",6811,27 Nov 2020

#32,"myositis:ti,ab,kw OR neuromyositis:ti,ab,kw OR myositis:tt OR neuromyositis:tt",15287,27 Nov 2020

#31,"'myositis'/exp",41179,27 Nov 2020

#30,"'libman sacks endocarditis'/de",112,27 Nov 2020

#29,"'libman sacks disease'/de",24,27 Nov 2020

#28,"'libman sacks disease*':ti,ab,kw OR 'libman sacks disease*':tt",19,27 Nov 2020

#27,"(sle:ti,ab,kw OR 's l e':ti,ab,kw OR sle:tt OR 's l e':tt) AND lupus",52367,27 Nov 2020

#26,"(('lupus erythemato*' NEAR/3 (systemic* OR disseminat* OR visceral*)):ti,ab,kw) OR (('lupus erythemato*' NEAR/3 (systemic* OR disseminat* OR visceral*)):tt)",77370,27 Nov 2020

#25,"'systemic lupus erythematosus'/exp",98941,27 Nov 2020

#24,"(('remitting seronegative' NEAR/3 synovitis NEAR/6 ('edem*' OR 'oedem*')):ti,ab,kw) OR (('remitting seronegative' NEAR/3 synovitis NEAR/6 ('edem*' OR 'oedem*')):tt)",255,27 Nov 2020

#23,"rs3pe*:ti,ab,kw OR rs3pe*:tt",242,27 Nov 2020

#22,"'rs3pe syndrome'/de",15,27 Nov 2020

#21,"'remitting seronegative symmetrical synovitis with pitting edema syndrome'/de",42,27 Nov 2020

#20,"'remitting seronegative symmetrical synovitis with pitting edema'/de",67,27 Nov 2020

#19,"(('forestier-certonciny' NEAR/1 syndrom*):ti,ab,kw) OR (('forestier certonciny' NEAR/1 syndrom*):tt)",2,27 Nov 2020

#18,"(((pseudopolyarthrit* OR 'pseudo polyarthrit*' OR 'inflammat* rheumat*' OR 'rheumat* inflammat*') NEAR/1 rhizomelic):ti,ab,kw) OR (((pseudopolyarthrit* OR 'pseudo polyarthrit*' OR 'inflammat* rheumat*' OR 'rheumat* inflammat*') NEAR/1 rhizomelic):tt)",88,27 Nov 2020

#17,"(((rheumat* OR arteritic*) NEAR/1 (polymyalgia* OR myalgia*)):ti,ab,kw) OR (((rheumat* OR arteritic*) NEAR/1 (polymyalgia* OR myalgia*)):tt)",4334,27 Nov 2020

#16,"'rheumatic polymyalgia'/de",5810,27 Nov 2020

#15,"('systemic scleros$s*':ti,ab,kw OR 'systemic scleros$s*':tt OR ssc:ti,ab,kw) AND ('interstitial lung disease$':ti,ab,kw,de OR 'interstitial pneumoni*':ti,ab,kw,de OR 'interstitial lung disease$':tt OR 'interstitial pneumoni*':tt)",3841,27 Nov 2020

#14,"'ssc-ild*':ti,ab,kw OR 'sscild*':ti,ab,kw",789,27 Nov 2020

#13,"#11 AND #12",4503,27 Nov 2020

#12,"'interstitial lung disease'/exp",85995,27 Nov 2020

#11,"'systemic sclerosis'/exp",33079,27 Nov 2020

#10,"'severe acute respiratory syndrome coronavirus 2':tt OR 'sars coronavirus 2':tt OR 'sars-cov-2*':tt OR 'sars-cov2*':tt OR 'sars-covid-2*':tt OR sarscov2*:tt OR 'sars-2-cov*':tt",665,27 Nov 2020

#9,"'severe acute respiratory syndrome coronavirus 2':ti,ab,kw OR 'sars coronavirus 2':ti,ab,kw OR 'sars-cov-2*':ti,ab,kw OR 'sars-cov2*':ti,ab,kw OR 'sars-covid-2*':ti,ab,kw OR sarscov2*:ti,ab,kw OR 'sars-2-cov*':ti,ab,kw",24248,27 Nov 2020

#8,"'severe acute respiratory syndrome coronavirus 2'/exp",17605,27 Nov 2020

#7,"((covid OR ncov OR 'n-cov' OR cov OR coronavir* OR 'corona vir*' OR betacoronavir*) NEAR/3 ('19$' OR '2019*' OR wuhan OR novel* OR new OR newly)):tt",3083,27 Nov 2020

#6,"((covid OR ncov OR 'n-cov' OR cov OR coronavir* OR 'corona vir*' OR betacoronavir*) NEAR/3 ('19$' OR '2019*' OR wuhan OR novel* OR new OR newly)):ti,ab,kw",68310,27 Nov 2020

#5,"'2019-ncov*':tt OR '2019-n-cov*':tt OR 2019ncov*:tt OR '2019-novel-cov*':tt OR ncov2019*:tt OR 'n-cov2019*':tt OR 'ncov-2019*':tt OR 'n-cov-2019*':tt OR 'novel cov-2019*':tt OR 'novel cov-19*':tt OR ncov19*:tt OR 'n-cov19*':tt OR 'ncov-19*':tt OR 'n-cov-19*':tt OR 'hcov-19':tt",21,27 Nov 2020

#4,"'2019-ncov*':ti,ab,kw OR '2019-n-cov*':ti,ab,kw OR 2019ncov*:ti,ab,kw OR '2019-novel-cov*':ti,ab,kw OR ncov2019*:ti,ab,kw OR 'n-cov2019*':ti,ab,kw OR 'ncov-2019*':ti,ab,kw OR 'n-cov-

2019*':ti,ab,kw OR 'novel cov-2019*':ti,ab,kw OR 'novel cov-19*':ti,ab,kw OR ncov19*:ti,ab,kw OR 'n-cov19*':ti,ab,kw OR 'ncov-19*':ti,ab,kw OR 'n-cov-19*':ti,ab,kw OR 'hcov-19':ti,ab,kw",1407,27 Nov 2020

#3,"'covid-19*':tt OR 'covid19*':tt OR 'covid-2019*':tt OR 'covid2019*':tt",3068,27 Nov 2020

#2,"'covid-19*':ti,ab,kw OR 'covid19*':ti,ab,kw OR 'covid-2019*':ti,ab,kw OR 'covid2019*':ti,ab,kw",64846,27 Nov 2020

#1,"'coronavirus disease 2019'/de",65249,27 Nov 2020

1.3.4.3 Cochrane CENTRAL

1 exp Coronavirus/ (10)

2 exp Coronavirus Infections/ (513)

3 (coronavirus* or corona virus* or OC43 or NL63 or 229E or HKU1 or HCoV* or ncov* or covid* or sars-cov* or sarscov* or Sars-coronavirus* or Severe Acute Respiratory Syndrome Coronavirus*).mp. (4896)

4 (or/1-3) and (20191* or 202*).up. (3779)

5 4 not (SARS or SARS-CoV or MERS or MERS-CoV or Middle East respiratory syndrome or camel* or dromedar* or equine or coronary or coronal or covidence* or covidien or influenza virus or HIV or bovine or calves or TGEV or feline or porcine or BCoV or PED or PEDV or PDCoV or FIPV or FCoV or SADS-CoV or canine or CCov or zoonotic or avian influenza or H1N1 or H5N1 or H5N6 or IBV or murine corona*).mp. (2117)

6 ((pneumonia or covid* or coronavirus* or corona virus* or ncov* or 2019-ncov or sars*).mp. or exp pneumonia/) and Wuhan.mp. (148)

7 (2019-ncov* or 2019-n-cov*or 2019ncov* or 2019n-cov* or ncov19 or ncov-19 or 2019-novel CoV*or ncov2019* or n-cov2019* or ncov-2019* or n-cov-2019* or novel cov-2019* or novel-cov-19* or ncov19* or n-cov19* or ncov-19* or n-cov-19* or sars-cov2* or sars-cov-2* or sarscov2* or sarscov-2* or sars-covid-2* or sars-2-cov* or Sars-coronavirus2* or Sars-coronavirus-2* or SARS-like coronavirus* or coronavirus-19 or covid19 or covid-19 or covid 2019 or ((novel or new or newly or nouveau or "19?" or "2019*" or wuhan) adj2 (CoV on nCoV or n-cov or covid or coronavirus* or corona virus or betacoronavirus* or Pandemi*2)) or ((covid or covid19 or covid-19) and pandemic*2) or (coronavirus* and pneumonia)).mp. (3582)

8 (severe acute respiratory syndrome coronavirus 2* or severe acute respiratory syndrome cov* 2* or severe acute respiratory syndrome coronavirus 2019* or severe acute respiratory syndrome cov* 2019* or severe acute respiratory syndrome coronavirus 19* or severe acute respiratory syndrome cov* 19*).mp. [mp=title, original title, abstract, mesh headings, heading words, keyword] (252)

9 6 or 7 or 8 (3585)

10 5 or 9 (3676)

11 10 and (20191* or 202*).up. (3649)

12 exp Lung Diseases, Interstitial/ (824)

13 exp Scleroderma, Systemic/ (559)

14 12 and 13 (40)

15 (((systemic adj3 (sclerosis* or sleroderma*)) or ssc) and (interstitial adj3 (lung disease* or pneumoni*))).mp. (255)

16 (ssc-ild* or sscild*).ti,ot,ab. (141)

17 Polymyalgia Rheumatica/ (81)

18 pmr.ti,ot,ab. and rheum*.mp. [mp=title, original title, abstract, mesh headings, heading words, keyword] (106)

19 ((rheumat* or arteritic*) adj1 (polymyalgia* or myalgia*)).mp. (224)

20 ((pseudopolyarthrit* or pseudo polyarthrit* or inflammat* rheumat* or rheumat* inflammat*) adj1 rhizomelic).mp. (0)

21 (forestier-certonciny adj1 syndrom*).mp. (0)

22 (remitting adj3 seronegative adj3 synovitis adj6 (edem* or oedem*)).mp. (1)

23 rs3pe*.ti,ot,ab. (1)

24 exp Synovitis/ (186)

25 Edema/ (1773)

26 24 and 25 (7)

27 (remitting or seronegativ* or symmetric*).mp. (7459)

28 26 and 27 (0)

29 exp Lupus Erythematosus, Systemic/ (1050)

30 (lupus erythemato* adj3 (systemic* or disseminat* or visceral*)).mp. (2518)

31 (sle or 's l e').ti,ot,ab. (2010)

32 (lupus or autoimmun* or auto immun* or autoantibod* or antibod* or inflamm* or immun* or rheumat* or arthrit* or nephritis or nephrol* or cutan* or dermatol* or venerol* or neuromyotoni* or neuroscience? or neurolog* or patient? or cell debris).mp,jw. (1135159)

33 31 and 32 (1981)

34 (libman sacks adj1 (disease* or endocarditis*)).mp. (3)

35 exp Myositis/ (193)

36 (myositis or neuromyositis or polymyositis or dermatomyositis).mp. (870)

37 (inflammat* adj1 (myopath* or muscle? or myosit*)).mp. (361)

38 (inflammat* adj3 idiopath* adj3 (myopath* or myosit*)).mp. (78)

39 ((infect* or focal or proliferativ*) adj3 (myopath* or myosit*)).ti,ot,ab. (10)

40 exp Amyloidosis/ (165)

41 (amyloidos#s or amyloidoma* or beta fibrillos#s or paraamyloidos#s).mp. (647)

42 Polychondritis, Relapsing/ (0)

43 (polychondrit* adj3 (relapsing* or atrophic*)).mp. (22)

44 (chondromalac* adj3 systemic*).mp. (0)

45 (von meyenburg adj3 disease*).mp. (0)

46 (periodic fever adj1 familial adj1 autosomal dominant).mp. (0)

47 ((tnf receptor or tnfr1 or tnfr) adj3 associated periodic syndrom*).mp. (23)

48 (traps and (tumo?r necrosis factor receptor or tnf receptor)).ti,ot,ab. (30)

49 (tumo?r necrosis factor receptor adj3 associated periodic adj2 (syndrom* or fever)).mp. (33)

50 (autosomal dominant adj3 periodic fever).mp. (0)

51 (hibernian fever adj3 familial).mp. (0)

52 ((tnf receptor associated periodic or tnfr1 associated or tumo?r necrosis factor receptor associated or tnfr associated) adj3 (syndrom* or fever*)).mp. (33)

53 Cryopyrin-Associated Periodic Syndromes/ (20)

54 (cinca or cinca syndrom*).mp. (16)

55 (chronic infantile adj3 neurolog* cutan* adj3 (articul* syndrom* or arthropath*)).mp. (2)

56 (neonatal onset adj3 multisyst* inflamm* adj1 (diseas* or disorder*)).mp. (3)

57 nomid.ti,ot,ab. (4)

58 iomid.ti,ot,ab. (0)

59 cryopyrin associated periodic syndrom*.mp. (42)

60 Neuromyelitis Optica/ (33)

61 (neuromyelit* adj1 optic*).mp. (222)

62 (myelooptic* adj1 neuropath*).mp. (164)

63 ((nmo or nmosd) and (neuromyelit* or neuropath*)).ti,ot,ab. (179)

64 ((devic or devics or devic's) adj2 (diseas* or syndrom* or neuromyelit* optic*)).mp. (5)

65 (myeloopticoneuropath* or myelooptic* neuropath* or neuroopticomyelit*).mp. (164)

66 exp Anti-Neutrophil Cytoplasmic Antibody-Associated Vasculitis/ (121)

67 ((anca or antineutrophil cytoplasmic or anti-neutrophil cytoplasmic) adj6 vasculit*).mp. (522)

68 (pauci immun* adj6 vasculit*).ti,ot,ab. (4)

69 exp Arthritis, Psoriatic/ (445)

70 (psoria* adj2 (arthrit* or polyarthrit* or poly arthrit* or arthropath*)).ti,ot,ab. (2204)

71 (psoria* adj1 rheumat*).ti,ot,ab. (65)

72 (psoria* adj6 (inflammat* arthrit* or inflammat* polyarthrit* or inflammat* poly arthrit*)).ti,ot,ab. (43)

73 Spondylitis, Ankylosing/ (687)

74 ((spondylitis* or spondylarthrit* or spndyloarthrit* or spine or vertebral) adj1 ankyl*).mp. (2309)

75 (rheumat* spondylit* or axial spondyl?arthrit* or bechterew* disease* or bechterews disease* or bechterew's disease* or marie struempell disease*).mp. (587)

76 (axspa* or ax-spa*).ti,ot,ab. (428)

77 (axial adj3 spondyl?arthrit*).ti,ot,ab. (598)

78 11 or 14 or 15 or 16 or 17 or 18 or 19 or 20 or 21 or 22 or 23 or 28 or 29 or 30 or 33 or 34 or 35 or 36 or 37 or 38 or 39 or 40 or 41 or 42 or 43 or 44 or 45 or 46 or 47 or 48 or 49 or 50 or 51 or 52 or 53 or 54 or 55 or 56 or 57 or 58 or 59 or 60 or 61 or 62 or 63 or 64 or 65 or 66 or 67 or 68 or 69 or 70 or 71 or 72 or 73 or 74 or 75 or 76 or 77 (13874)

79 (tocilizumab* or actemra* or atlizumab* or lusinex* or r1569 or r 1569 or roactemra* or mra or 375823 41 9 or i031v2h011* or rhpm-1 or rhpm1 or rg-1569 or rg1569 or msb-11456 or msb11456 or ro-4877533 or ro4877533).mp. (1989)

80 (sarilumab* or kevzara* or regn 88 or regn88 or sar 153191 or sar153191 or 1189541 98 7 or nu90v55f8i*).mp. (255)

81 (levilimab* or BCD-089 or bcd089 or 2035008 70 7 or P7UV3L2H80*).mp. (5)

82 (olokizumab* or cdp 6038 or cdp6038 or 1007223 17 7 or pai71r1d2w*).mp. (35)

83 (clazakizumab* or ald 518 or ald518 or bms 945429 or bms945429 or 1236278 28 6 or 4s38z8ra9o*).mp. (38)

84 (sirukumab* or cnto 136 or cnto136 or pilvensia or 1194585 53 9 or 640443FU93*).mp. (118)

85 (siltuximab* or sylvant or cnto 328 or cnto-328 or cnto328 or cllb8 or ccib8 monoclonal antibody or sylvant or t4h8fma7im* or 541502 14 1).mp. (78)

86 (ziltivekimab* or zilti or "cor-001" or "2226654 05 1").mp. (5)

87 (elsilimomab* or "b-e8" or 468715 71 1).mp. (2)

88 79 or 80 or 81 or 82 or 83 or 84 or 85 or 86 or 87 (2464)

89 78 and 88 (282)

90 Interleukin 1 Receptor Antagonist Protein/ (305)

91 (anakinra* or kineret or ((interleukin 1 receptor or il1 receptor or il 1 receptor) adj1 antagonist protein*) or (recombinant interleukin 1 receptor adj1 (antagonist* or blocker* or blocking agent*)) or antril or il 1ra or il1ra or il 1 ra or ((urin* or febrile) adj2 (interleukin 1 inhibitor* or il1 inhibitor* or il 1 inhibitor*)) or "143090 92 0" or 9013duq28k*).mp. (1087)

92 Infliximab/ (732)

93 (infliximab* or ifx or avakine or flixabi or inflectra or inflecta or ixifi or remicade or remsima or revellex or renflexis or zessly or mab ca2 or monoclonal antibody ca2 or antibody ca2 monoclonal or ca2 monoclonal antibody or ct p13 or ctp13 or ct-p-13 gp-1111 or gp1111 or ta-650 or ta650 or abp 710 or abp710 or bow-015 or bow015 or 170277 31 3 or b72hh48flu* or sb2 or "pf 06438179" or pf 6438179 or pf06438179 or pf6438179 or hsdb 7850 or hsdb7850).mp. (2769)

94 Etanercept/ (758)

95 (etanercept* or benepali or embrel or enbrel or enia 11 or enia11 or erelzi or lifmior or opinercept or recombinant tumo?r necrosis factor receptor fc fusion protein or "tnr 001" or tnr001 or tnfr-fc or tnrfr:fc or rhu tnfr:fc or rhu-tnfr:fc or tnfr-immunoadhesin or tnt receptor fusion protein or tum?or necrosis factor receptor fc fusion protein or tunex or "185243 69 0" or op401g7ojc* or brenzys or tnf receptor fusion protein or tnfr fc or recombinant human tnf or recombinant human dimeric tnf receptor type ii igg fusion protein or chs-0214 or chs0214 or dwp-422 or dwp422 or enia-11 or enia11 or gp-2015 or gp2015 or gp2015c or gp-2015c or hd-203 or hd203 or lbec-0101 or lbec0101 or sb-4 or sb4).mp. (2451)

96 Adalimumab/ (752)

97 (adalimumab* or humira or abp 501 or abp501 or abtd2e7 or amjevita or amgevita or bax 2923 or bax2923 gp 2017 or gp2017 or halimatoz or hefiya or hulio or hyrimoz or ibi 303 or ibi303 or imraldi or m 923 or m923 or msb 11022 or msb11022 or ons3010 or ons 3010 or "pf 06410293" or pf 6410293 or pf06410293 or pf6410293 or d2e7 or truxeda or cyltezo or solymbic or fys6t7f842* or 331731 18 1 or lu 200134 or lu200134 or d2e7 or hsdb 7851 or hsdb7851 or abp-501 or abp501 or bcd-057 or bcd057 or bi-695501 or bi695501 or chs-1420 or chs1420 or gp-2017 or gp2017 or sb-5 or sb5).mp. (3403)

98 (golimumab* or cnto 148 or cnto148 or simponi or 476181 74 5 or 91x1klu43e*).mp. (751)

99 Certolizumab Pegol/ (167)

100 (certolizumab* or cdp 870 or cdp870 or cimzia or pegylated tumor necrosis factor alpha antibody fab fragment or pegylated tumour necrosis factor alpha antibody fab fragment or pha 738144 or pha738144 or czp or 428863 50 7 or g6adw90r16* or umd07x179e* or hsdb 7848 or hsdb7848).mp. (705)

101 Rituximab/ (1268)

102 (rituximab* or ct p10 or ctp10 or idec 102 or idec102 or idecc2b8 or idec c2b8 or monoclonal antibody idec c2b8 or gp2013 or gp 2013 or "pf 05280586" or pf05280586 or mabthera or mab thera or r 105 or r105 or reditux or rg 105 or rg105 or rituxan or rituxin or ritemvia or rituzena or rixathon or riximyo or ro 452294 or ro452294 or truxima or 174722 31 7 or 4f4x42syq6* or hsdb 7455 or hsdb7455).mp. (5326)

103 (ofatumumab* or humax cd20 or humax-cd20 or humaxcd20 or humax-cd20-2f2 or arzerra or gsk 1841157 or gsk1841157 or gski841157 or humax cd20 or omb 157 or omb157 or 679818 59 8 or m95kg522r0* or hsdb 8170 or hsdb8170).mp. (298)

104 Abatacept/ (280)

105 (abatacept* or ctla4 ig or ctla4 immunglobulin or ctla4 immunoglobulin g or ctla4ig or 'ctla 4 ig' or ctla-igg4m or ctla4-fc or bms 188667 or bms188667 or orencia or '332348 12 6' or 7d0yb67s97* or rg 2077 or rg2077 or rg-1046 or rg1046 or nulojix or lea29y or lea 29y or ctl4 fc or ctl4fc or belatacept or bms 224818 or bms224818 or belatacept or cytotoxic t lymphocyte associat* antigen 4).mp. (1151)

106 (tabalumab* or ly 2127399 or ly2127399 or 1143503 67 6 or pqp8vh3mjw*).mp. (70)

107 (pateclizumab* or mlta 3698a or mlta3698a or pro 283698 or pro283698 or 12202526 59 7 or qok1yyh7j2* or rg 7415 or rg7415).mp. (7)

108 (secukinumab* or cosentyx or ain 457 or ain457 or 1229022 83 6 or dlg4eml025*).mp. (917)

109 (ixekizumab* or ly 2439821 or ly2439821 or taltz or 1329632 62 3 or 1143503 69 8 or bty153760o*).mp. (489)

110 (brodalumab* or amg 827 or amg827 or khk 4827 or khk4827 or siliq or kyntheum or 1174395 19 7 or 6za31y954z*).mp. (178)

111 (guselkumab* or cnto 1959 or cnto1959 or 1350289 85 8 or tremfya or 1350289 85 8 or 089658a12d*).mp. (227)

112 Ustekinumab/ (195)

113 (ustekinumab* or cnto 1275 or cnto1275 or stelara or "815610 63 0" or fu77b4u5z0* or 1275 cnto or 1275cnto or l04ac05 or tt-20 or tt20).mp. (897)

114 (mavrilimumab or "1085337 57 0" or 1158jdp9a* or cam 3001 or cam3001).mp. (53)

115 exp Antirheumatic Agents/ (34893)

116 (dmard* or csdmard* or cs-dmard* or sdmard* or s-dmard* or tsdmard* or ts-dmard* or bdmard* or b-dmard*).mp. (3147)

117 (disease modifying adj3 (antirheum* or anti rheum*) adj3 (drug? or agent?)).mp. (2123)

118 ((antirheum* or anti rheum*) adj5 (drug? or agent?)).mp. (4248)

119 Methotrexate/ (4137)

120 (mxt or methotrexat* or metotrexat* or methoblastin or methylaminopterin* or amet?opterin* or abitrexate or antifolan or brimexate or a-met?opterin* or a-methpterin* or ai3-25299 or ai325299 or alpha-methopterin or biotrexate or emtexate or emtrexate* or emthexat* or enthexate* or farmitrexat* or farmotrex or fauldexato or folex or folex pfs or hdmtx or ifamet or imeth or maxtrex or metotressat* or methotrate* or methohexate* or mexate* or metrotex or metatrexato* or metex or metrex or metatrexan or maxtrex or meticil or metoject or metotrexin or novatrex* or neotrexate* or nordimet or lumexon or ledertrexate* or lanterel or rasuvo or r?eumatrex* or reditrex or rasuvo or texate or texate-t or tremetex or trexeron or trixilem or texorate or trexall or xaken or xatmep or otrexup or mpi-2505 or mpi2505 or mpi 5004 or mpi5004 or amethopterin* or 133073-73-1 or 15475-56-6 or 3ig1e710zn* or yl5fz2y5u1* or cl 14377 or cl14377 or ccris 1109 or ccris1109 or emt 25299 or emt25299 or nsc-740 or nsc740 or r-9985 or r9885 or x-133 or wr-19039 or wr19039).mp. (12111)

121 Leflunomide/ (153)

122 (leflunomid* or hwa 486 or hwa486 or su101 or su 101 or arava or arabloc or hsdb 7289 or hsdb7289 or g162gk9u4w* or 75706-12-6 or rs 34821 or rs34821).mp. (688)

123 lef.ti,ot,ab. (173)

124 Sulfasalazine/ (469)

125 (sulfasalazin* or sasp or ssz or salicylazosulfapyridin* or salicyl azo sulfapyridin* or sulphasalazin* or salazosulfapyridin* or salazosulfapiridin* or 'pyralin en' or azulfadin* or azulfidin* or asulfidin* or azulfide* or azulfin or colo-pleon or colopleon or disalazin or gastropyrin or pleon or "pleon ra" or "pyralin en" or rorasul or rosulfant or ulcol or ucine or salazopyrin* or ratio-sulfosalazin* or sulfasalazin* or 3xc8guz6cb* or accucol or asulfidin* or azopyrin* or azosulfidin* or benzosulfa or "brn 0356241" or brn0356241 or ccris 4713 or ccris4713 or einecs 209-974-3 or hsdb 3395 or hsdb3395 or nsc 203730 or nsc203730 or nsc 667219 or nsc667219 or reupirin or rorasul or salazo-sulfapyridin* or salazosulfpyridin* or salazopiridazin* or salazopyridin* or salazopirin* or salazodin or salisulf or salipyr or saridine or si-88 or si88 or sulculon or sulfasalazin* or sulphasalazin* or w-t sasp oral or 599-79-1 or "s.a.s. 500" or "s.a.s.-500" or sas-500 or zopyrin*).mp. (1596)

126 Hydroxychloroquine/ (505)

127 (hydroxychloroquin* or hcq or 747-3-4 or 8q2869cnvh* or 118-42-3 or 4qwg6n8qkh* or hydroxychlorochin* or hydrochloroquin* or hydrocloroquin* or oxychlorochin or oxychloroquin* or plaquenil or plaquinol or "brn 0253894" or brn0253894 or einecs 204-249-8 or einecs204-249-8 or hidroxicloroquin* or idrossiclorochin* or oxichlorochin* or oxichloroquin* or oxiklorin or win1258 or win 1258 or z0188 or einecs 212-019-3 or einecs212-019-3 or chloroquinol or dimard or ercoquin or evoquin or erquin or geniquin or quensyl or sn 8137 or sn8137 or toremonil or yuma).mp. (1666)

128 Gold Sodium Thiomalate/ (91)

129 (gold sodium thiomalate* or aurothiomalate* or aurolate* or gold thiomalate* or gold disodium thiomalate* or gold thiomalic acid* or miocrin or miocrisin or monogold disodium thiomalate* or myochrysin* or myocrisin* or myocrysin* or sodium aurothiomalate* or sodium gold thiomalate* or sodium thiomalate* gold or sodium thiomalatoaurate* or tauredon or taureodon or tauredone or thiomalate gold or thiomalatoaurate sodium or 1244-57-4 or 39377-38-3 or 4846-27-9 or 74916-57-7 or e4768zy6gm* or aurothiomala-natrium or aurot?iomalato sodico or monogold* sodium salt or dinatrium 2-aurothio-succinat or einecs 235-479-7 or einecs235-479-7 or hsdb 7173 or hsdb7173 or kidon or natrii aurothiomalas* or shiosol or gold mercatpsuccinate*).mp. (156)

130 Aurothioglucose/ (36)

131 (thioglucosoaurate* or gold thioglucose* or gold thio glucose* or thioglucose gold or aureotan or solganal or solganol or gold-50 or gold50 or solganal b or b oleosum solganal or auromyose* or aurothioglucose* or auro thioglucose* or aurotan or aurumine or aurumin or authron or brenol or ccris 59 or ccris59 or einecs 235-365-7 or einecs235-365-7 or glysanol b or goldthioglucose* or goldtioglucose* or hsdb 7174 or hsdb7174 or oronol or romosol or skf 10056 or skf10056 or 2p2v9q0e78* or 12192-57-3).mp. (49)

132 ((gold* or auro*) adj10 (inject* or intramuscular* or im or intra muscular*)).ti,ot,ab. (210)

133 Organogold Compounds/ (15)

134 (aurothiopropanol* or allochrysine or allocrisine or aurotiopoe or aurotioprol or 27279-43-2 or g7097j63e9* or auromercapto propanol sulfonate sodium or auromercaptopropanolsulfonate sodium or aurothioisopropanolsulfonate sodium or sodium aurothiopropanolsulfonate or gold sodium thiopropanol sulfonate or sodium auromercaptopropanol sulfonate).mp. (3)

135 Chloroquine/ (709)

136 (chloroquin* or cq or 54-04-7 or 886u3h6uff* or aralen or aralene or arechin or arechine or arequin or chingamin or chlorochin* or khingamin or nivaquine or oe48649kh6n* or amokin or amokine or anaclor or aralan or arequine or artriquine or avloclor or avoclor or arthrochin or artrichin or bemaco or bemaphata or bemaphate or bemasulph or benaquin or bipiquin or cadiquin or "brn 0482809" or brn0482809 or capquin or ccris 3439 or ccris3439 or chemochin or chemochine or chingamine or chingaminum or chloraquin* or chlorquin or chlorquine or choloquine or choroquine sulfate or choroquine sulphate or cindacin or clo-kit junior or clorichina or clorichine or cloriquine or clorochina or delagil or delagyl or diclokin or chlorofoz or cindachin or clorochina or cloroquina or cocatrit or dichinalex or diquinalex or diroquine or emquin or genocin or gontochin or gontochine or gontoquine or einecs 200-191-2 or einecs200-191-2 or elestol or gontochin or heliopar or hsdb 3029 or hsdb3029 or imagon or iroquine or klorokin or klorokine or klorokinfosfat or lagaquin or lapaquin or malaquin or malaren or malarex or malarivon or malaviron or maliaquine or maquine or mesylith or mexaquin or mirquin or neochin or nivaquin or nivachine or nivaquine b or nivaquine dp or nivaquine forte or "p roquine" or nsc 187208 or nsc187208 or pfizerquine or quensyl or quinachlor or quinercyl or quingamine or quinilon or quinoline or quinoscan or repal or resochen or resochene or resochin or resochin junior or resochina or resochine or resochinon or resoquina or resoquine or reumachlor or ronaquine or roquine or rp 3377 or rp3377 or sanoquin or sanoquine or silbesan or siragan or sirajan or sn 7618 or sn7618 or solprina or solprine or sopaquin or st 21 or tresochin or tresoquine or trochin or trochine or troquine or weimerquin or w 7618 or w7618 or win 244 or win244).mp. (2086)

137 exp Adrenal Cortex Hormones/ (27587)

138 exp Prednisolone/ (4835)

139 Prednisone/ (3978)

140 Cortisone/ (148)

141 Hydrocortisone/ (5944)

142 exp Steroids/ (53553)

143 adrenal cortex hormones.mp. (2224)

144 prednisolone*.mp. (7414)

145 methylprednisolone*.mp. (5485)

146 (prednisolone* or predonine or di-adreson-f or diadresonf or 9phq9y1olm* or prednisolona* or prednisolonum* or nsc-9120 or nsc9120 or nsc 9900 or nsc9900 or delta-dehydrocortisol* or delta-dehydrocortisone* or delta-hydrocortisone or hydroretrocortine or metacortandralone* or brn 1354103 or brn1354103 or bubbli-pred or ccris 980 or ccris980 or co-hydeltra or codelcortone or cordrol or cortalone or cotogesic or cotolone or decaprednil or decortin h or delcortol or dehydrohydrocortisone* or delta-cortef or deltacortef or delta-ef-cortelan or delta-stab or deltacortenol or deltacortril or deltahydrocortisone* or deltisilone or derpo pd or dexa-dortidelt hostacortin h or di-adreson f or dicortol or donisolone or dydeltrone or eazolin d or ec 200-021-7 or ec200-021-7 or einecs 200-021-7 or einecs200-021-7 or erbacort or erbasona or estilsona or fernisolone or fernisolone p or hostacortin h or hsdb 3385 or hsdb3385 or hydeltra or hydeltrone or hydrodeltalone or hydrodeltisone or hydroretrocortin or hydroretrocortine* or k 1557 or k1557 or lentosone or meti-drm or meticortelone or orapred or orapred odt or paracortol or paracotol or pediapred or precortancyl or precortilon or precortisyl or predne-dome or prednelan or prednicen or predniliderm or predniretard or prednis or predonin or predonine* or prelon or prelone or prenolone or rolisone or scherisolon? or solone or sterolone or ulacort or ultracorten h or ultracortene h adelcort or antisolon or antisolone or aprednisolon? or benisolon? or caberdelta or co hydeltra or codelcortone or ompresolon).mp. (7432)

147 (cortadeltona or cortelinter or cortisolone or dacortin or dacortin h or dacrotin or decaprednil or decortil or delta cortril or delta hycortol or deltacortenolo or deltacortil or deltacortoil or deltaderm or deltadglycortril or deltahycortol or deltahydrocortison? or deltaophticor or deltasolone or deltastab or deltidrosol or deltisilone or deltisolon? or deltolasson? or deltosona or deltosone or depo-predate or dermosolon or dhasolone or di adreson? f or diadreson? f or dicortol or domucortone or encortelon? or encortolon or equisolon or glistelone or hefasolon or hydrelta or hydrocortancyl or hydrocortidelt or hydradeltalone or hydradeltisone or inflanefran or insolone or keteocort h or key-pred or key-pred sp or lenisolone or leocortol or liquipred or mediasolone or meprisolon? or metacortalon? or metacortelone or meti derm or metiderm or meticortelone or morlone or mydrapred or nisolon? or opredsone or panafcortelone or panafcortolone or panafort or paracortol pr phlogex or pre cortisyl or preconin or precortalon or precortancyl or precortisyl or pred ject 50 or predacort 50 or predaject 50 or predalone 50 or predartrina or predartrine or predate 50 or predeltilone or predisole or predisyr or predne dome or prednecort or prednedome or prednelan or predni coelin or predni h tablinen or predni helvacort or prednicoelin or prednicort or prednicortelone or prednifor drops or predniment or prednorsolon? or predorgasolon? or prenilone or preventan or prezolon or rubycort or serilone or solonda or solupren? or spiricort or spolotane or supercorti#ol or taracortelone or walesolone or wysolone).mp. (23)

148 (methylprednisolone * or methylprednisolona* or methylprednisolonum* or metipred or urbason or medrol or x4w7zr7023* or 83-43-2 or besonia or brn 23403300 or brn2340300 or a-methapred or artisone-wyeth or besonia or depo-medrol or dopomedrol ec 201-4764 pr ec2014764 or einecs 201-476-4 or einecs201-476-4 or esametone or firmacort or hsdb3127 or hsdb3127 or lemod or medesone or medixon or medlone 21 or medrate or medrol or medrone or mesopren or metastab or methyleneprednisolone* or metilbetasone or metrisone or metrocort or metysolon or moderin or nirypan or noretona or nsc 19987 or nsc19987 or predni n tablinen or prednol-l or promacortine or reactenol or sieropresol or solomet or summicort or suprametil or u 7532 or u7532 or "u 67 590a" or urbason or urbasone or wyacort or adlone 40 or adlone 80 or beta methylprednisolone or medalone 80 or depmedalone or deproject 80 or depopred

or esametone or firmacort or med-jec-40 or medixon or mednin or medralone or mepredprednisolone or meprelon or mesopren or methacort 40 or methacort 80 or methyxlcotol or methylcotolone or methylpred dp or methylsterolone or metidrol or metycortin or metypred or metypresol or neomedrone or solu decortin or urbason).mp. (1215)

149 (prednison or prednisone or prednisona or prednisonum or dehydrocortisone or delta cortisone or rectodelt or sterapred or ultracorten or winpred or apo prednisone or cortan or cartancyl or panafcort or cutason or decortin or dacortin or decortisyl or deltasone or encortone or encorton or enkorton or enkortolon or kortancyl or liquid pred or meticorten or orasone or panasol or predni tablinen or prednidib or predniment or pronisone or vb0r961hzt* or 53-03-2 or adasone or al3-52939 or ancortone or bicortone or cartancyl or ccris 2646 or ccrs2646 or colisone or cortan or cortidelt or cotone or dacorten or dacortin or decortancyl or dekortin or dellacort or delta cortelan or delta cortisone or delta dome or deltacortene or deltacortisone or deltacortone or deltasone or deltison or deltisona or deltra or di adreson or diadreson or econosone or einecs 200-160-3 or einecs200-160-3 or fernisone or fiasone or hostacortin or hsdb 3168 or hsdb3168 or incocortyl or juvason or lisacort or lodotra or lodtra).mp. (10119)

150 (me-korti or metacortandracin or metacorten or nci c04897 or ncic04897 or nisona or nizon or novoprednisone or nsc 10023 or nsc10023 or nurison or orasone or panafcort or paracort or prarmenison or pehacort or predeltin or prednicen-m or prednicorm or prednicort or prednicot or prednilonga or prednitone or prednizon or prednovister or presone or pronison or rayos or rectodelt or retrocortine or servisone or sterapred or supercortio or u 6020 or u6020 or ultracorten or ultracortene or winpred or wojtab or zenadrid).mp. (19)

151 cortisone?.mp. (569)

152 corticosteroid*.mp. (23083)

153 corticoid*.mp. (556)

154 glucocorticoid*.mp. (8890)

155 corticosterone*.mp. (153)

156 hydrocortisone*.mp. (9702)

157 dexamethasone*.mp. (12333)

158 beclomethasone*.mp. (2295)

159 (triamcinolone* or 1zk20vi6ty* or 124-94-7 or aristocort or volon or fluoxyprednisolon* or tiamcinolonum or triamcinolonum* or triamcinolona* or brn 2341955 or brn2341955 or cl 19823 or cl19823 or einecs 204-718-7 or einecs204-718-7 or hsdb 3194 or hsdb3194 or nsc 13397 or nsc13397).mp. (3241)

160 (steroid? adj10 (therap* or treat* or drug? or inject* or intramuscular* or intra muscular* or im or intravenous* or intra venous* or iv or intraarticular* or intra articular* or ia or administrat* or oral or orally or parenteral*)).mp. (14182)

161 Janus Kinase Inhibitors/ (50)

162 ((jak or jak1 or jak2 or jak3 or janus kinase) adj10 inhibitor*).mp. (1538)

163 (janus adj4 kinase adj10 inhibitor?).mp. (927)

164 exp Janus Kinases/ai [Antagonists & Inhibitors] (95)

165 (baricitinib* or 1187594-09-7 or isp4442i3y* or "incb 028050" or incb028050 or ly-3009104 or ly3009104).mp. (409)

166 (tofacitinib* or 477600-75-2 or 87la6fu830* or hsdb 8311 or hsdb8311 or cp-690 550 or cp 690550 or cp690550).mp. (825)

167 (filgotinib* or 1206161-97-8 or 3xvl385q0m* or "glpg 0634" or glpg0634).mp. (173)

168 (upadacitinib* or 1310726-60-3 or 4RA0KN46E0* or abt 494 or abt494).mp. (267)

169 (ruxolitinib* or rux or 941678-49-5 or 82s8x8xx8h* or hsdb 8259 or hsdb8259 or inc 424 or inc424 or incb424 or "incb 018424" or incb018424 or incb 18424 or incb18424 or r-ruxolitinib* or jakafi or jakavi).mp. (486)

170 (itacitinib or "incb 039110" or incb039110 or incb 39110 or incb39110 or 1334298-90-6 or 19j3781lpm*).mp. (34)

171 (ag490 or ag 490 or tyrphostin b42 or tyrphostin ag 490 or tyrphostin ag490 or 133550-30-8).mp. (1)

172 (fedratinib* or tg101348 or tg 101348 or sar302503 or sar 302503 or 936091-26-8 or 6l1xp550i6* or inrebic).mp. (29)

173 (ritlecitinib* or pf-06651600 or pf06651600 or pf 6651600 or pf6651600 or 2140301-97-7 or 132lf5wgh4* or 2192215-81-7 or eag4t1459k* or ritlecitinib malonate or ritlecitinib toslate or ritlecitinib tosylate or ritlecitinib propanedioate).mp. (24)

174 (peficitinib* or peficitinib hydrobromide).mp. (26)

175 or/90-174 (150408)

176 78 and 175 (6585)

177 89 or 176 (6689)

178 ae.fs. (128872)

179 (adverse adj3 (event* or effect* or reaction* or incident* or outcome*)).mp. (205801)

180 ((serious* or severe* or severit*) adj3 (adverse or saes or aes or adrs or sae or "ae" or adr)).mp. (34986)

181 (saes or aes or adrs or sae or "ae" or adr).ti,ot,ab. and adverse.mp. (21053)

182 (adverseevent* or adversedrugreaction* or adversereaction* or adverseincident* or adverseoutcome*).mp. (27)

183 side effect*.mp. (150434)

184 exp "Drug-Related Side Effects and Adverse Reactions"/ (3172)

185 Adverse Drug Reaction Reporting Systems/ (89)

186 exp Product Surveillance, Postmarketing/ (207)

187 Substance Withdrawal Syndrome/ (2005)

188 withdraw*.mp. (44162)

189 safety.mp. (251564)

190 exp Drug Hypersensitivity/ (983)

191 (hypersensitiv* or hyper sensitiv*).mp. (10145)

192 Anaphylaxis/ (183)

193 anaphyla*.mp. (2258)

194 (toxic* adj6 (drug* or medication*)).mp. (8807)

195 intoxication*.mp. (3388)

196 Abnormalities, Drug-Induced/ (45)

197 Teratogens/ (9)

198 teratogen*.mp. (374)

199 ((drug or chemical*) adj1 induced).mp. (7968)

200 exp substance related disorders/ (15176)

201 (treatment adj3 emergent).mp. (8208)

202 exp Mortality/ (13372)

203 exp Death, Sudden/ (859)

204 exp Death/ (2028)

205 (fatal or fatalit*).mp. (10324)

206 ((mortality or death?) adj4 (rate? or ratio or caus* or risk or sudden or number* or amount* or count*)).mp. (60394)

207 mortality.mp. (83073)

208 exp *Infection/ (14338)

209 infection rate?.mp. (4145)

210 ((serious* or severe* or severit*) adj8 (infect* or bacter?emi* or septic?emi* or sepsis or fung?emi* or fungal infect* or fungal disease* or mycoses or mycosis or mycotic)).mp. (11905)

211 exp Opportunistic Infections/ (1005)

212 ((opportunistic* or opportune) adj6 (infect* or bacter?emi* or septic?emi* or bacterial infect* or bacterial diseas* or viral infect* or virus diseas* or virus infect* or microbial infect* or mycos#s or fung* infect* or mycotic diseas* or mycos#s infect* or fung?emi*)).mp. (2625)

213 ((overall or rate or rates or risk?) adj8 (infect* or bacter?emi* or septic?emi* or sepsis or bacterial infect* or bacterial diseas* or bacterious infect* or viral infect* or virus diseas* or virus infect* or microbial infect* or mycos#s or fung* infect* or mycotic diseas* or mycos#s infect* or fung?emi*)).mp. (27132)

214 infect*.mp. (137368)

215 exp Tuberculosis/ (2387)

216 (tuberculosis or tuberculous).mp. (7367)

217 exp Cytomegalovirus Infections/ (777)

218 ((cytomegalovirus or cytomegaly virus or cytomegaloviral or cmv) adj (infection? or disease?)).mp. (2019)

219 (cytomegalic inclusion adj3 disease?).mp. (4)

220 cytomegaloinfection?.mp. (0)

221 congenital cytomegalic inclusion.mp. (0)

222 (cmv and (virus or viral or infect*)).mp. (1614)

223 exp Mycobacterium Infections, Nontuberculous/ (153)

224 exp Nontuberculous Mycobacteria/ (62)

225 ((atypical or nontuberculous* or non-tuberculous*) adj3 (mycobacterios* or mycobacter*)).mp. (205)

226 (mycobacter* adj3 intracellulare adj3 infection*).mp. (125)

227 exp Mycoses/ (2785)

228 (mycosis or mycoses or mycotic or fung*).mp. (5837)

229 (aspergillos#s or (aspergill* adj3 infect*)).mp. (664)

230 (candidias#s* or candidemi* or (candid* adj3 infect*)).mp. (2778)

231 pneumocyst*.mp. (723)

232 exp Hepatitis B/ (2724)

233 Hepatitis B virus/ (776)

234 hepatitis b.mp. (9349)

235 (hepatitis adj1 (type b or injection or serum)).mp. (523)

236 (hepatitis adj3 "b").ti,ot,ab. (8799)

237 hbv.ti,ot,ab. (4611)

238 exp Infection/ (24558)

239 (opportunistic or serious* or severe* or severit*).mp. (235906)

240 238 and 239 (5313)

241 exp Herpes Zoster/ (563)

242 ((herpes or varicella*) adj1 zoster*).mp. (2376)

243 (zoster or shingles or varicellovirus infect*).mp. (2443)

244 exp Herpes Simplex/ (976)

245 (herpes adj3 (simplex or vulgaris or infection*)).mp. (2126)

246 herpes.mp. (4620)

247 hsv.ti,ot,ab. and (infect* or herpes).mp. (781)

248 241 or 242 or 243 or 244 or 245 or 246 or 247 (4962)

249 Virus Activation/ (85)

250 (reactivat* or re-activat*).mp. (1660)

251 (activat* adj3 (virus or viral* or vir?emi*)).mp. (259)

252 249 or 250 or 251 (1846)

253 248 and 252 (245)

254 C-Reactive Protein/ (4676)

255 9007-41-4.mp. (1)

256 ((c-reactive or creactive or c reaction or creaction) adj2 protein).mp. (18672)

257 crp.mp. (15495)

258 Blood Sedimentation/ (507)

259 ((erythrocyte or blood) adj3 sedimentation*).mp. (2798)

260 esr.ti,ot,ab. and sedimentation.mp. (1248)

261 (DAS28-ESR or DAS-28-ESR or DAS28ESR).mp. (605)

262 ((DAS28 or "DAS 28" or disease activity score or das or disease activity) and ESR).mp. (1537)

263 esr.ti,ot,ab. (3034)

264 Procalcitonin/ (22)

265 Calcitonin/ (645)

266 (procalcitonin or pro-calcitonin or pct).mp. (1707)

267 (calcitonin adj3 precursor?).mp. (4)

268 exp Leukocyte Count/ (5342)

269 ((leukocyte* or white blood cell or white cell or white blood or wbc) adj3 count*).mp. (8905)

270 wbc.ti,ot,ab. (3986)

271 exp Neoplasms/ (79793)

272 exp Hematologic Neoplasms/ (481)

273 exp histiocytic disorders, malignant/ or exp leukemia/ or exp lymphoma/ (7510)

274 exp Multiple Myeloma/ (1618)

275 (lymphom* or leuk?em* or myeloma*).mp. (29835)

276 ((h?ematolog* or blood disease) adj6 (malignan* or neoplas*)).mp. (3004)

277 (cancer or cancers).mp. (176613)

278 neoplas*.mp. (85051)

279 (tumor? or tumour?).mp. (81133)

280 (carcinom* or karzinom*).mp. (44961)

281 hodgkin*.mp. (5949)

282 (non-hodgkin* or nonhodgkin*).mp. (3882)

283 (mesotheliom* or mesoteliom*).mp. (842)

284 malignan*.mp. (29048)

285 adenocarcinom*.mp. (11782)

286 metasta*.mp. (46561)

287 sarcom*.mp. (2964)

288 adenosarcom*.mp. (8)

289 osteosarcom*.mp. (616)

290 reticulosarcom*.mp. (5)

291 lymphosarcom*.mp. (27)

292 (blastom* or neuroblastom*).mp. (823)

293 (gliom* or glioblastom*).mp. (3695)

294 melanom*.mp. (6084)

295 ((non melanom* or nonmelanom*) adj3 skin adj3 (cancer or cancers or neoplas* or tumo?r* or carcino*)).mp. (634)

296 (solid adj6 (cancer or cancers or neoplas* or tumo?r* or carcino* or malignan*)).mp. (8239)

297 exp Diverticulitis/ (91)

298 diverticulitis*.mp. (568)

299 Intestinal Perforation/ (140)

300 ((digestive system or gastrointestinal* or gastro intestinal* or intestinal*) adj3 perforation*).mp. (805)

301 exp Liver/ (3141)

302 Liver Function Tests/ (1160)

303 ((liver or hepat*) adj1 function?).mp. (10870)

304 (lft or lfts).ti,ot,ab. (570)

305 (hypertransaminasemi* or hyper transaminasemi*).mp. (1157)

306 uln.ti,ot,ab. (2730)

307 exp Drug-Induced Liver Injury/ (10)

308 (dili or drug induc* liver injur*).mp. (484)

309 Alanine Transaminase/ (1605)

310 exp Aspartate Aminotransferases/ (1021)

311 (transaminas* or alanin* aminotransaminas* or alt or aspartat* aminotransaminas* or ast or serum glutam* pyruv* transaminas* or sgpt or serum glutam* oxalacet* transaminas* or sgot or liver enzyme* or hepat* enzyme*).mp. (17316)

312 transaminitis*.mp. (164)

313 (hepat*tox* or ((liver or hepat*) adj3 toxic*)).mp. (3522)

314 exp Triglycerides/ (6344)

315 exp Lipoproteins/bl [Blood] (1639)

316 exp "lipoprotein(a)"/ or exp lipoproteins, hdl/ or exp lipoproteins, ldl/ (7556)

317 exp Cholesterol/ (10207)

318 ((lipid? or cholesterol* or cholesterin* or triglycerid* or lipoprotein*) adj3 (level? or blood or plasma or serum)).mp. (37529)

319 (ldl or low density lipoprotein*).mp. (27032)

320 (hdl or high density lipoprotein*).mp. (22341)

321 (hdl2 or "hdl(2)" or "hdl 2").mp. (425)

322 (hdl3 or "hdl(3)" or "hdl 3").mp. (342)

323 (lipoprotein a or lipoproteina).mp. (22259)

324 alpha lipoprotein.mp. (13)

325 (ldl1 or "ldl(1)" or "ldl 1").mp. (48)

326 (ldl2 or "ldl(2)" or "ldl 2").mp. (65)

327 exp Lipids/bl [Blood] (3506)

328 exp Hyperlipidemias/ (6430)

329 (hyperlipid?emi* or lipid?emi*).mp. (6732)

330 exp Diabetes Mellitus/ (31627)

331 exp Obesity/ (14018)

332 (metabolic adj3 (effect? or alterat* or chang* or risk? or transition* or transformat* or parameter? or marker? or biomarker?)).mp. (14742)

333 (diabetes* or diabetic*).mp. (99239)

334 Glycated Hemoglobin A/ (5742)

335 (hba1c or hbaic or "hb a1c" or "hb a 1c" or "hb aic" or "hb a ic" or "hba 1c" or "hba ic").ti,ot,ab. (19312)

336 ("h?emoglobin a1c" or "h?emoglobin a 1c" or h?emoglobin aic or "h?emoglobin a ic").mp. (8888)

337 body mass index/ (10319)

338 (body mass index or bmi).mp. (64171)

339 Venous Thromboembolism/ (640)

340 ((venous* or venos* or vein*) adj1 (thrombos#s* or thrombotic*or thromboemboli* or thrombo emboli* or thrombus or thrombi or microthromb*)).mp. (8802)

341 (dvt or dvts or vte or vtes).ti,ot,ab. (4121)

342 exp Pulmonary Embolism/ (968)

343 ((lung* or pulmonary) adj3 (embol* or thromboemboli* or thromb* emboli* or microemboli* or clot* or thrombus or thrombi or microthromb* or infarct*)).mp. (6183)

344 (pe or pes).ti,ot,ab. and (lung* or pulmo* or pneumo* or embol* or thromboembol* or thrombot* or thrombus or thrombi or microembol).mp. (1709)

345 exp Anemia/ (5209)

346 an?emi*.mp. (21980)

347 exp Thrombocytopenia/ (1259)

348 (thrombocytopeni* or thrombopeni*).mp. (11302)

349 exp Neutropenia/ (1712)

350 neutropeni*.mp. (14658)

351 exp Lymphopenia/ (87)

352 (lymphocytopeni* or lymphopeni*).mp. (1515)

353 Macrophage Activation Syndrome/ (7)

354 mas.ti,ot,ab. (2036)

355 (macrophage activat* adj3 syndrome?).mp. (55)

356 353 or 354 or 355 (2071)

357 (Rheumatoid Arthritis adj3 Systemic adj3 Juvenile).mp. (18)

358 Arthritis, Juvenile/ (299)

359 ((arthrit* or artrit*) adj6 juvenile).mp. (1001)

360 JIA.ti,ot,ab. (627)

361 358 or 359 or 360 (1123)

362 (systemic or polyart* or poly art* or oligoart* or oligo art*).mp. (52595)

363 361 and 362 (515)

364 ((systemic adj6 jia) or sjia or s-jia).ti,ot,ab. (167)

365 357 or 363 or 364 (526)

366 356 and 365 (32)

367 exp Heart Failure/ (9513)

368 (congestive adj2 (heart or cardiac) adj2 failure*).mp. (6600)

369 (heart failure* or cardiac failure* or myocard* failure*).mp. (31749)

370 ((heart or cardiac or cordis or cardio* or myocard*) adj1 decompensatio*).mp. (144)

371 (chf or ccf or hf).ti,ot,ab. and (heart or cardiac or cardio* or myocard*).mp. (10356)

372 exp Myocardial Ischemia/ (28078)

373 ((heart or cardio* or cardiac or myocardial) adj1 infarct*).mp. (36483)

374 exp Myocardial Infarction/ (10847)

375 cardiovascular* stroke?.mp. (29)

376 (MACE or MACEs).ti,ot,ab. (3217)

377 major adverse card* event*.mp. (4170)

378 acute coronary syndrome*.mp. (7735)

379 (heart attack or heart attacks).mp. (1232)

380 (mi or mis).ti,ot,ab. and (myocard* or cardio* or coronary or heart or cardiac or mace or maces).mp. (6685)

381 (angina or anginas or stenocardia?).mp. (14212)

382 exp Stroke/ (9836)

383 ((cerebrovascular* or cerebr* vascular*) adj1 (accident? or failure? or injury or injuries or insult? or insufficienc* or apoplex*)).mp. (15128)

384 (stroke or strokes or apoplexi* or apoplexy*).mp. (59567)

385 (cva or cvas).ti,ot,ab. (594)

386 (brain insult* or brain isch?emic attack? or cerebral insult* or brain vascular accident? or isch?emic cerebr* attack? or isch?emic seizure? or cerebr* vascular accident?).mp. (210)

387 exp Myocardial Revascularization/ (9219)

388 (revascularizat* or re-vascularizat* or revascularisat* or re-vascularisat*).mp. (13109)

389 Peripheral Arterial Disease/ (1066)

390 Arterial Occlusive Diseases/ (863)

391 (peripher* adj2 (arter* or vascular*) adj2 disease*).mp. (5834)

392 ((pad or pvd) adj6 (peripher* or arter* or vascular*)).ti,ot,ab. (1342)

393 exp Kidney Function Tests/ (4263)

394 ((kidney or renal) adj1 function*).mp. (20279)

395 Creatinine/bl [Blood] (132)

396 (creatinin* and (serum or plasma or blood)).mp. (22626)

397 (creatinin* adj15 (level? or concentration? or ratio or clearance or value?)).mp. (18351)

398 (creatinin* adj15 (decreas* or increas* or elevat* or lower* or low or high or higher)).ti,ot,ab. (8238)

399 AYI8EX34EU.af. (0)

400 (estimated glomerul* filtrat* rate? or estimated glomerulofiltration rate?).mp. (5087)

401 (egfr or egfrs or e-gfr or e-gfrs or estimat* gfr or estimat* gfrs or estimat*gfr or estimat*gfrs).mp. (9842)

402 Glomerular Filtration Rate/ (2787)

403 exp Renal Insufficiency/ (9129)

404 ((renal or kidney) adj1 (insufficien* or failure ?or injur*)).mp. (5694)

405 ((cardio renal or cardiorenal or renocardiac or reno cardiac) adj1 syndrom*).mp. (132)

406 exp Hemoglobins/ (3695)

407 h?emoglobin*.mp. (39881)

408 exp Osteoporosis/ (4109)

409 (osteoporos* or osteoporotic*).mp. (11834)

410 exp Leukoencephalopathies/ (371)

411 (leukoencephalopath* or leuko encephalopath*).mp. (354)

412 exp Demyelinating Diseases/ (3812)

413 ((demyelinat* or demyeliniz*) adj3 (disease* or disorder* or cns or central nervous system* or encephalopath* or autoimmune disease*)).mp. (640)

414 (demyelinat* or de-myelinat*).mp. (1319)

415 (demyeliniz* or de-myeliniz*).mp. (13)

416 (demyelinis* or de-myelinis*).mp. (9)

417 exp Multiple Sclerosis/ (3525)

418 multiple scleros#s.mp. (10869)

419 exp Optic Neuritis/ (167)

420 (optic* adj3 (neuritis* or neuritides*)).mp. (495)

421 (optic* adj3 (inflammat* adj3 neuropath*)).mp. (6)

422 exp Myelitis, Transverse/ (43)

423 (transverse adj3 (myelitis* or myelitides* or myelopath* syndrome*)).mp. (67)

424 ((necrotiz* or necrotis* or necrotic) adj3 (myelitis* or myelitides*)).mp. (0)

425 ((demyelinat* or de-myelinat*) adj3 (myelitis* or myelitides*)).mp. (2)

426 ((postinfect* or infect* or postvaccin* or vaccin*) adj10 (myelitis or myelitides)).mp. (21)

427 exp Peripheral Nervous System Diseases/ (5421)

428 (peripheral adj2 (nervous system or nerve) adj2 (disease* or disorder* or neuropath* or mononeuropath* or mono neuropath* or polyneuropath* or poly neuropath* or polyradiculaneuropath* or poly radiculoneuropath* or polyradiculo neuropath*)).mp. (1199)

429 exp Guillain-Barre Syndrome/ (48)

430 (guillain barre* or fisher? syndrome*).mp. (398)

431 ((landry? or landry guillain barre?) adj3 (syndrom* or paralys*)).mp. (0)

432 (miller fisher? adj3 (syndrom* or polyneuritis* or poly neuritis* or variant?)).mp. (1)

433 ((polyradiculoneuropath* or poly radiculoneuropath* or polyradiculo neuropath* or polyneuropath* or poly neuropath* or neuronitis* or polyneuritis* or poly neuritis*) adj15 (inflammat* or postinfect* or infect* or postvaccin* or vaccin*)).mp. (380)

434 Polyradiculoneuropathy, Chronic Inflammatory Demyelinating/ (63)

435 ((polyradiculoneuropath* or poly radiculoneuropath* or polyradiculo neuropath* or polyneuropath* or poly neuropath* or polyradiculopath* or poly radiculopath*) adj6 chronic* adj3 inflammat*).mp. (303)

436 cidp.ti,ot,ab. (256)

437 exp Polyradiculoneuropathy/ (207)

438 (polyradiculoneuropath* or poly radiculoneuropath* or polyradiculo neuropath*).mp. (249)

439 ((demyelinat* or inflammat*) adj1 (neuropath* or polyneuropath* or poly neuropath* or polyradiculoneuropath* or poly radiculoneuropath* or polyradiculo neuropath*)).mp. (427)

440 (multifocal motor neuropath* adj6 conduction block*).mp. (8)

441 (motor adj3 neuropath* adj6 conduction block*).mp. (12)

442 exp Motor Neuron Disease/ (712)

443 (motor adj3 neuropath*).mp. (538)

444 exp Mononeuropathies/ (1097)

445 (mononeuropath* or mono neuropath* or mononeuritis* or mono neuritis* or mononeuritides* or mono neuritides*).mp. (76)

446 exp Polyneuropathies/ (499)

447 ((polyneuropath* or poly neuropath*) adj6 axonal adj3 (sensor* motor or sensorimotor* or sensorymotor)).mp. (4)

448 (polyneuropath* or poly neuropath*).mp. (1515)

449 exp Vaccination/ (2544)

450 exp Vaccines/ (12922)

451 vaccinat*.mp. (16843)

452 (active adj2 immuni#at*).mp. (351)

453 vaccine*.mp. (24496)

454 exp Pregnancy/ (21326)

455 exp Pregnancy Complications/ (11338)

456 pregnan*.mp. (68002)

457 tolerabilit*.mp. (68763)

458 safe.ti,ot,ab. (68592)

459 ((injurious* or undesirabl*) adj3 (effect? or reaction* or event? or outcome? or incident?)).ti,ot,ab. (1226)

460 exp Postoperative Complications/ (39488)

461 exp Intraoperative Complications/ (4298)

462 complication*.mp. (147097)

463 co.fs. (54137)

464 risk.ti,ot,ab. (222306)

465 de.fs. (120976)

466 exp risk/ (38514)

467 178 or 179 or 180 or 181 or 182 or 183 or 184 or 185 or 186 or 187 or 188 or 189 or 190 or 191 or 192 or 193 or 194 or 195 or 196 or 197 or 198 or 199 or 200 or 201 or 202 or 203 or 204 or 205 or 206 or 207 or 208 or 209 or 210 or 211 or 212 or 213 or 214 or 215 or 216 or 217 or 218 or 219 or 220 or 221 or 222 or 223 or 224 or 225 or 226 or 227 or 228 or 229 or 230 or 231 or 232 or 233 or 234 or 235 or 236 or 237 or 240 or 253 or 254 or 255 or 256 or 257 or 258 or 259 or 260 or 261 or 262 or 263 or 264 or 265 or 266 or 267 or 268 or 269 or 270 or 271 or 272 or 273 or 274 or 275 or 276 or 277 or 278 or 279 or 280 or

281 or 282 or 283 or 284 or 285 or 286 or 287 or 288 or 289 or 290 or 291 or 292 or 293 or 294 or 295 or 296 or 297 or 298 or 299 or 300 or 301 or 302 or 303 or 304 or 305 or 306 or 307 or 308 or 309 or 310 or 311 or 312 or 313 or 314 or 315 or 316 or 317 or 318 or 319 or 320 or 321 or 322 or 323 or 324 or 325 or 326 or 327 or 328 or 329 or 330 or 331 or 332 or 333 or 334 or 335 or 336 or 337 or 338 or 339 or 340 or 341 or 342 or 343 or 344 or 345 or 346 or 347 or 348 or 349 or 350 or 351 or 352 or 366 or 367 or 368 or 369 or 370 or 371 or 372 or 373 or 374 or 375 or 376 or 377 or 378 or 379 or 380 or 381 or 382 or 383 or 384 or 385 or 386 or 387 or 388 or 389 or 390 or 391 or 392 or 393 or 394 or 395 or 396 or 397 or 398 or 399 or 400 or 401 or 402 or 403 or 404 or 405 or 406 or 407 or 408 or 409 or 410 or 411 or 412 or 413 or 414 or 415 or 416 or 417 or 418 or 419 or 420 or 421 or 422 or 423 or 424 or 425 or 426 or 427 or 428 or 429 or 430 or 431 or 432 or 433 or 434 or 435 or 436 or 437 or 438 or 439 or 440 or 441 or 442 or 443 or 444 or 445 or 446 or 447 or 448 or 449 or 450 or 451 or 452 or 453 or 454 or 455 or 456 or 457 or 458 or 459 or 460 or 461 or 462 or 463 or 464 or 465 or 466 (1196458)

468 177 and 467 (5613)

469 ((eular or (european adj4 rheumat*)) adj8 (meeting* or conference* or congress*) adj8 annual).af. (704)

470 ((acr or (american college adj4 rheumat*)) adj8 (meeting* or conference* or congress*) adj8 annual).af. (806)

471 469 or 470 (1506)

472 468 and 471 (247)

473 limit 472 to yr="2019-2020" (0)

474 limit 468 to yr="2012-2020" (4245)

475 limit 474 to english (2689)

476 exp Registries/ (1029)

477 (register or registry or registries or registered or registration*).mp. (101650)

478 Cohort Studies/ (7646)

479 (cohort* adj6 (study* or studies* or analy*)).mp. (43916)

480 (cohortstudy* or cohortstudies* or cohortanaly*).mp. (6)

481 exp case-control studies/ (13933)

482 (case? adj3 control* adj3 (study or match*)).mp. (8904)

483 case referent.mp. (12)

484 (case? adj2 control?).mp. (19185)

485 Prospective Studies/ (93051)

486 exp Longitudinal studies/ (142382)

487 Retrospective Studies/ (8831)

488 (longitudinal or prospective* or retrospective*).mp. (301383)

489 Follow-Up Studies/ (60349)

490 (follow up or followup).mp. (267009)

491 489 or 490 (267009)

492 (long term or longterm).mp. (103284)

493 (extend* or extension?).mp. (49928)

494 (continuing or continue* or continuat*).mp. (73980)

495 492 or 493 or 494 (204772)

496 491 and 495 (62283)

497 ((long term or longterm) adj3 data).ti,ot,ab. (3554)

498 496 or 497 (63887)

499 476 or 477 or 478 or 479 or 480 or 481 or 482 or 483 or 484 or 485 or 486 or 487 or 488 or 498 (475261)

500 475 and 499 (1032)

501 randomized controlled trial.pt. (507674)

502 exp randomized controlled trial/ (131)

503 exp Randomized Controlled Trials as Topic/ (8017)

504 randomi*.mp. (1025624)

505 randomly.ti,ot,ab. (259754)

506 trial.ti. (336020)

507 trial.ab. and random*.mp. (412280)

508 (rct or rcts).ti,ot,ab. (34111)

509 Random Allocation/ (20645)

510 controlled clinical trial/ (36)

511 Controlled Clinical Trials as Topic/ (71)

512 Single-Blind Method/ (21220)

513 Double-Blind Method/ (139303)

514 ((singl* or doubl* or tripl* or trebl*) adj3 (blind* or mask*)).mp. (390906)

515 (singl*blind* or doubl*blind* or trebl*blind* or tripl*blind*).ti,ot,ab. (2483)

516 (singl*mask* or doubl*mask* or tripl*mask* or trebl*mask*).ti,ot,ab. (72)

517 (controlled adj8 (study or design or trial)).mp. (819745)

518 (parallel adj1 group*).ti,ot,ab. (43843)

519 exp placebo effect/ (1548)

520 Placebos/ (24107)

521 placebo*.mp. (331869)

522 (allocated or allocation or assigned).ti,ot,ab. and random*.mp. (230516)

523 groups.ti,ot,ab. (489767)

524 dt.fs. (200514)

525 ((assign* or match or matched or allocation) adj6 (alternate or group or groups or intervention? or patient? or subject? or participant?)).ti,ot,ab. (164078)

526 ('head to head' adj3 (study or comparison or trial)).ti,ot,ab. and random*.mp. (1119)

527 or/501-526 (1451142)

528 495 and 527 (186976)

529 475 and 528 (863)

530 500 or 529 (1524)

531 review.pt. (3088)

532 conference abstract.pt. (16804)

533 conference review.pt. (173)

534 conference paper.pt. (134)

535 congress.pt. (44)

536 congresses.pt. (5)

537 editorial.pt. (462)

538 letter.pt. (7334)

539 journal conference abstract.pt. (163818)

540 journal conference paper.pt. (6517)

541 "journal conference review".pt. (814)

542 journal editorial.pt. (2344)

543 journal letter.pt. (4901)

544 or/531-543 (206417)

545 (530 not 544) or 473 (653)

1.3.5 Patient adherence and economic aspects

1.3.5.1 Medline

1 Arthritis, Rheumatoid/ (100454)

2 ((rheumat* or reumat* or revmat* or rheumo or reumo) adj3 (arthrit* or artrit* or polyarthrit* or polyartrit*)).ti,ot,ab,kw,kf. (116756)

3 (r?eumarthrit* or r?eumartrit* or r?eum arthrit* or r?eum artrit* or r?evmarthrit* or r?evmartrit* or r?evm arthrit* or r?evm artrit* or r?eum polyarthrit* or r?eum polyartrit* or r?eum poly arthrit* or r?eum poly artrit* or r?eumpolyarthrit* or r?eumpolyartrit*).ti,ot,ab,kf,kw. (5)

4 (chronic* adj2 (polyarthrit* or poly arthrit* or polyartrit* or poly artrit*)).ti,ot,ab,kf,kw. (2455)

5 (inflammator* adj2 (arthrit* or artrit*)).ti,ot,ab,kf,kw. (6862)

6 ((r?eum* or r?evm*) adj2 (chronic* adj2 articular*)).ti,ot,ab,kf,kw. (42)

7 ((rheumat* or reumat* or revmat* or rheumo or reumo) adj2 (condition* or diseas*)).ti,ot,ab,kf,kw. (33919)

8 Arthritis, Juvenile/ (10545)

9 ((arthrit* or artrit*) adj6 juvenile*).ti,ab,kf,kw. (10753)

10 JIA.ti,ot,ab,kf,kw. (4157)

11 8 or 9 or 10 (14218)

12 (systemic or polyart* or poly art* or oligoart* or oligo art*).ti,ab,kf,kw,hw. (537798)

13 11 and 12 (4851)

14 ((polyarthr* or polyartrit* or poly arthrit* or poly artrit* or oligoarthrit* or oligoartrit* or oligo arthrit* or oligo artrit*) adj6 juvenile*).ti,ab,kf,kw. (233)

15 (((stills or still's or still) adj disease) and juvenile*).ti,ab,kf,kw. (313)

16 ((stills or still's or still) adj disease adj6 child*).ti,ab,kf,kw. (59)

17 ((systemic adj6 JIA) or sJIA or s-JIA).ti,ab,kf. (878)

18 (((polyart* or oligoart* or poly art* or oligo art*) and JIA) or pJIA).ti,ab,kf. (1066)

19 Giant Cell Arteritis/ (6562)

20 (giant cell adj6 (arterit* or aortit* or horton*)).ti,ab,kf. (5255)

21 ((temporal or cranial) adj2 arterit*).ti,ab,kf. (2432)

22 ((horton or horton's or hortons) adj3 disease?).ti,ab,kf. (490)

23 (large vessel adj3 (vasculit* or arterit*)).ti,ab,kf. (1098)

24 GCA.ti,ab,kf. and (arterit* or vasculit* or rheum*).mp,jw. (1894)

25 Takayasu Arteritis/ (4037)

26 (takayasu* adj3 (arterit* or syndrom* or disease*)).ti,ab,kf. (4488)

27 (young female adj6 arterit*).ti,ab,kf. (21)

28 (brachiocephal* adj6 arterit*).ti,ab,kf. (21)

29 (or/1-7) or (or/13-28) (179405)

30 (tocilizumab* or actemra* or atlizumab* or lusinex* or r1569 or r 1569 or roactemra* or mra or 375823 41 9 or i031v2h011* or rhpm-1 or rhpm1 or rg-1569 or rg1569 or msb-11456 or msb11456 or ro-4877533 or ro4877533).mp,rn. (12832)

31 (sarilumab* or kevzara* or regn 88 or regn88 or sar 153191 or sar153191 or 1189541 98 7 or nu90v55f8i*).mp,rn. (183)

32 (levilimab* or BCD-089 or bcd089 or 2035008 70 7 or P7UV3L2H80*).mp,rn. (0)

33 (olokizumab* or cdp 6038 or cdp6038 or 1007223 17 7 or pai71r1d2w*).mp,rn. (18)

34 (clazakizumab* or ald 518 or ald518 or bms 945429 or bms945429 or 1236278 28 6 or 4s38z8ra9o*).mp,rn. (31)

35 (sirukumab* or cnto 136 or cnto136 or pilvensia or 1194585 53 9 or 640443FU93*).mp,rn. (55)

36 (siltuximab* or sylvant or cnto 328 or cnto-328 or cnto328 or cllb8 or ccib8 monoclonal antibody or sylvant or t4h8fma7im* or 541502 14 1).mp,rn. (185)

37 (ziltivekimab* or zilti or "cor-001" or "2226654 05 1").mp,rn. (3)

38 (elsilimomab* or "b-e8" or 468715 71 1).mp,rn. (12)

39 30 or 31 or 32 or 33 or 34 or 35 or 36 or 37 or 38 (13122)

40 29 and 39 (2356)

41 Interleukin 1 Receptor Antagonist Protein/ (5254)

42 (anakinra* or kineret or ((interleukin 1 receptor or il1 receptor or il 1 receptor) adj1 antagonist protein*) or (recombinant interleukin 1 receptor adj1 (antagonist* or blocker* or blocking agent*)) or antril or il 1ra or il1ra or il 1 ra or ((urin* or febrile) adj2 (interleukin 1 inhibitor* or il1 inhibitor* or il 1 inhibitor*)) or "143090 92 0" or 9013duq28k*).mp,rn. (9744)

43 Infliximab/ (10447)

44 (infliximab* or ifx or avakine or flixabi or inflectra or inflecta or ixifi or remicade or remsima or revellex or renflexis or zessly or mab ca2 or monoclonal antibody ca2 or antibody ca2 monoclonal or ca2 monoclonal antibody or ct p13 or ctp13 or ct-p-13 gp-1111 or gp1111 or ta-650 or ta650 or abp 710 or abp710 or bow-015 or bow015 or 170277 31 3 or b72hh48flu* or sb2 or "pf 06438179" or pf 6438179 or pf06438179 or pf6438179 or hsdb 7850 or hsdb7850).mp,rn. (16036)

45 Etanercept/ (5925)

46 (etanercept* or benepali or embrel or enbrel or enia 11 or enia11 or erelzi or lifmior or opinercept or recombinant tumo?r necrosis factor receptor fc fusion protein or "tnr 001" or tnr001 or tnfr-fc or tnrfr:fc or rhu tnfr:fc or rhu-tnfr:fc or tnfr-immunoadhesin or tnt receptor fusion protein or tum?or necrosis factor receptor fc fusion protein or tunex or "185243 69 0" or op401g7ojc* or brenzys or tnf receptor fusion protein or tnfr fc or recombinant human tnf or recombinant human dimeric tnf receptor type ii igg fusion protein or chs-0214 or chs0214 or dwp-422 or dwp422 or enia-11 or enia11 or gp-2015 or gp2015 or gp2015c or gp-2015c or hd-203 or hd203 or lbec-0101 or lbec0101 or sb-4 or sb4).mp,rn. (9565)

47 Adalimumab/ (5520)

48 (adalimumab* or humira or abp 501 or abp501 or abtd2e7 or amjevita or amgevita or bax 2923 or bax2923 gp 2017 or gp2017 or halimatoz or hefiya or hulio or hyrimoz or ibi 303 or ibi303 or imraldi or m 923 or m923 or msb 11022 or msb11022 or ons3010 or ons 3010 or "pf 06410293" or pf 6410293 or pf06410293 or pf6410293 or d2e7 or truxeda or cyltezo or solymbic or fys6t7f842* or 331731 18 1 or lu 200134 or lu200134 or d2e7 or hsdb 7851 or hsdb7851 or abp-501 or abp501 or bcd-057 or bcd057 or bi-695501 or bi695501 or chs-1420 or chs1420 or gp-2017 or gp2017 or sb-5 or sb5).mp,rn. (9270)

49 (golimumab* or cnto 148 or cnto148 or simponi or 476181 74 5 or 91x1klu43e*).mp,rn. (1343)

50 Certolizumab Pegol/ (621)

51 (certolizumab* or cdp 870 or cdp870 or cimzia or pegylated tumor necrosis factor alpha antibody fab fragment or pegylated tumour necrosis factor alpha antibody fab fragment or pha 738144 or pha738144 or czp or 428863 50 7 or g6adw90r16* or umd07x179e* or hsdb 7848 or hsdb7848).mp,rn. (1591)

52 Rituximab/ (15139)

53 (rituximab* or ct p10 or ctp10 or idec 102 or idec102 or idecc2b8 or idec c2b8 or monoclonal antibody idec c2b8 or gp2013 or gp 2013 or "pf 05280586" or pf05280586 or mabthera or mab thera or r 105 or r105 or reditux or rg 105 or rg105 or rituxan or rituxin or ritemvia or rituzena or rixathon or riximyo or ro 452294 or ro452294 or truxima or 174722 31 7 or 4f4x42syq6* or hsdb 7455 or hsdb7455).mp,rn. (25221)

54 (ofatumumab* or humax cd20 or humax-cd20 or humaxcd20 or humax-cd20-2f2 or arzerra or gsk 1841157 or gsk1841157 or gski841157 or human cd20 or omb 157 or omb157 or 679818 59 8 or m95kg522r0* or hsdb 8170 or hsdb8170).mp,rn. (780)

55 Abatacept/ (2960)

56 (abatacept* or ctla4 ig or ctla4 immunglobulin or ctla4 immunoglobulin g or ctla4ig or 'ctla 4 ig' or ctla-igg4m or ctla4-fc or bms 188667 or bms188667 or orencia or '332348 12 6' or 7d0yb67s97* or rg 2077 or rg2077 or rg-1046 or rg1046 or nulojix or lea29y or lea 29y or ctl4 fc or ctl4fc or belatacept or bms 224818 or bms224818 or belatacept or cytotoxic t lymphocyte associat* antigen 4).mp,rn. (5512)

57 (tabalumab* or ly 2127399 or ly2127399 or 1143503 67 6 or pqp8vh3mjw*).mp,rn. (58)

58 (pateclizumab* or mlta 3698a or mlta3698a or pro 283698 or pro283698 or 12202526 59 7 or qok1yyh7j2* or rg 7415 or rg7415).mp,rn. (6)

59 (secukinumab* or cosentyx or ain 457 or ain457 or 1229022 83 6 or dlg4eml025*).mp,rn. (1285)

60 (ixekizumab* or ly 2439821 or ly2439821 or taltz or 1329632 62 3 or 1143503 69 8 or bty153760o*).mp,rn. (644)

61 (brodalumab* or amg 827 or amg827 or khk 4827 or khk4827 or siliq or kyntheum or 1174395 19 7 or 6za31y954z*).mp,rn. (368)

62 (guselkumab* or cnto 1959 or cnto1959 or 1350289 85 8 or tremfya or 1350289 85 8 or 089658a12d*).mp,rn. (283)

63 Ustekinumab/ (1125)

64 (ustekinumab* or cnto 1275 or cnto1275 or stelara or "815610 63 0" or fu77b4u5z0* or 1275 cnto or 1275cnto or l04ac05 or tt-20 or tt20).mp,rn. (2261)

65 (mavrilimumab or "1085337 57 0" or 1158jdp9a* or cam 3001 or cam3001).mp,rn. (33)

66 exp Antirheumatic Agents/ (432542)

67 (dmard* or csdmard* or cs-dmard* or sdmard* or s-dmard* or tsdmard* or ts-dmard* or bdmard* or b-dmard*).ti,ab,kf. (5967)

68 (disease modifying adj3 (antirheum* or anti rheum*) adj3 (drug? or agent?)).ti,ab,kf. (6574)

69 ((antirheum* or anti rheum*) adj5 (drug? or agent?)).ti,ab,kf. (9206)

70 Methotrexate/ (38328)

71 (mxt or methotrexat* or metotrexat* or methoblastin or methylaminopterin* or amet?opterin* or abitrexate or antifolan or brimexate or a-met?opterin* or a-methpterin* or ai3-25299 or ai325299 or alpha-methopterin or biotrexate or emtexate or emtrexate* or emthexat* or enthexate* or farmitrexat* or farmotrex or fauldexato or folex or folex pfs or hdmtx or ifamet or imeth or maxtrex or metotressat* or methotrate* or methohexate* or mexate* or metrotex or metatrexato* or metex or metrex or metatrexan or maxtrex or meticil or metoject or metotrexin or novatrex* or neotrexate* or nordimet or lumexon or ledertrexate* or lanterel or rasuvo or r?eumatrex* or reditrex or rasuvo or texate or texate-t or tremetex or trexeron or trixilem or texorate or trexall or xaken or xatmep or otrexup or mpi-2505 or mpi2505 or mpi 5004 or mpi5004 or amethopterin* or 133073-73-1 or 15475-56-6 or 3ig1e710zn* or yl5fz2y5u1* or cl 14377 or cl14377 or ccris 1109 or ccris1109 or emt 25299 or emt25299 or nsc-740 or nsc740 or r-9985 or r9885 or x-133 or wr-19039 or wr19039).mp,rn. (55959)

72 Leflunomide/ (1537)

73 (leflunomid* or hwa 486 or hwa486 or su101 or su 101 or arava or arabloc or hsdb 7289 or hsdb7289 or g162gk9u4w* or 75706-12-6 or rs 34821 or rs34821).mp,rn. (2633)

74 lef.ti,ab,kf. (2570)

75 Sulfasalazine/ (4145)

76 (sulfasalazin* or sasp or ssz or salicylazosulfapyridin* or salicyl azo sulfapyridin* or sulphasalazin* or salazosulfapyridin* or salazosulfapiridin* or 'pyralin en' or azulfadin* or azulfidin* or asulfidin* or azulfide* or azulfin or colo-pleon or colopleon or disalazin or gastropyrin or pleon or "pleon ra" or "pyralin en" or rorasul or rosulfant or ulcol or ucine or salazopyrin* or ratio-sulfosalazin* or sulfasalazin* or 3xc8guz6cb* or accucol or asulfidin* or azopyrin* or azosulfidin* or benzosulfa or "brn 0356241" or brn0356241 or ccris 4713 or ccris4713 or einecs 209-974-3 or hsdb 3395 or hsdb3395 or nsc 203730 or nsc203730 or nsc 667219 or nsc667219 or reupirin or rorasul or salazo-sulfapyridin* or salazosulfpyridin* or salazopiridazin* or salazopyridin* or salazopirin* or salazodin or salisulf or salipyr or saridine or si-88 or si88 or sulculon or sulfasalazin* or sulfasalizin* or sulphasalazin* or w-t sasp oral or 599-79-1 or "s.a.s. 500" or "s.a.s.-500" or sas-500 or zopyrin*).mp,rn. (8144)

77 Hydroxychloroquine/ (4150)

78 (hydroxychloroquin* or hcq or 747-3-4 or 8q2869cnvh* or 118-42-3 or 4qwg6n8qkh* or hydroxychlorochin* or hydrochloroquin* or hydrocloroquin* or oxychlorochin or oxychloroquin* or plaquenil or plaquinol or "brn 0253894" or brn0253894 or einecs 204-249-8 or einecs204-249-8 or hidroxicloroquin* or idrossiclorochin* or oxichlorochin* or oxichloroquin* or oxiklorin or win1258 or win 1258 or z0188 or einecs 212-019-3 or einecs212-019-3 or chloroquinol or dimard or ercoquin or evoquin or erquin or geniquin or quensyl or sn 8137 or sn8137 or toremonil or yuma).mp,rn. (7944)

79 Gold Sodium Thiomalate/ (1262)

80 (gold sodium thiomalate* or aurothiomalate* or aurolate* or gold thiomalate* or gold disodium thiomalate* or gold thiomalic acid* or miocrin or miocrisin or monogold disodium thiomalate* or myochrysin* or myocrisin* or myocrysin* or sodium aurothiomalate* or sodium gold thiomalate* or sodium thiomalate* gold or sodium thiomalatoaurate* or tauredon or taureodon or tauredone or thiomalate gold or thiomalatoaurate sodium or 1244-57-4 or 39377-38-3 or 4846-27-9 or 74916-57-7 or e4768zy6gm* or aurothiomala-natrium or aurot?iomalato sodico or monogold* sodium salt or dinatrium 2-aurothio-succinat or einecs 235-479-7 or einecs235-479-7 or hsdb 7173 or hsdb7173 or kidon or natrii aurothiomalas* or shiosol or gold mercatpsuccinate*).mp,rn. (1547)

81 Aurothioglucose/ (834)

82 (thioglucosoaurate* or gold thioglucose* or gold thio glucose* or thioglucose gold or aureotan or solganal or solganol or gold-50 or gold50 or solganal b or b oleosum solganal or auromyose* or aurothioglucose* or auro thioglucose* or aurotan or aurumine or aurumin or authron or brenol or ccris 59 or ccris59 or einecs 235-365-7 or einecs235-365-7 or glysanol b or goldthioglucose* or goldtioglucose* or hsdb 7174 or hsdb7174 or oronol or romosol or skf 10056 or skf10056 or 2p2v9q0e78* or 12192-57-3).mp,rn. (1134)

83 ((gold* or auro*) adj10 (inject* or intramuscular* or im or intra muscular*)).ti,ab,kf,kw. (2774)

84 Organogold Compounds/ (942)

85 (aurothiopropanol* or allochrysine or allocrisine or aurotiopoe or aurotioprol or 27279-43-2 or g7097j63e9* or auromercapto propanol sulfonate sodium or auromercaptopropanolsulfonate sodium or aurothioisopropanolsulfonate sodium or sodium aurothiopropanolsulfonate or gold sodium thiopropanol sulfonate or sodium auromercaptopropanol sulfonate).mp,rn. (91)

86 Chloroquine/ (14646)

87 (chloroquin* or cq or 54-04-7 or 886u3h6uff* or aralen or aralene or arechin or arechine or arequin or chingamin or chlorochin* or khingamin or nivaquine or oe48649kh6n* or amokin or amokine or anaclor or aralan or arequine or artriquine or avloclor or avoclor or arthrochin or artrichin or bemaco or bemaphata or bemaphate or bemasulph or benaquin or bipiquin or cadiquin or "brn 0482809" or brn0482809 or capquin or ccris 3439 or ccris3439 or chemochin or chemochine or chingamine or chingaminum or chloraquin* or chlorquin or chlorquine or choloquine or choroquine sulfate or choroquine sulphate or cindacin or clo-kit junior or clorichina or clorichine or cloriquine or clorochina or delagil or delagyl or diclokin or chlorofoz or cindachin or clorochina or cloroquina or cocatrit or dichinalex or diquinalex or diroquine or emquin or genocin or gontochin or gontochine or gontoquine or einecs 200-191-2 or einecs200-191-2 or elestol or gontochin or heliopar or hsdb 3029 or hsdb3029 or imagon or iroquine or klorokin or klorokine or klorokinfosfat or lagaquin or lapaquin or malaquin or malaren or malarex or malarivon or malaviron or maliaquine or maquine or mesylith or mexaquin or mirquin or neochin or nivaquin or nivachine or nivaquine b or nivaquine dp or nivaquine forte or "p roquine" or nsc 187208 or nsc187208 or pfizerquine or quensyl or quinachlor or quinercyl or quingamine or quinilon or quinoline or quinoscan or repal or resochen or resochene or resochin or resochin junior or resochina or resochine or resochinon or resoquina or resoquine or reumachlor or ronaquine or roquine or rp 3377 or rp3377 or sanoquin or sanoquine or silbesan or siragan or sirajan or sn 7618 or sn7618 or solprina or solprine or sopaquin or st 21 or tresochin or tresoquine or trochin or trochine or troquine or weimerquin or w 7618 or w7618 or win 244 or win244).mp,rn. (35357)

88 exp Adrenal Cortex Hormones/ (400140)

89 exp Prednisolone/ (51556)

90 Prednisone/ (39684)

91 Cortisone/ (19620)

92 Hydrocortisone/ (72560)

93 exp Steroids/ (863736)

94 adrenal cortex hormones.ti,ab,kf,rn. (65571)

95 prednisolone*.mp,rn. (46828)

96 methylprednisolone*.mp,rn. (27239)

97 (prednisolone* or predonine or di-adreson-f or diadresonf or 9phq9y1olm* or prednisolona* or prednisolonum* or nsc-9120 or nsc9120 or nsc 9900 or nsc9900 or delta-dehydrocortisol* or delta-dehydrocortisone* or delta-hydrocortisone or hydroretrocortine or metacortandralone* or brn 1354103 or brn1354103 or bubbli-pred or ccris 980 or ccris980 or co-hydeltra or codelcortone or cordrol or cortalone or cotogesic or cotolone or decaprednil or decortin h or delcortol or dehydrohydrocortisone* or delta-cortef or deltacortef or delta-ef-cortelan or delta-stab or deltacortenol or deltacortril or deltahydrocortisone* or deltisilone or derpo pd or dexa-dortidelt hostacortin h or di-adreson f or dicortol or donisolone or dydeltrone or eazolin d or ec 200-021-7 or ec200-021-7 or einecs 200-021-7 or einecs200-021-7 or erbacort or erbasona or estilsona or fernisolone or fernisolone p or hostacortin h or hsdb 3385 or hsdb3385 or hydeltra or hydeltrone or hydrodeltalone or hydrodeltisone or hydroretrocortin or hydroretrocortine* or k 1557 or k1557 or lentosone or meti-drm or meticortelone or orapred or orapred odt or paracortol or paracotol or pediapred or precortancyl or precortilon or precortisyl or predne-dome or prednelan or prednicen or predniliderm or predniretard or prednis or predonin or predonine* or prelon or prelone or prenolone or rolisone or scherisolon? or solone or sterolone or ulacort or ultracorten h or ultracortene h adelcort or antisolon or antisolone or aprednisolon? or benisolon? or caberdelta or co hydeltra or codelcortone or ompresolon).mp,rn. (46916)

98 (cortadeltona or cortelinter or cortisolone or dacortin or dacortin h or dacrotin or decaprednil or decortil or delta cortril or delta hycortol or deltacortenolo or deltacortil or deltacortoil or deltaderm or deltadglycortril or deltahycortol or deltahydrocortison? or deltaophticor or deltasolone or deltastab or deltidrosol or deltisilone or deltisolon? or deltolasson? or deltosona or deltosone or depo-predate or dermosolon or dhasolone or di adreson? f or diadreson? f or dicortol or domucortone or encortelon? or encortolon or equisolon or glistelone or hefasolon or hydrelta or hydrocortancyl or hydrocortidelt or hydradeltalone or hydradeltisone or inflanefran or insolone or keteocort h or key-pred or key-pred sp or lenisolone or leocortol or liquipred or mediasolone or meprisolon? or metacortalon? or metacortelone or meti derm or metiderm or meticortelone or morlone or mydrapred or nisolon? or opredsone or panafcortelone or panafcortolone or panafort or paracortol pr phlogex or pre cortisyl or preconin or precortalon or precortancyl or precortisyl or pred ject 50 or predacort 50 or predaject 50 or predalone 50 or predartrina or predartrine or predate 50 or predeltilone or predisole or predisyr or predne dome or prednecort or prednedome or prednelan or predni coelin or predni h tablinen or predni helvacort or prednicoelin or prednicort or prednicortelone or prednifor drops or predniment or prednorsolon? or predorgasolon? or prenilone or preventan or prezolon or rubycort or serilone or solonda or solupren? or spiricort or spolotane or supercorti#ol or taracortelone or walesolone or wysolone).mp,rn. (63)

99 (methylprednisolone * or methylprednisolona* or methylprednisolonum* or metipred or urbason or medrol or x4w7zr7023* or 83-43-2 or besonia or brn 23403300 or brn2340300 or a-methapred or artisone-wyeth or besonia or depo-medrol or dopomedrol ec 201-4764 pr ec2014764 or einecs 201-476-4 or einecs201-476-4 or esametone or firmacort or hsdb3127 or hsdb3127 or lemod or medesone or medixon or medlone 21 or medrate or medrol or medrone or mesopren or metastab or methyleneprednisolone* or metilbetasone or metrisone or metrocort or metysolon or moderin or nirypan or noretona or nsc 19987 or nsc19987 or predni n tablinen or prednol-l or promacortine or reactenol or sieropresol or solomet or summicort or suprametil or u 7532 or u7532 or "u 67 590a" or urbason or urbasone or wyacort or adlone 40 or adlone 80 or beta methylprednisolone or medalone 80 or depmedalone or deproject 80 or depopred

or esametone or firmacort or med-jec-40 or medixon or mednin or medralone or mepredprednisolone or meprelon or mesopren or methacort 40 or methacort 80 or methyxlcotol or methylcotolone or methylpred dp or methylsterolone or metidrol or metycortin or metypred or metypresol or neomedrone or solu decortin or urbason).mp,rn. (19873)

100 (prednison or prednisone or prednisona or prednisonum or dehydrocortisone or delta cortisone or rectodelt or sterapred or ultracorten or winpred or apo prednisone or cortan or cartancyl or panafcort or cutason or decortin or dacortin or decortisyl or deltasone or encortone or encorton or enkorton or enkortolon or kortancyl or liquid pred or meticorten or orasone or panasol or predni tablinen or prednidib or predniment or pronisone or vb0r961hzt* or 53-03-2 or adasone or al3-52939 or ancortone or bicortone or cartancyl or ccris 2646 or ccrs2646 or colisone or cortan or cortidelt or cotone or dacorten or dacortin or decortancyl or dekortin or dellacort or delta cortelan or delta cortisone or delta dome or deltacortene or deltacortisone or deltacortone or deltasone or deltison or deltisona or deltra or di adreson or diadreson or econosone or einecs 200-160-3 or einecs200-160-3 or fernisone or fiasone or hostacortin or hsdb 3168 or hsdb3168 or incocortyl or juvason or lisacort or lodotra or lodtra).mp,rn. (54312)

101 (me-korti or metacortandracin or metacorten or nci c04897 or ncic04897 or nisona or nizon or novoprednisone or nsc 10023 or nsc10023 or nurison or orasone or panafcort or paracort or prarmenison or pehacort or predeltin or prednicen-m or prednicorm or prednicort or prednicot or prednilonga or prednitone or prednizon or prednovister or presone or pronison or rayos or rectodelt or retrocortine or servisone or sterapred or supercortio or u 6020 or u6020 or ultracorten or ultracortene or winpred or wojtab or zenadrid).mp,rn. (356)

102 cortisone?.mp,rn. (23582)

103 corticosteroid*.mp,rn. (107301)

104 corticoid*.mp,rn. (6616)

105 glucocorticoid*.mp,rn. (115010)

106 corticosterone*.mp,rn. (34972)

107 hydrocortisone*.mp,rn. (78072)

108 dexamethasone*.mp,rn. (73300)

109 beclomethasone*.mp,rn. (3852)

110 (triamcinolone* or 1zk20vi6ty* or 124-94-7 or aristocort or volon or fluoxyprednisolon* or tiamcinolonum or triamcinolonum* or triamcinolona* or brn 2341955 or brn2341955 or cl 19823 or cl19823 or einecs 204-718-7 or einecs204-718-7 or hsdb 3194 or hsdb3194 or nsc 13397 or nsc13397).mp,rn. (12020)

111 (steroid? adj10 (therap* or treat* or drug? or inject* or intramuscular* or intra muscular* or im or intravenous* or intra venous* or iv or intraarticular* or intra articular* or ia or administrat* or oral or orally or parenteral*)).ti,ab,kf. (74695)

112 Janus Kinase Inhibitors/ (476)

113 ((jak or jak1 or jak2 or jak3 or janus kinase) adj10 inhibitor*).mp,rn. (6622)

114 (janus adj4 kinase adj10 inhibitor?).mp,rn. (2647)

115 exp Janus Kinases/ai [Antagonists & Inhibitors] (2325)

116 (baricitinib* or 1187594-09-7 or isp4442i3y* or "incb 028050" or incb028050 or ly-3009104 or ly3009104).mp,rn. (464)

117 (tofacitinib* or 477600-75-2 or 87la6fu830* or hsdb 8311 or hsdb8311 or cp-690 550 or cp 690550 or cp690550).mp,rn. (1683)

118 (filgotinib* or 1206161-97-8 or 3xvl385q0m* or "glpg 0634" or glpg0634).mp,rn. (117)

119 (upadacitinib* or 1310726-60-3 or 4RA0KN46E0* or abt 494 or abt494).mp,rn. (150)

120 (ruxolitinib* or rux or 941678-49-5 or 82s8x8xx8h* or hsdb 8259 or hsdb8259 or inc 424 or inc424 or incb424 or "incb 018424" or incb018424 or incb 18424 or incb18424 or r-ruxolitinib* or jakafi or jakavi).mp,rn. (1761)

121 (itacitinib or "incb 039110" or incb039110 or incb 39110 or incb39110 or 1334298-90-6 or 19j3781lpm*).mp,rn. (26)

122 (ag490 or ag 490 or tyrphostin b42 or tyrphostin ag 490 or tyrphostin ag490 or 133550-30-8).mp,rn. (1530)

123 (fedratinib* or tg101348 or tg 101348 or sar302503 or sar 302503 or 936091-26-8 or 6l1xp550i6* or inrebic).mp,rn. (158)

124 (ritlecitinib* or pf-06651600 or pf06651600 or pf 6651600 or pf6651600 or 2140301-97-7 or 132lf5wgh4* or 2192215-81-7 or eag4t1459k* or ritlecitinib malonate or ritlecitinib toslate or ritlecitinib tosylate or ritlecitinib propanedioate).mp,rn. (10)

125 (peficitinib* or peficitinib hydrobromide).mp,rn. (66)

126 or/41-125 (1535816)

127 29 and 126 (54066)

128 40 or 127 (54602)

129 exp "Treatment Adherence and Compliance"/ (250084)

130 (adhere* or adhering*).ti,ab,kf. (202757)

131 (complian* or complying*).ti,ab,kf. (138490)

132 (prefer* adj10 (patient? or patients*)).ti,ab,kf. (50259)

133 noncomplian*.ti,ab,kf. (8544)

134 nonadheren*.ti,ab,kf. (10018)

135 ((treatment* or therap* or drug? or medication? or agent? or formulation?) adj1 refus* adj10 (patient? or patients*)).ti,ab,kf. (976)

136 Attitude to Health/ (84202)

137 Health Knowledge, Attitudes, Practice/ (114570)

138 attitude*.ti,ab,kf. (158796)

139 (interrupt* adj3 (treatment* or therapy* or therapeutic*)).ti,ab,kf. (7231)

140 ((drug$ or agent$ or medication* or formulation$ or biologic* or biosimilar$ or therapy* or treatment* or therapeutic*) adj3 prefer*).ti,ab,kf. (24992)

141 138 or 139 or 140 (190183)

142 (patient? or patients*).mp. (7315755)

143 141 and 142 (81352)

144 Decision Making, Shared/ (690)

145 shared decision*.ti,ab,kw. (9872)

146 Decision Making/ (97186)

147 ((decision* or decide* or choice? or chosen) adj3 (patient? or patients*)).ti,ab,kf,kw. (42879)

148 ((decision* or decide* or choice? or chosen) adj3 (treatment? or therap* or drug* or agent* or medication* or formulation* or biosimilar? or bio similar? or biologic*) adj10 (patient? or patients*)).ti,ab,kw,kf. (32225)

149 Refusal to Participate/ (628)

150 ((patient? or patients*) adj10 (perception* perceive* or perspective*)).ti,ab,kf. (30250)

151 (drop out? or dropout?).ti,ab,kf. (18268)

152 motivat*.ti,ab,kf. (141787)

153 exp Motivation/ (173012)

154 barrier?.ti,ab,kf. (315511)

155 ((patient? or patients*) adj6 satisfaction*).ti,ab,kf,kw. (57774)

156 Maintenance/ (2214)

157 Maintenance Chemotherapy/ (1777)

158 maintenance*.ti,ab,kf. (284576)

159 (maintain* adj10 (therap* or treatment* or drug? or agent? or medication* or chemotherap* or dose? or dosage?)).ti,ab,kf. (59958)

160 persistenc*.ti,ab,kf. (95589)

161 Survival/ (4768)

162 exp survival analysis/ (304869)

163 Survival Rate/ (177118)

164 survival*.ti,ab,kf. (990471)

165 (drug? adj8 retention*).ti,ab,kf. (3860)

166 retention rate?.ti,ab,kf. (5342)

167 retention*.ti,ab,kf. and (dt.fs. or drug?.mp.) (42453)

168 "Early Termination of Clinical Trials"/ (659)

169 exp therapeutic index/ (65)

170 exp treatment failure/ (35728)

171 "duration of therapy"/ (209)

172 treatment outcome/ (1002694)

173 Withholding Treatment/ (12089)

174 Substance Withdrawal Syndrome/ (21498)

175 ((duration* or length) adj3 (treatment* or therapy* or therapies* or therapeutic* or medication*)).ti,ab,kf. (55575)

176 ((treatment* or therapy* or therapeutic*) adj1 failure*).ti,ab,kf. (35644)

177 ((treatment* or therap* or chemotherap*) adj3 lack adj3 efficacy*).ti,ab,kf. (375)

178 cessation*.ti,ab,kf. (76774)

179 withdraw*.ti,ab,kf. (129830)

180 (response* or respond*).ti,ab,kf. (3047902)

181 dose-response relationship, drug/ or hormesis/ (407069)

182 (discontinu* or dis-continu*).ti,ab,kf. (131590)

183 switch*.ti,ab,kf. (174269)

184 (continuing or continue*).ti,ab,kf. (488925)

185 economics/ (27279)

186 exp "Costs and Cost Analysis"/ (241622)

187 Economics, Pharmaceutical/ (2969)

188 exp Economics, Hospital/ (24895)

189 exp Economics, Medical/ (14237)

190 Economics, Nursing/ (4002)

191 (economic? or oeconomic? or cost or costs or costly or costing or price or prices or pricing or pharmaco?economic?).ti,ab,kf. (819808)

192 (value adj2 money).ti,ab,kf. (1856)

193 Health Expenditures/ (20889)

194 expenditure*.ti,ab,kf. (59886)

195 exp Budgets/ (13780)

196 budget?.ti,ab,kf. (27599)

197 quality-adjusted life years/ (12775)

198 (quality adjusted or adjusted life year?).ti,ab,kf. (18093)

199 (qaly or qalys).ti,ab,kf. (11259)

200 (utility or utilities).ti,ab,kf. (217859)

201 (health utility index* or hui or hui1 or hui2 or hui3 or hui-1 or hui-2 or hui-3).ti,ab,kf. (1710)

202 "Quality of Life"/ and ec.fs. (10356)

203 "Quality of Life"/ and (("quality of life" or qol) adj1 (score? or measure?)).ti,ab,kf. (13368)

204 "Quality of Life"/ and (health adj3 status).ti,ab,kf. (9363)

205 "Quality of Life"/ and (("quality of life" or qol) adj3 (improv* or chang*)).ti,ab,kf. (28885)

206 ((dose? or dosage?) adj8 (adjust* or modificat*)).ti,ab,kf. (26737)

207 exp Drug Administration Schedule/ (103311)

208 (interval? adj8 adjust*).ti,ab,kf. (49227)

209 ((dose? or dosage?) adj8 (taper or tapers or tapering or tapered)).ti,ab,kf. (2818)

210 129 or 130 or 131 or 132 or 133 or 134 or 135 or 136 or 137 or 143 or 144 or 145 or 146 or 147 or 148 or 149 or 150 or 151 or 152 or 153 or 154 or 155 or 156 or 157 or 158 or 159 or 160 or 161 or 162 or 163 or 164 or 165 or 166 or 167 or 168 or 169 or 170 or 171 or 172 or 173 or 174 or 175 or 176 or 177 or 178 or 179 or 180 or 181 or 182 or 183 or 184 or 185 or 186 or 187 or 188 or 189 or 190 or 191 or 192 or 193 or 194 or 195 or 196 or 197 or 198 or 199 or 200 or 201 or 202 or 203 or 204 or 205 or 206 or 207 or 208 or 209 (7962551)

211 128 and 210 (24372)

212 exp Registries/ (100173)

213 (register or registry or registries or registered or registration*).ti,ab,kf. (409544)

214 Cohort Studies/ (275542)

215 (cohort* adj6 (study* or studies* or analy*)).ti,ab,kf. (318268)

216 (cohortstudy* or cohortstudies* or cohortanaly*).ti,ab,kf. (4)

217 cohort?.ti,ab,kf. and (study* or studies or analy* or design or trial or trials).mp. (616233)

218 Prospective Studies/ (560411)

219 Retrospective Studies/ (864397)

220 exp Longitudinal Studies/ (141239)

221 (longitudinal or prospective* or retrospective*).ti,ab,kf. (1674909)

222 Follow-Up Studies/ (654383)

223 ((follow up or followup) adj6 (trial or trials or study or studies or analy*)).ti,ab,kf. (147400)

224 "Surveys and Questionnaires"/ (480388)

225 exp health care surveys/ or patient reported outcome measures/ or health surveys/ or behavioral risk factor surveillance system/ or exp health status indicators/ (412263)

226 (questionnaire* or questioning or questioned).ti,ab,kf. (574839)

227 interview/ (29502)

228 Interviews as Topic/ (63989)

229 exp qualitative research/ (59636)

230 interview*.ti,ab,kf. (374006)

231 qualitative.ti,ab,kf. (247442)

232 Focus Groups/ (31103)

233 focus group?.ti,ab,kf. (49770)

234 212 or 213 or 214 or 215 or 216 or 217 or 218 or 219 or 220 or 221 or 222 or 223 or 224 or 225 or 226 or 227 or 228 or 229 or 230 or 231 or 232 or 233 (4391251)

235 234 and 211 (9787)

236 limit 235 to yr="2012 - 2020" (5420)

237 limit 236 to english (5302)

238 exp animals/ not humans/ (4777411)

239 237 not 238 (5284)

240 ((eular or (european adj4 rheumat*)) adj8 (meeting* or conference* or congress*) adj8 annual).af. (70)

241 ((acr or (american college adj4 rheumat*)) adj8 (meeting* or conference* or congress*) adj8 annual).af. (142)

242 240 or 241 (178)

243 limit 242 to yr="2019 - 2020" (46)

244 239 and 243 (5)

245 239 not 244 (5279)

246 review.pt. (2750992)

247 congress.pt. (66508)

248 editorial.pt. (554796)

249 letter.pt. (1118652)

250 case reports.pt. (2150414)

251 246 or 247 or 248 or 249 or 250 (6269711)

252 245 not 251 (4613)

253 case report.ti. (250492)

254 case series.ti. (21897)

255 253 or 254 (272362)

256 252 not 255 (4590)

257 244 or 256 (4595)

1.3.5.2 EMBASE

#296,"#286 OR #295",5919,13 Jan 2021

#295,"#289 NOT #294",4807,13 Jan 2021

#294,"#290 OR #291 OR #292 OR #293",357745,13 Jan 2021

#293,"'case series':ti OR 'case series':tt",31092,13 Jan 2021

#292,"'case study'/exp",74658,13 Jan 2021

#291,"'case report':ti OR 'case report':tt",320500,13 Jan 2021

#290,"'case report'/de",2644470,13 Jan 2021

#289,"#287 NOT #288",5350,13 Jan 2021

#288,"#281 NOT #286 AND ([conference abstract]/lim OR [conference paper]/lim OR [conference review]/lim OR [editorial]/lim OR [letter]/lim OR [review]/lim)",8661,13 Jan 2021

#287,"#281 NOT #286",14011,13 Jan 2021

#286,"#281 AND #284 AND [2019-2020]/py",1112,13 Jan 2021

#285,"#281 AND #284",5238,13 Jan 2021

#284,"#282 OR #283",50862,13 Jan 2021

#283,"acr:nc AND annual:nc AND meeting:nc AND rheumatology:nc",19451,13 Jan 2021

#282,"'eular':nc AND 'annual european congress of rheumatology':nc",31411,13 Jan 2021

#281,"#276 NOT #280",15123,13 Jan 2021

#280,"#278 NOT #279",1065666,13 Jan 2021

#279,"human:ti OR humans:ti OR patient$:ti OR client$:ti OR participant$:ti OR human:tt OR humans:tt OR patient$:tt OR client$:tt OR participant$:tt",4106499,13 Jan 2021

#278,"#264 AND #277",1098719,13 Jan 2021

#277,"#267 OR #268",2462765,13 Jan 2021

#276,"#262 NOT #275",15129,13 Jan 2021

#275,"#269 NOT #274",7184071,13 Jan 2021

#274,"#270 OR #271 OR #272 OR #273",23367961,13 Jan 2021

#273,"human:tt OR humans:tt OR patient$:tt OR client$:tt OR participant$:tt",27790,13 Jan 2021

#272,"human:ti,ab OR humans:ti,ab OR patient$:ti,ab OR client$:ti,ab OR participant$:ti,ab",13477123,13 Jan 2021

#271,"'human experiment'/exp",530062,13 Jan 2021

#270,"'human'/exp",22896144,13 Jan 2021

#269,"#263 OR #264 OR #265 OR #266 OR #267 OR #268",30183289,13 Jan 2021

#268,"mouse:tt OR mice:tt OR rat:tt OR rats:tt OR animal:tt OR animals:tt OR dog:tt OR dogs:tt OR cat:tt OR cats:tt OR bovine:tt OR sheep:tt OR swine:tt OR porcine:tt OR pigs:tt OR piglets:tt OR rabbit$:tt OR lamb:tt OR lambs:tt OR cattle:tt OR monkey:tt OR monkeys:tt OR trout:tt OR marmoset*:tt OR zebrafish:tt OR 'zebra fish':tt",13792,13 Jan 2021

#267,"mouse:ti OR mice:ti OR rat:ti OR rats:ti OR animal:ti OR animals:ti OR dog:ti OR dogs:ti OR cat:ti OR cats:ti OR bovine:ti OR sheep:ti OR swine:ti OR porcine:ti OR pigs:ti OR piglets:ti OR rabbit$:ti OR lamb:ti OR lambs:ti OR cattle:ti OR monkey:ti OR monkeys:ti OR trout:ti OR marmoset*:ti OR zebrafish:ti OR 'zebra fish':ti",2461810,13 Jan 2021

#266,"'nonhuman'/de",6432717,13 Jan 2021

#265,"'animal model'/exp",1448726,13 Jan 2021

#264,"'animal experiment'/exp",2649777,13 Jan 2021

#263,"'animal'/exp",28447779,13 Jan 2021

#262,"#233 AND #259 AND [2012-2020]/py AND [english]/lim",15136,13 Jan 2021

#261,"#233 AND #259 AND [2012-2020]/py",15263,13 Jan 2021

#260,"#233 AND #259",20306,13 Jan 2021

#259,"#234 OR #235 OR #236 OR #237 OR #238 OR #239 OR #240 OR #241 OR #242 OR #243 OR #244 OR #245 OR #246 OR #247 OR #248 OR #249 OR #250 OR #251 OR #252 OR #253 OR #254 OR #255 OR #256 OR #257 OR #258",5816107,13 Jan 2021

#258,"'focus group$':ti,ab,kw OR 'focus group$':tt",62045,13 Jan 2021

#257,"'focus group'/de",32,13 Jan 2021

#256,"'focus group discussion'/de",26,13 Jan 2021

#255,"'focus group interview'/de",11,13 Jan 2021

#254,"qualitative:ti,ab,kw OR qualitative:tt",307935,13 Jan 2021

#253,"interview$:ti,ab,kw OR interview$:tt",386317,13 Jan 2021

#252,"'qualitative research'/exp",83239,13 Jan 2021

#251,"'interview'/exp",297131,13 Jan 2021

#250,"questionnaire*:tt OR questioning:tt OR questioned:tt",1122,13 Jan 2021

#249,"questionnaire*:ti,ab,kw OR questioning:ti,ab,kw OR questioned:ti,ab,kw",830976,13 Jan 2021

#248,"'questionnaire design'/de",59,13 Jan 2021

#247,"'questionnaire'/exp",744095,13 Jan 2021

#246,"(('follow up' OR followup) NEAR/6 (trial OR trials OR study OR studies OR analy* OR design)):ti,ab,kw",237558,13 Jan 2021

#245,"'follow up'/de",1622453,13 Jan 2021

#244,"'long term follow up'/de",27,13 Jan 2021

#243,"'interview'/exp",297131,13 Jan 2021

#242,"longitudinal:tt OR prospective*:tt OR retrospective*:tt",5814,13 Jan 2021

#241,"longitudinal:ti,ab,kw OR prospective*:ti,ab,kw OR retrospective*:ti,ab,kw",2590536,13 Jan 2021

#240,"'retrospective study'/de",1015923,13 Jan 2021

#239,"'longitudinal study'/exp",147182,13 Jan 2021

#238,"'prospective study'/de",642001,13 Jan 2021

#237,"((cohort* NEAR/6 (study* OR studies* OR analy*)):ti,ab,kw) OR ((cohort* NEAR/6 (study* OR studies* OR analy*)):tt) OR cohortstudy*:ti,ab,kw OR cohortstudies*:ti,ab,kw OR cohortanaly*:ti,ab,kw OR cohortstudy:tt OR cohortstudies:tt OR cohortanaly*:tt",489026,13 Jan 2021

#236,"'cohort analysis'/de",637513,13 Jan 2021

#235,"register:ti,ab,kw OR registry:ti,ab,kw OR registries:ti,ab,kw OR registered:ti,ab,kw OR registration$:ti,ab,kw OR register:tt OR registry:tt OR registries:tt OR registered:tt OR registration$:tt",565775,13 Jan 2021

#234,"'register'/de",113724,13 Jan 2021

#233,"#136 AND #232",52207,13 Jan 2021

#232,"#137 OR #138 OR #139 OR #140 OR #141 OR #142 OR #143 OR #144 OR #145 OR #146 OR #147 OR #148 OR #149 OR #150 OR #151 OR #152 OR #158 OR #159 OR #160 OR #161 OR #162 OR #163 OR #164 OR #165 OR #166 OR #167 OR #168 OR #169 OR #170 OR #171 OR #172 OR #173 OR #174 OR #175 OR #176 OR #177 OR #178 OR #179 OR #180 OR #181 OR #182 OR #183 OR #184 OR #185 OR #186 OR #187 OR #188 OR #189 OR #190 OR #191 OR #192 OR #193 OR #194 OR #195 OR #196 OR #197 OR #198 OR #199 OR #200 OR #201 OR #202 OR #203 OR #204 OR #205 OR #206 OR #207 OR #208 OR #209 OR #210 OR #211 OR #212 OR #213 OR #214 OR #215 OR #216 OR #217 OR #218 OR #219 OR #220 OR #221 OR #222 OR #223 OR #224 OR #225 OR #226 OR #227 OR #228 OR #229 OR #230 OR #231",10088921,13 Jan 2021

#231,"(interval$ NEAR/8 adjust*):tt",0,13 Jan 2021

#230,"(interval$ NEAR/8 adjust*):ti,ab,kw",54583,13 Jan 2021

#229,"'drug dose regimen'/de",34785,13 Jan 2021

#228,"'drug response'/exp",1144683,13 Jan 2021

#227,"((dose$ OR dosage$) NEAR/8 (adjust* OR modificat*)):tt",31,13 Jan 2021

#226,"((dose$ OR dosage$) NEAR/8 (adjust* OR modificat*)):ti,ab,kw",45261,13 Jan 2021

#225,"'quality of life'/de AND ((health NEAR/3 status):tt)",1,13 Jan 2021

#224,"'quality of life'/de AND ((health NEAR/3 status):ti,ab,kw)",16810,13 Jan 2021

#223,"'quality of life'/de AND ((('quality of life' OR qol) NEAR/1 (score$ OR measure$)):tt)",4,13 Jan 2021

#222,"'quality of life'/de AND ((('quality of life' OR qol) NEAR/1 (score$ OR measure$)):ti,ab,kw)",28933,13 Jan 2021

#221,"'health utility index*':tt OR hui:tt OR hu1:tt OR hui2:tt OR hui3:tt",971,13 Jan 2021

#220,"'health utility index*':ti,ab,kw OR hui:ti,ab,kw OR hu1:ti,ab,kw OR hui2:ti,ab,kw OR hui3:ti,ab,kw",2781,13 Jan 2021

#219,"utility:tt OR utilities:tt",23,13 Jan 2021

#218,"utility:ti,ab,kw OR utilities:ti,ab,kw",301792,13 Jan 2021

#217,"'cost utility analysis'/de",10098,13 Jan 2021

#216,"qaly:ti,ab,kw OR qalys:ti,ab,kw OR qaly:tt OR qualys:tt",21131,13 Jan 2021

#215,"'quality adjusted':ti,ab,kw OR 'adjusted life year$':ti,ab,kw OR 'quality adjusted':tt OR 'adjusted life year$':tt",26322,13 Jan 2021

#214,"'quality adjusted life year'/de",27936,13 Jan 2021

#213,"budget$:ti,ab,kw OR budget$:tt",36570,13 Jan 2021

#212,"expenditure$:tt NOT energy:tt",3,13 Jan 2021

#211,"expenditure$:ti,ab,kw NOT energy:ti,ab,kw",42960,13 Jan 2021

#210,"'expenditures'/de",120,13 Jan 2021

#209,"(value NEAR/2 money):tt",1,13 Jan 2021

#208,"(value NEAR/2 money):ti,ab,kw",2533,13 Jan 2021

#207,"economic$:tt OR oeconomic$:tt OR cost:tt OR costs:tt OR costly:tt OR costing:tt OR price:tt OR prices:tt OR pricing:tt OR pharmaco$economic$:tt",1566,13 Jan 2021

#206,"economic$:ti,ab,kw OR oeconomic$:ti,ab,kw OR cost:ti,ab,kw OR costs:ti,ab,kw OR costly:ti,ab,kw OR costing:ti,ab,kw OR price:ti,ab,kw OR prices:ti,ab,kw OR pricing:ti,ab,kw OR pharmaco$economic$:ti,ab,kw",1058579,13 Jan 2021

#205,"'pharmacoeconomics'/de",7484,13 Jan 2021

#204,"'economic evaluation'/exp",313199,13 Jan 2021

#203,"'health care cost'/exp",299369,13 Jan 2021

#202,"continuing:ti,ab,kw OR continue*:ti,ab,kw OR continuing:tt OR continue*:tt",692860,13 Jan 2021

#201,"switch*:ti,ab,kw OR switch*:tt",220737,13 Jan 2021

#200,"discontinu*:ti,ab,kw OR 'dis-continue*':ti,ab,kw OR discontinu*:tt OR 'dis-continue*':tt",213846,13 Jan 2021

#199,"response$:tt OR respond*:tt",798,13 Jan 2021

#198,"response$:ti,ab,kw OR respond*:ti,ab,kw",3903155,13 Jan 2021

#197,"withdraw*:ti,ab,kw OR withdraw*:tt",182883,13 Jan 2021

#196,"'cessation*':ti,ab,kw OR 'cessation*':tt",103134,13 Jan 2021

#195,"'cessation'/de",32,13 Jan 2021

#194,"((treatment* OR therap* OR therapeutic OR chemotherap*) NEAR/3 lack NEAR/3 efficacy*):tt",0,13 Jan 2021

#193,"((treatment* OR therap* OR therapeutic* OR chemotherap*) NEAR/3 lack NEAR/3 efficacy*):ti,ab,kw",963,13 Jan 2021

#192,"((treatment* OR therapy* OR therapeutic*) NEAR/1 failure*):tt",4,13 Jan 2021

#191,"((treatment* OR therapy* OR therapeutic*) NEAR/1 failure*):ti,ab,kw",54428,13 Jan 2021

#190,"((duration* OR length) NEAR/3 (treatment* OR therapy* OR therapies* OR therapeutic*)):tt",1,13 Jan 2021

#189,"((duration* OR length) NEAR/3 (treatment* OR therapy* OR therapies* OR therapeutic*)):ti,ab,kw",93444,13 Jan 2021

#188,"'drug retention'/de",6827,13 Jan 2021

#187,"'treatment response'/de",274103,13 Jan 2021

#186,"'treatment failure'/exp",171207,13 Jan 2021

#185,"'treatment withdrawal'/exp",220949,13 Jan 2021

#184,"'treatment duration'/de",240572,13 Jan 2021

#183,"survival*:ti,ab,kw OR survival*:tt",1460204,13 Jan 2021

#182,"'survival'/exp",1163039,13 Jan 2021

#181,"'drug survival'/de",28,13 Jan 2021

#180,"persistenc*:ti,ab,kw OR persistenc*:tt",123850,13 Jan 2021

#179,"'drug persistence'/de",19,13 Jan 2021

#178,"'persistence'/de",78,13 Jan 2021

#177,"(maintain* NEAR/10 (therap* OR treatment* OR therapeutic$ OR drug$ OR agent$ OR medication$ OR chemotherap* OR dose$)):tt",2,13 Jan 2021

#176,"(maintain* NEAR/10 (therap* OR treatment* OR therapeutic$ OR drug$ OR agent$ OR medication$ OR chemotherap* OR dose$)):ti,ab,kw",89248,13 Jan 2021

#175,"maintenance*:ti,ab,kw OR maintenance*:tt",383431,13 Jan 2021

#174,"'maintenance therapy'/de OR 'maintenance chemotherapy'/de OR 'maintenance drug dose'/de",50984,13 Jan 2021

#173,"((patient OR patients* OR 'patient s*') NEAR/6 satisfaction*):tt",115,13 Jan 2021

#172,"((patient OR patients* OR 'patient s*') NEAR/6 satisfaction*):ti,ab,kw",86383,13 Jan 2021

#171,"'patient satisfaction'/de",142272,13 Jan 2021

#170,"barrier$:ti,ab,kw OR barrier$:tt",391187,13 Jan 2021

#169,"motivat*:ti,ab,kw OR motivat*:tt",169719,13 Jan 2021

#168,"'drop out$':ti,ab,kw OR 'dropout$':ti,ab,kw OR 'drop out$':tt OR 'dropout$':tt",26791,13 Jan 2021

#167,"((patient OR patients* OR 'patient s*') NEAR/10 (perception* OR perceive* OR perspective*)):tt",128,13 Jan 2021

#166,"((patient OR patients* OR 'patient s*') NEAR/10 (perception* OR perceive* OR perspective*)):ti,ab,kw",120491,13 Jan 2021

#165,"'refusal to participate'/de",1262,13 Jan 2021

#164,"((decision OR decide* OR choice$ OR chosen) NEAR/3 (treatment$ OR therap* OR therapeutic* OR drug* OR agent* OR medication*) NEAR/10 (patient OR patients* OR 'patient s*')):tt",4,13 Jan 2021

#163,"((decision OR decide* OR choice$ OR chosen) NEAR/3 (treatment$ OR therap* OR therapeutic* OR drug* OR agent* OR medication*) NEAR/10 (patient OR patients* OR 'patient s*')):ti,ab,kw",43869,13 Jan 2021

#162,"((decision* OR decide* OR choice$ OR chosen) NEAR/3 (patient OR patients* OR 'patient s*')):tt",21,13 Jan 2021

#161,"((decision* OR decide* OR choice$ OR chosen) NEAR/3 (patient OR patients* OR 'patient s*')):ti,ab,kw",69003,13 Jan 2021

#160,"'shared decision*':ti,ab,kw OR 'shared decision*':tt",13740,13 Jan 2021

#159,"'shared decision making'/de",7989,13 Jan 2021

#158,"#156 AND #157",87604,13 Jan 2021

#157,"patient:ti,ab,kw,de OR 'patient s*':ti,ab,kw,de OR patients*:ti,ab,kw,de OR patient:tt OR 'patient s*':tt OR patients*:tt",10853540,13 Jan 2021

#156,"#153 OR #154 OR #155",205675,13 Jan 2021

#155,"'treatment interruption'/de",2304,13 Jan 2021

#154,"'drug preference'/de",2659,13 Jan 2021

#153,"attitude*:ti,ab,kw OR attitude*:tt",201458,13 Jan 2021

#152,"'patient attitude'/de",69348,13 Jan 2021

#151,"'patient participation'/de",28333,13 Jan 2021

#150,"(((treatment* OR therap* OR therapeutic* OR drug$ OR medication* OR agent$) NEAR/1 refus* NEAR/10 (patient OR patients* OR 'patient s*')):ti,ab,kw) OR (((treatment* OR therap* OR therapeutic* OR drug$ OR medication$ OR agent$) NEAR/1 refus* NEAR/10 (patient OR patients* OR 'patient s*')):tt)",2690,13 Jan 2021

#149,"nonadheren*:ti,ab,kw OR nonadheren*:tt",29427,13 Jan 2021

#148,"noncomplian*:ti,ab,kw OR noncomplian*:tt",24471,13 Jan 2021

#147,"'patient dropout'/de",1696,13 Jan 2021

#146,"'treatment refusal'/de",19425,13 Jan 2021

#145,"complian*:tt OR complying*:tt",1098,13 Jan 2021

#144,"complian*:ti,ab,kw OR complying*:ti,ab,kw",219797,13 Jan 2021

#143,"adhere*:tt OR adhering*:tt",1139,13 Jan 2021

#142,"adhere*:ti,ab,kw OR adhering*:ti,ab,kw",291126,13 Jan 2021

#141,"((patient OR 'patient s' OR patients*) NEAR/10 prefer*):tt",28,13 Jan 2021

#140,"((patient OR 'patient s' OR patients*) NEAR/10 prefer*):ti,ab,kw",77563,13 Jan 2021

#139,"'medication compliance'/de",33850,13 Jan 2021

#138,"'patient compliance'/de",134920,13 Jan 2021

#137,"'patient preference'/de",19679,13 Jan 2021

#136,"#58 OR #135",103985,13 Jan 2021

#135,"#47 AND #134",102766,13 Jan 2021

#134,"#59 OR #60 OR #61 OR #62 OR #63 OR #64 OR #65 OR #66 OR #67 OR #68 OR #69 OR #70 OR #71 OR #72 OR #73 OR #74 OR #75 OR #76 OR #77 OR #78 OR #79 OR #80 OR #81 OR #82 OR #83 OR #84 OR #85 OR #86 OR #87 OR #88 OR #89 OR #90 OR #91 OR #92 OR #93 OR #94 OR #95 OR #96 OR #97 OR #98 OR #99 OR #100 OR #101 OR #102 OR #103 OR #104 OR #105 OR #106 OR #107 OR #108 OR #109 OR #110 OR #111 OR #112 OR #113 OR #114 OR #115 OR #116 OR #117 OR #118 OR #119 OR #120 OR #121 OR #122 OR #123 OR #124 OR #125 OR #126 OR #127 OR #128 OR #129 OR #130 OR #131 OR #132 OR #133",1474704,13 Jan 2021

#133,"'peficitinib'/de OR '4 [(5 hydroxyadamantan 2 yl) amino] 1h pyrrolo [2, 3 b] pyridine 5 carboxamide' OR '4 [(5 hydroxytricyclo [3.3.1.1 3, 7] dec 2 yl) amino] 1h pyrrolo [2, 3 b] pyridine 5 carboxamide' OR 'asp 015k' OR 'asp015k' OR 'peficitinib' OR 'peficitinib hydrobromide'",193,13 Jan 2021

#132,"'ritlecitinib'/exp OR '1 [2 methyl 5 (7h pyrrolo [2, 3 d] pyrimidin 4 ylamino) 1 piperidinyl] prop 2 en 1 one' OR '1 [2 methyl 5 (7h pyrrolo [2, 3 d] pyrimidin 4 ylamino) piperidin 1 yl] prop 2 en 1 one' OR '1 [2 methyl 5 [(7h pyrrolo [2, 3 d] pyrimidin 4 yl) amino] 1 piperidinyl] prop 2 en 1 one' OR '1 [2 methyl 5 [(7h pyrrolo [2, 3 d] pyrimidin 4 yl) amino] piperidin 1 yl] prop 2 en 1 one' OR '1 [5 (7h pyrrolo [2, 3 d] pyrimidin 4 ylamino) 2 methyl 1 piperidinyl] prop 2 en 1 one' OR '1 [5 (7h pyrrolo [2, 3 d] pyrimidin 4 ylamino) 2 methylpiperidin 1 yl] prop 2 en 1 one' OR '1 [5 [(7h pyrrolo [2, 3 d] pyrimidin 4 yl) amino] 2 methyl 1 piperidinyl] prop 2 en 1 one' OR '1 [5 [(7h pyrrolo [2, 3 d] pyrimidin 4 yl) amino] 2 methylpiperidin 1 yl] prop 2 en 1 one' OR 'pf 06651600' OR 'pf 6651600' OR 'pf06651600' OR 'pf6651600' OR 'ritlecitinib' OR 'ritlecitinib malonate' OR 'ritlecitinib propanedioate'",39,13 Jan 2021

#131,"'fedratinib'/de OR 'fedratinib' OR 'fedratinib dihydrochloride' OR 'fedratinib dihydrochloride monohydrate' OR 'fedratinib hydrochloride' OR 'inrebic' OR 'n (1, 1 dimethylethyl) 3 [[5 methyl 2 [[4 [2 (1 pyrrolidinyl) ethoxy] phenyl] amino] 4 pyrimidinyl] amino] benzenesulfonamide' OR 'n tert butyl 3 [[5 methyl 2 [[4 (2 pyrrolidin 1 ylethoxy) phenyl] amino] pyrimidin 4 yl] amino] benzenesulfonamide' OR 'n tert butyl 3 [[5 methyl 2 [4 [2 (pyrrolidin 1 yl) ethoxy] anilino] pyrimidin 4 yl] amino] benzenesulfonamide' OR 'n tert butyl 3 [5 methyl 2 [4 (2 pyrrolidin 1 ylethoxy) phenylamino] pyrimidin 4 ylamino] benzenesulfonamide' OR 'n tert butyl 3 [5 methyl 2 [4 [2 (1 pyrrolidinyl) ethoxy] phenylamino] 4 pyrimidinylamino] benzenesulfonamide' OR 'sar 302503' OR 'sar 302503a' OR 'sar302503' OR 'sar302503a' OR 'tg 101348' OR 'tg101348'",648,13 Jan 2021

#130,"'n benzyl 2 cyano 3 (3,4 dihydroxyphenyl)acrylamide'/exp OR '2 cyano 3 (3, 4 dihydroxyphenyl) n (phenylmethyl) 2 propenamide' OR 'ag 490' OR 'ag490' OR 'n benzyl 2 cyano 3 (3,4 dihydroxyphenyl)acrylamide' OR 'tyrphostin ag 490' OR 'tyrphostin ag490'",2477,13 Jan 2021
#129,"'itacitinib'/de OR '1 [1 [[3 fluoro 2 (trifluoromethyl) 4 pyridinyl] carbonyl] 4 piperidinyl] 3 [4 (7h pyrrolo [2, 3 d] pyrimidin 4 yl) 1h pyrazol 1 yl] 3 azetidineacetonitrile' OR '1 [1 [[3 fluoro 2 (trifluoromethyl) pyridin 4 yl] carbonyl] piperidin 4 yl] 3 [4 (7h pyrrolo [2, 3 d] pyrimidin 4 yl) 1h pyrazol 1 yl] 3 azetidineacetonitrile' OR '[1 [1 (3 fluoro 2 trifluoromethylpyridine 4 carbonyl) 4 piperidinyl] 3 [4 (7h pyrrolo [2, 3 d] pyrimidin 4 yl) 1h pyrazol 1 yl] 3 azetidinyl] ethanenitrile' OR '[1 [1 [3 fluoro 2 (trifluoromethyl) pyridine 4 carbonyl] piperidin 4 yl] 3 [4 (7h pyrrolo [2, 3 d] pyrimidin 4 yl) 1h pyrazol 1 yl] azetidin 3 yl] ethanenitrile' OR 'incb 039110' OR 'incb 39110' OR 'incb039110' OR 'incb39110' OR 'itacitinib' OR 'itacitinib adipate' OR 'itacitinib hexanedioate'",183,13 Jan 2021
#128,"'ruxolitinib'/de OR '3 [4 (7h pyrrolo [2, 3 d] pyrimidin 4 yl) 1h pyrazol 1 yl] 3 cyclopentylpropanenitrile' OR '3 [4 (7h pyrrolo [2, 3 d] pyrimidin 4 yl) 1h pyrazol 1 yl] 3 cyclopentylpropanenitrile phosphate' OR '3 cyclopentyl 3 [4 (7h pyrrolo [2, 3 d] pyrimidin 4 yl) 1h pyrazol 1 yl] propanenitrile' OR 'beta cyclopentyl 4 (7h pyrrolo [2, 3 d] pyrimidin 4 yl) 1h pyrazole 1 propanenitrile' OR 'incb 018424' OR 'incb 18424' OR 'incb 424' OR 'incb018424' OR 'incb18424' OR 'incb424' OR 'jakafi' OR 'jakavi' OR 'ruxolitinib' OR 'ruxolitinib maleate' OR 'ruxolitinib phosphate'",5334,13 Jan 2021
#127,"'upadacitinib'/de OR '3 ethyl 4 (3h imidazo [1, 2 a] pyrrolo [2, 3 e] pyrazin 8 yl) n (2, 2, 2 trifluoroethyl) 1 pyrrolidinecarboxamide' OR '3 ethyl 4 (3h imidazo [1, 2 a] pyrrolo [2, 3 e] pyrazin 8 yl) n (2, 2, 2 trifluoroethyl) 1 pyrrolidinecarboxamide 2, 3 dihydroxybutanedioate' OR '3 ethyl 4 (3h imidazo [1, 2 a] pyrrolo [2, 3 e] pyrazin 8 yl) n (2, 2, 2 trifluoroethyl) 1 pyrrolidinecarboxamide tartrate' OR '3

ethyl 4 (3h imidazo [1, 2 a] pyrrolo [2, 3 e] pyrazin 8 yl) n (2, 2, 2 trifluoroethyl) pyrrolidine 1 carboxamide' OR '3 ethyl 4 (3h imidazo [1, 2 a] pyrrolo [2, 3 e] pyrazin 8 yl) n (2, 2, 2 trifluoroethyl) pyrrolidine 1 carboxamide 2, 3 dihydroxybutanedioate' OR '3 ethyl 4 (3h imidazo [1, 2 a] pyrrolo [2, 3 e] pyrazin 8 yl) n (2, 2, 2 trifluoroethyl) pyrrolidine 1 carboxamide tartrate' OR 'abt 494' OR 'abt494' OR 'rinvoq' OR 'upadacitinib' OR 'upadacitinib 2, 3 dihydroxybutanedioate' OR 'upadacitinib hemihydrate' OR 'upadacitinib hydrate' OR 'upadacitinib tartrate'",547,13 Jan 2021

#126,"'filgotinib'/de OR 'filgotinib' OR 'filgotinib 2 butenedioate' OR 'filgotinib hydrochloride' OR 'filgotinib maleate' OR 'g 146034' OR 'g 146034 101' OR 'g 146034-101' OR 'g146034' OR 'g146034 101' OR 'g146034-101' OR 'glpg 0634' OR 'glpg0634' OR 'gs 6034' OR 'gs6034' OR 'n [5 [4 (1, 1 dioxothiomorpholinomethyl) phenyl] 1, 2, 4 triazolo [1, 5 a] pyridin 2 yl] cyclopropanecarboxamide' OR 'n [5 [4 (1, 1 dioxothiomorpholinomethyl) phenyl] 1, 2, 4 triazolo [1, 5 a] pyridin 2 yl] cyclopropanecarboxamide 2 butenedioate' OR 'n [5 [4 (1, 1 dioxothiomorpholinomethyl) phenyl] 1, 2, 4 triazolo [1, 5 a] pyridin 2 yl] cyclopropanecarboxamide but 2 enedioate' OR 'n [5 [4 [(1, 1 dioxido 4 thiomorpholinyl) methyl] phenyl] 1, 2, 4 triazolo [1, 5 a] pyridin 2 yl] cyclopropanecarboxamide' OR 'n [5 [4 [(1, 1 dioxido 4 thiomorpholinyl) methyl] phenyl] 1, 2, 4 triazolo [1, 5 a] pyridin 2 yl] cyclopropanecarboxamide 2 butenedioate' OR 'n [5 [4 [(1, 1 dioxothiomorpholin 4 yl) methyl] phenyl] 1, 2, 4 triazolo [1, 5 a] pyridin 2 yl] cyclopropanecarboxamide' OR 'n [5 [4 [(1, 1 dioxothiomorpholin 4 yl) methyl] phenyl] 1, 2, 4 triazolo [1, 5 a] pyridin 2 yl] cyclopropanecarboxamide but 2 enedioate' OR 'n [5 [4 [(1, 1 dioxothiomorpholin 4 yl) methyl] phenyl] [1, 2, 4] triazolo [1, 5 a] pyridin 2 yl] cyclopropanecarboxamide' OR 'n [5 [4 [(1, 1 dioxothiomorpholin 4 yl) methyl] phenyl] [1, 2, 4] triazolo [1, 5 a] pyridin 2 yl] cyclopropanecarboxamide but 2 enedioate'",504,13 Jan 2021

#125,"'tofacitinib'/de OR '1 cyanoacetyl 4 methyl n methyl n (1h pyrrolo [2, 3 d] pyrimidin 4 yl) 3 piperidinamine' OR '3 [4 methyl 3 [methyl (7h pyrrolo [2, 3 d] pyrimidin 4 yl) amino] 1 piperidinyl] 3 oxopropanenitrile' OR '4 [n [1 (2 cyano 1 oxoethyl) 4 methyl 3 piperidinyl] n methylamino] pyrrolo [2, 3 d] pyrimidine' OR '4 methyl 3 [methyl (7h pyrrolo [2, 3 d] pyrimidin 4 yl) amino] beta oxo 1 piperidinepropanenitrile' OR 'cp 690 550' OR 'cp 690, 550' OR 'cp 690550' OR 'cp 690550 10' OR 'cp 690550-10' OR 'cp690 550' OR 'cp690, 550' OR 'cp690550' OR 'cp690550 10' OR 'cp690550-10' OR 'tasocitinib' OR 'tasocitinib citrate' OR 'tofacitinib' OR 'tofacitinib citrate' OR 'xeljanz' OR 'xeljanz xr'",5247,13 Jan 2021

#124,"'baricitinib'/de OR '1 (ethylsulfonyl) 3 [4 (7h pyrrolo [2, 3 d] pyrimidin 4 yl) 1h pyrazol 1 yl] 3 azetidineacetonitrile' OR '[1 (ethanesulfonyl) 3 [4 (7h pyrrolo [2, 3 d] pyrimidin 4 yl) 1h pyrazol 1 yl] azetidin 3 yl] ethanenitrile' OR '[1 (ethylsulfonyl) 3 [4 (1h pyrrolo [2, 3 d] pyrimidin 4 yl) 1h pyrazol 1 yl] 3 azetidinyl] acetonitrile' OR '[1 (ethylsulfonyl) 3 [4 (7h pyrrolo [2, 3 d] pyrimidin 4 yl) 1h pyrazol 1 yl] azetidin 3 yl] ethanenitrile' OR 'baricitinib' OR 'incb 028050' OR 'incb 28050' OR 'incb028050' OR 'incb28050' OR 'ly 3009104' OR 'ly3009104' OR 'olumiant'",1531,13 Jan 2021

#123,"(janus NEAR/4 kinase NEAR/10 inhibitor$):tt",4,13 Jan 2021

#122,"(janus NEAR/4 kinase NEAR/10 inhibitor$):ti,ab,kw,tn",3313,13 Jan 2021

#121,"((jak OR jak1 OR jak2 OR jak3 OR 'janus kinase') NEAR/10 inhibitor*):tt",21,13 Jan 2021

#120,"((jak OR jak1 OR jak2 OR jak3 OR 'janus kinase') NEAR/10 inhibitor*):ti,ab,kw,tn",11233,13 Jan 2021

#119,"'janus kinase inhibitor'/exp OR 'jak inhibitor' OR 'janus kinase inhibitor' OR 'janus kinase inhibitors' OR 'janus tyrosine kinase inhibitor'",16150,13 Jan 2021

#118,"(steroid$ NEAR/10 (therap* OR treat* OR drug$ OR inject* OR intramuscular* OR 'intra muscular*' OR im OR intravenous* OR 'intra venous*' OR iv OR intraarticular* OR 'intra articular*' OR ia OR administrat* OR oral OR orally OR parenteral*)):tt",147,13 Jan 2021
#117,"(steroid$ NEAR/10 (therap* OR treat* OR drug$ OR inject* OR intramuscular* OR 'intra muscular*' OR im OR intravenous* OR 'intra venous*' OR iv OR intraarticular* OR 'intra articular*' OR ia OR administrat* OR oral OR orally OR parenteral*)):ti,ab,kw",118085,13 Jan 2021
#116,"'triamcinolone'/exp OR '9alpha fluoro 1, 4 pregnadiene 3, 20 dione 11beta, 16alpha, 17alpha, 21 tetrol' OR '9alpha fluoro 11beta, 16alpha, 17alpha, 21 tetrahydroxypregna 1, 4 diene 3, 20 dione' OR '9alpha fluoro 16alpha hydroxyhydrocortisone' OR '9alpha fluoro 16alpha hydroxyprednisolone' OR 'acetocot' OR 'adcortyl' OR 'aristocort' OR 'aristocort forte' OR 'aristodan' OR 'azmacor' OR 'celeste' OR 'cl 19823' OR 'cl19823' OR 'clinacort' OR 'clinalog' OR 'delphicort' OR 'fluoxiprednisolone' OR 'fluoxyprednisolone' OR 'ken-jec 40' OR 'kenacort' OR 'kenacort retard' OR 'korticoid' OR 'ledercort' OR 'omcilon' OR 'polcortolon' OR 'rp 8357' OR 'rp8357' OR 'simacort' OR 'sterocort' OR 'tac 3' OR 'tramcinolone' OR 'triacortyl' OR 'triam-a' OR 'triam-forte' OR 'triamcinolon' OR 'triamcinolona' OR 'triamcinolone*' OR 'triamcort' OR 'triamcot' OR 'triamonide 40' OR 'triamsicort' OR 'triancinolon' OR 'u-tri-lone' OR 'volon'",39561,13 Jan 2021
#115,"'beclometasone'/exp OR '9 chloro 11beta, 17, 21 trihydroxy 16beta methylpregna 1, 4 diene 3, 20 dione' OR '9alpha chloro 16beta methylprednisolone' OR 'beclometasone' OR 'beclomethasone' OR 'prednisolone, 9alpha chloro 16beta methyl' OR 'pregna 1, 4 diene 11beta, 17, 21 triol 3, 20 dione, 9 chloro 16beta methyl'",15089,13 Jan 2021
#114,"'dexamethasone'/de OR '16alpha methyl 9alpha fluoroprednisolone' OR '9 alpha fluoro 16 alpha methyl delta corticosterone' OR '9alpha fluoro 11beta, 17alpha, 21 trihydroxy 16alpha methyl 1, 4

pregnadiene 3, 20 dione' OR '9alpha fluoro 11beta, 17alpha, 21 trihydroxy 16alpha methylpregna 1, 4 diene 3, 20 dione' OR '9alpha fluoro 16alpha methyl delta corticosterone' OR 'adrecort' OR 'adrenocot' OR 'aeroseb dex' OR 'aeroseb-dex' OR 'aflucoson' OR 'aflucosone' OR 'alfalyl' OR 'anaflogistico' OR 'anaflogistico novobios' OR 'arcodexan' OR 'arcodexane' OR 'artrosone' OR 'azium' OR 'bidexol' OR 'calonat' OR 'cebedex' OR 'cetadexon' OR 'colofoam' OR 'corsona' OR 'cortastat' OR 'cortastat 10' OR 'cortastat la' OR 'cortidex' OR 'cortidexason' OR 'cortidrona' OR 'cortidrone' OR 'cortisumman' OR 'dacortina fuerte' OR 'dacortine fuerte' OR 'dalalone' OR 'dalalone d.p.' OR 'dalalone l.a.' OR 'danasone' OR 'de-sone la' OR 'decacortin' OR 'decadeltosona' OR 'decadeltosone' OR 'decaderm' OR 'decadion' OR 'decadran' OR 'decadron' OR 'decadron 5-12 pak' OR 'decadron la' OR 'decadronal' OR 'decadrone' OR 'decaesadril' OR 'decaject' OR 'decamethasone' OR 'decasone' OR 'decaspray' OR 'decasterolone' OR 'decdan' OR 'decilone' OR 'decilone forte' OR 'decofluor' OR 'dectancyl' OR 'dekacort' OR 'delladec' OR 'deltafluoren' OR 'deltafluorene' OR 'dergramin' OR 'deronil' OR 'desacort' OR 'desacortone' OR 'desadrene' OR 'desalark' OR 'desameton' OR 'desametone' OR 'desigdron' OR 'dexa cortisyl' OR 'dexa dabrosan' OR 'dexa korti' OR 'dexa scherosan' OR 'dexa scherozon' OR 'dexa scherozone' OR 'dexa-p' OR 'dexacen 4' OR 'dexacen-4' OR 'dexachel' OR 'dexacort' OR 'dexacortal' OR 'dexacorten' OR 'dexacortin' OR 'dexacortisyl' OR 'dexadabroson' OR 'dexadecadrol' OR 'dexadrol' OR 'dexagel' OR 'dexagen' OR 'dexahelvacort' OR 'dexakorti' OR 'dexalien' OR 'dexalocal' OR 'dexame' OR 'dexamecortin' OR 'dexameson' OR 'dexamesone' OR 'dexametason' OR 'dexametasone' OR 'dexameth' OR 'dexamethason' OR 'dexamethasone' OR 'dexamethasone alcohol' OR 'dexamethasone intensol' OR 'dexamethazon' OR 'dexamethazone' OR 'dexamethonium' OR 'dexamonozon' OR 'dexan' OR 'dexane' OR 'dexano' OR 'dexapot' OR 'dexascheroson' OR 'dexascherozon' OR 'dexascherozone' OR 'dexason' OR 'dexasone' OR 'dexasone la' OR 'dexasone s' OR 'dexinoral' OR 'dexionil' OR 'dexmethsone' OR

'dexona' OR 'dexone' OR 'dexone 0.5' OR 'dexone 0.75' OR 'dexone 1.5' OR 'dexone 4' OR 'dexpak taperpak' OR 'dextelan' OR 'dextenza' OR 'dextrasone' OR 'dexycu' OR 'dezone' OR 'dibasona' OR 'doxamethasone' OR 'esacortene' OR 'ex s1' OR 'exadion' OR 'exadione' OR 'firmalone' OR 'fluormethyl prednisolone' OR 'fluormethylprednisolon' OR 'fluormethylprednisolone' OR 'fluormone' OR 'fluorocort' OR 'fluorodelta' OR 'fluoromethylprednisolone' OR 'fortecortin' OR 'gammacorten' OR 'gammacortene' OR 'grosodexon' OR 'grosodexone' OR 'hemady' OR 'hexadecadiol' OR 'hexadecadrol' OR 'hexadiol' OR 'hexadrol' OR 'isnacort' OR 'isopto dex' OR 'isopto maxidex' OR 'isopto-dex' OR 'isopto-maxidex' OR 'isoptodex' OR 'isoptomaxidex' OR 'lokalison f' OR 'loverine' OR 'luxazone' OR 'marvidione' OR 'maxidex' OR 'mediamethasone' OR 'megacortin' OR 'mephameson' OR 'mephamesone' OR 'metasolon' OR 'metasolone' OR 'methazon ion' OR 'methazone ion' OR 'methazonion' OR 'methazonione' OR 'metisone lafi' OR 'mexasone' OR 'millicorten' OR 'millicortenol' OR 'mk 125' OR 'mk125' OR 'mymethasone' OR 'neoforderx' OR 'neofordex' OR 'nisomethasona' OR 'novocort' OR 'nsc 34521' OR 'nsc34521' OR 'oftan-dexa' OR 'opticorten' OR 'opticortinol' OR 'oradexan' OR 'oradexon' OR 'oradexone' OR 'orgadrone' OR 'ozurdex' OR 'pidexon' OR 'policort' OR 'posurdex' OR 'predni f tablinen' OR 'predni-f' OR 'prednisolone f' OR 'prodexona' OR 'prodexone' OR 'sanamethasone' OR 'santenson' OR 'santeson' OR 'sawasone' OR 'solurex' OR 'solurex la' OR 'spoloven' OR 'sterasone' OR 'thilodexine' OR 'triamcimetil' OR 'vexamet' OR 'visumetazone' OR 'visumethazone'",171013,13 Jan 2021
#113,"'hydrocortisone'/de OR '11beta, 17, 21 trihydroxypregn 4 ene 3, 20 dione' OR '11beta, 17alpha, 21 trihydroxypregn 4 ene 3, 20 dione' OR '17 hydroxycorticosterone' OR '4 pregnene 11beta, 17alpha, 21 triol 3, 20 dione' OR '4 pregnene 3, 20 dione n beta, 17alpha, 21 triol' OR 'acticort' OR 'acticort 100' OR 'aeroseb hc' OR 'aeroseb-hc' OR 'ala-cort' OR 'ala-scalp' OR 'ala-scalp hp' OR 'alfacort' OR 'algicortis' OR 'alkindi' OR 'alpha derm' OR 'alphaderm' OR 'anucort-hc' OR 'anumed-hc' OR 'anutone-hc' OR

'aquanil hc' OR 'balneol-hc' OR 'barseb hc' OR 'beta-hc' OR 'biacort' OR 'cetacort' OR 'cobadex' OR 'colocort' OR 'compound f' OR 'cordicare lotion' OR 'coripen' OR 'cort dome' OR 'cort-dome' OR 'cort-dome high potency' OR 'cortef' OR 'cortef cream' OR 'cortenema' OR 'cortibel' OR 'corticorenol' OR 'cortifan' OR 'cortiphate' OR 'cortisol' OR 'cortisole' OR 'cortispray' OR 'cortoderm' OR 'cortril' OR 'cotacort' OR 'covocort' OR 'cremicort-h' OR 'cutaderm' OR 'derm-aid cream' OR 'dermacrin hc lotion' OR 'dermaid' OR 'dermaid soft cream' OR 'dermocare' OR 'dermocortal' OR 'dermolate' OR 'dioderm' OR 'eczacort' OR 'ef cortelan' OR 'efcortelan' OR 'egocort' OR 'egocort cream' OR 'eksalb' OR 'eldecort' OR 'emo-cort' OR 'epicort' OR 'ficortril' OR 'filocot' OR 'flexicort' OR 'gly-cort' OR 'glycort' OR 'h-cort' OR 'hc (hydrocortisone)' OR 'hc no. 1' OR 'hc no. 4' OR 'hebcort' OR 'hebcort v' OR 'hemorrhoidal hc' OR 'hemril-30' OR 'hemril-hc uniserts' OR 'hi-cor' OR 'hidrotisona' OR 'hycor' OR 'hycort' OR 'hydracort' OR 'hydrasson' OR 'hydro ricortex' OR 'hydro-rx' OR 'hydrocort' OR 'hydrocorticosteroid' OR 'hydrocortisate' OR 'hydrocortison' OR 'hydrocortisone' OR 'hydrocortisone 1% in absorbase' OR 'hydrocortisone acetonide' OR 'hydrocortisone astier' OR 'hydrocortisone in absorbase' OR 'hydrocortisone ointment' OR 'hydrocortisone plus saline' OR 'hydrocortisone steroid' OR 'hydrocortisone, topical' OR 'hydrocortisonum' OR 'hydrocortisyl' OR 'hydrocortone' OR 'hydrogalen' OR 'hydrokort' OR 'hydrokortison' OR 'hydrotopic' OR 'hysone' OR 'hytisone' OR 'hytone' OR 'hytone lotion' OR 'incortin h' OR 'instacort 10' OR 'kyypakkaus' OR 'lacticare hc' OR 'lacticare-hc' OR 'lemnis fatty cream hc' OR 'lenirit' OR 'medihaler cort' OR 'medihaler duo' OR 'medrocil' OR 'mildison' OR 'mildison fet krem' OR 'mildison lipocream' OR 'mildison-fatty' OR 'mitocortyl demangeaisons' OR 'munitren' OR 'nogenic hc' OR 'novohydrocort' OR 'nsc 10483' OR 'nsc 741' OR 'nsc10483' OR 'nutracort' OR 'optef' OR 'otosone f' OR 'penecort' OR 'plenadren' OR 'prepcort' OR 'prevex hc' OR 'pro cort' OR 'procort' OR 'procto-kit 1%' OR 'procto-kit 2.5%' OR 'proctocort' OR 'proctosert hc' OR

'proctosol-hc' OR 'proctosone' OR 'proctozone hc' OR 'procutan' OR 'rectasol-hc' OR 'rectocort' OR 'rederm' OR 'sanatison' OR 'scalp-aid' OR 'schericur' OR 'schericur 0.25%' OR 'scherosone f' OR 'sistral hydrocort' OR 'skincalm' OR 'stie-cort' OR 'substance m' OR 'synacort' OR 'texacort' OR 'triburon-hc' OR 'unicort' OR 'vasocort'",165746,13 Jan 2021

#112,"'corticosterone'/de OR '11 beta, 21 dihydroxy pregn 4 ene 3, 20 dione' OR '11beta, 21 dihydroxy 4 pregnene 3, 20 dione' OR '11beta, 21 dihydroxypregn 4 ene 3, 20 dione' OR '4 pregnene 11beta, 21 diol 3, 20 dione' OR 'compound b' OR 'corticosterone*' OR 'corticosterone function' OR 'corticosterone response' OR 'kendall compound b' OR 'nsc 9705' OR 'nsc9705' OR 'pregn 4 ene 11beta, 21 diol 3, 20 dione' OR 'reichstein substance h'",45684,13 Jan 2021

#111,"'glucocorticoid'/exp OR 'glucocorticoid*' OR 'glucocorticoid drug' OR 'glucocorticoid hormone' OR 'glucocorticoid steroid' OR 'glucocorticoids' OR 'glucocorticoids, synthetic' OR 'glucocorticoids, topical' OR 'glucocorticoidsteroid' OR 'glucocorticosteroid' OR 'glucocortoid' OR 'glycocorticoid' OR 'glycocorticosteroid'",796659,13 Jan 2021

#110,"'methylprednisolone'/de OR '11beta, 17alpha, 21 trihydroxy 6alpha methyl 1, 4 pregnadiene 3, 20 dione' OR '2 methylprednisolone' OR '5 methylprednisolone' OR '6 alpha methylprednisolone' OR '6 methyl delta 1 hydrocortisone' OR '6 methyl prednisolone' OR '6 methylprednisolone' OR '6alpha methyl delta1 hydrocortisone' OR '6alpha methylprednisolone' OR 'adlone-40' OR 'adlone-80' OR 'beta methylprednisolone' OR 'dep medalone 80' OR 'depmedalone' OR 'depoject-80' OR 'depopred' OR 'esametone' OR 'firmacort' OR 'med-jec-40' OR 'medixon' OR 'mednin' OR 'medralone 80' OR 'medrate' OR 'medrol' OR 'medrol a' OR 'medrol adt pak' OR 'medrol compositum' OR 'medrol dosepak' OR 'medrol medules' OR 'medrol pak' OR 'medrone' OR 'meprednisolone' OR 'meprelon' OR 'mesopren' OR 'methacort 40' OR 'methacort 80' OR 'methyl prednisolone' OR 'methylcotol' OR 'methylcotolone' OR

'methylpred dp' OR 'methylprednisolone*' OR 'methylsterolone' OR 'metidrol' OR 'metrisone' OR 'metycortin' OR 'metypred' OR 'metypresol' OR 'neomedrone' OR 'nsc 19987' OR 'nsc19987' OR 'prednol' OR 'solomet' OR 'solu decortin' OR 'urbason' OR '83 43 2':rn",109147,13 Jan 2021

#109,"'cortisone'/de OR '11 dehydro 17 hydroxy corticosterone' OR '11 dehydro 17 hydroxycorticosterone' OR '17 hydroxy 11 dehydrocortisone' OR '17alpha, 21 dihydroxy 4 pregnene 3, 11, 20 trione' OR '4 pregnene 17alpha, 21 diol 3, 11, 20 trione' OR 'adrenalex' OR 'compound e (kendall)' OR 'corlin' OR 'cortadren' OR 'cortagen' OR 'cortandren' OR 'cortane' OR 'cortisal' OR 'cortisate' OR 'cortison' OR 'cortisone$' OR 'cortisone sodium' OR 'cortistal' OR 'cortivite' OR 'cortogen' OR 'cortone' OR 'delta4 pregnene 17alpha, 21 diol 3, 11, 20 trione' OR 'incorlin' OR 'incortin' OR 'kendall compound e' OR 'nsc 9703' OR 'nsc9703' OR 'pregn 4 en 17 alpha, 21 diol 3, 11, 20 trione' OR 'pregn 4 ene 17alpha, 21 diol 3, 11, 20 trione' OR 'reichstein substance fa' OR 'ricortex' OR 'scheroson' OR 'scherosone' OR 'wintersteiner compound f'",32700,13 Jan 2021

#108,"'cortisone therapeutic use'/de",3404,13 Jan 2021

#107,"'prednisone'/de OR '1, 2 dehydrocortisone' OR '17, 21 dihydroxypregna 1, 4 diene 3, 11, 20 trione' OR 'ancortone' OR 'apo-prednisone' OR 'biocortone' OR 'colisone' OR 'cortan' OR 'cortidelt' OR 'cortiprex' OR 'cutason' OR 'dacorten' OR 'de cortisyl' OR 'decortancyl' OR 'decortin' OR 'decortin e merck' OR 'decortine' OR 'decortisyl' OR 'dehydrocortisone' OR 'dekortin' OR 'delitisone' OR 'dellacort a' OR 'delta 1 dehydrocortisone' OR 'delta cortelan' OR 'delta cortisone' OR 'delta dome' OR 'delta e' OR 'delta prenovis' OR 'delta-dome' OR 'deltacorten' OR 'deltacortene' OR 'deltacortisone' OR 'deltacortone' OR 'deltasone' OR 'deltison' OR 'deltisona' OR 'deltra' OR 'di adreson' OR 'di-adreson' OR 'diadreson' OR 'drazone' OR 'encorton' OR 'encortone' OR 'enkorton' OR 'fernisone' OR 'hostacortin' OR 'insone' OR 'liquid pred' OR 'lodotra' OR 'me-korti' OR 'meprison' OR 'metacortandracin' OR

'meticorten' OR 'meticortine' OR 'nisona' OR 'nsc 10023' OR 'nsc10023' OR 'orasone' OR 'orisane' OR 'panafcort' OR 'paracort' OR 'pehacort' OR 'precort' OR 'precortal' OR 'prednicen-m' OR 'prednicorm' OR 'prednicot' OR 'prednidib' OR 'prednison' OR 'prednisone*' OR 'prednisone alcohol' OR 'prednisone intensol' OR 'prednisone test' OR 'prednitone' OR 'pregna 1, 4 diene 3, 11, 20 trione 17, 21 diol' OR 'pronison' OR 'pronisone' OR 'pronizone' OR 'pulmison' OR 'rayos' OR 'rectodelt' OR 'servisone' OR 'steerometz' OR 'sterapred' OR 'sterapred ds' OR 'ultracorten' OR 'urtilone' OR 'winpred'",187180,13 Jan 2021

#106,"'prednisolone'/de OR '1, 2 dehydro hydrocortison' OR '1, 4 pregnadien 11beta, 17alpha, 21 triol 3, 20 dion' OR '1, 4 pregnadiene 11beta, 17alpha, 21 triol 3, 20 dione' OR '1, 4 pregnadiene 3, 20 dione 11beta, 17alpha, 21 triol' OR '11beta, 17, 21 trihydroxypregna 1, 4 diene 3, 20 dione' OR '11beta, 17alpha, 21 trihydroxy 1, 4 pregnadien 3, 20 dion' OR '11beta, 17alpha, 21 trihydroxypregna 1, 4 diene 3, 20 dione' OR '3, 20 dioxo 11beta, 17alpha, 21 trihydroxy 1, 4 pregnadiene' OR 'adelcort' OR 'antisolon' OR 'antisolone' OR 'aprednislon' OR 'aprednislone' OR 'benisolon' OR 'benisolone' OR 'berisolon' OR 'berisolone' OR 'caberdelta' OR 'capsoid' OR 'co hydeltra' OR 'codelcortone' OR 'compresolon' OR 'cortadeltona' OR 'cortadeltone' OR 'cortalone' OR 'cortelinter' OR 'cortisolone' OR 'cotolone' OR 'dacortin' OR 'dacortin h' OR 'dacrotin' OR 'decaprednil' OR 'decortin h' OR 'decortril' OR 'dehydro cortex' OR 'dehydro hydrocortison' OR 'dehydro hydrocortisone' OR 'dehydrocortex' OR 'dehydrocortisol' OR 'dehydrocortisole' OR 'dehydrohydrocortison' OR 'dehydrohydrocortisone' OR 'delcortol' OR 'delta 1 17 hydroxycorticosterone 21 acetate' OR 'delta 1 hydrocortisone' OR 'delta cortef' OR 'delta cortril' OR 'delta ef cortelan' OR 'delta f' OR 'delta hycortol' OR 'delta hydrocortison' OR 'delta hydrocortisone' OR 'delta ophticor' OR 'delta stab' OR 'delta-cortef' OR 'delta1 dehydrocortisol' OR 'delta1 dehydrohydrocortisone' OR 'delta1 hydrocortisone' OR 'deltacortef' OR

'deltacortenolo' OR 'deltacortil' OR 'deltacortoil' OR 'deltacortril' OR 'deltaderm' OR 'deltaglycortril' OR 'deltahycortol' OR 'deltahydrocortison' OR 'deltahydrocortisone' OR 'deltaophticor' OR 'deltasolone' OR 'deltastab' OR 'deltidrosol' OR 'deltisilone' OR 'deltisolon' OR 'deltisolone' OR 'deltolasson' OR 'deltolassone' OR 'deltosona' OR 'deltosone' OR 'depo-predate' OR 'dermosolon' OR 'dhasolone' OR 'di adreson f' OR 'di adresone f' OR 'di-adreson-f' OR 'diadreson f' OR 'diadresone f' OR 'dicortol' OR 'domucortone' OR 'encortelon' OR 'encortelone' OR 'encortolon' OR 'equisolon' OR 'fernisolone-p' OR 'glistelone' OR 'hefasolon' OR 'hostacortin h' OR 'hostacortin h vet' OR 'hydeltra' OR 'hydeltrone' OR 'hydrelta' OR 'hydrocortancyl' OR 'hydrocortidelt' OR 'hydrodeltalone' OR 'hydrodeltisone' OR 'hydroretrocortin' OR 'hydroretrocortine' OR 'inflanefran' OR 'insolone' OR 'keteocort h' OR 'key-pred' OR 'key-pred sp' OR 'lenisolone' OR 'leocortol' OR 'liquipred' OR 'lygal kopftinktur n' OR 'mediasolone' OR 'meprisolon' OR 'meprisolone' OR 'metacortalon' OR 'metacortalone' OR 'metacortandralon' OR 'metacortandralone' OR 'metacortelone' OR 'meti derm' OR 'meti-derm' OR 'meticortelone' OR 'metiderm' OR 'morlone' OR 'mydrapred' OR 'neo delta' OR 'nisolon' OR 'nisolone' OR 'nsc 9120' OR 'nsc9120' OR 'opredsone' OR 'panafcortelone' OR 'panafcortolone' OR 'panafort' OR 'paracortol' OR 'phlogex' OR 'pre cortisyl' OR 'preconin' OR 'precortalon' OR 'precortancyl' OR 'precortisyl' OR 'pred-ject-50' OR 'predacort 50' OR 'predaject-50' OR 'predalone 50' OR 'predartrina' OR 'predartrine' OR 'predate-50' OR 'predeltilone' OR 'predisole' OR 'predisyr' OR 'predne dome' OR 'prednecort' OR 'prednedome' OR 'prednelan' OR 'predni coelin' OR 'predni h tablinen' OR 'predni-helvacort' OR 'prednicoelin' OR 'prednicort' OR 'prednicortelone' OR 'prednifor drops' OR 'predniment' OR 'predniretard' OR 'prednis' OR 'prednisil' OR 'prednisolon' OR 'prednisolona' OR 'prednisolone*' OR 'prednisolone alcohol' OR 'prednisolone h' OR 'prednisolone oleosae sr 82' OR 'prednisolone, topical' OR 'prednivet' OR 'prednorsolon' OR 'prednorsolone' OR 'predonine' OR 'predorgasolona' OR

'predorgasolone' OR 'pregna 1, 4 diene 11beta, 17alpha, 21 triol 3, 20 dione' OR 'prelon' OR 'prelone' OR 'prenilone' OR 'prenin' OR 'prenolone' OR 'preventan' OR 'prezolon' OR 'rubycort' OR 'scherisolon' OR 'scherisolona' OR 'serilone' OR 'solondo' OR 'solone' OR 'solupren' OR 'soluprene' OR 'spiricort' OR 'spolotane' OR 'sterane' OR 'sterolone' OR 'supercortisol' OR 'supercortizol' OR 'taracortelone' OR 'walesolone' OR 'wysolone'",147306,13 Jan 2021

#105,"'corticosteroid'/exp OR 'adrenal cortex hormone' OR 'adrenal cortex hormones' OR 'adrenal cortical hormone' OR 'adrenal cortical hormones' OR 'adrenal cortical steroid' OR 'adrenal steroid' OR 'adrenal steroid hormone' OR 'adreno cortical steroid' OR 'adreno corticosteroid' OR 'adrenocortical hormone' OR 'adrenocortical steroid' OR 'adrenocorticosteroid' OR 'cortical steroid' OR 'cortico steroid' OR 'corticoid*' OR 'corticosteroid*' OR 'corticosteroid agent' OR 'corticosteroid calcium' OR 'corticosteroid hormone' OR 'corticosteroids' OR 'corticosteroids, inhalation' OR 'corticosteroids, ophthalmic' OR 'corticosteroids, otic' OR 'corticosteroids, systemic' OR 'corticosteroids, topical' OR 'dermocorticosteroid' OR 'fluorinated corticosteroid'",1039304,13 Jan 2021

#104,"chloroquin*:ti,ab,kw,rn,tn OR cq:ti,ab,kw,rn,tn OR '54-04-7':ti,ab,kw,rn,tn OR 886u3h6uff*:ti,ab,kw,rn,tn OR arequin:ti,ab,kw,rn,tn OR chingamin:ti,ab,kw,rn,tn OR chlorochin*:ti,ab,kw,rn,tn OR khingamin:ti,ab,kw,rn,tn OR oe48649kh6n*:ti,ab,kw,rn,tn OR anaclor:ti,ab,kw,rn,tn OR bemaco:ti,ab,kw,rn,tn OR benaquin:ti,ab,kw,rn,tn OR 'brn 0482809':ti,ab,kw,rn,tn OR brn0482809:ti,ab,kw,rn,tn OR capquin:ti,ab,kw,rn,tn OR 'ccris 3439':ti,ab,kw,rn,tn OR ccris3439:ti,ab,kw,rn,tn OR chloraquin*:ti,ab,kw,rn,tn OR cindacin:ti,ab,kw,rn,tn OR cindachin:ti,ab,kw,rn,tn OR cloroquina:ti,ab,kw,rn,tn OR cocatrit:ti,ab,kw,rn,tn OR 'einecs 200-191-2':ti,ab,kw,rn,tn OR 'einecs200 191 2':ti,ab,kw,rn,tn OR elestol:ti,ab,kw,rn,tn OR 'hsdb 3029':ti,ab,kw,rn,tn OR hsdb3029:ti,ab,kw,rn,tn OR

lapaquin:ti,ab,kw,rn,tn OR malaren:ti,ab,kw,rn,tn OR neochin:ti,ab,kw,rn,tn OR 'nsc 187208':ti,ab,kw,rn,tn OR nsc187208:ti,ab,kw,rn,tn OR pfizerquine:ti,ab,kw,rn,tn OR quensyl:ti,ab,kw,rn,tn OR quinercyl:ti,ab,kw,rn,tn OR quinilon:ti,ab,kw,rn,tn OR quinoline:ti,ab,kw,rn,tn OR quinoscan:ti,ab,kw,rn,tn OR ronaquine:ti,ab,kw,rn,tn OR sopaquin:ti,ab,kw,rn,tn OR 'st 21':ti,ab,kw,rn,tn OR weimerquin:ti,ab,kw,rn,tn",36677,13 Jan 2021

#103,"'chloroquine'/de OR '4 (4 diethylamino 1 methylbutylamino) 7 chlorchinolin diphosphate' OR '4 (4 diethylamino 1 methylbutylamino) 7 chlorchinolin sulfate' OR '4 (4 diethylamino 1 methylbutylamino) 7 chlorchinolin sulphate' OR '4 (4 diethylamino 1 methylbutylamino) 7 chloroquinoline' OR '7 chloro 4 (4 diethylamino 1 methylbutylamino) quinoline' OR '7 chloro 4 (4 diethylamino 1 methylbutylamino) quinoline diphosphate' OR 'a-cq' OR 'amokin' OR 'amokine' OR 'anoclor' OR 'aralan' OR 'aralen' OR 'aralen hydrochloride' OR 'aralen phosphate' OR 'aralene' OR 'arechin' OR 'arechine' OR 'arequine' OR 'arthrochin' OR 'arthrochine' OR 'arthroquine' OR 'artrichin' OR 'artrichine' OR 'artriquine' OR 'avloclor' OR 'avoclor' OR 'bemaphata' OR 'bemaphate' OR 'bemasulph' OR 'bipiquin' OR 'cadiquin' OR 'chemochin' OR 'chemochine' OR 'chingamine' OR 'chingaminum' OR 'chloraquine' OR 'chlorochin' OR 'chlorochine' OR 'chlorofoz' OR 'chloroquin' OR 'chloroquin phosphate' OR 'chloroquine' OR 'chloroquine diphosphate' OR 'chloroquine disulfate' OR 'chloroquine disulphate' OR 'chloroquine hydrochloride' OR 'chloroquine phosphate' OR 'chloroquine streuli' OR 'chloroquine sulfate' OR 'chloroquine sulphate' OR 'chloroquinesulphate' OR 'chloroquini diphosphas' OR 'chloroquinum diphosphoricum' OR 'chlorquin' OR 'chlorquine' OR 'choloquine' OR 'choroquine sulfate' OR 'choroquine sulphate' OR 'cidanchin' OR 'clo-kit junior' OR 'clorichina' OR 'clorichine' OR 'cloriquine' OR 'clorochina' OR 'delagil' OR 'delagyl' OR 'dichinalex' OR 'diclokin' OR 'diquinalex' OR 'diroquine' OR 'emquin' OR 'genocin' OR 'gontochin' OR 'gontochine' OR 'gontoquine' OR 'heliopar' OR 'imagon' OR

'iroquine' OR 'klorokin' OR 'klorokine' OR 'klorokinfosfat' OR 'lagaquin' OR 'malaquin' OR 'malarex' OR 'malarivon' OR 'malaviron' OR 'maliaquine' OR 'maquine' OR 'mesylith' OR 'mexaquin' OR 'mirquin' OR 'nivachine' OR 'nivaquin' OR 'nivaquine' OR 'nivaquine (b)' OR 'nivaquine b' OR 'nivaquine dp' OR 'nivaquine forte' OR 'p roquine' OR 'quinachlor' OR 'quingamine' OR 'repal' OR 'resochen' OR 'resochene' OR 'resochin' OR 'resochin junior' OR 'resochina' OR 'resochine' OR 'resochinon' OR 'resoquina' OR 'resoquine' OR 'reumachlor' OR 'roquine' OR 'rp 3377' OR 'rp3377' OR 'sanoquin' OR 'sanoquine' OR 'silbesan' OR 'siragan' OR 'sirajan' OR 'sn 7618' OR 'sn7618' OR 'solprina' OR 'solprine' OR 'tresochin' OR 'tresochine' OR 'tresoquine' OR 'trochin' OR 'trochine' OR 'troquine' OR 'w 7618' OR 'w7618' OR 'win 244' OR 'win244'",44503,13 Jan 2021

#102,"'organogold compound'/exp OR 'organogold compound' OR 'organogold compounds' OR 'organogold derivative'",482,13 Jan 2021

#101,"aurothiopropanol*:ti,ab,kw,rn,tn OR allochrysine:ti,ab,kw,rn,tn OR allocrisine:ti,ab,kw,rn,tn OR aurotiopoe:ti,ab,kw,rn,tn OR aurotioprol:ti,ab,kw,rn,tn OR '27279-43-2':ti,ab,kw,rn,tn OR g7097j63e9*:ti,ab,kw,rn,tn OR 'sodium aurothiopropanolsulfonate':ti,ab,kw,rn,tn OR 'gold sodium thiopropanol sulfonate':ti,ab,kw,rn,tn OR 'sodium auromercaptopropanol sulfonate':ti,ab,kw,rn,tn",274,13 Jan 2021

#100,"'allochrysine'/de OR '1 auromercapto 2 propanol 3 sulfonate' OR '1 auromercapto 2 propanol 3 sulfonate sodium' OR '1 auromercepto 2 propanol 3 sulfonate' OR 'allochrysine' OR 'auromercapto propanol sulfonate sodium' OR 'auromercaptopropanolsulfonate sodium' OR 'aurothioisopropanolsulfonate sodium' OR 'aurothiopropanol sulfonate' OR 'aurothiopropanol sulfonate sodium' OR 'gold sodium thiopropanol sulfonate' OR 'sodium 1 auromercapto 2 propanol 3

sulfonate' OR 'sodium auromercapto 2 propanol 3 sulfonate' OR 'sodium auromercaptopropanol sulfonate' OR 'sodium aurothiopropanol sulfonate'",272,13 Jan 2021

#99,"((gold* OR auro*) NEAR/10 (inject* OR intramuscular* OR 'im' OR 'intra muscular*')):ti,ab,kw",3122,13 Jan 2021

#98,"(thioglucosoaurate*:ti,ab,kw,rn,tn OR 'gold thioglucose*':ti,ab,kw,rn,tn OR 'gold thio glucose*':ti,ab,kw,rn,tn OR 'thioglucose gold':ti,ab,kw,rn,tn OR aureotan:ti,ab,kw,rn,tn OR 'gold-50':ti,ab,kw,rn,tn OR gold50:ti,ab,kw,rn,tn OR 'b oleosum solganal':ti,ab,kw,rn,tn OR aurotan:ti,ab,kw,rn,tn OR authron:ti,ab,kw,rn,tn OR brenol:ti,ab,kw,rn,tn OR 'ccris 59':ti,ab,kw,rn,tn OR ccris59:ti,ab,kw,rn,tn OR 'einecs 235-365-7':ti,ab,kw,rn,tn OR 'einecs235 365 7':ti,ab,kw,rn,tn OR glysanol:ti,ab,kw,rn,tn) AND b:ti,ab,kw,rn,tn OR goldthioglucose*:ti,ab,kw,rn,tn OR goldtioglucose*:ti,ab,kw,rn,tn OR 'hsdb 7174':ti,ab,kw,rn,tn OR hsdb7174:ti,ab,kw,rn,tn OR oronol:ti,ab,kw,rn,tn OR romosol:ti,ab,kw,rn,tn OR 'skf 10056':ti,ab,kw,rn,tn OR skf10056:ti,ab,kw,rn,tn OR 2p2v9q0e78*:ti,ab,kw,rn,tn OR '12192-57-3':ti,ab,kw,rn,tn",1509,13 Jan 2021

#97,"'aurothioglucose sodium'/de",40,13 Jan 2021

#96,"'aurothioglucose'/de OR '(1 d glucosylthio) gold' OR '(1 glucosylthio) gold' OR 'anitur' OR 'aureotan' OR 'auro thioglucose*' OR 'auromyose*' OR 'aurothioglucose*' OR 'aurumin' OR 'aurumine' OR 'gold thio glucose' OR 'gold thioglucose' OR 'gold-50' OR 'goldthioglucose' OR 'goldtioglucose' OR 'oronol' OR 'solganal' OR 'solganal b' OR 'solganol'",1709,13 Jan 2021

#95,"aurolate*:ti,ab,kw,rn,tn OR 'gold disodium thiomalate*':ti,ab,kw,rn,tn OR 'gold thiomalic acid*':ti,ab,kw,rn,tn OR miocrisin:ti,ab,kw,rn,tn OR 'monogold disodium thiomalate*':ti,ab,kw,rn,tn OR myochrysin*:ti,ab,kw,rn,tn OR myocrisin*:ti,ab,kw,rn,tn OR myocrysin*:ti,ab,kw,rn,tn OR 'sodium aurothiomalate*':ti,ab,kw,rn,tn OR 'sodium gold thiomalate*':ti,ab,kw,rn,tn OR 'sodium thiomalate*

gold':ti,ab,kw,rn,tn OR 'sodium thiomalatoaurate*':ti,ab,kw,rn,tn OR taureodon:ti,ab,kw,rn,tn OR 'thiomalatoaurate sodium':ti,ab,kw,rn,tn OR '1244-57-4':ti,ab,kw,rn,tn OR '39377-38-3':ti,ab,kw,rn,tn OR '4846-27-9':ti,ab,kw,rn,tn OR '74916-57-7':ti,ab,kw,rn,tn OR e4768zy6gm*:ti,ab,kw,rn,tn OR 'aurothiomala-natrium':ti,ab,kw,rn,tn OR 'aurot$iomalato sodico':ti,ab,kw,rn,tn OR 'monogold* sodium salt':ti,ab,kw,rn,tn OR 'dinatrium 2-aurothio-succinat':ti,ab,kw,rn,tn OR 'einecs 235-479-7':ti,ab,kw,rn,tn OR 'einecs235 479 7':ti,ab,kw,rn,tn OR 'hsdb 7173':ti,ab,kw,rn,tn OR hsdb7173:ti,ab,kw,rn,tn OR kidon:ti,ab,kw,rn,tn OR 'natrii aurothiomalas*':ti,ab,kw,rn,tn OR shiosol:ti,ab,kw,rn,tn OR 'gold mercatpsuccinate*':ti,ab,kw,rn,tn",885,13 Jan 2021

#94,"'aurothiomalate'/de OR '(1, 2 dicarboxyethylthio) gold disodium' OR '(1, 2 dicarboxyethylthio) gold disodium salt' OR 'aurothio' OR 'aurothiomalate*' OR 'aurothiomalate sodium' OR 'disodium aurothiomalate' OR 'gold mercaptosuccinate' OR 'gold sodium thiomalate*' OR 'gold thiomalate*' OR 'gold thiomalate sodium' OR 'miocrin' OR 'myochrisin' OR 'myochrisine' OR 'myochrysin' OR 'myochrysine' OR 'myocrisin' OR 'myocrysin' OR 'shiosol' OR 'sodium aurothiomalate' OR 'sodium gold thiomalate' OR 'tauredon' OR 'tauredone' OR 'thiomalate gold'",3454,13 Jan 2021

#93,"hydroxychloroquin*:ti,ab,kw,rn,tn OR hcq:ti,ab,kw,rn,tn OR '747-3-4':ti,ab,kw,rn,tn OR 8q2869cnvh*:ti,ab,kw,rn,tn OR '118-42-3':ti,ab,kw,rn,tn OR 4qwg6n8qkh*:ti,ab,kw,rn,tn OR hydroxychlorochin*:ti,ab,kw,rn,tn OR hydrochloroquin*:ti,ab,kw,rn,tn OR hydrocloroquin*:ti,ab,kw,rn,tn OR oxychlorochin:ti,ab,kw,rn,tn OR oxychloroquin*:ti,ab,kw,rn,tn OR plaquinol:ti,ab,kw,rn,tn OR 'brn 0253894':ti,ab,kw,rn,tn OR brn0253894:ti,ab,kw,rn,tn OR 'einecs 204-249-8':ti,ab,kw,rn,tn OR 'einecs204 249 8':ti,ab,kw,rn,tn OR hidroxicloroquin*:ti,ab,kw,rn,tn OR idrossiclorochin*:ti,ab,kw,rn,tn OR oxichlorochin*:ti,ab,kw,rn,tn OR oxichloroquin*:ti,ab,kw,rn,tn OR win1258:ti,ab,kw,rn,tn OR 'win 1258':ti,ab,kw,rn,tn OR z0188:ti,ab,kw,rn,tn OR 'einecs 212-019-

3':ti,ab,kw,rn,tn OR 'einecs212-019-3':ti,ab,kw,rn,tn OR erquin:ti,ab,kw,rn,tn OR quensyl:ti,ab,kw,rn,tn OR 'sn 8137':ti,ab,kw,rn,tn OR sn8137:ti,ab,kw,rn,tn",29630,13 Jan 2021

#92,"'hydroxychloroquine sulfate'/de OR '1 (7 chloro 4 quinolylamino) 3 diethylamino 2 propanol sulfate' OR '1 (7 chloro 4 quinolylamino) 3 diethylamino 2 propanol sulphate' OR '2 [[4 [(7 chloro 4 quinolinyl) amino] pentyl] (ethyl) amino] ethanol sulfate' OR '2 [[4 [(7 chloro 4 quinolinyl) amino] pentyl] (ethyl) amino] ethanol sulphate' OR '2 [[4 [(7 chloroquinolin 4 yl) amino] pentyl] (ethyl) amino] ethanol sulfate' OR '2 [[4 [(7 chloroquinolin 4 yl) amino] pentyl] (ethyl) amino] ethanol sulphate' OR '7 chloro 4 (3 diethylamino 2 hydroxypropylamino) quinoline sulfate' OR '7 chloro 4 (3 diethylamino 2 hydroxypropylamino) quinoline sulphate' OR 'dimard' OR 'erquin' OR 'evoquin' OR 'geniquin' OR 'hydroxychloroquine sulfate' OR 'hydroxychloroquine sulphate' OR 'oxiklorin' OR 'oxychloroquine sulfate' OR 'oxychloroquine sulphate' OR 'plaquenil' OR 'plaquenil sulfate' OR 'plaquenil sulphate' OR 'plaquinol' OR 'toremonil' OR 'yuma'",3645,13 Jan 2021

#91,"'hydroxychloroquine'/de OR '7 chloro 4 [4 [ethyl (2 hydroxyethyl) amino] 1 methylbutylamino] quinoline' OR '7 chloro 4 [4 [ethyl (2 hydroxyethyl) amino] 1 methylbutylamino] quinoline diphosphate' OR 'apo-hydroxychloroquine' OR 'chloroquinol' OR 'ercoquin' OR 'hydrochloroquine' OR 'hydrocloroquine' OR 'hydroxychloroquine' OR 'oxychloroquine' OR 'quensyl' OR 'sn 8137'",29798,13 Jan 2021

#90,"sasp:ti,ab,kw,rn,tn OR ssz:ti,ab,kw,rn,tn OR salicylazosulfapyridin*:ti,ab,kw,rn,tn OR 'salicyl azo sulfapyridin*':ti,ab,kw,rn,tn OR salazosulfapyridin*:ti,ab,kw,rn,tn OR salazosulfapiridin*:ti,ab,kw,rn,tn OR azulfadin*:ti,ab,kw,rn,tn OR azulfidin*:ti,ab,kw,rn,tn OR pleon:ti,ab,kw,rn,tn OR ulcol:ti,ab,kw,rn,tn OR ucine:ti,ab,kw,rn,tn OR salazopyrin*:ti,ab,kw,rn,tn OR 'ratio-sulfosalazin*':ti,ab,kw,rn,tn OR 3xc8guz6cb*:ti,ab,kw,rn,tn OR accucol:ti,ab,kw,rn,tn OR asulfidin*:ti,ab,kw,rn,tn OR

azopyrin*:ti,ab,kw,rn,tn OR azosulfidin*:ti,ab,kw,rn,tn OR 'brn 0356241':ti,ab,kw,rn,tn OR brn0356241:ti,ab,kw,rn,tn OR 'ccris 4713':ti,ab,kw,rn,tn OR ccris4713:ti,ab,kw,rn,tn OR 'einecs 209-974-3':ti,ab,kw,rn,tn OR 'hsdb 3395':ti,ab,kw,rn,tn OR hsdb3395:ti,ab,kw,rn,tn OR 'nsc 203730':ti,ab,kw,rn,tn OR nsc203730:ti,ab,kw,rn,tn OR 'nsc 667219':ti,ab,kw,rn,tn OR nsc667219:ti,ab,kw,rn,tn OR reupirin:ti,ab,kw,rn,tn OR 'salazo-sulfapyridin*':ti,ab,kw,rn,tn OR salazosulfpyridin*:ti,ab,kw,rn,tn OR salazopiridazin*:ti,ab,kw,rn,tn OR salazopyridin*:ti,ab,kw,rn,tn OR salazopirin*:ti,ab,kw,rn,tn OR salipyr:ti,ab,kw,rn,tn OR 'si-88':ti,ab,kw,rn,tn OR si88:ti,ab,kw,rn,tn OR sulfasalazin*:ti,ab,kw,rn,tn OR sulphasalazin*:ti,ab,kw,rn,tn OR 'w-t sasp oral':ti,ab,kw,rn,tn OR '599-79-1':ti,ab,kw,rn,tn",28709,13 Jan 2021

#89,"'salazosulfapyridine'/de OR '4 (2 pyridylaminosulfonyl) 3` carboxy 4` hydroxyazobenzene' OR '4 hydroxy 4` (pyrid 2 ylsulfamoyl) azobenzene 3 carboxylic acid' OR '5 [4 (2 pyridylsulfamoyl) phenylazo] 2 hydroxybenzoic acid' OR '5 [para (2 pyridylsulfamoyl) phenylazo] salicylic acid' OR 'azlufidine en-tabs' OR 'azopyrin' OR 'azopyrine' OR 'azosulfidine' OR 'azulfide' OR 'azulfidina' OR 'azulfidine' OR 'azulfidine en tabs' OR 'azulfidine en-tabs' OR 'azulfidine ra' OR 'azulfin' OR 'benzosulfa' OR 'colo pleon' OR 'colo-pleon' OR 'colopleon' OR 'disalazin' OR 'gastropyrin' OR 'pleon ra' OR 'pyralin en' OR 'rorasul' OR 'rosulfant' OR 's.a.s.-500' OR 'salazine' OR 'salazo sulfapyridine' OR 'salazodin' OR 'salazopirina' OR 'salazopyridin' OR 'salazopyridine' OR 'salazopyrin' OR 'salazopyrin entabs' OR 'salazopyrin-en' OR 'salazopyrina' OR 'salazopyrine' OR 'salazopyrine ec' OR 'salazosulfa pyridine' OR 'salazosulfapyridine' OR 'salazosulfpyridine' OR 'salicyl azo sulfapyridine' OR 'salicylazosulfapyridin' OR 'salicylazosulfapyridine' OR 'salisulf' OR 'salopyr' OR 'saridine' OR 'sas 500' OR 'sulcolon' OR 'sulfasalazine' OR 'sulfasalizine' OR 'sulfosalazine' OR 'sulphasalazine' OR 'zopyrin'",26746,13 Jan 2021

#88,"'lef':ti,ab,kw",4107,13 Jan 2021

#87,"'leflunomide'/de OR '5 methyl 4` trifluoromethyl 4 isoxazolecarboxanilide' OR '5 methyl n [4 (trifluoromethyl) phenyl] 4 isoxazolecarboxamide' OR '5 methyl n [4 (trifluoromethyl) phenyl] isoxazole 4 carboxamide' OR '5 methyl n [para (trifluoromethyl) phenyl] 4 isoxazolecarboxamide' OR 'alpha, alpha, alpha trifluoro 5 methyl 4 isoxazolecarboxy para toluidide' OR 'arabloc' OR 'arava' OR 'hwa 486' OR 'hwa486' OR 'leflunomid*' OR 'leflunomide winthrop' OR 'n (4 trifluoromethylphenyl) 5 methylisoxazole 4 carboxamide' OR 'repso' OR 'rs 34821' OR 'rs34821' OR 'su 101' OR 'su101' OR 'hsdb 7289' OR hsdb7289 OR g162gk9u4w* OR '75706-12-6'",12973,13 Jan 2021

#86,"'mtx':ti,ab,kw,rn,tn OR methotrex*:ti,ab,kw,rn,tn OR metotrexat*:ti,ab,kw,rn,tn OR methylaminopterin*:ti,ab,kw,rn,tn OR amet$opterin*:ti,ab,kw,rn,tn OR brimexate:ti,ab,kw,rn,tn OR 'a-met$opterin*':ti,ab,kw,rn,tn OR 'a-methpterin*':ti,ab,kw,rn,tn OR 'ai3-25299':ti,ab,kw,rn,tn OR ai325299:ti,ab,kw,rn,tn OR 'alpha-methopterin':ti,ab,kw,rn,tn OR farmitrexat*:ti,ab,kw,rn,tn OR fauldexato:ti,ab,kw,rn,tn OR hdmtx:ti,ab,kw,rn,tn OR metotressat*:ti,ab,kw,rn,tn OR methotrate*:ti,ab,kw,rn,tn OR methohexate*:ti,ab,kw,rn,tn OR mexat*:ti,ab,kw,rn,tn OR metrotex:ti,ab,kw,rn,tn OR metatrexato*:ti,ab,kw,rn,tn OR metatrexan:ti,ab,kw,rn,tn OR maxtrex:ti,ab,kw,rn,tn OR meticil:ti,ab,kw,rn,tn OR metoject:ti,ab,kw,rn,tn OR metotrexin:ti,ab,kw,rn,tn OR lumexon:ti,ab,kw,rn,tn OR r$eumatrex*:ti,ab,kw,rn,tn OR tremetex:ti,ab,kw,rn,tn OR trexeron:ti,ab,kw,rn,tn OR trixilem:ti,ab,kw,rn,tn OR 'mpi-2505':ti,ab,kw,rn,tn OR mpi2505:ti,ab,kw,rn,tn OR amethopterin*:ti,ab,kw,rn,tn OR '133073-73-1':ti,ab,kw,rn,tn OR '15475-56-6':ti,ab,kw,rn,tn OR 3ig1e710zn*:ti,ab,kw,rn,tn OR yl5fz2y5u1*:ti,ab,kw,rn,tn OR 'ccris 1109':ti,ab,kw,rn,tn OR ccris1109:ti,ab,kw,rn,tn OR 'emt 25299':ti,ab,kw,rn,tn OR emt25299:ti,ab,kw,rn,tn OR 'r-9985':ti,ab,kw,rn,tn OR r9885:ti,ab,kw,rn,tn OR 'x-133':ti,ab,kw,rn,tn OR 'wr-19039':ti,ab,kw,rn,tn OR wr19039:ti,ab,kw,rn,tn",191043,13 Jan 2021

#85,"'methotrexate'/de OR '2 [[4 [[(2, 4 diamino 6 pteridinyl) methyl] (methyl) amino] benzoyl] amino] pentanedioic acid' OR '2 [[4 [[(2, 4 diaminopteridin 6 yl) methyl] (methyl) amino] benzoyl] amino] pentanedioic acid' OR '4 amino 10 methylfolic acid' OR '4 amino 10 methylpteroylglutamic acid' OR '4 amino n10 methylpteroylglutamic acid' OR 'mtx' OR 'a methopterine' OR 'abitrexate' OR 'amethopterin' OR 'amethopterine' OR 'ametopterine' OR 'antifolan' OR 'biotrexate' OR 'canceren' OR 'cl 14377' OR 'cl14377' OR 'emtexate' OR 'emthexat' OR 'emthexate' OR 'emtrexate' OR 'enthexate' OR 'farmitrexat' OR 'farmitrexate' OR 'farmotrex' OR 'folex' OR 'folex pfs' OR 'ifamet' OR 'imeth' OR 'intradose mtx' OR 'jylamvo' OR 'lantarel' OR 'ledertrexate' OR 'maxtrex' OR 'metex' OR 'methoblastin' OR 'methohexate' OR 'methotrate' OR 'methotrexat' OR 'methotrexat ebewe' OR 'methotrexate' OR 'methotrexate lpf' OR 'methotrexate preservative free' OR 'methotrexate sodium' OR 'methotrexate sodium preservative free' OR 'methotrexato' OR 'methoxtrexate' OR 'methrotrexate' OR 'methylaminopterin' OR 'methylaminopterine' OR 'meticil' OR 'metoject' OR 'metothrexate' OR 'metothrexate sodium' OR 'metotrexat' OR 'metotrexate' OR 'metotrexin' OR 'metrex' OR 'mexate' OR 'mexate-aq' OR 'mexate-aq preserved' OR 'mpi 5004' OR 'mpi5004' OR 'n [4 [(2, 4 diamino 6 pteridylmethyl) methylamino] benzoyl] glutamic acid' OR 'neotrexate' OR 'nordimet' OR 'novatrex' OR 'nsc 740' OR 'nsc740' OR 'otrexup' OR 'otrexup pfs' OR 'rasuvo' OR 'reditrex' OR 'reumatrex' OR 'rheumatrex' OR 'rheumatrex dose pack' OR 'sodium methotrexate' OR 'texate' OR 'texate-t' OR 'texorate' OR 'trexall' OR 'xaken' OR 'xatmep' OR 'zexate'",191311,13 Jan 2021

#84,"(('disease modifying' NEAR/3 (antir$eum* OR 'anti r$eum*') NEAR/3 (drug$ OR agent$)):ti,ab,kw) OR (('disease modifying' NEAR/3 (antir$eum* OR 'anti r$eum*') NEAR/3 (drug$ OR agent$)):tt)",10971,13 Jan 2021

#83,"dmard*:ti,ab,kw OR csdmard*:ti,ab,kw OR 'cs-dmard*':ti,ab,kw OR sdmard*:ti,ab,kw OR 's-dmard*':ti,ab,kw OR tsdmard*:ti,ab,kw OR 'ts-dmard*':ti,ab,kw OR bdmard*:ti,ab,kw OR 'b-dmard*':ti,ab,kw OR dmard*:tt OR csdmard*:tt OR 'cs-dmard*':tt OR sdmard*:tt OR 's-dmard*':tt OR tsdmard*:tt OR 'ts-dmard*':tt OR bdmard*:tt OR 'b-dmard*':tt",17420,13 Jan 2021

#82,"'disease modifying antirheumatic drug'/de OR 'disease modifying antirheumatic agent' OR 'disease modifying antirheumatic drug' OR 'disease modifying antirheumatic drugs'",20081,13 Jan 2021

#81,"'mavrilimumab'/de OR 'mavrilimumab*' OR '1085337 57 0' OR 1158jdp9a* OR 'cam 3001' OR cam3001",130,13 Jan 2021

#80,"'ustekinumab'/de OR 'cnto 1275' OR 'cnto1275' OR 'monoclonal antibody cnto 1275' OR 'stelara' OR 'ustekinumab*' OR '815610 63 0' OR fu77b4u5z0* OR 'stelera'",7688,13 Jan 2021

#79,"'guselkumab'/de OR 'cnto 1959' OR 'cnto1959' OR 'guselkumab' OR 'tremfya' OR '1350289 85 8' OR 089658a12d*",825,13 Jan 2021

#78,"'brodalumab'/de OR 'amg 827' OR 'amg827' OR 'brodalumab*' OR 'kyntheum' OR 'siliq' OR 'khk 4827' OR khk4827 OR '1174395 19 7' OR 6za31y954z*",1170,13 Jan 2021

#77,"'ixekizumab'/de OR 'ixekizumab' OR 'ly 2439821' OR 'ly2439821' OR 'taltz' OR '1329632 62 3' OR '1143503 69 8' OR bty153760o*",1953,13 Jan 2021

#76,"'secukinumab'/de OR 'ain 457' OR 'ain457' OR 'cosentyx' OR 'secukinumab*' OR '1229022 83 6' OR dlg4eml025*",4059,13 Jan 2021

#75,"'pateclizumab'/de OR 'mlta 3698a' OR 'mlta3698a' OR 'pateclizumab' OR 'pro 283698' OR 'pro283698' OR '12202526 59 7' OR qok1yyh7j2* OR 'rg 7415' OR rg7415",28,13 Jan 2021

#74,"'tabalumab'/de OR 'ly 2127399' OR 'ly2127399' OR 'tabalumab*' OR '1143503 67 6' OR pqp8vh3mjw*",272,13 Jan 2021

#73,"abatacept*:ti,ab,kw,rn,tn OR 'ctla 4 ig':ti,ab,kw,rn,tn OR 'ctla-igg4m':ti,ab,kw,rn,tn OR 'ctla4-fc':ti,ab,kw,rn,tn OR '332348 12 6':ti,ab,kw,rn,tn OR 7d0yb67s97*:ti,ab,kw,rn,tn OR 'rg 2077':ti,ab,kw,rn,tn OR rg2077:ti,ab,kw,rn,tn OR 'rg-1046':ti,ab,kw,rn,tn OR rg1046:ti,ab,kw,rn,tn OR nulojix:ti,ab,kw,rn,tn OR lea29y:ti,ab,kw,rn,tn OR 'lea 29y':ti,ab,kw,rn,tn OR 'ctl4 fc':ti,ab,kw,rn,tn OR ctl4fc:ti,ab,kw,rn,tn OR belatacept:ti,ab,kw,rn,tn OR 'bms 224818':ti,ab,kw,rn,tn OR bms224818:ti,ab,kw,rn,tn OR 'cytotoxic t lymphocyte associat* antigen 4 immunglobulin*':ti,ab,kw,rn,tn",11018,13 Jan 2021

#72,"'abatacept'/de OR 'ctla4 ig' OR 'ctla4 immunoglobulin' OR 'ctla4 immunoglobulin g' OR 'ctla4ig' OR 'abatacept' OR 'bms 188667' OR 'bms188667' OR 'orencia'",11017,13 Jan 2021

#71,"'ofatumumab'/de OR 'humax cd20' OR 'humax-cd20' OR 'humaxcd20' OR 'arzerra' OR 'gsk 1841157' OR 'gsk1841157' OR 'humac cd20' OR 'ofatumumab*' OR 'omb 157' OR 'omb157' OR 'humax-cd20-2f2' OR '679818 59 8' OR m95kg522r0* OR 'hsdb 8170' OR hsdb8170",3081,13 Jan 2021

#70,"'rituximab'/de OR 'abp 798' OR 'abp798' OR 'blitzima' OR 'ct p10' OR 'ctp10' OR 'gp 2013' OR 'gp2013' OR 'hlx 01' OR 'hlx01' OR 'idec 102' OR 'idec c2b8' OR 'idec102' OR 'idecc2b8' OR 'mabthera' OR 'mab thera' OR 'mk 8808' OR 'mk8808' OR 'monoclonal antibody idec c2b8' OR 'pf 05280586' OR 'pf 5280586' OR 'pf05280586' OR 'pf5280586' OR 'r 105' OR 'r105' OR 'reditux' OR 'rg 105' OR 'rg105' OR 'ritemvia' OR 'ritumax' OR 'rituxan' OR 'rituximab*' OR 'rituximab abbs' OR 'rituximab pvvr' OR 'rituximab-abbs' OR 'rituximab-pvvr' OR 'rituxin' OR 'rituzena' OR 'rixathon' OR 'riximyo' OR 'ro 452294' OR 'ro452294' OR 'ruxience' OR 'truxima' OR 'tuxella' OR '174722 31 7' OR 4f4x42syq6* OR 'hsdb 7455' OR hsdb7455",85946,13 Jan 2021

#69,"'czp':ti,ab,kw,rn,tn OR '428863 50 7':ti,ab,kw,rn,tn OR g6adw90r16*:ti,ab,kw,rn,tn OR umd07x179e*:ti,ab,kw,rn,tn OR 'hsdb 7848':ti,ab,kw,rn,tn OR hsdb7848:ti,ab,kw,rn,tn",6870,13 Jan 2021

#68,"'certolizumab pegol'/de OR 'cdp 870' OR 'cdp870' OR 'certolizumab pegol' OR 'cimzia' OR 'pegylated tumor necrosis factor alpha antibody fab fragment' OR 'pegylated tumour necrosis factor alpha antibody fab fragment' OR 'pha 738144' OR 'pha738144'",6949,13 Jan 2021

#67,"'golimumab'/de OR 'cnto 148' OR 'cnto148' OR 'golimumab*' OR 'simponi' OR 'simponi aria' OR '476181 74 5' OR 91x1klu43e*",7366,13 Jan 2021

#66,"adalimumab*:ti,ab,kw,rn,tn OR fys6t7f842*:ti,ab,kw,rn,tn OR '331731 18 1':ti,ab,kw,rn,tn OR d2e7:ti,ab,kw,rn,tn OR 'hsdb 7851':ti,ab,kw,rn,tn OR hsdb7851:ti,ab,kw,rn,tn OR 'bcd-057':ti,ab,kw,rn,tn OR bcd057:ti,ab,kw,rn,tn OR 'chs-1420':ti,ab,kw,rn,tn OR chs1420:ti,ab,kw,rn,tn OR 'sb-5':ti,ab,kw,rn,tn OR sb5:ti,ab,kw,rn,tn",35435,13 Jan 2021

#65,"'adalimumab'/de OR 'abp 501' OR 'abp501' OR 'abrilada' OR 'abt d2e7' OR 'abtd2e7' OR 'adalimumab' OR 'adalimumab adaz' OR 'adalimumab adbm' OR 'adalimumab afzb' OR 'adalimumab atto' OR 'adalimumab beta' OR 'adalimumab bwwd' OR 'adalimumab-adaz' OR 'adalimumab-adbm' OR 'adalimumab-afzb' OR 'adalimumab-atto' OR 'adalimumab-bwwd' OR 'adaly' OR 'amgevita' OR 'amjevita' OR 'amsparity' OR 'avt 02' OR 'avt02' OR 'bat 1406' OR 'bat1406' OR 'bax 2923' OR 'bax 923' OR 'bax2923' OR 'bax923' OR 'bi 695501' OR 'bi695501' OR 'chs 1420' OR 'chs1420' OR 'cinnora' OR 'ct p17' OR 'ctp17' OR 'cyltezo' OR 'da 3113' OR 'da3113' OR 'dmb 3113' OR 'dmb3113' OR 'exemptia' OR 'fkb 327' OR 'fkb327' OR 'fyzoclad' OR 'gp 2017' OR 'gp2017' OR 'hadlima' OR 'halimatoz' OR 'hefiya' OR 'hlx 03' OR 'hlx03' OR 'hulio' OR 'humira' OR 'hyrimoz' OR 'ibi 303' OR 'ibi303' OR 'idacio' OR 'imraldi' OR 'kromeya' OR 'lu 200134' OR 'lu200134' OR 'm 923' OR 'm923' OR 'mabura' OR 'monoclonal antibody

d2e7' OR 'msb 11022' OR 'msb11022' OR 'ons 3010' OR 'ons3010' OR 'pf 06410293' OR 'pf 6410293' OR 'pf06410293' OR 'pf6410293' OR 'raheara' OR 'solymbic' OR 'trudexa' OR 'zrc 3197' OR 'zrc3197'",35367,13 Jan 2021

#64,"etanercept*:ti,ab,kw,tn,rn OR 'rhu tnfr fc':ti,ab,kw,tn,rn OR 'tnfr-immunoadhesin':ti,ab,kw,tn,rn OR 'tnt receptor fusion protein':ti,ab,kw,tn,rn OR 'tum$or necrosis factor receptor fc fusion protein':ti,ab,kw,tn,rn OR 'tnf receptor fusion protein':ti,ab,kw,tn,rn OR 'tnfr fc':ti,ab,kw,tn,rn OR 'recombinant human tnf':ti,ab,kw,tn,rn OR 'recombinant human dimeric tnf receptor type ii igg fusion protein':ti,ab,kw,tn,rn OR 'dwp-422':ti,ab,kw,tn,rn OR dwp422:ti,ab,kw,tn,rn OR 'hd-203':ti,ab,kw,tn,rn OR hd203:ti,ab,kw,tn,rn OR 'lbec 0101':ti,ab,kw,tn,rn OR lbec0101:ti,ab,kw,tn,rn OR 'sb 4':ti,ab,kw,tn,rn OR 'sb4':ti,ab,kw,tn,rn",16020,13 Jan 2021

#63,"'etanercept'/de OR 'avent' OR 'benepali' OR 'brenzys' OR 'chs 0214' OR 'chs0214' OR 'embrel' OR 'enbrel' OR 'enerceptan' OR 'enia 11' OR 'enia11' OR 'erelzi' OR 'etanercept*' OR 'etanercept szzs' OR 'etanercept ykro' OR 'etanercept-szzs' OR 'etanercept-ykro' OR 'eticovo' OR 'gp 2015' OR 'gp 2015c' OR 'gp2015' OR 'gp2015c' OR 'hd 203' OR 'hd203' OR 'infinitam' OR 'lbec 0101' OR 'lbec0101' OR 'lifmior' OR 'nepexto' OR 'opinercept' OR 'recombinant tumor necrosis factor receptor fc fusion protein' OR 'recombinant tumour necrosis factor receptor fc fusion protein' OR 'tnr 001' OR 'tnr001' OR 'tumor necrosis factor receptor fc fusion protein' OR 'tumour necrosis factor receptor fc fusion protein' OR 'tunex' OR 'ylb 113' OR 'ylb113' OR '185243 69 0' OR 'op401g7ojc*'",33171,13 Jan 2021

#62,"ifx:ti,ab,kw,de,tn,rn OR 'mab ca2':ti,ab,kw,de,tn,rn OR 'monoclonal antibody ca2':ti,ab,kw,de,tn,rn OR 'antibody ca2 monoclonal':ti,ab,kw,de,tn,rn OR 'ca2 monoclonal antibody':ti,ab,kw,de,tn,rn OR 'ct p13':ti,ab,kw,de,tn,rn OR ctp13:ti,ab,kw,de,tn,rn OR 'ct-p-13':ti,ab,kw,de,tn,rn OR sb2:ti,ab,kw,de,tn,rn OR 'hsdb 7850':ti,ab,kw,de,tn,rn OR hsdb7850:ti,ab,kw,de,tn,rn",5759,13 Jan 2021

#61,"'infliximab'/de OR 'abp 710' OR 'abp710' OR 'avakine' OR 'avsola' OR 'flixabi' OR 'gp 1111' OR 'gp1111' OR 'inflectra' OR 'infliximab*' OR 'infliximab abda' OR 'infliximab axxq' OR 'infliximab dyyb' OR 'infliximab qbtx' OR 'infliximab-abda' OR 'infliximab-axxq' OR 'infliximab-dyyb' OR 'infliximab-qbtx' OR 'ixifi' OR 'pf 06438179' OR 'pf 6438179' OR 'pf06438179' OR 'pf6438179' OR 'remicade' OR 'remsima' OR 'renflexis' OR 'revellex' OR 'ta 650' OR 'ta650' OR 'zessly' OR '170277 31 3' OR 'b72hh48flu*' OR 'inflecta'",52734,13 Jan 2021

#60,"((('il-1ra' OR il1ra OR 'il-1-ra') NEAR/10 (antagonist* OR 'blocking agent*' OR blocker* OR inhibitor* OR anakinra*)):ti,ab,kw,tn) AND (therap*:ti,ab,kw,de OR treat*:ti,ab,kw,de OR trial$:ti,ab,kw,de)",2220,13 Jan 2021

#59,"'anakinra'/de OR 'anakinra*' OR 'kineret' OR 'recombinant interleukin 1 receptor antagonist' OR 'recombinant interleukin 1 receptor blocker' OR 'recombinant interleukin 1 receptor blocking agent' OR '143090 92 0'",9231,13 Jan 2021

#58,"#47 AND #57",9443,13 Jan 2021

#57,"#48 OR #49 OR #50 OR #51 OR #52 OR #53 OR #54 OR #55 OR #56",30942,13 Jan 2021

#56,"'elsilimomab'/de OR 'b e8' OR 'elsilimomab*' OR '468715 71 1'",38,13 Jan 2021

#55,"'ziltivekimab'/de OR 'cor 001' OR 'cor001' OR 'ziltivekimab*' OR ziltivekimab* OR zilti OR '2226654 05 1'",6,13 Jan 2021

#54,"'siltuximab'/de OR 'cnto 328' OR 'cnto328' OR 'monoclonal antibody cnto 328' OR 'monoclonal antibody cnto328' OR 'siltuximab*' OR 'sylvant' OR cllb8 OR 'ccib8 monoclonal antibody' OR t4h8fma7im* OR '541502 14 1'",891,13 Jan 2021

#53,"'sirukumab'/exp OR 'cnto 136' OR 'cnto136' OR 'plivensia' OR 'sirukumab*' OR '1194585 53 9' OR 640443fu93*",321,13 Jan 2021

#52,"'clazakizumab'/de OR 'ald 518' OR 'ald518' OR 'bms 945429' OR 'bms945429' OR 'clazakizumab*' OR '1236278 28 6' OR 4s38z8ra9o*",224,13 Jan 2021

#51,"'olokizumab'/de OR 'olokizumab*' OR 'cdp 6038' OR cdp6038 OR '1007223 17 7' OR pai71r1d2w*",96,13 Jan 2021

#50,"'levilimab'/de OR 'bcd 089' OR 'bcd089' OR 'levilimab*' OR '2035008 70 7' OR p7uv3l2h80*",5,13 Jan 2021

#49,"'sarilumab'/de OR 'kevzara*' OR 'regn 88' OR 'regn88' OR 'sar 153191' OR 'sar153191' OR 'sarilumab*' OR '1189541 98 7' OR nu90v55f8i*",888,13 Jan 2021

#48,"'tocilizumab'/de OR 'actemra*' OR 'actemra 200' OR 'atlizumab*' OR 'lusinex*' OR 'r1569' OR 'roactemra*' OR 'tocilizumab*' OR r1569 OR 'r 1569' OR 'mra':ti,ab,kw,tn OR mra:tt OR '375823 41 9' OR i031v2h011* OR 'rhpm-1' OR rhpm1 OR 'rg-1569' OR rg1569 OR 'msb-11456' OR msb11456 OR 'ro-4877533' OR ro4877533",29836,13 Jan 2021

#47,"#1 OR #2 OR #3 OR #4 OR #5 OR #6 OR #7 OR #8 OR #9 OR #10 OR #11 OR #12 OR #13 OR #14 OR #15 OR #16 OR #17 OR #23 OR #24 OR #25 OR #26 OR #27 OR #28 OR #29 OR #30 OR #31 OR #32 OR #33 OR #34 OR #35 OR #36 OR #37 OR #38 OR #39 OR #40 OR #41 OR #42 OR #43 OR #44 OR #45 OR #46",313713,13 Jan 2021

#46,"(('brachiocephal*' NEAR/6 arterit*):ti,ab,kw) OR (('brachiocephal*' NEAR/6 arterit*):tt)",29,13 Jan 2021

#45,"(('young female' NEAR/6 arterit*):ti,ab,kw) OR (('young female' NEAR/6 arterit*):tt)",29,13 Jan 2021

#44,"(takayasu* NEAR/3 (arterit* OR syndrom* OR disease*)):tt",255,13 Jan 2021

#43,"(takayasu* NEAR/3 (arterit* OR syndrom* OR disease*)):ti,ab,kw",6517,13 Jan 2021

#42,"'aortic arch syndrome'/de",9191,13 Jan 2021

#41,"(('large vessel' NEAR/3 (vasculit* OR arterit*)):ti,ab,kw) OR (('large vessel' NEAR/3 (vasculit* OR arterit*)):tt)",2150,13 Jan 2021

#40,"'large vessel vasculitis'/de",236,13 Jan 2021

#39,"(gca:ti,ab,kw OR gca:tt) AND (arterit*:ti,ab,kw,de OR vasculit*:ti,ab,kw,de OR r$eum*:ti,ab,kw,de,jt)",3607,13 Jan 2021

#38,"'temporal arteritis'/de",4264,13 Jan 2021

#37,"((horton OR 'horton s' OR hortons) NEAR/3 disease*):tt",0,13 Jan 2021

#36,"((horton OR 'horton s' OR hortons) NEAR/3 disease*):ti,ab,kw",578,13 Jan 2021

#35,"((temporal OR cranial) NEAR/2 arterit*):ti,ab,kw",3161,13 Jan 2021

#34,"('giant cell' NEAR/6 (arterit* OR aortit* OR horton*)):tt",5,13 Jan 2021

#33,"('giant cell' NEAR/6 (arterit* OR aortit* OR horton*)):ti,ab,kw",7766,13 Jan 2021

#32,"'giant cell arteritis'/de",8369,13 Jan 2021

#31,"(polyart*:tt OR oligoart*:tt OR 'poly art*':tt OR 'oligo art*':tt) AND (jia:tt OR 'juvenile idiopathic arthritis':tt OR 'juvenile onset idiopathic arthritis':tt) OR pjia:tt OR 'p-jia':tt",1,13 Jan 2021

#30,"(polyart*:ti,ab,kw OR oligoart*:ti,ab,kw OR 'poly art*':ti,ab,kw OR 'oligo art*':ti,ab,kw) AND (jia:ti,ab,kw OR 'juvenile idiopathic arthritis':ti,ab,kw OR 'juvenile onset idiopathic arthritis':ti,ab,kw) OR pjia:ti,ab,kw OR 'p-jia':ti,ab,kw",3386,13 Jan 2021

#29,"(('stills disease*' OR 'still s disease*' OR 'still disease*') NEAR/6 child*):tt",0,13 Jan 2021

#28,"(('stills disease*' OR 'still s disease*' OR 'still disease*') NEAR/6 child*):ti,ab,kw",89,13 Jan 2021

#27,"('stills disease*':tt OR 'still s disease*':tt OR 'still disease*':tt) AND juvenile*:tt",0,13 Jan 2021

#26,"('stills disease*':ti,ab,kw OR 'still s disease*':ti,ab,kw OR 'still disease*':ti,ab,kw) AND juvenile*:ti,ab,kw",516,13 Jan 2021

#25,"((polyarthr* OR polyartrit* OR 'poly arthrit*' OR 'poly artrit*' OR oligoarthrit* OR oligoartrit* OR 'oligo arthrit*' OR 'oligo artrit*') NEAR/6 juvenile*):tt",80,13 Jan 2021

#24,"((polyarthr* OR polyartrit* OR 'poly arthrit*' OR 'poly artrit*' OR oligoarthrit* OR oligoartrit* OR 'oligo arthrit*' OR 'oligo artrit*') NEAR/6 juvenile*):ti,ab,kw",326,13 Jan 2021

#23,"#21 AND #22",9491,13 Jan 2021

#22,"systemic:ti,ab,kw OR polyart*:ti,ab,kw OR 'poly art*':ti,ab,kw OR oligoart*:ti,ab,kw OR 'oligo art*':ti,ab,kw OR systemic:tt OR polyart*:tt OR 'poly art*':tt OR oligoart*:tt OR 'oligo art*':tt",727145,13 Jan 2021

#21,"#18 OR #19 OR #20",26056,13 Jan 2021

#20,"jia:ti,ab,kw OR jia:tt",9549,13 Jan 2021

#19,"(((arthrit* OR artrit*) NEAR/6 juvenile*):ti,ab,kw) OR (((arthrit* OR artrit*) NEAR/6 juvenile*):tt)",17967,13 Jan 2021

#18,"'juvenile rheumatoid arthritis'/de",21478,13 Jan 2021

#17,"((systemic NEAR/6 (jia OR 'juvenile idiopathic arthritis' OR 'juvenile onset idiopathic arthriti')):tt) OR sjia:tt OR 's-jia':tt",3,13 Jan 2021

#16,"((systemic NEAR/6 (jia OR 'juvenile idiopathic arthritis' OR 'juvenile onset idiopathic arthriti')):ti,ab,kw) OR sjia:ti,ab,kw OR 's-jia':ti,ab,kw",3365,13 Jan 2021

#15,"'systemic juvenile idiopathic arthritis'/de",1420,13 Jan 2021

#14,"'rheumatic disease'/de",50593,13 Jan 2021

#13,"((rheumat* OR reumat* OR revmat* OR rheumo OR reumo) NEAR/2 (condition* OR diseas*)):tt",13,13 Jan 2021

#12,"((rheumat* OR reumat* OR revmat* OR rheumo OR reumo) NEAR/2 (condition* OR diseas*)):ti,ab,kw",51268,13 Jan 2021

#11,"((r$eum* OR r$evm*) NEAR/2 (chronic* OR articular*)):tt",87,13 Jan 2021

#10,"((r$eum* OR r$evm*) NEAR/2 (chronic* OR articular*)):ti,ab,kw",5714,13 Jan 2021

#9,"(inflammator* NEAR/2 (arthrit* OR artrit*)):tt",3,13 Jan 2021

#8,"(inflammator* NEAR/2 (arthrit* OR artrit*)):ti,ab,kw",12485,13 Jan 2021

#7,"(chronic* NEAR/2 (polyarthrit* OR 'poly arthrit*' OR polyartrit* OR 'poly artrit*')):tt",86,13 Jan 2021

#6,"(chronic* NEAR/2 (polyarthrit* OR 'poly arthrit*' OR polyartrit* OR 'poly artrit*')):ti,ab,kw",2522,13 Jan 2021

#5,"r$eumarthrit*:tt OR r$eumartrit*:tt OR 'r$eum arthrit*':tt OR 'r$eum artrit*':tt OR r$evmarthrit*:tt OR r$evmartrit*:tt OR 'r$evm arthrit*':tt OR 'r$evm artrit*':tt OR 'r$eum polyarthrit*':tt OR 'r$eum polyartrit*':tt OR 'r$eum poly arthrit*':tt OR 'r$eum poly artrit*':tt OR r$eumpolyarthrit*:tt OR r$eumpolyartrit*:tt",0,13 Jan 2021

#4,"r$eumarthrit*:ti,ab,kw OR r$eumartrit*:ti,ab,kw OR 'r$eum arthrit*':ti,ab,kw OR 'r$eum artrit*':ti,ab,kw OR r$evmarthrit*:ti,ab,kw OR r$evmartrit*:ti,ab,kw OR 'r$evm arthrit*':ti,ab,kw OR 'r$evm artrit*':ti,ab,kw OR 'r$eum polyarthrit*':ti,ab,kw OR 'r$eum polyartrit*':ti,ab,kw OR 'r$eum poly arthrit*':ti,ab,kw OR 'r$eum poly artrit*':ti,ab,kw OR r$eumpolyarthrit*:ti,ab,kw OR r$eumpolyartrit*:ti,ab,kw",10,13 Jan 2021

#3,"((rheumat* OR reumat* OR revmat* OR rheumo OR reumo) NEAR/3 (arthrit* OR artrit* OR polyarthrit* OR polyartrit*)):tt",6622,13 Jan 2021

#2,"((rheumat* OR reumat* OR revmat* OR rheumo OR reumo) NEAR/3 (arthrit* OR artrit* OR polyarthrit* OR polyartrit*)):ti,ab,kw",172469,13 Jan 2021

#1,"'rheumatoid arthritis'/de",200422,13 Jan 2021

1.3.5.3 Cochrane CENTRAL

1 Arthritis, Rheumatoid/ (5819)

2 ((rheumat* or reumat* or revmat* or rheumo or reumo) adj3 (arthrit* or artrit* or polyarthrit* or polyartrit*)).mp. (17097)

3 (r?eumarthrit* or r?eumartrit* or r?eum arthrit* or r?eum artrit* or r?evmarthrit* or r?evmartrit* or r?evm arthrit* or r?evm artrit* or r?eum polyarthrit* or r?eum polyartrit* or r?eum poly arthrit* or r?eum poly artrit* or r?eumpolyarthrit* or r?eumpolyartrit*).mp. (3)

4 (chronic* adj2 (polyarthrit* or poly arthrit* or polyartrit* or poly artrit*)).mp. (143)

5 (inflammator* adj2 (arthrit* or artrit*)).mp. (668)

6 ((r?eum* or r?evm*) adj2 (chronic* adj2 articular*)).mp. (14)

7 ((rheumat* or reumat* or revmat* or rheumo or reumo) adj2 (condition* or diseas*)).mp. (5045)

8 Arthritis, Juvenile/ (299)

9 ((arthrit* or artrit*) adj6 juvenile*).mp. (1001)

10 JIA.ti,ot,ab. (627)

11 8 or 9 or 10 (1123)

12 (systemic or polyart* or poly art* or oligoart* or oligo art*).mp. (52595)

13 11 and 12 (515)

14 ((polyarthr* or polyartrit* or poly arthrit* or poly artrit* or oligoarthrit* or oligoartrit* or oligo arthrit* or oligo artrit*) adj6 juvenile*).mp. (9)

15 (((stills or still's or still) adj disease) and juvenile*).mp. (31)

16 ((stills or still's or still) adj disease adj6 child*).mp. (1)

17 ((systemic adj6 JIA) or sJIA or s-JIA).mp. (167)

18 (((polyart* or oligoart* or poly art* or oligo art*) and JIA) or pJIA or p-JIA).mp. (211)

19 Giant Cell Arteritis/ (90)

20 (giant cell adj6 (arterit* or aortit* or horton*)).mp. (297)

21 ((temporal or cranial) adj2 arterit*).mp. (44)

22 ((horton or horton's or hortons) adj3 disease?).mp. (4)

23 (large vessel adj3 (vasculit* or arterit*)).mp. (55)

24 GCA.ti,ot,ab. and (arterit* or vasculit* or rheum*).mp,jw. (209)

25 Takayasu Arteritis/ (27)

26 (takayasu* adj3 (arterit* or syndrom* or disease*)).mp. (102)

27 (young female adj6 arterit*).mp. (0)

28 (brachiocephal* adj6 arterit*).mp. (0)

29 (or/1-7) or (or/13-28) (20006)

30 (tocilizumab* or actemra* or atlizumab* or lusinex* or r1569 or r 1569 or roactemra* or mra or 375823 41 9 or i031v2h011* or rhpm-1 or rhpm1 or rg-1569 or rg1569 or msb-11456 or msb11456 or ro-4877533 or ro4877533).mp. (1989)

31 (sarilumab* or kevzara* or regn 88 or regn88 or sar 153191 or sar153191 or 1189541 98 7 or nu90v55f8i*).mp. (255)

32 (levilimab* or BCD-089 or bcd089 or 2035008 70 7 or P7UV3L2H80*).mp. (5)

33 (olokizumab* or cdp 6038 or cdp6038 or 1007223 17 7 or pai71r1d2w*).mp. (35)

34 (clazakizumab* or ald 518 or ald518 or bms 945429 or bms945429 or 1236278 28 6 or 4s38z8ra9o*).mp. (38)

35 (sirukumab* or cnto 136 or cnto136 or pilvensia or 1194585 53 9 or 640443FU93*).mp. (118)

36 (siltuximab* or sylvant or cnto 328 or cnto-328 or cnto328 or cllb8 or ccib8 monoclonal antibody or sylvant or t4h8fma7im* or 541502 14 1).mp. (78)

37 (ziltivekimab* or zilti or "cor-001" or "2226654 05 1").mp. (5)

38 (elsilimomab* or "b-e8" or 468715 71 1).mp. (2)

39 30 or 31 or 32 or 33 or 34 or 35 or 36 or 37 or 38 (2464)

40 29 and 39 (1230)

41 Interleukin 1 Receptor Antagonist Protein/ (305)

42 (anakinra* or kineret or ((interleukin 1 receptor or il1 receptor or il 1 receptor) adj1 antagonist protein*) or (recombinant interleukin 1 receptor adj1 (antagonist* or blocker* or blocking agent*)) or antril or il 1ra or il1ra or il 1 ra or ((urin* or febrile) adj2 (interleukin 1 inhibitor* or il1 inhibitor* or il 1 inhibitor*)) or "143090 92 0" or 9013duq28k*).mp. (1087)

43 Infliximab/ (732)

44 (infliximab* or ifx or avakine or flixabi or inflectra or inflecta or ixifi or remicade or remsima or revellex or renflexis or zessly or mab ca2 or monoclonal antibody ca2 or antibody ca2 monoclonal or ca2 monoclonal antibody or ct p13 or ctp13 or ct-p-13 gp-1111 or gp1111 or ta-650 or ta650 or abp 710 or abp710 or bow-015 or bow015 or 170277 31 3 or b72hh48flu* or sb2 or "pf 06438179" or pf 6438179 or pf06438179 or pf6438179 or hsdb 7850 or hsdb7850).mp. (2769)

45 Etanercept/ (758)

46 (etanercept* or benepali or embrel or enbrel or enia 11 or enia11 or erelzi or lifmior or opinercept or recombinant tumo?r necrosis factor receptor fc fusion protein or "tnr 001" or tnr001 or tnfr-fc or tnrfr:fc or rhu tnfr:fc or rhu-tnfr:fc or tnfr-immunoadhesin or tnt receptor fusion protein or tum?or necrosis factor receptor fc fusion protein or tunex or "185243 69 0" or op401g7ojc* or brenzys or tnf receptor fusion protein or tnfr fc or recombinant human tnf or recombinant human dimeric tnf receptor type ii igg fusion protein or chs-0214 or chs0214 or dwp-422 or dwp422 or enia-11 or enia11 or gp-2015 or gp2015 or gp2015c or gp-2015c or hd-203 or hd203 or lbec-0101 or lbec0101 or sb-4 or sb4).mp. (2451)

47 Adalimumab/ (752)

48 (adalimumab* or humira or abp 501 or abp501 or abtd2e7 or amjevita or amgevita or bax 2923 or bax2923 gp 2017 or gp2017 or halimatoz or hefiya or hulio or hyrimoz or ibi 303 or ibi303 or imraldi or m 923 or m923 or msb 11022 or msb11022 or ons3010 or ons 3010 or "pf 06410293" or pf 6410293 or pf06410293 or pf6410293 or d2e7 or truxeda or cyltezo or solymbic or fys6t7f842* or 331731 18 1 or lu 200134 or lu200134 or d2e7 or hsdb 7851 or hsdb7851 or abp-501 or abp501 or bcd-057 or bcd057 or bi-695501 or bi695501 or chs-1420 or chs1420 or gp-2017 or gp2017 or sb-5 or sb5).mp. (3403)

49 (golimumab* or cnto 148 or cnto148 or simponi or 476181 74 5 or 91x1klu43e*).mp. (751)

50 Certolizumab Pegol/ (167)

51 (certolizumab* or cdp 870 or cdp870 or cimzia or pegylated tumor necrosis factor alpha antibody fab fragment or pegylated tumour necrosis factor alpha antibody fab fragment or pha 738144 or pha738144 or czp or 428863 50 7 or g6adw90r16* or umd07x179e* or hsdb 7848 or hsdb7848).mp. (705)

52 Rituximab/ (1268)

53 (rituximab* or ct p10 or ctp10 or idec 102 or idec102 or idecc2b8 or idec c2b8 or monoclonal antibody idec c2b8 or gp2013 or gp 2013 or "pf 05280586" or pf05280586 or mabthera or mab thera or r 105 or r105 or reditux or rg 105 or rg105 or rituxan or rituxin or ritemvia or rituzena or rixathon or riximyo or ro 452294 or ro452294 or truxima or 174722 31 7 or 4f4x42syq6* or hsdb 7455 or hsdb7455).mp. (5326)

54 (ofatumumab* or humax cd20 or humax-cd20 or humaxcd20 or humax-cd20-2f2 or arzerra or gsk 1841157 or gsk1841157 or gski841157 or humax cd20 or omb 157 or omb157 or 679818 59 8 or m95kg522r0* or hsdb 8170 or hsdb8170).mp. (298)

55 Abatacept/ (280)

56 (abatacept* or ctla4 ig or ctla4 immunglobulin or ctla4 immunoglobulin g or ctla4ig or 'ctla 4 ig' or ctla-igg4m or ctla4-fc or bms 188667 or bms188667 or orencia or '332348 12 6' or 7d0yb67s97* or rg 2077 or rg2077 or rg-1046 or rg1046 or nulojix or lea29y or lea 29y or ctl4 fc or ctl4fc or belatacept or bms 224818 or bms224818 or belatacept or cytotoxic t lymphocyte associat* antigen 4).mp. (1151)

57 (tabalumab* or ly 2127399 or ly2127399 or 1143503 67 6 or pqp8vh3mjw*).mp. (70)

58 (pateclizumab* or mlta 3698a or mlta3698a or pro 283698 or pro283698 or 12202526 59 7 or qok1yyh7j2* or rg 7415 or rg7415).mp. (7)

59 (secukinumab* or cosentyx or ain 457 or ain457 or 1229022 83 6 or dlg4eml025*).mp. (917)

60 (ixekizumab* or ly 2439821 or ly2439821 or taltz or 1329632 62 3 or 1143503 69 8 or bty153760o*).mp. (489)

61 (brodalumab* or amg 827 or amg827 or khk 4827 or khk4827 or siliq or kyntheum or 1174395 19 7 or 6za31y954z*).mp. (178)

62 (guselkumab* or cnto 1959 or cnto1959 or 1350289 85 8 or tremfya or 1350289 85 8 or 089658a12d*).mp. (227)

63 Ustekinumab/ (195)

64 (ustekinumab* or cnto 1275 or cnto1275 or stelara or "815610 63 0" or fu77b4u5z0* or 1275 cnto or 1275cnto or l04ac05 or tt-20 or tt20).mp. (897)

65 (mavrilimumab or "1085337 57 0" or 1158jdp9a* or cam 3001 or cam3001).mp. (53)

66 exp Antirheumatic Agents/ (34893)

67 (dmard* or csdmard* or cs-dmard* or sdmard* or s-dmard* or tsdmard* or ts-dmard* or bdmard* or b-dmard*).mp. (3147)

68 (disease modifying adj3 (antirheum* or anti rheum*) adj3 (drug? or agent?)).mp. (2123)

69 ((antirheum* or anti rheum*) adj5 (drug? or agent?)).mp. (4248)

70 Methotrexate/ (4137)

71 (mxt or methotrexat* or metotrexat* or methoblastin or methylaminopterin* or amet?opterin* or abitrexate or antifolan or brimexate or a-met?opterin* or a-methpterin* or ai3-25299 or ai325299 or alpha-methopterin or biotrexate or emtexate or emtrexate* or emthexat* or enthexate* or farmitrexat* or farmotrex or fauldexato or folex or folex pfs or hdmtx or ifamet or imeth or maxtrex or metotressat* or methotrate* or methohexate* or mexate* or metrotex or metatrexato* or metex or metrex or metatrexan or maxtrex or meticil or metoject or metotrexin or novatrex* or neotrexate* or nordimet or lumexon or ledertrexate* or lanterel or rasuvo or r?eumatrex* or reditrex or rasuvo or texate or texate-t or tremetex or trexeron or trixilem or texorate or trexall or xaken or xatmep or otrexup or mpi-2505 or mpi2505 or mpi 5004 or mpi5004 or amethopterin* or 133073-73-1 or 15475-56-6 or 3ig1e710zn* or yl5fz2y5u1* or cl 14377 or cl14377 or ccris 1109 or ccris1109 or emt 25299 or emt25299 or nsc-740 or nsc740 or r-9985 or r9885 or x-133 or wr-19039 or wr19039).mp. (12111)

72 Leflunomide/ (153)

73 (leflunomid* or hwa 486 or hwa486 or su101 or su 101 or arava or arabloc or hsdb 7289 or hsdb7289 or g162gk9u4w* or 75706-12-6 or rs 34821 or rs34821).mp. (688)

74 lef.ti,ot,ab. (173)

75 Sulfasalazine/ (469)

76 (sulfasalazin* or sasp or ssz or salicylazosulfapyridin* or salicyl azo sulfapyridin* or sulphasalazin* or salazosulfapyridin* or salazosulfapiridin* or 'pyralin en' or azulfadin* or azulfidin* or asulfidin* or azulfide* or azulfin or colo-pleon or colopleon or disalazin or gastropyrin or pleon or "pleon ra" or "pyralin en" or rorasul or rosulfant or ulcol or ucine or salazopyrin* or ratio-sulfosalazin* or sulfasalazin* or 3xc8guz6cb* or accucol or asulfidin* or azopyrin* or azosulfidin* or benzosulfa or "brn 0356241" or brn0356241 or ccris 4713 or ccris4713 or einecs 209-974-3 or hsdb 3395 or hsdb3395 or nsc 203730 or nsc203730 or nsc 667219 or nsc667219 or reupirin or rorasul or salazo-sulfapyridin* or salazosulfpyridin* or salazopiridazin* or salazopyridin* or salazopirin* or salazodin or salisulf or salipyr or saridine or si-88 or si88 or sulculon or sulfasalazin* or sulphasalazin* or w-t sasp oral or 599-79-1 or "s.a.s. 500" or "s.a.s.-500" or sas-500 or zopyrin*).mp. (1596)

77 Hydroxychloroquine/ (505)

78 (hydroxychloroquin* or hcq or 747-3-4 or 8q2869cnvh* or 118-42-3 or 4qwg6n8qkh* or hydroxychlorochin* or hydrochloroquin* or hydrocloroquin* or oxychlorochin or oxychloroquin* or plaquenil or plaquinol or "brn 0253894" or brn0253894 or einecs 204-249-8 or einecs204-249-8 or hidroxicloroquin* or idrossiclorochin* or oxichlorochin* or oxichloroquin* or oxiklorin or win1258 or win 1258 or z0188 or einecs 212-019-3 or einecs212-019-3 or chloroquinol or dimard or ercoquin or evoquin or erquin or geniquin or quensyl or sn 8137 or sn8137 or toremonil or yuma).mp. (1666)

79 Gold Sodium Thiomalate/ (91)

80 (gold sodium thiomalate* or aurothiomalate* or aurolate* or gold thiomalate* or gold disodium thiomalate* or gold thiomalic acid* or miocrin or miocrisin or monogold disodium thiomalate* or myochrysin* or myocrisin* or myocrysin* or sodium aurothiomalate* or sodium gold thiomalate* or sodium thiomalate* gold or sodium thiomalatoaurate* or tauredon or taureodon or tauredone or thiomalate gold or thiomalatoaurate sodium or 1244-57-4 or 39377-38-3 or 4846-27-9 or 74916-57-7 or e4768zy6gm* or aurothiomala-natrium or aurot?iomalato sodico or monogold* sodium salt or dinatrium 2-aurothio-succinat or einecs 235-479-7 or einecs235-479-7 or hsdb 7173 or hsdb7173 or kidon or natrii aurothiomalas* or shiosol or gold mercatpsuccinate*).mp. (156)

81 Aurothioglucose/ (36)

82 (thioglucosoaurate* or gold thioglucose* or gold thio glucose* or thioglucose gold or aureotan or solganal or solganol or gold-50 or gold50 or solganal b or b oleosum solganal or auromyose* or aurothioglucose* or auro thioglucose* or aurotan or aurumine or aurumin or authron or brenol or ccris 59 or ccris59 or einecs 235-365-7 or einecs235-365-7 or glysanol b or goldthioglucose* or goldtioglucose* or hsdb 7174 or hsdb7174 or oronol or romosol or skf 10056 or skf10056 or 2p2v9q0e78* or 12192-57-3).mp. (49)

83 ((gold* or auro*) adj10 (inject* or intramuscular* or im or intra muscular*)).ti,ot,ab. (210)

84 Organogold Compounds/ (15)

85 (aurothiopropanol* or allochrysine or allocrisine or aurotiopoe or aurotioprol or 27279-43-2 or g7097j63e9* or auromercapto propanol sulfonate sodium or auromercaptopropanolsulfonate sodium or aurothioisopropanolsulfonate sodium or sodium aurothiopropanolsulfonate or gold sodium thiopropanol sulfonate or sodium auromercaptopropanol sulfonate).mp. (3)

86 Chloroquine/ (709)

87 (chloroquin* or cq or 54-04-7 or 886u3h6uff* or aralen or aralene or arechin or arechine or arequin or chingamin or chlorochin* or khingamin or nivaquine or oe48649kh6n* or amokin or amokine or anaclor or aralan or arequine or artriquine or avloclor or avoclor or arthrochin or artrichin or bemaco or bemaphata or bemaphate or bemasulph or benaquin or bipiquin or cadiquin or "brn 0482809" or brn0482809 or capquin or ccris 3439 or ccris3439 or chemochin or chemochine or chingamine or chingaminum or chloraquin* or chlorquin or chlorquine or choloquine or choroquine sulfate or choroquine sulphate or cindacin or clo-kit junior or clorichina or clorichine or cloriquine or clorochina or delagil or delagyl or diclokin or chlorofoz or cindachin or clorochina or cloroquina or cocatrit or dichinalex or diquinalex or diroquine or emquin or genocin or gontochin or gontochine or gontoquine or einecs 200-191-2 or einecs200-191-2 or elestol or gontochin or heliopar or hsdb 3029 or hsdb3029 or imagon or iroquine or klorokin or klorokine or klorokinfosfat or lagaquin or lapaquin or malaquin or malaren or malarex or malarivon or malaviron or maliaquine or maquine or mesylith or mexaquin or mirquin or neochin or nivaquin or nivachine or nivaquine b or nivaquine dp or nivaquine forte or "p roquine" or nsc 187208 or nsc187208 or pfizerquine or quensyl or quinachlor or quinercyl or quingamine or quinilon or quinoline or quinoscan or repal or resochen or resochene or resochin or resochin junior or resochina or resochine or resochinon or resoquina or resoquine or reumachlor or ronaquine or roquine or rp 3377 or rp3377 or sanoquin or sanoquine or silbesan or siragan or sirajan or sn 7618 or sn7618 or solprina or solprine or sopaquin or st 21 or tresochin or tresoquine or trochin or trochine or troquine or weimerquin or w 7618 or w7618 or win 244 or win244).mp. (2086)

88 exp Adrenal Cortex Hormones/ (27587)

89 exp Prednisolone/ (4835)

90 Prednisone/ (3978)

91 Cortisone/ (148)

92 Hydrocortisone/ (5944)

93 exp Steroids/ (53553)

94 adrenal cortex hormones.mp. (2224)

95 prednisolone*.mp. (7414)

96 methylprednisolone*.mp. (5485)

97 (prednisolone* or predonine or di-adreson-f or diadresonf or 9phq9y1olm* or prednisolona* or prednisolonum* or nsc-9120 or nsc9120 or nsc 9900 or nsc9900 or delta-dehydrocortisol* or delta-dehydrocortisone* or delta-hydrocortisone or hydroretrocortine or metacortandralone* or brn 1354103 or brn1354103 or bubbli-pred or ccris 980 or ccris980 or co-hydeltra or codelcortone or cordrol or cortalone or cotogesic or cotolone or decaprednil or decortin h or delcortol or dehydrohydrocortisone* or delta-cortef or deltacortef or delta-ef-cortelan or delta-stab or deltacortenol or deltacortril or deltahydrocortisone* or deltisilone or derpo pd or dexa-dortidelt hostacortin h or di-adreson f or dicortol or donisolone or dydeltrone or eazolin d or ec 200-021-7 or ec200-021-7 or einecs 200-021-7 or einecs200-021-7 or erbacort or erbasona or estilsona or fernisolone or fernisolone p or hostacortin h or hsdb 3385 or hsdb3385 or hydeltra or hydeltrone or hydrodeltalone or hydrodeltisone or hydroretrocortin or hydroretrocortine* or k 1557 or k1557 or lentosone or meti-drm or meticortelone or orapred or orapred odt or paracortol or paracotol or pediapred or precortancyl or precortilon or precortisyl or predne-dome or prednelan or prednicen or predniliderm or predniretard or prednis or predonin or predonine* or prelon or prelone or prenolone or rolisone or scherisolon? or solone or sterolone or ulacort or ultracorten h or ultracortene h adelcort or antisolon or antisolone or aprednisolon? or benisolon? or caberdelta or co hydeltra or codelcortone or ompresolon).mp. (7432)

98 (cortadeltona or cortelinter or cortisolone or dacortin or dacortin h or dacrotin or decaprednil or decortil or delta cortril or delta hycortol or deltacortenolo or deltacortil or deltacortoil or deltaderm or deltadglycortril or deltahycortol or deltahydrocortison? or deltaophticor or deltasolone or deltastab or deltidrosol or deltisilone or deltisolon? or deltolasson? or deltosona or deltosone or depo-predate or dermosolon or dhasolone or di adreson? f or diadreson? f or dicortol or domucortone or encortelon? or encortolon or equisolon or glistelone or hefasolon or hydrelta or hydrocortancyl or hydrocortidelt or hydradeltalone or hydradeltisone or inflanefran or insolone or keteocort h or key-pred or key-pred sp or lenisolone or leocortol or liquipred or mediasolone or meprisolon? or metacortalon? or metacortelone or meti derm or metiderm or meticortelone or morlone or mydrapred or nisolon? or opredsone or panafcortelone or panafcortolone or panafort or paracortol pr phlogex or pre cortisyl or preconin or precortalon or precortancyl or precortisyl or pred ject 50 or predacort 50 or predaject 50 or predalone 50 or predartrina or predartrine or predate 50 or predeltilone or predisole or predisyr or predne dome or prednecort or prednedome or prednelan or predni coelin or predni h tablinen or predni helvacort or prednicoelin or prednicort or prednicortelone or prednifor drops or predniment or prednorsolon? or predorgasolon? or prenilone or preventan or prezolon or rubycort or serilone or solonda or solupren? or spiricort or spolotane or supercorti#ol or taracortelone or walesolone or wysolone).mp. (23)

99 (methylprednisolone * or methylprednisolona* or methylprednisolonum* or metipred or urbason or medrol or x4w7zr7023* or 83-43-2 or besonia or brn 23403300 or brn2340300 or a-methapred or artisone-wyeth or besonia or depo-medrol or dopomedrol ec 201-4764 pr ec2014764 or einecs 201-476-4 or einecs201-476-4 or esametone or firmacort or hsdb3127 or hsdb3127 or lemod or medesone or medixon or medlone 21 or medrate or medrol or medrone or mesopren or metastab or methyleneprednisolone* or metilbetasone or metrisone or metrocort or metysolon or moderin or nirypan or noretona or nsc 19987 or nsc19987 or predni n tablinen or prednol-l or promacortine or reactenol or sieropresol or solomet or summicort or suprametil or u 7532 or u7532 or "u 67 590a" or urbason or urbasone or wyacort or adlone 40 or adlone 80 or beta methylprednisolone or medalone 80 or depmedalone or deproject 80 or depopred

or esametone or firmacort or med-jec-40 or medixon or mednin or medralone or mepredprednisolone or meprelon or mesopren or methacort 40 or methacort 80 or methyxlcotol or methylcotolone or methylpred dp or methylsterolone or metidrol or metycortin or metypred or metypresol or neomedrone or solu decortin or urbason).mp. (1215)

100 (prednison or prednisone or prednisona or prednisonum or dehydrocortisone or delta cortisone or rectodelt or sterapred or ultracorten or winpred or apo prednisone or cortan or cartancyl or panafcort or cutason or decortin or dacortin or decortisyl or deltasone or encortone or encorton or enkorton or enkortolon or kortancyl or liquid pred or meticorten or orasone or panasol or predni tablinen or prednidib or predniment or pronisone or vb0r961hzt* or 53-03-2 or adasone or al3-52939 or ancortone or bicortone or cartancyl or ccris 2646 or ccrs2646 or colisone or cortan or cortidelt or cotone or dacorten or dacortin or decortancyl or dekortin or dellacort or delta cortelan or delta cortisone or delta dome or deltacortene or deltacortisone or deltacortone or deltasone or deltison or deltisona or deltra or di adreson or diadreson or econosone or einecs 200-160-3 or einecs200-160-3 or fernisone or fiasone or hostacortin or hsdb 3168 or hsdb3168 or incocortyl or juvason or lisacort or lodotra or lodtra).mp. (10119)

101 (me-korti or metacortandracin or metacorten or nci c04897 or ncic04897 or nisona or nizon or novoprednisone or nsc 10023 or nsc10023 or nurison or orasone or panafcort or paracort or prarmenison or pehacort or predeltin or prednicen-m or prednicorm or prednicort or prednicot or prednilonga or prednitone or prednizon or prednovister or presone or pronison or rayos or rectodelt or retrocortine or servisone or sterapred or supercortio or u 6020 or u6020 or ultracorten or ultracortene or winpred or wojtab or zenadrid).mp. (19)

102 cortisone?.mp. (569)

103 corticosteroid*.mp. (23083)

104 corticoid*.mp. (556)

105 glucocorticoid*.mp. (8890)

106 corticosterone*.mp. (153)

107 hydrocortisone*.mp. (9702)

108 dexamethasone*.mp. (12333)

109 beclomethasone*.mp. (2295)

110 (triamcinolone* or 1zk20vi6ty* or 124-94-7 or aristocort or volon or fluoxyprednisolon* or tiamcinolonum or triamcinolonum* or triamcinolona* or brn 2341955 or brn2341955 or cl 19823 or cl19823 or einecs 204-718-7 or einecs204-718-7 or hsdb 3194 or hsdb3194 or nsc 13397 or nsc13397).mp. (3241)

111 (steroid? adj10 (therap* or treat* or drug? or inject* or intramuscular* or intra muscular* or im or intravenous* or intra venous* or iv or intraarticular* or intra articular* or ia or administrat* or oral or orally or parenteral*)).mp. (14182)

112 Janus Kinase Inhibitors/ (50)

113 ((jak or jak1 or jak2 or jak3 or janus kinase) adj10 inhibitor*).mp. (1538)

114 (janus adj4 kinase adj10 inhibitor?).mp. (927)

115 exp Janus Kinases/ai [Antagonists & Inhibitors] (95)

116 (baricitinib* or 1187594-09-7 or isp4442i3y* or "incb 028050" or incb028050 or ly-3009104 or ly3009104).mp. (409)

117 (tofacitinib* or 477600-75-2 or 87la6fu830* or hsdb 8311 or hsdb8311 or cp-690 550 or cp 690550 or cp690550).mp. (825)

118 (filgotinib* or 1206161-97-8 or 3xvl385q0m* or "glpg 0634" or glpg0634).mp. (173)

119 (upadacitinib* or 1310726-60-3 or 4RA0KN46E0* or abt 494 or abt494).mp. (267)

120 (ruxolitinib* or rux or 941678-49-5 or 82s8x8xx8h* or hsdb 8259 or hsdb8259 or inc 424 or inc424 or incb424 or "incb 018424" or incb018424 or incb 18424 or incb18424 or r-ruxolitinib* or jakafi or jakavi).mp. (486)

121 (itacitinib or "incb 039110" or incb039110 or incb 39110 or incb39110 or 1334298-90-6 or 19j3781lpm*).mp. (34)

122 (ag490 or ag 490 or tyrphostin b42 or tyrphostin ag 490 or tyrphostin ag490 or 133550-30-8).mp. (1)

123 (fedratinib* or tg101348 or tg 101348 or sar302503 or sar 302503 or 936091-26-8 or 6l1xp550i6* or inrebic).mp. (29)

124 (ritlecitinib* or pf-06651600 or pf06651600 or pf 6651600 or pf6651600 or 2140301-97-7 or 132lf5wgh4* or 2192215-81-7 or eag4t1459k* or ritlecitinib malonate or ritlecitinib toslate or ritlecitinib tosylate or ritlecitinib propanedioate).mp. (24)

125 (peficitinib* or peficitinib hydrobromide).mp. (26)

126 or/41-125 (150408)

127 29 and 126 (11897)

128 40 or 127 (12066)

129 exp patient compliance/ (11947)

130 (adhere* or adhering*).mp. (38129)

131 (complian* or complying).ti,ot,ab. (33195)

132 (complian* adj3 (medication? or patient? or treatment* or therapy*)).mp. (31584)

133 patient participation/ (1380)

134 patient preference/ (784)

135 (prefer* adj10 (patient? or patients*)).mp. (14041)

136 noncomplian*.mp. (1267)

137 nonadheren*.mp. (1114)

138 Treatment Refusal/ (264)

139 ((treatment* or therap* or drug? or medication? or agent? or formulation?) adj1 refus* adj10 (patient? or patients*)).mp. (261)

140 attitude/ or exp attitude to health/ (35193)

141 attitude*.mp. (26832)

142 (interrupt* adj3 (treatment* or therapy* or therapeutic*)).mp. (2059)

143 ((drug? or agent? or medication* or formulation? or biologic* or biosimilar? or biosimilar? or therapy* or treatment* or therapeutic*) adj3 prefer*).ti,ot,ab. (6130)

144 141 or 142 or 143 (34706)

145 (patient? or patients*).mp. (1034494)

146 144 and 145 (20642)

147 exp decision making/ (3917)

148 shared decision*.mp. (1522)

149 (decision making adj3 shared).mp. (1510)

150 ((decision* or decide* or choice? or chosen) adj3 (patient? or patients*)).mp. (11263)

151 ((decision* or decide* or choice? or chosen) adj3 (treatment? or therap* or drug* or agent* or medication* or formulation* or biosimilar? or bio similar? or biologic*) adj10 (patient? or patients*)).mp. (7071)

152 Refusal to Participate/ (31)

153 patient dropouts/ (1890)

154 (drop out? or dropout?).mp. (8427)

155 ((patient? or patients*) adj10 (perception* perceive* or perspective*)).mp. (3866)

156 motivation/ or achievement/ or "conflict (psychology)"/ or drive/ or exploratory behavior/ or goals/ or "handling (psychology)"/ or instinct/ or intention/ or "power (psychology)"/ (6689)

157 motivat*.mp. (22235)

158 barrier?.mp. (19496)

159 patient satisfaction/ (11522)

160 ((patient? or patients*) adj6 satisfaction*).mp. (34051)

161 maintenance chemotherapy/ (342)

162 maintenance*.mp. (45726)

163 (maintain* adj10 (therap* or treatment* or drug? or agent? or medication* or chemotherap* or dose? or dosage?)).ti,ot,ab. (21011)

164 persistenc*.mp. (5238)

165 survival/ (134)

166 exp survival analysis/ (20574)

167 survival rate/ (10321)

168 survival*.mp. (114620)

169 retention rate?.mp. (2107)

170 retention*.ti,ot,ab. and (drug?.mp. or dt.fs.) (5605)

171 (drug? adj8 retention*).mp. (1228)

172 "early termination of clinical trials"/ (304)

173 treatment failure/ (3270)

174 treatment outcome/ (134037)

175 withholding treatment/ (367)

176 Substance Withdrawal Syndrome/ (2005)

177 ((duration* or length) adj3 (treatment* or therapy* or therapies* or therapeutic* or medication*)).mp. (44838)

178 ((treatment* or therapy* or therapeutic*) adj1 failure*).mp. (20017)

179 ((treatment* or therap* or chemotherap*) adj3 lack adj3 efficacy*).mp. (353)

180 cessation*.mp. (17589)

181 withdraw*.mp. (44162)

182 dose-response relationship, drug/ (30453)

183 (response* or respond*).mp. (301389)

184 (discontinu* or dis-continu*).mp. (38733)

185 switch*.mp. (16296)

186 (continuing or continue*).mp. (70259)

187 Economics/ (45)

188 exp "costs and cost analysis"/ (10678)

189 economics, pharmaceutical/ (65)

190 exp economics, hospital/ (724)

191 exp economics, medical/ (61)

192 economics, nursing/ (12)

193 (economic? or oeconomic? or cost or costs or costly or costing or price or prices or pricing or pharmaco?economic?).mp. (84536)

194 (value adj2 money).ti,ot,ab. (269)

195 Health Expenditures/ (215)

196 expenditure*.mp. (6886)

197 Budgets/ (27)

198 budget?.mp. (1092)

199 quality-adjusted life years/ (1282)

200 (quality adjusted or adjusted life year?).mp. (5785)

201 (qaly or qalys).ti,ot,ab. (3926)

202 (utility or utilities).mp. (16809)

203 health utility index*.mp. (115)

204 (hui or hui1 or hui2 or hui3 or hui-1 or hui-2 or hui-3).ti,ot,ab. (262)

205 "Quality of Life"/ and ec.fs. (1621)

206 "Quality of Life"/ and (("quality of life" or qol) adj1 (score? or measure?)).ti,ot,ab. (3375)

207 "Quality of Life"/ and (health adj3 status).mp. (2660)

208 "Quality of Life"/ and (("quality of life" or qol) adj3 (improv* or chang*)).ti,ot,ab. (7089)

209 ((dose? or dosage?) adj8 (adjust* or modificat*)).mp. (10149)

210 exp drug administration schedule/ (23891)

211 (interval? adj8 adjust*).ti,ot,ab. (6394)

212 ((dose? or dosage? or treatment* or therapy*) adj8 (taper or tapers or tapering or tapered)).ti,ot,ab. (2236)

213 129 or 130 or 131 or 132 or 133 or 134 or 135 or 136 or 137 or 138 or 139 or 140 or 146 or 147 or 148 or 149 or 150 or 151 or 152 or 153 or 154 or 155 or 156 or 157 or 158 or 159 or 160 or 161 or 162 or 163 or 164 or 165 or 166 or 167 or 168 or 169 or 170 or 171 or 172 or 173 or 174 or 175 or 176 or 177 or 178 or 179 or 180 or 181 or 182 or 183 or 184 or 185 or 186 or 187 or 188 or 189 or 190 or 191 or 192 or 193 or 194 or 195 or 196 or 197 or 198 or 199 or 200 or 201 or 202 or 203 or 204 or 205 or 206 or 207 or 208 or 209 or 210 or 211 or 212 (790720)

214 128 and 213 (8552)

215 exp Registries/ (1029)

216 (register or registry or registries or registered or registration*).mp. (101650)

217 Cohort Studies/ (7646)

218 (cohort* adj6 (study* or studies* or analy*)).mp. (43916)

219 (cohortstudy* or cohortstudies* or cohortanaly*).mp. (6)

220 cohort?.ti,ot,ab. and (study* or studies or analy* or design or trial or trials).mp. (60771)

221 Prospective Studies/ (93051)

222 Retrospective Studies/ (8831)

223 exp Longitudinal Studies/ (142382)

224 (longitudinal or prospective* or retrospective*).mp. (301383)

225 Follow-Up Studies/ (60349)

226 ((follow up or followup) adj6 (trial or trials or study or studies or analy*)).mp. (150671)

227 "Surveys and Questionnaires"/ (26053)

228 exp health care surveys/ or patient reported outcome measures/ or health surveys/ or behavioral risk factor surveillance system/ or exp health status indicators/ (23494)

229 (questionnaire* or questioning or questioned).mp. (140803)

230 interview/ (7)

231 Interviews as Topic/ (1893)

232 exp qualitative research/ (1123)

233 interview*.mp. (39691)

234 qualitative.mp. (16260)

235 Focus Groups/ (636)

236 focus group?.mp. (4340)

237 215 or 216 or 217 or 218 or 219 or 220 or 221 or 222 or 223 or 224 or 225 or 226 or 227 or 228 or 229 or 230 or 231 or 232 or 233 or 234 or 235 or 236 (620372)

238 214 and 237 (4038)

239 limit 238 to yr="2012 - 2020" (2815)

240 limit 239 to english (2411)

241 (mouse or mice or rat or rats or cat or cats or dog or dogs or bovine or swine or pigs or piglets or marmoset? or monkey or monkeys or murine? or sheep or lamb or lambs or cattle or animal or animals).ti. (4151)

242 (patient? or human or humans or participiant? or client?).mp. (1439102)

243 241 not 242 (1221)

244 240 not 243 (2411)

245 ((eular or (european adj4 rheumat*)) adj8 (meeting* or conference* or congress*) adj8 annual).af. (704)

246 ((acr or (american college adj4 rheumat*)) adj8 (meeting* or conference* or congress*) adj8 annual).af. (806)

247 245 or 246 (1506)

248 244 and 247 (215)

249 limit 248 to yr="2019 - 2020" (0)

250 244 not 249 (2411)

251 review.pt. (3088)

252 congress.pt. (44)

253 editorial.pt. (462)

254 letter.pt. (7334)

255 case reports.pt. (1591)

256 journal review.pt. (23539)

257 journal editorial.pt. (2344)

258 journal letter.pt. (4901)

259 case report.ti. (310)

260 case series.ti. (527)

261 congresses.pt. (5)

262 conference abstract.pt. (16804)

263 conference review.pt. (173)

264 conference paper.pt. (134)

265 or/251-264 (61021)

266 250 not 265 (2198)

267 266 or 249 (2198)
